# Zwitterionic Heavier Pnictinidenes in Redox Catalysis

**DOI:** 10.1002/anie.202505697

**Published:** 2025-06-30

**Authors:** Selwin Fernando, Yi Chen Chan, Sergio Fernandez, Enric Sabater, Graham Tizzard, Simon J. Coles, Diego M. Andrada, Oriol Planas

**Affiliations:** ^1^ Department of Chemistry, Molecular Sciences Research Hub Imperial College London 82 Wood Lane, Shepherds Bush London W12 0BZ UK; ^2^ Department of Chemistry Queen Mary University of London Mile End Road London E1 4NS UK; ^3^ General and Inorganic Chemistry Department University of Saarland Campus C4.1 66123 Saarbrücken Germany; ^4^ Institut de Química Computacional i Catàlisi, Departament de Química Universitat de Girona C/M. Aurèlia Capmany 69 Girona 17003 Spain; ^5^ EPSRC National Crystallography Service, School of Chemistry and Chemical Engineering University of Southampton Southampton SO17 1BJ UK

**Keywords:** Antimony, Bismuth, N‐Heterocyclic carbene ligands, Organometallic chemistry, Redox catalysis

## Abstract

Herein, we describe a new class of zwitterionic heavier pnictogen species with *bis*(*N*‐heterocyclic carbene)borate as ligands, enabling the isolation of stable Sb and Bi species in multiple oxidation states. Computational analysis of zwitterionic pnictinidenes revealed their cationic character at the metal centre while holding unique electronic properties that contribute to their nucleophilicity and stability. These systems participate in oxidative addition and reductive elimination processes, and display redox catalytic activity in hydrodefluorination reactions, marking a unique example of cationic pnictinidene catalysing a redox transformation and providing reactivity beyond the constraints of pincer ligands. Additionally, we report on a novel dehydrogenative thiolation of silanes. This work expands the scope of low‐valent pnictogen chemistry, providing a novel platform for main group redox catalysis.

## Graphical abstract

**Zwitterionic pnictinidenes**: A new class of zwitterionic heavier pnictogen species stabilized by bis(*N*‐heterocyclic carbene)borate ligands is reported, enabling the isolation of Sb and Bi in multiple oxidation states. These thoroughly characterized systems exhibit cationic character and engage in redox catalysis, facilitating catalytic hydrodefluorination and dehydrogenative thiolation of silanes, expanding the scope of main group redox chemistry.

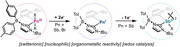

## Introduction

Ligand design has enabled the engagement of main group elements in substrate coordination, small molecule activation and redox catalysis, areas that were traditionally limited to the realm of transition metals.^[^ ^1^, ^2^, ^3^, ^4^, ^5^ ^]^ Group 15 elements (pnictogens, Pn) have shown significant potential in this area,^[^ ^6^ ^]^ prompted by strategies such as the introduction of geometric constraints,^[^ ^7^, ^8^ ^]^ and the reduction to low‐valent species. Regarding the latter, low‐valent antimony (Sb) and bismuth (Bi) are particularly powerful nucleophiles due to an easily oxidizable lone‐pair of electrons located in a p‐orbital. Over the past decade, a variety of neutral low‐valent Sb and Bi species have been reported using diverse ligand frameworks, enabling the isolation of base‐stabilized pnictinidenes,^[^ ^9^, ^10^, ^11^, ^12^, ^13^ ^]^ as well as rare monocoordinated triplet pnictinidene species,^[^ ^14^, ^15^ ^]^ which were long considered challenging to access. Among neutral low‐valent heavier pnictogen species, the use of *N,C,N*‐pincer scaffolds has resulted particularly successful, resulting in pnictinidene systems capable of engaging in redox reactivity and metal–ligand cooperativity,^[^ ^16^, ^17^, ^18^, ^19^ ^]^ displaying halogen‐bond acceptor properties,^[^ ^20^ ^]^ and allowing the possibility to merge pnictinidene chemistry with light‐induced processes.^[^ ^21^, ^22^, ^23^, ^24^ ^]^ Nonetheless, tridentate *N,C,N*‐pincer ligands present some limitations, such as a saturated pnictinidene centre with no available coordination sites, which can lead to catalytically unproductive ligand‐centred reactivity.^[^ ^25^, ^26^ ^]^ Moreover, the structural rigidity of these frameworks often limits the extent of electronic and steric tunability, which may restrict access to reactive intermediates or hinder the fine‐tuning of reactivity towards specific transformations. A compelling example of the importance of ligand diversity can be found in phosphorus redox catalysis, where the development of a wide range of ligand architectures has been instrumental in advancing P(III)/P(V) cycles.^[^ ^27^, ^28^, ^29^ ^]^ These observations underscore the need for alternative ligand platforms that can effectively enable redox catalysis with heavier pnictogens.^[^ ^30^ ^]^

**Figure 1:**
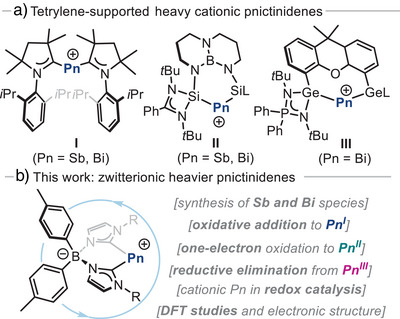
a) Tetrylene‐supported low‐valent Pn cations (Sb, Bi). b) This work: bis(*N*‐heterocyclic carbene) ligands for the synthesis of zwitterionic pnictogen species in multiple oxidation states.

Recent accomplishments in low‐valent double bonded species,^[^ ^31^, ^32^, ^33^ ^]^ and particularly heavier Pn cations,^[^ ^34^ ^]^ have provided paramount insight into how to access heavier pnictinidene species with unique electronic structure and reactivity patterns. Indeed, low‐valent Pn cations are intriguing due to their isoelectronic relationship with highly‐reduced group 14 element compounds, so‐called tetrylones.^[^ ^35^ ^]^ On one hand, Majumdar and co‐workers reported a unique example of bis(phosphines)‐supported Sb(I) cation, which was shown to coordinate transition metals and undergo nucleophilic reactions.^[^ ^36^, ^37^ ^]^ Tetrylene‐based ligands have been particularly successful stabilizing platforms (Figure 1a). While attempts to isolate carbene‐supported species with *N*‐heterocyclic carbenes (NHC) proved challenging,^[^ ^38^ ^]^ Roesky and co‐workers successfully reported the synthesis of the first ylidene‐supported Sb(I) and Bi(I) cations (**I**, Figure 1a) using cyclic (alkyl)(amino)carbenes (cAAC) as ligand.^[^ ^39^ ^]^ This seminal study set the precedent for further investigations on other ylidene ligands, including *N*‐heterocyclic silylenes (**II**, Figure 1a),^[^ ^40^ ^]^ and *N*‐heterocyclic germylenes (**III**, Figure 1a).^[^ ^41^ ^]^ Their particular bonding led to stable stibinidene and bismuthinidene cations with nucleophilic properties, as the pnictogen centre in these species are endowed with two lone pairs. Despite the significant progress achieved, the organometallic reactivity of such pnictinidene cations remain underexplored, particularly their potential applications in redox catalysis.

Bulky anionic bis(NHC)borate ligands, introduced by Hofmann,^[^ ^42^ ^]^ have recently gained attention in Fe organometallics,^[^ ^43^, ^44^, ^45^, ^46^ ^]^ and have also found use to stabilize germylene species.^[^ ^47^, ^48^ ^]^ Based on the chelating effect and anionic charge of the bis(NHC)borate ligand, together with the strong *σ*‐donor ability and lower *π*‐acidity of NHCs compared to cAAC scaffolds,^[^ ^49^ ^]^ we hypothesized they would allow the isolation of Pn(I) zwitterions. We envisioned that in these species, the positive charge would primarily reside on the Pn centre, while the negative charge would be localized on the borate unit,^[^ ^50^, ^51^, ^52^ ^]^ yielding an overall neutral compound with ylidone nature. In addition, bis(NHC)borate ligands offer versatility, as they are easily modified to introduce different substituents on both the borate and the imidazole residues.^[^ ^53^ ^]^ Herein, we report on the synthesis and full characterization of a new class of zwitterionic heavier pnictogen species in oxidation states +1, +2 and +3 stabilized by a bis(NHC)borate scaffold. Pnictinidene species have been investigated for their organometallic reactivity, including key oxidative addition and reductive elimination processes. Furthermore, they have been successfully applied to redox catalytic transformations, offering an electronically distinct and structurally versatile alternative to the current state‐of‐the‐art pnictogen‐based catalysts.

## Results and Discussion

### Synthesis and Characterization of Bis(NHC)Borate‐Supported Low‐Valent Pnictogen Complexes

**Scheme 1:**
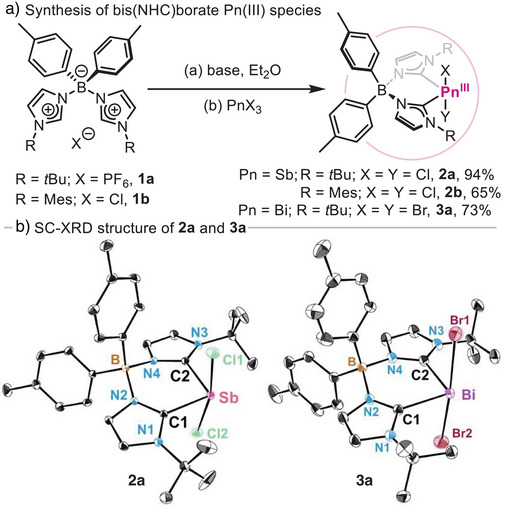
a) Synthesis of Pn(III) species. b) Molecular structure of **2a** and **3a**. Hydrogen atoms and solvent molecules are omitted for clarity, thermal ellipsoids are displayed at 50% probability.

Initially, a family of differently substituted bis(NHC)borate ligands was synthesized adapting a previously reported protocol.^[^ ^42^ ^]^ Reaction of ditolylboron chloride with *N*‐alkyl and *N*‐aryl substituted imidazoles resulted in the formation of ligands **1a** and **1b**.^[^ ^54^ ^]^ The synthesis of bis(NHC)borate Sb(III) and Bi(III) complexes **2a‐b** and **3a** (Scheme 1a) was performed by deprotonation of the corresponding salts with 2.0 equiv. of an appropriate alkyllithium base, followed by reaction with pnictogen trihalide salts (PnX_3_; Pn═Sb, Bi and X═Cl, Br) in diethyl ether. The resulting Pn(III) species represent a unique example of two‐coordinated heavy pnictogen compounds supported by a bis(NHC) ligand.^[^ ^55^ ^]^ Single‐crystal X‐ray diffraction (SC‐XRD)^[^ ^56^ ^]^ analysis of **2a** resulted in a Sb centre displaying a disphenoidal coordination, where the bis(NHC)borate ligand occupies equatorial positions and chloride ligands are positioned axially (see Scheme 1b). The Sb–C1 and Sb–C2 bond distances are 2.1596(10) and 2.1586(10) Å, respectively, representing the shortest Sb(III)─C_carbene_ bond distance reported to date.^[^ ^11^, ^57^, ^58^, ^59^, ^60^, ^61^, ^62^, ^63^ ^]^ In addition, the Sb(III)─C bond distance obtained is consistent with the sum of single‐bond atomic radii for Sb and C.^[^ ^64^ ^]^ Interestingly, the heavier Bi analogue **3a**, also shows unusually short Bi(III)─C_carbene_ bond distances [2.2730(10) and 2.2894(10) Å] compared to other carbene‐Bi complexes (2.31–2.46 Å).^[^ ^58^, ^65^, ^66^ ^]^

**Scheme 2:**
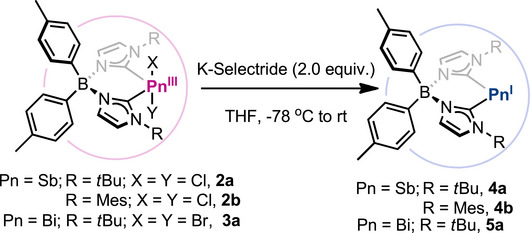
Reduction of Pn(III) to obtain Pn(I) species.

Reduction of Pn(III) species **2a‐b** and **3a** with 2.0 equiv. of K‐Selectride at −78 °C in THF resulted in the formation of **4a‐b** and **5a** (Scheme 2), with concomitant formation of potassium salts and dihydrogen, which was detected by ^1^H NMR (*δ* = 4.49 ppm in THF‐d_8,_ Figure S13).^[^ ^54^ ^]^ Reduced species **4a‐b** and **5a** are air‐sensitive and decompose in chloroalkanes, but remain stable in ethers, acetonitrile, and hydrocarbons and exhibit tolerance to small amounts of water. Importantly, they are also remarkably stable at high temperatures, showing no signs of decomposition when stirred in benzene at 80 °C for several days. The UV/Vis spectrum of pnictinidene species exhibits two main absorptions at *λ*_max_ = 335 and 400 nm (for **4a**) and *λ*_max_ = 330 and 385 nm (for **5a**), which are outlined in Figure S51. Based on TD‐DFT analysis, these may be assigned to HOMO→LUMO + 1 and HOMO→LUMO + 5 transitions (Tables S114 and S115), respectively. The ^1^H NMR of **4a** exhibits four sets of aromatic signals assigned to the imidazole and tolylborate moieties of the ligand backbone, indicating C*_2v_* symmetric structures. In addition, ^13^C NMR unveils a Sb─C_carbene_ chemical shift of *δ* = 155.8 ppm, which is similar to Sb(III) species **2a** (*δ* = 153.5 ppm) and **2b** (*δ* = 156.5 ppm), suggesting unchanged electronic environments after reduction. On the contrary, the Bi analogue **5a** shows a slightly upfield‐shifted Bi─C_carbene_^13^C NMR signal (*δ* = 168.1 ppm), compared to **3a** (*δ* = 175.0 ppm).

**Figure 2:**
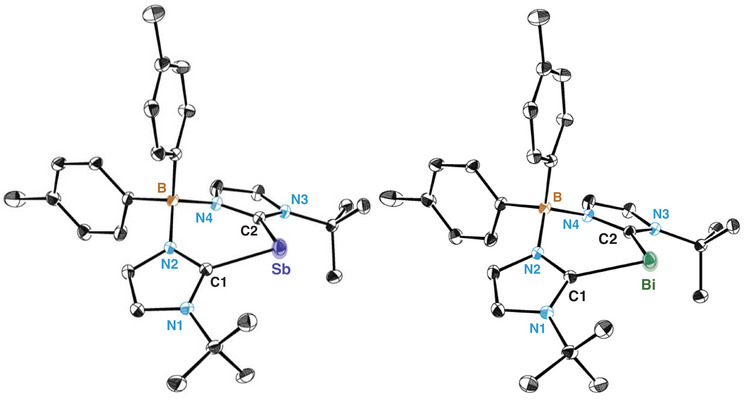
Molecular structure of bis(NHC)borate‐supported stibinidene **4a** (left) and bis(NHC)borate‐supported bismuthinidene species **5a** (right). Hydrogen atoms and solvent molecules are omitted for clarity and thermal ellipsoids are displayed at 50% probability. Selected experimental and calculated [PBE0‐D3(BJ)/def2/SVP] bond length (Å) and angles (°) of **4a**: Sb‐C1 2.1563(9) [2.158], Sb‐C2 2.1521(8) [2.158], C1‐Sb‐C2 84.03(4) [88.3]; **5a**: Bi‐C1 2.2805(11) [2.269], Bi‐C2: 2.2878(11) [2.269], C1‐Bi‐C2: 85.30(4) [86.1].

Pnictinidene species **4a‐b** and **5a** were further characterized by SC‐XRD (Figure 2). The solid‐state structure of **4a‐b** and **5a** shows an endocyclic Pn(I) centre that adopts a bent geometry. Interestingly, the Sb(I)─C_carbene_ bond distance in **4a** remains similar to its Sb(III) counterpart **2a**, with values of 2.1563(9) and 2.1521(8) Å for C1─Sb and C2─Sb, respectively. These bond lengths fall within the expected sum of covalent radii for a C─Sb single bond (2.15 Å),^[^ ^67^ ^]^ and suggest a small degree of *p*‐backdonation from the Sb(I) p lone pair into the formally empty p orbital of the carbene carbon. This is in sharp contrast to the significant *π*‐backdonation contribution observed for previously reported carbene‐supported stibinidene systems. For example, the use of one diamidocarbene (DAC) and cAAC ligands results in neutral (DAC)─Sb(Ph) and cAAC─SbCl species with Sb(I)─C_carbene_ bond distances of 2.068(7) and 2.082(5) Å, respectively.^[^ ^11^, ^12^ ^]^ The Bi analogue **5a** similarly exhibits an unchanged Bi(I)─C_carbene_ bond distance after reduction, with values of 2.2805(11) and 2.2878(11) Å for C1─Bi and C2─Bi, respectively. The structural parameters of **5a** can also be evaluated against neutral cAAC‐supported Bi(I) species ^Et2^cAAC─Bi(Ph) reported by Gilliard and co‐workers,^[^ ^68^ ^]^ which shows a significantly shorter Bi(I)─C_carbene_ bond distance [2.199(2) Å] due to a strong *π*‐backdonation. Intriguingly, Pn(I)─C_carbene_ bond distances in **4a** and **5a** closely resemble those reported by Roesky and co‐workers in **I‐Sb** [2.145(2) and 2.1493(2) Å] and **I‐Bi** cations [2.270(3) and 2.314(3) Å] (Figure 1),^[^ ^39^ ^]^ suggesting a similar bonding situation between carbene and Pn centre.

### Electronic Structure Analysis

The electronic structure of zwitterionic pnictinidenes **4a** and **5a** was investigated by density functional theory (DFT) calculations at the PBE0‐D3(BJ)/def2‐SVP level of theory (see Tables S98 and S99).^[^ ^69^, ^70^, ^71^, ^72^, ^73^ ^]^ Their computed structural parameters show good agreement with those obtained by SC‐XRD analysis (see Figure 2), and they exhibit singlet–triplet gap energies (at PBE0‐D3(BJ)/def2‐TZVPP) of 39.0 (**4a**) and 31.8 (**5a**) kcal mol^−1^.^[^ ^54^ ^]^ The diradical character of pnictinidenes **4a** and **5a** was investigated at the CASSCF(2,2) level (Table S109), displaying CI coefficients for closed shell wavefunction (20) and the double excitation (02) of *c*_0_ = 0.9946 and *c*_d_ = −0.1037 for **4a**, and *c*_0_ = −0.9929 and *c*_d_ = 0.1185 for **5a**. These values are consistent with a diradical character of 14.7% and 16.8% for **4a** and **5a**, respectively,^[^ ^74^ ^]^ according to Neese's formulation.^[^ ^75^, ^76^ ^]^ Despite the non‐negligible diradical character, our attempts to find a broken‐symmetry solution were unsuccessful.

**Figure 3:**
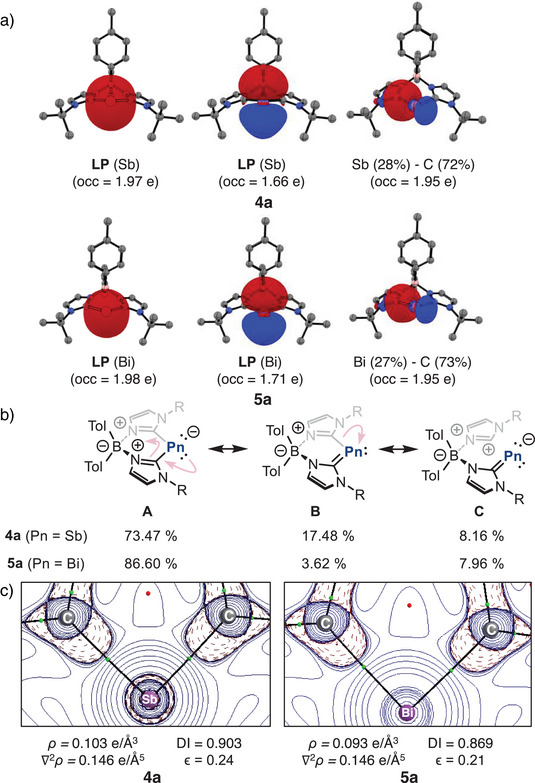
a) Selected NBOs for **4a** and **5a**. b) NRT weight of the main resonance structures of bis(NHC)borate‐stabilized low‐valent Sb and Bi species **4a** and **5a**. c) Laplacian distribution of the electron density of **4a** and **5a** at PBE0‐D3(BJ)/def2‐TZVPP//PBE0‐D3(BJ)/def2‐SVP level of theory. Contour line diagrams of the Laplacian distribution ∇^2^ *ρ(r)*. Dashed red lines indicate areas of charge concentration (∇^2^ *ρ(r)* < 0) while solid blue lines show areas of charge depletion (∇^2^ *ρ(r)* > 0). The thick solid lines connecting the atomic nuclei are the bond paths and the small dots are the critical points. Bond Critical Points (in green) and Ring Critical Point (in red).

The molecular Kohn–Sham (KS) orbitals and Natural Bond Orbital (NBO) analysis reveal two lone pairs at the pnictogen centre, together with two highly polarized *σ*(Pn─C_carbene_) bonds (Figure 3a, Tables S103 and S104). The first is a *σ*‐type lone pair and possesses strong s character (**4a** 83.11% and **5a** 89.69%), while the second lone pair is a *π*‐type pair of electrons endowed with strong p character (**4a** 99.80% and **5a** 99.87%) with lower occupancy (1.66 e for **4a** and 1.72 for **5a**). These two lone pairs are majorly presented by HOMO‐6(**4a**)/HOMO‐8(**5a**) and HOMO frontier orbitals (see Figure S40), respectively. In addition, the two Pn─C_carbene_ *σ*‐bonding orbitals have significant contributions from the sp^2^ orbital of the carbene carbon, accounting for 72% for **4a** and 73% for **5a**. The Wiberg bond indices (WBI) of Pn─C_carbene_ bonds in **4a** and **5a** (Table S102) are consistent with a single bond character, with values of 0.91 and 0.86, respectively. Strikingly, Pn─C_carbene_ WBI are lower than those reported for pnictinidene cations **I** (Figure 1), for which computed values of 1.02 (**Sb**) and 0.94 (**Bi**) were obtained.^[^ ^39^ ^]^ Additionally, the natural population analysis (NPA) indicates positive partial charges of +0.22*e* at Sb and +0.21*e* at Bi in **4a** and **5a**, respectively. In this case, the positive charge located at the pnictogen centre is lower than with cAAC ligands (+0.72*e* at **I‐Sb** and +0.69*e* at **I‐Bi**),^[^ ^39^ ^]^ but higher than those cations directly bonded to other heteroatoms: Sb (+0.05*e*) in Majumdar's Sb species,^[^ ^36^ ^]^ Sb (−0.34*e*) and Bi (−0.28*e*) in **II**,^[^ ^40^ ^]^ and Bi (−0.23*e*) in **III**.^[^ ^41^ ^]^

Natural Resonance Theory (NRT) was used to estimate significant Lewis structures, which are shown in Figure 3b. The dominant resonance structure in **4a** (73.47%) and **5a** (86.60%), depicted as **A**, features the pnictogen atom bearing two lone pairs and forming two single bonds with each adjacent carbene carbon atom. Additionally, two other resonance structures contribute to a lesser extent (**B** and **C**), exhibiting *π*‐conjugation between Pn and C_carbene_ atoms. In **B**, the Pn─C_carbene_ bond is characterized by electron sharing (**4a**: 17.48%, **5a**: 3.62%), while in **C**, the electron pair of the Pn─C_carbene_ bond is assigned to the Pn element (**4a**: 8.16%, **5a**: 7.96%). These results support a description in which the bonding is predominantly of donor–acceptor character, featuring a Pn centre with two nonbonding electron pairs and limited π‐conjugation with the ligand framework. Note that resonance structures display formal charges, which should be distinguished from computed partial charges discussed above.^[^ ^77^ ^]^

Electronic density distribution was analysed using the quantum theory of atoms in molecules (QTAIM) framework.^[^ ^78^, ^79^ ^]^ The Laplacian distribution ∇^2^ *ρ(r)* on the C─Pn─C plane is depicted in Figure 3c for **4a** and **5a** and shows electron accumulation in the bonding region around the carbene carbon atoms pointing towards the pnictogen centre. The electron density and Laplacian values at the bond critical points (BCPs) are 0.103e Å^−3^ and 0.146e Å^−5^ for **4a**, and 0.093e Å^−3^ and 0.146e Å^−5^ for **5a**, respectively. These values are consistent with an ionic interaction, as indicated by the low electron density (*ρ(r)*) and positive Laplacian values (∇^2^ *ρ(r)*) at the bond critical points, along with a local electron density H close to zero (Table S109) and a weak double bond character (*ϵ *= 0.24 (**4a**) ϵ = 0.21 (**5a**)).^[^ ^80^ ^]^ Notably, the Laplacian distribution does not exhibit electron density accumulation around the Bi atom, neither a shell structure nor features consistent with two lone pairs are observed. This is consistent with the diffuse character of the valence shell in heavier elements, as previously discussed in the context of the Laplacian and other electron pair localization functions.^[^ ^81^ ^]^ Similar results are obtained within the representative series of PnH_3_ and PnPh_3_ (Pn = N─Bi) as outlined in Figure S46, and also on the related systems **Ia** and **Ib**.^[^ ^41^ ^]^ The oxidation state of the pnictogen atoms was determined by effective oxidation state analysis (EOS),^[^ ^82^, ^83^ ^]^ treating pnictogen and ligands as distinct fragments. Calculations indicate an oxidation state of +1 for both Sb and Bi congeners, with a reliability index (R%) of 82 and 87, respectively, using both TFVC and NAO atomic definitions (Table S110). To validate this assignment, we extended the analysis to other pnictogen species previously assigned as +1 oxidation state, observing good agreement with the reported values. The frontier effective fragment orbitals (EFOs)^[^ ^84^, ^85^ ^]^ and their occupations (Table S111) further confirm that the two lone pairs are primarily localized on the Sb and Bi atoms, consistent with their expected electronic configuration in the +1 oxidation state.

To further investigate the nature of the pnictogen‐ligand bonds of **4a** and **5a** species, we performed energy decomposition analysis (EDA)^[^ ^86^, ^87^, ^88^ ^]^ in combination with the natural orbitals for chemical valence (NOCV)^[^ ^89^, ^90^, ^91^ ^]^ method.^[^ ^89^, ^92^ ^]^ In **4a** and **5a**, we selected the pnictogen centre and the bis(NHC)borate ligand as distinct fragments. While multiple electronic reference states can be considered, NBO and QTAIM analyses indicate an ionic C_carbene_─Pn (Sb, Bi) interaction, with two lone pairs localized on the pnictogen centre. Based on this, we adopted a closed‐shell fragment description, where Sb(1+) and Bi(1+) are assigned as ¹D reference state, and the ligand carries a formal negative charge with a *σ*‐lone pair localized on each carbene carbon atom. Table 1 summarizes the numerical results of EDA‐NOCV calculations.

**Table 1:** EDA‐NOCV results at BP86‐D3(BJ)/TZ2P. All values are in kcal mol^−1^.^a)^

	**4a**	**5a**
**Fragmentation**	L^−^ (S)^b)^; Sb^+^ (^1^D, 5s^2^5p=^0^5p_║_^0^5p_┴_^2^)	L^−^ (S)^b)^; Bi^+^ (^1^D, 6s^2^6p=^0^6p_║_^0^6p_┴_^2^)
**Δ*E*_int_**	−319.2	−294.2
**Δ*E*_Pauli_**	361.5	319.7
**Δ*E*_disp_** ^c)^	−18.1 (2.7%)	−19.2 (3.1%)
**Δ*E*_elst_** ^c)^	−370.2 (54.4%)	−345.6 (56.3%)
**Δ*E*_orb_** ^c)^	−292.4 (43.0%)	−249.2 (40.6%)
**Δ*E*_orb‐σ(+,+)‐donation_** ^d)^	−151.6 (51.9%)	−124.0 (49.8%)
**Δ*E*_orb‐σ(+,−)‐donation_** ^d)^	−68.2 (23.3%)	−62.2 (25.0%)
**Δ*E*_orb‐π‐backdonation_** ^d)^	−22.4 (7.7%)	−19.9 (8.0%)
**Δ*E*_orb rest_** ^d)^	−50.1 (17.1%)	−43.1 (17.3%)
**Δ*E*_prep_**	31.0	29.3
***D*_e_**	288.2	264.9

^a)^ All calculations were performed on the PBE0‐D3(BJ)/def2‐SVP optimized structures.

^b)^ S stands for singlet electronic state.

^c)^ The value in parenthesis gives the percentage contribution to the total attractive interactions Δ*E*_elst_ + Δ*E*_orb_ + Δ*E*_disp_.

^d)^ The value in parenthesis gives the percentage contribution to the total orbital interaction term.

**Figure 4:**
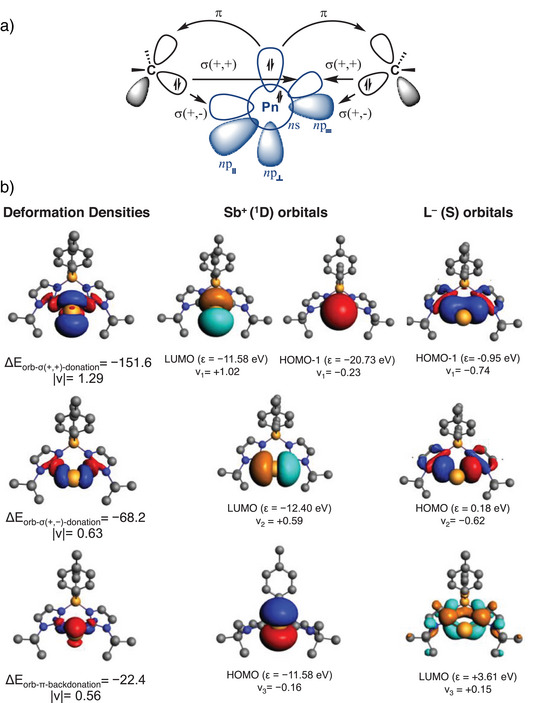
a) General orbital scheme. b) Plot of deformation densities ∆*ρ* (isocontour value = 0.003) of the pairwise orbital interactions (isocontour value = 0.03) between Sb^+^(^1^D) and bis(NHC)borate ligand (L^‒^(S)) in the singlet state, associated energies ∆*E* in kcal mol^−1^ and eigenvalues *ν* in a.u. The red colour shows the charge outflow, whereas blue shows charge density accumulation. Hydrogen atoms are omitted for clarity.

The bond dissociation energies LPn→L^–^ + Pn^+^(^3^P) (D_e_) of 288.2 (**4a**) and 264.9 (**5a**) kcal mol^−1^ suggest a thermodynamic stability with respect to pnictogen atom release. These values are notably higher than the bond dissociation energy of **I‐Sb** (223.4 kcal mol^−1^) and **I‐Bi** (203.6 kcal mol^−1^),^[^ ^93^ ^]^ suggesting stronger ligand‐pnictogen interactions. The interaction energy (Δ*E*_int_) follows a similar trend to *D*_e_, as the preparation energy (Δ*E*_prep_) remains low due to minimal geometric distortion in the ligand. However, for the pnictogen atom, *ΔE*_prep_ includes the excitation energy associated with the Pn(^3^P)→Pn(^1^D) transition. A detailed decomposition of the interaction energy reveals that the electrostatic interaction contributes the most to stabilization (54.4% for **4a** and 56.3% for **5a**), reinforcing the ionic nature of the bonding. Orbital interactions account for approximately 40% of the stabilization, while dispersion interactions represent only 3%. Interestingly, the orbital interaction energy is numerically lower for **4a** (−319.2 kcal mol^−1^) and **5a** (−294.2 kcal mol^−1^) than for **I‐Sb** (−276.1  kcal mol^−1^) and **I‐Bi** (−232.9 kcal mol^−1^) (see Table S112), indicating greater stabilization.^[^ ^93^ ^]^ A further breakdown of orbital interactions reveals three dominant donor–acceptor orbital pairs, which contribute differently to bonding based on their deformation densities. The corresponding deformation densities and fragment orbitals for **4a** are depicted in Figure 4, while those for **5a** are provided in Figure S48.

The first deformation density and orbital interaction corresponds to *σ*‐donation, arising from the in‐phase (+,+) combination of the ligand lone pair (HOMO‐1) donating into the vacant (*n*)*p*_║_^0^ orbital of the pnictogen centre. A minor contribution from the *ns* ^2^ orbital of the pnictogen atoms is observed due to orbital hybridization on wavefunction relaxation step of EDA calculation.^[^ ^54^ ^]^ It is important to note that the eigenvalue of the total deformation density is 1.29e, suggesting that alternative representations could be also suitable.^[^ ^94^ ^]^ The second orbital interaction is related to the out‐of‐phase (+,−) combination of the lone pairs (HOMO) interacting with the vacant (*n*)*p* = ^0^ orbital. The third orbital interaction contribution is associated with the *π*‐backdonation, where the (*n*)*p_┴_* ^2^ orbital of the Pn donates into the vacant *π*‐orbital of the ligand (LUMO). The orbital interaction energies indicate a strong *σ*‐donation from the ligand to the Pn atom, with values of −151.6 and −68.2 kcal mol^−1^ for **4a**, and −124.0 and −62.2 kcal mol^−1^ for **5a**. Notably, these values exceed those observed for cAAC ligands (−115.4 and −58.5 kcal mol^−1^ for **I‐Sb** and −91.4 and −54.2 kcal mol^−1^ for **I‐Bi**),^[^ ^93^ ^]^ likely due to the anionic nature of the bis(NHC)borate scaffold. In contrast, the stabilization energy due to *π*‐backdonation is −22.4 kcal mol^−1^ for **4a** and −19.9 kcal mol^−1^ for **5a**, which is less than a half of the reported *π*‐backdonation for cAAC‐stabilized cationic pnictinidenes.^[^ ^39^ ^]^ This is expected given the lower *π*‐acidity of the bis(NHC)borate ligand.^[^ ^95^ ^]^

### Stoichiometric Reactivity of Bis(NHC)Borate‐Supported Stibinidenes

**Scheme 3:**
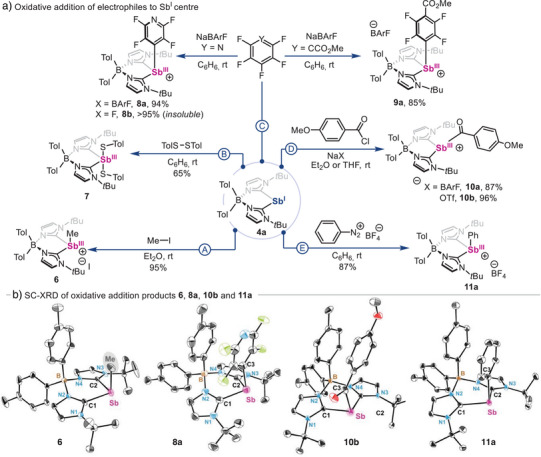
a) Oxidative addition of polar electrophiles to stibinidene **4a**. b) Molecular structures of species **6, 8a 10b** and **11a**. H‐atoms and solvent molecules are omitted for clarity and ellipsoids are displayed at 50% probability.

After experimentally and computationally characterizing pnictinidene species **4a** and **5a**, their reactivity was investigated. These studies were performed using Sb(I) species **4a** (Scheme 3) as model, given that stibinidene‐mediated organometallic redox processes remain underexplored compared to its heavier analog.^[^ ^96^, ^97^ ^]^ First, **4a** was reacted with methyl iodide (MeI) in order to examine its nucleophilic character (Scheme 3a, pathway A), resulting in compound **6** (95% yield). Reactivity of **5a** with MeI resulted in decomposition. SC‐XRD analysis (Scheme 3b) reveals an Sb centre that adopts a see–saw geometry, with a long Sb–I distance in the range of 3.7022(9)–3.9327(7) Å, which remains within the sum of the van der Waals radii of both atoms.^[^ ^68^ ^]^ In addition, the Sb–Me distance is in the range of 2.138(10)–2.186(8) Å, similar to SbMe_3_ (2.16 Å). Interestingly, SC‐XRD analysis also shows that the Me residue in compound **6** lies in close proximity to a tolyl ring of the ligand backbone (3.521–4.090 Å), which likely contributes to the unusually high‐field ^1^H NMR chemical shift (*δ* = 0.57 ppm) observed for the Sb─Me moiety.^[^ ^98^ ^]^ In fact, the methyl group in cationic species **6** appears significantly more shielded than in the neutral parent compound SbMe₃ (*δ* = 0.65 ppm) and related Sb–methyl halide species (Me₂SbI, *δ* = 1.25 ppm; Me₂SbBr, *δ* = 1.02 ppm; MeSbBr_2_, *δ* = 1.49 ppm), supporting the idea that magnetic anisotropy from the nearby tolyl ring is a plausible cause for the observed high‐field shift.^[^ ^99^, ^100^ ^]^ The nucleophilicity of **4a** was also evaluated using disulfides, offering a comparison point with *N,C,N*‐pincer supported stibinidenes.^[^ ^18^, ^101^ ^]^ Thus, compound **4a** was reacted with 1.0 equiv. of ditolyl disulfide in benzene at room temperature (Scheme 3a, pathway B), affording **7** as a yellow powder in 65% yield. Crystals of **7** could not be obtained, but ^1^H NMR analysis in THF‐d_8_ revealed a fully symmetric bis(NHC)borate scaffold, with two pairs of signals corresponding to borate‐ (*δ* = 7.07 and 6.96 ppm) and thiol‐bonded (*δ* = 7.05 and 6.82 ppm) tolyl residues,^[^ ^54^ ^]^ suggesting that both thiolate ligands are attached to the Sb centre.

Given the growing relevance of polyfluorinated arenes in pnictogen redox catalysis,^[^ ^16^, ^102^, ^103^ ^]^ their reactivity with **4a** was also tested (Scheme 3a, pathway C), as the activation of C─X bonds with Sb species is yet to be reported. Initially, the characteristic yellow colour of **4a** rapidly fades after the addition of 1.0 equiv. of pentafluoropyridine in presence of 1.0 equiv. of sodium tetrakis[3,5‐bis(trifluoromethyl)phenyl]borate (NaBArF), resulting in **8a**, which was fully characterized. If the reaction is performed in absence of NaBArF, highly insoluble **8b** is obtained instead. Similarly to **6**, the ^1^H NMR spectrum of **8a** in CDCl_3_ also shows two non‐equivalent tolyl moieties. Indeed, SC‐XRD analysis of **8a** allowed its unambiguous structural determination. Interestingly, the electron‐poor Sb─PyrF_4_ ring is in close proximity to one of the electron‐rich tolyl residues from the ligand scaffold, showing face‐centred pairing with a centroid–centroid distance of 3.3560(15) Å, which is consistent with a stabilizing polar *π–π* stacking interaction.^[^ ^104^ ^]^ This is less pronounced in species **9a** (centroid–centroid distance of 3.4777(11) Å, which was obtained in 85% yield. Noteworthy, both Sb─ArF monocations **8a** and **9a** possess similar Sb─C_aryl_ bond distances, with values of 2.170(2) and 2.1835(19), respectively. These are unique examples of C─F oxidative addition to an Sb(I) centre, promising new avenues for the development of innovative catalytic processes beyond the use of prototypical *N,C,N*‐pincer‐supported species.

The reactivity of zwitterionic stibinidene **4a** was also tested towards aroyl chlorides to synthesize acylstibine species, heavier analogs of amides. While acyl‐substituted species of group 14 elements have become important reagents in organic synthesis,^[^ ^105^ ^]^ their group 15 counterparts remain virtually unknown.^[^ ^106^, ^107^, ^108^ ^]^ Thus, when anisoyl chloride was added to a 1:1 mixture of **4a** and NaX (X═BArF, OTf), monocationic anisostibides **10a** and **10b** were obtained in excellent yields (Scheme 3a, pathway D).^[^ ^54^ ^]^ Given the fundamental interest in such species, we also attempted the synthesis of monocationic anisobismuthides starting from **5a**. However, these efforts were unsuccessful, yielding a complex mixture of products. Noteworthy, ^1^H NMR of anistibides **10a** and **10b** reveals shielded *ortho*‐C─H bonds respect to the carbonyl group (*δ* = 7.01 and 6.85 ppm for **10b**) when compared to the starting anisoyl chloride (*δ* = 7.9 and 6.4 ppm), indicative of the aromatic current shielding from the borate–tolyl ring. The ^13^C NMR spectrum for the Sb─C(O)Ar carbonyl group in **10a** and **10b** exhibits a chemical shift of *δ* = 211.6 (C_6_D_6_) and 220.8 (THF‐d_8_) ppm, downfield‐shifted from anisoyl chloride (165 ppm). This feature indicates an electron‐deficient carbonyl group, consistent with the cationic Sb centre and analogous to heavier acyl‐substituted group 14 metalloids.^[^ ^109^, ^110^ ^]^ In addition, the computed carbonyl stretching frequency for **10a** (1742 cm^−1^) is estimated red shifted with respect to anisoyl chloride (1894 cm^−1^) indicating a strong Sb–C(O) conjugation.^[^ ^54^ ^]^ SC‐XRD analysis of **10b** revealed a disphenoidal geometry (Scheme 3b), with anisoyl and tolyl rings in close proximity (3.706 Å). Interestingly, monocation **10b** shows an unusually long Sb─C*sp* ^2^ bond distance of 2.2490(8) Å when compared to **8a**, **9a**, or **11a**, but similar to other acyl‐metal species.^[^ ^111^, ^112^, ^113^, ^114^ ^]^ Overall, **10a** and **10b** constitute rare examples of stable and cationic heavier analogs of amides.

**Scheme 4:**
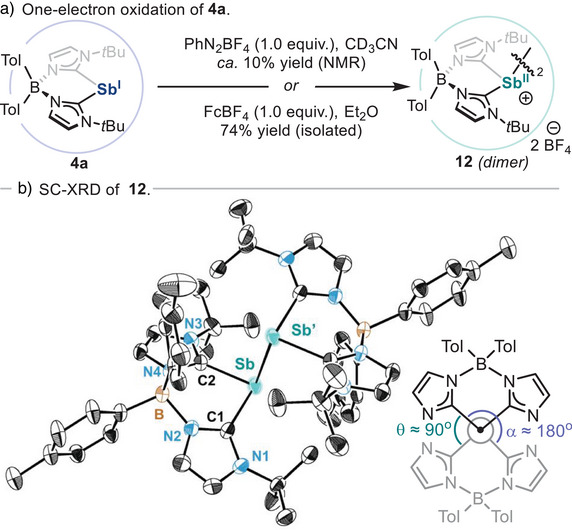
a) Synthesis of bis(NHC)borate‐Sb(II) dimeric species **12**. b) Molecular structure of **12** and Newman projection along the Sb─Sb’ axis with dihedral angles (*t*Bu groups on imidazoles not shown for clarity). H‐atoms and tetrafluoroborate anions are omitted for clarity and ellipsoids are displayed at 50% probability.

Finally, stibinidene **4a** was also submitted to oxidative addition with aryl diazonium salts (Scheme 3a, pathway E).^[^ ^23^ ^]^ Reaction of **4a** with 1.0 equiv. of benzenediazonium tetrafluoroborate resulted in the formation of the corresponding aryl‐Sb(III) species **11a** (87% yield by ^1^H NMR, Figure S1) together with trace amounts of benzene and other unidentified byproducts (Figures S2 and S3).^[^ ^54^ ^]^ Isolation of **11a** was attempted by crystallization of the reaction crude, but this resulted in the serendipitous isolation of dicationic Sb(II)─Sb(II) dimer **12** (Scheme 4). Firstly, structural and spectroscopic features of monocationic **11a** were confirmed by synthesizing it via an alternative route.^[^ ^54^ ^]^ SC‐XRD analysis of **11a** (Scheme 3b) reveals structural parameters similar to those of **8a** and **9a**, with the most notable difference being the shorter Sb─C_aryl_ bond distance [2.1455(10)Å/2.1566(14)Å] due to increased electron density of the aryl group in **11a**. Similarly, to unambiguously confirm the formation of **12**, its synthesis was attempted via the one‐electron oxidation of an ethereal solution of **4a** with ferrocenium tetrafluoroborate (FcBF_4_), successfully forging the target homobimetallic compound in 74% yield. Spectroscopic detection of Sb(II) dimer **12** when Sb(I) species **4a** is reacted with benzenediazonium tetrafluoroborate indicates this transformation begins with a single electron transfer, forming monomeric Sb(II) open‐shell species, together with phenyl radical. While recombination of monomeric Sb(II) open‐shell species with phenyl radicals results in oxidative addition product **11a**, we hypothesize that the formation of dimer **12** stems from the recombination of two monomeric Sb(II) open‐shell species (see Scheme S1). In addition, detection of benzene in the crude mixture after reaction of **4a** with benzenediazonium tetrafluoroborate also suggests the intermediacy of phenyl radical species, which are known to engage in hydrogen atom abstraction reactions with organic molecules.^[^ ^115^ ^]^ The presence of phenyl radicals was confirmed when the reaction of **4a** with benzenediazonium tetrafluoroborate is performed in the presence of bis(pinacolato)diboron, resulting in their interception and formation of phenyl pinacolborane in 77% yield (Figure S4).^[^ ^54^ ^]^ SC‐XRD analysis of species **12** (Scheme 4b) reveals that the dimer adopts a perfectly staggered *trans*‐conformation, which is typical of tetraorganyldipnictanes.^[^ ^116^, ^117^ ^]^ Furthermore, the Sb─Sb’ bond distance is unusually long [2.9798(5) Å] compared to tetraphenyldistibane (2.84 Å) and other distibane species,^[^ ^118^ ^]^ indicating steric clash of bis(NHC)borate ligand residues.^[^ ^119^ ^]^ This compound represents a unique example of carbene‐supported dimeric Sb(II)─Sb(II) species,^[^ ^120^ ^]^ and its formation suggests that well‐defined organostibinidene can engage in one‐electron processes with organic reagents, leading to productive bond‐forming reactivity.^[^ ^121^ ^]^ Noteworthy, Majumdar and co‐workers have recently reported the synthesis of a tetra‐cationic distibane species supported by bis(*α*‐iminopyridine) ligands.^[^ ^122^ ^]^ Interestingly, such compounds show a similarly long Sb─Sb’ bond distance [2.9978(6) Å] compared to **12**, and the ability to dissociate to monomeric stibinyl radical dications due to Coulombic repulsion. We speculate that in dimeric species **12**, the presence of the bis(NHC)borate ligand mitigates potential Coulombic repulsion between the two adjacent cationic Sb centres, rendering them resistant to dissociation.^[^ ^54^ ^]^ Additionally, SC‐XRD analysis of **12** reveals short intramolecular H─H contacts (<2.5 Å) between C─H bonds from the two ligands, suggesting that London dispersion interactions may also contribute to the stabilization of the dimeric form over the monomeric open‐shell species.^[^ ^123^ ^]^ Further studies on the electronic structure of **12** and the potential cleavage of the Sb─Sb’ bond in solution are currently under investigation in our laboratory.

### Stoichiometric Reductive Elimination From Sb(III) Centres

**Scheme 5:**
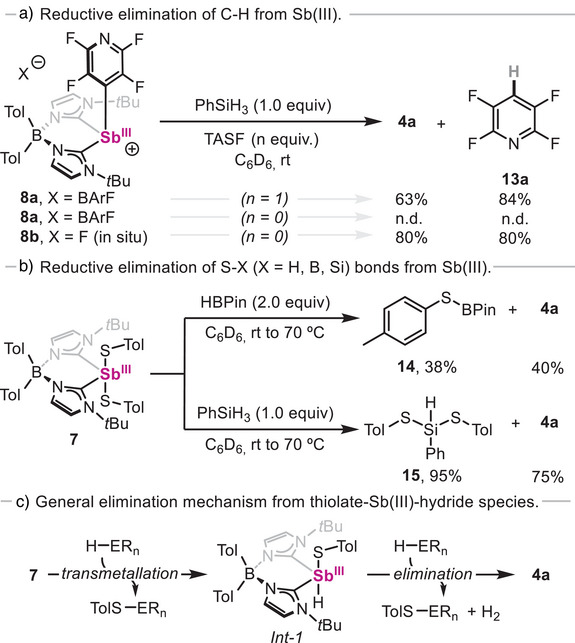
a) Reactivity of **8a** and **8b** towards reductive elimination with phenylsilane. b) Reactivity of **7** towards reductive elimination with hydride sources. c) General mechanism for reductive dehydrogenative coupling and formation of S─E (E═Si, B) bonds.

After evaluating the ability of stibinidene species **4a** to undergo stoichiometric oxidative addition, the possibility of Sb(III) compounds participating in reductive elimination to form new bonds was assessed. Such processes are rare and remain limited to H─H,^[^ ^9^, ^124^ ^]^ Te─Te,^[^ ^125^ ^]^ S─H, and S─B bond formation,^[^ ^18^ ^]^ being the formation of **4a** using hydride sources an example of the former (Scheme 2). Thus, an unprecedented C─H reductive elimination from Sb(III) centres was first studied from **8a** (Scheme 5a). Indeed, when **8a** was mixed with 1.0 equiv. of phenylsilane in the presence of tris(dimethylamino)sulfonium difluorotrimethylsilicate (TASF), reductive elimination was observed, resulting in the formation of 2,3,5,6‐tetrafluoropyridine **13a** and **4a** in 84% and 63% yield, respectively. In contrast, in absence of TASF reductive elimination does not proceed and **8a** remains unreacted. The importance of fluoride anions and their role on silane activation prior to hydride transmetallation was further demonstrated when the reaction was conducted from in situ generated **8b**, which showed formation of both **4a** and **13a** in 80% yield.

Reductive elimination was also investigated from dithiolate species **7** (Scheme 5b). Interestingly, reaction of **7** with 2.0 equiv. of pinacolborane was sluggish at 70 °C after several hours, resulting in the formation of pinBSTol **14** and **4a** in 38% and 40% yield, respectively, with only trace amounts of HSTol.^[^ ^54^ ^]^ This result, which suggests that S─H bond reductive elimination is not occurring, is in sharp contrast with the results reported for *N,C,N*‐pincer‐supported antimony species,^[^ ^18^ ^]^ where the formation of HSTol, pinBSTol and low‐valent Sb species in equal proportions is observed. Notably, when 1.0 equiv. of phenylsilane is mixed with **7**, complete conversion is observed at 70 °C after 1 h, yielding species **15** and **4a** in 95% and 75% yield, respectively. During the reaction, concomitant formation of dihydrogen was observed by ^1^H NMR (δ = 4.47 ppm in C_6_D_6_). In contrast to observations reported with *N,C,N*‐pincer‐supported antimony species, *p*‐toluenethiol was not detected after reaction completion. We rationalized that when using phenylsilane, the formation of two new S─Si bonds, along with dihydrogen and stibinidene **4a**, suggests a reductive dehydrogenative Si─S coupling (Scheme 5c). This constitutes a rare example of a main‐group‐mediated hydrogen evolution reaction involving silanes.

### Stibinidene‐Catalysed Redox Transformations

After demonstrating that zwitterionic stibinidene **4a** can undergo stoichiometric oxidative addition and reductive elimination, its application towards Sb(I)‐catalysed redox transformations was pursued. Sb presents a compelling platform for redox catalysis, a field that has only recently been explored in the hydroboration of disulfides.^[^ ^18^ ^]^ In addition, the broad organometallic reactivity exhibited by **4a** presents an opportunity to expand the catalytic scope of pnictinidenes beyond *N,C,N*‐pincer‐supported species in a broader range of catalytic transformations and explore the first C─X coupling catalysed by Sb. Building on the stoichiometric results obtained in the previous section, we set out to develop an Sb‐catalysed HDF of electron‐deficient arenes, leveraging the Sb(I)/Sb(III) redox cycle and the robustness of the zwitterionic species reported herein. After a brief optimization using pentafluoropyridine as substrate and phenylsilane as hydride source, a catalyst loading of 2 mol% of **4a** resulted in the formation of **13a** in quantitative yield under 2 h (Table 2), while when **2a** was used as catalyst no reactivity was observed. Noteworthy, **13a** was also obtained in quantitative yield when **5a** was used as catalyst, while employing sterically crowded stibinidene **4b** resulted in a moderate yield under similar reaction conditions. In addition, *N,C,N*‐pincer‐supported Sb(I) species afforded diminished yields under standard reaction conditions,^[^ ^54^ ^]^ highlighting the superior reactivity of zwitterionic species **4a** and underscoring their potential as viable alternative to conventional *N,C,N*‐pincer ligated Pn(I) platforms.

**Table 2:** Sb(I)‐catalysed HDF of polyfluorinated aromatic species.^a)^

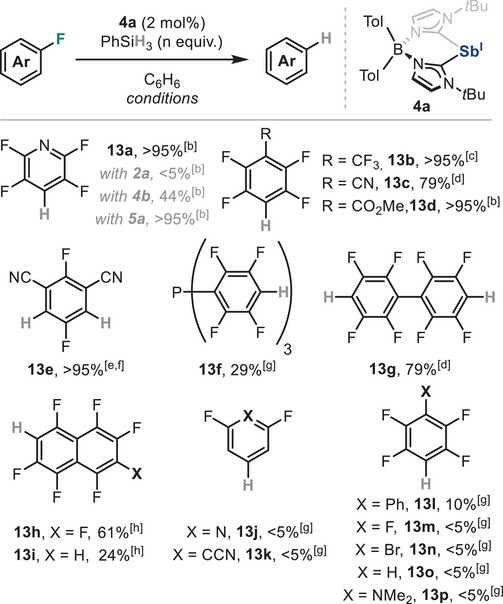

^a)^Yields calculated by quantitative ^19^F NMR using *α*,*α*,*α*‐trifluorotoluene as internal standard unless otherwise stated.

^b)^Reaction was run at rt for 2 h.

^c)^Reaction was run at rt for 4 h.

^d)^Reaction was run with 3.0 equiv. of PhSiH_3_ at 60 °C for 16 h.

^e)^Reaction was run with 2.0 equiv. of PhSiH_3_ at rt for 1 h.

^f)^Isolated yield after silica gel chromatography.

^g)^Reaction was run at 60 °C for 4 days.

^h)^Reaction was run with 2.0 equiv. of PhSiH_3_ at 60 °C for 2 days.

With the optimized reaction conditions in hand, a variety of electron‐deficient perfluoroarenes was submitted to the HDF protocol using **4a** as Sb(I) catalyst. As shown in Table 2, mono‐ and polyhydrodefluorination products **13a‐e** and **13g** were afforded with good to excellent yields under relatively mild reaction conditions, while **13f** was obtained in 29% yield under higher temperatures and larger reaction times. In the case of perfluoronaphthalene, a mixture of hydrodefluorinated isomers was obtained, consisting primarily of compounds **13h** and **13i** in 61% and 24% yield, respectively. In contrast to other heavy low‐valent p‐block‐mediated catalytic HDF reactions,^[^ ^16^, ^126^ ^]^ stibinidene **4a** was less active for the hydrodefluorination of less activated (hetero)arenes or other haloperfluorobenzenes, as products **13j‐p** were observed in low to traces amounts even under more forcing conditions. We hypothesize this is likely a consequence of the partial positive charge on the antimony atom in **4a**, which overall makes the pnictinidene centre slightly less nucleophilic compared to other neutral heavy main group species. Radical pathways were ruled out as formation of **13a** in presence of radical traps proceeded with identical yields to those observed in their absence (Table S3).^[^ ^54^ ^]^ Notably, fluoride anions were found to be essential for the catalytic reaction to proceed, in line with observations from stoichiometric reactivity studies, as the addition of Lewis acids acting as fluoride scavengers significantly reduced the yield of **13a** (Table S4).^[^ ^54^ ^]^ In addition, C(sp^3^)─F bond activation and the use of other organosilanes to forge other C(sp^2^)─X bonds [X═C(sp), C(sp^3^),N] resulted unfruitful (see Table S5), highlighting the limitations of the protocol.

**Scheme 6:**
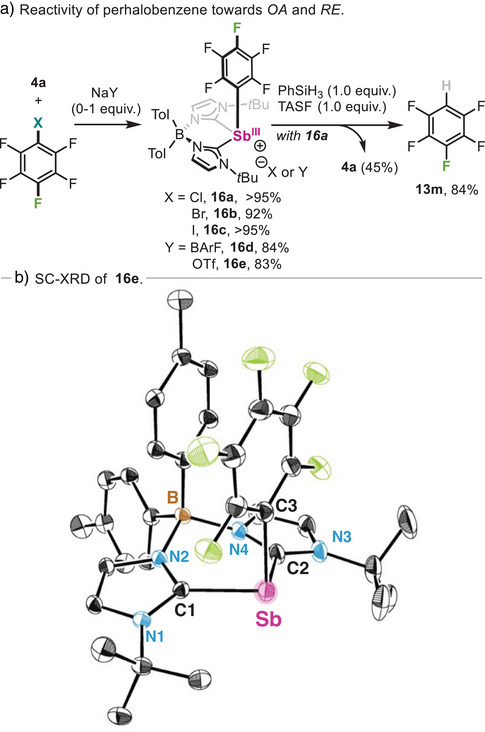
a) Reaction of stibinidene **4a** with halopentafluoroarenes and b) SC‐XRD structure of pentafluorophenyl stibine **16e**. Hydrogen atoms, anion, and solvent molecules are omitted for clarity and ellipsoids are displayed at 50% probability.

The lack of reactivity of bromoperfluorobenzene to obtain **13n** prompted us to gain further insight into its reactivity. When equimolar amounts of **4a** and chloro‐, bromo‐ or iodopentafluorobenzene were reacted at rt in benzene, rapid precipitation of complexes **16a‐c** was observed in quantitative yields (Scheme 6a). Unfortunately, the structure of halide‐containing compounds **16a‐c** could not be unambiguously confirmed by SC‐XRD experiments due to their intrinsic instability, leading to decomposition via protodemetallation with trace amounts of water.^[^ ^127^, ^128^ ^]^ However, NMR and HRMS experiments confirmed a rare C(sp^2^)─X (X═Cl, Br and I) oxidative addition event with a heavy main group species.^[^ ^102^ ^]^ Full characterization was achieved carrying out the reaction in presence of 1.0 equivalent of NaBArF or NaOTf, which resulted in thermally stable **16d** and **16e** in 84% and 83% yield, respectively. SC‐XRD analysis of **16e** (Scheme 6b) displays a long Sb─C_aryl_ bond distance in **16e**, with a value of 2.215(2) Å, compared to its non‐fluorinated analogue **11a**, which may be responsible of its facile decomposition via protodemetallation. The importance of fluoride anions in the reductive elimination of aryl─H bonds was further highlighted when **16a** was mixed with phenylsilane, undergoing reductive elimination only in presence of 1.0 equiv. of TASF (Scheme 6a). This stoichiometric protocol also overcomes the limited catalytic reactivity of **4a** with hexafluorobenzene (see Table 2), providing an effective Sb‐mediated method for the synthesis of pentafluorobenzene **13m**.

**Figure 5:**
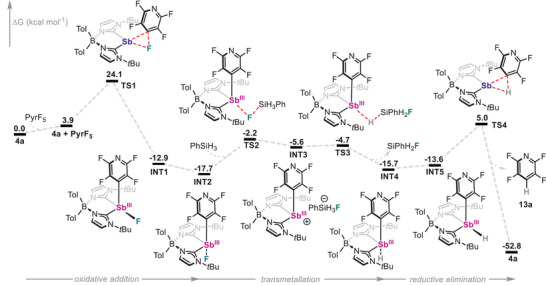
Gibbs energy (Δ*G*) for the reaction mechanism of the hydrodefluorination of pentafluoropyridine with PhSiH_3_ catalysed by **4a**. All calculations were performed at the CPCM(benzene)‐PBE0‐D3/def2‐TZVPP// CPCM(benzene)‐PBE0‐D3/def2‐SVP level of theory.

To gain deeper insight into the role of the **4a** in the redox‐catalysed hydrodefluorination reaction, DFT calculations were carried out on several plausible mechanistic pathways. To do so, the transformation of pentafluoropyridine with phenylsilane (PhSiH_3_) was selected as a representative case study. The mechanism with the lowest overall energy barrier is depicted in Figure 5, while alternative pathways displaying significantly larger activation energies are detailed in the Supporting Information (Figures S53–S55). The reaction begins with the oxidative addition of Sb(I) species **4a** to the C─F bond at the 4‐position of the pyridine ring, resulting in Sb(III) intermediate **INT1**. This step is thermodynamically favourable (Δ*G* = −12.9 kcal mol^−1^), with a connecting transition state (**TS1**) located at +24.1 kcal mol⁻¹ relative to the reactants, consistent with the need for mild heating and relatively long reaction times. After oxidative addition, **INT1** features the fluorine atom in the equatorial position and can undergo isomerization to **INT2**, in which the fluorine occupies an axial site. **INT2** is calculated to be 4.8 kcal mol^−1^ more stable than **INT1**. Subsequent ligand metathesis proceeds in a stepwise manner with the involvement of PhSiH_3_. Transmetallation begins with silane‐mediated fluoride abstraction, forming the fluorosilicate anion [PhSiH_3_F]^−^ and generating the corresponding cationic Sb(III) intermediate (**INT3**). This step is endergonic by +12.1 kcal mol^−1^ and proceeds via **TS2** with a barrier of +15.5 kcal mol^−1^. **INT3** is predicted to be a relatively high‐energy intermediate and rapidly evolves to a hydride species (**INT4**) by overcoming a low‐energy barrier (**TS3**) of only +0.9 kcal mol^−1^. The resulting **INT4**, with the hydride ligand in axial position, undergoes isomerization to **INT5**, placing the hydride in an equatorial site. Finally, C─H reductive elimination from **INT5** yields tetrafluoropyridine **13a** and regenerates the catalyst **4a**, with a barrier (**TS4**) of +18.6 kcal mol^−1^. Overall, the reaction is exergonic by −52.8 kcal mol^−1^, with the oxidative addition step identified as rate determining. Notably, the proposed mechanism, reminiscent of that reported for simple phosphines,^[^ ^102^ ^]^ accounts for the rapid C─H reductive elimination from **8a** and **16a** in the presence of both fluoride and PhSiH_3_ (Schemes 5a and 6a) and the experimentally observed crucial role of fluoride anions. It also rationalizes the observed instability of a putative Sb(III)─H intermediate, which could not be detected spectroscopically, and supports a mechanistic scenario involving an Sb(I)/Sb(III) redox cycle.

**Scheme 7:**
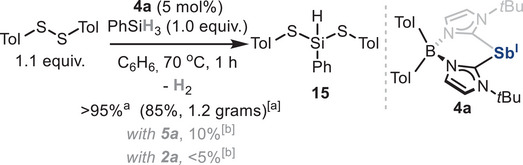
Dehydrogenative thiolation of silanes. a) Isolated yield after column chromatography. b) Yield determined by ^1^H NMR using 1,3,5‐trimethoxybenzene as internal standard.

After establishing the catalytic ability of stibinidene **4a** and based on the stoichiometric results depicted in Scheme 5b, we hypothesized a dehydrogenative thiolation of silanes could also be readily rendered catalytic. Thus, under the reaction conditions outlined in Scheme 7, phenylbis(*p*‐tolylthio)silane **15** was detected in almost quantitative yield by ^1^H NMR. Interestingly, using 5 mol% of bismuthinidene **5a** as the catalyst afforded compound **15** in only 10% yield due to catalyst decomposition, highlighting the greater stability and reactivity of Sb(I) species **4a** in this transformation. It is important to note that the use of Sb(III) species **2a** proved completely inactive. As the inherent moisture‐ and air‐sensitivity of silane **15** prevented its isolation on a small scale, a gram‐scale reaction was performed, obtaining 1.2 g (85% yield) of product after distillation, showing the robustness of the methodology. While similar reactions have been reported under transition‐metal and Lewis acid catalysis,^[^ ^129^ ^]^ this protocol contributes to the broader landscape of main‐group redox‐catalysed dehydrogenative coupling with silanes,^[^ ^130^, ^131^ ^]^ providing access to phenylbis(arylthiol)silanes—a class of compounds that remains largely unexplored and is not readily accessible through other dehydrogenative methods. This transformation may also find potential applications in on‐demand molecular hydrogen production.^[^ ^132^ ^]^ The mechanistic intricacies and broader substrate scope are currently under investigation.

## Conclusion

In this work, we have introduced a novel heavy pnictogen species stabilized by monoanionic bis(NHC)borate scaffolds. Their remarkable stability facilitates the isolation and characterization of zwitterionic Sb and Bi species in multiple oxidation states. Pnictinidene species exhibit unusually long Pn(I)─C_carbene_ lengths and DFT calculations reveal a moderate positive charge and two lone pairs at the pnictogen centres. Quantitative evaluation of the donor–acceptor interaction in **4a** and **5a** points towards strong *σ*‐donation and weak *π*‐accepting properties but with an overall stronger stabilizing effect than the one provided by cAAC ligands. The ability of zwitterionic stibinidene **4a** to undergo oxidative addition was investigated, resulting in oxidative addition to S─S, C(sp^3^)─X, and C(sp^2^)─X bonds. Furthermore, we have shown that the reaction with benzenediazonium tetrafluoroborate proceeds through a series of consecutive one‐electron transfer events, resulting in the isolation of dicationic distibane **12**. Reductive elimination from Sb(III) centres provided novel strategies for the stoichiometric construction of H─H, C─H, S─B, and S─Si bonds, and laid the foundation for the development of two proof‐of‐concept catalytic cycles based on the Sb(I)/Sb(III) redox couple, including a hydrodefluorination of polyfluoroarenes and a dehydrogenative thiolation of silanes. Overall, this work represents the first example of a formally cationic, low‐valent group 15 compound participating in redox catalysis, expanding the scope of heavy pnictogen redox catalysis beyond the constraints of conventional pincer scaffolds and offering a new approach in main group catalysis.

## Conflict of Interests

The authors declare no conflict of interest.

## Supporting information



Supporting Information

Supporting Information

## Data Availability

The data that support the findings of this study are available in the Supporting Information of this article.

## References

[1] T. Chu , G. I. Nikonov , Chem. Rev. 2018, 118, 3608–3680. 29558125 10.1021/acs.chemrev.7b00572

[2] L. Greb , Eur. J. Inorg. Chem. 2022, 2022, e202100871. 35910784 10.1002/ejic.202100871 PMC9306562

[3] P. P. Power , Nature 2010, 463, 171–177. 20075912 10.1038/nature08634

[4] C. Weetman , S. Inoue , ChemCatChem 2018, 10, 4213–4228.

[5] R. L. Melen , Science 2019, 363, 479. 30705183 10.1126/science.aau5105

[6] J. M. Lipshultz , G. Li , A. T. Radosevich , J. Am. Chem. Soc. 2021, 143, 1699–1721. 33464903 10.1021/jacs.0c12816 PMC7934640

[7] J. Abbenseth , J. M. Goicoechea , Chem. Sci. 2020, 11, 9728–9740. 34094237 10.1039/d0sc03819a PMC8162179

[8] S. Kundu , Chem. Asian J. 2020, 15, 3209–3224. 32794320 10.1002/asia.202000800

[9] P. Šimon , F. de Proft , R. Jambor , A. Růžička , L. Dostál , Angew. Chem. Int. Ed. 2010, 49, 5468–5471. 10.1002/anie.201002209 20602393

[10] C. Liu , Y. Dai , Q. Han , C. Liu , Y. Su , Chem. Commun. 2023, 59, 2161–2164. 10.1039/d2cc05736k 36727589

[11] C. L. Dorsey , R. M. Mushinski , T. W. Hudnall , Chem. ‐ Eur. J. 2014, 20, 8914–8917. 24925469 10.1002/chem.201403578

[12] R. Kretschmer , D. A. Ruiz , C. E. Moore , A. L. Rheingold , G. Bertrand , Angew. Chem. Int. Ed. 2014, 53, 8176–8179. 10.1002/anie.201404849 24961494

[13] D. Raiser , K. Eichele , H. Schubert , L. Wesemann , Chem. ‐ Eur. J. 2021, 27, 14073. 34291518 10.1002/chem.202102320 PMC8518042

[14] M. Wu , H. Li , W. Chen , D. Wang , Y. He , L. Xu , S. Ye , G. Tan , Chem 2023, 9, 2573.

[15] Y. Pang , N. Nöthling , M. Leutzsch , L. Kang , M. Van Gastel , E. Reijerse , R. Goddard , L. Wagner , D. Santalucia , S. Debeer , F. Neese , J. Cornella , Science 2023, 380, 1043–1048. 37200451 10.1126/science.adg2833

[16] Y. Pang , M. Leutzsch , N. Nöthling , F. Katzenburg , J. Cornella , J. Am. Chem. Soc. 2021, 143, 12487–12493. 34358426 10.1021/jacs.1c06735 PMC8377712

[17] F. Wang , O. Planas , J. Cornella , J. Am. Chem. Soc. 2019, 141, 4235–4240. 30816708 10.1021/jacs.9b00594 PMC6728098

[18] M. Huang , K. Li , Z. Zhang , J. Zhou , J. Am. Chem. Soc. 2024, 146, 20432–20438. 38981106 10.1021/jacs.4c05905

[19] Y. Pang , M. Leutzsch , N. Nöthling , J. Cornella , J. Am. Chem. Soc. 2020, 142, 19473–19479. 33146996 10.1021/jacs.0c10092 PMC7677929

[20] L. P. Griffin , T.‐N. Streit , R. Sievers , S. Aldridge , R. M. Gomila , A. Frontera , M. Malischewski , J. Am. Chem. Soc. 2024, 146, 29877–29882. 39423030 10.1021/jacs.4c11901 PMC11528405

[21] S. Ni , D. Spinnato , J. Cornella , J. Am. Chem. Soc. 2024, 146, 22140–22144. 39102564 10.1021/jacs.4c07262 PMC11328130

[22] T. Tsuruta , D. Spinnato , H. W. Moon , M. Leutzsch , J. Cornella , J. Am. Chem. Soc. 2023, 145, 25538–25544. 37963280 10.1021/jacs.3c10333 PMC10690797

[23] M. Mato , P. C. Bruzzese , F. Takahashi , M. Leutzsch , E. J. Reijerse , A. Schnegg , J. Cornella , J. Am. Chem. Soc. 2023, 145, 18742–18747. 37603853 10.1021/jacs.3c06651 PMC10472430

[24] J. Ramler , J. Schwarzmann , A. Stoy , C. Lichtenberg , Eur. J. Inorg. Chem. 2022, 2022, 1434. 10.1002/ejic.202100934 PMC9300068 35873275

[25] M. Kořenková , M. Hejda , M. Erben , R. Jirásko , R. Jambor , A. Růžička , E. Rychagova , S. Ketkov , L. Dostál , Chem. ‐ Eur. J. 2019, 25, 12884. 31353625 10.1002/chem.201902968

[26] V. Kremlacek , E. Kertesz , M. Erben , A. Ruzicka , R. Jambor , Z. Benko , L. Dostál , Chem. ‐ Eur. J. 2025, 31, e202404751. 39981902 10.1002/chem.202404751

[27] T. J. Hannah , S. S. Chitnis , Chem. Soc. Rev. 2024, 53, 764–792. 38099873 10.1039/d3cs00765k

[28] J. Abbenseth , J. Goicoechea , Chem. Sci. 2020, 11, 9728–9740. 34094237 10.1039/d0sc03819a PMC8162179

[29] D. Bawari , D. Toami , R. Dobrovetsky , Chem. Commun. 2025, 61, 5871–5882. 10.1039/d5cc00723b 40135433

[30] S. P. N. Gautam , S. Maji , K. Bhattacharyya , S. K. Mandal , J. Am. Chem. Soc. 2024, 146, 16743–16752. 10.1021/jacs.4c04376 38843466

[31] P. Palui , S. Ghosh , R. M. Gomila , G. Schnakenburg , A. Frontera , A. Bismuto , J. Am. Chem. Soc. 2025, 147, 1421–1426. 39772460 10.1021/jacs.4c15626

[32] P. Dabringhaus , A. Molino , R. J. Gilliard , J. Am. Chem. Soc. 2024, 146, 27186–27195. 39298432 10.1021/jacs.4c10834

[33] D. Meleschko , P. Palui , R. M. Gomila , G. Schnakenburg , A. C. Filippou , A. Frontera , A. Bismuto , Angew. Chem. Int. Ed. 2024, 63, e202405400. 10.1002/anie.202405400 38727609

[34] P. B. Deb , M. Majumdar , Chem. Asian J. 2022, 17, e202101133. 34786856 10.1002/asia.202101133

[35] P. K. Majhi , T. Sasamori , Chem. ‐ Eur. J. 2018, 24, 9441–9455. 29437260 10.1002/chem.201800142

[36] V. Kumar , R. G. Gonnade , C. B. Yildiz , M. Majumdar , Angew. Chem. Int. Ed. 2021, 60, 25522–25529. 10.1002/anie.202111339 34505340

[37] N. Mukherjee , V. Kumar , C. B. Yildiz , M. Majumdar , Inorg. Chem. 2024, 63, 24306–24312. 39661746 10.1021/acs.inorgchem.4c04257

[38] J. Zhou , H. Kim , L. L. Liu , L. L. Cao , D. W. Stephan , Chem. Commun. 2020, 56, 12953–12956. 10.1039/d0cc02710c 32985631

[39] M. M. Siddiqui , S. K. Sarkar , M. Nazish , M. Morganti , C. Köhler , J. Cai , L. Zhao , R. Herbst‐Irmer , D. Stalke , G. Frenking , H. W. Roesky , J. Am. Chem. Soc. 2021, 143, 1301–1306. 33434020 10.1021/jacs.0c12084

[40] X. Wang , B. Lei , Z. Zhang , M. Chen , H. Rong , H. Song , L. Zhao , Z. Mo , Nat. Commun. 2023, 14, 2968. 37221189 10.1038/s41467-023-38606-2 PMC10206093

[41] J. Xu , S. Pan , S. Yao , C. Lorent , C. Teutloff , Z. Zhang , J. Fan , A. Molino , K. B. Krause , J. Schmidt , R. Bittl , C. Limberg , L. Zhao , G. Frenking , M. Driess , J. Am. Chem. Soc. 2024, 146, 6025–6036. 38408197 10.1021/jacs.3c13016 PMC10921399

[42] I. V. Shishkov , F. Rominger , P. Hofmann , Organometallics 2009, 28, 3532.

[43] J. L. Martinez , S. A. Lutz , H. Yang , J. Xie , J. Telser , B. M. Hoffman , V. Carta , M. Pink , Y. Losovyj , J. M. Smith , Science 2020, 370, 356. 33060362 10.1126/science.abd3054

[44] Y. Gao , X. Li , J. E. Stevens , H. Tang , J. M. Smith , J. Am. Chem. Soc. 2023, 145, 11978–11987. 37227372 10.1021/jacs.2c13350

[45] Y. Gao , M. Pink , J. M. Smith , J. Am. Chem. Soc. 2022, 144, 1786–1794. 35076249 10.1021/jacs.1c11429

[46] Y. Gao , M. Pink , V. Carta , J. M. Smith , J. Am. Chem. Soc. 2022, 144, 17165–17172. 36070477 10.1021/jacs.2c07462

[47] Y. Xiong , S. Yao , T. Szilvási , E. Ballestero‐Martínez , H. Grützmacher , M. Driess , Angew. Chem. Int. Ed. 2017, 56, 4333–4336. 10.1002/anie.201701337 28295977

[48] Y. Xiong , T. Szilvási , S. Yao , G. Tan , M. Driess , J. Am. Chem. Soc. 2014, 136, 11300–11303. 25073089 10.1021/ja506824s

[49] V. Nesterov , D. Reiter , P. Bag , P. Frisch , R. Holzner , A. Porzelt , S. Inoue , Chem. Rev. 2018, 118, 9678–9842. 29969239 10.1021/acs.chemrev.8b00079

[50] C. Hu , L. L. Liu , Inorg. Chem. 2023, 62, 3592–3600. 36763989 10.1021/acs.inorgchem.2c04258

[51] J. W. Dube , C. L. B. Macdonald , P. J. Ragogna , Angew. Chem. Int. Ed. 2012, 51, 13026–13030. 10.1002/anie.201205744 23148013

[52] S. C. Kosnik , J. F. Binder , M. C. Nascimento , A. Swidan , C. L. B. Macdonald , Chem. ‐ Eur. J. 2019, 25, 1208–1211. 30468552 10.1002/chem.201805711

[53] J. C. Thomas , J. C. Peters , Inorg. Chem. 2003, 42, 5055. 12924877 10.1021/ic034150x

[54] See Supporting Information for Further Details.

[55] H. M. Weinert , Y. Schulte , A. Gehlhaar , C. Wölper , G. Haberhauer , S. Schulz , Chem. Commun. 2023, 59, 7755. 10.1039/d3cc01844j 37272311

[56] Deposition numbers 2423149 (for **1c**), 2423150 (for **2a**), 2423151 (for **3a**), 2423152 (for **4a**), 2423153 (for **4b**), 2423154 (for **5a**), 2423155 (for **6**), 2423156 (for **8a**), 2423157 (for **9a**), 2423159 (for **10b**), 2423158 (for **11a**), 2423160 (for **12**), for 2423161 (for **16b**) and 2423162 (for **16e**) contain the supplementary crystallographic data for this paper. These data are provided free of charge by the joint Cambridge Crystallographic Data Centre and Fachinformationszentrum Karlsruhe Access Structures service.

[57] A. Sidiropoulos , B. Osborne , A. N. Simonov , D. Dange , A. M. Bond , A. Stasch , C. Jones , Dalton Trans. 2014, 43, 14858–14864. 25166429 10.1039/c4dt02074j

[58] G. Wang , L. A. Freeman , D. A. Dickie , R. Mokrai , Z. Benkő , R. J. Gilliard Jr. , Inorg. Chem. 2018, 57, 11687. 30160485 10.1021/acs.inorgchem.8b01813

[59] M. S. M. Philipp , M. J. Krahfuss , K. Radacki , U. Radius , Eur. J. Inorg. Chem. 2021, 2021, 4007–4019.

[60] J. B. Waters , Q. Chen , T. A. Everitt , J. M. Goicoechea , Dalton Trans. 2017, 46, 12053–12066. 28766627 10.1039/c7dt02431b

[61] D. Bawari , I. Malahov , R. Dobrovetsky , Angew. Chem. Int. Ed. 2025, 64, e202419772. 10.1002/anie.202419772 39570789

[62] F. D. Henne , A. T. Dickschat , F. Hennersdorf , K.‐O. Feldmann , J. J. Weigand , Inorg. Chem. 2015, 54, 6849–6861. 26151669 10.1021/acs.inorgchem.5b00765

[63] L. P. Ho , A. Nasr , P. G. Jones , A. Altun , F. Neese , G. Bistoni , M. Tamm , Chem. ‐ Eur. J. 2018, 24, 18922–18932. 30357989 10.1002/chem.201804714

[64] P. Pykkö , J. Phys. Chem. A 2015, 119, 2326–2337. 25162610 10.1021/jp5065819

[65] A. Aprile , R. Corbo , K. V. Tan , D. J. D. Wilson , J. L. Dutton , Dalton Trans. 2013, 43, 764–768. 24150033 10.1039/c3dt52715h

[66] L. P. Ho , M. Tamm , Dalton Trans. 2021, 50, 1202–1205. 33480906 10.1039/d1dt00140j

[67] M. Mantina , A. C. Chamberlin , R. Valero , C. J. Cramer , D. G. Truhlar , J. Phys. Chem. A 2009, 113, 5806–5812. 19382751 10.1021/jp8111556 PMC3658832

[68] G. Wang , L. A. Freeman , D. A. Dickie , R. Mokrai , Z. Benkő , R. J. Gilliard Jr. , Chem. ‐ Eur. J. 2019, 25, 4335–4339. 30706565 10.1002/chem.201900458 PMC6593863

[69] S. Grimme , S. Ehrlich , L. Goerigk , J. Comput. Chem. 2011, 32, 1456–1465. 21370243 10.1002/jcc.21759

[70] S. Grimme , J. Antony , S. Ehrlich , H. Krieg , J. Chem. Phys. 2010, 132, 154104. 20423165 10.1063/1.3382344

[71] F. Weigend , R. Ahlrichs , Phys. Chem. Chem. Phys. 2005, 7, 3297. 16240044 10.1039/b508541a

[72] M. Ernzerhof , G. E. Scuseria , J. Chem. Phys. 1999, 110, 5029–5036.

[73] C. Adamo , V. Barone , J. Chem. Phys. 1999, 110, 6158.

[74] V. Bachler , G. Olbrich , F. Neese , K. Wieghardt , Inorg. Chem. 2002, 41, 4179–4193. 12160406 10.1021/ic0113101

[75] D. Herebian , E. Bothe , F. Neese , T. Weyhermuller , K. Wieghardt , J. Am. Chem. Soc. 2003, 125, 9116–9128. 15369369 10.1021/ja030123u

[76] F. Neese , J. Phys. Chem. Solids 2004, 65, 781.

[77] G. Parkin Valence , J. Chem. Educ. 2006, 83, 791.

[78] R. F. W. Bader , Chem. Rev. 1991, 91, 893.

[79] R. F. W. Bader , Atoms in Molecules: A Quantum Theory. Oxford University Press: Oxford, 1990.

[80] D. Cremer , E. Kraka , Angew. Chem. Int. Ed. Engl. 1984, 23, 627–628.

[81] M. Menéndez‐Herrero , J. Munárriz , E. Francisco , Á. M. Pendás , J. Chem. Phys. 2022, 156, 164103. 35489996 10.1063/5.0089438

[82] E. Ramos‐Cordoba , V. Postils , P. Salvador , J. Chem. Theory Comput. 2015, 11, 1501–1508. 26574361 10.1021/ct501088v

[83] P. Salvador , E. Ramos‐Cordoba , M. Montilla , L. Pujal , M. Gimferrer , J. Chem. Phys. 2024, 160, 172502. 38748009 10.1063/5.0206187

[84] I. Mayer , Chem. Phys. Lett. 2013, 585, 198–200.

[85] E. Ramos‐Cordoba , P. Salvador , I. Mayer , J. Chem. Phys. 2013, 138, 214107. 23758358 10.1063/1.4807775

[86] K. Morokuma , J. Chem. Phys. 1971, 55, 1236–1244.

[87] T. Ziegler , A. Rauk , Inorg. Chem. 1979, 18, 1755–1759.

[88] T. Ziegler , A. Rauk , Inorg. Chem. 1979, 18, 1558–1565.

[89] M. P. Mitoraj , A. Michalak , T. Ziegler , J. Chem. Theory Comput. 2009, 5, 962–975. 26609605 10.1021/ct800503d

[90] A. Michalak , M. Mitoraj , T. Ziegler , J. Phys. Chem. A 2008, 112, 1933–1939. 18266342 10.1021/jp075460u

[91] M. Mitoraj , A. Michalak , Organometallics 2007, 26, 6576–6580.

[92] L. Zhao , M. von Hopffgarten , D. M. Andrada , G. Frenking , WIREs Comput. Mol. Sci. 2018, 8, e1345.

[93] To compare [Pn(cAAC)_2_]^+^ species with 4a and 5a, the bis(cAAC) system was computed at the same level of theory. See Supporting Information for computational details.

[94] M. Gimferrer , S. Danes , E. Vos , C. B. Yildiz , I. Corral , A. Jana , P. Salvador , D. M. Andrada , Chem. Sci. 2022, 13, 6583–6591. 35756523 10.1039/d2sc01401g PMC9172369

[95] D. M. Andrada , N. Holzmann , G. Frenking , Can. J. Chem. 2016, 94, 1006–1014.

[96] Y. Pang , M. Leutzsch , N. Nöthling , J. Cornella , Angew. Chem. Int. Ed. 2023, 62, e202302071. 10.1002/anie.202302071 37265121

[97] B. Zhou , F. P. Gabbaï , J. Am. Chem. Soc. 2023, 145, 13758–13767. 37306561 10.1021/jacs.3c02223 PMC10863049

[98] D. Sarkar , P. Vasko , T. Gkuharev , L. P. Griffin , C. Bogle , J. Strujis , J. Tang , A. F. Roper , A. E. Crumpton , S. Aldridge , Angew. Chem. Int. Ed. 2024, 63, e202407427. 10.1002/anie.202407427 38775385

[99] H. J. Breunig , H. Jawad , J. Organomet. Chem. 1983, 243, 417–422.

[100] S. L. Benjamin , W. Levanson , G. Reid , R. P. Warr , Organometallics 2012, 31, 1025–1034.

[101] C. Ganesamoorthy , C. Wölper , L. Dostál , S. Schulz , J. Organomet. Chem. 2017, 845, 38–43.

[102] S. Bonfante , C. Lorber , J. M. Lynam , A. Simonneau , J. M. Slattery , J. Am. Chem. Soc. 2024, 146, 2005. 38207215 10.1021/jacs.3c10614 PMC10811696

[103] K. Chulsky , I. Malahov , D. Bawari , R. Dobrovetsky , J. Am. Chem. Soc. 2023, 145, 3786–3794. 36738474 10.1021/jacs.2c13318 PMC9936586

[104] C. R. Martinez , B. L. Iverson , Chem. Sci. 2012, 3, 2191.

[105] Q. Zhao , Q. Geng , Y. Li , J. Li , Z. Liu , Org. Chem. Front. 2023, 10, 1316–1321.

[106] A. Asthana , R. C. Srivastava , J. Organomet. Chem. 1989, 366, 281–285.

[107] J. Durkin , D. E. Hibbs , P. B. Hitchcock , M. B. Hursthouse , C. Jones , J. Jones , K. M. A. Malik , J. F. Nixon , G. Parry , J. Chem. Soc. Dalton Trans. 1996, 15, 3277.

[108] P. B. Hitchcock , C. Jones , J. F. Nixon , Angew. Chem. Int. Ed. Engl. 1995, 34, 492–493.

[109] R. Villazana , H. Sharma , F. Cervantes‐Lee , K. H. Pannell , Organometallics 1993, 12, 4278.

[110] Q. Lu , C. Yan , X.‐Q. Xiao , Z. Li , N. Wei , G. Lai , M. Kira , Organometallics 2017, 36, 3633–3637.

[111] A. Anaby , M. Feller , Y. Ben‐David , G. Leitus , Y. Diskin‐Posner , L. J. W. Shimon , D. Milstein , J. Am. Chem. Soc. 2016, 138, 9941–9950. 27400288 10.1021/jacs.6b05128

[112] T. C. Wiessner , S. A. Fosu , R. Parveen , N. P. Rath , B. Vlaisavljevich , W. B. Tolman , Organometallics 2020, 39, 3992.

[113] J. S. Quesnel , B. A. Arndtsen , J. Am. Chem. Soc. 2013, 135, 16841–16844. 24144068 10.1021/ja4098093

[114] K. S. Chan , C. M. Lau , Organometallics 2006, 25, 260–265.

[115] S. Sarkar , K. P. Shing Cheung , V. Gevorgyan , Chem. Sci. 2020, 11, 12974–12993. 34123240 10.1039/d0sc04881j PMC8163321

[116] F. Calderazzo , R. Poli , G. Pelizzi , J. Chem. Soc. Dalton Trans. 1984, 11, 2365.

[117] I.‐P. Lorenz , S. Rudolph , H. Piotrowski , K. Polborn , Eur. J. Inorg. Chem. 2005, 2005, 82–85.

[118] A. J. Ashe , Adv. Organomet. Chem 1990, 30, 77–97.

[119] S. Ishida , F. Hirakawa , K. Furukawa , K. Yoza , T. Iwamoto , Angew. Chem. Int. Ed. 2014, 53, 11172. 10.1002/anie.201405509 25066471

[120] R. J. Schwamm , M. P. Coles , Chem. ‐ Eur. J. 2019, 25, 14183–14191. 31452283 10.1002/chem.201903175

[121] T. Li , H. Wei , Y. Fang , L. Wang , S. Chen , Z. Zhang , Y. Zhao , G. Tan , X. Wang , Angew. Chem. Int. Ed. 2017, 56, 632. 10.1002/anie.201610334 27930850

[122] H. Haldar , S. Das , H. T. A. Wiedemann , K. Beuthert , C. W. M. Kay , S. Dehnen , C. B. Yildiz , M. Majumdar , J. Am. Chem. Soc. 2025, 147, 3140. 39818741 10.1021/jacs.4c12354

[123] D. Liptrot , P. Power , Nat. Rev. Chem. 2017, 1, 0004.

[124] I. Vránová , M. Alonso , R. Jambor , A. Růžička , J. Turek , L. Dostál , Chem. ‐ Eur. J. 2017, 23, 2340. 27943507 10.1002/chem.201604142

[125] P. Šimon , R. Jambor , A. Růžička , L. Dostál , Organometallics 2013, 32, 239–248.

[126] Z. Liu , Z. Wang , H. Mu , Y. Zhou , J. Zhou , Z. Dong , Nat. Commun. 2024, 15, 9849. 39537615 10.1038/s41467-024-54321-y PMC11561055

[127] M. Fild , O. Glemser , G. Christoph , Angew. Chem. Int. Ed. Engl. 1964, 3, 801.

[128] W. Tyrra , S. Aboulkacem , B. Hoge , W. Wiebe , I. Pantenburg , J. Fluor. Chem. 2006, 127, 213–217.

[129] K. Kuciński , G. Hreczycho , ChemCatChem 2017, 9, 1868.

[130] R. J. Schwamm , M. Lein , M. P. Coles , C. M. Fitchett , Chem. Commun. 2018, 54, 916–919. 10.1039/c7cc08402a 29318242

[131] J. Ramler , I. Krummenacher , C. Lichtenberg , Chem. ‐ Eur. J. 2020, 26, 14551–14555. 32573876 10.1002/chem.202002219 PMC7821184

[132] W.‐S. Han , T.‐J. Kim , S.‐K. Kim , Y. Kim , Y. Kim , S.‐W. Nam , S. O. Kang , Int. J. Hydrog. Energy 2011, 36, 12305–12312.

