# Supplementary material for: Zwitterionic Heavier Pnictinidenes in Redox Catalysis
Source: Angew Chem Int Ed Engl. 2025 Jun 30;64(34):e202505697. doi: 10.1002/anie.202505697 (PMC12363621; doi:10.1002/anie.202505697)
Supplement: Supplementary file 1 — Supporting Information [file ANIE-64-e202505697-s001.pdf]

# Supporting Information

# Zwitterionic Heavier Pnictinidenes in Redox Catalysis

Selwin Fernando,[a] Yi Chen Chan,[a,b] Sergio Fernandez,[a,b,*] Enric Sabater,[c,d] Graham Tizzard,[e] Simon J. Coles,[e] Diego M. Andrada,[c,*] Oriol Planas[a,b,*]

[a]School of Physical and Chemical Sciences, Department of Chemistry, Queen Mary University of London, Mile End Road, London, E1 4NS, UK.

[b]Department of Chemistry, Molecular Sciences Research Hub, Imperial College London, 82 Wood Lane, Shepherds Bush, London, W12 0BZ, UK.

[c]Department of Chemistry, Faculty of Natural Sciences and Technology, Saarland University, Saarbrücken, Germany

[d]Institut de Química Computacional i Catàlisi and Departament de Química, Universitat de Girona, C/ M. Aurèlia Capmany 69, 17003, Girona, Spain.

[e]EPSRC National Crystallography Service, School of Chemistry and Chemical Engineering, University of Southampton, Southampton SO17 1BJ, UK

*corresponding authors:
Dr Sergio Fernandez, s.fernandezgonzalez@imperial.ac.uk
Dr Diego M Andrada, diego.andrada@uni-saarland.de
Dr Oriol Planas, o.planas@imperial.ac.uk

TABLE OF CONTENTS


*1 General methods* ..... *5*

1.1 Experimental Methods ..... 5

*2 Ligand synthesis* ..... *7*

2.1 Synthesis of imidazolium chloride salts ..... 7

2.2 Synthesis of imidazolium hexafluorophosphate salt **1a** ..... 9

*3 Synthesis of Sb(III) and Bi(III) complexes* ..... *10*

3.1 Synthesis of Sb(III) complex **2a** ..... 10

3.2 Synthesis of Sb(III) complex **2b** ..... 11

3.3 Synthesis of Bi(III) complex **3a** ..... 12

3.4 Synthesis of Sb(III) complex **11b** ..... 13

3.5 Synthesis of Sb(III) complex **11a** ..... 14

*4 Synthesis of Sb(I) and Bi(I) complexes* ..... *15*

4.1 Synthesis of Sb(I) complex **4a** ..... 15

4.2 Synthesis of Sb(I) complex **4b** ..... 16

4.3 Synthesis of Bi(I) complex **5a** ..... 17

*5 Oxidative addition to Sb(I) complex 4a* ..... *18*

5.1 Reaction of 4a with methyl iodide ..... 18

5.2 Reaction of 4a with 4-tolyldisulfide ..... 19

5.3 Reaction of 4a with perfluoro(hetero)arene derivatives ..... 20

5.4 Reaction of 4a with phenyldiazonium tetrafluoroborate ..... 22

5.5 Reaction of 4a with 4-methoxybenzoyl chloride ..... 28

5.6 Reaction of 4a with (halo)pentafluorobenzene derivatives ..... 31

5.7 Reaction of 4a with (halo)pentafluorobenzene derivatives and $NaBAr^F_4$ ..... 36

5.8 Reaction of 4a with (halo)pentafluorobenzene derivatives and NaOTf ..... 39

5.9 Synthesis of Sb(II) dimer **12** ..... 40

*6 Reductive elimination from Sb(III) species* ..... *41*

6.1 Stoichiometric reductive elimination of H-H bonds ..... 41

6.2 Stoichiometric reductive elimination of C-H bonds from **8a** ..... 42

6.3 Stoichiometric reductive elimination of C-H bonds from **8b** ..... 44

6.4 Stoichiometric reduction of C-H bonds from **16a** ..... 46

6.5 Stoichiometric reductive elimination of Si-S bonds from **7** ..... 48

6.6 Stoichiometric reductive elimination of B-S bonds from **7** ..... 50

*7 Catalytic hydrodefluorination reactions* ..... *51*

7.1 General Method ..... 51

7.2 Optimisation of reaction conditions ..... 52

7.3 Results and spectroscopic data for hydrodefluorination products ..........................53
7.4 Radical trap tests for HDF reaction ........................................................................56
7.5 Radical trap test for Oxidative Addition to Perfluoropyridine ..................................57
7.6 Fluoride scavenging tests for HDF reaction ...........................................................59
7.7 Unsuccessful reagents for C-X/C-C couplings .......................................................60
*8 Dehydrogenative dithiolation of silanes* ....................................................................*61*
8.1 General method .........................................................................................................61
8.2 Optimisation of the Sb(I)-Sb(III) redox catalytic dehydrogenative dithiolation of silanes with *p*-tolyl disulfide ..........................................................................................61
8.3 Results and spectroscopic data for products from dehydrogenative thiolation ......63
*9 Crystallographic data* .................................................................................................*64*
9.1 Single crystal structure analysis of **1c** ......................................................................64
9.2 Single crystal structure analysis of **2a** ......................................................................75
9.3 Single crystal structure analysis of **3a** ......................................................................88
9.4 Single crystal structure analysis of **4a** ....................................................................100
9.5 Single crystal structure analysis of **4b** ....................................................................113
9.6 Single crystal structure analysis of **5a** ....................................................................127
9.7 Single crystal structure analysis of **6** ......................................................................140
9.8 Single crystal structure analysis of **8a** ....................................................................176
9.9 Single crystal structure analysis of **9a** ....................................................................201
9.10 Single crystal structure analysis of **10b** ..................................................................224
9.11 Single crystal structure analysis of **11a** ..................................................................240
9.12 Single crystal structure analysis of **12** ....................................................................257
9.13 Single crystal structure analysis of **16d** ..................................................................276
9.14 Single crystal structure analysis of **16e** ..................................................................300
*10 Computational details* ..............................................................................................*315*
10.1 Benchmark ..............................................................................................................317
10.2 Frontier Molecular Orbitals (FMO) ..........................................................................318
10.3 Natural Bond Orbitals (NBO) ...................................................................................319
10.4 Natural Resonance Theory (NRT) ...........................................................................321
10.5 Atoms in Molecule (AIM) .........................................................................................330
10.6 Effective Oxidation States (EOS) and Singlet-triplet Gaps ....................................332
10.7 Energy Decomposition Analysis (EDA) ...................................................................335
10.8 CASSCF calculations ...............................................................................................339
10.9 TD-DFT results .........................................................................................................340
10.10 Reaction profile of hydrodefluorination of pentafluoropyridine ..............................350
10.11 xyz coordinates (in Å) and Energies (in Hartree) ...................................................354

***11 NMR Characterisation data*** ........................................................................... *418*
11.1 NMR characterisation of imidazolium chloride ligands........................................ 418
11.2 NMR characterisation of imidazolium hexafluorophosphate salt **1a** .................. 428
11.3 NMR characterisation of Pn(III) complexes........................................................ 435
11.4 NMR characterisation of Pn(I) complexes........................................................... 462
11.5 NMR characterisation of oxidative addition products ........................................... 477
11.6 NMR characterisation of Sb(II) dimer **12**............................................................ 536
11.7 $^{19}$F NMR of crude reactions after Sb(I)-catalysed hydrodefluorination with phenylsilane ................................................................................................................ 542
11.8 Characterisation of dehydrogenative silylation product........................................ 551
***12 References***.................................................................................................. *556*

# 1 General methods

## 1.1 Experimental Methods

Unless otherwise stated, all manipulations were performed using standard Schlenk techniques under dry argon (Ar) in oven-dried glassware or in $N_2$-filled MBraun Unilab SP glovebox. Celite was dried at 150 °C under vacuum for 3 days and stored in the glovebox prior to use. Anhydrous solvents were distilled from appropriate drying agents, transferred under Ar and stored over activated 4Å molecular sieves (MS): tetrahydrofuran (Na-benzophenone), acetonitrile ($CaH_2$) and hexane ($CaH_2$). Diethyl ether, toluene and dichloromethane were collected from a MBraun SPS-800 solvent purification system under Ar and stored over activated 4Å MS. Deuterated solvents were stored over activated 4Å MS under Ar prior to use.

Commercially available antimony trichloride, bismuth tribromide, triphenylantimony, *p*-tolyl disulfide, and silver tetrafluoroborate were stored inside a $N_2$-filled glovebox and used as received. Sodium tetrakis[3,5-bis(trifluoromethyl)phenyl]borate was purchased from Fluorochem, washed with dry dichloromethane and dried under vacuum at 120 °C overnight prior to use. Octafluorotoluene, pentafluoropyridine and methyl pentafluorobenzoate were distilled and stored over activated 4Å molecular sieves. Phenylsilane, methyl iodide and all the other perfluorinated (hetero)arenes utilized in this work were obtained from Sigma-Aldrich and used without further purification. Phenylantimony dichloride,[1] phenyldiazonium tetrafluoroborate,[2] and ferrocenium tetrafluoroborate[3] were prepared according to reported procedures. Ferrocenium tetrafluoroborate was dried under vacuum at 120 °C overnight prior to use. N-*tert*-butylimidazole and 1*H*-1-mesitylimidazole,[4] and chlorodi-*p*-tolylborane[5] were synthesized according to reported procedures. For catalytic hydrodefluorination (HDF) processes, $\alpha,\alpha,\alpha$-trifluorotoluene was distilled under vacuum and stored over activated 4Å MS prior to be used as internal standard.

IR spectra were recorded using Bruker ALPHA FT-IR spectrometer under ambient conditions with 16 scans on a diamond ATR unit. NMR spectra were recorded using 300 MHz Bruker Avance III. $^{1}H$ NMR spectra (300.13 MHz, 500.1 Hz) were referenced to the residual protons of the corresponding deuterated solvent:[6] $CDCl_3$ (δ = 7.26 ppm), $C_6D_6$ (δ = 7.16 ppm), THF-$d_8$ (δ = 3.58 ppm) or $CD_3CN$ (δ = 1.94 ppm). $^{13}C\{^{1}H\}$ NMR spectra (75.47 MHz, 125 MHz) were referenced internally to the D-coupled $^{13}C$ resonances of the corresponding deuterated solvent:[6] $CDCl_3$ (δ = 77.16 ppm), $C_6D_6$ (δ = 128.06 ppm), THF-$d_8$ (δ = 67.21 ppm) or $CD_3CN$ (δ = 118.26 ppm). $^{19}F$ and $^{31}P$ NMR spectra are $^{1}H$ decoupled. Chemical shifts (δ) are given in ppm, relative to deuterated solvent residual peak, and coupling constants (*J*) provided in Hz. NMR data are represented according to the following structure: chemical shift, multiplicity (s = singlet, d = doublet, dd = doublet of doublet, t = triplet, q = quartet, hept = heptuplet, m =

multiplet, bs = broad singlet), coupling constants (Hz), integration and assigned atom (labelled according to the depicted structure).

Atmospheric Solid Probe Analysis coupled with Capillary Heated Electrospray Ionisation (ASAP-HESI) was used to acquire mass spectra at the University College London (UCL) Chemistry Mass Spectrometry Facility. The generated gas phase ions were measured using the Exactive Plus mass spectrometer. HESI parameters were the auxiliary gas to 5 and the sheath and sweep gases to 0 and the auxiliary gas heater to 400 °C and typical operating temperatures were between 100 °C and 450 °C but higher temperatures are needed for some compounds and lower more appropriate for others. The discharge voltage was set to between 3.5 and 4.0 kV to give a stable background ion signal would depend on the structure of analyte. Elemental analysis was performed at the London Metropolitan University using a ThermoFlash 2000 instrument.

# 2 Ligand synthesis

## 2.1 Synthesis of imidazolium chloride salts

Imidazolium salts **1a-c** were prepared following adaptations of the procedure reported by Hofmann and coworkers.[7]

### 2.1.1 Synthesis of imidazolium chloride salt **1c**

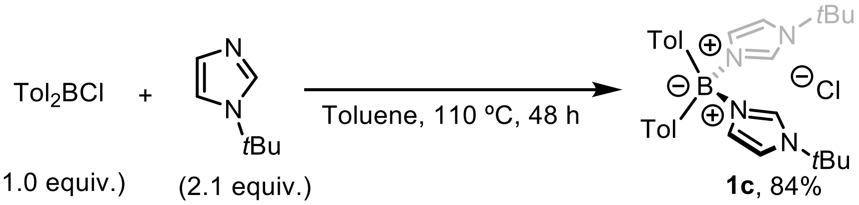


(1.0 equiv.) (2.1 equiv.) **1c**, 84%

To a solution of 1-(*tert*-butyl)-1*H*-imidazole (19.4 g, 2.1 equiv., 156.2 mmol) in toluene (150 mL) at 0 °C was added slowly a solution of chlorodi-*p*-tolylborane (17.0 g, 1.0 equiv., 74.4 mmol) in toluene (30 mL). The reaction mixture was stirred at rt for 15 min and then at 110 °C for 48 h. The white suspension formed was filtered and washed with toluene (2 × 40 mL) and *n*-hexane (3 × 40 mL). Residue was dried in vacuo to obtain the desired product **1c** (29.8 g, 74.4 mmol, 84 %) as a white solid. Crystals suitable for SC-XRD were obtained from a bilayer of ethyl acetate and *n*-hexane solvent system at rt under air.

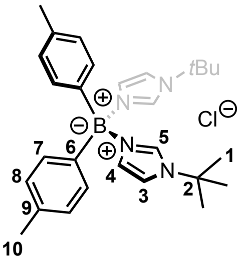


**$^1$H NMR – (400 MHz, CDCl$_3$) δ** = 8.19 (t, $J_{H\text{-}H}$ = 1.6 Hz, 2H, H-5), 7.60 (t, $J_{H\text{-}H}$ = 1.8 Hz, 2H, H-3), 7.14 (t, $J_{H\text{-}H}$ = 1.8 Hz, 2H, H-4), 7.09 (d, $J_{H\text{-}H}$ = 7.6 Hz, 4H, H-8), 6.97 (d, $J_{H\text{-}H}$ = 8.0 Hz, 4H, H-7), 2.30 (s, 6H, H-10), 1.65 (s, 18H, H-1).

**$^{11}$B NMR – (128 MHz, CDCl$_3$) δ =** 1.96 (bs).

**$^{13}$C NMR – (101 MHz, CDCl$_3$) δ =** 141.5 (2 × C, C-B, C-6), 136.9 (2 × C, C-9), 135.5 (2 × CH, C-5), 133.4 (4 × CH, C-7), 128.8 (4 × CH, C-8), 125.9 (2 × CH, C-4), 119.8 (2 × CH, C-3), 59.0 (2 × C, C-2), 30.3 (6 × $CH_3$, C-1), 21.2 (2 × $CH_3$, C-10). Carbon atoms connected to B could be identified analysing the cross peaks in the HMBC spectrum.

**HRMS (ASAP-HESI, m/z):** calculated for $C_{28}H_{38}BN_4^+$ $[M\text{-}Cl]^+$ 441.3184; found 413.2873. This m/z corresponds to the target molecule missing two methyl groups, with molecular formula (calculated m/z for $[M\text{-}C_2H_6\text{+}2H]^+$ = 413.2871). No other peaks were observed.

2.1.2 Synthesis of imidazolium chloride salt **1b**

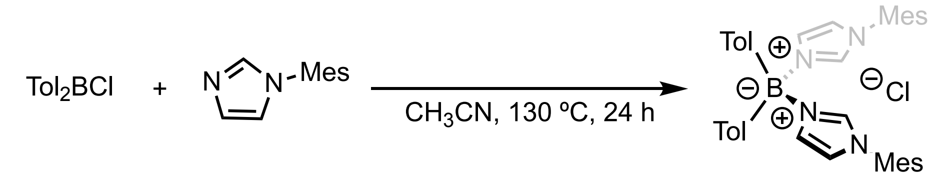


**1b**, 80%

To a mixture of 1-mesityl-1*H*-imidazole (2.57 g, 2.1 equiv., 13.8 mmol) and chlorodi-*p*-tolylborane (1.50 g, 1.0 equiv., 6.56 mmol) was added acetonitrile (20 mL). The resulting solution was stirred at 130 °C in a sealed J-Young Schlenk for 24 h. The solvent was then evaporated in vacuo, and diethyl ether (10 mL) was added resulting in the formation of a white precipitate. The solid material was collected by filtration and washed with dry diethyl ether (3 × 5 mL), and finally dried under vacuo to afford the desired product **1b** (3.14 g, 5.22 mmol, 80 %) as a white solid.

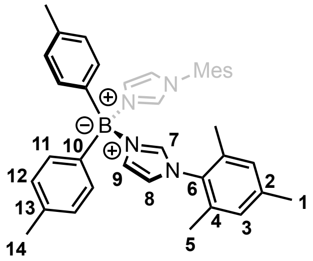


**$^{1}$H NMR – (400 MHz, CDCl$_3$) δ =** 7.99 (t, $J_{H\text{-}H}$ = 1.6 Hz, 2H, H-7), 7.70 (t, $J_{H\text{-}H}$ = 1.7 Hz, 2H, H-9), 7.58 (t, $J_{H\text{-}H}$ = 1.7 Hz, 2H, H-8), 7.11 (d, $J_{H\text{-}H}$ = 7.7 Hz, 4H, H-12), 7.01 (d, $J_{H\text{-}H}$ = 7.9 Hz, 4H, H-11), 6.96 (s, 4H, H-3), 2.30 (s, 6H, H-14), 2.29 (s, 6H, H-1), 2.06 (s, 12H, H-5).

**$^{11}$B NMR – (128 MHz, CDCl$_3$) δ =** 1.45 (bs).

**$^{13}$C NMR – (101 MHz, CDCl$_3$) δ =** 141.4 (2 × C, C-B, C-10), 140.9 (2 × C, C-6), 138.3 (2 × CH, C-7), 137.5 (2 × C, C-13), 134.5 (4 × C, C-4), 133.2 (4 × CH, C-11), 131.3 (2 × C, C-4), 129.7 (4 × CH, C-12), 129.1 (4 × CH, C-3), 126.8 (2 × CH, C-9), 124.4 (2 × CH, C-8), 21.2 (2 × CH$_3$, C-14), 21.1 (2 × CH$_3$, C-1), 17.7 (4 × CH$_3$, C-5). Carbon atoms connected to B could be identified analysing the cross peaks in the HMBC spectrum.

**HRMS (ASAP-HESI, m/z):** calc'd for $C_{38}H_{42}BClN_4$ [M-Cl]$^+$ 565.3497; found 565.3492.

## 2.2 Synthesis of imidazolium hexafluorophosphate salt 1a

### 2.2.1 Synthesis of imidazolium hexafluorophosphate salt **1a**

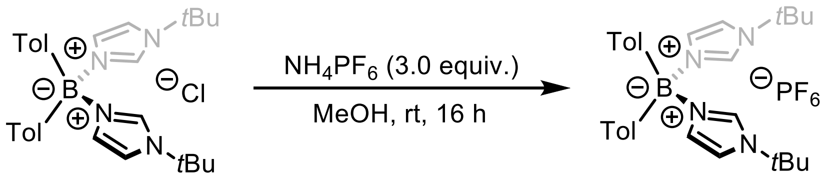


**1c** (1.0 equiv.) **1a**, 72%

This reaction was performed in the absence of inert atmosphere and using wet solvents as reported by Hofmann and coworkers.[7] To a solution of **1c** (29.8 g, 1.0 equiv., 62.5 mmol) in methanol (300 mL), ammonium hexafluorophosphate (30.5 g, 3.0 equiv., 186.5 mmol) was added in one portion. An off-white precipitate was immediately formed, and the suspension was stirred at rt overnight. The reaction mixture was filtered under vacuum washing with methanol (3 × 50 mL) and diethyl ether (2 × 50 mL). The residue was then dried in vacuo at 120 °C overnight to obtain **1a** (28.3 g, 62.5 mmol, 72%) as a white solid.

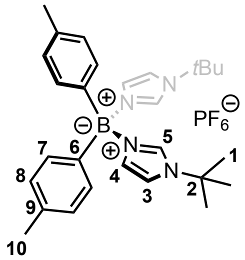


**$^1$H NMR – (400 MHz, $CDCl_3$) δ =** 7ii (t, $J_{H\text{-}H}$ = 1.6 Hz, 2H, H-5), 7.32 (t, $J_{H\text{-}H}$ = 1.8 Hz, 2H, H-3), 7.13 (d, $J_{H\text{-}H}$ = 8.1 Hz, 4H, H-8), 7.06 (t, $J_{H\text{-}H}$ = 1.7 Hz, 2H, H-4), 7.01 (d, $J_{H\text{-}H}$ = 7.9 Hz, 4H, H-7), 2.34 (s, 6H, H-10), 1.60 (s, 18H, H-1).

**$^{11}$B NMR – (128 MHz, $CDCl_3$) δ =** 2.05 (bs).

**$^{13}$C NMR – (101 MHz, $CDCl_3$) δ =** 141.6 (2 × C, C-B, C-6), 137.1 (2 × C, C-9), 134.8 (2 × CH, C-5), 133.6 (4 × CH, C-7), 128.9 (4 × CH, C-8), 125.9 (2 × CH, C-4), 119.5 (2 × CH, C-3), 59.0 (2 × C, C-2), 29.9 (6 × $CH_3$, C-1), 21.3 (2 × $CH_3$, C-10). Carbon atoms connected to B could be identified analysing the cross peaks in the HMBC spectrum.

**$^{19}$F NMR – (377 MHz, $CDCl_3$) δ =** -72.6 (d, *J* = 712.6 Hz, 6F, $PF_6$).

**$^{31}$P NMR** – **(162 MHz, $CDCl_3$) δ** = -144.5 (hept, *J* = 713.1 Hz, 1P, $PF_6$).

**HRMS (ASAP-HESI, m/z):** calc'd for $C_{28}H_{38}BN_4$ [M-$PF_6$]$^+$ 441.3195; found 441.3221.

## 3 Synthesis of Sb(III) and Bi(III) complexes

### 3.1 Synthesis of Sb(III) complex 2a

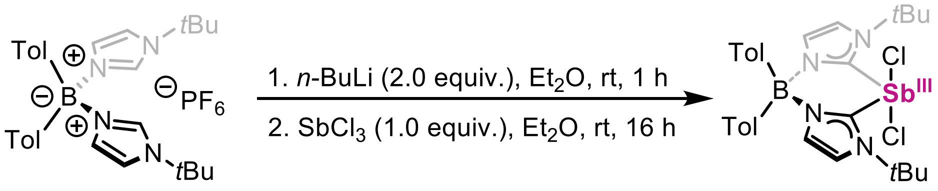


To a stirred solution of **1a** (2.50 g, 1.0 equiv., 4.26 mmol) in diethyl ether (50 mL) was added *n*-butyllithium (3.41 mL, 2.5 molar, 2.0 equiv., 8.53 mmol) at rt. The reaction mixture was stirred at that temperature for 1 h to form a clear yellow solution. A solution of antimony trichloride (0.98 g, 1.0 equiv., 4.31 mmol) in diethyl ether (15 mL) was then added slowly at rt to immediately form a white precipitate. The reaction mixture was stirred vigorously for at least 4 h. The precipitate was collected under Ar and washed with diethyl ether (3 × 15 mL) and *n*-hexane (3 × 15 mL). The product was extracted using dichloromethane (2 × 10 mL) to remove remaining LiCl. Removal of the solvent under vacuum afforded product **2a** (2.67 g, 4.20 mmol, 94 %) as a white solid. Crystals suitable for SC-XRD were grown from a dichloromethane solution of the title compound by slow vapour diffusion of diethyl ether at rt under air.

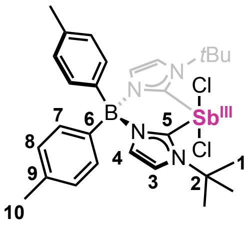

**$^{1}$H NMR – (400 MHz, CDCl$_3$) δ** = 7.22 – 7.13 (m, 6H, H-3 + H-8), 7.09 – 6.96 (m, 6H, H-4 + H-7), 2.29 (s, 6H, H10), 1.91 (s, 18H, H-1).

**$^{11}$B NMR – (128 MHz, CDCl$_3$) δ =** 4.15 (bs).

**$^{13}$C NMR – (101 MHz, CDCl$_3$) δ =** 153.5 (2 × C, C-Sb, C-5), 145.1 (2 × C, C-B, C-6), 136.1 (2 × C, C-9), 134.2 (4 × CH, C-7), 128.3 (4 × CH, C-8), 127.2 (2 × CH, C-3), 119.0 (2 × CH, C-4), 60.4 (2 × C, C-2), 32.1 (6 × CH$_3$, C-1), 21.4 (2 × CH$_3$, C-10). Carbon atoms connected to B could be identified analysing the cross peaks in the HMBC spectrum.

**HRMS (ASAP-HESI, m/z):** calc'd for $C_{28}H_{36}BClN_4Sb$ $[M-Cl]^+$ 595.1765; found 595.1768.

**CHN:** $C_{28}H_{36}BCl_2N_4Sb$ calcd. C 53.21, H 5.74, N 8.86 %; exp. C 53.27, H 5.41, N 8.77 %.

## 3.2 Synthesis of Sb(III) complex 2b

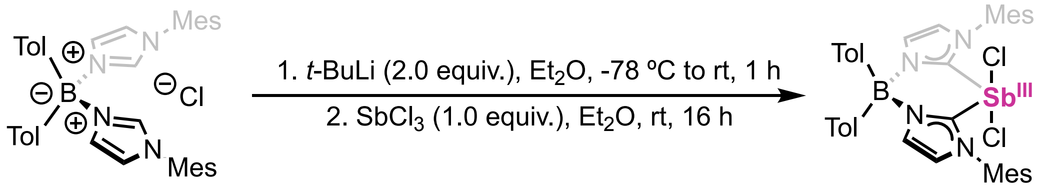


**1b** **2b,** 65%

To a suspension of **1b** (1.30 g, 1.0 equiv., 2.16 mmol) in diethyl ether (25 mL), a *tert*-butyllithium solution (2.54 mL, 1.7 molar in hexane, 2.0 equiv., 4.33 mmol) was added at -78 °C. The cooling bath was removed and the reaction mixture was stirred at rt for 1 h, forming an orangish-red solution with white precipitate. Salts were filtered off using a cannula filter. To the filtrate, antimony trichloride (0.49 g, 1.0 equiv., 2.16 mmol) in diethyl ether (10 mL) was added slowly and the reaction mixture was stirred at rt overnight. The precipitate was collected by filtration washing with diethyl ether (2 × 10 mL) and *n*-hexane (2 × 10 mL). The residue was suspended in dichloromethane, filtered through a celite plug, evaporated and dried in vacuo to obtain **2b** (1.07 g, 1.41 mmol, 65 %) as an off-white solid.

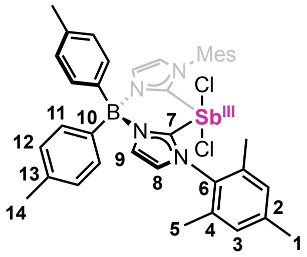


**$^{1}$H NMR – (400 MHz, CDCl$_3$) δ =** 7.38 (d, $J_{H\text{-}H}$ = 8.0 Hz, 2H, H-12), 7.28 (d, $J_{H\text{-}H}$ = 1.8 Hz, 2H, H-8), 7.14 (d, $J_{H\text{-}H}$ = 7.6 Hz, 2H, H-11), 7.00 (d, $J_{H\text{-}H}$ = 1.7 Hz, 2H, H-9), 6.96 (s, 2H, H-3), 2.35 (s, 6H, H-14), 2.31 (s, 6H, H-1), 2.22 (s, 12H, H-5).

**$^{11}$B NMR – (128 MHz, CDCl$_3$) δ =** 4.69 (bs).

**$^{13}$C NMR – (101 MHz, CDCl$_3$) δ =** 156.5 (2 × C, C-Sb, C-7), 144.5 (2 × C, C-B, C-10), 140.6 (2 × C, C-6), 137.1 (4 × C, C-4), 136.2 (2 × C, C-13), 134.7 (4 × CH, C-12), 132.3 (2 × C, C-2), 129.7 (4 × CH, C-3), 128.9 (4 × CH, C-11), 127.9 (2 × CH, C-8), 121.7 (2 × CH, C-9), 21.3 (2 × CH$_3$, C-14), 20.9 (2 × CH$_3$, C-1), 19.1 (4 × CH$_3$, C-5). Carbon atoms connected to B and Sb could be identified analysing the cross peaks in the HMBC spectrum.

**HRMS (ASAP-HESI, m/z):** calc'd for $C_{38}H_{40}BClN_4Sb$ [M-Cl]$^+$ 719.2067; m/z not found

**CHN:** $C_{38}H_{40}BCl_2N_4Sb$ calcd. C 60.35, H 5.33 N 7.41 %; exp. C 60.44, H 4.98, N 7.71 %.

## 3.3 Synthesis of Bi(III) complex 3a

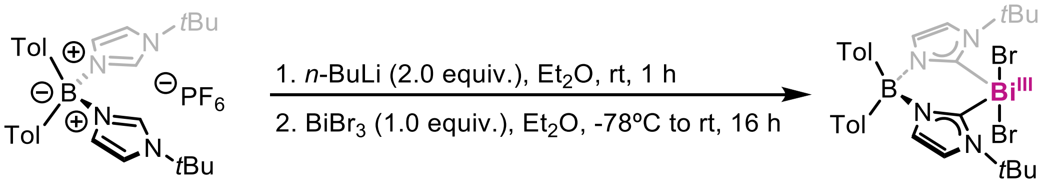


**1a** **3a**, 73%

To a stirred suspension of **1a** (0.45 g, 1.0 equiv., 0.77 mmol) in diethyl ether (10 mL), *n*-butyllithium (0.098 g, 2.5 molar in *n*-hexane, 2.0 equiv., 1.50 mmol) was added dropwise. The reaction mixture was stirred for an additional 1 h at rt to give a pale-yellow solution. After cooling down to -78 °C, a solution of bismuth tribromide (0.34 g, 1.0 equiv., 0.77 mmol) in diethyl ether (5 mL) was added slowly. The cooling bath was then removed, and the reaction mixture was allowed to stir while warming to rt for overnight. After completion, the mixture was filtered and the solid was extracted with dichloromethane (3 × 5mL). The filtrate was concentrated under vacuo. An off-white solid was precipitated when *n*-hexane was added, which was then filtered and dried under vacuum to afford complex **3a** (0.50 g, 1.2 mmol, 73%). Crystals suitable for SC-XRD were grown from a dichloromethane/pentane bilayer solution of the title compound at rt under air.

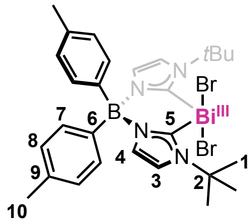

**$^{1}$H NMR – (400 MHz, CDCl$_3$) δ =** 7.28 (d, $J_{H\text{-}H}$ = 1.9 Hz, 2H, H-3), 7.15 (d, $J_{H\text{-}H}$ = 1.9 Hz, 2H, H-4), 7.08 (d, $J_{H\text{-}H}$ = 8.1 Hz, 4H, H-7), 7.04 (d, $J_{H\text{-}H}$ = 8.0 Hz, 4H, H-8), 2.30 (s, 6H, H-10), 1.86 (s, 18H, H-1).

**$^{11}$B NMR – (128 MHz, CDCl$_3$) δ =** 0.75 (bs).

**$^{13}$C NMR – (101 MHz, CDCl$_3$) δ =** 175.0 (2 × C, C-Bi, C-5), 145.2 (2 × C, C-B, C-6), 136.0 (2 × C, C-9), 134.0 (4 × CH, C-7), 129.3 (2 × CH, C-4), 128.3 (4 × CH, C-8), 120.5 (2 × CH, C-3), 60.4 (2 × C, C-2), 31.6 (6 × CH$_3$, C-1), 21.3 (2 × CH$_3$, C-10). Carbon atoms connected to B and Bi could be identified analysing the cross peaks in the HMBC spectrum.

**HRMS (ASAP-HESI, m/z):** calc'd for $C_{28}H_{37}BBiBrN_4$ [M+H]$^+$ 807.12764; found 807.1256.

**CHN:** $C_{28}H_{36}BBr_2N_4Bi$ calcd. C 41.61, H 4.49 N 6.93 %; exp. C 41.94, H 4.75, N 5.55 %.

### 3.4 Synthesis of Sb(III) complex 11b

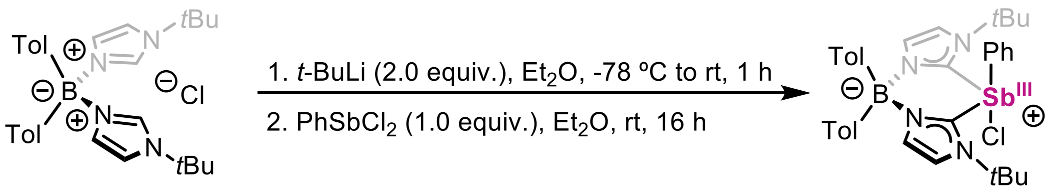


**1a** **11b**, 33%

To a suspension of **1c** (0.48 g, 1.0 equiv., 1.00 mmol) in dry diethyl ether (10 mL), a *tert*-butyllithium solution (1.05 mL, 1.9 molar in hexane, 2.0 equiv., 2.00 mmol) was added at -78 °C. The cooling bath was removed and the reaction mixture was stirred at rt for 1 h, forming an orangish solution with white precipitate. Phenyldichlorostibane (0.27 g, 1.0 equiv., 1.00 mmol) in diethyl ether (2.5 mL) was added slowly and the reaction mixture was stirred at rt overnight. The precipitate was collected by filtration washing with diethyl ether (2 × 5 mL) and *n*-hexane (2 × 5 mL). The solid was resuspended in dichloromethane, filtered through a celite plug, evaporated and dried in vacuo to obtain **11b** (0.22 g, 0.33 mmol, 33 %) as an off-white solid.

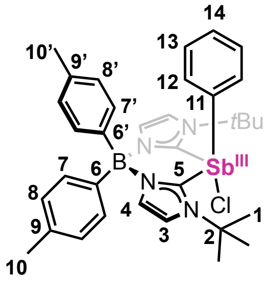


**$^{1}$H NMR – (400 MHz, $CD_3CN$) δ =** 7.66 (d, $J_{H\text{-}H}$ = 1.9 Hz, 2H, H-3), 7.26 – 7.19 (m, 1H, H-14), 7.13 (dd, $J_{H\text{-}H}$ = 8.0, 1.5 Hz, 2H, H-13), 7.06 (d, $J_{H\text{-}H}$ = 7.7 Hz, 4H, H-8 + H-12), 7.00 – 6.94 (m, 4H, H-4 + H-8'), 6.79 (d, $J_{H\text{-}H}$ = 7.9 Hz, 2H, H-7), 6.76 (d, $J_{H\text{-}H}$ = 7.9 Hz, 2H, H-7'), 2.29 (s, 3H, H-10), 2.28 (s, 3H, H-10'), 1.60 (s, 18H, H-1).

**$^{11}$B NMR – (128 MHz, $CDCl_3$) δ =** 1.71 (bs).

**$^{13}$C NMR – (101 MHz, $CD_3CN$) δ =** 147.3 (2 × C, C-Sb, C-5), 147.2 (C, C-B, C-6) 144.8 (C, C-B, C-6'), 140.3 (C, C-Sb, C-11), 137.8 (2 × CH, C-13), 137.4 (C, C-9), 137.2 (C, C-9'), 134.5 (2 × CH, C-7), 133.8 (2 × CH, C-7'), 130.5 (CH, C-14), 130.3 (2 × CH, C-12), 129.4 (2 × CH, C-8), 129.3 (2 × CH, C-8'), 129.2 (2 × CH, C-4), 122.8 (2 × CH, C-3), 61.5 (2 × C, C-2), 31.6 (6 × $CH_3$, C-1), 21.3 ($CH_3$, C-10), 21.2 ($CH_3$, C-10'). Carbon atoms connected to B and Sb could be identified analysing the cross peaks in the HMBC spectrum.

**HRMS (ASAP-HESI, m/z):** calc'd for $C_{34}H_{41}BN_4Sb^+$ $[M\text{-}Cl]^+$ 637.2457; found 637.2474

## 3.5 Synthesis of Sb(III) complex 11a

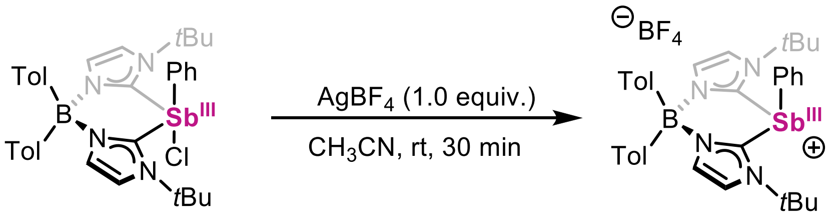


**11b** **11a**, >99%

To a solution of **11b** (67 mg, 1.0 equiv., 0.10 mmol) in acetonitrile (1 mL), a solution of silver tetrafluoroborate (20 mg, 1.0 equiv., 0.10 mmol) in acetonitrile (1 mL) was added dropwise at rt. The solution was stirred at this temperature for 30 min, observing the precipitation of AgCl. The pale orangish suspension was filtered through a celite plug and dried in vacuo to afford **11a** (72 mg, 0.10 mmol, >99 %) as a pale orange solid. Crystals suitable for SC-XRD were grown at -35 °C from a saturated diethyl ether solution of the title compound under inert atmosphere.

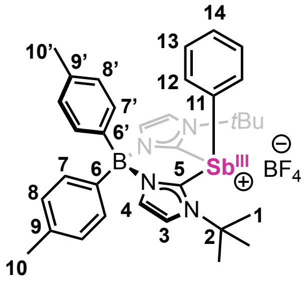


**$^{1}$H NMR – (400 MHz, $CD_3CN$) δ =** 7.63 (d, $J_{H-H}$ = 1.9 Hz, 2H, H-3), 7.36 – 7.28 (m, 1H, H-14), 7.22 – 7.15 (m, 4H, H-12 + H-13), 7.08 (d, $J_{H-H}$ = 7.6 Hz, 2H, H-8), 7.07 – 6.99 (m, 4H, H-4 + H-8'), 6.84 (d, $J_{H-H}$ = 7.9 Hz, 2H, H-7), 6.62 (d, $J_{H-H}$ = 7.9 Hz, 2H, H-7'), 2.31 (s, 6H, H-10 + H-10'), 1.54 (s, 18H, H-1).

**$^{11}$B NMR – (128 MHz, $CDCl_3$) δ =** 3.09 (bs, $BTol_2$), -1.18 (s, $BF_4$)

**$^{13}$C NMR – (101 MHz, $CD_3CN$) δ =** 147.2 (C, C-B, C-6), 146.3 (2 × C, C-Sb, C-5), 142.9 (C, C-B, C-6'), 138.0 (2 × CH, C-13), 137.9 (C, C-Sb, C-11), 137.7 (2 × C, C-9 + C-9'), 134.2 (2 × CH, C-7), 133.9 (2 × CH, C-7'), 131.2 (CH, C-14), 130.8 (2 × CH, C-12), 129.7 (2 × CH, C-8), 129.5 (2 × CH, C-8'), 129.4 (2 × CH, C-4), 123.3 (2 × CH, C-3), 61.8 (2 × C, C-2), 31.3 (6 × $CH_3$, C-1), 21.2 (2 × $CH_3$, C-10 + C-10'). Carbon atoms connected to B and Sb could be identified analysing the cross peaks in the HMBC spectrum.

**$^{19}$F NMR – (377 MHz, $CDCl_3$) δ =** -151.76 (s), -151.81 (s). The signals correspond to the F atoms attached to $^{10}$B and $^{11}$B isotopes respectively.

**HRMS (ASAP-HESI, m/z):** calc'd for $C_{34}H_{41}BN_4Sb^+$ $[M-BF_4]^+$ 637.2457; found 637.2448

## 4 Synthesis of Sb(I) and Bi(I) complexes

### 4.1 Synthesis of Sb(I) complex 4a

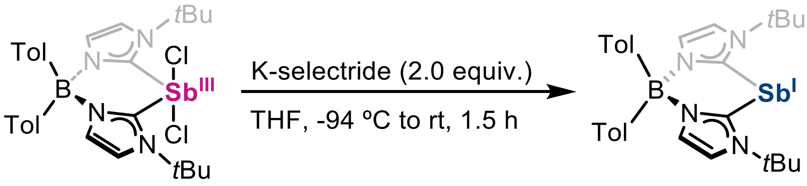


**2a** **4a**, 84%

A solution of **2a** (2.50 g, 1.0 equiv., 3.96 mmol) in THF (10 mL) was cooled down to -94 °C in an acetone/$N_2$(l) cooling bath. A solution of K-Selectride (7.91 mL, 1.0 molar in THF, 2.0 equiv., 7.91 mmol) was then added dropwise to the reaction mixture. After stirring at -94 °C for 15 minutes, the solution was warmed to rt whilst stirring for a further 1.5 h. The solvent was then evaporated in vacuo and diethyl ether (20 mL) was added to the reaction mixture. The insoluble species were removed by filtration to obtain an orange solution. After evaporation of the solvent in vacuo, the residue was washed with minimal amounts of hexane (3 × 4 mL), resulting in a yellow solid corresponding to **4a** (1.87 g, 3.33 mmol, 84 %). Crystals suitable for SC-XRD were grown from a saturated solution in hexane at rt under inert atmosphere.

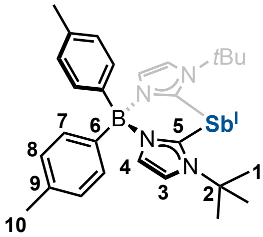


**$^1$H NMR – (400 MHz, $C_6D_6$, ppm) δ** = 7.30 (d, $J_{H-H}$ = 7.6 Hz, 4H, H-7), 7.20 (d, $J_{H-H}$ = 7.7 Hz, 4H, H-8), 6.92 (d, $J_{H-H}$ = 2.0 Hz, 2H, H-4), 6.58 (d, $J_{H-H}$ = 2.0 Hz, 2H, H-3), 2.26 (s, 6H, H-10), 1.28 (s, 18H, H-4).

**$^{11}$B NMR – (128 MHz, $C_6D_6$) δ =** 2.37 (bs).

**$^{13}$C NMR – (101 MHz, $C_6D_6$, ppm) δ =** 155.8 (2 × C, C-Sb, C-5), 146.2 (2 × C, C-B, C-6), 135.2 (2 × C, C-9), 135.1 (4 × CH, C-7), 128.4 (4 × CH, C-8), 126.7 (2 × CH, C-4), 117.4 (2 × CH, C-3), 57.6 (2 × C, C-2), 29.0 (6 × $CH_3$, C-1), 21.5 (2 × $CH_3$, C-10). Carbon atoms connected to B and Sb could be identified analysing the cross peaks in the HMBC spectrum.

**$^{19}$F NMR – (377 MHz, $CDCl_3$) δ =** -151.76 (s), -151.81 (s). The signals correspond to the F atoms attached to $^{10}$B and $^{11}$B isotopes respectively.

**HRMS (ASAP-HESI, m/z):** calc'd for $C_{28}H_{37}BN_4Sb$ $[M+H]^+$ 561.2144; found 561.2125

**CHN:** $C_{28}H_{36}BN_4Sb$ calcd. C 59.93, H 6.47 N 9.98 %; exp. C 59.89, H 6.96, N 9.71 %.

**$\lambda_{max}$ (UVvis, benzene):** 335 nm (ε = 3.1 x $10^2$ $M^{-1}$ $cm^{-1}$), 400 nm (ε = 1.7 x $10^2$ $M^{-1}$ $cm^{-1}$).

### 4.2 Synthesis of Sb(I) complex 4b

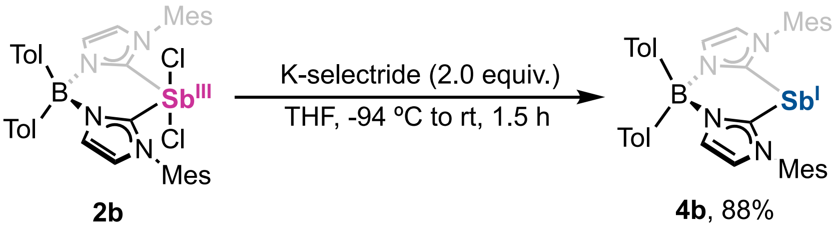


A solution of **2b** (1.07 g, 1.0 equiv., 1.41 mmol) in THF (30 mL) was cooled down to -94 °C in an acetone/$N_2$(l) cooling bath. A solution K-Selectride (2.84 mL, 1 molar in THF, 2.0 equiv., 2.84 mmol) was then added dropwise to the reaction mixture. After stirring at -94 °C for 15 minutes, the solution was warmed to rt whilst stirring for a further 1.5 h. After completion, the solvent was evaporated in vacuo and diethyl ether (20 mL) was added to the reaction mixture. The insoluble species were removed by filtration to obtain an orange solution. After evaporation of the solvent in vacuo, the residue was washed with minimal amounts of hexane (3 × 4 mL), resulting in a yellow solid corresponding to **4b** (0.85 g, 1.25 mmol, 88 %). Crystals suitable for SC-XRD were obtained by slow evaporation of a tetrahydrofuran-diethyl ether solution at rt under inert atmosphere.

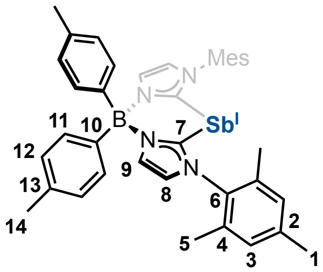


**$^{1}$H NMR – (400 MHz, $C_6D_6$, ppm) δ =** 7.45 (d, $J_{H\text{-}H}$ = 7.5 Hz, 4H, H-11), 7.25 (d, $J_{H\text{-}H}$ = 7.5 Hz, 4H, H-12), 7.06 (d, $J_{H\text{-}H}$ = 1.8 Hz, 2H, H-9), 6.53 (s, 4H, H-3), 6.22 (d, $J_{H\text{-}H}$ = 1.9 Hz, 2H, H-8), 2.31 (s, 6H, H-14), 1.98 (s, 12H, H-5), 1.94 (s, 6H, H-1).

**$^{11}$B NMR – (128 MHz, $C_6D_6$) δ =** 2.81 (bs).

**$^{13}$C NMR – (101 MHz, $C_6D_6$, ppm) δ =** 160.3 (2 × C, C-Sb, C-7), 146.0 (2 × C, C-B, C-10), 139.2 (2 × C, C-2), 135.7 (4 × C, C-4), 135.4 (2 × C, C-6), 134.8 (4 × CH, C-11), 134.1 (2 × C, C-13), 129.7 (4 × CH, C-3), 128.4 (2 × CH, C-9), 128.4 (4 × CH, C-12), 119.7 (2 × CH, C-8), 21.5 (2 × $CH_3$, C-14), 21.0 (2 × $CH_3$, C-1), 18.2 (4 × $CH_3$, C-5). Carbon atoms connected to B and Sb could be identified analysing the cross peaks in the HMBC spectrum.

**HRMS (ASAP-HESI, m/z):** calc'd for $C_{38}H_{41}BN_4Sb$ $[M+H]^+$ 684.2379; found 684.2380

**CHN:** $C_{38}H_{40}BN_4Sb$ calcd. C 66.60, H 5.88 N 8.18 %; exp. C 66.12, H 6.22, N 8.03 %.

## 4.3 Synthesis of Bi(I) complex 5a

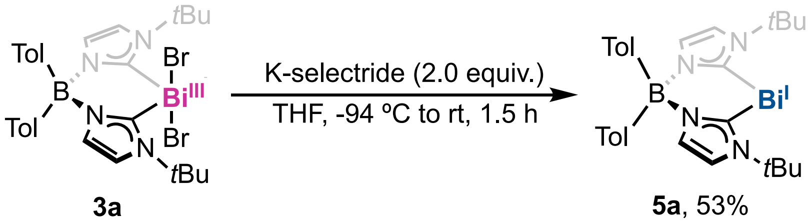

A solution of **3a** (0.40 g, 1.0 equiv., 0.50 mmol) in THF (20 mL) was cooled down to -94 °C in an acetone/$N_2$(l) cooling bath. A solution of K-Selectride (1.0 mL, 1 molar in THF, 2.0 equiv., 1.00 mmol) was then added dropwise to the reaction mixture. After stirring at -94 °C for 15 minutes, the solution was warmed to rt whilst stirring for a further 1.5 h. The solvent was then evaporated in vacuo and diethyl ether (10 mL) was added to the reaction mixture. The insoluble species were removed by filtration to obtain an orangish-red solution. After evaporation of the solvent in vacuo, the residue was washed with minimal amounts of cold *n*-hexane (3 × 2 mL), resulting in an orange-brown solid corresponding to **5a** (0.17 g, 0. 27 mmol, 53%). Crystals suitable for SC-XRD were grown from a saturated solution in *n*-hexane at rt under inert atmosphere.

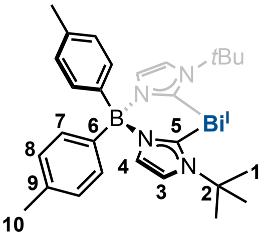

**$^1$H NMR – (400 MHz, $C_6D_6$) δ =** 7.30 (d, $J_{H\text{-}H}$ = 7.9 Hz, 4H, H-7), 7.19 (d, $J_{H\text{-}H}$ = 7.4 Hz, 4H, H-8), 7.06 (d, $J_{H\text{-}H}$ = 1.9 Hz, 2H, H-4), 6.69 (d, $J_{H\text{-}H}$ = 1.9 Hz, 2H, H-3), 2.26 (s, 6H, H-10), 1.17 (s, 18H, H-1).

**$^{11}$B NMR – (128 MHz, $C_6D_6$) δ =** 2.96 (bs).

**$^{13}$C NMR – (101 MHz, $C_6D_6$) δ =** 168.1 (2 × C, C-Bi, C-5), 147.0 (2 × C, C-B, C-6) 135.7(4 × CH, C-7), 135.0 (2 x C, C-9), 130.1 (2 x CH, C-4), 127.9 (4 × CH, C-8) 118.5 (2 × CH, C-3), 57.2 (2 × C, C-2), 30.7 (6 × $CH_3$, C-1), 21.5 (2 × $CH_3$, C-10). Carbon atoms connected to B and Bi could be identified analysing the cross peaks in the HMBC spectrum.

**HRMS (ASAP-HESI, m/z):** calc'd for $C_{28}H_{36}BBiN_4$ $[M]^+$ 648.2831; found 648.2829

**CHN:** $C_{28}H_{36}BN_4Bi$ calcd. C 51.87, H 5.60 N 8.64 %; exp. C 51.47, H 5.77, N 8.03 %.

**$\lambda_{max}$ (UVvis, benzene):** 330 nm (ε = 4.5 x $10^2$ $M^{-1}$ $cm^{-1}$), 85 nm (ε = 1.8 x $10^2$ $M^{-1}$ $cm^{-1}$).

## 5 Oxidative addition to Sb(I) complex 4a

### 5.1 Reaction of 4a with methyl iodide

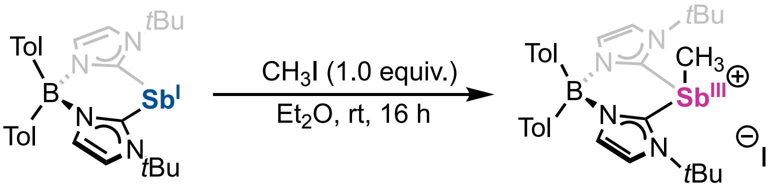


To a stirred solution of **4a** (56 mg, 1.0 equiv., 0.10 mmol) in diethyl ether (2 mL) was added methyl iodide (14 mg, 1.0 equiv., 0.10 mmol) at rt, resulting in the precipitation of an off-white solid. After stirring overnight at the same temperature, the precipitate was collected by filtration and washed with diethyl ether (3 × 5 mL) to obtain complex **6** (67 mg, 0.095 mmol, 95%) as a white solid. Single crystal suitable for SC-XRD was obtained employing a bilayer of dichloromethane /pentane at rt under air.

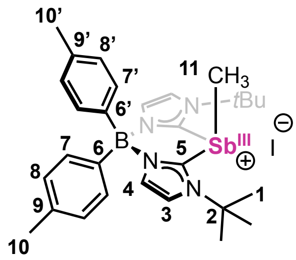

**$^{1}$H NMR – (400 MHz, CDCl$_3$) δ** = 7.38 (d, $J_{H\text{-}H}$ = 1.9 Hz, 2H, H-3), 7.14-7.03 (m, 4H, H-7 + H-8), 6.99 – 6.87 (m, 4H, H-4 + H-8'), 6.30 (d, $J_{H\text{-}H}$ = 7.5 Hz, 2H, H-7'), 2.31 (s, 3H, C-10), 2.26 (s, 3H, H-10'), 1.85 (s, 18H, H-1), 0.57 (s, 3H, H-11).

**$^{11}$B NMR – (128 MHz, CDCl$_3$) δ =** 2.82 (bs).

**$^{13}$C NMR – (101 MHz, CDCl$_3$) δ =** 146.9 (C, C-B, C-6') 143.5 (2 × C, C-Sb, C-5), 143.5 (C, C-B, C-6) 137.2 (C, C-9), 136.0 (C, C-9'), 135.6 (2 x CH), 130.6 (2 × CH, C-7'), 128.8 (2 × CH), 128.8 (2 × CH), 128.7 (2 x C-H, C-4), 120.2 (2 × CH, C-3), 60.3 (2 × C, C-2), 32.2 (6 × CH$_3$, C-11), 21.4 (CH$_3$), 21.2 (CH$_3$, C-10'), 2.7 (CH$_3$, H$_3$C-Sb, C-11). Carbon atoms connected to B and Sb could be identified analysing the cross peaks in the HMBC spectrum

**HRMS (ASAP-HESI, m/z):** calc'd for $C_{29}H_{39}BN_4Sb^+$ [M-I]+ 575.2300; found 575.2304

## 5.2 Reaction of 4a with 4-tolyldisulfide

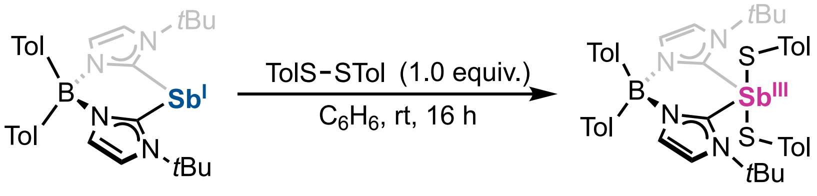


To a stirred solution of **4a** (0.10 g, 1.0 equiv., 0.18 mmol) in benzene (2 mL) was added *p*-tolyl disulfide (46 mg, 1.0 equiv., 0.18 mmol) at rt, and the reaction mixture was stirred overnight. After solvent evaporation, the residue was washed with *n*-hexane (3 × 5 mL) to obtain **7** (94 mg, 0.12 mmol, 65 %) as a yellow solid.

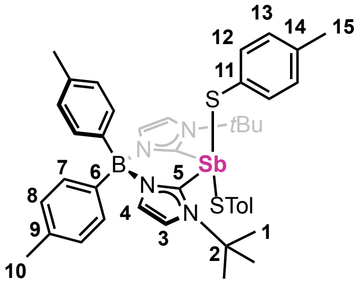


**$^1$H NMR – (400 MHz, THF-*d*$_8$) δ =** 7.45 (d, $J_{H\text{-}H}$ = 1.9 Hz, 2H, H-3), 7.07 (d, $J_{H\text{-}H}$ = 7.7 Hz, 4H, H-8), 7.05 (d, $J_{H\text{-}H}$ = 8.0 Hz, 4H, H-12), 6.96 (d, $J_{H\text{-}H}$ = 7.7 Hz, 4H, H-7), 6.93 (d, $J_{H\text{-}H}$ = 1.9 Hz, 2H, H-4), 6.82 (d, $J_{H\text{-}H}$ = 7.8 Hz, 4H, H-13), 2.25 (s, 6H, H-10), 2.20 (s, 6H, H-15), 1.68 (s, 18H, H-1).

**$^{11}$B NMR – (128 MHz, CDCl$_3$) δ =** 2.76 (bs)

**$^{13}$C NMR – (101 MHz, THF-*d*$_8$) δ =** 151.9 (2 × C, C-Sb, C-5), 147.4 (2 × C, C-B, C-6), 141.9 (2 × C, C-11), 135.8 (2 × C, C-9), 135.0 (4 × CH, C-8), 134.5 (4 × CH, C-12), 132.9 (2 × C, C-14), 129.6 (4 × CH, C-13), 128.8 (4 × CH, C-7), 128.6 (2 × CH, C-4), 120.3 (2 × CH, C-3), 61.1 (2 × C, C-2), 31.8 (6 × $CH_3$, C-1), 21.6 ($CH_3$, C-10), 21.1 ($CH_3$, C-15). Carbon atoms connected to B and Sb could be identified analysing the cross peaks in the HMBC spectrum.

**HRMS (ASAP-HESI, m/z):** calc'd for $C_{35}H_{43}BN_4SSb^+$ [M-STol]$^+$ 683.2334; found 683.2338

## 5.3 Reaction of 4a with perfluoro(hetero)arene derivatives

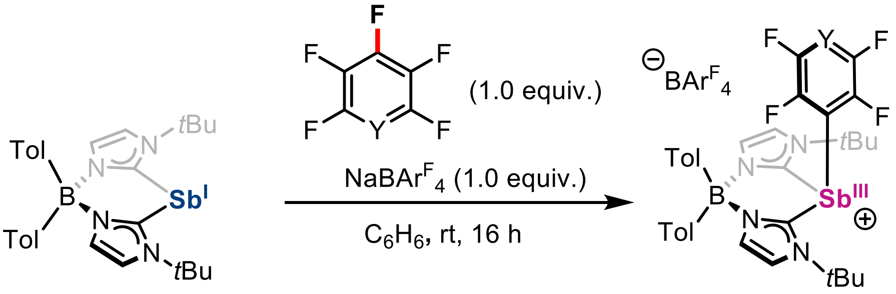


**4a**

Y = N, **8a**, 94%

Y = $CCO_2Me$, **9a**, 83%

To a stirred solution of **4a** (56 mg, 1.0 equiv., 0.10 mmol) and sodium tetrakis(3,5-bis(trifluoromethyl)phenyl)borate (89 mg, 1.0 equiv., 0.10 mmol) in benzene (2 mL) was added perfluoropyridine (17 mg, 1.0 equiv., 0.10 mmol) at rt. After stirring overnight, the reaction mixture was filtered and dried in vacuo to obtain complex **8a** (0.15 g, 0.094 mmol, 94%) as a white solid. Crystals suitable for SC-XRD were obtained from a bilayer of dichloromethane and *n*-pentane solvent system at rt under inert atmosphere.

Characterisation data for **8a**

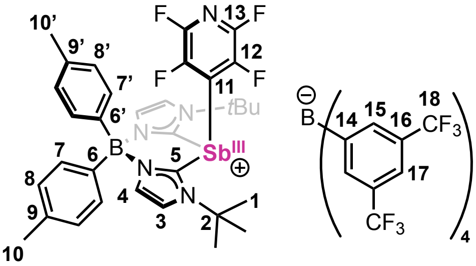

**$^1$H NMR – (400 MHz, $CDCl_3$) δ =** 7.70 (bs, 8H, H-15), 7.51 (bs, 4H, H-17), 7.49 (d, $J_{H\text{-}H}$ =1.9 Hz, 2H, H-3), 7.18 (d, $J_{H\text{-}H}$ = 1.9 Hz, 2H, H-4), 7.06 (d, $J_{H\text{-}H}$ = 7.5 Hz, 2H, H-8), 6.76 (d, $J$ = 7.5 Hz, 2H, H-8’), 6.76 (d, $J_{H\text{-}H}$ = 7.5 Hz, 2H, H-7) 6.33 (d, $J_{H\text{-}H}$ = 7.5 Hz, 2H, H-7’), 2.31 (s, 3H, H-10), 2.18 (s, 3H, H-10’), 1.76 (s, 18H, H-1).

**$^{11}$B NMR – (128 MHz, $CDCl_3$) δ =** 4.22 (bs, $BTol_2$), -6.65 (s, BArF)

**$^{13}$C NMR – (101 MHz, $CDCl_3$) δ =** 161.8 (q, $J_{C\text{-}B}$ = 49.7 Hz, 4 × C, $BAr^F{}_4$, C-14), 142.2 (C-B, C-6), 141.4 (C-B, C-6’), 138.5 (2 x C, C-Sb, C-5) , 138.1 (C, C-9), 138.0 (C, C-9’), 134.9 (bs, 8 × CH, $BAr^F{}_4$, C-15), 134.9 (2 x CH, C-7), 131.3 (2 × CH, C-4), 131.0 (2 × CH, C-7’), 129. (qq, $J_{C\text{-}F}$ = 30.3, 2.9 Hz, 8 × C, $BAr^F{}_4$, C-16), 129.2 (2 × CH, C-8), 128.2 (2 × CH, C-8’), 124.7 (q, $J_{C\text{-}F}$ = 272.5 Hz, 8 × C, $BAr^F{}_4$, C-18), 122.7 (2 × CH, C-3), 117.6 (hept, $J_{C\text{-}F}$ = 3.8 Hz, 4 × CH, $BAr^F{}_4$, C-17), 62.4 (2 × C, C-2), 31.9 (6 × $CH_3$, C-1), 21.3 ($CH_3$, C-10), 20.8 ($CH_3$, C-10’). Carbon atoms connected to B and Sb could be identified analysing cross peaks in the $^1$H-$^{13}$C HMBC spectrum. Peaks at 134.9 ppm could be identified in the $^1$H-$^{13}$C HSQC spectrum. Carbons of tetrafluoropyridyl moeity directly bonded to fluorines could not be detected due to a complex coupling pattern. <u>*Unambiguous structural determination was achieved by SC-XRD (see section 9.8).*</u>

**$^{19}$F NMR – (377 MHz, $CDCl_3$) δ =** -62.3 (s, 24F, F-18), -88.6 (m, 2F, F-12), -124.5 (m, 2F, F-13)

**HRMS (ASAP-HESI, m/z):** calc'd for $C_{33}H_{36}BF_4N_5Sb$ $[M+H]^+$ 710.2033; found 710.2048

To obtain the analogous complex **9a** (0.14 g, 0.083 mmol, 83%) as a white solid, the same protocol was followed using methyl 2,3,4,5,6-pentafluorobenzoate (23 mg, 1.0 equiv., 0.10 mmol) instead of perfluoropyridine. Crystals suitable for SC-XRD were obtained from a bilayer of tetrahydrofuran and *n*-pentane solvent system at rt under inert atmosphere.

Characterisation data for **9a**

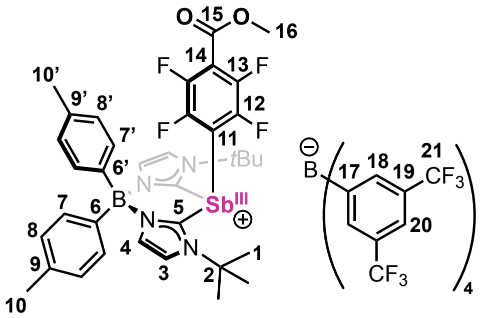


**$^{1}$H NMR – (400 MHz, $CDCl_3$) δ =** 7.65 – 7.54 (m, 8H, H-17), 7.41 (bs, 4H, H-19), 7.36 (d, *J* = 1.9 Hz, 2H, H-3), 7.07 (d, *J* = 1.9 Hz, 2H, H-4), 6.93 (d, J = 7.7 Hz, 2H, H-8), 6.73 (d, *J* = 7.8 Hz, 2H, H-8'), 6.51 (d, *J* = 7.8 Hz, 2H, H-7), 6.37 (d, *J* = 7.6 Hz, 2H, H-7'), 3.84 (s, 3H, H-15), 2.19 (s, 3H, H-10), 2.07 (s, 3H, H-10'), 1.61 (s, 18H, H-1).

**$^{13}$C NMR – (101 MHz, $CDCl_3$) δ =** 161.8 (q, $J_{C\text{-}B}$ = 49.7 Hz, 4 × C, $BAr^F_4$, C-17), 158.7 (C, C=O, C-15), 143.3 (C-B, C-6), 140.3 (C-B, C-6'), 139.5 (2 × C, C-Sb, C-5), 138.4 (C, C-9), 137.5 (C, C-9'), 134.9 (bs, 8 × CH, $BAr^F_4$, C-18), 134.4 (2 × CH, C-7), 131.4 (2 × CH, C-7'), 131.0 (2 × CH, C-4), 129.0 (qq, $J_{C\text{-}F}$ = 30.3, 2.9 Hz, 8 × C, $BAr^F_4$, C-19), 128.9 (2 × CH, C-8), 128.3 (2 × CH, C-8'), 124.7 (q, $J_{C\text{-}F}$ = 272.5 Hz, 8 × C, $BAr^F_4$, C-21), 122.3 (2 × CH, C-3), 117.6 (hept, $J_{C\text{-}F}$ = 3.8 Hz, 4 × CH, $BAr^F_4$, C-20), 62.0 (2 × C, C-2), 53.5 ($CH_3$, C-16), 31.8 (6 × $CH_3$, C-1), 21.2 ($CH_3$, C-10), 21.0 ($CH_3$, C-10'). Carbon atoms connected to B and Sb and carboxyl carbon could be identified analysing cross peaks in $^{1}$H-$^{13}$C HMBC. Peaks at 128.9 ppm could be identified in $^{1}$H-$^{13}$C HSQC. Carbons of tetrafluoroaryl moiety directly bonded to fluorine (C-11, C-12, C-13 and C-14) could not be detected due to a complex coupling pattern. *Unambiguous structural determination was achieved by SC-XRD (see section 9.9).*

**$^{11}$B NMR – (128 MHz, $CDCl_3$) δ =** 2.54 (bs, $BTol_2$), -6.64 (s, BArF)

**$^{19}$F NMR (377 MHz, $CDCl_3$) δ =** -62.34 (s, 24F, F-20), -121.55 (m, 2F, F-12), -135.01 (m, 2F, F-13).

**HRMS (ASAP-HESI, m/z):** calc'd for $C_{36}H_{43}BN_4O_2Sb^+$ $[M\text{-}BAr^F_4]^+$ 767.2135; found 767.2153

## 5.4 Reaction of 4a with phenyldiazonium tetrafluoroborate

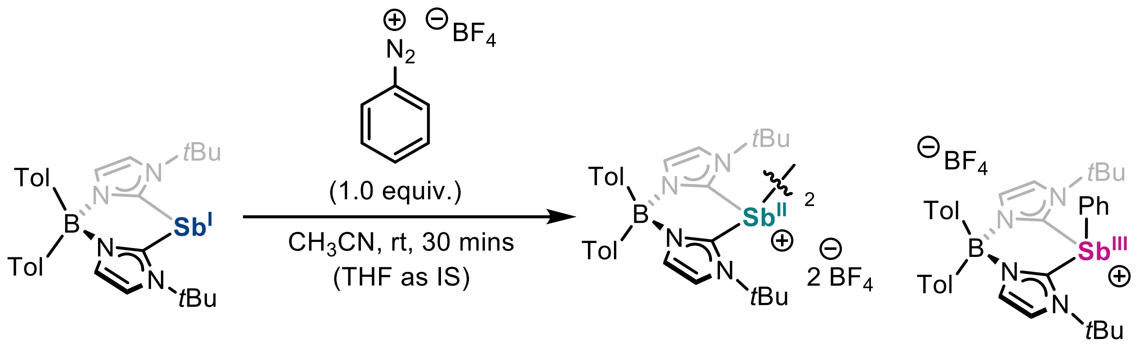


**4a** **12**, >90% **11a**, <10%

In two separate flasks under Ar, a solution of **4a** (14 mg, 1.0 equiv., 0.025 mmol) in dry $CD_3CN$ (0.25 mL) and a solution of phenyldiazonium tetrafluoroborate (5 mg, 1.0 equiv., 0.025 mmol) in dry $CD_3CN$ (0.25 mL) were prepared. Then, the solution of the phenyldiazonium tetrafluoroborate was added dropwise to that of the Sb(I) complex **4a**, resulting in a colour change to pale orange and formation of bubbles, which indicated $N_2$ evolution. The mixture was stirred for an additional 30 minutes. After that time, tetrahydrofuran (4 μL, 0.050 mmol, 2.0 equiv.) was added as internal standard and the mixture was analysed by $^1$H NMR, which showed formation of desired product in 87% yield (see Figure S1)

Crystallisation attempts of the reaction crude by slow diffusion of diethyl ether into the NMR sample at rt under inert atmosphere resulted in the unexpected crystallisation of dimeric Sb(II) complex **12**. Formation of dimer **12** and oxidative addition product **11a** with diazonium salts were confirmed obtaining these compounds by alternative synthetic routes (see sections 3.5 and 5.9 for further details) and comparing them to the crude NMR obtained from the reaction with diazonium salts (see Figure S2 and Figure S3).

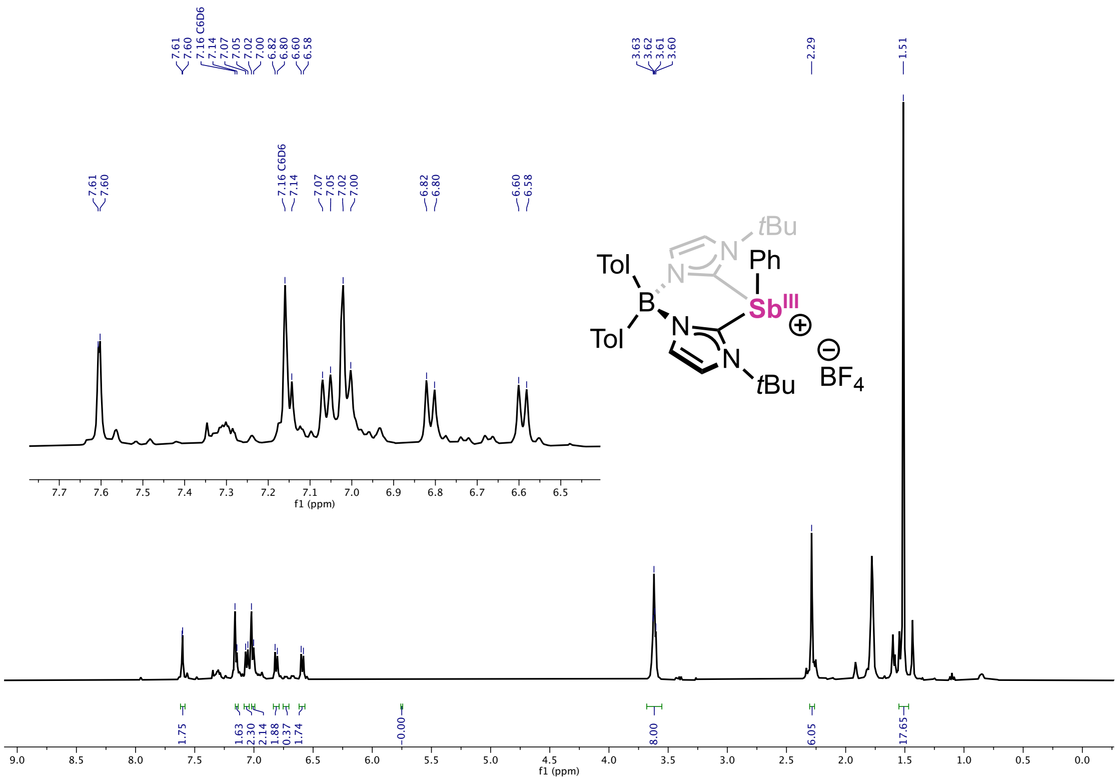

Figure S1. ¹H NMR spectra of the crude reaction of **4a** with phenyldiazonium tetrafluoroborate showing the formation of a major product corresponding to **11a**.

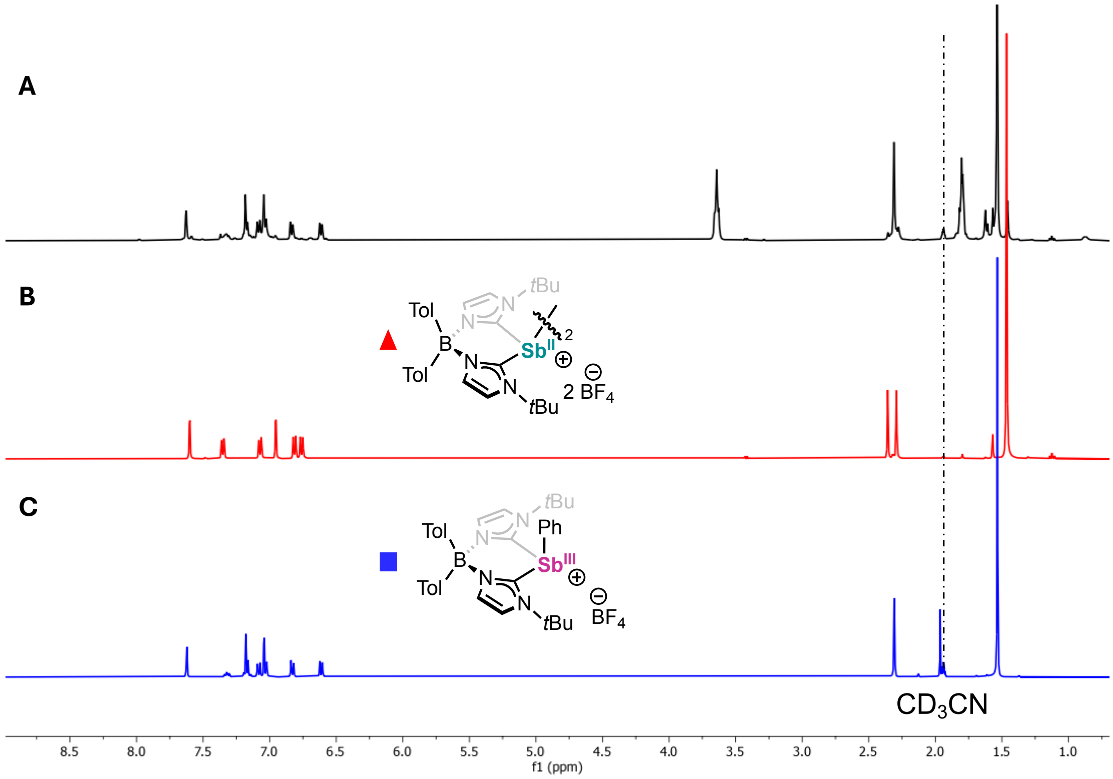

Figure S2. ¹H NMR spectra of the crude reaction of **4a** with phenyldiazonium tetrafluoroborate (**A**) and comparison with species **12** (**B**) and **11a** (**C**) which were synthesised using alternative routes (see sections 5.9 and 3.5 respectively).

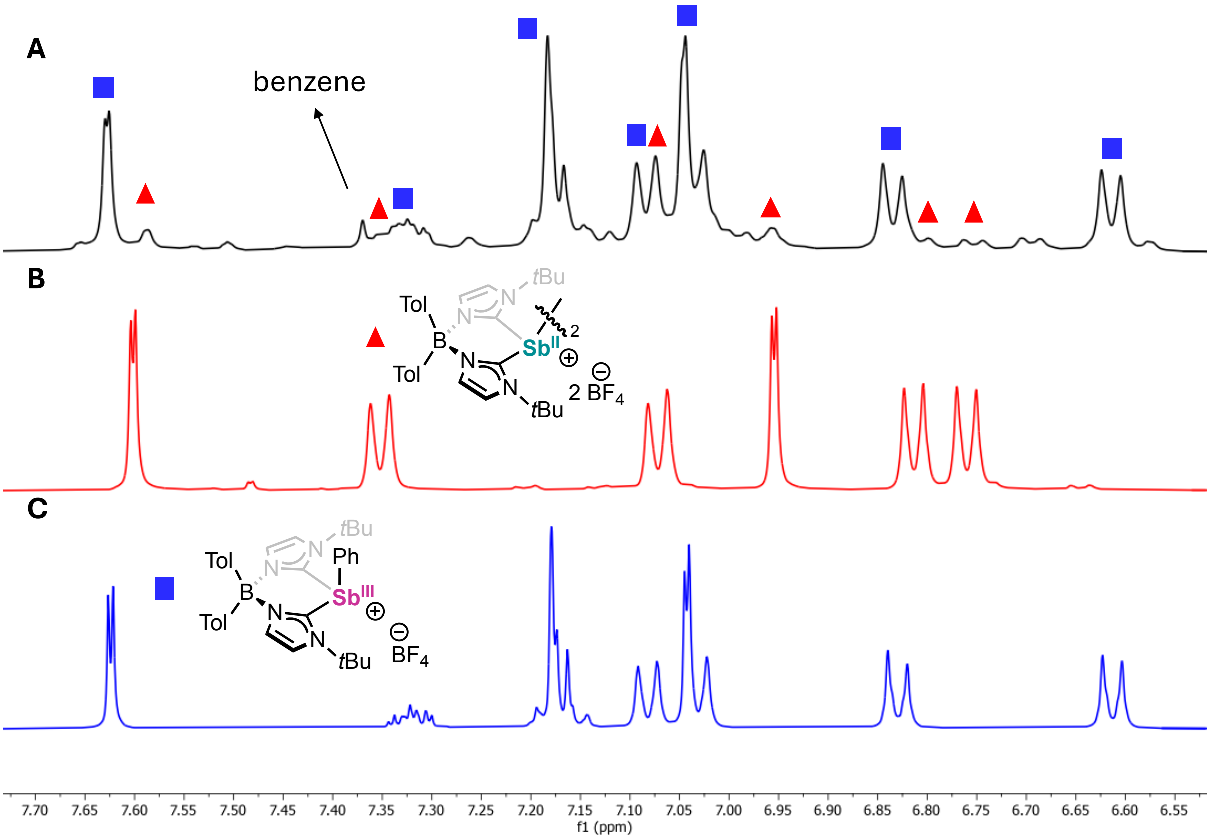


Figure S3. $^1$H NMR spectra (aromatic region) of the crude reaction of **4a** with phenyldiazonium tetrafluoroborate (**A**) and comparison with isolated species **12** (**B,** red triangle) and **11a** (**C**, blue square), which were synthesised using alternative routes (see sections 5.9 and 3.5 respectively).

5.4.1 Radical interception experiments with $B_2pin_2$

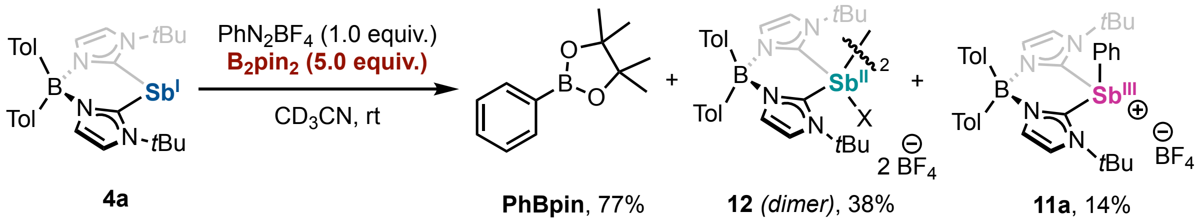


In two separate flasks under Ar, a solution of **4a** (15.0 mg, 1.0 equiv., 0.027 mmol) and bis(pinacolato)diborane (33.9 mg, 5.0 equiv., 0.134 mmol) in dry $CD_3CN$ (0.5 mL) and a solution of phenyldiazonium tetrafluoroborate (5.1 mg, 1.0 equiv., 0.027 mmol) in dry $CD_3CN$ (0.25 mL) were prepared. Then, the solution of the phenyldiazonium tetrafluoroborate was added dropwise to that of the Sb(I) complex **4a**, resulting in a colour change to pale orange and formation of bubbles, which indicated $N_2$ evolution. The mixture was stirred for an additional 30 minutes. After that time, 1,3,5-trimethoxybenzene (3.0 mg, 0.66 equiv., 0.018 mmol) was added as internal standard and the mixture was analysed by $^1H$ NMR, which showed formation of PhBpin (77%), **12** (38%) and **11a** (14%) (see above).

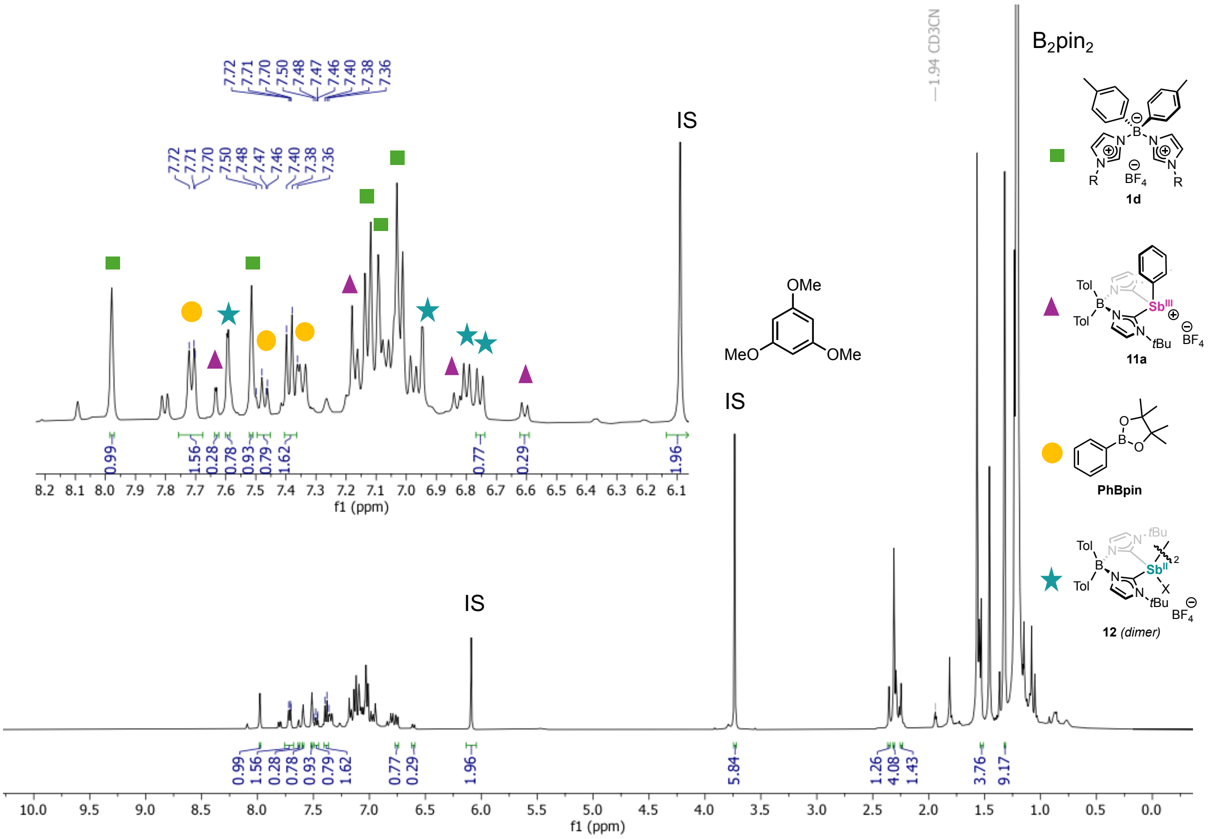


Figure S4. $^1H$ NMR in $CD_3CN$ after reaction of stibinidene **4a** with phenyldiazonium tetrafluoroborate in presence of $B_2pin_2$, showing the formation of phenyl stibine species **11a**, resulting phenyl pinacolborane (PhBpin), distibane **12** and ligand due to decomposition. 1,3,5-trimethoxybenzene was used as internal standard (IS).

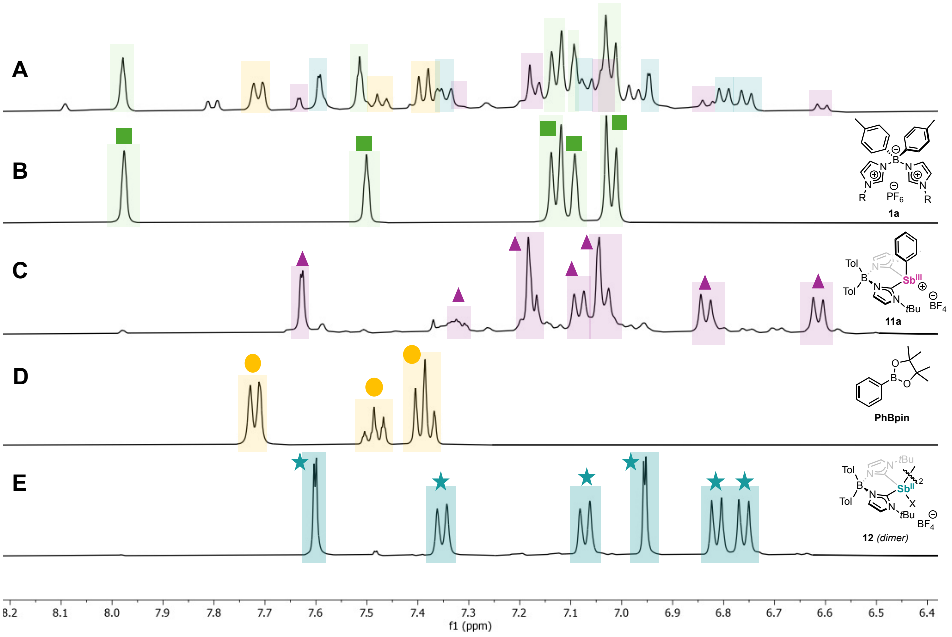


Figure S5. $^{1}$H NMR in $CD_3CN$ showing the aromatic region after reaction of stibinidene **4a** with phenyldiazonium tetrafluoroborate in presence of $B_2pin_2$ (A) and comparison with ligand **1a** (B), phenyl stibine species **11a** resulting from reaction of **4a** and benzenediazonium tetrafluoroborate (C), phenyl pinacolborane (D) and distibane **12** (E). 1,3,5-trimethoxybenzene was used as internal standard (IS).

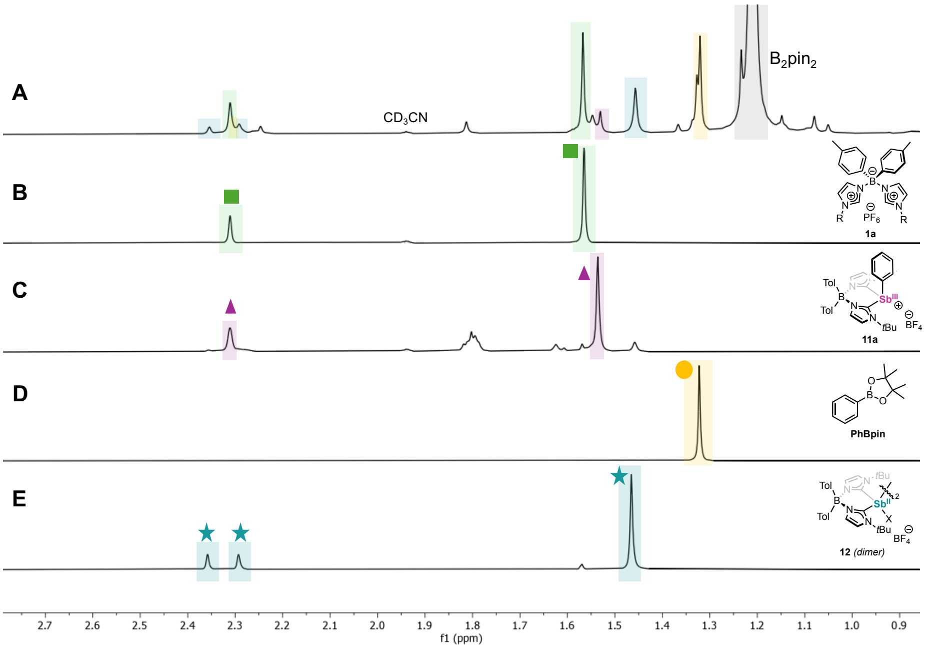


Figure S6. $^{1}$H NMR in $CD_3CN$ showing the aliphatic region after reaction of stibinidene **4a** with phenyldiazonium tetrafluoroborate in presence of $B_2pin_2$ (A) and comparison with ligand **1a** (B), phenyl stibine species **11a** (C), phenyl pinacolborane (D) and distibane **12** (E). 1,3,5-trimethoxybenzene was used as internal standard.

In the presence of $B_2pin_2$, the formation of **11a** was significantly suppressed (14% yield), while distibane **12** was obtained in 38% yield. The free ligand was isolated as the major product (48%), indicating decomposition pathways are more prevalent under these conditions. These results stand in stark contrast to the reaction conducted in the absence of a radical scavenger (see above), where **11a** is formed in 87% yield, accompanied by 10% of distibane **12** and detectable amounts of benzene. These observations support a mechanism involving an initial single-electron transfer (SET) event, generating an open-shell Sb(II) species (**Int-1**) and a phenyl radical (Scheme S1). In the absence of radical traps, rapid recombination of **Int-1** with the phenyl radical (pathway A) affords **11a** as the major product, while dimerisation of **Int-1** to form **12** (pathway B) proceeds more slowly, likely due to steric repulsion — as reflected by the long Sb–Sb distance observed in the crystal structure of **12** (see main text discussion). In the presence of $B_2pin_2$, the phenyl radical is intercepted,[8] resulting in the formation of PhBpin (pathway C). This disrupts the recombination pathway (A) and diverts **Int-1** toward decomposition and dimerisation, thereby accounting for the observed shift in product distribution.

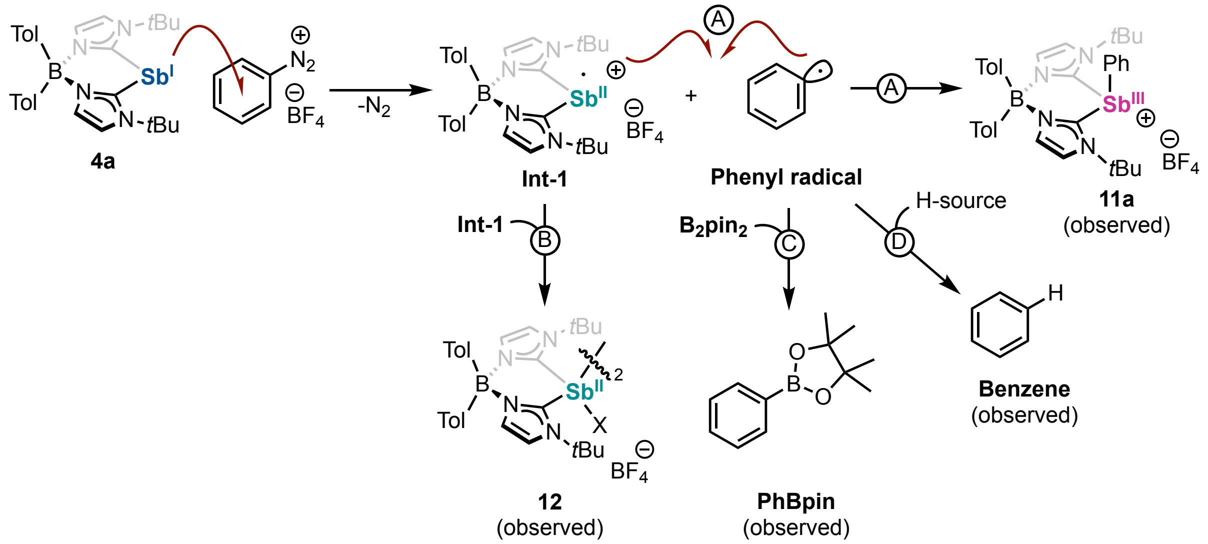


Scheme S1. Potential pathways for the reaction of stibinidene **4a** with phenyldiazonium tetrafluoroborate in presence or absence of $B_2pin_2$ as radical scavenger.

## 5.5 Reaction of 4a with 4-methoxybenzoyl chloride

### 5.5.1 Synthesis of **10a** using sodium tetrakis(3,5-bis(trifluoromethyl)phenyl)borate

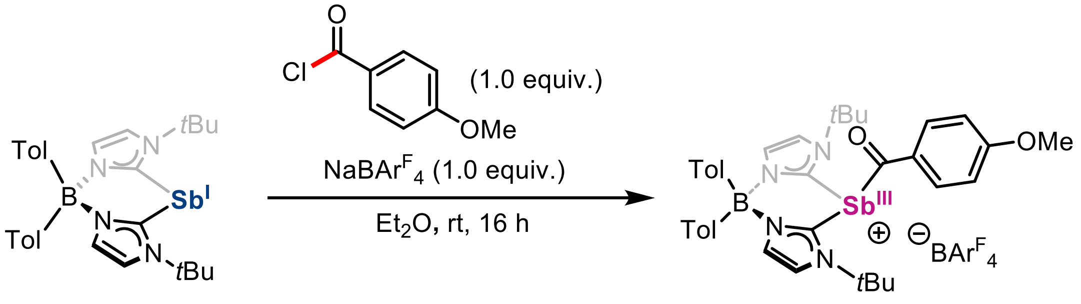


**4a** **10a**, 87%

To a stirred solution of **4a** (56 mg, 1.0 equiv., 0.10 mmol) and sodium tetrakis(3,5-bis(trifluoromethyl)phenyl)borate (89 mg, 1.0 equiv., 0.10 mmol) in diethyl ether (2 mL) was added 4-methoxybenzoyl chloride (17 mg, 1.0 equiv., 0.10 mmol) at rt. After stirring overnight, the reaction mixture was filtered and concentrated to obtain complex **10a** (0.10 g, 0.13 mmol, 87%) as pale-yellow solid.

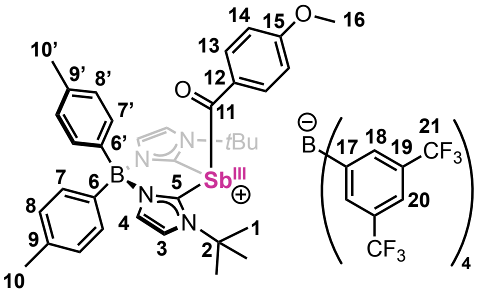

**$^1$H NMR – (400 MHz, $C_6D_6$) δ =** 8.34 (s, 8H, H-18), 7.67 (s, 4H, H-20), 7.10 (d, $J_{H-H}$ = 7.6 Hz, 2H, H-8), 6.90 – 6.81 (m, 4H, H7 + H-13), 6.78 (bs, 2H, H-3), 6.63 (d, $J_{H-H}$ = 7.6 Hz, 2H, H-8'), 6.54 – 6.42 (m, 4H, H-4 + H-7' + H-14), 3.22 (s, 3H, H-16), 2.22 (s, 3H, H-10), 1.91 (s, 3H, H-10'), 1.15 (s, 18H, H-1).

**$^{11}$B NMR – (128 MHz, $CDCl_3$) δ =** 2.93 (bs, $BTol_2$), -6.50 (s, BArF).

**$^{13}$C NMR – (101 MHz, $C_6D_6$) δ =** 211.6 (C, C=O, C-11), 165.9 (C,C-15), 162.7 (q, $J_{C-B}$ = 49.7 Hz, 4 × C, $BAr^F_4$, C-17), 144.7 (2 × C, C-Sb, C-5), 143.7 (C-B, C-6), 140.3 (C-B, C-6'), 138.2 (C, C-10), 136.9 (C, C-10'), 135.4 (bs, 8 × CH, $BAr^F_4$, C-18), 134.7 (2 × CH, C-8), 133.6 (2 × CH, C-4), 132.6 (2 × CH, C-13), 131.1 (C, C-12), 130.0 (qq, $J_{C-F}$ = 30.3, 2.9 Hz, 8 × C, $BAr^F_4$, C-19), 129.5 (2 × CH, C-8), 129.3 (2 × CH, C-3), 128.7 (2 × CH, C-8'), 125.3 (q, $J$ = 272.5 Hz, 8 × C, $BAr^F_4$, C-21), 121.2 (2 × CH, C-3), 118.1 (hept, $J_{C-F}$ = 3.8 Hz, 4 × CH, $BAr^F_4$, C-20), 114.1 (2 × CH, C-14), 60.7 (2 × C, C-2), 55.3 ($CH_3$, C-16), 31.3 (6 × $CH_3$, C-1), 21.1 ($CH_3$, C-10), 21.0 ($CH_3$, C-10'). Carbon atoms connected to B and Sb could be identified analysing the cross peaks in the HMBC spectrum.

**$^{19}$F NMR – (377 MHz, $C_6D_6$) δ =** -62.12 (s, 24F).

**IR (ATR): ῡ =** 2978, 1670, 1595, 1354, 1273, 1112, 1026, 887, 839, 769 $cm^{-1}$.

**HRMS (ASAP-HESI, m/z):** calc'd for $C_{36}H_{43}BN_4O_2Sb^+$ $[M-BarF_4]^+$ 695.2512; found 695.2500.

**CHN:** $C_{68}H_{55}B_2F_{24}N_4O_2Sb$ calcd. C 52.37 H 3.55 N 3.59 %; exp. C 52.68, H 3.12, N 3.26 %.

### 5.5.2 Synthesis of **10b** using sodium trifluoromethanesulfonate

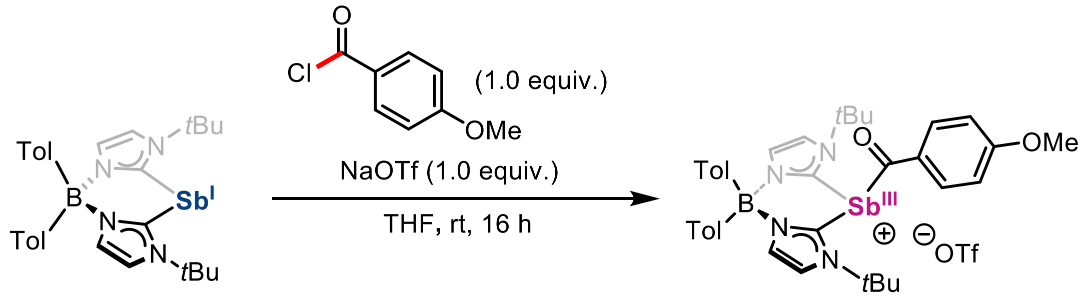


**4a** **10b**, 96%

To a stirred solution of **4a** (56 mg, 1.0 equiv., 0.10 mmol) and sodium trifluoromethanesulfonate (17 mg, 1.0 equiv., 0.10 mmol) in tetrahydrofuran (2 mL) was added 4-methoxybenzoyl chloride (17 mg, 1.0 equiv., 0.10 mmol) at rt. After stirring overnight, the reaction mixture was filtered and concentrated to obtain **10b** (82 mg, 0.096 mmol, 96%) as pale-yellow solid. Crystals suitable for SC-XRD were obtained from a bilayer of benzene and *n*-hexane solvent system at rt under inert atmosphere.

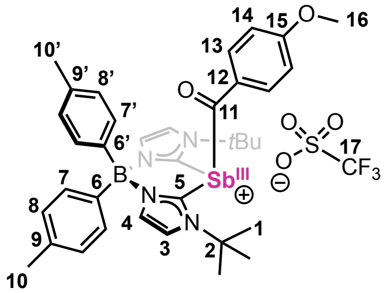

**$^{1}$H NMR – (400 MHz, THF-$d_8$) δ =** δ 7.81 (d, $J_{H\text{-}H}$ = 1.9 Hz, 2H, H-3), 7.01 (d, $J_{H\text{-}H}$ = 8.8 Hz, 2H, H-13), 6.96 (d, $J$ = 7.9 Hz, 2H, H-8), 6.92 (d, $J$ = 8.0 Hz, 2H, H-7), 6.87 (d, $J_{H\text{-}H}$ = 1.9 Hz, 2H, H-4), 6.85 (d, $J$ = 8.8 Hz, 2H, H-14), 6.41 (d, $J_{H\text{-}H}$ = 7.6 Hz, 2H, H-8'), 6.26 (d, $J_{H\text{-}H}$ = 7.7 Hz, 2H, H-7'), 3.84 (s, 3H, H-16), 2.24 (s, 3H, H-10), 1.94 (s, 18H, H-1), 1.92 (s, 3H, H-10').

**$^{11}$B NMR – (128 MHz, $CDCl_3$) δ =** 2.52 (bs)

**$^{13}$C NMR – (101 MHz, THF-$d_8$) δ =** 220.8 (C, C=O, C-11), 165.8 (C, C-15), 148.0 (2 × C, C-Sb, C-5), 146.3 (C, C-B, C-6), 143.7 (C, C-B, C-6'), 137.2 (C, C-9), 136.2 (2 × CH, C-7), 135.9 (C, C-9'), 134.1 (C, C-12), 133.7 (2 × CH, C-7'), 132.8 (2 × CH, C-13), 129.8 (2 × CH, C-4), 129.1 (2 × CH, C-8), 128.7 (2 × CH, C-8'), 123.0 (2 × CH, C-3), 122.3 (q, $J_{C\text{-}F}$ = 322.5 Hz, $CF_3$, C-17), 114.5 (2 × CH, C-14), 62.5 (2 × C, C-2), 56.4 ($CH_3$, C-16), 32.5 (6 × $CH_3$, C-1), 21.5 ($CH_3$, C-10), 21.3 ($CH_3$, C-10'). Carbon atoms connected to B and Sb could be identified analysing the cross peaks in the HMBC spectrum.

**$^{19}$F NMR – (377 MHz, THF-$d_8$) δ =** -78.99 (s, 3F).

**IR (ATR): ū =** 3132, 2970, 1620, 1581, 1257, 1141, 1026, 879, 833, 839, 771, 632 cm$^{-1}$.

**HRMS (ASAP-HESI, m/z):** calc'd for $C_{36}H_{39}BF_4N_4O_2Sb^+$ [M-Bar$^F_4$]$^+$ 767.2135; found 767.2153.

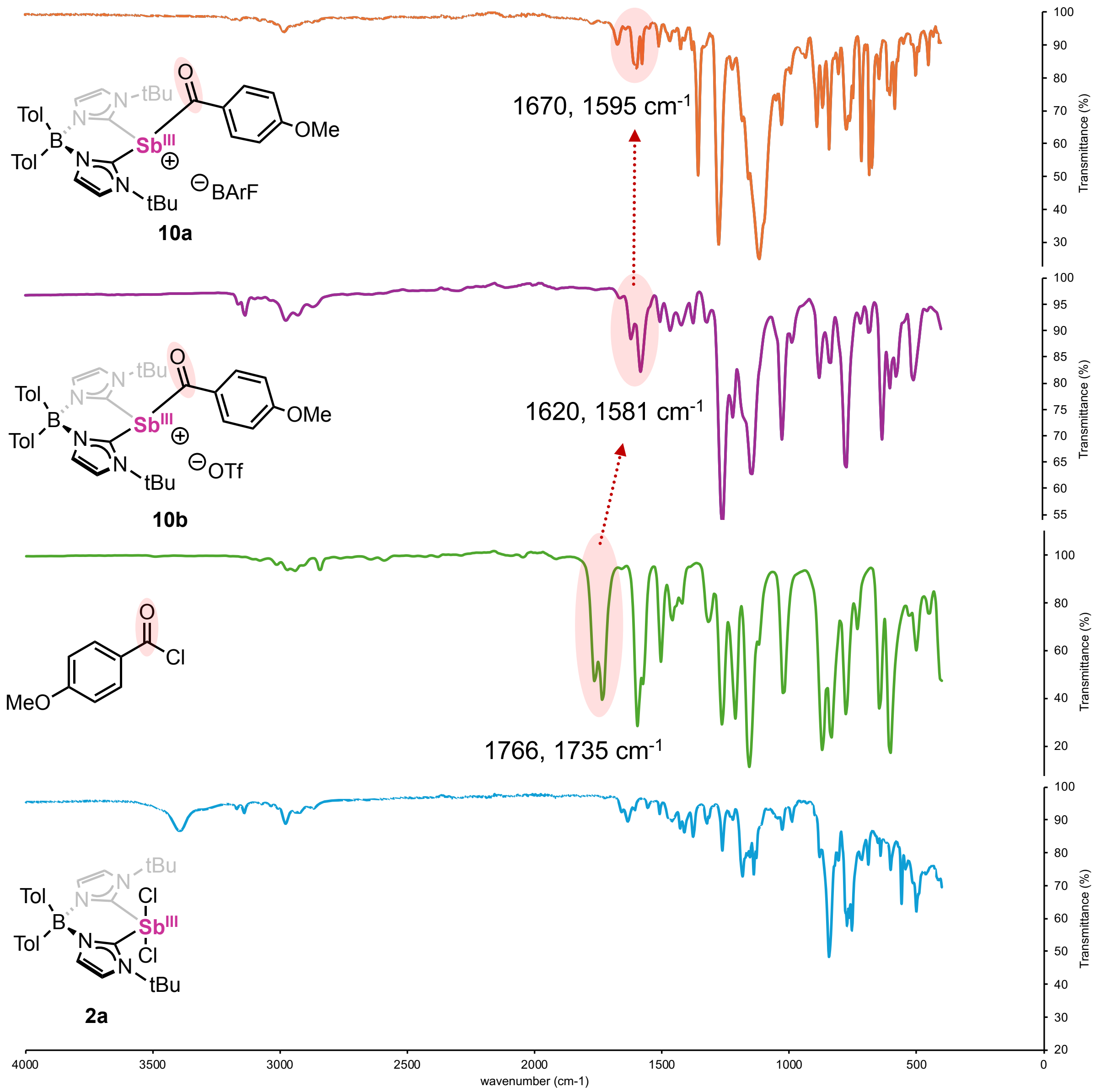


Figure S7. FT-IR spectra of compounds **10a** (orange), **10b** (purple), **anisoyl chloride** (green), **2a** (blue). C=O stretch is highlighted in red.

## 5.6 Reaction of 4a with (halo)pentafluorobenzene derivatives

### 5.6.1 Characterisation of pentafluorobenzene stibines

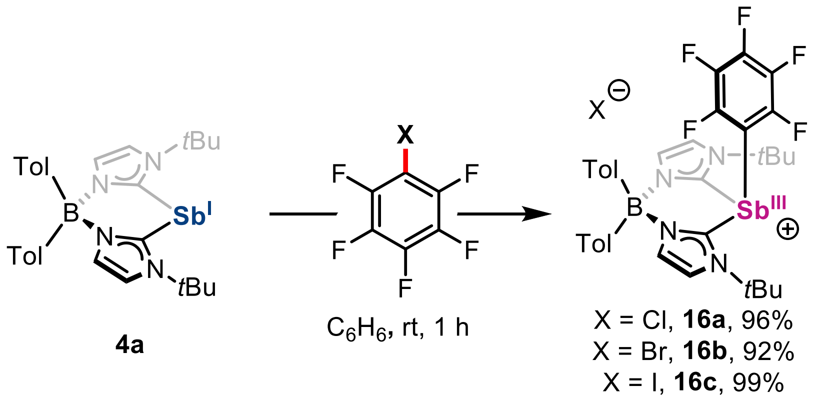


General procedure. To a solution of **4a** (50 mg, 1.0 equiv., 0.089 mmol) in tetrahydrofuran (2 mL) was added chloropentafluorobenzene (11.3 µL, 1.0 equiv., 0.087 mmol) and reaction mixture was stirred at rt for 1 h. Solvent was evaporated and residue was washed with *n*-hexane (2 × 8 mL) to obtain **16a** (65 mg, 0.086 mmol, 96%) as a white solid

Characterisation data for **16a**

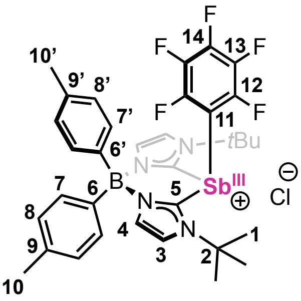


**$^{1}$H NMR – (400 MHz, THF-*d*$_8$) δ =** 7.67 (d, $J_{H\text{-}H}$ = 2.0 Hz, 2H, H-3), 6.93 (d, $J_{H\text{-}H}$ = 7.6 Hz, 2H, H-8), 6.79 (d, $J_{H\text{-}H}$ = 7.6 Hz, 2H, H-7), 6.74 (d, $J_{H\text{-}H}$ = 1.9 Hz, 2H, H-4), 6.66 (d, $J_{H\text{-}H}$ = 7.6 Hz, 2H, H-8'), 6.39 (d, $J_{H\text{-}H}$ = 7.6 Hz, 2H, H-7'), 2.24 (s, 3H, H-10), 2.15 (s, 3H, H-10'), 1.97 (s, 18H, H-1).

**$^{11}$B NMR – (128 MHz, CDCl$_3$) δ =** 2.63 (bs).

**$^{13}$C NMR – (101 MHz, THF-*d*$_8$) δ =** 151.0 (2 x C, C-Sb, C-5), 148.9 (C, C-B, C-6), 145.8 (C, C-B, C-6'), 136.9 (C, C-9), 136.0 (2 × CH, C-7), 135.5 (C, C-9'), 131.6 (2 × CH, C-7'), 129.8 (2 × CH, C-4), 129.0 (2 × CH, C-8), 128.0 (2 × CH, C-8'), 121.3 (2 × CH, C-3), 62.5 (2 × C, C-2), 31.1 (6 × $CH_3$, C-1), 21.5 ($CH_3$, C-10), 21.1 ($CH_3$, C-10'). Carbons of pentafluorophenyl moiety directly bonded to fluorines could not be detected due to a combination of low solubility and a complex coupling pattern.

**$^{19}$F NMR – (377 MHz, THF-*d*$_8$) δ =** -124.26 (m, 2F, F-12), -155.96 (m, 1F, F-14), -160.81 (m, 2F, F-13).

**HRMS (ASAP-HESI, m/z):** calc'd for $C_{34}H_{36}BF_5N_4Sb^+$ [M-Cl]+ 727.1986; found 727.1983

Characterisation data for **16b**

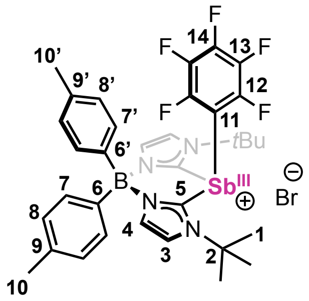


Following the general procedure, reaction with bromopentafluorobenzene resulted in the analogous compound **16b** (66 mg, 0.082 mmol, 92%) as highly insoluble white solid.

**$^{1}$H NMR – (400 MHz, CDCl$_3$) δ =** 7.30 (d, $J_{H\text{-}H}$ = 1.9 Hz, 2H), 6.99 (d, $J_{H\text{-}H}$ = 7.6 Hz, 2H), 6.89 (d, $J_{H\text{-}H}$ = 7.5 Hz, 2H), 6.86 (d, $J_{H\text{-}H}$ = 1.9 Hz, 2H), 6.72 (d, $J_{H\text{-}H}$ = 7.6 Hz, 2H), 6.34 (d, $J_{H\text{-}H}$ = 7.6 Hz, 2H), 2.28 (s, 3H), 2.19 (s, 3H), 1.99 (s, 18H).

**$^{11}$B NMR – (128 MHz, CDCl$_3$) δ =** 2.29 (bs).

**$^{13}$C NMR – (101 MHz, CDCl$_3$).** Due to low solubility and stability issues, $^{13}$C NMR of this compound was not of sufficient quality to be identified.

**$^{19}$F NMR – (377 MHz, CDCl$_3$) δ =** -123.47 (m, 2F), -153.46 (m, 1F), -159.98 (m, 2F).

**HRMS (ASAP-HESI, m/z):** calc'd for $C_{34}H_{36}BF_5N_4Sb^+$ [M-Br]$^+$ 727.1986; found 727.1980

Characterisation data for **16c**

Following the general procedure, reaction with bromopentafluorobenzene resulted in the analogous compound **16c** (75 mg, 0.088 μmol, 99%) as a highly insoluble white solid.

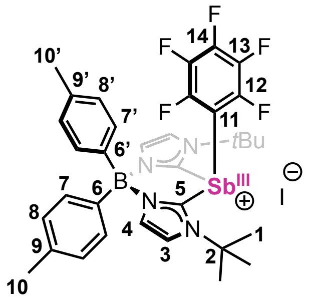


**$^{1}$H NMR – (400 MHz, CDCl$_3$) δ =** 7.35 (d, $J_{H\text{-}H}$ = 1.9 Hz, 2H), 7.02 (d, $J_{H\text{-}H}$ = 7.6 Hz, 2H), 6.96 – 6.90 (m, 4H), 6.74 (d, $J_{H\text{-}H}$ = 7.5 Hz, 2H), 6.33 (d, $J_{H\text{-}H}$ = 7.5 Hz, 2H), 2.31 (s, 3H), 2.21 (s, 3H), 2.03 (s, 18H).

**$^{11}$B NMR – (128 MHz, CDCl$_3$) δ =** 2.47 (bs).

**$^{13}$C NMR – (101 MHz, CDCl$_3$).** Due to low solubility and stability issues, $^{13}$C NMR of this compound was not of sufficient quality to be identified.

**$^{19}$F NMR – (377 MHz, CDCl3) δ =** -122.79 (m, 2F), -151.76(m, 1F), -161.04 (m, 2F).

**HRMS (ASAP-HESI, m/z):** calc'd for $C_{34}H_{36}BF_5N_4Sb^+$ [M-I]$^+$ 727.1986; found 727.1995

### 5.6.2 Comparison of pentafluorobenzene stibine chloride, bromide and iodide **16a-c**

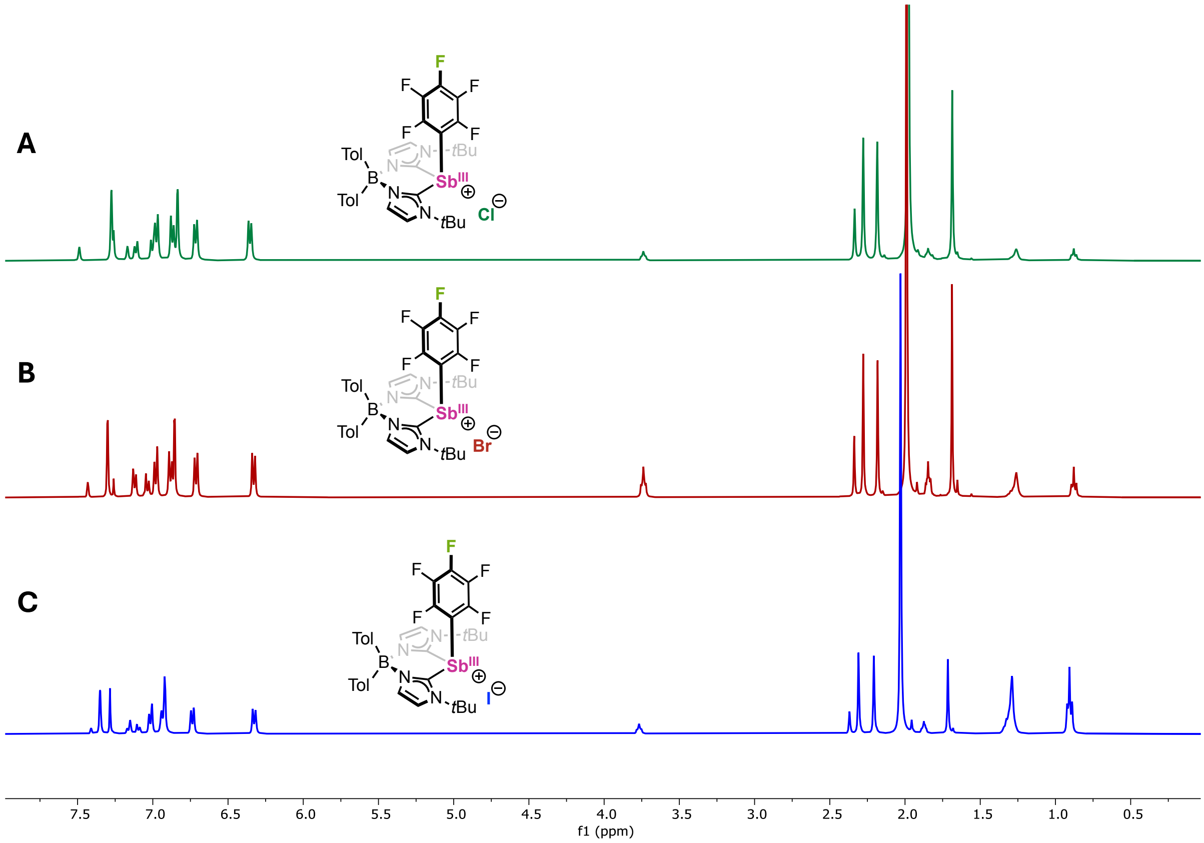


Figure S8. $^1$H NMR spectra of the reaction of **4a** with pentafluorochlorobenzene (**A**), pentafluorobromobenzene (**B**) and pentafluoroiodobenzene (**C**) in $CDCl_3$. In all cases, insoluble pentafluorobenzene stibanes (black triangles) are formed, stemming from the oxidative addition of C–X (X = Cl, Br and I) bonds to the Sb(I) centre, resulting in **16a-c**. Noteworthy, products are formed together with impurities stemming from protodemetallation and decomposition to the corresponding bis(imidazole)borate ligands (see below).

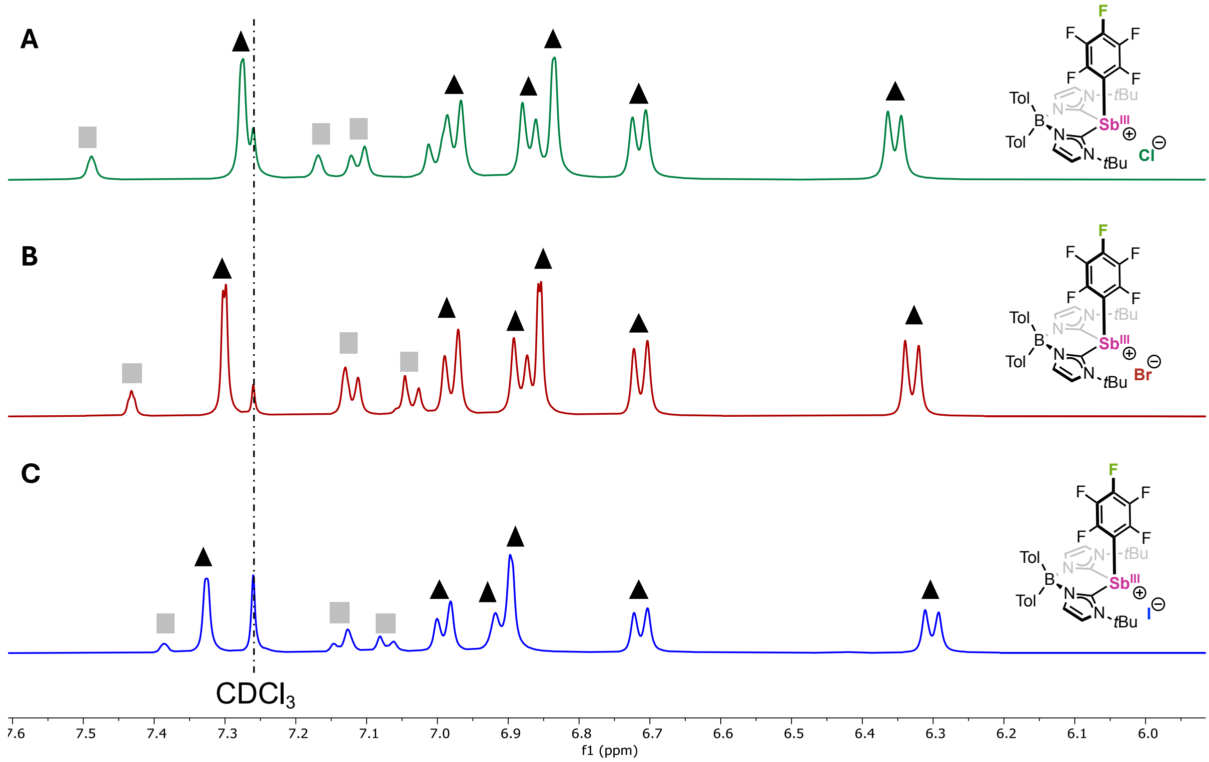


Figure S9. $^{1}$H NMR spectra (aromatic region) of the reaction of **4a** with pentafluorochlorobenzene (**A**), pentafluorobromobenzene (**B**) and pentafluoroiodobenzene (**C**) in $CDCl_3$. In all cases, insoluble pentafluorobenzene stibanes (black triangles) are formed, stemming from the oxidative addition of C–X (X = Cl, Br and I) bonds to the Sb(I) centre, resulting in **16a-c**. Noteworthy, products are formed together with impurities stemming from protodemetallation and decomposition to the corresponding bis(imidazole)borate ligands (highlighted as grey squares).

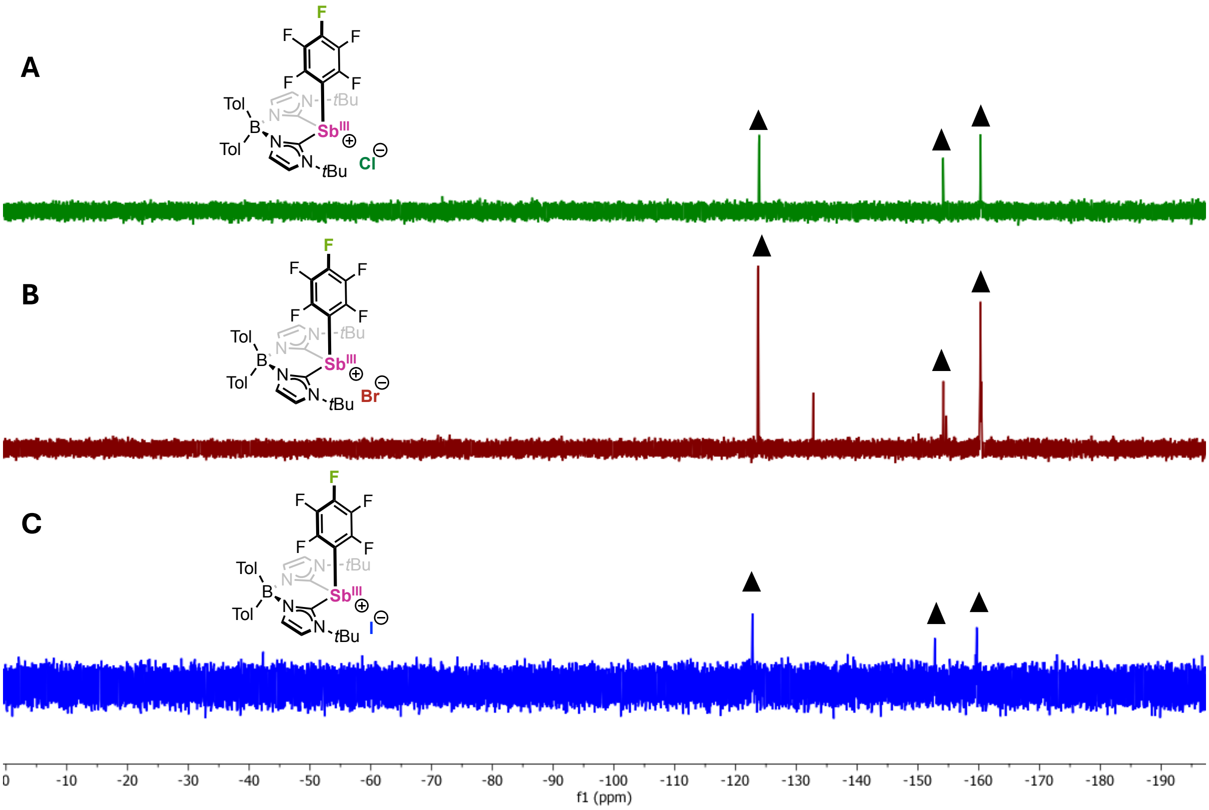

Figure S10. $^{19}$F NMR spectra of the reaction of **4a** with pentafluorochlorobenzene (**A**), pentafluorobromobenzene (**B**) and pentafluoroiodobenzene (**C**) in $CDCl_3$. In all cases, highly insoluble pentafluorobenzene stibanes (black triangles) are formed, stemming from the oxidative addition of C–X (X = Cl, Br and I) bonds to the Sb(I) centre, resulting in **16a-c**.

## 5.7 Reaction of 4a with (halo)pentafluorobenzene derivatives and $NaBAr^F_4$

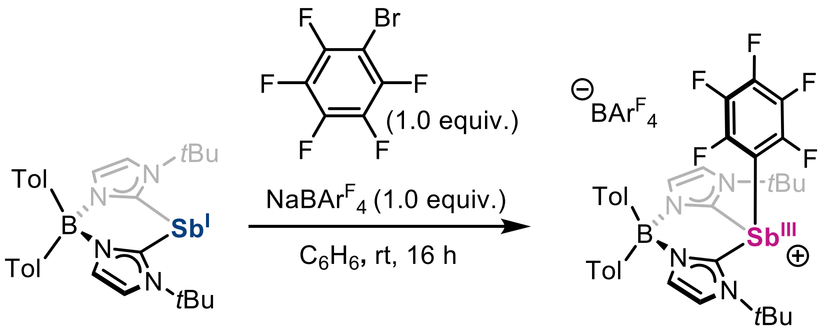


**4a** (1.0 equiv.) **16d**, 84%

To a stirred solution of Sb(I) complex **4a** (84 mg, 1.0 equiv., 0.15 mmol) and sodium tetrakis(3,5-bis(trifluoromethyl)phenyl)borate (133 mg, 1.0 equiv., 0.15 mmol) in diethyl ether (2 mL) was added 1-bromo-2,3,4,5,6-pentafluorobenzene (37 mg, 1.0 equiv., 0.15 mmol) at rt. After stirring overnight, the reaction mixture was filtered and concentrated to obtain pentafluorophenyl Sb(III) complex **16d** (200 mg, 84%) as white solid.

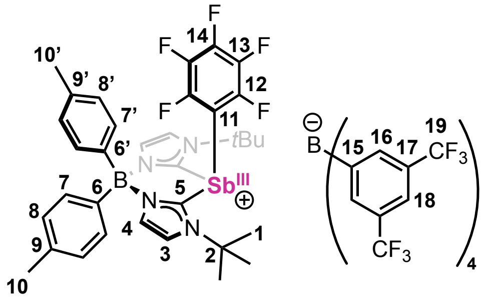


**$^1$H NMR – (400 MHz, $C_6D_6$) δ =** δ 8.31 (bs, 8H, H-16), 7.66 (bs, 4H, H-18), 7.06 (d, $J_{H-H}$ = 7.7 Hz, 2H, H-8), 6.81 (d, $J_{H-H}$ = 7.7 Hz, 2H, H-8'), 6.77 (d, $J_{H-H}$ = 7.8 Hz, 2H, H-7), 6.67 (d, $J_{H-H}$ = 1.9 Hz, 2H, H-3), 6.40 (d, $J_{H-H}$ = 7.6 Hz, 2H, H-7'), 6.36 (d, $J_{H-H}$ = 1.9 Hz, 2H, H-4), 2.19 (s, 3H, H-10), 2.13 (s, 3H, H-10'), 1.05 (s, 18H, H-1).

**$^{11}$B NMR – (128 MHz, $CDCl_3$) δ =** -2.32 (bs), -6.03 (s, BArF).

**$^{13}$C NMR – (101 MHz, $C_6D_6$) δ =** 162.7 (q, $J_{C-B}$ = 49.7 Hz, 4 × C, $BAr^F_4$, C-15), 144.4 (C-B, C-6), 142.2 (C-B, C-6'), 138.8 (2 × C, C-Sb, C-5), 138.6 (C, C-9), 137.2 (C, C-9'), 135.4 (bs, 8 × CH, $BAr^F_4$, C-16), 134.8 (2 × CH, C-7), 129.8 (qq, $J_{C-F}$ = 30.3, 2.9 Hz, 8 × C, $BAr^F_4$, C-17), 130.5 (2 × CH, C-7'), 130.0 (2 × CH, C-4), 129.4 (2 × CH, C-8), 128.3 (2 × CH, C-8'), 125.2 (q, $J_{C-F}$ = 272.5 Hz, 8 × C, $BAr^F_4$, C-19), 122.0 (2 × CH, C-3), 118.1 (hept, $J_{C-F}$ = 3.8 Hz, 4 × CH, $BAr^F_4$), 61.3 (2 × C), 30.9 (6 × $CH_3$), 21.1 ($CH_3$, C-10), 20.7 ($CH_3$, C-10'). Carbons of pentafluorophenyl moiety directly bonded to fluorines could not be detected due to partial decomposition of the complex.

**$^{19}$F NMR – (377 MHz, $C_6D_6$) δ =** -62.14 (s, 24F, F-19), -122.59 – -122.70 (m, 2F, F-12), -147.30 – -147.43 (m, 1F, F-14), -157.52 – -157.65 (m, 2F, F-13).

**HRMS (ASAP-HESI, m/z):** calc'd for $C_{34}H_{36}BF_5N_4Sb^+$ $[M-BAr^F_4]^+$ 727.1979; found 727.1986

Complex **16d** was obtained by the same procedure, using 1-chloro-2,3,4,5,6-pentafluorobenzene or 1-iodo-2,3,4,5,6-pentafluorobenzene, and its formation was confirmed by NMR (see Figure S11 and Figure S12). Using 1-chloro-2,3,4,5,6-pentafluorobenzene and 1-iodo-2,3,4,5,6-pentafluorobenzene, impurities belonging to ligand byproducts stemming from protodeelementation were observed due to rapid decomposition of **16d** at room temperature.

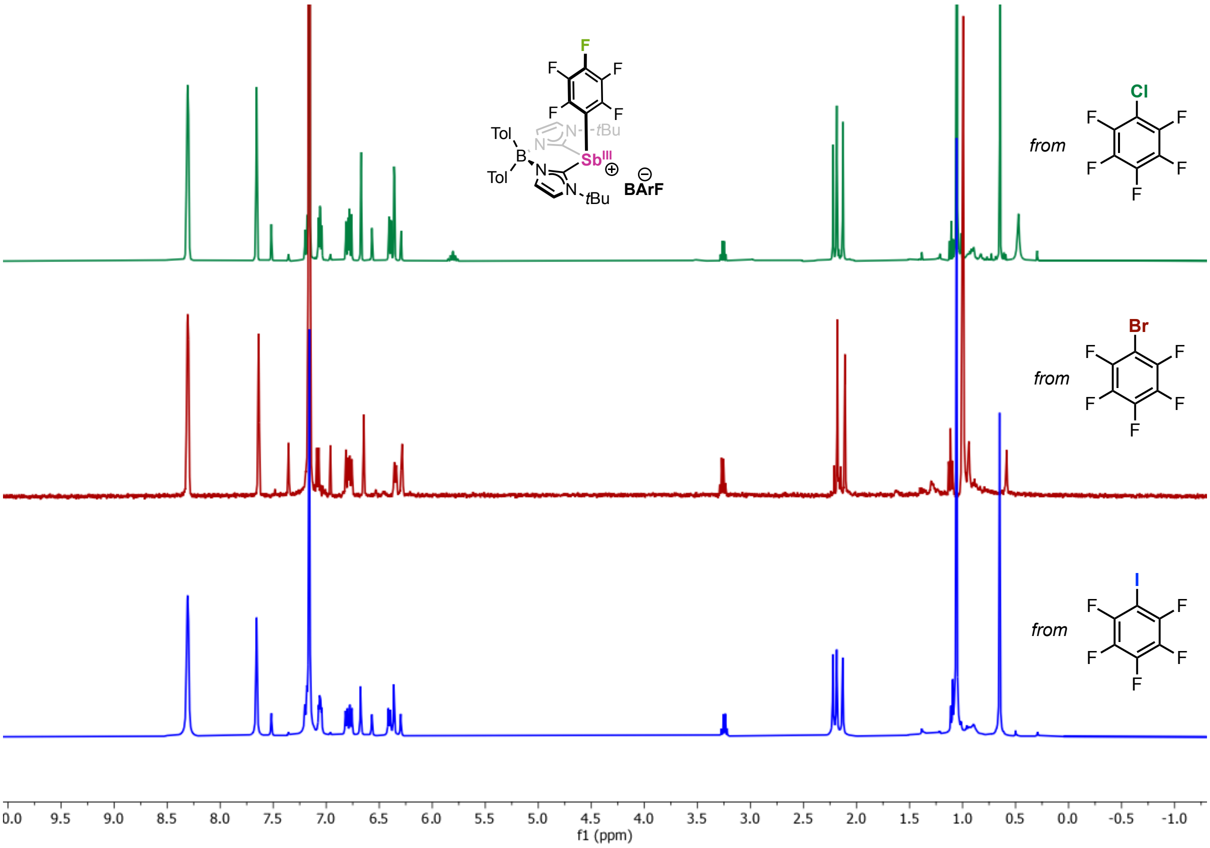


Figure S11. $^{1}$H NMR spectra of the reaction of **4a** with pentafluorochlorobenzene (**A**), pentafluorobromobenzene (**B**) and pentafluoroiodobenzene (**C**) and NaBArF in $CDCl_3$. In all cases, **16d** (black triangles) is formed, stemming from the oxidative addition of C–X (X = Cl, Br and I) bonds to the Sb(I) centre. Noteworthy, when **4a** is reacted with pentafluorobromobenzene (**B**) and pentafluoroiodobenzene (**C**) in presence of NaBArF, **16d** is observed together with impurities stemming from protodemetallation and partial decomposition of the target product.

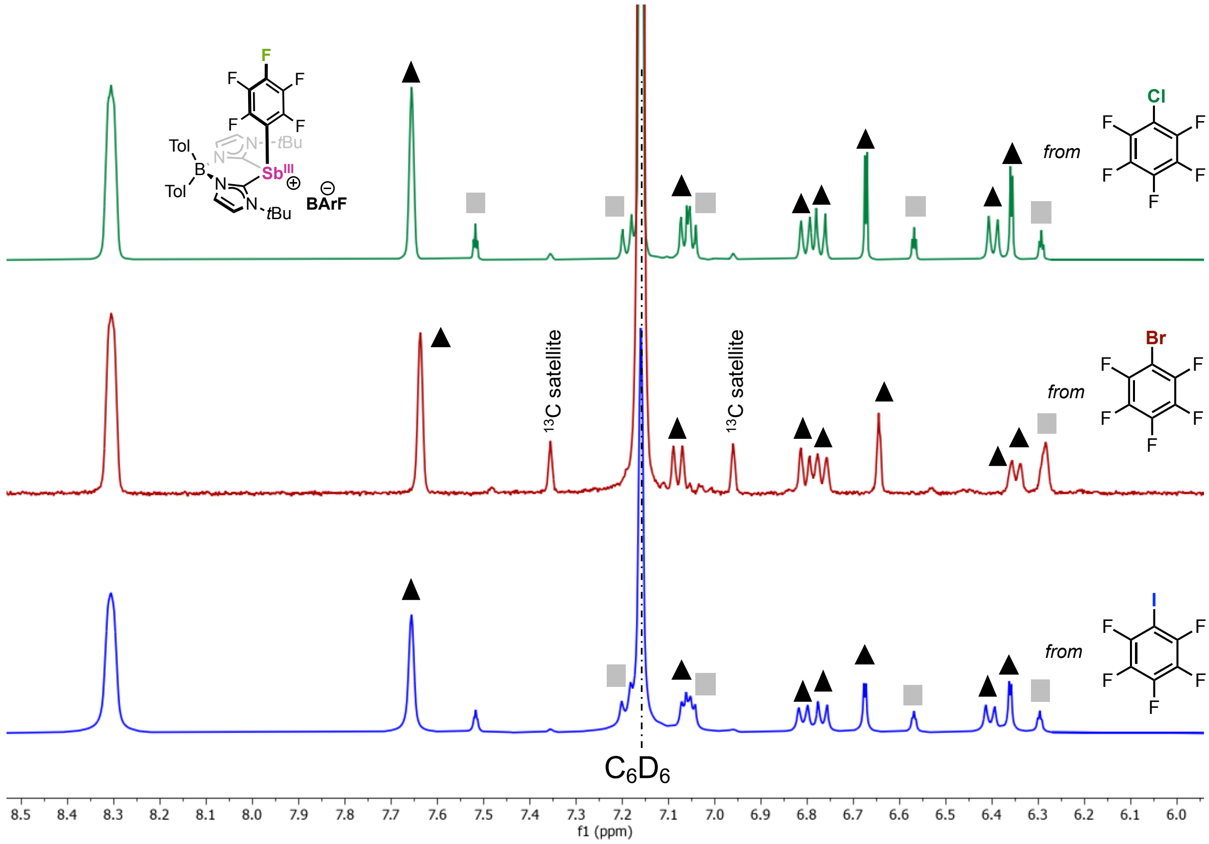


Figure S12. $^{1}$H NMR spectra (aromatic region) of the reaction of **4a** with pentafluorochlorobenzene (**A**), pentafluorobromobenzene (**B**) and pentafluoroiodobenzene (**C**) and NaBArF in $CDCl_3$. In all cases, **16d** (black triangles) is formed, stemming from the oxidative addition of C–X (X = Cl, Br and I) bonds to the Sb(I) centre. Noteworthy, when **4a** is reacted with pentafluorobromobenzene (**B**) and pentafluoroiodobenzene (**C**) in presence of NaBArF, **16d** is observed together with impurities stemming from protodemetallation and partial decomposition of the target product (grey squares).

## 5.8 Reaction of 4a with (halo)pentafluorobenzene derivatives and NaOTf

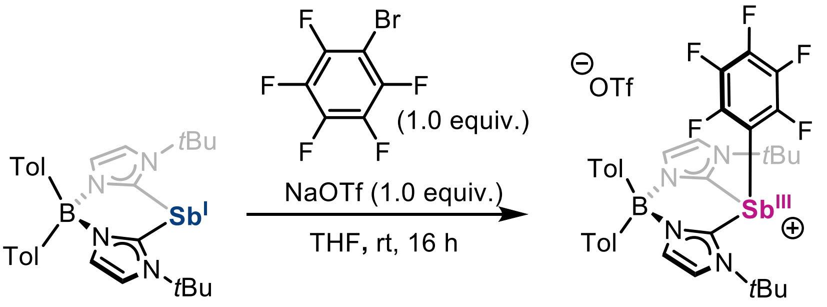


**4a** (1.0 equiv.) **16e**, 84%

To a stirred solution of **4a** (56 mg, 1.0 equiv., 0.10 mmol) and sodium trifluoromethanesulfonate (17 mg, 1.0 equiv., 0.10 mmol) in tetrahydrofuran (2 mL) was added 1-bromo-2,3,4,5,6-pentafluorobenzene (25 mg, 1.0 equiv., 0.10 mmol) at rt. After stirring overnight, the reaction mixture was filtered and concentrated to obtain **16e** (74 mg, 84%) as white solid. Crystals suitable for SC-XRD were obtained from a bilayer of ethyl acetate and *n*-hexane solvent system under inert atmosphere. This compound resulted to be highly unstable and decomposed over time even under inert atmosphere and low temperatures (-20 °C).

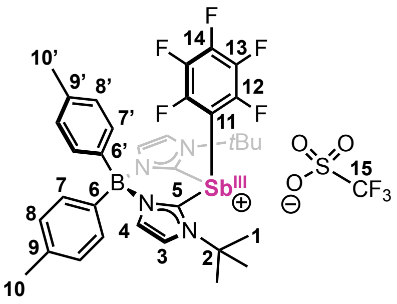

**$^{1}$H NMR – (400 MHz, THF-$d_8$) δ =** 7.75 (d, $J_{H-H}$ = 1.9 Hz, 2H, H-3), 6.93 (d, $J_{H-H}$ = 7.7 Hz, 2H, H-8), 6.84 (d, $J_{H-H}$ = 7.5 Hz, 2H, H-7), 6.78 (d, $J_{H-H}$ = 1.9 Hz, 2H, H-4), 6.68 (d, $J_{H-H}$ = 7.6 Hz, 2H, H-8’), 6.37 (d, $J_{H-H}$ = 7.7 Hz, 2H, H-7’), 2.23 (s, 3H, H-10), 2.15 (s, 3H, H-10’), 1.96 (s, 18H, H-1).

**$^{11}$B NMR – (128 MHz, THF-$d_8$) δ =** 1.40 (bs).

**$^{13}$C NMR – (101 MHz, THF-$d_8$) δ =** 148.5 – 148.0 (m, 2 × C, C-F, C-12/C-13), 148.4 (C, C-B, C-6), 148.0 (2 × C, C-Sb, C-5), 146.3 – 145.7 (m, 2 × C, C-F, C-12/C-13), 145.8 (C, C-B, C-6), 143.4 – 142.7 (m, C, C-F/S-Sb, C-14/C-11), 140.9 – 140.3 (m, C, C-F/C-Sb, C-14/C-11), 137.0 (C, C-9), 136.1 (2 × CH, C-7), 135.7 (C, C-9’), 131.6 (2 x CH, C-7’), 130.2 (2 × CH, C-4), 129.1 (2 × CH, C-8), 128.1 (2 × CH, C-8’), 122.3 (2 × CH, C-3), 121.9 (q, $J_{C-F}$ = 320.4 Hz, $CF_3$, C-15) 62.9 (2 × C, C-2), 31.2 (6 × $CH_3$, C-1), 21.5 ($CH_3$, C-10), 21.1 ($CH_3$, C-10’). Carbon atoms connected to B and Sb could be identified analysing the cross peaks in the HMBC spectrum.

**$^{19}$F NMR – (377 MHz, THF-$d_8$) δ =** -79.24 (s, 3F, F-15), -123.54 – -123.67 (m, 2F, F-12), -156.36 – -156.48 (m, 1F, F-14), -161.99 – -162.17 (m, 2F, F-13).

**HRMS (ASAP-HESI, m/z):** calc’d for $C_{34}H_{36}BF_5N_4Sb^+$ $[M-OTf]^+$ 727.1986; found 727.1965.

## 5.9 Synthesis of Sb(II) dimer 12

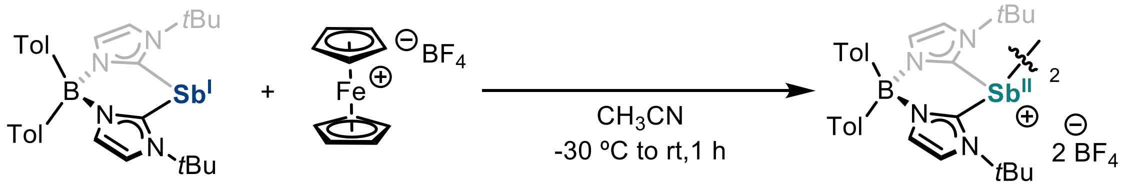


**4a** (1.05 equiv.) 1.0 equiv. **12**, 74%

To a stirred solution of Sb(I) complex **4a** (59 mg, 1.05 equiv., 0.11 mmol) in acetonitrile (2 mL) was added dropwise a solution of ferrocenium tetrafluoroborate (27 mg, 1.0 equiv., 0.10 mmol) in acetonitrile (2 mL) at -30 °C under Ar. The mixture was stirred for 5 min, warmed up to rt and stirred for an additional hour. Then, diethyl ether (5 mL) was added to induce precipitation of the desired product. The supernatant was removed using a syringe and the solid was washed with additional diethyl ether (2 × 3 mL). After drying in vacuo, Sb(II)-Sb(II) dimer **12** (48 mg, 0.037 mmol, 74 %) was obtained as a pale yellow solid. Crystals suitable for SC-XRD could be grown via slow diffusion of diethyl ether to an acetonitrile solution of the compound at rt under inert atmosphere, providing unambiguous characterisation of this dimeric species.

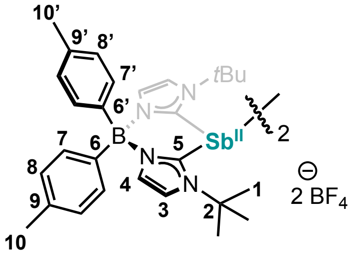


**$^{1}$H NMR – (400 MHz, $CD_3CN$) δ =** 7.60 (d, $J_{H\text{-}H}$ = 1.9 Hz, 4H, H-3), 7.35 (d, $J_{H\text{-}H}$ = 7.5 Hz, 4H, H-8), 7.07 (d, $J_{H\text{-}H}$ = 7.6 Hz, 4H, H-8'), 6.95 (d, $J_{H\text{-}H}$ = 1.9 Hz, 4H, H-4), 6.81 (d, $J_{H\text{-}H}$ = 7.8 Hz, 4H, H-7), 6.76 (d, $J_{H\text{-}H}$ = 7.7 Hz, 4H, H-7'), 2.36 (s, 6H, H-10), 2.29 (s, 6H, H-10'), 1.47 (s, 36H, H-1).

**$^{11}$B NMR – (128 MHz, $CDCl_3$) δ =** 1.64 (bs, $BTol_2$), -1.19 (s, $BF_4$).

**$^{13}$C NMR – (101 MHz, $CD_3CN$) δ =** 145.0 (2 × C, C-B, C-6), 141.5 (2 × C, C-B, C-6'), 138.5 (4 × C, C-9 + C-9'), 138.3 (4 × C, C-Sb, C-5), 135.7 (4 × CH, C-7'), 133.8 (4 × CH, C-7), 131.6 (4 × CH, C-8), 131.5 (4 × CH, C-4), 129.4 (4 × CH, C-8'), 124.1 (4 × CH, C-3), 61.9 (4 × C, C-2), 32.3 (12 × $CH_3$, C-1), 21.3 (2 × $CH_3$, C-10), 21.2 (2 × $CH_3$, C-10').

**$^{19}$F NMR – (377 MHz, $CD_3CN$) δ =** -63.06 – -63.39 (m, 4F). The signal includes both the F atoms attached to $^{10}$B and $^{11}$B isotopes.

**HRMS (ASAP-HESI, m/z)**: Peaks corresponding to **12** could not be assigned, possibly due to decompositon of 12 under analysis conditions.

**CHN**: $C_{56}H_{72}B_2F_8N_8Sb_2$ calcd. C 51.90 H 5.60 N 8.65 %; exp. C 51.65, H 5.30, N 8.95 %.

## 6 Reductive elimination from Sb(III) species

### 6.1 Stoichiometric reductive elimination of H-H bonds

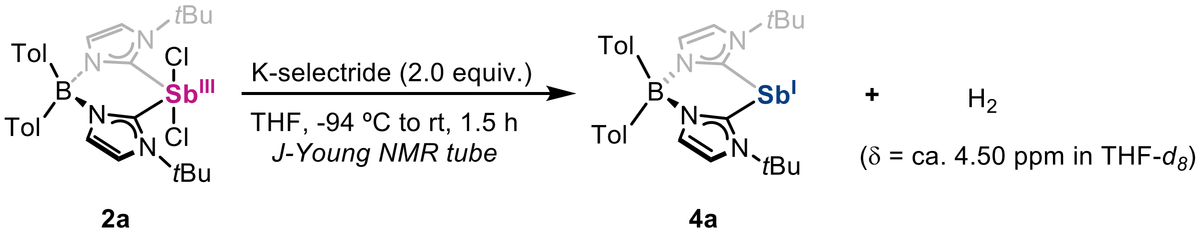


A solution of Sb(III) complex **2a** (20 mg, 1.0 equiv., 0.023 mmol) in THF-$d_8$ (0.4 mL) in a sealed J-Young NMR tube under argon atmosphere was frozen using liquid $N_2$. The tube was then carefully opened and K-Selectride (46 μL, 1 molar in THF, 2 equiv., 0.046 mmol) was added quickly whilst frozen. The J-Young NMR tube was closed and cycled with argon 3 times whilst the reaction mixture was frozen. The tube was then warmed to rt and kept at that temperature for 30 mins before the NMR spectrum was acquired. The $^1$H NMR spectrum showed the formation of the Sb(I) complex **4a** and $H_2$ (Figure S13).

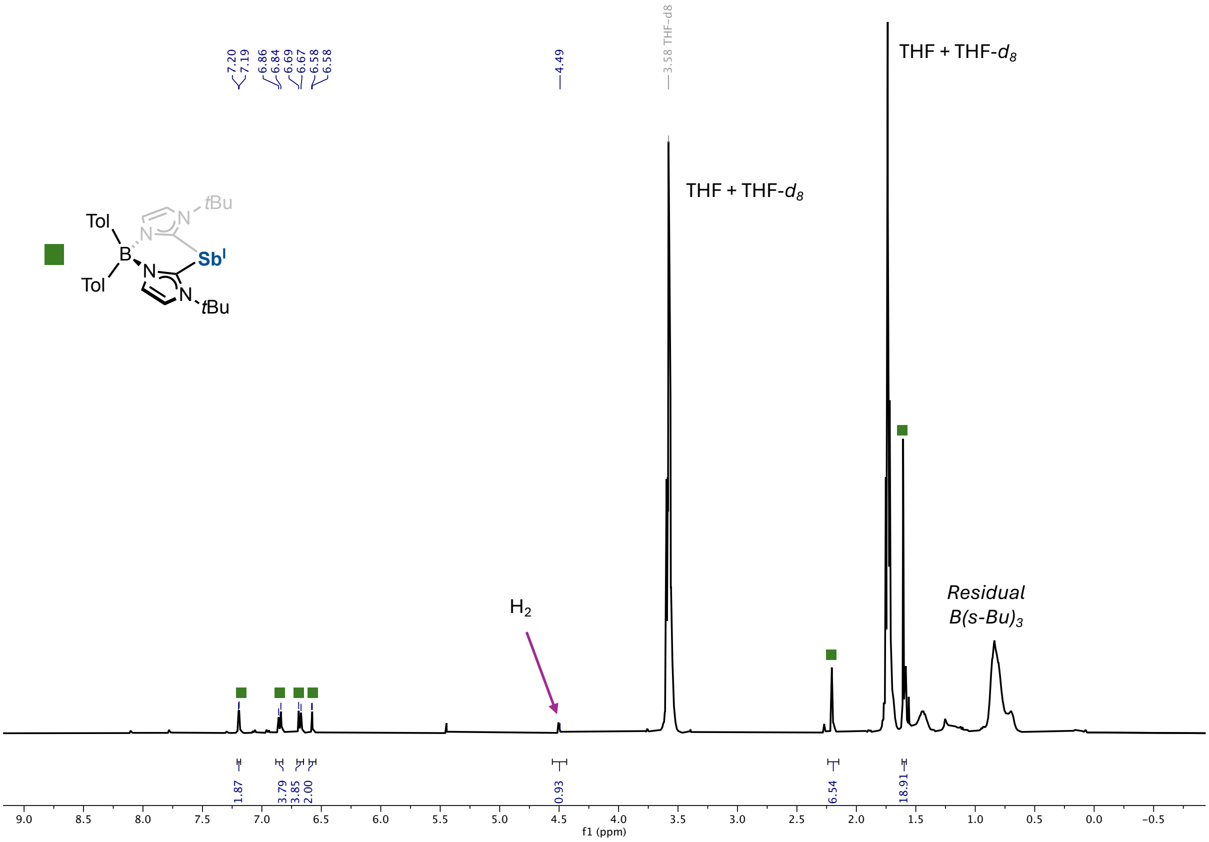


Figure S13. $^1$H NMR spectra of the reaction of **2a** with K-Selectride in a sealed J-Young NMR tube, showing a signal at $\delta \approx 4.5$ ppm corresponding to $H_2$ in solution. Residual THF signals are observed due to K-Selectride (1.0 M solution in THF).

### 6.2 Stoichiometric reductive elimination of C-H bonds from 8a

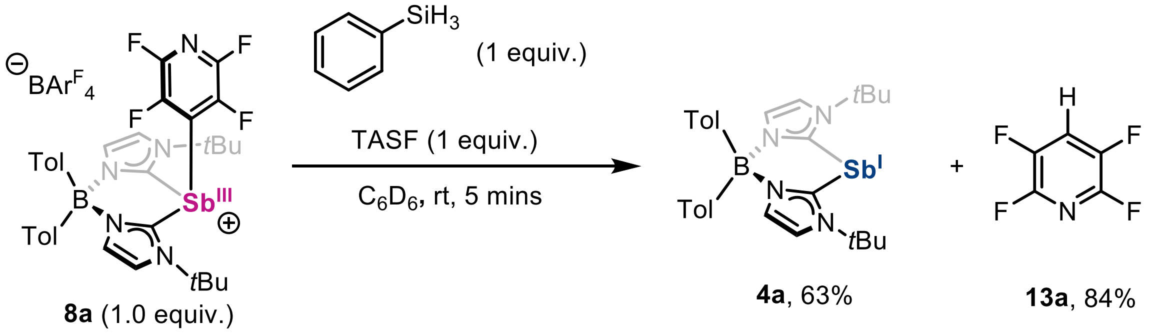


To a solution of Sb(III) complex **8a** (39 mg, 1.0 equiv., 0.025 mmol) and 1,3,5-trimethoxybenzene (2.4 mg, 0.5 equiv., 0.0125 mmol) in 0.3 mL of $C_6D_6$ in a vial was added phenyl silane (3.5 g, 1.0 equiv., 0.025 mmol). The reaction mixture was stirred and then a solution of tris(dimethylamino)sulfonium difluorotrimethylsilicate (7.4 mg, 1.0 equiv., 0.025 mmol) in 0.2 mL of $C_6D_6$ was added. The resulting mixture was stirred for a further 5 min and analysed by $^1$H NMR, which showed the formation of **4a** (63 %) and 2,3,5,6-tetrafluoropyridine **13a** (84%) (Figure S14 and Figure S15).

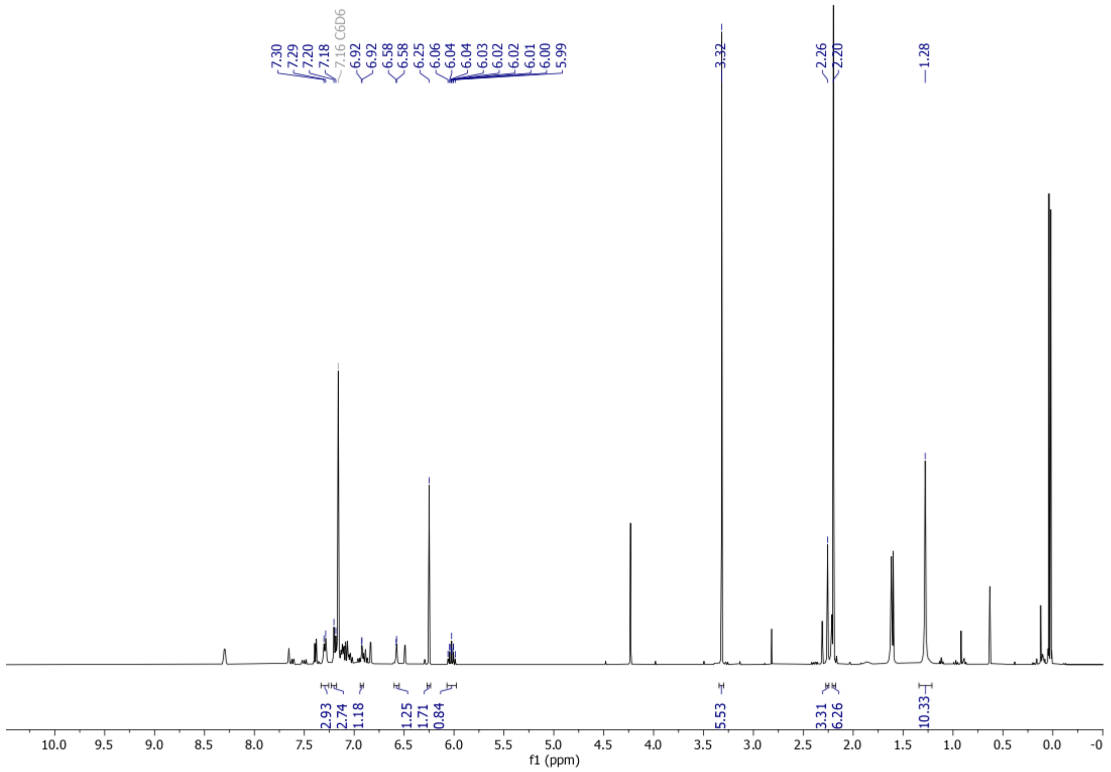


Figure S14. $^{1}$H NMR spectrum of the reaction mixture of isolated **8a** with phenyl silane, TASF and 1,3,5-trimethoxybenzene (internal standard, IS) in $C_6D_6$.

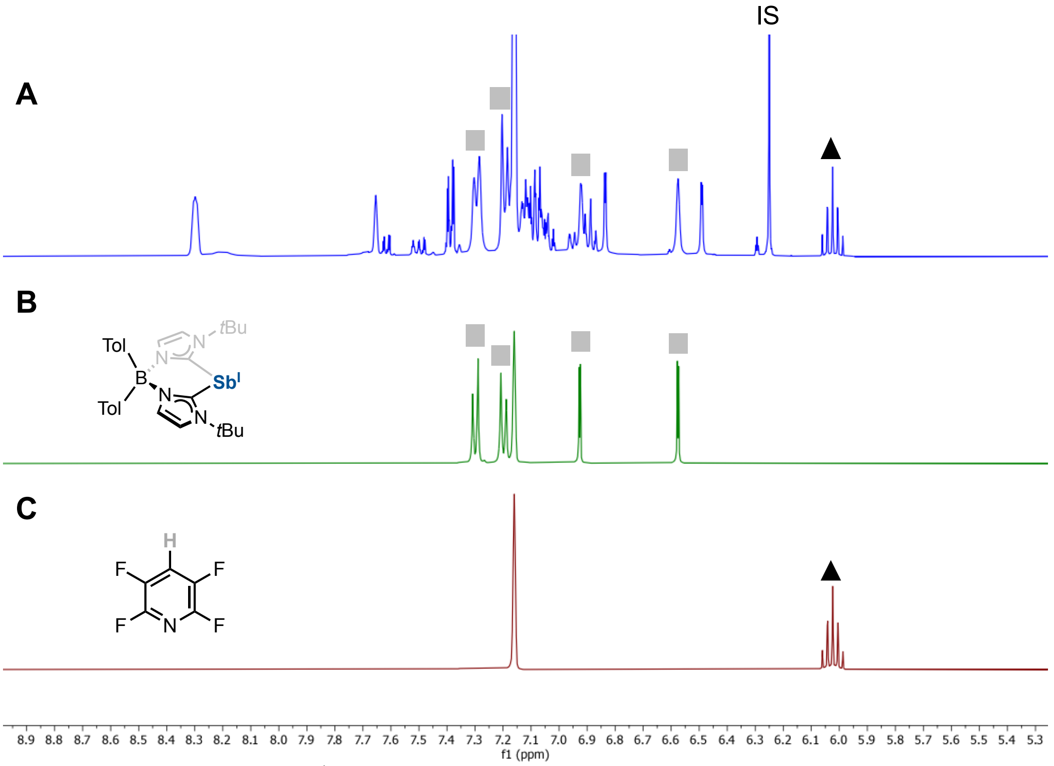


Figure S15. Stacking of the $^{1}$H NMR spectra of (**A**) reaction mixture of isolated **8a**, phenyl silane and internal standard (IS) 1,3,5-trimethoxybenzene after addition of fluoride source TASF (**B**), pure **4a** (grey squares) and (**C**) pure 2,3,5,6-tetrafluoropyridine **13a** (black triangle) in $C_6D_6$.

## 6.3 Stoichiometric reductive elimination of C-H bonds from 8b

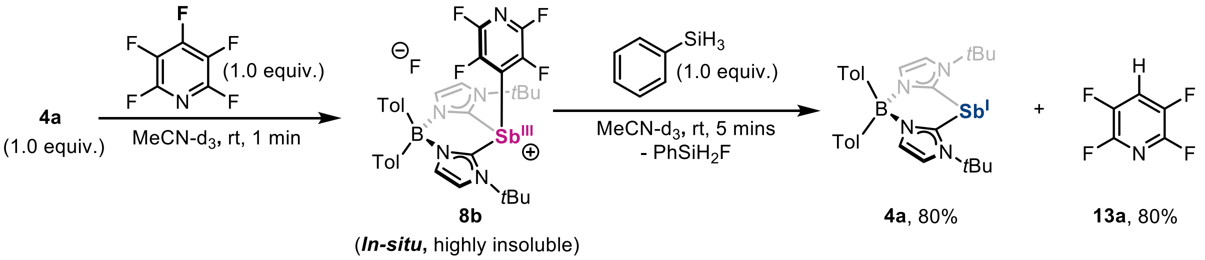


To a solution of Sb(III) complex **4a** (14 mg, 1.0 equiv., 0.025 mmol) and 1,3,5-trimethoxybenzene (2.2 mg, 0.513 equiv., 0.0125 mmol) in 0.5 mL of $CD_3CN$ in a J-Young NMR tube was added pentafluoropyridine (6.2 g, 1.0 equiv., 0.025 mmol) and the mixture was shaken for a minute, resulting in the formation of a white precipitate that couldn't be analysed by NMR. After this, phenylsilane (3.1 mg, 1.0 equiv., 0.025 mmol) was added and the reaction mixture was then checked with $^1$H NMR to obtain the yield of regenerated **4a** (80%) and 2,3,5,6-tetrafluoropyridine **13a** (80%) (Figure S16 and Figure S17).

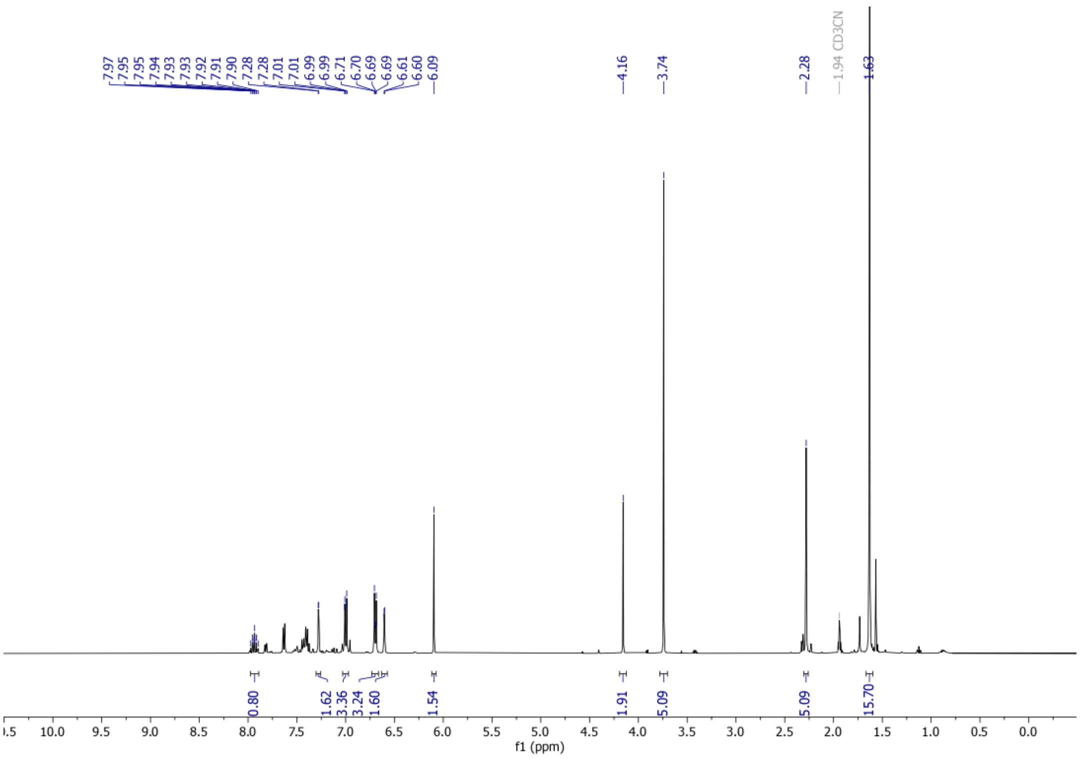

Figure S16. ¹H NMR spectrum of the reaction mixture of **4a** with pentafluoropyridine, phenyl silane and 1,3,5-trimethoxybenzene (internal standard, IS) in $CD_3CN$.

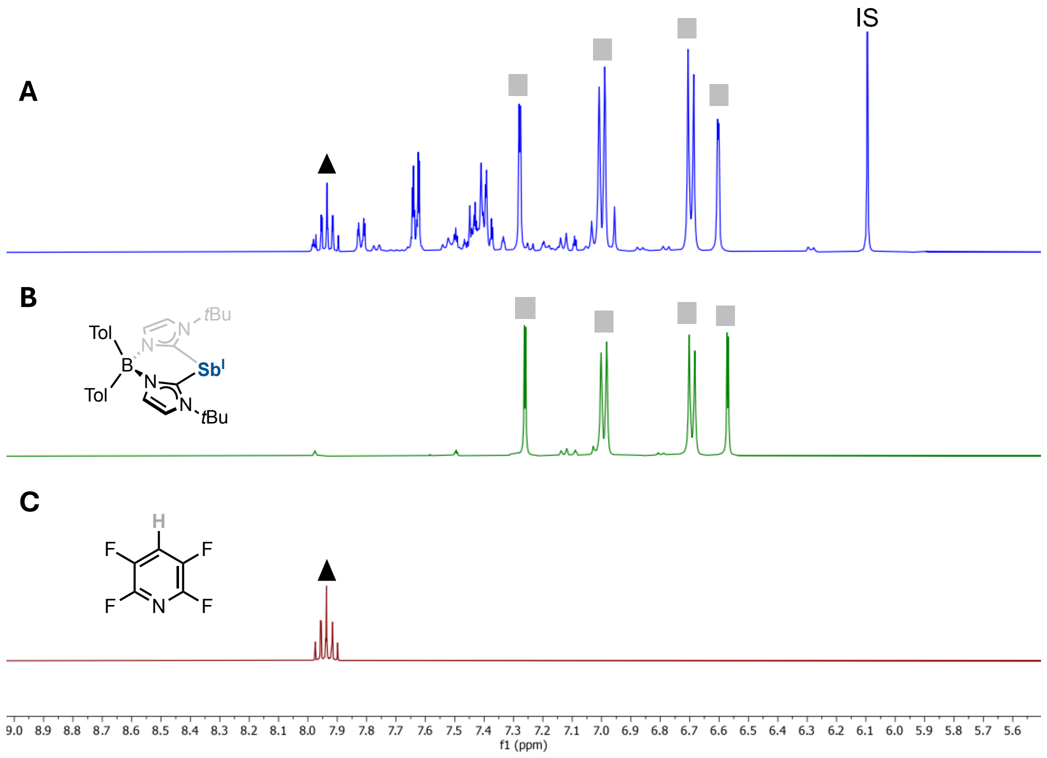


Figure S17. Stacking of the ¹H NMR spectra of (**A**) reaction mixture of **4a**, pentafluoropyridine and 1,3,5-trimethoxybenzene as internal standard (IS) after addition of phenylsilane, (**B**) **4a** (grey squares) and (**C**) 2,3,5,6-tetrafluoropyridine **13a** (black triangle) in $CD_3CN$.

## 6.4 Stoichiometric reduction of C-H bonds from 16a

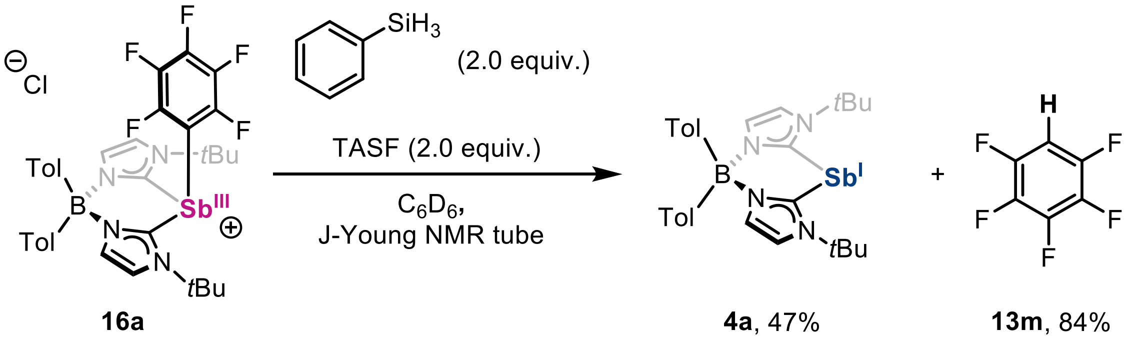


To a stirred solution of complex **16a** (8.5 mg, 1.0 equiv., 0.011 mmol) and 1,3,5-trimethoxybenzene (1.9 mg, 1.0 equiv., 0.011 mmol) as internal standard in $C_6D_6$ (0.4 mL) was added phenylsilane (2.7 μL, 2.0 equiv., 0.022 mmol). The reaction mixture was immediately transferred to a J-Young NMR tube and analysed by $^1$H NMR, which suggested no formation of pentafluorobenzene or **13m**.

To this crude mixture, a solution of tris(dimethylamino)sulfonium difluorotrimethylsilicate(IV) (6.1 mg, 2 equiv., 0.022 mmol) in $C_6D_6$ (0.2 mL) was added inside the glovebox and followed up by $^1$H NMR. After 30 minutes, pentafluorobenzene was detected in 33 % yield along with species **4a** in 7% yield. The reaction was left stirring for a further 3 h. After this time, pentafluorobenzene was detected in 42 % yield along with **4a** in 14% yield. The reaction mixture was heated at 40 °C overnight, resulting in 57% and 14% yield of pentafluorobenzene **13m** and **4a** respectively. Further heating at 60 °C overnight resulted in the formation of **4a** at 47% yield and pentafluorobenzene **13m** at 84% yield (Figure S18 and Figure S19). Black precipitate was observed during the course of the reaction, suggesting some decomposition of **4a** to Sb black.

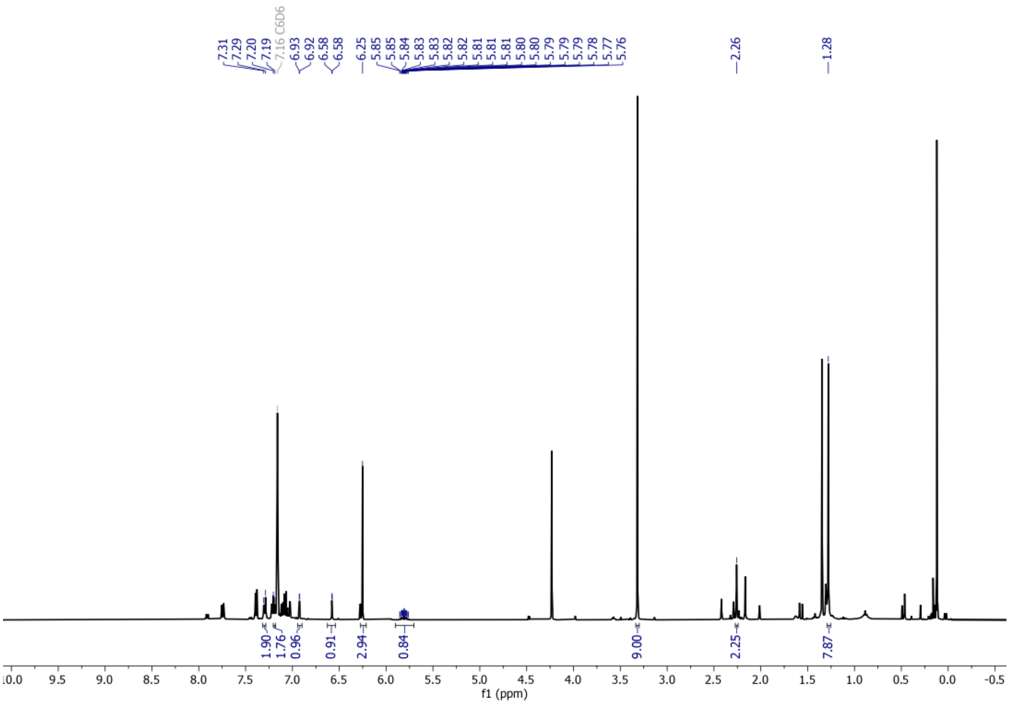


Figure S18. $^1$H NMR spectrum of the reaction mixture of isolated **16a** with phenyl silane, 1,3,5-trimethoxybenzene after addition of TASF in $C_6D_6$.

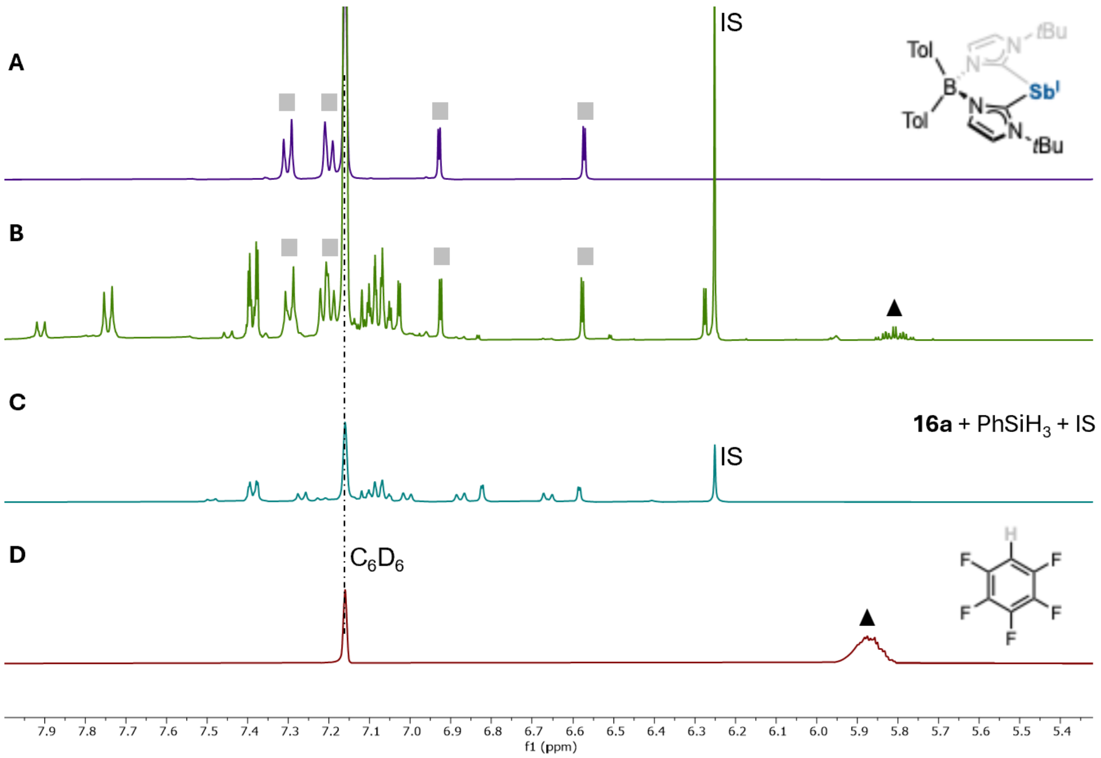


Figure S19. Stacking of the $^1$H NMR spectra of (**A**) pure **4a** (grey squares), (**B**) crude reaction of **16a**, phenyl silane and internal standard (IS) 1,3,5-trimethoxybenzene after addition of fluoride source TASF, (**C**) crude reaction of isolated **16a**, phenyl silane and internal standard (IS) 1,3,5-trimethoxybenzene and (**D**) pure **13m** (black triangle) in $C_6D_6$.

## 6.5 Stoichiometric reductive elimination of Si-S bonds from 7

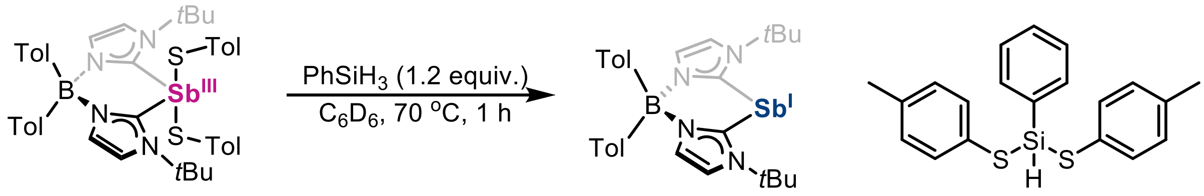


**7** **4a,** 75% **15**, >95%

To a solution of complex **7** (21 mg, 1.0 equiv., 0.025 mmol) in $C_6D_6$, phenylsilane (5.1 μL, 1.2 equiv.) was added in one portion at rt. The mixture was then transferred to a J-Young NMR tube and analysed by $^1$H NMR. No conversion of the starting materials was observed at rt after 16 h. Upon heating the same sample to 70 ºC over 1h, the $^1$H NMR spectrum indicated full conversion of **7** and the formation of a new product in 95% yield, which was identified as phenylbis(*p*-tolylthio)silane **15** (compared with isolated pure material). Silane **15** was obtained together with concomitant formation of Sb(I) complex **4a** in 75% yield and $H_2$ (peak detected at $\delta \approx 4.5$ ppm).

Analytical data for **15** (For isolation and full characterization see section 8.3):

**$^1$H NMR – (400 MHz, $C_6D_6$) δ =** 7.65 (dd, $J_{H\text{-}H}$ = 7.8, 1.8 Hz, 2H, H-8), 7.35 (d, $J_{H\text{-}H}$ = 8.2 Hz, 4H, H-4), 7.15 – 6.98 (m, 3H, H-7 + H-9), 6.74 (d, $J_{H\text{-}H}$ = 8.2 Hz, 4H, H-3), 5.97 (s, 1H, Si-H), 1.94 (s, 6H, H-1).

**$^{13}$C NMR – (101 MHz, $C_6D_6$) δ =** 137.5 (2 × C, C-5), 135.1 (4 × CH, C-4), 135.0 (2 × CH, C-8), 132.2 (C, C-6), 131.2 (CH, C-9), 130.2 (4 × CH, C-3), 128.5 (2 × CH, C-7), 126.2 (2 × C, C-2), 21.0 (2 × $CH_3$, C-1).

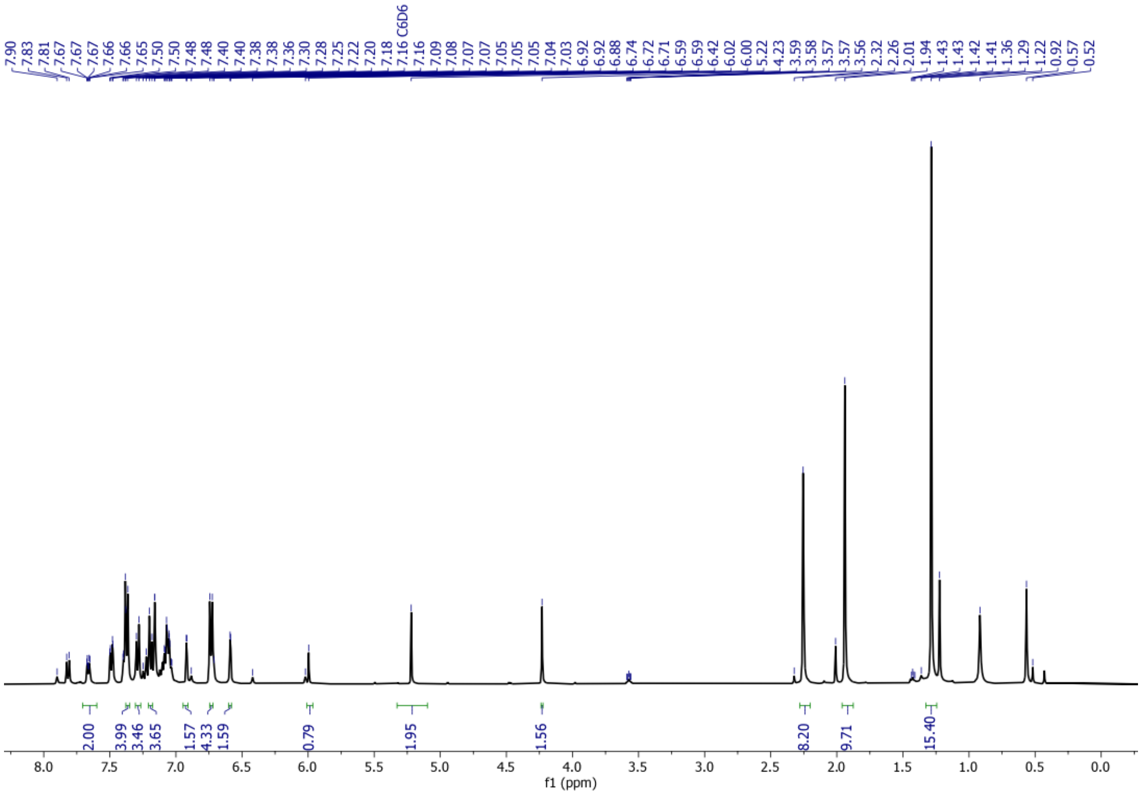

Figure S20. $^{1}$H NMR spectrum of the reaction mixture of isolated **7** with phenyl silane after 1h in $C_6D_6$.

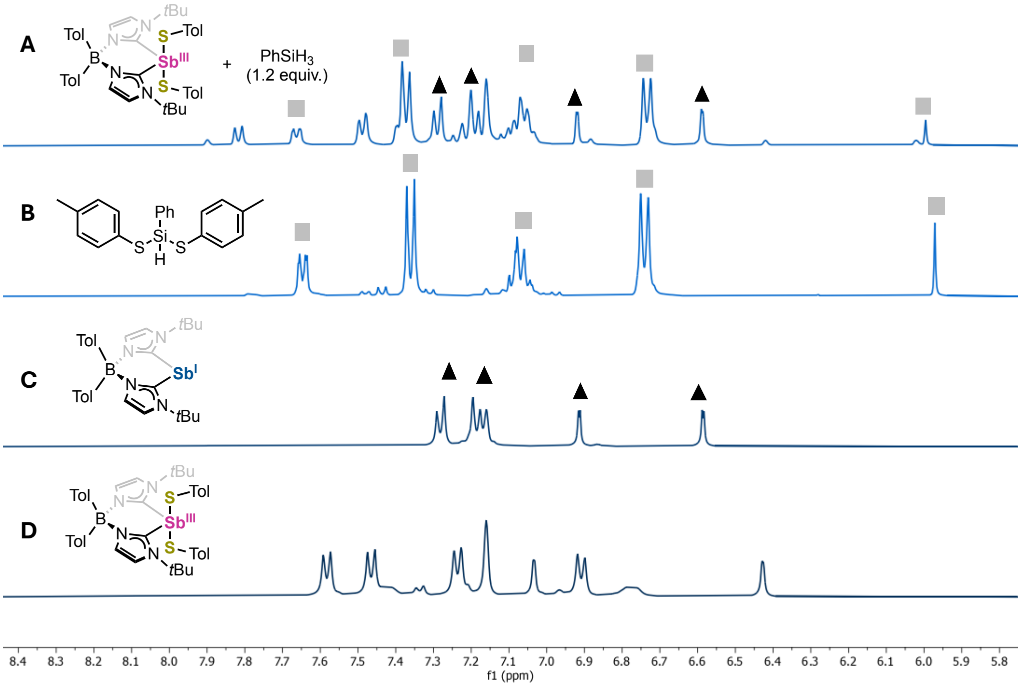


Figure S21. Stacking of the $^{1}$H NMR spectra of (**A**) crude reaction of **7** and phenyl silane (**B**) **15** (grey squares), (**C**) **4a** (black triangles) and (**D**) **7** in $C_6D_6$.

## 6.6 Stoichiometric reductive elimination of B-S bonds from 7

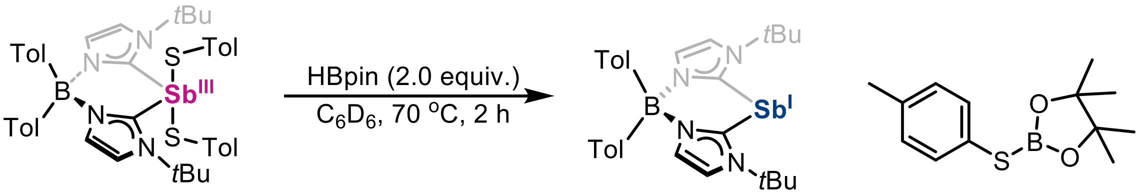


**7** (1.0 equiv.) **4a**, ~40% **14**, ~38%

To a solution of complex **7** (21 mg, 1.0 equiv., 0.025 mmol) in $C_6D_6$, pinacol borane (5.1 mL, 2.0 equiv.) was added in one portion at rt. The mixture was then transferred to a J-Young NMR tube and analysed by $^1$H NMR. No conversion of the starting materials was observed at rt after 16 h. Upon heating the same sample to 70 °C over 2h, the $^1$H NMR spectrum indicated partial conversion of **7** to **4a** (approximately 40%) and the formation of a new product, which was identified as 4,4,5,5-tetramethyl-2-(*p*-tolylthio)-1,3,2-dioxaborolane **14** (approximately 38%) compared with reported sample).[9] The quality of the NMR spectra could not be improved after several trials and the presence of impurities and decomposition products made very difficult the quantification of products (see Figure S22).

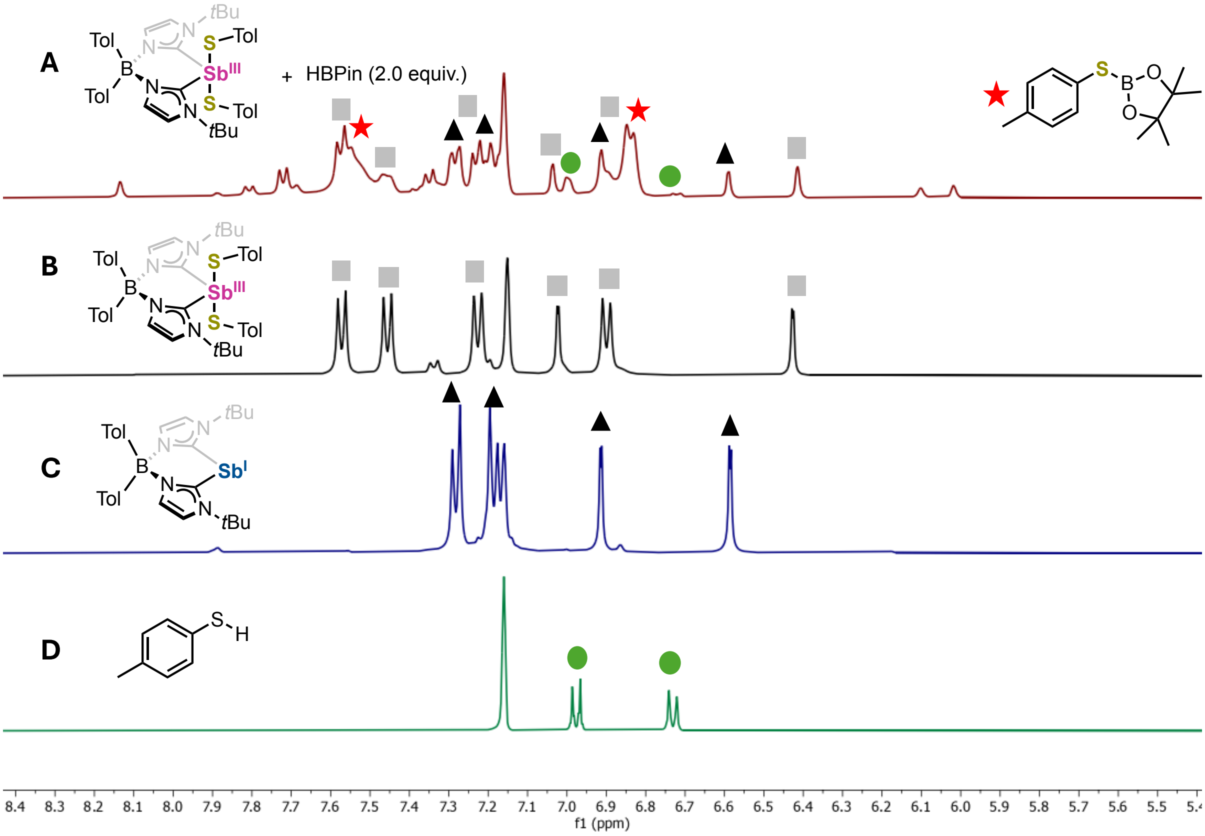


Figure S22. Stacking of the $^1$H NMR spectra of (**A**) crude reaction of **7** and phenyl silane, showing the formation of 4,4,5,5-tetramethyl-2-(*p*-tolylthio)-1,3,2-dioxaborolane **14** (red stars) (**B**) **7** (grey squares), (**C**) **4a** (black triangles) and (**D**) **p-thiocresol** (green circles) in $C_6D_6$.

# 7 Catalytic hydrodefluorination reactions

## 7.1 General Method

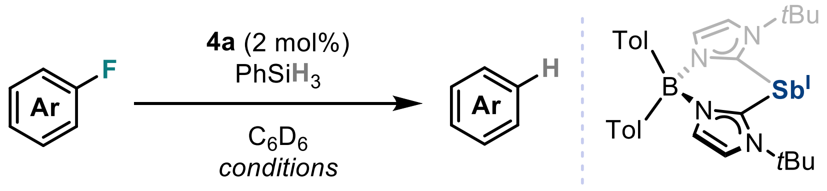


0.3 mmol

**4a**

To a solution of the corresponding perfluoro(hetero)arene (0.30 mmol) and phenylsilane (1.0 to 4.0 equiv.) in benzene (0.25 mL) was added a solution of the Sb(I) complex **4a** (2 mol%) in benzene (0.25 mL) as catalyst. After this, $\alpha,\alpha,\alpha$-trifluorotoluene (0.10 mmol) was added to the mixture as internal standard and the reaction was monitored by $^{19}F$ NMR.

## 7.2 Optimisation of reaction conditions

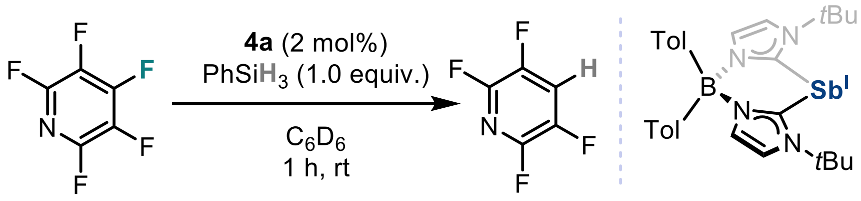


0.3 mmol **13a** **4a**

Table S1. Table showing the optimisation of the catalytic hydrodefluorination of pentafluoropyridine.

| Entry | Deviation from standard conditions | Yield of 13a (%)[a] |
|---|---|---|
| 1 | none | 81 |
| 2 | No $PhSiH_3$ | 0 |
| 3 | 0.5 equiv. $PhSiH_3$ | 67 |
| 4 | 1.0 equiv. TMDSO and 2 h | 73 |
| 5 | 1.0 equiv. $Et_3SiH$ instead of $PhSiH_3$ | 0 |
| 6 | 3.0 equiv. $Ph_3SiH$ instead of $PhSiH_3$ | 13 |
| 7 | 1.0 equiv. $Ph_3SiH$ instead of $PhSiH_3$ | 10 |
| 8 | 1.5 equiv. $Ph_2SiH_2$ instead of $PhSiH_3$ | 24 |
| 9 | 1.0 equiv. $Ph_2SiH_2$ instead of $PhSiH_3$ | 19 |
| 10 | No **4a** | 0 |
| 11 | 0.5 mol% **4a** | 4 |
| 12 | 5 mol% **4a** | >95 |
| 13 | 2 h | >95 |
| 14 | **4b** instead of **4a** | 44 |
| 15 | **2a** instead of **4a** | 0 |
| 16 | **5a** instead of **4a** | 71 |
| 17 | **5a** instead of **4a**, 2 h | >95 |
| 18 | TAS-F instead of **4a** | 0 |
| 19 | *N,C,N*-Sb(I)[9] instead of **4a** | 50 |

[a]Calculated by $^{19}F$ NMR using $\alpha,\alpha,\alpha$-trifluorotoluene (0.33 equiv.) as IS.

## 7.3 Results and spectroscopic data for hydrodefluorination products

### 7.3.1 2,3,5,6-tetrafluoropyridine (**13a**)

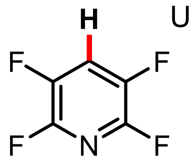


Using the general method with pentafluoropyridine and 1.0 equiv. phenylsilane resulted in the formation of **13a** at >95% NMR yield after 2 h at rt.

**$^{19}$F NMR – (377 MHz, $C_6D_6$) δ =** -91.40 – -91.87 (m, 2F), -139.55 – -140.70 (m, 2F).

### 7.3.2 1,2,4,5-tetrafluoro-3-(trifluoromethyl)benzene (**13b**)

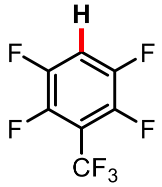


Using the general method with 1,2,3,4,5-pentafluoro-6-(trifluoromethyl)benzene and 1.0 equiv. phenylsilane resulted in the formation of **13b** at >95% NMR yield after 4 h at rt.

**$^{19}$F NMR – (377 MHz, $C_6D_6$) δ =** -56.84 (t, *J* = 22.1 Hz, 3F), -136.99 – -137.25 (m, 2F), -140.92 – -141.82 (m, 2F)

### 7.3.3 Methyl 2,3,5,6-tetrafluorobenzoate (**13c**)

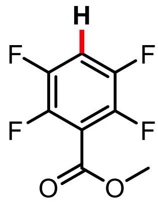


Using the general method with 1.0 equiv. phenylsilane resulted in the formation of **13c** at 79% NMR yield after 16 h at 60 °C.

**$^{19}$F NMR – (377 MHz, $C_6D_6$) δ =** -138.01 – -138.29 (m, 2F), -140.31 – -140.50 (m, 2F).

### 7.3.4 2,3,5,6-tetrafluorobenzonitrile (**13d**)

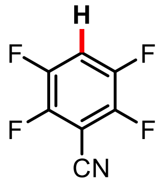


Using the general method with 1.0 equiv. phenylsilane resulted in the formation of **13d** at >95% NMR yield after 2 h at rt.

**$^{19}$F NMR – (377 MHz, $C_6D_6$) δ =** -133.32 – -133.47 (m, 2F), -136.29 – -136.53 (m, 2F)

### 7.3.5 2,5-difluoroisophthalonitrile (**13e**)

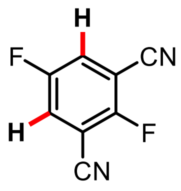


Using the general method with 2.0 equiv. phenylsilane resulted in the formation of **13e** at >95% NMR yield after 1 h at rt. The product was isolated in 93% yield running the reaction at 0.65 mmol scale and the obtained spectral data agreed with previous reports.[10]

**$^{1}$H NMR – (400 MHz, $C_6D_6$) δ =** 6.21 (dd, *J* = 7.1, 4.9 Hz, 2H).

**$^{19}$F NMR – (377 MHz, $C_6D_6$) δ =** -107.11 (d, *J* = 16.2 Hz), -112.77 (d, *J* = 16.2 Hz).

### 7.3.6 Tris(2,3,5,6-tetrafluorophenyl)phosphane (**13f**)

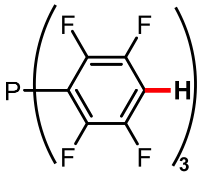

Using the general method with 3.0 equiv. phenylsilane resulted in the formation of **13f** at 29% NMR yield after 4 d at 60 °C.

**$^{19}$F NMR – (377 MHz, $C_6D_6$) δ =** -130.26 – -130.70 (m, 6F), -136.75 – -137.50 (m, 6F)

### 7.3.7 2,2',3,3',5,5',6,6'-octafluoro-1,1'-biphenyl (**13g**)

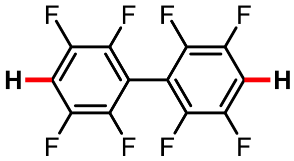

Using the general method with 2.0 equiv. phenylsilane resulted in the formation of **13g** at 79% NMR yield after 16 h at rt.

**$^{19}$F NMR – (377 MHz, $C_6D_6$) δ =** -137.70 – -138.02 (m, 4F), -139.10 – -139.30 (m, 4F)

### 7.3.8 1,2,3,4,5,6,8-heptafluoronaphthalene (**13h**) and 1,2,4,5,6,8-hexafluoronaphthalene (**13i**)

Using the general method with 2.0 equiv. phenylsilane resulted in the formation of

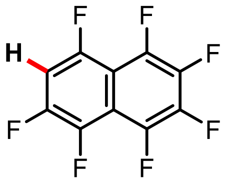

**13h** at 61% NMR yield after 2 d at 60 °C.

**$^{19}$F NMR – (377 MHz, $C_6D_6$) δ =** -117.77 – -119.26 (m), -135.45 – -138.00 (m), -146.78 (ddd, $J$ = 64.4, 19.0, 15.2 Hz), -148.04 – -148.89 (m), -151.83 (dddd, $J$ = 58.2, 23.6, 13.4, 5.0 Hz), -154.90 – -156.30 (m), -158.37 – -159.66 (m).

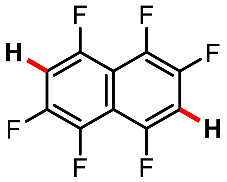

**13i** at 24% NMR yield after 2 d at 60 °C.

**$^{19}$F NMR – (377 MHz, $C_6D_6$) δ =** -119.11 – -119.91 (m), -137.57 – -140.61 (m), -150.05 – -151.21 (m).

Together with traces of 1,2,3,4,5,8-hexafluoronaphthalene (**13j**) and unreacted perfluoronaphtalene (12%).

### 7.3.9 Unsuccessful substrates

The following substrates were obtained in less than 10% yield under 4 days at 60 ºC: 2,4,6-trifluoropyridine, 2,4,6-trifluorobenzonitrile, 2,3,4,5,6-pentafluoro-1,1'-biphenyl, perfluorobenzene and 1-bromo-2,3,4,5,6-pentafluorobenzene and 1-fluoroheptane

Table S2. Products obtained in low yields or trace (<5%) amounts.

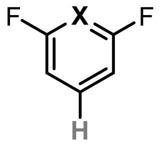

X = N, **13j**, <5%
X = CCN, **13k**, <5%

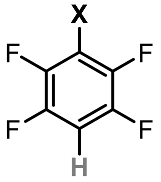

X = Ph, **13l**, <5%
X = F, **13m**, <5%
X = Br, **13n**, <5%
X = H, **13o**, <5%
X = $NH_2$, **13p**, <5%

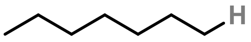

**13q**, <5%

## 7.4 Radical trap tests for HDF reaction

Different radical trap inhibition experiments were conducted to explore the mechanism of this transformation. The HDF of pentafluoropyridine with 1 equiv. of the reagents listed below could proceed smoothly affording the expected products in the yields shown below, suggesting that SET processes do not take place in the present transformation.

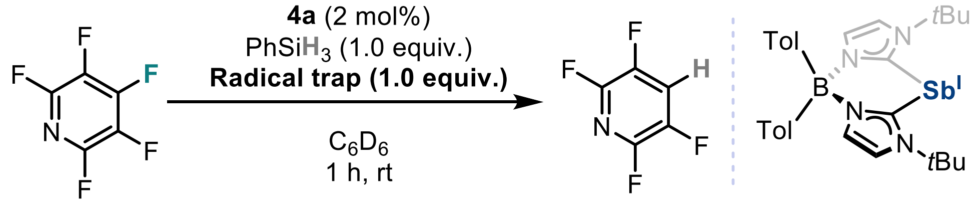


0.3 mmol **13a** **4a**

Table S3. Table showing catalytic hydrodefluorination of pentafluoropyridine using **4a** in the presence of different radical traps

| Entry | Reagent | Yield (%)[a] | Note |
| --- | --- | --- | --- |
| 1 | 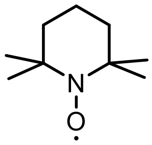 | >95 | / |
| 2 | 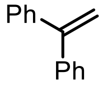 | >95 | / |
| 3 | 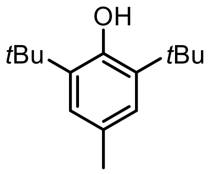 | 53 | >95 after 16 h at rt |
| 4 | $P(OEt)_3$ | 42 | 77 after 16 h at rt |
| 5 | 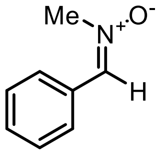 | 52 | >95 after 16 h at rt |
| 6 | $B_2pin_2$ | <5 | $B_2pin_2$ is known to react with fluoride anions,[11] quenching reactivity towards hydrodefluorination. See *section 6.11*. |

[a]Estimated by $^{19}F$ NMR using $\alpha,\alpha,\alpha$-trifluorotoluene (0.33 equiv.) as IS.

## 7.5 Radical trap test for Oxidative Addition to Perfluoropyridine

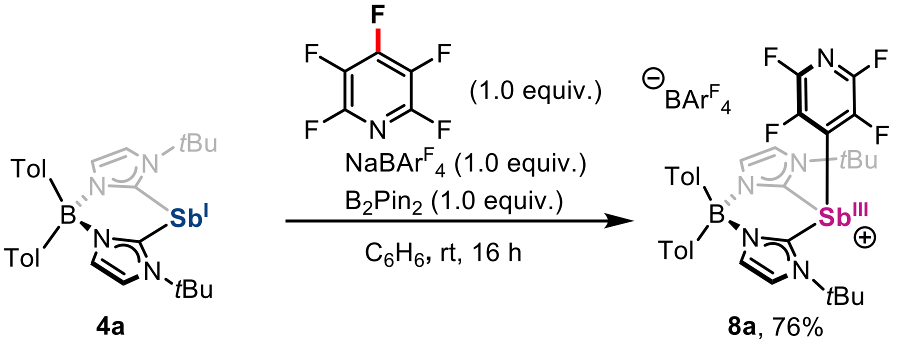


The oxidative addition of pentafluoropyridine to stibidine **4a** was performed as described previously (see section 5.7) but in the presence of $B_2pin_2$ (1 equivalent) as radical scavenger and $\alpha,\alpha,\alpha$-trifluorotoluene (0.33 equivalent) was added as internal standard. The corresponding oxidative addition product **8a** was observed in 69% yield.

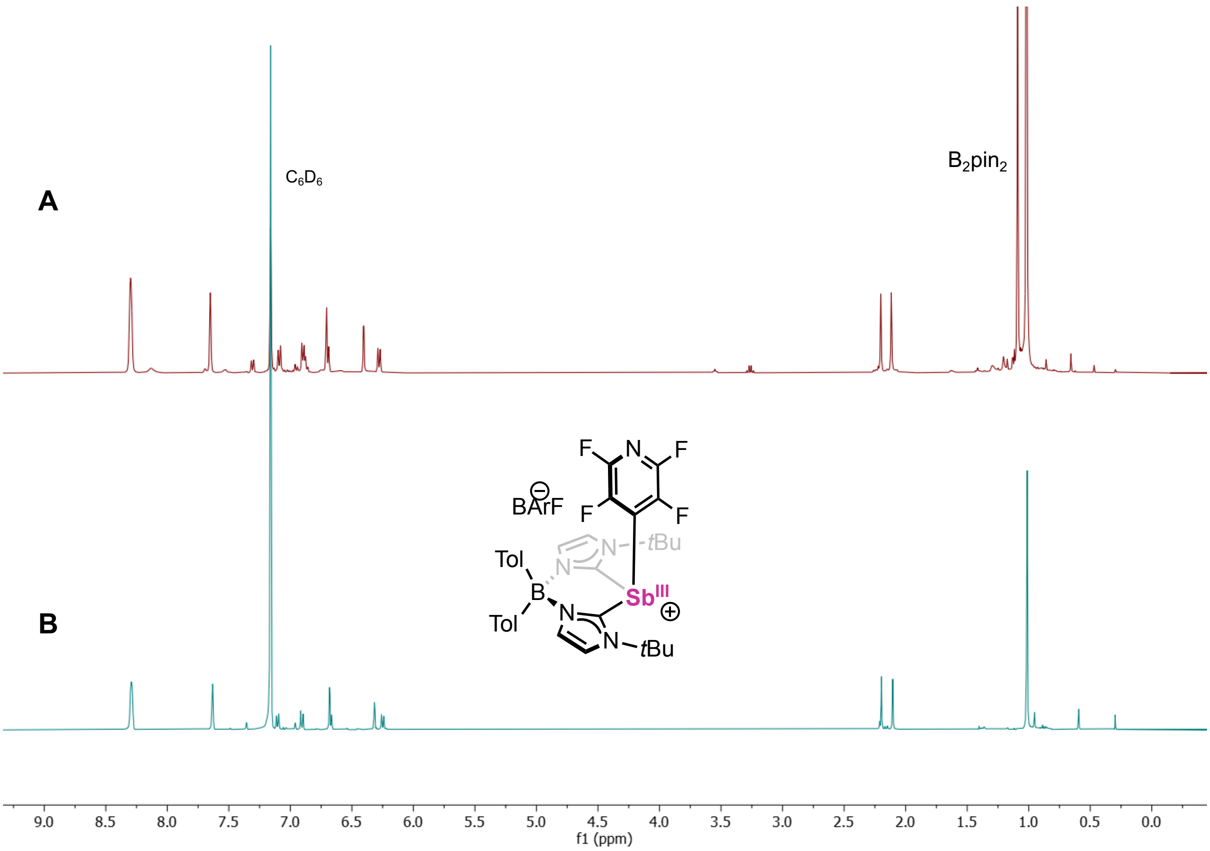


Figure S23. Stacking of the $^{1}$H NMR spectra of (**A**) crude reaction of **4a** and pentafluoropyridine in the presence of $B_2pin_2$, showing the formation of Sb(III) complex **8a**, and (**B**) isolated **8a** in $C_6D_6$.

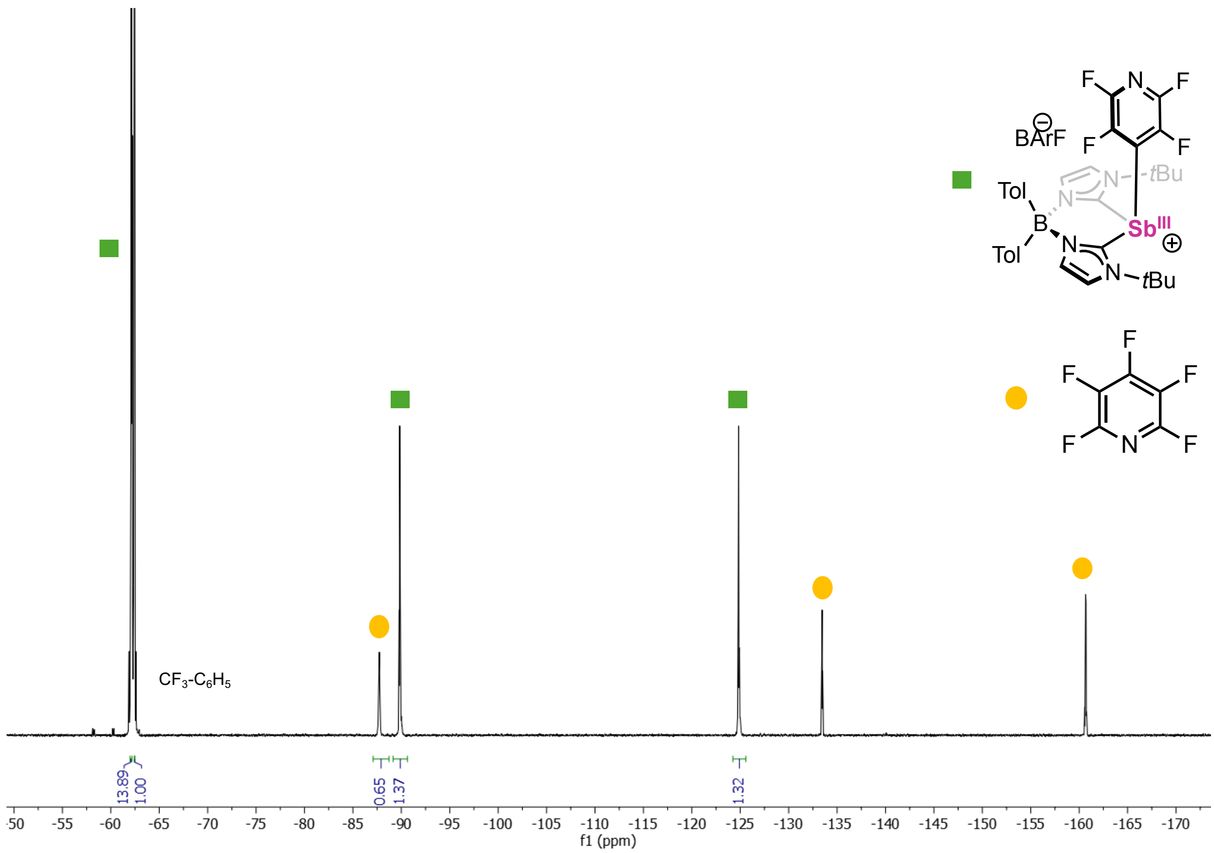


Figure S24. $^{19}F$ NMR spectra of crude reaction of **4a** and pentafluoropyridine in the presence of $B_2pin_2$, showing the formation of Sb(III) complex **8a** in ca. 69% yield in $C_6D_6$.

## 7.6 Fluoride scavenging tests for HDF reaction

Different fluoride inhibition experiments were conducted to explore the mechanism of this transformation. The HDF of pentafluoropyridine with 1 equiv. of the reagents listed below proceed in the presence of those with a lower Fluoride Ion Affinity number (FIA), affording the expected products in the yields shown below, while those reagents with higher FIA numbers completely inhibited it. These results suggest that the participation of s $F^-$ anion is key for the present transformation.

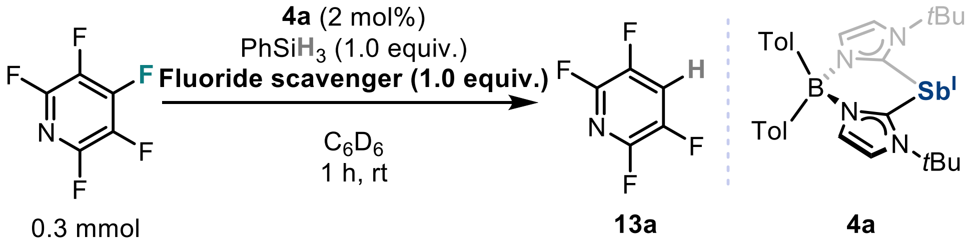


Table S4. Table showing catalytic hydrodefluorination of pentafluoropyridine using **4a** in the presence of fluoride scavengers

| Entry | Reagent | FIA | Yield (%)[a] |
|---|---|---|---|
| 1 | $Al(OTf)_3$ | 368[12] | - |
| 2 | $BF_3$ | 258[12] | - |
| 3 | $B_2pin_2$ | 247[13] | 9 |
| 4 | $BBu_3$ | 132 for $BMe_3$[12] | 47 |
| 5 | PhBpin | 81[14] | >95 |

[a]Estimated by $^{19}$F NMR using $\alpha,\alpha,\alpha$-trifluorotoluene (0.33 equiv.) as IS.

## 7.7 Unsuccessful reagents for C-X/C-C couplings

Following a similar methodology to the described General Method for the catalytic HDF of perfluoro(hetero)arenes (see Section 7.1), different reagents were tested aiming at expanding the scope of this transformation to C-X and C-C cross-coupling reagents. However, these transformations proved unsuccessful.

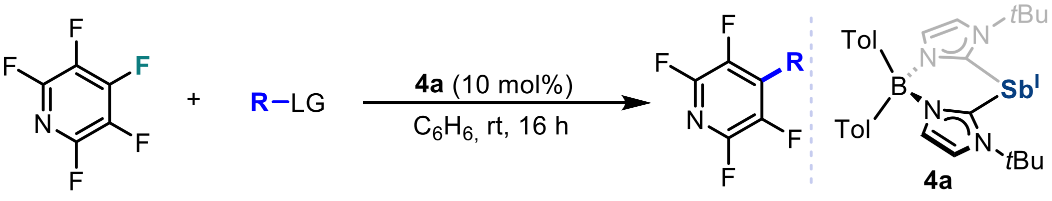


Table S5. Table showing attempts of C-C/C-X couplings to pentafluoropyridine with 10 mol% **4a**

| Entry | Reagent | Yield (%)[a] | Note |
|---|---|---|---|
| 1 | 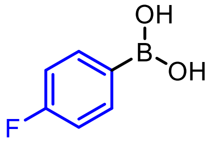 | NR | / |
| 2 | 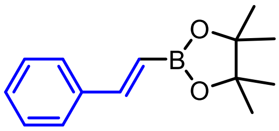 | NR | / |
| 3 | 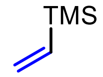 | NR | / |
| 4 | 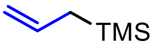 | NR | / |
| 5 | 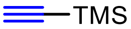 | NR | / |
| 6 | 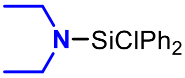 | NR | Reported to react at higher T in absence of catalyst[15] |
| 7 | 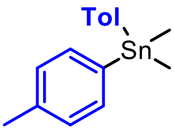 | NR | / |
| 8 | 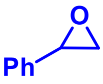 | NR | / |
| 9 | 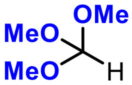 | NR | / |

[a]Estimated by $^{19}F$ NMR using $\alpha,\alpha,\alpha$-trifluorotoluene (0.33 equiv.) as IS.

## 8 Dehydrogenative dithiolation of silanes

### 8.1 General method

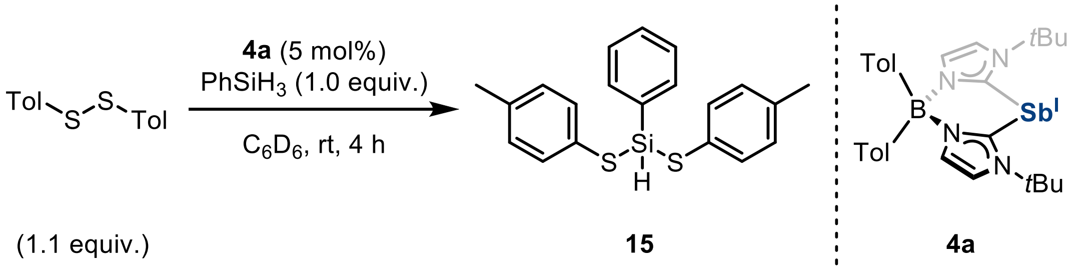


(1.1 equiv.) **15** **4a**

To a vial containing a mixture of disulfide (1.1 equiv., 0.220 mmol) and phenylsilane (24.8 μL, 1 equiv., 0.200 mmol), a solution of Sb(I) complex **4a** (5.6 mg, 5 mol%, 0.010 mmol) in $C_6H_6$ (1 mL) was added in one portion at rt. After stirring for 4 h at the same temperature, the solvent was removed under vacuum and the crude redissolved in $C_6D_6$. The crude mixture was characterised directly due to the instability of product **15** to different purification methods (e.g. filtration over celite, silica, alumina, deactivated alumina, extraction).

The yields were determined in a different experiment, repeating the procedure mentioned above, adding TMB (11.1 mg, 0.33 equiv., 0.066 mmol) as IS at the end of the reaction and analysing the crude mixture by $^1$H NMR in $C_6D_6$.

### 8.2 Optimisation of the Sb(I)-Sb(III) redox catalytic dehydrogenative dithiolation of silanes with *p*-tolyl disulfide

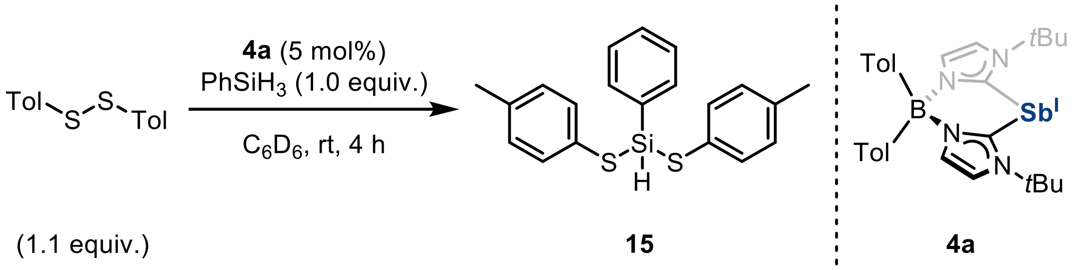


(1.1 equiv.) **15** **4a**

Table S6. Table showing the optimisation of the catalytic dehydrogenative silylation of *p*-tolyl disulfide.

| Entry | Deviation from standard conditions | Yield of 15 (%)[a] |
|---|---|---|
| 1 | None | >95 |
| 2 | No **4a** | 0 |
| 3 | **4b** instead of **4a** | 78 |
| 4 | **5a** instead of **4a** | 10 |
| 5 | **2a** instead of **4a** | 0 |
| 6 | In presence of **$B_2$pin$_2$** (1.1 equiv.) | 73 |

[a]Estimated by $^1$H NMR using 1,3,5-trimethoxybenzene (0.33 equiv.) as IS.

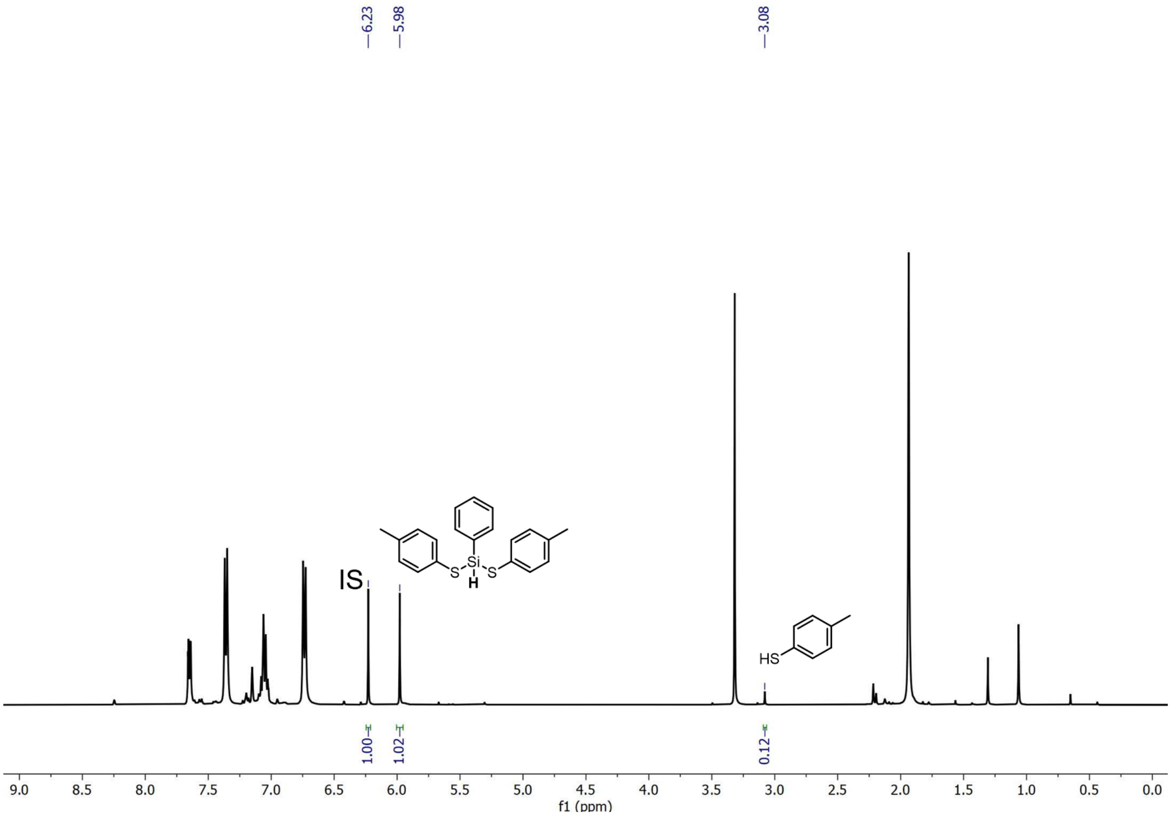


Figure S25. $^1$H NMR Spectra showing the crude reaction of catalytic dehydrogenative silylation of *p*-tolyl disulfide in $C_6D_6$ (see entry 1 in Table S6).

## 8.3 Results and spectroscopic data for products from dehydrogenative thiolation

### 8.3.1 Phenylbis(*p*-tolylthio)silane (**15**)

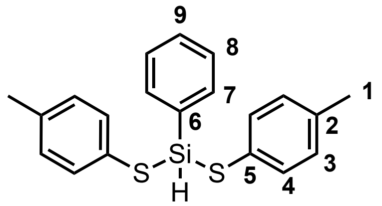


The crude yield for this compound was found to be >95% using the general procedure. In an attempt to isolate a significant amount, a multigram scale reaction was performed using the general procedure from phenylsilane (0.50 mL, 4.00 mmol, 1.0 equiv.), di-*p*-tolyl disulfide (1.03 g, 4.20 mmol, 1.05 equiv.), Sb(I) complex **4a** (112 mg, 0.20 mmol, 0.05 equiv.) and benzene (10 mL). Then, after removal of the volatile species under vacuum, the product **15** was isolated as a colourless oil (1.20 g, 4.00 mmol, 85%) by vacuum distillation (220 ºC, 0.005 mbar). Due to its air- and moisture-sensitivity, some decomposition products could be observed in $^{1}$H NMR, but the purity of the product was enough to unambiguously assign the obtained peaks.

**$^{1}$H NMR – (400 MHz, $C_6D_6$) δ =** 7.65 (dd, $J_{H\text{-}H}$ = 7.8, 1.8 Hz, 2H, H-8), 7.35 (d, $J_{H\text{-}H}$ = 8.2 Hz, 4H, H-4), 7.15 – 6.98 (m, 3H, H-7 + H-9), 6.74 (d, $J_{H\text{-}H}$ = 8.2 Hz, 4H, H-3), 5.97 (s, 1H, Si-H), 1.94 (s, 6H, H-1).

**$^{13}$C NMR – (101 MHz, $C_6D_6$) δ =** 137.5 (2 × C, C-5), 135.1 (4 × CH, C-4), 135.0 (2 × CH, C-8), 132.2 (C, C-6), 131.2 (CH, C-9), 130.2 (4 × CH, C-3), 128.5 (2 × CH, C-7), 126.2 (2 × C, C-2), 21.0 (2 × $CH_3$, C-1).

**HRMS (ASAP-HESI, m/z, m/z):** calc'd for $[M]^+$ 352.0776; Peaks corresponding to **15** could not be assigned, possibility due to decomposition of **15** under analysis conditions.

## 9 Crystallographic data

### 9.1 Single crystal structure analysis of 1c

CCDC Deposition number 2423149

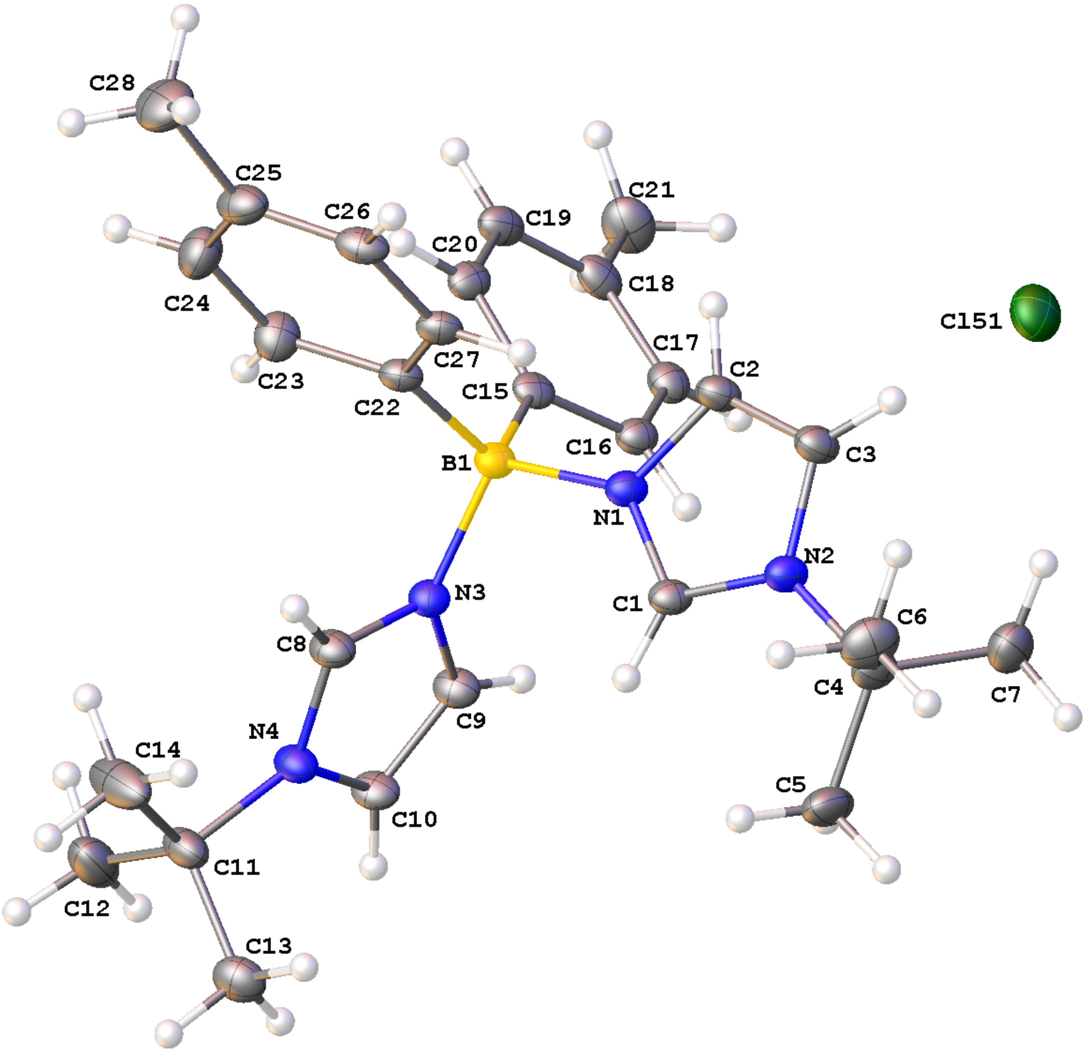


Figure S26. Olex-2 representation of the molecular structure of ligand **1c**

#### 9.1.1 Experimental

Single colourless plate-shaped crystals of **1c** The crystal used as supplied. A suitable crystal with dimensions 0.03 × 0.03 × 0.01 $mm^3$ was selected and mounted on a MITIGEN holder in oil on a Rigaku 007HF diffractometer equipped with Arc)Sec VHF Varimax confocal mirrors and a UG2 goniometer and HyPix Arc-100 detector diffractometer. The crystal was kept at a steady $T$ = 100(2) K during data collection. The structure was solved with the ShelXT 2018/2 (Sheldrick, 2015) solution program using dual methods and by using Olex2 1.5-dev (Dolomanov et al., 2009) as the graphical interface. The model was refined with olex2.refine 1.5-dev (Bourhis et al., 2015) using full matrix least squares minimisation on $F^2$.

### 9.1.2 Crystal Data

$C_{28}H_{38}BN_4Cl$, $M_r$ = 476.918, monoclinic, *I*2/*a* (No. 15), a = 22.6761(6) Å, b = 11.8252(3) Å, c = 20.2428(5) Å, $\beta$ = 95.740(2)°, $\alpha = \gamma$ = 90°, $V$ = 5400.9(2) Å$^3$, $T$ = 100.(2) K, $Z$ = 8, $Z'$ = 1, $\mu$(Cu $K_\alpha$) = 1.410, 51580 reflections measured, 5425 unique ($R_{int}$ = 0.0562) which were used in all calculations. The final $wR_2$ was 0.1074 (all data) and $R_1$ was 0.0409 (I≥2 $\sigma$(I)).

| Compound | **1c** |
|---|---|
| | |
| Formula | $C_{28}H_{38}BN_4Cl$ |
| $D_{calc.}$/ g cm$^{-3}$ | 1.173 |
| $\mu$/mm$^{-1}$ | 1.410 |
| Formula Weight | 476.918 |
| Colour | colourless |
| Shape | plate-shaped |
| Size/mm$^3$ | 0.03×0.03×0.01 |
| *T*/K | 100.(2) |
| Crystal System | monoclinic |
| Space Group | *I*2/*a* |
| *a*/Å | 22.6761(6) |
| *b*/Å | 11.8252(3) |
| *c*/Å | 20.2428(5) |
| $\alpha$/° | 90 |
| $\beta$/° | 95.740(2) |
| $\gamma$/° | 90 |
| V/Å$^3$ | 5400.9(2) |
| *Z* | 8 |
| *Z'* | 1 |
| Wavelength/Å | 1.54184 |
| Radiation type | Cu $K_\alpha$ |
| $\Theta_{min}$/° | 3.92 |
| $\Theta_{max}$/° | 74.02 |
| Measured Refl's. | 51580 |
| Indep't Refl's | 5425 |
| Refl's I≥2 $\sigma$(I) | 4381 |
| $R_{int}$ | 0.0562 |
| Parameters | 315 |
| Restraints | 0 |
| Largest Peak | 0.4307 |
| Deepest Hole | -0.3757 |
| GooF | 1.0395 |
| $wR_2$ (all data) | 0.1074 |
| $wR_2$ | 0.0999 |
| $R_1$ (all data) | 0.0553 |
| $R_1$ | 0.0409 |

### 9.1.3 Structure Quality Indicators

| | | | | |
|---|---|---|---|---|
| Reflections: | d min (CuKα) 2Θ=148.0° 0.80 | I/σ(I) 37.6 | Rint 5.62% | Full 135.4° 99% to 148.0° 100 |
| Refinement: | Shift 0.001 | Max Peak 0.4 | Min Peak -0.4 | GooF 1.040 |

A colourless plate-shaped crystal with dimensions 0.03 × 0.03 × 0.01 $mm^3$ was mounted on a MITIGEN holder in oil. Data were collected using a Rigaku 007HF diffractometer equipped with Arc)Sec VHF Varimax confocal mirrors and a UG2 goniometer and HyPix Arc-100 detector diffractometer equipped with an Oxford Cryosystems low-temperature device operating at $T$ = 100.(2) K.

Data were measured using profile data from $\omega$-scans with Cu $K_\alpha$ radiation. The diffraction pattern was indexed and the total number of runs and images was based on the strategy calculation from the program CrysAlisPro system (CCD 43.134a 64-bit (release 09-08-2024)). The maximum resolution that was achieved was $\Theta$ = 74.02° (0.80 Å).

The unit cell was refined using CrysAlisPro 1.171.43.135a (Rigaku OD, 2024) on 18259 reflections, 35% of the observed reflections.

Data reduction, scaling and absorption corrections were performed using CrysAlisPro 1.171.43.135a (Rigaku OD, 2024). The final completeness is 100.00 % out to 74.02° in $\Theta$. A multi-scan absorption correction was performed using CrysAlisPro 1.171.43.135a (Rigaku OD, 2024) Empirical absorption correction using spherical harmonics, implemented in SCALE3 ABSPACK scaling algorithm. The absorption coefficient $\mu$ of this material is 1.410 $mm^{-1}$ at this wavelength ($\lambda$ = 1.54184Å) and the minimum and maximum transmissions are 0.935 and 1.000.

The structure was solved and the space group *I*2/*a* (# 15) determined by the ShelXT 2018/2 (Sheldrick, 2015) structure solution program using dual methods and refined by full matrix least squares minimisation on $F^2$ using version of olex2.refine 1.5-dev (Bourhis et al., 2015). All non-hydrogen atoms were refined anisotropically. Hydrogen atom positions were calculated geometrically and refined using the riding model.

*_exptl_absorpt_process_details*: CrysAlisPro 1.171.43.135a (Rigaku OD, 2024) using spherical harmonics, implemented in SCALE3 ABSPACK scaling algorithm.

There is a single formula unit in the asymmetric unit, which is represented by the reported sum formula. In other words: Z is 8 and Z' is 1. The moiety formula is C28 H38 B N4, Cl.

## 9.1.4 Data Plots: Diffraction Data

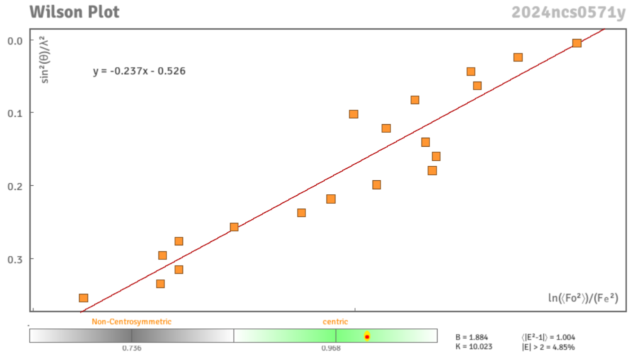


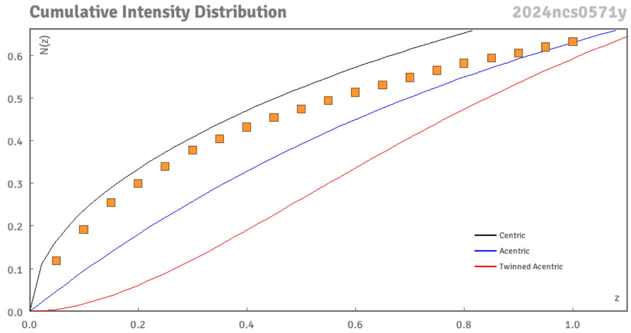


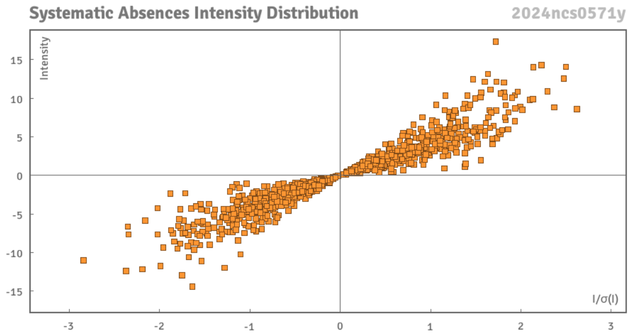


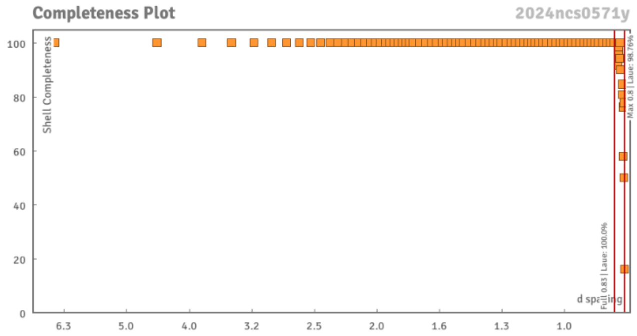


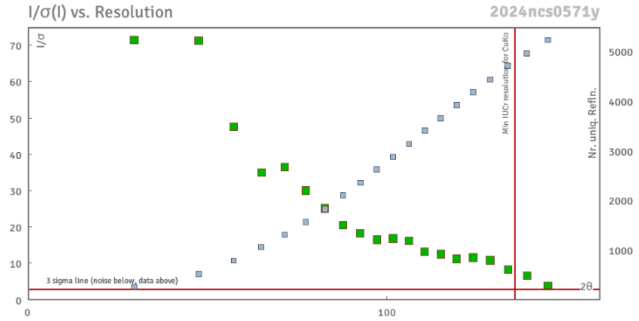


## 9.1.5 Data Plots: Refinement and Data

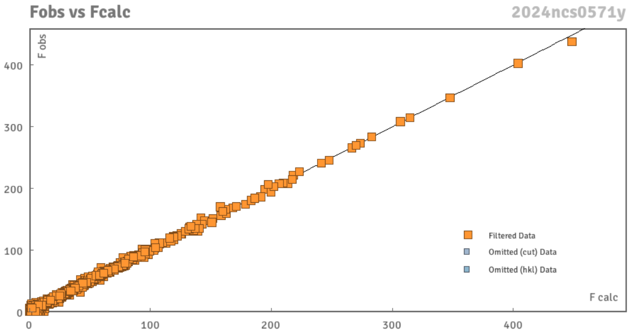


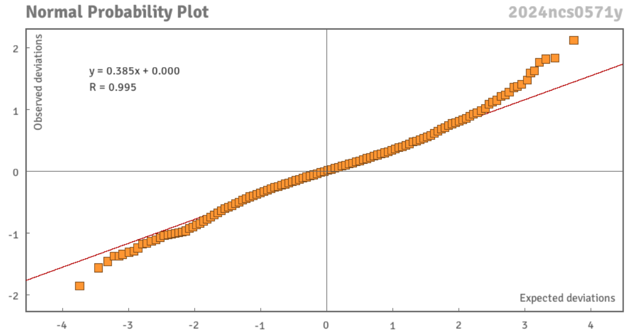

### 9.1.6 Reflection Statistics

| | | | |
|---|---|---|---|
| Total reflections (after filtering) | 53094 | Unique reflections | 5425 |
| Completeness | 0.988 | Mean I/$\sigma$ | 24.36 |
| $hkl_{max}$ collected | (27, 14, 23) | $hkl_{min}$ collected | (-27, -14, -25) |
| $hkl_{max}$ used | (27, 14, 25) | $hkl_{min}$ used | (-27, 0, 0) |
| Lim $d_{max}$ collected | 100.0 | Lim $d_{min}$ collected | 0.77 |
| $d_{max}$ used | 15.83 | $d_{min}$ used | 0.8 |
| Friedel pairs | 6031 | Friedel pairs merged | 1 |
| Inconsistent equivalents | 0 | $R_{int}$ | 0.0562 |
| $R_{sigma}$ | 0.0266 | Intensity transformed | 0 |
| Omitted reflections | 0 | Omitted by user (OMIT hkl) | 0 |
| Multiplicity | (4381, 4427, 2600, 1701, 1103, 682, 456, 367, 275, 182, 122, 72, 75, 42, 37, 24, 14, 8, 5, 2) | Maximum multiplicity | 37 |
| Removed systematic absences | 1514 | Filtered off (Shel/OMIT) | 0 |

### 9.1.7 Fractional Atomic Coordinates and Equivalent Isotropic Displacement Parameters

Table S7. Fractional Atomic Coordinates (×$10^4$) and Equivalent Isotropic Displacement Parameters (Å$^2$×$10^3$) for **1c**. $U_{eq}$ is defined as 1/3 of the trace of the orthogonalised $U_{ij}$.

| Atom | x | y | z | $U_{eq}$ |
|---|---|---|---|---|
| N1 | 5829.8(6) | 5112.3(11) | 3922.4(6) | 17.0(3) |
| N2 | 5564.4(6) | 4239.2(12) | 2989.1(6) | 19.8(3) |
| N3 | 6630.4(5) | 6600.2(11) | 4184.1(6) | 16.8(3) |
| N4 | 7111.5(6) | 7981.5(11) | 3747.9(7) | 20.1(3) |
| C1 | 5903.4(7) | 5064.8(14) | 3277.7(8) | 18.6(3) |
| C2 | 5419.4(7) | 4286.9(14) | 4038.0(8) | 20.9(3) |
| C3 | 5248.7(7) | 3751.0(14) | 3465.5(8) | 21.7(3) |
| C4 | 5537.0(8) | 3840.7(15) | 2283.7(8) | 23.6(4) |
| C5 | 6056.2(9) | 4337.6(17) | 1961.3(9) | 32.3(4) |
| C6 | 4950.9(8) | 4242.4(18) | 1925.2(9) | 32.9(4) |
| C7 | 5572.2(9) | 2550.9(16) | 2300.6(9) | 31.2(4) |
| C8 | 6593.5(7) | 7652.8(14) | 3948.0(8) | 21.2(3) |
| C9 | 7202.2(7) | 6241.0(14) | 4119.1(8) | 20.2(3) |
| C10 | 7498.8(7) | 7085.8(14) | 3846.4(8) | 20.9(3) |
| C11 | 7268.6(8) | 9122.2(14) | 3493.2(9) | 25.2(4) |
| C12 | 7782.0(10) | 9576.3(17) | 3968.5(10) | 38.7(5) |
| C13 | 7454.6(8) | 8986.1(15) | 2798.5(9) | 27.4(4) |
| C14 | 6726.0(10) | 9881.0(17) | 3473.0(13) | 46.6(6) |
| C15 | 6365.6(7) | 5144.0(13) | 5106.8(8) | 17.4(3) |
| C16 | 6690.1(7) | 4163.1(14) | 5004.4(8) | 18.8(3) |
| C17 | 6940.9(7) | 3496.2(14) | 5524.4(8) | 20.5(3) |
| C18 | 6872.8(7) | 3771.2(14) | 6181.2(8) | 22.0(3) |
| C19 | 6526.9(7) | 4708.2(14) | 6293.7(8) | 22.0(4) |
| C20 | 6279.7(7) | 5376.5(14) | 5771.1(8) | 19.5(3) |
| C21 | 7178.8(9) | 3090.6(16) | 6745.0(9) | 31.7(4) |
| C22 | 5607.0(7) | 6835.3(13) | 4663.5(7) | 17.5(3) |
| C23 | 5732.4(8) | 7674.1(15) | 5147.4(9) | 25.0(4) |
| C24 | 5300.4(8) | 8379.0(15) | 5354.2(9) | 29.2(4) |
| C25 | 4713.2(8) | 8291.6(15) | 5077.8(9) | 26.0(4) |
| C26 | 4584.6(7) | 7500.2(14) | 4582.2(8) | 21.8(3) |
| C27 | 5019.0(7) | 6787.5(14) | 4383.9(7) | 18.3(3) |
| C28 | 4229.6(9) | 8988.9(18) | 5337.1(11) | 39.2(5) |
| B1 | 6105.4(8) | 5938.5(15) | 4487.2(8) | 16.8(4) |
| Cl51 | 3910.37(19) | 2415.6(4) | 2831.5(2) | 33.33(13) |

9.1.8 Anisotropic Displacement Parameters

Table S8. Anisotropic Displacement Parameters (×$10^4$) for **1c**. The anisotropic displacement factor exponent takes the form: $-2\pi^2[h^2a^{*2} \times U_{11}+ ... +2hka^* \times b^* \times U_{12}]$

| Atom | $U_{11}$ | $U_{22}$ | $U_{33}$ | $U_{23}$ | $U_{13}$ | $U_{12}$ |
|---|---|---|---|---|---|---|
| N1 | 13.4(6) | 22.0(7) | 15.6(6) | -0.6(5) | 1.0(5) | 0.5(5) |
| N2 | 17.5(7) | 25.5(7) | 16.6(6) | -3.7(6) | 2.7(5) | -1.9(5) |
| N3 | 11.9(6) | 19.7(7) | 18.9(6) | 1.0(5) | 1.3(5) | 0.6(5) |
| N4 | 15.4(7) | 20.0(7) | 25.5(7) | 0.8(5) | 5.4(5) | 2.6(5) |
| C1 | 13.4(7) | 24.2(8) | 18.4(8) | -1.9(6) | 2.8(6) | -0.7(6) |
| C2 | 19.4(8) | 25.4(8) | 18.3(8) | -5.0(7) | 3.8(6) | 2.0(6) |
| C3 | 19.7(8) | 25.6(8) | 20.1(8) | -6.1(7) | 3.3(6) | 0.2(6) |
| C4 | 25.7(9) | 29.8(9) | 15.7(8) | -5.5(7) | 3.4(7) | -4.6(7) |
| C5 | 35.3(10) | 40.5(11) | 23.1(9) | -10.0(9) | 12.6(8) | -8.7(8) |
| C6 | 32.4(10) | 43.9(11) | 21.8(9) | 2.6(9) | -1.1(8) | -0.8(8) |
| C7 | 39.2(11) | 29.9(10) | 24.9(9) | -4.2(8) | 4.9(8) | -6.5(7) |
| C8 | 15.5(8) | 22.9(8) | 25.9(8) | 2.0(7) | 5.8(7) | 2.2(7) |
| C9 | 14.1(8) | 22.9(8) | 23.7(8) | 3.3(6) | 2.0(6) | 3.6(6) |
| C10 | 12.2(7) | 24.8(8) | 26.1(8) | 3.1(6) | 3.9(6) | 3.2(7) |
| C11 | 22.3(9) | 19.5(8) | 35.3(10) | -0.6(7) | 10.5(7) | 4.6(7) |
| C12 | 51.3(13) | 34.3(11) | 31.7(10) | -18.3(10) | 9.7(9) | -0.8(8) |
| C13 | 25.7(9) | 27.7(9) | 29.1(9) | 1.3(7) | 3.8(7) | 7.6(7) |
| C14 | 38.1(12) | 24.9(10) | 82.6(17) | 10.1(9) | 34.2(12) | 14.1(10) |
| C15 | 10.1(7) | 22.8(8) | 19.2(7) | -4.7(6) | -0.0(6) | -0.4(6) |
| C16 | 15.3(7) | 23.2(8) | 18.2(7) | -2.9(6) | 2.6(6) | -1.1(6) |
| C17 | 15.8(8) | 20.7(8) | 25.0(8) | -2.2(6) | 1.9(6) | 0.7(6) |
| C18 | 18.5(8) | 24.3(8) | 22.0(8) | -7.3(7) | -3.5(6) | 3.3(7) |
| C19 | 19.8(8) | 29.1(9) | 16.8(8) | -7.8(7) | -0.1(6) | -1.2(6) |
| C20 | 13.2(7) | 25.4(8) | 19.8(8) | -3.7(6) | 0.7(6) | -3.1(6) |
| C21 | 36.6(11) | 31.6(10) | 25.3(9) | -1.8(8) | -5.6(8) | 5.8(7) |
| C22 | 15.0(8) | 21.5(8) | 16.4(7) | -0.3(6) | 3.5(6) | 3.5(6) |
| C23 | 18.8(8) | 26.6(9) | 28.8(9) | 0.9(7) | -1.2(7) | -2.8(7) |
| C24 | 31.6(10) | 25.7(9) | 30.5(9) | 1.8(8) | 4.0(8) | -6.4(7) |
| C25 | 25.2(9) | 25.2(9) | 29.1(9) | 5.8(7) | 10.6(7) | 4.8(7) |
| C26 | 13.3(8) | 28.6(9) | 23.8(8) | 2.5(7) | 3.6(6) | 8.6(7) |
| C27 | 16.2(8) | 23.7(8) | 15.3(7) | -0.0(6) | 2.7(6) | 4.6(6) |
| C28 | 34.0(11) | 37.2(11) | 48.8(12) | 10.5(9) | 16.0(9) | -2.5(9) |
| B1 | 12.4(8) | 21.2(9) | 16.7(8) | -2.1(7) | 0.9(7) | -0.9(7) |
| Cl51 | 25.1(2) | 33.9(2) | 40.1(3) | -6.08(18) | -0.85(18) | -5.09(19) |

### 9.1.9 Bond Lengths

Table S9. Bond Lengths in Å for **1c**.

| Atom | Atom | Length/Å |
|---|---|---|
| N1 | C1 | 1.333(2) |
| N1 | C2 | 1.385(2) |
| N1 | B1 | 1.584(2) |
| N2 | C1 | 1.340(2) |
| N2 | C3 | 1.384(2) |
| N2 | C4 | 1.4991(19) |
| N3 | C8 | 1.333(2) |
| N3 | C9 | 1.3831(19) |
| N3 | B1 | 1.598(2) |
| N4 | C8 | 1.338(2) |
| N4 | C10 | 1.378(2) |
| N4 | C11 | 1.500(2) |
| C2 | C3 | 1.343(2) |
| C4 | C5 | 1.520(2) |
| C4 | C6 | 1.525(2) |
| C4 | C7 | 1.528(2) |
| C9 | C10 | 1.353(2) |
| C11 | C12 | 1.531(3) |
| C11 | C13 | 1.517(2) |
| C11 | C14 | 1.520(3) |
| C15 | C16 | 1.400(2) |
| C15 | C20 | 1.405(2) |
| C15 | B1 | 1.630(2) |
| C16 | C17 | 1.390(2) |
| C17 | C18 | 1.392(2) |
| C18 | C19 | 1.390(2) |
| C18 | C21 | 1.508(2) |
| C19 | C20 | 1.392(2) |
| C22 | C23 | 1.403(2) |
| C22 | C27 | 1.397(2) |
| C22 | B1 | 1.615(2) |
| C23 | C24 | 1.383(2) |
| C24 | C25 | 1.396(3) |
| C25 | C26 | 1.382(2) |
| C25 | C28 | 1.508(2) |
| C26 | C27 | 1.386(2) |

9.1.10 Bond angles

Table S10. Bond Angles in ° for **1c**.

| Atom | Atom | Atom | Angle/° |
|---|---|---|---|
| C2 | N1 | C1 | 106.72(13) |
| B1 | N1 | C1 | 130.81(13) |
| B1 | N1 | C2 | 122.42(12) |
| C3 | N2 | C1 | 108.33(13) |
| C4 | N2 | C1 | 127.71(13) |
| C4 | N2 | C3 | 123.91(13) |
| C9 | N3 | C8 | 106.17(13) |
| B1 | N3 | C8 | 124.93(13) |
| B1 | N3 | C9 | 128.89(13) |
| C10 | N4 | C8 | 107.41(13) |
| C11 | N4 | C8 | 127.69(14) |
| C11 | N4 | C10 | 124.84(13) |
| N2 | C1 | N1 | 109.57(13) |
| C3 | C2 | N1 | 109.11(14) |
| C2 | C3 | N2 | 106.26(14) |
| C5 | C4 | N2 | 109.10(13) |
| C6 | C4 | N2 | 107.85(14) |
| C6 | C4 | C5 | 110.55(15) |
| C7 | C4 | N2 | 107.18(13) |
| C7 | C4 | C5 | 110.77(15) |
| C7 | C4 | C6 | 111.28(15) |
| N4 | C8 | N3 | 110.75(14) |
| C10 | C9 | N3 | 108.74(14) |
| C9 | C10 | N4 | 106.91(14) |
| C12 | C11 | N4 | 106.94(14) |
| C13 | C11 | N4 | 108.72(14) |
| C13 | C11 | C12 | 110.81(14) |
| C14 | C11 | N4 | 108.70(13) |
| C14 | C11 | C12 | 111.87(17) |
| C14 | C11 | C13 | 109.69(16) |
| C20 | C15 | C16 | 115.40(14) |
| B1 | C15 | C16 | 121.27(13) |
| B1 | C15 | C20 | 123.33(14) |
| C17 | C16 | C15 | 122.62(15) |
| C18 | C17 | C16 | 120.96(15) |
| C19 | C18 | C17 | 117.45(15) |
| C21 | C18 | C17 | 120.77(16) |
| C21 | C18 | C19 | 121.76(15) |
| C20 | C19 | C18 | 121.27(15) |
| C19 | C20 | C15 | 122.15(15) |
| C27 | C22 | C23 | 115.48(14) |
| B1 | C22 | C23 | 121.39(14) |
| B1 | C22 | C27 | 122.99(14) |
| C24 | C23 | C22 | 122.50(16) |
| C25 | C24 | C23 | 120.69(17) |
| C26 | C25 | C24 | 117.70(16) |
| C28 | C25 | C24 | 121.20(17) |
| C28 | C25 | C26 | 121.01(17) |
| C27 | C26 | C25 | 121.18(15) |
| C26 | C27 | C22 | 122.37(15) |
| N3 | B1 | N1 | 106.27(12) |
| C15 | B1 | N1 | 106.63(13) |
| C15 | B1 | N3 | 110.58(12) |
| C22 | B1 | N1 | 109.28(12) |
| C22 | B1 | N3 | 109.14(13) |
| C22 | B1 | C15 | 114.58(13) |

9.1.11 Torsion angles

Table S11. Torsion Angles in ° for **1c**.

| Atom | Atom | Atom | Atom | Angle/° |
|---|---|---|---|---|
| N1 | C1 | N2 | C3 | 1.40(15) |
| N1 | C1 | N2 | C4 | -175.79(12) |
| N1 | C2 | C3 | N2 | 0.89(15) |
| N1 | B1 | N3 | C8 | 101.03(14) |
| N1 | B1 | N3 | C9 | -80.11(15) |
| N1 | B1 | C15 | C16 | 43.80(14) |
| N1 | B1 | C15 | C20 | -135.58(12) |
| N1 | B1 | C22 | C23 | -179.65(12) |
| N1 | B1 | C22 | C27 | 4.79(16) |
| N3 | C8 | N4 | C10 | 1.37(16) |
| N3 | C8 | N4 | C11 | -175.78(12) |
| N3 | C9 | C10 | N4 | 0.66(14) |
| N3 | B1 | C15 | C16 | -71.32(14) |
| N3 | B1 | C15 | C20 | 109.31(13) |
| N3 | B1 | C22 | C23 | -63.82(15) |
| N3 | B1 | C22 | C27 | 120.62(12) |
| C15 | C16 | C17 | C18 | 0.85(18) |
| C15 | C20 | C19 | C18 | -0.05(18) |
| C15 | B1 | C22 | C23 | 60.78(16) |
| C15 | B1 | C22 | C27 | -114.78(14) |
| C16 | C17 | C18 | C19 | 2.44(18) |
| C16 | C17 | C18 | C21 | -175.93(15) |
| C17 | C18 | C19 | C20 | -2.82(18) |
| C22 | C23 | C24 | C25 | -0.9(2) |
| C22 | C27 | C26 | C25 | -0.82(18) |
| C23 | C24 | C25 | C26 | -1.6(2) |
| C23 | C24 | C25 | C28 | 174.98(17) |
| C24 | C25 | C26 | C27 | 2.42(19) |

9.1.12 Hydrogen Fractional Atomic Coordinates and Equivalent Isotropic Displacement Parameters

Table S12. Hydrogen Fractional Atomic Coordinates (×10[4]) and Equivalent Isotropic Displacement Parameters (Å$^2$×10$^3$) for **1c**. $U_{eq}$ is defined as 1/3 of the trace of the orthogonalised $U_{ij}$.

| Atom | x | y | z | $U_{eq}$ |
|---|---|---|---|---|
| H1 | 6157.3(7) | 5542.9(14) | 3056.8(8) | 22.3(4) |
| H2 | 5280.7(7) | 4125.3(14) | 4455.4(8) | 25.1(4) |
| H3 | 4967.4(7) | 3155.8(14) | 3400.8(8) | 26.1(4) |
| H5a | 6025(3) | 5164.3(18) | 1956(6) | 48.4(7) |
| H5b | 6428.4(9) | 4113(10) | 2215(4) | 48.4(7) |
| H5c | 6050(4) | 4056(9) | 1505(2) | 48.4(7) |
| H6a | 4621.6(9) | 3929(9) | 2146(4) | 49.4(6) |
| H6b | 4934(3) | 5070.2(18) | 1937(6) | 49.4(6) |
| H6c | 4920(3) | 3986(10) | 1463(2) | 49.4(6) |
| H7a | 5912(4) | 2316.0(16) | 2607(5) | 46.9(6) |
| H7b | 5207(3) | 2243.5(16) | 2451(6) | 46.9(6) |
| H7c | 5620(6) | 2265.6(17) | 1854.5(16) | 46.9(6) |
| H8 | 6246.7(7) | 8107.6(14) | 3925.0(8) | 25.4(4) |
| H9 | 7360.4(7) | 5520.0(14) | 4245.1(8) | 24.3(4) |
| H10 | 7898.1(7) | 7065.5(14) | 3742.6(8) | 25.1(4) |
| H12a | 7665(3) | 9588(12) | 4421.6(15) | 58.1(7) |
| H12b | 8129(2) | 9086(7) | 3952(6) | 58.1(7) |
| H12c | 7881(4) | 10345(5) | 3836(5) | 58.1(7) |
| H13a | 7809(3) | 8508(9) | 2814.6(12) | 41.1(6) |
| H13b | 7132(2) | 8632(10) | 2512.4(18) | 41.1(6) |
| H13c | 7543(5) | 9730.5(18) | 2620(3) | 41.1(6) |
| H14a | 6403(2) | 9548(7) | 3178(6) | 69.9(9) |
| H14b | 6602(4) | 9954(11) | 3921.1(18) | 69.9(9) |
| H14c | 6823(2) | 10630(5) | 3307(8) | 69.9(9) |
| H16 | 6741.0(7) | 3944.4(14) | 4562.1(8) | 22.6(4) |
| H17 | 7161.9(7) | 2843.6(14) | 5430.0(8) | 24.6(4) |
| H19 | 6457.6(7) | 4896.6(14) | 6735.5(8) | 26.4(4) |
| H20 | 6045.3(7) | 6010.4(14) | 5866.9(8) | 23.4(4) |
| H21a | 7589(2) | 3349(8) | 6837(4) | 47.6(6) |
| H21b | 6970(4) | 3191(9) | 7142(2) | 47.6(6) |
| H2**1c** | 7175(5) | 2289(2) | 6622(3) | 47.6(6) |
| H23 | 6129.2(8) | 7761.1(15) | 5340.3(9) | 30.0(4) |
| H24 | 5404.3(8) | 8928.5(15) | 5688.0(9) | 35.0(5) |
| H26 | 4191.4(7) | 7443.5(14) | 4373.7(8) | 26.2(4) |
| H27 | 4913.0(7) | 6247.0(14) | 4045.5(7) | 22.0(4) |
| H28a | 3902(3) | 9079(11) | 4987(3) | 58.8(7) |
| H28b | 4086(5) | 8607(6) | 5720(5) | 58.8(7) |
| H28c | 4387(2) | 9735(5) | 5473(7) | 58.8(7) |

## 9.2 Single crystal structure analysis of 2a

CCDC Deposition number 2423150

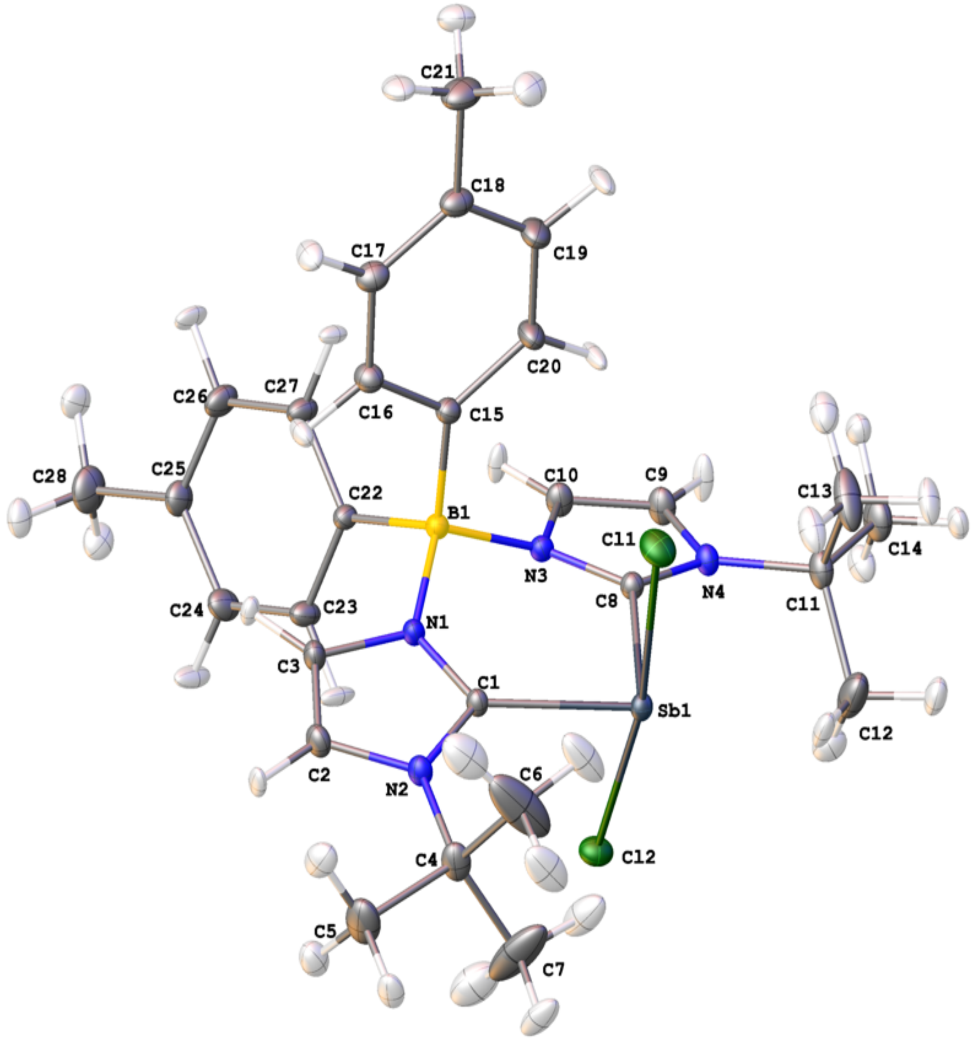


Figure S27. Olex-2 representation of the molecular structure of complex **2a**

### 9.2.1 Experimental.

Single colourless plate-shaped crystals of **2a** were recrystallised by solvent layering of pentane and on a saturated solution of DCM. A suitable crystal 0.08×0.07×0.03 mm$^3$ was selected and mounted on a MITIGEN holder in oil on a Rigaku FRE+ diffractometer with Arc)Sec VHF Varimax confocal mirrors, a UG2 goniometer and HyPix 6000HE detector. The crystal was kept at a steady $T$ = 100(2) K during data collection. The structure was solved with the ShelXT (Sheldrick, 2015) structure solution program using the using dual methods solution method and by using Olex2 1.5-alpha (Dolomanov et al., 2009) as the graphical interface. The model was refined with olex2.refine 1.5-alpha (Bourhis et al., 2015) using full matrix least squares minimisation on $\boldsymbol{F}^2$ minimisation.

### 9.2.2 Crystal Data.

$C_{28}H_{36}BN_4Cl_2Sb$, $M_r$ = 632.114, orthorhombic, *Pbca* (No. 61), a = 14.6318(2) Å, b = 12.2290(2) Å, c = 31.9850(5) Å, $a = b = g = 90^{\circ}$, $V$ = 5723.15(15) Å$^3$, $T$ = 100(2) K, $Z$ = 8, $Z'$ = 1, $m$(Mo $K_a$) = 1.175 mm$^{-1}$, 138287 reflections measured, 15081 unique ($R_{int}$ = 0.0606) which were used in all calculations. The final $wR_2$ was 0.0519 (all data) and $R_1$ was 0.0317 (I≥2 $s$(I)).

| **Compound** | **2a** |
|---|---|
| | |
| Formula | $C_{28}H_{36}BN_4Cl_2Sb$ |
| $D_{calc.}$/ g cm$^{-3}$ | 1.467 |
| $m$/mm$^{-1}$ | 1.175 |
| Formula Weight | 632.114 |
| Colour | colourless |
| Shape | plate-shaped |
| Size/mm$^3$ | 0.08×0.07×0.03 |
| $T$/K | 100(2) |
| Crystal System | orthorhombic |
| Space Group | Pbca |
| $a$/Å | 14.6318(2) |
| $b$/Å | 12.2290(2) |
| $c$/Å | 31.9850(5) |
| $a/^{\circ}$ | 90 |
| $b/^{\circ}$ | 90 |
| $g/^{\circ}$ | 90 |
| V/Å$^3$ | 5723.15(15) |
| $Z$ | 8 |
| $Z'$ | 1 |
| Wavelength/Å | 0.71073 |
| Radiation type | Mo $K_a$ |
| $Q_{min}/^{\circ}$ | 1.89 |
| $Q_{max}/^{\circ}$ | 38.15 |
| Measured Refl's. | 138287 |
| Indep't Refl's | 15081 |
| Refl's I≥2 $s$(I) | 11709 |
| $R_{int}$ | 0.0606 |
| Parameters | 649 |
| Restraints | 642 |
| Largest Peak | 2.3730 |
| Deepest Hole | -1.0683 |
| GooF | 1.0313 |
| $wR_2$ (all data) | 0.0519 |
| $wR_2$ | 0.0480 |
| $R_1$ (all data) | 0.0524 |
| $R_1$ | 0.0317 |

### 9.2.3 Structure Quality Indicators

| | | | | |
|---|---|---|---|---|
| Reflections: | d min (MoKα) 2Θ=76.3° 0.58 | I/σ(I) 27.4 | Rint m=9.63 6.06% | Full 50.5° 96% to 76.3° 100 |
| Refinement: | Shift 0.001 | Max Peak 2.4 | Min Peak -1.1 | GooF 1.031 |

A colourless plate-shaped crystal with dimensions 0.08×0.07×0.03 mm$^3$ was mounted on a MITIGEN holder in oil. X-ray diffraction data were collected using a Rigaku FRE+ diffractometer with Arc)Sec VHF Varimax confocal mirrors, a UG2 goniometer and HyPix 6000HE detector equipped with an Oxford Cryosystems low-temperature device, operating at $T$ = 100(2) K.

Data were measured using profile data from *w*-scans of 0.5 ° per frame for 7.0 s using Mo $K_a$ radiation (Rotating Anode, 45.0 kV, 55.0 mA). The total number of runs and images was based on the strategy calculation from the program CrysAlisPro system (CCD 43.121a 64-bit (release 24-04-2024)). The maximum resolution achieved was $Q$ = 38.15°.

Cell parameters were retrieved using the CrysAlisPro 1.171.43.124a (Rigaku OD, 2024) software and refined using CrysAlisPro 1.171.43.124a (Rigaku OD, 2024) on 42969 reflections, 31 % of the observed reflections. Data reduction was performed using the CrysAlisPro 1.171.43.124a (Rigaku OD, 2024) software which corrects for Lorentz polarisation. The final completeness is 99.98 % out to 38.15° in $Q$.

A gaussian absorption correction was performed using CrysAlisPro 1.171.43.124a (Rigaku OD, 2024) Numerical absorption correction based on gaussian integration over a multifaceted crystal model Empirical absorption correction using spherical harmonics, implemented in SCALE3 ABSPACK scaling algorithm.. The absorption coefficient $m$ of this material is 1.175 mm$^{-1}$ at this wavelength ($l$ = 0.71073Å) and the minimum and maximum transmissions are 0.802 and 1.000.

The structure was solved in the space group *Pbca* (# 61) by using dual methods using the ShelXT (Sheldrick, 2015) structure solution program and refined by full matrix least squares minimisation on $\boldsymbol{F}^2$ using version of olex2.refine 1.5-alpha (Bourhis et al., 2015). All non-hydrogen atoms were refined anisotropically.

*_refine_special_details*: Thermal restraints (RIGU) applied to all atoms.

*_olex2_refine_details*: Refinement using NoSpherA2, an implementation of NOn-SPHERical Atom-form-factors in Olex2. Please cite: F. Kleemiss et al. Chem. Sci. DOI 10.1039/D0SC05526C – 2021 NoSpherA2 implementation of HAR makes use of tailor-made aspherical atomic form factors calculated on-the-fly from a Hirshfeld-partitioned electron density (ED) - not from spherical-atom form factors. The ED is calculated from a gaussian basis set single determinant SCF wavefunction - either Hartree-Fock or DFT using selected functionals - for a fragment of the crystal. This fragment can be embedded in an electrostatic crystal field by employing cluster charges or modelled using implicit solvation models, depending on the software used. The following options were used: SOFTWARE: ORCA 5.0 ::: PARTITIONING: NoSpherA2 ::: INT ACCURACY: Normal ::: METHOD:

R2SCAN ::: BASIS SET: x2c-TZVP ::: CHARGE: 0 ::: MULTIPLICITY: 1 ::: RELATIVISTIC: DKH2 ::: DATE: 2024-05-29_20-16-28

*_exptl_absorpt_process_details*: CrysAlisPro 1.171.43.124a (Rigaku OD, 2024). Numerical absorption correction based on gaussian integration over a multifaceted crystal model. Empirical absorption correction using spherical harmonics, implemented in SCALE3 ABSPACK scaling algorithm.

There is a single formula unit in the asymmetric unit, which is represented by the reported sum formula. In other words: Z is 8 and Z' is 1. The moiety formula is C28 H36 B Cl2 N4 Sb.

## 9.2.4 Data Plots: Diffraction Data

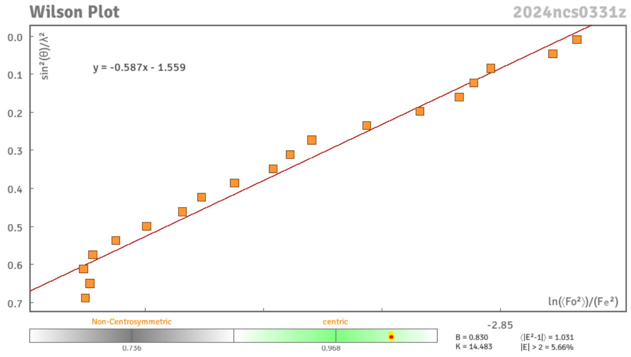


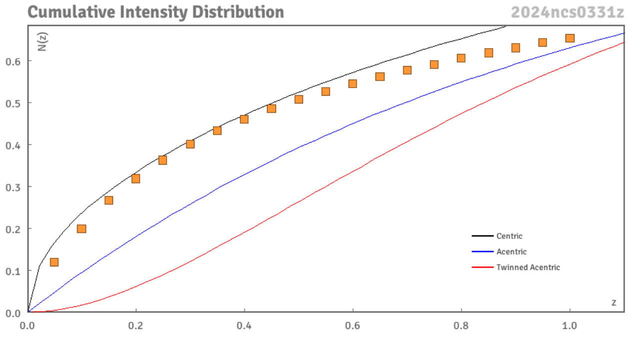


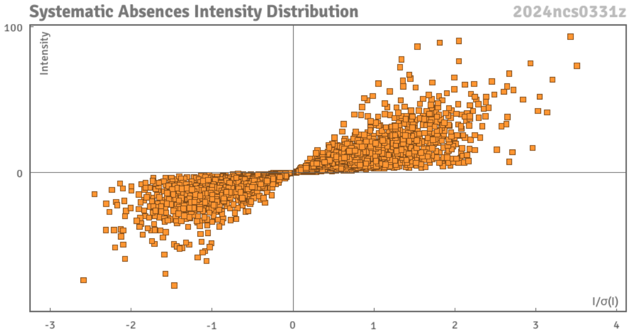


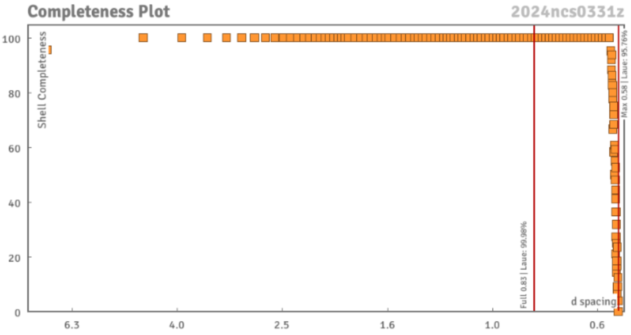


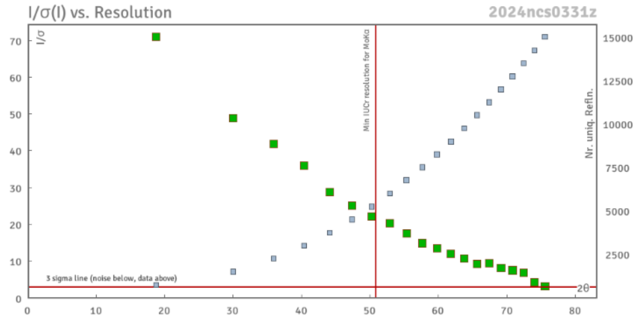


## 9.2.5 Data Plots: Refinement and Data

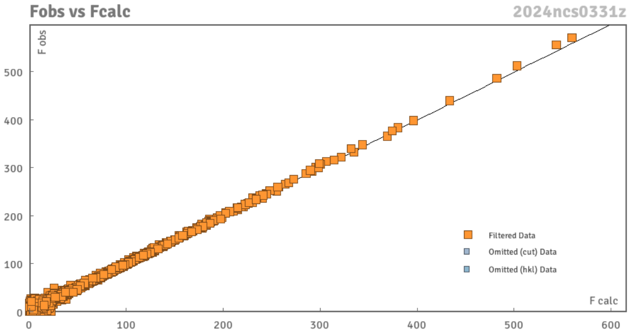


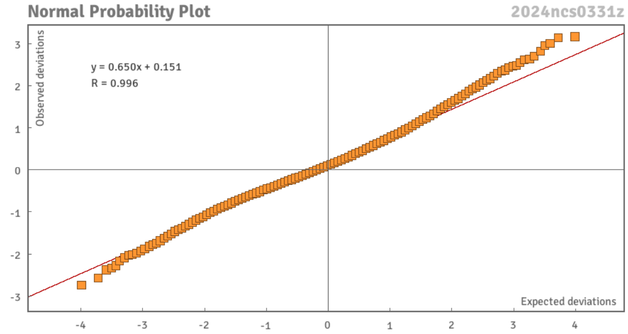

### 9.2.6 Reflection Statistics

| | | | |
|---|---|---|---|
| Total reflections (after filtering) | 145194 | Unique reflections | 15081 |
| Completeness | 0.958 | Mean I/*s* | 21.27 |
| $hkl_{max}$ collected | (25, 21, 53) | $hkl_{min}$ collected | (-24, -21, -52) |
| $hkl_{max}$ used | (25, 21, 53) | $hkl_{min}$ used | (0, 0, 0) |
| Lim $d_{max}$ collected | 100.0 | Lim $d_{min}$ collected | 0.36 |
| $d_{max}$ used | 12.23 | $d_{min}$ used | 0.58 |
| Friedel pairs | 35738 | Friedel pairs merged | 1 |
| Inconsistent equivalents | 0 | $R_{int}$ | 0.0606 |
| $R_{sigma}$ | 0.0365 | Intensity transformed | 0 |
| Omitted reflections | 0 | Omitted by user (OMIT hkl) | 0 |
| Multiplicity | (53377, 26242, 10285, 1453, 389, 105, 13) | Maximum multiplicity | 32 |
| Removed systematic absences | 6907 | Filtered off (Shel/OMIT) | 0 |

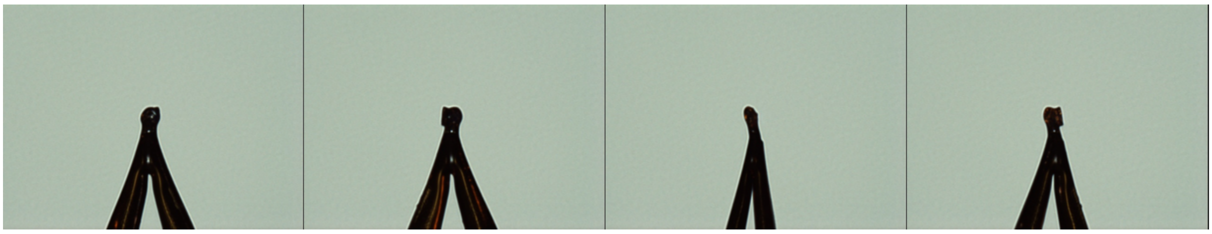

9.2.7 Fractional Atomic Coordinates and Equivalent Isotropic Displacement Parameters

Table S13. Fractional Atomic Coordinates (×10[4]) and Equivalent Isotropic Displacement Parameters (Å[2]×10[3]) for **2a**. $U_{eq}$ is defined as 1/3 of the trace of the orthogonalised $U_{ij}$.

| Atom | x | y | z | $U_{eq}$ |
|---|---|---|---|---|
| Sb1 | 6113.35(4) | 5912.43(5) | 3727.824(19) | 10.431(16) |
| Cl1 | 6245.5(2) | 7172.9(2) | 4346.44(8) | 20.69(5) |
| Cl2 | 5647.70(19) | 4695.1(2) | 3071.58(8) | 16.75(5) |
| N1 | 4035.1(6) | 5723.3(7) | 3927.5(3) | 11.13(15) |
| N2 | 4761.1(6) | 4368.6(7) | 4231.8(3) | 12.62(15) |
| N3 | 4455.9(6) | 7150.2(7) | 3360.5(3) | 10.96(14) |
| N4 | 5702.9(6) | 7741.5(7) | 3051.4(3) | 12.41(15) |
| C1 | 4863.0(7) | 5279.5(8) | 3992.7(3) | 10.63(16) |
| C2 | 3845.1(7) | 4252.8(9) | 4322.1(3) | 15.03(18) |
| C3 | 3396.3(7) | 5088.1(9) | 4132.7(3) | 14.08(18) |
| C4 | 5485.0(8) | 3603.6(10) | 4392.3(4) | 18.5(2) |
| C5 | 5027.1(10) | 2652.6(12) | 4613.7(5) | 28.7(3) |
| C6 | 6101.3(15) | 4213.4(15) | 4689.6(8) | 56.3(6) |
| C7 | 6029.2(14) | 3130.3(18) | 4029.4(6) | 52.4(6) |
| C8 | 5372.8(7) | 7121.0(8) | 3371.2(3) | 10.89(16) |
| C9 | 4966.2(7) | 8157.3(10) | 2833.9(3) | 16.4(2) |
| C10 | 4198.0(7) | 7787.1(9) | 3026.0(3) | 15.07(18) |
| C11 | 6678.3(7) | 8074.9(10) | 2981.2(3) | 16.71(19) |
| C12 | 7266.0(10) | 7080.5(13) | 2875.8(5) | 31.3(3) |
| C13 | 7006.0(10) | 8652.8(15) | 3374.5(4) | 33.3(3) |
| C14 | 6731.6(9) | 8871.0(11) | 2615.7(4) | 23.0(2) |
| C15 | 3760.4(6) | 7805.4(8) | 4069.4(3) | 12.19(17) |
| C16 | 3248.6(7) | 7686.6(9) | 4437.7(3) | 14.93(18) |
| C17 | 3203.2(8) | 8503.2(10) | 4741.2(3) | 16.54(19) |
| C18 | 3671.3(8) | 9489.6(10) | 4689.8(4) | 17.5(2) |
| C19 | 4172.9(8) | 9625.1(10) | 4323.0(4) | 21.2(2) |
| C20 | 4215.7(8) | 8802.6(9) | 4020.3(4) | 18.1(2) |
| C21 | 3631.8(10) | 10368.2(12) | 5018.2(4) | 25.7(3) |
| C22 | 2746.3(7) | 6697.3(8) | 3508.8(3) | 11.74(17) |
| C23 | 2510.7(8) | 5768.0(9) | 3274.8(4) | 16.42(19) |
| C24 | 1637.6(8) | 5632.8(10) | 3108.7(4) | 19.1(2) |
| C25 | 959.7(7) | 6424.2(10) | 3167.8(4) | 17.3(2) |
| C26 | 1196.0(8) | 7372.5(10) | 3384.0(4) | 19.1(2) |
| C27 | 2072.1(7) | 7497.5(9) | 3552.2(4) | 16.1(2) |
| C28 | 8.4(9) | 6255.3(13) | 3001.1(5) | 26.5(3) |
| B1 | 3747.0(7) | 6851.2(9) | 3717.2(4) | 11.28(18) |

9.2.8 Anisotropic Displacement Parameters

Table S14. Anisotropic Displacement Parameters (×$10^4$) for **2a**. The anisotropic displacement factor exponent takes the form: $-2p^2[h^2a^{*2} \times U_{11} + ... + 2hka^* \times b^* \times U_{12}]$

| Atom | $U_{11}$ | $U_{22}$ | $U_{33}$ | $U_{23}$ | $U_{13}$ | $U_{12}$ |
|---|---|---|---|---|---|---|
| Sb1 | 8.39(2) | 11.92(3) | 10.99(3) | -0.21(2) | -0.82(2) | 0.94(2) |
| Cl1 | 26.59(14) | 20.50(12) | 14.99(11) | -4.25(11) | -2.72(9) | -3.78(9) |
| Cl2 | 20.08(11) | 14.75(11) | 15.42(11) | 0.78(9) | -3.15(9) | -3.58(9) |
| N1 | 9.5(3) | 9.8(4) | 14.1(4) | -0.3(3) | 0.4(3) | 1.3(3) |
| N2 | 12.0(4) | 12.2(4) | 13.6(4) | -0.5(3) | -0.4(3) | 3.2(3) |
| N3 | 10.2(3) | 10.4(4) | 12.3(4) | 0.4(3) | -0.3(3) | 0.6(3) |
| N4 | 11.8(4) | 13.3(4) | 12.2(4) | -1.9(3) | 0.1(3) | 2.1(3) |
| C1 | 9.3(4) | 10.7(4) | 11.9(4) | -0.5(3) | -0.1(3) | 2.2(3) |
| C2 | 14.2(4) | 13.1(4) | 17.8(5) | -2.3(4) | 0.6(4) | 4.0(3) |
| H2 | 20(7) | 23(6) | 40(9) | -2(3) | 6(4) | 17(4) |
| C3 | 10.4(4) | 12.7(4) | 19.2(5) | -1.7(3) | 1.4(3) | 2.1(3) |
| H3 | 14(4) | 29(8) | 32(9) | 2(2) | 2(2) | 8(5) |
| C4 | 15.4(5) | 17.9(5) | 22.2(5) | 0.4(4) | -3.2(4) | 9.6(4) |
| C5 | 24.0(6) | 22.3(6) | 39.8(8) | 0.6(5) | 0.2(5) | 17.0(6) |
| H5a | 49(8) | 42(8) | 46(5) | 10(3) | 7(3) | 18(3) |
| H5b | 35(7) | 38(7) | 66(7) | -1(3) | -8(3) | 8(3) |
| H5c | 32(6) | 30(6) | 62(9) | 1(3) | -3(3) | 21(3) |
| C6 | 55.6(11) | 29.4(9) | 84.1(15) | -11.3(8) | -54.9(11) | 18.7(9) |
| H6a | 69(9) | 46(6) | 113(10) | -19(3) | -58(4) | 31(3) |
| H6b | 76(7) | 59(10) | 85(6) | -5(3) | -47(3) | 9(3) |
| H6c | 64(7) | 49(7) | 95(10) | -5(3) | -53(3) | 24(4) |
| C7 | 50.3(11) | 61.3(12) | 45.6(10) | 42.0(9) | 22.2(8) | 30.7(8) |
| H7a | 80(8) | 93(10) | 78(7) | 41(3) | 2(3) | 7(3) |
| H7b | 77(9) | 73(7) | 63(8) | 38(3) | 31(4) | 34(3) |
| H7c | 53(7) | 61(8) | 54(8) | 36(3) | 19(3) | 26(3) |
| C8 | 10.2(4) | 11.1(4) | 11.3(4) | -0.2(3) | -0.2(3) | 1.1(3) |
| C9 | 15.2(4) | 18.0(5) | 16.2(5) | -1.0(4) | -1.6(4) | 7.2(4) |
| H9 | 21(8) | 62(10) | 42(6) | -5(4) | -5(3) | 39(4) |
| C10 | 12.6(4) | 15.5(5) | 17.1(5) | 0.5(4) | -2.4(3) | 4.9(4) |
| H10 | 13(4) | 48(11) | 42(9) | 2(2) | -2(2) | 26(6) |
| C11 | 14.2(4) | 20.2(5) | 15.8(5) | -4.2(4) | 0.6(3) | 4.4(4) |
| C12 | 20.0(6) | 30.6(7) | 43.4(8) | 5.8(5) | 13.1(5) | 13.1(6) |
| H12a | 22(7) | 44(6) | 55(5) | 11(3) | 15(3) | 20(3) |
| H12b | 44(8) | 48(8) | 63(6) | 11(4) | 2(3) | -1(3) |
| H12c | 27(4) | 50(8) | 61(9) | 3(2) | 15(2) | 16(4) |
| C13 | 27.8(7) | 51.2(10) | 20.9(6) | -23.8(7) | -1.2(5) | -0.9(6) |
| H13a | 46(7) | 59(6) | 46(8) | -17(3) | 3(3) | -8(3) |
| H13b | 49(9) | 64(7) | 29(5) | -26(4) | -3(3) | 3(3) |
| H13c | 34(4) | 66(9) | 43(8) | -27(2) | 0(2) | 0(4) |
| C14 | 21.6(6) | 25.0(6) | 22.2(6) | -4.5(5) | 3.8(4) | 8.5(4) |
| H14a | 45(8) | 43(7) | 27(4) | -10(3) | 2(2) | 6(2) |
| H14b | 31(6) | 33(5) | 43(8) | -1(2) | 4(3) | 7(3) |
| H14c | 26(4) | 29(7) | 42(8) | -5(2) | 5(2) | 10(4) |
| C15 | 11.5(4) | 11.0(4) | 14.1(4) | -0.0(3) | -0.4(3) | -1.7(3) |
| C16 | 17.5(5) | 13.7(4) | 13.6(4) | -0.4(4) | 0.6(3) | -1.1(3) |
| H16 | 40(9) | 22(5) | 30(8) | -12(3) | 10(4) | -3(3) |
| C17 | 18.9(5) | 17.4(5) | 13.3(5) | 2.9(4) | -1.1(4) | -2.7(4) |
| H17 | 46(9) | 36(8) | 26(5) | -8(5) | 14(3) | -8(3) |
| C18 | 16.4(4) | 16.9(5) | 19.0(5) | 2.7(4) | -3.3(4) | -6.3(4) |
| C19 | 21.1(5) | 15.3(5) | 27.1(6) | -4.8(4) | 4.2(4) | -8.5(4) |

| | | | | | | |
|---|---|---|---|---|---|---|
| H19 | 64(11) | 27(5) | 59(9) | -24(4) | 30(5) | -20(3) |
| C20 | 18.9(5) | 12.8(4) | 22.5(5) | -4.9(4) | 5.2(4) | -5.5(4) |
| H20 | 58(11) | 22(8) | 41(6) | -20(5) | 28(4) | -14(3) |
| C21 | 25.0(6) | 24.3(6) | 28.0(6) | 3.7(5) | -1.4(5) | -13.7(5) |
| H21a | 54(8) | 31(7) | 42(5) | 1(3) | 9(3) | -18(3) |
| H21b | 40(4) | 50(9) | 55(8) | -2(2) | -9(2) | -18(4) |
| H21c | 54(8) | 35(5) | 57(8) | 12(3) | -7(4) | -11(3) |
| C22 | 9.5(4) | 10.8(4) | 14.9(4) | 0.5(3) | -0.2(3) | -2.1(3) |
| C23 | 13.7(4) | 12.2(5) | 23.4(5) | 1.7(3) | -3.6(4) | -5.2(4) |
| H23 | 27(6) | 30(6) | 63(11) | 15(3) | -19(4) | -26(5) |
| C24 | 14.8(5) | 15.9(5) | 26.5(6) | -1.1(4) | -6.2(4) | -4.8(4) |
| H24 | 27(8) | 35(6) | 87(12) | 9(3) | -24(5) | -36(4) |
| C25 | 11.0(4) | 19.8(5) | 21.0(5) | -0.7(4) | -3.5(3) | -1.0(4) |
| C26 | 12.1(4) | 21.1(5) | 24.1(5) | 5.0(4) | -3.9(4) | -5.1(4) |
| H26 | 21(6) | 35(6) | 64(11) | 15(3) | -12(4) | -18(4) |
| C27 | 12.3(4) | 14.6(5) | 21.5(5) | 3.4(3) | -3.2(4) | -5.4(4) |
| H27 | 20(7) | 22(5) | 48(10) | 7(3) | -12(4) | -18(3) |
| C28 | 14.4(5) | 34.0(7) | 31.2(7) | -1.9(5) | -7.7(5) | -0.5(6) |
| H28a | 25(7) | 51(6) | 46(7) | -8(3) | -7(3) | 5(3) |
| H28b | 25(7) | 46(5) | 61(9) | 5(2) | -11(4) | -3(3) |
| H28c | 34(8) | 52(9) | 39(4) | -2(4) | -10(2) | -5(2) |
| B1 | 10.2(4) | 9.8(4) | 13.8(5) | 0.1(3) | 0.4(3) | -0.2(4) |

9.2.9 Bond lengths

Table S15. Bond Lengths in Å for **2a**.

| Atom | Atom | Length/Å |
|---|---|---|
| Sb1 | Cl1 | 2.5157(3) |
| Sb1 | Cl2 | 2.6620(3) |
| Sb1 | C1 | 2.1596(10) |
| Sb1 | C8 | 2.1586(10) |
| N1 | C1 | 1.3436(12) |
| N1 | C3 | 1.3811(13) |
| N1 | B1 | 1.5914(14) |
| N2 | C1 | 1.3593(13) |
| N2 | C2 | 1.3785(14) |
| N2 | C4 | 1.5036(14) |
| N3 | C8 | 1.3425(13) |
| N3 | C10 | 1.3762(13) |
| N3 | B1 | 1.5847(14) |
| N4 | C8 | 1.3621(13) |
| N4 | C9 | 1.3799(14) |
| N4 | C11 | 1.5011(14) |
| C2 | C3 | 1.3572(15) |
| C4 | C5 | 1.5177(17) |
| C4 | C6 | 1.508(2) |
| C4 | C7 | 1.522(2) |
| C9 | C10 | 1.3586(15) |
| C11 | C12 | 1.5270(18) |
| C11 | C13 | 1.5206(18) |
| C11 | C14 | 1.5234(16) |
| C15 | C16 | 1.4034(15) |
| C15 | C20 | 1.3985(15) |
| C15 | B1 | 1.6221(15) |
| C16 | C17 | 1.3941(15) |
| C17 | C18 | 1.3968(17) |
| C18 | C19 | 1.3938(17) |
| C18 | C21 | 1.5036(17) |
| C19 | C20 | 1.3975(16) |
| C22 | C23 | 1.4038(14) |
| C22 | C27 | 1.3965(14) |
| C22 | B1 | 1.6197(15) |
| C23 | C24 | 1.3934(16) |
| C24 | C25 | 1.3986(17) |
| C25 | C26 | 1.3937(17) |
| C25 | C28 | 1.5048(16) |
| C26 | C27 | 1.3985(15) |

9.2.10 Bond angles

Table S16. Bond Angles in ° for **2a**.

| Atom | Atom | Atom | Angle/° |
|---|---|---|---|
| I2 | Sb1 | Cl1 | 169.274(9) |
| C1 | Sb1 | Cl1 | 88.63(3) |
| C1 | Sb1 | Cl2 | 83.80(3) |
| C8 | Sb1 | Cl1 | 91.99(3) |
| C8 | Sb1 | Cl2 | 80.66(3) |
| C8 | Sb1 | C1 | 91.57(4) |
| C3 | N1 | C1 | 108.01(8) |
| B1 | N1 | C1 | 130.88(8) |
| B1 | N1 | C3 | 120.60(8) |
| C2 | N2 | C1 | 107.98(8) |
| C4 | N2 | C1 | 128.66(9) |
| C4 | N2 | C2 | 123.33(9) |
| C10 | N3 | C8 | 107.99(9) |
| B1 | N3 | C8 | 129.03(8) |
| B1 | N3 | C10 | 120.72(8) |
| C9 | N4 | C8 | 107.87(8) |
| C11 | N4 | C8 | 126.92(8) |
| C11 | N4 | C9 | 124.55(9) |
| N1 | C1 | Sb1 | 123.93(7) |
| N2 | C1 | Sb1 | 127.39(7) |
| N2 | C1 | N1 | 108.63(8) |
| C3 | C2 | N2 | 107.42(9) |
| C2 | C3 | N1 | 107.95(9) |
| C5 | C4 | N2 | 108.98(9) |
| C6 | C4 | N2 | 109.20(11) |
| C6 | C4 | C5 | 110.40(13) |
| C7 | C4 | N2 | 110.17(10) |
| C7 | C4 | C5 | 107.19(13) |
| C7 | C4 | C6 | 110.88(17) |
| N3 | C8 | Sb1 | 122.23(7) |
| N4 | C8 | Sb1 | 126.89(7) |
| N4 | C8 | N3 | 108.69(9) |
| C10 | C9 | N4 | 107.19(9) |
| C9 | C10 | N3 | 108.26(9) |
| C12 | C11 | N4 | 110.61(10) |
| C13 | C11 | N4 | 107.60(9) |
| C13 | C11 | C12 | 112.04(12) |
| C14 | C11 | N4 | 109.69(9) |
| C14 | C11 | C12 | 108.10(10) |
| C14 | C11 | C13 | 108.77(11) |
| C20 | C15 | C16 | 116.02(10) |
| B1 | C15 | C16 | 120.14(9) |
| B1 | C15 | C20 | 123.71(9) |
| C17 | C16 | C15 | 122.39(10) |
| C18 | C17 | C16 | 120.89(10) |
| C19 | C18 | C17 | 117.37(10) |
| C21 | C18 | C17 | 121.07(11) |
| C21 | C18 | C19 | 121.55(11) |
| C20 | C19 | C18 | 121.42(11) |
| C19 | C20 | C15 | 121.90(11) |
| C27 | C22 | C23 | 116.54(9) |
| B1 | C22 | C23 | 122.38(9) |
| B1 | C22 | C27 | 121.09(9) |
| C24 | C23 | C22 | 121.64(10) |
| C25 | C24 | C23 | 121.10(10) |
| C26 | C25 | C24 | 117.83(10) |
| C28 | C25 | C24 | 120.87(11) |
| C28 | C25 | C26 | 121.30(11) |
| C27 | C26 | C25 | 120.61(10) |
| C26 | C27 | C22 | 122.19(10) |
| N3 | B1 | N1 | 109.33(8) |
| C15 | B1 | N1 | 109.06(8) |
| C15 | B1 | N3 | 109.03(8) |
| C22 | B1 | N1 | 108.22(8) |
| C22 | B1 | N3 | 108.80(8) |
| C22 | B1 | C15 | 112.35(8) |

9.2.11 Torsion Angles

Table S17. Torsion Angles in ° for **2a**.

| Atom | Atom | Atom | Atom | Angle/° |
|---|---|---|---|---|
| Sb1 | C1 | N1 | C3 | 178.09(8) |
| Sb1 | C1 | N1 | B1 | -10.28(11) |
| Sb1 | C1 | N2 | C2 | -178.20(9) |
| Sb1 | C1 | N2 | C4 | 3.54(12) |
| Sb1 | C8 | N3 | C10 | -163.47(8) |
| Sb1 | C8 | N3 | B1 | 33.86(10) |
| Sb1 | C8 | N4 | C9 | 162.65(9) |
| Sb1 | C8 | N4 | C11 | -26.38(11) |
| N1 | C1 | N2 | C2 | -0.83(10) |
| N1 | C1 | N2 | C4 | -179.09(8) |
| N1 | C3 | C2 | N2 | -0.36(10) |
| N1 | B1 | N3 | C8 | -46.25(11) |
| N1 | B1 | N3 | C10 | 152.99(8) |
| N1 | B1 | C15 | C16 | -55.14(9) |
| N1 | B1 | C15 | C20 | 129.10(9) |
| N1 | B1 | C22 | C23 | -43.84(10) |
| N1 | B1 | C22 | C27 | 136.60(9) |
| N3 | C8 | N4 | C9 | -0.64(10) |
| N3 | C8 | N4 | C11 | 170.33(8) |
| N3 | C10 | C9 | N4 | 0.20(10) |
| N3 | B1 | C15 | C16 | -174.46(8) |
| N3 | B1 | C15 | C20 | 9.78(11) |
| N3 | B1 | C22 | C23 | 74.86(10) |
| N3 | B1 | C22 | C27 | -104.71(9) |
| C15 | C16 | C17 | C18 | 0.13(13) |
| C15 | C20 | C19 | C18 | -0.05(14) |
| C15 | B1 | C22 | C23 | -164.32(9) |
| C15 | B1 | C22 | C27 | 16.12(11) |
| C16 | C17 | C18 | C19 | 0.70(13) |
| C16 | C17 | C18 | C21 | -179.39(11) |
| C17 | C18 | C19 | C20 | -0.74(13) |
| C22 | C23 | C24 | C25 | 0.25(14) |
| C22 | C27 | C26 | C25 | 0.70(14) |
| C23 | C24 | C25 | C26 | 2.49(14) |
| C23 | C24 | C25 | C28 | -177.77(12) |
| C24 | C25 | C26 | C27 | -2.95(13) |

9.2.12 Hydrogen Fractional Atomic Coordinate and Equivalent Isotropic Displacement Parameters

Table S18. Hydrogen Fractional Atomic Coordinates (×$10^4$) and Equivalent Isotropic Displacement Parameters ($Å^2$×$10^3$) for **2a**. $U_{eq}$ is defined as 1/3 of the trace of the orthogonalised $U_{ij}$.

| Atom | x | y | z | $U_{eq}$ |
|---|---|---|---|---|
| H2 | 3586(10) | 3594(13) | 4509(5) | 28(4) |
| H3 | 2683(9) | 5276(13) | 4125(5) | 25(4) |
| H5a | 4682(12) | 2957(15) | 4889(5) | 45(4) |
| H5b | 4552(12) | 2221(15) | 4411(6) | 46(4) |
| H5c | 5572(12) | 2073(14) | 4723(6) | 41(4) |
| H6a | 6402(15) | 4928(18) | 4553(7) | 76(5) |
| H6b | 5664(17) | 4381(19) | 4939(7) | 73(5) |
| H6c | 6626(14) | 3687(17) | 4811(7) | 69(4) |
| H7a | 5585(18) | 2780(20) | 3823(7) | 84(5) |
| H7b | 6356(15) | 3770(20) | 3854(7) | 71(4) |
| H7c | 6525(13) | 2568(17) | 4163(6) | 56(4) |
| H9 | 5037(10) | 8658(15) | 2575(5) | 42(5) |
| H10 | 3492(10) | 7928(14) | 2967(5) | 34(5) |
| H12a | 7315(11) | 6568(14) | 3130(5) | 40(3) |
| H12b | 6999(13) | 6657(16) | 2603(6) | 52(4) |
| H12c | 7952(12) | 7381(15) | 2821(6) | 46(4) |
| H13a | 6578(14) | 9334(17) | 3441(6) | 50(4) |
| H13b | 6997(13) | 8146(16) | 3646(5) | 47(4) |
| H13c | 7682(12) | 8942(16) | 3323(5) | 48(4) |
| H14a | 6511(12) | 8519(14) | 2336(5) | 38(3) |
| H14b | 6327(11) | 9598(15) | 2670(5) | 35(3) |
| H14c | 7430(11) | 9124(13) | 2590(5) | 33(3) |
| H16 | 2866(11) | 6933(13) | 4482(5) | 31(4) |
| H17 | 2806(12) | 8369(14) | 5019(5) | 36(4) |
| H19 | 4555(13) | 10357(14) | 4275(6) | 50(5) |
| H20 | 4626(13) | 8934(13) | 3748(5) | 40(5) |
| H21a | 3278(12) | 10094(14) | 5293(5) | 42(4) |
| H21b | 4298(13) | 10605(16) | 5123(6) | 48(4) |
| H21c | 3271(13) | 11075(15) | 4915(6) | 49(4) |
| H23 | 3010(11) | 5148(14) | 3223(5) | 40(5) |
| H24 | 1448(11) | 4937(14) | 2934(6) | 50(5) |
| H26 | 701(11) | 8009(14) | 3423(5) | 40(5) |
| H27 | 2234(10) | 8234(13) | 3717(5) | 30(4) |
| H28a | -364(11) | 5687(15) | 3186(5) | 41(4) |
| H28b | -370(11) | 7009(15) | 3013(6) | 44(4) |
| H28c | 8(12) | 5942(15) | 2687(5) | 42(4) |

## 9.3 Single crystal structure analysis of 3a

CCDC Deposition number 2423151

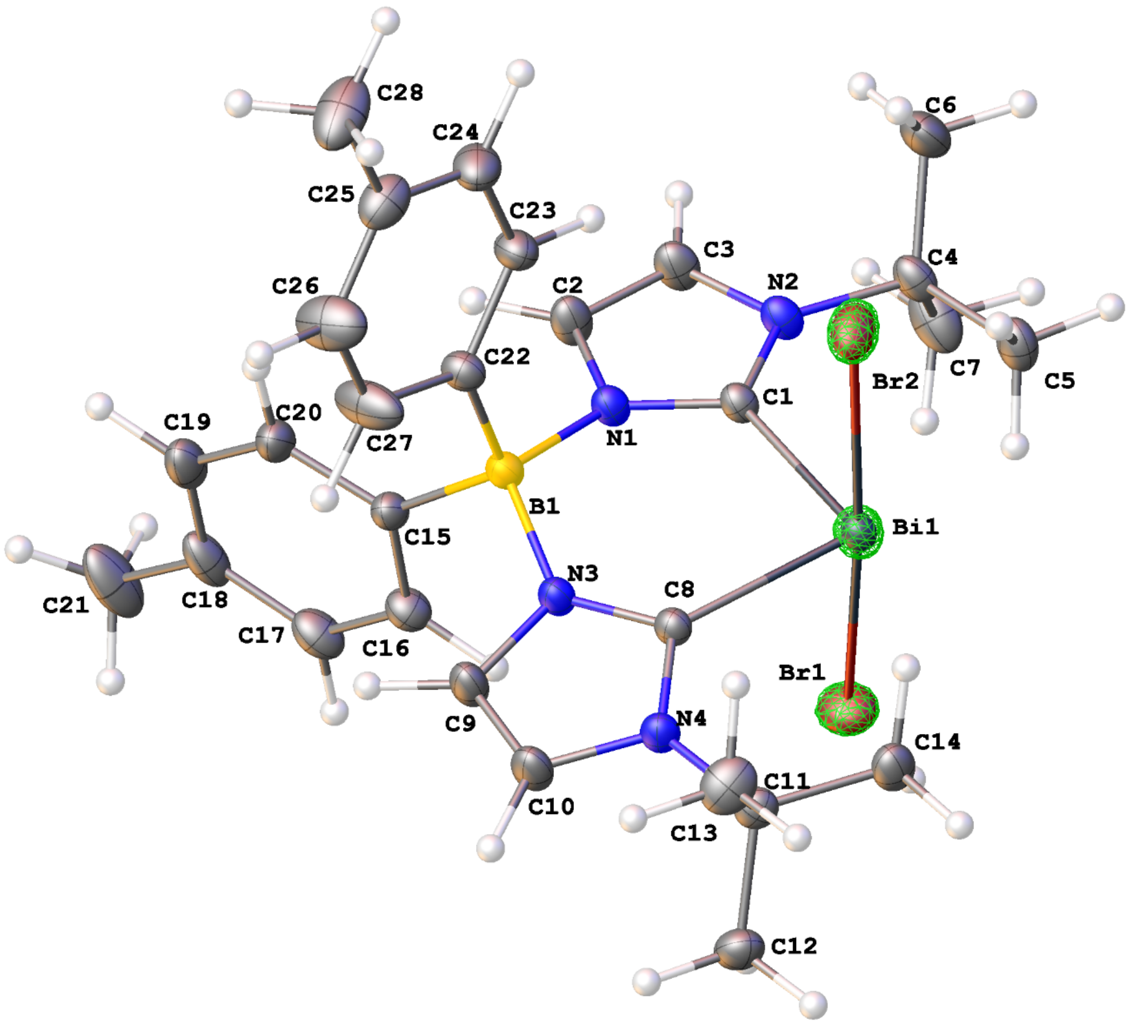


Figure S28. Olex-2 representation of the molecular structure of complex **3a**

### 9.3.1 Experimental.

Single colourless block-shaped crystals of **3a** were used as supplied. A suitable crystal with dimensions 0.24 × 0.15 × 0.11 mm$^3$ was selected and mounted on a MITIGEN holder in oil on a Rigaku FRE+ diffractometer with HF Varimax confocal mirrors, a UG2 goniometer and HyPix 6000HE detector diffractometer. The crystal was kept at a steady $T$ = 100(2) K during data collection. The structure was solved with the ShelXT 2018/2 (Sheldrick, 2015) solution program using dual methods and by using Olex2 1.5-dev (Dolomanov et al., 2009) as the graphical interface. The model was refined with olex2.refine 1.5-dev (Bourhis et al., 2015) using full matrix least squares minimisation on $\boldsymbol{F}^2$.

### 9.3.2 Crystal Data.

$C_{28}H_{36}BN_4Br_2Bi$, $M_r$ = 808.237, monoclinic, $P2_1/c$ (No. 14), a = 16.1734(2) Å, b = 12.5541(1) Å, c = 14.8272(1) Å, $\beta$ = 92.151(1)°, $\alpha = \gamma$ = 90°, $V$ = 3008.43(5) Å$^3$, $T$ = 100(2) K, $Z$ = 4, $Z'$ = 1, $\mu$(Mo $K_\alpha$) = 8.538, 237420 reflections measured, 42500 unique ($R_{int}$ = 0.0696) which were used in all calculations. The final $wR_2$ was 0.0665 (all data) and $R_1$ was 0.0328 (I≥2 $\sigma$(I)).

| Compound | **3a** |
|---|---|
| | |
| Formula | $C_{28}H_{36}BN_4Br_2Bi$ |
| $D_{calc.}$/ g cm$^{-3}$ | 1.784 |
| $\mu$/mm$^{-1}$ | 8.538 |
| Formula Weight | 808.237 |
| Colour | colourless |
| Shape | block-shaped |
| Size/mm$^3$ | 0.24×0.15×0.11 |
| $T$/K | 100(2) |
| Crystal System | monoclinic |
| Space Group | $P2_1/c$ |
| $a$/Å | 16.1734(2) |
| $b$/Å | 12.5541(1) |
| $c$/Å | 14.8272(1) |
| $\alpha$/° | 90 |
| $\beta$/° | 92.151(1) |
| $\gamma$/° | 90 |
| V/Å$^3$ | 3008.43(5) |
| $Z$ | 4 |
| $Z'$ | 1 |
| Wavelength/Å | 0.71073 |
| Radiation type | Mo $K_\alpha$ |
| $\Theta_{min}$/° | 2.05 |
| $\Theta_{max}$/° | 57.79 |
| Measured Refl's. | 237420 |
| Indep't Refl's | 42500 |
| Refl's I≥2 $\sigma$(I) | 28943 |
| $R_{int}$ | 0.0696 |
| Parameters | 408 |
| Restraints | 0 |
| Largest Peak | 3.6270 |
| Deepest Hole | -3.0949 |
| GooF | 0.9808 |
| $wR_2$ (all data) | 0.0665 |
| $wR_2$ | 0.0584 |
| $R_1$ (all data) | 0.0630 |
| $R_1$ | 0.0328 |

### 9.3.3 Structure Quality Indicators

| | | | | |
|---|---|---|---|---|
| Reflections: | d min (MoKα) 2Θ=115.6° 0.42 | I/σ(I) 23.4 | Rint m=5.68 6.96% | Full 50.5° 99.9 |
| Refinement: | Shift 0.001 | Max Peak 3.6 | Min Peak -3.1 | GooF 0.981 |

A colourless block-shaped-shaped crystal with dimensions 0.24 × 0.15 × 0.11 mm$^3$ was mounted on a MITIGEN holder in oil. Data were collected using a Rigaku FRE+ diffractometer with HF Varimax confocal mirrors, a UG2 goniometer and HyPix 6000HE detector diffractometer equipped with an Oxford Cryosystems low-temperature device operating at $T$ = 100(2) K.

Data were measured using profile data from $\omega$-scans with Mo $K_{\alpha}$ radiation. The diffraction pattern was indexed and the total number of runs and images was based on the strategy calculation from the program CrysAlisPro system (CCD 43.143a 64-bit (release 25-10-2024)). The maximum resolution that was achieved was $\Theta$ = 57.79° (0.42 Å).

The unit cell was refined using CrysAlisPro 1.171.43.143a (Rigaku OD, 2024) on 83664 reflections, 35% of the observed reflections.

Data reduction, scaling and absorption corrections were performed using CrysAlisPro 1.171.43.143a (Rigaku OD, 2024). The final completeness is 99.89 % out to 57.79° in $\Theta$. A gaussian absorption correction was performed using CrysAlisPro 1.171.43.143a (Rigaku OD, 2024) Numerical absorption correction was based on gaussian integration over a multifaceted crystal model. Empirical absorption correction using spherical harmonics as implemented in SCALE3 ABSPACK scaling algorithm. The absorption coefficient $\mu$ of this material is 8.538 mm$^{-1}$ at this wavelength ($\lambda$ = 0.71073Å) and the minimum and maximum transmissions are 0.276 and 0.843.

The structure was solved and the space group $P2_1/c$ (# 14) determined by the ShelXT 2018/2 (Sheldrick, 2015) structure solution program using dual methods and refined by full matrix least squares minimisation on $\boldsymbol{F^2}$ using version of olex2.refine 1.5-dev (Bourhis et al., 2015). All non-hydrogen atoms were refined anisotropically. Hydrogen atom positions were calculated geometrically and refined using the riding model.

*_refine_special_details*: Bi and Br atoms refined anharmonically

*_olex2_refine_details*: Refinement using NoSpherA2, an implementation of NOn-SPHERical Atom-form-factors in Olex2.Please cite: F. Kleemiss et al. Chem. Sci. DOI 10.1039/D0SC05526C – 2021 NoSpherA2 implementation of HAR makes use of tailor-made aspherical atomic form factors calculated on-the-fly from a Hirshfeld-partitioned electron density (ED) - not from spherical-atom form factors. The ED is calculated from a gaussian basis set single determinant SCF wavefunction - either Hartree-Fock or DFT using selected functionals- for a fragment of the crystal. This fragment can be embedded in an electrostatic crystal field by employing cluster charges or modelled using implicit solvation models, depending on the software used. The following options were used: SOFTWARE: ORCA 6.0 ::: PARTITIONING: NoSpherA2 ::: INT ACCURACY: Normal ::: METHOD:

r2SCAN ::: BASIS SET: x2c-TZVP ::: CHARGE: 0 ::: MULTIPLICITY: 1 ::: RELATIVISTIC: DKH2 ::: DATE: 2024-11-01_15-10-41

*_exptl_absorpt_process_details*: CrysAlisPro 1.171.43.143a (Rigaku OD, 2024). Numerical absorption correction based on gaussian integration over a multifaceted crystal model. Empirical absorption correction using spherical harmonics as implemented in SCALE3 ABSPACK scaling algorithm.

There is a single formula unit in the asymmetric unit, which is represented by the reported sum formula. In other words: Z is 4 and Z' is 1. The moiety formula is C28 H36 B Bi Br2 N4.

### 9.3.4 Data Plots: Diffraction Data

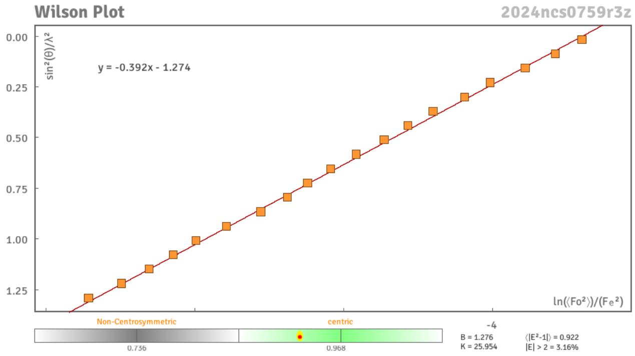


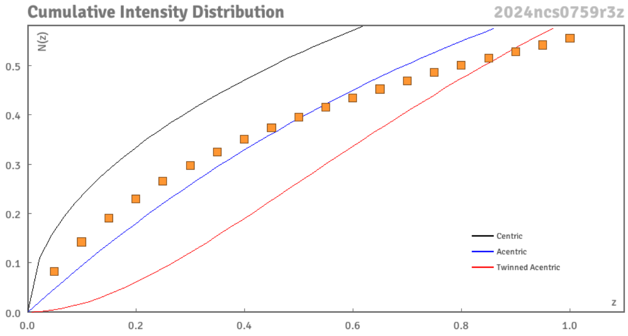


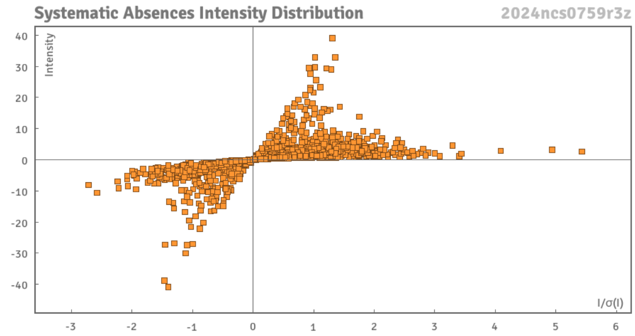


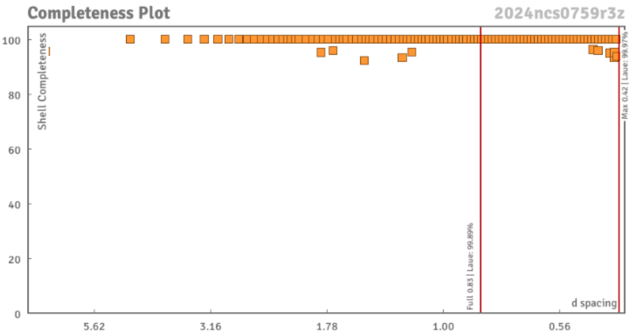


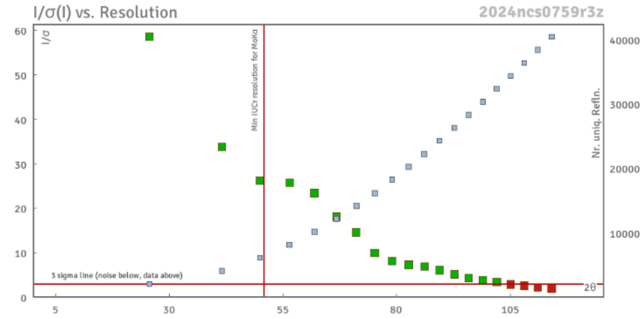


### 9.3.5 Data Plots: Refinement and Data

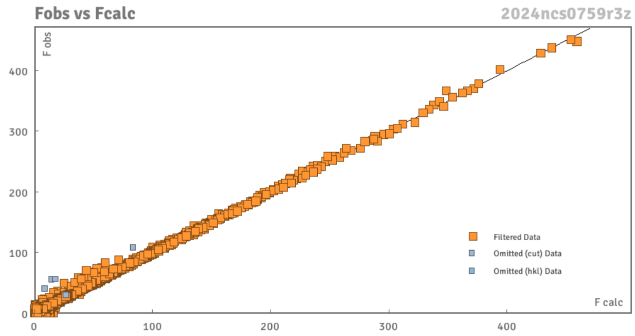


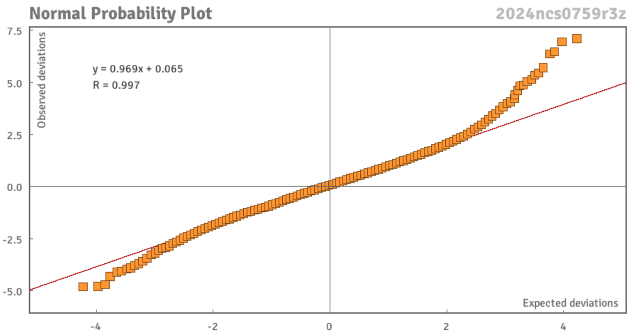

### 9.3.6 Reflection Statistics

| | | | |
|---|---|---|---|
| Total reflections (after filtering) | 241415 | Unique reflections | 42500 |
| Completeness | 1.0 | Mean I/$\sigma$ | 13.2 |
| $hkl_{max}$ collected | (38, 29, 34) | $hkl_{min}$ collected | (-34, -29, -35) |
| $hkl_{max}$ used | (38, 29, 35) | $hkl_{min}$ used | (-38, 0, 0) |
| Lim $d_{max}$ collected | 100.0 | Lim $d_{min}$ collected | 0.36 |
| $d_{max}$ used | 11.13 | $d_{min}$ used | 0.42 |
| Friedel pairs | 36042 | Friedel pairs merged | 1 |
| Inconsistent equivalents | 199 | $R_{int}$ | 0.0696 |
| $R_{sigma}$ | 0.0427 | Intensity transformed | 0 |
| Omitted reflections | 0 | Omitted by user (OMIT hkl) | 20 |
| Multiplicity | (47690, 31773, 18860, 10010, 4005, 1532, 515, 80, 13) | Maximum multiplicity | 19 |
| Removed systematic absences | 4015 | Filtered off (Shel/OMIT) | 0 |

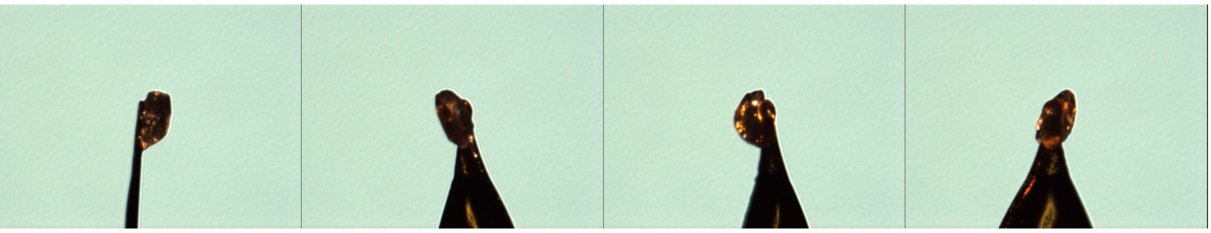

### 9.3.7 Fractional Atomic Coordinates and Equivalent Isotropic Displacement Parameters

Table S19. Fractional Atomic Coordinates (×$10^4$) and Equivalent Isotropic Displacement Parameters (Å$^2$×$10^3$) for **3a**. $U_{eq}$ is defined as 1/3 of the trace of the orthogonalised $U_{ij}$.

| Atom | x | y | z | $U_{eq}$ |
|---|---|---|---|---|
| Bi1 | 8622.12(5) | 5255.01(6) | 5705.69(6) | 16.74(2) |
| Br1 | 7826.04(17) | 6907.6(2) | 6619.52(19) | 31.34(5) |
| Br2 | 9402.09(13) | 3436.06(17) | 4987.30(15) | 27.81(4) |
| N1 | 7686.4(6) | 3710.0(8) | 7066.2(6) | 18.85(13) |
| N2 | 8875.1(6) | 4161.1(9) | 7709.8(6) | 20.80(14) |
| N3 | 6791.6(5) | 4238.7(8) | 5616.2(6) | 19.16(13) |
| N4 | 6911.9(6) | 5232.0(8) | 4414.1(7) | 19.74(14) |
| C1 | 8385.7(6) | 4259.3(9) | 6950.2(7) | 17.75(14) |
| C2 | 7737.1(8) | 3252.2(11) | 7913.0(8) | 25.2(2) |
| C3 | 8471.5(8) | 3529.0(12) | 8312.7(8) | 26.0(2) |
| C4 | 9707.0(7) | 4621.4(11) | 7958.4(8) | 23.43(19) |
| C5 | 10055.4(9) | 5273.9(12) | 7196.4(10) | 28.3(2) |
| C6 | 10288.7(9) | 3694.5(14) | 8174.2(13) | 36.8(3) |
| C7 | 9602.9(11) | 5364.2(15) | 8766.5(11) | 37.3(3) |
| C8 | 7325.3(6) | 4824.4(8) | 5149.6(7) | 17.29(14) |
| C9 | 6030.1(7) | 4272.6(11) | 5164.6(9) | 25.4(2) |
| C10 | 6102.0(8) | 4878.7(11) | 4415.7(10) | 25.8(2) |
| C11 | 7190.6(7) | 5929.6(9) | 3664.6(7) | 21.19(16) |
| C12 | 6575.7(9) | 6854.4(11) | 3566.8(10) | 27.8(2) |
| C13 | 7193.2(12) | 5261.5(12) | 2804.0(10) | 33.0(3) |
| C14 | 8054.1(8) | 6375.7(11) | 3865.7(9) | 27.0(2) |
| C15 | 6126.1(7) | 3404.2(9) | 7020.2(8) | 19.79(16) |
| C16 | 5827.1(8) | 4368.1(11) | 7361.5(9) | 23.77(19) |
| C17 | 5211.5(8) | 4386.3(13) | 7996.0(10) | 29.0(2) |
| C18 | 4880.5(8) | 3434.3(14) | 8327.3(9) | 31.2(3) |
| C19 | 5157.8(8) | 2476.7(13) | 7971.4(11) | 31.4(3) |
| C20 | 5764.4(8) | 2464.1(11) | 7326.7(10) | 26.2(2) |
| C21 | 4251.4(10) | 3456(2) | 9050.9(12) | 48.0(5) |
| C22 | 7124.1(7) | 2256.7(9) | 5938.6(8) | 21.00(16) |
| C23 | 7848.9(8) | 1682.9(10) | 6095.2(10) | 24.55(19) |
| C24 | 7984.0(9) | 701.9(11) | 5680.9(12) | 30.9(3) |
| C25 | 7408.9(11) | 255.2(11) | 5082.8(11) | 30.2(3) |
| C26 | 6684.9(13) | 823.7(15) | 4908.0(14) | 43.9(4) |
| C27 | 6544.9(11) | 1791.5(14) | 5328.4(14) | 41.9(4) |
| C28 | 7549.6(14) | -798.0(14) | 4638.3(15) | 45.7(5) |
| B1 | 6933.6(7) | 3391.1(10) | 6403.4(8) | 18.31(17) |

### 9.3.8 Anisotropic displacement parameters

Table S20. Anisotropic Displacement Parameters (×10$^4$) for **3a**. The anisotropic displacement factor exponent takes the form: $-2\pi^2[h^2a^{*2} \times U_{11} + ... + 2hka^* \times b^* \times U_{12}]$

| Atom | $U_{11}$ | $U_{22}$ | $U_{33}$ | $U_{23}$ | $U_{13}$ | $U_{12}$ |
|---|---|---|---|---|---|---|
| Bi1 | 14.84(3) | 19.82(4) | 15.55(3) | -1.26(3) | 0.43(2) | -0.01(3) |
| Br1 | 36.72(11) | 25.54(11) | 31.71(11) | 5.48(9) | 0.70(9) | -9.82(10) |
| Br2 | 28.39(8) | 25.63(8) | 30.26(8) | -3.92(7) | 12.27(6) | -8.19(7) |
| N1 | 16.4(3) | 24.1(4) | 16.0(3) | -0.4(3) | 0.4(2) | 2.1(3) |
| N2 | 18.0(3) | 29.2(4) | 15.1(3) | 0.4(3) | -0.9(2) | 1.0(3) |
| N3 | 15.5(3) | 22.6(4) | 19.3(3) | -0.9(3) | -1.0(2) | 4.3(3) |
| N4 | 18.7(3) | 22.7(4) | 17.6(3) | -1.2(3) | -2.0(2) | 3.6(3) |
| C1 | 15.4(3) | 22.9(4) | 14.9(3) | 0.0(3) | 0.4(2) | 0.4(3) |
| C2 | 21.6(4) | 36.2(6) | 17.8(4) | -4.3(4) | -0.1(3) | 7.0(4) |
| C3 | 22.4(5) | 38.4(6) | 17.0(4) | -1.5(4) | -1.6(3) | 5.6(4) |
| C4 | 17.3(4) | 34.5(6) | 18.2(4) | 0.4(3) | -2.1(3) | 0.0(4) |
| C5 | 21.3(5) | 38.4(7) | 24.8(5) | -6.9(4) | -2.8(4) | 2.3(5) |
| C6 | 21.8(5) | 42.0(8) | 46.4(9) | 3.5(5) | -3.5(5) | 11.7(7) |
| C7 | 27.9(6) | 57.1(10) | 26.7(6) | -5.1(6) | -0.5(5) | -14.5(6) |
| C8 | 16.4(3) | 19.0(4) | 16.4(3) | -0.9(3) | -1.4(3) | 1.0(3) |
| C9 | 16.8(4) | 32.7(5) | 26.5(5) | -3.3(4) | -3.3(3) | 8.8(4) |
| C10 | 19.1(4) | 33.1(6) | 24.8(5) | -2.6(4) | -5.8(4) | 8.0(4) |
| C11 | 24.1(4) | 22.8(4) | 16.5(3) | -0.2(3) | -1.5(3) | 2.0(3) |
| C12 | 29.7(6) | 24.8(5) | 28.6(5) | 1.9(4) | -3.6(4) | 5.6(4) |
| C13 | 45.5(9) | 33.8(7) | 19.9(4) | -3.1(5) | 2.7(5) | -3.8(4) |
| C14 | 24.7(5) | 32.0(6) | 24.2(5) | -3.8(4) | -0.2(4) | 8.8(4) |
| C15 | 16.7(3) | 20.7(4) | 22.1(4) | 0.4(3) | 2.2(3) | 4.1(3) |
| C16 | 21.2(4) | 26.7(5) | 23.5(4) | 2.9(4) | 2.6(3) | -1.0(4) |
| C17 | 22.0(5) | 40.1(7) | 25.0(5) | 5.6(4) | 2.8(4) | -3.1(5) |
| C18 | 17.2(4) | 54.4(9) | 22.0(5) | 0.6(5) | 2.4(3) | 6.2(5) |
| C19 | 20.6(5) | 41.2(7) | 32.5(6) | -3.1(4) | 2.7(4) | 14.2(6) |
| C20 | 19.7(4) | 25.8(5) | 33.1(6) | -1.0(3) | 3.2(4) | 8.9(4) |
| C21 | 24.7(6) | 92.6(16) | 27.1(6) | 1.0(8) | 8.3(5) | 5.8(8) |
| C22 | 20.0(4) | 23.0(4) | 19.8(4) | 1.9(3) | -1.9(3) | -0.7(3) |
| C23 | 20.4(4) | 21.8(4) | 31.2(5) | 2.4(3) | -1.0(4) | -0.7(4) |
| C24 | 26.5(5) | 23.0(5) | 43.6(8) | 2.7(4) | 6.1(5) | -3.7(5) |
| C25 | 40.5(8) | 25.3(5) | 25.6(5) | -2.7(5) | 9.9(5) | -3.5(4) |
| C26 | 52.5(10) | 36.8(8) | 41.0(9) | 5.9(7) | -19.2(8) | -14.7(7) |
| C27 | 38.8(8) | 37.3(7) | 47.8(9) | 10.7(6) | -22.3(7) | -17.7(7) |
| C28 | 57.6(11) | 31.2(7) | 50.1(10) | -9.1(7) | 26.6(9) | -14.7(7) |
| B1 | 15.8(4) | 20.3(4) | 18.8(4) | 0.4(3) | -0.1(3) | 2.2(3) |

9.3.9 Bond Lengths

Table S21. Bond Lengths in Å for **3a**.

| Atom | Atom | Length/Å |
|---|---|---|
| Bi1 | Br1 | 2.8158(3) |
| Bi1 | Br2 | 2.8357(2) |
| Bi1 | C1 | 2.2730(10) |
| Bi1 | C8 | 2.2894(10) |
| N1 | C1 | 1.3413(14) |
| N1 | C2 | 1.3804(14) |
| N1 | B1 | 1.5871(15) |
| N2 | C1 | 1.3576(13) |
| N2 | C3 | 1.3780(16) |
| N2 | C4 | 1.4975(15) |
| N3 | C8 | 1.3451(14) |
| N3 | C9 | 1.3803(14) |
| N3 | B1 | 1.5898(15) |
| N4 | C8 | 1.3576(14) |
| N4 | C10 | 1.3830(16) |
| N4 | C11 | 1.4977(15) |
| C2 | C3 | 1.3528(18) |
| C4 | C5 | 1.5208(19) |
| C4 | C6 | 1.5228(19) |
| C4 | C7 | 1.533(2) |
| C9 | C10 | 1.3547(18) |
| C11 | C12 | 1.5320(18) |
| C11 | C13 | 1.5271(18) |
| C11 | C14 | 1.5237(18) |
| C15 | C16 | 1.4045(17) |
| C15 | C20 | 1.4005(16) |
| C15 | B1 | 1.6224(16) |
| C16 | C17 | 1.3955(18) |
| C17 | C18 | 1.406(2) |
| C18 | C19 | 1.394(2) |
| C18 | C21 | 1.506(2) |
| C19 | C20 | 1.395(2) |
| C22 | C23 | 1.3881(16) |
| C22 | C27 | 1.4047(19) |
| C22 | B1 | 1.6167(17) |
| C23 | C24 | 1.3971(19) |
| C24 | C25 | 1.380(2) |
| C25 | C26 | 1.388(3) |
| C25 | C28 | 1.499(2) |
| C26 | C27 | 1.388(2) |

9.3.10 Bond Angles

Table S22. Bond Angles in ° for **3a**.

| Atom | Atom | Atom | Angle/° |
|---|---|---|---|
| Br2 | Bi1 | Br1 | 172.728(8) |
| C1 | Bi1 | Br1 | 85.45(3) |
| C1 | Bi1 | Br2 | 87.30(3) |
| C8 | Bi1 | Br1 | 85.30(3) |
| C8 | Bi1 | Br2 | 95.11(3) |
| C8 | Bi1 | C1 | 89.01(4) |
| C2 | N1 | C1 | 108.01(9) |
| B1 | N1 | C1 | 132.94(9) |
| B1 | N1 | C2 | 118.43(9) |
| C3 | N2 | C1 | 108.35(9) |
| C4 | N2 | C1 | 131.27(10) |
| C4 | N2 | C3 | 120.37(10) |
| C9 | N3 | C8 | 108.17(9) |
| B1 | N3 | C8 | 131.74(9) |
| B1 | N3 | C9 | 118.59(9) |
| C10 | N4 | C8 | 108.40(10) |
| C11 | N4 | C8 | 131.57(10) |
| C11 | N4 | C10 | 120.02(10) |
| N1 | C1 | Bi1 | 123.73(7) |
| N2 | C1 | Bi1 | 127.79(8) |
| N2 | C1 | N1 | 108.37(9) |
| C3 | C2 | N1 | 108.22(10) |
| C2 | C3 | N2 | 107.06(10) |
| C5 | C4 | N2 | 112.30(10) |
| C6 | C4 | N2 | 107.41(11) |
| C6 | C4 | C5 | 108.93(12) |
| C7 | C4 | N2 | 107.65(11) |
| C7 | C4 | C5 | 107.92(13) |
| C7 | C4 | C6 | 112.69(13) |
| N3 | C8 | Bi1 | 122.73(7) |
| N4 | C8 | Bi1 | 128.11(7) |
| N4 | C8 | N3 | 108.24(9) |
| C10 | C9 | N3 | 108.20(10) |
| C9 | C10 | N4 | 106.98(10) |
| C12 | C11 | N4 | 107.52(10) |
| C13 | C11 | N4 | 108.01(10) |
| C13 | C11 | C12 | 111.05(11) |
| C14 | C11 | N4 | 111.65(9) |
| C14 | C11 | C12 | 109.09(10) |
| C14 | C11 | C13 | 109.52(12) |
| C20 | C15 | C16 | 117.10(11) |
| B1 | C15 | C16 | 120.47(10) |
| B1 | C15 | C20 | 121.95(10) |
| C17 | C16 | C15 | 121.40(12) |
| C18 | C17 | C16 | 120.82(13) |
| C19 | C18 | C17 | 117.96(12) |
| C21 | C18 | C17 | 120.65(17) |
| C21 | C18 | C19 | 121.38(16) |
| C20 | C19 | C18 | 120.91(13) |
| C19 | C20 | C15 | 121.72(13) |
| C27 | C22 | C23 | 115.50(12) |
| B1 | C22 | C23 | 123.99(10) |
| B1 | C22 | C27 | 120.51(11) |
| C24 | C23 | C22 | 121.93(13) |
| C25 | C24 | C23 | 121.87(14) |
| C26 | C25 | C24 | 117.05(13) |
| C28 | C25 | C24 | 122.10(17) |
| C28 | C25 | C26 | 120.84(17) |
| C27 | C26 | C25 | 121.13(15) |
| C26 | C27 | C22 | 122.49(15) |
| N3 | B1 | N1 | 111.85(9) |
| C15 | B1 | N1 | 105.27(9) |
| C15 | B1 | N3 | 108.05(8) |
| C22 | B1 | N1 | 109.40(9) |
| C22 | B1 | N3 | 107.53(9) |
| C22 | B1 | C15 | 114.80(9) |

9.3.11 Torsion Angles

Table S23. Torsion Angles in ° for **3a**.

| Atom | Atom | Atom | Atom | Angle/° |
|---|---|---|---|---|
| Bi1 | C1 | N1 | C2 | -176.63(10) |
| Bi1 | C1 | N1 | B1 | 12.79(12) |
| Bi1 | C1 | N2 | C3 | 176.47(11) |
| Bi1 | C1 | N2 | C4 | -2.36(13) |
| Bi1 | C8 | N3 | C9 | 170.19(10) |
| Bi1 | C8 | N3 | B1 | -24.42(12) |
| Bi1 | C8 | N4 | C10 | -169.98(11) |
| Bi1 | C8 | N4 | C11 | 11.15(12) |
| N1 | C1 | N2 | C3 | 0.33(11) |
| N1 | C1 | N2 | C4 | -178.50(9) |
| N1 | C2 | C3 | N2 | 0.05(12) |
| N1 | B1 | N3 | C8 | 36.14(13) |
| N1 | B1 | N3 | C9 | -159.70(10) |
| N1 | B1 | C15 | C16 | 68.28(10) |
| N1 | B1 | C15 | C20 | -103.58(10) |
| N1 | B1 | C22 | C23 | -1.30(12) |
| N1 | B1 | C22 | C27 | 179.23(13) |
| N3 | C8 | N4 | C10 | -0.85(11) |
| N3 | C8 | N4 | C11 | -179.72(8) |
| N3 | C9 | C10 | N4 | -0.81(12) |
| N3 | B1 | C15 | C16 | -51.37(11) |
| N3 | B1 | C15 | C20 | 136.76(10) |
| N3 | B1 | C22 | C23 | 120.37(10) |
| N3 | B1 | C22 | C27 | -59.10(14) |
| C15 | C16 | C17 | C18 | 1.18(15) |
| C15 | C20 | C19 | C18 | 0.84(16) |
| C15 | B1 | C22 | C23 | -119.37(11) |
| C15 | B1 | C22 | C27 | 61.17(15) |
| C16 | C17 | C18 | C19 | -2.98(16) |
| C16 | C17 | C18 | C21 | 176.48(14) |
| C17 | C18 | C19 | C20 | 1.99(15) |
| C22 | C23 | C24 | C25 | 0.92(17) |
| C22 | C27 | C26 | C25 | 0.9(3) |
| C23 | C24 | C25 | C26 | -0.0(2) |
| C23 | C24 | C25 | C28 | -179.84(15) |
| C24 | C25 | C26 | C27 | -0.85(19) |

9.3.12 Hydrogen Fractional Atomic Coordinates and Equivalent Isotropic Displacement Parameters

Table S24. Hydrogen Fractional Atomic Coordinates (×$10^4$) and Equivalent Isotropic Displacement Parameters (Å$^2$×$10^3$) for **3a**. $U_{eq}$ is defined as 1/3 of the trace of the orthogonalised $U_{ij}$.

| Atom | x | y | z | $U_{eq}$ |
|---|---|---|---|---|
| H2 | 7269.5(8) | 2756.8(11) | 8206.7(8) | 30.3(2) |
| H3 | 8699.2(8) | 3297.6(12) | 8980.4(8) | 31.2(2) |
| H5a | 10072(8) | 4793(4) | 6596(3) | 42.4(4) |
| H5b | 9668(5) | 5959(6) | 7067(6) | 42.4(4) |
| H5c | 10673(3) | 5532(9) | 7387(4) | 42.4(4) |
| H6a | 10091(6) | 3289(8) | 8769(6) | 55.3(5) |
| H6b | 10279(7) | 3148(6) | 7614(4) | 55.3(5) |
| H6c | 10908.0(19) | 3991.4(19) | 8293(11) | 55.3(5) |
| H7a | 9173(7) | 5989(7) | 8582(3) | 55.9(5) |
| H7b | 9370(9) | 4917(3) | 9323(4) | 55.9(5) |
| H7c | 10193(2) | 5708(10) | 8963(7) | 55.9(5) |
| H9 | 5471.5(7) | 3882.1(11) | 5373.0(9) | 30.5(2) |
| H10 | 5617.4(8) | 5053.5(11) | 3913.7(10) | 31.0(3) |
| H12a | 5979(2) | 6552.6(17) | 3343(9) | 41.7(3) |
| H12b | 6524(6) | 7245(7) | 4209(2) | 41.7(3) |
| H12c | 6792(4) | 7416(6) | 3080(7) | 41.7(3) |
| H13a | 7604(7) | 4594(7) | 2904(3) | 49.6(4) |
| H13b | 6577(2) | 4977(10) | 2645(6) | 49.6(4) |
| H13c | 7400(9) | 5743(4) | 2256(2) | 49.6(4) |
| H14a | 8057(2) | 6822(9) | 4486(5) | 40.5(3) |
| H14b | 8490.4(15) | 5729.6(13) | 3930(9) | 40.5(3) |
| H14c | 8225(3) | 6891(8) | 3323(4) | 40.5(3) |
| H16 | 6079.7(8) | 5113.1(11) | 7127.2(9) | 28.5(2) |
| H17 | 4985.8(8) | 5143.3(13) | 8236.2(10) | 34.8(3) |
| H19 | 4898.8(8) | 1732.2(13) | 8198.3(11) | 37.7(3) |
| H20 | 5960.7(8) | 1707.7(11) | 7056.3(10) | 31.4(3) |
| H21a | 3875(7) | 2749(7) | 9003(7) | 71.9(7) |
| H21b | 4564.9(11) | 3486(14) | 9704.2(13) | 71.9(7) |
| H21c | 3864(7) | 4149(7) | 8963(7) | 71.9(7) |
| H23 | 8323.7(8) | 2005.1(10) | 6552.0(10) | 29.5(2) |
| H24 | 8556.3(9) | 280.0(11) | 5833.6(12) | 37.1(3) |
| H26 | 6220.9(13) | 506.9(15) | 4435.4(14) | 52.7(5) |
| H27 | 5967.4(11) | 2203.6(14) | 5180.5(14) | 50.3(5) |
| H28a | 7604(11) | -682(2) | 3923(2) | 68.6(7) |
| H28b | 8110(6) | -1150(6) | 4917(8) | 68.6(7) |
| H28c | 7035(5) | -1318(5) | 4756(10) | 68.6(7) |

## 9.4 Single crystal structure analysis of 4a

CCDC Deposition number 2423152

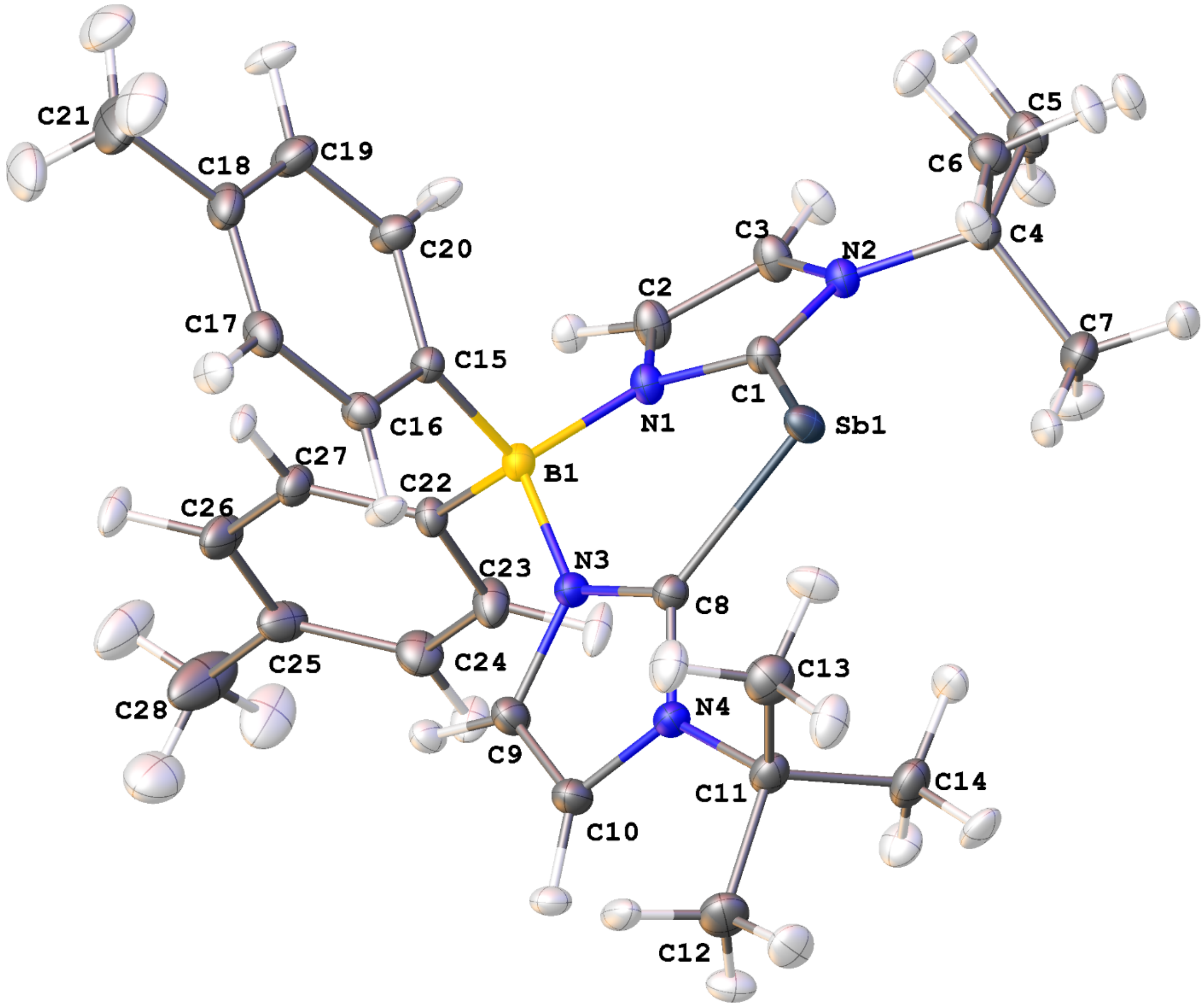


Figure S29. Olex-2 representation of the molecular structure of complex **4a**

### 9.4.1 Experimental

Single yellow block-shaped crystals of **4a** were recrystallised from diethyl ether by slow evaporation. A suitable crystal fragment 0.08×0.06×0.01 $mm^3$ was selected and mounted on a MITIGEN holder in oil on a Rigaku FRE+ diffractometer with Arc)Sec VHF Varimax confocal mirrors, a UG2 goniometer and HyPix 6000HE detector. The crystal was kept at a steady $T$ = 100.06(13) K during data collection. The structure was solved with the ShelXT 2018/2 (Sheldrick, 2018) structure solution program using the dual methods solution method and by using Olex2 1.5-alpha (Dolomanov et al., 2009) as the graphical interface. The model was refined with version of olex2.refine 1.5-alpha (Bourhis et al., 2015) using full matrix least squares minimisation on $\boldsymbol{F}^2$ minimisation.

### 9.4.2 Crystal Data

$C_{28}H_{36}BN_4Sb$, $M_r$ = 561.209, monoclinic, $P2_1/c$ (No. 14), a = 10.2536(2) Å, b = 12.3211(2) Å, c = 22.3697(4) Å, $\beta$ = 102.985(2)°, $\alpha = \gamma$ = 90°, $V$ = 2753.82(9) Å$^3$, $T$ = 100.06(13) K, $Z$ = 4, $Z'$ = 1, $\mu$(Mo $K_\alpha$) = 1.024 mm$^{-1}$, 44639 reflections measured, 9795 unique ($R_{int}$ = 0.0340) which were used in all calculations. The final $wR_2$ was 0.0319 (all data) and $R_1$ was 0.0206 (I≥2 $\sigma$(I)).

| Compound | **4a** |
|---|---|
| | |
| Formula | $C_{28}H_{36}BN_4Sb$ |
| $D_{calc.}$/ g cm$^{-3}$ | 1.354 |
| $\mu$/mm$^{-1}$ | 1.024 |
| Formula Weight | 561.209 |
| Colour | yellow |
| Shape | chip-shaped |
| Size/mm$^3$ | 0.08×0.06×0.01 |
| $T$/K | 100.06(13) |
| Crystal System | monoclinic |
| Space Group | $P2_1/c$ |
| $a$/Å | 10.2536(2) |
| $b$/Å | 12.3211(2) |
| $c$/Å | 22.3697(4) |
| $\alpha$/° | 90 |
| $\beta$/° | 102.985(2) |
| $\gamma$/° | 90 |
| V/Å$^3$ | 2753.82(9) |
| $Z$ | 4 |
| $Z'$ | 1 |
| Wavelength/Å | 0.71075 |
| Radiation type | Mo $K_\alpha$ |
| $\Theta_{min}$/° | 1.90 |
| $\Theta_{max}$/° | 33.58 |
| Measured Refl's. | 44639 |
| Indep't Refl's | 9795 |
| Refl's I≥2 $\sigma$(I) | 8152 |
| $R_{\text{int}}$ | 0.0340 |
| Parameters | 631 |
| Restraints | 216 |
| Largest Peak | 0.6722 |
| Deepest Hole | -0.5934 |
| GooF | 0.9982 |
| $wR_2$ (all data) | 0.0319 |
| $wR_2$ | 0.0294 |
| $R_1$ (all data) | 0.0325 |
| $R_1$ | 0.0206 |

### 9.4.3 Structure Quality Indicators

| | | | | |
|---|---|---|---|---|
| Reflections: | d min (MoKα) 2Θ=67.2° 0.64 | I/σ(I) 32.1 | Rint m=4.71 3.40% | Full 50.5° 90% to 67.2° 100 |
| Refinement: | Shift 0.001 | Max Peak 0.7 | Min Peak -0.6 | GooF 0.998 |

A yellow chip-shaped-shaped crystal with dimensions 0.08×0.06×0.01 $mm^3$ was mounted on a MITIGEN holder in oil. X-ray diffraction data were collected using a Rigaku FRE+ diffractometer with Arc)Sec VHF Varimax confocal mirrors, a UG2 goniometer and HyPix 6000HE detector equipped with an Oxford Cryosystems low-temperature device, operating at $T$ = 100.06(13) K.

Data were measured using profile data from $\omega$-scans of 0.5 ° per frame for 20.0 s using Mo $K_\alpha$ radiation (Rotating Anode, 45.0 kV, 55.0 mA). The total number of runs and images was based on the strategy calculation from the program CrysAlisPro 1.171.42.99a (Rigaku OD, 2023). The maximum resolution achieved was $\Theta$ = 33.58°.

Cell parameters were retrieved using the CrysAlisPro 1.171.42.99a (Rigaku OD, 2023) software and refined using CrysAlisPro 1.171.42.99a (Rigaku OD, 2023) on 23029 reflections, 52 % of the observed reflections. Data reduction was performed using the CrysAlisPro 1.171.42.99a (Rigaku OD, 2023) software which corrects for Lorentz polarisation. The final completeness is 99.98 % out to 33.58° in $\Theta$.

A multi-scan absorption correction was performed using CrysAlisPro 1.171.42.99a (Rigaku OD 2023) Empirical absorption correction using spherical harmonics, implemented in SCALE3 ABSPACK scaling algorithm. The absorption coefficient $\mu$ of this material is 1.024 $mm^{-1}$ at this wavelength ($\lambda$ = 0.71075Å) and the minimum and maximum transmissions are 0.904 and 1.000.

The structure was solved in the space group $P2_1/c$ (# 14) by using dual methods using the ShelXT 2018/2 (Sheldrick, 2018) structure solution program and refined by full matrix least squares minimisation on $\boldsymbol{F^2}$ using olex2.refine 1.5-alpha (Bourhis et al., 2015). All non-hydrogen atoms were refined anisotropically.

*_refine_special_details*: Thermal restraints applied to both MeBz substituents.

*_olex2_refine_details*: Refinement using NoSpherA2, an implementation of NOn-SPHERical Atom-form-factors in Olex2.Please cite: F. Kleemiss et al. Chem. Sci. DOI 10.1039/D0SC05526C – 2021 NoSpherA2 implementation of HAR makes use of tailor-made aspherical atomic form factors calculated on-the-fly from a Hirshfeld-partitioned electron density (ED) - not from spherical-atom form factors. The ED is calculated from a gaussian basis set single determinant SCF wavefunction - either Hartree-Fock or DFT using selected functionals - for a fragment of the crystal. This fragment can be embedded in an electrostatic crystal field by employing cluster charges or modelled using implicit solvation models, depending on the software used. The following options were used: SOFTWARE: ORCA 5.0; PARTITIONING: NoSpherA2; INT ACCURACY: Normal; METHOD:

R2SCAN; BASIS SET: x2c-TZVP; CHARGE: 0; MULTIPLICITY: 1; RELATIVISTIC: DKH2; DATE: 2023-08-16_11-51-35

*_exptl_absorpt_process_details*: CrysAlisPro 1.171.42.99a (Rigaku OD, 2023) using spherical harmonics, implemented in SCALE3 ABSPACK scaling algorithm.

There is a single formula unit in the asymmetric unit, which is represented by the reported sum formula. In other words: Z is 4 and Z' is 1. The moiety formula is C28 H36 B N4 Sb.

## 9.4.4 Data Plots: Diffraction Data

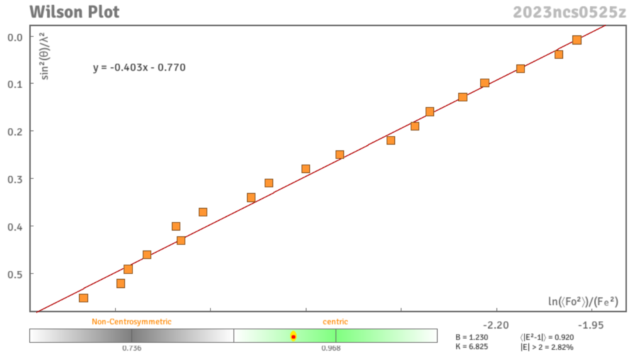


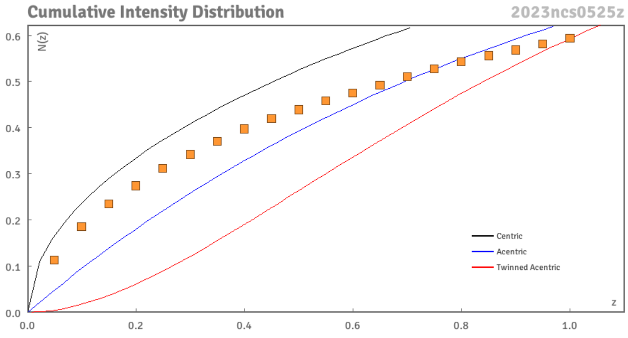


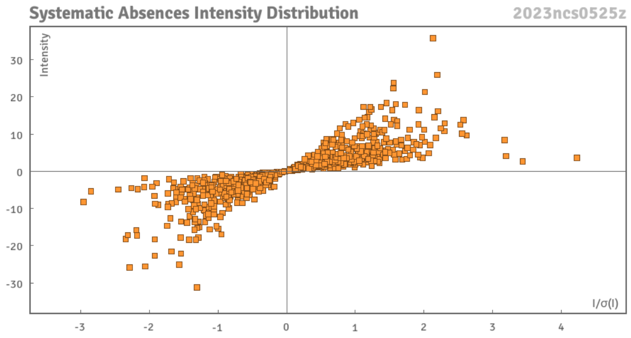


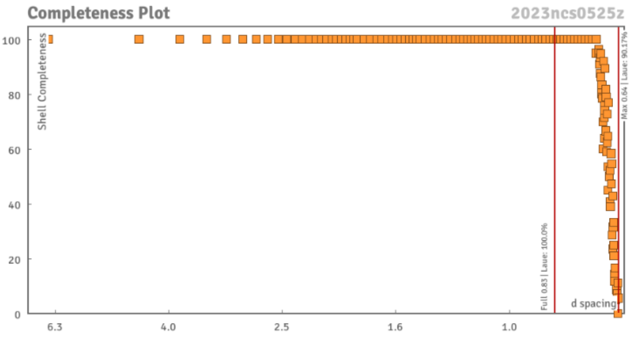


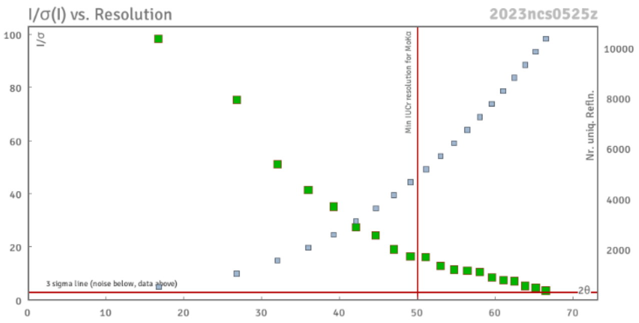


## 9.4.5 Data Plots: Refinement and Data

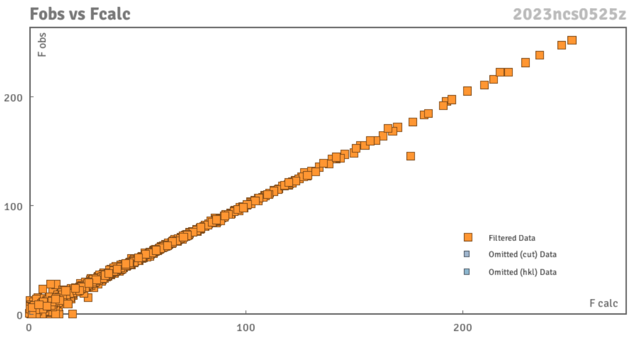


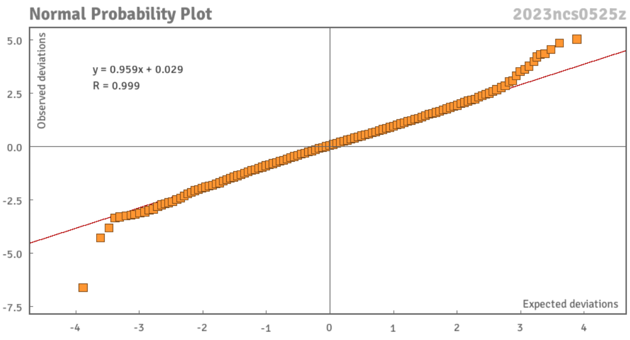

### 9.4.6 Reflection Statistics

| | | | |
|---|---|---|---|
| Total reflections (after filtering) | 46158 | Unique reflections | 9795 |
| Completeness | 0.902 | Mean I/$\sigma$ | 26.52 |
| $hkl_{max}$ collected | (14, 18, 34) | $hkl_{min}$ collected | (-15, -18, -33) |
| $hkl_{max}$ used | (15, 18, 34) | $hkl_{min}$ used | (-15, 0, 0) |
| Lim $d_{max}$ collected | 100.0 | Lim $d_{min}$ collected | 0.36 |
| $d_{max}$ used | 10.9 | $d_{min}$ used | 0.64 |
| Friedel pairs | 10120 | Friedel pairs merged | 1 |
| Inconsistent equivalents | 3 | $R_{int}$ | 0.034 |
| $R_{sigma}$ | 0.0312 | Intensity transformed | 0 |
| Omitted reflections | 0 | Omitted by user (OMIT hkl) | 2 |
| Multiplicity | (16412, 7767, 2183, 977, 422, 163, 55, 24, 10) | Maximum multiplicity | 20 |
| Removed systematic absences | 1519 | Filtered off (Shel/OMIT) | 0 |

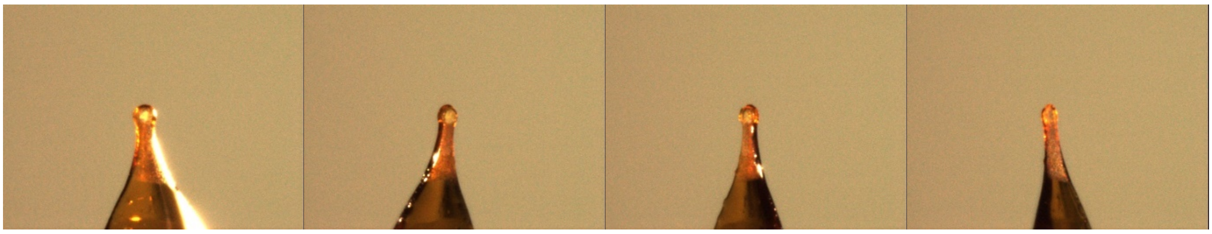

### 9.4.7 Fractional Atomic Coordinates and Equivalent Isotropic Displacement Parameters

Table S25. Fractional Atomic Coordinates (×10[4]) and Equivalent Isotropic Displacement Parameters (Å$^2$×10$^3$) for **4a**. $U_{eq}$ is defined as 1/3 of the trace of the orthogonalised $U_{ij}$.

| Atom | x | y | z | $U_{eq}$ |
|---|---|---|---|---|
| Sb1 | 2747.68(5) | 6081.58(5) | 2463.79(3) | 16.246(18) |
| N1 | 1743.4(7) | 4266.2(6) | 3213.8(3) | 14.39(14) |
| N2 | 448.3(7) | 4273.0(6) | 2288.0(3) | 14.61(15) |
| N3 | 4176.4(7) | 4814.1(6) | 3625.3(3) | 14.60(15) |
| N4 | 5716.2(7) | 5533.1(7) | 3208.6(3) | 16.33(16) |
| C1 | 1528.2(8) | 4762.3(8) | 2661.4(4) | 14.20(17) |
| C2 | 788.8(9) | 3466.9(8) | 3190.5(4) | 18.32(19) |
| C3 | -19.7(9) | 3465.5(8) | 2618.6(4) | 18.15(19) |
| C4 | -100.4(8) | 4481.5(8) | 1620.2(4) | 15.91(18) |
| C5 | -1404.8(10) | 3846.3(10) | 1405.2(5) | 21.6(2) |
| C6 | -397.7(10) | 5691.2(9) | 1524.3(5) | 19.43(19) |
| C7 | 920.4(11) | 4102.4(11) | 1261.0(5) | 24.4(2) |
| C8 | 4367.9(8) | 5448.1(8) | 3159.9(4) | 14.60(17) |
| C9 | 5412.4(9) | 4487.9(9) | 3966.0(4) | 19.04(19) |
| C10 | 6368.2(9) | 4928.6(9) | 3710.0(4) | 20.2(2) |
| C11 | 6404.5(8) | 6095.4(9) | 2774.1(4) | 18.74(18) |
| C12 | 7917.4(10) | 6091.9(12) | 3041.5(5) | 26.3(2) |
| C13 | 5927.3(11) | 7274.9(10) | 2694.7(5) | 24.6(2) |
| C14 | 6107.2(11) | 5471.5(11) | 2166.7(5) | 24.1(2) |
| C15 | 2327.5(8) | 5619.6(8) | 4148.4(4) | 14.05(17) |
| C16 | 3211.9(9) | 6423.2(8) | 4441.5(4) | 16.49(18) |
| C17 | 2829.8(9) | 7195.9(8) | 4824.5(4) | 20.20(19) |
| C18 | 1543.9(10) | 7199.9(9) | 4933.8(4) | 22.2(2) |
| C19 | 636.4(10) | 6425.6(9) | 4631.1(4) | 23.4(2) |
| C20 | 1024.5(9) | 5658.9(9) | 4247.2(4) | 19.38(19) |
| C21 | 1141.5(14) | 8008.2(12) | 5364.6(5) | 38.2(3) |
| C22 | 3034.5(8) | 3526.3(8) | 4267.1(4) | 14.65(17) |
| C23 | 3448.9(9) | 2529.9(8) | 4068.3(4) | 21.3(2) |
| C24 | 3667.3(9) | 1617.2(9) | 4443.3(4) | 22.1(2) |
| C25 | 3497.8(9) | 1662.4(9) | 5045.0(4) | 23.5(2) |
| C26 | 3087.0(9) | 2645.0(9) | 5250.1(4) | 24.6(2) |
| C27 | 2856.1(9) | 3550.9(9) | 4867.1(4) | 18.34(19) |
| C28 | 3793.9(15) | 699.1(12) | 5462.7(7) | 47.3(4) |
| B1 | 2813.5(9) | 4578.8(9) | 3816.1(4) | 13.97(19) |

9.4.8 Anisotropic Displacement Parameters

Table S26. Anisotropic Displacement Parameters (×10^4) for **4a**. The anisotropic displacement factor exponent takes the form: $-2\pi^2[h^2a^{*2} \times U_{11}+ ... +2hka^* \times b^* \times U_{12}]$

| Atom | $U_{11}$ | $U_{22}$ | $U_{33}$ | $U_{23}$ | $U_{13}$ | $U_{12}$ |
|---|---|---|---|---|---|---|
| Sb1 | 14.50(3) | 17.25(3) | 16.41(3) | -0.37(3) | 2.255(19) | 5.29(3) |
| N1 | 16.8(3) | 14.1(4) | 11.6(3) | -1.6(3) | 2.0(3) | 0.7(3) |
| N2 | 16.1(3) | 14.7(4) | 12.5(3) | -0.6(3) | 2.0(3) | 0.1(3) |
| N3 | 13.7(3) | 17.5(4) | 12.7(3) | 1.8(3) | 3.4(3) | 2.1(3) |
| N4 | 13.6(3) | 21.4(4) | 14.4(3) | 0.9(3) | 4.1(3) | 0.3(3) |
| C1 | 15.5(4) | 14.6(5) | 12.6(4) | -0.3(3) | 3.3(3) | 0.4(3) |
| C2 | 22.7(4) | 17.0(5) | 14.5(4) | -4.9(4) | 2.6(3) | 2.0(4) |
| H2 | 33(7) | 32(9) | 32(7) | -9(6) | 11(5) | 4(6) |
| C3 | 20.4(4) | 16.8(5) | 15.8(4) | -5.1(4) | 1.1(3) | -0.1(4) |
| H3 | 39(7) | 33(9) | 44(7) | -17(7) | -6(6) | 4(6) |
| C4 | 16.4(4) | 19.0(5) | 11.8(4) | 1.3(4) | 2.1(3) | -1.4(3) |
| C5 | 21.1(5) | 23.7(6) | 17.9(5) | -1.9(5) | -0.1(4) | -2.7(5) |
| H5a | 44(8) | 40(10) | 47(8) | -3(7) | -1(6) | 8(7) |
| H5b | 30(7) | 52(10) | 33(7) | 6(6) | 14(6) | -7(6) |
| H5c | 41(8) | 66(11) | 26(7) | -11(7) | -6(6) | 8(7) |
| C6 | 19.3(5) | 19.5(5) | 18.6(5) | 1.6(4) | 2.3(4) | 3.4(4) |
| H6a | 61(9) | 38(10) | 47(8) | -1(7) | 29(7) | 6(7) |
| H6b | 35(7) | 37(9) | 31(7) | -13(6) | 2(6) | 4(6) |
| H6c | 54(8) | 32(9) | 27(7) | -5(6) | -6(6) | 8(6) |
| C7 | 23.1(5) | 34.4(7) | 16.4(5) | 5.4(5) | 5.8(4) | -3.7(5) |
| H7a | 36(8) | 61(11) | 27(7) | 4(7) | 1(6) | -12(7) |
| H7b | 46(9) | 37(10) | 75(10) | 2(8) | 22(7) | -3(8) |
| H7c | 32(7) | 80(12) | 29(7) | -2(7) | 6(6) | -4(7) |
| C8 | 14.2(4) | 16.2(5) | 13.9(4) | 0.8(3) | 4.1(3) | 1.6(3) |
| C9 | 16.1(4) | 25.3(6) | 15.3(4) | 2.9(4) | 2.5(3) | 4.3(4) |
| H9 | 31(7) | 48(10) | 33(7) | 8(6) | 8(5) | 14(6) |
| C10 | 14.3(4) | 28.6(6) | 17.6(4) | 3.1(4) | 3.3(3) | 2.5(4) |
| H10 | 27(7) | 47(10) | 44(7) | 11(6) | 9(6) | 7(7) |
| C11 | 16.7(4) | 23.6(5) | 17.3(4) | -2.1(4) | 6.9(3) | -0.7(4) |
| C12 | 17.6(4) | 35.9(7) | 26.6(5) | -3.5(5) | 7.6(4) | 0.3(6) |
| H12a | 24(7) | 71(12) | 44(8) | 3(7) | 5(6) | -19(8) |
| H12b | 32(8) | 38(10) | 61(9) | 8(7) | 9(6) | 10(8) |
| H12c | 43(8) | 66(11) | 52(8) | -8(8) | 22(7) | 13(8) |
| C13 | 25.2(5) | 23.4(6) | 26.6(6) | -3.5(4) | 9.1(5) | 2.4(5) |
| H13a | 27(7) | 48(10) | 65(9) | -7(7) | 2(6) | 3(7) |
| H13b | 74(10) | 48(11) | 42(8) | 0(8) | 17(7) | -12(7) |
| H13c | 80(10) | 42(10) | 42(8) | -1(8) | 26(7) | 4(7) |
| C14 | 24.3(5) | 31.8(7) | 19.0(5) | -2.5(5) | 10.8(4) | -2.3(5) |
| H14a | 59(9) | 30(9) | 49(8) | 4(7) | 18(7) | -4(7) |
| H14b | 29(7) | 63(11) | 37(7) | -11(7) | 3(6) | -17(7) |
| H14c | 41(8) | 64(11) | 48(8) | -5(7) | 31(7) | -7(7) |
| C15 | 14.7(4) | 15.0(5) | 12.6(4) | 0.5(3) | 3.3(3) | -0.6(3) |
| C16 | 16.2(4) | 16.1(5) | 16.5(4) | 0.0(3) | 2.0(3) | -1.1(3) |
| H16 | 21(4) | 44(8) | 48(7) | -6(2) | 13(2) | -14(5) |
| C17 | 24.3(5) | 16.8(5) | 17.1(4) | 2.0(4) | -0.3(3) | -3.3(4) |
| H17 | 40(5) | 37(6) | 44(6) | -8(3) | 5(3) | -21(3) |
| C18 | 28.7(5) | 22.3(5) | 15.6(4) | 6.6(4) | 5.0(3) | -2.0(4) |
| C19 | 21.4(5) | 26.5(6) | 25.1(5) | 3.4(4) | 10.9(4) | -3.0(4) |
| H19 | 29(4) | 43(8) | 72(7) | -4(3) | 27(2) | -20(5) |

| | | | | | | |
|---|---|---|---|---|---|---|
| C20 | 16.0(4) | 20.1(5) | 22.9(4) | -0.6(4) | 6.1(3) | -4.0(4) |
| H20 | 25(5) | 43(6) | 59(7) | -12(3) | 19(3) | -26(4) |
| C21 | 49.0(7) | 40.0(8) | 26.2(6) | 14.1(6) | 9.6(5) | -12.2(5) |
| H21a | 105(7) | 90(9) | 43(4) | 28(4) | 3(2) | -21(2) |
| H21b | 122(10) | 52(5) | 75(8) | 14(3) | 36(4) | -12(2) |
| H21c | 69(4) | 80(9) | 84(8) | 13(2) | 29(2) | -28(4) |
| C22 | 17.6(4) | 15.0(5) | 11.2(4) | 0.4(3) | 2.8(3) | 1.2(3) |
| C23 | 31.5(5) | 17.4(5) | 14.5(4) | 5.3(4) | 4.1(4) | 1.0(4) |
| H23 | 95(9) | 31(7) | 26(4) | 21(5) | 26(2) | 5(2) |
| C24 | 25.9(5) | 16.8(5) | 23.1(5) | 4.7(4) | 4.3(4) | 4.4(4) |
| H24 | 71(9) | 25(5) | 40(6) | 17(3) | 19(4) | 5(2) |
| C25 | 22.1(4) | 24.6(6) | 26.1(5) | 6.5(4) | 10.2(4) | 12.3(4) |
| C26 | 27.6(5) | 29.8(6) | 20.2(5) | 8.7(4) | 13.1(4) | 10.3(4) |
| H26 | 88(9) | 51(8) | 31(4) | 29(5) | 32(2) | 16(2) |
| C27 | 21.3(4) | 20.4(5) | 15.0(4) | 3.3(4) | 7.7(3) | 3.4(4) |
| H27 | 48(7) | 29(4) | 19(5) | 13(3) | 14(3) | 6(2) |
| C28 | 60.2(8) | 42.1(8) | 51.4(8) | 29.3(7) | 37.6(7) | 31.5(7) |
| H28a | 138(10) | 49(5) | 79(7) | 32(3) | 45(4) | 28(2) |
| H28b | 91(5) | 86(10) | 90(7) | 37(3) | 28(2) | 29(4) |
| H28c | 127(7) | 79(9) | 92(5) | 40(4) | 76(3) | 45(3) |
| B1 | 15.9(4) | 14.4(5) | 11.7(4) | 0.3(4) | 3.2(3) | 0.3(4) |

### 9.4.9 Bond lengths

Table S27. Bond Lengths in Å for **4a**.

| Atom | Atom | Length/Å |
|---|---|---|
| Sb1 | C1 | 2.1563(9) |
| Sb1 | C8 | 2.1521(8) |
| N1 | C1 | 1.3516(10) |
| N1 | C2 | 1.3812(12) |
| N1 | B1 | 1.5823(12) |
| N2 | C1 | 1.3681(11) |
| N2 | C3 | 1.3880(11) |
| N2 | C4 | 1.4954(10) |
| N3 | C8 | 1.3507(11) |
| N3 | C9 | 1.3837(11) |
| N3 | B1 | 1.5771(11) |
| N4 | C8 | 1.3662(10) |
| N4 | C10 | 1.3866(12) |
| N4 | C11 | 1.4938(11) |
| C2 | C3 | 1.3591(12) |
| C4 | C5 | 1.5309(13) |
| C4 | C6 | 1.5268(14) |
| C4 | C7 | 1.5289(12) |
| C9 | C10 | 1.3548(13) |
| C11 | C12 | 1.5321(13) |
| C11 | C13 | 1.5309(15) |
| C11 | C14 | 1.5309(14) |
| C15 | C16 | 1.4019(13) |
| C15 | C20 | 1.4032(11) |
| C15 | B1 | 1.6161(13) |
| C16 | C17 | 1.3945(13) |
| C17 | C18 | 1.3941(13) |
| C18 | C19 | 1.3969(14) |
| C18 | C21 | 1.5064(14) |
| C19 | C20 | 1.3935(13) |
| C22 | C23 | 1.4038(13) |
| C22 | C27 | 1.3951(11) |
| C22 | B1 | 1.6273(13) |
| C23 | C24 | 1.3906(13) |
| C24 | C25 | 1.3964(13) |
| C25 | C26 | 1.3930(15) |
| C25 | C28 | 1.4988(15) |
| C26 | C27 | 1.3946(14) |

9.4.10 Bond angles

Table S28. Bond Angles in ° for **4a**.

| Atom | Atom | Atom | Angle/° |
|---|---|---|---|
| C8 | Sb1 | C1 | 88.12(3) |
| C2 | N1 | C1 | 108.62(7) |
| B1 | N1 | C1 | 127.44(7) |
| B1 | N1 | C2 | 123.76(7) |
| C3 | N2 | C1 | 108.66(7) |
| C4 | N2 | C1 | 127.25(7) |
| C4 | N2 | C3 | 123.91(7) |
| C9 | N3 | C8 | 108.59(7) |
| B1 | N3 | C8 | 127.30(7) |
| B1 | N3 | C9 | 123.72(7) |
| C10 | N4 | C8 | 108.58(7) |
| C11 | N4 | C8 | 126.96(7) |
| C11 | N4 | C10 | 124.27(7) |
| N1 | C1 | Sb1 | 123.05(6) |
| N2 | C1 | Sb1 | 129.36(6) |
| N2 | C1 | N1 | 107.60(7) |
| C3 | C2 | N1 | 108.28(8) |
| C2 | C3 | N2 | 106.84(8) |
| C5 | C4 | N2 | 109.39(7) |
| C6 | C4 | N2 | 109.06(7) |
| C6 | C4 | C5 | 108.79(8) |
| C7 | C4 | N2 | 108.92(7) |
| C7 | C4 | C5 | 109.37(8) |
| C7 | C4 | C6 | 111.29(8) |
| N3 | C8 | Sb1 | 123.03(6) |
| N4 | C8 | Sb1 | 129.25(6) |
| N4 | C8 | N3 | 107.62(7) |
| C10 | C9 | N3 | 108.10(8) |
| C9 | C10 | N4 | 107.11(8) |
| C12 | C11 | N4 | 109.24(8) |
| C13 | C11 | N4 | 109.29(7) |
| C13 | C11 | C12 | 108.49(9) |
| C14 | C11 | N4 | 108.39(8) |
| C14 | C11 | C12 | 109.11(8) |
| C14 | C11 | C13 | 112.27(9) |
| C20 | C15 | C16 | 116.15(8) |
| B1 | C15 | C16 | 122.79(7) |
| B1 | C15 | C20 | 120.18(8) |
| C17 | C16 | C15 | 121.87(8) |
| C18 | C17 | C16 | 121.17(9) |
| C19 | C18 | C17 | 117.76(9) |
| C21 | C18 | C17 | 121.36(10) |
| C21 | C18 | C19 | 120.88(9) |
| C20 | C19 | C18 | 120.69(9) |
| C19 | C20 | C15 | 122.30(9) |
| C27 | C22 | C23 | 115.99(9) |
| B1 | C22 | C23 | 120.70(7) |
| B1 | C22 | C27 | 123.30(8) |
| C24 | C23 | C22 | 122.36(8) |
| C25 | C24 | C23 | 120.74(10) |
| C26 | C25 | C24 | 117.68(9) |
| C28 | C25 | C24 | 121.17(10) |
| C28 | C25 | C26 | 121.11(9) |
| C27 | C26 | C25 | 121.02(8) |
| C26 | C27 | C22 | 122.20(9) |
| N3 | B1 | N1 | 107.54(6) |
| C15 | B1 | N1 | 111.00(7) |
| C15 | B1 | N3 | 111.24(7) |
| C22 | B1 | N1 | 107.89(7) |
| C22 | B1 | N3 | 107.81(7) |
| C22 | B1 | C15 | 111.20(7) |

9.4.11 Torsion angles

Table S29. Torsion Angles in ° for **4a**.

| Atom | Atom | Atom | Atom | Angle/° |
|---|---|---|---|---|
| Sb1 | C1 | N1 | C2 | 179.56(7) |
| Sb1 | C1 | N1 | B1 | 4.35(9) |
| Sb1 | C1 | N2 | C3 | -179.50(8) |
| Sb1 | C1 | N2 | C4 | 5.24(10) |
| Sb1 | C8 | N3 | C9 | 176.09(7) |
| Sb1 | C8 | N3 | B1 | -10.92(9) |
| Sb1 | C8 | N4 | C10 | -175.72(8) |
| Sb1 | C8 | N4 | C11 | -0.54(10) |
| N1 | C1 | N2 | C3 | 0.57(8) |
| N1 | C1 | N2 | C4 | -174.68(6) |
| N1 | C2 | C3 | N2 | 0.10(8) |
| N1 | B1 | N3 | C8 | 48.25(10) |
| N1 | B1 | N3 | C9 | -139.74(7) |
| N1 | B1 | C15 | C16 | -143.16(7) |
| N1 | B1 | C15 | C20 | 47.98(8) |
| N1 | B1 | C22 | C23 | 57.62(8) |
| N1 | B1 | C22 | C27 | -123.60(7) |
| N3 | C8 | N4 | C10 | 0.70(8) |
| N3 | C8 | N4 | C11 | 175.88(7) |
| N3 | C9 | C10 | N4 | 0.14(9) |
| N3 | B1 | C15 | C16 | -23.46(9) |
| N3 | B1 | C15 | C20 | 167.68(7) |
| N3 | B1 | C22 | C23 | -58.25(8) |
| N3 | B1 | C22 | C27 | 120.53(7) |
| C15 | C16 | C17 | C18 | 0.16(11) |
| C15 | C20 | C19 | C18 | 0.38(11) |
| C15 | B1 | C22 | C23 | 179.57(7) |
| C15 | B1 | C22 | C27 | -1.66(9) |
| C16 | C17 | C18 | C19 | -2.04(11) |
| C16 | C17 | C18 | C21 | 177.81(10) |
| C17 | C18 | C19 | C20 | 1.77(11) |
| C22 | C23 | C24 | C25 | -0.92(11) |
| C22 | C27 | C26 | C25 | -0.65(10) |
| C23 | C24 | C25 | C26 | 0.87(11) |
| C23 | C24 | C25 | C28 | -177.03(11) |
| C24 | C25 | C26 | C27 | -0.11(10) |

### 9.4.12 Hydrogen Fractional Atomic Coordinates and Equivalent Isotropic Displacement Parameters

Table S30. Hydrogen Fractional Atomic Coordinates (×10$^4$) and Equivalent Isotropic Displacement Parameters (Å$^2$×10$^3$) for **4a**. $U_{eq}$ is defined as 1/3 of the trace of the orthogonalised $U_{ij}$.

| Atom | x | y | z | $U_{eq}$ |
|---|---|---|---|---|
| H2 | 764(10) | 2971(10) | 3583(5) | 32(3) |
| H3 | -844(11) | 2982(10) | 2427(5) | 41(3) |
| H5a | -1247(11) | 2978(11) | 1432(5) | 45(3) |
| H5b | -2144(10) | 4070(10) | 1666(5) | 37(3) |
| H5c | -1809(11) | 4064(11) | 927(5) | 46(4) |
| H6a | -1037(12) | 5983(10) | 1818(5) | 46(4) |
| H6b | 528(11) | 6184(10) | 1610(5) | 35(3) |
| H6c | -902(11) | 5841(10) | 1052(5) | 40(3) |
| H7a | 1886(11) | 4497(11) | 1427(5) | 42(4) |
| H7b | 1052(12) | 3238(12) | 1321(6) | 51(4) |
| H7c | 535(11) | 4263(12) | 774(5) | 47(4) |
| H9 | 5505(10) | 3996(10) | 4364(5) | 37(3) |
| H10 | 7443(10) | 4869(10) | 3855(5) | 39(3) |
| H12a | 8166(10) | 6446(11) | 3492(5) | 47(4) |
| H12b | 8305(11) | 5278(12) | 3066(5) | 44(3) |
| H12c | 8371(12) | 6570(12) | 2738(5) | 52(4) |
| H13a | 4911(12) | 7311(10) | 2457(5) | 48(3) |
| H13b | 6049(12) | 7661(11) | 3129(5) | 54(4) |
| H13c | 6482(13) | 7718(11) | 2426(5) | 53(4) |
| H14a | 6406(12) | 4637(11) | 2262(5) | 45(3) |
| H14b | 5044(11) | 5490(11) | 1942(5) | 44(4) |
| H14c | 6647(11) | 5821(11) | 1858(5) | 47(4) |
| H16 | 4224(10) | 6439(10) | 4372(5) | 37(3) |
| H17 | 3534(11) | 7805(10) | 5042(5) | 41(3) |
| H19 | -369(11) | 6400(10) | 4695(5) | 45(3) |
| H20 | 306(10) | 5035(10) | 4039(5) | 41(3) |
| H21a | 1700(14) | 7949(13) | 5792(6) | 81(4) |
| H21b | 1264(16) | 8862(14) | 5215(7) | 81(4) |
| H21c | 124(15) | 7930(13) | 5366(6) | 76(4) |
| H23 | 3595(12) | 2475(10) | 3603(5) | 49(3) |
| H24 | 3986(12) | 859(10) | 4270(5) | 44(3) |
| H26 | 2965(12) | 2710(11) | 5717(5) | 53(4) |
| H27 | 2571(10) | 4287(9) | 5048(4) | 31(3) |
| H28a | 3788(17) | -41(14) | 5210(7) | 85(4) |
| H28b | 4847(17) | 712(14) | 5724(7) | 88(4) |
| H28c | 3330(16) | 657(13) | 5795(7) | 91(4) |

## 9.5 Single crystal structure analysis of 4b

CCDC Deposition number 2423153

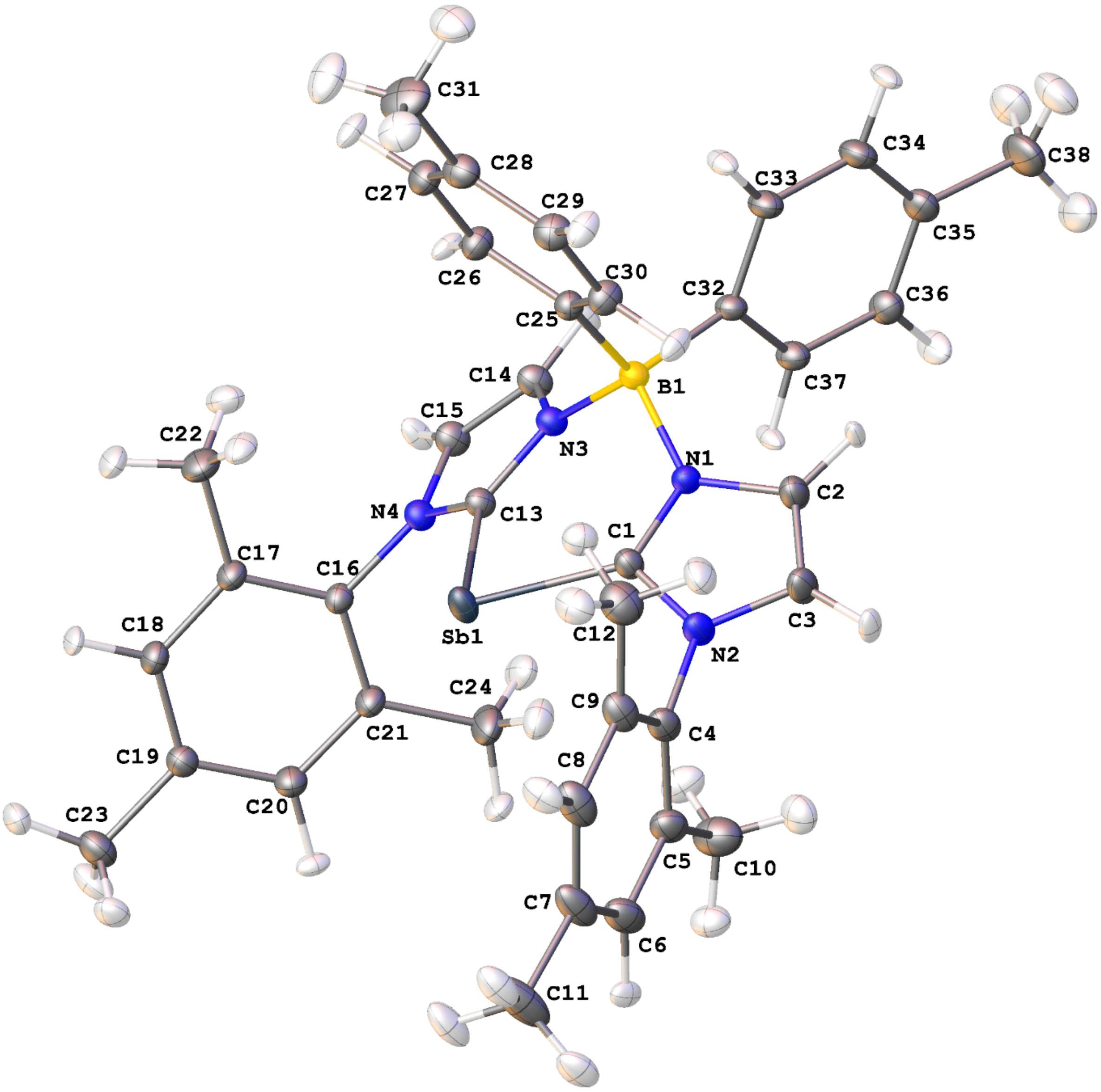


Figure S30. Olex-2 representation of the molecular structure of complex **4b**

### 9.5.1 Experimental

Single yellow lath-shaped crystals of **4b** were recrystallised from hexane. A suitable crystal 0.13×0.03×0.02 $mm^3$ was selected and mounted on a MITIGEN holder in oil on a Rigaku FRE+ diffractometer with Arc)Sec VHF Varimax confocal mirrors, a UG2 goniometer and HyPix 6000HE detector. The crystal was kept at a steady $T$ = 100(2) K during data collection. The structure was solved with the ShelXT 2018/2 (Sheldrick, 2015) structure solution program using the using dual methods solution method and by using Olex2 1.5-alpha (Dolomanov et al., 2009) as the graphical interface. The model was refined with version of olex2.refine 1.5-alpha (Bourhis et al., 2015) using full matrix least squares minimisation on $\boldsymbol{F}^2$ minimisation.

### 9.5.2 Crystal Data

$C_{38}H_{40}BN_4Sb$, $M_r$ = 685.352, monoclinic, $P2_1/n$ (No. 14), a = 8.0754(2) Å, b = 22.7176(5) Å, c = 18.7799(5) Å, $\beta$ = 98.925(3)°, $\alpha = \gamma$ = 90°, $V$ = 3403.53(15) Å$^3$, $T$ = 100(2) K, $Z$ = 4, $Z'$ = 1, $\mu$(Mo $K_\alpha$) = 0.842 mm$^{-1}$, 98108 reflections measured, 12980 unique ($R_{int}$ = 0.0735) which were used in all calculations. The final $wR_2$ was 0.0496 (all data) and $R_1$ was 0.0255 (I≥2 $\sigma$(I)).

| Compound | 4b |
|---|---|
| | |
| Formula | $C_{38}H_{40}BN_4Sb$ |
| $D_{calc.}$/ g cm$^{-3}$ | 1.337 |
| $\mu$/mm$^{-1}$ | 0.842 |
| Formula Weight | 685.352 |
| Colour | yellow |
| Shape | lath-shaped |
| Size/mm$^3$ | 0.13×0.03×0.02 |
| $T$/K | 100(2) |
| Crystal System | monoclinic |
| Space Group | $P2_1/n$ |
| $a$/Å | 8.0754(2) |
| $b$/Å | 22.7176(5) |
| $c$/Å | 18.7799(5) |
| $\alpha$/° | 90 |
| $\beta$/° | 98.925(3) |
| $\gamma$/° | 90 |
| V/Å$^3$ | 3403.53(15) |
| $Z$ | 4 |
| $Z'$ | 1 |
| Wavelength/Å | 0.71073 |
| Radiation type | Mo $K_\alpha$ |
| $\Theta_{min}$/° | 2.10 |
| $\Theta_{max}$/° | 33.14 |
| Measured Refl's. | 98108 |
| Indep't Refl's | 12980 |
| Refl's I≥2 $\sigma$(I) | 9961 |
| $R_{\rm int}$ | 0.0735 |
| Parameters | 757 |
| Restraints | 741 |
| Largest Peak | 1.0774 |
| Deepest Hole | -0.6495 |
| GooF | 0.9365 |
| $wR_2$ (all data) | 0.0496 |
| $wR_2$ | 0.0444 |
| $R_1$ (all data) | 0.0446 |
| $R_1$ | 0.0255 |

### 9.5.3 Structure Quality Indicators

| | | | | |
|---|---|---|---|---|
| Reflections: | d min (MoKα) 2Θ=66.3° 0.65 | I/σ(I) 23.4 | Rint m=7.64 7.35% | Full 50.5° 100 |
| Refinement: | Shift -0.001 | Max Peak 1.1 | Min Peak -0.6 | GooF 0.936 |

A yellow lath-shaped crystal with dimensions 0.13×0.03×0.02 $mm^3$ was mounted on a MITIGEN holder in oil. X-ray diffraction data were collected using a Rigaku FRE+ diffractometer with Arc)Sec VHF Varimax confocal mirrors, a UG2 goniometer and HyPix 6000HE detector equipped with an Oxford Cryosystems low-temperature device, operating at $T$ = 100(2) K.

Data were measured using profile data from $\omega$-scans of 0.5 ° per frame for 16.0 s using Mo $K_{\alpha}$ radiation (Rotating Anode, 45.0 kV, 55.0 mA). The total number of runs and images was based on the strategy calculation from the program CrysAlisPro system (CCD 43.121a 64-bit (release 24-04-2024)). The maximum resolution achieved was $\Theta$ = 33.14°.

Cell parameters were retrieved using the CrysAlisPro 1.171.43.119a (Rigaku OD, 2024) software and refined using CrysAlisPro 1.171.43.119a (Rigaku OD, 2024) on 37135 reflections, 38 % of the observed reflections. Data reduction was performed using the CrysAlisPro 1.171.43.119a (Rigaku OD, 2024) software which corrects for Lorentz polarisation. The final completeness is 99.97 % out to 33.14° in $\Theta$.

A multi-scan absorption correction was performed using CrysAlisPro 1.171.43.119a (Rigaku OD, 2024) Empirical absorption correction using spherical harmonics, implemented in SCALE3 ABSPACK scaling algorithm. The absorption coefficient $\mu$ of this material is 0.842 $mm^{-1}$ at this wavelength ($\lambda$ = 0.71073Å) and the minimum and maximum transmissions are 0.834 and 1.000.

The structure was solved in the space group $P2_1/n$ (# 14) by using dual methods using the ShelXT 2018/2 (Sheldrick, 2015) structure solution program and refined by full matrix least squares minimisation on $F^2$ using version of olex2.refine 1.5-alpha (Bourhis et al., 2015). All non-hydrogen atoms were refined anisotropically. Hydrogen atom positions were calculated geometrically and refined using the riding model.

*_olex2_refine_details*: Refinement using NoSpherA2, an implementation of NOn-SPHERical Atom-form-factors in Olex2. Please cite: F. Kleemiss et al. Chem. Sci. DOI 10.1039/D0SC05526C – 2021 NoSpherA2 implementation of HAR makes use of tailor-made aspherical atomic form factors calculated on-the-fly from a Hirshfeld-partitioned electron density (ED) - not from spherical-atom form factors. The ED is calculated from a gaussian basis set single determinant SCF wavefunction - either Hartree-Fock or DFT using selected functionals - for a fragment of the crystal. This fragment can be embedded in an electrostatic crystal field by employing cluster charges or modelled using implicit solvation models, depending on the software used. The following options were used: SOFTWARE: ORCA 5.0 ::: PARTITIONING: NoSpherA2 ::: INT ACCURACY: Normal ::: METHOD: R2SCAN ::: BASIS SET: x2c-TZVP CHARGE: 0 ::: MULTIPLICITY: 1 ::: RELATIVISTIC: DKH2 ::: DATE: 2024-05-02_14-39-30

*_exptl_absorpt_process_details*: CrysAlisPro 1.171.43.119a (Rigaku Oxford Diffraction, 2024) using spherical harmonics, implemented in SCALE3 ABSPACK scaling algorithm.

There is a single formula unit in the asymmetric unit, which is represented by the reported sum formula. In other words: Z is 4 and Z' is 1. The moiety formula is C38 H40 B N4 Sb.

## 9.5.4 Data Plots: Diffraction Data

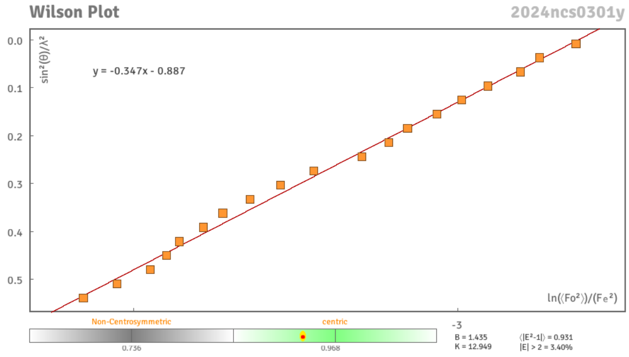


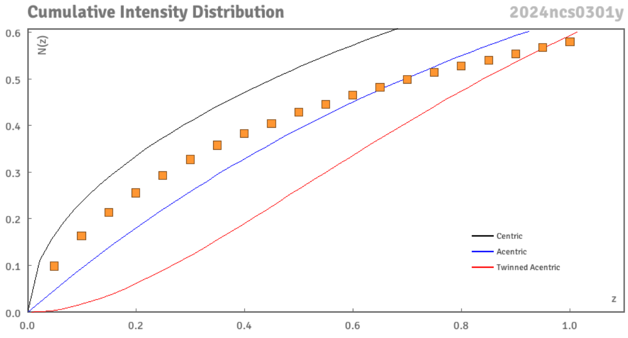


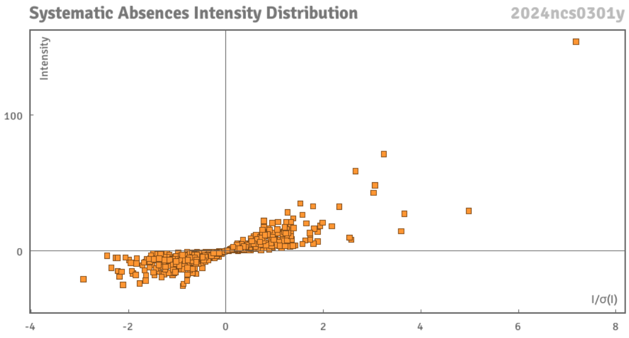


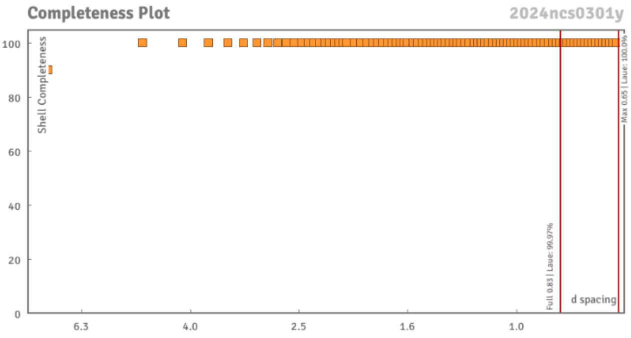


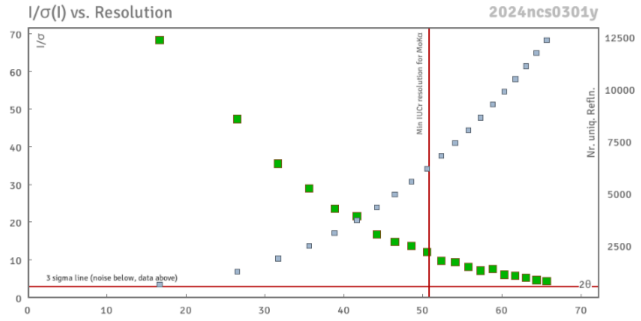


## 9.5.5 Data Plots: Refinement and Data

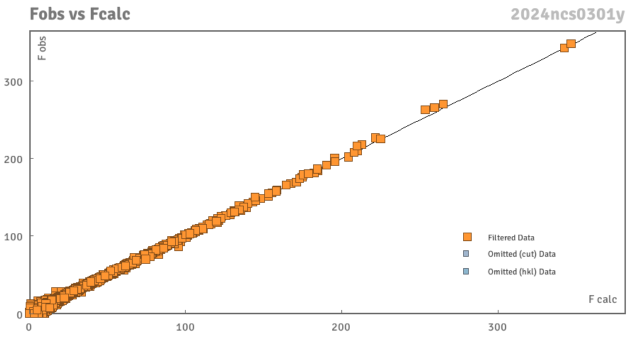


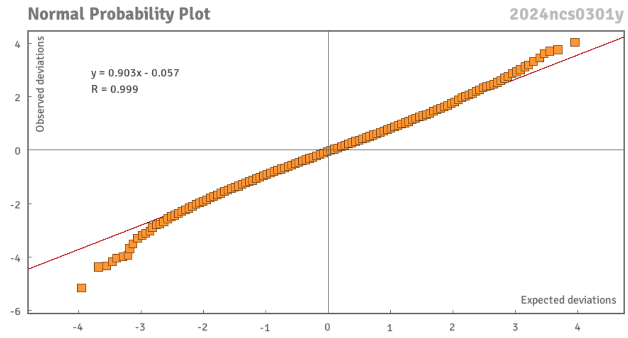

### 9.5.6 Reflection Statistics

| | | | |
|---|---|---|---|
| Total reflections (after filtering) | 99135 | Unique reflections | 12980 |
| Completeness | 1.0 | Mean I/$\sigma$ | 17.52 |
| $hkl_{max}$ collected | (12, 34, 28) | $hkl_{min}$ collected | (-12, -34, -28) |
| $hkl_{max}$ used | (12, 34, 28) | $hkl_{min}$ used | (-12, 0, 0) |
| Lim $d_{max}$ collected | 100.0 | Lim $d_{min}$ collected | 0.36 |
| $d_{max}$ used | 9.69 | $d_{min}$ used | 0.65 |
| Friedel pairs | 21722 | Friedel pairs merged | 1 |
| Inconsistent equivalents | 15 | $R_{int}$ | 0.0735 |
| $R_{sigma}$ | 0.0427 | Intensity transformed | 0 |
| Omitted reflections | 0 | Omitted by user (OMIT hkl) | 0 |
| Multiplicity | (16190, 17565, 9291, 2964, 1009, 330, 106, 33, 5, 1) | Maximum multiplicity | 25 |
| Removed systematic absences | 1027 | Filtered off (Shel/OMIT) | 0 |

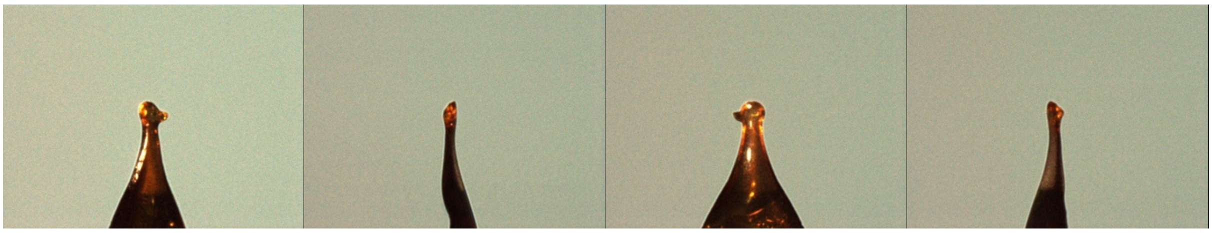

### 9.5.7 Fractional Atomic Coordinates and Equivalent Isotropic Displacement Parameters

Table S31. Fractional Atomic Coordinates (×10^4^) and Equivalent Isotropic Displacement Parameters (Å$^2$×10$^3$) for **4b**. $U_{eq}$ is defined as 1/3 of the trace of the orthogonalised $U_{ij}$.

| Atom | x | y | z | $U_{eq}$ |
|---|---|---|---|---|
| Sb1 | 5389.14(10) | 5776.16(3) | 6958.37(4) | 18.78(2) |
| N1 | 2504.4(11) | 6723.2(4) | 6737.1(5) | 15.06(17) |
| N2 | 1715.0(12) | 5889.6(4) | 6211.7(5) | 18.68(19) |
| N3 | 4826.5(11) | 6965.1(4) | 7760.8(5) | 14.84(17) |
| N4 | 6515.5(12) | 6367.2(4) | 8440.8(5) | 15.63(17) |
| C1 | 3030.7(14) | 6181.1(4) | 6588.8(6) | 16.1(2) |
| C2 | 816.9(15) | 6770.1(5) | 6450.5(7) | 20.6(2) |
| C3 | 320.3(15) | 6252.3(5) | 6122.0(7) | 22.6(2) |
| C4 | 1852.4(15) | 5303.6(4) | 5943.9(6) | 19.4(2) |
| C5 | 1451.3(15) | 4826.6(5) | 6356.3(7) | 22.6(2) |
| C6 | 1757.8(16) | 4261.6(5) | 6114.5(7) | 25.5(3) |
| C7 | 2450.9(17) | 4172.4(5) | 5486.4(7) | 27.3(3) |
| C8 | 2774.5(17) | 4659.2(5) | 5077.5(7) | 27.3(3) |
| C9 | 2480.0(15) | 5231.4(5) | 5295.9(6) | 22.3(2) |
| C10 | 753(2) | 4921.3(6) | 7042.3(8) | 33.2(3) |
| C11 | 2886(3) | 3561.2(6) | 5266.7(9) | 41.1(4) |
| C12 | 2818.2(19) | 5752.2(6) | 4844.8(8) | 29.9(3) |
| C13 | 5593.7(13) | 6436.7(4) | 7774.6(6) | 14.91(19) |
| C14 | 5294.4(15) | 7233.5(5) | 8425.3(6) | 17.9(2) |
| C15 | 6351.8(15) | 6865.8(5) | 8850.1(6) | 18.7(2) |
| C16 | 7436.2(13) | 5842.3(4) | 8669.2(6) | 14.97(19) |
| C17 | 9110.6(14) | 5801.3(4) | 8571.2(6) | 16.65(19) |
| C18 | 9979.2(14) | 5283.9(5) | 8787.4(6) | 18.0(2) |
| C19 | 9215.8(14) | 4814.0(4) | 9083.9(6) | 17.5(2) |
| C20 | 7535.2(15) | 4869.1(5) | 9171.4(6) | 19.2(2) |
| C21 | 6623.6(14) | 5380.1(5) | 8968.0(6) | 17.9(2) |
| C22 | 9945.5(17) | 6298.5(5) | 8240.6(8) | 26.6(3) |
| C23 | 10178.5(17) | 4257.9(5) | 9298.4(7) | 24.6(2) |
| C24 | 4803.5(16) | 5428.2(6) | 9046.4(9) | 28.9(3) |
| C25 | 4864.4(14) | 7447.0(4) | 6493.9(6) | 15.0(2) |
| C26 | 6480.3(14) | 7671.3(5) | 6710.2(6) | 18.5(2) |
| C27 | 7454.3(15) | 7884.0(5) | 6215.3(7) | 20.5(2) |
| C28 | 6856.5(16) | 7880.9(5) | 5478.7(7) | 21.4(2) |
| C29 | 5246.8(16) | 7659.9(5) | 5252.0(7) | 21.7(2) |
| C30 | 4277.4(15) | 7449.3(5) | 5750.8(6) | 18.9(2) |
| C31 | 7899(2) | 8115.3(7) | 4945.0(9) | 35.1(3) |
| C32 | 2526.8(14) | 7763.0(4) | 7309.3(6) | 15.5(2) |
| C33 | 2355.9(15) | 8314.5(5) | 6971.5(7) | 19.7(2) |
| C34 | 1179.1(16) | 8727.3(5) | 7125.7(7) | 23.7(2) |
| C35 | 102.0(15) | 8604.4(5) | 7620.5(7) | 22.1(2) |
| C36 | 284.6(15) | 8062.3(5) | 7972.4(7) | 20.3(2) |
| C37 | 1481.7(14) | 7658.0(4) | 7825.6(6) | 18.0(2) |
| C38 | -1229(2) | 9032.7(6) | 7762.4(10) | 36.5(3) |
| B1 | 3699.0(15) | 7240.1(5) | 7074.6(7) | 14.5(2) |

9.5.8 Anisotropic Displacement Parameters

Table S32. Anisotropic Displacement Parameters (×$10^4$) for **4b**. The anisotropic displacement factor exponent takes the form: $-2\pi^2[h^2a^{*2} \times U_{11}+ ... +2hka^* \times b^* \times U_{12}]$

| Atom | $U_{11}$ | $U_{22}$ | $U_{33}$ | $U_{23}$ | $U_{13}$ | $U_{12}$ |
|---|---|---|---|---|---|---|
| Sb1 | 17.80(3) | 17.48(3) | 19.58(4) | 5.05(3) | -1.81(3) | -4.90(3) |
| N1 | 14.3(4) | 13.2(4) | 17.2(4) | -0.4(3) | 0.8(4) | -0.4(3) |
| N2 | 17.5(4) | 16.0(4) | 20.8(5) | 0.3(3) | -2.5(4) | -3.9(3) |
| N3 | 16.7(4) | 13.6(4) | 13.9(4) | 0.2(3) | 1.3(4) | 0.1(3) |
| N4 | 16.7(4) | 15.6(4) | 14.2(4) | 0.9(3) | 1.0(4) | 1.5(3) |
| C1 | 15.4(5) | 14.4(4) | 17.6(5) | 1.3(4) | -0.0(4) | -1.2(4) |
| C2 | 16.1(5) | 17.3(5) | 26.8(6) | 2.0(4) | -1.4(5) | -1.8(4) |
| H2 | 19(6) | 21(5) | 57(11) | 5(2) | -5(5) | -10(3) |
| C3 | 16.5(5) | 19.4(5) | 29.3(6) | 0.8(4) | -5.0(5) | -4.2(4) |
| H3 | 21(4) | 39(7) | 46(10) | 2(3) | -9(3) | -16(5) |
| C4 | 19.1(5) | 16.8(5) | 20.7(6) | 0.5(4) | -2.0(4) | -3.9(4) |
| C5 | 23.9(6) | 18.0(5) | 24.7(6) | -1.6(4) | -0.0(5) | -2.5(4) |
| C6 | 29.4(6) | 17.6(5) | 26.6(6) | 0.8(5) | -4.5(5) | -3.6(4) |
| H6 | 35(9) | 30(5) | 31(7) | -8(3) | -12(4) | 1(2) |
| C7 | 33.8(6) | 20.5(5) | 23.6(6) | 8.2(5) | -8.2(5) | -7.4(4) |
| C8 | 31.6(7) | 27.0(6) | 21.0(6) | 9.0(5) | -3.0(5) | -6.3(5) |
| H8 | 72(12) | 43(7) | 31(5) | 13(5) | 16(4) | -7(3) |
| C9 | 24.0(6) | 22.5(5) | 18.7(6) | 3.9(4) | -2.3(5) | -4.5(4) |
| C10 | 40.5(9) | 26.4(6) | 35.5(8) | -2.5(6) | 14.3(7) | -1.5(5) |
| H10a | 68(7) | 59(7) | 48(6) | -17(3) | 17(3) | -14(3) |
| H10b | 57(5) | 56(7) | 60(9) | 9(2) | 19(3) | -6(4) |
| H10c | 62(9) | 45(5) | 49(7) | -6(3) | 19(4) | 3(2) |
| C11 | 62.5(11) | 25.9(6) | 28.5(8) | 18.6(7) | -13.4(8) | -9.8(6) |
| H11a | 78(9) | 47(8) | 37(4) | 21(4) | -7(3) | -12(3) |
| H11b | 85(6) | 55(8) | 51(7) | 34(3) | -19(3) | -7(3) |
| H11c | 78(6) | 36(6) | 53(9) | 11(3) | -2(3) | -14(3) |
| C12 | 33.3(7) | 31.1(6) | 24.7(6) | 3.3(6) | 3.0(6) | 2.3(5) |
| H12a | 47(5) | 55(7) | 58(8) | 11(2) | 4(3) | 15(3) |
| H12b | 50(6) | 44(6) | 42(7) | -2(3) | 5(3) | -1(3) |
| H12c | 66(8) | 52(7) | 45(6) | 6(3) | 20(3) | 0(3) |
| C13 | 15.8(5) | 14.5(4) | 13.9(5) | 0.4(4) | 0.6(4) | 0.2(3) |
| C14 | 22.0(5) | 16.6(5) | 14.4(5) | 0.9(4) | 0.7(4) | -1.6(4) |
| H14 | 44(9) | 25(4) | 28(8) | 14(3) | -9(5) | -11(3) |
| C15 | 20.8(5) | 19.4(5) | 14.9(5) | 0.2(4) | -0.2(5) | -1.0(4) |
| H15 | 46(9) | 31(7) | 22(4) | 12(5) | -10(3) | -7(2) |
| C16 | 13.3(4) | 16.8(4) | 14.7(5) | -0.3(4) | 1.9(4) | 2.3(4) |
| C17 | 14.2(5) | 17.4(4) | 19.1(5) | -1.4(4) | 4.6(4) | 2.6(4) |
| C18 | 13.3(5) | 19.4(5) | 21.8(5) | 0.1(4) | 4.3(4) | 1.2(4) |
| H18 | 19(4) | 37(7) | 58(10) | 3(2) | 16(3) | 10(5) |
| C19 | 17.5(5) | 16.4(4) | 18.5(5) | 0.5(4) | 2.3(4) | 0.3(4) |
| C20 | 18.1(5) | 17.4(5) | 22.6(6) | -0.3(4) | 4.6(5) | 4.1(4) |
| H20 | 40(7) | 30(5) | 22(8) | -13(3) | 1(4) | 7(3) |
| C21 | 14.5(5) | 18.9(5) | 20.7(5) | -0.7(4) | 4.6(4) | 4.2(4) |
| C22 | 22.9(6) | 23.9(6) | 34.7(7) | -4.5(5) | 9.6(6) | 7.3(5) |
| H22a | 39(7) | 50(7) | 45(4) | -9(4) | 9(2) | 18(2) |
| H22b | 56(9) | 36(4) | 73(7) | -9(3) | 24(4) | -2(2) |
| H22c | 28(4) | 48(7) | 76(9) | -3(2) | 16(2) | 11(4) |
| C23 | 27.0(6) | 18.5(5) | 28.0(6) | 5.6(5) | 3.0(5) | -0.0(5) |
| H23a | 54(7) | 30(5) | 54(7) | 3(3) | 7(3) | -6(3) |

| | | | | | | |
|---|---|---|---|---|---|---|
| H23b | 36(4) | 37(7) | 76(9) | 10(2) | 14(2) | 5(4) |
| H23c | 74(9) | 44(8) | 44(4) | 24(4) | 13(2) | 11(2) |
| C24 | 17.5(6) | 30.6(6) | 40.5(8) | 2.0(5) | 10.9(6) | 11.4(6) |
| H24a | 41(8) | 49(5) | 78(7) | -2(3) | 29(3) | -3(3) |
| H24b | 35(6) | 73(9) | 57(5) | -1(3) | 5(2) | 17(2) |
| H24c | 31(7) | 43(5) | 54(8) | -6(3) | 15(3) | 11(3) |
| C25 | 14.3(5) | 15.1(4) | 15.7(5) | 0.3(4) | 2.6(4) | 0.9(4) |
| C26 | 16.1(5) | 20.7(5) | 18.6(5) | -2.0(4) | 2.4(4) | 1.2(4) |
| H26 | 28(7) | 41(8) | 22(4) | -18(5) | 1(2) | 4(2) |
| C27 | 17.4(5) | 21.8(5) | 23.3(6) | -0.8(4) | 6.3(5) | 1.4(4) |
| H27 | 27(4) | 59(10) | 32(7) | -16(3) | 5(2) | 4(4) |
| C28 | 23.8(6) | 20.1(5) | 22.1(6) | 0.6(4) | 9.5(5) | 1.1(4) |
| C29 | 25.3(6) | 23.5(5) | 17.0(5) | -0.4(4) | 5.5(5) | 0.7(4) |
| H29 | 34(7) | 63(10) | 22(4) | -12(5) | 3(2) | 5(2) |
| C30 | 19.1(5) | 22.1(5) | 15.1(5) | -1.4(4) | 1.5(4) | 0.5(4) |
| H30 | 28(4) | 54(9) | 22(7) | -14(3) | -4(2) | 7(4) |
| C31 | 37.7(8) | 39.0(7) | 33.6(8) | -6.6(7) | 21.7(7) | 2.4(6) |
| H31a | 69(9) | 68(7) | 45(5) | -6(4) | 28(3) | -2(3) |
| H31b | 81(9) | 60(5) | 73(9) | 2(3) | 34(4) | 17(3) |
| H31c | 49(4) | 114(10) | 60(8) | -11(2) | 20(2) | 9(4) |
| C32 | 17.7(5) | 11.9(4) | 17.4(5) | -0.7(4) | 4.2(4) | 1.5(4) |
| C33 | 21.9(5) | 13.7(4) | 24.8(6) | 0.6(4) | 7.4(5) | 3.6(4) |
| H33 | 44(7) | 26(6) | 47(7) | 8(4) | 28(4) | 14(4) |
| C34 | 26.6(6) | 13.1(5) | 32.3(7) | 2.2(4) | 7.5(5) | 2.7(4) |
| H34 | 47(9) | 23(5) | 62(9) | 11(3) | 23(5) | 19(3) |
| C35 | 22.9(6) | 15.7(5) | 28.2(6) | 2.0(4) | 5.8(5) | -3.7(4) |
| C36 | 20.6(5) | 18.5(5) | 23.0(6) | 0.3(4) | 6.9(5) | -2.9(4) |
| H36 | 54(8) | 46(8) | 61(8) | 11(4) | 41(4) | 13(4) |
| C37 | 19.9(5) | 14.7(4) | 20.6(5) | -0.1(4) | 7.0(4) | 1.4(4) |
| H37 | 39(9) | 25(4) | 56(9) | 12(3) | 30(5) | 18(3) |
| C38 | 35.9(8) | 23.0(6) | 53.9(10) | 10.3(6) | 17.6(8) | -4.4(6) |
| H38a | 73(9) | 55(7) | 88(6) | 11(3) | 25(3) | -20(3) |
| H38b | 90(10) | 57(6) | 88(6) | 30(4) | 29(3) | 13(3) |
| H38c | 64(6) | 60(8) | 125(10) | 8(3) | 50(3) | 3(4) |
| B1 | 15.2(5) | 13.4(5) | 15.3(5) | 0.3(4) | 3.0(5) | 1.0(4) |

### 9.5.9 Bond lengths

Table S33. Bond Lengths in Å for **4b**.

| Atom | Atom | Length/Å |
|---|---|---|
| Sb1 | C1 | 2.1319(11) |
| Sb1 | C13 | 2.1332(10) |
| N1 | C1 | 1.3459(13) |
| N1 | C2 | 1.3895(14) |
| N1 | B1 | 1.5878(14) |
| N2 | C1 | 1.3548(14) |
| N2 | C3 | 1.3846(14) |
| N2 | C4 | 1.4335(13) |
| N3 | C13 | 1.3492(13) |
| N3 | C14 | 1.3877(14) |
| N3 | B1 | 1.5861(15) |
| N4 | C13 | 1.3621(13) |
| N4 | C15 | 1.3868(14) |
| N4 | C16 | 1.4352(13) |
| C2 | C3 | 1.3595(15) |
| C4 | C5 | 1.3989(16) |
| C4 | C9 | 1.3985(17) |
| C5 | C6 | 1.3962(16) |
| C5 | C10 | 1.4999(19) |
| C6 | C7 | 1.3966(19) |
| C7 | C8 | 1.3940(19) |
| C7 | C11 | 1.5056(17) |
| C8 | C9 | 1.3947(16) |
| C9 | C12 | 1.5047(18) |
| C14 | C15 | 1.3609(15) |
| C16 | C17 | 1.3957(15) |
| C16 | C21 | 1.4008(15) |
| C17 | C18 | 1.3965(15) |
| C17 | C22 | 1.4985(16) |
| C18 | C19 | 1.3914(15) |
| C19 | C20 | 1.3978(16) |
| C19 | C23 | 1.5051(15) |
| C20 | C21 | 1.3958(15) |
| C21 | C24 | 1.5037(17) |
| C25 | C26 | 1.4011(15) |
| C25 | C30 | 1.4026(15) |
| C25 | B1 | 1.6169(16) |
| C26 | C27 | 1.3938(16) |
| C27 | C28 | 1.3926(17) |
| C28 | C29 | 1.3969(17) |
| C28 | C31 | 1.5032(18) |
| C29 | C30 | 1.3953(16) |
| C32 | C33 | 1.4015(14) |
| C32 | C37 | 1.4011(16) |
| C32 | B1 | 1.6220(16) |
| C33 | C34 | 1.3970(16) |
| C34 | C35 | 1.3960(18) |
| C35 | C36 | 1.3946(16) |
| C35 | C38 | 1.5039(18) |
| C36 | C37 | 1.3917(16) |

9.5.10 Bond angles

Table S34. Bond Angles in ° for **4b**.

| Atom | Atom | Atom | Angle/° |
|---|---|---|---|
| C13 | Sb1 | C1 | 84.03(4) |
| C2 | N1 | C1 | 108.19(9) |
| B1 | N1 | C1 | 124.57(9) |
| B1 | N1 | C2 | 126.62(9) |
| C3 | N2 | C1 | 109.26(9) |
| C4 | N2 | C1 | 122.71(9) |
| C4 | N2 | C3 | 128.01(9) |
| C14 | N3 | C13 | 108.27(9) |
| B1 | N3 | C13 | 124.75(9) |
| B1 | N3 | C14 | 126.83(8) |
| C15 | N4 | C13 | 109.20(9) |
| C16 | N4 | C13 | 123.71(9) |
| C16 | N4 | C15 | 127.05(9) |
| N1 | C1 | Sb1 | 128.48(8) |
| N2 | C1 | Sb1 | 123.21(7) |
| N2 | C1 | N1 | 107.96(9) |
| C3 | C2 | N1 | 108.18(10) |
| C2 | C3 | N2 | 106.41(10) |
| C5 | C4 | N2 | 119.21(11) |
| C9 | C4 | N2 | 118.19(10) |
| C9 | C4 | C5 | 122.49(10) |
| C6 | C5 | C4 | 117.65(12) |
| C10 | C5 | C4 | 120.97(11) |
| C10 | C5 | C6 | 121.37(11) |
| C7 | C6 | C5 | 121.50(12) |
| C8 | C7 | C6 | 118.95(11) |
| C11 | C7 | C6 | 120.43(13) |
| C11 | C7 | C8 | 120.60(14) |
| C9 | C8 | C7 | 121.49(13) |
| C8 | C9 | C4 | 117.81(11) |
| C12 | C9 | C4 | 121.31(10) |
| C12 | C9 | C8 | 120.88(12) |
| N3 | C13 | Sb1 | 128.90(8) |
| N4 | C13 | Sb1 | 123.28(7) |
| N4 | C13 | N3 | 107.74(9) |
| C15 | C14 | N3 | 108.42(10) |
| C14 | C15 | N4 | 106.35(10) |
| C17 | C16 | N4 | 118.92(9) |
| C21 | C16 | N4 | 119.30(10) |
| C21 | C16 | C17 | 121.76(9) |
| C18 | C17 | C16 | 118.10(10) |
| C22 | C17 | C16 | 120.78(10) |
| C22 | C17 | C18 | 121.12(10) |
| C19 | C18 | C17 | 121.89(10) |
| C20 | C19 | C18 | 118.49(10) |
| C23 | C19 | C18 | 120.56(11) |
| C23 | C19 | C20 | 120.95(10) |
| C21 | C20 | C19 | 121.52(10) |
| C20 | C21 | C16 | 118.24(10) |
| C24 | C21 | C16 | 120.69(10) |
| C24 | C21 | C20 | 121.05(10) |
| C30 | C25 | C26 | 116.29(10) |
| B1 | C25 | C26 | 121.57(10) |
| B1 | C25 | C30 | 122.01(10) |
| C27 | C26 | C25 | 121.96(11) |
| C28 | C27 | C26 | 121.07(11) |
| C29 | C28 | C27 | 117.87(11) |
| C31 | C28 | C27 | 121.12(12) |
| C31 | C28 | C29 | 121.01(12) |
| C30 | C29 | C28 | 120.73(11) |
| C29 | C30 | C25 | 122.08(11) |
| C37 | C32 | C33 | 115.97(10) |
| B1 | C32 | C33 | 123.53(10) |
| B1 | C32 | C37 | 120.19(9) |
| C34 | C33 | C32 | 121.91(11) |
| C35 | C34 | C33 | 121.19(10) |
| C36 | C35 | C34 | 117.46(11) |
| C38 | C35 | C34 | 121.65(11) |
| C38 | C35 | C36 | 120.88(12) |
| C37 | C36 | C35 | 120.96(11) |
| C36 | C37 | C32 | 122.43(10) |
| N3 | B1 | N1 | 105.65(8) |
| C25 | B1 | N1 | 109.08(9) |
| C25 | B1 | N3 | 109.98(9) |
| C32 | B1 | N1 | 107.82(9) |
| C32 | B1 | N3 | 110.69(9) |
| C32 | B1 | C25 | 113.31(8) |

9.5.11 Torsion Angles

Table S35. Torsion Angles in ° for **4b**.

| Atom | Atom | Atom | Atom | Angle/° |
|---|---|---|---|---|
| Sb1 | C1 | N1 | C2 | -172.83(10) |
| Sb1 | C1 | N1 | B1 | 15.67(12) |
| Sb1 | C1 | N2 | C3 | 173.34(10) |
| Sb1 | C1 | N2 | C4 | -8.50(11) |
| Sb1 | C13 | N3 | C14 | 177.68(10) |
| Sb1 | C13 | N3 | B1 | -6.47(12) |
| Sb1 | C13 | N4 | C15 | -178.21(9) |
| Sb1 | C13 | N4 | C16 | -0.56(11) |
| N1 | C1 | N2 | C3 | -0.36(11) |
| N1 | C1 | N2 | C4 | 177.80(9) |
| N1 | C2 | C3 | N2 | 0.12(11) |
| N1 | B1 | N3 | C13 | 45.43(11) |
| N1 | B1 | N3 | C14 | -139.49(8) |
| N1 | B1 | C25 | C26 | -149.59(8) |
| N1 | B1 | C25 | C30 | 34.78(10) |
| N1 | B1 | C32 | C33 | -112.24(9) |
| N1 | B1 | C32 | C37 | 61.12(10) |
| N2 | C4 | C5 | C6 | -173.75(11) |
| N2 | C4 | C5 | C10 | 4.91(14) |
| N2 | C4 | C9 | C8 | 173.43(11) |
| N2 | C4 | C9 | C12 | -6.95(13) |
| N3 | C13 | N4 | C15 | -1.15(10) |
| N3 | C13 | N4 | C16 | 176.51(8) |
| N3 | C14 | C15 | N4 | -0.49(10) |
| N3 | B1 | C25 | C26 | -34.16(10) |
| N3 | B1 | C25 | C30 | 150.20(8) |
| N3 | B1 | C32 | C33 | 132.66(9) |
| N3 | B1 | C32 | C37 | -53.99(10) |
| N4 | C16 | C17 | C18 | -179.10(10) |
| N4 | C16 | C17 | C22 | 0.67(13) |
| N4 | C16 | C21 | C20 | 178.60(10) |
| N4 | C16 | C21 | C24 | 0.36(13) |
| C4 | C5 | C6 | C7 | 0.44(14) |
| C4 | C9 | C8 | C7 | 0.28(14) |
| C5 | C6 | C7 | C8 | -2.78(15) |
| C5 | C6 | C7 | C11 | 175.50(13) |
| C6 | C7 | C8 | C9 | 2.41(15) |
| C7 | C8 | C9 | C12 | -179.34(12) |
| C16 | C17 | C18 | C19 | 0.90(12) |
| C16 | C21 | C20 | C19 | 0.12(13) |
| C17 | C18 | C19 | C20 | -0.63(13) |
| C17 | C18 | C19 | C23 | 178.83(11) |
| C18 | C19 | C20 | C21 | 0.10(13) |
| C19 | C20 | C21 | C24 | 178.35(12) |
| C25 | C26 | C27 | C28 | -0.15(13) |
| C25 | C30 | C29 | C28 | -0.22(13) |
| C25 | B1 | C32 | C33 | 8.58(12) |
| C25 | B1 | C32 | C37 | -178.06(9) |
| C26 | C27 | C28 | C29 | 0.26(13) |
| C26 | C27 | C28 | C31 | 179.42(11) |
| C27 | C28 | C29 | C30 | -0.07(12) |
| C32 | C33 | C34 | C35 | -0.74(14) |

| | | | | |
|---|---|---|---|---|
| C32 | C37 | C36 | C35 | -1.78(14) |
| C33 | C34 | C35 | C36 | 2.12(14) |
| C33 | C34 | C35 | C38 | -176.79(13) |
| C34 | C35 | C36 | C37 | -0.90(14) |

## 9.5.12 Hydrogen Fractional Atomic Coordinates and Equivalent Isotropic Displacement Parameters

Table S36. Hydrogen Fractional Atomic Coordinates (×$10^4$) and Equivalent Isotropic Displacement Parameters (Å$^2$×$10^3$) for **4b**. $U_{eq}$ is defined as 1/3 of the trace of the orthogonalised $U_{ij}$.

| Atom | x | y | z | $U_{eq}$ |
|---|---|---|---|---|
| H2 | 130(17) | 7170(5) | 6498(8) | 34(4) |
| H3 | -861(17) | 6105(6) | 5852(8) | 37(4) |
| H6 | 1485(18) | 3889(6) | 6436(8) | 34(4) |
| H8 | 3260(20) | 4597(6) | 4583(8) | 48(5) |
| H10a | 1500(20) | 5193(7) | 7401(9) | 57(4) |
| H10b | -380(20) | 5146(7) | 6977(9) | 57(4) |
| H10c | 580(20) | 4500(7) | 7316(9) | 51(4) |
| H11a | 3250(20) | 3543(7) | 4729(9) | 56(4) |
| H11b | 3930(20) | 3384(7) | 5635(10) | 67(4) |
| H11c | 1800(20) | 3285(7) | 5277(9) | 57(4) |
| H12a | 1730(20) | 5980(7) | 4629(9) | 54(4) |
| H12b | 3700(20) | 6066(7) | 5139(9) | 45(4) |
| H12c | 3460(20) | 5619(7) | 4410(9) | 53(4) |
| H14 | 4797(19) | 7664(6) | 8529(8) | 34(4) |
| H15 | 6955(19) | 6906(6) | 9387(7) | 35(4) |
| H18 | 11236(17) | 5249(6) | 8688(8) | 37(4) |
| H20 | 6924(19) | 4491(6) | 9394(7) | 31(3) |
| H22a | 9360(20) | 6379(7) | 7704(9) | 44(3) |
| H22b | 9850(20) | 6711(7) | 8513(10) | 54(4) |
| H22c | 11264(19) | 6193(7) | 8249(9) | 50(4) |
| H23a | 9700(20) | 3904(6) | 8960(9) | 46(3) |
| H23b | 11480(20) | 4293(6) | 9212(10) | 49(4) |
| H23c | 10180(20) | 4145(6) | 9854(9) | 53(4) |
| H24a | 4530(20) | 5792(7) | 9352(10) | 54(4) |
| H24b | 4010(20) | 5471(8) | 8533(10) | 55(4) |
| H24c | 4356(19) | 5043(6) | 9287(9) | 42(3) |
| H26 | 6984(18) | 7682(6) | 7284(7) | 30(3) |
| H27 | 8735(18) | 8053(7) | 6400(8) | 39(4) |
| H29 | 4747(19) | 7650(7) | 4682(8) | 40(4) |
| H30 | 3037(18) | 7275(6) | 5551(7) | 36(4) |
| H31a | 7820(20) | 7878(7) | 4452(9) | 59(4) |
| H31b | 7460(30) | 8558(8) | 4752(10) | 69(4) |
| H31c | 9160(20) | 8142(9) | 5145(10) | 73(4) |
| H33 | 3113(19) | 8427(5) | 6581(8) | 37(4) |
| H34 | 1010(20) | 9143(5) | 6847(9) | 42(4) |
| H36 | -540(20) | 7966(6) | 8365(9) | 50(5) |
| H37 | 1543(18) | 7228(6) | 8102(8) | 38(4) |
| H38a | -800(30) | 9329(8) | 8193(12) | 71(4) |
| H38b | -1530(30) | 9353(8) | 7360(12) | 77(4) |
| H38c | -2220(20) | 8831(8) | 7969(11) | 79(5) |

## 9.6 Single crystal structure analysis of 5a

CCDC Deposition number 2423154

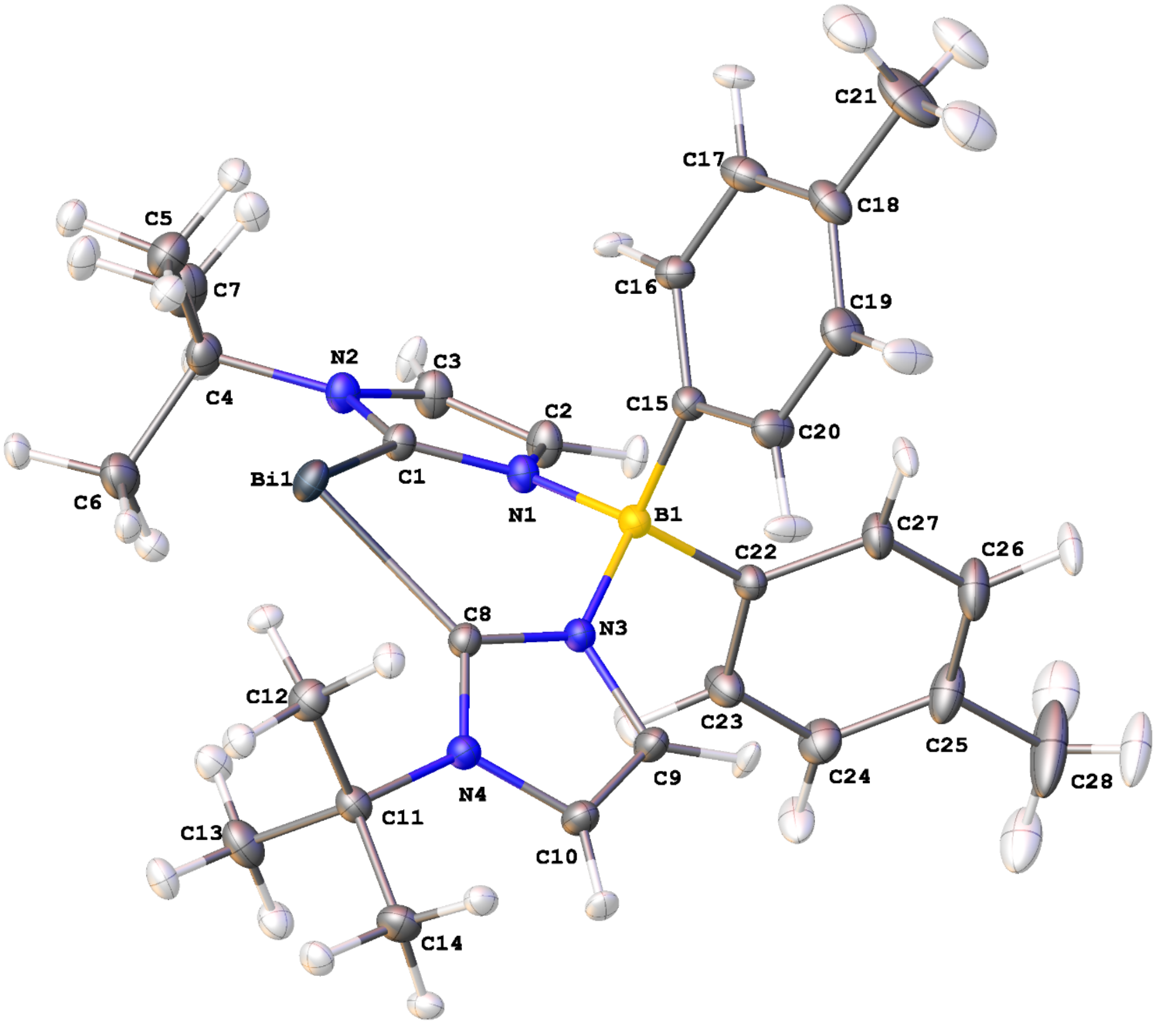


Figure S31. Olex-2 representation of the molecular structure of complex **5a**

### 9.6.1 Experimental.

Single yellow plate-shaped-shaped crystals of **5a** were The crystal was chosen from the sample as supplied.. A suitable crystal 0.12×0.10×0.05 mm$^3$ was selected and mounted on a MITIGEN holder in oil on a Rigaku FRE+ diffractometer with Arc)Sec VHF Varimax confocal mirrors, a UG2 goniometer and HyPix 6000HE detector. The crystal was kept at a steady $T$ = 100(2) K during data collection. The structure was solved with the ShelXT (Sheldrick, 2015) structure solution program using the using dual methods solution method and by using Olex2 1.5-alpha (Dolomanov et al., 2009) as the graphical interface. The model was refined with version of olex2.refine 1.5-alpha (Bourhis et al., 2015) using full matrix least squares minimisation on $\boldsymbol{F}^2$ minimisation.

9.6.2 Crystal Data.

$C_{28}H_{36}BBiN_4$, $M_r$ = 648.429, monoclinic, $P2_1/c$ (No. 14), a = 10.3422(2) Å, b = 12.2864(2) Å, c = 22.3222(4) Å, $\beta$ = 102.815(2)°, $\alpha = \gamma$ = 90°, $V$ = 2765.79(9) Å$^3$, $T$ = 100(2) K, $Z$ = 4, $Z'$ = 1, $\mu$(Mo $K_\alpha$) = 6.396 mm$^{-1}$, 120888 reflections measured, 22888 unique ($R_{int}$ = 0.0648) which were used in all calculations. The final $wR_2$ was 0.0419 (all data) and $R_1$ was 0.0340 (I≥2 $\sigma$(I)).

| Compound | **5a** |
|---|---|
| | |
| Formula | $C_{28}H_{36}BBiN_4$ |
| $D_{calc.}$/ g cm$^{-3}$ | 1.557 |
| $\mu$/mm$^{-1}$ | 6.396 |
| Formula Weight | 648.429 |
| Colour | yellow |
| Shape | plate-shaped |
| Size/mm$^3$ | 0.12×0.10×0.05 |
| $T$/K | 100(2) |
| Crystal System | monoclinic |
| Space Group | $P2_1/c$ |
| $a$/Å | 10.3422(2) |
| $b$/Å | 12.2864(2) |
| $c$/Å | 22.3222(4) |
| $\alpha$/° | 90 |
| $\beta$/° | 102.815(2) |
| $\gamma$/° | 90 |
| V/Å$^3$ | 2765.79(9) |
| $Z$ | 4 |
| $Z'$ | 1 |
| Wavelength/Å | 0.71073 |
| Radiation type | Mo $K_\alpha$ |
| $\Theta_{min}$/° | 1.90 |
| $\Theta_{max}$/° | 45.16 |
| Measured Refl's. | 120888 |
| Indep't Refl's | 22888 |
| Refl's I≥2 $\sigma$(I) | 16048 |
| $R_{int}$ | 0.0648 |
| Parameters | 656 |
| Restraints | 621 |
| Largest Peak | 1.9263 |
| Deepest Hole | -1.6143 |
| GooF | 0.9908 |
| $wR_2$ (all data) | 0.0419 |
| $wR_2$ | 0.0361 |
| $R_1$ (all data) | 0.0692 |
| $R_1$ | 0.0340 |

### 9.6.3 Structure Quality Indicators

| | | | | |
|---|---|---|---|---|
| Reflections: | d min (MoKα) 2Θ=90.3° 0.50 | I/σ(I) 18.8 | Rint m=5.38 6.48% | Full 50.5° 100 |
| Refinement: | Shift -0.001 | Max Peak 1.9 | Min Peak -1.6 | GooF 0.991 |

A yellow plate-shaped-shaped crystal with dimensions 0.12×0.10×0.05 mm$^3$ was mounted on a MITIGEN holder in oil. X-ray diffraction data were collected using a Rigaku FRE+ diffractometer with Arc)Sec VHF Varimax confocal mirrors, a UG2 goniometer and HyPix 6000HE detector equipped with an Oxford Cryosystems low-temperature device, operating at $T$ = 100(2) K.

Data were measured using profile data from $\omega$-scans of 0.5 ° per frame for 1.5 s using Mo $K_\alpha$ radiation (Rotating Anode, 45.0 kV, 55.0 mA). The total number of runs and images was based on the strategy calculation from the program CrysAlisPro system (CCD 43.127a 64-bit (release 16-06-2024)).The maximum resolution achieved was $\Theta$ = 45.16°.

Cell parameters were retrieved using the CrysAlisPro 1.171.43.130a (Rigaku OD, 2024) software and refined using CrysAlisPro 1.171.43.130a (Rigaku OD, 2024) on 29932 reflections, 25 % of the observed reflections. Data reduction was performed using the CrysAlisPro 1.171.43.130a (Rigaku OD, 2024) software which corrects for Lorentz polarisation. The final completeness is 99.96 % out to 45.16° in $\Theta$.

A gaussian absorption correction was performed using CrysAlisPro 1.171.43.130a (Rigaku Oxford Diffraction, 2024) Numerical absorption correction based on gaussian integration over a multifaceted crystal model Empirical absorption correction using spherical harmonics, implemented in SCALE3 ABSPACK scaling algorithm.. The absorption coefficient $\mu$ of this material is 6.396 mm$^{-1}$ at this wavelength ($\lambda$ = 0.71073Å) and the minimum and maximum transmissions are 0.643 and 0.991.

The structure was solved in the space group $P2_1/c$ (# 14) by using dual methods using the ShelXT (Sheldrick, 2015) structure solution program and refined by full matrix least squares minimisation on $F^2$ using version of olex2.refine 1.5-alpha (Bourhis et al., 2015). All non-hydrogen atoms were refined anisotropically. Hydrogen atom positions were calculated geometrically and refined using the riding model.

*_refine_special_details*: NoSpherA2 refinement. Thermal restraints (RIGU) applied to all atoms. Bi refined anisotropically with anharmonic component.

*_olex2_refine_details*: Refinement using NoSpherA2, an implementation ofNOn-SPHERical Atom-form-factors in Olex2.Please cite:F. Kleemiss et al. Chem. Sci. DOI 10.1039/D0SC05526C - 2021NoSpherA2 implementation of HAR makes use oftailor-made aspherical atomic form factors calculatedon-the-fly from a Hirshfeld-partitioned electron density (ED) - not fromspherical-atom form factors.The ED is calculated from a gaussian basis set single determinant SCFwavefunction - either Hartree-Fock or DFT using selected funtionals - for a fragment of the crystal.This fragment can be

embedded in an electrostatic crystal field by employing cluster chargesor modelled using implicit solvation models, depending on the software used.The following options were used: SOFTWARE: ORCA 5.0 PARTITIONING: NoSpherA2 INT ACCURACY: Normal METHOD: R2SCAN BASIS SET: x2c-TZVP CHARGE: 0 MULTIPLICITY: 1 RELATIVISTIC: DKH2 DATE: 2024-07-12_13-25-47

*_exptl_absorpt_process_details*: CrysAlisPro 1.171.43.130a (Rigaku Oxford Diffraction, 2024)Numerical absorption correction based on gaussian integration overa multifaceted crystal modelEmpirical absorption correction using spherical harmonics,implemented in SCALE3 ABSPACK scaling algorithm.

There is a single formula unit in the asymmetric unit, which is represented by the reported sum formula. In other words: Z is 4 and Z' is 1. The moiety formula is C28 H36 B Bi N4.

## 9.6.4 Data Plots: Diffraction Data

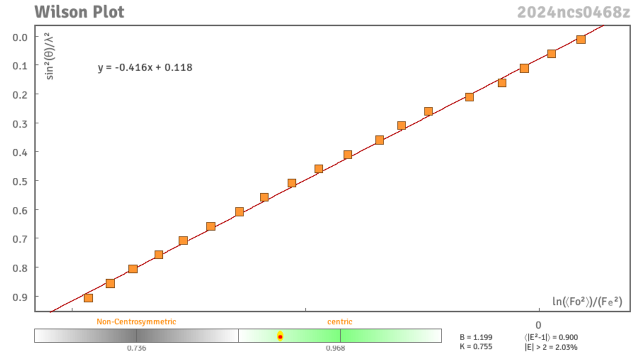


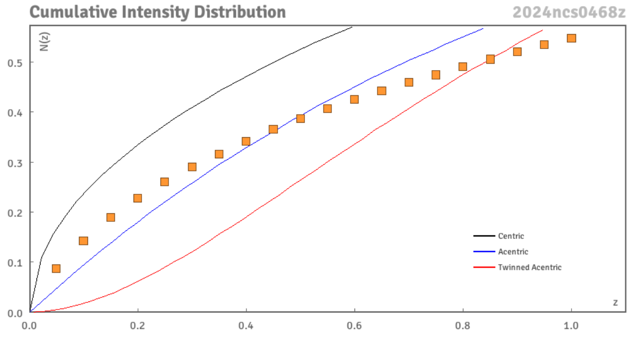


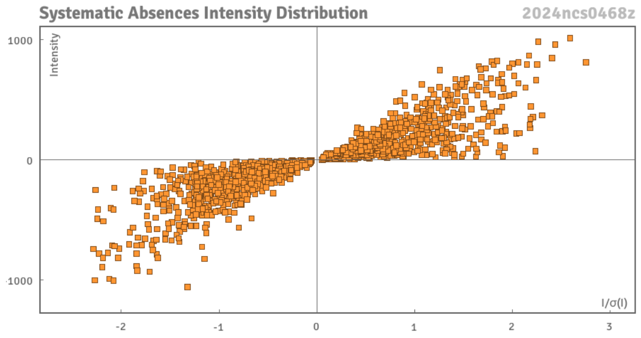


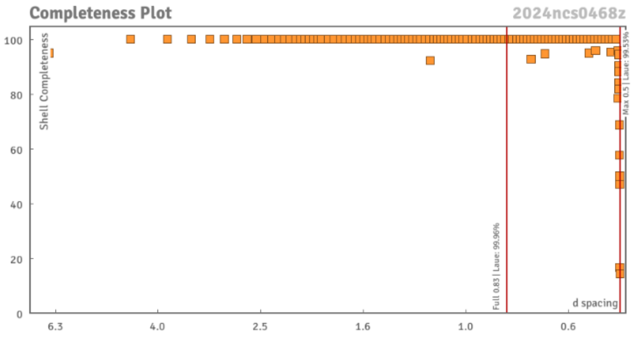


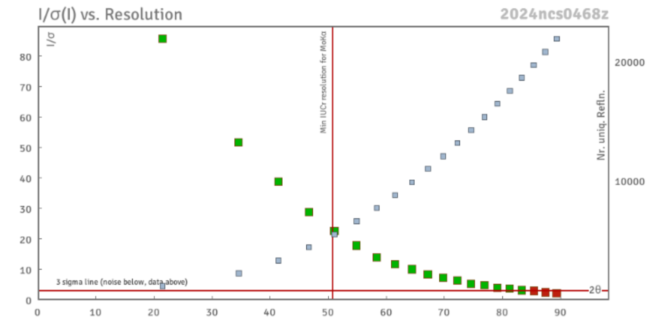


## 9.6.5 Data Plots: Refinement and Data

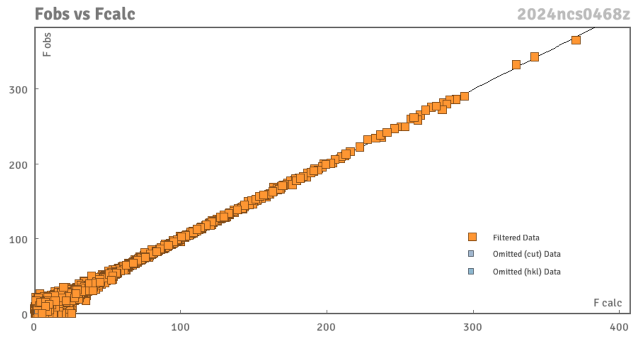


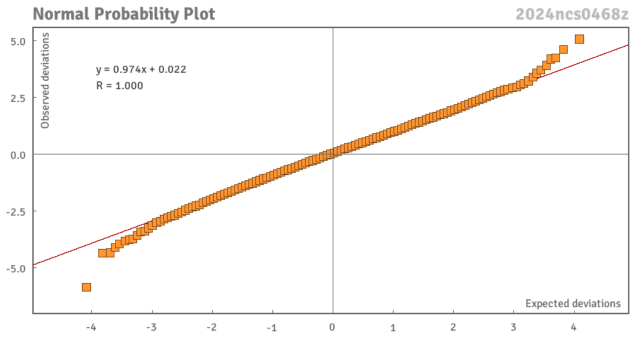

### 9.6.6 Reflection Statistics

| | | | |
|---|---|---|---|
| Total reflections (after filtering) | 123188 | Unique reflections | 22888 |
| Completeness | 0.995 | Mean $I/\sigma$ | 16.51 |
| $hkl_{max}$ collected | (20, 24, 44) | $hkl_{min}$ collected | (-20, -24, -44) |
| $hkl_{max}$ used | (20, 24, 44) | $hkl_{min}$ used | (-20, 0, 0) |
| Lim $d_{max}$ collected | 100.0 | Lim $d_{min}$ collected | 0.36 |
| $d_{max}$ used | 10.7 | $d_{min}$ used | 0.5 |
| Friedel pairs | 27435 | Friedel pairs merged | 1 |
| Inconsistent equivalents | 0 | $R_{int}$ | 0.0648 |
| $R_{sigma}$ | 0.0532 | Intensity transformed | 0 |
| Omitted reflections | 0 | Omitted by user (OMIT hkl) | 0 |
| Multiplicity | (36969, 22566, 8476, 2593, 749, 199, 44, 5) | Maximum multiplicity | 15 |
| Removed systematic absences | 2300 | Filtered off (Shel/OMIT) | 0 |

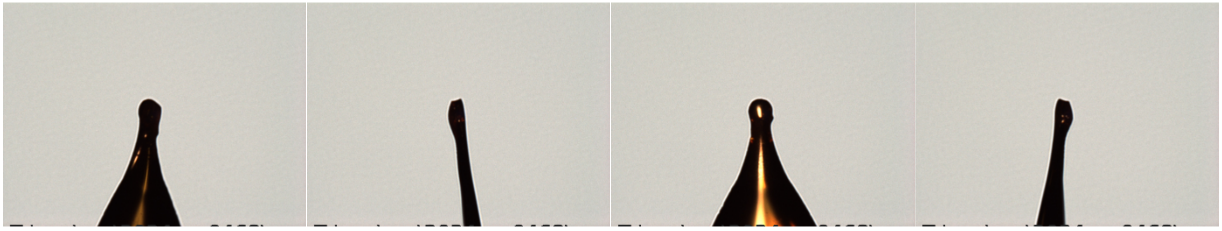

### 9.6.7 Fractional Atomic Coordinates and Equivalent Isotropic Displacement Parameters

Table S37. Fractional Atomic Coordinates (×10[4]) and Equivalent Isotropic Displacement Parameters (Å[2]×10[3]) for **5a**. $U_{eq}$ is defined as 1/3 of the trace of the orthogonalised $U_{ij}$.

| Atom | x | y | z | $U_{eq}$ |
|---|---|---|---|---|
| Bi1 | 7182.27(8) | 6213.85(7) | 7566.71(4) | 16.90(3) |
| N1 | 5763.9(9) | 4834.4(8) | 6377.1(4) | 14.32(17) |
| N2 | 4187.4(9) | 5537.3(8) | 6766.3(5) | 15.82(18) |
| N3 | 8193.9(9) | 4283.9(8) | 6805.9(4) | 13.72(17) |
| N4 | 9493.0(9) | 4282.9(8) | 7724.3(4) | 14.13(17) |
| C1 | 5527.4(11) | 5480.7(9) | 6829.3(5) | 14.18(19) |
| C2 | 4561.4(12) | 4482.8(11) | 6026.3(6) | 19.0(2) |
| C3 | 3580.7(12) | 4909.8(11) | 6266.2(6) | 19.4(2) |
| C4 | 3471.6(12) | 6093.7(10) | 7190.5(6) | 18.4(2) |
| C5 | 3913.9(14) | 7286.6(11) | 7270.0(7) | 23.7(3) |
| C6 | 3754.4(14) | 5479.0(12) | 7801.1(6) | 23.9(3) |
| C7 | 1979.3(13) | 6064.8(13) | 6913.5(7) | 26.1(3) |
| C8 | 8432.5(11) | 4785.2(9) | 7359.1(5) | 13.83(19) |
| C9 | 9126.9(12) | 3468.2(10) | 6826.0(5) | 17.4(2) |
| C10 | 9934.9(12) | 3459.9(10) | 7393.5(6) | 17.6(2) |
| C11 | 10059.4(11) | 4503.7(10) | 8387.9(5) | 15.8(2) |
| C12 | 10366.1(13) | 5716.9(10) | 8475.3(6) | 18.8(2) |
| C13 | 9057.8(14) | 4143.2(12) | 8760.6(6) | 22.7(3) |
| C14 | 11347.3(13) | 3860.5(11) | 8601.2(6) | 21.0(2) |
| C15 | 7630.1(11) | 5629.1(9) | 5863.3(5) | 14.09(19) |
| C16 | 6766.6(12) | 6440.2(9) | 5568.8(5) | 16.4(2) |
| C17 | 7154.8(13) | 7200.2(10) | 5178.1(6) | 20.5(2) |
| C18 | 8428.6(14) | 7184.0(11) | 5065.1(6) | 22.2(2) |
| C19 | 9313.8(13) | 6402.9(11) | 5370.8(6) | 23.7(3) |
| C20 | 8918.9(12) | 5649.9(11) | 5760.7(6) | 19.4(2) |
| C21 | 8837(2) | 7971.9(16) | 4629.7(8) | 40.1(4) |
| C22 | 6932.3(11) | 3530.7(9) | 5753.4(5) | 14.89(19) |
| C23 | 6507.0(13) | 2538.1(10) | 5955.4(6) | 20.2(2) |
| C24 | 6316.9(13) | 1609.6(11) | 5588.7(6) | 21.9(2) |
| C25 | 6529.5(13) | 1635.4(12) | 4993.7(7) | 25.3(3) |
| C26 | 6946.8(14) | 2617.3(12) | 4783.8(7) | 26.5(3) |
| C27 | 7148.5(12) | 3539.1(10) | 5157.7(6) | 19.1(2) |
| C28 | 6285(2) | 651.0(17) | 4588.5(10) | 55.1(6) |
| B1 | 7134.7(13) | 4595.2(10) | 6200.9(6) | 13.6(2) |

## 9.6.8 Anisotropic Displacement Parameters

Table S38. Anisotropic Displacement Parameters (×$10^4$) for **5a**. The anisotropic displacement factor exponent takes the form: $-2\pi^2[h^2a^{*2} \times U_{11}+ ... +2hka^* \times b^* \times U_{12}]$

| Atom | $U_{11}$ | $U_{22}$ | $U_{33}$ | $U_{23}$ | $U_{13}$ | $U_{12}$ |
|---|---|---|---|---|---|---|
| Bi1 | 14.91(5) | 16.51(5) | 18.93(5) | 0.36(4) | 3.02(3) | -6.17(4) |
| N1 | 14.7(4) | 16.1(4) | 12.3(4) | -1.2(3) | 3.3(3) | -1.7(3) |
| N2 | 13.5(4) | 19.4(5) | 14.9(4) | -0.8(3) | 4.0(3) | -1.3(3) |
| N3 | 15.8(4) | 13.9(4) | 11.3(4) | 1.6(3) | 2.7(3) | -0.5(3) |
| N4 | 15.1(4) | 14.2(4) | 12.8(4) | 0.8(3) | 2.6(3) | -0.1(3) |
| C1 | 13.2(5) | 15.3(5) | 14.4(5) | -0.5(4) | 3.8(4) | -1.4(4) |
| C2 | 16.5(5) | 24.3(6) | 15.8(5) | -2.0(4) | 2.5(4) | -6.1(4) |
| H2 | 29(8) | 41(9) | 28(6) | -3(4) | 6(3) | -19(4) |
| C3 | 13.6(5) | 25.8(6) | 18.6(5) | -2.5(4) | 3.0(4) | -4.4(4) |
| H3 | 13(4) | 48(11) | 40(9) | -2(2) | 2(2) | -23(6) |
| C4 | 15.2(5) | 22.8(6) | 18.4(5) | 1.7(4) | 6.3(4) | -0.7(4) |
| C5 | 23.7(6) | 22.6(6) | 25.8(7) | 2.7(5) | 7.7(5) | -2.9(5) |
| H5a | 32(4) | 35(8) | 38(7) | 1(2) | 7(2) | -4(3) |
| H5b | 53(8) | 30(7) | 33(4) | 4(4) | 14(2) | 0(2) |
| H5c | 35(6) | 38(7) | 35(7) | 6(3) | 12(3) | -6(3) |
| C6 | 23.8(6) | 29.3(7) | 21.0(6) | 2.1(5) | 10.2(5) | 2.7(5) |
| H6a | 38(7) | 34(4) | 31(7) | 1(2) | 9(4) | 4(2) |
| H6b | 26(4) | 37(8) | 24(6) | 5(2) | 9.0(19) | -1(4) |
| H6c | 33(7) | 45(8) | 26(6) | 7(3) | 15(3) | 3(3) |
| C7 | 15.6(5) | 35.7(8) | 27.9(7) | 2.1(5) | 6.6(5) | -3.1(6) |
| H7a | 26(7) | 52(8) | 32(4) | 1(4) | 5(2) | 1(2) |
| H7b | 32(7) | 44(4) | 49(8) | -4(2) | 14(4) | -6(2) |
| H7c | 30(7) | 60(8) | 43(7) | 9(3) | 13(3) | -8(3) |
| C8 | 14.6(5) | 13.7(5) | 13.5(5) | 0.2(4) | 3.6(4) | -0.9(4) |
| C9 | 21.3(5) | 15.7(5) | 14.6(5) | 5.2(4) | 2.7(4) | -1.6(4) |
| H9 | 38(9) | 42(9) | 26(5) | 20(5) | -2(4) | -15(3) |
| C10 | 19.3(5) | 16.6(5) | 16.2(5) | 4.9(4) | 2.4(4) | -0.3(4) |
| H10 | 30(6) | 39(8) | 31(8) | 18(3) | 4(3) | 3(4) |
| C11 | 16.4(5) | 17.4(5) | 13.5(5) | -1.1(4) | 3.1(4) | 0.9(4) |
| C12 | 18.5(6) | 18.5(5) | 18.6(6) | -2.1(4) | 2.1(4) | -3.1(4) |
| H12a | 34(6) | 29(7) | 31(6) | -6(3) | 11(3) | -3(3) |
| H12b | 25(4) | 21(6) | 36(8) | -1(2) | 6(2) | 1(3) |
| H12c | 32(7) | 31(7) | 28(4) | -2(3) | 2(2) | -5(2) |
| C13 | 22.3(6) | 30.0(7) | 16.6(6) | -4.5(5) | 5.6(5) | 2.9(5) |
| H13a | 30(4) | 48(7) | 38(8) | 0(2) | 9(2) | 7(4) |
| H13b | 43(8) | 37(4) | 36(8) | -8(2) | 13(4) | 2(2) |
| H13c | 37(7) | 51(8) | 24(4) | -6(4) | 7(2) | 0(2) |
| C14 | 21.4(6) | 22.1(6) | 17.9(5) | 3.4(5) | 0.8(4) | 3.5(5) |
| H14a | 38(8) | 27(4) | 28(8) | 2(2) | 0(4) | 5(2) |
| H14b | 30(6) | 33(8) | 33(6) | 6(3) | 9(3) | 7(3) |
| H14c | 33(7) | 39(8) | 24(4) | 1(4) | 0(2) | 1(2) |
| C15 | 15.0(5) | 13.9(5) | 13.7(5) | 0.1(4) | 4.0(4) | 0.5(4) |
| C16 | 17.3(5) | 14.3(5) | 16.9(5) | 0.9(4) | 2.5(4) | 1.2(4) |
| H16 | 23(4) | 24(8) | 49(10) | 7(3) | 12(2) | 13(5) |
| C17 | 25.1(6) | 16.6(5) | 17.2(5) | -2.7(4) | -0.5(4) | 3.3(4) |
| H17 | 36(6) | 26(7) | 36(9) | 3(3) | 2(3) | 13(4) |
| C18 | 28.9(7) | 21.8(6) | 15.7(5) | -7.5(5) | 4.6(5) | 2.2(4) |
| C19 | 22.9(6) | 26.2(7) | 24.4(6) | -4.4(5) | 10.6(5) | 2.5(5) |
| H19 | 34(4) | 60(11) | 78(12) | 9(3) | 32(3) | 37(7) |

| | | | | | | |
|---|---|---|---|---|---|---|
| C20 | 16.7(5) | 20.4(6) | 22.2(6) | 0.1(4) | 6.4(4) | 3.7(4) |
| H20 | 21(6) | 37(7) | 67(11) | 7(3) | 15(4) | 25(5) |
| C21 | 51.3(11) | 42.3(10) | 27.0(8) | -18.2(8) | 9.5(7) | 12.9(7) |
| H21a | 104(10) | 54(5) | 64(9) | -20(3) | 23(4) | 11(2) |
| H21b | 68(4) | 78(10) | 73(10) | -17(3) | 27(2) | 24(4) |
| H21c | 84(7) | 83(10) | 43(5) | -29(4) | 5(2) | 23(3) |
| C22 | 18.0(5) | 14.3(5) | 12.2(4) | 0.1(4) | 3.0(4) | -0.9(3) |
| C23 | 29.2(6) | 16.1(5) | 15.0(5) | -4.2(5) | 4.3(5) | -1.5(4) |
| H23 | 83(13) | 30(8) | 23(4) | -15(5) | 24(3) | -4(3) |
| C24 | 25.4(6) | 17.0(6) | 22.9(6) | -4.0(5) | 4.9(5) | -4.7(4) |
| H24 | 92(14) | 26(5) | 53(9) | -20(3) | 39(5) | -9(3) |
| C25 | 24.8(6) | 25.6(6) | 29.0(7) | -9.4(5) | 13.7(5) | -15.4(5) |
| C26 | 30.3(7) | 30.8(7) | 23.0(6) | -11.1(5) | 15.8(5) | -12.6(5) |
| H26 | 85(13) | 44(9) | 33(4) | -26(5) | 36(3) | -19(3) |
| C27 | 23.3(6) | 20.9(6) | 15.3(5) | -3.2(4) | 8.7(4) | -4.0(4) |
| H27 | 61(11) | 27(5) | 22(7) | -16(3) | 20(5) | -7(3) |
| C28 | 72.2(14) | 47.4(11) | 62.5(13) | -37.5(10) | 51.0(11) | -41.4(9) |
| H28a | 108(10) | 53(5) | 95(8) | -36(3) | 62(4) | -33(3) |
| H28b | 95(5) | 65(10) | 92(8) | -43(3) | 42(3) | -39(4) |
| H28c | 110(7) | 69(9) | 97(7) | -46(4) | 78(3) | -54(4) |
| B1 | 16.0(5) | 13.0(5) | 11.7(5) | 0.5(4) | 3.1(4) | 0.0(4) |

9.6.9 Bond lengths

Table S39. Bond Lengths in Å for **5a**.

| Atom | Atom | Length/Å |
|---|---|---|
| Bi1 | C1 | 2.2805(11) |
| Bi1 | C8 | 2.2878(11) |
| N1 | C1 | 1.3485(14) |
| N1 | C2 | 1.3837(15) |
| N1 | B1 | 1.5807(15) |
| N2 | C1 | 1.3633(14) |
| N2 | C3 | 1.3870(15) |
| N2 | C4 | 1.4910(14) |
| N3 | C8 | 1.3526(14) |
| N3 | C9 | 1.3850(15) |
| N3 | B1 | 1.5856(16) |
| N4 | C8 | 1.3608(14) |
| N4 | C10 | 1.3878(15) |
| N4 | C11 | 1.4921(15) |
| C2 | C3 | 1.3534(17) |
| C4 | C5 | 1.5339(18) |
| C4 | C6 | 1.5286(18) |
| C4 | C7 | 1.5304(18) |
| C9 | C10 | 1.3541(17) |
| C11 | C12 | 1.5274(17) |
| C11 | C13 | 1.5309(16) |
| C11 | C14 | 1.5310(17) |
| C15 | C16 | 1.4008(16) |
| C15 | C20 | 1.4018(16) |
| C15 | B1 | 1.6172(17) |
| C16 | C17 | 1.3961(17) |
| C17 | C18 | 1.3953(18) |
| C18 | C19 | 1.3956(19) |
| C18 | C21 | 1.4976(19) |
| C19 | C20 | 1.3921(17) |
| C22 | C23 | 1.4044(17) |
| C22 | C27 | 1.3965(16) |
| C22 | B1 | 1.6308(17) |
| C23 | C24 | 1.3922(17) |
| C24 | C25 | 1.3940(19) |
| C25 | C26 | 1.397(2) |
| C25 | C28 | 1.498(2) |
| C26 | C27 | 1.3948(17) |

9.6.10 Bond angles

Table S40. Bond Angles in ° for **5a**.

| Atom | Atom | Atom | Angle/° |
|---|---|---|---|
| C8 | Bi1 | C1 | 85.30(4) |
| C2 | N1 | C1 | 108.54(10) |
| B1 | N1 | C1 | 128.12(9) |
| B1 | N1 | C2 | 122.97(9) |
| C3 | N2 | C1 | 108.82(10) |
| C4 | N2 | C1 | 126.48(10) |
| C4 | N2 | C3 | 124.50(10) |
| C9 | N3 | C8 | 108.37(9) |
| B1 | N3 | C8 | 128.27(9) |
| B1 | N3 | C9 | 123.15(9) |
| C10 | N4 | C8 | 109.17(10) |
| C11 | N4 | C8 | 126.81(10) |
| C11 | N4 | C10 | 123.89(10) |
| N1 | C1 | Bi1 | 122.76(8) |
| N2 | C1 | Bi1 | 129.59(8) |
| N2 | C1 | N1 | 107.57(10) |
| C3 | C2 | N1 | 108.26(11) |
| C2 | C3 | N2 | 106.80(11) |
| C5 | C4 | N2 | 109.52(10) |
| C6 | C4 | N2 | 108.42(10) |
| C6 | C4 | C5 | 112.07(11) |
| C7 | C4 | N2 | 109.33(10) |
| C7 | C4 | C5 | 108.49(11) |
| C7 | C4 | C6 | 108.98(11) |
| N3 | C8 | Bi1 | 122.51(8) |
| N4 | C8 | Bi1 | 130.03(8) |
| N4 | C8 | N3 | 107.46(9) |
| C10 | C9 | N3 | 108.46(10) |
| C9 | C10 | N4 | 106.53(11) |
| C12 | C11 | N4 | 109.20(9) |
| C13 | C11 | N4 | 108.84(10) |
| C13 | C11 | C12 | 111.06(10) |
| C14 | C11 | N4 | 109.74(10) |
| C14 | C11 | C12 | 108.80(10) |
| C14 | C11 | C13 | 109.18(10) |
| C20 | C15 | C16 | 116.15(11) |
| B1 | C15 | C16 | 122.86(10) |
| B1 | C15 | C20 | 120.09(10) |
| C17 | C16 | C15 | 121.81(11) |
| C18 | C17 | C16 | 121.15(12) |
| C19 | C18 | C17 | 117.69(11) |
| C21 | C18 | C17 | 121.47(14) |
| C21 | C18 | C19 | 120.84(14) |
| C20 | C19 | C18 | 120.73(12) |
| C19 | C20 | C15 | 122.39(12) |
| C27 | C22 | C23 | 116.09(11) |
| B1 | C22 | C23 | 120.44(10) |
| B1 | C22 | C27 | 123.47(10) |
| C24 | C23 | C22 | 122.43(12) |
| C25 | C24 | C23 | 120.66(12) |
| C26 | C25 | C24 | 117.71(12) |
| C28 | C25 | C24 | 121.06(14) |
| C28 | C25 | C26 | 121.21(14) |
| C27 | C26 | C25 | 121.13(12) |
| C26 | C27 | C22 | 121.97(12) |
| N3 | B1 | N1 | 108.74(9) |
| C15 | B1 | N1 | 111.36(9) |
| C15 | B1 | N3 | 110.86(9) |
| C22 | B1 | N1 | 107.53(9) |
| C22 | B1 | N3 | 107.39(9) |
| C22 | B1 | C15 | 110.82(9) |

9.6.11 Torsion angles

Table S41. Torsion Angles in ° for **5a**.

| Atom | Atom | Atom | Atom | Angle/° |
|---|---|---|---|---|
| Bi1 | C1 | N1 | C2 | 177.07(9) |
| Bi1 | C1 | N1 | B1 | -9.76(12) |
| Bi1 | C1 | N2 | C3 | -176.48(11) |
| Bi1 | C1 | N2 | C4 | -1.53(13) |
| Bi1 | C8 | N3 | C9 | 179.25(9) |
| Bi1 | C8 | N3 | B1 | 4.36(12) |
| Bi1 | C8 | N4 | C10 | -179.34(10) |
| Bi1 | C8 | N4 | C11 | 4.72(12) |
| N1 | C1 | N2 | C3 | 0.23(11) |
| N1 | C1 | N2 | C4 | 175.19(8) |
| N1 | C2 | C3 | N2 | 0.49(11) |
| N1 | B1 | N3 | C8 | -45.76(12) |
| N1 | B1 | N3 | C9 | 140.04(9) |
| N1 | B1 | C15 | C16 | -21.71(12) |
| N1 | B1 | C15 | C20 | 169.56(9) |
| N1 | B1 | C22 | C23 | -58.21(11) |
| N1 | B1 | C22 | C27 | 121.14(10) |
| N3 | C8 | N4 | C10 | 0.55(11) |
| N3 | C8 | N4 | C11 | -175.39(8) |
| N3 | C9 | C10 | N4 | -0.17(11) |
| N3 | B1 | C15 | C16 | -142.91(9) |
| N3 | B1 | C15 | C20 | 48.36(11) |
| N3 | B1 | C22 | C23 | 58.66(11) |
| N3 | B1 | C22 | C27 | -121.99(9) |
| C15 | C16 | C17 | C18 | -0.12(14) |
| C15 | C20 | C19 | C18 | 0.44(15) |
| C15 | B1 | C22 | C23 | 179.87(10) |
| C15 | B1 | C22 | C27 | -0.78(12) |
| C16 | C17 | C18 | C19 | -1.92(14) |
| C16 | C17 | C18 | C21 | 177.84(13) |
| C17 | C18 | C19 | C20 | 1.77(14) |
| C22 | C23 | C24 | C25 | -0.83(16) |
| C22 | C27 | C26 | C25 | -0.67(15) |
| C23 | C24 | C25 | C26 | 0.49(16) |
| C23 | C24 | C25 | C28 | -177.98(16) |
| C24 | C25 | C26 | C27 | 0.24(16) |

9.6.12 Hydrogen Fractional Atomic Coordinates and Equivalent Isotropic Displacement Parameters

Table S42. Hydrogen Fractional Atomic Coordinates (×10$^4$) and Equivalent Isotropic Displacement Parameters (Å$^2$×10$^3$) for **5a**. $U_{eq}$ is defined as 1/3 of the trace of the orthogonalised $U_{ij}$.

| Atom | x | y | z | $U_{eq}$ |
|---|---|---|---|---|
| H2 | 4491(15) | 3995(13) | 5638(7) | 33(4) |
| H3 | 2513(14) | 4829(13) | 6121(7) | 34(4) |
| H5a | 4925(16) | 7401(13) | 7508(7) | 35(3) |
| H5b | 3802(16) | 7679(13) | 6835(7) | 38(4) |
| H5c | 3297(16) | 7709(13) | 7529(7) | 35(4) |
| H6a | 3475(15) | 4637(13) | 7721(7) | 34(3) |
| H6b | 4796(15) | 5473(13) | 8026(7) | 28(3) |
| H6c | 3176(16) | 5837(14) | 8111(7) | 33(4) |
| H7a | 1758(15) | 6392(14) | 6460(8) | 37(3) |
| H7b | 1596(15) | 5245(14) | 6887(8) | 41(4) |
| H7c | 1462(16) | 6508(15) | 7218(8) | 43(4) |
| H9 | 9141(15) | 2978(14) | 6434(7) | 37(4) |
| H10 | 10774(15) | 2982(13) | 7582(7) | 34(4) |
| H12a | 10953(15) | 6011(12) | 8163(7) | 31(3) |
| H12b | 9440(15) | 6183(11) | 8377(7) | 27(3) |
| H12c | 10884(15) | 5855(12) | 8936(7) | 31(3) |
| H13a | 8120(16) | 4543(14) | 8602(7) | 38(4) |
| H13b | 8890(16) | 3281(14) | 8710(7) | 38(3) |
| H13c | 9448(15) | 4323(14) | 9237(7) | 37(3) |
| H14a | 11171(15) | 2971(13) | 8582(7) | 32(3) |
| H14b | 12059(15) | 4058(12) | 8330(7) | 32(3) |
| H14c | 11755(16) | 4052(13) | 9088(7) | 33(3) |
| H16 | 5772(15) | 6482(12) | 5623(7) | 31(4) |
| H17 | 6463(15) | 7781(12) | 4950(7) | 33(4) |
| H19 | 10295(17) | 6370(14) | 5294(9) | 55(6) |
| H20 | 9620(14) | 5034(13) | 5971(8) | 41(5) |
| H21a | 8840(20) | 8802(17) | 4782(10) | 73(5) |
| H21b | 9790(20) | 7915(17) | 4573(9) | 71(4) |
| H21c | 8230(20) | 7979(18) | 4210(9) | 71(4) |
| H23 | 6362(17) | 2495(13) | 6415(7) | 43(5) |
| H24 | 5987(19) | 874(13) | 5759(8) | 53(5) |
| H26 | 7116(18) | 2672(14) | 4327(7) | 51(5) |
| H27 | 7483(16) | 4266(13) | 4970(7) | 35(4) |
| H28a | 6190(20) | -77(18) | 4838(11) | 79(5) |
| H28b | 5300(20) | 661(19) | 4302(11) | 80(4) |
| H28c | 6840(20) | 555(16) | 4305(10) | 83(4) |

## 9.7 Single crystal structure analysis of 6

CCDC Deposition number 2423155

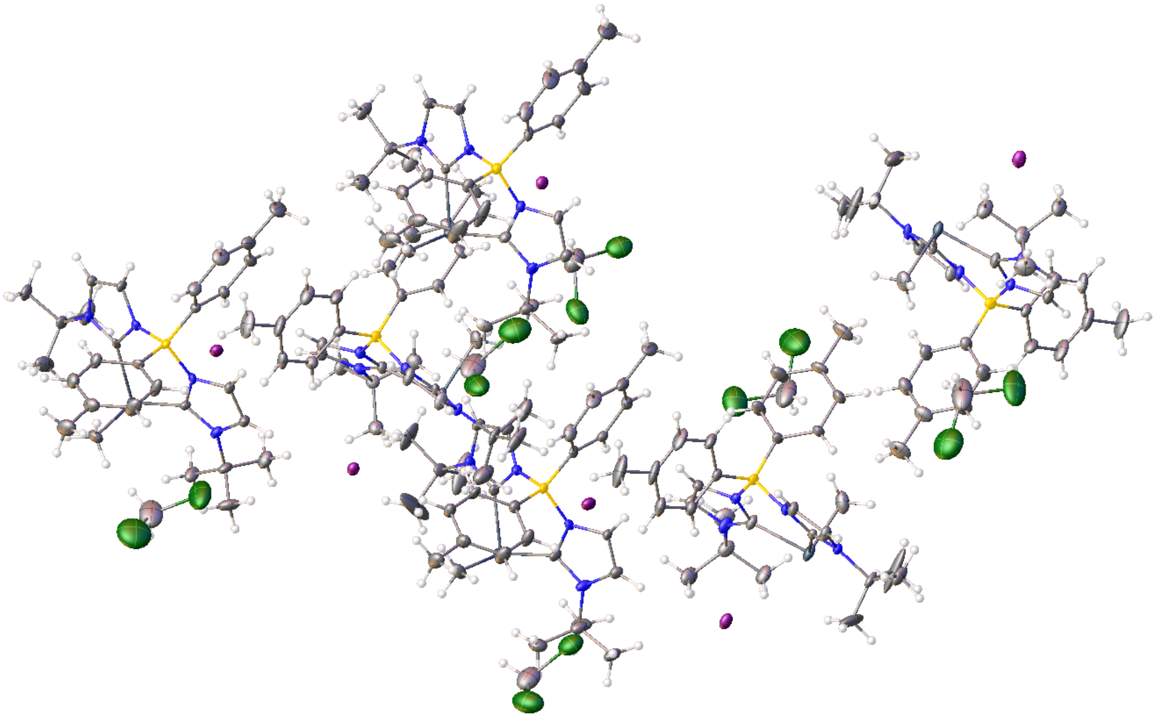

Figure S32. Olex-2 representation of the molecular structure of complex **6**

### 9.7.1 Experimental

Single colourless plate-shaped crystals of **6** were chosen from the sample as supplied. A suitable crystal 0.19×0.13×0.03 $mm^3$ was selected and mounted on a MITIGEN holder in oil on a Rigaku FRE+ diffractometer with Arc)Sec VHF Varimax confocal mirrors, a UG2 goniometer and HyPix 6000HE detector. The crystal was kept at a steady $T$ = 100(2) K during data collection. The structure was solved with the ShelXT (Sheldrick, 2015) structure solution program using the using dual methods solution method and by using Olex2 1.5-alpha (Dolomanov et al., 2009) as the graphical interface. The model was refined with ShelXL 2018/3 (Sheldrick, 2015) using full matrix least squares minimisation on $\boldsymbol{F}^2$ minimisation.

### 9.7.2 Crystal Data

$C_{30}H_{41}BCl_2IN_4Sb$, $M_r$ = 788.03, triclinic, *P*-1 (No. 2), a = 18.9347(2) Å, b = 23.6896(3) Å, c = 26.2232(4) Å, $\alpha$ = 63.4380(10)°, $\beta$ = 84.4850(10)°, $\gamma$ = 75.9090(10)°, *V* = 10203.4(2) Å$^3$, *T* = 100(2) K, *Z* = 12, *Z'* = 6, $\mu$(Mo $K_\alpha$) = 1.901 mm$^{-1}$, 201969 reflections measured, 52480 unique ($R_{int}$ = 0.0830) which were used in all calculations. The final $wR_2$ was 0.2244 (all data) and $R_1$ was 0.0827 (I≥2 $\sigma$(I)).

| Compound | **6** |
|---|---|
| | |
| Formula | $C_{30}H_{41}BCl_2IN_4Sb$ |
| $D_{calc.}$/ g cm$^{-3}$ | 1.539 |
| $\mu$/mm$^{-1}$ | 1.901 |
| Formula Weight | 788.03 |
| Colour | colourless |
| Shape | plate-shaped |
| Size/mm$^3$ | 0.19×0.13×0.03 |
| *T*/K | 100(2) |
| Crystal System | triclinic |
| Space Group | *P*-1 |
| *a*/Å | 18.9347(2) |
| *b*/Å | 23.6896(3) |
| *c*/Å | 26.2232(4) |
| $\alpha$/° | 63.4380(10) |
| $\beta$/° | 84.4850(10) |
| $\gamma$/° | 75.9090(10) |
| V/Å$^3$ | 10203.4(2) |
| *Z* | 12 |
| *Z'* | 6 |
| Wavelength/Å | 0.71073 |
| Radiation type | Mo $K_\alpha$ |
| $\Theta_{min}$/° | 1.642 |
| $\Theta_{max}$/° | 28.700 |
| Measured Refl's. | 201969 |
| Indep't Refl's | 52480 |
| Refl's I≥2 $\sigma$(I) | 42444 |
| $R_{int}$ | 0.0830 |
| Parameters | 2161 |
| Restraints | 60 |
| Largest Peak | 7.538 |
| Deepest Hole | -2.404 |
| GooF | 1.065 |
| $wR_2$ (all data) | 0.2244 |
| $wR_2$ | 0.2121 |
| $R_1$ (all data) | 0.0987 |
| $R_1$ | 0.0827 |

### 9.7.3 Structure Quality Indicators

| | | | | |
|---|---|---|---|---|
| Reflections: | d min (MoKα) 2Θ=57.4° 0.74 | I/σ(I) 16.0 | Rint m=3.85 8.30% | Full 50.5° 99.7 |
| Refinement: | Shift -0.001 | Max Peak 7.5 | Min Peak -2.4 | GooF 1.065 |

A colourless plate-shaped crystal with dimensions 0.19×0.13×0.03 $mm^3$ was mounted on a MITIGEN holder in oil. X-ray diffraction data were collected using a Rigaku FRE+ diffractometer with Arc)Sec VHF Varimax confocal mirrors, a UG2 goniometer and HyPix 6000HE detector equipped with an Oxford Cryosystems low-temperature device, operating at $T$ = 100(2) K.

Data were measured using profile data from $\omega$-scans of 0.4 ° per frame for 7.5 s using Mo $K_{\alpha}$ radiation (Rotating Anode, 45.0 kV, 55.0 mA). The total number of runs and images was based on the strategy calculation from the program CrysAlisPro system (CCD 43.121a 64-bit (release 24-04-2024)). The maximum resolution achieved was $\Theta$ = 28.700°.

Cell parameters were retrieved using the CrysAlisPro 1.171.43.121a (Rigaku OD, 2024) software and refined using CrysAlisPro 1.171.43.121a (Rigaku OD, 2024) on 89676 reflections, 44 % of the observed reflections. Data reduction was performed using the CrysAlisPro 1.171.43.121a (Rigaku OD, 2024) software which corrects for Lorentz polarisation. The final completeness is 99.70 % out to 28.700° in $\Theta$.

A gaussian absorption correction was performed using CrysAlisPro 1.171.43.121a (Rigaku OD, 2024) Numerical absorption correction based on gaussian integration over a multifaceted crystal model. Empirical absorption correction using spherical harmonics, implemented in SCALE3 ABSPACK scaling algorithm. The absorption coefficient $\mu$ of this material is 1.901 $mm^{-1}$ at this wavelength ($\lambda$ = 0.71073Å) and the minimum and maximum transmissions are 0.696 and 1.000.

The structure was solved in the space group *P*-1 (# 2) by using dual methods using the ShelXT (Sheldrick, 2015) structure solution program and refined by full matrix least squares minimisation on $F^2$ using ShelXL 2018/3 (Sheldrick, 2015). All non-hydrogen atoms were refined anisotropically. Hydrogen atom positions were calculated geometrically and refined using the riding model.

*_diffrn_special_details*: The structure appears to be a monoclinic C-centred structure, incommensurately modulated along the b-axis. It is also possibly twinned. This is a triclinic super cell solution using standard crystallographic solution and refinement programs.

*_refine_special_details*: Thermal restraints (RIGU) applied to two tBu groups.

*_exptl_absorpt_process_details*: CrysAlisPro 1.171.43.121a (Rigaku OD, 2024). Numerical absorption correction based on gaussian integration over a multifaceted crystal model. Empirical absorption correction using spherical harmonics, implemented in SCALE3 ABSPACK scaling algorithm.

The value of Z' is 6. The moiety formula is C29 H39 B N4 Sb, C H2 Cl2, I.

### 9.7.4 Data Plots: Diffraction Data

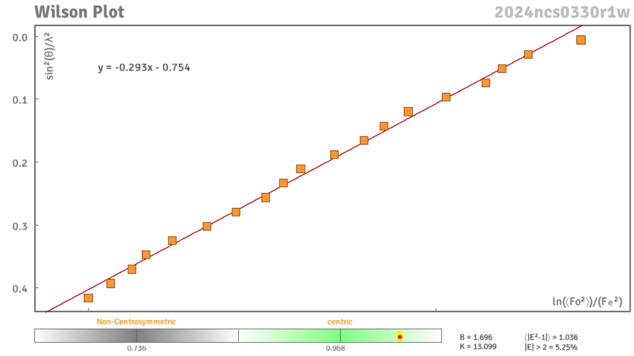


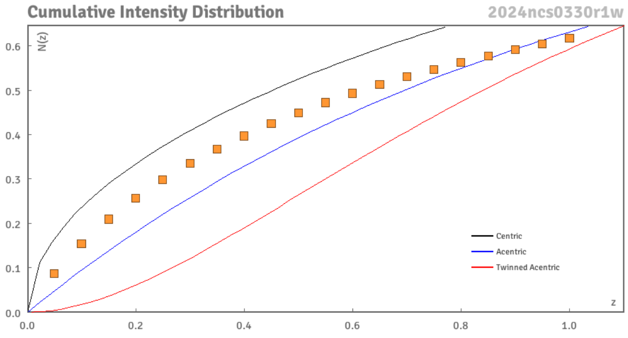


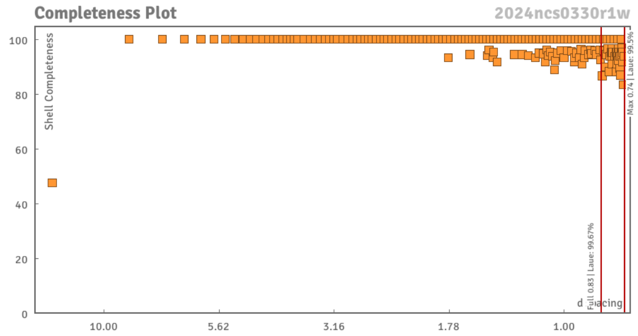


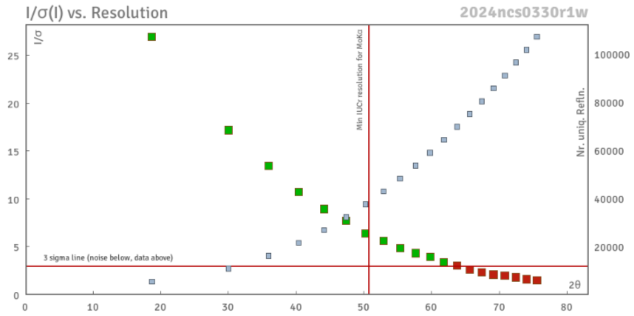


### 9.7.5 Data Plots: Refinement and Data

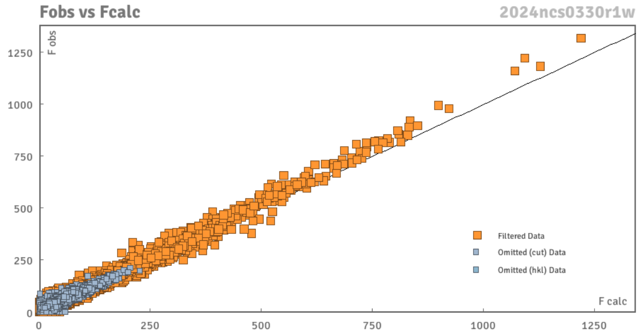


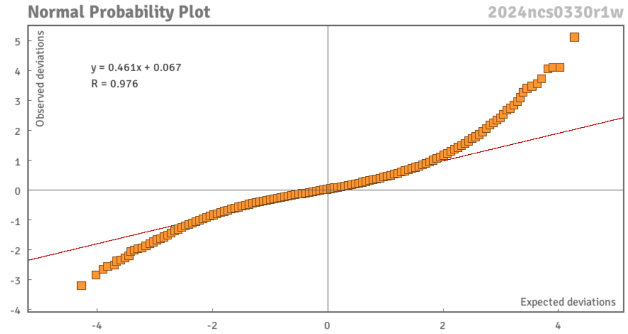

### 9.7.6 Reflection Statistics

| | | | |
|---|---|---|---|
| Total reflections (after filtering) | 201969 | Unique reflections | 52480 |
| Completeness | 0.995 | Mean I/$\sigma$ | 11.02 |
| $hkl_{max}$ collected | (32, 39, 42) | $hkl_{min}$ collected | (-31, -41, -44) |
| $hkl_{max}$ used | (25, 32, 35) | $hkl_{min}$ used | (-25, -28, 0) |
| Lim $d_{max}$ collected | 999.0 | Lim $d_{min}$ collected | 0.74 |
| $d_{max}$ used | 12.4 | $d_{min}$ used | 0.74 |
| Friedel pairs | 43714 | Friedel pairs merged | 1 |
| Inconsistent equivalents | 944 | $R_{int}$ | 0.083 |
| $R_{sigma}$ | 0.0624 | Intensity transformed | 0 |
| Omitted reflections | 0 | Omitted by user (OMIT hkl) | 0 |
| Multiplicity | (50656, 46219, 26565, 12721, 5415, 2934, 952, 269, 94, 21, 1) | Maximum multiplicity | 14 |
| Removed systematic absences | 0 | Filtered off (Shel/OMIT) | 126266 |

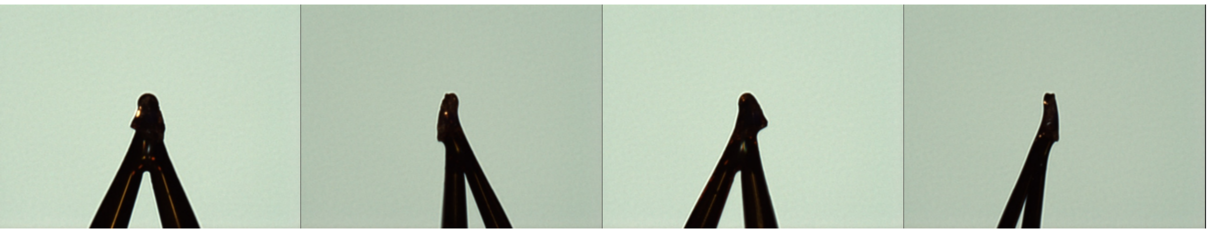

### 9.7.7 Fractional Atomic Coordinates and Equivalent Isotropic Displacement Parameters

Table S43. Fractional Atomic Coordinates (×10[4]) and Equivalent Isotropic Displacement Parameters (Å[2]×10[3]) for **6**. $U_{eq}$ is defined as 1/3 of the trace of the orthogonalised $U_{ij}$.

| Atom | x | y | z | $U_{eq}$ |
|---|---|---|---|---|
| Sb1_1 | 2640.1(3) | 3101.4(2) | 9259.8(2) | 26.96(12) |
| N1_1 | 3265(3) | 3886(2) | 8083(2) | 14.8(10) |
| N2_1 | 3892(3) | 2883(3) | 8430(3) | 19.3(12) |
| N3_1 | 2378(3) | 4583(2) | 8490(2) | 14.8(10) |
| N4_1 | 1852(4) | 4413(3) | 9312(3) | 23.7(13) |
| C1_1 | 3315(4) | 3293(3) | 8525(3) | 16.3(12) |
| C2_1 | 3821(4) | 3848(3) | 7715(3) | 23.1(15) |
| C3_1 | 4216(4) | 3221(3) | 7929(3) | 23.1(15) |
| C4_1 | 4179(5) | 2173(3) | 8803(4) | 28.4(16) |
| C5_1 | 4594(7) | 1855(4) | 8447(4) | 59(3) |
| C6_1 | 4618(11) | 2097(6) | 9269(6) | 102(6) |
| C7_1 | 3524(8) | 1857(5) | 8982(10) | 137(9) |
| C8_1 | 2229(4) | 4126(3) | 8989(3) | 23.1(15) |
| C9_1 | 2110(4) | 5177(3) | 8495(3) | 17.2(13) |
| C10_1 | 1778(4) | 5073(3) | 9002(3) | 22.3(14) |
| C11_1 | 1524(5) | 4075(4) | 9890(4) | 33.4(19) |
| C12_1 | 1091(5) | 3621(4) | 9857(4) | 39(2) |
| C13_1 | 994(5) | 4585(4) | 10027(4) | 36(2) |
| C14_1 | 2116(5) | 3707(4) | 10340(4) | 36(2) |
| C15_1 | 1928(4) | 4398(3) | 7695(3) | 19.6(13) |
| C16_1 | 1995(4) | 3975(4) | 7443(3) | 23.4(15) |
| C17_1 | 1389(4) | 3871(4) | 7263(4) | 26.4(16) |
| C18_1 | 698(4) | 4186(4) | 7323(4) | 27.9(16) |
| C19_1 | 617(5) | 4612(5) | 7568(4) | 36(2) |
| C20_1 | 1218(4) | 4715(4) | 7745(4) | 29.9(17) |
| C21_1 | 38(5) | 4068(5) | 7136(4) | 36(2) |
| C22_1 | 2949(4) | 5119(3) | 7480(3) | 16.5(12) |
| C23_1 | 2743(5) | 5456(3) | 6910(3) | 26.6(16) |
| C24_1 | 3078(6) | 5946(4) | 6522(3) | 34(2) |
| C25_1 | 3622(5) | 6119(3) | 6698(3) | 28.4(17) |
| C26_1 | 3983(6) | 6656(4) | 6293(4) | 42(2) |
| C27_1 | 3836(4) | 5786(3) | 7268(3) | 24.9(15) |
| C28_1 | 3501(4) | 5292(3) | 7652(3) | 18.7(13) |
| C29_1 | 1793(5) | 2942(4) | 8872(4) | 35(2) |
| B1_1 | 2623(4) | 4506(3) | 7927(3) | 14.3(13) |
| Sb1_2 | 2785.5(3) | -138.5(2) | 5997.7(2) | 17.12(10) |
| N1_2 | 3141(3) | 598(2) | 4713(2) | 12.8(10) |
| N2_2 | 3771(3) | -403(3) | 5035(3) | 16.4(11) |
| N3_2 | 2328(3) | 1315(2) | 5154(2) | 13.3(10) |
| N4_2 | 2008(3) | 1213(3) | 6010(2) | 17.6(11) |
| C1_2 | 3252(3) | 22(3) | 5170(3) | 13.8(12) |
| C2_2 | 3596(4) | 550(3) | 4278(3) | 18.9(13) |
| C3_2 | 3984(4) | -75(3) | 4484(3) | 17.8(13) |
| C4_2 | 4116(4) | -1093(3) | 5425(3) | 22.4(15) |
| C5_2 | 4439(5) | -1444(3) | 5058(4) | 27.5(16) |
| C6_2 | 4710(6) | -1076(4) | 5762(4) | 43(2) |
| C7_2 | 3566(6) | -1437(4) | 5809(5) | 52(3) |
| C8_2 | 2320(4) | 886(3) | 5702(3) | 14.7(12) |
| C9_2 | 2024(4) | 1926(3) | 5114(3) | 17.8(13) |
| C10_2 | 1824(4) | 1865(3) | 5643(3) | 20.3(14) |

| | | | | |
|---|---|---|---|---|
| C11_2 | 1872(4) | 990(3) | 6637(3) | 23.8(15) |
| C12_2 | 2144(5) | 257(4) | 6976(3) | 31.1(18) |
| C13_2 | 1061(5) | 1185(5) | 6711(4) | 38(2) |
| C14_2 | 2281(5) | 1333(4) | 6842(4) | 30.0(17) |
| C15_2 | 1798(3) | 1146(3) | 4369(3) | 16.3(12) |
| C16_2 | 1788(4) | 623(3) | 4257(3) | 22.4(14) |
| C17_2 | 1147(4) | 540(4) | 4107(3) | 26.6(16) |
| C18_2 | 487(4) | 981(4) | 4057(4) | 27.8(16) |
| C19_2 | 492(4) | 1513(5) | 4145(4) | 34.6(19) |
| C20_2 | 1129(4) | 1594(4) | 4298(4) | 29.4(17) |
| C21_2 | -208(5) | 876(5) | 3914(4) | 40(2) |
| C22_2 | 2857(4) | 1839(3) | 4147(3) | 15.1(12) |
| C23_2 | 2640(4) | 2216(3) | 3578(3) | 23.6(15) |
| C24_2 | 2961(5) | 2725(4) | 3213(4) | 33.5(19) |
| C25_2 | 3509(5) | 2872(3) | 3408(4) | 30.3(18) |
| C26_2 | 3853(6) | 3433(4) | 3032(5) | 48(3) |
| C27_2 | 3745(4) | 2503(3) | 3974(4) | 24.2(15) |
| C28_2 | 3426(4) | 1994(3) | 4332(3) | 19.4(13) |
| C29_2 | 1785(5) | -316(4) | 5816(4) | 30.0(17) |
| B1_2 | 2524(4) | 1234(3) | 4585(3) | 12.8(12) |
| Sb1_3 | 7791.0(3) | 9215.3(2) | 7293.2(2) | 19.4(1) |
| N1_3 | 7409(3) | 9805(3) | 8156(2) | 15.1(10) |
| N2_3 | 7042(3) | 10566(3) | 7309(2) | 17.9(11) |
| N3_3 | 8238(3) | 8654(2) | 8569(2) | 15.0(10) |
| N4_3 | 8847(3) | 7992(3) | 8221(3) | 16.9(11) |
| C1_3 | 7359(4) | 9932(3) | 7605(3) | 19.3(13) |
| C2_3 | 7126(4) | 10373(3) | 8203(3) | 19.3(13) |
| C3_3 | 6894(4) | 10845(3) | 7681(3) | 21.0(14) |
| C4_3 | 6859(5) | 10979(3) | 6680(3) | 26.7(16) |
| C5_3 | 6038(5) | 11238(5) | 6633(4) | 39(2) |
| C6_3 | 7250(5) | 11532(4) | 6467(4) | 33.6(19) |
| C7_3 | 7114(5) | 10605(4) | 6330(3) | 33.0(19) |
| C8_3 | 8317(4) | 8539(3) | 8107(3) | 16.1(12) |
| C9_3 | 8724(4) | 8179(3) | 8985(3) | 24.0(15) |
| C10_3 | 9114(4) | 7773(3) | 8762(3) | 21.0(14) |
| C11_3 | 9171(4) | 7691(3) | 7831(3) | 24.9(15) |
| C12_3 | 8605(7) | 7777(6) | 7426(6) | 75(5) |
| C13_3 | 9476(5) | 6973(3) | 8186(4) | 30.7(18) |
| C14_3 | 9781(7) | 8024(5) | 7503(5) | 60(4) |
| C15_3 | 6905(4) | 8844(3) | 8917(3) | 19.8(13) |
| C16_3 | 6224(5) | 9228(4) | 8946(4) | 32.4(18) |
| C17_3 | 5599(5) | 8994(4) | 9096(4) | 35.3(19) |
| C18_3 | 5613(4) | 8356(4) | 9232(4) | 29.9(17) |
| C19_3 | 6279(5) | 7959(4) | 9219(4) | 32.8(18) |
| C20_3 | 6901(4) | 8197(3) | 9064(4) | 25.8(16) |
| C21_3 | 4922(5) | 8103(5) | 9372(5) | 41(2) |
| C22_3 | 7960(4) | 9286(3) | 9171(3) | 16.5(12) |
| C23_3 | 7756(5) | 9063(4) | 9741(3) | 27.4(16) |
| C24_3 | 8084(6) | 9179(4) | 10127(4) | 41(2) |
| C25_3 | 8618(6) | 9525(4) | 9966(4) | 37(2) |
| C26_3 | 8958(8) | 9675(5) | 10373(5) | 57(3) |
| C27_3 | 8839(5) | 9740(4) | 9404(4) | 28.6(17) |
| C28_3 | 8521(4) | 9620(3) | 9017(3) | 20.3(13) |
| C29_3 | 6794(5) | 8853(4) | 7487(4) | 31.5(18) |
| B1_3 | 7617(4) | 9144(3) | 8712(3) | 15.0(13) |
| Sb1_4 | 2518.5(2) | 4347.3(2) | 5834.8(2) | 16.52(9) |

| | | | | |
|---|---|---|---|---|
| N1_4 | 1811(3) | 4705(2) | 4679(2) | 14.7(10) |
| N2_4 | 1198(3) | 5367(3) | 5025(2) | 13.7(10) |
| N3_4 | 2736(3) | 3602(2) | 5095(2) | 15.2(10) |
| N4_4 | 3310(3) | 2973(3) | 5914(2) | 16.2(11) |
| C1_4 | 1788(3) | 4873(3) | 5108(3) | 14.7(12) |
| C2_4 | 1222(4) | 5091(3) | 4328(3) | 24.4(15) |
| C3_4 | 842(4) | 5502(3) | 4536(3) | 22.9(15) |
| C4_4 | 946(4) | 5715(3) | 5389(3) | 25.2(16) |
| C5_4 | 439(7) | 6353(5) | 5052(4) | 54(3) |
| C6_4 | 654(7) | 5308(5) | 5930(5) | 58(3) |
| C7_4 | 1627(6) | 5934(5) | 5463(5) | 48(3) |
| C8_4 | 2926(4) | 3580(3) | 5582(3) | 15.6(12) |
| C9_4 | 2999(4) | 3000(3) | 5110(3) | 17.4(13) |
| C10_4 | 3352(4) | 2605(3) | 5624(3) | 18.6(13) |
| C11_4 | 3650(4) | 2739(3) | 6485(3) | 18.0(13) |
| C12_4 | 4068(4) | 3235(4) | 6455(3) | 24.1(15) |
| C13_4 | 4195(4) | 2098(3) | 6618(3) | 26.1(16) |
| C14_4 | 3066(4) | 2648(4) | 6937(3) | 24.5(15) |
| C15_4 | 3137(4) | 4582(3) | 4281(3) | 17.9(13) |
| C16_4 | 3059(4) | 5253(3) | 4048(3) | 18.9(13) |
| C17_4 | 3654(4) | 5542(3) | 3868(3) | 21.1(14) |
| C18_4 | 4348(4) | 5170(3) | 3917(3) | 23.5(15) |
| C19_4 | 4436(4) | 4504(4) | 4155(4) | 31.0(18) |
| C20_4 | 3846(4) | 4219(4) | 4330(4) | 29.4(17) |
| C21_4 | 4993(4) | 5482(4) | 3737(4) | 31.0(18) |
| C22_4 | 2106(4) | 4057(3) | 4095(3) | 16.3(12) |
| C23_4 | 2240(5) | 4335(4) | 3508(3) | 26.5(16) |
| C24_4 | 1870(5) | 4236(4) | 3134(4) | 32.5(19) |
| C25_4 | 1366(5) | 3856(4) | 3318(4) | 32.0(19) |
| C26_4 | 965(7) | 3737(5) | 2919(4) | 48(3) |
| C27_4 | 1221(4) | 3580(3) | 3900(4) | 24.4(15) |
| C28_4 | 1585(4) | 3679(3) | 4278(3) | 18.6(13) |
| C29_4 | 3349(4) | 4885(4) | 5437(4) | 27.8(16) |
| B1_4 | 2452(4) | 4230(3) | 4531(3) | 13.8(13) |
| Sb1_5 | 2466.6(3) | 6500.4(2) | 2462.4(2) | 17.88(10) |
| N1_5 | 3092(3) | 7323(2) | 1293(2) | 14.2(10) |
| N2_5 | 3716(3) | 6316(2) | 1618(2) | 14.8(10) |
| N3_5 | 2188(3) | 7991(2) | 1730(2) | 14.4(10) |
| N4_5 | 1650(3) | 7784(3) | 2559(3) | 18.1(11) |
| C1_5 | 3152(3) | 6722(3) | 1722(3) | 13.4(11) |
| C2_5 | 3635(4) | 7290(3) | 916(3) | 21.0(14) |
| C3_5 | 4030(4) | 6666(3) | 1111(3) | 21.5(14) |
| C4_5 | 3993(4) | 5607(3) | 1978(3) | 19.8(14) |
| C5_5 | 4525(5) | 5315(4) | 1642(4) | 33.2(19) |
| C6_5 | 4334(6) | 5503(5) | 2515(4) | 47(3) |
| C7_5 | 3339(5) | 5280(4) | 2098(4) | 37(2) |
| C8_5 | 2033(4) | 7516(3) | 2219(3) | 17.1(12) |
| C9_5 | 1916(4) | 8572(3) | 1754(3) | 18.8(13) |
| C10_5 | 1582(4) | 8448(3) | 2263(3) | 20.2(13) |
| C11_5 | 1339(4) | 7435(3) | 3128(3) | 22.6(14) |
| C12_5 | 935(4) | 6957(4) | 3104(4) | 28.4(16) |
| C13_5 | 786(5) | 7927(4) | 3273(4) | 32.2(19) |
| C14_5 | 1942(5) | 7084(4) | 3575(3) | 28.6(16) |
| C15_5 | 1768(4) | 7851(3) | 900(3) | 19.1(13) |
| C16_5 | 1821(4) | 7387(3) | 702(3) | 20.2(13) |
| C17_5 | 1216(4) | 7280(4) | 522(3) | 25.0(15) |

| | | | | |
|---|---|---|---|---|
| C18_5 | 527(4) | 7648(5) | 527(4) | 32.1(18) |
| C19_5 | 459(5) | 8111(6) | 726(6) | 58(3) |
| C20_5 | 1073(5) | 8212(6) | 897(6) | 55(3) |
| C21_5 | -130(5) | 7522(6) | 351(4) | 42(2) |
| C22_5 | 2787(4) | 8552(3) | 750(3) | 18.1(13) |
| C23_5 | 2645(6) | 8902(4) | 164(3) | 33.8(19) |
| C24_5 | 2997(7) | 9379(4) | -189(4) | 48(3) |
| C25_5 | 3521(6) | 9540(4) | 28(4) | 42(2) |
| C26_5 | 3923(9) | 10056(5) | -349(6) | 75(5) |
| C27_5 | 3673(5) | 9212(3) | 611(4) | 30.0(18) |
| C28_5 | 3321(4) | 8721(3) | 961(3) | 21.7(14) |
| C29_5 | 1622(4) | 6341(4) | 2078(4) | 27.9(16) |
| B1_5 | 2449(4) | 7942(3) | 1155(3) | 15.7(13) |
| Sb1_6 | 2368.4(3) | 7495.0(2) | 9241.3(2) | 22.63(11) |
| N1_6 | 1937(3) | 8024(2) | 7966(2) | 14.4(10) |
| N2_6 | 1315(3) | 8712(2) | 8285(2) | 14.8(10) |
| N3_6 | 2774(3) | 6871(3) | 8409(2) | 14.9(10) |
| N4_6 | 3142(4) | 6125(3) | 9268(3) | 23.2(13) |
| C1_6 | 1849(3) | 8155(3) | 8418(3) | 13.1(11) |
| C2_6 | 1453(4) | 8497(3) | 7545(3) | 18.9(13) |
| C3_6 | 1067(4) | 8923(3) | 7745(3) | 20.3(14) |
| C4_6 | 995(4) | 9017(3) | 8671(3) | 22.8(14) |
| C5_6 | 641(5) | 9722(4) | 8312(4) | 32.9(19) |
| C6_6 | 452(9) | 8654(6) | 9038(7) | 94(6) |
| C7_6 | 1594(8) | 9004(8) | 9014(8) | 112(7) |
| C8_6 | 2818(4) | 6758(3) | 8955(3) | 18.7(13) |
| C9_6 | 3079(4) | 6292(3) | 8381(3) | 17.8(13) |
| C10_6 | 3314(4) | 5830(3) | 8913(3) | 20.7(14) |
| C11_6 | 3310(5) | 5726(4) | 9898(3) | 28.0(16) |
| C12_6 | 3040(5) | 6107(4) | 10237(3) | 31.8(18) |
| C13_6 | 4135(5) | 5473(4) | 9957(4) | 36(2) |
| C14_6 | 2927(6) | 5162(4) | 10115(4) | 38(2) |
| C15_6 | 3257(4) | 7823(3) | 7613(3) | 17.2(13) |
| C16_6 | 3277(4) | 8453(3) | 7510(3) | 22.2(14) |
| C17_6 | 3905(4) | 8689(4) | 7355(4) | 28.1(16) |
| C18_6 | 4558(4) | 8298(4) | 7307(4) | 27.3(16) |
| C19_6 | 4553(5) | 7679(4) | 7403(5) | 39(2) |
| C20_6 | 3921(4) | 7444(4) | 7551(4) | 32.4(19) |
| C21_6 | 5253(5) | 8546(5) | 7171(4) | 39(2) |
| C22_6 | 2188(4) | 7339(3) | 7416(3) | 15.5(12) |
| C23_6 | 2341(5) | 7555(4) | 6844(3) | 26.9(16) |
| C24_6 | 1981(6) | 7430(4) | 6476(4) | 39(2) |
| C25_6 | 1458(5) | 7057(4) | 6698(4) | 37(2) |
| C26_6 | 1068(8) | 6898(6) | 6311(6) | 65(4) |
| C27_6 | 1298(5) | 6835(4) | 7269(4) | 29.1(17) |
| C28_6 | 1649(4) | 6976(3) | 7619(3) | 22.1(14) |
| C29_6 | 3347(5) | 7845(4) | 9059(4) | 37(2) |
| B1_6 | 2545(4) | 7518(3) | 7838(3) | 14.7(13) |
| Cl51_13 | 4693(3) | 10587.6(16) | 7983.0(15) | 99.7(16) |
| Cl52_13 | 3633(3) | 9790(3) | 8180.5(19) | 102.2(15) |
| C51_13 | 4421(11) | 10004(7) | 7845(5) | 90(6) |
| Cl51_14 | 4559.2(19) | 7066.9(15) | 4667.7(12) | 62.0(8) |
| Cl52_14 | 3152(2) | 6748.8(15) | 4764.5(13) | 64.3(8) |
| C51_14 | 4021(8) | 6693(6) | 4451(6) | 62(3) |
| Cl51_15 | -280.5(18) | 5089.9(16) | 8702.8(12) | 58.4(7) |
| Cl52_15 | -1705(2) | 4860(2) | 8636.7(16) | 83.5(11) |

| | | | | |
|---|---|---|---|---|
| C51_15 | -826(8) | 4507(6) | 8902(5) | 64(4) |
| Cl51_16 | -1948(2) | 8251(2) | 1864.8(14) | 74.1(10) |
| Cl52_16 | -505(3) | 8380(2) | 1985.1(16) | 88.5(12) |
| C51_16 | -1103(9) | 7813(6) | 2185(5) | 76(5) |
| Cl51_17 | 5385(3) | 6121.8(18) | 8639.9(15) | 83.8(12) |
| Cl52_17 | 6523(3) | 6822(3) | 8430.3(19) | 106.0(16) |
| C51_17 | 5679(10) | 6697(7) | 8762(5) | 78(5) |
| Cl51_18 | -265(3) | 1897(2) | 5355.4(16) | 99.2(16) |
| Cl52_18 | -1393(3) | 1363(2) | 5135.2(19) | 113.7(18) |
| C51_18 | -548(10) | 1210(7) | 5467(5) | 81(5) |
| I61_7 | 9199.3(3) | 10141.3(2) | 7106.0(2) | 27.03(11) |
| I61_8 | 3893.4(2) | 7308.4(2) | 2713.7(2) | 19.61(10) |
| I61_9 | 1087.2(2) | 3296.6(2) | 6081.6(2) | 19.79(10) |
| I61_10 | 977.5(3) | 6551.3(2) | 9405.5(2) | 22.99(10) |
| I61_11 | 4189.4(3) | 603.4(2) | 6186.5(2) | 24.08(11) |
| I61_12 | 4085.5(3) | 3835.2(2) | 9533.7(2) | 27.88(12) |

### 9.7.8 Anisotropic Displacement Parameters

Table S44. Anisotropic Displacement Parameters (×10$^4$) for **6**. The anisotropic displacement factor exponent takes the form: $-2\pi^2[h^2a^{*2} \times U_{11}+ ... +2hka^* \times b^* \times U_{12}]$

| Atom | $U_{11}$ | $U_{22}$ | $U_{33}$ | $U_{23}$ | $U_{13}$ | $U_{12}$ |
|---|---|---|---|---|---|---|
| Sb1_1 | 29.8(3) | 11.34(19) | 29.1(3) | -3.02(18) | 14.1(2) | -3.24(18) |
| N1_1 | 17(3) | 11(2) | 15(3) | -5(2) | 3(2) | -2.9(19) |
| N2_1 | 16(3) | 11(2) | 30(3) | -9(2) | 2(2) | -2(2) |
| N3_1 | 15(3) | 8(2) | 20(3) | -5(2) | 3(2) | -3.7(19) |
| N4_1 | 27(3) | 18(3) | 18(3) | -5(2) | 7(2) | 2(2) |
| C1_1 | 13(3) | 11(3) | 24(3) | -9(2) | 3(2) | 0(2) |
| C2_1 | 25(4) | 15(3) | 27(4) | -11(3) | 11(3) | -2(3) |
| C3_1 | 25(4) | 21(3) | 25(4) | -14(3) | 9(3) | -3(3) |
| C4_1 | 34(4) | 9(3) | 35(4) | -6(3) | 13(3) | -2(3) |
| C5_1 | 98(9) | 21(4) | 42(5) | -19(4) | -13(5) | 30(5) |
| C6_1 | 188(16) | 35(6) | 70(8) | -31(6) | -75(10) | 48(8) |
| C7_1 | 64(8) | 15(4) | 280(20) | -31(9) | 75(10) | -14(5) |
| C8_1 | 29(4) | 12(3) | 25(4) | -9(3) | 12(3) | -2(3) |
| C9_1 | 20(3) | 13(3) | 21(3) | -11(2) | 8(3) | -5(2) |
| C10_1 | 21(3) | 19(3) | 25(4) | -11(3) | 5(3) | 0(3) |
| C11_1 | 41(5) | 25(4) | 28(4) | -10(3) | 16(4) | -5(3) |
| C12_1 | 41(5) | 38(5) | 36(5) | -14(4) | 18(4) | -17(4) |
| C13_1 | 46(5) | 29(4) | 31(4) | -16(3) | 20(4) | -6(4) |
| C14_1 | 39(5) | 36(4) | 22(4) | -5(3) | 12(3) | -9(4) |
| C15_1 | 20(3) | 15(3) | 24(3) | -9(3) | 3(3) | -3(2) |
| C16_1 | 18(3) | 25(3) | 34(4) | -23(3) | 7(3) | -1(3) |
| C17_1 | 22(4) | 27(4) | 39(4) | -22(3) | 6(3) | -7(3) |
| C18_1 | 21(4) | 35(4) | 35(4) | -20(3) | 7(3) | -11(3) |
| C19_1 | 19(4) | 47(5) | 55(6) | -37(5) | 2(4) | -1(3) |
| C20_1 | 20(4) | 32(4) | 47(5) | -30(4) | -2(3) | 2(3) |
| C21_1 | 23(4) | 51(5) | 49(5) | -34(5) | 7(4) | -15(4) |
| C22_1 | 20(3) | 8(2) | 20(3) | -7(2) | 2(2) | 0(2) |
| C23_1 | 36(4) | 17(3) | 22(4) | -7(3) | -6(3) | -1(3) |
| C24_1 | 63(6) | 19(3) | 12(3) | -2(3) | 3(3) | -5(4) |
| C25_1 | 42(5) | 15(3) | 27(4) | -10(3) | 13(3) | -5(3) |
| C26_1 | 65(7) | 18(4) | 36(5) | -7(3) | 29(5) | -16(4) |
| C27_1 | 28(4) | 17(3) | 31(4) | -13(3) | 10(3) | -8(3) |
| C28_1 | 21(3) | 15(3) | 21(3) | -10(2) | 8(3) | -4(2) |
| C29_1 | 24(4) | 26(4) | 58(6) | -20(4) | 12(4) | -11(3) |
| B1_1 | 15(3) | 11(3) | 19(3) | -9(3) | 2(3) | -1(2) |
| Sb1_2 | 20.4(2) | 11.22(18) | 19.1(2) | -6.61(16) | 6.43(17) | -4.84(15) |
| N1_2 | 9(2) | 12(2) | 18(3) | -8(2) | 1(2) | -3.8(18) |
| N2_2 | 13(3) | 12(2) | 24(3) | -10(2) | 7(2) | -4(2) |
| N3_2 | 16(3) | 11(2) | 16(3) | -7(2) | 2(2) | -6.0(19) |
| N4_2 | 16(3) | 20(3) | 18(3) | -12(2) | 1(2) | 0(2) |
| C1_2 | 7(3) | 9(2) | 26(3) | -8(2) | 3(2) | -2(2) |
| C2_2 | 21(3) | 13(3) | 23(3) | -11(3) | 7(3) | -2(2) |
| C3_2 | 16(3) | 14(3) | 24(3) | -10(3) | 7(3) | -4(2) |
| C4_2 | 22(4) | 12(3) | 32(4) | -11(3) | 6(3) | 0(2) |
| C5_2 | 35(4) | 15(3) | 33(4) | -15(3) | 4(3) | 1(3) |
| C6_2 | 48(6) | 30(4) | 48(6) | -23(4) | -18(4) | 13(4) |
| C7_2 | 49(6) | 17(4) | 74(7) | -14(4) | 31(5) | -3(4) |
| C8_2 | 14(3) | 12(3) | 16(3) | -7(2) | 6(2) | -2(2) |
| C9_2 | 18(3) | 14(3) | 23(3) | -10(2) | 4(3) | -3(2) |

| | | | | | | |
|---|---|---|---|---|---|---|
| C10_2 | 23(4) | 18(3) | 24(3) | -13(3) | 8(3) | -7(3) |
| C11_2 | 27(4) | 23(3) | 20(3) | -11(3) | 10(3) | -4(3) |
| C12_2 | 48(5) | 19(3) | 21(4) | -7(3) | 9(3) | -5(3) |
| C13_2 | 27(4) | 44(5) | 30(4) | -11(4) | 13(3) | 0(4) |
| C14_2 | 37(5) | 29(4) | 26(4) | -16(3) | -4(3) | -2(3) |
| C15_2 | 9(3) | 23(3) | 21(3) | -12(3) | 3(2) | -7(2) |
| C16_2 | 21(4) | 24(3) | 27(4) | -17(3) | -3(3) | -1(3) |
| C17_2 | 29(4) | 29(4) | 30(4) | -19(3) | 2(3) | -11(3) |
| C18_2 | 22(4) | 40(4) | 28(4) | -18(3) | 3(3) | -13(3) |
| C19_2 | 14(4) | 51(5) | 53(6) | -38(5) | -1(3) | -3(3) |
| C20_2 | 18(4) | 36(4) | 50(5) | -35(4) | -3(3) | 0(3) |
| C21_2 | 28(5) | 60(6) | 46(6) | -31(5) | 4(4) | -18(4) |
| C22_2 | 16(3) | 13(3) | 16(3) | -6(2) | 3(2) | -4(2) |
| C23_2 | 29(4) | 19(3) | 18(3) | -6(3) | -1(3) | -2(3) |
| C24_2 | 43(5) | 20(3) | 23(4) | 0(3) | -2(3) | -2(3) |
| C25_2 | 34(4) | 14(3) | 36(4) | -9(3) | 15(3) | -4(3) |
| C26_2 | 67(7) | 21(4) | 48(6) | -9(4) | 28(5) | -19(4) |
| C27_2 | 20(3) | 17(3) | 36(4) | -12(3) | 9(3) | -6(3) |
| C28_2 | 20(3) | 15(3) | 24(3) | -8(3) | 3(3) | -5(2) |
| C29_2 | 29(4) | 31(4) | 35(4) | -15(3) | 11(3) | -19(3) |
| B1_2 | 15(3) | 13(3) | 12(3) | -7(2) | 2(2) | -3(2) |
| Sb1_3 | 24.1(2) | 15.90(19) | 19.4(2) | -10.94(17) | -7.53(17) | 2.70(17) |
| N1_3 | 18(3) | 13(2) | 16(3) | -8(2) | -1(2) | -2(2) |
| N2_3 | 22(3) | 13(2) | 15(3) | -5(2) | -5(2) | 3(2) |
| N3_3 | 19(3) | 11(2) | 15(3) | -7(2) | -4(2) | 0(2) |
| N4_3 | 15(3) | 12(2) | 26(3) | -11(2) | -8(2) | 1(2) |
| C1_3 | 22(3) | 15(3) | 22(3) | -11(3) | -6(3) | 2(2) |
| C2_3 | 21(3) | 15(3) | 23(3) | -11(3) | 0(3) | 0(2) |
| C3_3 | 22(3) | 14(3) | 25(4) | -9(3) | -5(3) | 3(2) |
| C4_3 | 37(4) | 17(3) | 18(3) | -3(3) | -9(3) | 2(3) |
| C5_3 | 24(4) | 45(5) | 34(5) | -9(4) | -12(3) | 4(4) |
| C6_3 | 47(5) | 18(3) | 28(4) | -6(3) | -2(4) | -1(3) |
| C7_3 | 47(5) | 24(4) | 21(4) | -8(3) | -7(3) | 4(3) |
| C8_3 | 20(3) | 11(3) | 19(3) | -9(2) | -4(2) | -2(2) |
| C9_3 | 31(4) | 14(3) | 22(3) | -8(3) | -11(3) | 7(3) |
| C10_3 | 23(3) | 17(3) | 24(3) | -11(3) | -11(3) | 4(3) |
| C11_3 | 31(4) | 15(3) | 27(4) | -13(3) | -6(3) | 7(3) |
| C12_3 | 75(8) | 70(7) | 105(10) | -79(8) | -65(8) | 49(7) |
| C13_3 | 40(5) | 14(3) | 35(4) | -13(3) | -8(4) | 6(3) |
| C14_3 | 75(8) | 33(5) | 66(7) | -26(5) | 43(7) | -11(5) |
| C15_3 | 18(3) | 15(3) | 25(3) | -7(3) | -2(3) | -1(2) |
| C16_3 | 26(4) | 21(3) | 47(5) | -15(3) | 0(4) | 0(3) |
| C17_3 | 24(4) | 31(4) | 44(5) | -12(4) | 3(4) | -4(3) |
| C18_3 | 20(4) | 34(4) | 28(4) | -5(3) | -7(3) | -9(3) |
| C19_3 | 32(4) | 22(4) | 43(5) | -11(3) | -2(4) | -10(3) |
| C20_3 | 22(4) | 15(3) | 37(4) | -11(3) | -1(3) | 0(3) |
| C21_3 | 18(4) | 50(5) | 53(6) | -15(5) | -6(4) | -15(4) |
| C22_3 | 22(3) | 12(3) | 13(3) | -6(2) | -2(2) | 1(2) |
| C23_3 | 41(5) | 25(3) | 16(3) | -10(3) | 5(3) | -7(3) |
| C24_3 | 75(7) | 32(4) | 19(4) | -16(3) | -5(4) | -4(4) |
| C25_3 | 56(6) | 25(4) | 35(5) | -22(4) | -17(4) | 6(4) |
| C26_3 | 98(10) | 43(5) | 43(6) | -31(5) | -31(6) | 2(6) |
| C27_3 | 36(4) | 21(3) | 34(4) | -18(3) | -15(3) | 1(3) |
| C28_3 | 25(4) | 17(3) | 21(3) | -11(3) | -4(3) | -2(3) |
| C29_3 | 28(4) | 30(4) | 39(5) | -16(3) | -15(3) | -4(3) |
| B1_3 | 14(3) | 10(3) | 16(3) | -4(2) | -2(3) | 3(2) |

| | | | | | | |
|---|---|---|---|---|---|---|
| Sb1_4 | 17.0(2) | 15.62(19) | 17.9(2) | -10.16(16) | -4.09(16) | 1.87(15) |
| N1_4 | 16(3) | 12(2) | 17(3) | -7(2) | -3(2) | -2(2) |
| N2_4 | 11(2) | 15(2) | 19(3) | -12(2) | -4(2) | 1.9(19) |
| N3_4 | 16(3) | 10(2) | 18(3) | -5(2) | -1(2) | -1.0(19) |
| N4_4 | 16(3) | 13(2) | 16(3) | -3(2) | 0(2) | -2(2) |
| C1_4 | 12(3) | 10(3) | 24(3) | -10(2) | -2(2) | -1(2) |
| C2_4 | 29(4) | 21(3) | 25(4) | -15(3) | -11(3) | 5(3) |
| C3_4 | 26(4) | 17(3) | 22(3) | -9(3) | -13(3) | 9(3) |
| C4_4 | 29(4) | 19(3) | 30(4) | -19(3) | -6(3) | 9(3) |
| C5_4 | 83(8) | 31(5) | 35(5) | -22(4) | -10(5) | 28(5) |
| C6_4 | 82(9) | 37(5) | 44(6) | -19(5) | 20(6) | 1(5) |
| C7_4 | 54(6) | 47(6) | 55(6) | -39(5) | -6(5) | 3(5) |
| C8_4 | 15(3) | 10(3) | 20(3) | -7(2) | -1(2) | 0(2) |
| C9_4 | 21(3) | 14(3) | 18(3) | -9(2) | -1(3) | -3(2) |
| C10_4 | 19(3) | 11(3) | 22(3) | -6(2) | 0(3) | 0(2) |
| C11_4 | 18(3) | 18(3) | 14(3) | -5(2) | -6(2) | 1(2) |
| C12_4 | 23(4) | 25(3) | 22(3) | -7(3) | -9(3) | -3(3) |
| C13_4 | 29(4) | 17(3) | 25(4) | -7(3) | -8(3) | 7(3) |
| C14_4 | 31(4) | 21(3) | 17(3) | -5(3) | -2(3) | -5(3) |
| C15_4 | 20(3) | 12(3) | 21(3) | -7(2) | 1(3) | -3(2) |
| C16_4 | 18(3) | 12(3) | 21(3) | -4(2) | -2(3) | 1(2) |
| C17_4 | 23(4) | 13(3) | 19(3) | 1(2) | -1(3) | -6(3) |
| C18_4 | 20(4) | 21(3) | 21(3) | -1(3) | -4(3) | -6(3) |
| C19_4 | 21(4) | 21(3) | 42(5) | -8(3) | -1(3) | 1(3) |
| C20_4 | 26(4) | 15(3) | 42(5) | -10(3) | -3(3) | 2(3) |
| C21_4 | 25(4) | 25(4) | 40(5) | -7(3) | -4(3) | -14(3) |
| C22_4 | 20(3) | 14(3) | 13(3) | -7(2) | 1(2) | 1(2) |
| C23_4 | 39(5) | 21(3) | 19(3) | -10(3) | 4(3) | -5(3) |
| C24_4 | 51(5) | 23(4) | 21(4) | -11(3) | -6(3) | 2(3) |
| C25_4 | 49(5) | 22(3) | 29(4) | -18(3) | -13(4) | 5(3) |
| C26_4 | 70(7) | 38(5) | 43(5) | -26(4) | -33(5) | 2(5) |
| C27_4 | 23(4) | 20(3) | 37(4) | -18(3) | -9(3) | -1(3) |
| C28_4 | 20(3) | 18(3) | 16(3) | -6(2) | -2(2) | -3(2) |
| C29_4 | 26(4) | 24(3) | 37(4) | -15(3) | -9(3) | -6(3) |
| B1_4 | 12(3) | 9(3) | 18(3) | -5(2) | -1(3) | 1(2) |
| Sb1_5 | 19.2(2) | 12.46(18) | 16.2(2) | -3.92(15) | 3.96(16) | 0.17(15) |
| N1_5 | 11(2) | 11(2) | 20(3) | -7(2) | 2(2) | 0.2(19) |
| N2_5 | 12(3) | 9(2) | 20(3) | -5(2) | 3(2) | -0.3(19) |
| N3_5 | 15(3) | 12(2) | 17(3) | -8(2) | 0(2) | -1.5(19) |
| N4_5 | 11(3) | 19(3) | 25(3) | -13(2) | 2(2) | 0(2) |
| C1_5 | 8(3) | 15(3) | 21(3) | -12(2) | 1(2) | -3(2) |
| C2_5 | 23(4) | 7(3) | 26(4) | -5(2) | 9(3) | 0(2) |
| C3_5 | 20(3) | 11(3) | 26(4) | -5(3) | 9(3) | 0(2) |
| C4_5 | 23(3) | 10(3) | 18(3) | -3(2) | 3(3) | 4(2) |
| C5_5 | 45(5) | 17(3) | 28(4) | -10(3) | 1(4) | 11(3) |
| C6_5 | 57(6) | 35(5) | 39(5) | -20(4) | -24(5) | 21(4) |
| C7_5 | 44(5) | 17(3) | 41(5) | -8(3) | 11(4) | -6(3) |
| C8_5 | 19(3) | 18(3) | 14(3) | -8(2) | 1(2) | -2(2) |
| C9_5 | 23(3) | 11(3) | 23(3) | -10(2) | -2(3) | 0(2) |
| C10_5 | 21(3) | 18(3) | 23(3) | -12(3) | 4(3) | -2(3) |
| C11_5 | 19(3) | 22(3) | 24(4) | -11(3) | 7(3) | -2(3) |
| C12_5 | 22(4) | 32(4) | 33(4) | -14(3) | 10(3) | -12(3) |
| C13_5 | 35(5) | 25(4) | 32(4) | -14(3) | 16(4) | -2(3) |
| C14_5 | 34(4) | 32(4) | 20(4) | -12(3) | 5(3) | -9(3) |
| C15_5 | 17(3) | 16(3) | 24(3) | -11(3) | -2(3) | 3(2) |
| C16_5 | 20(3) | 21(3) | 22(3) | -13(3) | -1(3) | -1(3) |

| | | | | | | |
|---|---|---|---|---|---|---|
| C17_5 | 27(4) | 28(4) | 26(4) | -15(3) | 1(3) | -10(3) |
| C18_5 | 20(4) | 59(5) | 29(4) | -27(4) | 5(3) | -16(4) |
| C19_5 | 18(4) | 91(9) | 100(10) | -79(8) | -5(5) | 3(5) |
| C20_5 | 32(5) | 66(7) | 94(9) | -69(7) | -23(5) | 21(5) |
| C21_5 | 22(4) | 74(7) | 42(5) | -34(5) | 2(4) | -17(4) |
| C22_5 | 23(3) | 10(3) | 21(3) | -8(2) | -5(3) | 1(2) |
| C23_5 | 56(6) | 17(3) | 21(4) | -4(3) | -7(4) | -1(3) |
| C24_5 | 90(8) | 16(4) | 24(4) | 1(3) | 12(5) | -11(4) |
| C25_5 | 63(7) | 16(3) | 42(5) | -12(3) | 23(5) | -10(4) |
| C26_5 | 112(12) | 29(5) | 78(9) | -21(5) | 61(9) | -35(6) |
| C27_5 | 31(4) | 13(3) | 42(5) | -10(3) | 12(4) | -6(3) |
| C28_5 | 22(3) | 16(3) | 26(4) | -9(3) | 4(3) | -3(3) |
| C29_5 | 27(4) | 26(4) | 33(4) | -13(3) | 9(3) | -12(3) |
| B1_5 | 16(3) | 14(3) | 18(3) | -9(3) | 2(3) | -3(3) |
| Sb1_6 | 25.4(3) | 22.1(2) | 22.6(2) | -16.04(19) | -8.95(18) | 6.84(18) |
| N1_6 | 17(3) | 10(2) | 18(3) | -8(2) | 2(2) | -4.1(19) |
| N2_6 | 17(3) | 12(2) | 17(3) | -9(2) | 3(2) | -2(2) |
| N3_6 | 13(3) | 16(2) | 18(3) | -10(2) | 1(2) | -3(2) |
| N4_6 | 25(3) | 19(3) | 22(3) | -11(2) | -6(2) | 8(2) |
| C1_6 | 10(3) | 15(3) | 18(3) | -10(2) | 1(2) | -3(2) |
| C2_6 | 21(3) | 14(3) | 21(3) | -10(2) | -4(3) | 1(2) |
| C3_6 | 18(3) | 16(3) | 27(4) | -12(3) | -3(3) | 2(2) |
| C4_6 | 34(4) | 15(3) | 18(3) | -11(2) | -1(3) | 6(3) |
| C5_6 | 48(5) | 17(3) | 26(4) | -12(3) | -3(3) | 12(3) |
| C6_6 | 128(11) | 37(5) | 101(11) | -33(6) | 98(9) | -24(7) |
| C7_6 | 93(9) | 124(11) | 169(15) | -138(12) | -95(10) | 74(8) |
| C8_6 | 20(3) | 18(3) | 19(3) | -12(3) | -5(3) | 4(2) |
| C9_6 | 20(3) | 14(3) | 19(3) | -8(2) | 4(3) | -5(2) |
| C10_6 | 18(3) | 18(3) | 24(3) | -10(3) | -1(3) | 4(2) |
| C11_6 | 28(4) | 27(4) | 23(4) | -11(3) | -7(3) | 7(3) |
| C12_6 | 44(5) | 29(4) | 21(4) | -14(3) | -8(3) | 3(3) |
| C13_6 | 26(4) | 36(4) | 31(4) | -9(4) | -11(3) | 10(3) |
| C14_6 | 50(6) | 19(4) | 33(5) | -7(3) | 3(4) | 3(3) |
| C15_6 | 12(3) | 14(3) | 26(3) | -10(2) | 4(2) | -2(2) |
| C16_6 | 20(3) | 19(3) | 31(4) | -13(3) | 10(3) | -10(3) |
| C17_6 | 28(4) | 25(4) | 38(4) | -15(3) | 9(3) | -16(3) |
| C18_6 | 17(4) | 34(4) | 29(4) | -9(3) | 4(3) | -15(3) |
| C19_6 | 20(4) | 28(4) | 64(6) | -16(4) | 12(4) | -8(3) |
| C20_6 | 17(4) | 19(3) | 60(6) | -18(4) | 9(4) | -4(3) |
| C21_6 | 25(4) | 41(5) | 47(5) | -8(4) | -2(4) | -20(4) |
| C22_6 | 16(3) | 13(3) | 18(3) | -9(2) | -1(2) | 1(2) |
| C23_6 | 41(5) | 21(3) | 21(4) | -14(3) | 2(3) | -2(3) |
| C24_6 | 68(7) | 30(4) | 19(4) | -16(3) | -9(4) | 2(4) |
| C25_6 | 47(5) | 26(4) | 46(5) | -28(4) | -23(4) | 14(4) |
| C26_6 | 87(9) | 48(6) | 75(8) | -50(6) | -41(7) | 18(6) |
| C27_6 | 31(4) | 26(4) | 39(5) | -24(3) | -8(3) | 1(3) |
| C28_6 | 24(4) | 20(3) | 26(4) | -15(3) | 0(3) | -4(3) |
| C29_6 | 47(5) | 24(4) | 40(5) | -13(3) | -18(4) | -5(4) |
| B1_6 | 13(3) | 10(3) | 20(3) | -8(3) | 2(3) | 1(2) |
| Cl51_13 | 191(5) | 43.6(16) | 47.6(18) | 1.9(14) | -24(2) | -32(2) |
| Cl52_13 | 134(4) | 115(4) | 63(2) | -45(2) | -11(3) | -22(3) |
| C51_13 | 187(19) | 53(7) | 31(6) | -11(5) | -6(9) | -39(10) |
| Cl51_14 | 70(2) | 60.2(17) | 34.1(13) | -6.9(12) | -1.5(13) | -4.0(14) |
| Cl52_14 | 88(2) | 51.2(15) | 44.1(15) | -3.9(12) | -11.5(15) | -28.2(15) |
| C51_14 | 85(10) | 48(6) | 51(7) | -26(5) | 1(6) | -2(6) |
| Cl51_15 | 66.2(19) | 66.1(17) | 36.6(13) | -22.5(13) | 2.8(12) | -5.0(14) |

| | | | | | | |
|---|---|---|---|---|---|---|
| Cl52_15 | 81(3) | 119(3) | 49.9(19) | -29(2) | 8.0(17) | -41(2) |
| C51_15 | 96(10) | 48(6) | 32(5) | -11(5) | 4(6) | -2(6) |
| Cl51_16 | 102(3) | 93(2) | 41.6(16) | -33.4(16) | 18.3(17) | -47(2) |
| Cl52_16 | 94(3) | 100(3) | 48.4(19) | -18.2(19) | -2.5(18) | -8(2) |
| C51_16 | 129(14) | 50(7) | 25(5) | -3(5) | 7(7) | -9(8) |
| Cl51_17 | 133(4) | 59.5(19) | 44.6(17) | -5.3(14) | -16(2) | -25(2) |
| Cl52_17 | 141(4) | 138(4) | 57(2) | -48(3) | 17(3) | -61(4) |
| C51_17 | 144(15) | 56(7) | 36(6) | -23(6) | 4(8) | -19(8) |
| Cl51_18 | 184(5) | 86(3) | 49.7(19) | -39.8(19) | 24(2) | -57(3) |
| Cl52_18 | 150(5) | 94(3) | 60(2) | 1(2) | 0(3) | -33(3) |
| C51_18 | 143(15) | 57(8) | 32(6) | -7(5) | 4(8) | -32(9) |
| I61_7 | 23.2(2) | 23.3(2) | 34.8(3) | -15.4(2) | -6.3(2) | 2.28(18) |
| I61_8 | 18.6(2) | 14.77(18) | 22.6(2) | -7.81(16) | -1.04(16) | 0.92(15) |
| I61_9 | 16.0(2) | 18.22(19) | 26.3(2) | -12.42(17) | 0.51(16) | -0.78(15) |
| I61_10 | 19.7(2) | 22.0(2) | 28.6(2) | -15.53(18) | -4.71(18) | 3.69(17) |
| I61_11 | 20.9(2) | 22.1(2) | 31.4(3) | -14.95(19) | 8.73(18) | -5.50(17) |
| I61_12 | 27.3(3) | 22.4(2) | 33.3(3) | -14.3(2) | 12.1(2) | -4.85(18) |

### 9.7.9 Bond lengths

Table S45. Bond Lengths in Å for **6**.

| Atom | Atom | Length/Å |
|---|---|---|
| Sb1_1 | C1_1 | 2.141(7) |
| Sb1_1 | C8_1 | 2.156(6) |
| Sb1_1 | C29_1 | 2.167(9) |
| N1_1 | C1_1 | 1.350(8) |
| N1_1 | C2_1 | 1.372(8) |
| N1_1 | B1_1 | 1.580(8) |
| N2_1 | C1_1 | 1.359(8) |
| N2_1 | C3_1 | 1.374(9) |
| N2_1 | C4_1 | 1.504(9) |
| N3_1 | C8_1 | 1.332(9) |
| N3_1 | C9_1 | 1.382(7) |
| N3_1 | B1_1 | 1.580(9) |
| N4_1 | C8_1 | 1.359(8) |
| N4_1 | C10_1 | 1.381(9) |
| N4_1 | C11_1 | 1.517(10) |
| C2_1 | C3_1 | 1.370(9) |
| C4_1 | C5_1 | 1.503(11) |
| C4_1 | C6_1 | 1.463(16) |
| C4_1 | C7_1 | 1.530(15) |
| C9_1 | C10_1 | 1.358(9) |
| C11_1 | C12_1 | 1.536(13) |
| C11_1 | C13_1 | 1.525(11) |
| C11_1 | C14_1 | 1.507(13) |
| C15_1 | C16_1 | 1.404(9) |
| C15_1 | C20_1 | 1.396(10) |
| C15_1 | B1_1 | 1.621(11) |
| C16_1 | C17_1 | 1.394(11) |
| C17_1 | C18_1 | 1.372(10) |
| C18_1 | C19_1 | 1.394(11) |
| C18_1 | C21_1 | 1.515(11) |
| C19_1 | C20_1 | 1.381(12) |
| C22_1 | C23_1 | 1.385(10) |
| C22_1 | C28_1 | 1.390(10) |
| C22_1 | B1_1 | 1.623(9) |
| C23_1 | C24_1 | 1.401(11) |
| C24_1 | C25_1 | 1.382(13) |
| C25_1 | C26_1 | 1.513(11) |
| C25_1 | C27_1 | 1.389(12) |
| C27_1 | C28_1 | 1.403(9) |
| Sb1_2 | C1_2 | 2.167(7) |
| Sb1_2 | C8_2 | 2.162(6) |
| Sb1_2 | C29_2 | 2.175(8) |
| N1_2 | C1_2 | 1.338(8) |
| N1_2 | C2_2 | 1.392(8) |
| N1_2 | B1_2 | 1.585(8) |
| N2_2 | C1_2 | 1.369(7) |
| N2_2 | C3_2 | 1.375(9) |
| N2_2 | C4_2 | 1.502(9) |
| N3_2 | C8_2 | 1.340(8) |
| N3_2 | C9_2 | 1.380(8) |
| N3_2 | B1_2 | 1.587(8) |
| N4_2 | C8_2 | 1.359(8) |
| N4_2 | C10_2 | 1.381(9) |
| N4_2 | C11_2 | 1.502(9) |
| C2_2 | C3_2 | 1.362(9) |
| C4_2 | C5_2 | 1.533(10) |
| C4_2 | C6_2 | 1.514(13) |
| C4_2 | C7_2 | 1.497(11) |
| C9_2 | C10_2 | 1.354(10) |
| C11_2 | C12_2 | 1.531(10) |
| C11_2 | C13_2 | 1.511(11) |
| | | |
| C11_2 | C14_2 | 1.525(11) |
| C15_2 | C16_2 | 1.401(9) |
| C15_2 | C20_2 | 1.408(10) |
| C15_2 | B1_2 | 1.626(10) |
| C16_2 | C17_2 | 1.393(11) |
| C17_2 | C18_2 | 1.393(11) |
| C18_2 | C19_2 | 1.382(11) |
| C18_2 | C21_2 | 1.508(12) |
| C19_2 | C20_2 | 1.387(11) |
| C22_2 | C23_2 | 1.393(10) |
| C22_2 | C28_2 | 1.408(10) |
| C22_2 | B1_2 | 1.613(9) |
| C23_2 | C24_2 | 1.398(11) |
| C24_2 | C25_2 | 1.379(13) |
| C25_2 | C26_2 | 1.512(11) |
| C25_2 | C27_2 | 1.394(12) |
| C27_2 | C28_2 | 1.390(9) |
| Sb1_3 | C1_3 | 2.158(6) |
| Sb1_3 | C8_3 | 2.165(7) |
| Sb1_3 | C29_3 | 2.186(8) |
| N1_3 | C1_3 | 1.346(9) |
| N1_3 | C2_3 | 1.376(8) |
| N1_3 | B1_3 | 1.582(9) |
| N2_3 | C1_3 | 1.353(8) |
| N2_3 | C3_3 | 1.377(9) |
| N2_3 | C4_3 | 1.516(9) |
| N3_3 | C8_3 | 1.345(8) |
| N3_3 | C9_3 | 1.377(8) |
| N3_3 | B1_3 | 1.585(9) |
| N4_3 | C8_3 | 1.360(8) |
| N4_3 | C10_3 | 1.373(9) |
| N4_3 | C11_3 | 1.501(9) |
| C2_3 | C3_3 | 1.350(10) |
| C4_3 | C5_3 | 1.521(12) |
| C4_3 | C6_3 | 1.520(12) |
| C4_3 | C7_3 | 1.516(11) |
| C9_3 | C10_3 | 1.373(9) |
| C11_3 | C12_3 | 1.496(12) |
| C11_3 | C13_3 | 1.518(10) |
| C11_3 | C14_3 | 1.530(14) |
| C15_3 | C16_3 | 1.403(10) |
| C15_3 | C20_3 | 1.404(9) |
| C15_3 | B1_3 | 1.610(10) |

| | | |
|---|---|---|
| C16_3 | C17_3 | 1.376(12) |
| C17_3 | C18_3 | 1.384(12) |
| C18_3 | C19_3 | 1.385(12) |
| C18_3 | C21_3 | 1.516(11) |
| C19_3 | C20_3 | 1.378(11) |
| C22_3 | C23_3 | 1.396(10) |
| C22_3 | C28_3 | 1.400(10) |
| C22_3 | B1_3 | 1.612(10) |
| C23_3 | C24_3 | 1.391(12) |
| C24_3 | C25_3 | 1.372(15) |
| C25_3 | C26_3 | 1.503(12) |
| C25_3 | C27_3 | 1.387(13) |
| C27_3 | C28_3 | 1.390(10) |
| Sb1_4 | C1_4 | 2.154(7) |
| Sb1_4 | C8_4 | 2.154(6) |
| Sb1_4 | C29_4 | 2.158(8) |
| N1_4 | C1_4 | 1.346(8) |
| N1_4 | C2_4 | 1.369(9) |
| N1_4 | B1_4 | 1.589(9) |
| N2_4 | C1_4 | 1.360(8) |
| N2_4 | C3_4 | 1.378(9) |
| N2_4 | C4_4 | 1.498(8) |
| N3_4 | C8_4 | 1.336(9) |
| N3_4 | C9_4 | 1.374(8) |
| N3_4 | B1_4 | 1.575(9) |
| N4_4 | C8_4 | 1.359(8) |
| N4_4 | C10_4 | 1.374(9) |
| N4_4 | C11_4 | 1.496(8) |
| C2_4 | C3_4 | 1.351(9) |
| C4_4 | C5_4 | 1.499(11) |
| C4_4 | C6_4 | 1.460(13) |
| C4_4 | C7_4 | 1.559(14) |
| C9_4 | C10_4 | 1.371(9) |
| C11_4 | C12_4 | 1.539(10) |
| C11_4 | C13_4 | 1.525(9) |
| C11_4 | C14_4 | 1.519(10) |
| C15_4 | C16_4 | 1.399(8) |
| C15_4 | C20_4 | 1.388(10) |
| C15_4 | B1_4 | 1.639(10) |
| C16_4 | C17_4 | 1.395(10) |
| C17_4 | C18_4 | 1.373(10) |
| C18_4 | C19_4 | 1.384(10) |
| C18_4 | C21_4 | 1.510(10) |
| C19_4 | C20_4 | 1.380(12) |
| C22_4 | C23_4 | 1.404(10) |
| C22_4 | C28_4 | 1.400(10) |
| C22_4 | B1_4 | 1.611(10) |
| C23_4 | C24_4 | 1.388(12) |
| C24_4 | C25_4 | 1.376(13) |
| C25_4 | C26_4 | 1.507(11) |
| C25_4 | C27_4 | 1.397(12) |
| C27_4 | C28_4 | 1.388(10) |
| Sb1_5 | C1_5 | 2.160(6) |
| Sb1_5 | C8_5 | 2.153(6) |
| Sb1_5 | C29_5 | 2.159(8) |
| N1_5 | C1_5 | 1.347(8) |
| N1_5 | C2_5 | 1.369(8) |
| N1_5 | B1_5 | 1.583(9) |
| N2_5 | C1_5 | 1.350(7) |
| N2_5 | C3_5 | 1.383(9) |
| N2_5 | C4_5 | 1.495(8) |
| N3_5 | C8_5 | 1.338(8) |
| N3_5 | C9_5 | 1.373(8) |
| N3_5 | B1_5 | 1.589(9) |
| N4_5 | C8_5 | 1.372(8) |
| N4_5 | C10_5 | 1.386(9) |
| N4_5 | C11_5 | 1.490(9) |
| C2_5 | C3_5 | 1.366(8) |
| C4_5 | C5_5 | 1.518(10) |
| C4_5 | C6_5 | 1.498(12) |
| C4_5 | C7_5 | 1.551(12) |
| C9_5 | C10_5 | 1.358(10) |
| C11_5 | C12_5 | 1.539(11) |
| C11_5 | C13_5 | 1.524(10) |
| C11_5 | C14_5 | 1.514(11) |
| C15_5 | C16_5 | 1.392(9) |
| C15_5 | C20_5 | 1.383(11) |
| C15_5 | B1_5 | 1.609(11) |
| C16_5 | C17_5 | 1.397(10) |
| C17_5 | C18_5 | 1.383(11) |
| C18_5 | C19_5 | 1.387(13) |
| C18_5 | C21_5 | 1.506(12) |
| C19_5 | C20_5 | 1.391(14) |
| C22_5 | C23_5 | 1.397(10) |
| C22_5 | C28_5 | 1.412(10) |
| C22_5 | B1_5 | 1.603(10) |
| C23_5 | C24_5 | 1.374(13) |
| C24_5 | C25_5 | 1.395(16) |
| C25_5 | C26_5 | 1.508(13) |
| C25_5 | C27_5 | 1.391(14) |
| C27_5 | C28_5 | 1.394(10) |
| Sb1_6 | C1_6 | 2.171(7) |
| Sb1_6 | C8_6 | 2.159(6) |
| Sb1_6 | C29_6 | 2.138(10) |
| N1_6 | C1_6 | 1.340(8) |
| N1_6 | C2_6 | 1.379(8) |
| N1_6 | B1_6 | 1.577(8) |
| N2_6 | C1_6 | 1.375(8) |
| N2_6 | C3_6 | 1.361(9) |
| N2_6 | C4_6 | 1.498(8) |
| N3_6 | C8_6 | 1.340(9) |
| N3_6 | C9_6 | 1.385(8) |
| N3_6 | B1_6 | 1.589(9) |
| N4_6 | C8_6 | 1.359(8) |
| N4_6 | C10_6 | 1.370(9) |
| N4_6 | C11_6 | 1.511(10) |
| C2_6 | C3_6 | 1.367(9) |
| C4_6 | C5_6 | 1.514(9) |
| C4_6 | C6_6 | 1.491(14) |
| C4_6 | C7_6 | 1.500(14) |
| C9_6 | C10_6 | 1.364(10) |
| C11_6 | C12_6 | 1.506(10) |

| | | |
|---|---|---|
| C11_6 | C13_6 | 1.528(11) |
| C11_6 | C14_6 | 1.530(13) |
| C15_6 | C16_6 | 1.401(9) |
| C15_6 | C20_6 | 1.399(9) |
| C15_6 | B1_6 | 1.623(10) |
| C16_6 | C17_6 | 1.385(10) |
| C17_6 | C18_6 | 1.386(11) |
| C18_6 | C19_6 | 1.374(12) |
| C18_6 | C21_6 | 1.517(11) |
| C19_6 | C20_6 | 1.389(11) |
| C22_6 | C23_6 | 1.377(10) |
| C22_6 | C28_6 | 1.407(10) |
| C22_6 | B1_6 | 1.603(10) |
| C23_6 | C24_6 | 1.401(12) |
| C24_6 | C25_6 | 1.398(15) |

| | | |
|---|---|---|
| C25_6 | C26_6 | 1.531(13) |
| C25_6 | C27_6 | 1.377(13) |
| C27_6 | C28_6 | 1.376(10) |
| Cl51_13 | C51_13 | 1.773(15) |
| Cl52_13 | C51_13 | 1.724(19) |
| Cl51_14 | C51_14 | 1.775(14) |
| Cl52_14 | C51_14 | 1.770(14) |
| Cl51_15 | C51_15 | 1.787(15) |
| Cl52_15 | C51_15 | 1.726(15) |
| Cl51_16 | C51_16 | 1.736(16) |
| Cl52_16 | C51_16 | 1.832(16) |
| Cl51_17 | C51_17 | 1.750(14) |
| Cl52_17 | C51_17 | 1.771(18) |
| Cl51_18 | C51_18 | 1.730(15) |
| Cl52_18 | C51_18 | 1.775(18) |

9.7.10 Bond angles

Table S46. Bond Angles in ° for **6**.

| Atom | Atom | Atom | Angle/° |
|---|---|---|---|
| C1_1 | Sb1_1 | C8_1 | 90.4(2) |
| C1_1 | Sb1_1 | C29_1 | 93.3(3) |
| C8_1 | Sb1_1 | C29_1 | 97.6(3) |
| C1_1 | N1_1 | C2_1 | 108.5(5) |
| C1_1 | N1_1 | B1_1 | 128.4(5) |
| C2_1 | N1_1 | B1_1 | 122.3(5) |
| C1_1 | N2_1 | C3_1 | 109.2(6) |
| C1_1 | N2_1 | C4_1 | 128.1(6) |
| C3_1 | N2_1 | C4_1 | 122.7(6) |
| C8_1 | N3_1 | C9_1 | 108.1(5) |
| C8_1 | N3_1 | B1_1 | 127.1(5) |
| C9_1 | N3_1 | B1_1 | 123.0(5) |
| C8_1 | N4_1 | C10_1 | 107.7(6) |
| C8_1 | N4_1 | C11_1 | 126.4(6) |
| C10_1 | N4_1 | C11_1 | 125.8(6) |
| N1_1 | C1_1 | Sb1_1 | 123.7(4) |
| N1_1 | C1_1 | N2_1 | 107.7(6) |
| N2_1 | C1_1 | Sb1_1 | 128.4(5) |
| C3_1 | C2_1 | N1_1 | 108.2(6) |
| C2_1 | C3_1 | N2_1 | 106.4(6) |
| N2_1 | C4_1 | C7_1 | 107.2(8) |
| C5_1 | C4_1 | N2_1 | 109.6(7) |
| C5_1 | C4_1 | C7_1 | 101.6(11) |
| C6_1 | C4_1 | N2_1 | 109.4(7) |
| C6_1 | C4_1 | C5_1 | 112.9(10) |
| C6_1 | C4_1 | C7_1 | 115.7(14) |
| N3_1 | C8_1 | Sb1_1 | 124.1(5) |
| N3_1 | C8_1 | N4_1 | 109.1(6) |
| N4_1 | C8_1 | Sb1_1 | 126.4(5) |
| C10_1 | C9_1 | N3_1 | 107.9(6) |
| C9_1 | C10_1 | N4_1 | 107.2(6) |
| N4_1 | C11_1 | C12_1 | 108.7(7) |
| N4_1 | C11_1 | C13_1 | 108.6(6) |
| C13_1 | C11_1 | C12_1 | 108.1(8) |
| C14_1 | C11_1 | N4_1 | 110.0(7) |
| C14_1 | C11_1 | C12_1 | 111.3(7) |
| C14_1 | C11_1 | C13_1 | 110.0(8) |
| C16_1 | C15_1 | B1_1 | 122.7(6) |
| C20_1 | C15_1 | C16_1 | 115.8(7) |
| C20_1 | C15_1 | B1_1 | 121.5(6) |
| C17_1 | C16_1 | C15_1 | 122.0(6) |
| C18_1 | C17_1 | C16_1 | 120.8(7) |
| C17_1 | C18_1 | C19_1 | 118.4(8) |
| C17_1 | C18_1 | C21_1 | 121.0(7) |
| C19_1 | C18_1 | C21_1 | 120.6(7) |
| C20_1 | C19_1 | C18_1 | 120.6(7) |
| C19_1 | C20_1 | C15_1 | 122.4(7) |
| C23_1 | C22_1 | C28_1 | 116.9(7) |
| C23_1 | C22_1 | B1_1 | 122.8(7) |
| C28_1 | C22_1 | B1_1 | 120.1(6) |
| C22_1 | C23_1 | C24_1 | 121.8(8) |
| C25_1 | C24_1 | C23_1 | 120.9(7) |
| C24_1 | C25_1 | C26_1 | 122.3(8) |
| C24_1 | C25_1 | C27_1 | 118.1(7) |
| C27_1 | C25_1 | C26_1 | 119.6(8) |
| C25_1 | C27_1 | C28_1 | 120.5(8) |
| C22_1 | C28_1 | C27_1 | 121.8(7) |
| N1_1 | B1_1 | C15_1 | 109.4(5) |
| N1_1 | B1_1 | C22_1 | 106.6(5) |
| N3_1 | B1_1 | N1_1 | 108.4(5) |
| N3_1 | B1_1 | C15_1 | 106.9(5) |
| N3_1 | B1_1 | C22_1 | 110.2(5) |
| C15_1 | B1_1 | C22_1 | 115.3(6) |
| C1_2 | Sb1_2 | C29_2 | 94.7(3) |
| C8_2 | Sb1_2 | C1_2 | 91.3(2) |
| C8_2 | Sb1_2 | C29_2 | 92.5(3) |
| C1_2 | N1_2 | C2_2 | 109.0(5) |
| C1_2 | N1_2 | B1_2 | 130.2(5) |
| C2_2 | N1_2 | B1_2 | 119.9(5) |
| C1_2 | N2_2 | C3_2 | 108.3(5) |
| C1_2 | N2_2 | C4_2 | 127.2(6) |
| C3_2 | N2_2 | C4_2 | 124.2(5) |
| C8_2 | N3_2 | C9_2 | 108.2(5) |
| C8_2 | N3_2 | B1_2 | 132.6(5) |
| C9_2 | N3_2 | B1_2 | 118.8(5) |
| C8_2 | N4_2 | C10_2 | 108.3(6) |
| C8_2 | N4_2 | C11_2 | 131.9(6) |
| C10_2 | N4_2 | C11_2 | 119.8(5) |
| N1_2 | C1_2 | Sb1_2 | 124.0(4) |
| N1_2 | C1_2 | N2_2 | 107.9(6) |
| N2_2 | C1_2 | Sb1_2 | 127.6(5) |
| C3_2 | C2_2 | N1_2 | 106.9(6) |
| C2_2 | C3_2 | N2_2 | 107.9(6) |
| N2_2 | C4_2 | C5_2 | 108.2(6) |
| N2_2 | C4_2 | C6_2 | 107.3(6) |
| C6_2 | C4_2 | C5_2 | 110.5(7) |
| C7_2 | C4_2 | N2_2 | 111.3(6) |
| C7_2 | C4_2 | C5_2 | 107.9(7) |
| C7_2 | C4_2 | C6_2 | 111.6(9) |
| N3_2 | C8_2 | Sb1_2 | 122.7(4) |
| N3_2 | C8_2 | N4_2 | 108.4(5) |
| N4_2 | C8_2 | Sb1_2 | 128.9(5) |
| C10_2 | C9_2 | N3_2 | 108.1(6) |
| C9_2 | C10_2 | N4_2 | 107.0(6) |
| N4_2 | C11_2 | C12_2 | 112.0(6) |
| N4_2 | C11_2 | C13_2 | 107.5(7) |
| N4_2 | C11_2 | C14_2 | 107.3(6) |
| C13_2 | C11_2 | C12_2 | 110.3(7) |
| C13_2 | C11_2 | C14_2 | 110.7(7) |
| C14_2 | C11_2 | C12_2 | 109.0(7) |
| C16_2 | C15_2 | C20_2 | 115.8(6) |
| C16_2 | C15_2 | B1_2 | 123.3(6) |
| C20_2 | C15_2 | B1_2 | 120.9(6) |
| C17_2 | C16_2 | C15_2 | 121.8(7) |
| C16_2 | C17_2 | C18_2 | 121.1(7) |

| | | | |
|---|---|---|---|
| C17_2 | C18_2 | C21_2 | 120.8(8) |
| C19_2 | C18_2 | C17_2 | 117.9(7) |
| C19_2 | C18_2 | C21_2 | 121.3(8) |
| C18_2 | C19_2 | C20_2 | 121.1(8) |
| C19_2 | C20_2 | C15_2 | 122.2(7) |
| C23_2 | C22_2 | C28_2 | 116.2(6) |
| C23_2 | C22_2 | B1_2 | 124.3(6) |
| C28_2 | C22_2 | B1_2 | 119.4(6) |
| C22_2 | C23_2 | C24_2 | 122.0(8) |
| C25_2 | C24_2 | C23_2 | 120.5(8) |
| C24_2 | C25_2 | C26_2 | 122.1(9) |
| C24_2 | C25_2 | C27_2 | 119.1(7) |
| C27_2 | C25_2 | C26_2 | 118.7(9) |
| C28_2 | C27_2 | C25_2 | 119.8(8) |
| C27_2 | C28_2 | C22_2 | 122.4(7) |
| N1_2 | B1_2 | N3_2 | 109.8(5) |
| N1_2 | B1_2 | C15_2 | 108.0(5) |
| N1_2 | B1_2 | C22_2 | 107.1(5) |
| N3_2 | B1_2 | C15_2 | 107.7(5) |
| N3_2 | B1_2 | C22_2 | 107.8(5) |
| C22_2 | B1_2 | C15_2 | 116.4(6) |
| C1_3 | Sb1_3 | C8_3 | 91.4(2) |
| C1_3 | Sb1_3 | C29_3 | 92.7(3) |
| C8_3 | Sb1_3 | C29_3 | 95.0(3) |
| C1_3 | N1_3 | C2_3 | 107.9(6) |
| C1_3 | N1_3 | B1_3 | 131.5(5) |
| C2_3 | N1_3 | B1_3 | 119.9(5) |
| C1_3 | N2_3 | C3_3 | 108.6(6) |
| C1_3 | N2_3 | C4_3 | 131.6(6) |
| C3_3 | N2_3 | C4_3 | 119.8(5) |
| C8_3 | N3_3 | C9_3 | 108.7(5) |
| C8_3 | N3_3 | B1_3 | 129.7(6) |
| C9_3 | N3_3 | B1_3 | 120.5(6) |
| C8_3 | N4_3 | C10_3 | 108.1(6) |
| C8_3 | N4_3 | C11_3 | 128.8(6) |
| C10_3 | N4_3 | C11_3 | 122.8(6) |
| N1_3 | C1_3 | Sb1_3 | 122.8(5) |
| N1_3 | C1_3 | N2_3 | 108.1(5) |
| N2_3 | C1_3 | Sb1_3 | 129.0(5) |
| C3_3 | C2_3 | N1_3 | 108.4(6) |
| C2_3 | C3_3 | N2_3 | 106.9(6) |
| N2_3 | C4_3 | C5_3 | 106.8(7) |
| N2_3 | C4_3 | C6_3 | 107.8(7) |
| C6_3 | C4_3 | C5_3 | 110.6(7) |
| C7_3 | C4_3 | N2_3 | 112.4(6) |
| C7_3 | C4_3 | C5_3 | 111.4(7) |
| C7_3 | C4_3 | C6_3 | 107.8(7) |
| N3_3 | C8_3 | Sb1_3 | 123.3(4) |
| N3_3 | C8_3 | N4_3 | 108.4(6) |
| N4_3 | C8_3 | Sb1_3 | 127.6(5) |
| C10_3 | C9_3 | N3_3 | 107.1(6) |
| N4_3 | C10_3 | C9_3 | 107.6(6) |
| N4_3 | C11_3 | C13_3 | 109.0(6) |
| N4_3 | C11_3 | C14_3 | 107.5(7) |
| C12_3 | C11_3 | N4_3 | 110.6(7) |
| C12_3 | C11_3 | C13_3 | 109.1(8) |

| | | | |
|---|---|---|---|
| C12_3 | C11_3 | C14_3 | 110.3(10) |
| C13_3 | C11_3 | C14_3 | 110.3(7) |
| C16_3 | C15_3 | C20_3 | 114.5(7) |
| C16_3 | C15_3 | B1_3 | 121.4(6) |
| C20_3 | C15_3 | B1_3 | 124.1(6) |
| C17_3 | C16_3 | C15_3 | 122.9(7) |
| C16_3 | C17_3 | C18_3 | 121.1(8) |
| C17_3 | C18_3 | C19_3 | 117.6(8) |
| C17_3 | C18_3 | C21_3 | 121.4(8) |
| C19_3 | C18_3 | C21_3 | 121.0(8) |
| C20_3 | C19_3 | C18_3 | 121.0(7) |
| C19_3 | C20_3 | C15_3 | 122.9(7) |
| C23_3 | C22_3 | C28_3 | 115.8(7) |
| C23_3 | C22_3 | B1_3 | 123.6(7) |
| C28_3 | C22_3 | B1_3 | 120.5(6) |
| C24_3 | C23_3 | C22_3 | 121.6(8) |
| C25_3 | C24_3 | C23_3 | 122.0(8) |
| C24_3 | C25_3 | C26_3 | 122.6(10) |
| C24_3 | C25_3 | C27_3 | 117.3(8) |
| C27_3 | C25_3 | C26_3 | 120.1(10) |
| C25_3 | C27_3 | C28_3 | 121.2(8) |
| C27_3 | C28_3 | C22_3 | 122.0(7) |
| N1_3 | B1_3 | N3_3 | 109.3(5) |
| N1_3 | B1_3 | C15_3 | 108.3(5) |
| N1_3 | B1_3 | C22_3 | 107.8(5) |
| N3_3 | B1_3 | C15_3 | 109.1(5) |
| N3_3 | B1_3 | C22_3 | 106.7(5) |
| C15_3 | B1_3 | C22_3 | 115.5(6) |
| C1_4 | Sb1_4 | C29_4 | 94.6(3) |
| C8_4 | Sb1_4 | C1_4 | 91.2(2) |
| C8_4 | Sb1_4 | C29_4 | 97.1(3) |
| C1_4 | N1_4 | C2_4 | 107.8(5) |
| C1_4 | N1_4 | B1_4 | 127.8(5) |
| C2_4 | N1_4 | B1_4 | 123.8(6) |
| C1_4 | N2_4 | C3_4 | 107.9(5) |
| C1_4 | N2_4 | C4_4 | 128.2(6) |
| C3_4 | N2_4 | C4_4 | 124.0(5) |
| C8_4 | N3_4 | C9_4 | 108.3(5) |
| C8_4 | N3_4 | B1_4 | 126.6(5) |
| C9_4 | N3_4 | B1_4 | 123.2(5) |
| C8_4 | N4_4 | C10_4 | 107.8(6) |
| C8_4 | N4_4 | C11_4 | 127.1(6) |
| C10_4 | N4_4 | C11_4 | 125.1(5) |
| N1_4 | C1_4 | Sb1_4 | 123.3(4) |
| N1_4 | C1_4 | N2_4 | 108.5(6) |
| N2_4 | C1_4 | Sb1_4 | 127.9(5) |
| C3_4 | C2_4 | N1_4 | 108.7(6) |
| C2_4 | C3_4 | N2_4 | 107.1(6) |
| N2_4 | C4_4 | C5_4 | 109.8(6) |
| N2_4 | C4_4 | C7_4 | 104.8(6) |
| C5_4 | C4_4 | C7_4 | 101.8(8) |
| C6_4 | C4_4 | N2_4 | 111.9(7) |
| C6_4 | C4_4 | C5_4 | 114.4(8) |
| C6_4 | C4_4 | C7_4 | 113.3(9) |
| N3_4 | C8_4 | Sb1_4 | 123.7(4) |
| N3_4 | C8_4 | N4_4 | 109.0(5) |

| | | | |
|---|---|---|---|
| N4_4 | C8_4 | Sb1_4 | 126.5(5) |
| C10_4 | C9_4 | N3_4 | 107.6(6) |
| C9_4 | C10_4 | N4_4 | 107.3(5) |
| N4_4 | C11_4 | C12_4 | 108.6(5) |
| N4_4 | C11_4 | C13_4 | 108.3(6) |
| N4_4 | C11_4 | C14_4 | 109.9(6) |
| C13_4 | C11_4 | C12_4 | 108.3(6) |
| C14_4 | C11_4 | C12_4 | 111.4(6) |
| C14_4 | C11_4 | C13_4 | 110.3(6) |
| C16_4 | C15_4 | B1_4 | 122.9(6) |
| C20_4 | C15_4 | C16_4 | 115.8(7) |
| C20_4 | C15_4 | B1_4 | 121.1(6) |
| C17_4 | C16_4 | C15_4 | 122.2(6) |
| C18_4 | C17_4 | C16_4 | 120.5(6) |
| C17_4 | C18_4 | C19_4 | 118.1(7) |
| C17_4 | C18_4 | C21_4 | 120.4(7) |
| C19_4 | C18_4 | C21_4 | 121.5(7) |
| C20_4 | C19_4 | C18_4 | 121.4(7) |
| C19_4 | C20_4 | C15_4 | 122.0(7) |
| C23_4 | C22_4 | B1_4 | 122.2(6) |
| C28_4 | C22_4 | C23_4 | 116.6(7) |
| C28_4 | C22_4 | B1_4 | 120.8(6) |
| C24_4 | C23_4 | C22_4 | 120.9(8) |
| C25_4 | C24_4 | C23_4 | 122.2(8) |
| C24_4 | C25_4 | C26_4 | 122.9(9) |
| C24_4 | C25_4 | C27_4 | 117.6(7) |
| C27_4 | C25_4 | C26_4 | 119.5(9) |
| C28_4 | C27_4 | C25_4 | 120.8(7) |
| C27_4 | C28_4 | C22_4 | 122.0(7) |
| N1_4 | B1_4 | C15_4 | 108.8(5) |
| N1_4 | B1_4 | C22_4 | 106.0(5) |
| N3_4 | B1_4 | N1_4 | 109.5(5) |
| N3_4 | B1_4 | C15_4 | 106.5(5) |
| N3_4 | B1_4 | C22_4 | 111.0(5) |
| C22_4 | B1_4 | C15_4 | 115.0(6) |
| C8_5 | Sb1_5 | C1_5 | 90.9(2) |
| C8_5 | Sb1_5 | C29_5 | 97.6(3) |
| C29_5 | Sb1_5 | C1_5 | 94.7(3) |
| C1_5 | N1_5 | C2_5 | 107.4(5) |
| C1_5 | N1_5 | B1_5 | 128.9(5) |
| C2_5 | N1_5 | B1_5 | 122.7(5) |
| C1_5 | N2_5 | C3_5 | 108.6(5) |
| C1_5 | N2_5 | C4_5 | 128.0(6) |
| C3_5 | N2_5 | C4_5 | 123.4(5) |
| C8_5 | N3_5 | C9_5 | 108.5(6) |
| C8_5 | N3_5 | B1_5 | 127.0(5) |
| C9_5 | N3_5 | B1_5 | 122.8(5) |
| C8_5 | N4_5 | C10_5 | 106.8(6) |
| C8_5 | N4_5 | C11_5 | 127.0(6) |
| C10_5 | N4_5 | C11_5 | 126.1(6) |
| N1_5 | C1_5 | Sb1_5 | 123.4(4) |
| N1_5 | C1_5 | N2_5 | 108.9(5) |
| N2_5 | C1_5 | Sb1_5 | 127.7(5) |
| C3_5 | C2_5 | N1_5 | 109.1(6) |
| C2_5 | C3_5 | N2_5 | 106.0(6) |
| N2_5 | C4_5 | C5_5 | 109.9(6) |

| | | | |
|---|---|---|---|
| N2_5 | C4_5 | C6_5 | 109.8(6) |
| N2_5 | C4_5 | C7_5 | 107.4(6) |
| C5_5 | C4_5 | C7_5 | 105.5(7) |
| C6_5 | C4_5 | C5_5 | 111.8(7) |
| C6_5 | C4_5 | C7_5 | 112.3(8) |
| N3_5 | C8_5 | Sb1_5 | 124.1(5) |
| N3_5 | C8_5 | N4_5 | 108.9(6) |
| N4_5 | C8_5 | Sb1_5 | 126.4(5) |
| C10_5 | C9_5 | N3_5 | 107.9(6) |
| C9_5 | C10_5 | N4_5 | 107.9(6) |
| N4_5 | C11_5 | C12_5 | 109.6(6) |
| N4_5 | C11_5 | C13_5 | 108.8(6) |
| N4_5 | C11_5 | C14_5 | 109.7(6) |
| C13_5 | C11_5 | C12_5 | 107.9(6) |
| C14_5 | C11_5 | C12_5 | 110.8(6) |
| C14_5 | C11_5 | C13_5 | 110.0(7) |
| C16_5 | C15_5 | B1_5 | 123.4(6) |
| C20_5 | C15_5 | C16_5 | 115.3(7) |
| C20_5 | C15_5 | B1_5 | 121.2(6) |
| C15_5 | C16_5 | C17_5 | 122.8(7) |
| C18_5 | C17_5 | C16_5 | 120.3(7) |
| C17_5 | C18_5 | C19_5 | 118.0(8) |
| C17_5 | C18_5 | C21_5 | 120.6(8) |
| C19_5 | C18_5 | C21_5 | 121.4(8) |
| C18_5 | C19_5 | C20_5 | 120.4(8) |
| C15_5 | C20_5 | C19_5 | 123.2(8) |
| C23_5 | C22_5 | C28_5 | 115.4(7) |
| C23_5 | C22_5 | B1_5 | 123.6(7) |
| C28_5 | C22_5 | B1_5 | 120.6(6) |
| C24_5 | C23_5 | C22_5 | 123.1(9) |
| C23_5 | C24_5 | C25_5 | 120.7(9) |
| C24_5 | C25_5 | C26_5 | 122.0(11) |
| C27_5 | C25_5 | C24_5 | 118.3(8) |
| C27_5 | C25_5 | C26_5 | 119.6(11) |
| C25_5 | C27_5 | C28_5 | 120.2(9) |
| C27_5 | C28_5 | C22_5 | 122.3(8) |
| N1_5 | B1_5 | N3_5 | 109.2(5) |
| N1_5 | B1_5 | C15_5 | 108.7(5) |
| N1_5 | B1_5 | C22_5 | 106.0(5) |
| N3_5 | B1_5 | C15_5 | 106.8(5) |
| N3_5 | B1_5 | C22_5 | 109.4(5) |
| C22_5 | B1_5 | C15_5 | 116.6(6) |
| C8_6 | Sb1_6 | C1_6 | 91.8(2) |
| C29_6 | Sb1_6 | C1_6 | 95.7(3) |
| C29_6 | Sb1_6 | C8_6 | 92.5(3) |
| C1_6 | N1_6 | C2_6 | 107.9(5) |
| C1_6 | N1_6 | B1_6 | 130.4(6) |
| C2_6 | N1_6 | B1_6 | 120.9(5) |
| C1_6 | N2_6 | C4_6 | 127.3(6) |
| C3_6 | N2_6 | C1_6 | 108.6(5) |
| C3_6 | N2_6 | C4_6 | 123.9(6) |
| C8_6 | N3_6 | C9_6 | 107.2(6) |
| C8_6 | N3_6 | B1_6 | 132.6(5) |
| C9_6 | N3_6 | B1_6 | 119.7(5) |
| C8_6 | N4_6 | C10_6 | 108.7(6) |
| C8_6 | N4_6 | C11_6 | 132.2(6) |

| | | | |
|---|---|---|---|
| C10_6 | N4_6 | C11_6 | 119.0(6) |
| N1_6 | C1_6 | Sb1_6 | 123.3(4) |
| N1_6 | C1_6 | N2_6 | 108.2(6) |
| N2_6 | C1_6 | Sb1_6 | 127.9(5) |
| C3_6 | C2_6 | N1_6 | 108.3(6) |
| N2_6 | C3_6 | C2_6 | 107.0(6) |
| N2_6 | C4_6 | C5_6 | 108.9(6) |
| N2_6 | C4_6 | C7_6 | 109.2(7) |
| C6_6 | C4_6 | N2_6 | 107.9(7) |
| C6_6 | C4_6 | C5_6 | 111.3(8) |
| C6_6 | C4_6 | C7_6 | 112.4(13) |
| C7_6 | C4_6 | C5_6 | 107.1(9) |
| N3_6 | C8_6 | Sb1_6 | 122.2(5) |
| N3_6 | C8_6 | N4_6 | 108.9(6) |
| N4_6 | C8_6 | Sb1_6 | 128.8(5) |
| C10_6 | C9_6 | N3_6 | 108.7(6) |
| C9_6 | C10_6 | N4_6 | 106.5(6) |
| N4_6 | C11_6 | C13_6 | 106.5(7) |
| N4_6 | C11_6 | C14_6 | 107.9(7) |
| C12_6 | C11_6 | N4_6 | 112.7(6) |
| C12_6 | C11_6 | C13_6 | 111.2(7) |
| C12_6 | C11_6 | C14_6 | 108.1(7) |
| C13_6 | C11_6 | C14_6 | 110.4(7) |
| C16_6 | C15_6 | B1_6 | 123.9(6) |
| C20_6 | C15_6 | C16_6 | 115.2(6) |
| C20_6 | C15_6 | B1_6 | 120.8(6) |
| C17_6 | C16_6 | C15_6 | 122.9(7) |
| C16_6 | C17_6 | C18_6 | 120.6(7) |
| C17_6 | C18_6 | C21_6 | 120.4(8) |
| C19_6 | C18_6 | C17_6 | 117.6(7) |
| C19_6 | C18_6 | C21_6 | 121.9(8) |

| | | | |
|---|---|---|---|
| C18_6 | C19_6 | C20_6 | 121.9(8) |
| C19_6 | C20_6 | C15_6 | 121.8(7) |
| C23_6 | C22_6 | C28_6 | 116.5(7) |
| C23_6 | C22_6 | B1_6 | 124.0(6) |
| C28_6 | C22_6 | B1_6 | 119.4(6) |
| C22_6 | C23_6 | C24_6 | 122.6(8) |
| C25_6 | C24_6 | C23_6 | 119.0(8) |
| C24_6 | C25_6 | C26_6 | 120.4(10) |
| C27_6 | C25_6 | C24_6 | 119.2(8) |
| C27_6 | C25_6 | C26_6 | 120.5(10) |
| C28_6 | C27_6 | C25_6 | 120.7(8) |
| C27_6 | C28_6 | C22_6 | 122.0(7) |
| N1_6 | B1_6 | N3_6 | 110.0(5) |
| N1_6 | B1_6 | C15_6 | 108.0(5) |
| N1_6 | B1_6 | C22_6 | 106.9(5) |
| N3_6 | B1_6 | C15_6 | 107.5(5) |
| N3_6 | B1_6 | C22_6 | 107.5(5) |
| C22_6 | B1_6 | C15_6 | 116.8(6) |
| Cl52_13 | C51_13 | Cl51_13 | 114.3(9) |
| Cl52_14 | C51_14 | Cl51_14 | 109.8(6) |
| Cl52_15 | C51_15 | Cl51_15 | 112.1(7) |
| Cl51_16 | C51_16 | Cl52_16 | 107.8(7) |
| Cl51_17 | C51_17 | Cl52_17 | 110.8(8) |
| Cl51_18 | C51_18 | Cl52_18 | 114.6(9) |

9.7.11 Torsion angles

Table S47. Torsion Angles in ° for **6**.

| Atom | Atom | Atom | Atom | Angle/° |
|---|---|---|---|---|
| N1_1 | C2_1 | C3_1 | N2_1 | 0.0(9) |
| N3_1 | C9_1 | C10_1 | N4_1 | 0.7(9) |
| C1_1 | N1_1 | C2_1 | C3_1 | 0.6(9) |
| C1_1 | N1_1 | B1_1 | N3_1 | 45.1(9) |
| C1_1 | N1_1 | B1_1 | C15_1 | -71.1(8) |
| C1_1 | N1_1 | B1_1 | C22_1 | 163.7(6) |
| C1_1 | N2_1 | C3_1 | C2_1 | -0.6(9) |
| C1_1 | N2_1 | C4_1 | C5_1 | 156.4(9) |
| C1_1 | N2_1 | C4_1 | C6_1 | -79.4(12) |
| C1_1 | N2_1 | C4_1 | C7_1 | 46.9(14) |
| C2_1 | N1_1 | C1_1 | Sb1_1 | 174.0(5) |
| C2_1 | N1_1 | C1_1 | N2_1 | -0.9(8) |
| C2_1 | N1_1 | B1_1 | N3_1 | -146.6(6) |
| C2_1 | N1_1 | B1_1 | C15_1 | 97.2(7) |
| C2_1 | N1_1 | B1_1 | C22_1 | -28.0(8) |
| C3_1 | N2_1 | C1_1 | Sb1_1 | -173.7(5) |
| C3_1 | N2_1 | C1_1 | N1_1 | 0.9(8) |
| C3_1 | N2_1 | C4_1 | C5_1 | -26.0(12) |
| C3_1 | N2_1 | C4_1 | C6_1 | 98.3(12) |
| C3_1 | N2_1 | C4_1 | C7_1 | -135.5(12) |
| C4_1 | N2_1 | C1_1 | Sb1_1 | 4.2(11) |
| C4_1 | N2_1 | C1_1 | N1_1 | 178.9(7) |
| C4_1 | N2_1 | C3_1 | C2_1 | -178.6(7) |
| C8_1 | N3_1 | C9_1 | C10_1 | -1.4(9) |
| C8_1 | N3_1 | B1_1 | N1_1 | -48.8(9) |
| C8_1 | N3_1 | B1_1 | C15_1 | 68.9(8) |
| C8_1 | N3_1 | B1_1 | C22_1 | -165.1(7) |
| C8_1 | N4_1 | C10_1 | C9_1 | 0.3(9) |
| C8_1 | N4_1 | C11_1 | C12_1 | -48.4(11) |
| C8_1 | N4_1 | C11_1 | C13_1 | -165.8(8) |
| C8_1 | N4_1 | C11_1 | C14_1 | 73.7(11) |
| C9_1 | N3_1 | C8_1 | Sb1_1 | -171.8(5) |
| C9_1 | N3_1 | C8_1 | N4_1 | 1.6(9) |
| C9_1 | N3_1 | B1_1 | N1_1 | 148.3(6) |
| C9_1 | N3_1 | B1_1 | C15_1 | -93.9(7) |
| C9_1 | N3_1 | B1_1 | C22_1 | 32.1(8) |
| C10_1 | N4_1 | C8_1 | Sb1_1 | 172.0(6) |
| C10_1 | N4_1 | C8_1 | N3_1 | -1.2(9) |
| C10_1 | N4_1 | C11_1 | C12_1 | 127.1(8) |
| C10_1 | N4_1 | C11_1 | C13_1 | 9.6(12) |
| C10_1 | N4_1 | C11_1 | C14_1 | -110.8(9) |
| C11_1 | N4_1 | C8_1 | Sb1_1 | -11.9(12) |
| C11_1 | N4_1 | C8_1 | N3_1 | 174.9(7) |
| C11_1 | N4_1 | C10_1 | C9_1 | -175.9(8) |
| C15_1 | C16_1 | C17_1 | C18_1 | 0.4(13) |
| C16_1 | C15_1 | C20_1 | C19_1 | 1.0(13) |
| C16_1 | C15_1 | B1_1 | N1_1 | -25.4(9) |
| C16_1 | C15_1 | B1_1 | N3_1 | -142.5(7) |
| C16_1 | C15_1 | B1_1 | C22_1 | 94.6(8) |
| C16_1 | C17_1 | C18_1 | C19_1 | 0.1(13) |
| C16_1 | C17_1 | C18_1 | C21_1 | -179.2(8) |
| C17_1 | C18_1 | C19_1 | C20_1 | 0.0(14) |

| | | | | |
|---|---|---|---|---|
| C18_1 | C19_1 | C20_1 | C15_1 | -0.6(15) |
| C20_1 | C15_1 | C16_1 | C17_1 | -0.9(12) |
| C20_1 | C15_1 | B1_1 | N1_1 | 153.3(7) |
| C20_1 | C15_1 | B1_1 | N3_1 | 36.1(9) |
| C20_1 | C15_1 | B1_1 | C22_1 | -86.8(8) |
| C21_1 | C18_1 | C19_1 | C20_1 | 179.3(9) |
| C22_1 | C23_1 | C24_1 | C25_1 | -1.2(12) |
| C23_1 | C22_1 | C28_1 | C27_1 | 0.0(10) |
| C23_1 | C22_1 | B1_1 | N1_1 | 110.2(7) |
| C23_1 | C22_1 | B1_1 | N3_1 | -132.3(7) |
| C23_1 | C22_1 | B1_1 | C15_1 | -11.3(9) |
| C23_1 | C24_1 | C25_1 | C26_1 | -178.5(8) |
| C23_1 | C24_1 | C25_1 | C27_1 | 1.1(12) |
| C24_1 | C25_1 | C27_1 | C28_1 | -0.5(11) |
| C25_1 | C27_1 | C28_1 | C22_1 | 0.0(10) |
| C26_1 | C25_1 | C27_1 | C28_1 | 179.1(7) |
| C28_1 | C22_1 | C23_1 | C24_1 | 0.6(11) |
| C28_1 | C22_1 | B1_1 | N1_1 | -64.4(7) |
| C28_1 | C22_1 | B1_1 | N3_1 | 53.0(8) |
| C28_1 | C22_1 | B1_1 | C15_1 | 174.1(6) |
| B1_1 | N1_1 | C1_1 | Sb1_1 | -16.4(10) |
| B1_1 | N1_1 | C1_1 | N2_1 | 168.6(6) |
| B1_1 | N1_1 | C2_1 | C3_1 | -169.8(6) |
| B1_1 | N3_1 | C8_1 | Sb1_1 | 23.3(11) |
| B1_1 | N3_1 | C8_1 | N4_1 | -163.3(6) |
| B1_1 | N3_1 | C9_1 | C10_1 | 164.2(6) |
| B1_1 | C15_1 | C16_1 | C17_1 | 177.8(7) |
| B1_1 | C15_1 | C20_1 | C19_1 | -177.7(8) |
| B1_1 | C22_1 | C23_1 | C24_1 | -174.2(7) |
| B1_1 | C22_1 | C28_1 | C27_1 | 175.0(6) |
| N1_2 | C2_2 | C3_2 | N2_2 | 0.0(8) |
| N3_2 | C9_2 | C10_2 | N4_2 | 0.1(8) |
| C1_2 | N1_2 | C2_2 | C3_2 | 0.3(8) |
| C1_2 | N1_2 | B1_2 | N3_2 | 36.7(9) |
| C1_2 | N1_2 | B1_2 | C15_2 | -80.4(8) |
| C1_2 | N1_2 | B1_2 | C22_2 | 153.5(6) |
| C1_2 | N2_2 | C3_2 | C2_2 | -0.3(8) |
| C1_2 | N2_2 | C4_2 | C5_2 | 159.7(7) |
| C1_2 | N2_2 | C4_2 | C6_2 | -81.0(9) |
| C1_2 | N2_2 | C4_2 | C7_2 | 41.3(11) |
| C2_2 | N1_2 | C1_2 | Sb1_2 | 171.8(5) |
| C2_2 | N1_2 | C1_2 | N2_2 | -0.5(7) |
| C2_2 | N1_2 | B1_2 | N3_2 | -155.0(6) |
| C2_2 | N1_2 | B1_2 | C15_2 | 87.9(7) |
| C2_2 | N1_2 | B1_2 | C22_2 | -38.2(8) |
| C3_2 | N2_2 | C1_2 | Sb1_2 | -171.4(5) |
| C3_2 | N2_2 | C1_2 | N1_2 | 0.5(7) |
| C3_2 | N2_2 | C4_2 | C5_2 | -27.0(9) |
| C3_2 | N2_2 | C4_2 | C6_2 | 92.3(8) |
| C3_2 | N2_2 | C4_2 | C7_2 | -145.4(8) |
| C4_2 | N2_2 | C1_2 | Sb1_2 | 2.8(10) |
| C4_2 | N2_2 | C1_2 | N1_2 | 174.7(6) |
| C4_2 | N2_2 | C3_2 | C2_2 | -174.7(6) |
| C8_2 | N3_2 | C9_2 | C10_2 | -0.6(8) |
| C8_2 | N3_2 | B1_2 | N1_2 | -32.6(9) |
| C8_2 | N3_2 | B1_2 | C15_2 | 84.7(8) |

| | | | | |
|---|---|---|---|---|
| C8_2 | N3_2 | B1_2 | C22_2 | -149.0(7) |
| C8_2 | N4_2 | C10_2 | C9_2 | 0.4(8) |
| C8_2 | N4_2 | C11_2 | C12_2 | 0.1(11) |
| C8_2 | N4_2 | C11_2 | C13_2 | -121.2(8) |
| C8_2 | N4_2 | C11_2 | C14_2 | 119.7(8) |
| C9_2 | N3_2 | C8_2 | Sb1_2 | -177.0(5) |
| C9_2 | N3_2 | C8_2 | N4_2 | 0.8(8) |
| C9_2 | N3_2 | B1_2 | N1_2 | 156.0(5) |
| C9_2 | N3_2 | B1_2 | C15_2 | -86.6(7) |
| C9_2 | N3_2 | B1_2 | C22_2 | 39.7(8) |
| C10_2 | N4_2 | C8_2 | Sb1_2 | 176.9(5) |
| C10_2 | N4_2 | C8_2 | N3_2 | -0.7(8) |
| C10_2 | N4_2 | C11_2 | C12_2 | -177.2(7) |
| C10_2 | N4_2 | C11_2 | C13_2 | 61.4(9) |
| C10_2 | N4_2 | C11_2 | C14_2 | -57.7(8) |
| C11_2 | N4_2 | C8_2 | Sb1_2 | -0.8(11) |
| C11_2 | N4_2 | C8_2 | N3_2 | -178.4(7) |
| C11_2 | N4_2 | C10_2 | C9_2 | 178.3(6) |
| C15_2 | C16_2 | C17_2 | C18_2 | 0.6(12) |
| C16_2 | C15_2 | C20_2 | C19_2 | 2.1(13) |
| C16_2 | C15_2 | B1_2 | N1_2 | -7.2(9) |
| C16_2 | C15_2 | B1_2 | N3_2 | -125.7(7) |
| C16_2 | C15_2 | B1_2 | C22_2 | 113.2(7) |
| C16_2 | C17_2 | C18_2 | C19_2 | 1.9(13) |
| C16_2 | C17_2 | C18_2 | C21_2 | -178.0(8) |
| C17_2 | C18_2 | C19_2 | C20_2 | -2.3(14) |
| C18_2 | C19_2 | C20_2 | C15_2 | 0.3(15) |
| C20_2 | C15_2 | C16_2 | C17_2 | -2.5(11) |
| C20_2 | C15_2 | B1_2 | N1_2 | 171.1(7) |
| C20_2 | C15_2 | B1_2 | N3_2 | 52.7(8) |
| C20_2 | C15_2 | B1_2 | C22_2 | -68.4(9) |
| C21_2 | C18_2 | C19_2 | C20_2 | 177.6(9) |
| C22_2 | C23_2 | C24_2 | C25_2 | -0.1(12) |
| C23_2 | C22_2 | C28_2 | C27_2 | 1.7(10) |
| C23_2 | C22_2 | B1_2 | N1_2 | 109.9(7) |
| C23_2 | C22_2 | B1_2 | N3_2 | -132.0(7) |
| C23_2 | C22_2 | B1_2 | C15_2 | -10.9(9) |
| C23_2 | C24_2 | C25_2 | C26_2 | -178.0(8) |
| C23_2 | C24_2 | C25_2 | C27_2 | 0.6(12) |
| C24_2 | C25_2 | C27_2 | C28_2 | 0.0(11) |
| C25_2 | C27_2 | C28_2 | C22_2 | -1.2(10) |
| C26_2 | C25_2 | C27_2 | C28_2 | 178.6(7) |
| C28_2 | C22_2 | C23_2 | C24_2 | -1.0(10) |
| C28_2 | C22_2 | B1_2 | N1_2 | -67.2(7) |
| C28_2 | C22_2 | B1_2 | N3_2 | 50.9(7) |
| C28_2 | C22_2 | B1_2 | C15_2 | 172.0(6) |
| B1_2 | N1_2 | C1_2 | Sb1_2 | -18.9(9) |
| B1_2 | N1_2 | C1_2 | N2_2 | 168.8(6) |
| B1_2 | N1_2 | C2_2 | C3_2 | -170.3(6) |
| B1_2 | N3_2 | C8_2 | Sb1_2 | 11.0(10) |
| B1_2 | N3_2 | C8_2 | N4_2 | -171.2(6) |
| B1_2 | N3_2 | C9_2 | C10_2 | 172.7(6) |
| B1_2 | C15_2 | C16_2 | C17_2 | 176.0(7) |
| B1_2 | C15_2 | C20_2 | C19_2 | -176.4(8) |
| B1_2 | C22_2 | C23_2 | C24_2 | -178.2(7) |
| B1_2 | C22_2 | C28_2 | C27_2 | 179.0(6) |

| | | | | |
|---|---|---|---|---|
| N1_3 | C2_3 | C3_3 | N2_3 | -0.6(8) |
| N3_3 | C9_3 | C10_3 | N4_3 | -2.0(9) |
| C1_3 | N1_3 | C2_3 | C3_3 | 0.8(8) |
| C1_3 | N1_3 | B1_3 | N3_3 | 37.1(10) |
| C1_3 | N1_3 | B1_3 | C15_3 | -81.7(9) |
| C1_3 | N1_3 | B1_3 | C22_3 | 152.7(7) |
| C1_3 | N2_3 | C3_3 | C2_3 | 0.1(8) |
| C1_3 | N2_3 | C4_3 | C5_3 | 119.5(9) |
| C1_3 | N2_3 | C4_3 | C6_3 | -121.6(8) |
| C1_3 | N2_3 | C4_3 | C7_3 | -3.0(12) |
| C2_3 | N1_3 | C1_3 | Sb1_3 | 176.0(5) |
| C2_3 | N1_3 | C1_3 | N2_3 | -0.7(8) |
| C2_3 | N1_3 | B1_3 | N3_3 | -153.5(6) |
| C2_3 | N1_3 | B1_3 | C15_3 | 87.7(7) |
| C2_3 | N1_3 | B1_3 | C22_3 | -37.9(8) |
| C3_3 | N2_3 | C1_3 | Sb1_3 | -176.1(5) |
| C3_3 | N2_3 | C1_3 | N1_3 | 0.4(8) |
| C3_3 | N2_3 | C4_3 | C5_3 | -62.1(9) |
| C3_3 | N2_3 | C4_3 | C6_3 | 56.8(9) |
| C3_3 | N2_3 | C4_3 | C7_3 | 175.5(7) |
| C4_3 | N2_3 | C1_3 | Sb1_3 | 2.5(12) |
| C4_3 | N2_3 | C1_3 | N1_3 | 178.9(7) |
| C4_3 | N2_3 | C3_3 | C2_3 | -178.6(7) |
| C8_3 | N3_3 | C9_3 | C10_3 | 1.1(9) |
| C8_3 | N3_3 | B1_3 | N1_3 | -40.9(9) |
| C8_3 | N3_3 | B1_3 | C15_3 | 77.4(8) |
| C8_3 | N3_3 | B1_3 | C22_3 | -157.2(6) |
| C8_3 | N4_3 | C10_3 | C9_3 | 2.1(8) |
| C8_3 | N4_3 | C11_3 | C12_3 | -35.4(12) |
| C8_3 | N4_3 | C11_3 | C13_3 | -155.3(7) |
| C8_3 | N4_3 | C11_3 | C14_3 | 85.2(10) |
| C9_3 | N3_3 | C8_3 | Sb1_3 | -171.1(5) |
| C9_3 | N3_3 | C8_3 | N4_3 | 0.2(8) |
| C9_3 | N3_3 | B1_3 | N1_3 | 152.1(6) |
| C9_3 | N3_3 | B1_3 | C15_3 | -89.6(8) |
| C9_3 | N3_3 | B1_3 | C22_3 | 35.8(8) |
| C10_3 | N4_3 | C8_3 | Sb1_3 | 169.4(5) |
| C10_3 | N4_3 | C8_3 | N3_3 | -1.5(8) |
| C10_3 | N4_3 | C11_3 | C12_3 | 151.7(9) |
| C10_3 | N4_3 | C11_3 | C13_3 | 31.7(10) |
| C10_3 | N4_3 | C11_3 | C14_3 | -87.8(9) |
| C11_3 | N4_3 | C8_3 | Sb1_3 | -4.4(10) |
| C11_3 | N4_3 | C8_3 | N3_3 | -175.2(7) |
| C11_3 | N4_3 | C10_3 | C9_3 | 176.3(7) |
| C15_3 | C16_3 | C17_3 | C18_3 | 0.6(15) |
| C16_3 | C15_3 | C20_3 | C19_3 | 0.8(12) |
| C16_3 | C15_3 | B1_3 | N1_3 | -46.4(9) |
| C16_3 | C15_3 | B1_3 | N3_3 | -165.3(7) |
| C16_3 | C15_3 | B1_3 | C22_3 | 74.6(9) |
| C16_3 | C17_3 | C18_3 | C19_3 | 0.8(14) |
| C16_3 | C17_3 | C18_3 | C21_3 | -177.3(9) |
| C17_3 | C18_3 | C19_3 | C20_3 | -1.3(14) |
| C18_3 | C19_3 | C20_3 | C15_3 | 0.5(14) |
| C20_3 | C15_3 | C16_3 | C17_3 | -1.4(13) |
| C20_3 | C15_3 | B1_3 | N1_3 | 132.1(7) |
| C20_3 | C15_3 | B1_3 | N3_3 | 13.2(10) |

| | | | | |
|---|---|---|---|---|
| C20_3 | C15_3 | B1_3 | C22_3 | -106.9(8) |
| C21_3 | C18_3 | C19_3 | C20_3 | 176.8(9) |
| C22_3 | C23_3 | C24_3 | C25_3 | 1.0(13) |
| C23_3 | C22_3 | C28_3 | C27_3 | -1.9(10) |
| C23_3 | C22_3 | B1_3 | N1_3 | 132.8(7) |
| C23_3 | C22_3 | B1_3 | N3_3 | -109.9(7) |
| C23_3 | C22_3 | B1_3 | C15_3 | 11.5(9) |
| C23_3 | C24_3 | C25_3 | C26_3 | 177.7(9) |
| C23_3 | C24_3 | C25_3 | C27_3 | -2.2(13) |
| C24_3 | C25_3 | C27_3 | C28_3 | 1.3(12) |
| C25_3 | C27_3 | C28_3 | C22_3 | 0.7(11) |
| C26_3 | C25_3 | C27_3 | C28_3 | -178.5(8) |
| C28_3 | C22_3 | C23_3 | C24_3 | 1.1(11) |
| C28_3 | C22_3 | B1_3 | N1_3 | -50.6(8) |
| C28_3 | C22_3 | B1_3 | N3_3 | 66.7(7) |
| C28_3 | C22_3 | B1_3 | C15_3 | -171.9(6) |
| B1_3 | N1_3 | C1_3 | Sb1_3 | -13.6(10) |
| B1_3 | N1_3 | C1_3 | N2_3 | 169.6(6) |
| B1_3 | N1_3 | C2_3 | C3_3 | -170.8(6) |
| B1_3 | N3_3 | C8_3 | Sb1_3 | 20.8(10) |
| B1_3 | N3_3 | C8_3 | N4_3 | -167.9(6) |
| B1_3 | N3_3 | C9_3 | C10_3 | 170.5(6) |
| B1_3 | C15_3 | C16_3 | C17_3 | 177.2(8) |
| B1_3 | C15_3 | C20_3 | C19_3 | -177.7(8) |
| B1_3 | C22_3 | C23_3 | C24_3 | 177.8(7) |
| B1_3 | C22_3 | C28_3 | C27_3 | -178.7(6) |
| N1_4 | C2_4 | C3_4 | N2_4 | -0.2(9) |
| N3_4 | C9_4 | C10_4 | N4_4 | -0.9(8) |
| C1_4 | N1_4 | C2_4 | C3_4 | 0.8(9) |
| C1_4 | N1_4 | B1_4 | N3_4 | 45.0(8) |
| C1_4 | N1_4 | B1_4 | C15_4 | -71.0(8) |
| C1_4 | N1_4 | B1_4 | C22_4 | 164.9(6) |
| C1_4 | N2_4 | C3_4 | C2_4 | -0.4(9) |
| C1_4 | N2_4 | C4_4 | C5_4 | 161.2(8) |
| C1_4 | N2_4 | C4_4 | C6_4 | -70.7(11) |
| C1_4 | N2_4 | C4_4 | C7_4 | 52.5(10) |
| C2_4 | N1_4 | C1_4 | Sb1_4 | 172.9(5) |
| C2_4 | N1_4 | C1_4 | N2_4 | -1.1(8) |
| C2_4 | N1_4 | B1_4 | N3_4 | -145.1(6) |
| C2_4 | N1_4 | B1_4 | C15_4 | 98.9(7) |
| C2_4 | N1_4 | B1_4 | C22_4 | -25.2(8) |
| C3_4 | N2_4 | C1_4 | Sb1_4 | -172.7(5) |
| C3_4 | N2_4 | C1_4 | N1_4 | 0.9(8) |
| C3_4 | N2_4 | C4_4 | C5_4 | -19.9(11) |
| C3_4 | N2_4 | C4_4 | C6_4 | 108.3(9) |
| C3_4 | N2_4 | C4_4 | C7_4 | -128.5(8) |
| C4_4 | N2_4 | C1_4 | Sb1_4 | 6.4(10) |
| C4_4 | N2_4 | C1_4 | N1_4 | 180.0(6) |
| C4_4 | N2_4 | C3_4 | C2_4 | -179.5(7) |
| C8_4 | N3_4 | C9_4 | C10_4 | 0.5(8) |
| C8_4 | N3_4 | B1_4 | N1_4 | -50.0(8) |
| C8_4 | N3_4 | B1_4 | C15_4 | 67.5(8) |
| C8_4 | N3_4 | B1_4 | C22_4 | -166.7(6) |
| C8_4 | N4_4 | C10_4 | C9_4 | 0.9(8) |
| C8_4 | N4_4 | C11_4 | C12_4 | -46.7(9) |
| C8_4 | N4_4 | C11_4 | C13_4 | -164.1(7) |

| | | | | |
|---|---|---|---|---|
| C8_4 | N4_4 | C11_4 | C14_4 | 75.4(8) |
| C9_4 | N3_4 | C8_4 | Sb1_4 | -170.5(5) |
| C9_4 | N3_4 | C8_4 | N4_4 | 0.0(8) |
| C9_4 | N3_4 | B1_4 | N1_4 | 147.4(6) |
| C9_4 | N3_4 | B1_4 | C15_4 | -95.1(7) |
| C9_4 | N3_4 | B1_4 | C22_4 | 30.7(8) |
| C10_4 | N4_4 | C8_4 | Sb1_4 | 169.6(5) |
| C10_4 | N4_4 | C8_4 | N3_4 | -0.6(8) |
| C10_4 | N4_4 | C11_4 | C12_4 | 131.4(7) |
| C10_4 | N4_4 | C11_4 | C13_4 | 14.0(9) |
| C10_4 | N4_4 | C11_4 | C14_4 | -106.6(7) |
| C11_4 | N4_4 | C8_4 | Sb1_4 | -12.1(10) |
| C11_4 | N4_4 | C8_4 | N3_4 | 177.8(6) |
| C11_4 | N4_4 | C10_4 | C9_4 | -177.5(6) |
| C15_4 | C16_4 | C17_4 | C18_4 | -0.1(12) |
| C16_4 | C15_4 | C20_4 | C19_4 | -0.5(12) |
| C16_4 | C15_4 | B1_4 | N1_4 | -21.2(9) |
| C16_4 | C15_4 | B1_4 | N3_4 | -139.1(7) |
| C16_4 | C15_4 | B1_4 | C22_4 | 97.5(8) |
| C16_4 | C17_4 | C18_4 | C19_4 | -0.9(12) |
| C16_4 | C17_4 | C18_4 | C21_4 | -178.4(7) |
| C17_4 | C18_4 | C19_4 | C20_4 | 1.2(13) |
| C18_4 | C19_4 | C20_4 | C15_4 | -0.5(14) |
| C20_4 | C15_4 | C16_4 | C17_4 | 0.8(11) |
| C20_4 | C15_4 | B1_4 | N1_4 | 153.9(7) |
| C20_4 | C15_4 | B1_4 | N3_4 | 35.9(9) |
| C20_4 | C15_4 | B1_4 | C22_4 | -87.5(8) |
| C21_4 | C18_4 | C19_4 | C20_4 | 178.7(8) |
| C22_4 | C23_4 | C24_4 | C25_4 | -1.0(12) |
| C23_4 | C22_4 | C28_4 | C27_4 | 0.4(10) |
| C23_4 | C22_4 | B1_4 | N1_4 | 103.8(7) |
| C23_4 | C22_4 | B1_4 | N3_4 | -137.4(6) |
| C23_4 | C22_4 | B1_4 | C15_4 | -16.5(9) |
| C23_4 | C24_4 | C25_4 | C26_4 | -179.3(8) |
| C23_4 | C24_4 | C25_4 | C27_4 | 1.6(12) |
| C24_4 | C25_4 | C27_4 | C28_4 | -1.1(11) |
| C25_4 | C27_4 | C28_4 | C22_4 | 0.1(11) |
| C26_4 | C25_4 | C27_4 | C28_4 | 179.8(7) |
| C28_4 | C22_4 | C23_4 | C24_4 | 0.0(10) |
| C28_4 | C22_4 | B1_4 | N1_4 | -68.4(7) |
| C28_4 | C22_4 | B1_4 | N3_4 | 50.4(8) |
| C28_4 | C22_4 | B1_4 | C15_4 | 171.4(6) |
| B1_4 | N1_4 | C1_4 | Sb1_4 | -15.9(9) |
| B1_4 | N1_4 | C1_4 | N2_4 | 170.1(6) |
| B1_4 | N1_4 | C2_4 | C3_4 | -170.8(6) |
| B1_4 | N3_4 | C8_4 | Sb1_4 | 24.8(9) |
| B1_4 | N3_4 | C8_4 | N4_4 | -164.7(6) |
| B1_4 | N3_4 | C9_4 | C10_4 | 165.9(6) |
| B1_4 | C15_4 | C16_4 | C17_4 | 176.1(7) |
| B1_4 | C15_4 | C20_4 | C19_4 | -175.9(8) |
| B1_4 | C22_4 | C23_4 | C24_4 | -172.5(7) |
| B1_4 | C22_4 | C28_4 | C27_4 | 173.0(6) |
| N1_5 | C2_5 | C3_5 | N2_5 | 0.0(9) |
| N3_5 | C9_5 | C10_5 | N4_5 | 0.3(8) |
| C1_5 | N1_5 | C2_5 | C3_5 | 0.4(8) |
| C1_5 | N1_5 | B1_5 | N3_5 | 42.8(9) |

| | | | | |
|---|---|---|---|---|
| C1_5 | N1_5 | B1_5 | C15_5 | -73.4(8) |
| C1_5 | N1_5 | B1_5 | C22_5 | 160.5(6) |
| C1_5 | N2_5 | C3_5 | C2_5 | -0.4(8) |
| C1_5 | N2_5 | C4_5 | C5_5 | 167.9(7) |
| C1_5 | N2_5 | C4_5 | C6_5 | -68.7(10) |
| C1_5 | N2_5 | C4_5 | C7_5 | 53.6(9) |
| C2_5 | N1_5 | C1_5 | Sb1_5 | 176.7(5) |
| C2_5 | N1_5 | C1_5 | N2_5 | -0.6(8) |
| C2_5 | N1_5 | B1_5 | N3_5 | -149.8(6) |
| C2_5 | N1_5 | B1_5 | C15_5 | 94.0(7) |
| C2_5 | N1_5 | B1_5 | C22_5 | -32.1(8) |
| C3_5 | N2_5 | C1_5 | Sb1_5 | -176.5(5) |
| C3_5 | N2_5 | C1_5 | N1_5 | 0.6(8) |
| C3_5 | N2_5 | C4_5 | C5_5 | -13.8(10) |
| C3_5 | N2_5 | C4_5 | C6_5 | 109.6(8) |
| C3_5 | N2_5 | C4_5 | C7_5 | -128.1(7) |
| C4_5 | N2_5 | C1_5 | Sb1_5 | 2.0(10) |
| C4_5 | N2_5 | C1_5 | N1_5 | 179.2(6) |
| C4_5 | N2_5 | C3_5 | C2_5 | -179.0(7) |
| C8_5 | N3_5 | C9_5 | C10_5 | -0.8(8) |
| C8_5 | N3_5 | B1_5 | N1_5 | -48.1(9) |
| C8_5 | N3_5 | B1_5 | C15_5 | 69.3(8) |
| C8_5 | N3_5 | B1_5 | C22_5 | -163.7(6) |
| C8_5 | N4_5 | C10_5 | C9_5 | 0.3(8) |
| C8_5 | N4_5 | C11_5 | C12_5 | -46.4(9) |
| C8_5 | N4_5 | C11_5 | C13_5 | -164.1(7) |
| C8_5 | N4_5 | C11_5 | C14_5 | 75.5(9) |
| C9_5 | N3_5 | C8_5 | Sb1_5 | -170.6(5) |
| C9_5 | N3_5 | C8_5 | N4_5 | 1.0(8) |
| C9_5 | N3_5 | B1_5 | N1_5 | 148.6(6) |
| C9_5 | N3_5 | B1_5 | C15_5 | -94.0(7) |
| C9_5 | N3_5 | B1_5 | C22_5 | 33.0(9) |
| C10_5 | N4_5 | C8_5 | Sb1_5 | 170.5(5) |
| C10_5 | N4_5 | C8_5 | N3_5 | -0.8(8) |
| C10_5 | N4_5 | C11_5 | C12_5 | 130.9(7) |
| C10_5 | N4_5 | C11_5 | C13_5 | 13.2(10) |
| C10_5 | N4_5 | C11_5 | C14_5 | -107.2(8) |
| C11_5 | N4_5 | C8_5 | Sb1_5 | -11.7(10) |
| C11_5 | N4_5 | C8_5 | N3_5 | 176.9(6) |
| C11_5 | N4_5 | C10_5 | C9_5 | -177.4(7) |
| C15_5 | C16_5 | C17_5 | C18_5 | 1.0(12) |
| C16_5 | C15_5 | C20_5 | C19_5 | 2.0(17) |
| C16_5 | C15_5 | B1_5 | N1_5 | -15.3(9) |
| C16_5 | C15_5 | B1_5 | N3_5 | -133.0(7) |
| C16_5 | C15_5 | B1_5 | C22_5 | 104.4(8) |
| C16_5 | C17_5 | C18_5 | C19_5 | -1.4(14) |
| C16_5 | C17_5 | C18_5 | C21_5 | -178.1(8) |
| C17_5 | C18_5 | C19_5 | C20_5 | 2.1(18) |
| C18_5 | C19_5 | C20_5 | C15_5 | -2(2) |
| C20_5 | C15_5 | C16_5 | C17_5 | -1.3(12) |
| C20_5 | C15_5 | B1_5 | N1_5 | 160.8(9) |
| C20_5 | C15_5 | B1_5 | N3_5 | 43.1(10) |
| C20_5 | C15_5 | B1_5 | C22_5 | -79.5(10) |
| C21_5 | C18_5 | C19_5 | C20_5 | 178.7(12) |
| C22_5 | C23_5 | C24_5 | C25_5 | -0.6(14) |
| C23_5 | C22_5 | C28_5 | C27_5 | 1.0(10) |

| | | | | |
|---|---|---|---|---|
| C23_5 | C22_5 | B1_5 | N1_5 | 105.5(8) |
| C23_5 | C22_5 | B1_5 | N3_5 | -136.9(7) |
| C23_5 | C22_5 | B1_5 | C15_5 | -15.7(10) |
| C23_5 | C24_5 | C25_5 | C26_5 | 179.4(9) |
| C23_5 | C24_5 | C25_5 | C27_5 | -0.5(14) |
| C24_5 | C25_5 | C27_5 | C28_5 | 1.8(12) |
| C25_5 | C27_5 | C28_5 | C22_5 | -2.1(11) |
| C26_5 | C25_5 | C27_5 | C28_5 | -178.2(8) |
| C28_5 | C22_5 | C23_5 | C24_5 | 0.3(12) |
| C28_5 | C22_5 | B1_5 | N1_5 | -67.2(7) |
| C28_5 | C22_5 | B1_5 | N3_5 | 50.4(8) |
| C28_5 | C22_5 | B1_5 | C15_5 | 171.6(6) |
| B1_5 | N1_5 | C1_5 | Sb1_5 | -14.4(9) |
| B1_5 | N1_5 | C1_5 | N2_5 | 168.3(6) |
| B1_5 | N1_5 | C2_5 | C3_5 | -169.4(6) |
| B1_5 | N3_5 | C8_5 | Sb1_5 | 24.1(9) |
| B1_5 | N3_5 | C8_5 | N4_5 | -164.3(6) |
| B1_5 | N3_5 | C9_5 | C10_5 | 165.2(6) |
| B1_5 | C15_5 | C16_5 | C17_5 | 175.1(7) |
| B1_5 | C15_5 | C20_5 | C19_5 | -174.4(11) |
| B1_5 | C22_5 | C23_5 | C24_5 | -172.7(8) |
| B1_5 | C22_5 | C28_5 | C27_5 | 174.3(6) |
| N1_6 | C2_6 | C3_6 | N2_6 | 0.0(8) |
| N3_6 | C9_6 | C10_6 | N4_6 | 1.0(8) |
| C1_6 | N1_6 | C2_6 | C3_6 | 0.3(8) |
| C1_6 | N1_6 | B1_6 | N3_6 | 37.0(9) |
| C1_6 | N1_6 | B1_6 | C15_6 | -80.0(8) |
| C1_6 | N1_6 | B1_6 | C22_6 | 153.5(6) |
| C1_6 | N2_6 | C3_6 | C2_6 | -0.3(8) |
| C1_6 | N2_6 | C4_6 | C5_6 | 159.8(7) |
| C1_6 | N2_6 | C4_6 | C6_6 | -79.2(11) |
| C1_6 | N2_6 | C4_6 | C7_6 | 43.2(12) |
| C2_6 | N1_6 | C1_6 | Sb1_6 | 171.3(5) |
| C2_6 | N1_6 | C1_6 | N2_6 | -0.5(7) |
| C2_6 | N1_6 | B1_6 | N3_6 | -154.3(6) |
| C2_6 | N1_6 | B1_6 | C15_6 | 88.6(7) |
| C2_6 | N1_6 | B1_6 | C22_6 | -37.9(8) |
| C3_6 | N2_6 | C1_6 | Sb1_6 | -170.8(5) |
| C3_6 | N2_6 | C1_6 | N1_6 | 0.5(7) |
| C3_6 | N2_6 | C4_6 | C5_6 | -26.3(10) |
| C3_6 | N2_6 | C4_6 | C6_6 | 94.7(10) |
| C3_6 | N2_6 | C4_6 | C7_6 | -142.9(11) |
| C4_6 | N2_6 | C1_6 | Sb1_6 | 3.9(9) |
| C4_6 | N2_6 | C1_6 | N1_6 | 175.2(6) |
| C4_6 | N2_6 | C3_6 | C2_6 | -175.2(6) |
| C8_6 | N3_6 | C9_6 | C10_6 | -0.6(8) |
| C8_6 | N3_6 | B1_6 | N1_6 | -33.3(10) |
| C8_6 | N3_6 | B1_6 | C15_6 | 84.1(8) |
| C8_6 | N3_6 | B1_6 | C22_6 | -149.4(7) |
| C8_6 | N4_6 | C10_6 | C9_6 | -1.0(9) |
| C8_6 | N4_6 | C11_6 | C12_6 | 2.3(13) |
| C8_6 | N4_6 | C11_6 | C13_6 | -119.9(9) |
| C8_6 | N4_6 | C11_6 | C14_6 | 121.5(9) |
| C9_6 | N3_6 | C8_6 | Sb1_6 | -176.8(5) |
| C9_6 | N3_6 | C8_6 | N4_6 | 0.0(8) |
| C9_6 | N3_6 | B1_6 | N1_6 | 156.0(6) |

| | | | | |
|---|---|---|---|---|
| C9_6 | N3_6 | B1_6 | C15_6 | -86.6(7) |
| C9_6 | N3_6 | B1_6 | C22_6 | 40.0(8) |
| C10_6 | N4_6 | C8_6 | Sb1_6 | 177.2(5) |
| C10_6 | N4_6 | C8_6 | N3_6 | 0.6(9) |
| C10_6 | N4_6 | C11_6 | C12_6 | -175.9(7) |
| C10_6 | N4_6 | C11_6 | C13_6 | 61.8(9) |
| C10_6 | N4_6 | C11_6 | C14_6 | -56.7(9) |
| C11_6 | N4_6 | C8_6 | Sb1_6 | -1.2(13) |
| C11_6 | N4_6 | C8_6 | N3_6 | -177.8(8) |
| C11_6 | N4_6 | C10_6 | C9_6 | 177.6(7) |
| C15_6 | C16_6 | C17_6 | C18_6 | -1.5(13) |
| C16_6 | C15_6 | C20_6 | C19_6 | 0.7(13) |
| C16_6 | C15_6 | B1_6 | N1_6 | -5.0(10) |
| C16_6 | C15_6 | B1_6 | N3_6 | -123.7(7) |
| C16_6 | C15_6 | B1_6 | C22_6 | 115.5(8) |
| C16_6 | C17_6 | C18_6 | C19_6 | 1.7(13) |
| C16_6 | C17_6 | C18_6 | C21_6 | -176.4(8) |
| C17_6 | C18_6 | C19_6 | C20_6 | -0.7(15) |
| C18_6 | C19_6 | C20_6 | C15_6 | -0.4(16) |
| C20_6 | C15_6 | C16_6 | C17_6 | 0.3(12) |
| C20_6 | C15_6 | B1_6 | N1_6 | 169.9(7) |
| C20_6 | C15_6 | B1_6 | N3_6 | 51.3(9) |
| C20_6 | C15_6 | B1_6 | C22_6 | -69.5(9) |
| C21_6 | C18_6 | C19_6 | C20_6 | 177.3(10) |
| C22_6 | C23_6 | C24_6 | C25_6 | -1.8(12) |
| C23_6 | C22_6 | C28_6 | C27_6 | 0.9(10) |
| C23_6 | C22_6 | B1_6 | N1_6 | 106.1(7) |
| C23_6 | C22_6 | B1_6 | N3_6 | -135.8(7) |
| C23_6 | C22_6 | B1_6 | C15_6 | -14.9(9) |
| C23_6 | C24_6 | C25_6 | C26_6 | -178.6(8) |
| C23_6 | C24_6 | C25_6 | C27_6 | 1.6(12) |
| C24_6 | C25_6 | C27_6 | C28_6 | -0.2(12) |
| C25_6 | C27_6 | C28_6 | C22_6 | -1.0(11) |
| C26_6 | C25_6 | C27_6 | C28_6 | 179.9(8) |
| C28_6 | C22_6 | C23_6 | C24_6 | 0.6(11) |
| C28_6 | C22_6 | B1_6 | N1_6 | -69.5(7) |
| C28_6 | C22_6 | B1_6 | N3_6 | 48.6(7) |
| C28_6 | C22_6 | B1_6 | C15_6 | 169.5(6) |
| B1_6 | N1_6 | C1_6 | Sb1_6 | -18.9(9) |
| B1_6 | N1_6 | C1_6 | N2_6 | 169.3(6) |
| B1_6 | N1_6 | C2_6 | C3_6 | -170.7(6) |
| B1_6 | N3_6 | C8_6 | Sb1_6 | 11.6(10) |
| B1_6 | N3_6 | C8_6 | N4_6 | -171.5(6) |
| B1_6 | N3_6 | C9_6 | C10_6 | 172.2(6) |
| B1_6 | C15_6 | C16_6 | C17_6 | 175.5(7) |
| B1_6 | C15_6 | C20_6 | C19_6 | -174.7(9) |
| B1_6 | C22_6 | C23_6 | C24_6 | -175.2(7) |
| B1_6 | C22_6 | C28_6 | C27_6 | 176.8(6) |

9.7.12 Hydrogen Fractional Atomic Coordinates and Equivalent Isotropic Displacement Parameters

Table S48. Hydrogen Fractional Atomic Coordinates (×10[4]) and Equivalent Isotropic Displacement Parameters (Å$^2$×10$^3$) for **6**. $U_{eq}$ is defined as 1/3 of the trace of the orthogonalised $U_{ij}$.

| Atom | x | y | z | $U_{eq}$ |
|---|---|---|---|---|
| H2_1 | 3915.17 | 4196.98 | 7371.11 | 28 |
| H3_1 | 4632.36 | 3053.21 | 7764.36 | 28 |
| H5A_1 | 5038.47 | 2016.18 | 8308.48 | 88 |
| H5B_1 | 4290.5 | 1955.9 | 8121.53 | 88 |
| H5C_1 | 4723.41 | 1384.69 | 8678.99 | 88 |
| H6A_1 | 4768.46 | 1635.76 | 9530.94 | 154 |
| H6B_1 | 4329.62 | 2330.53 | 9474.5 | 154 |
| H6C_1 | 5050.92 | 2271.83 | 9112.73 | 154 |
| H7A_1 | 3100.88 | 2155.69 | 8742.76 | 205 |
| H7B_1 | 3415.25 | 1757.73 | 9382.41 | 205 |
| H7C_1 | 3637.79 | 1456.7 | 8936.12 | 205 |
| H9_1 | 2149.52 | 5586.34 | 8197.03 | 21 |
| H10_1 | 1540.33 | 5395.3 | 9122.2 | 27 |
| H12A_1 | 757.01 | 3506.28 | 10178.38 | 58 |
| H12B_1 | 1428.44 | 3227.29 | 9873.35 | 58 |
| H12C_1 | 811.3 | 3840.78 | 9498.09 | 58 |
| H13A_1 | 650 | 4860.13 | 9708.23 | 55 |
| H13B_1 | 1266.24 | 4852.89 | 10089.58 | 55 |
| H13C_1 | 726.76 | 4371.18 | 10372.77 | 55 |
| H14A_1 | 2319.21 | 4013.76 | 10404.15 | 54 |
| H14B_1 | 2502.19 | 3440.63 | 10212.98 | 54 |
| H14C_1 | 1913.35 | 3427.55 | 10696.39 | 54 |
| H16_1 | 2467.08 | 3753.1 | 7393.46 | 28 |
| H17_1 | 1455.56 | 3578.92 | 7096.55 | 32 |
| H19_1 | 142.91 | 4834.68 | 7614.15 | 43 |
| H20_1 | 1146.36 | 5011.59 | 7907.33 | 36 |
| H21A_1 | 168.65 | 3953.44 | 6817.57 | 54 |
| H21B_1 | -351.74 | 4461.78 | 7013.04 | 54 |
| H21C_1 | -130.25 | 3712.4 | 7454.82 | 54 |
| H23_1 | 2363.59 | 5352.56 | 6778.93 | 32 |
| H24_1 | 2928.85 | 6161.77 | 6132.51 | 41 |
| H26A_1 | 3944.99 | 6973.99 | 6443.38 | 64 |
| H26B_1 | 3740.3 | 6868.49 | 5918.93 | 64 |
| H26C_1 | 4497.65 | 6474.01 | 6255.27 | 64 |
| H27_1 | 4211.74 | 5892.95 | 7399.52 | 30 |
| H28_1 | 3655.77 | 5070.86 | 8040.64 | 22 |
| H29A_1 | 1401.36 | 3334.01 | 8726.72 | 53 |
| H29B_1 | 1598.58 | 2579.62 | 9158.05 | 53 |
| H29C_1 | 1997.11 | 2840.8 | 8557.31 | 53 |
| H2_2 | 3629.95 | 887.42 | 3908.83 | 23 |
| H3_2 | 4339.69 | -253.1 | 4281.99 | 21 |
| H5A_2 | 4814.43 | -1236.49 | 4812.11 | 41 |
| H5B_2 | 4053.33 | -1421.73 | 4821.74 | 41 |
| H5C_2 | 4656.95 | -1898.28 | 5305.8 | 41 |
| H6A_2 | 4958.81 | -1519.68 | 6013.4 | 64 |
| H6B_2 | 4494.97 | -846.93 | 5991.57 | 64 |
| H6C_2 | 5061.97 | -850.15 | 5498.72 | 64 |
| H7A_2 | 3137.36 | -1356.06 | 5583.52 | 78 |

| | | | | |
|---|---|---|---|---|
| H7B_2 | 3421.81 | -1277.57 | 6098.45 | 78 |
| H7C_2 | 3779.38 | -1903.56 | 5996.23 | 78 |
| H9_2 | 1965.3 | 2319.84 | 4774.34 | 21 |
| H10_2 | 1600.44 | 2206.88 | 5742.01 | 24 |
| H12A_2 | 2046.99 | 132.07 | 7382.02 | 47 |
| H12B_2 | 2669.42 | 137.36 | 6922.65 | 47 |
| H12C_2 | 1891.15 | 33.58 | 6841.39 | 47 |
| H13A_2 | 806.78 | 971.26 | 6565.05 | 57 |
| H13B_2 | 895.76 | 1655.69 | 6498.17 | 57 |
| H13C_2 | 955.69 | 1053.65 | 7115.85 | 57 |
| H14A_2 | 2110.91 | 1803 | 6625.84 | 45 |
| H14B_2 | 2804.35 | 1208.69 | 6782.83 | 45 |
| H14C_2 | 2192.39 | 1207.98 | 7248.52 | 45 |
| H16_2 | 2229.58 | 315.89 | 4284.1 | 27 |
| H17_2 | 1160.26 | 177.43 | 4037.16 | 32 |
| H19_2 | 52.89 | 1828.56 | 4100.58 | 41 |
| H20_2 | 1112.67 | 1964.7 | 4357.21 | 35 |
| H21A_2 | -106.86 | 694.78 | 3637.06 | 61 |
| H21B_2 | -563.59 | 1291.42 | 3749.49 | 61 |
| H21C_2 | -404.58 | 575.4 | 4260.96 | 61 |
| H23_2 | 2262.89 | 2123.5 | 3434.14 | 28 |
| H24_2 | 2800.34 | 2970.84 | 2826.6 | 40 |
| H26A_2 | 3644.16 | 3802.57 | 3115.67 | 72 |
| H26B_2 | 3758.62 | 3555.1 | 2630.56 | 72 |
| H26C_2 | 4379.94 | 3303.4 | 3104.53 | 72 |
| H27_2 | 4121.98 | 2599.13 | 4114.89 | 29 |
| H28_2 | 3598.65 | 1741.7 | 4714.45 | 23 |
| H29A_2 | 1401.24 | -234.8 | 6069.93 | 45 |
| H29B_2 | 1867.9 | -767.41 | 5878.9 | 45 |
| H29C_2 | 1634.88 | -26.67 | 5418.98 | 45 |
| H2_3 | 7098.7 | 10423.71 | 8543.94 | 23 |
| H3_3 | 6671.13 | 11284.61 | 7588.38 | 25 |
| H5A_3 | 5888.76 | 11467.18 | 6870.91 | 58 |
| H5B_3 | 5795.99 | 10876.17 | 6761.71 | 58 |
| H5C_3 | 5899.99 | 11536.66 | 6234.6 | 58 |
| H6A_3 | 7145.81 | 11803.79 | 6059.11 | 50 |
| H6B_3 | 7775.45 | 11355.07 | 6528.47 | 50 |
| H6C_3 | 7079.13 | 11793.32 | 6676.15 | 50 |
| H7A_3 | 6906.65 | 10220.42 | 6483.37 | 49 |
| H7B_3 | 7646.62 | 10470.63 | 6348.01 | 49 |
| H7C_3 | 6953.04 | 10880.68 | 5932.93 | 49 |
| H9_3 | 8779.08 | 8138.41 | 9357.06 | 29 |
| H10_3 | 9499.3 | 7407.67 | 8947.27 | 25 |
| H12A_3 | 8819.04 | 7556.23 | 7189.78 | 113 |
| H12B_3 | 8425.31 | 8239.78 | 7181.15 | 113 |
| H12C_3 | 8199.88 | 7591.53 | 7641.94 | 113 |
| H13A_3 | 9106.23 | 6782.77 | 8452.78 | 46 |
| H13B_3 | 9906.78 | 6912.83 | 8400.44 | 46 |
| H13C_3 | 9612.92 | 6759.19 | 7934.62 | 46 |
| H14A_3 | 10149.99 | 7960.59 | 7773.35 | 90 |
| H14B_3 | 9578.87 | 8488.82 | 7282.31 | 90 |
| H14C_3 | 10004.85 | 7836.83 | 7244.48 | 90 |
| H16_3 | 6192.78 | 9667.73 | 8859.45 | 39 |
| H17_3 | 5151.19 | 9276.32 | 9105.48 | 42 |
| H19_3 | 6307.29 | 7516.14 | 9318.64 | 39 |
| H20_3 | 7347.4 | 7911.43 | 9057.58 | 31 |

| | | | | |
|---|---|---|---|---|
| H21A_3 | 4582.27 | 8328.96 | 9562.62 | 62 |
| H21B_3 | 5040.62 | 7637.03 | 9625.21 | 62 |
| H21C_3 | 4696.46 | 8177.96 | 9019.61 | 62 |
| H23_3 | 7383.49 | 8826.66 | 9869.81 | 33 |
| H24_3 | 7934.94 | 9013.31 | 10512.74 | 49 |
| H26A_3 | 9473.06 | 9449.41 | 10433.81 | 86 |
| H26B_3 | 8705.35 | 9529.69 | 10737.33 | 86 |
| H26C_3 | 8918.95 | 10142.83 | 10211.85 | 86 |
| H27_3 | 9214.6 | 9972.73 | 9280.93 | 34 |
| H28_3 | 8689.41 | 9769.97 | 8635.55 | 24 |
| H29A_3 | 6672.99 | 8734.27 | 7888.53 | 47 |
| H29B_3 | 6867.18 | 8470.63 | 7415.01 | 47 |
| H29C_3 | 6393.95 | 9190.36 | 7244.39 | 47 |
| H2_4 | 1101.24 | 5072.28 | 3992.98 | 29 |
| H3_4 | 409.74 | 5823.1 | 4375.74 | 27 |
| H5A_4 | -31.98 | 6278.9 | 5003 | 81 |
| H5B_4 | 647.01 | 6572.56 | 4676.52 | 81 |
| H5C_4 | 371.23 | 6623 | 5254.18 | 81 |
| H6A_4 | 534.56 | 5538.49 | 6168.56 | 87 |
| H6B_4 | 1019.05 | 4905.28 | 6126.86 | 87 |
| H6C_4 | 213.9 | 5206.21 | 5856.68 | 87 |
| H7A_4 | 1836.16 | 6155.26 | 5089 | 72 |
| H7B_4 | 1990.7 | 5553.73 | 5706.18 | 72 |
| H7C_4 | 1482.87 | 6230.7 | 5640.9 | 72 |
| H9_4 | 2946.42 | 2879.76 | 4818.07 | 21 |
| H10_4 | 3583.49 | 2159.59 | 5755.78 | 22 |
| H12A_4 | 4439.08 | 3024.43 | 6759.66 | 36 |
| H12B_4 | 3727.31 | 3592.39 | 6503.07 | 36 |
| H12C_4 | 4303.14 | 3404.97 | 6084.46 | 36 |
| H13A_4 | 4502.09 | 2139.21 | 6286.11 | 39 |
| H13B_4 | 3932.63 | 1756.79 | 6709.2 | 39 |
| H13C_4 | 4501.87 | 1987.47 | 6945.24 | 39 |
| H14A_4 | 2818.41 | 2322.84 | 6952.06 | 37 |
| H14B_4 | 2712.2 | 3060.23 | 6840.63 | 37 |
| H14C_4 | 3291.8 | 2502.04 | 7308.9 | 37 |
| H16_4 | 2585.02 | 5520.94 | 4010.86 | 23 |
| H17_4 | 3579.54 | 5999.71 | 3711.22 | 25 |
| H19_4 | 4911.87 | 4238.1 | 4198.82 | 37 |
| H20_4 | 3927.56 | 3761.14 | 4488.92 | 35 |
| H21A_4 | 4845.49 | 5917.93 | 3425.36 | 47 |
| H21B_4 | 5381.13 | 5220.15 | 3606.21 | 47 |
| H21C_4 | 5171.83 | 5510.13 | 4061.06 | 47 |
| H23_4 | 2589.78 | 4595.67 | 3364.15 | 32 |
| H24_4 | 1967.29 | 4437.44 | 2737.92 | 39 |
| H26A_4 | 939.59 | 3280.75 | 3090.94 | 71 |
| H26B_4 | 1223.91 | 3842.96 | 2556.87 | 71 |
| H26C_4 | 471.1 | 4010.96 | 2848.39 | 71 |
| H27_4 | 870.1 | 3321.5 | 4039.89 | 29 |
| H28_4 | 1476.29 | 3484.92 | 4672.3 | 22 |
| H29A_4 | 3746.03 | 4623.79 | 5313.66 | 42 |
| H29B_4 | 3539.21 | 4989.76 | 5711.87 | 42 |
| H29C_4 | 3139.62 | 5287.2 | 5106.33 | 42 |
| H2_5 | 3722 | 7644.24 | 573.88 | 25 |
| H3_5 | 4438.03 | 6505.6 | 933.68 | 26 |
| H5A_5 | 4958.74 | 5497.1 | 1558.6 | 50 |
| H5B_5 | 4293.4 | 5415.65 | 1283.75 | 50 |

| | | | | |
|---|---|---|---|---|
| H5C_5 | 4667.43 | 4844.25 | 1866.63 | 50 |
| H6A_5 | 4510.17 | 5037.31 | 2751.34 | 70 |
| H6B_5 | 3971.71 | 5685.93 | 2724.99 | 70 |
| H6C_5 | 4743.66 | 5716.3 | 2418.57 | 70 |
| H7A_5 | 3118.23 | 5379.58 | 1736.1 | 55 |
| H7B_5 | 2976.87 | 5444.38 | 2321.2 | 55 |
| H7C_5 | 3509.18 | 4810.09 | 2313.13 | 55 |
| H9_5 | 1954.5 | 8986.6 | 1466 | 23 |
| H10_5 | 1343.41 | 8760.27 | 2393.31 | 24 |
| H12A_5 | 638.86 | 6802.8 | 3445.29 | 43 |
| H12B_5 | 1290.25 | 6587.98 | 3087.2 | 43 |
| H12C_5 | 619.16 | 7175.36 | 2763.68 | 43 |
| H13A_5 | 478.77 | 8231.85 | 2937.62 | 48 |
| H13B_5 | 1042.99 | 8164.07 | 3389.52 | 48 |
| H13C_5 | 480.99 | 7700.56 | 3586.18 | 48 |
| H14A_5 | 2152.36 | 7401.4 | 3615.5 | 43 |
| H14B_5 | 2320.24 | 6803.03 | 3457.79 | 43 |
| H14C_5 | 1742.65 | 6821.36 | 3940.63 | 43 |
| H16_5 | 2288.14 | 7132.13 | 688.08 | 24 |
| H17_5 | 1277.17 | 6953.17 | 395.13 | 30 |
| H19_5 | -9.1 | 8361.52 | 746.02 | 70 |
| H20_5 | 1010.63 | 8544.11 | 1016.88 | 66 |
| H21A_5 | 2.26 | 7388.02 | 43.79 | 63 |
| H21B_5 | -516.67 | 7917.66 | 215.21 | 63 |
| H21C_5 | -302.3 | 7176.76 | 678.04 | 63 |
| H23_5 | 2287.62 | 8807.35 | 2.35 | 41 |
| H24_5 | 2882.27 | 9599.76 | -585.47 | 58 |
| H26A_5 | 3902.8 | 10357.34 | -183.18 | 112 |
| H26B_5 | 3694.02 | 10293.04 | -728.98 | 112 |
| H26C_5 | 4431.63 | 9853.08 | -379.08 | 112 |
| H27_5 | 4018.12 | 9321.88 | 770.23 | 36 |
| H28_5 | 3444.99 | 8492.68 | 1355.55 | 26 |
| H29A_5 | 1250.29 | 6744.61 | 1902.79 | 42 |
| H29B_5 | 1397.7 | 6004.73 | 2371.2 | 42 |
| H29C_5 | 1833.07 | 6202.44 | 1785.21 | 42 |
| H2_6 | 1396.41 | 8522.32 | 7178.86 | 23 |
| H3_6 | 696.76 | 9296.36 | 7545.76 | 24 |
| H5A_6 | 304.14 | 9752.49 | 8034.42 | 49 |
| H5B_6 | 1017.48 | 9958.78 | 8109.68 | 49 |
| H5C_6 | 372.49 | 9909.94 | 8558.54 | 49 |
| H6A_6 | 323.22 | 8771.31 | 9354.68 | 140 |
| H6B_6 | 661.42 | 8187.72 | 9189.33 | 140 |
| H6C_6 | 13.83 | 8764.7 | 8811 | 140 |
| H7A_6 | 1961.01 | 9212.42 | 8755.72 | 168 |
| H7B_6 | 1821.07 | 8554.35 | 9266.81 | 168 |
| H7C_6 | 1392.93 | 9237.97 | 9242.1 | 168 |
| H9_6 | 3117.31 | 6227.78 | 8045.65 | 21 |
| H10_6 | 3550.92 | 5390.54 | 9016.76 | 25 |
| H12A_6 | 3169.25 | 5829.18 | 10640.62 | 48 |
| H12B_6 | 2510.12 | 6259.86 | 10194.32 | 48 |
| H12C_6 | 3266.89 | 6478.94 | 10097.02 | 48 |
| H13A_6 | 4372.21 | 5838.66 | 9813.93 | 54 |
| H13B_6 | 4294.09 | 5224.23 | 9735.53 | 54 |
| H13C_6 | 4268.43 | 5192.68 | 10359.35 | 54 |
| H14A_6 | 3137.28 | 4877.18 | 9934.22 | 57 |
| H14B_6 | 2406.2 | 5332.17 | 10020.67 | 57 |

| | | | | |
|---|---|---|---|---|
| H14C_6 | 2992.58 | 4917.35 | 10529.77 | 57 |
| H16_6 | 2840.69 | 8729.92 | 7548.46 | 27 |
| H17_6 | 3887.4 | 9123.15 | 7281.44 | 34 |
| H19_6 | 4992.92 | 7404.58 | 7367.95 | 47 |
| H20_6 | 3941.54 | 7014.37 | 7611.06 | 39 |
| H21A_6 | 5143.62 | 9005.21 | 6898.42 | 59 |
| H21B_6 | 5604.34 | 8298.62 | 7003.73 | 59 |
| H21C_6 | 5460.17 | 8493.86 | 7521.53 | 59 |
| H23_6 | 2705.04 | 7798.54 | 6692.6 | 32 |
| H24_6 | 2089.54 | 7596.89 | 6081.4 | 47 |
| H26A_6 | 1128.58 | 6427.4 | 6468.31 | 97 |
| H26B_6 | 1276.13 | 7060.9 | 5928.82 | 97 |
| H26C_6 | 547.95 | 7103.33 | 6286.93 | 97 |
| H27_6 | 942.76 | 6582.13 | 7423.12 | 35 |
| H28_6 | 1522.79 | 6823.34 | 8010.48 | 27 |
| H29A_6 | 3536.51 | 7862.32 | 8691.99 | 55 |
| H29B_6 | 3709.64 | 7553.39 | 9360.31 | 55 |
| H29C_6 | 3242.29 | 8280.18 | 9039.46 | 55 |
| H51A_13 | 4349.88 | 10176.15 | 7428.14 | 108 |
| H51B_13 | 4819.35 | 9609.15 | 7966.86 | 108 |
| H51A_14 | 3967.08 | 6908.77 | 4030.45 | 75 |
| H51B_14 | 4263.94 | 6231.84 | 4569.47 | 75 |
| H51A_15 | -600.65 | 4184.39 | 8756.72 | 77 |
| H51B_15 | -837.94 | 4275.62 | 9322.65 | 77 |
| H51A_16 | -897.25 | 7475.45 | 2053.09 | 91 |
| H51B_16 | -1150.09 | 7599.04 | 2604.19 | 91 |
| H51A_17 | 5731.25 | 6546.17 | 9177.89 | 94 |
| H51B_17 | 5309.95 | 7111.2 | 8610.68 | 94 |
| H51A_18 | -587.39 | 947.77 | 5882.19 | 97 |
| H51B_18 | -169.69 | 947.95 | 5323.06 | 97 |

## 9.8 Single crystal structure analysis of 8a

CCDC Deposition number 2423156

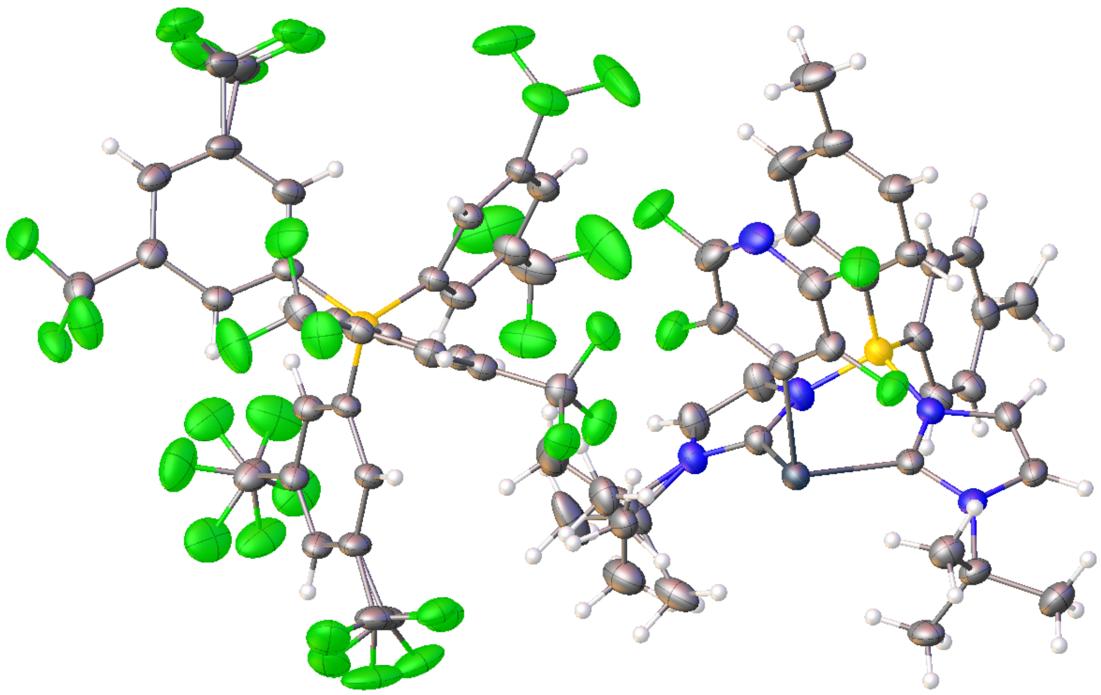

Figure S33. Olex-2 representation of the molecular structure of complex **8a**

### 9.8.1 Experimental

Single colourless plate-shaped crystals of **8a** were used as supplied.. A suitable crystal 0.10×0.07×0.03 mm$^3$ was selected and mounted on a MITIGEN holder in oil on a Rigaku FRE+ diffractometer with HF Varimax confocal mirrors, a UG2 goniometer and HyPix 6000HE detector. The crystal was kept at a steady $T$ = 100(2) K during data collection. The structure was solved with the ShelXT 2018/2 (Sheldrick, 2015) structure solution program using the using dual methods solution method and by using Olex2 1.5-alpha (Dolomanov et al., 2009) as the graphical interface. The model was refined with olex2.refine 1.5-alpha (Bourhis et al., 2015) using full matrix least squares minimisation on $\boldsymbol{F}^2$ minimisation.

### 9.8.2 Crystal data

$C_{66}H_{50}B_2Cl_2F_{28}N_5Sb$, $M_r$ = 1659.435, monoclinic, $P2_1/c$ (No. 14), a = 10.9725(2) Å, b = 29.0178(5) Å, c = 21.9761(3) Å, $\beta$ = 99.206(2)°, $\alpha = \gamma$ = 90°, $V$ = 6907.0(2) Å$^3$, $T$ = 100(2) K, $Z$ = 4, $Z'$ = 1, $\mu$(Mo $K_\alpha$) = 0.598 mm$^{-1}$, 198779 reflections measured, 25145 unique ($R_{int}$ = 0.0796) which were used in all calculations. The final $wR_2$ was 0.1450 (all data) and $R_1$ was 0.0541 (I≥2 $\sigma$(I)).

| Compound | **8a** |
|---|---|
| | |
| Formula | $C_{66}H_{50}B_2Cl_2F_{28}N_5Sb$ |
| $D_{calc.}$/ g cm$^{-3}$ | 1.596 |
| $\mu$/mm$^{-1}$ | 0.598 |
| Formula Weight | 1659.435 |
| Colour | colourless |
| Shape | plate-shaped |
| Size/mm$^3$ | 0.10×0.07×0.03 |
| $T$/K | 100(2) |
| Crystal System | monoclinic |
| Space Group | $P2_1/c$ |
| $a$/Å | 10.9725(2) |
| $b$/Å | 29.0178(5) |
| $c$/Å | 21.9761(3) |
| $\alpha$/° | 90 |
| $\beta$/° | 99.206(2) |
| $\gamma$/° | 90 |
| V/Å$^3$ | 6907.0(2) |
| $Z$ | 4 |
| $Z'$ | 1 |
| Wavelength/Å | 0.71073 |
| Radiation type | Mo $K_\alpha$ |
| $\Theta_{min}$/° | 1.69 |
| $\Theta_{max}$/° | 32.58 |
| Measured Refl's. | 198779 |
| Indep't Refl's | 25145 |
| Refl's I≥2 $\sigma$(I) | 17616 |
| $R_{int}$ | 0.0796 |
| Parameters | 1060 |
| Restraints | 882 |
| Largest Peak | 1.9386 |
| Deepest Hole | -1.3428 |
| GooF | 1.0262 |
| $wR_2$ (all data) | 0.1450 |
| $wR_2$ | 0.1288 |
| $R_1$ (all data) | 0.0851 |
| $R_1$ | 0.0541 |

### 9.8.3 Structure Quality Indicators

| | | | | |
|---|---|---|---|---|
| Reflections: | d min (MoKα) 2Θ=65.2° 0.66 | I/σ(I) 19.3 | Rint m=7.99 7.96% | Full 50.5° 100 |
| Refinement: | Shift 0.001 | Max Peak 1.9 | Min Peak -1.3 | GooF 1.026 |

A colourless plate-shaped crystal with dimensions 0.10×0.07×0.03 $mm^3$ was mounted on a MITIGEN holder in oil. X-ray diffraction data were collected using a Rigaku FRE+ diffractometer with HF Varimax confocal mirrors, a UG2 goniometer and HyPix 6000HE detector equipped with an Oxford Cryosystems low-temperature device, operating at $T$ = 100(2) K.

Data were measured using profile data from $\omega$-scans of 0.5 ° per frame for 7.0 s using Mo $K_\alpha$ radiation (Rotating Anode, 45.0 kV, 55.0 mA). The total number of runs and images was based on the strategy calculation from the program CrysAlisPro system (CCD 43.131a 64-bit (release 19-07-2024)). The maximum resolution achieved was $\Theta$ = 32.58°.

Cell parameters were retrieved using the CrysAlisPro 1.171.43.130a (Rigaku OD, 2024) software and refined using CrysAlisPro 1.171.43.130a (Rigaku OD, 2024) on 48186 reflections, 24 % of the observed reflections. Data reduction was performed using the CrysAlisPro 1.171.43.130a (Rigaku OD, 2024) software which corrects for Lorentz polarisation. The final completeness is 99.98 % out to 32.58° in $\Theta$.

A multi-scan absorption correction was performed using CrysAlisPro 1.171.43.130a (Rigaku OD, 2024). Empirical absorption correction using spherical harmonics, implemented in SCALE3 ABSPACK scaling algorithm. The absorption coefficient $\mu$ of this material is 0.598 $mm^{-1}$ at this wavelength ($\lambda$ = 0.71073Å) and the minimum and maximum transmissions are 0.764 and 1.000.

The structure was solved in the space group $P2_1/c$ (# 14) by using dual methods using the ShelXT 2018/2 (Sheldrick, 2015) structure solution program and refined by full matrix least squares minimisation on $F^2$ using olex2.refine 1.5-alpha (Bourhis et al., 2015). All non-hydrogen atoms were refined anisotropically. Hydrogen atom positions were calculated geometrically and refined using the riding model.

*_refine_special_details*: One tBu group and three CF3 groups are disordered (ca. 70:30, 59:41, 74:26, 87:13). 1, 2 and 1, 3 equal distance geometric restraints (SADI) applied to all chemically equivalent atom pairs of each disorder component. Thermal restraints (RIGU) applied to all disorder components. Solvent making applied to eliminate electronic contribution equivalent to one DCM molecule per formula unit.

*_exptl_absorpt_process_details*: CrysAlisPro 1.171.43.130a (Rigaku OD, 2024) using spherical harmonics, implemented in SCALE3 ABSPACK scaling algorithm.

*_smtbx_masks_special_details*: A solvent mask was calculated and 172 electrons were found in a volume of 588Å$^3$ in 1 void per unit cell. This is consistent with the presence of 1[CH2Cl2] per formula unit which account for 168 electrons per unit cell.

There is a single formula unit in the asymmetric unit, which is represented by the reported sum formula. In other words: Z is 4 and Z' is 1. The moiety formula is C33 H36 B F4 N5 Sb, C32 H12 B F24, 1[CH2Cl2].

## 9.8.4 Data Plots: Diffraction Data

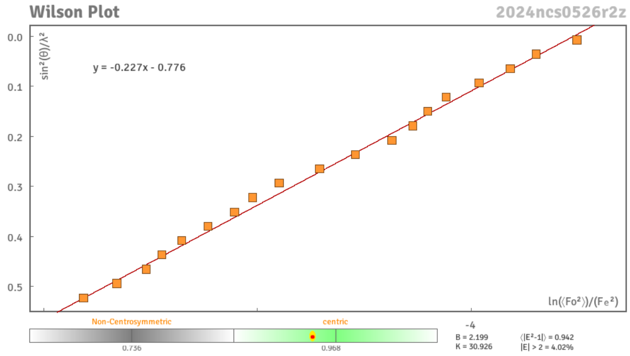


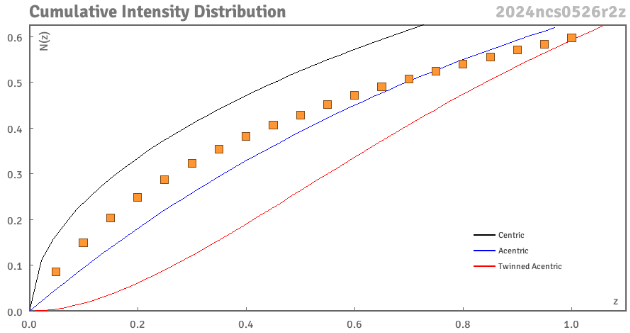


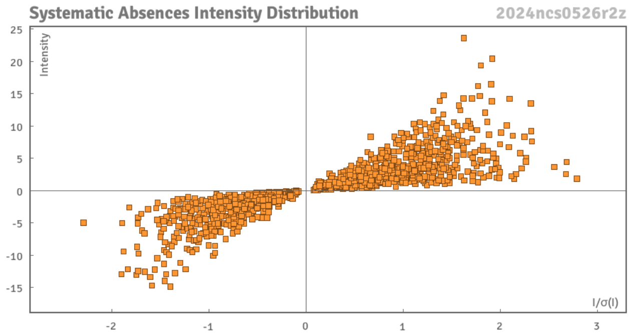


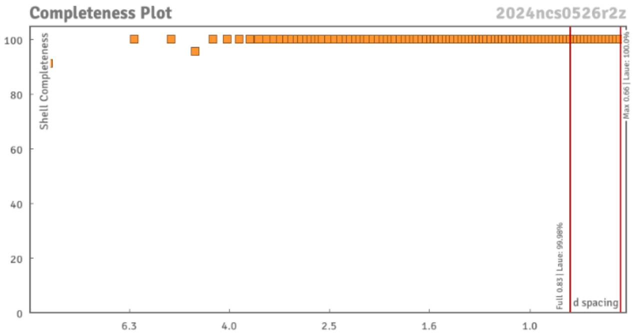


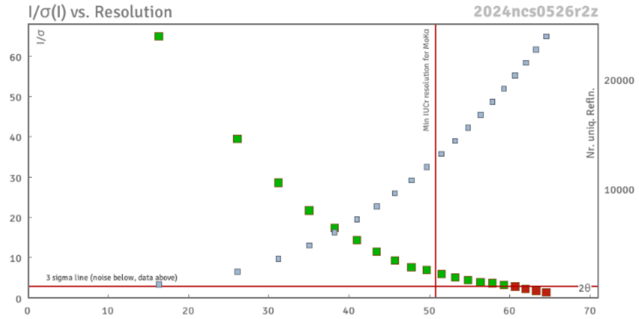


## 9.8.5 Data Plots: Refinement and Data

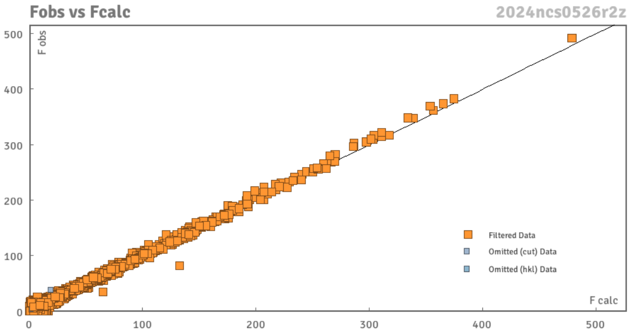


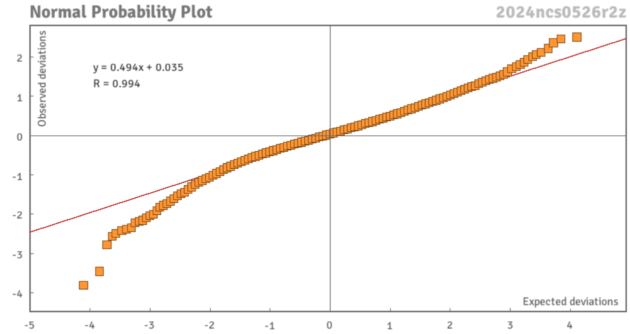

### 9.8.6 Reflection Statistics

| | | | |
|---|---|---|---|
| Total reflections (after filtering) | 200958 | Unique reflections | 25145 |
| Completeness | 1.0 | Mean I/$\sigma$ | 12.78 |
| $hkl_{max}$ collected | (16, 43, 33) | $hkl_{min}$ collected | (-16, -43, -33) |
| $hkl_{max}$ used | (16, 43, 33) | $hkl_{min}$ used | (-16, 0, 0) |
| Lim $d_{max}$ collected | 100.0 | Lim $d_{min}$ collected | 0.36 |
| $d_{max}$ used | 12.06 | $d_{min}$ used | 0.66 |
| Friedel pairs | 37601 | Friedel pairs merged | 1 |
| Inconsistent equivalents | 17 | $R_{int}$ | 0.0796 |
| $R_{sigma}$ | 0.0519 | Intensity transformed | 0 |
| Omitted reflections | 10 | Omitted by user (OMIT hkl) | 0 |
| Multiplicity | (30349, 27255, 14745, 7346, 3736, 1592, 952, 487, 239, 108, 37, 5) | Maximum multiplicity | 29 |
| Removed systematic absences | 2189 | Filtered off (Shel/OMIT) | 0 |

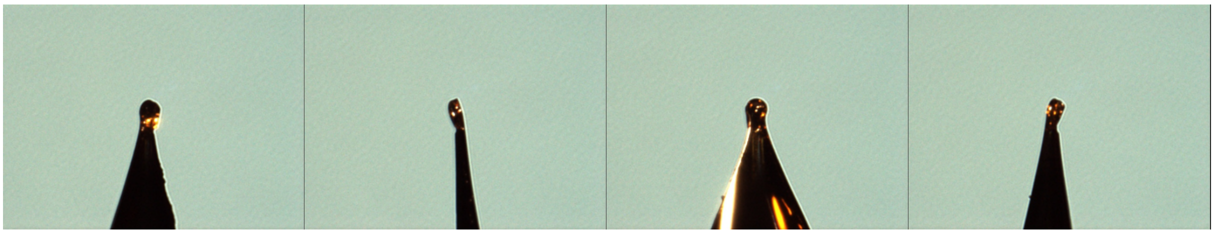

### 9.8.7 Fractional Atomic Coordinates and Equivalent Isotropic Displacement Parameters

Table S49. Fractional Atomic Coordinates (×10$^4$) and Equivalent Isotropic Displacement Parameters (Å$^2$×10$^3$) for **8a**. $U_{eq}$ is defined as 1/3 of the trace of the orthogonalised $U_{ij}$.

| Atom | x | y | z | $U_{eq}$ |
|---|---|---|---|---|
| Sb1 | 1065.54(13) | 4962.98(5) | 2755.74(7) | 27.19(5) |
| F1 | 2217.9(13) | 4082.0(5) | 2145.0(8) | 40.9(3) |
| F2 | 4536.7(15) | 3886.3(6) | 1966.8(9) | 50.7(4) |
| F3 | 3597.0(15) | 5593.1(6) | 2679.8(8) | 46.6(4) |
| F4 | 5817.4(15) | 5335.1(7) | 2435.2(10) | 58.0(5) |
| N1 | -234.9(18) | 5020.1(7) | 1399.4(9) | 27.6(4) |
| N2 | -935.9(17) | 4414.0(7) | 1838.4(9) | 28.4(4) |
| N3 | 585.0(19) | 5790.3(7) | 1871.4(9) | 31.5(4) |
| N4 | 934.6(19) | 6055.3(7) | 2818.8(9) | 31.6(4) |
| N5 | 5165.6(19) | 4615.1(9) | 2201.0(10) | 40.2(5) |
| C1 | -139.5(19) | 4776.9(8) | 1927.2(10) | 26.8(4) |
| C2 | -1077(2) | 4797.3(9) | 964.6(11) | 31.5(5) |
| C3 | -1505(2) | 4427.3(9) | 1233.6(11) | 33.4(5) |
| C4 | -1272(2) | 4090.1(9) | 2325.6(12) | 32.6(5) |
| C5 | -1800(2) | 4375.1(10) | 2804.3(12) | 39.2(6) |
| C6 | -2258(3) | 3756.7(11) | 2016.8(15) | 46.5(7) |
| C7 | -145(2) | 3815.3(9) | 2614.8(13) | 38.5(6) |
| C8 | 850(2) | 5666.7(8) | 2467.8(10) | 28.5(4) |
| C9 | 516(3) | 6264.0(9) | 1849.0(12) | 38.5(6) |
| C10 | 728(3) | 6425.6(9) | 2428.7(12) | 37.8(6) |
| C11a | 1046(8) | 6078(3) | 3508(4) | 39.4(14) |
| C11b | 1260(20) | 6123(8) | 3514(9) | 43(3) |
| C12a | 2187(5) | 5817.5(19) | 3809(2) | 42.0(12) |
| C12b | 2255(11) | 6514(4) | 3614(5) | 56(3) |
| C13a | 1113(6) | 6574.4(16) | 3716(2) | 63.7(17) |
| C13b | 1769(11) | 5707(4) | 3850(5) | 38(2) |
| C14a | -153(4) | 5852.7(17) | 3671.4(18) | 48.2(12) |
| C14b | 99(10) | 6283(4) | 3743(5) | 53(3) |
| C15 | -413(2) | 5759.4(8) | 726.5(10) | 30.4(5) |
| C16 | -16(2) | 5907.2(8) | 186.6(11) | 31.3(5) |
| C17 | -786(2) | 6151.4(9) | -264.0(11) | 34.0(5) |
| C18 | -1986(2) | 6261.7(8) | -194.2(11) | 32.3(5) |
| C19 | -2392(2) | 6117.9(9) | 346.3(11) | 34.8(5) |
| C20 | -1620(2) | 5873.9(9) | 788.1(11) | 34.6(5) |
| C21 | -2829(3) | 6529.5(9) | -677.3(12) | 39.9(6) |
| C22 | 1819(2) | 5364.5(8) | 1111.7(11) | 30.6(5) |
| C23 | 2133(2) | 4938.1(8) | 896.9(11) | 30.9(5) |
| C24 | 3314(2) | 4843.1(10) | 774.8(12) | 35.9(5) |
| C25 | 4226(2) | 5179.8(11) | 858.8(13) | 41.8(6) |
| C26 | 3911(3) | 5614.5(11) | 1039.6(15) | 47.8(7) |
| C27 | 2733(2) | 5704.9(9) | 1163.7(14) | 41.0(6) |
| C28 | 5542(3) | 5065.6(13) | 782.7(16) | 53.8(8) |
| C29 | 2800(2) | 4847.8(9) | 2424.2(11) | 29.7(5) |
| C30 | 3090(2) | 4411.8(9) | 2236.5(11) | 32.5(5) |
| C31 | 4267(2) | 4312.7(10) | 2138.7(12) | 37.8(5) |
| C32 | 3754(2) | 5162.1(9) | 2494.8(12) | 35.4(5) |
| C33 | 4904(2) | 5029.2(10) | 2370.7(14) | 41.3(6) |
| B1 | 470(2) | 5482.1(9) | 1259.5(12) | 28.7(5) |
| F51 | 10335.3(15) | 5670.8(7) | 5178.1(9) | 56.1(5) |
| F52 | 9496.1(19) | 5844.1(7) | 5960.1(8) | 62.1(5) |

| | | | | |
|---|---|---|---|---|
| F53 | 9522.1(17) | 5143.6(6) | 5652.3(8) | 53.2(4) |
| F54 | 4386.1(15) | 5112.2(6) | 3935.9(9) | 48.9(4) |
| F55 | 6002.0(17) | 4790.1(6) | 3688.4(7) | 47.6(4) |
| F56 | 5488.1(15) | 4657.6(5) | 4572.2(7) | 42.5(4) |
| F57 | 6331(2) | 6270.9(12) | 1590.4(9) | 101.4(10) |
| F58 | 7570.6(18) | 6034.7(6) | 2362.8(8) | 54.5(4) |
| F59 | 7786(2) | 6691.8(8) | 2028.4(13) | 85.3(7) |
| F60 | 2498.4(17) | 7423.5(8) | 2746.9(9) | 67.0(6) |
| F61 | 2807(3) | 7245.0(11) | 1859.4(11) | 106.5(10) |
| F62 | 3748(2) | 7814.8(9) | 2323.2(14) | 95.0(8) |
| F63a | 3825(3) | 8280.1(8) | 4538.8(13) | 71.5(8) |
| F63b | 3437(17) | 8202(5) | 5636(8) | 64(3) |
| F64a | 4652(2) | 8289.8(8) | 5474.6(15) | 68.6(7) |
| F64b | 2740(14) | 8188(5) | 4752(10) | 66(3) |
| F65a | 2709(2) | 8184.6(7) | 5230.7(16) | 64.3(7) |
| F65b | 4645(14) | 8353(5) | 4997(10) | 63(3) |
| F66a | 1990(5) | 6643.5(14) | 5909(3) | 71.7(15) |
| F66b | 1559(5) | 6606.9(17) | 5505(4) | 59.2(17) |
| F67a | 3673(5) | 6324.3(18) | 6256.2(16) | 61.2(12) |
| F67b | 3046(9) | 6517(3) | 6241(2) | 72(2) |
| F68a | 2631(11) | 6087(3) | 5415(6) | 49.1(19) |
| F68b | 2865(15) | 6049(4) | 5473(8) | 39.7(18) |
| F69 | 10646.7(16) | 7767.0(8) | 5968.5(9) | 62.9(5) |
| F70 | 9716(2) | 7165.3(7) | 6217.9(8) | 63.1(5) |
| F71 | 8809.1(16) | 7816.7(7) | 6150.7(8) | 54.0(5) |
| F72a | 11151(3) | 7677.5(16) | 3848.5(17) | 66.9(11) |
| F72b | 10679(11) | 7913(4) | 3727(5) | 72(3) |
| F73a | 9659(6) | 8147.4(10) | 3567(2) | 74.7(15) |
| F73b | 8879(11) | 8104(3) | 3328(5) | 62(2) |
| F74a | 9691(8) | 7480(3) | 3151(3) | 51.0(14) |
| F74b | 9440(20) | 7461(7) | 3105(9) | 48(3) |
| C51 | 6711.4(19) | 6220.0(8) | 4507.2(9) | 23.8(4) |
| C52 | 7811(2) | 6111.0(8) | 4897.2(10) | 26.3(4) |
| C53 | 8156(2) | 5658.2(8) | 5044.5(10) | 28.9(4) |
| C54 | 7433(2) | 5291.9(8) | 4810.9(11) | 30.6(5) |
| C55 | 6329(2) | 5389.4(8) | 4420.9(10) | 28.8(4) |
| C56 | 5986(2) | 5841.7(8) | 4275.0(10) | 26.4(4) |
| C57 | 5555(2) | 4991.0(8) | 4156.5(12) | 34.6(5) |
| C58 | 9369(3) | 5579.5(9) | 5456.5(12) | 38.1(5) |
| C59 | 5745(2) | 6773.6(8) | 3582.7(10) | 26.4(4) |
| C60 | 6395(2) | 6547.8(8) | 3169.5(10) | 28.2(4) |
| C61 | 6102(2) | 6598.6(8) | 2538.2(10) | 29.7(4) |
| C62 | 5100(2) | 6864.5(9) | 2281.1(11) | 34.4(5) |
| C63 | 4422(2) | 7082.1(10) | 2674.5(11) | 36.7(5) |
| C64 | 4735(2) | 7038.3(9) | 3314.2(11) | 33.0(5) |
| C65 | 3375(3) | 7392.9(12) | 2409.2(14) | 52.3(7) |
| C66 | 6927(2) | 6399.1(10) | 2127.0(11) | 37.2(5) |
| C67 | 5217.8(19) | 6930.5(8) | 4724.6(10) | 25.3(4) |
| C68 | 4520(2) | 6637.0(8) | 5037.3(10) | 27.7(4) |
| C69 | 3601(2) | 6804.7(9) | 5352.1(11) | 32.1(5) |
| C70 | 3347(2) | 7271.9(9) | 5367.1(11) | 32.3(5) |
| C71 | 4023(2) | 7568.7(8) | 5064.1(11) | 30.4(5) |
| C72 | 4942(2) | 7401.4(8) | 4748.7(11) | 28.5(4) |
| C73 | 3799(2) | 8076.4(9) | 5081.4(14) | 40.7(6) |
| C74a | 2981(8) | 6463(3) | 5727(4) | 42.0(14) |
| C74b | 2769(12) | 6502(4) | 5628(6) | 40.7(17) |

| | | | | |
|---|---|---|---|---|
| C75 | 7482.9(19) | 7094.2(7) | 4466.8(10) | 25.1(4) |
| C76 | 7979(2) | 7206.4(8) | 5073.6(10) | 27.1(4) |
| C77 | 9072(2) | 7457.9(8) | 5223.7(11) | 30.8(5) |
| C78 | 9697(2) | 7619.2(9) | 4767.9(13) | 36.9(5) |
| C79 | 9203(3) | 7521.1(9) | 4161.9(13) | 37.9(5) |
| C80 | 8130(2) | 7266.2(8) | 4014.8(11) | 31.4(5) |
| C81 | 9557(2) | 7549.7(10) | 5888.1(13) | 38.5(5) |
| C82a | 9908(6) | 7707(2) | 3687(3) | 47.3(11) |
| C82b | 9558(17) | 7739(6) | 3567(8) | 54(2) |
| B51 | 6272(2) | 6752.5(9) | 4324.1(11) | 24.2(4) |

### 9.8.8 Anisotropic Displacement Parameters

Table S50. Anisotropic Displacement Parameters (×10[4]) for **8a**. The anisotropic displacement factor exponent takes the form: $-2\pi^2[h^2a^{*2} \times U_{11} + ... +2hka^* \times b^* \times U_{12}]$

| Atom | $U_{11}$ | $U_{22}$ | $U_{33}$ | $U_{23}$ | $U_{13}$ | $U_{12}$ |
|---|---|---|---|---|---|---|
| Sb1 | 25.25(8) | 30.27(8) | 25.83(7) | 2.05(6) | 3.39(5) | -0.93(6) |
| F1 | 27.5(7) | 39.0(8) | 56.3(9) | -1.0(6) | 7.1(6) | -10.6(7) |
| F2 | 34.1(8) | 52.9(10) | 65.7(11) | 13.6(7) | 9.8(7) | -13.7(8) |
| F3 | 37.0(8) | 40.6(9) | 61.1(11) | -5.8(7) | 4.3(7) | -8.2(8) |
| F4 | 30.5(8) | 63.4(12) | 79.7(13) | -13.6(8) | 7.6(8) | 1.3(10) |
| N1 | 24.2(9) | 34.6(10) | 24.6(9) | 1.0(7) | 5.7(7) | -3.6(7) |
| N2 | 21.8(8) | 33.3(10) | 31.5(10) | -0.4(7) | 8.4(7) | -4.5(8) |
| N3 | 37.0(11) | 29.3(10) | 27.2(9) | 2.3(8) | 2.3(8) | -1.5(7) |
| N4 | 33.9(10) | 31.8(10) | 28.4(9) | 3.3(8) | 3.4(7) | -3.2(7) |
| N5 | 23.5(10) | 57.6(15) | 39.0(12) | 3.0(9) | 3.4(8) | 0.6(10) |
| C1 | 20.4(9) | 32.8(11) | 27.9(10) | 1.2(8) | 6.3(8) | -3.6(8) |
| C2 | 26.1(11) | 40.0(13) | 28.2(11) | -0.8(9) | 4.2(8) | -7.6(9) |
| C3 | 23.4(10) | 42.6(13) | 35.0(12) | -3.0(9) | 7.3(9) | -9.7(10) |
| C4 | 25.6(11) | 36.6(12) | 37.5(12) | -3.5(9) | 10.5(9) | -0.2(10) |
| C5 | 27.6(12) | 55.1(16) | 38.0(13) | 2.3(11) | 14.6(10) | -0.4(12) |
| C6 | 36.8(14) | 48.8(16) | 54.4(17) | -12.8(12) | 9.1(12) | -0.1(13) |
| C7 | 36.0(13) | 35.8(13) | 44.3(14) | 0.5(10) | 8.4(11) | 2.5(11) |
| C8 | 27.2(10) | 29.8(11) | 28.4(11) | 1.9(8) | 4.2(8) | -2.6(8) |
| C9 | 52.1(15) | 30.0(12) | 31.1(12) | 4.2(11) | -0.8(11) | 1.5(9) |
| C10 | 47.9(15) | 27.9(12) | 35.9(13) | 4.3(10) | 1.8(11) | -2.9(10) |
| C11a | 49(3) | 41(2) | 27(2) | 13.9(17) | 3.6(14) | -5.8(13) |
| C11b | 52(4) | 52(5) | 23(5) | 7(2) | 3.4(17) | -6(2) |
| C12a | 41(3) | 49(3) | 33(2) | 5.5(18) | -5.2(18) | -6.6(17) |
| C12b | 66(6) | 64(6) | 35(5) | -17(3) | 0(3) | -3(3) |
| C13a | 112(5) | 41(2) | 35(2) | 19(2) | -1(2) | -12.9(15) |
| C13b | 33(5) | 52(5) | 27(4) | -4(3) | 3(3) | -4(3) |
| C14a | 48(2) | 67(3) | 30.6(19) | 25.2(17) | 10.4(14) | 6.2(17) |
| C14b | 48(5) | 74(7) | 39(5) | 12(3) | 11(3) | -1(4) |
| C15 | 35.1(12) | 31.8(11) | 24.4(10) | 3.3(9) | 5.5(9) | -3.4(8) |
| C16 | 33.8(12) | 32.1(12) | 28.6(10) | 1.2(9) | 7.2(9) | -2.8(9) |
| C17 | 42.6(13) | 32.6(12) | 27.6(11) | -1.6(10) | 8.0(10) | -0.1(9) |
| C18 | 41.0(13) | 26.6(11) | 28.1(11) | 1.2(9) | 1.8(9) | -2.8(9) |
| C19 | 33.8(12) | 39.6(13) | 31.2(12) | 8.6(10) | 6.2(9) | -2.0(10) |
| C20 | 37.1(13) | 43.0(14) | 24.5(11) | 6.7(10) | 7.5(9) | 0.1(9) |
| C21 | 44.6(15) | 37.5(13) | 35.2(13) | 2.0(11) | -0.6(11) | 3.6(10) |
| C22 | 30.0(11) | 32.9(12) | 28.6(11) | -0.3(9) | 4.1(9) | 3.7(9) |
| C23 | 27.6(11) | 36.1(12) | 30.2(11) | -1.5(9) | 8.7(8) | 0.4(9) |
| C24 | 30.9(12) | 42.1(14) | 36.7(13) | 1.5(10) | 11.4(10) | 1.5(11) |
| C25 | 29.2(12) | 57.2(17) | 40.8(14) | -1.1(11) | 11.4(10) | 10.8(12) |
| C26 | 35.8(14) | 47.5(16) | 59.8(18) | -11.0(12) | 7.0(12) | 12.3(14) |
| C27 | 35.4(13) | 33.0(13) | 54.6(16) | -5.1(10) | 7.3(11) | 3.9(11) |
| C28 | 30.0(13) | 79(2) | 55.5(18) | 0.6(13) | 14.6(12) | 13.6(16) |
| C29 | 22.4(10) | 38.2(12) | 27.4(11) | 1.7(9) | 1.0(8) | -0.5(9) |
| C30 | 22.9(10) | 40.6(13) | 33.1(12) | 1.1(9) | 2.1(8) | -2.9(10) |
| C31 | 27.8(11) | 47.5(15) | 37.2(13) | 6.2(10) | 2.2(9) | -2.8(11) |
| C32 | 28.7(12) | 37.6(13) | 38.8(13) | -1.7(10) | 2.3(10) | -1.1(10) |
| C33 | 25.7(11) | 54.5(17) | 42.6(14) | -5.7(11) | 2.1(10) | 4.3(12) |
| B1 | 31.1(12) | 29.4(12) | 25.7(11) | 0.1(10) | 5.2(9) | -1.6(9) |
| F51 | 27.8(8) | 77.1(13) | 59.7(11) | 5.1(8) | -4.1(7) | 11.2(9) |

| | | | | | | |
|---|---|---|---|---|---|---|
| F52 | 70.8(12) | 64.8(12) | 40.9(9) | 27.8(9) | -21.0(8) | -12.0(8) |
| F53 | 57.3(11) | 45.5(9) | 50.5(10) | 16.5(8) | -10.5(8) | 13.1(7) |
| F54 | 36.3(8) | 34.9(8) | 70.5(12) | 1.1(6) | -6.5(7) | -3.1(8) |
| F55 | 61.2(11) | 42.3(9) | 39.8(8) | 0.4(8) | 9.9(7) | -11.1(7) |
| F56 | 50.4(9) | 29.2(7) | 48.8(9) | -2.5(6) | 10.7(7) | 3.2(6) |
| F57 | 58.9(13) | 201(3) | 41.0(10) | 38.0(15) | -3.8(9) | -51.4(13) |
| F58 | 62.6(11) | 54.2(10) | 54.0(10) | 10.9(8) | 31.8(8) | -3.2(8) |
| F59 | 81.8(14) | 60.1(13) | 135(2) | -8.9(10) | 82.0(14) | -1.4(12) |
| F60 | 45.2(10) | 95.8(16) | 61.4(11) | 33.4(10) | 12.8(8) | 28.3(10) |
| F61 | 96.7(18) | 156(3) | 54.4(13) | 67.5(17) | -25.9(11) | -6.6(13) |
| F62 | 67.4(14) | 72.9(14) | 151(2) | 32.3(10) | 37.3(14) | 60.1(14) |
| F63a | 101.4(19) | 39.0(12) | 80.8(16) | 25.9(11) | 34.9(11) | 9.6(9) |
| F63b | 73(5) | 31(6) | 92(4) | 11(2) | 30.1(17) | -11(2) |
| F64a | 58.2(13) | 39.9(12) | 104(2) | -0.9(9) | 1.7(11) | -23.3(11) |
| F64b | 62(4) | 37(6) | 102(4) | 17(2) | 18.8(17) | -7(2) |
| F65a | 41.9(11) | 41.6(12) | 118(2) | 10.5(8) | 37.5(11) | -10.4(12) |
| F65b | 51(4) | 37(6) | 107(4) | 14(2) | 28.9(18) | -7(2) |
| F66a | 65(2) | 56(2) | 112(4) | 9.0(16) | 71(2) | 12(2) |
| F66b | 31(2) | 45(2) | 110(5) | 1.6(13) | 36.6(17) | 7(2) |
| F67a | 73(3) | 69(3) | 45.1(17) | -16.9(17) | 17.8(14) | 12.9(13) |
| F67b | 96(5) | 85(4) | 41(2) | -35(3) | 33.8(17) | -3.2(16) |
| F68a | 46(5) | 47(3) | 59(3) | -8(2) | 25(2) | -2.2(18) |
| F68b | 26(4) | 37(3) | 60(4) | 2.1(15) | 19(2) | 3.1(17) |
| F69 | 35.9(9) | 87.9(15) | 62.9(12) | -16.4(8) | 1.8(8) | -23.4(10) |
| F70 | 86.3(14) | 53.3(11) | 42.5(10) | 5.3(9) | -11.8(9) | -2.8(8) |
| F71 | 41.7(9) | 72.5(12) | 47.5(9) | 10.0(8) | 5.9(7) | -26.0(8) |
| F72a | 51.3(17) | 92(3) | 66.9(18) | -31.2(13) | 37.6(12) | -25.1(16) |
| F72b | 64(4) | 82(6) | 78(5) | -33(2) | 33(2) | 7(3) |
| F73a | 130(4) | 39.2(14) | 72(2) | -10.9(14) | 67(2) | 1.7(11) |
| F73b | 78(5) | 52(4) | 67(4) | -10(2) | 44(3) | 12(2) |
| F74a | 57(3) | 58(2) | 47.7(19) | -17.8(18) | 34.8(17) | -12.3(13) |
| F74b | 51(7) | 48(5) | 51(4) | -11(3) | 27(3) | 7(2) |
| C51 | 22.7(9) | 30.4(10) | 20.3(9) | 4.0(7) | 9.0(7) | 0.6(7) |
| C52 | 26.3(10) | 30.5(10) | 23.1(9) | 3.0(7) | 6.8(7) | 1.5(8) |
| C53 | 29.3(10) | 34.6(11) | 23.3(10) | 7.4(8) | 6.1(7) | 3.3(8) |
| C54 | 33.9(11) | 29.7(11) | 29.5(11) | 6.2(8) | 8.9(8) | 3.6(8) |
| C55 | 29.6(11) | 30.7(11) | 27.9(10) | 3.8(7) | 9.8(8) | -0.2(8) |
| C56 | 23.0(10) | 30.8(10) | 26.5(10) | 4.8(7) | 7.0(7) | 0.6(8) |
| C57 | 35.5(12) | 29.4(11) | 38.1(12) | 3.2(8) | 3.9(9) | -0.2(8) |
| C58 | 39.6(13) | 39.5(13) | 32.8(12) | 10.0(9) | -1.5(9) | 1.9(9) |
| C59 | 26.1(10) | 30.7(11) | 23.4(9) | 3.2(8) | 7.5(7) | 2.6(7) |
| C60 | 25.0(10) | 33.8(11) | 26.6(10) | 2.2(8) | 6.6(7) | -0.4(8) |
| C61 | 29.1(11) | 36.4(12) | 24.9(10) | -2.3(8) | 8.3(7) | -1.8(8) |
| C62 | 34.0(12) | 46.0(14) | 23.2(10) | 0.5(9) | 4.4(8) | 4.6(9) |
| C63 | 34.4(12) | 47.1(14) | 28.4(11) | 9.5(9) | 4.2(8) | 8.2(9) |
| C64 | 31.1(11) | 41.7(13) | 27.6(11) | 9.4(9) | 9.1(8) | 4.2(9) |
| C65 | 48.0(16) | 72(2) | 37.6(14) | 24.2(12) | 7.4(10) | 19.4(12) |
| C66 | 34.9(12) | 52.9(15) | 26.0(11) | -0.9(9) | 11.6(8) | -2.4(9) |
| C67 | 22.9(9) | 31.2(10) | 23.1(9) | 3.9(7) | 7.4(7) | -0.4(7) |
| C68 | 27.7(10) | 30.4(11) | 26.9(10) | 1.2(8) | 10.2(7) | -3.5(8) |
| C69 | 29.5(11) | 35.7(11) | 34.9(11) | 0.3(8) | 16.3(8) | -3.8(8) |
| C70 | 25.0(10) | 36.0(12) | 38.6(12) | 1.7(8) | 13.4(9) | -9.8(9) |
| C71 | 25.1(10) | 32.0(11) | 35.4(12) | 3.1(8) | 8.6(8) | -7.1(8) |
| C72 | 24.4(10) | 32.3(11) | 30.1(11) | 3.7(8) | 8.0(8) | 0.0(8) |
| C73 | 31.6(12) | 33.8(12) | 59.8(15) | 3.7(8) | 16.5(9) | -7.2(9) |
| C74a | 42(2) | 43(3) | 49(3) | -1.4(12) | 29.3(14) | -1.8(12) |

| | | | | | | |
|---|---|---|---|---|---|---|
| C74b | 42(3) | 41(3) | 47(3) | -0.3(12) | 29.8(14) | -3.8(14) |
| C75 | 23.8(10) | 24.9(10) | 28.7(10) | 4.9(7) | 10.7(7) | 1.9(7) |
| C76 | 25.0(10) | 28.7(10) | 29.2(10) | 4.1(8) | 9.6(7) | 0.9(8) |
| C77 | 26.8(10) | 28.6(11) | 38.5(12) | 4.3(8) | 9.4(8) | -6.7(8) |
| C78 | 30.2(11) | 31.3(12) | 53.7(14) | -3.4(9) | 19.8(9) | -9.7(10) |
| C79 | 44.0(13) | 31.5(12) | 44.9(13) | -6.4(9) | 27.5(9) | -6.0(8) |
| C80 | 38.8(12) | 26.6(11) | 32.3(11) | -0.3(8) | 16.5(9) | -1.2(8) |
| C81 | 26.6(11) | 43.7(14) | 44.3(14) | 1.9(9) | 3.2(9) | -10.1(10) |
| C82a | 61(3) | 39.9(19) | 50(2) | -19.8(13) | 34.1(13) | -11.5(12) |
| C82b | 61(3) | 51(4) | 58(3) | -21.8(16) | 32.4(14) | -0.5(16) |
| B51 | 22.9(10) | 29.6(11) | 21.3(10) | 4.7(8) | 7.3(7) | 0.9(8) |

9.8.9 Bond lengths

Table S51. Bond Lengths in Å for **8a**.

| Atom | Atom | Length/Å |
|---|---|---|
| Sb1 | C1 | 2.141(2) |
| Sb1 | C8 | 2.140(2) |
| Sb1 | C29 | 2.170(2) |
| F1 | C30 | 1.346(3) |
| F2 | C31 | 1.341(3) |
| F3 | C32 | 1.335(3) |
| F4 | C33 | 1.330(3) |
| N1 | C1 | 1.347(3) |
| N1 | C2 | 1.379(3) |
| N1 | B1 | 1.602(3) |
| N2 | C1 | 1.362(3) |
| N2 | C3 | 1.376(3) |
| N2 | C4 | 1.514(3) |
| N3 | C8 | 1.345(3) |
| N3 | C9 | 1.377(3) |
| N3 | B1 | 1.603(3) |
| N4 | C8 | 1.361(3) |
| N4 | C10 | 1.371(3) |
| N4 | C11a | 1.502(8) |
| N4 | C11b | 1.525(19) |
| N5 | C31 | 1.310(4) |
| N5 | C33 | 1.304(4) |
| C2 | C3 | 1.346(4) |
| C4 | C5 | 1.523(4) |
| C4 | C6 | 1.527(4) |
| C4 | C7 | 1.523(4) |
| C9 | C10 | 1.343(4) |
| C11a | C12a | 1.520(10) |
| C11a | C13a | 1.509(9) |
| C11a | C14a | 1.562(9) |
| C11b | C12b | 1.57(2) |
| C11b | C13b | 1.48(2) |
| C11b | C14b | 1.51(2) |
| C15 | C16 | 1.395(3) |
| C15 | C20 | 1.393(3) |
| C15 | B1 | 1.611(3) |
| C16 | C17 | 1.389(3) |
| C17 | C18 | 1.386(4) |
| C18 | C19 | 1.397(4) |
| C18 | C21 | 1.508(3) |
| C19 | C20 | 1.378(3) |
| C22 | C23 | 1.387(3) |
| C22 | C27 | 1.399(3) |
| C22 | B1 | 1.602(4) |
| C23 | C24 | 1.392(3) |
| C24 | C25 | 1.390(4) |
| C25 | C26 | 1.383(4) |
| C25 | C28 | 1.516(4) |
| C26 | C27 | 1.387(4) |
| C29 | C30 | 1.384(3) |
| C29 | C32 | 1.378(3) |
| C30 | C31 | 1.373(3) |
| C32 | C33 | 1.388(4) |
| F51 | C58 | 1.333(3) |
| F52 | C58 | 1.336(3) |
| F53 | C58 | 1.338(3) |
| F54 | C57 | 1.344(3) |
| F55 | C57 | 1.341(3) |
| F56 | C57 | 1.341(3) |
| F57 | C66 | 1.307(3) |
| F58 | C66 | 1.330(3) |
| F59 | C66 | 1.312(3) |
| F60 | C65 | 1.309(4) |
| F61 | C65 | 1.338(4) |
| F62 | C65 | 1.314(4) |
| F63a | C73 | 1.335(4) |
| F63b | C73 | 1.389(15) |
| F64a | C73 | 1.322(4) |
| F64b | C73 | 1.308(15) |
| F65a | C73 | 1.328(3) |
| F65b | C73 | 1.263(15) |
| F66a | C74a | 1.324(10) |
| F66b | C74b | 1.347(12) |
| F67a | C74a | 1.345(9) |
| F67b | C74b | 1.334(13) |
| F68a | C74a | 1.314(14) |
| F68b | C74b | 1.365(17) |
| F69 | C81 | 1.338(3) |
| F70 | C81 | 1.326(3) |
| F71 | C81 | 1.326(3) |
| F72a | C82a | 1.356(6) |
| F72b | C82b | 1.324(17) |
| F73a | C82a | 1.323(6) |
| F73b | C82b | 1.351(18) |
| F74a | C82a | 1.337(7) |
| F74b | C82b | 1.29(2) |
| C51 | C52 | 1.400(3) |
| C51 | C56 | 1.403(3) |
| C51 | B51 | 1.649(3) |
| C52 | C53 | 1.391(3) |
| C53 | C54 | 1.376(3) |
| C53 | C58 | 1.502(3) |
| C54 | C55 | 1.396(3) |
| C55 | C56 | 1.389(3) |
| C55 | C57 | 1.496(3) |
| C59 | C60 | 1.404(3) |
| C59 | C64 | 1.399(3) |
| C59 | B51 | 1.640(3) |
| C60 | C61 | 1.381(3) |
| C61 | C62 | 1.387(3) |
| C61 | C66 | 1.494(3) |
| C62 | C63 | 1.380(4) |
| C63 | C64 | 1.399(3) |
| C63 | C65 | 1.503(4) |
| C67 | C68 | 1.397(3) |

| | | |
|---|---|---|
| C67 | C72 | 1.402(3) |
| C67 | B51 | 1.644(3) |
| C68 | C69 | 1.398(3) |
| C69 | C70 | 1.386(3) |
| C69 | C74a | 1.517(10) |
| C69 | C74b | 1.467(13) |
| C70 | C71 | 1.376(3) |
| C71 | C72 | 1.398(3) |
| C71 | C73 | 1.495(3) |
| C75 | C76 | 1.396(3) |

| | | |
|---|---|---|
| C75 | C80 | 1.402(3) |
| C75 | B51 | 1.648(3) |
| C76 | C77 | 1.397(3) |
| C77 | C78 | 1.383(4) |
| C77 | C81 | 1.496(4) |
| C78 | C79 | 1.385(4) |
| C79 | C80 | 1.384(4) |
| C79 | C82a | 1.496(6) |
| C79 | C82b | 1.557(17) |

9.8.10 Bond Angles

Table S52. Bond Angles in ° for **8a**.

| Atom | Atom | Atom | Angle/° |
|---|---|---|---|
| C8 | Sb1 | C1 | 88.00(9) |
| C29 | Sb1 | C1 | 97.53(8) |
| C29 | Sb1 | C8 | 96.26(9) |
| C2 | N1 | C1 | 107.6(2) |
| B1 | N1 | C1 | 129.06(19) |
| B1 | N1 | C2 | 123.36(19) |
| C3 | N2 | C1 | 107.4(2) |
| C4 | N2 | C1 | 127.01(19) |
| C4 | N2 | C3 | 125.18(19) |
| C9 | N3 | C8 | 107.6(2) |
| B1 | N3 | C8 | 130.1(2) |
| B1 | N3 | C9 | 122.0(2) |
| C10 | N4 | C8 | 107.79(19) |
| C11a | N4 | C8 | 126.5(4) |
| C11a | N4 | C10 | 125.1(4) |
| C11b | N4 | C8 | 131.2(9) |
| C11b | N4 | C10 | 121.0(9) |
| C11b | N4 | C11a | 9.9(10) |
| C33 | N5 | C31 | 116.8(2) |
| N1 | C1 | Sb1 | 123.53(16) |
| N2 | C1 | Sb1 | 127.69(17) |
| N2 | C1 | N1 | 108.77(19) |
| C3 | C2 | N1 | 108.2(2) |
| C2 | C3 | N2 | 108.1(2) |
| C5 | C4 | N2 | 108.3(2) |
| C6 | C4 | N2 | 108.3(2) |
| C6 | C4 | C5 | 109.3(2) |
| C7 | C4 | N2 | 110.27(19) |
| C7 | C4 | C5 | 111.7(2) |
| C7 | C4 | C6 | 108.9(2) |
| N3 | C8 | Sb1 | 122.63(16) |
| N4 | C8 | Sb1 | 128.96(17) |
| N4 | C8 | N3 | 108.4(2) |
| C10 | C9 | N3 | 108.3(2) |
| C9 | C10 | N4 | 107.8(2) |
| C12a | C11a | N4 | 110.2(6) |
| C13a | C11a | N4 | 109.8(6) |
| C13a | C11a | C12a | 110.2(6) |
| C14a | C11a | N4 | 106.1(5) |
| C14a | C11a | C12a | 110.9(6) |
| C14a | C11a | C13a | 109.4(6) |
| C12b | C11b | N4 | 106.1(13) |
| C13b | C11b | N4 | 113.8(15) |
| C13b | C11b | C12b | 108.6(15) |
| C14b | C11b | N4 | 107.5(14) |
| C14b | C11b | C12b | 109.6(15) |
| C14b | C11b | C13b | 111.1(15) |
| C20 | C15 | C16 | 116.1(2) |
| B1 | C15 | C16 | 122.6(2) |
| B1 | C15 | C20 | 121.2(2) |
| C17 | C16 | C15 | 121.8(2) |
| C18 | C17 | C16 | 121.2(2) |
| C19 | C18 | C17 | 117.6(2) |
| C21 | C18 | C17 | 121.8(2) |
| C21 | C18 | C19 | 120.6(2) |
| C20 | C19 | C18 | 120.6(2) |
| C19 | C20 | C15 | 122.8(2) |
| C27 | C22 | C23 | 116.3(2) |
| B1 | C22 | C23 | 123.1(2) |
| B1 | C22 | C27 | 120.4(2) |
| C24 | C23 | C22 | 122.1(2) |
| C25 | C24 | C23 | 120.5(3) |
| C26 | C25 | C24 | 118.1(3) |
| C28 | C25 | C24 | 120.7(3) |
| C28 | C25 | C26 | 121.2(3) |
| C27 | C26 | C25 | 120.8(3) |
| C26 | C27 | C22 | 121.9(3) |
| C30 | C29 | Sb1 | 119.81(18) |
| C32 | C29 | Sb1 | 123.45(19) |
| C32 | C29 | C30 | 115.6(2) |
| C29 | C30 | F1 | 120.2(2) |
| C31 | C30 | F1 | 119.5(2) |
| C31 | C30 | C29 | 120.4(2) |
| N5 | C31 | F2 | 116.8(2) |
| C30 | C31 | F2 | 119.5(2) |
| C30 | C31 | N5 | 123.7(3) |
| C29 | C32 | F3 | 121.5(2) |
| C33 | C32 | F3 | 119.1(2) |
| C33 | C32 | C29 | 119.5(3) |
| N5 | C33 | F4 | 116.7(2) |
| C32 | C33 | F4 | 119.2(3) |
| C32 | C33 | N5 | 124.2(2) |
| N3 | B1 | N1 | 106.37(18) |
| C15 | B1 | N1 | 107.78(19) |
| C15 | B1 | N3 | 106.79(19) |
| C22 | B1 | N1 | 110.52(19) |
| C22 | B1 | N3 | 109.72(19) |
| C22 | B1 | C15 | 115.2(2) |
| C56 | C51 | C52 | 115.4(2) |
| B51 | C51 | C52 | 123.45(19) |
| B51 | C51 | C56 | 121.16(18) |
| C53 | C52 | C51 | 122.2(2) |
| C54 | C53 | C52 | 121.5(2) |
| C58 | C53 | C52 | 117.8(2) |
| C58 | C53 | C54 | 120.7(2) |
| C55 | C54 | C53 | 117.7(2) |
| C56 | C55 | C54 | 120.6(2) |
| C57 | C55 | C54 | 117.7(2) |
| C57 | C55 | C56 | 121.7(2) |
| C55 | C56 | C51 | 122.6(2) |
| F55 | C57 | F54 | 106.4(2) |
| F56 | C57 | F54 | 106.3(2) |
| F56 | C57 | F55 | 106.00(19) |
| C55 | C57 | F54 | 112.81(19) |
| C55 | C57 | F55 | 112.3(2) |

| | | | |
|---|---|---|---|
| C55 | C57 | F56 | 112.5(2) |
| F52 | C58 | F51 | 106.3(2) |
| F53 | C58 | F51 | 105.5(2) |
| F53 | C58 | F52 | 106.6(2) |
| C53 | C58 | F51 | 112.7(2) |
| C53 | C58 | F52 | 112.1(2) |
| C53 | C58 | F53 | 113.0(2) |
| C64 | C59 | C60 | 115.7(2) |
| B51 | C59 | C60 | 119.36(19) |
| B51 | C59 | C64 | 124.7(2) |
| C61 | C60 | C59 | 122.6(2) |
| C62 | C61 | C60 | 120.7(2) |
| C66 | C61 | C60 | 120.0(2) |
| C66 | C61 | C62 | 119.1(2) |
| C63 | C62 | C61 | 118.1(2) |
| C64 | C63 | C62 | 121.2(2) |
| C65 | C63 | C62 | 119.1(2) |
| C65 | C63 | C64 | 119.6(2) |
| C63 | C64 | C59 | 121.6(2) |
| F61 | C65 | F60 | 104.8(3) |
| F62 | C65 | F60 | 107.0(3) |
| F62 | C65 | F61 | 106.2(3) |
| C63 | C65 | F60 | 114.0(2) |
| C63 | C65 | F61 | 112.0(3) |
| C63 | C65 | F62 | 112.2(3) |
| F58 | C66 | F57 | 106.4(3) |
| F59 | C66 | F57 | 107.7(3) |
| F59 | C66 | F58 | 103.2(2) |
| C61 | C66 | F57 | 113.1(2) |
| C61 | C66 | F58 | 114.1(2) |
| C61 | C66 | F59 | 111.7(2) |
| C72 | C67 | C68 | 115.9(2) |
| B51 | C67 | C68 | 124.0(2) |
| B51 | C67 | C72 | 120.1(2) |
| C69 | C68 | C67 | 121.8(2) |
| C70 | C69 | C68 | 121.1(2) |
| C74a | C69 | C68 | 117.6(4) |
| C74a | C69 | C70 | 121.0(4) |
| C74b | C69 | C68 | 122.8(5) |
| C74b | C69 | C70 | 115.8(5) |
| C74b | C69 | C74a | 11.8(7) |
| C71 | C70 | C69 | 118.3(2) |
| C72 | C71 | C70 | 120.7(2) |
| C73 | C71 | C70 | 120.0(2) |
| C73 | C71 | C72 | 119.3(2) |
| C71 | C72 | C67 | 122.3(2) |
| F63b | C73 | F63a | 135.8(6) |
| F64a | C73 | F63a | 105.2(3) |
| F64a | C73 | F63b | 64.9(8) |
| F64b | C73 | F63a | 62.6(9) |
| F64b | C73 | F63b | 93.9(11) |
| F64b | C73 | F64a | 136.0(7) |
| F65a | C73 | F63a | 105.4(3) |
| F65a | C73 | F63b | 47.6(7) |
| F65a | C73 | F64a | 107.4(3) |
| F65a | C73 | F64b | 47.4(9) |

| | | | |
|---|---|---|---|
| F65b | C73 | F63a | 57.7(10) |
| F65b | C73 | F63b | 106.3(11) |
| F65b | C73 | F64a | 48.6(10) |
| F65b | C73 | F64b | 111.7(12) |
| F65b | C73 | F65a | 126.7(6) |
| C71 | C73 | F63a | 112.7(2) |
| C71 | C73 | F63b | 110.6(6) |
| C71 | C73 | F64a | 112.1(2) |
| C71 | C73 | F64b | 111.4(7) |
| C71 | C73 | F65a | 113.4(2) |
| C71 | C73 | F65b | 119.7(6) |
| F67a | C74a | F66a | 103.6(7) |
| F68a | C74a | F66a | 107.5(9) |
| F68a | C74a | F67a | 106.2(8) |
| C69 | C74a | F66a | 111.6(6) |
| C69 | C74a | F67a | 115.1(6) |
| C69 | C74a | F68a | 112.2(9) |
| F67b | C74b | F66b | 104.6(10) |
| F68b | C74b | F66b | 106.3(12) |
| F68b | C74b | F67b | 105.6(11) |
| C69 | C74b | F66b | 116.1(8) |
| C69 | C74b | F67b | 110.0(9) |
| C69 | C74b | F68b | 113.3(13) |
| C80 | C75 | C76 | 115.4(2) |
| B51 | C75 | C76 | 120.12(19) |
| B51 | C75 | C80 | 124.3(2) |
| C77 | C76 | C75 | 122.4(2) |
| C78 | C77 | C76 | 120.8(2) |
| C81 | C77 | C76 | 118.8(2) |
| C81 | C77 | C78 | 120.4(2) |
| C79 | C78 | C77 | 117.6(2) |
| C80 | C79 | C78 | 121.5(2) |
| C82a | C79 | C78 | 115.5(3) |
| C82a | C79 | C80 | 123.0(3) |
| C82b | C79 | C78 | 127.7(7) |
| C82b | C79 | C80 | 109.8(7) |
| C82b | C79 | C82a | 16.4(7) |
| C79 | C80 | C75 | 122.2(2) |
| F70 | C81 | F69 | 106.7(2) |
| F71 | C81 | F69 | 105.6(2) |
| F71 | C81 | F70 | 106.8(2) |
| C77 | C81 | F69 | 112.6(2) |
| C77 | C81 | F70 | 112.3(2) |
| C77 | C81 | F71 | 112.3(2) |
| F73a | C82a | F72a | 106.3(4) |
| F74a | C82a | F72a | 103.3(5) |
| F74a | C82a | F73a | 107.4(6) |
| C79 | C82a | F72a | 113.9(4) |
| C79 | C82a | F73a | 111.9(4) |
| C79 | C82a | F74a | 113.4(5) |
| F73b | C82b | F72b | 103.7(13) |
| F74b | C82b | F72b | 115.0(17) |
| F74b | C82b | F73b | 101.7(15) |
| C79 | C82b | F72b | 106.2(11) |
| C79 | C82b | F73b | 116.6(13) |
| C79 | C82b | F74b | 113.6(15) |

| | | | |
|---|---|---|---|
| C59 | B51 | C51 | 108.68(18) |
| C67 | B51 | C51 | 111.53(17) |
| C67 | B51 | C59 | 110.72(17) |
| C75 | B51 | C51 | 108.76(17) |
| C75 | B51 | C59 | 108.17(18) |
| C75 | B51 | C67 | 108.90(18) |

9.8.11 Torsion angles

Table S53. Torsion Angles in ° for **8a**.

| Atom | Atom | Atom | Atom | Angle/° |
|---|---|---|---|---|
| Sb1 | C1 | N1 | C2 | 179.13(18) |
| Sb1 | C1 | N1 | B1 | -0.4(2) |
| Sb1 | C1 | N2 | C3 | -179.2(2) |
| Sb1 | C1 | N2 | C4 | 8.2(2) |
| Sb1 | C8 | N3 | C9 | -179.8(2) |
| Sb1 | C8 | N3 | B1 | -5.0(3) |
| Sb1 | C8 | N4 | C10 | 179.9(2) |
| Sb1 | C8 | N4 | C11a | -8.4(5) |
| Sb1 | C8 | N4 | C11b | 2.9(11) |
| Sb1 | C29 | C30 | F1 | 12.88(19) |
| Sb1 | C29 | C30 | C31 | -167.50(19) |
| Sb1 | C29 | C32 | F3 | -11.6(2) |
| Sb1 | C29 | C32 | C33 | 168.4(2) |
| F1 | C30 | C29 | C32 | -179.0(2) |
| F1 | C30 | C31 | F2 | -1.5(3) |
| F1 | C30 | C31 | N5 | 178.2(2) |
| F2 | C31 | N5 | C33 | -179.7(2) |
| F2 | C31 | C30 | C29 | 178.9(2) |
| F3 | C32 | C29 | C30 | -179.3(2) |
| F3 | C32 | C33 | F4 | -0.5(3) |
| F3 | C32 | C33 | N5 | 178.4(2) |
| F4 | C33 | N5 | C31 | 179.8(2) |
| F4 | C33 | C32 | C29 | 179.5(2) |
| N1 | C1 | N2 | C3 | 1.9(2) |
| N1 | C1 | N2 | C4 | -170.63(17) |
| N1 | C2 | C3 | N2 | -0.1(2) |
| N1 | B1 | N3 | C8 | 40.7(2) |
| N1 | B1 | N3 | C9 | -145.1(2) |
| N1 | B1 | C15 | C16 | -126.91(19) |
| N1 | B1 | C15 | C20 | 54.5(2) |
| N1 | B1 | C22 | C23 | 23.5(2) |
| N1 | B1 | C22 | C27 | -160.1(2) |
| N3 | C8 | N4 | C10 | -0.5(2) |
| N3 | C8 | N4 | C11a | 171.2(4) |
| N3 | C8 | N4 | C11b | -177.6(11) |
| N3 | C9 | C10 | N4 | 0.2(3) |
| N3 | B1 | C15 | C16 | 119.1(2) |
| N3 | B1 | C15 | C20 | -59.5(2) |
| N3 | B1 | C22 | C23 | 140.48(19) |
| N3 | B1 | C22 | C27 | -43.1(2) |
| N5 | C31 | C30 | C29 | -1.4(3) |
| N5 | C33 | C32 | C29 | -1.7(3) |
| C15 | C16 | C17 | C18 | 0.4(3) |
| C15 | C20 | C19 | C18 | 0.3(3) |
| C15 | B1 | C22 | C23 | -99.0(2) |
| C15 | B1 | C22 | C27 | 77.4(2) |
| C16 | C17 | C18 | C19 | -0.0(3) |
| C16 | C17 | C18 | C21 | 179.5(2) |
| C17 | C18 | C19 | C20 | -0.3(3) |
| C22 | C23 | C24 | C25 | -0.8(3) |
| C22 | C27 | C26 | C25 | -0.1(3) |

| | | | | |
|---|---|---|---|---|
| C23 | C24 | C25 | C26 | -2.8(3) |
| C23 | C24 | C25 | C28 | 174.4(3) |
| C24 | C25 | C26 | C27 | 3.2(3) |
| F51 | C58 | C53 | C52 | -70.9(2) |
| F51 | C58 | C53 | C54 | 107.6(2) |
| F52 | C58 | C53 | C52 | 49.0(3) |
| F52 | C58 | C53 | C54 | -132.5(2) |
| F53 | C58 | C53 | C52 | 169.6(2) |
| F53 | C58 | C53 | C54 | -12.0(3) |
| F54 | C57 | C55 | C54 | 162.6(2) |
| F54 | C57 | C55 | C56 | -19.0(3) |
| F55 | C57 | C55 | C54 | -77.2(2) |
| F55 | C57 | C55 | C56 | 101.2(2) |
| F56 | C57 | C55 | C54 | 42.3(3) |
| F56 | C57 | C55 | C56 | -139.28(19) |
| F57 | C66 | C61 | C60 | 148.9(3) |
| F57 | C66 | C61 | C62 | -36.2(3) |
| F58 | C66 | C61 | C60 | 27.1(3) |
| F58 | C66 | C61 | C62 | -158.0(2) |
| F59 | C66 | C61 | C60 | -89.4(3) |
| F59 | C66 | C61 | C62 | 85.5(3) |
| F60 | C65 | C63 | C62 | 152.4(3) |
| F60 | C65 | C63 | C64 | -31.1(4) |
| F61 | C65 | C63 | C62 | 33.6(4) |
| F61 | C65 | C63 | C64 | -149.9(3) |
| F62 | C65 | C63 | C62 | -85.8(3) |
| F62 | C65 | C63 | C64 | 90.8(3) |
| F63a | C73 | C71 | C70 | -139.4(2) |
| F63a | C73 | C71 | C72 | 42.0(3) |
| F63b | C73 | C71 | C70 | 31.9(9) |
| F63b | C73 | C71 | C72 | -146.8(9) |
| F64a | C73 | C71 | C70 | 102.2(3) |
| F64a | C73 | C71 | C72 | -76.5(3) |
| F64b | C73 | C71 | C70 | -71.2(10) |
| F64b | C73 | C71 | C72 | 110.1(10) |
| F65a | C73 | C71 | C70 | -19.7(3) |
| F65a | C73 | C71 | C72 | 161.6(3) |
| F65b | C73 | C71 | C70 | 155.9(12) |
| F65b | C73 | C71 | C72 | -22.7(12) |
| F66a | C74a | C69 | C68 | -168.7(5) |
| F66a | C74a | C69 | C70 | 17.1(8) |
| F66a | C74a | C69 | C74b | -49.7(18) |
| F66b | C74b | C69 | C68 | -132.2(8) |
| F66b | C74b | C69 | C70 | 41.6(11) |
| F66b | C74b | C69 | C74a | 161(2) |
| F67a | C74a | C69 | C68 | 73.7(8) |
| F67a | C74a | C69 | C70 | -100.6(6) |
| F67a | C74a | C69 | C74b | -167.3(16) |
| F67b | C74b | C69 | C68 | 109.3(9) |
| F67b | C74b | C69 | C70 | -77.0(9) |
| F67b | C74b | C69 | C74a | 42.1(14) |
| F68a | C74a | C69 | C68 | -48.0(9) |
| F68a | C74a | C69 | C70 | 137.8(7) |
| F68a | C74a | C69 | C74b | 71.0(18) |
| F68b | C74b | C69 | C68 | -8.7(13) |
| F68b | C74b | C69 | C70 | 165.1(9) |

| | | | | |
|---|---|---|---|---|
| F68b | C74b | C69 | C74a | -75.9(17) |
| F69 | C81 | C77 | C76 | 175.9(2) |
| F69 | C81 | C77 | C78 | -4.0(3) |
| F70 | C81 | C77 | C76 | 55.4(3) |
| F70 | C81 | C77 | C78 | -124.5(2) |
| F71 | C81 | C77 | C76 | -65.0(3) |
| F71 | C81 | C77 | C78 | 115.1(2) |
| F72a | C82a | C79 | C78 | 39.6(5) |
| F72a | C82a | C79 | C80 | -140.2(4) |
| F72a | C82a | C79 | C82b | -179(2) |
| F72b | C82b | C79 | C78 | 21.2(17) |
| F72b | C82b | C79 | C80 | -170.3(10) |
| F72b | C82b | C79 | C82a | -24.2(14) |
| F73a | C82a | C79 | C78 | -81.0(4) |
| F73a | C82a | C79 | C80 | 99.2(4) |
| F73a | C82a | C79 | C82b | 60(2) |
| F73b | C82b | C79 | C78 | -93.7(12) |
| F73b | C82b | C79 | C80 | 74.8(13) |
| F73b | C82b | C79 | C82a | -139(3) |
| F74a | C82a | C79 | C78 | 157.3(5) |
| F74a | C82a | C79 | C80 | -22.5(7) |
| F74a | C82a | C79 | C82b | -61(2) |
| F74b | C82b | C79 | C78 | 148.5(14) |
| F74b | C82b | C79 | C80 | -43.0(18) |
| F74b | C82b | C79 | C82a | 103(3) |
| C51 | C52 | C53 | C54 | 0.2(3) |
| C51 | C52 | C53 | C58 | 178.7(2) |
| C51 | C56 | C55 | C54 | -0.2(3) |
| C51 | C56 | C55 | C57 | -178.6(2) |
| C51 | B51 | C59 | C60 | -43.8(2) |
| C51 | B51 | C59 | C64 | 142.28(19) |
| C51 | B51 | C67 | C68 | -17.7(2) |
| C51 | B51 | C67 | C72 | 164.61(18) |
| C51 | B51 | C75 | C76 | -73.67(19) |
| C51 | B51 | C75 | C80 | 102.07(19) |
| C52 | C53 | C54 | C55 | -0.3(3) |
| C53 | C54 | C55 | C56 | 0.3(3) |
| C53 | C54 | C55 | C57 | 178.7(2) |
| C59 | C60 | C61 | C62 | -2.8(3) |
| C59 | C60 | C61 | C66 | 172.0(2) |
| C59 | C64 | C63 | C62 | 0.1(3) |
| C59 | C64 | C63 | C65 | -176.4(3) |
| C59 | B51 | C67 | C68 | 103.4(2) |
| C59 | B51 | C67 | C72 | -74.2(2) |
| C59 | B51 | C75 | C76 | 168.46(17) |
| C59 | B51 | C75 | C80 | -15.8(2) |
| C60 | C61 | C62 | C63 | 1.1(3) |
| C61 | C62 | C63 | C64 | 0.2(3) |
| C61 | C62 | C63 | C65 | 176.7(3) |
| C67 | C68 | C69 | C70 | -0.1(3) |
| C67 | C68 | C69 | C74a | -174.3(4) |
| C67 | C68 | C69 | C74b | 173.3(6) |
| C67 | C72 | C71 | C70 | 0.0(3) |
| C67 | C72 | C71 | C73 | 178.7(2) |
| C67 | B51 | C75 | C76 | 48.1(2) |
| C67 | B51 | C75 | C80 | -136.19(18) |

| | | | | |
|---|---|---|---|---|
| C68 | C69 | C70 | C71 | -0.1(3) |
| C69 | C70 | C71 | C72 | 0.1(3) |
| C69 | C70 | C71 | C73 | -178.5(2) |
| C75 | C76 | C77 | C78 | 1.8(3) |
| C75 | C76 | C77 | C81 | -178.1(2) |
| C75 | C80 | C79 | C78 | 0.1(3) |
| C75 | C80 | C79 | C82a | 179.9(3) |
| C75 | C80 | C79 | C82b | -169.3(8) |
| C76 | C77 | C78 | C79 | -0.2(3) |
| C77 | C78 | C79 | C80 | -0.8(3) |
| C77 | C78 | C79 | C82a | 179.4(3) |
| C77 | C78 | C79 | C82b | 166.6(9) |

9.8.12 Hydrogen Fractional Atomic Coordinates and Equivalent Isotropic Displacement Parameters

Table S54. Hydrogen Fractional Atomic Coordinates (×10$^4$) and Equivalent Isotropic Displacement Parameters (Å$^2$×10$^3$) for **8a**. $U_{eq}$ is defined as 1/3 of the trace of the orthogonalised $U_{ij}$.

| Atom | x | y | z | $U_{eq}$ |
|---|---|---|---|---|
| H2 | -1313(2) | 4889.1(9) | 547.5(11) | 37.8(6) |
| H3 | -2097(2) | 4212.8(9) | 1039.6(11) | 40.0(6) |
| H5a | -2096(17) | 4168.9(10) | 3102(6) | 58.8(9) |
| H5b | -1155(6) | 4577(5) | 3020(7) | 58.8(9) |
| H5c | -2487(12) | 4563(5) | 2599.1(16) | 58.8(9) |
| H6a | -2509(15) | 3551(5) | 2328(2) | 69.7(10) |
| H6b | -2975(9) | 3931.4(11) | 1816(9) | 69.7(10) |
| H6c | -1921(7) | 3573(5) | 1708(7) | 69.7(10) |
| H7a | 452(8) | 4022.2(15) | 2857(8) | 57.7(8) |
| H7b | -397(4) | 3577(5) | 2885(7) | 57.7(8) |
| H7c | 236(11) | 3670(6) | 2289.1(13) | 57.7(8) |
| H9 | 347(3) | 6445.4(9) | 1485.4(12) | 46.3(7) |
| H10 | 733(3) | 6740.5(9) | 2547.4(12) | 45.3(7) |
| H12a | 2250(17) | 5835(9) | 4259(2) | 63.0(17) |
| H12b | 2122(14) | 5494(3) | 3679(12) | 63.0(17) |
| H12c | 2923(5) | 5955(7) | 3684(12) | 63.0(17) |
| H12d | 3030(20) | 6397(10) | 3510(40) | 84(4) |
| H12e | 1970(40) | 6777(12) | 3350(30) | 84(4) |
| H12f | 2380(60) | 6610(20) | 4046(10) | 84(4) |
| H13a | 1180(40) | 6587.0(16) | 4166(3) | 96(3) |
| H13b | 1840(20) | 6722(4) | 3591(17) | 96(3) |
| H13c | 365(18) | 6737(4) | 3527(15) | 96(3) |
| H13d | 1111(17) | 5479(10) | 3860(30) | 56(4) |
| H13e | 2420(40) | 5575(14) | 3643(19) | 56(4) |
| H13f | 2120(60) | 5790(6) | 4274(11) | 56(4) |
| H14a | -204(16) | 5532(4) | 3532(14) | 72.3(17) |
| H14b | -133(14) | 5863(10) | 4119(2) | 72.3(17) |
| H14c | -876(4) | 6023(7) | 3466(12) | 72.3(17) |
| H14d | -150(40) | 6585(14) | 3560(30) | 80(4) |
| H14e | -570(20) | 6061(15) | 3620(30) | 80(4) |
| H14f | 260(20) | 6310(30) | 4193(5) | 80(4) |
| H16 | 802(2) | 5839.1(8) | 125.5(11) | 37.5(6) |
| H17 | -486(2) | 6244.6(9) | -627.0(11) | 40.8(6) |
| H19 | -3207(2) | 6189.0(9) | 409.7(11) | 41.7(6) |
| H20 | -1923(2) | 5779.7(9) | 1149.9(11) | 41.6(6) |
| H21a | -3679(4) | 6420(5) | -695(7) | 59.8(9) |
| H21b | -2787(15) | 6857.8(13) | -571(5) | 59.8(9) |
| H21c | -2570(12) | 6485(6) | -1080(2) | 59.8(9) |
| H23 | 1522(2) | 4703.3(8) | 831.0(11) | 37.0(6) |
| H24 | 3497(2) | 4545.7(10) | 632.9(12) | 43.1(6) |
| H26 | 4508(3) | 5854.3(11) | 1079.2(15) | 57.3(8) |
| H27 | 2542(2) | 6006.5(9) | 1287.6(14) | 49.2(7) |
| H28a | 6041(6) | 5018(8) | 1189.1(17) | 80.7(12) |
| H28b | 5547(4) | 4784(5) | 537(9) | 80.7(12) |
| H28c | 5887(8) | 5321(4) | 572(10) | 80.7(12) |
| H52 | 8338(2) | 6354.1(8) | 5066.9(10) | 31.6(5) |
| H54 | 7676(2) | 4983.4(8) | 4911.5(11) | 36.8(5) |

| | | | | |
|---|---|---|---|---|
| H56 | 5231(2) | 5897.1(8) | 4007.5(10) | 31.7(5) |
| H60 | 7064(2) | 6352.2(8) | 3330.5(10) | 33.8(5) |
| H62 | 4886(2) | 6895.9(9) | 1846.8(11) | 41.3(6) |
| H64 | 4249(2) | 7192.0(9) | 3572.8(11) | 39.6(6) |
| H68 | 4674(2) | 6314.9(8) | 5036.0(10) | 33.2(5) |
| H70 | 2721(2) | 7384.6(9) | 5581.0(11) | 38.8(6) |
| H72 | 5395(2) | 7614.1(8) | 4543.7(11) | 34.2(5) |
| H76 | 7559(2) | 7108.1(8) | 5396.5(10) | 32.5(5) |
| H78 | 10439(2) | 7791.1(9) | 4867.1(13) | 44.3(6) |
| H80 | 7822(2) | 7205.8(8) | 3593.4(11) | 37.7(6) |

9.8.13 Atomic Occupancies for all atoms that are not fully occupied

Table S55. Atomic Occupancies for all atoms that are not fully occupied in **8a**.

| Atom | Occupancy |
|---|---|
| C11a | 0.700(6) |
| C11b | 0.300(6) |
| C12a | 0.700(6) |
| H12a | 0.700(6) |
| H12b | 0.700(6) |
| H12c | 0.700(6) |
| C12b | 0.300(6) |
| H12d | 0.300(6) |
| H12e | 0.300(6) |
| H12f | 0.300(6) |
| C13a | 0.700(6) |
| H13a | 0.700(6) |
| H13b | 0.700(6) |
| H13c | 0.700(6) |
| C13b | 0.300(6) |
| H13d | 0.300(6) |
| H13e | 0.300(6) |
| H13f | 0.300(6) |
| C14a | 0.700(6) |
| H14a | 0.700(6) |
| H14b | 0.700(6) |
| H14c | 0.700(6) |
| C14b | 0.300(6) |
| H14d | 0.300(6) |
| H14e | 0.300(6) |
| H14f | 0.300(6) |
| F63a | 0.866(3) |
| F63b | 0.134(3) |
| F64a | 0.866(3) |
| F64b | 0.134(3) |
| F65a | 0.866(3) |
| F65b | 0.134(3) |
| F66a | 0.582(8) |
| F66b | 0.418(8) |
| F67a | 0.582(8) |
| F67b | 0.418(8) |
| F68a | 0.582(8) |
| F68b | 0.418(8) |
| F72a | 0.727(8) |
| F72b | 0.273(8) |
| F73a | 0.727(8) |
| F73b | 0.273(8) |
| F74a | 0.727(8) |
| F74b | 0.273(8) |
| C74a | 0.582(8) |
| C74b | 0.418(8) |
| C82a | 0.727(8) |
| C82b | 0.273(8) |

### 9.8.14 Solvent masking (PLATON/SQUEEZE) information

Table S56. Solvent masking (PLATON/SQUEEZE) information for **8a**.

| No | x | y | z | V | e | Content |
|---|---|---|---|---|---|---|
| 1 | 0.339 | 0.179 | 0.812 | 147.4 | 42.8 | 1DCM |
| 2 | 0.339 | 0.321 | 0.312 | 147.4 | 43.2 | 1DCM |
| 3 | 0.661 | 0.679 | 0.688 | 147.4 | 43.2 | 1DCM |
| 4 | 0.661 | 0.821 | 0.188 | 147.4 | 43.6 | 1DCM |

## 9.9 Single crystal structure analysis of 9a

CCDC Deposition number 2423157

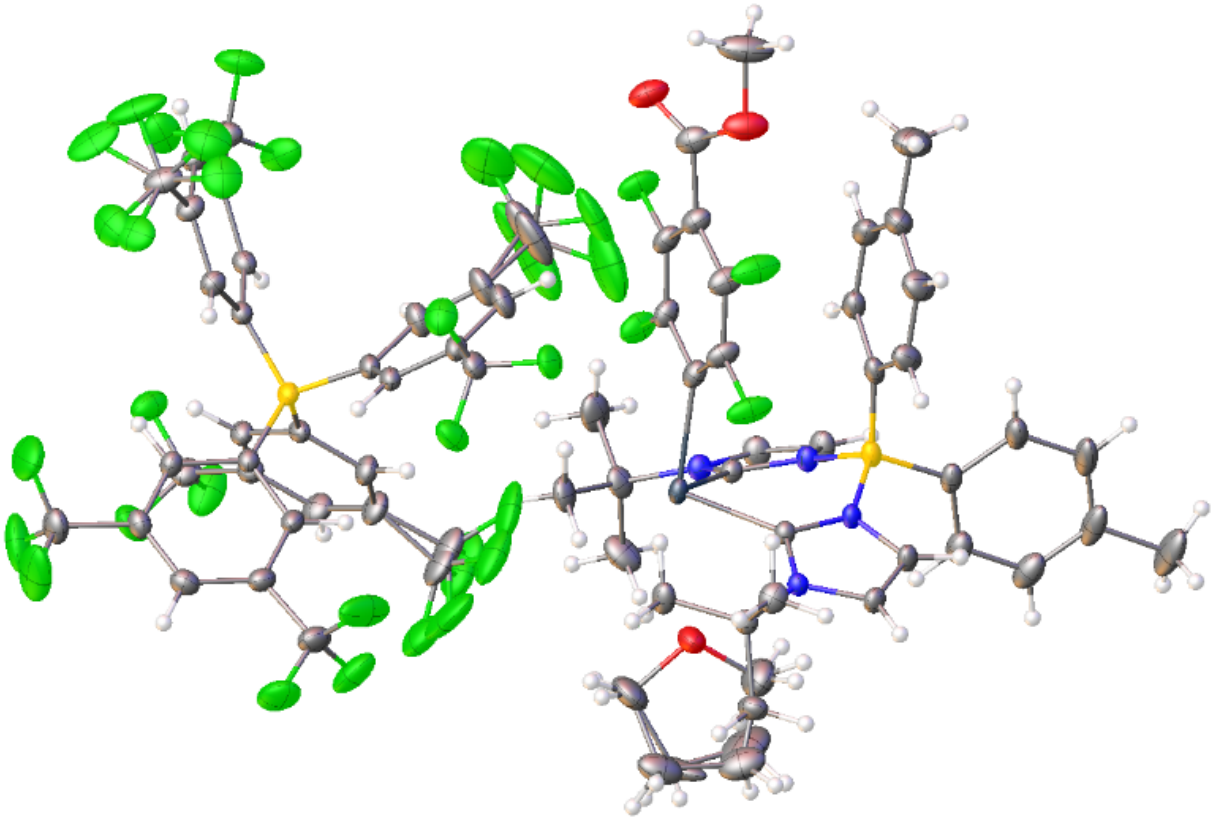

Figure S34. Olex-2 representation of the molecular structure of complex **9a**

### 9.9.1 Experimental

Single colourless plate-shaped crystals of **9a** were used as supplied. A suitable crystal $0.10\times0.05\times0.02$ mm$^3$ was selected and mounted on a MITIGEN holder in oil on a Rigaku FRE+ diffractometer with Arc)Sec VHF Varimax confocal mirrors, a UG2 goniometer and HyPix 6000HE detector. The crystal was kept at a steady $T$ = 100(2) K during data collection. The structure was solved with the ShelXT 2018/2 (Sheldrick, 2015) structure solution program using dual methods solution method and by using Olex2 1.5-alpha (Dolomanov et al., 2009) as the graphical interface. The model was refined with olex2.refine 1.5-alpha (Bourhis et al., 2015) using full matrix least squares minimisation on $\boldsymbol{F}^2$ minimisation.

### 9.9.2 Crystal Data

$C_{72}H_{59}B_2F_{28}N_4O_3Sb$, $M_r$ = 1703.659, monoclinic, *I*2/*a* (No. 15), a = 35.9316(6) Å, b = 12.3239(2) Å, c = 34.6274(7) Å, $\beta$ = 103.410(2)°, $\alpha = \gamma$ = 90°, *V* = 14915.6(5) Å$^3$, *T* = 100(2) K, *Z* = 8, *Z'* = 1, $\mu$(Mo $K_\alpha$) = 0.489 mm$^{-1}$, 198747 reflections measured, 25975 unique ($R_{int}$ = 0.0715) which were used in all calculations. The final $wR_2$ was 0.1024 (all data) and $R_1$ was 0.0425 (I≥2 $\sigma$(I)).

| Compound | **9a** |
|---|---|
| | |
| Formula | $C_{72}H_{59}B_2F_{28}N_4O_3Sb$ |
| $D_{calc.}$/ g cm$^{-3}$ | 1.517 |
| $\mu$/mm$^{-1}$ | 0.489 |
| Formula Weight | 1703.659 |
| Colour | colourless |
| Shape | plate-shaped |
| Size/mm$^3$ | 0.10×0.05×0.02 |
| *T*/K | 100(2) |
| Crystal System | monoclinic |
| Space Group | *I*2/*a* |
| *a*/Å | 35.9316(6) |
| *b*/Å | 12.3239(2) |
| *c*/Å | 34.6274(7) |
| $\alpha$/° | 90 |
| $\beta$/° | 103.410(2) |
| $\gamma$/° | 90 |
| V/Å$^3$ | 14915.6(5) |
| *Z* | 8 |
| *Z'* | 1 |
| Wavelength/Å | 0.71073 |
| Radiation type | Mo $K_\alpha$ |
| $\Theta_{min}$/° | 1.86 |
| $\Theta_{max}$/° | 32.03 |
| Measured Refl's. | 198747 |
| Indep't Refl's | 25975 |
| Refl's I≥2 $\sigma$(I) | 19192 |
| $R_{\rm int}$ | 0.0715 |
| Parameters | 1121 |
| Restraints | 794 |
| Largest Peak | 0.9833 |
| Deepest Hole | -0.9217 |
| GooF | 1.0369 |
| $wR_2$ (all data) | 0.1024 |
| $wR_2$ | 0.0907 |
| $R_1$ (all data) | 0.0700 |
| $R_1$ | 0.0425 |

### 9.9.3 Structure Quality Indicators

| | | | | |
|---|---|---|---|---|
| Reflections: | d min (MoKα) 2Θ=64.1° 0.67 | I/σ(I) 21.5 | Rint m=7.82 7.15% | Full 50.5° 100 |
| Refinement: | Shift 0.001 | Max Peak 1.0 | Min Peak -0.9 | GooF 1.037 |

A colourless plate-shaped crystal with dimensions 0.10×0.05×0.02 $mm^3$ was mounted on a MITIGEN holder in oil. X-ray diffraction data were collected using a Rigaku FRE+ diffractometer with Arc)Sec VHF Varimax confocal mirrors, a UG2 goniometer and HyPix 6000HE detector equipped with an Oxford Cryosystems low-temperature device, operating at $T$ = 100(2) K.

Data were measured using profile data from $\omega$-scans of 0.4 ° per frame for 15.0 s using Mo $K_{\alpha}$ radiation (Rotating Anode, 45.0 kV, 55.0 mA). The total number of runs and images was based on the strategy calculation from the program CrysAlisPro system (CCD 43.131a 64-bit (release 19-07-2024)). The maximum resolution achieved was $\Theta$ = 32.03°.

Cell parameters were retrieved using the CrysAlisPro 1.171.43.130a (Rigaku OD, 2024) software and refined using CrysAlisPro 1.171.43.130a (Rigaku OD, 2024) on 54658 reflections, 28 % of the observed reflections. Data reduction was performed using the CrysAlisPro 1.171.43.130a (Rigaku OD, 2024) software which corrects for Lorentz polarisation. The final completeness is 99.96 % out to 32.03° in $\Theta$.

A multi-scan absorption correction was performed using CrysAlisPro 1.171.43.130a (Rigaku OD, 2024) Empirical absorption correction using spherical harmonics, implemented in SCALE3 ABSPACK scaling algorithm.. The absorption coefficient $\mu$ of this material is 0.489 $mm^{-1}$ at this wavelength ($\lambda$ = 0.71073Å) and the minimum and maximum transmissions are 0.849 and 1.000.

The structure was solved in the space group *I*2/*a* (# 15) by using dual methods using the ShelXT 2018/2 (Sheldrick, 2015) structure solution program and refined by full matrix least squares minimisation on $F^2$ using olex2.refine 1.5-alpha (Bourhis et al., 2015). All non-hydrogen atoms were refined anisotropically. Hydrogen atom positions were calculated geometrically and refined using the riding model.

*_refine_special_details*: Three CF3 groups and solvent THF are disordered (ca. 52:48, 80:20, 81:19, 72:28). 1,2 and 1,3 equal distance restraints (SADI) were applied to all chemically equivalent atom pairs of each disorder component. Thermal restraints (RIGU, SIMU) were applied to disordered THF. Thermal restraints (RIGU) were applied to BArF anion.

*_exptl_absorpt_process_details*: CrysAlisPro 1.171.43.130a (Rigaku OD, 2024) using spherical harmonics, implemented in SCALE3 ABSPACK scaling algorithm.

There is a single formula unit in the asymmetric unit, which is represented by the reported sum formula. In other words: Z is 8 and Z' is 1. The moiety formula is C36 H39 B F4 N4 O2 Sb, C32 H12 B F24, C4 H8 O.

### 9.9.4 Data Plots: Diffraction Data

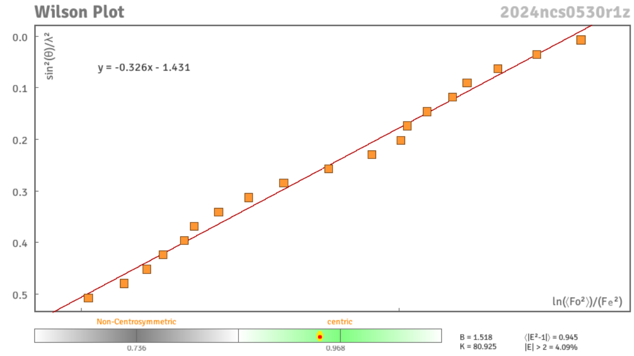


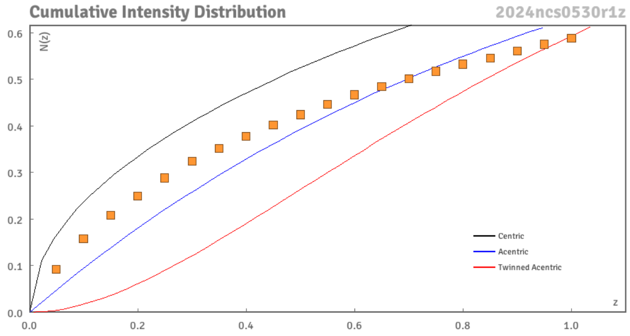


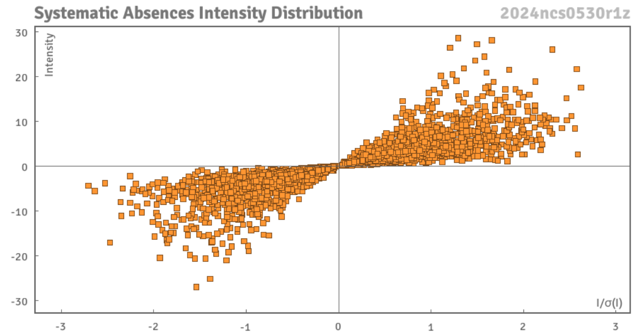


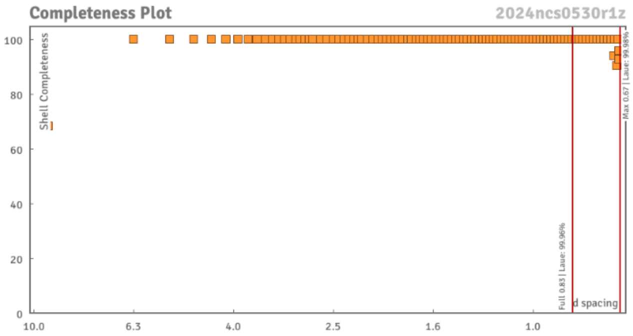


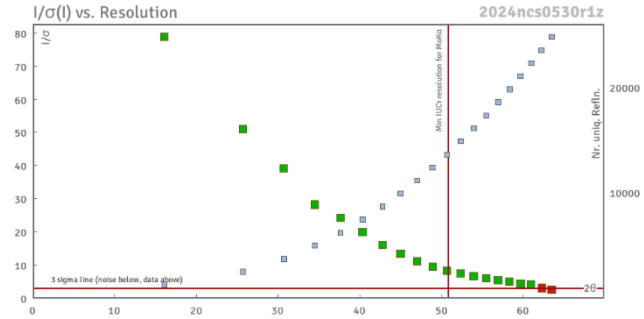


### 9.9.5 Data Plots: Refinement and Data

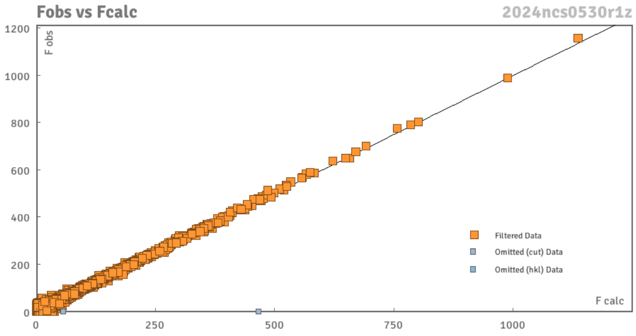


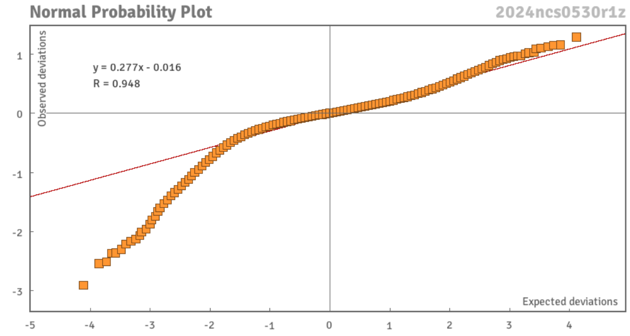

### 9.9.6 Reflection Statistics

| | | | |
|---|---|---|---|
| Total reflections (after filtering) | 203141 | Unique reflections | 25975 |
| Completeness | 1.0 | Mean $I/\sigma$ | 17.14 |
| $hkl_{max}$ collected | (53, 18, 51) | $hkl_{min}$ collected | (-53, -17, -51) |
| $hkl_{max}$ used | (52, 18, 51) | $hkl_{min}$ used | (-53, 0, 0) |
| Lim $d_{max}$ collected | 100.0 | Lim $d_{min}$ collected | 0.36 |
| $d_{max}$ used | 11.49 | $d_{min}$ used | 0.67 |
| Friedel pairs | 38125 | Friedel pairs merged | 1 |
| Inconsistent equivalents | 11 | $R_{int}$ | 0.0715 |
| $R_{sigma}$ | 0.0466 | Intensity transformed | 0 |
| Omitted reflections | 5 | Omitted by user (OMIT hkl) | 0 |
| Multiplicity | (30828, 26692, 17996, 8199, 2615, 1308, 738, 360, 212, 104, 19, 2) | Maximum multiplicity | 25 |
| Removed systematic absences | 4399 | Filtered off (Shel/OMIT) | 0 |

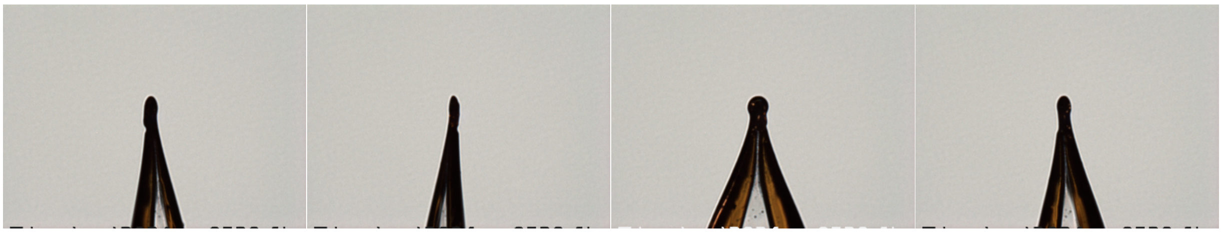

### 9.9.7 Fractional Atomic Coordinates (×10^4) and Equivalent Isotropic Displacement Parameters

Table S57. Fractional Atomic Coordinates (×10^4) and Equivalent Isotropic Displacement Parameters (Å^2×10^3) for **9a**. $U_{eq}$ is defined as 1/3 of the trace of the orthogonalised $U_{ij}$.

| Atom | x | y | z | $U_{eq}$ |
|---|---|---|---|---|
| Sb1 | 4029.06(3) | 1500.91(9) | 3587.68(4) | 18.10(3) |
| F1 | 4429.7(4) | 1634.1(9) | 2817.7(5) | 39.8(3) |
| F2 | 4560.6(5) | 3227.9(10) | 2364.9(5) | 46.0(4) |
| F3 | 3929.7(4) | 5661.2(9) | 3061.6(4) | 38.9(3) |
| F4 | 3779.1(4) | 4028.6(9) | 3494.2(4) | 34.5(3) |
| O1 | 4360.5(5) | 6404.2(12) | 2585.2(6) | 40.2(4) |
| O2 | 4403.6(5) | 5196.2(13) | 2116.4(5) | 41.8(4) |
| N1 | 3169.1(4) | 1491.1(12) | 3160.4(4) | 18.9(3) |
| N2 | 3222.9(4) | 2205.0(12) | 3751.6(5) | 20.6(3) |
| N3 | 3581.6(4) | 123.6(11) | 2888.4(4) | 18.0(3) |
| N4 | 4131.1(4) | -687.4(11) | 3126.9(4) | 16.8(3) |
| C1 | 3421.1(5) | 1760.8(14) | 3499.0(5) | 18.2(3) |
| C2 | 2808.8(5) | 1787.0(16) | 3197.9(6) | 25.7(4) |
| C3 | 2840.2(5) | 2223.1(16) | 3559.0(6) | 25.7(4) |
| C4 | 3346.2(6) | 2574.3(17) | 4176.0(6) | 27.3(4) |
| C5 | 3163.6(7) | 1803(2) | 4423.1(7) | 40.6(5) |
| C6 | 3204.1(8) | 3739(2) | 4193.5(7) | 41.5(6) |
| C7 | 3777.1(6) | 2558.8(18) | 4330.2(6) | 30.8(4) |
| C8 | 3919.6(5) | 211.7(13) | 3154.8(5) | 17.1(3) |
| C9 | 3584.0(5) | -839.0(14) | 2687.0(6) | 21.8(4) |
| C10 | 3921.2(5) | -1342.9(14) | 2833.4(5) | 20.8(3) |
| C11 | 4529.9(5) | -1027.1(14) | 3339.3(5) | 19.3(3) |
| C12 | 4502.0(6) | -2124.8(16) | 3535.6(6) | 27.8(4) |
| C13 | 4715.6(5) | -203.3(16) | 3653.5(6) | 26.6(4) |
| C14 | 4766.6(6) | -1116.1(17) | 3027.3(6) | 27.5(4) |
| C15 | 2860.8(5) | 319.3(15) | 2547.3(6) | 24.1(4) |
| C16 | 2702.4(6) | -480.0(16) | 2747.4(7) | 29.6(4) |
| C17 | 2388.9(6) | -1100.7(18) | 2557.4(8) | 37.3(5) |
| C18 | 2220.4(6) | -940.0(18) | 2155.8(8) | 42.3(6) |
| C19 | 2367.6(7) | -137.5(18) | 1959.6(8) | 41.0(6) |
| C20 | 2683.1(6) | 479.6(17) | 2147.9(6) | 31.0(5) |
| C21 | 1893.5(7) | -1649(2) | 1943.5(11) | 62.3(10) |
| C22 | 3353.1(5) | 1908.5(15) | 2482.5(5) | 21.6(3) |
| C23 | 3220.1(6) | 2978.2(15) | 2482.6(6) | 25.0(4) |
| C24 | 3278.4(6) | 3740.6(16) | 2205.9(7) | 30.9(4) |
| C25 | 3472.9(7) | 3472.4(18) | 1916.0(7) | 34.8(5) |
| C26 | 3609.2(7) | 2414.9(18) | 1914.2(7) | 36.1(5) |
| C27 | 3550.4(6) | 1658.6(16) | 2190.1(6) | 29.4(4) |
| C28 | 3530.0(9) | 4295(2) | 1612.2(8) | 50.8(7) |
| C29 | 4094.1(5) | 2770.3(14) | 3168.4(6) | 22.0(4) |
| C30 | 4295.8(6) | 2631.4(15) | 2878.3(7) | 27.1(4) |
| C31 | 4368.6(6) | 3468.3(16) | 2642.2(7) | 29.4(4) |
| C32 | 4254.3(5) | 4528.3(15) | 2696.5(6) | 25.0(4) |
| C33 | 4057.8(5) | 4677.8(14) | 2991.2(6) | 24.7(4) |
| C34 | 3979.2(5) | 3825.8(15) | 3217.6(6) | 23.5(4) |
| C35 | 4345.1(6) | 5485.2(16) | 2467.7(7) | 29.7(4) |
| C36 | 4496.0(9) | 6083(2) | 1879.5(10) | 56.2(8) |
| B1 | 3241.1(6) | 983.9(16) | 2763.7(6) | 19.8(4) |
| F51 | 7543.7(5) | 4565.1(15) | 5593.1(5) | 66.3(5) |
| F52 | 7705.1(5) | 2963.5(16) | 5779.5(6) | 77.4(6) |

| | | | | |
|---|---|---|---|---|
| F53 | 7267.2(5) | 3793.2(17) | 5985.3(5) | 65.4(5) |
| F54 | 6867.5(7) | 469.6(12) | 4424.7(5) | 76.7(6) |
| F55 | 6492.0(5) | 58.5(11) | 4798.4(6) | 58.6(5) |
| F56 | 7091.8(5) | -54.4(11) | 5016.2(5) | 54.3(4) |
| F57 | 5771.7(4) | 9040.1(11) | 4157.0(5) | 47.0(4) |
| F58 | 5404.9(4) | 7842.1(11) | 4323.7(5) | 45.4(4) |
| F59 | 5779.2(4) | 8789.7(12) | 4766.2(5) | 47.2(4) |
| F60a | 7115.9(6) | 6791(2) | 3982.9(6) | 46.7(6) |
| F60b | 7082(3) | 7382(11) | 3982(3) | 58(2) |
| F61a | 7367.1(9) | 6882(3) | 4600.5(11) | 49.6(8) |
| F61b | 7366(4) | 6611(12) | 4501(4) | 43(2) |
| F62a | 7119.5(7) | 8308.7(14) | 4277.4(12) | 62.5(9) |
| F62b | 7223(2) | 8187(7) | 4551(4) | 55.0(18) |
| F63 | 6734.0(4) | 2032.1(10) | 3542.5(4) | 37.3(3) |
| F64 | 6834.1(4) | 3509.4(11) | 3253.6(4) | 38.8(3) |
| F65 | 6399.5(4) | 2400.8(10) | 2957.3(3) | 34.5(3) |
| F66a | 5151.0(15) | 4036(8) | 2917.0(17) | 98(2) |
| F66b | 5218.7(17) | 4672(6) | 2839.2(13) | 75.1(19) |
| F67a | 5100.6(19) | 5247(6) | 3358.2(16) | 72.5(18) |
| F67b | 5019(2) | 4735(9) | 3355(2) | 108(3) |
| F68a | 5426.7(15) | 5545(6) | 2910.3(18) | 107(2) |
| F68b | 5379.3(16) | 6106(4) | 3213(2) | 107(3) |
| F69 | 5675.5(5) | 4280.3(12) | 5976.4(4) | 50.8(4) |
| F70 | 5482.0(4) | 5737.8(10) | 5666.3(4) | 38.9(3) |
| F71 | 5076.4(5) | 4525.6(14) | 5733.7(5) | 57.1(4) |
| F72a | 4937.5(9) | 1946(4) | 4187.9(9) | 69.5(11) |
| F72b | 5100(5) | 1393(13) | 4322(5) | 72(4) |
| F73a | 4659.5(9) | 1977(4) | 4647.4(14) | 50.0(8) |
| F73b | 4723(5) | 1711(16) | 4727(6) | 51(3) |
| F74a | 5169.9(9) | 955.6(19) | 4688.4(18) | 88.0(17) |
| F74b | 5095(4) | 987(8) | 4925(5) | 60(3) |
| C51 | 6531.5(5) | 3501.5(14) | 4814.1(5) | 18.6(3) |
| C52 | 6538.8(6) | 2389.6(14) | 4725.5(6) | 22.2(4) |
| C53 | 6825.4(6) | 1707.4(14) | 4924.4(6) | 24.6(4) |
| C54 | 7116.2(6) | 2082.2(16) | 5232.0(6) | 26.5(4) |
| C55 | 7111.1(5) | 3169.6(16) | 5331.1(6) | 24.1(4) |
| C56 | 6827.2(5) | 3861.6(15) | 5123.5(6) | 21.2(3) |
| C57 | 7407.2(6) | 3600.7(19) | 5673.2(7) | 33.8(5) |
| C58 | 6821.0(7) | 551.4(17) | 4790.7(7) | 35.2(5) |
| C59 | 6281.0(5) | 5557.2(14) | 4576.9(5) | 18.7(3) |
| C60 | 6633.0(5) | 5899.6(15) | 4509.8(6) | 22.1(4) |
| C61 | 6698.4(5) | 6965.5(15) | 4414.3(6) | 24.0(4) |
| C62 | 6414.6(5) | 7744.6(15) | 4379.5(6) | 23.2(4) |
| C63 | 6064.9(5) | 7434.5(14) | 4450.5(6) | 20.9(3) |
| C64 | 6001.6(5) | 6366.0(14) | 4551.0(5) | 20.0(3) |
| C65 | 5757.2(6) | 8268.4(16) | 4422.4(7) | 27.8(4) |
| C66 | 7075.3(6) | 7255.2(17) | 4331.0(8) | 33.2(5) |
| C67 | 6086.0(5) | 4015.1(14) | 4093.4(5) | 21.0(3) |
| C68 | 6341.5(5) | 3513.4(14) | 3903.2(5) | 20.9(3) |
| C69 | 6269.3(5) | 3414.5(15) | 3491.6(5) | 22.9(3) |
| C70 | 5942.1(6) | 3857.7(19) | 3252.2(6) | 32.7(4) |
| C71 | 5687.0(6) | 4384(2) | 3435.1(7) | 37.8(5) |
| C72 | 5754.8(6) | 4441.1(19) | 3844.3(6) | 31.6(4) |
| C73 | 6557.5(6) | 2844.4(16) | 3312.8(6) | 27.2(4) |
| C74a | 5352(3) | 4813(10) | 3170(3) | 62(2) |
| C74b | 5322(4) | 5055(12) | 3200(3) | 63(2) |

| | | | | |
|---|---|---|---|---|
| C75 | 5815.1(5) | 3976.2(14) | 4761.6(5) | 19.4(3) |
| C76 | 5758.1(5) | 4456.8(14) | 5109.2(5) | 20.6(3) |
| C77 | 5460.6(5) | 4143.8(15) | 5282.3(6) | 23.6(4) |
| C78 | 5212.1(6) | 3317.6(16) | 5117.7(7) | 29.9(4) |
| C79 | 5267.3(6) | 2817.4(17) | 4776.7(7) | 31.3(4) |
| C80 | 5559.9(5) | 3142.3(16) | 4602.3(6) | 26.0(4) |
| C81 | 5421.9(6) | 4667.1(17) | 5659.3(6) | 29.1(4) |
| C82a | 5006.9(15) | 1922(4) | 4586.6(17) | 44.4(11) |
| C82b | 5065(7) | 1744(19) | 4654(7) | 44(3) |
| B51 | 6180.4(6) | 4260.5(16) | 4573.0(6) | 18.8(4) |
| O91 | 3806.6(5) | -285.4(12) | 3992.8(5) | 38.3(4) |
| C91 | 3517.8(8) | -1077(2) | 3845.9(9) | 52.1(7) |
| C92a | 3642.0(15) | -2093(4) | 4105.6(16) | 48.9(12) |
| C92b | 3471(7) | -1796(15) | 4116(4) | 76(5) |
| C93a | 3975(2) | -1747(3) | 4427.4(13) | 47.4(13) |
| C93b | 3811(5) | -1716(13) | 4423(5) | 76(4) |
| C94 | 3982.2(9) | -577(2) | 4395.3(7) | 49.4(7) |

9.9.8 Anisotropic Displacement Parameters

Table S58. Anisotropic Displacement Parameters (×$10^4$) for **9a**. The anisotropic displacement factor exponent takes the form: $-2\pi^2[h^2a^{*2} \times U_{11}+ ... +2hka^* \times b^* \times U_{12}]$

| Atom | $U_{11}$ | $U_{22}$ | $U_{33}$ | $U_{23}$ | $U_{13}$ | $U_{12}$ |
|---|---|---|---|---|---|---|
| Sb1 | 15.56(5) | 19.07(5) | 18.45(6) | -0.25(4) | 1.44(4) | -5.46(5) |
| F1 | 51.6(8) | 19.6(5) | 61.2(9) | 6.6(5) | 39.5(7) | 1.7(5) |
| F2 | 65.7(10) | 26.5(6) | 62.8(10) | 6.3(6) | 49.6(8) | 3.2(6) |
| F3 | 45.8(8) | 19.1(5) | 59.2(9) | 4.9(5) | 27.2(7) | -3.6(6) |
| F4 | 45.4(7) | 24.6(6) | 41.2(8) | 0.1(5) | 25.6(6) | -8.2(5) |
| O1 | 45.1(9) | 22.1(7) | 56.4(11) | -0.3(6) | 17.6(8) | 3.4(7) |
| O2 | 51.0(10) | 32.0(8) | 51.4(11) | 10.1(7) | 30.5(9) | 11.1(7) |
| N1 | 15.7(6) | 20.7(7) | 18.9(7) | 2.9(6) | 1.3(5) | -2.7(6) |
| N2 | 20.4(7) | 22.6(7) | 19.3(8) | 2.1(6) | 5.6(6) | -2.7(6) |
| N3 | 18.2(7) | 18.4(6) | 15.6(7) | 1.1(5) | 0.5(5) | -3.7(5) |
| N4 | 16.1(6) | 17.6(6) | 15.9(7) | 0.8(5) | 2.3(5) | -0.9(5) |
| C1 | 19.0(8) | 18.2(7) | 17.3(8) | -0.1(6) | 4.1(6) | -2.0(6) |
| C2 | 16.8(8) | 29.8(9) | 28.9(10) | 4.0(7) | 2.0(7) | -4.8(8) |
| C3 | 19.8(8) | 30.4(9) | 27.9(10) | 4.3(7) | 7.8(7) | -4.1(8) |
| C4 | 30.9(10) | 34.5(10) | 17.7(9) | 6.0(8) | 7.7(8) | -5.5(8) |
| C5 | 38.8(12) | 60.4(15) | 26.2(11) | 7.7(11) | 14.9(10) | 8.5(10) |
| C6 | 48.1(14) | 41.8(12) | 32.8(12) | 12.0(11) | 5.7(10) | -17.0(10) |
| C7 | 31.5(10) | 40.3(11) | 19.1(10) | 1.5(9) | 3.0(8) | -10.4(8) |
| C8 | 16.5(7) | 17.7(7) | 16.4(8) | 0.0(6) | 2.7(6) | -1.6(6) |
| C9 | 22.5(8) | 21.4(8) | 19.1(9) | 1.8(7) | 0.3(7) | -6.0(7) |
| C10 | 23.3(8) | 18.7(8) | 19.9(8) | 0.4(6) | 3.8(7) | -5.0(6) |
| C11 | 15.3(7) | 20.7(8) | 21.0(9) | 2.3(6) | 2.5(6) | -0.5(7) |
| C12 | 27.5(10) | 24.8(9) | 29.5(11) | 2.9(8) | 3.7(8) | 6.4(8) |
| C13 | 18.2(8) | 29.1(9) | 29.0(10) | 4.4(7) | -1.5(7) | -6.9(8) |
| C14 | 22.6(9) | 30.4(9) | 31.7(11) | 0.1(8) | 10.7(8) | -3.9(8) |
| C15 | 19.9(8) | 22.7(8) | 26.0(10) | 4.4(7) | -2.2(7) | -8.0(7) |
| C16 | 22.1(9) | 28.3(9) | 35.3(12) | 1.1(8) | 0.2(8) | -6.1(8) |
| C17 | 23.6(10) | 28.6(10) | 57.6(16) | -1.2(8) | 5.1(10) | -9.5(10) |
| C18 | 21.6(10) | 30.8(11) | 63.6(17) | 5.9(8) | -12.1(10) | -19.5(11) |
| C19 | 32.7(11) | 34.5(11) | 43.4(14) | 9.4(9) | -16.8(10) | -13.9(10) |
| C20 | 28.9(10) | 28.0(9) | 29.2(11) | 5.8(8) | -7.2(8) | -7.5(8) |
| C21 | 29.1(12) | 41.4(14) | 98(3) | -0.3(10) | -22.1(14) | -23.9(15) |
| C22 | 21.4(8) | 23.4(8) | 17.0(8) | 3.2(7) | -1.8(7) | -3.0(7) |
| C23 | 24.5(9) | 24.3(9) | 23.1(9) | 2.1(7) | -0.8(7) | -3.5(7) |
| C24 | 33.4(11) | 22.8(9) | 31.4(11) | 2.3(8) | -3.2(9) | 0.9(8) |
| C25 | 42.7(12) | 31.8(10) | 26.2(10) | -3.6(9) | 0.4(9) | 4.7(9) |
| C26 | 47.5(13) | 36.4(11) | 26.1(11) | 3.8(10) | 12.0(10) | 0.7(9) |
| C27 | 38.6(11) | 26.7(10) | 23.6(10) | 6.7(8) | 8.4(8) | -0.5(8) |
| C28 | 73(2) | 40.0(13) | 39.5(15) | -1.5(13) | 14.2(14) | 11.5(11) |
| C29 | 17.1(8) | 20.7(8) | 27.9(10) | -2.2(6) | 4.3(7) | -4.9(7) |
| C30 | 26.4(9) | 17.8(8) | 41.0(12) | 2.1(7) | 15.9(9) | -2.0(8) |
| C31 | 32.0(10) | 24.0(9) | 38.4(12) | 0.4(8) | 20.3(9) | -2.5(8) |
| C32 | 21.8(8) | 20.1(8) | 34.1(11) | -0.4(7) | 8.8(8) | -0.4(7) |
| C33 | 21.5(9) | 18.0(8) | 35.0(11) | 0.3(7) | 7.5(8) | -5.4(7) |
| C34 | 21.6(8) | 21.8(8) | 28.1(10) | -1.4(7) | 8.0(7) | -7.3(7) |
| C35 | 21.9(9) | 25.4(9) | 43.4(13) | 3.3(7) | 10.8(8) | 4.3(9) |
| C36 | 66.9(19) | 44.5(14) | 71(2) | 23.6(13) | 45.3(17) | 30.7(14) |
| B1 | 18.5(9) | 21.3(9) | 17.4(9) | 2.9(7) | -0.5(7) | -4.2(7) |
| F51 | 53.2(10) | 68.0(11) | 64.8(11) | -32.8(8) | -12.2(8) | -2.0(8) |

| | | | | | | |
|---|---|---|---|---|---|---|
| F52 | 44.2(9) | 79.7(12) | 83.9(13) | 26.1(8) | -35.1(9) | -28.3(10) |
| F53 | 48.0(9) | 112.6(14) | 31.6(8) | -17.6(9) | 1.2(7) | -27.9(9) |
| F54 | 169(2) | 28.2(7) | 45.2(9) | 11.8(10) | 49.9(11) | -4.4(6) |
| F55 | 54.5(9) | 26.1(7) | 94.0(13) | -9.5(6) | 14.7(9) | -14.8(7) |
| F56 | 60.8(10) | 25.2(6) | 72.6(11) | 12.1(6) | 6.7(8) | 6.2(7) |
| F57 | 44.2(8) | 41.2(7) | 60.9(10) | 17.0(6) | 22.8(7) | 27.7(7) |
| F58 | 20.9(6) | 36.7(7) | 75.0(11) | 3.8(5) | 3.7(6) | 7.8(7) |
| F59 | 48.6(8) | 47.3(8) | 47.2(9) | 18.8(7) | 14.2(7) | -8.9(6) |
| F60a | 37.5(10) | 63.7(16) | 47.9(11) | -4.3(10) | 28.2(8) | -0.2(9) |
| F60b | 50(4) | 70(3) | 64(3) | 0.2(18) | 30.6(18) | 10.2(14) |
| F61a | 23.5(10) | 69(2) | 55.3(18) | -10.0(10) | 6.9(10) | 6.3(12) |
| F61b | 20(3) | 53(4) | 58(4) | -9.2(16) | 14.3(17) | 11.4(14) |
| F62a | 44.1(12) | 22.0(8) | 137(3) | -0.6(7) | 51.8(15) | 14.2(10) |
| F62b | 28(3) | 35(3) | 114(4) | -13.9(17) | 42(2) | -5.5(16) |
| F63 | 49.8(8) | 38.6(7) | 22.9(6) | 11.9(6) | 7.3(6) | 1.9(5) |
| F64 | 34.7(7) | 47.4(8) | 37.3(7) | -10.1(6) | 14.9(6) | -3.6(6) |
| F65 | 46.2(7) | 38.6(7) | 17.6(6) | -1.7(6) | 5.0(5) | -6.4(5) |
| F66a | 34(2) | 189(6) | 58(3) | 18(3) | -14.2(18) | -24(3) |
| F66b | 56(3) | 135(5) | 23.9(18) | 47(3) | -12.3(16) | -7(2) |
| F67a | 60(3) | 124(5) | 27(2) | 62(3) | -2.5(18) | -1(2) |
| F67b | 35(2) | 229(9) | 52(3) | 31(3) | -7.2(16) | 0(4) |
| F68a | 83(3) | 150(5) | 78(4) | 50(3) | 0(2) | 61(3) |
| F68b | 100(4) | 76(3) | 118(5) | 51(2) | -31(3) | 13(2) |
| F69 | 75.4(11) | 49.1(8) | 24.9(7) | 13.1(7) | 5.6(7) | 0.5(6) |
| F70 | 55.4(8) | 27.5(6) | 39.7(8) | -3.7(6) | 23.3(7) | -3.6(5) |
| F71 | 50.7(9) | 73.6(10) | 60.2(10) | -27.7(8) | 39.7(8) | -25.7(8) |
| F72a | 56.4(16) | 96(2) | 58.0(15) | -50.6(16) | 16.7(11) | -42.2(14) |
| F72b | 76(8) | 74(7) | 76(6) | -52(5) | 35(4) | -40(3) |
| F73a | 28.4(12) | 55.3(19) | 63.4(19) | -23.9(11) | 4.6(11) | -3.6(13) |
| F73b | 42(5) | 58(8) | 57(7) | -27(3) | 18(3) | -14(4) |
| F74a | 60.3(16) | 30.3(11) | 159(5) | -19.1(9) | -3.9(19) | -17.5(15) |
| F74b | 60(6) | 39(4) | 79(6) | -24(3) | 11(3) | -10(3) |
| C51 | 19.4(7) | 20.0(7) | 16.3(8) | -3.3(6) | 4.0(6) | -0.7(6) |
| C52 | 28.0(9) | 19.2(8) | 19.0(9) | -5.2(6) | 4.3(7) | 0.1(6) |
| C53 | 31.2(9) | 18.9(8) | 24.6(9) | -0.5(6) | 8.4(7) | 1.4(6) |
| C54 | 26.4(9) | 27.1(9) | 25.9(10) | 3.7(7) | 5.9(7) | 4.4(7) |
| C55 | 20.5(8) | 30.2(9) | 20.0(9) | -0.4(7) | 1.6(6) | -1.4(7) |
| C56 | 21.4(8) | 20.8(8) | 20.6(9) | -1.9(6) | 3.3(6) | -3.2(6) |
| C57 | 24.3(9) | 41.3(11) | 31.0(11) | 0.8(8) | -3.8(7) | -4.9(8) |
| C58 | 49.0(13) | 21.3(9) | 35.7(12) | 1.8(8) | 10.5(9) | 0.9(8) |
| C59 | 19.2(8) | 20.5(8) | 16.1(8) | -2.6(6) | 3.3(6) | 0.1(6) |
| C60 | 21.8(8) | 20.7(8) | 24.5(9) | -0.7(6) | 6.9(7) | -1.4(7) |
| C61 | 22.0(8) | 22.7(8) | 29.8(10) | -4.2(6) | 11.1(7) | -0.8(7) |
| C62 | 25.2(9) | 19.9(8) | 26.1(10) | -2.1(6) | 8.9(7) | -0.3(7) |
| C63 | 20.8(8) | 21.9(8) | 20.1(9) | 1.3(6) | 4.9(6) | 1.5(6) |
| C64 | 18.1(7) | 24.2(8) | 18.0(8) | -1.8(6) | 4.6(6) | 1.7(6) |
| C65 | 23.7(9) | 26.3(9) | 34.1(11) | 2.2(7) | 8.4(7) | 6.0(7) |
| C66 | 26.7(9) | 25.7(9) | 52.3(13) | -1.5(7) | 19.5(8) | 5.2(8) |
| C67 | 21.8(8) | 22.1(8) | 17.3(8) | -4.7(6) | 0.6(6) | -0.1(6) |
| C68 | 22.4(8) | 21.4(8) | 17.4(8) | -4.3(7) | 1.4(6) | 0.6(6) |
| C69 | 26.2(9) | 24.1(8) | 17.6(8) | -6.1(7) | 3.5(6) | -1.2(6) |
| C70 | 33.1(10) | 46.0(12) | 16.0(9) | -0.3(8) | -0.4(7) | -0.3(8) |
| C71 | 29.4(10) | 56.9(14) | 22.3(10) | 7.9(9) | -3.6(7) | 0.4(9) |
| C72 | 26.5(10) | 43.9(12) | 21.6(10) | 3.0(8) | 0.2(7) | -2.3(8) |
| C73 | 33.3(10) | 30.3(9) | 17.2(9) | -2.5(7) | 4.1(7) | 0.2(7) |
| C74a | 45(3) | 108(4) | 27(3) | 40.9(17) | -5.6(15) | -2.5(17) |

| | | | | | | |
|---|---|---|---|---|---|---|
| C74b | 51(4) | 104(4) | 26(3) | 41.0(17) | -2.2(17) | -0.7(18) |
| C75 | 17.4(8) | 20.6(8) | 18.5(8) | -2.8(6) | 0.5(6) | 2.3(6) |
| C76 | 19.2(8) | 21.5(8) | 20.4(8) | -5.5(6) | 3.3(6) | 0.6(6) |
| C77 | 22.7(8) | 25.1(8) | 23.4(9) | -3.6(7) | 6.5(7) | 2.4(7) |
| C78 | 24.0(9) | 31.1(10) | 36.3(11) | -9.9(7) | 10.4(8) | -0.4(8) |
| C79 | 24.7(9) | 32.5(10) | 36.5(12) | -13.4(7) | 6.8(8) | -6.9(8) |
| C80 | 23.3(9) | 27.7(9) | 26.0(10) | -7.7(7) | 3.6(7) | -5.1(7) |
| C81 | 31.2(10) | 29.9(9) | 28.7(10) | -6.7(7) | 12.2(8) | 1.1(7) |
| C82a | 34.2(19) | 42.0(19) | 59(2) | -20.1(13) | 13.8(14) | -18.1(14) |
| C82b | 33(4) | 40(4) | 59(4) | -22.3(16) | 13.1(19) | -17.9(17) |
| B51 | 18.4(8) | 19.1(8) | 18.0(9) | -3.2(6) | 2.5(6) | -0.3(6) |
| O91 | 49.2(10) | 33.1(8) | 26.3(8) | -8.9(7) | -4.3(7) | 8.2(6) |
| C91 | 49.0(15) | 50.8(15) | 52.9(17) | -18.6(11) | 4.2(12) | -0.8(12) |
| C92a | 42(3) | 38(2) | 75(3) | -3.4(14) | 29.6(19) | 8.9(15) |
| C92b | 110(12) | 57(8) | 77(8) | -41(7) | 50(7) | -2(6) |
| C93a | 63(3) | 48(2) | 38(2) | 15.9(17) | 26.0(19) | 15.5(14) |
| C93b | 40(7) | 88(8) | 105(9) | 18(5) | 27(6) | 82(6) |
| C94 | 57.7(16) | 58.4(15) | 27.5(12) | 0.7(12) | 0.2(10) | 15.1(10) |

9.9.9 Bond lengths

Table S59. Bond Lengths in Å for **9a**.

| Atom | Atom | Length/Å |
|---|---|---|
| Sb1 | C1 | 2.1584(17) |
| Sb1 | C8 | 2.1570(17) |
| Sb1 | C29 | 2.1835(19) |
| F1 | C30 | 1.354(2) |
| F2 | C31 | 1.339(2) |
| F3 | C33 | 1.339(2) |
| F4 | C34 | 1.348(2) |
| O1 | C35 | 1.200(3) |
| O2 | C35 | 1.330(3) |
| O2 | C36 | 1.451(3) |
| N1 | C1 | 1.346(2) |
| N1 | C2 | 1.380(2) |
| N1 | B1 | 1.584(3) |
| N2 | C1 | 1.364(2) |
| N2 | C3 | 1.382(2) |
| N2 | C4 | 1.504(2) |
| N3 | C8 | 1.348(2) |
| N3 | C9 | 1.377(2) |
| N3 | B1 | 1.601(2) |
| N4 | C8 | 1.360(2) |
| N4 | C10 | 1.378(2) |
| N4 | C11 | 1.510(2) |
| C2 | C3 | 1.342(3) |
| C4 | C5 | 1.525(3) |
| C4 | C6 | 1.530(3) |
| C4 | C7 | 1.516(3) |
| C9 | C10 | 1.351(3) |
| C11 | C12 | 1.528(3) |
| C11 | C13 | 1.524(3) |
| C11 | C14 | 1.526(3) |
| C15 | C16 | 1.399(3) |
| C15 | C20 | 1.396(3) |
| C15 | B1 | 1.620(3) |
| C16 | C17 | 1.394(3) |
| C17 | C18 | 1.396(4) |
| C18 | C19 | 1.373(4) |
| C18 | C21 | 1.511(3) |
| C19 | C20 | 1.395(3) |
| C22 | C23 | 1.402(3) |
| C22 | C27 | 1.398(3) |
| C22 | B1 | 1.609(3) |
| C23 | C24 | 1.392(3) |
| C24 | C25 | 1.390(3) |
| C25 | C26 | 1.393(3) |
| C25 | C28 | 1.509(3) |
| C26 | C27 | 1.386(3) |
| C29 | C30 | 1.379(3) |
| C29 | C34 | 1.387(3) |
| C30 | C31 | 1.378(3) |
| C31 | C32 | 1.395(3) |
| C32 | C33 | 1.381(3) |
| C32 | C35 | 1.498(3) |
| C33 | C34 | 1.378(3) |
| F51 | C57 | 1.339(3) |
| F52 | C57 | 1.310(3) |
| F53 | C57 | 1.315(3) |
| F54 | C58 | 1.320(3) |
| F55 | C58 | 1.335(3) |
| F56 | C58 | 1.327(3) |
| F57 | C65 | 1.332(2) |
| F58 | C65 | 1.340(2) |
| F59 | C65 | 1.339(3) |
| F60a | C66 | 1.372(3) |
| F60b | C66 | 1.224(10) |
| F61a | C66 | 1.315(4) |
| F61b | C66 | 1.337(13) |
| F62a | C66 | 1.326(3) |
| F62b | C66 | 1.412(9) |
| F63 | C73 | 1.343(2) |
| F64 | C73 | 1.340(2) |
| F65 | C73 | 1.346(2) |
| F66a | C74a | 1.383(13) |
| F66b | C74b | 1.306(11) |
| F67a | C74a | 1.340(10) |
| F67b | C74b | 1.377(15) |
| F68a | C74a | 1.345(14) |
| F68b | C74b | 1.310(15) |
| F69 | C81 | 1.341(3) |
| F70 | C81 | 1.336(2) |
| F71 | C81 | 1.336(2) |
| F72a | C82a | 1.345(6) |
| F72b | C82b | 1.26(2) |
| F73a | C82a | 1.315(5) |
| F73b | C82b | 1.31(2) |
| F74a | C82a | 1.338(6) |
| F74b | C82b | 1.31(2) |
| C51 | C52 | 1.406(2) |
| C51 | C56 | 1.395(2) |
| C51 | B51 | 1.636(3) |
| C52 | C53 | 1.383(3) |
| C53 | C54 | 1.387(3) |
| C53 | C58 | 1.497(3) |
| C54 | C55 | 1.385(3) |
| C55 | C56 | 1.395(3) |
| C55 | C57 | 1.494(3) |
| C59 | C60 | 1.403(2) |
| C59 | C64 | 1.403(2) |
| C59 | B51 | 1.638(3) |
| C60 | C61 | 1.388(3) |
| C61 | C62 | 1.385(3) |
| C61 | C66 | 1.492(3) |
| C62 | C63 | 1.389(3) |
| C63 | C64 | 1.394(2) |
| C63 | C65 | 1.496(3) |
| C67 | C68 | 1.393(3) |

| | | |
|---|---|---|
| C67 | C72 | 1.400(3) |
| C67 | B51 | 1.644(3) |
| C68 | C69 | 1.393(3) |
| C69 | C70 | 1.384(3) |
| C69 | C73 | 1.499(3) |
| C70 | C71 | 1.390(3) |
| C71 | C72 | 1.383(3) |
| C71 | C74a | 1.436(10) |
| C71 | C74b | 1.604(11) |
| C75 | C76 | 1.399(3) |
| C75 | C80 | 1.402(2) |
| C75 | B51 | 1.634(3) |
| C76 | C77 | 1.396(2) |
| C77 | C78 | 1.387(3) |
| C77 | C81 | 1.491(3) |
| C78 | C79 | 1.387(3) |
| C79 | C80 | 1.387(3) |
| C79 | C82a | 1.496(5) |
| C79 | C82b | 1.52(2) |
| O91 | C91 | 1.429(3) |
| O91 | C94 | 1.436(3) |
| C91 | C92a | 1.547(5) |
| C91 | C92b | 1.328(13) |
| C92a | C93a | 1.497(7) |
| C92b | C93b | 1.42(2) |
| C93a | C94 | 1.446(5) |
| C93b | C94 | 1.544(14) |

9.9.10 Bond Angles

Table S60. Bond Angles in ° for **9a**.

| Atom | Atom | Atom | Angle/° |
|---|---|---|---|
| C8 | Sb1 | C1 | 89.72(6) |
| C29 | Sb1 | C1 | 93.44(7) |
| C29 | Sb1 | C8 | 95.51(7) |
| C36 | O2 | C35 | 114.90(19) |
| C2 | N1 | C1 | 108.00(15) |
| B1 | N1 | C1 | 129.84(14) |
| B1 | N1 | C2 | 122.09(15) |
| C3 | N2 | C1 | 107.82(15) |
| C4 | N2 | C1 | 131.97(15) |
| C4 | N2 | C3 | 120.09(15) |
| C9 | N3 | C8 | 107.79(14) |
| B1 | N3 | C8 | 129.95(14) |
| B1 | N3 | C9 | 121.87(14) |
| C10 | N4 | C8 | 108.12(14) |
| C11 | N4 | C8 | 132.38(14) |
| C11 | N4 | C10 | 119.46(14) |
| N1 | C1 | Sb1 | 122.77(12) |
| N2 | C1 | Sb1 | 129.05(13) |
| N2 | C1 | N1 | 108.18(15) |
| C3 | C2 | N1 | 108.39(17) |
| C2 | C3 | N2 | 107.61(16) |
| C5 | C4 | N2 | 106.99(17) |
| C6 | C4 | N2 | 107.29(17) |
| C6 | C4 | C5 | 111.80(19) |
| C7 | C4 | N2 | 112.69(16) |
| C7 | C4 | C5 | 109.37(18) |
| C7 | C4 | C6 | 108.72(18) |
| N3 | C8 | Sb1 | 121.65(12) |
| N4 | C8 | Sb1 | 129.41(12) |
| N4 | C8 | N3 | 108.38(14) |
| C10 | C9 | N3 | 108.43(15) |
| C9 | C10 | N4 | 107.27(15) |
| C12 | C11 | N4 | 108.03(14) |
| C13 | C11 | N4 | 111.83(14) |
| C13 | C11 | C12 | 109.59(16) |
| C14 | C11 | N4 | 107.27(15) |
| C14 | C11 | C12 | 110.87(16) |
| C14 | C11 | C13 | 109.24(15) |
| C20 | C15 | C16 | 116.43(18) |
| B1 | C15 | C16 | 121.56(17) |
| B1 | C15 | C20 | 121.96(19) |
| C17 | C16 | C15 | 122.0(2) |
| C18 | C17 | C16 | 120.6(2) |
| C19 | C18 | C17 | 117.9(2) |
| C21 | C18 | C17 | 120.7(3) |
| C21 | C18 | C19 | 121.4(3) |
| C20 | C19 | C18 | 121.7(2) |
| C19 | C20 | C15 | 121.4(2) |
| C27 | C22 | C23 | 115.93(18) |
| B1 | C22 | C23 | 122.18(17) |
| B1 | C22 | C27 | 121.46(16) |
| C24 | C23 | C22 | 121.92(19) |
| C25 | C24 | C23 | 121.19(19) |
| C26 | C25 | C24 | 117.5(2) |
| C28 | C25 | C24 | 121.1(2) |
| C28 | C25 | C26 | 121.4(2) |
| C27 | C26 | C25 | 121.1(2) |
| C26 | C27 | C22 | 122.35(19) |
| C30 | C29 | Sb1 | 123.43(13) |
| C34 | C29 | Sb1 | 120.61(14) |
| C34 | C29 | C30 | 115.33(17) |
| C29 | C30 | F1 | 119.44(17) |
| C31 | C30 | F1 | 117.65(17) |
| C31 | C30 | C29 | 122.91(17) |
| C30 | C31 | F2 | 117.40(17) |
| C32 | C31 | F2 | 121.36(18) |
| C32 | C31 | C30 | 121.22(18) |
| C33 | C32 | C31 | 116.19(18) |
| C35 | C32 | C31 | 123.97(18) |
| C35 | C32 | C33 | 119.80(17) |
| C32 | C33 | F3 | 120.78(17) |
| C34 | C33 | F3 | 117.48(17) |
| C34 | C33 | C32 | 121.73(17) |
| C29 | C34 | F4 | 118.98(17) |
| C33 | C34 | F4 | 118.45(16) |
| C33 | C34 | C29 | 122.57(18) |
| O2 | C35 | O1 | 123.9(2) |
| C32 | C35 | O1 | 124.2(2) |
| C32 | C35 | O2 | 111.89(17) |
| N3 | B1 | N1 | 107.20(14) |
| C15 | B1 | N1 | 108.09(15) |
| C15 | B1 | N3 | 107.45(14) |
| C22 | B1 | N1 | 111.06(14) |
| C22 | B1 | N3 | 110.46(15) |
| C22 | B1 | C15 | 112.37(15) |
| C56 | C51 | C52 | 115.36(16) |
| B51 | C51 | C52 | 119.82(15) |
| B51 | C51 | C56 | 124.76(15) |
| C53 | C52 | C51 | 122.23(17) |
| C54 | C53 | C52 | 121.42(17) |
| C58 | C53 | C52 | 118.12(18) |
| C58 | C53 | C54 | 120.43(18) |
| C55 | C54 | C53 | 117.51(18) |
| C56 | C55 | C54 | 120.94(18) |
| C57 | C55 | C54 | 119.53(18) |
| C57 | C55 | C56 | 119.51(18) |
| C55 | C56 | C51 | 122.50(17) |
| F52 | C57 | F51 | 106.0(2) |
| F53 | C57 | F51 | 103.8(2) |
| F53 | C57 | F52 | 108.7(2) |
| C55 | C57 | F51 | 112.26(19) |
| C55 | C57 | F52 | 113.25(19) |
| C55 | C57 | F53 | 112.21(18) |
| F55 | C58 | F54 | 107.1(2) |
| F56 | C58 | F54 | 106.5(2) |

| | | | |
|---|---|---|---|
| F56 | C58 | F55 | 105.31(18) |
| C53 | C58 | F54 | 112.03(18) |
| C53 | C58 | F55 | 111.88(19) |
| C53 | C58 | F56 | 113.56(19) |
| C64 | C59 | C60 | 115.79(16) |
| B51 | C59 | C60 | 119.86(15) |
| B51 | C59 | C64 | 122.64(15) |
| C61 | C60 | C59 | 122.20(17) |
| C62 | C61 | C60 | 121.00(17) |
| C66 | C61 | C60 | 118.94(17) |
| C66 | C61 | C62 | 120.01(17) |
| C63 | C62 | C61 | 118.15(17) |
| C64 | C63 | C62 | 120.72(16) |
| C65 | C63 | C62 | 119.00(16) |
| C65 | C63 | C64 | 120.28(16) |
| C63 | C64 | C59 | 122.09(16) |
| F58 | C65 | F57 | 106.84(17) |
| F59 | C65 | F57 | 105.51(17) |
| F59 | C65 | F58 | 105.81(17) |
| C63 | C65 | F57 | 112.88(17) |
| C63 | C65 | F58 | 112.80(16) |
| C63 | C65 | F59 | 112.43(17) |
| F60b | C66 | F60a | 32.4(5) |
| F61a | C66 | F60a | 104.3(2) |
| F61a | C66 | F60b | 123.4(5) |
| F61b | C66 | F60a | 84.7(6) |
| F61b | C66 | F60b | 108.5(8) |
| F61b | C66 | F61a | 20.8(6) |
| F62a | C66 | F60a | 104.2(2) |
| F62a | C66 | F60b | 72.9(6) |
| F62a | C66 | F61a | 109.9(3) |
| F62a | C66 | F61b | 122.4(6) |
| F62b | C66 | F60a | 135.2(4) |
| F62b | C66 | F60b | 109.8(7) |
| F62b | C66 | F61a | 76.3(5) |
| F62b | C66 | F61b | 95.0(7) |
| F62b | C66 | F62a | 40.3(5) |
| C61 | C66 | F60a | 110.87(19) |
| C61 | C66 | F60b | 116.6(5) |
| C61 | C66 | F61a | 112.9(2) |
| C61 | C66 | F61b | 115.2(7) |
| C61 | C66 | F62a | 113.90(18) |
| C61 | C66 | F62b | 109.6(4) |
| C72 | C67 | C68 | 115.76(17) |
| B51 | C67 | C68 | 123.72(16) |
| B51 | C67 | C72 | 120.01(17) |
| C69 | C68 | C67 | 122.39(17) |
| C70 | C69 | C68 | 120.59(18) |
| C73 | C69 | C68 | 118.75(17) |
| C73 | C69 | C70 | 120.65(17) |
| C71 | C70 | C69 | 118.01(19) |
| C72 | C71 | C70 | 120.9(2) |
| C74a | C71 | C70 | 115.2(4) |
| C74a | C71 | C72 | 123.9(4) |
| C74b | C71 | C70 | 123.9(4) |
| C74b | C71 | C72 | 115.0(4) |

| | | | |
|---|---|---|---|
| C74b | C71 | C74a | 11.2(8) |
| C71 | C72 | C67 | 122.3(2) |
| F64 | C73 | F63 | 106.34(17) |
| F65 | C73 | F63 | 106.26(16) |
| F65 | C73 | F64 | 106.19(16) |
| C69 | C73 | F63 | 112.43(16) |
| C69 | C73 | F64 | 112.61(16) |
| C69 | C73 | F65 | 112.51(17) |
| F67a | C74a | F66a | 105.9(10) |
| F68a | C74a | F66a | 101.3(8) |
| F68a | C74a | F67a | 108.7(8) |
| C71 | C74a | F66a | 112.7(8) |
| C71 | C74a | F67a | 113.3(7) |
| C71 | C74a | F68a | 114.0(9) |
| F67b | C74b | F66b | 101.2(11) |
| F68b | C74b | F66b | 113.5(10) |
| F68b | C74b | F67b | 113.7(9) |
| C71 | C74b | F66b | 108.4(7) |
| C71 | C74b | F67b | 106.3(9) |
| C71 | C74b | F68b | 112.9(9) |
| C80 | C75 | C76 | 115.83(17) |
| B51 | C75 | C76 | 123.01(15) |
| B51 | C75 | C80 | 120.85(16) |
| C77 | C76 | C75 | 122.16(16) |
| C78 | C77 | C76 | 120.71(18) |
| C81 | C77 | C76 | 119.52(17) |
| C81 | C77 | C78 | 119.70(17) |
| C79 | C78 | C77 | 118.09(18) |
| C80 | C79 | C78 | 120.99(18) |
| C82a | C79 | C78 | 120.3(3) |
| C82a | C79 | C80 | 118.7(3) |
| C82b | C79 | C78 | 118.1(10) |
| C82b | C79 | C80 | 119.3(10) |
| C82b | C79 | C82a | 13.4(10) |
| C79 | C80 | C75 | 122.20(19) |
| F70 | C81 | F69 | 105.07(17) |
| F71 | C81 | F69 | 106.31(18) |
| F71 | C81 | F70 | 106.19(18) |
| C77 | C81 | F69 | 112.31(18) |
| C77 | C81 | F70 | 113.28(17) |
| C77 | C81 | F71 | 113.05(17) |
| F73a | C82a | F72a | 101.9(4) |
| F74a | C82a | F72a | 104.8(4) |
| F74a | C82a | F73a | 112.2(5) |
| C79 | C82a | F72a | 112.5(4) |
| C79 | C82a | F73a | 114.3(4) |
| C79 | C82a | F74a | 110.5(4) |
| F73b | C82b | F72b | 117(2) |
| F74b | C82b | F72b | 114(2) |
| F74b | C82b | F73b | 76.4(16) |
| C79 | C82b | F72b | 114.3(17) |
| C79 | C82b | F73b | 112.4(19) |
| C79 | C82b | F74b | 117.6(17) |
| C59 | B51 | C51 | 114.27(14) |
| C67 | B51 | C51 | 111.37(15) |
| C67 | B51 | C59 | 100.44(14) |

| | | | |
|---|---|---|---|
| C75 | B51 | C51 | 105.64(14) |
| C75 | B51 | C59 | 113.72(15) |
| C75 | B51 | C67 | 111.56(14) |
| C94 | O91 | C91 | 107.08(19) |
| C92a | C91 | O91 | 105.0(3) |
| C92b | C91 | O91 | 113.9(7) |
| C92b | C91 | C92a | 27.8(11) |

| | | | |
|---|---|---|---|
| C93a | C92a | C91 | 106.0(3) |
| C93b | C92b | C91 | 104.6(10) |
| C94 | C93a | C92a | 104.6(4) |
| C94 | C93b | C92b | 107.6(9) |
| C93a | C94 | O91 | 108.2(3) |
| C93b | C94 | O91 | 101.3(6) |
| C93b | C94 | C93a | 22.4(5) |

9.9.11 Torsion Angles

Table S61. Torsion Angles in ° for **9a**.

| Atom | Atom | Atom | Atom | Angle/° |
|---|---|---|---|---|
| Sb1 | C1 | N1 | C2 | -178.57(15) |
| Sb1 | C1 | N1 | B1 | -1.73(19) |
| Sb1 | C1 | N2 | C3 | 178.58(16) |
| Sb1 | C1 | N2 | C4 | -5.6(2) |
| Sb1 | C8 | N3 | C9 | -173.08(14) |
| Sb1 | C8 | N3 | B1 | 14.21(19) |
| Sb1 | C8 | N4 | C10 | 172.14(16) |
| Sb1 | C8 | N4 | C11 | -10.2(2) |
| Sb1 | C29 | C30 | F1 | 7.08(17) |
| Sb1 | C29 | C30 | C31 | -173.09(17) |
| Sb1 | C29 | C34 | F4 | -9.10(15) |
| Sb1 | C29 | C34 | C33 | 171.66(14) |
| F1 | C30 | C29 | C34 | 177.99(19) |
| F1 | C30 | C31 | F2 | 1.2(2) |
| F1 | C30 | C31 | C32 | -177.35(19) |
| F2 | C31 | C30 | C29 | -178.62(18) |
| F2 | C31 | C32 | C33 | 179.9(2) |
| F2 | C31 | C32 | C35 | -2.4(3) |
| F3 | C33 | C32 | C31 | -178.76(19) |
| F3 | C33 | C32 | C35 | 3.5(2) |
| F3 | C33 | C34 | F4 | 0.1(2) |
| F3 | C33 | C34 | C29 | 179.34(17) |
| F4 | C34 | C29 | C30 | 179.71(18) |
| F4 | C34 | C33 | C32 | -178.59(17) |
| O1 | C35 | O2 | C36 | 1.0(3) |
| O1 | C35 | C32 | C31 | -155.3(2) |
| O1 | C35 | C32 | C33 | 22.2(3) |
| O2 | C35 | C32 | C31 | 25.2(2) |
| O2 | C35 | C32 | C33 | -157.21(17) |
| N1 | C1 | N2 | C3 | -1.01(17) |
| N1 | C1 | N2 | C4 | 174.84(14) |
| N1 | C2 | C3 | N2 | 0.08(18) |
| N1 | B1 | N3 | C8 | -45.59(19) |
| N1 | B1 | N3 | C9 | 142.58(14) |
| N1 | B1 | C15 | C16 | -53.55(17) |
| N1 | B1 | C15 | C20 | 129.19(15) |
| N1 | B1 | C22 | C23 | -29.62(18) |
| N1 | B1 | C22 | C27 | 158.25(15) |
| N3 | C8 | N4 | C10 | 0.70(17) |
| N3 | C8 | N4 | C11 | 178.37(13) |
| N3 | C9 | C10 | N4 | -0.24(17) |
| N3 | B1 | C15 | C16 | 61.84(18) |
| N3 | B1 | C15 | C20 | -115.42(16) |
| N3 | B1 | C22 | C23 | -148.43(14) |
| N3 | B1 | C22 | C27 | 39.45(18) |
| C15 | C16 | C17 | C18 | -0.1(2) |
| C15 | C20 | C19 | C18 | -0.9(2) |
| C15 | B1 | C22 | C23 | 91.60(17) |
| C15 | B1 | C22 | C27 | -80.53(18) |
| C16 | C17 | C18 | C19 | -1.6(2) |
| C16 | C17 | C18 | C21 | 176.9(2) |

| | | | | |
|---|---|---|---|---|
| C17 | C18 | C19 | C20 | 2.1(3) |
| C22 | C23 | C24 | C25 | -0.1(2) |
| C22 | C27 | C26 | C25 | 0.1(3) |
| C23 | C24 | C25 | C26 | -0.4(2) |
| C23 | C24 | C25 | C28 | 179.0(2) |
| C24 | C25 | C26 | C27 | 0.4(3) |
| C29 | C30 | C31 | C32 | 2.8(3) |
| C29 | C34 | C33 | C32 | 0.7(2) |
| C30 | C31 | C32 | C33 | -1.5(3) |
| C30 | C31 | C32 | C35 | 176.1(2) |
| C31 | C32 | C33 | C34 | -0.1(2) |
| F51 | C57 | C55 | C54 | 137.57(19) |
| F51 | C57 | C55 | C56 | -44.3(2) |
| F52 | C57 | C55 | C54 | 17.6(3) |
| F52 | C57 | C55 | C56 | -164.3(2) |
| F53 | C57 | C55 | C54 | -106.0(2) |
| F53 | C57 | C55 | C56 | 72.2(2) |
| F54 | C58 | C53 | C52 | 63.6(2) |
| F54 | C58 | C53 | C54 | -114.6(2) |
| F55 | C58 | C53 | C52 | -56.7(2) |
| F55 | C58 | C53 | C54 | 125.1(2) |
| F56 | C58 | C53 | C52 | -175.73(18) |
| F56 | C58 | C53 | C54 | 6.1(2) |
| F57 | C65 | C63 | C62 | 31.3(2) |
| F57 | C65 | C63 | C64 | -149.48(17) |
| F58 | C65 | C63 | C62 | 152.49(17) |
| F58 | C65 | C63 | C64 | -28.2(2) |
| F59 | C65 | C63 | C62 | -87.96(19) |
| F59 | C65 | C63 | C64 | 91.30(18) |
| F60a | C66 | C61 | C60 | 68.5(2) |
| F60a | C66 | C61 | C62 | -108.8(2) |
| F60b | C66 | C61 | C60 | 103.5(7) |
| F60b | C66 | C61 | C62 | -73.8(7) |
| F61a | C66 | C61 | C60 | -48.2(3) |
| F61a | C66 | C61 | C62 | 134.5(2) |
| F61b | C66 | C61 | C60 | -25.5(7) |
| F61b | C66 | C61 | C62 | 157.2(7) |
| F62a | C66 | C61 | C60 | -174.4(3) |
| F62a | C66 | C61 | C62 | 8.3(3) |
| F62b | C66 | C61 | C60 | -131.1(6) |
| F62b | C66 | C61 | C62 | 51.6(6) |
| F63 | C73 | C69 | C68 | 33.69(19) |
| F63 | C73 | C69 | C70 | -147.87(17) |
| F64 | C73 | C69 | C68 | -86.42(18) |
| F64 | C73 | C69 | C70 | 92.02(19) |
| F65 | C73 | C69 | C68 | 153.61(15) |
| F65 | C73 | C69 | C70 | -27.9(2) |
| F66a | C74a | C71 | C70 | 55.4(10) |
| F66a | C74a | C71 | C72 | -122.5(6) |
| F66a | C74a | C71 | C74b | -162(2) |
| F66b | C74b | C71 | C70 | 25.7(13) |
| F66b | C74b | C71 | C72 | -160.1(8) |
| F66b | C74b | C71 | C74a | -15.4(19) |
| F67a | C74a | C71 | C70 | 175.6(8) |
| F67a | C74a | C71 | C72 | -2.2(14) |
| F67a | C74a | C71 | C74b | -41(2) |

| | | | | |
|---|---|---|---|---|
| F67b | C74b | C71 | C70 | 133.8(7) |
| F67b | C74b | C71 | C72 | -52.0(11) |
| F67b | C74b | C71 | C74a | 93(2) |
| F68a | C74a | C71 | C70 | -59.4(9) |
| F68a | C74a | C71 | C72 | 122.7(7) |
| F68a | C74a | C71 | C74b | 84(2) |
| F68b | C74b | C71 | C70 | -100.9(8) |
| F68b | C74b | C71 | C72 | 73.3(10) |
| F68b | C74b | C71 | C74a | -142(2) |
| F69 | C81 | C77 | C76 | -77.11(19) |
| F69 | C81 | C77 | C78 | 99.87(19) |
| F70 | C81 | C77 | C76 | 41.7(2) |
| F70 | C81 | C77 | C78 | -141.28(18) |
| F71 | C81 | C77 | C76 | 162.59(17) |
| F71 | C81 | C77 | C78 | -20.4(2) |
| F72a | C82a | C79 | C78 | 143.6(3) |
| F72a | C82a | C79 | C80 | -35.6(4) |
| F72a | C82a | C79 | C82b | -132(3) |
| F72b | C82b | C79 | C78 | 173.5(15) |
| F72b | C82b | C79 | C80 | -21(2) |
| F72b | C82b | C79 | C82a | 71(3) |
| F73a | C82a | C79 | C78 | 28.0(5) |
| F73a | C82a | C79 | C80 | -151.2(3) |
| F73a | C82a | C79 | C82b | 113(3) |
| F73b | C82b | C79 | C78 | 37(2) |
| F73b | C82b | C79 | C80 | -157.5(13) |
| F73b | C82b | C79 | C82a | -66(3) |
| F74a | C82a | C79 | C78 | -99.7(5) |
| F74a | C82a | C79 | C80 | 81.2(5) |
| F74a | C82a | C79 | C82b | -15(4) |
| F74b | C82b | C79 | C78 | -49(2) |
| F74b | C82b | C79 | C80 | 116.5(19) |
| F74b | C82b | C79 | C82a | -152(5) |
| C51 | C52 | C53 | C54 | 2.0(2) |
| C51 | C52 | C53 | C58 | -176.19(19) |
| C51 | C56 | C55 | C54 | 1.4(2) |
| C51 | C56 | C55 | C57 | -176.77(19) |
| C51 | B51 | C59 | C60 | 44.22(19) |
| C51 | B51 | C59 | C64 | -151.28(15) |
| C51 | B51 | C67 | C68 | -19.54(18) |
| C51 | B51 | C67 | C72 | 169.02(16) |
| C51 | B51 | C75 | C76 | 83.30(16) |
| C51 | B51 | C75 | C80 | -90.01(16) |
| C52 | C53 | C54 | C55 | -0.5(2) |
| C53 | C54 | C55 | C56 | -1.1(2) |
| C53 | C54 | C55 | C57 | 177.02(19) |
| C59 | C60 | C61 | C62 | -0.2(2) |
| C59 | C60 | C61 | C66 | -177.5(2) |
| C59 | C64 | C63 | C62 | -1.4(2) |
| C59 | C64 | C63 | C65 | 179.38(18) |
| C59 | B51 | C67 | C68 | 101.85(15) |
| C59 | B51 | C67 | C72 | -69.59(17) |
| C59 | B51 | C75 | C76 | -42.81(19) |
| C59 | B51 | C75 | C80 | 143.89(16) |
| C60 | C61 | C62 | C63 | 1.3(2) |
| C61 | C62 | C63 | C64 | -0.5(2) |

| | | | | |
|---|---|---|---|---|
| C61 | C62 | C63 | C65 | 178.79(18) |
| C67 | C68 | C69 | C70 | 2.6(2) |
| C67 | C68 | C69 | C73 | -178.92(17) |
| C67 | C72 | C71 | C70 | 2.4(3) |
| C67 | C72 | C71 | C74a | -179.8(7) |
| C67 | C72 | C71 | C74b | -172.1(7) |
| C67 | B51 | C75 | C76 | -155.54(15) |
| C67 | B51 | C75 | C80 | 31.15(19) |
| C68 | C69 | C70 | C71 | -1.1(2) |
| C69 | C70 | C71 | C72 | -1.4(3) |
| C69 | C70 | C71 | C74a | -179.3(7) |
| C69 | C70 | C71 | C74b | 172.6(7) |
| C75 | C76 | C77 | C78 | 1.3(2) |
| C75 | C76 | C77 | C81 | 178.30(18) |
| C75 | C80 | C79 | C78 | 0.7(2) |
| C75 | C80 | C79 | C82a | 179.8(3) |
| C75 | C80 | C79 | C82b | -164.9(11) |
| C76 | C77 | C78 | C79 | -0.2(2) |
| C77 | C78 | C79 | C80 | -0.7(2) |
| C77 | C78 | C79 | C82a | -179.9(3) |
| C77 | C78 | C79 | C82b | 165.0(11) |
| O91 | C91 | C92a | C93a | 7.7(3) |
| O91 | C91 | C92b | C93b | -18.0(9) |
| O91 | C94 | C93a | C92a | -26.1(3) |
| O91 | C94 | C93b | C92b | -20.9(10) |
| C91 | C92a | C93a | C94 | 10.9(4) |
| C91 | C92b | C93b | C94 | 23.8(18) |
| C92a | C93a | C94 | C93b | 48.9(18) |
| C92b | C93b | C94 | C93a | -132(3) |

9.9.12 Hydrogen Fractional Atomic Coordinates and Equivalent Isotropic Displacement Parameters

Table S62. Hydrogen Fractional Atomic Coordinates (×10[4]) and Equivalent Isotropic Displacement Parameters (Å[2]×10[3]) for **9a**. $U_{eq}$ is defined as 1/3 of the trace of the orthogonalised $U_{ij}$.

| Atom | x | y | z | $U_{eq}$ |
|---|---|---|---|---|
| H2 | 2577.7(5) | 1697.1(16) | 3001.3(6) | 30.8(5) |
| H3 | 2636.3(5) | 2495.0(16) | 3663.4(6) | 30.8(5) |
| H5a | 2885.0(8) | 1815(11) | 4325(3) | 60.9(8) |
| H5b | 3259(4) | 1065(3) | 4402(4) | 60.9(8) |
| H5c | 3230(4) | 2034(9) | 4701.5(11) | 60.9(8) |
| H6a | 3314(4) | 4201(4) | 4018(4) | 62.3(9) |
| H6b | 2924.4(8) | 3754(3) | 4107(5) | 62.3(9) |
| H6c | 3282(5) | 4009(6) | 4466.5(13) | 62.3(9) |
| H7a | 3871.0(8) | 1813(3) | 4326(4) | 46.1(7) |
| H7b | 3896.0(7) | 3018(10) | 4161(3) | 46.1(7) |
| H7c | 3842.9(6) | 2835(12) | 4603.0(17) | 46.1(7) |
| H9 | 3382.6(5) | -1103.9(14) | 2480.2(6) | 26.1(4) |
| H10 | 3999.2(5) | -2022.7(14) | 2749.4(5) | 25.0(4) |
| H12a | 4396(4) | -2663(3) | 3331.5(7) | 41.6(6) |
| H12b | 4757.5(8) | -2355(6) | 3680(4) | 41.6(6) |
| H12c | 4335(4) | -2060(3) | 3721(3) | 41.6(6) |
| H13a | 4569(3) | -160(9) | 3859(2) | 39.9(6) |
| H13b | 4978.1(15) | -430(7) | 3774(3) | 39.9(6) |
| H13c | 4719(4) | 510(3) | 3529.5(10) | 39.9(6) |
| H14a | 5030.8(11) | -1307(12) | 3156.8(8) | 41.3(6) |
| H14b | 4658(3) | -1679(9) | 2834(3) | 41.3(6) |
| H14c | 4763(4) | -419(4) | 2891(3) | 41.3(6) |
| H16 | 2811.7(6) | -603.5(16) | 3021.0(7) | 35.6(5) |
| H17 | 2289.0(6) | -1637.7(18) | 2702.4(8) | 44.8(6) |
| H19 | 2251.5(7) | 0.9(18) | 1688.5(8) | 49.2(7) |
| H20 | 2779.2(6) | 1020.4(17) | 2001.3(6) | 37.2(5) |
| H21a | 1995.6(8) | -2288(10) | 1835(6) | 93.5(14) |
| H21b | 1743(4) | -1881(16) | 2131.0(19) | 93.5(14) |
| H21c | 1730(4) | -1237(7) | 1727(4) | 93.5(14) |
| H23 | 3086.3(6) | 3188.7(15) | 2677.1(6) | 30.0(5) |
| H24 | 3183.5(6) | 4457.2(16) | 2215.4(7) | 37.1(5) |
| H26 | 3744.8(7) | 2208.7(18) | 1720.6(7) | 43.3(6) |
| H27 | 3647.6(6) | 944.8(16) | 2180.3(6) | 35.3(5) |
| H28a | 3300(2) | 4325(12) | 1395(3) | 76.2(10) |
| H28b | 3577(6) | 5011(4) | 1737.4(17) | 76.2(10) |
| H28c | 3750(4) | 4084(9) | 1507(4) | 76.2(10) |
| H36a | 4534(6) | 5799(3) | 1627(3) | 84.2(12) |
| H36b | 4286(3) | 6609(9) | 1828(6) | 84.2(12) |
| H36c | 4731(4) | 6440(11) | 2024(3) | 84.2(12) |
| H52 | 6340.2(6) | 2097.0(14) | 4522.1(6) | 26.7(4) |
| H54 | 7311.5(6) | 1610.4(16) | 5369.5(6) | 31.8(5) |
| H56 | 6835.5(5) | 4606.5(15) | 5195.5(6) | 25.4(4) |
| H60 | 6833.4(5) | 5384.5(15) | 4530.3(6) | 26.5(4) |
| H62 | 6458.0(5) | 8470.8(15) | 4309.0(6) | 27.9(4) |
| H64 | 5761.6(5) | 6179.7(14) | 4603.7(5) | 24.0(4) |
| H68 | 6573.4(5) | 3228.2(14) | 4059.8(5) | 25.1(4) |
| H70 | 5893.4(6) | 3803.9(19) | 2971.3(6) | 39.3(5) |
| H72 | 5570.1(6) | 4781.6(19) | 3960.5(6) | 37.9(5) |

| | | | | |
|---|---|---|---|---|
| H76 | 5927.4(5) | 5014.6(14) | 5231.5(5) | 24.7(4) |
| H78 | 5009.8(6) | 3100.5(16) | 5235.2(7) | 35.9(5) |
| H80 | 5588.3(5) | 2788.0(16) | 4367.2(6) | 31.2(5) |
| H91a | 3264.3(8) | -817(2) | 3872.9(9) | 62.6(8) |
| H91b | 3504.2(8) | -1238(2) | 3562.9(9) | 62.6(8) |
| H91c | 3271.8(8) | -699(2) | 3742.2(9) | 62.6(8) |
| H91d | 3585.0(8) | -1467(2) | 3621.6(9) | 62.6(8) |
| H92a | 3430.2(15) | -2354(4) | 4220.3(16) | 58.7(15) |
| H92b | 3717.3(15) | -2685(4) | 3945.8(16) | 58.7(15) |
| H92c | 3243(7) | -1619(15) | 4219(4) | 92(6) |
| H92d | 3441(7) | -2537(15) | 4003(4) | 92(6) |
| H93a | 3940(2) | -1970(3) | 4691.2(13) | 56.9(16) |
| H93b | 4216(2) | -2067(3) | 4386.9(13) | 56.9(16) |
| H93c | 3751(5) | -1817(13) | 4685(5) | 91(5) |
| H93d | 3996(5) | -2282(13) | 4388(5) | 91(5) |
| H94a | 4249.5(9) | -312(2) | 4467.1(7) | 59.3(8) |
| H94b | 3840.5(9) | -245(2) | 4578.6(7) | 59.3(8) |
| H94c | 4264.5(9) | -605(2) | 4441.0(7) | 59.3(8) |
| H94d | 3910.5(9) | -68(2) | 4586.8(7) | 59.3(8) |

9.9.13 Atomic Occupancies for all atoms that are not fully occupied

Table S63. Atomic Occupancies for all atoms that are not fully occupied in **9a**.

| Atom | Occupancy |
|---|---|
| F60a | 0.797(5) |
| F60b | 0.203(5) |
| F61a | 0.797(5) |
| F61b | 0.203(5) |
| F62a | 0.797(5) |
| F62b | 0.203(5) |
| F66a | 0.517(7) |
| F66b | 0.483(7) |
| F67a | 0.517(7) |
| F67b | 0.483(7) |
| F68a | 0.517(7) |
| F68b | 0.483(7) |
| F72a | 0.812(9) |
| F72b | 0.188(9) |
| F73a | 0.812(9) |
| F73b | 0.188(9) |
| F74a | 0.812(9) |
| F74b | 0.188(9) |
| C74a | 0.517(7) |
| C74b | 0.483(7) |
| C82a | 0.812(9) |
| C82b | 0.188(9) |
| H91a | 0.722(14) |
| H91b | 0.722(14) |
| H91c | 0.278(14) |
| H91d | 0.278(14) |
| C92a | 0.722(14) |
| H92a | 0.722(14) |
| H92b | 0.722(14) |
| C92b | 0.278(14) |
| H92c | 0.278(14) |
| H92d | 0.278(14) |
| C93a | 0.722(14) |
| H93a | 0.722(14) |
| H93b | 0.722(14) |
| C93b | 0.278(14) |
| H93c | 0.278(14) |
| H93d | 0.278(14) |
| H94a | 0.722(14) |
| H94b | 0.722(14) |
| H94c | 0.278(14) |
| H94d | 0.278(14) |

## 9.10 Single crystal structure analysis of 10b

CCDC Deposition number 2423159

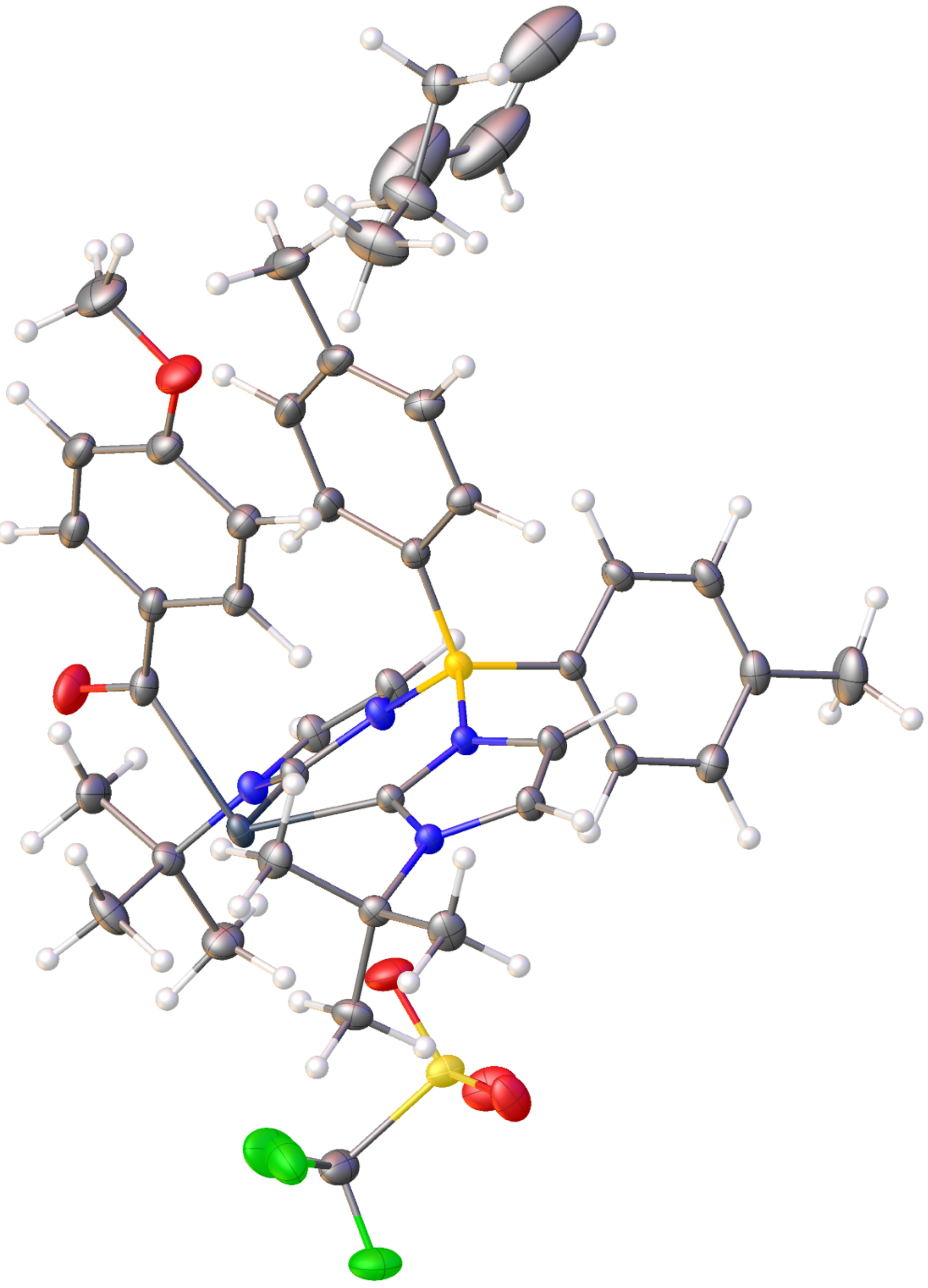

Figure S35. Olex-2 representation of the molecular structure of complex **10b**

### 9.10.1 Experimental.

Single yellow block-shaped crystals of **10b** were recrystallised from a mixture of hexane and benzene. A suitable crystal with dimensions 0.14 × 0.13 × 0.12 mm$^3$ was selected and mounted on a MITIGEN holder in oil on a Rigaku FRE+ diffractometer with Arc)Sec VHF Varimax confocal mirrors, a UG2 goniometer and HyPix 6000HE detector diffractometer. The crystal was kept at a steady $T$ = 100(2) K during data collection. The structure was solved with the ShelXT 2018/2 (Sheldrick, 2015) solution program using dual methods and by using Olex2 1.5-dev (Dolomanov et al., 2009) as the graphical interface. The model was refined with olex2.refine 1.5-dev (Bourhis et al., 2015) using full matrix least squares minimisation on $\boldsymbol{F^2}$.

9.10.2 Crystal Data.

$C_{40}H_{49}BF_3N_4O_5SSb$, $M_r$ = 887.504, triclinic, *P*-1 (No. 2), a = 10.9066(1) Å, b = 14.9551(2) Å, c = 15.1257(1) Å, $\alpha$ = 118.156(1)°, $\beta$ = 103.675(1)°, $\gamma$ = 96.569(1)°, *V* = 2038.69(5) Å$^3$, *T* = 100(2) K, *Z* = 2, *Z'* = 1, $\mu$(Mo $K_\alpha$) = 0.788, 174063 reflections measured, 33820 unique ($R_{int}$ = 0.0284) which were used in all calculations. The final $wR_2$ was 0.0534 (all data) and $R_1$ was 0.0236 (I≥2 $\sigma$(I)).

| Compound | **10b** |
|---|---|
| | |
| Formula | $C_{40}H_{49}BF_3N_4O_5$ SSb |
| $D_{calc.}$/ g cm$^{-3}$ | 1.446 |
| $\mu$/mm$^{-1}$ | 0.788 |
| Formula Weight | 887.504 |
| Colour | yellow |
| Shape | block-shaped |
| Size/mm$^3$ | 0.14×0.13×0.12 |
| *T*/K | 100(2) |
| Crystal System | triclinic |
| Space Group | *P*-1 |
| *a*/Å | 10.9066(1) |
| *b*/Å | 14.9551(2) |
| *c*/Å | 15.1257(1) |
| $\alpha$/° | 118.156(1) |
| $\beta$/° | 103.675(1) |
| $\gamma$/° | 96.569(1) |
| V/Å$^3$ | 2038.69(5) |
| *Z* | 2 |
| *Z'* | 1 |
| Wavelength/Å | 0.71073 |
| Radiation type | Mo $K_\alpha$ |
| $\Theta_{min}$/° | 1.58 |
| $\Theta_{max}$/° | 45.19 |
| Measured Refl's. | 174063 |
| Indep't Refl's | 33820 |
| Refl's I≥2 $\sigma$(I) | 30351 |
| $R_{int}$ | 0.0284 |
| Parameters | 558 |
| Restraints | 21 |
| Largest Peak | 1.2714 |
| Deepest Hole | -0.8124 |
| GooF | 1.0380 |
| $wR_2$ (all data) | 0.0534 |
| $wR_2$ | 0.0514 |
| $R_1$ (all data) | 0.0295 |
| $R_1$ | 0.0236 |

### 9.10.3 Structure Quality Indicators

| | | | | |
|---|---|---|---|---|
| Reflections: | d min (MoKα) 2Θ=90.4° 0.50 | I/σ(I) 46.9 | Rint m=5.15 2.84% | Full 50.5° 99.9 |
| Refinement: | Shift 0.001 | Max Peak 1.3 | Min Peak -0.8 | GooF 1.038 |

A yellow block-shaped crystal with dimensions 0.14 × 0.13 × 0.12 $mm^3$ was mounted on a MITIGEN holder in oil. Data were collected using a Rigaku FRE+ diffractometer with Arc)Sec VHF Varimax confocal mirrors, a UG2 goniometer and HyPix 6000HE detector diffractometer equipped with an Oxford Cryosystems low-temperature device operating at $T$ = 100(2) K.

Data were measured using profile data from $\omega$-scans with Mo $K_\alpha$ radiation. The diffraction pattern was indexed and the total number of runs and images was based on the strategy calculation from the program CrysAlisPro system (CCD 43.143a 64-bit (release 25-10-2024)). The maximum resolution that was achieved was $\Theta$ = 45.19° (0.50 Å).

The unit cell was refined using CrysAlisPro 1.171.43.143a (Rigaku OD, 2024) on 84907 reflections, 49% of the observed reflections.

Data reduction, scaling and absorption corrections were performed using CrysAlisPro 1.171.43.143a (Rigaku OD, 2024). The final completeness is 99.90 % out to 45.19° in $\Theta$. A multi-scan absorption correction was performed using CrysAlisPro 1.171.43.143a (Rigaku OD, 2024) Empirical absorption correction using spherical harmonics, implemented in SCALE3 ABSPACK scaling algorithm. The absorption coefficient $\mu$ of this material is 0.788 $mm^{-1}$ at this wavelength ($\lambda$ = 0.71073Å) and the minimum and maximum transmissions are 0.898 and 1.000.

The structure was solved and the space group $P$-1 (# 2) determined by the ShelXT 2018/2 (Sheldrick, 2018) structure solution program using dual methods and refined by full matrix least squares minimisation on $F^2$ using version of olex2.refine 1.5-dev (Bourhis et al., 2015). All non-hydrogen atoms were refined anisotropically. Hydrogen atom positions were calculated geometrically and refined using the riding model. Hydrogen atom positions were calculated geometrically and refined using the riding model.

*_refine_special_details*: Sb1 has been refined with an anharmonic component to the anisotropic refinement. The structure includes a mixed solvent site over a symmetry site with solvent occupancies initially refined then fixed to 75:25 hexane:benzene. Partially occupied solvent benzene has thermal restraints (RIGU) applied.

*_exptl_absorpt_process_details*: CrysAlisPro 1.171.43.143a (Rigaku OD, 2024) using spherical harmonics, implemented in SCALE3 ABSPACK scaling algorithm.

There is a single formula unit in the asymmetric unit, which is represented by the reported sum formula. In other words: Z is 2 and Z' is 1. The moiety formula is C36 H43 B N4 O2 Sb, C F3 O3 S, 0.75(C3 H7), 0.25(C3 H3).

*_olex2_refine_details*: Refinement using NoSpherA2, an implementation of NOn-SPHERical Atom-form-factors in Olex2.Please cite: F. Kleemiss et al. Chem. Sci. DOI 10.1039/D0SC05526C – 2021 NoSpherA2 implementation of HAR makes use of tailor-made aspherical atomic form factors calculated on-the-fly from a Hirshfeld-partitioned electron density (ED) - not from spherical-atom form factors. The ED is calculated from a gaussian basis set single determinant SCF wavefunction - either Hartree-Fock or DFT using selected functionals- for a fragment of the crystal. This fragment can be embedded in an electrostatic crystal field by employing cluster charges or modelled using implicit solvation models, depending on the software used. The following options were used: SOFTWARE: ORCA 6.0.1 ::: PARTITIONING: NoSpherA2 ::: INT ACCURACY: Normal ::: METHOD: PBE ::: BASIS SET: x2c-TZVP ::: CHARGE: 0 ::: MULTIPLICITY: 1 ::: RELATIVISTIC: DKH2 ::: DATE: 2024-11-10_04-07-09

## 9.10.4 Data Plots: Diffraction Data

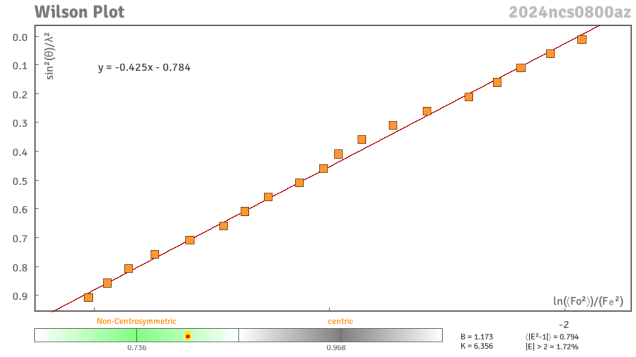


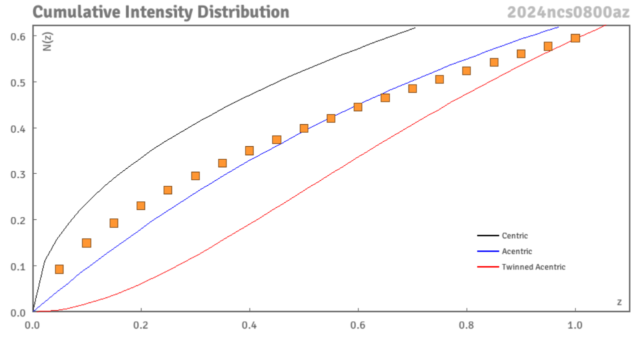


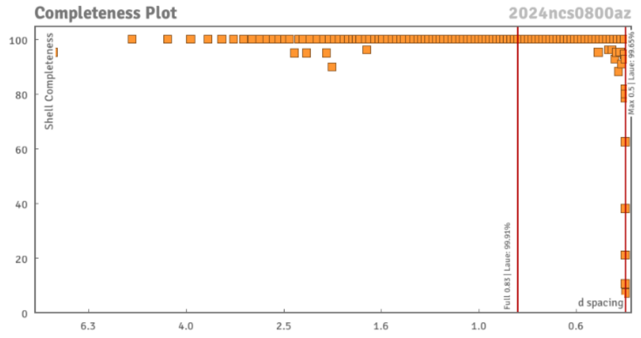


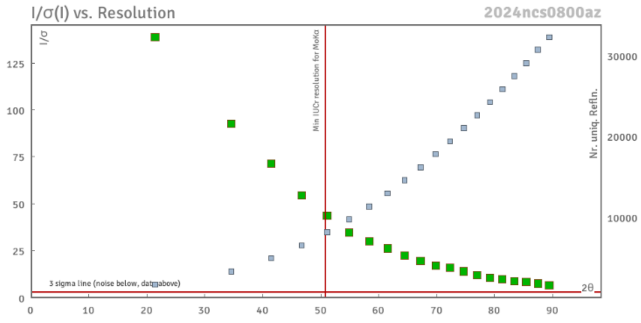


## 9.10.5 Data Plots: Refinement and Data

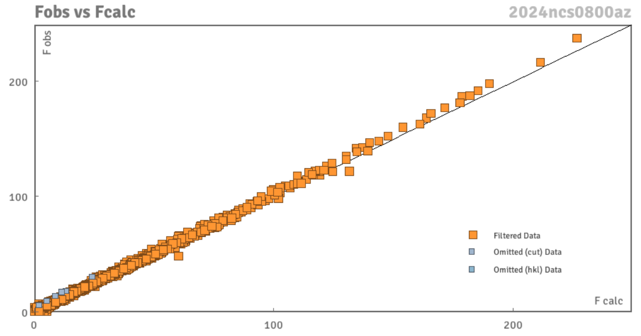


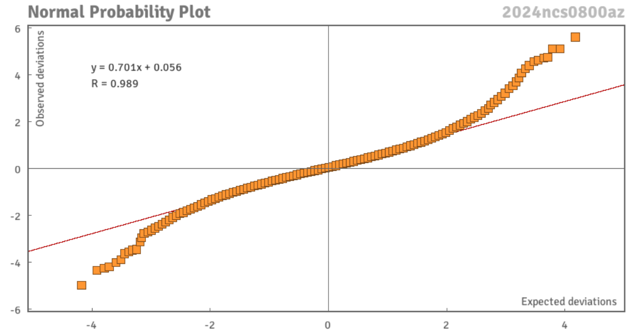

### 9.10.6 Reflection Statistics

| | | | |
|---|---|---|---|
| Total reflections (after filtering) | 174010 | Unique reflections | 33820 |
| Completeness | 0.996 | Mean $I/\sigma$ | 32.24 |
| $hkl_{max}$ collected | (21, 29, 30) | $hkl_{min}$ collected | (-21, -29, -29) |
| $hkl_{max}$ used | (20, 26, 30) | $hkl_{min}$ used | (-21, -29, 0) |
| Lim $d_{max}$ collected | 100.0 | Lim $d_{min}$ collected | 0.36 |
| $d_{max}$ used | 12.87 | $d_{min}$ used | 0.5 |
| Friedel pairs | 22819 | Friedel pairs merged | 1 |
| Inconsistent equivalents | 30 | $R_{int}$ | 0.0284 |
| $R_{sigma}$ | 0.0213 | Intensity transformed | 0 |
| Omitted reflections | 0 | Omitted by user (OMIT hkl) | 53 |
| Multiplicity | (14127, 15649, 9806, 5565, 3819, 2660, 1982, 1324, 727, 421, 271, 182, 76, 27, 7, 1, 2) | Maximum multiplicity | 18 |
| Removed systematic absences | 0 | Filtered off (Shel/OMIT) | 0 |

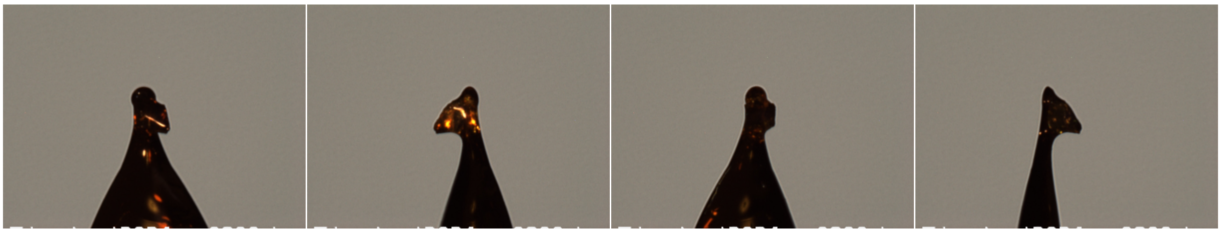

9.10.7 Fractional Atomic Coordinates and Equivalent Isotropic Displacement Parameters

Table S64. Fractional Atomic Coordinates (×$10^4$) and Equivalent Isotropic Displacement Parameters (Å$^2$×$10^3$) for **10b**. $U_{eq}$ is defined as 1/3 of the trace of the orthogonalised $U_{ij}$.

| Atom | x | y | z | $U_{eq}$ |
|---|---|---|---|---|
| Sb1 | 9854.14(8) | 3885.33(5) | 6040.43(6) | 15.89(3) |
| O1 | 8816.8(7) | 5457.2(4) | 5787.5(5) | 35.50(13) |
| O2 | 4842.3(6) | 2370.4(5) | 1104.3(5) | 34.39(12) |
| N1 | 7816.8(5) | 3612.8(3) | 7068.4(3) | 15.57(7) |
| N2 | 9160.4(5) | 5187.4(3) | 8155.8(3) | 16.26(7) |
| N3 | 7898.6(4) | 1871.4(3) | 5502.2(3) | 13.84(6) |
| N4 | 9289.0(4) | 1413.7(3) | 4636.3(3) | 14.10(6) |
| C1 | 8876.6(5) | 4282.9(4) | 7206.0(4) | 14.67(7) |
| C2 | 7409.1(6) | 4107.7(4) | 7937.7(4) | 19.80(9) |
| C3 | 8233.9(6) | 5081.6(4) | 8610.9(4) | 19.66(9) |
| C4 | 10317.8(6) | 6133.2(4) | 8798.4(4) | 18.58(8) |
| C5 | 11238.6(7) | 6133.5(5) | 8188.0(5) | 28.92(13) |
| C6 | 11049.5(8) | 6095.8(6) | 9776.1(5) | 27.95(12) |
| C7 | 9806.9(8) | 7112.5(5) | 9129.6(6) | 27.35(12) |
| C8 | 8912.7(5) | 2241.9(4) | 5301.4(4) | 13.35(7) |
| C9 | 7617.3(6) | 791.2(4) | 4948.7(4) | 17.22(8) |
| C10 | 8471.9(6) | 500.9(4) | 4404.9(4) | 17.18(8) |
| C11 | 10404.4(5) | 1449.6(4) | 4217.6(4) | 16.26(8) |
| C12 | 10405.4(7) | 325.0(5) | 3444.5(5) | 23.85(11) |
| C13 | 11697.5(6) | 1980.1(5) | 5142.5(5) | 21.96(10) |
| C14 | 10203.2(7) | 2032.6(5) | 3617.9(5) | 22.39(10) |
| C15 | 5832.6(5) | 2574.8(4) | 5294.0(4) | 15.81(7) |
| C16 | 5573.4(6) | 3538.5(4) | 5501.3(5) | 21.13(9) |
| C17 | 4623.0(7) | 3617.5(5) | 4763.5(6) | 24.53(11) |
| C18 | 3893.3(6) | 2737.8(6) | 3775.4(5) | 22.92(10) |
| C19 | 4097.5(6) | 1764.8(5) | 3573.7(5) | 22.4(1) |
| C20 | 5039.8(6) | 1686.5(4) | 4318.3(4) | 19.11(8) |
| C21 | 2956.1(8) | 2854.9(8) | 2954.1(7) | 33.54(16) |
| C22 | 6496.9(5) | 1861.4(4) | 6662.5(4) | 14.95(7) |
| C23 | 7411.2(6) | 1616.3(4) | 7283.2(4) | 17.99(8) |
| C24 | 7061.6(7) | 1225.4(5) | 7886.6(5) | 21.69(10) |
| C25 | 5782.7(7) | 1078.2(5) | 7909.2(5) | 22.72(10) |
| C26 | 4863.6(6) | 1306.1(5) | 7287.1(5) | 22.37(10) |
| C27 | 5216.0(6) | 1681.8(4) | 6670.1(5) | 18.92(8) |
| C28 | 5406.9(11) | 703.9(7) | 8598.2(7) | 37.63(19) |
| C29 | 8526.7(7) | 4503.0(4) | 5212.5(5) | 21.00(9) |
| C30 | 7566.6(6) | 3945.8(4) | 4135.2(5) | 18.76(8) |
| C31 | 6984.5(7) | 4527.8(5) | 3735.9(5) | 22.59(10) |
| C32 | 6069.4(7) | 4036.4(6) | 2724.6(6) | 24.78(11) |
| C33 | 5723.6(7) | 2935.1(6) | 2094.5(5) | 23.58(10) |
| C34 | 6293.0(7) | 2348.1(5) | 2487.9(5) | 22.46(10) |
| C35 | 7200.8(6) | 2849.1(4) | 3499.1(4) | 19.08(8) |
| C36 | 4214.9(11) | 2913.8(9) | 653.8(8) | 44.8(2) |
| B1 | 6980.5(6) | 2471.3(4) | 6112.7(4) | 14.16(8) |
| S41 | 11843.02(15) | 2920.81(11) | 8122.91(10) | 19.40(3) |
| F41 | 13596.7(6) | 3704.8(5) | 7556.6(5) | 42.60(13) |
| F42 | 14416.0(5) | 3557.1(4) | 8899.8(4) | 36.93(11) |
| F43 | 13478.1(7) | 4788.4(4) | 9078.6(6) | 54.34(18) |
| O41 | 10921.5(6) | 3288.7(5) | 7612.0(5) | 34.79(12) |
| O42 | 11952.6(6) | 1890.8(4) | 7441.0(4) | 29.59(11) |

| | | | | |
|---|---|---|---|---|
| O43 | 11817.5(7) | 3141.0(5) | 9148.6(4) | 35.27(13) |
| C41 | 13419.9(7) | 3788.6(5) | 8433.0(5) | 27.55(12) |
| C51a | 2557.0(13) | -108.7(13) | -686.7(10) | 46.1(3) |
| C52a | 1835.3(12) | 205.7(11) | 103.0(9) | 36.6(2) |
| C53a | 365.3(10) | -105.1(7) | -392.4(7) | 24.87(15) |
| C51b | 1344(10) | 388(6) | 348(5) | 106(3) |
| C52b | 764(7) | 165(5) | 966(4) | 94(2) |
| C53b | -625(7) | -271(5) | 589(4) | 94(2) |

9.10.8 Anisotropic Displacement Parameters

Table S65. Anisotropic Displacement Parameters (×$10^4$) for **10b**. The anisotropic displacement factor exponent takes the form: $-2\pi^2[h^2a^{*2} \times U_{11} + ... +2hka^* \times b^* \times U_{12}]$

| Atom | $U_{11}$ | $U_{22}$ | $U_{33}$ | $U_{23}$ | $U_{13}$ | $U_{12}$ |
|---|---|---|---|---|---|---|
| Sb1 | 16.68(4) | 10.89(4) | 17.73(4) | 2.20(3) | 9.13(3) | 4.38(3) |
| O1 | 50.9(4) | 13.88(18) | 32.0(2) | 8.5(2) | 5.4(2) | 8.49(17) |
| O2 | 34.7(3) | 40.4(3) | 29.4(2) | 12.3(2) | 3.9(2) | 21.6(2) |
| N1 | 16.93(18) | 11.27(14) | 15.27(15) | 2.75(12) | 8.08(13) | 3.38(12) |
| N2 | 18.11(18) | 11.65(14) | 14.14(14) | 2.39(12) | 5.95(13) | 3.06(11) |
| N3 | 14.29(16) | 10.56(13) | 14.98(14) | 3.60(11) | 6.96(12) | 4.31(11) |
| N4 | 15.60(17) | 12.26(14) | 13.98(14) | 5.02(12) | 7.18(12) | 5.12(11) |
| C1 | 15.45(19) | 11.02(15) | 14.22(16) | 2.63(13) | 6.19(13) | 3.66(12) |
| C2 | 22.5(2) | 14.29(18) | 18.71(19) | 2.67(16) | 12.24(17) | 3.59(15) |
| C3 | 23.8(2) | 13.99(18) | 16.32(18) | 3.42(16) | 10.32(17) | 2.86(14) |
| C4 | 19.6(2) | 12.98(17) | 15.89(17) | 2.08(15) | 4.32(15) | 3.21(14) |
| C5 | 27.0(3) | 21.2(2) | 23.9(2) | -5.3(2) | 9.8(2) | 2.52(19) |
| C6 | 27.2(3) | 24.8(3) | 21.5(2) | 4.4(2) | 0.2(2) | 8.3(2) |
| C7 | 29.1(3) | 13.4(2) | 31.5(3) | 4.69(19) | 6.2(2) | 7.52(19) |
| C8 | 14.02(18) | 11.01(15) | 13.96(15) | 3.76(13) | 6.42(13) | 4.77(12) |
| C9 | 18.7(2) | 10.98(16) | 20.23(19) | 3.74(14) | 10.19(16) | 5.12(14) |
| C10 | 20.0(2) | 11.23(16) | 19.08(18) | 5.18(14) | 10.36(16) | 4.89(14) |
| C11 | 17.4(2) | 16.79(18) | 15.19(17) | 6.56(15) | 8.74(15) | 6.73(14) |
| C12 | 27.4(3) | 19.8(2) | 24.7(2) | 10.4(2) | 16.6(2) | 7.29(18) |
| C13 | 17.0(2) | 26.7(3) | 20.3(2) | 7.27(19) | 8.19(17) | 9.51(19) |
| C14 | 27.2(3) | 24.8(2) | 21.4(2) | 8.8(2) | 13.2(2) | 13.74(19) |
| C15 | 16.9(2) | 13.32(17) | 17.88(18) | 5.84(14) | 7.72(15) | 7.32(14) |
| C16 | 21.1(2) | 14.77(19) | 29.0(2) | 7.40(17) | 9.98(19) | 11.01(18) |
| C17 | 22.4(3) | 22.8(2) | 38.7(3) | 11.1(2) | 13.6(2) | 20.9(2) |
| C18 | 19.5(2) | 32.3(3) | 30.2(3) | 12.6(2) | 13.1(2) | 22.6(2) |
| C19 | 20.7(2) | 27.5(3) | 20.0(2) | 10.1(2) | 6.95(18) | 12.04(19) |
| C20 | 20.1(2) | 16.92(19) | 17.40(19) | 7.19(16) | 5.11(16) | 6.69(15) |
| C21 | 24.8(3) | 56.7(5) | 43.4(4) | 19.9(3) | 16.9(3) | 39.7(4) |
| C22 | 14.15(18) | 12.96(16) | 16.69(17) | 3.32(13) | 6.98(14) | 6.09(13) |
| C23 | 17.3(2) | 18.27(19) | 19.45(19) | 5.23(16) | 7.54(16) | 9.82(16) |
| C24 | 27.3(3) | 20.2(2) | 21.3(2) | 8.10(19) | 10.80(19) | 11.90(18) |
| C25 | 31.4(3) | 16.8(2) | 23.5(2) | 6.17(19) | 16.6(2) | 9.77(17) |
| C26 | 21.5(2) | 19.1(2) | 27.0(2) | 3.44(18) | 14.4(2) | 9.82(18) |
| C27 | 15.1(2) | 18.6(2) | 22.6(2) | 3.90(15) | 9.05(16) | 9.16(16) |
| C28 | 57.2(6) | 32.8(4) | 41.1(4) | 15.5(4) | 33.6(4) | 24.2(3) |
| C29 | 28.5(3) | 12.96(18) | 21.5(2) | 5.57(17) | 9.39(19) | 8.32(16) |
| C30 | 22.2(2) | 16.77(19) | 22.8(2) | 7.62(17) | 11.26(18) | 12.13(17) |
| C31 | 25.8(3) | 21.2(2) | 30.7(3) | 10.3(2) | 14.1(2) | 17.8(2) |
| C32 | 25.5(3) | 28.9(3) | 33.4(3) | 12.4(2) | 13.6(2) | 23.3(2) |
| C33 | 23.5(3) | 28.8(3) | 25.9(2) | 10.2(2) | 9.3(2) | 18.5(2) |
| C34 | 24.4(3) | 21.5(2) | 22.4(2) | 7.62(19) | 6.25(19) | 12.29(19) |
| C35 | 22.0(2) | 17.02(19) | 20.4(2) | 7.13(17) | 7.50(17) | 10.72(16) |
| C36 | 45.6(5) | 56.0(6) | 40.3(4) | 18.1(4) | 4.2(4) | 34.0(4) |
| B1 | 14.3(2) | 11.33(17) | 15.22(18) | 3.52(15) | 6.65(15) | 4.84(15) |
| S41 | 21.65(6) | 17.10(5) | 13.58(5) | 8.45(4) | 4.44(4) | 3.46(4) |
| F41 | 35.3(3) | 47.2(3) | 41.1(3) | -5.1(2) | 4.1(2) | 26.9(2) |
| F42 | 24.6(2) | 41.6(3) | 32.5(2) | 9.27(19) | -0.41(17) | 14.5(2) |
| F43 | 42.2(3) | 18.4(2) | 62.9(4) | 2.9(2) | 2.3(3) | -0.6(2) |
| O41 | 26.9(3) | 41.4(3) | 34.8(3) | 14.1(2) | 2.4(2) | 21.2(2) |

| | | | | | | |
|---|---|---|---|---|---|---|
| O42 | 33.3(3) | 15.64(17) | 30.5(2) | 5.61(17) | 17.1(2) | 2.18(15) |
| O43 | 41.4(3) | 45.6(3) | 18.68(19) | 20.8(3) | 14.4(2) | 12.5(2) |
| C41 | 24.7(3) | 18.3(2) | 25.3(3) | 3.0(2) | -1.3(2) | 5.38(19) |
| C51a | 31.6(6) | 68.3(9) | 29.8(5) | 7.3(5) | 15.1(4) | 17.9(5) |
| C52a | 29.5(5) | 47.0(6) | 22.9(4) | 3.3(4) | 10.5(3) | 11.0(4) |
| C53a | 28.9(4) | 23.5(3) | 22.3(3) | 6.3(3) | 10.9(3) | 11.0(3) |
| C51b | 187(8) | 95(5) | 46(3) | 80(5) | 41(3) | 32(3) |
| C52b | 145(6) | 91(4) | 44(2) | 68(4) | 13(3) | 34(2) |
| C53b | 158(6) | 81(4) | 32(2) | 65(4) | 16(3) | 22(2) |

9.10.9 Bond lengths

Table S66. Bond Lengths in Å for **10b**.

| Atom | Atom | Length/Å |
|---|---|---|
| Sb1 | C1 | 2.1548(5) |
| Sb1 | C8 | 2.1521(5) |
| Sb1 | C29 | 2.2490(6) |
| O1 | C29 | 1.2200(7) |
| O2 | C33 | 1.3492(9) |
| O2 | C36 | 1.4220(10) |
| N1 | C1 | 1.3446(7) |
| N1 | C2 | 1.3812(6) |
| N1 | B1 | 1.5897(7) |
| N2 | C1 | 1.3599(6) |
| N2 | C3 | 1.3839(7) |
| N2 | C4 | 1.5010(7) |
| N3 | C8 | 1.3417(6) |
| N3 | C9 | 1.3761(6) |
| N3 | B1 | 1.5878(7) |
| N4 | C8 | 1.3602(6) |
| N4 | C10 | 1.3798(7) |
| N4 | C11 | 1.5050(7) |
| C2 | C3 | 1.3545(8) |
| C4 | C5 | 1.5165(9) |
| C4 | C6 | 1.5353(9) |
| C4 | C7 | 1.5272(9) |
| C9 | C10 | 1.3608(7) |
| C11 | C12 | 1.5301(8) |
| C11 | C13 | 1.5295(8) |
| C11 | C14 | 1.5255(8) |
| C15 | C16 | 1.4014(7) |
| C15 | C20 | 1.4050(8) |
| C15 | B1 | 1.6178(8) |
| C16 | C17 | 1.3926(9) |
| C17 | C18 | 1.3930(10) |
| C18 | C19 | 1.3955(9) |
| C18 | C21 | 1.5049(9) |
| C19 | C20 | 1.3940(8) |
| C22 | C23 | 1.4057(8) |
| C22 | C27 | 1.3959(7) |
| C22 | B1 | 1.6171(8) |
| C23 | C24 | 1.3918(8) |
| C24 | C25 | 1.3981(10) |
| C25 | C26 | 1.3934(10) |
| C25 | C28 | 1.5058(9) |
| C26 | C27 | 1.3962(8) |
| C29 | C30 | 1.4692(9) |
| C30 | C31 | 1.4024(8) |
| C30 | C35 | 1.3980(8) |
| C31 | C32 | 1.3876(10) |
| C32 | C33 | 1.4034(10) |
| C33 | C34 | 1.3976(9) |
| C34 | C35 | 1.3834(9) |
| S41 | O41 | 1.4390(6) |
| S41 | O42 | 1.4325(5) |
| S41 | O43 | 1.4340(5) |
| S41 | C41 | 1.8427(8) |
| F41 | C41 | 1.3341(10) |
| F42 | C41 | 1.3239(9) |
| F43 | C41 | 1.3282(8) |
| C51a | C52a | 1.5055(16) |
| C52a | C53a | 1.5102(16) |
| C53a | C53a[1] | 1.5152(16) |
| C51b | C52b | 1.382(9) |
| C51b | C53b[1] | 1.366(7) |
| C52b | C53b | 1.436(9) |

----
[1]-x,-y,-z

9.10.10 Bond Angles

Table S67. Bond Angles in ° for **10b**.

| Atom | Atom | Atom | Angle/° |
|---|---|---|---|
| C8 | Sb1 | C1 | 90.774(18) |
| C29 | Sb1 | C1 | 90.39(2) |
| C29 | Sb1 | C8 | 106.00(2) |
| C36 | O2 | C33 | 118.69(7) |
| C2 | N1 | C1 | 108.11(4) |
| B1 | N1 | C1 | 132.51(4) |
| B1 | N1 | C2 | 119.24(4) |
| C3 | N2 | C1 | 108.10(4) |
| C4 | N2 | C1 | 132.38(5) |
| C4 | N2 | C3 | 118.91(4) |
| C9 | N3 | C8 | 108.05(4) |
| B1 | N3 | C8 | 129.92(4) |
| B1 | N3 | C9 | 121.50(4) |
| C10 | N4 | C8 | 108.14(4) |
| C11 | N4 | C8 | 127.49(4) |
| C11 | N4 | C10 | 124.36(4) |
| N1 | C1 | Sb1 | 122.28(3) |
| N2 | C1 | Sb1 | 129.29(4) |
| N2 | C1 | N1 | 108.43(4) |
| C3 | C2 | N1 | 108.15(5) |
| C2 | C3 | N2 | 107.19(4) |
| C5 | C4 | N2 | 112.45(4) |
| C6 | C4 | N2 | 106.60(5) |
| C6 | C4 | C5 | 109.03(6) |
| C7 | C4 | N2 | 107.94(5) |
| C7 | C4 | C5 | 110.01(6) |
| C7 | C4 | C6 | 110.77(5) |
| N3 | C8 | Sb1 | 124.21(3) |
| N4 | C8 | Sb1 | 126.99(4) |
| N4 | C8 | N3 | 108.60(4) |
| C10 | C9 | N3 | 108.28(5) |
| C9 | C10 | N4 | 106.92(4) |
| C12 | C11 | N4 | 108.99(5) |
| C13 | C11 | N4 | 109.38(4) |
| C13 | C11 | C12 | 108.95(5) |
| C14 | C11 | N4 | 108.95(4) |
| C14 | C11 | C12 | 108.67(5) |
| C14 | C11 | C13 | 111.85(5) |
| C20 | C15 | C16 | 116.15(5) |
| B1 | C15 | C16 | 122.84(5) |
| B1 | C15 | C20 | 121.01(5) |
| C17 | C16 | C15 | 121.92(6) |
| C18 | C17 | C16 | 121.26(6) |
| C19 | C18 | C17 | 117.57(6) |
| C21 | C18 | C17 | 120.35(7) |
| C21 | C18 | C19 | 122.04(7) |
| C20 | C19 | C18 | 120.97(6) |
| C19 | C20 | C15 | 121.97(5) |
| C27 | C22 | C23 | 116.76(5) |
| B1 | C22 | C23 | 120.10(5) |
| B1 | C22 | C27 | 122.59(5) |
| C24 | C23 | C22 | 121.70(5) |
| C25 | C24 | C23 | 120.83(6) |
| C26 | C25 | C24 | 118.00(5) |
| C28 | C25 | C24 | 121.35(7) |
| C28 | C25 | C26 | 120.64(7) |
| C27 | C26 | C25 | 120.86(6) |
| C26 | C27 | C22 | 121.82(6) |
| O1 | C29 | Sb1 | 107.60(5) |
| C30 | C29 | Sb1 | 129.68(4) |
| C30 | C29 | O1 | 122.39(6) |
| C31 | C30 | C29 | 119.20(5) |
| C35 | C30 | C29 | 121.93(5) |
| C35 | C30 | C31 | 118.86(6) |
| C32 | C31 | C30 | 121.28(6) |
| C33 | C32 | C31 | 119.01(6) |
| C32 | C33 | O2 | 124.38(6) |
| C34 | C33 | O2 | 115.49(6) |
| C34 | C33 | C32 | 120.12(6) |
| C35 | C34 | C33 | 120.20(6) |
| C34 | C35 | C30 | 120.52(5) |
| N3 | B1 | N1 | 109.49(4) |
| C15 | B1 | N1 | 109.36(4) |
| C15 | B1 | N3 | 108.91(4) |
| C22 | B1 | N1 | 105.45(4) |
| C22 | B1 | N3 | 107.79(4) |
| C22 | B1 | C15 | 115.69(4) |
| O42 | S41 | O41 | 115.04(4) |
| O43 | S41 | O41 | 114.92(4) |
| O43 | S41 | O42 | 115.45(4) |
| C41 | S41 | O41 | 102.17(4) |
| C41 | S41 | O42 | 103.44(3) |
| C41 | S41 | O43 | 103.21(4) |
| F41 | C41 | S41 | 111.48(5) |
| F42 | C41 | S41 | 111.49(5) |
| F42 | C41 | F41 | 107.33(7) |
| F43 | C41 | S41 | 110.85(6) |
| F43 | C41 | F41 | 107.51(7) |
| F43 | C41 | F42 | 107.98(6) |
| C53a | C52a | C51a | 114.26(9) |
| C53a[1] | C53a | C52a | 114.18(10) |
| C53b[1] | C51b | C52b | 121.9(9) |
| C53b | C52b | C51b | 119.9(6) |
| C52b | C53b | C51b[1] | 118.1(7) |

----

[1]-x,-y,-z

9.10.11 Torsion Angles

Table S68. Torsion Angles in ° for **10b**.

| Atom | Atom | Atom | Atom | Angle/° |
|---|---|---|---|---|
| Sb1 | C1 | N1 | C2 | -178.60(5) |
| Sb1 | C1 | N1 | B1 | -2.96(6) |
| Sb1 | C1 | N2 | C3 | 178.43(5) |
| Sb1 | C1 | N2 | C4 | -10.87(7) |
| Sb1 | C8 | N3 | C9 | -175.72(4) |
| Sb1 | C8 | N3 | B1 | 12.67(6) |
| Sb1 | C8 | N4 | C10 | 175.84(4) |
| Sb1 | C8 | N4 | C11 | -3.39(6) |
| Sb1 | C29 | C30 | C31 | -170.73(5) |
| Sb1 | C29 | C30 | C35 | 10.16(6) |
| O1 | C29 | C30 | C31 | 1.82(8) |
| O1 | C29 | C30 | C35 | -177.30(7) |
| O2 | C33 | C32 | C31 | 179.57(7) |
| O2 | C33 | C34 | C35 | -179.42(6) |
| N1 | C1 | N2 | C3 | -1.15(5) |
| N1 | C1 | N2 | C4 | 169.55(4) |
| N1 | C2 | C3 | N2 | -0.21(6) |
| N1 | B1 | N3 | C8 | -35.66(6) |
| N1 | B1 | N3 | C9 | 153.70(4) |
| N1 | B1 | C15 | C16 | -6.56(5) |
| N1 | B1 | C15 | C20 | 172.89(4) |
| N1 | B1 | C22 | C23 | -66.82(5) |
| N1 | B1 | C22 | C27 | 104.40(5) |
| N3 | C8 | N4 | C10 | 0.88(5) |
| N3 | C8 | N4 | C11 | -178.36(4) |
| N3 | C9 | C10 | N4 | 0.48(5) |
| N3 | B1 | C15 | C16 | -126.17(4) |
| N3 | B1 | C15 | C20 | 53.27(5) |
| N3 | B1 | C22 | C23 | 50.07(5) |
| N3 | B1 | C22 | C27 | -138.71(4) |
| C15 | C16 | C17 | C18 | -0.73(7) |
| C15 | C20 | C19 | C18 | -0.69(7) |
| C15 | B1 | C22 | C23 | 172.21(4) |
| C15 | B1 | C22 | C27 | -16.57(5) |
| C16 | C17 | C18 | C19 | 3.48(7) |
| C16 | C17 | C18 | C21 | -174.60(6) |
| C17 | C18 | C19 | C20 | -2.78(7) |
| C22 | C23 | C24 | C25 | -1.05(6) |
| C22 | C27 | C26 | C25 | -1.11(7) |
| C23 | C24 | C25 | C26 | 1.78(7) |
| C23 | C24 | C25 | C28 | -177.11(6) |
| C24 | C25 | C26 | C27 | -0.73(7) |
| C29 | C30 | C31 | C32 | -179.78(6) |
| C29 | C30 | C35 | C34 | 179.96(6) |
| C30 | C31 | C32 | C33 | 0.08(7) |
| C30 | C35 | C34 | C33 | -0.50(7) |
| C31 | C32 | C33 | C34 | 0.28(8) |
| C32 | C33 | C34 | C35 | -0.07(7) |
| C51a | C52a | C53a | C53a$^1$ | 173.70(13) |
| C52a | C53a | C53a$^1$ | C52a$^1$ | -180.00(15) |
| C51b | C52b | C53b | C51b$^1$ | -4.2(6) |
| C51b$^1$ | C53b | C52b | C51b | -4.2(6) |

### 9.10.12 Hydrogen Fractional Atomic Coordinates and Equivalent Isotropic Displacement Parameters

Table S69. Hydrogen Fractional Atomic Coordinates (×10[4]) and Equivalent Isotropic Displacement Parameters (Å[2]×10[3]) for **10b**. $U_{eq}$ is defined as 1/3 of the trace of the orthogonalised $U_{ij}$.

| Atom | x | y | z | $U_{eq}$ |
|---|---|---|---|---|
| H2 | 6574.6(6) | 3774.9(4) | 8059.8(4) | 23.76(11) |
| H3 | 8178.6(6) | 5667.8(4) | 9364.4(4) | 23.59(11) |
| H5a | 11626(5) | 5458(3) | 7987(5) | 43.4(2) |
| H5b | 10713.5(18) | 6103(6) | 7471(3) | 43.4(2) |
| H5c | 12030(4) | 6840(3) | 8672(2) | 43.4(2) |
| H6a | 10440(3) | 6187(6) | 10263(3) | 41.92(18) |
| H6b | 11283(6) | 5351(2) | 9522.2(5) | 41.92(18) |
| H6c | 11939(4) | 6722(3) | 10229(3) | 41.92(18) |
| H7a | 9234(6) | 7092(3) | 8431.1(6) | 41.03(18) |
| H7b | 9206(5) | 7132(3) | 9607(4) | 41.03(18) |
| H7c | 10620.1(8) | 7804.1(5) | 9584(5) | 41.03(18) |
| H9 | 6847.8(6) | 262.8(4) | 4946.2(4) | 20.66(10) |
| H10 | 8504.4(6) | -294.9(4) | 3889.0(4) | 20.62(9) |
| H12a | 10529(6) | -100.5(16) | 3854.4(14) | 35.77(16) |
| H12b | 9490(3) | -60.0(17) | 2798(3) | 35.77(16) |
| H12c | 11197(4) | 343.7(5) | 3137(4) | 35.77(16) |
| H13a | 11723(3) | 2783.4(15) | 5673(3) | 32.94(14) |
| H13b | 11780(3) | 1567(3) | 5566(3) | 32.94(14) |
| H13c | 12499.6(6) | 1964(4) | 4839.5(6) | 32.94(14) |
| H14a | 9256(3) | 1666(3) | 3009(3) | 33.59(15) |
| H14b | 10260(6) | 2839.5(13) | 4163.3(10) | 33.59(15) |
| H14c | 10951(4) | 2004(4) | 3258(4) | 33.59(15) |
| H16 | 6128.4(6) | 4242.3(4) | 6257.3(5) | 25.36(11) |
| H17 | 4447.9(7) | 4377.4(5) | 4961.7(6) | 29.44(13) |
| H19 | 3515.3(6) | 1060.2(5) | 2827.7(5) | 26.88(12) |
| H20 | 5164.7(6) | 917.8(4) | 4139.3(4) | 22.93(10) |
| H21a | 2408(5) | 3391(5) | 3327.0(13) | 50.3(2) |
| H21b | 3495.7(9) | 3158(6) | 2598(4) | 50.3(2) |
| H21c | 2294(5) | 2099.9(13) | 2349(3) | 50.3(2) |
| H23 | 8413.4(6) | 1734.6(4) | 7292.1(4) | 21.58(10) |
| H24 | 7789.5(7) | 1033.0(5) | 8344.4(5) | 26.03(11) |
| H26 | 3862.8(6) | 1190.4(5) | 7282.0(5) | 26.84(12) |
| H27 | 4475.1(6) | 1838.1(4) | 6183.9(5) | 22.7(1) |
| H28a | 5766(7) | 29(4) | 8485(5) | 56.4(3) |
| H28b | 5828(7) | 1324(2) | 9419.8(9) | 56.4(3) |
| H28c | 4357.5(11) | 491(6) | 8383(5) | 56.4(3) |
| H31 | 7254.5(7) | 5377.4(5) | 4226.3(5) | 27.11(12) |
| H32 | 5628.6(7) | 4495.8(6) | 2426.0(6) | 29.74(13) |
| H34 | 6022.8(7) | 1498.3(5) | 1999.5(5) | 26.95(12) |
| H35 | 7631.5(6) | 2388.2(4) | 3800.4(4) | 22.9(1) |
| H36a | 3538(6) | 2353.8(10) | -130(3) | 67.3(3) |
| H36b | 3693(7) | 3374(6) | 1147(4) | 67.3(3) |
| H36c | 4938.6(14) | 3425(5) | 603(7) | 67.3(3) |
| H51a | 2304(9) | 232(8) | -1171(7) | 69.2(5) |
| H51c | 3595.0(13) | 170(9) | -268.7(10) | 69.2(5) |
| H51d | 2288(9) | -952.5(14) | -1185(6) | 69.2(5) |
| H52a | 2089.3(12) | -153.4(11) | 579.9(9) | 43.9(3) |
| H52c | 2167.8(12) | 1056.9(11) | 638.2(9) | 43.9(3) |

| | | | | |
|---|---|---|---|---|
| H53a | 43.6(10) | -941.9(7) | -992.9(7) | 29.85(17) |
| H53c | 104.8(10) | 323.3(7) | -795.2(7) | 29.85(17) |
| H51b | 2400(10) | 664(6) | 616(5) | 128(3) |
| H52b | 1356(7) | 319(5) | 1731(4) | 113(3) |
| H53b | -1090(7) | -500(5) | 1037(4) | 112(3) |

9.10.13 Atomic Occupancies for all atoms that are not fully occupied

Table S70. Atomic Occupancies for all atoms that are not fully occupied in **10b**.

| Atom | Occupancy |
|---|---|
| C51a | 0.750000 |
| H51a | 0.750000 |
| H51c | 0.750000 |
| H51d | 0.750000 |
| C52a | 0.750000 |
| H52a | 0.750000 |
| H52c | 0.750000 |
| C53a | 0.750000 |
| H53a | 0.750000 |
| H53c | 0.750000 |
| C51b | 0.250000 |
| H51b | 0.250000 |
| C52b | 0.250000 |
| H52b | 0.250000 |
| C53b | 0.250000 |
| H53b | 0.250000 |

## 9.11 Single crystal structure analysis of 11a

CCDC Deposition number 2423158

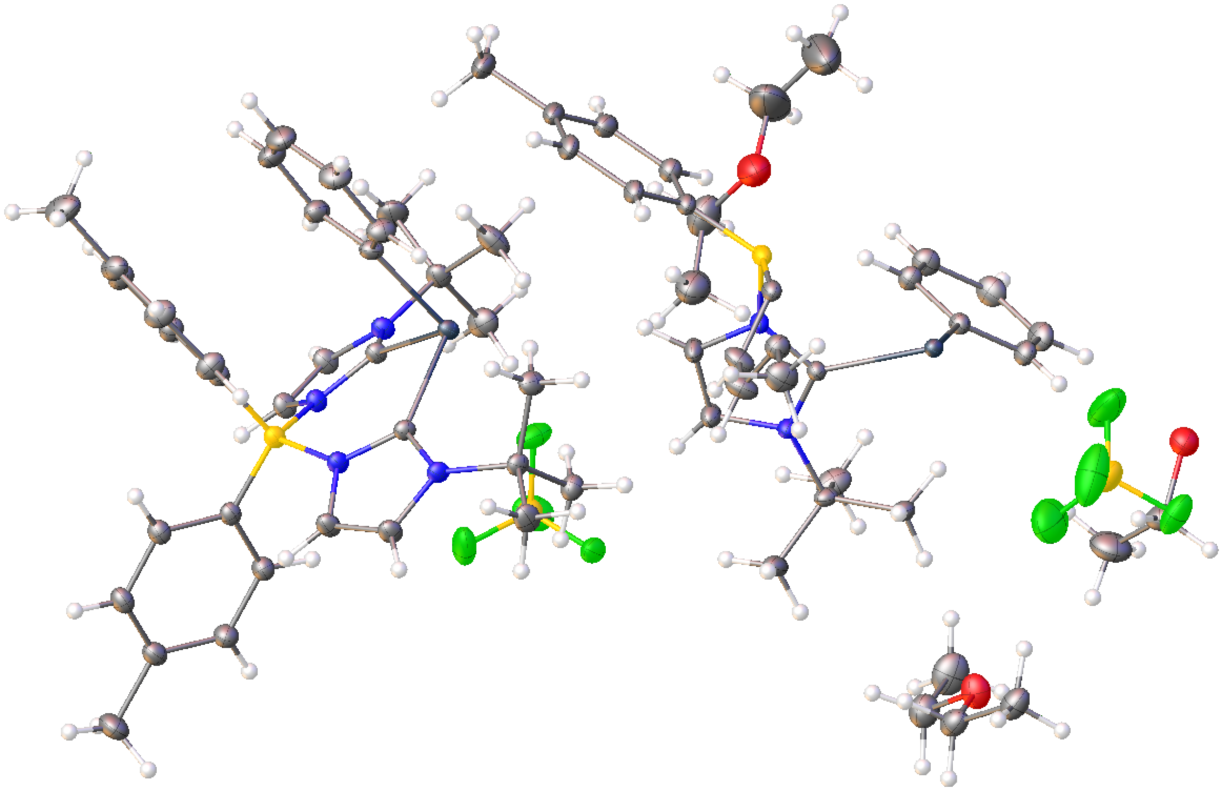

Figure S36. Olex-2 representation of the molecular structure of complex **11a**

### 9.11.1 Experimental

Single colourless block-shaped crystals of **11a** were recrystallised by slow diffusion of diethyl ether and into a saturated solution of THF at -30degs C. A suitable crystal with dimensions 0.15 × 0.08 × 0.08 mm3 was selected and mounted on a MITIGEN holder in oil on a Rigaku FRE+ diffractometer with Arc)Sec VHF Varimax confocal mirrors, a UG2 goniometer and HyPix 6000HE detector diffractometer. The crystal was kept at a steady T = 100(2) K during data collection. The structure was solved with the ShelXT 2018/2 (Sheldrick, 2015) solution program using dual methods and by using Olex2 1.5-dev (Dolomanov et al., 2009) as the graphical interface. The model was refined with olex2.refine 1.5-dev (Bourhis et al., 2015) using full matrix least squares minimisation on F2.

9.11.2 Crystal Data.

$C_{39.33}H_{54.33}B_2F_4N_4O_{1.33}Sb$, $M_r$ = 823.965, orthorhombic, *Pnma* (No. 62), a = 28.1188(3) Å, b = 31.1696(3) Å, c = 13.7378(1) Å, $\alpha = \beta = \gamma = 90^{\circ}$, $V$ = 12040.52(19) Å$^3$, $T$ = 100(2) K, $Z$ = 12, $Z'$ = 1.5, $\mu$(Mo $K_{\alpha}$) = 0.741, 410672 reflections measured, 32300 unique ($R_{int}$ = 0.0398) which were used in all calculations. The final $wR_2$ was 0.0754 (all data) and $R_1$ was 0.0300 (I≥2 $\sigma$(I)).

| Compound | **11a** |
|---|---|
| | |
| Formula | $C_{39.33}H_{54.33}B_2F_4N_4O_{1.33}Sb$ |
| $D_{calc.}$/ g cm$^{-3}$ | 1.364 |
| *m*/mm$^{-1}$ | 0.741 |
| Formula Weight | 823.965 |
| Colour | colourless |
| Shape | block-shaped |
| Size/mm$^3$ | 0.15×0.08×0.08 |
| *T*/K | 100(2) |
| Crystal System | orthorhombic |
| Space Group | *Pnma* |
| *a*/Å | 28.1188(3) |
| *b*/Å | 31.1696(3) |
| *c*/Å | 13.7378(1) |
| *a*/° | 90 |
| *b*/° | 90 |
| *g*/° | 90 |
| V/Å$^3$ | 12040.52(19) |
| *Z* | 12 |
| *Z'* | 1.5 |
| Wavelength/Å | 0.71073 |
| Radiation type | Mo $K_a$ |
| $Q_{min}$/° | 1.59 |
| $Q_{max}$/° | 38.01 |
| Measured Refl's. | 410672 |
| Indep't Refl's | 32300 |
| Refl's I≥2 *s*(I) | 27379 |
| $R_{int}$ | 0.0398 |
| Parameters | 800 |
| Restraints | 75 |
| Largest Peak | 1.6780 |
| Deepest Hole | -0.5535 |
| GooF | 1.0324 |
| $wR_2$ (all data) | 0.0754 |
| $wR_2$ | 0.0713 |
| $R_1$ (all data) | 0.0402 |
| $R_1$ | 0.300 |

### 9.11.3 Structure Quality Indicators

| | | | | |
|---|---|---|---|---|
| Reflections: | d min (MoKα) 2Θ=76.0° 0.58 | I/σ(I) 52.1 | Rint m=13.03 3.98% | Full 50.5° 97% to 76.0° 100 |
| Refinement: | Shift -0.000 | Max Peak 1.7 | Min Peak -0.6 | GooF 1.032 |

A colourless block-shaped-shaped crystal with dimensions 0.15 × 0.08 × 0.08 mm$^3$ was mounted on a MITIGEN holder in oil. Data were collected using a Rigaku FRE+ diffractometer with Arc)Sec VHF Varimax confocal mirrors, a UG2 goniometer and HyPix 6000HE detector diffractometer equipped with an Oxford Cryosystems low-temperature device operating at $T$ = 100(2) K.

Data were measured using profile data from $\omega$-scans with Mo $K_{\alpha}$ radiation. The diffraction pattern was indexed and the total number of runs and images was based on the strategy calculation from the program CrysAlisPro system (CCD 43.143a 64-bit (release 25-10-2024)). The maximum resolution that was achieved was $\Theta$ = 38.01° (0.58 Å).

The unit cell was refined using CrysAlisPro 1.171.43.143a (Rigaku OD, 2024) on 158106 reflections, 38% of the observed reflections.

Data reduction, scaling and absorption corrections were performed using CrysAlisPro 1.171.43.143a (Rigaku OD, 2024). The final completeness is 99.97 % out to 38.01° in $\Theta$. A multi-scan absorption correction was performed using CrysAlisPro 1.171.43.143a (Rigaku OD, 2024) Empirical absorption correction using spherical harmonics, implemented in SCALE3 ABSPACK scaling algorithm. The absorption coefficient $\mu$ of this material is 0.741 mm$^{-1}$ at this wavelength ($\lambda$ = 0.71073Å) and the minimum and maximum transmissions are 0.930 and 1.000.

The structure was solved and the space group *Pnma* (# 62) determined by the ShelXT 2018/2 (Sheldrick, 2018) structure solution program using dual methods and refined by full matrix least squares minimisation on $F^2$ using version of olex2.refine 1.5-dev (Bourhis et al., 2015). All non-hydrogen atoms were refined anisotropically. Hydrogen atom positions were calculated geometrically and refined using the riding model.

*_refine_special_details*: The structure is Z' = 3/2 with one molecule of the structure, one BF4- counterion and two solvent Et2O molecules lying along the crystallographic mirror plane. One BF4- and Et2O are modelled as disordered with PART -1 with equal distance restraints (SADI) between chemically equivalent disordered atom pairs and thermal restraints (RIGU).

*_exptl_absorpt_process_details*: CrysAlisPro 1.171.43.143a (Rigaku OD, 2024) using spherical harmonics, implemented in SCALE3 ABSPACK scaling algorithm.

The value of Z' is 1.5. The moiety formula is C34 H41 B N4 Sb, 1.333(C4 H10 O), B F4.

# 9.11.4 Data Plots: Diffraction Data

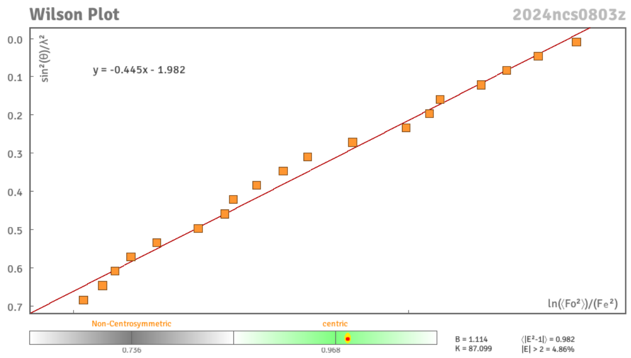


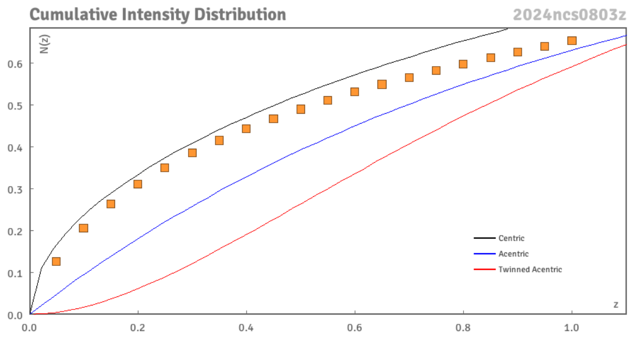


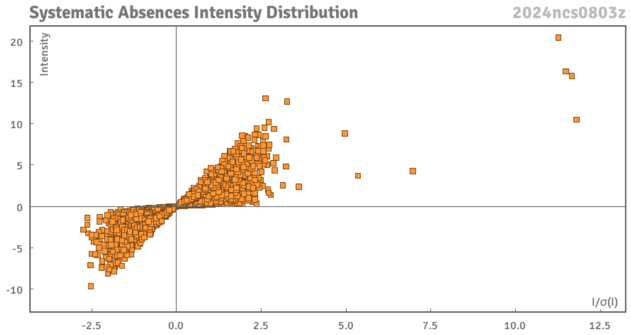


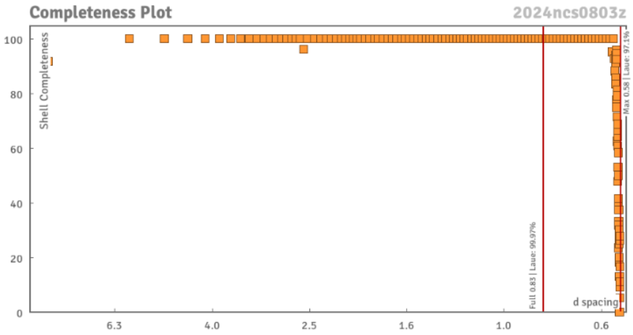


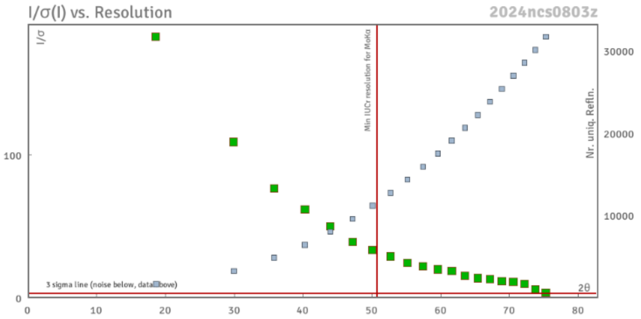


# 9.11.5 Data Plots: Refinement and Data

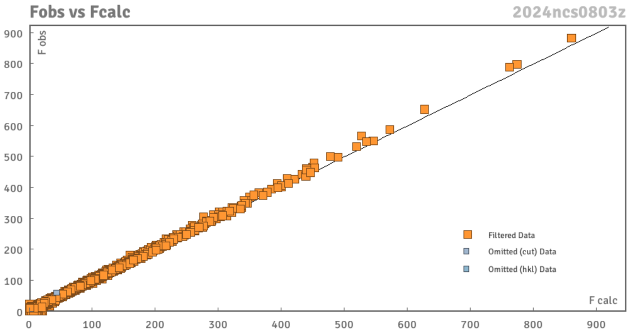


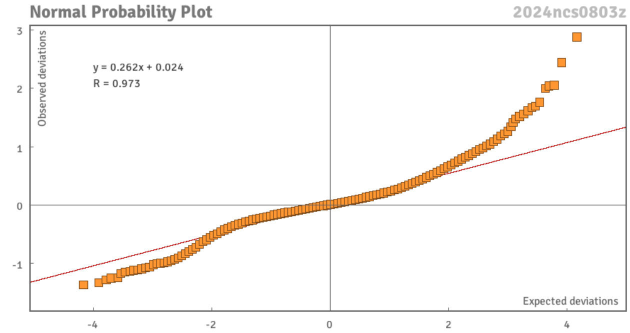

### 9.11.6 Reflection Statistics

| | | | |
|---|---|---|---|
| Total reflections (after filtering) | 420750 | Unique reflections | 32300 |
| Completeness | 0.971 | Mean I/$\sigma$ | 38.63 |
| $hkl_{max}$ collected | (47, 53, 22) | $hkl_{min}$ collected | (-48, -53, -23) |
| $hkl_{max}$ used | (48, 53, 23) | $hkl_{min}$ used | (0, 0, 0) |
| Lim $d_{max}$ collected | 100.0 | Lim $d_{min}$ collected | 0.36 |
| $d_{max}$ used | 13.63 | $d_{min}$ used | 0.58 |
| Friedel pairs | 103188 | Friedel pairs merged | 1 |
| Inconsistent equivalents | 10 | $R_{int}$ | 0.0398 |
| $R_{sigma}$ | 0.0192 | Intensity transformed | 0 |
| Omitted reflections | 0 | Omitted by user (OMIT hkl) | 10 |
| Multiplicity | (91778, 89165, 29089, 10564, 2846, 736, 241, 78, 9, 8, 1) | Maximum multiplicity | 46 |
| Removed systematic absences | 10088 | Filtered off (Shel/OMIT) | 0 |

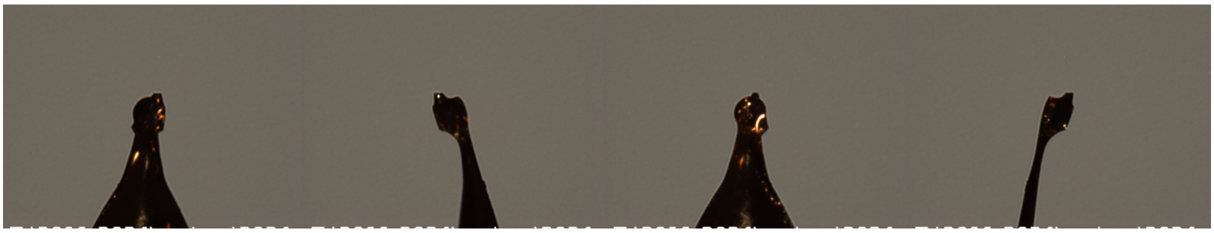

9.11.7 Fractional Atomic Coordinates and Equivalent Isotropic Displacement Parameters

Table S71. Fractional Atomic Coordinates (×$10^4$) and Equivalent Isotropic Displacement Parameters (Å$^2$×$10^3$) for **11a**. $U_{eq}$ is defined as 1/3 of the trace of the orthogonalised $U_{ij}$.

| Atom | x | y | z | $U_{eq}$ |
|---|---|---|---|---|
| Sb1 | 8175.55(5) | 3862.61(5) | 3951.98(10) | 14.163(16) |
| N1 | 8582.2(3) | 4730.9(3) | 4698.9(6) | 15.49(13) |
| N2 | 8304.2(3) | 4371.1(3) | 5946.1(6) | 15.34(13) |
| N3 | 8386.7(3) | 4715.1(3) | 2867.7(6) | 14.89(13) |
| N4 | 7951.4(3) | 4313.3(3) | 1906.5(6) | 16.28(14) |
| C1 | 8372.6(3) | 4361.7(3) | 4963.6(6) | 14.38(14) |
| C2 | 8654.3(4) | 4973.0(3) | 5524.3(7) | 19.74(17) |
| C3 | 8482.4(4) | 4752.3(3) | 6296.6(7) | 19.36(17) |
| C4 | 8024.5(4) | 4058.8(3) | 6555.7(7) | 16.36(15) |
| C5 | 8206.6(4) | 3602.6(4) | 6393.4(8) | 21.89(18) |
| C6 | 7503.2(4) | 4107.9(4) | 6276.1(8) | 21.45(18) |
| C7 | 8090.3(5) | 4172.8(4) | 7628.9(7) | 24.6(2) |
| C8 | 8172.5(3) | 4329.8(3) | 2787.5(7) | 14.64(14) |
| C9 | 8301.3(4) | 4941.0(3) | 2021.2(7) | 18.36(16) |
| C10 | 8031.9(4) | 4695.6(3) | 1429.3(7) | 19.17(17) |
| C11 | 7656.9(4) | 3971.7(4) | 1427.1(7) | 20.11(17) |
| C12 | 7879.7(6) | 3881.7(5) | 433.0(9) | 31.9(3) |
| C13 | 7152.6(5) | 4152.8(5) | 1298.8(10) | 29.4(2) |
| C14 | 7640.6(6) | 3564.9(4) | 2018.8(9) | 31.9(3) |
| C15 | 9277.2(4) | 4705.3(3) | 3415.5(7) | 16.77(15) |
| C16 | 9589.7(4) | 4564.4(4) | 4138.0(8) | 23.6(2) |
| C17 | 10046.5(4) | 4420.4(5) | 3923.3(9) | 27.6(2) |
| C18 | 10212.5(4) | 4412.1(4) | 2971.2(9) | 25.1(2) |
| C19 | 9908.8(4) | 4559.4(4) | 2243.0(8) | 23.75(19) |
| C20 | 9454.2(4) | 4704.0(4) | 2462.0(7) | 20.10(17) |
| C21 | 10699.3(5) | 4236.8(6) | 2742.3(12) | 37.7(3) |
| C22 | 8738.2(4) | 5408.4(3) | 3681.5(7) | 16.38(15) |
| C23 | 9154.3(4) | 5647.7(3) | 3805.9(7) | 18.61(16) |
| C24 | 9149.2(4) | 6093.4(4) | 3909.1(8) | 21.11(18) |
| C25 | 8725.0(4) | 6321.3(3) | 3874.0(8) | 21.48(18) |
| C26 | 8304.9(4) | 6086.2(4) | 3746.1(9) | 22.65(19) |
| C27 | 8310.9(4) | 5641.7(3) | 3665.6(8) | 20.12(17) |
| C28 | 8721.6(5) | 6804.7(4) | 3957.3(11) | 32.5(3) |
| C29 | 8881.0(4) | 3636.0(3) | 3652.3(7) | 18.20(16) |
| C30 | 9119.8(4) | 3401.1(4) | 4368.2(9) | 24.1(2) |
| C31 | 9565.6(5) | 3227.1(4) | 4180.2(10) | 30.5(2) |
| C32 | 9769.5(5) | 3279.8(4) | 3269.4(11) | 31.6(3) |
| C33 | 9532.6(5) | 3504.6(4) | 2549.7(10) | 28.7(2) |
| C34 | 9090.0(4) | 3684.1(4) | 2739.2(8) | 22.41(19) |
| B1 | 8754.2(4) | 4888.5(4) | 3657.5(7) | 15.65(17) |
| Sb51 | 5839.84(7) | 2500 | 6486.53(15) | 13.178(18) |
| N51 | 6702.7(3) | 2910.8(3) | 5424.4(6) | 15.93(13) |
| N52 | 6159.1(3) | 3409.6(3) | 5585.2(6) | 15.65(13) |
| C51 | 6268.8(3) | 2994.6(3) | 5790.8(7) | 13.98(14) |
| C52 | 6867.1(4) | 3277.1(3) | 4974.9(9) | 22.35(19) |
| C53 | 6531.6(4) | 3585.4(3) | 5068.0(9) | 22.43(19) |
| C54 | 5721.4(4) | 3667.8(3) | 5813.1(7) | 17.44(16) |
| C55 | 5886.5(5) | 4105.5(4) | 6193.1(10) | 27.1(2) |
| C56 | 5423.1(4) | 3453.0(3) | 6594.9(8) | 20.76(18) |
| C57 | 5435.6(5) | 3713.9(5) | 4874.7(9) | 30.1(2) |

| | | | | |
|---|---|---|---|---|
| C58 | 7460.6(5) | 2500 | 4876.8(11) | 17.4(2) |
| C59 | 7935.7(5) | 2500 | 5175.3(12) | 19.6(2) |
| C60 | 8311.5(6) | 2500 | 4507.2(14) | 24.3(3) |
| C61 | 8224.4(6) | 2500 | 3510.6(13) | 24.3(3) |
| C62 | 7750.7(6) | 2500 | 3201.6(12) | 23.9(3) |
| C63 | 7377.6(6) | 2500 | 3868.3(11) | 21.1(3) |
| C64 | 8628.8(7) | 2500 | 2791.9(16) | 32.3(4) |
| C65 | 7174.0(5) | 2500 | 6765.3(11) | 16.8(2) |
| C66 | 7265.3(5) | 2878.1(4) | 7275.0(9) | 25.6(2) |
| C67 | 7440.2(5) | 2879.2(4) | 8222.8(9) | 28.0(2) |
| C68 | 7528.7(6) | 2500 | 8720.1(12) | 22.2(3) |
| C69 | 7709.6(8) | 2500 | 9752.3(13) | 32.7(4) |
| C70 | 5379.0(5) | 2500 | 5231.8(10) | 15.9(2) |
| C71 | 5577.7(5) | 2500 | 4297.7(10) | 19.0(2) |
| C72 | 5284.5(6) | 2500 | 3484.1(12) | 23.9(3) |
| C73 | 4793.3(6) | 2500 | 3595.1(13) | 27.2(3) |
| C74 | 4594.5(6) | 2500 | 4518.3(13) | 25.9(3) |
| C75 | 4887.0(5) | 2500 | 5341.4(12) | 20.4(2) |
| B51 | 7019.6(5) | 2500 | 5631.3(12) | 16.4(2) |
| O91 | 4346.5(4) | 4249.4(3) | 6876.8(7) | 32.41(19) |
| C91 | 4621.9(4) | 4343.7(4) | 7718.1(9) | 27.7(2) |
| C92 | 4544.1(5) | 3992.0(5) | 8452.1(10) | 31.4(2) |
| C93 | 4373.2(5) | 4580.5(5) | 6172.8(11) | 34.7(3) |
| C94 | 4080.4(6) | 4450.9(7) | 5299.9(12) | 45.5(4) |
| O101 | 6318.4(9) | 2573.9(10) | 2169(2) | 53.7(9) |
| C101 | 6339.9(16) | 2991.6(18) | 1813(3) | 48.1(8) |
| C102 | 6400.8(15) | 3279.2(12) | 2681(3) | 47.4(8) |
| C103 | 6235(3) | 2286.3(19) | 1372(5) | 99(2) |
| C104 | 6220(3) | 1846(2) | 1754(8) | 119(3) |
| O111 | 3817.9(5) | 2500 | 6247.2(11) | 31.5(3) |
| C111 | 3829.1(6) | 2878.1(5) | 6826.6(12) | 38.3(3) |
| C112 | 3840.5(8) | 3257.6(5) | 6164.9(15) | 49.7(4) |
| F41 | 7188.9(3) | 4276.2(3) | 3857.7(6) | 33.96(18) |
| F42 | 6704.2(4) | 4795.2(3) | 3271.9(6) | 40.8(2) |
| F43 | 7321.0(3) | 4978.9(3) | 4262.4(7) | 42.4(2) |
| F44 | 6684.6(3) | 4624.6(2) | 4871.8(6) | 27.76(14) |
| B41 | 6977.6(5) | 4672.5(4) | 4059.7(8) | 19.76(19) |
| F81 | 4998.4(13) | 2923.3(9) | 8306(3) | 66.3(8) |
| F82 | 4446.2(5) | 2500 | 8999.6(9) | 43.0(3) |
| F83 | 4931.4(11) | 2245.2(12) | 7817(2) | 76.2(9) |
| F84 | 5229.4(7) | 2422.1(19) | 9311.8(17) | 72.1(16) |
| B81 | 4900.9(8) | 2500 | 8618.2(17) | 33.9(4) |

9.11.8 Anisotropic Displacement Parameters

Table S72. Anisotropic Displacement Parameters (×10^4) for **11a**. The anisotropic displacement factor exponent takes the form: $-2\pi^2[h^2a^{*2} \times U_{11} + ... +2hka^* \times b^* \times U_{12}]$

| Atom | $U_{11}$ | $U_{22}$ | $U_{33}$ | $U_{23}$ | $U_{13}$ | $U_{12}$ |
|---|---|---|---|---|---|---|
| Sb1 | 18.56(3) | 13.00(3) | 10.93(3) | -0.370(19) | 0.206(18) | 0.437(17) |
| N1 | 19.3(3) | 16.1(3) | 11.2(3) | -1.4(3) | 0.3(2) | 0.0(2) |
| N2 | 19.0(3) | 16.3(3) | 10.8(3) | 0.0(3) | 0.9(2) | 0.3(2) |
| N3 | 17.9(3) | 15.1(3) | 11.7(3) | -1.2(3) | -0.7(2) | 1.8(2) |
| N4 | 20.5(4) | 16.6(3) | 11.7(3) | -1.0(3) | -2.0(3) | -0.3(2) |
| C1 | 17.5(4) | 15.0(3) | 10.6(3) | 0.8(3) | 0.1(3) | 0.4(3) |
| C2 | 27.8(5) | 18.6(4) | 12.9(4) | -4.1(3) | 0.6(3) | -1.9(3) |
| C3 | 26.0(5) | 19.2(4) | 12.9(3) | -3.0(3) | 0.5(3) | -2.0(3) |
| C4 | 19.7(4) | 17.7(4) | 11.6(3) | -0.2(3) | 1.4(3) | 1.8(3) |
| C5 | 27.8(5) | 19.0(4) | 18.8(4) | 3.0(4) | 2.2(4) | 4.1(3) |
| C6 | 19.7(4) | 23.1(5) | 21.6(4) | -0.3(4) | 1.8(3) | 2.8(4) |
| C7 | 33.7(6) | 27.9(5) | 12.1(4) | -5.5(4) | 2.2(4) | 0.6(3) |
| C8 | 17.6(4) | 15.1(3) | 11.2(3) | -0.5(3) | -0.6(3) | 0.3(3) |
| C9 | 23.8(4) | 18.1(4) | 13.2(3) | -1.9(3) | -1.7(3) | 3.8(3) |
| C10 | 25.2(5) | 19.4(4) | 12.9(4) | -0.8(3) | -3.2(3) | 2.8(3) |
| C11 | 25.0(5) | 20.8(4) | 14.5(4) | -3.3(3) | -1.9(3) | -3.4(3) |
| C12 | 41.5(7) | 33.5(6) | 20.7(5) | -4.4(5) | 5.0(5) | -10.1(4) |
| C13 | 26.5(5) | 34.5(6) | 27.4(5) | -1.1(5) | -5.6(4) | -5.3(5) |
| C14 | 48.0(8) | 22.2(5) | 25.6(5) | -8.9(5) | -10.2(5) | 0.2(4) |
| C15 | 18.4(4) | 17.3(4) | 14.6(4) | -1.6(3) | -0.4(3) | -0.3(3) |
| C16 | 22.4(5) | 31.7(5) | 16.6(4) | 3.3(4) | -2.3(3) | -0.2(4) |
| C17 | 22.4(5) | 35.0(6) | 25.4(5) | 5.5(4) | -5.6(4) | -3.5(4) |
| C18 | 19.5(4) | 26.6(5) | 29.1(5) | 0.2(4) | -0.7(4) | -7.5(4) |
| C19 | 23.2(5) | 27.1(5) | 20.9(4) | -1.2(4) | 3.1(4) | -4.1(4) |
| C20 | 21.4(4) | 23.1(4) | 15.8(4) | -0.8(4) | 0.7(3) | -0.1(3) |
| C21 | 24.5(6) | 46.8(8) | 41.8(7) | 7.6(5) | -0.2(5) | -12.7(6) |
| C22 | 19.3(4) | 17.3(4) | 12.5(3) | -2.1(3) | 0.1(3) | 1.1(3) |
| C23 | 20.7(4) | 18.1(4) | 17.0(4) | -2.3(3) | -2.0(3) | 1.0(3) |
| C24 | 24.1(5) | 19.0(4) | 20.3(4) | -5.0(3) | -1.7(3) | 0.3(3) |
| C25 | 27.2(5) | 17.3(4) | 19.9(4) | -2.3(4) | 1.5(4) | -0.8(3) |
| C26 | 23.2(5) | 18.7(4) | 26.1(5) | 0.6(4) | 2.4(4) | -0.5(4) |
| C27 | 20.6(4) | 17.9(4) | 21.9(4) | -1.7(3) | 1.4(3) | 0.4(3) |
| C28 | 37.2(7) | 17.7(5) | 42.6(7) | -2.6(4) | 2.2(5) | -3.4(4) |
| C29 | 21.6(4) | 14.9(4) | 18.0(4) | 2.4(3) | 1.5(3) | -1.0(3) |
| C30 | 27.1(5) | 23.1(5) | 22.0(5) | 5.7(4) | -0.4(4) | 1.5(4) |
| C31 | 28.2(6) | 28.5(6) | 34.7(6) | 9.7(4) | -2.8(5) | 0.4(5) |
| C32 | 25.2(5) | 26.8(6) | 42.9(7) | 6.7(4) | 5.6(5) | -4.4(5) |
| C33 | 29.7(6) | 25.5(5) | 31.0(6) | 2.8(4) | 11.3(4) | -3.1(4) |
| C34 | 26.9(5) | 19.6(4) | 20.7(4) | 1.9(4) | 4.7(4) | -0.5(3) |
| B1 | 19.3(4) | 16.5(4) | 11.2(4) | -2.0(3) | -0.6(3) | 0.8(3) |
| Sb51 | 14.18(3) | 12.30(3) | 13.05(3) | -0 | 1.80(3) | 0 |
| N51 | 12.9(3) | 13.2(3) | 21.6(4) | -0.4(2) | 2.2(3) | 1.2(3) |
| N52 | 15.8(3) | 12.6(3) | 18.6(3) | 1.1(2) | 2.6(3) | 1.2(3) |
| C51 | 13.6(3) | 12.5(3) | 15.8(3) | -0.4(3) | 0.8(3) | 0.3(3) |
| C52 | 18.2(4) | 15.6(4) | 33.3(5) | -0.6(3) | 8.0(4) | 4.1(4) |
| C53 | 21.2(4) | 14.8(4) | 31.3(5) | 0.9(3) | 8.9(4) | 4.7(4) |
| C54 | 19.3(4) | 14.4(4) | 18.7(4) | 4.5(3) | 3.4(3) | 0.2(3) |
| C55 | 34.2(6) | 14.8(4) | 32.4(6) | -1.1(4) | 13.4(5) | -2.9(4) |
| C56 | 21.3(4) | 16.4(4) | 24.6(5) | 1.2(3) | 7.2(4) | -1.7(3) |
| C57 | 29.9(6) | 35.2(6) | 25.3(5) | 13.8(5) | -2.8(4) | 4.2(5) |
| C58 | 14.4(5) | 14.2(5) | 23.7(6) | -0 | 3.1(4) | 0 |

| | | | | | | |
|---|---|---|---|---|---|---|
| C59 | 13.5(5) | 18.0(6) | 27.3(7) | -0 | 3.0(5) | 0 |
| C60 | 15.3(6) | 21.3(6) | 36.4(8) | -0 | 6.9(6) | 0 |
| C61 | 23.5(7) | 15.8(6) | 33.7(8) | -0 | 12.8(6) | 0 |
| C62 | 27.6(7) | 18.9(6) | 25.3(7) | -0 | 7.9(6) | 0 |
| C63 | 19.4(6) | 19.5(6) | 24.4(6) | -0 | 3.0(5) | 0 |
| C64 | 30.4(8) | 25.0(7) | 41.4(10) | -0 | 19.8(7) | 0 |
| C65 | 14.1(5) | 14.1(5) | 22.4(6) | -0 | 2.3(4) | 0 |
| C66 | 33.3(6) | 14.7(4) | 28.7(5) | -0.7(4) | -6.2(4) | 0.7(4) |
| C67 | 37.7(6) | 17.5(4) | 28.9(5) | 1.2(4) | -6.1(5) | -3.5(4) |
| C68 | 22.8(7) | 21.7(6) | 22.3(6) | -0 | 1.3(5) | 0 |
| C69 | 40.9(10) | 34.0(9) | 23.3(7) | -0 | -2.3(7) | 0 |
| C70 | 14.0(5) | 15.3(5) | 18.3(5) | -0 | -1.2(4) | 0 |
| C71 | 18.1(6) | 21.8(6) | 17.1(6) | -0 | -0.7(4) | 0 |
| C72 | 26.3(7) | 26.5(7) | 18.9(6) | -0 | -3.2(5) | 0 |
| C73 | 24.1(7) | 29.7(8) | 27.7(7) | -0 | -10.2(6) | 0 |
| C74 | 16.2(6) | 29.6(8) | 32.1(8) | -0 | -4.3(5) | 0 |
| C75 | 15.1(5) | 21.8(6) | 24.2(6) | -0 | 0.4(5) | 0 |
| B51 | 12.8(6) | 13.4(5) | 23.1(7) | -0 | 1.2(5) | 0 |
| O91 | 31.3(5) | 37.0(5) | 28.9(4) | -6.0(4) | -5.6(4) | 2.6(4) |
| C91 | 22.3(5) | 34.0(6) | 26.9(5) | -0.4(4) | 0.8(4) | -5.6(4) |
| C92 | 24.1(5) | 43.3(7) | 26.7(5) | 1.8(5) | 2.2(4) | -1.1(5) |
| C93 | 33.1(7) | 37.7(7) | 33.5(6) | -0.9(5) | 1.5(5) | 3.5(5) |
| C94 | 43.7(8) | 63.6(11) | 29.1(7) | -0.4(8) | -4.2(6) | 7.1(7) |
| O101 | 44.0(11) | 41(2) | 75.9(15) | -2.9(10) | 18.0(11) | -12.2(12) |
| C101 | 43(2) | 63(3) | 38.6(16) | -2.8(16) | 7.4(13) | 3.7(14) |
| C102 | 49.9(19) | 37.3(15) | 55(2) | 4.2(13) | 12.5(15) | 4.1(13) |
| C103 | 127(5) | 70(3) | 102(4) | -41(3) | 61(3) | -49(2) |
| C104 | 71(4) | 55(3) | 232(10) | -13(2) | 63(5) | -36(3) |
| O111 | 31.3(7) | 26.0(6) | 37.1(7) | -0 | 3.5(5) | 0 |
| C111 | 41.0(8) | 32.1(7) | 41.9(7) | 7.9(6) | 5.5(6) | -7.6(6) |
| C112 | 61.5(11) | 26.7(7) | 61.0(11) | 8.3(7) | 0.3(9) | -2.5(7) |
| F41 | 39.7(4) | 32.8(4) | 29.4(4) | 13.9(3) | 9.8(3) | 2.0(3) |
| F42 | 58.0(6) | 38.4(5) | 26.0(4) | 6.7(4) | -15.3(4) | 7.5(3) |
| F43 | 40.1(5) | 51.6(5) | 35.5(4) | -25.4(4) | 9.1(4) | -9.4(4) |
| F44 | 33.4(4) | 23.7(3) | 26.2(3) | -2.8(3) | 12.6(3) | -1.0(3) |
| B41 | 23.7(5) | 20.9(5) | 14.7(4) | -0.6(4) | 0.4(4) | 2.6(4) |
| F81 | 72.1(18) | 47.9(14) | 79(2) | -1.9(11) | 22.8(16) | 20.8(11) |
| F82 | 29.2(6) | 70.3(10) | 29.5(6) | -0 | 9.6(4) | 0 |
| F83 | 53.0(15) | 101(2) | 74.7(18) | -22.4(14) | 33.6(14) | -50.9(14) |
| F84 | 34.5(8) | 112(4) | 70.2(13) | 0.0(13) | 2.6(8) | 48(2) |
| B81 | 30.5(9) | 40.1(11) | 31.0(9) | -0 | 11.2(7) | 0 |

9.11.9 Bond lengths

Table S73. Bond Lengths in Å for **11a**.

| Atom | Atom | Length/Å |
|---|---|---|
| Sb1 | C1 | 2.1583(9) |
| Sb1 | C8 | 2.1632(9) |
| Sb1 | C29 | 2.1455(10) |
| N1 | C1 | 1.3433(12) |
| N1 | C2 | 1.3770(13) |
| N1 | B1 | 1.5881(13) |
| N2 | C1 | 1.3637(12) |
| N2 | C3 | 1.3766(13) |
| N2 | C4 | 1.5057(13) |
| N3 | C8 | 1.3480(12) |
| N3 | C9 | 1.3806(12) |
| N3 | B1 | 1.5929(13) |
| N4 | C8 | 1.3617(12) |
| N4 | C10 | 1.3787(13) |
| N4 | C11 | 1.5012(13) |
| C2 | C3 | 1.3537(14) |
| C4 | C5 | 1.5277(15) |
| C4 | C6 | 1.5231(15) |
| C4 | C7 | 1.5278(14) |
| C9 | C10 | 1.3491(14) |
| C11 | C12 | 1.5285(16) |
| C11 | C13 | 1.5363(18) |
| C11 | C14 | 1.5067(17) |
| C15 | C16 | 1.3966(15) |
| C15 | C20 | 1.4014(14) |
| C15 | B1 | 1.6121(15) |
| C16 | C17 | 1.3923(17) |
| C17 | C18 | 1.3890(18) |
| C18 | C19 | 1.3932(17) |
| C18 | C21 | 1.5070(18) |
| C19 | C20 | 1.3883(16) |
| C22 | C23 | 1.3979(14) |
| C22 | C27 | 1.4045(15) |
| C22 | B1 | 1.6215(15) |
| C23 | C24 | 1.3966(15) |
| C24 | C25 | 1.3891(17) |
| C25 | C26 | 1.4013(16) |
| C25 | C28 | 1.5111(17) |
| C26 | C27 | 1.3901(15) |
| C29 | C30 | 1.3979(15) |
| C29 | C34 | 1.3934(15) |
| C30 | C31 | 1.3902(17) |
| C31 | C32 | 1.386(2) |
| C32 | C33 | 1.383(2) |
| C33 | C34 | 1.3890(17) |
| Sb51 | C51 | 2.1782(9) |
| Sb51 | C51[1] | 2.1782(9) |
| Sb51 | C70 | 2.1566(14) |
| N51 | C51 | 1.3454(12) |
| N51 | C52 | 1.3778(13) |
| N51 | B51 | 1.5858(12) |
| N52 | C51 | 1.3596(12) |
| N52 | C53 | 1.3794(13) |
| N52 | C54 | 1.5034(13) |
| C52 | C53 | 1.3527(15) |
| C54 | C55 | 1.5329(16) |
| C54 | C56 | 1.5185(14) |
| C54 | C57 | 1.5257(16) |
| C58 | C59 | 1.398(2) |
| C58 | C63 | 1.405(2) |
| C58 | B51 | 1.616(2) |
| C59 | C60 | 1.400(2) |
| C60 | C61 | 1.391(3) |
| C61 | C62 | 1.398(3) |
| C61 | C64 | 1.506(2) |
| C62 | C63 | 1.393(2) |
| C65 | C66[1] | 1.3946(14) |
| C65 | C66 | 1.3946(14) |
| C65 | B51 | 1.617(2) |
| C66 | C67 | 1.3919(17) |
| C67 | C68 | 1.3876(15) |
| C68 | C69 | 1.507(2) |
| C70 | C71 | 1.400(2) |
| C70 | C75 | 1.392(2) |
| C71 | C72 | 1.389(2) |
| C72 | C73 | 1.390(2) |
| C73 | C74 | 1.386(3) |
| C74 | C75 | 1.398(2) |
| O91 | C91 | 1.4221(15) |
| O91 | C93 | 1.4164(18) |
| C91 | C92 | 1.506(2) |
| C93 | C94 | 1.510(2) |
| O101 | C101 | 1.393(6) |
| O101 | C103 | 1.434(6) |
| C101 | C102 | 1.501(6) |
| C103 | C104 | 1.470(10) |
| O111 | C111[1] | 1.4225(17) |
| O111 | C111 | 1.4225(17) |
| C111 | C112 | 1.492(2) |
| F41 | B41 | 1.3984(15) |
| F42 | B41 | 1.3816(14) |
| F43 | B41 | 1.3863(15) |
| F44 | B41 | 1.3950(14) |
| F81 | B81[1] | 1.415(3) |
| F82 | B81 | 1.382(2) |
| F83 | B81 | 1.360(3) |
| F84 | B81[1] | 1.349(3) |

----
[1]+x,1/2-y,+z

9.11.10 Bond angles

Table S74. Bond Angles in ° for **11a**.

| Atom | Atom | Atom | Angle/° |
|---|---|---|---|
| C8 | Sb1 | C1 | 89.54(3) |
| C29 | Sb1 | C1 | 97.10(4) |
| C29 | Sb1 | C8 | 94.78(4) |
| C2 | N1 | C1 | 108.13(8) |
| B1 | N1 | C1 | 129.98(8) |
| B1 | N1 | C2 | 121.84(8) |
| C3 | N2 | C1 | 108.27(8) |
| C4 | N2 | C1 | 127.61(8) |
| C4 | N2 | C3 | 123.62(8) |
| C9 | N3 | C8 | 107.93(8) |
| B1 | N3 | C8 | 130.38(8) |
| B1 | N3 | C9 | 120.90(8) |
| C10 | N4 | C8 | 108.37(8) |
| C11 | N4 | C8 | 131.96(8) |
| C11 | N4 | C10 | 119.67(8) |
| N1 | C1 | Sb1 | 123.77(6) |
| N2 | C1 | Sb1 | 128.07(7) |
| N2 | C1 | N1 | 108.14(8) |
| C3 | C2 | N1 | 108.32(9) |
| C2 | C3 | N2 | 107.13(8) |
| C5 | C4 | N2 | 110.22(8) |
| C6 | C4 | N2 | 107.30(8) |
| C6 | C4 | C5 | 112.29(9) |
| C7 | C4 | N2 | 108.85(8) |
| C7 | C4 | C5 | 108.47(9) |
| C7 | C4 | C6 | 109.66(9) |
| N3 | C8 | Sb1 | 122.52(6) |
| N4 | C8 | Sb1 | 129.32(7) |
| N4 | C8 | N3 | 108.07(8) |
| C10 | C9 | N3 | 108.43(9) |
| C9 | C10 | N4 | 107.19(8) |
| C12 | C11 | N4 | 107.23(9) |
| C13 | C11 | N4 | 107.39(9) |
| C13 | C11 | C12 | 110.07(10) |
| C14 | C11 | N4 | 112.15(9) |
| C14 | C11 | C12 | 109.88(10) |
| C14 | C11 | C13 | 110.06(11) |
| C20 | C15 | C16 | 116.10(10) |
| B1 | C15 | C16 | 122.61(9) |
| B1 | C15 | C20 | 121.19(9) |
| C17 | C16 | C15 | 122.10(10) |
| C18 | C17 | C16 | 121.04(11) |
| C19 | C18 | C17 | 117.64(11) |
| C21 | C18 | C17 | 120.57(12) |
| C21 | C18 | C19 | 121.76(12) |
| C20 | C19 | C18 | 121.05(10) |
| C19 | C20 | C15 | 122.04(10) |
| C27 | C22 | C23 | 116.22(9) |
| B1 | C22 | C23 | 120.81(9) |
| B1 | C22 | C27 | 122.74(9) |
| C24 | C23 | C22 | 122.31(10) |
| C25 | C24 | C23 | 120.94(10) |
| C26 | C25 | C24 | 117.43(10) |
| C28 | C25 | C24 | 120.86(11) |
| C28 | C25 | C26 | 121.71(11) |
| C27 | C26 | C25 | 121.41(11) |
| C26 | C27 | C22 | 121.67(10) |
| C30 | C29 | Sb1 | 118.79(8) |
| C34 | C29 | Sb1 | 121.82(8) |
| C34 | C29 | C30 | 119.15(10) |
| C31 | C30 | C29 | 120.45(11) |
| C32 | C31 | C30 | 119.63(12) |
| C33 | C32 | C31 | 120.41(12) |
| C34 | C33 | C32 | 120.12(12) |
| C33 | C34 | C29 | 120.21(11) |
| N3 | B1 | N1 | 108.13(8) |
| C15 | B1 | N1 | 110.73(8) |
| C15 | B1 | N3 | 109.33(8) |
| C22 | B1 | N1 | 106.40(8) |
| C22 | B1 | N3 | 109.57(8) |
| C22 | B1 | C15 | 112.56(8) |
| C51[1] | Sb51 | C51 | 90.10(5) |
| C70 | Sb51 | C51[1] | 88.97(4) |
| C70 | Sb51 | C51 | 88.97(4) |
| C52 | N51 | C51 | 108.13(8) |
| B51 | N51 | C51 | 126.81(9) |
| B51 | N51 | C52 | 124.12(9) |
| C53 | N52 | C51 | 108.22(8) |
| C54 | N52 | C51 | 130.69(8) |
| C54 | N52 | C53 | 121.08(8) |
| N51 | C51 | Sb51 | 121.94(7) |
| N52 | C51 | Sb51 | 129.70(7) |
| N52 | C51 | N51 | 108.22(8) |
| C53 | C52 | N51 | 108.20(9) |
| C52 | C53 | N52 | 107.23(9) |
| C55 | C54 | N52 | 107.42(9) |
| C56 | C54 | N52 | 111.31(8) |
| C56 | C54 | C55 | 108.60(9) |
| C57 | C54 | N52 | 107.79(8) |
| C57 | C54 | C55 | 111.32(10) |
| C57 | C54 | C56 | 110.37(10) |
| C63 | C58 | C59 | 116.62(13) |
| B51 | C58 | C59 | 123.04(13) |
| B51 | C58 | C63 | 120.34(13) |
| C60 | C59 | C58 | 121.95(15) |
| C61 | C60 | C59 | 120.85(15) |
| C62 | C61 | C60 | 117.81(14) |
| C64 | C61 | C60 | 120.84(17) |
| C64 | C61 | C62 | 121.35(17) |
| C63 | C62 | C61 | 121.19(16) |
| C62 | C63 | C58 | 121.57(15) |
| C66[1] | C65 | C66 | 115.34(14) |
| B51 | C65 | C66 | 122.21(7) |
| B51 | C65 | C66[1] | 122.21(7) |
| C67 | C66 | C65 | 122.47(11) |

| | | | |
|---|---|---|---|
| C68 | C67 | C66 | 121.46(11) |
| C67[1] | C68 | C67 | 116.80(15) |
| C69 | C68 | C67[1] | 121.60(8) |
| C69 | C68 | C67 | 121.60(8) |
| C71 | C70 | Sb51 | 119.53(10) |
| C75 | C70 | Sb51 | 120.72(11) |
| C75 | C70 | C71 | 119.75(13) |
| C72 | C71 | C70 | 120.05(14) |
| C73 | C72 | C71 | 120.11(15) |
| C74 | C73 | C72 | 120.09(15) |
| C75 | C74 | C73 | 120.19(15) |
| C74 | C75 | C70 | 119.82(14) |
| N51[1] | B51 | N51 | 107.71(11) |
| C58 | B51 | N51 | 108.43(8) |
| C58 | B51 | N51[1] | 108.43(8) |
| C65 | B51 | N51[1] | 108.87(8) |
| C65 | B51 | N51 | 108.87(8) |
| C65 | B51 | C58 | 114.33(12) |
| C93 | O91 | C91 | 112.04(11) |
| C92 | C91 | O91 | 108.33(11) |
| C94 | C93 | O91 | 108.57(13) |
| C103 | O101 | C101 | 108.8(4) |
| C102 | C101 | O101 | 106.5(3) |
| C104 | C103 | O101 | 108.4(7) |
| C111 | O111 | C111[1] | 111.89(17) |
| C112 | C111 | O111[1] | 108.44(14) |
| F42 | B41 | F41 | 109.00(10) |
| F43 | B41 | F41 | 110.63(11) |

| | | | |
|---|---|---|---|
| F43 | B41 | F42 | 110.75(10) |
| F44 | B41 | F41 | 108.37(9) |
| F44 | B41 | F42 | 109.11(10) |
| F44 | B41 | F43 | 108.93(9) |
| F81 | B81 | F81[1] | 137.8(3) |
| F82 | B81 | F81 | 107.13(17) |
| F82 | B81 | F81[1] | 107.13(17) |
| F83 | B81 | F81 | 106.7(3) |
| F83[1] | B81 | F81 | 36.62(18) |
| F83[1] | B81 | F81[1] | 106.7(3) |
| F83 | B81 | F81[1] | 36.62(18) |
| F83 | B81 | F82 | 111.4(2) |
| F83[1] | B81 | F82 | 111.4(2) |
| F83[1] | B81 | F83 | 71.4(4) |
| F84 | B81 | F81[1] | 85.1(3) |
| F84[1] | B81 | F81 | 85.1(3) |
| F84 | B81 | F81 | 104.5(3) |
| F84[1] | B81 | F81[1] | 104.5(3) |
| F84 | B81 | F82 | 111.45(19) |
| F84[1] | B81 | F82 | 111.45(19) |
| F84 | B81 | F83[1] | 129.3(3) |
| F84[1] | B81 | F83[1] | 115.1(3) |
| F84 | B81 | F83 | 115.1(3) |
| F84[1] | B81 | F83 | 129.3(3) |
| F84[1] | B81 | F84 | 20.7(5) |

––––
[1]+x,1/2-y,+z

9.11.11 Torsion angles

Table S75. Torsion Angles in ° for **11a**.

| Atom | Atom | Atom | Atom | Angle/° |
|---|---|---|---|---|
| Sb1 | C1 | N1 | C2 | 179.73(8) |
| Sb1 | C1 | N1 | B1 | -2.85(11) |
| Sb1 | C1 | N2 | C3 | -179.57(9) |
| Sb1 | C1 | N2 | C4 | -7.63(11) |
| Sb1 | C8 | N3 | C9 | -177.40(8) |
| Sb1 | C8 | N3 | B1 | 12.93(10) |
| Sb1 | C8 | N4 | C10 | 176.90(9) |
| Sb1 | C8 | N4 | C11 | -2.65(12) |
| Sb1 | C29 | C30 | C31 | 176.13(9) |
| Sb1 | C29 | C34 | C33 | -175.06(9) |
| N1 | C1 | N2 | C3 | -0.91(9) |
| N1 | C1 | N2 | C4 | 171.03(7) |
| N1 | C2 | C3 | N2 | 0.15(10) |
| N1 | B1 | N3 | C8 | -41.81(11) |
| N1 | B1 | N3 | C9 | 149.67(8) |
| N1 | B1 | C15 | C16 | -20.77(11) |
| N1 | B1 | C15 | C20 | 163.24(8) |
| N1 | B1 | C22 | C23 | 101.56(8) |
| N1 | B1 | C22 | C27 | -72.76(9) |
| N3 | C8 | N4 | C10 | 0.26(9) |
| N3 | C8 | N4 | C11 | -179.29(8) |
| N3 | C9 | C10 | N4 | -0.37(10) |
| N3 | B1 | C15 | C16 | -139.80(9) |
| N3 | B1 | C15 | C20 | 44.20(10) |
| N3 | B1 | C22 | C23 | -141.78(8) |
| N3 | B1 | C22 | C27 | 43.91(10) |
| C15 | C16 | C17 | C18 | 0.19(15) |
| C15 | C20 | C19 | C18 | -0.64(13) |
| C15 | B1 | C22 | C23 | -19.90(10) |
| C15 | B1 | C22 | C27 | 165.78(8) |
| C16 | C17 | C18 | C19 | 1.04(16) |
| C16 | C17 | C18 | C21 | -177.19(13) |
| C17 | C18 | C19 | C20 | -0.82(14) |
| C22 | C23 | C24 | C25 | -1.21(12) |
| C22 | C27 | C26 | C25 | -1.79(13) |
| C23 | C24 | C25 | C26 | 0.92(13) |
| C23 | C24 | C25 | C28 | -178.26(11) |
| C24 | C25 | C26 | C27 | 0.54(12) |
| C29 | C30 | C31 | C32 | -1.33(14) |
| C29 | C34 | C33 | C32 | -0.46(14) |
| C30 | C31 | C32 | C33 | 0.11(16) |
| C31 | C32 | C33 | C34 | 0.78(16) |
| Sb51 | C51 | N51 | C52 | 175.71(9) |
| Sb51[1] | C51 | N51 | C52 | 175.71(9) |
| Sb51 | C51 | N51 | B51 | -15.10(11) |
| Sb51[1] | C51 | N51 | B51 | -15.10(11) |
| Sb51 | C51 | N52 | C53 | -175.02(9) |
| Sb51[1] | C51 | N52 | C53 | -175.02(9) |
| Sb51 | C51 | N52 | C54 | 3.61(11) |
| Sb51[1] | C51 | N52 | C54 | 3.61(11) |
| Sb51 | C70 | C71 | C72 | -180.0 |

| | | | | |
|---|---|---|---|---|
| Sb51 | C70 | C75 | C74 | 180.0 |
| N51 | C51 | N52 | C53 | 0.77(10) |
| N51 | C51 | N52 | C54 | 179.39(7) |
| N51 | C52 | C53 | N52 | 0.48(11) |
| N51 | B51 | N51[1] | C51[1] | -52.14(13) |
| N51 | B51 | N51[1] | C52[1] | 140.29(10) |
| N51 | B51 | C58 | C59 | 121.66(8) |
| N51 | B51 | C58 | C63 | -58.34(8) |
| N51 | B51 | C65 | C66 | -34.31(12) |
| N51 | B51 | C65 | C66[1] | 151.48(9) |
| C58 | C59 | C60 | C61 | 0.0 |
| C58 | C63 | C62 | C61 | 0.0 |
| C58 | B51 | C65 | C66[1] | -87.11(8) |
| C58 | B51 | C65 | C66 | 87.11(8) |
| C59 | C60 | C61 | C62 | 0.0 |
| C59 | C60 | C61 | C64 | 180.0 |
| C60 | C61 | C62 | C63 | 0.0 |
| C65[1] | C66 | C67 | C68 | -0.77(17) |
| C65 | C66 | C67 | C68 | -0.77(17) |
| C66 | C67 | C68 | C67[1] | 0.80(10) |
| C66 | C67 | C68 | C69 | -178.88(15) |
| C70 | C71 | C72 | C73 | 0.0 |
| C70 | C75 | C74 | C73 | 0.0 |
| C71 | C72 | C73 | C74 | 0.0 |
| C72 | C73 | C74 | C75 | 0.0 |

----

[1]+x,1/2-y,+z

9.11.12 Hydrogen Fractional Atomic Coordinates and Equivalent Isotropic Displacement Parameters

Table S76. Hydrogen Fractional Atomic Coordinates (×10[4]) and Equivalent Isotropic Displacement Parameters (Å[2]×10[3]) for **11a**. $U_{eq}$ is defined as 1/3 of the trace of the orthogonalised $U_{ij}$.

| Atom | x | y | z | $U_{eq}$ |
|---|---|---|---|---|
| H2 | 8799.7(4) | 5247.9(3) | 5547.8(7) | 23.7(2) |
| H3 | 8484.4(4) | 4843.4(3) | 6956.5(7) | 23.2(2) |
| H5a | 8549.9(8) | 3593.1(8) | 6514(7) | 32.8(3) |
| H5b | 8044(3) | 3406.3(6) | 6841(5) | 32.8(3) |
| H5c | 8142(3) | 3515.6(13) | 5721(2) | 32.8(3) |
| H6a | 7308.7(5) | 3917(2) | 6680(5) | 32.2(3) |
| H6b | 7403.8(11) | 4405.8(8) | 6379(7) | 32.2(3) |
| H6c | 7461.7(7) | 4032(3) | 5589(2) | 32.2(3) |
| H7a | 7959(4) | 4458.8(15) | 7752.7(19) | 36.9(3) |
| H7b | 7924(3) | 3962(2) | 8033.5(8) | 36.9(3) |
| H7c | 8429.9(5) | 4171(4) | 7789(2) | 36.9(3) |
| H9 | 8413.2(4) | 5221.7(3) | 1879.6(7) | 22.0(2) |
| H10 | 7918.9(4) | 4771.9(3) | 801.0(7) | 23.0(2) |
| H12a | 7703(3) | 3652(3) | 108(4) | 47.8(4) |
| H12b | 7867(4) | 4142.0(12) | 34(3) | 47.8(4) |
| H12c | 8211.6(14) | 3794(4) | 519.4(11) | 47.8(4) |
| H13a | 6951.8(12) | 3938.6(15) | 977(8) | 44.2(4) |
| H13b | 7018.5(17) | 4222(4) | 1938.2(11) | 44.2(4) |
| H13c | 7165.9(7) | 4413(2) | 900(7) | 44.2(4) |
| H14a | 7964.8(6) | 3458(2) | 2120(8) | 47.9(4) |
| H14b | 7492(4) | 3623.7(9) | 2650(4) | 47.9(4) |
| H14c | 7454(4) | 3348.1(13) | 1671(4) | 47.9(4) |
| H16 | 9487.3(4) | 4566.9(4) | 4797.0(8) | 28.3(2) |
| H17 | 10247.8(4) | 4326.4(5) | 4436.0(9) | 33.1(3) |
| H19 | 10014.5(4) | 4561.0(4) | 1586.1(8) | 28.5(2) |
| H20 | 9257.1(4) | 4805.2(4) | 1949.5(7) | 24.1(2) |
| H21a | 10667.8(5) | 3954(2) | 2437(9) | 56.6(5) |
| H21b | 10863.5(19) | 4432(2) | 2296(8) | 56.6(5) |
| H21c | 10883.1(18) | 4210(4) | 3345.3(17) | 56.6(5) |
| H23 | 9450.9(4) | 5501.9(3) | 3820.8(7) | 22.3(2) |
| H24 | 9439.9(4) | 6242.8(4) | 4004.7(8) | 25.3(2) |
| H26 | 8009.7(4) | 6233.7(4) | 3713.7(9) | 27.2(2) |
| H27 | 8018.5(4) | 5491.9(3) | 3598.1(8) | 24.1(2) |
| H28a | 9039.6(12) | 6905.6(5) | 4138(9) | 48.8(4) |
| H28b | 8631(4) | 6930.3(4) | 3330(3) | 48.8(4) |
| H28c | 8492(3) | 6891.8(4) | 4457(6) | 48.8(4) |
| H30 | 8976.5(4) | 3360.2(4) | 4987.5(9) | 28.9(2) |
| H31 | 9729.6(5) | 3072.8(4) | 4673.2(10) | 36.6(3) |
| H32 | 10073.8(5) | 3160.7(4) | 3138.7(11) | 37.9(3) |
| H33 | 9672.8(5) | 3536.4(4) | 1924.7(10) | 34.5(3) |
| H34 | 8929.3(4) | 3840.1(4) | 2244.6(8) | 26.9(2) |
| H52 | 7164.7(4) | 3307.8(3) | 4654.9(9) | 26.8(2) |
| H53 | 6549.3(4) | 3870.0(3) | 4822.9(9) | 26.9(2) |
| H55a | 5609.0(5) | 4277.0(14) | 6380(8) | 40.7(3) |
| H55b | 6092(3) | 4064.3(4) | 6761(5) | 40.7(3) |
| H55c | 6064(3) | 4255.2(16) | 5681(3) | 40.7(3) |
| H56a | 5163(2) | 3644.5(14) | 6786(6) | 31.1(3) |
| H56b | 5291(3) | 3184.5(17) | 6340(3) | 31.1(3) |

| | | | | |
|---|---|---|---|---|
| H56c | 5622.6(9) | 3391(3) | 7163(3) | 31.1(3) |
| H57a | 5344(4) | 3429.1(5) | 4639(5) | 45.2(4) |
| H57b | 5149(2) | 3884(3) | 5001(2) | 45.2(4) |
| H57c | 5630.3(15) | 3858(3) | 4381(3) | 45.2(4) |
| H59 | 8005.7(5) | 2500 | 5851.8(12) | 23.5(3) |
| H60 | 8630.0(6) | 2500 | 4737.4(14) | 29.2(3) |
| H62 | 7682.0(6) | 2500 | 2524.5(12) | 28.7(3) |
| H63 | 7059.4(6) | 2500 | 3635.7(11) | 25.3(3) |
| H64a | 8882(3) | 2310(5) | 3026(7) | 48.4(6) |
| H64b | 8514.3(18) | 2398(6) | 2159(4) | 48.4(6) |
| H64c | 8753(5) | 2792.0(11) | 2723(11) | 48.4(6) |
| H66 | 7205.9(5) | 3144.9(4) | 6963.5(9) | 30.7(3) |
| H67 | 7500.3(5) | 3145.4(4) | 8536.1(9) | 33.6(3) |
| H69a | 7690(7) | 2209.0(13) | 10019(5) | 49.1(6) |
| H69b | 8041(2) | 2597(6) | 9761(2) | 49.1(6) |
| H69c | 7515(5) | 2694(5) | 10148(4) | 49.1(6) |
| H71 | 5913.5(5) | 2500 | 4220.2(10) | 22.8(3) |
| H72 | 5420.0(6) | 2500 | 2850.6(12) | 28.7(3) |
| H73 | 4593.4(6) | 2500 | 3037.5(13) | 32.6(4) |
| H74 | 4258.6(6) | 2500 | 4592.1(13) | 31.1(4) |
| H75 | 4750.2(5) | 2500 | 5973.7(12) | 24.5(3) |
| H91a | 4963.0(4) | 4361.1(4) | 7543.9(9) | 33.3(3) |
| H91b | 4523.6(4) | 4623.2(4) | 7995.3(9) | 33.3(3) |
| H92a | 4607(4) | 3713.3(6) | 8148(3) | 47.1(4) |
| H92b | 4760(3) | 4033(2) | 9004(4) | 47.1(4) |
| H92c | 4214.3(13) | 4001(3) | 8682(7) | 47.1(4) |
| H93a | 4249.6(5) | 4851.7(5) | 6449.3(11) | 41.7(3) |
| H93b | 4708.2(5) | 4626.4(5) | 5976.9(11) | 41.7(3) |
| H94a | 4094(4) | 4678(2) | 4807(5) | 68.2(6) |
| H94b | 4207(3) | 4184(3) | 5026(7) | 68.2(6) |
| H94c | 3749.4(13) | 4406(5) | 5500(2) | 68.2(6) |
| H10a | 6611.9(16) | 3023.9(18) | 1361(3) | 57.7(10) |
| H10b | 6043.7(16) | 3064.1(18) | 1460(3) | 57.7(10) |
| H10c | 6427(11) | 3577.7(15) | 2463(3) | 71.1(12) |
| H10d | 6125(5) | 3250(7) | 3112(12) | 71.1(12) |
| H10e | 6690(6) | 3198(6) | 3034(13) | 71.1(12) |
| H10f | 5930(3) | 2357.4(19) | 1050(5) | 119(2) |
| H10g | 6494(3) | 2313.4(19) | 886(5) | 119(2) |
| H10h | 6539(5) | 1759(8) | 1960(50) | 179(5) |
| H10i | 6003(19) | 1834(5) | 2310(30) | 179(5) |
| H10j | 6110(20) | 1651(4) | 1244(17) | 179(5) |
| H11a | 3543.8(6) | 2890.7(5) | 7248.0(12) | 46.0(4) |
| H11b | 4114.6(6) | 2876.9(5) | 7248.2(12) | 46.0(4) |
| H11c | 4110(3) | 3231(3) | 5716(8) | 74.6(6) |
| H11d | 3544(2) | 3271(3) | 5793(9) | 74.6(6) |
| H11e | 3876(6) | 3519.9(7) | 6551.5(17) | 74.6(6) |

### 9.11.13 Atomic Occupancies for all atoms that are not fully occupied

Table S77. Atomic Occupancies for all atoms that are not fully occupied in **11a**.

| Atom | Occupancy |
|---|---|
| H64a | 0.500000 |
| H64b | 0.500000 |
| H64c | 0.500000 |
| H69a | 0.500000 |
| H69b | 0.500000 |
| H69c | 0.500000 |
| O101 | 0.500000 |
| C101 | 0.500000 |
| H10a | 0.500000 |
| H10b | 0.500000 |
| C102 | 0.500000 |
| H10c | 0.500000 |
| H10d | 0.500000 |
| H10e | 0.500000 |
| C103 | 0.500000 |
| H10f | 0.500000 |
| H10g | 0.500000 |
| C104 | 0.500000 |
| H10h | 0.500000 |
| H10i | 0.500000 |
| H10j | 0.500000 |
| F81 | 0.500000 |
| F82 | 0.5 |
| F83 | 0.500000 |
| F84 | 0.500000 |
| B81 | 0.5 |

## 9.12 Single crystal structure analysis of 12

CCDC Deposition number 2423160

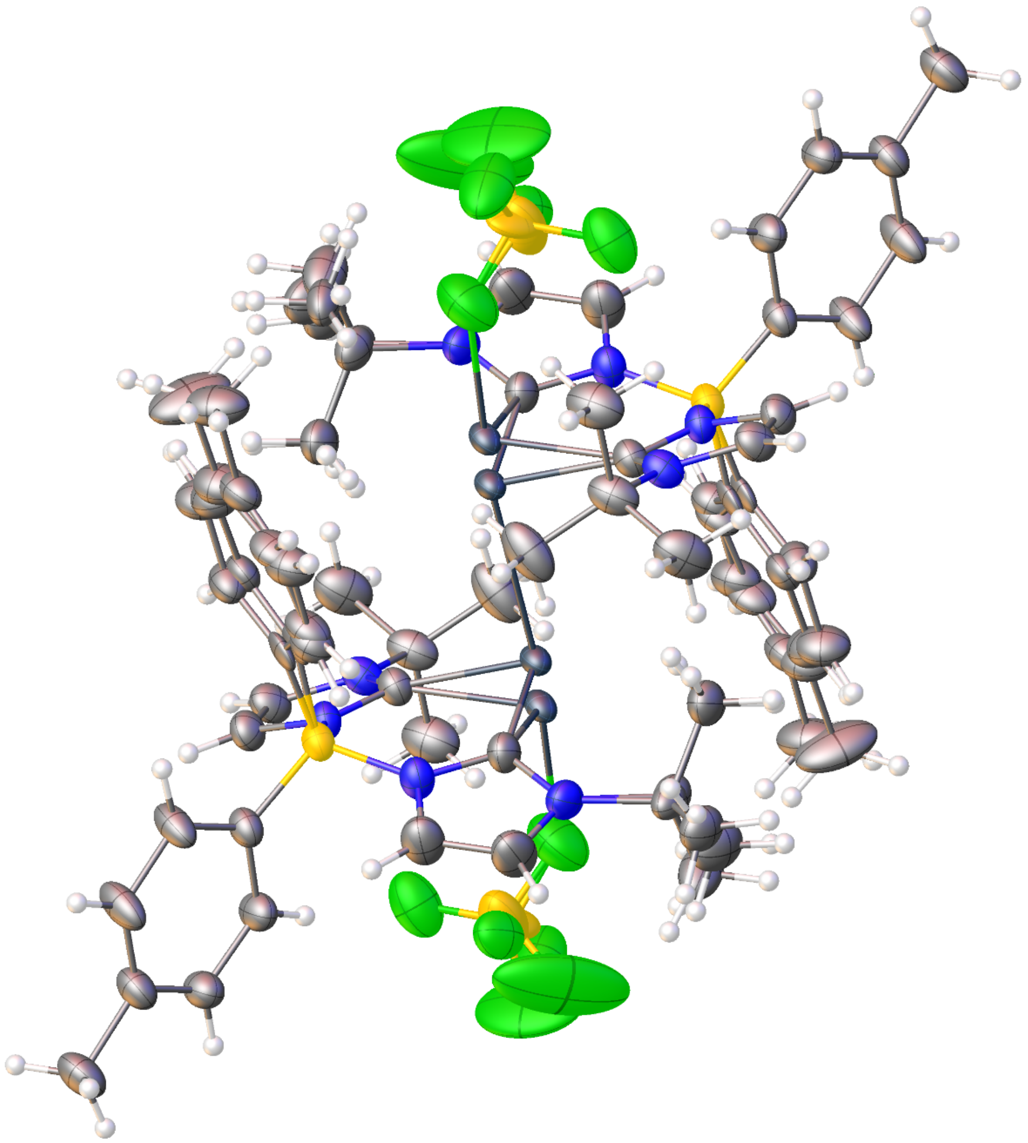

Figure S37. Olex-2 representation of the molecular structure of complex **12**

### 9.12.1 Experimental

Single colourless plate-shaped crystals of **12** were recrystallised by solvent layering of diethyl ether into a saturated solution of CDCl3. A suitable crystal with dimensions 0.15 × 0.11 × 0.02 $mm^3$ was selected and mounted on a MITIGEN holder in oil on a Rigaku FRE+ diffractometer with Arc)Sec VHF Varimax confocal mirrors, a UG2 goniometer and HyPix 6000HE detector diffractometer. The crystal was kept at a steady $T$ = 100(2) K during data collection. The structure was solved with the ShelXT 2018/2 (Sheldrick, 2015) solution program using dual methods and by using Olex2 1.5-dev (Dolomanov et al., 2009) as the graphical interface. The model was refined with olex2.refine 1.5-dev (Bourhis et al., 2015) using full matrix least squares minimisation on $\boldsymbol{F}^2$.

9.12.2 Crystal Data.

$C_{61}H_{79.5}B_4F_8N_{10.5}Sb_2$, $M_r$ = 1398.686, triclinic, *P*-1 (No. 2), a = 12.4854(2) Å, b = 12.5060(3) Å, c = 12.7115(2) Å, $\alpha$ = 91.576(2)°, $\beta$ = 112.299(2)°, $\gamma$ = 113.773(2)°, *V* = 1641.94(7) Å$^3$, *T* = 100(2) K, *Z* = 1, *Z'* = 0.5, $\mu$(Mo $K_\alpha$) = 0.890, 78817 reflections measured, 15888 unique ($R_{int}$ = 0.0490) which were used in all calculations. The final $wR_2$ was 0.1618 (all data) and $R_1$ was 0.0582 (I≥2 $\sigma$(I)).

| Compound | **12** |
|---|---|
| | |
| Formula | $C_{61}H_{79.5}B_4F_8N_{10.5}Sb_2$ |
| $D_{calc.}$/ g cm$^{-3}$ | 1.415 |
| $\mu$/mm$^{-1}$ | 0.890 |
| Formula Weight | 1398.686 |
| Colour | colourless |
| Shape | plate-shaped |
| Size/mm$^3$ | 0.15×0.11×0.02 |
| *T*/K | 100(2) |
| Crystal System | triclinic |
| Space Group | *P*-1 |
| *a*/Å | 12.4854(2) |
| *b*/Å | 12.5060(3) |
| *c*/Å | 12.7115(2) |
| $\alpha$/° | 91.576(2) |
| $\beta$/° | 112.299(2) |
| $\gamma$/° | 113.773(2) |
| V/Å$^3$ | 1641.94(7) |
| *Z* | 1 |
| *Z'* | 0.5 |
| Wavelength/Å | 0.71073 |
| Radiation type | Mo $K_\alpha$ |
| $\Theta_{min}$/° | 1.77 |
| $\Theta_{max}$/° | 36.32 |
| Measured Refl's. | 78817 |
| Indep't Refl's | 15888 |
| Refl's I≥2 $\sigma$(I) | 12377 |
| $R_{int}$ | 0.0490 |
| Parameters | 486 |
| Restraints | 607 |
| Largest Peak | 2.5481 |
| Deepest Hole | -1.2172 |
| GooF | 1.0226 |
| $wR_2$ (all data) | 0.1618 |
| $wR_2$ | 0.1509 |
| $R_1$ (all data) | 0.0770 |
| $R_1$ | 0.0582 |

### 9.12.3 Structure Quality Indicators

| | | | | |
|---|---|---|---|---|
| Reflections: | d min (MoKα) 2Θ=72.6° 0.60 | I/σ(I) 22.7 | Rint m=4.96 4.90% | Full 50.5° 100 |
| Refinement: | Shift 0.001 | Max Peak 2.5 | Min Peak -1.2 | GooF 1.023 |

A colourless plate-shaped crystal with dimensions 0.15 × 0.11 × 0.02 $mm^3$ was mounted on a MITIGEN holder in oil. Data were collected using a Rigaku FRE+ diffractometer with Arc)Sec VHF Varimax confocal mirrors, a UG2 goniometer and HyPix 6000HE detector diffractometer equipped with an Oxford Cryosystems low-temperature device operating at $T$ = 100(2) K.

Data were measured using profile data from $\omega$-scans with Mo $K_{\alpha}$ radiation. The diffraction pattern was indexed and the total number of runs and images was based on the strategy calculation from the program CrysAlisPro system (CCD 43.136a 64-bit (release 23-08-2024)). The maximum resolution that was achieved was $\Theta$ = 36.32° (0.60 Å).

The unit cell was refined using CrysAlisPro 1.171.43.138a (Rigaku OD, 2024) on 26291 reflections, 33% of the observed reflections.

Data reduction, scaling and absorption corrections were performed using CrysAlisPro 1.171.43.138a (Rigaku OD, 2024). The final completeness is 99.97 % out to 36.32° in $\Theta$. A gaussian absorption correction was performed using CrysAlisPro 1.171.43.138a (Rigaku Oxford Diffraction, 2024) Numerical absorption correction based on gaussian integration over a multifaceted crystal model Empirical absorption correction using spherical harmonics, implemented in SCALE3 ABSPACK scaling algorithm. The absorption coefficient $\mu$ of this material is 0.890 $mm^{-1}$ at this wavelength ($\lambda$ = 0.71073Å) and the minimum and maximum transmissions are 0.687 and 1.000.

The structure was solved and the space group $P$-1 (# 2) determined by the ShelXT 2018/2 (Sheldrick, 2015) structure solution program using dual methods and refined by full matrix least squares minimisation on $F^2$ using version of olex2.refine 1.5-dev (Bourhis et al., 2015). All non-hydrogen atoms were refined anisotropically. Hydrogen atom positions were calculated geometrically and refined using the riding model. Hydrogen atom positions were calculated geometrically and refined using the riding model.

*_refine_special_details*: Sb is disordered (ca. 84:16). BF4 is partially disordered (ca. 50:50). One tBu group is disordered (ca. 75:25). One pTolyl group is disordered (ca. 52:48). Standard 1,2 and 1,3 equal distance restraints (SAD) were applied to all chemically equivalent atom pairs of each disorder component. Thermal restraints (RIGU) were applied to all disordered atoms. EADP constraints were applied to C22a and C22b disordered atom pair. Solvent masking was applied to eliminate the electron contribution equivalent to 1.25 molecules of C2D3N per ASU

*_exptl_absorpt_process_details*: CrysAlisPro 1.171.43.138a (Rigaku OD, 2024). Numerical absorption correction based on gaussian integration over a multifaceted crystal model Empirical

absorption correction using spherical harmonics, implemented in SCALE3 ABSPACK scaling algorithm.

The value of Z' is 0.5. This means that only half of the formula unit is present in the asymmetric unit, with the other half consisting of symmetry equivalent atoms. The moiety formula is C56 H72 B4 F8 N8 Sb2, 2.5[C2H3N].

## 9.12.4 Data Plots: Diffraction Data

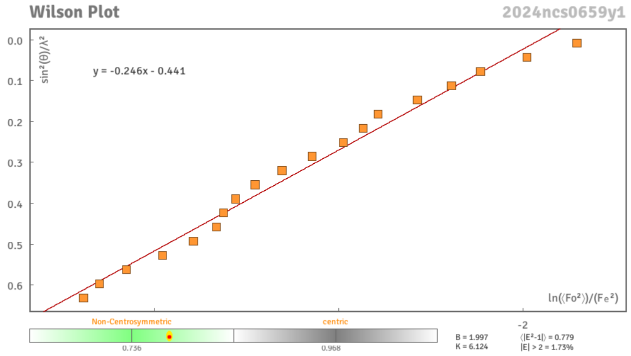


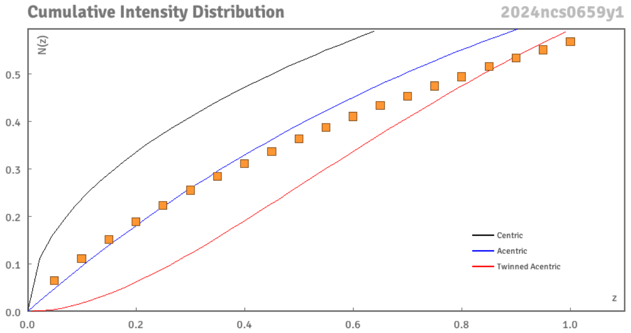


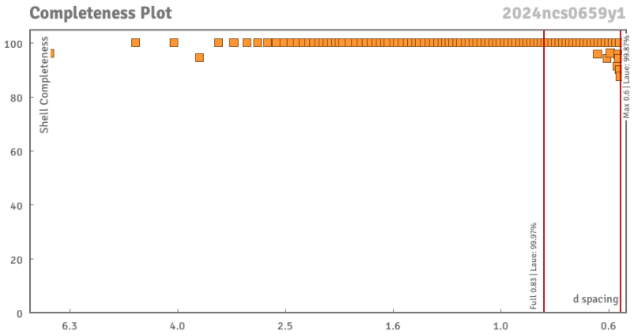


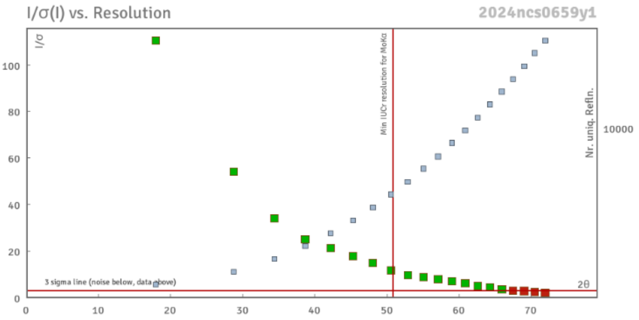


## 9.12.5 Data Plots: Refinement and Data

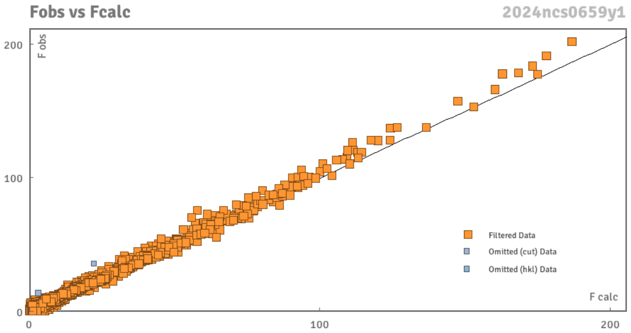


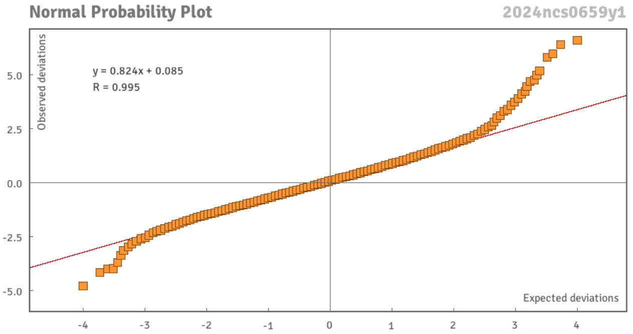

9.12.6 Reflection Statistics

| | | | |
|---|---|---|---|
| Total reflections (after filtering) | 78801 | Unique reflections | 15888 |
| Completeness | 0.999 | Mean I/$\sigma$ | 17.58 |
| $hkl_{max}$ collected | (20, 20, 21) | $hkl_{min}$ collected | (-20, -20, -21) |
| $hkl_{max}$ used | (18, 20, 21) | $hkl_{min}$ used | (-20, -20, 0) |
| Lim $d_{max}$ collected | 100.0 | Lim $d_{min}$ collected | 0.36 |
| $d_{max}$ used | 11.49 | $d_{min}$ used | 0.6 |
| Friedel pairs | 15024 | Friedel pairs merged | 1 |
| Inconsistent equivalents | 17 | $R_{int}$ | 0.049 |
| $R_{sigma}$ | 0.0441 | Intensity transformed | 0 |
| Omitted reflections | 0 | Omitted by user (OMIT hkl) | 16 |
| Multiplicity | (6879, 11164, 6998, 3479, 1200, 496, 305, 157, 119, 68, 26, 12, 6, 2, 2, 1) | Maximum multiplicity | 20 |
| Removed systematic absences | 0 | Filtered off (Shel/OMIT) | 0 |

9.12.7 Fractional Atomic Coordinates and Equivalent Isotropic Displacement Parameters

Table S78. Fractional Atomic Coordinates (×$10^4$) and Equivalent Isotropic Displacement Parameters (Å$^2$×$10^3$) for **12**. $U_{eq}$ is defined as 1/3 of the trace of the orthogonalised $U_{ij}$.

| Atom | x | y | z | $U_{eq}$ |
|---|---|---|---|---|
| Sb1A | 4083.3(2) | 4328.75(15) | 5497.99(16) | 25.07(7) |
| Sb1B | 3619.5(12) | 4139.2(9) | 5712.1(8) | 25.5(3) |
| N1 | 3434(2) | 6466.2(19) | 5724.3(18) | 34.0(4) |
| N2 | 1806(2) | 5094(2) | 4221.1(18) | 39.1(4) |
| N3 | 5317.2(18) | 6516.1(17) | 7544.6(15) | 28.2(3) |
| N4 | 5936(2) | 5184.1(19) | 8260.5(17) | 32.9(4) |
| C1 | 3015(2) | 5394(2) | 5081(2) | 34.2(4) |
| C2 | 2495(3) | 6850(3) | 5259(3) | 48.1(6) |
| C3 | 1495(3) | 5996(3) | 4338(3) | 52.4(7) |
| C7 | 1611(3) | 3537(3) | 2828(2) | 46.3(6) |
| C8 | 5232(2) | 5435(2) | 7263.7(18) | 29.9(4) |
| C9 | 6090(2) | 6954(2) | 8727.1(19) | 34.5(4) |
| C10 | 6468(3) | 6141(3) | 9163(2) | 36.7(5) |
| C11 | 6072(3) | 4064(3) | 8486(2) | 41.1(5) |
| C12 | 5187(4) | 3422(3) | 9048(3) | 54.9(7) |
| C13 | 7486(4) | 4454(4) | 9399(3) | 57.6(8) |
| C14 | 5844(4) | 3325(3) | 7409(3) | 58.4(9) |
| C15 | 4495(2) | 8123(2) | 7539.3(19) | 31.6(4) |
| C16 | 4903(3) | 9342(2) | 7596(2) | 42.9(6) |
| C17 | 4468(4) | 9987(3) | 8121(3) | 50.3(7) |
| C18 | 3667(3) | 9446(2) | 8640(2) | 39.4(5) |
| C19 | 3305(3) | 8246(2) | 8637(2) | 38.7(5) |
| C20 | 3696(3) | 7592(2) | 8090(2) | 37.9(5) |
| C21 | 3180(4) | 10132(3) | 9182(3) | 51.3(7) |
| C4A | 870(4) | 3999(5) | 3286(4) | 34.0(9) |
| C5A | 115(7) | 3099(6) | 3825(4) | 60.5(17) |
| C6A | -30(4) | 4297(5) | 2272(4) | 47.0(12) |
| C22A | 5780(12) | 8014(15) | 6218(12) | 27.7(15) |
| C23A | 5322(16) | 8501(16) | 5266(15) | 38(2) |
| C24A | 6122(13) | 9172(12) | 4783(11) | 46(2) |
| C25A | 7448(13) | 9516(9) | 5301(11) | 42.2(18) |
| C26A | 7924(13) | 9150(14) | 6304(14) | 50(2) |
| C27A | 7112(12) | 8383(15) | 6777(13) | 34.1(17) |
| C28A | 8332(15) | 10242(11) | 4766(15) | 66(3) |
| C4B | 980(16) | 3789(17) | 3461(15) | 41(3) |
| C5B | 547(15) | 2821(13) | 4062(12) | 44(3) |
| C6B | -203(15) | 3850(20) | 2521(15) | 61(4) |
| C22b | 5823(13) | 8190(16) | 6316(14) | 27.7(15) |
| C23b | 5510(20) | 8567(18) | 5252(16) | 38(2) |
| C24b | 6448(16) | 9312(13) | 4918(11) | 42(2) |
| C25b | 7762(16) | 9625(13) | 5593(15) | 49(2) |
| C26b | 8073(15) | 9168(16) | 6551(15) | 49(3) |
| C27b | 7145(15) | 8533(18) | 6940(16) | 40(3) |
| C28b | 8705(17) | 10395(18) | 5135(18) | 90(5) |
| B1 | 4792(3) | 7320(2) | 6769(2) | 29.4(4) |
| B51A | 2004(17) | 3892(18) | 7258(15) | 85(5) |
| B51B | 1983(11) | 3627(13) | 7404(8) | 60(3) |
| F51 | 2389(3) | 3226(3) | 6718(2) | 83.7(8) |
| F52 | 3049(3) | 4596(2) | 8275.4(19) | 72.5(6) |

| | | | | |
|---|---|---|---|---|
| F53A | 1023(11) | 3249(9) | 7640(12) | 153(4) |
| F54A | 1577(4) | 4549(5) | 6597(3) | 58.9(15) |
| F53B | 1695(6) | 2692(6) | 8031(4) | 90(2) |
| F54B | 753(10) | 3391(15) | 6745(7) | 210(6) |

9.12.8 Anisotropic Displacement Parameters

Table S79. Anisotropic Displacement Parameters (×10[4]) for **12**. The anisotropic displacement factor exponent takes the form: $-2\pi^2[h^2a^{*2} \times U_{11} + ... +2hka^* \times b^* \times U_{12}]$

| Atom | $U_{11}$ | $U_{22}$ | $U_{33}$ | $U_{23}$ | $U_{13}$ | $U_{12}$ |
|---|---|---|---|---|---|---|
| Sb1A | 29.28(10) | 22.11(8) | 25.75(8) | 8.38(6) | 17.15(7) | 2.78(5) |
| Sb1B | 33.6(6) | 23.1(4) | 22.9(4) | 10.9(4) | 17.2(3) | 4.4(3) |
| N1 | 34.8(9) | 30.5(9) | 32.0(8) | 15.4(7) | 9.2(7) | 1.3(6) |
| N2 | 28.7(8) | 47.5(12) | 30.8(8) | 12.7(7) | 7.7(7) | -2.3(7) |
| N3 | 31.5(8) | 23.8(8) | 24.8(7) | 9.9(6) | 10.4(6) | 2.2(5) |
| N4 | 41.1(9) | 36.7(10) | 28.8(8) | 22.0(7) | 17.3(7) | 11.6(7) |
| C1 | 31.8(10) | 34.6(11) | 27.2(9) | 11.3(8) | 8.0(7) | -1.1(7) |
| C2 | 44.9(13) | 49.8(15) | 46.8(14) | 29.2(11) | 8.8(11) | 2.7(11) |
| C3 | 38.8(12) | 66.6(19) | 44.9(14) | 30.1(11) | 4.8(10) | -1.5(12) |
| C7 | 37.3(12) | 50.9(16) | 35.6(11) | 12.9(10) | 9.0(8) | -4.8(10) |
| C8 | 34.9(10) | 27.5(9) | 27.6(8) | 14.4(7) | 13.2(7) | 4.7(7) |
| C9 | 38.0(11) | 36.1(11) | 25.1(8) | 13.8(8) | 12.6(8) | 1.9(7) |
| C10 | 40.9(11) | 46.6(13) | 25.2(9) | 22.2(9) | 13.8(8) | 7.7(8) |
| C11 | 58.0(14) | 45.2(13) | 44.1(12) | 34.9(11) | 31.7(10) | 23.9(10) |
| C12 | 74(2) | 52.7(17) | 65.0(18) | 38.1(15) | 44.4(15) | 30.1(14) |
| C13 | 66.8(18) | 78(2) | 48.4(15) | 50.4(16) | 25.3(13) | 24.0(15) |
| C14 | 102(3) | 53.2(17) | 43.3(14) | 54.0(17) | 32.1(14) | 17.9(12) |
| C15 | 40.9(11) | 24.4(9) | 30.4(9) | 13.1(7) | 17.9(8) | 4.4(7) |
| C16 | 72.2(17) | 27.9(10) | 44.2(12) | 21.4(10) | 41.0(12) | 10.5(8) |
| C17 | 88(2) | 29.6(11) | 55.2(14) | 27.9(12) | 50.3(13) | 12.8(10) |
| C18 | 54.3(14) | 33.9(11) | 38.2(11) | 21.4(10) | 26.2(10) | 5.5(8) |
| C19 | 42.3(12) | 33.6(11) | 45.6(12) | 14.7(9) | 26.5(10) | 6.6(9) |
| C20 | 42.7(12) | 25.4(10) | 48.2(13) | 11.2(8) | 26.7(9) | 5.0(8) |
| C21 | 71.8(19) | 48.2(16) | 52.5(15) | 33.9(14) | 37.4(14) | 8.4(12) |
| C4A | 26.0(13) | 40.9(19) | 23.0(14) | 5.3(10) | 9.3(10) | 1.1(10) |
| C5A | 60(3) | 62(3) | 34.1(18) | 0.8(18) | 24.2(16) | 5.6(15) |
| C6A | 33.8(16) | 58(3) | 35.5(18) | 15.7(14) | 6.5(12) | 4.6(14) |
| C22A | 45.3(12) | 15(4) | 26(2) | 13.7(15) | 18.6(11) | -3(2) |
| C23A | 46(4) | 41(4) | 36(3) | 23(3) | 23(2) | 12(2) |
| C24A | 55(4) | 46(4) | 55(4) | 27(3) | 35(3) | 18(2) |
| C25A | 55(4) | 39(3) | 43(3) | 21(3) | 32(3) | 8(2) |
| C26A | 48(4) | 49(4) | 57(5) | 17(2) | 31(3) | 13(3) |
| C27A | 40(3) | 31(3) | 33(4) | 15.2(18) | 19.0(19) | -1(2) |
| C28A | 70(5) | 71(5) | 92(6) | 39(4) | 60(4) | 40(4) |
| C4B | 33(4) | 45(4) | 29(4) | 8.8(16) | 7.9(19) | 2.8(15) |
| C5B | 43(6) | 38(5) | 31(4) | 3(3) | 14(3) | -5(2) |
| C6B | 42(5) | 83(10) | 44(6) | 22(3) | 9(3) | 10(4) |
| C22b | 45.3(12) | 15(4) | 26(2) | 13.7(15) | 18.6(11) | -3(2) |
| C23b | 58(5) | 38(4) | 36(3) | 26(3) | 33(3) | 16(2) |
| C24b | 61(5) | 43(4) | 41(3) | 24(3) | 37(3) | 20(3) |
| C25b | 57(4) | 50(5) | 56(5) | 25(3) | 38(3) | 23(3) |
| C26b | 54(4) | 55(5) | 53(5) | 26(3) | 35(3) | 25(3) |
| C27b | 49(4) | 33(6) | 36(5) | 11(3) | 26(3) | 4(4) |
| C28b | 63(5) | 127(10) | 96(7) | 37(4) | 53(4) | 76(6) |
| B1 | 34.8(10) | 25.2(10) | 26.0(9) | 12.0(7) | 12.7(7) | 3.1(7) |
| B51A | 64(7) | 97(8) | 79(9) | 51(6) | 5(5) | -28(6) |
| B51B | 48(4) | 113(9) | 27(3) | 36(4) | 25(3) | 5(4) |
| F51 | 108(2) | 91(2) | 71.1(15) | 50.0(17) | 51.2(15) | 11.6(14) |
| F52 | 102.9(18) | 70.7(15) | 49.8(11) | 50.8(14) | 25.6(12) | 11.7(10) |

| | | | | | | |
|---|---|---|---|---|---|---|
| F53A | 172(8) | 153(8) | 234(10) | 80(6) | 172(8) | 137(8) |
| F54A | 52(2) | 85(4) | 34.3(19) | 32(2) | 12.2(16) | 2.6(19) |
| F53B | 88(4) | 88(4) | 56(3) | -4(3) | 40(2) | 15(2) |
| F54B | 184(8) | 476(17) | 128(6) | 263(10) | 91(6) | 151(9) |

9.12.9 Bond Lengths

Table S80. Bond Lengths in Å for **12**.

| Atom | Atom | Length/Å |
|---|---|---|
| Sb1A | Sb1A[1] | 2.9799(5) |
| Sb1A | C1 | 2.184(3) |
| Sb1A | C8 | 2.190(2) |
| Sb1B | C1 | 2.060(3) |
| Sb1B | C8 | 2.179(2) |
| Sb1B | F51 | 2.336(3) |
| N1 | C1 | 1.338(3) |
| N1 | C2 | 1.377(3) |
| N1 | B1 | 1.591(3) |
| N2 | C1 | 1.372(3) |
| N2 | C3 | 1.354(4) |
| N2 | C4A | 1.490(5) |
| N2 | C4B | 1.569(18) |
| N3 | C8 | 1.340(3) |
| N3 | C9 | 1.385(3) |
| N3 | B1 | 1.584(3) |
| N4 | C8 | 1.369(3) |
| N4 | C10 | 1.376(3) |
| N4 | C11 | 1.501(3) |
| C2 | C3 | 1.348(4) |
| C7 | C4A | 1.534(6) |
| C7 | C4B | 1.428(18) |
| C9 | C10 | 1.336(4) |
| C11 | C12 | 1.514(4) |
| C11 | C13 | 1.556(4) |
| C11 | C14 | 1.493(4) |
| C15 | C16 | 1.389(3) |
| C15 | C20 | 1.396(3) |
| C15 | B1 | 1.620(3) |
| C16 | C17 | 1.409(4) |
| C17 | C18 | 1.369(4) |
| C18 | C19 | 1.382(4) |
| C18 | C21 | 1.510(4) |
| C19 | C20 | 1.392(4) |
| C4A | C5A | 1.517(6) |
| C4A | C6A | 1.527(6) |
| C22A | C23A | 1.399(18) |
| C22A | C27A | 1.396(15) |
| C22A | B1 | 1.611(12) |
| C23A | C24A | 1.378(12) |
| C24A | C25A | 1.394(11) |
| C25A | C26A | 1.365(12) |
| C25A | C28A | 1.517(12) |
| C26A | C27A | 1.421(16) |
| C4B | C5B | 1.476(19) |
| C4B | C6B | 1.543(18) |
| C22b | C23b | 1.409(19) |
| C22b | C27b | 1.398(17) |
| C22b | B1 | 1.625(14) |
| C23b | C24b | 1.393(13) |
| C24b | C25b | 1.406(13) |
| C25b | C26b | 1.349(12) |
| C25b | C28b | 1.513(14) |
| C26b | C27b | 1.390(16) |
| B51A | F51 | 1.392(16) |
| B51A | F52 | 1.372(17) |
| B51A | F53A | 1.435(19) |
| B51A | F54A | 1.310(19) |
| B51B | F51 | 1.336(9) |
| B51B | F52 | 1.405(13) |
| B51B | F53B | 1.436(13) |
| B51B | F54B | 1.338(13) |

----
[1]1-x,1-y,1-z

9.12.10 Bond Angles

Table S81. Bond Angles in ° for **12**.

| Atom | Atom | Atom | Angle/° |
|---|---|---|---|
| C8 | Sb1A | C1 | 89.09(8) |
| C8 | Sb1B | C1 | 92.70(9) |
| F51 | Sb1B | C1 | 104.64(12) |
| F51 | Sb1B | C8 | 93.52(10) |
| C2 | N1 | C1 | 108.1(2) |
| B1 | N1 | C1 | 131.1(2) |
| B1 | N1 | C2 | 120.5(2) |
| C3 | N2 | C1 | 108.4(2) |
| C4A | N2 | C1 | 131.2(3) |
| C4A | N2 | C3 | 120.3(3) |
| C4B | N2 | C1 | 118.8(7) |
| C4B | N2 | C3 | 131.9(7) |
| C4B | N2 | C4A | 14.0(6) |
| C9 | N3 | C8 | 108.15(19) |
| B1 | N3 | C8 | 131.53(18) |
| B1 | N3 | C9 | 120.16(19) |
| C10 | N4 | C8 | 108.2(2) |
| C11 | N4 | C8 | 130.8(2) |
| C11 | N4 | C10 | 120.7(2) |
| Sb1B | C1 | Sb1A | 18.45(4) |
| N1 | C1 | Sb1A | 123.31(16) |
| N1 | C1 | Sb1B | 123.44(17) |
| N2 | C1 | Sb1A | 128.84(18) |
| N2 | C1 | Sb1B | 122.89(19) |
| N2 | C1 | N1 | 107.7(2) |
| C3 | C2 | N1 | 108.1(3) |
| C2 | C3 | N2 | 107.7(2) |
| C4B | C7 | C4A | 14.2 |
| Sb1B | C8 | Sb1A | 18.21(4) |
| N3 | C8 | Sb1A | 123.07(15) |
| N3 | C8 | Sb1B | 120.92(16) |
| N4 | C8 | Sb1A | 129.21(17) |
| N4 | C8 | Sb1B | 126.52(17) |
| N4 | C8 | N3 | 107.69(19) |
| C10 | C9 | N3 | 108.4(2) |
| C9 | C10 | N4 | 107.6(2) |
| C12 | C11 | N4 | 107.4(2) |
| C13 | C11 | N4 | 107.1(3) |
| C13 | C11 | C12 | 107.3(3) |
| C14 | C11 | N4 | 111.8(2) |
| C14 | C11 | C12 | 114.4(3) |
| C14 | C11 | C13 | 108.5(3) |
| C20 | C15 | C16 | 116.2(2) |
| B1 | C15 | C16 | 122.6(2) |
| B1 | C15 | C20 | 120.9(2) |
| C17 | C16 | C15 | 121.6(2) |
| C18 | C17 | C16 | 121.3(3) |
| C19 | C18 | C17 | 117.5(2) |
| C21 | C18 | C17 | 121.3(3) |
| C21 | C18 | C19 | 121.2(3) |
| C20 | C19 | C18 | 121.7(2) |
| C19 | C20 | C15 | 121.6(2) |
| C7 | C4A | N2 | 109.4(3) |
| C5A | C4A | N2 | 106.6(4) |
| C5A | C4A | C7 | 112.2(4) |
| C6A | C4A | N2 | 109.9(4) |
| C6A | C4A | C7 | 107.8(3) |
| C6A | C4A | C5A | 110.9(4) |
| C27A | C22A | C23A | 117.8(13) |
| B1 | C22A | C23A | 116.9(11) |
| B1 | C22A | C27A | 123.6(12) |
| C24A | C23A | C22A | 121.0(12) |
| C25A | C24A | C23A | 121.6(10) |
| C26A | C25A | C24A | 117.3(8) |
| C28A | C25A | C24A | 121.8(8) |
| C28A | C25A | C26A | 120.9(9) |
| C27A | C26A | C25A | 122.5(10) |
| C26A | C27A | C22A | 119.1(14) |
| C7 | C4B | N2 | 110.8(11) |
| C5B | C4B | N2 | 116.7(12) |
| C5B | C4B | C7 | 111.0(13) |
| C6B | C4B | N2 | 103.1(13) |
| C6B | C4B | C7 | 104.3(12) |
| C6B | C4B | C5B | 109.9(14) |
| C27b | C22b | C23b | 113.6(14) |
| B1 | C22b | C23b | 125.8(12) |
| B1 | C22b | C27b | 120.3(14) |
| C24b | C23b | C22b | 122.7(15) |
| C25b | C24b | C23b | 120.2(10) |
| C26b | C25b | C24b | 118.0(10) |
| C28b | C25b | C24b | 116.5(9) |
| C28b | C25b | C26b | 125.4(10) |
| C27b | C26b | C25b | 120.8(13) |
| C26b | C27b | C22b | 123.8(16) |
| N3 | B1 | N1 | 108.41(18) |
| C15 | B1 | N1 | 105.97(19) |
| C15 | B1 | N3 | 108.33(17) |
| C22A | B1 | N1 | 107.8(5) |
| C22A | B1 | N3 | 108.6(6) |
| C22A | B1 | C15 | 117.4(6) |
| C22b | B1 | N1 | 112.3(6) |
| C22b | B1 | N3 | 112.1(7) |
| C22b | B1 | C15 | 109.5(7) |
| C22b | B1 | C22A | 8.0(11) |
| F52 | B51A | F51 | 107.5(11) |
| F53A | B51A | F51 | 117.4(15) |
| F53A | B51A | F52 | 103.0(13) |
| F54A | B51A | F51 | 112.3(13) |
| F54A | B51A | F52 | 110.7(14) |
| F54A | B51A | F53A | 105.6(12) |
| F52 | B51B | F51 | 108.8(7) |
| F53B | B51B | F51 | 102.4(10) |
| F53B | B51B | F52 | 102.9(7) |
| F54B | B51B | F51 | 106.8(8) |
| F54B | B51B | F52 | 134.4(12) |

| | | | |
|---|---|---|---|
| F54B | B51B | F53B | 96.2(9) |
| B51A | F51 | Sb1B | 119.2(9) |
| B51B | F51 | Sb1B | 133.8(6) |
| B51B | F51 | B51A | 15.9(11) |
| B51B | F52 | B51A | 15.7(11) |

9.12.11 Torsion Angles

Table S82. Torsion Angles in ° for **12**.

| Atom | Atom | Atom | Atom | Angle/° |
|---|---|---|---|---|
| Sb1A | C1 | N1 | C2 | -176.3(2) |
| Sb1A | C1 | N1 | B1 | 10.1(3) |
| Sb1A | C1 | N2 | C3 | 175.6(3) |
| Sb1A | C1 | N2 | C4A | -2.7(4) |
| Sb1A | C1 | N2 | C4B | 5.3(8) |
| Sb1A | C8 | N3 | C9 | 178.67(19) |
| Sb1A | C8 | N3 | B1 | -6.1(3) |
| Sb1A | C8 | N4 | C10 | -178.6(2) |
| Sb1A | C8 | N4 | C11 | -4.9(3) |
| Sb1B | C1 | N1 | C2 | -154.2(2) |
| Sb1B | C1 | N1 | B1 | 32.3(3) |
| Sb1B | C1 | N2 | C3 | 154.0(2) |
| Sb1B | C1 | N2 | C4A | -24.3(3) |
| Sb1B | C1 | N2 | C4B | -16.4(8) |
| Sb1B | C8 | N3 | C9 | 157.31(18) |
| Sb1B | C8 | N3 | B1 | -27.4(2) |
| Sb1B | C8 | N4 | C10 | -155.7(2) |
| Sb1B | C8 | N4 | C11 | 17.9(3) |
| Sb1B | F51 | B51A | F52 | -78.1(7) |
| Sb1B | F51 | B51A | F53A | 166.5(10) |
| Sb1B | F51 | B51A | F54A | 43.9(5) |
| Sb1B | F51 | B51B | F52 | -51.6(5) |
| Sb1B | F51 | B51B | F53B | -160.1(9) |
| Sb1B | F51 | B51B | F54B | 99.4(10) |
| N1 | C1 | N2 | C3 | 0.5(3) |
| N1 | C1 | N2 | C4A | -177.8(3) |
| N1 | C1 | N2 | C4B | -169.8(7) |
| N1 | C2 | C3 | N2 | -0.6(3) |
| N1 | B1 | N3 | C8 | 35.6(3) |
| N1 | B1 | N3 | C9 | -149.64(18) |
| N1 | B1 | C15 | C16 | -110.8(2) |
| N1 | B1 | C15 | C20 | 62.5(2) |
| N1 | B1 | C22A | C23A | 45.5(10) |
| N1 | B1 | C22A | C27A | -149.4(9) |
| N1 | B1 | C22b | C23b | 25.7(11) |
| N1 | B1 | C22b | C27b | -147.5(11) |
| N2 | C4A | C7 | C4B | 87(3) |
| N2 | C4B | C7 | C4A | -73(2) |
| N3 | C8 | N4 | C10 | -0.5(2) |
| N3 | C8 | N4 | C11 | 173.09(18) |
| N3 | C9 | C10 | N4 | -0.0(2) |
| N3 | B1 | C15 | C16 | 133.0(2) |
| N3 | B1 | C15 | C20 | -53.6(2) |
| N3 | B1 | C22A | C23A | 162.7(11) |
| N3 | B1 | C22A | C27A | -32.1(8) |
| N3 | B1 | C22b | C23b | 148.0(12) |
| N3 | B1 | C22b | C27b | -25.2(9) |
| C15 | C16 | C17 | C18 | 2.8(4) |
| C15 | C20 | C19 | C18 | 1.3(3) |
| C15 | B1 | C22A | C23A | -74.0(11) |
| C15 | B1 | C22A | C27A | 91.1(9) |
| C15 | B1 | C22b | C23b | -91.8(12) |

| | | | | |
|---|---|---|---|---|
| C15 | B1 | C22b | C27b | 95.0(9) |
| C16 | C17 | C18 | C19 | 0.3(4) |
| C16 | C17 | C18 | C21 | -178.8(3) |
| C17 | C18 | C19 | C20 | -2.3(4) |
| C22A | C23A | C24A | C25A | -8(2) |
| C22A | C27A | C26A | C25A | -2.0(15) |
| C22A | B1 | C22b | C23b | 82(8) |
| C22A | B1 | C22b | C27b | -91(8) |
| C23A | C24A | C25A | C26A | 0.7(14) |
| C23A | C24A | C25A | C28A | 179.2(12) |
| C24A | C25A | C26A | C27A | 4.2(11) |
| C22b | C23b | C24b | C25b | 6(2) |
| C22b | C27b | C26b | C25b | 8(2) |
| C23b | C24b | C25b | C26b | 1.0(15) |
| C23b | C24b | C25b | C28b | 177.5(15) |
| C24b | C25b | C26b | C27b | -8.1(14) |
| B51A | F51 | B51B | F52 | -77(4) |
| B51A | F51 | B51B | F53B | 175(4) |
| B51A | F51 | B51B | F54B | 74(4) |
| B51A | F52 | B51B | F51 | 86(4) |
| B51A | F52 | B51B | F53B | -166(3) |
| B51A | F52 | B51B | F54B | -53(4) |

9.12.12 Hydrogen Fractional Atomic Coordinates and Equivalent Isotropic Displacement Parameters

Table S83. Hydrogen Fractional Atomic Coordinates (×10[4]) and Equivalent Isotropic Displacement Parameters (Å[2]×10[3]) for **12**. $U_{eq}$ is defined as 1/3 of the trace of the orthogonalised $U_{ij}$.

| Atom | x | y | z | $U_{eq}$ |
|---|---|---|---|---|
| H2 | 2544(3) | 7589(3) | 5538(3) | 57.7(8) |
| H3 | 713(3) | 6022(3) | 3858(3) | 62.9(9) |
| H7bd | 1800(100) | 4150(60) | 2370(70) | 69.4(9) |
| H7be | 2420(50) | 3540(100) | 3373(5) | 69.4(9) |
| H7bf | 1040(40) | 2750(40) | 2310(70) | 69.4(9) |
| H7aa | 2270(20) | 4206(6) | 2690(30) | 69.4(9) |
| H7ab | 2040(30) | 3160(30) | 3402(14) | 69.4(9) |
| H7ac | 1006(6) | 2950(20) | 2096(16) | 69.4(9) |
| H9 | 6314(2) | 7706(2) | 9155.3(19) | 41.4(5) |
| H10 | 7006(3) | 6210(3) | 9953(2) | 44.1(6) |
| H12a | 5381(19) | 3970(8) | 9734(15) | 82.3(11) |
| H12b | 4284(4) | 3150(20) | 8490(9) | 82.3(11) |
| H12c | 5320(20) | 2731(16) | 9280(20) | 82.3(11) |
| H13a | 8087(4) | 4870(20) | 9062(9) | 86.4(12) |
| H13b | 7670(10) | 4990(20) | 10089(11) | 86.4(12) |
| H13c | 7591(9) | 3746(4) | 9620(20) | 86.4(12) |
| H14a | 4939(8) | 3030(20) | 6845(10) | 87.6(13) |
| H14b | 6410(20) | 3816(8) | 7070(14) | 87.6(13) |
| H14c | 6030(30) | 2648(15) | 7605(5) | 87.6(13) |
| H16 | 5488(3) | 9752(2) | 7274(2) | 51.5(7) |
| H17 | 4737(4) | 10813(3) | 8114(3) | 60.3(8) |
| H19 | 2776(3) | 7857(2) | 9017(2) | 46.5(6) |
| H20 | 3413(3) | 6764(2) | 8092(2) | 45.4(6) |
| H21a | 2570(20) | 9584(5) | 9450(20) | 77.0(10) |
| H21b | 3912(5) | 10757(15) | 9845(14) | 77.0(10) |
| H21c | 2750(20) | 10500(20) | 8603(7) | 77.0(10) |
| H5Aa | 696(12) | 2850(30) | 4420(30) | 91(3) |
| H5Ab | -260(40) | 3465(14) | 4190(30) | 91(3) |
| H5Ac | -580(30) | 2397(18) | 3219(8) | 91(3) |
| H6Aa | -600(20) | 4490(30) | 2515(10) | 70.5(18) |
| H6Ab | 485(4) | 4980(20) | 2030(20) | 70.5(18) |
| H6Ac | -560(20) | 3606(12) | 1616(11) | 70.5(18) |
| H23A | 4445(16) | 8366(16) | 4948(15) | 46(3) |
| H24A | 5762(13) | 9405(12) | 4079(11) | 56(3) |
| H26A | 8832(13) | 9419(14) | 6702(14) | 60(3) |
| H27A | 7470(12) | 8124(15) | 7463(13) | 41(2) |
| H28a | 8490(60) | 9707(14) | 4330(60) | 99(4) |
| H28b | 7920(40) | 10660(60) | 4240(50) | 99(4) |
| H28c | 9160(30) | 10830(50) | 5383(16) | 99(4) |
| H5Ba | 117(15) | 3015(13) | 4490(12) | 66(5) |
| H5Bb | -61(15) | 2069(13) | 3488(12) | 66(5) |
| H5Bc | 1297(15) | 2737(13) | 4608(12) | 66(5) |
| H6Ba | -695(15) | 4030(20) | 2889(15) | 92(6) |
| H6Bb | 90(15) | 4490(20) | 2117(15) | 92(6) |
| H6Bc | -757(15) | 3090(20) | 1960(15) | 92(6) |
| H23b | 4620(20) | 8301(18) | 4742(16) | 46(3) |
| H24b | 6199(16) | 9611(13) | 4231(11) | 51(3) |
| H26b | 8937(15) | 9280(16) | 6965(15) | 59(3) |

| | | | | |
|---|---|---|---|---|
| H27b | 7426(15) | 8321(18) | 7673(16) | 48(3) |
| H28d | 8870(80) | 9883(19) | 4680(80) | 135(7) |
| H28e | 8340(50) | 10860(80) | 4640(80) | 135(7) |
| H28f | 9520(40) | 10940(70) | 5790(18) | 135(7) |

9.12.13 Atomic Occupancies for all atoms that are not fully occupied

Table S84. Atomic Occupancies for all atoms that are not fully occupied in **12**.

| Atom | Occupancy |
|---|---|
| Sb1A | 0.8421(11) |
| Sb1B | 0.1579(11) |
| H7bd | 0.246(15) |
| H7be | 0.246(15) |
| H7bf | 0.246(15) |
| H7aa | 0.754(15) |
| H7ab | 0.754(15) |
| H7ac | 0.754(15) |
| C4A | 0.754(15) |
| C5A | 0.754(15) |
| H5Aa | 0.754(15) |
| H5Ab | 0.754(15) |
| H5Ac | 0.754(15) |
| C6A | 0.754(15) |
| H6Aa | 0.754(15) |
| H6Ab | 0.754(15) |
| H6Ac | 0.754(15) |
| C22A | 0.53(3) |
| C23A | 0.53(3) |
| H23A | 0.53(3) |
| C24A | 0.53(3) |
| H24A | 0.53(3) |
| C25A | 0.53(3) |
| C26A | 0.53(3) |
| H26A | 0.53(3) |
| C27A | 0.53(3) |
| H27A | 0.53(3) |
| C28A | 0.53(3) |
| H28a | 0.53(3) |
| H28b | 0.53(3) |
| H28c | 0.53(3) |
| C4B | 0.246(15) |
| C5B | 0.246(15) |
| H5Ba | 0.246(15) |
| H5Bb | 0.246(15) |
| H5Bc | 0.246(15) |
| C6B | 0.246(15) |
| H6Ba | 0.246(15) |
| H6Bb | 0.246(15) |
| H6Bc | 0.246(15) |
| C22b | 0.47(3) |
| C23b | 0.47(3) |
| H23b | 0.47(3) |
| C24b | 0.47(3) |
| H24b | 0.47(3) |
| C25b | 0.47(3) |
| C26b | 0.47(3) |
| H26b | 0.47(3) |
| C27b | 0.47(3) |
| H27b | 0.47(3) |
| C28b | 0.47(3) |
| H28d | 0.47(3) |
| H28e | 0.47(3) |
| H28f | 0.47(3) |
| B51A | 0.465(7) |
| B51B | 0.535(7) |
| F53A | 0.465(7) |
| F54A | 0.465(7) |
| F53B | 0.535(7) |
| F54B | 0.535(7) |

### 9.12.14 Solvent masking (PLATON/SQUEEZE) information

Table S85. Solvent masking (PLATON/SQUEEZE) information for **12**.

| No | x | y | z | V | e | Content |
|---|---|---|---|---|---|---|
| 1 | 0.000 | -0.335 | 0.000 | 277.9 | 53.5 | 2.5C2H3N |

## 9.13 Single crystal structure analysis of 16d

CCDC Deposition number 2423161

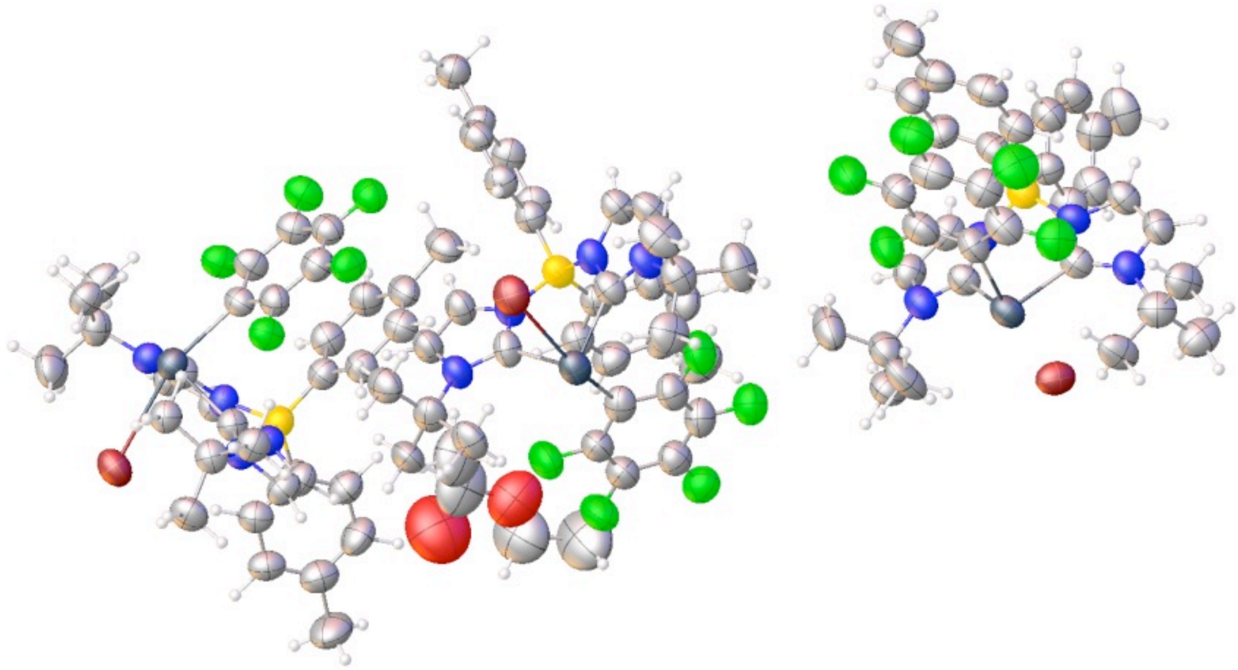

Figure S38. Olex-2 representation of the molecular structure of complex **16d**

### 9.13.1 Experimental.

Single colourless lath-shaped crystals of **16d** recrystallised from ethyl acetate. A suitable crystal with dimensions 0.12 × 0.03 × 0.01 $mm^3$ was selected and mounted on a MITIGEN holder in oil on a Rigaku 007HF diffractometer with HF Varimax confocal mirrors, an UG2 goniometer and HyPix Arc-100 detector diffractometer. The crystal was kept at a steady $T$ = 100(2) K during data collection. The structure was solved with the ShelXT 2018/2 (Sheldrick, 2015) solution program using dual methods and by using Olex2 1.5-dev (Dolomanov et al., 2009) as the graphical interface. The model was refined with olex2.refine 1.5-dev (Bourhis et al., 2015) using full matrix least squares minimisation on $\boldsymbol{F}^2$.

### 9.13.2 Crystal Data

$C_{35.33}H_{38.67}BBrF_5N_4O_{0.67}Sb$, $M_r$ = 837.540, triclinic, *P*-1 (No. 2), a = 13.4299(4) Å, b = 20.3137(7) Å, c = 20.8491(4) Å, $\alpha$ = 87.097(2)°, $\beta$ = 83.235(2)°, $\gamma$ = 75.270(3)°, *V* = 5461.4(3) Å$^3$, *T* = 100(2) K, *Z* = 6, *Z'* = 3, $\mu$(Cu $K_\alpha$) = 7.779, 74640 reflections measured, 19656 unique ($R_{int}$ = 0.0907) which were used in all calculations. The final $wR_2$ was 0.2379 (all data) and $R_1$ was 0.0772 (I≥2 $\sigma$(I)).

| **Compound** | **16d** |
|---|---|
| | |
| Formula | $C_{35.33}H_{38.67}BBrF_5N_4O_{0.67}Sb$ |
| $D_{calc.}$/ g cm$^{-3}$ | 1.528 |
| $\mu$/mm$^{-1}$ | 7.779 |
| Formula Weight | 837.540 |
| Colour | colourless |
| Shape | lath-shaped |
| Size/mm$^3$ | 0.12×0.03×0.01 |
| *T*/K | 100(2) |
| Crystal System | triclinic |
| Space Group | *P*-1 |
| *a*/Å | 13.4299(4) |
| *b*/Å | 20.3137(7) |
| *c*/Å | 20.8491(4) |
| $\alpha$/° | 87.097(2) |
| $\beta$/° | 83.235(2) |
| $\gamma$/° | 75.270(3) |
| V/Å$^3$ | 5461.4(3) |
| *Z* | 6 |
| *Z'* | 3 |
| Wavelength/Å | 1.54184 |
| Radiation type | Cu $K_\alpha$ |
| $\Theta_{min}$/° | 3.07 |
| $\Theta_{max}$/° | 70.69 |
| Measured Refl's. | 74640 |
| Indep't Refl's | 19656 |
| Refl's I≥2 $\sigma$(I) | 12193 |
| $R_{int}$ | 0.0907 |
| Parameters | 1323 |
| Restraints | 30 |
| Largest Peak | 3.2166 |
| Deepest Hole | -0.9289 |
| GooF | 1.0139 |
| $wR_2$ (all data) | 0.2379 |
| $wR_2$ | 0.2008 |
| $R_1$ (all data) | 0.1187 |
| $R_1$ | 0.0772 |

### 9.13.3 Structure Quality Indicators

| | | | | |
|---|---|---|---|---|
| Reflections: | d min (CuKα) 2Θ=141.4° 0.82 | I/σ(I) 13.3 | Rint m=3.80 9.07% | Full 135.4° 94% to 141.4° 96.4 |
| Refinement: | Shift -0.001 | Max Peak 3.2 | Min Peak -0.9 | GooF 1.014 |

A colourless lath-shaped crystal with dimensions 0.12 × 0.03 × 0.01 $mm^3$ was mounted on a MITIGEN holder in oil. Data were collected using a Rigaku 007HF diffractometer with HF Varimax confocal mirrors, an UG2 goniometer and HyPix Arc-100 detector diffractometer equipped with an Oxford Cryosystems low-temperature device operating at $T$ = 100(2) K.

Data were measured using profile data from $\omega$-scans with Cu $K_\alpha$ radiation. The diffraction pattern was indexed and the total number of runs and images was based on the strategy calculation from the program CrysAlisPro system (CCD 43.143a 64-bit (release 25-10-2024)). The maximum resolution that was achieved was $\Theta$ = 70.69° (0.82 Å).

The unit cell was refined using CrysAlisPro 1.171.43.143a (Rigaku OD, 2024) on 18183 reflections, 24% of the observed reflections.

Data reduction, scaling and absorption corrections were performed using CrysAlisPro 1.171.43.143a (Rigaku OD, 2024). The final completeness is 96.37 % out to 70.69° in $\Theta$. A sphere absorption correction was performed using CrysAlisPro 1.171.43.143a (Rigaku Oxford Diffraction, 2024) Spherical absorption correction using equivalent radius and absorption coefficient. Empirical absorption correction using spherical harmonics, implemented in SCALE3 ABSPACK scaling algorithm. The absorption coefficient $\mu$ of this material is 7.779 $mm^{-1}$ at this wavelength ($\lambda$ = 1.54184Å) and the minimum and maximum transmissions are 0.024 and 0.106.

The structure was solved and the space group *P*-1 (# 2) determined by the ShelXT 2018/2 (Sheldrick, 2015) structure solution program using dual methods and refined by full matrix least squares minimisation on $F^2$ using version of olex2.refine 1.5-dev (Bourhis et al., 2015). All non-hydrogen atoms were refined anisotropically. Hydrogen atom positions were calculated geometrically and refined using the riding model.

*_refine_special_details*: Standard temperature restraints (RIGU) applied to solvent ethyl acetate

*_olex2_refine_details*: Refinement using NoSpherA2, an implementation of NOn-SPHERical Atom-form-factors in Olex2.Please cite: F. Kleemiss et al. Chem. Sci. DOI 10.1039/D0SC05526C – 2021 NoSpherA2 implementation of HAR makes use of tailor-made aspherical atomic form factors calculated on-the-fly from a Hirshfeld-partitioned electron density (ED) - not from spherical-atom form factors. The ED is calculated from a gaussian basis set single determinant SCF wavefunction - either Hartree-Fock or DFT using selected functionals- for a fragment of the crystal. This fragment can be embedded in an electrostatic crystal field by employing cluster charges or modelled using implicit solvation models, depending on the software used. The following options were used: SOFTWARE: ORCA 6.0 ::: PARTITIONING: NoSpherA2 ::: INT ACCURACY: Low ::: METHOD: r2SCAN ::: BASIS SET: jorge-DZP-DKH ::: CHARGE: 0 ::: MULTIPLICITY: 1 ::: RELATIVISTIC: ZORA ::: DATE: 2025-02-05_14-05-48

*_exptl_absorpt_process_details*: CrysAlisPro 1.171.43.143a (Rigaku Oxford Diffraction, 2024) Spherical absorption correction using equivalent radius and absorption coefficient. Empirical absorption correction using spherical harmonics, implemented in SCALE3 ABSPACK scaling algorithm.

The value of Z' is 3. The moiety formula is 0.333(C34 H36 B F5 N4 Sb), 0.333(Br), 0.667(C34 H36 B Br F5 N4 Sb), 0.333(C4 H8 O2).

## 9.13.4 Data Plots: Diffraction Data

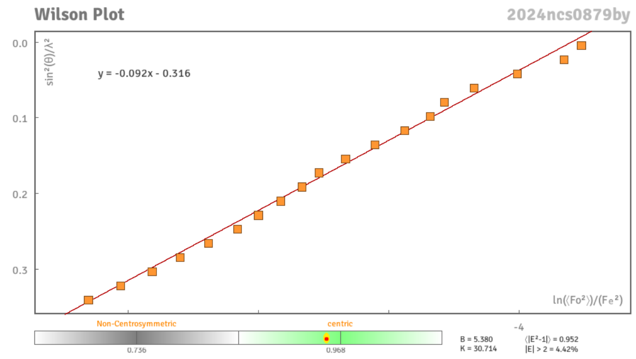


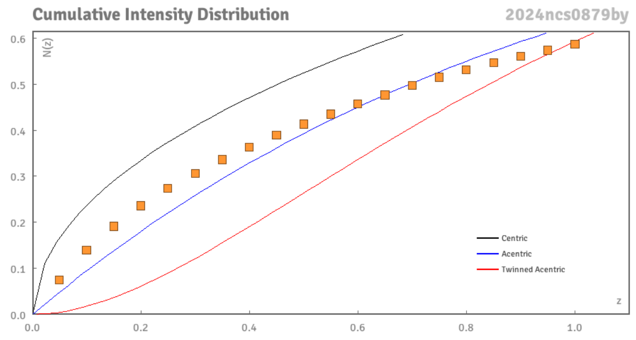


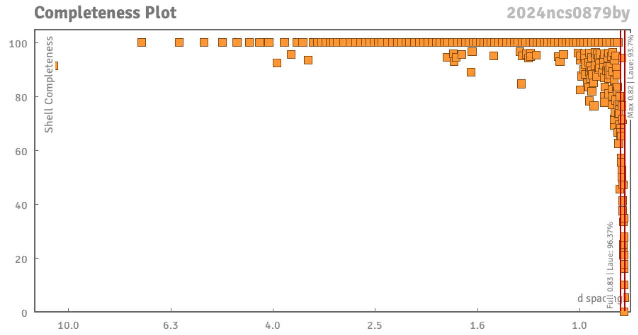


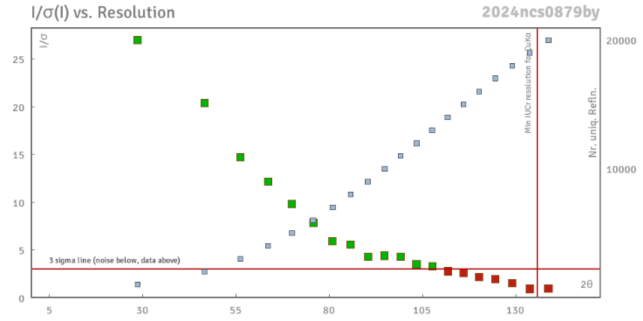


## 9.13.5 Data Plots: Refinement and Data

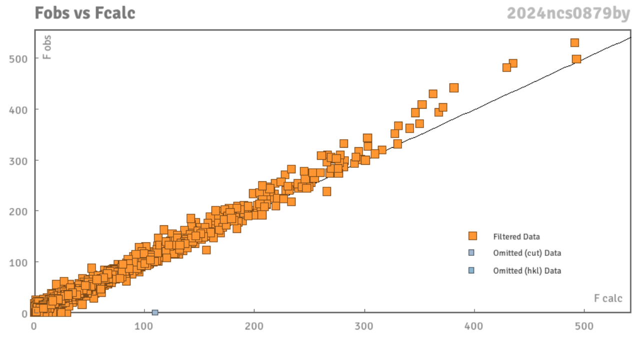


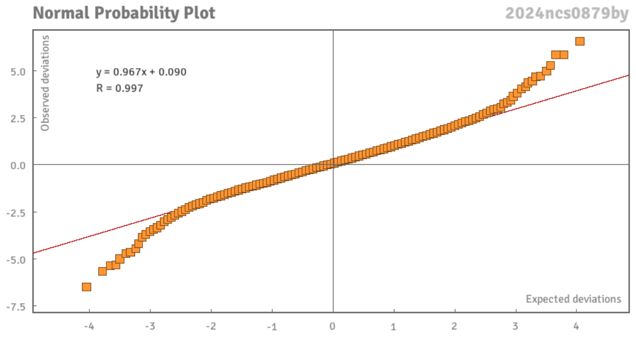

### 9.13.6 Reflection Statistics

| | | | |
|---|---|---|---|
| Total reflections (after filtering) | 74639 | Unique reflections | 19656 |
| Completeness | 0.937 | Mean I/$\sigma$ | 7.11 |
| $hkl_{max}$ collected | (16, 23, 15) | $hkl_{min}$ collected | (-16, -24, -25) |
| $hkl_{max}$ used | (16, 24, 25) | $hkl_{min}$ used | (-16, -24, 0) |
| Lim $d_{max}$ collected | 100.0 | Lim $d_{min}$ collected | 0.77 |
| $d_{max}$ used | 14.4 | $d_{min}$ used | 0.82 |
| Friedel pairs | 5560 | Friedel pairs merged | 1 |
| Inconsistent equivalents | 49 | $R_{int}$ | 0.0907 |
| $R_{sigma}$ | 0.075 | Intensity transformed | 0 |
| Omitted reflections | 0 | Omitted by user (OMIT hkl) | 1 |
| Multiplicity | (10097, 6095, 2585, 1714, 1232, 987, 691, 499, 300, 237, 171, 99, 88, 50, 51, 44, 42, 55, 39, 65, 41, 32, 3) | Maximum multiplicity | 24 |
| Removed systematic absences | 0 | Filtered off (Shel/OMIT) | 0 |

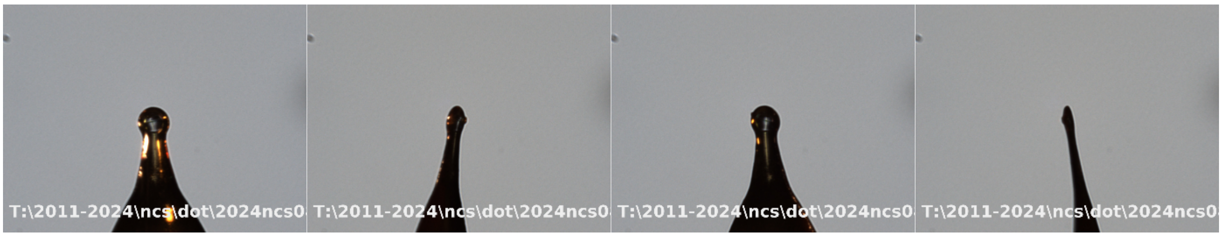

9.13.7 Fractional Atomic Coordinates and Equivalent Isotropic Displacement Parameters

Table S86. Fractional Atomic Coordinates (×10^4) and Equivalent Isotropic Displacement Parameters (Å^2×10^3) for **16d**. $U_{eq}$ is defined as 1/3 of the trace of the orthogonalised $U_{ij}$.

| Atom | x | y | z | $U_{eq}$ |
|---|---|---|---|---|
| Sb31 | 8030.6(5) | 1154.1(3) | 7178.3(3) | 70.7(2) |
| Br31 | 6955.5(8) | 2002.6(6) | 8359.5(5) | 82.2(3) |
| F301 | 7244(5) | 1004(3) | 5717(2) | 97.3(19) |
| F302 | 7951(6) | 149(3) | 4745(3) | 109(2) |
| F303 | 9631(6) | -916(3) | 4850(3) | 107(2) |
| F304 | 10545(6) | -1126(4) | 5946(3) | 119(2) |
| F305 | 9855(5) | -260(3) | 6928(3) | 97.4(18) |
| N301 | 5866(6) | 856(4) | 7292(3) | 65.8(18) |
| N302 | 5747(7) | 1859(4) | 6818(3) | 74(2) |
| N303 | 7192(6) | 19(4) | 7933(3) | 66.2(18) |
| N304 | 8604(6) | 84(4) | 8331(3) | 69.4(19) |
| C301 | 6396(7) | 1298(5) | 7057(4) | 65(2) |
| C302 | 4852(7) | 1143(6) | 7185(4) | 74(2) |
| C303 | 4763(8) | 1759(6) | 6889(4) | 76(3) |
| C304 | 5933(10) | 2498(6) | 6495(5) | 89(3) |
| C305 | 7037(10) | 2532(6) | 6489(7) | 112(4) |
| C306 | 5659(12) | 2514(6) | 5805(4) | 110(4) |
| C307 | 5223(10) | 3093(6) | 6896(5) | 94(3) |
| C308 | 7973(7) | 344(5) | 7874(4) | 67(2) |
| C309 | 7372(7) | -450(5) | 8424(4) | 71(2) |
| C310 | 8230(9) | -418(5) | 8677(4) | 79(3) |
| C311 | 9542(8) | 267(6) | 8533(5) | 84(3) |
| C312 | 9272(10) | 500(7) | 9236(5) | 102(4) |
| C313 | 9801(8) | 848(6) | 8121(5) | 88(3) |
| C314 | 10436(8) | -353(6) | 8468(5) | 88(3) |
| C315 | 6559(8) | -383(5) | 6894(4) | 73(2) |
| C316 | 5933(9) | -260(5) | 6396(4) | 82(3) |
| C317 | 6202(10) | -632(6) | 5823(4) | 89(3) |
| C318 | 7090(10) | -1143(6) | 5748(5) | 88(3) |
| C319 | 7702(9) | -1292(5) | 6246(5) | 87(3) |
| C320 | 7429(8) | -913(5) | 6817(4) | 74(2) |
| C321 | 7406(12) | -1540(6) | 5125(5) | 109(4) |
| C322 | 5308(8) | -64(5) | 8037(4) | 71(2) |
| C323 | 4906(9) | -612(6) | 7995(5) | 83(3) |
| C324 | 4142(9) | -755(6) | 8478(5) | 88(3) |
| C325 | 3757(9) | -327(7) | 8989(5) | 88(3) |
| C326 | 4137(9) | 243(6) | 9034(5) | 88(3) |
| C327 | 4891(8) | 367(5) | 8571(4) | 78(3) |
| C328 | 2934(10) | -474(8) | 9511(5) | 114(4) |
| C329 | 8501(8) | 396(5) | 6375(4) | 77(3) |
| C330 | 8065(10) | 487(6) | 5803(5) | 88(3) |
| C331 | 8415(9) | 50(5) | 5279(4) | 78(3) |
| C332 | 9268(10) | -488(6) | 5338(5) | 94(3) |
| C333 | 9757(9) | -586(6) | 5895(5) | 90(3) |
| C334 | 9377(9) | -134(6) | 6390(5) | 88(3) |
| B301 | 6222(9) | 97(6) | 7533(5) | 70(3) |
| Sb21 | 5420.8(5) | 3929.6(3) | 2886.1(3) | 71.3(2) |
| Br21 | 6041.2(9) | 3176.0(6) | 1641.0(5) | 88.4(3) |
| F201 | 5137(5) | 3371(3) | 4463(3) | 98.4(19) |

| | | | | |
|---|---|---|---|---|
| F202 | 4224(5) | 3997(3) | 5568(2) | 96.0(18) |
| F203 | 3254(5) | 5326(3) | 5563(3) | 94.0(17) |
| F204 | 3164(6) | 6037(3) | 4438(3) | 107(2) |
| F205 | 4000(6) | 5430(3) | 3319(2) | 100(2) |
| N201 | 4146(6) | 2850(4) | 3113(3) | 70.2(19) |
| N202 | 5739(6) | 2352(4) | 3273(3) | 73(2) |
| N203 | 3194(5) | 3976(4) | 2594(3) | 63.4(17) |
| N204 | 3710(6) | 4777(4) | 2002(3) | 68.8(19) |
| C201 | 5084(8) | 2949(5) | 3140(4) | 72(2) |
| C202 | 4213(7) | 2158(5) | 3227(4) | 73(2) |
| C203 | 5198(8) | 1862(5) | 3318(5) | 82(3) |
| C204 | 6880(8) | 2159(6) | 3365(5) | 88(3) |
| C205 | 7320(9) | 2774(6) | 3308(6) | 99(3) |
| C206 | 7417(9) | 1650(6) | 2826(6) | 107(4) |
| C207 | 6950(10) | 1831(7) | 4047(5) | 113(4) |
| C208 | 3994(7) | 4263(5) | 2447(4) | 68(2) |
| C209 | 2421(8) | 4291(5) | 2227(4) | 76(3) |
| C210 | 2735(8) | 4793(5) | 1863(4) | 74(2) |
| C211 | 4308(8) | 5239(6) | 1622(4) | 79(3) |
| C212 | 4460(10) | 5027(6) | 914(4) | 88(3) |
| C213 | 5348(9) | 5174(6) | 1867(5) | 89(3) |
| C214 | 3661(10) | 5963(5) | 1710(5) | 89(3) |
| C215 | 2628(7) | 3677(5) | 3809(4) | 67(2) |
| C216 | 2799(8) | 3238(5) | 4353(4) | 76(3) |
| C217 | 2361(9) | 3442(6) | 4973(4) | 91(3) |
| C218 | 1744(9) | 4094(7) | 5076(4) | 87(3) |
| C219 | 1579(9) | 4537(6) | 4541(5) | 89(3) |
| C220 | 2019(7) | 4326(5) | 3913(4) | 69(2) |
| C221 | 1307(12) | 4319(8) | 5748(5) | 118(5) |
| C222 | 2269(7) | 3013(5) | 2816(4) | 64(2) |
| C223 | 1289(7) | 3044(5) | 3115(4) | 66(2) |
| C224 | 595(7) | 2742(5) | 2865(4) | 74(2) |
| C225 | 870(8) | 2383(5) | 2297(4) | 70(2) |
| C226 | 1873(8) | 2341(5) | 1982(4) | 73(2) |
| C227 | 2533(8) | 2648(5) | 2240(4) | 70(2) |
| C228 | 153(9) | 2050(6) | 2026(4) | 82(3) |
| C229 | 4589(8) | 4377(6) | 3842(4) | 75(3) |
| C230 | 4633(8) | 4038(6) | 4427(5) | 81(3) |
| C231 | 4172(8) | 4344(6) | 5013(4) | 83(3) |
| C232 | 3668(8) | 5017(6) | 5011(5) | 79(3) |
| C233 | 3631(9) | 5383(5) | 4429(5) | 80(3) |
| C234 | 4093(9) | 5058(5) | 3872(4) | 78(3) |
| B201 | 3074(8) | 3378(6) | 3095(5) | 64(2) |
| Sb11 | -1179.2(4) | 6771.6(3) | 33.0(2) | 61.75(18) |
| Br11 | -1594.4(9) | 8285.1(6) | -174.4(5) | 83.5(3) |
| F101 | -2717(4) | 5736(3) | 756(2) | 78.4(14) |
| F102 | -2291(4) | 4549(3) | 1403(2) | 84.1(15) |
| F103 | -316(5) | 3906(3) | 1589(2) | 82.0(15) |
| F104 | 1258(4) | 4445(3) | 1068(2) | 83.5(15) |
| F105 | 859(4) | 5637(3) | 431(2) | 76.0(13) |
| N101 | -2603(5) | 7331(4) | 1258(3) | 59.6(16) |
| N102 | -3586(6) | 7316(4) | 497(3) | 63.4(17) |
| N103 | -628(6) | 7213(4) | 1311(3) | 63.5(17) |
| N104 | 696(6) | 7172(4) | 591(3) | 64.5(18) |
| C101 | -2598(6) | 7142(5) | 647(4) | 63(2) |
| C102 | -3617(7) | 7619(5) | 1504(4) | 70(2) |

| | | | | |
|---|---|---|---|---|
| C103 | -4220(7) | 7616(5) | 1033(4) | 71(2) |
| C104 | -4050(7) | 7246(5) | -105(4) | 72(2) |
| C105 | -3275(7) | 6772(5) | -584(4) | 73(2) |
| C106 | -4393(11) | 7948(6) | -406(5) | 108(4) |
| C107 | -4960(8) | 6923(7) | 80(5) | 96(4) |
| C108 | -271(7) | 7071(4) | 699(4) | 63(2) |
| C109 | 114(7) | 7384(5) | 1615(4) | 68(2) |
| C110 | 952(8) | 7371(5) | 1170(4) | 73(2) |
| C111 | 1467(7) | 7137(5) | -8(4) | 69(2) |
| C112 | 1656(9) | 7849(5) | -141(5) | 84(3) |
| C113 | 1078(7) | 6918(5) | -589(4) | 68(2) |
| C114 | 2486(7) | 6631(5) | 136(4) | 74(2) |
| C115 | -1618(7) | 6447(5) | 2075(3) | 64(2) |
| C116 | -2497(8) | 6215(5) | 2292(4) | 74(2) |
| C117 | -2445(8) | 5598(5) | 2607(4) | 77(3) |
| C118 | -1505(9) | 5165(5) | 2738(4) | 75(3) |
| C119 | -637(8) | 5391(5) | 2545(4) | 75(2) |
| C120 | -686(8) | 6019(5) | 2220(4) | 72(2) |
| C121 | -1465(10) | 4474(5) | 3061(5) | 92(3) |
| C122 | -1974(7) | 7822(5) | 2203(4) | 69(2) |
| C123 | -1945(8) | 7769(6) | 2864(4) | 76(3) |
| C124 | -2116(9) | 8338(7) | 3246(4) | 90(3) |
| C125 | -2327(9) | 8990(6) | 2980(5) | 86(3) |
| C126 | -2382(8) | 9063(5) | 2312(4) | 74(2) |
| C127 | -2189(7) | 8490(5) | 1940(4) | 72(2) |
| C128 | -2539(13) | 9627(7) | 3390(6) | 120(5) |
| C129 | -954(7) | 5753(4) | 567(3) | 60(2) |
| C130 | -1722(7) | 5446(5) | 828(4) | 67(2) |
| C131 | -1524(8) | 4824(5) | 1161(4) | 71(2) |
| C132 | -522(8) | 4496(5) | 1258(4) | 70(2) |
| C133 | 265(8) | 4774(5) | 1002(4) | 73(2) |
| C134 | 41(8) | 5395(5) | 673(4) | 68(2) |
| B101 | -1698(9) | 7185(6) | 1723(4) | 66(3) |
| O401 | 551(17) | 7385(12) | 3347(10) | 264(9) |
| O402 | 772(10) | 6702(8) | 4117(6) | 174(4) |
| C401 | 1437(14) | 6260(11) | 3036(8) | 172(7) |
| C402 | 804(13) | 6749(14) | 3398(10) | 165(6) |
| C403 | 189(18) | 7229(12) | 4513(8) | 185(7) |
| C404 | 430(20) | 7012(12) | 5236(8) | 205(9) |

9.13.8 Anisotropic Displacement Parameters

Table S87. Anisotropic Displacement Parameters (×10[4]) for **16d**. The anisotropic displacement factor exponent takes the form: $-2\pi^2[h^2a^{*2} \times U_{11} + ... + 2hka^* \times b^* \times U_{12}]$

| Atom | $U_{11}$ | $U_{22}$ | $U_{33}$ | $U_{23}$ | $U_{13}$ | $U_{12}$ |
|---|---|---|---|---|---|---|
| Sb31 | 65.0(4) | 63.4(4) | 79.5(3) | -15.7(3) | 5.0(3) | 6.3(3) |
| Br31 | 63.4(6) | 79.3(7) | 102.7(6) | -15.3(5) | -4.3(5) | -16.8(5) |
| F301 | 109(5) | 84(4) | 84(3) | 3(4) | -11(3) | 5(3) |
| F302 | 144(7) | 89(5) | 87(3) | -18(4) | -8(4) | -4(3) |
| F303 | 109(5) | 99(5) | 108(4) | -23(4) | 14(4) | -16(3) |
| F304 | 85(5) | 109(5) | 139(5) | 12(4) | 7(4) | -23(4) |
| F305 | 65(4) | 110(5) | 106(4) | -6(3) | 1(3) | -6(3) |
| N301 | 58(4) | 65(5) | 73(4) | -13(4) | -5(3) | -2(3) |
| N302 | 80(6) | 67(5) | 70(4) | -13(4) | 4(4) | 4(3) |
| N303 | 63(5) | 62(4) | 76(4) | -23(4) | -1(3) | 3(3) |
| N304 | 61(5) | 69(5) | 74(4) | -11(4) | -6(3) | 4(3) |
| C301 | 58(5) | 66(6) | 72(4) | -20(4) | 1(4) | 5(4) |
| C302 | 52(5) | 90(7) | 76(5) | -12(5) | -2(4) | -10(5) |
| C303 | 66(6) | 82(7) | 75(5) | -7(5) | -11(4) | -4(5) |
| C304 | 100(9) | 72(7) | 87(6) | -10(6) | -10(6) | 4(5) |
| C305 | 86(9) | 78(8) | 166(11) | -19(6) | -18(8) | 48(7) |
| C306 | 144(12) | 89(8) | 75(6) | 4(8) | -8(6) | 11(5) |
| C307 | 99(9) | 72(7) | 108(7) | -12(6) | -16(6) | -2(5) |
| C308 | 61(5) | 68(6) | 70(4) | -16(4) | 5(4) | 1(4) |
| C309 | 61(6) | 67(6) | 79(5) | -14(4) | 1(4) | 9(4) |
| C310 | 82(7) | 70(6) | 79(5) | -9(5) | -12(5) | 9(4) |
| C311 | 67(6) | 86(7) | 93(6) | -15(5) | 5(5) | -11(5) |
| C312 | 84(8) | 102(9) | 114(7) | -5(7) | -11(6) | -27(7) |
| C313 | 51(6) | 81(7) | 134(8) | -20(5) | -10(5) | 10(6) |
| C314 | 59(6) | 88(8) | 114(7) | -10(5) | -15(5) | 0(6) |
| C315 | 80(7) | 58(5) | 72(5) | -9(5) | 9(4) | -1(4) |
| C316 | 90(8) | 79(7) | 75(5) | -17(6) | -5(5) | -14(5) |
| C317 | 114(9) | 78(7) | 71(5) | -17(7) | -2(5) | -1(5) |
| C318 | 105(9) | 75(7) | 83(6) | -29(6) | 16(6) | -7(5) |
| C319 | 91(8) | 67(6) | 98(7) | -20(6) | 18(6) | -8(5) |
| C320 | 71(6) | 69(6) | 76(5) | -16(5) | 11(4) | -2(4) |
| C321 | 156(13) | 85(8) | 83(6) | -35(8) | 17(7) | -7(5) |
| C322 | 66(6) | 77(6) | 72(5) | -25(5) | 2(4) | -1(4) |
| C323 | 84(7) | 78(7) | 84(6) | -18(6) | -4(5) | 5(5) |
| C324 | 78(7) | 88(8) | 104(7) | -34(6) | -11(6) | 13(6) |
| C325 | 76(7) | 107(9) | 81(6) | -24(6) | -1(5) | 2(6) |
| C326 | 76(7) | 99(8) | 80(5) | -18(6) | 14(5) | 0(5) |
| C327 | 66(6) | 83(7) | 84(5) | -25(5) | 11(4) | -3(5) |
| C328 | 83(8) | 147(12) | 103(7) | -32(8) | 19(6) | 30(7) |
| C329 | 76(7) | 68(6) | 81(5) | -8(5) | -9(5) | 12(4) |
| C330 | 96(8) | 74(7) | 92(6) | -26(6) | 4(6) | 9(5) |
| C331 | 83(7) | 75(7) | 71(5) | -16(5) | 4(5) | -1(4) |
| C332 | 85(8) | 85(8) | 102(7) | -19(6) | 26(6) | -12(6) |
| C333 | 70(7) | 104(9) | 91(7) | -17(6) | 1(5) | -7(6) |
| C334 | 76(7) | 82(7) | 94(6) | -8(6) | 15(5) | 5(5) |
| B301 | 69(7) | 68(7) | 70(5) | -14(5) | 3(5) | -1(5) |
| Sb21 | 57.8(4) | 77.1(4) | 78.4(3) | -19.5(3) | -4.9(3) | 10.6(3) |
| Br21 | 77.9(7) | 102.9(9) | 78.3(5) | -21.2(6) | 10.2(5) | 4.7(5) |
| F201 | 91(4) | 103(5) | 88(3) | -1(4) | -16(3) | 14(3) |
| F202 | 92(4) | 108(5) | 82(3) | -16(4) | -8(3) | 6(3) |

| | | | | | | |
|---|---|---|---|---|---|---|
| F203 | 100(5) | 100(5) | 85(3) | -30(4) | -6(3) | -8(3) |
| F204 | 143(6) | 71(4) | 102(4) | -19(4) | -12(4) | 4(3) |
| F205 | 135(6) | 84(4) | 80(3) | -27(4) | -8(3) | 13(3) |
| N201 | 59(5) | 79(5) | 69(4) | -16(4) | 0(3) | 12(3) |
| N202 | 56(4) | 69(5) | 86(4) | -3(4) | -2(3) | 11(4) |
| N203 | 55(4) | 69(5) | 66(3) | -19(3) | -1(3) | 10(3) |
| N204 | 67(5) | 67(5) | 71(4) | -19(4) | -6(3) | 13(3) |
| C201 | 67(6) | 69(6) | 73(5) | -13(5) | 4(4) | 7(4) |
| C202 | 57(5) | 71(6) | 86(5) | -12(5) | -2(4) | 11(4) |
| C203 | 70(7) | 70(6) | 102(6) | -16(5) | 0(5) | 18(5) |
| C204 | 66(6) | 91(8) | 97(6) | -10(6) | -3(5) | 11(6) |
| C205 | 58(6) | 101(9) | 126(8) | 1(6) | -10(6) | -4(7) |
| C206 | 77(8) | 92(9) | 131(8) | 5(6) | 16(6) | 1(7) |
| C207 | 78(8) | 131(11) | 113(8) | -2(7) | -7(6) | 26(7) |
| C208 | 56(5) | 78(6) | 63(4) | -5(4) | -5(4) | 13(4) |
| C209 | 74(6) | 80(6) | 74(5) | -23(5) | -9(4) | 17(4) |
| C210 | 78(7) | 72(6) | 74(5) | -23(5) | -12(4) | 16(4) |
| C211 | 73(7) | 92(7) | 80(5) | -35(6) | -6(5) | 14(5) |
| C212 | 109(9) | 84(7) | 69(5) | -29(6) | 6(5) | 5(5) |
| C213 | 85(7) | 92(8) | 90(6) | -35(6) | 4(5) | 26(5) |
| C214 | 120(10) | 68(6) | 88(6) | -38(6) | -22(6) | 19(5) |
| C215 | 58(5) | 75(6) | 69(4) | -23(5) | -3(4) | 6(4) |
| C216 | 82(7) | 77(7) | 66(5) | -19(5) | -3(4) | 7(4) |
| C217 | 98(8) | 103(9) | 66(5) | -20(7) | 4(5) | -4(5) |
| C218 | 88(8) | 111(9) | 66(5) | -33(7) | 3(5) | -15(5) |
| C219 | 99(8) | 91(8) | 80(6) | -28(6) | 0(5) | -17(5) |
| C220 | 57(5) | 76(6) | 70(5) | -13(5) | 0(4) | 1(4) |
| C221 | 126(12) | 142(12) | 85(6) | -37(9) | 6(7) | -29(7) |
| C222 | 56(5) | 70(5) | 62(4) | -17(4) | 1(3) | 3(4) |
| C223 | 49(5) | 76(6) | 74(5) | -20(4) | 6(4) | -6(4) |
| C224 | 55(5) | 91(7) | 75(5) | -24(5) | 2(4) | 1(5) |
| C225 | 69(6) | 75(6) | 67(4) | -24(5) | -7(4) | 0(4) |
| C226 | 85(7) | 65(6) | 68(5) | -20(5) | 2(4) | -1(4) |
| C227 | 65(6) | 77(6) | 70(5) | -25(5) | 3(4) | 5(4) |
| C228 | 79(7) | 90(7) | 80(5) | -29(6) | -8(5) | 4(5) |
| C229 | 64(6) | 93(8) | 71(5) | -24(5) | -6(4) | 9(5) |
| C230 | 70(6) | 84(7) | 85(6) | -12(5) | -13(5) | 12(5) |
| C231 | 76(7) | 96(8) | 73(5) | -14(6) | -12(4) | 8(5) |
| C232 | 72(6) | 80(7) | 85(6) | -16(5) | -12(5) | 2(5) |
| C233 | 90(7) | 74(7) | 80(6) | -26(6) | -12(5) | 7(5) |
| C234 | 89(7) | 69(6) | 78(5) | -25(5) | -15(5) | 7(5) |
| B201 | 48(5) | 69(6) | 75(5) | -15(5) | -3(4) | 5(5) |
| Sb11 | 56.4(3) | 71.6(4) | 56.1(3) | -15.7(3) | -0.4(2) | -5.3(2) |
| Br11 | 82.9(7) | 75.4(7) | 83.7(6) | -11.9(5) | 1.9(5) | 13.5(5) |
| F101 | 62(3) | 73(3) | 99(3) | -18(3) | -2(3) | -2(3) |
| F102 | 73(4) | 79(4) | 97(3) | -21(3) | 8(3) | 2(3) |
| F103 | 87(4) | 76(4) | 79(3) | -15(3) | -4(3) | -1(3) |
| F104 | 73(4) | 81(4) | 94(3) | -9(3) | -20(3) | -8(3) |
| F105 | 65(3) | 72(3) | 89(3) | -14(3) | -6(2) | -2(2) |
| N101 | 53(4) | 68(4) | 55(3) | -11(3) | -3(3) | -3(3) |
| N102 | 62(4) | 68(5) | 62(3) | -20(3) | -1(3) | -3(3) |
| N103 | 60(4) | 66(4) | 65(3) | -16(3) | -5(3) | -7(3) |
| N104 | 67(5) | 66(4) | 61(3) | -20(4) | -3(3) | 1(3) |
| C101 | 48(5) | 69(5) | 65(4) | -6(4) | -2(3) | -2(4) |
| C102 | 49(5) | 87(6) | 70(5) | -12(4) | 8(4) | -12(4) |
| C103 | 59(6) | 79(6) | 70(5) | -9(5) | -6(4) | -4(4) |

| | | | | | | |
|---|---|---|---|---|---|---|
| C104 | 57(5) | 95(7) | 64(4) | -18(5) | -9(4) | 8(4) |
| C105 | 61(6) | 89(7) | 74(5) | -23(5) | -8(4) | -6(4) |
| C106 | 113(10) | 95(9) | 100(7) | 9(7) | -33(7) | 12(6) |
| C107 | 62(6) | 146(11) | 84(6) | -36(7) | 8(5) | -28(6) |
| C108 | 56(5) | 67(5) | 62(4) | -11(4) | -5(3) | 1(4) |
| C109 | 64(6) | 83(6) | 58(4) | -19(5) | -6(4) | -7(4) |
| C110 | 63(6) | 88(7) | 72(5) | -24(5) | -7(4) | -11(4) |
| C111 | 58(5) | 76(6) | 69(4) | -12(4) | 1(4) | -2(4) |
| C112 | 79(7) | 82(7) | 91(6) | -34(6) | 7(5) | 8(5) |
| C113 | 62(5) | 74(6) | 66(4) | -15(4) | -2(4) | -2(4) |
| C114 | 64(6) | 85(7) | 75(5) | -24(5) | -2(4) | -1(4) |
| C115 | 62(5) | 71(6) | 59(4) | -17(4) | -3(4) | -4(4) |
| C116 | 62(6) | 79(7) | 79(5) | -15(5) | -2(4) | -1(5) |
| C117 | 69(6) | 85(7) | 80(5) | -28(5) | -6(4) | 7(5) |
| C118 | 98(8) | 67(6) | 58(4) | -19(5) | -2(4) | -3(4) |
| C119 | 71(6) | 76(6) | 74(5) | -16(5) | 4(4) | -5(4) |
| C120 | 75(6) | 73(6) | 65(4) | -17(5) | -2(4) | -2(4) |
| C121 | 114(9) | 67(6) | 86(6) | -16(6) | 2(6) | -6(5) |
| C122 | 63(5) | 80(7) | 67(4) | -22(5) | -6(4) | -5(4) |
| C123 | 77(6) | 84(7) | 68(5) | -20(5) | -3(4) | -14(4) |
| C124 | 95(8) | 107(9) | 67(5) | -16(7) | -12(5) | -22(5) |
| C125 | 81(7) | 85(8) | 95(7) | -23(6) | -7(5) | -18(6) |
| C126 | 68(6) | 68(6) | 88(6) | -19(5) | 0(4) | -17(5) |
| C127 | 65(6) | 71(6) | 78(5) | -18(5) | 1(4) | -6(4) |
| C128 | 150(13) | 95(9) | 114(8) | -16(8) | -26(8) | -41(7) |
| C129 | 65(5) | 56(5) | 56(4) | -14(4) | 3(3) | -3(3) |
| C130 | 53(5) | 70(6) | 80(5) | -20(4) | 0(4) | 2(4) |
| C131 | 64(6) | 71(6) | 78(5) | -19(5) | 2(4) | -3(4) |
| C132 | 74(6) | 65(6) | 68(4) | -16(5) | -3(4) | -4(4) |
| C133 | 71(6) | 82(7) | 67(4) | -18(5) | -5(4) | -6(4) |
| C134 | 74(6) | 68(6) | 60(4) | -18(5) | -6(4) | 2(4) |
| B101 | 69(7) | 72(7) | 59(5) | -19(5) | -6(4) | -3(4) |
| O401 | 224(19) | 246(14) | 281(13) | 2(8) | 7(9) | -10(7) |
| O402 | 122(9) | 222(11) | 169(7) | -28(7) | 5(5) | -37(6) |
| C401 | 102(12) | 222(15) | 166(11) | 8(9) | -20(7) | -13(7) |
| C402 | 77(9) | 229(14) | 170(8) | -2(8) | -3(5) | -22(6) |
| C403 | 176(17) | 206(14) | 153(8) | -40(9) | 48(7) | -19(7) |
| C404 | 240(20) | 220(20) | 131(8) | -31(14) | 50(7) | -31(7) |

9.13.9 Bond Lengths

Table S88. Bond Lengths in Å for **16d**.

| Atom | Atom | Length/Å |
|---|---|---|
| Sb31 | C301 | 2.182(9) |
| Sb31 | C308 | 2.148(9) |
| Sb31 | C329 | 2.257(10) |
| F301 | C330 | 1.338(13) |
| F302 | C331 | 1.321(12) |
| F303 | C332 | 1.337(12) |
| F304 | C333 | 1.326(13) |
| F305 | C334 | 1.338(13) |
| N301 | C301 | 1.324(11) |
| N301 | C302 | 1.378(11) |
| N301 | B301 | 1.569(13) |
| N302 | C301 | 1.358(11) |
| N302 | C303 | 1.378(13) |
| N302 | C304 | 1.496(13) |
| N303 | C308 | 1.366(12) |
| N303 | C309 | 1.362(11) |
| N303 | B301 | 1.599(14) |
| N304 | C308 | 1.349(11) |
| N304 | C310 | 1.383(12) |
| N304 | C311 | 1.509(13) |
| C302 | C303 | 1.351(14) |
| C304 | C305 | 1.500(17) |
| C304 | C306 | 1.523(15) |
| C304 | C307 | 1.548(15) |
| C309 | C310 | 1.339(14) |
| C311 | C312 | 1.537(14) |
| C311 | C313 | 1.514(14) |
| C311 | C314 | 1.503(14) |
| C315 | C316 | 1.387(14) |
| C315 | C320 | 1.373(13) |
| C315 | B301 | 1.640(14) |
| C316 | C317 | 1.407(13) |
| C317 | C318 | 1.367(16) |
| C318 | C319 | 1.374(16) |
| C318 | C321 | 1.522(14) |
| C319 | C320 | 1.411(13) |
| C322 | C323 | 1.367(15) |
| C322 | C327 | 1.423(13) |
| C322 | B301 | 1.607(13) |
| C323 | C324 | 1.426(14) |
| C324 | C325 | 1.375(15) |
| C325 | C326 | 1.389(16) |
| C325 | C328 | 1.532(14) |
| C326 | C327 | 1.376(13) |
| C329 | C330 | 1.374(15) |
| C329 | C334 | 1.379(14) |
| C330 | C331 | 1.401(14) |
| C331 | C332 | 1.380(15) |
| C332 | C333 | 1.382(16) |
| C333 | C334 | 1.380(15) |
| Sb21 | Br21 | 3.0036(13) |
| Sb21 | C201 | 2.178(10) |
| Sb21 | C208 | 2.159(9) |
| Sb21 | C229 | 2.278(9) |
| F201 | C230 | 1.354(12) |
| F202 | C231 | 1.325(10) |
| F203 | C232 | 1.331(11) |
| F204 | C233 | 1.316(11) |
| F205 | C234 | 1.347(10) |
| N201 | C201 | 1.333(12) |
| N201 | C202 | 1.395(12) |
| N201 | B201 | 1.566(12) |
| N202 | C201 | 1.343(11) |
| N202 | C203 | 1.368(13) |
| N202 | C204 | 1.514(13) |
| N203 | C208 | 1.345(12) |
| N203 | C209 | 1.369(12) |
| N203 | B201 | 1.588(12) |
| N204 | C208 | 1.371(10) |
| N204 | C210 | 1.366(12) |
| N204 | C211 | 1.521(11) |
| C202 | C203 | 1.338(14) |
| C204 | C205 | 1.506(17) |
| C204 | C206 | 1.549(15) |
| C204 | C207 | 1.544(14) |
| C209 | C210 | 1.362(13) |
| C211 | C212 | 1.535(13) |
| C211 | C213 | 1.516(15) |
| C211 | C214 | 1.514(15) |
| C215 | C216 | 1.411(12) |
| C215 | C220 | 1.377(13) |
| C215 | B201 | 1.623(13) |
| C216 | C217 | 1.394(12) |
| C217 | C218 | 1.383(15) |
| C218 | C219 | 1.400(15) |
| C218 | C221 | 1.501(13) |
| C219 | C220 | 1.411(12) |
| C222 | C223 | 1.377(11) |
| C222 | C227 | 1.406(12) |
| C222 | B201 | 1.629(14) |
| C223 | C224 | 1.398(13) |
| C224 | C225 | 1.384(12) |
| C225 | C226 | 1.412(13) |
| C225 | C228 | 1.485(14) |
| C226 | C227 | 1.374(14) |
| C229 | C230 | 1.371(13) |
| C229 | C234 | 1.374(14) |
| C230 | C231 | 1.401(14) |
| C231 | C232 | 1.362(14) |
| C232 | C233 | 1.390(13) |
| C233 | C234 | 1.368(13) |
| Sb11 | Br11 | 3.0000(13) |
| Sb11 | C101 | 2.151(8) |
| Sb11 | C108 | 2.156(9) |
| Sb11 | C129 | 2.267(8) |

| | | |
|---|---|---|
| F101 | C130 | 1.338(10) |
| F102 | C131 | 1.330(10) |
| F103 | C132 | 1.334(10) |
| F104 | C133 | 1.350(11) |
| F105 | C134 | 1.351(10) |
| N101 | C101 | 1.349(10) |
| N101 | C102 | 1.386(10) |
| N101 | B101 | 1.602(12) |
| N102 | C101 | 1.354(11) |
| N102 | C103 | 1.388(10) |
| N102 | C104 | 1.494(11) |
| N103 | C108 | 1.329(9) |
| N103 | C109 | 1.364(11) |
| N103 | B101 | 1.597(12) |
| N104 | C108 | 1.356(11) |
| N104 | C110 | 1.398(11) |
| N104 | C111 | 1.519(10) |
| C102 | C103 | 1.345(13) |
| C104 | C105 | 1.533(13) |
| C104 | C106 | 1.510(14) |
| C104 | C107 | 1.532(14) |
| C109 | C110 | 1.367(12) |
| C111 | C112 | 1.537(14) |
| C111 | C113 | 1.503(13) |
| C111 | C114 | 1.539(13) |
| C115 | C116 | 1.399(13) |
| C115 | C120 | 1.388(13) |
| C115 | B101 | 1.620(14) |
| C116 | C117 | 1.375(13) |
| C117 | C118 | 1.391(14) |
| C118 | C119 | 1.368(14) |
| C118 | C121 | 1.518(13) |
| C119 | C120 | 1.403(13) |
| C122 | C123 | 1.381(12) |
| C122 | C127 | 1.409(13) |
| C122 | B101 | 1.616(14) |
| C123 | C124 | 1.394(14) |
| C124 | C125 | 1.384(16) |
| C125 | C126 | 1.402(14) |
| C125 | C128 | 1.533(15) |
| C126 | C127 | 1.384(13) |
| C129 | C130 | 1.382(12) |
| C129 | C134 | 1.386(13) |
| C130 | C131 | 1.392(13) |
| C131 | C132 | 1.375(13) |
| C132 | C133 | 1.364(13) |
| C133 | C134 | 1.385(13) |
| O401 | C402 | 1.25(3) |
| O402 | C402 | 1.49(2) |
| O402 | C403 | 1.40(2) |
| C401 | C402 | 1.33(2) |
| C403 | C404 | 1.60(3) |

9.13.10 Bond Angles

Table S89. Bond Angles in ° for **16d**.

| Atom | Atom | Atom | Angle/° |
|---|---|---|---|
| C308 | Sb31 | C301 | 91.2(3) |
| C329 | Sb31 | C301 | 91.2(4) |
| C329 | Sb31 | C308 | 90.3(3) |
| C302 | N301 | C301 | 106.2(8) |
| B301 | N301 | C301 | 131.8(8) |
| B301 | N301 | C302 | 121.1(8) |
| C303 | N302 | C301 | 108.2(8) |
| C304 | N302 | C301 | 132.2(9) |
| C304 | N302 | C303 | 119.6(9) |
| C309 | N303 | C308 | 107.2(8) |
| B301 | N303 | C308 | 131.6(7) |
| B301 | N303 | C309 | 121.2(8) |
| C310 | N304 | C308 | 108.6(8) |
| C311 | N304 | C308 | 132.4(8) |
| C311 | N304 | C310 | 118.9(8) |
| N301 | C301 | Sb31 | 122.2(6) |
| N302 | C301 | Sb31 | 127.5(7) |
| N302 | C301 | N301 | 109.8(8) |
| C303 | C302 | N301 | 110.4(9) |
| C302 | C303 | N302 | 105.3(8) |
| C305 | C304 | N302 | 112.0(9) |
| C306 | C304 | N302 | 107.7(10) |
| C306 | C304 | C305 | 109.8(10) |
| C307 | C304 | N302 | 106.1(8) |
| C307 | C304 | C305 | 108.9(11) |
| C307 | C304 | C306 | 112.3(9) |
| N303 | C308 | Sb31 | 122.1(7) |
| N304 | C308 | Sb31 | 130.0(7) |
| N304 | C308 | N303 | 107.8(7) |
| C310 | C309 | N303 | 109.7(9) |
| C309 | C310 | N304 | 106.6(8) |
| C312 | C311 | N304 | 107.3(8) |
| C313 | C311 | N304 | 111.2(8) |
| C313 | C311 | C312 | 108.2(10) |
| C314 | C311 | N304 | 108.2(9) |
| C314 | C311 | C312 | 111.7(9) |
| C314 | C311 | C313 | 110.2(9) |
| C320 | C315 | C316 | 116.6(9) |
| B301 | C315 | C316 | 119.1(8) |
| B301 | C315 | C320 | 124.3(9) |
| C317 | C316 | C315 | 122.0(10) |
| C318 | C317 | C316 | 120.3(11) |
| C319 | C318 | C317 | 118.7(10) |
| C321 | C318 | C317 | 121.0(12) |
| C321 | C318 | C319 | 120.2(11) |
| C320 | C319 | C318 | 120.5(10) |
| C319 | C320 | C315 | 121.8(10) |
| C327 | C322 | C323 | 116.4(9) |
| B301 | C322 | C323 | 123.5(9) |
| B301 | C322 | C327 | 120.1(9) |
| C324 | C323 | C322 | 121.2(10) |
| C325 | C324 | C323 | 120.7(11) |
| C326 | C325 | C324 | 119.0(10) |
| C328 | C325 | C324 | 121.2(12) |
| C328 | C325 | C326 | 119.7(11) |
| C327 | C326 | C325 | 119.7(10) |
| C326 | C327 | C322 | 122.9(10) |
| C330 | C329 | Sb31 | 123.4(7) |
| C334 | C329 | Sb31 | 120.3(8) |
| C334 | C329 | C330 | 115.4(10) |
| C329 | C330 | F301 | 121.1(10) |
| C331 | C330 | F301 | 115.2(10) |
| C331 | C330 | C329 | 123.8(11) |
| C330 | C331 | F302 | 121.5(10) |
| C332 | C331 | F302 | 120.8(10) |
| C332 | C331 | C330 | 117.7(10) |
| C331 | C332 | F303 | 119.8(12) |
| C333 | C332 | F303 | 119.5(11) |
| C333 | C332 | C331 | 120.7(10) |
| C332 | C333 | F304 | 119.0(10) |
| C334 | C333 | F304 | 122.3(11) |
| C334 | C333 | C332 | 118.6(11) |
| C329 | C334 | F305 | 119.5(10) |
| C333 | C334 | F305 | 116.6(10) |
| C333 | C334 | C329 | 123.7(11) |
| N303 | B301 | N301 | 108.9(8) |
| C315 | B301 | N301 | 107.8(7) |
| C315 | B301 | N303 | 110.0(8) |
| C322 | B301 | N301 | 108.1(8) |
| C322 | B301 | N303 | 106.0(7) |
| C322 | B301 | C315 | 115.9(9) |
| C201 | Sb21 | Br21 | 77.1(2) |
| C208 | Sb21 | Br21 | 80.1(2) |
| C208 | Sb21 | C201 | 90.4(4) |
| C229 | Sb21 | Br21 | 164.9(3) |
| C229 | Sb21 | C201 | 91.6(3) |
| C229 | Sb21 | C208 | 90.1(3) |
| C202 | N201 | C201 | 107.8(8) |
| B201 | N201 | C201 | 130.0(8) |
| B201 | N201 | C202 | 121.2(8) |
| C203 | N202 | C201 | 107.8(9) |
| C204 | N202 | C201 | 132.3(9) |
| C204 | N202 | C203 | 119.9(9) |
| C209 | N203 | C208 | 108.3(7) |
| B201 | N203 | C208 | 129.9(7) |
| B201 | N203 | C209 | 121.9(8) |
| C210 | N204 | C208 | 108.3(8) |
| C211 | N204 | C208 | 131.9(8) |
| C211 | N204 | C210 | 119.4(7) |
| N201 | C201 | Sb21 | 121.7(7) |
| N202 | C201 | Sb21 | 128.9(8) |
| N202 | C201 | N201 | 109.0(9) |
| C203 | C202 | N201 | 106.8(9) |

| | | | |
|---|---|---|---|
| C202 | C203 | N202 | 108.5(9) |
| C205 | C204 | N202 | 111.3(9) |
| C206 | C204 | N202 | 106.0(9) |
| C206 | C204 | C205 | 110.4(9) |
| C207 | C204 | N202 | 105.7(8) |
| C207 | C204 | C205 | 110.7(11) |
| C207 | C204 | C206 | 112.5(10) |
| N203 | C208 | Sb21 | 122.6(6) |
| N204 | C208 | Sb21 | 129.5(7) |
| N204 | C208 | N203 | 107.9(8) |
| C210 | C209 | N203 | 108.3(9) |
| C209 | C210 | N204 | 107.2(8) |
| C212 | C211 | N204 | 106.9(8) |
| C213 | C211 | N204 | 111.5(7) |
| C213 | C211 | C212 | 110.3(9) |
| C214 | C211 | N204 | 107.1(8) |
| C214 | C211 | C212 | 112.0(8) |
| C214 | C211 | C213 | 109.1(9) |
| C220 | C215 | C216 | 117.3(8) |
| B201 | C215 | C216 | 119.0(8) |
| B201 | C215 | C220 | 123.5(7) |
| C217 | C216 | C215 | 122.0(10) |
| C218 | C217 | C216 | 120.6(10) |
| C219 | C218 | C217 | 118.0(9) |
| C221 | C218 | C217 | 120.3(10) |
| C221 | C218 | C219 | 121.6(11) |
| C220 | C219 | C218 | 121.3(10) |
| C219 | C220 | C215 | 120.9(9) |
| C227 | C222 | C223 | 115.1(9) |
| B201 | C222 | C223 | 123.3(8) |
| B201 | C222 | C227 | 121.6(7) |
| C224 | C223 | C222 | 122.9(8) |
| C225 | C224 | C223 | 121.1(8) |
| C226 | C225 | C224 | 117.1(9) |
| C228 | C225 | C224 | 122.2(9) |
| C228 | C225 | C226 | 120.7(8) |
| C227 | C226 | C225 | 120.2(8) |
| C226 | C227 | C222 | 123.5(9) |
| C230 | C229 | Sb21 | 124.9(8) |
| C234 | C229 | Sb21 | 119.8(6) |
| C234 | C229 | C230 | 114.8(9) |
| C229 | C230 | F201 | 120.3(9) |
| C231 | C230 | F201 | 116.3(8) |
| C231 | C230 | C229 | 123.4(10) |
| C230 | C231 | F202 | 121.5(10) |
| C232 | C231 | F202 | 119.3(9) |
| C232 | C231 | C230 | 119.2(9) |
| C231 | C232 | F203 | 120.3(9) |
| C233 | C232 | F203 | 120.5(10) |
| C233 | C232 | C231 | 119.2(9) |
| C232 | C233 | F204 | 118.4(9) |
| C234 | C233 | F204 | 122.4(9) |
| C234 | C233 | C232 | 119.2(10) |
| C229 | C234 | F205 | 118.9(9) |
| C233 | C234 | F205 | 116.8(9) |
| C233 | C234 | C229 | 124.2(9) |

| | | | |
|---|---|---|---|
| N203 | B201 | N201 | 109.4(7) |
| C215 | B201 | N201 | 110.8(7) |
| C215 | B201 | N203 | 110.7(8) |
| C222 | B201 | N201 | 109.0(8) |
| C222 | B201 | N203 | 105.9(7) |
| C222 | B201 | C215 | 110.9(7) |
| C101 | Sb11 | Br11 | 77.3(2) |
| C108 | Sb11 | Br11 | 76.5(2) |
| C108 | Sb11 | C101 | 92.3(3) |
| C129 | Sb11 | Br11 | 159.05(18) |
| C129 | Sb11 | C101 | 89.4(3) |
| C129 | Sb11 | C108 | 88.2(3) |
| C102 | N101 | C101 | 108.6(7) |
| B101 | N101 | C101 | 130.9(7) |
| B101 | N101 | C102 | 120.0(6) |
| C103 | N102 | C101 | 108.0(7) |
| C104 | N102 | C101 | 132.3(7) |
| C104 | N102 | C103 | 119.7(7) |
| C109 | N103 | C108 | 109.6(7) |
| B101 | N103 | C108 | 131.8(8) |
| B101 | N103 | C109 | 118.6(7) |
| C110 | N104 | C108 | 108.2(7) |
| C111 | N104 | C108 | 133.2(7) |
| C111 | N104 | C110 | 118.5(8) |
| N101 | C101 | Sb11 | 121.9(6) |
| N102 | C101 | Sb11 | 129.4(6) |
| N102 | C101 | N101 | 108.1(7) |
| C103 | C102 | N101 | 107.4(7) |
| C102 | C103 | N102 | 107.9(8) |
| C105 | C104 | N102 | 111.6(7) |
| C106 | C104 | N102 | 107.9(9) |
| C106 | C104 | C105 | 110.2(8) |
| C107 | C104 | N102 | 108.0(7) |
| C107 | C104 | C105 | 107.2(9) |
| C107 | C104 | C106 | 111.9(10) |
| N103 | C108 | Sb11 | 122.5(6) |
| N104 | C108 | Sb11 | 129.2(5) |
| N104 | C108 | N103 | 108.2(7) |
| C110 | C109 | N103 | 108.0(7) |
| C109 | C110 | N104 | 106.1(9) |
| C112 | C111 | N104 | 108.0(7) |
| C113 | C111 | N104 | 112.0(8) |
| C113 | C111 | C112 | 109.3(7) |
| C114 | C111 | N104 | 107.9(7) |
| C114 | C111 | C112 | 109.4(9) |
| C114 | C111 | C113 | 110.1(8) |
| C120 | C115 | C116 | 115.1(9) |
| B101 | C115 | C116 | 122.0(8) |
| B101 | C115 | C120 | 122.8(9) |
| C117 | C116 | C115 | 122.8(9) |
| C118 | C117 | C116 | 121.7(10) |
| C119 | C118 | C117 | 116.4(9) |
| C121 | C118 | C117 | 120.7(10) |
| C121 | C118 | C119 | 122.9(10) |
| C120 | C119 | C118 | 122.1(10) |
| C119 | C120 | C115 | 121.9(10) |

| | | | |
|---|---|---|---|
| C127 | C122 | C123 | 115.7(9) |
| B101 | C122 | C123 | 124.9(9) |
| B101 | C122 | C127 | 119.3(7) |
| C124 | C123 | C122 | 122.2(10) |
| C125 | C124 | C123 | 121.3(9) |
| C126 | C125 | C124 | 117.9(9) |
| C128 | C125 | C124 | 122.5(10) |
| C128 | C125 | C126 | 119.5(11) |
| C127 | C126 | C125 | 119.7(10) |
| C126 | C127 | C122 | 123.2(8) |
| C130 | C129 | Sb11 | 126.6(7) |
| C134 | C129 | Sb11 | 118.9(6) |
| C134 | C129 | C130 | 114.4(8) |
| C129 | C130 | F101 | 120.6(8) |
| C131 | C130 | F101 | 116.0(8) |
| C131 | C130 | C129 | 123.3(8) |
| C130 | C131 | F102 | 121.1(9) |
| C132 | C131 | F102 | 119.3(9) |
| C132 | C131 | C130 | 119.5(9) |
| C131 | C132 | F103 | 120.6(9) |
| C133 | C132 | F103 | 120.2(9) |
| C133 | C132 | C131 | 119.3(9) |
| C132 | C133 | F104 | 120.2(9) |
| C134 | C133 | F104 | 120.2(9) |
| C134 | C133 | C132 | 119.7(9) |
| C129 | C134 | F105 | 119.9(8) |
| C133 | C134 | F105 | 116.3(8) |
| C133 | C134 | C129 | 123.7(9) |
| N103 | B101 | N101 | 109.7(6) |
| C115 | B101 | N101 | 109.0(8) |
| C115 | B101 | N103 | 110.9(8) |
| C122 | B101 | N101 | 106.0(7) |
| C122 | B101 | N103 | 105.7(8) |
| C122 | B101 | C115 | 115.3(7) |
| C403 | O402 | C402 | 122.2(17) |
| O402 | C402 | O401 | 97(2) |
| C401 | C402 | O401 | 134(2) |
| C401 | C402 | O402 | 120(2) |
| C404 | C403 | O402 | 107.0(18) |

9.13.11 Torsion Angles

Table S90. Torsion Angles in ° for **16d**.

| Atom | Atom | Atom | Atom | Angle/° |
|---|---|---|---|---|
| Sb31 | C301 | N301 | C302 | 173.9(7) |
| Sb31 | C301 | N301 | B301 | -17.0(10) |
| Sb31 | C301 | N302 | C303 | -173.9(7) |
| Sb31 | C301 | N302 | C304 | 8.7(10) |
| Sb31 | C308 | N303 | C309 | -178.4(7) |
| Sb31 | C308 | N303 | B301 | 3.1(9) |
| Sb31 | C308 | N304 | C310 | 177.9(8) |
| Sb31 | C308 | N304 | C311 | 1.8(10) |
| Sb31 | C329 | C330 | F301 | -7.4(9) |
| Sb31 | C329 | C330 | C331 | 174.0(8) |
| Sb31 | C329 | C334 | F305 | 10.5(9) |
| Sb31 | C329 | C334 | C333 | -175.0(8) |
| F301 | C330 | C329 | C334 | -176.6(11) |
| F301 | C330 | C331 | F302 | -0.5(11) |
| F301 | C330 | C331 | C332 | 179.2(10) |
| F302 | C331 | C330 | C329 | 178.2(10) |
| F302 | C331 | C332 | F303 | -0.1(11) |
| F302 | C331 | C332 | C333 | 179.4(11) |
| F303 | C332 | C331 | C330 | -179.8(10) |
| F303 | C332 | C333 | F304 | -3.5(12) |
| F303 | C332 | C333 | C334 | 179.2(10) |
| F304 | C333 | C332 | C331 | 177.0(10) |
| F304 | C333 | C334 | F305 | 0.9(13) |
| F304 | C333 | C334 | C329 | -173.8(11) |
| F305 | C334 | C329 | C330 | -180.0(10) |
| F305 | C334 | C333 | C332 | 178.1(10) |
| N301 | C301 | N302 | C303 | -1.6(8) |
| N301 | C301 | N302 | C304 | -179.0(7) |
| N301 | C302 | C303 | N302 | -0.8(8) |
| N301 | B301 | N303 | C308 | -31.8(9) |
| N301 | B301 | N303 | C309 | 149.9(6) |
| N301 | B301 | C315 | C316 | -44.4(10) |
| N301 | B301 | C315 | C320 | 136.2(8) |
| N301 | B301 | C322 | C323 | 128.8(8) |
| N301 | B301 | C322 | C327 | -52.7(9) |
| N303 | C308 | N304 | C310 | 0.8(8) |
| N303 | C308 | N304 | C311 | -175.3(6) |
| N303 | C309 | C310 | N304 | -0.3(8) |
| N303 | B301 | C315 | C316 | -163.0(8) |
| N303 | B301 | C315 | C320 | 17.7(10) |
| N303 | B301 | C322 | C323 | -114.6(8) |
| N303 | B301 | C322 | C327 | 63.9(9) |
| C315 | C316 | C317 | C318 | 1.6(12) |
| C315 | C320 | C319 | C318 | -0.8(12) |
| C315 | B301 | C322 | C323 | 7.8(11) |
| C315 | B301 | C322 | C327 | -173.7(8) |
| C316 | C317 | C318 | C319 | 0.9(13) |
| C316 | C317 | C318 | C321 | -178.6(10) |
| C317 | C318 | C319 | C320 | -1.4(12) |
| C322 | C323 | C324 | C325 | 2.7(13) |
| C322 | C327 | C326 | C325 | 0.2(12) |
| C323 | C324 | C325 | C326 | -1.2(12) |

| | | | | |
|---|---|---|---|---|
| C323 | C324 | C325 | C328 | 179.9(10) |
| C324 | C325 | C326 | C327 | -0.3(12) |
| C329 | C330 | C331 | C332 | -2.1(13) |
| C329 | C334 | C333 | C332 | 3.4(14) |
| C330 | C331 | C332 | C333 | -0.3(12) |
| C331 | C332 | C333 | C334 | -0.3(13) |
| Sb21 | C201 | N201 | C202 | 172.9(6) |
| Sb21 | C201 | N201 | B201 | -18.4(9) |
| Sb21 | C201 | N202 | C203 | -171.8(8) |
| Sb21 | C201 | N202 | C204 | 7.7(11) |
| Sb21 | C208 | N203 | C209 | -178.8(7) |
| Sb21 | C208 | N203 | B201 | 1.3(10) |
| Sb21 | C208 | N204 | C210 | 179.5(8) |
| Sb21 | C208 | N204 | C211 | 6.4(11) |
| Sb21 | C229 | C230 | F201 | -4.9(9) |
| Sb21 | C229 | C230 | C231 | 175.3(9) |
| Sb21 | C229 | C234 | F205 | 8.0(8) |
| Sb21 | C229 | C234 | C233 | -175.6(8) |
| F201 | C230 | C229 | C234 | -177.4(10) |
| F201 | C230 | C231 | F202 | -0.4(12) |
| F201 | C230 | C231 | C232 | 178.8(10) |
| F202 | C231 | C230 | C229 | 179.4(10) |
| F202 | C231 | C232 | F203 | 1.5(12) |
| F202 | C231 | C232 | C233 | 178.9(10) |
| F203 | C232 | C231 | C230 | -177.7(10) |
| F203 | C232 | C233 | F204 | -0.9(12) |
| F203 | C232 | C233 | C234 | 177.8(10) |
| F204 | C233 | C232 | C231 | -178.3(10) |
| F204 | C233 | C234 | F205 | -3.7(13) |
| F204 | C233 | C234 | C229 | 179.9(10) |
| F205 | C234 | C229 | C230 | -179.1(10) |
| F205 | C234 | C233 | C232 | 177.6(9) |
| N201 | C201 | N202 | C203 | 1.1(8) |
| N201 | C201 | N202 | C204 | -179.4(7) |
| N201 | C202 | C203 | N202 | 0.9(8) |
| N201 | B201 | N203 | C208 | -34.9(11) |
| N201 | B201 | N203 | C209 | 145.2(8) |
| N201 | B201 | C215 | C216 | -36.6(10) |
| N201 | B201 | C215 | C220 | 148.5(8) |
| N201 | B201 | C222 | C223 | 130.5(7) |
| N201 | B201 | C222 | C227 | -50.3(8) |
| N203 | C208 | N204 | C210 | -1.6(8) |
| N203 | C208 | N204 | C211 | -174.6(7) |
| N203 | C209 | C210 | N204 | 0.9(8) |
| N203 | B201 | C215 | C216 | -158.2(8) |
| N203 | B201 | C215 | C220 | 27.0(10) |
| N203 | B201 | C222 | C223 | -111.9(7) |
| N203 | B201 | C222 | C227 | 67.3(8) |
| C215 | C216 | C217 | C218 | -0.6(13) |
| C215 | C220 | C219 | C218 | -0.2(12) |
| C215 | B201 | C222 | C223 | 8.3(9) |
| C215 | B201 | C222 | C227 | -172.6(7) |
| C216 | C217 | C218 | C219 | -0.1(14) |
| C216 | C217 | C218 | C221 | -177.8(12) |
| C217 | C218 | C219 | C220 | 0.5(13) |
| C222 | C223 | C224 | C225 | 0.8(11) |

| | | | | |
|---|---|---|---|---|
| C222 | C227 | C226 | C225 | -0.1(11) |
| C223 | C224 | C225 | C226 | -0.6(11) |
| C223 | C224 | C225 | C228 | 179.2(9) |
| C224 | C225 | C226 | C227 | 0.2(10) |
| C229 | C230 | C231 | C232 | -1.4(14) |
| C229 | C234 | C233 | C232 | 1.1(13) |
| C230 | C231 | C232 | C233 | -0.3(13) |
| C231 | C232 | C233 | C234 | 0.5(13) |
| Sb11 | C101 | N101 | C102 | 173.1(7) |
| Sb11 | C101 | N101 | B101 | -15.9(10) |
| Sb11 | C101 | N102 | C103 | -171.7(8) |
| Sb11 | C101 | N102 | C104 | 7.6(11) |
| Sb11 | C108 | N103 | C109 | 179.8(7) |
| Sb11 | C108 | N103 | B101 | 2.5(10) |
| Sb11 | C108 | N104 | C110 | 179.3(8) |
| Sb11 | C108 | N104 | C111 | 2.1(11) |
| Sb11 | C129 | C130 | F101 | 2.9(7) |
| Sb11 | C129 | C130 | C131 | -178.7(7) |
| Sb11 | C129 | C134 | F105 | -3.4(6) |
| Sb11 | C129 | C134 | C133 | 179.0(6) |
| F101 | C130 | C129 | C134 | -179.5(8) |
| F101 | C130 | C131 | F102 | -1.4(9) |
| F101 | C130 | C131 | C132 | -179.6(8) |
| F102 | C131 | C130 | C129 | -179.9(8) |
| F102 | C131 | C132 | F103 | 0.2(9) |
| F102 | C131 | C132 | C133 | 179.0(8) |
| F103 | C132 | C131 | C130 | 178.5(8) |
| F103 | C132 | C133 | F104 | 1.5(9) |
| F103 | C132 | C133 | C134 | -178.4(8) |
| F104 | C133 | C132 | C131 | -177.2(8) |
| F104 | C133 | C134 | F105 | 0.2(9) |
| F104 | C133 | C134 | C129 | 177.9(7) |
| F105 | C134 | C129 | C130 | 178.8(8) |
| F105 | C134 | C133 | C132 | -179.8(7) |
| N101 | C101 | N102 | C103 | -0.5(8) |
| N101 | C101 | N102 | C104 | 178.8(7) |
| N101 | C102 | C103 | N102 | 1.0(8) |
| N101 | B101 | N103 | C108 | -29.8(11) |
| N101 | B101 | N103 | C109 | 153.1(7) |
| N101 | B101 | C115 | C116 | -38.8(8) |
| N101 | B101 | C115 | C120 | 145.5(6) |
| N101 | B101 | C122 | C123 | 133.7(7) |
| N101 | B101 | C122 | C127 | -51.3(9) |
| N103 | C108 | N104 | C110 | 1.3(8) |
| N103 | C108 | N104 | C111 | -175.9(6) |
| N103 | C109 | C110 | N104 | -1.2(8) |
| N103 | B101 | C115 | C116 | -159.7(7) |
| N103 | B101 | C115 | C120 | 24.6(9) |
| N103 | B101 | C122 | C123 | -109.9(8) |
| N103 | B101 | C122 | C127 | 65.1(8) |
| C115 | C116 | C117 | C118 | 0.8(10) |
| C115 | C120 | C119 | C118 | -0.1(10) |
| C115 | B101 | C122 | C123 | 13.0(11) |
| C115 | B101 | C122 | C127 | -172.0(8) |
| C116 | C117 | C118 | C119 | 1.0(11) |
| C116 | C117 | C118 | C121 | -177.4(9) |

| | | | | |
|---|---|---|---|---|
| C117 | C118 | C119 | C120 | -1.3(10) |
| C122 | C123 | C124 | C125 | 0.2(12) |
| C122 | C127 | C126 | C125 | 2.5(12) |
| C123 | C124 | C125 | C126 | 1.0(13) |
| C123 | C124 | C125 | C128 | 178.8(12) |
| C124 | C125 | C126 | C127 | -2.3(13) |
| C129 | C130 | C131 | C132 | 1.9(10) |
| C129 | C134 | C133 | C132 | -2.1(10) |
| C130 | C131 | C132 | C133 | -2.7(10) |
| C131 | C132 | C133 | C134 | 2.9(10) |
| O401 | C402 | O402 | C403 | 23(2) |

### 9.13.12 Hydrogen Fractional Atomic Coordinates (×10$^4$) and Equivalent Isotropic Displacement Parameters

Table S91. Hydrogen Fractional Atomic Coordinates (×10$^4$) and Equivalent Isotropic Displacement Parameters (Å$^2$×10$^3$) for **16d**. $U_{eq}$ is defined as 1/3 of the trace of the orthogonalised $U_{ij}$.

| Atom | x | y | z | $U_{eq}$ |
|---|---|---|---|---|
| H302 | 4216(7) | 908(6) | 7320(4) | 89(3) |
| H303 | 4066(8) | 2101(6) | 6739(4) | 92(3) |
| H30g | 7542(11) | 2110(30) | 6220(40) | 168(6) |
| H30h | 7131(19) | 3010(20) | 6260(40) | 168(6) |
| H30i | 7230(20) | 2500(50) | 6980(7) | 168(6) |
| H30d | 5810(70) | 2960(30) | 5554(15) | 164(7) |
| H30e | 6120(60) | 2070(30) | 5561(16) | 164(7) |
| H30f | 4850(20) | 2530(50) | 5813(4) | 164(7) |
| H30a | 5350(50) | 3568(6) | 6700(30) | 141(5) |
| H30b | 4425(10) | 3090(30) | 6880(30) | 141(5) |
| H30c | 5400(50) | 3030(20) | 7389(11) | 141(5) |
| H309 | 6897(7) | -793(5) | 8585(4) | 85(3) |
| H310 | 8562(9) | -724(5) | 9073(4) | 94(3) |
| H31g | 9910(30) | 670(50) | 9387(16) | 153(5) |
| H31h | 8590(40) | 920(30) | 9270(10) | 153(5) |
| H31i | 9130(70) | 83(15) | 9543(9) | 153(5) |
| H31a | 10510(30) | 940(30) | 8250(30) | 132(4) |
| H31b | 9900(60) | 718(17) | 7616(6) | 132(4) |
| H31c | 9180(30) | 1303(11) | 8200(30) | 132(4) |
| H31d | 10630(40) | -480(20) | 7964(7) | 132(4) |
| H31e | 11099(19) | -248(13) | 8650(30) | 132(4) |
| H31f | 10220(20) | -774(11) | 8740(30) | 132(4) |
| H316 | 5215(9) | 133(5) | 6449(4) | 98(3) |
| H317 | 5706(10) | -513(6) | 5441(4) | 107(4) |
| H319 | 8400(9) | -1703(5) | 6200(5) | 104(4) |
| H320 | 7919(8) | -1044(5) | 7205(4) | 89(3) |
| H32a | 6726(15) | -1630(40) | 4950(30) | 164(7) |
| H32b | 7780(70) | -1250(20) | 4766(17) | 164(7) |
| H32c | 7940(60) | -2020(20) | 5217(12) | 164(7) |
| H323 | 5172(9) | -943(6) | 7588(5) | 100(3) |
| H324 | 3862(9) | -1205(6) | 8444(5) | 106(4) |
| H326 | 3841(9) | 587(6) | 9431(5) | 105(4) |
| H327 | 5179(8) | 813(5) | 8616(4) | 93(3) |
| H32d | 2720(60) | -930(30) | 9390(30) | 171(7) |
| H32e | 3240(30) | -550(50) | 9972(10) | 171(7) |
| H32f | 2260(30) | -50(20) | 9530(30) | 171(7) |
| H202 | 3591(7) | 1910(5) | 3240(4) | 87(3) |
| H203 | 5514(8) | 1325(5) | 3412(5) | 99(3) |
| H20a | 7270(60) | 2990(30) | 2825(13) | 148(5) |
| H20b | 6880(40) | 3148(18) | 3660(30) | 148(5) |
| H20c | 8120(20) | 2624(10) | 3400(40) | 148(5) |
| H20g | 7090(50) | 1220(20) | 2870(30) | 160(6) |
| H20h | 7310(60) | 1895(16) | 2361(6) | 160(6) |
| H20i | 8233(14) | 1480(40) | 2870(30) | 160(6) |
| H20d | 6410(60) | 2160(30) | 4393(9) | 169(7) |
| H20e | 6760(80) | 1350(30) | 4054(15) | 169(7) |
| H20f | 7730(20) | 1760(50) | 4170(20) | 169(7) |
| H209 | 1686(8) | 4164(5) | 2225(4) | 91(3) |

| | | | | |
|---|---|---|---|---|
| H210 | 2294(8) | 5139(5) | 1527(4) | 89(3) |
| H21a | 3714(11) | 5050(40) | 752(13) | 132(5) |
| H21b | 4830(60) | 5370(30) | 618(7) | 132(5) |
| H21c | 4940(60) | 4511(17) | 871(8) | 132(5) |
| H21g | 5790(20) | 4648(9) | 1840(30) | 133(5) |
| H21h | 5770(30) | 5490(30) | 1570(20) | 133(5) |
| H21i | 5229(9) | 5330(40) | 2364(13) | 133(5) |
| H21d | 4060(30) | 6309(6) | 1450(30) | 134(5) |
| H21e | 2930(20) | 6016(11) | 1530(30) | 134(5) |
| H21f | 3530(50) | 6080(14) | 2216(6) | 134(5) |
| H216 | 3282(8) | 2729(5) | 4287(4) | 91(3) |
| H217 | 2504(9) | 3088(6) | 5378(4) | 109(4) |
| H219 | 1108(9) | 5048(6) | 4610(5) | 107(4) |
| H220 | 1876(7) | 4680(5) | 3509(4) | 83(3) |
| H22d | 760(60) | 4030(40) | 5940(20) | 176(7) |
| H22e | 1928(15) | 4230(50) | 6054(14) | 176(7) |
| H22f | 920(80) | 4856(15) | 5737(9) | 176(7) |
| H223 | 1044(7) | 3314(5) | 3564(4) | 80(3) |
| H224 | -170(7) | 2790(5) | 3119(4) | 88(3) |
| H226 | 2122(8) | 2069(5) | 1538(4) | 88(3) |
| H227 | 3297(8) | 2605(5) | 1986(4) | 84(3) |
| H22a | 90(40) | 1600(20) | 2320(20) | 123(4) |
| H22b | -604(16) | 2402(13) | 2030(30) | 123(4) |
| H22c | 450(30) | 1900(30) | 1534(12) | 123(4) |
| H102 | -3877(7) | 7812(5) | 1986(4) | 84(3) |
| H103 | -5048(7) | 7811(5) | 1066(4) | 85(3) |
| H10a | -3674(11) | 6650(30) | -964(18) | 110(4) |
| H10b | -2690(30) | 7021(14) | -790(20) | 110(4) |
| H10c | -2920(40) | 6310(15) | -336(8) | 110(4) |
| H10d | -4760(70) | 7915(10) | -830(30) | 162(6) |
| H10e | -4940(60) | 8278(15) | -62(18) | 162(6) |
| H10f | -3726(14) | 8150(20) | -540(40) | 162(6) |
| H10g | -5530(30) | 7250(20) | 410(30) | 144(5) |
| H10h | -5300(40) | 6860(40) | -348(7) | 144(5) |
| H10i | -4686(13) | 6433(18) | 310(30) | 144(5) |
| H109 | 50(7) | 7508(5) | 2120(4) | 82(3) |
| H110 | 1668(8) | 7490(5) | 1250(4) | 88(3) |
| H11g | 1970(50) | 7997(17) | 264(16) | 125(4) |
| H11h | 2190(40) | 7841(11) | -570(20) | 125(4) |
| H11i | 934(13) | 8210(9) | -210(30) | 125(4) |
| H11d | 1660(20) | 6890(30) | -998(8) | 102(3) |
| H11e | 920(50) | 6425(15) | -491(12) | 102(3) |
| H11f | 380(30) | 7282(18) | -692(19) | 102(3) |
| H11a | 2349(13) | 6131(8) | 240(30) | 111(4) |
| H11b | 3058(17) | 6610(30) | -280(13) | 111(4) |
| H11c | 2770(30) | 6799(18) | 550(20) | 111(4) |
| H116 | -3247(8) | 6533(5) | 2208(4) | 89(3) |
| H117 | -3154(8) | 5445(5) | 2757(4) | 93(3) |
| H119 | 106(8) | 5077(5) | 2646(4) | 90(3) |
| H120 | 21(8) | 6172(5) | 2080(4) | 86(3) |
| H12a | -1650(60) | 4144(14) | 2731(15) | 137(5) |
| H12b | -698(19) | 4254(18) | 3190(30) | 137(5) |
| H12c | -2010(40) | 4533(7) | 3490(20) | 137(5) |
| H123 | -1784(8) | 7268(6) | 3093(4) | 92(3) |
| H124 | -2083(9) | 8270(7) | 3760(4) | 108(4) |
| H126 | -2574(8) | 9566(5) | 2088(4) | 89(3) |

| | | | | |
|---|---|---|---|---|
| H127 | -2205(7) | 8557(5) | 1426(4) | 86(3) |
| H12d | -2900(80) | 9526(19) | 3870(18) | 180(7) |
| H12e | -1811(16) | 9760(30) | 3430(40) | 180(7) |
| H12f | -3060(70) | 10050(16) | 3160(30) | 180(7) |
| H40c | 2120(60) | 6040(60) | 3270(40) | 257(10) |
| H40d | 1050(50) | 5870(40) | 2970(70) | 257(10) |
| H40e | 1660(110) | 6474(19) | 2570(30) | 257(10) |
| H40a | 401(18) | 7697(12) | 4375(8) | 222(9) |
| H40b | -625(18) | 7304(12) | 4472(8) | 222(9) |
| H40f | 1200(70) | 7060(110) | 5300(30) | 308(14) |
| H40g | -140(110) | 7340(70) | 5570(9) | 308(14) |
| H40h | 390(170) | 6490(40) | 5330(40) | 308(14) |

## 9.14 Single crystal structure analysis of 16e

CCDC Deposition number 2423162

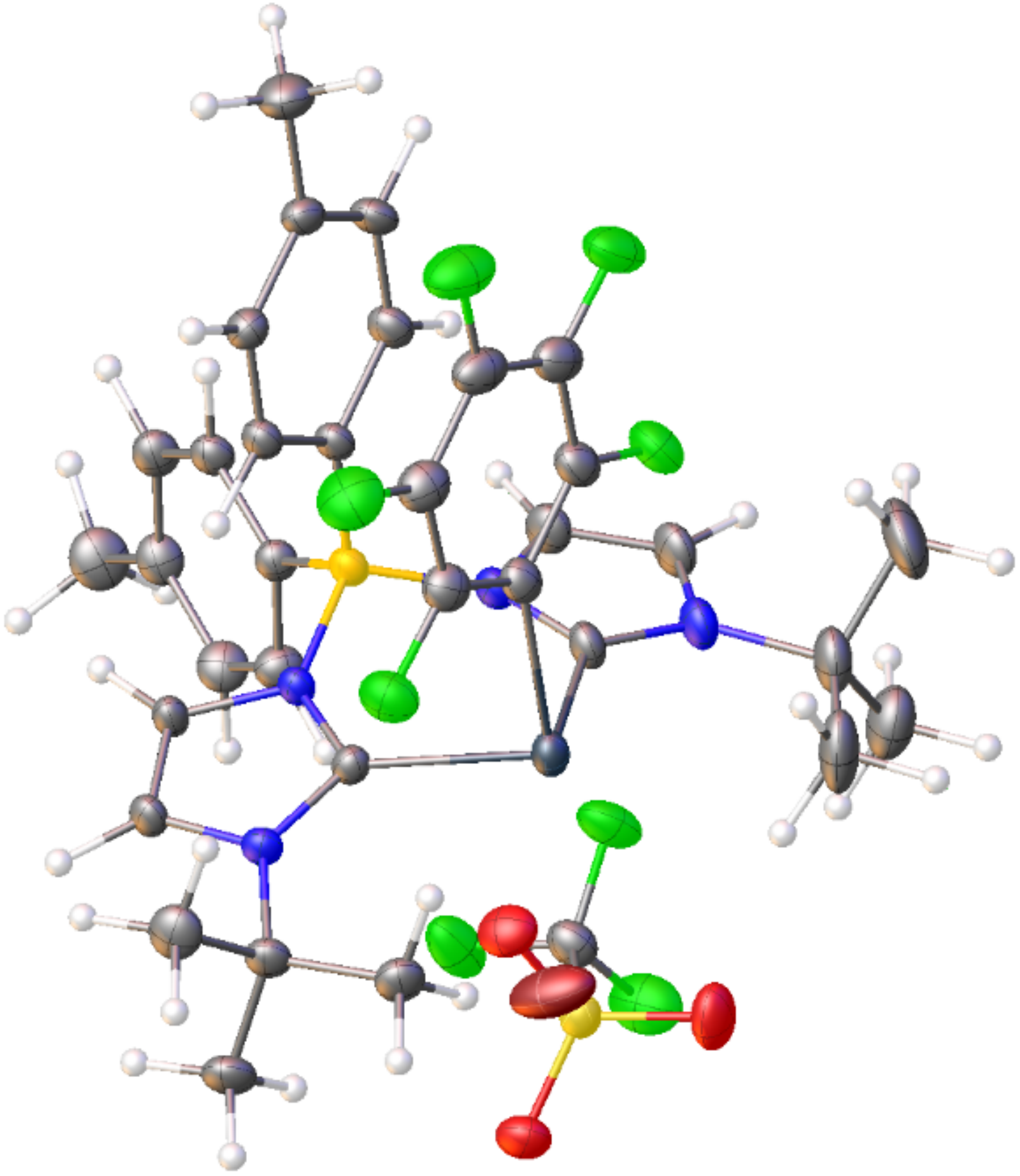

Figure S39. Olex-2 representation of the molecular structure of complex **16e**

### 9.14.1 Experimental.

Single colourless plate-shaped crystals of **16e** The crystal was chosen from the sample as supplied. A suitable crystal with dimensions 0.10 × 0.08 × 0.01 $mm^3$ was selected and mounted on a MITIGEN holder in oil on a Rigaku FRE+ diffractometer with Arc)Sec VHF Varimax confocal mirrors, a UG2 goniometer and HyPix 6000HE detector diffractometer. The crystal was kept at a steady $T$ = 100(2) K during data collection. The structure was solved with the ShelXT 2018/2 (Sheldrick, 2015) solution program using dual methods and by using Olex2 1.5-dev (Dolomanov et al., 2009) as the graphical interface. The model was refined with olex2.refine 1.5-dev (Bourhis et al., 2015) using full matrix least squares minimisation on $\boldsymbol{F}^2$.

9.14.2 Crystal Data.

$C_{36.5}H_{39.2}BBr_{0.1}F_{7.69}N_4O_{3.49}S_{0.9}Sb$, $M_r$ = 905.457, triclinic, $P$-1 (No. 2), a = 10.6884(4) Å, b = 12.8699(4) Å, c = 14.6458(5) Å, $\alpha$ = 82.607(3)°, $\beta$ = 73.092(3)°, $\gamma$ = 84.724(3)°, $V$ = 1908.39(12) Å$^3$, $T$ = 100(2) K, $Z$ = 2, $Z'$ = 1, $\mu$(Mo $K_\alpha$) = 0.960, 68971 reflections measured, 13273 unique ($R_{int}$ = 0.0746) which were used in all calculations. The final $wR_2$ was 0.0796 (all data) and $R_1$ was 0.0400 (I≥2 $\sigma$(I)).

| Compound | **16e** |
|---|---|
| | |
| Formula | $C_{36.5}H_{39.2}BBr_{0.1}F_{7.69}N_4O_{3.49}S_{0.9}Sb$ |
| $D_{calc.}$/ g cm$^{-3}$ | 1.576 |
| $\mu$/mm$^{-1}$ | 0.960 |
| Formula Weight | 905.457 |
| Colour | colourless |
| Shape | plate-shaped |
| Size/mm$^3$ | 0.10×0.08×0.01 |
| $T$/K | 100(2) |
| Crystal System | triclinic |
| Space Group | $P$-1 |
| $a$/Å | 10.6884(4) |
| $b$/Å | 12.8699(4) |
| $c$/Å | 14.6458(5) |
| $\alpha$/° | 82.607(3) |
| $\beta$/° | 73.092(3) |
| $\gamma$/° | 84.724(3) |
| V/Å$^3$ | 1908.39(12) |
| $Z$ | 2 |
| $Z'$ | 1 |
| Wavelength/Å | 0.71073 |
| Radiation type | Mo $K_\alpha$ |
| $\Theta_{min}$/° | 1.60 |
| $\Theta_{max}$/° | 32.03 |
| Measured Refl's. | 68971 |
| Indep't Refl's | 13273 |
| Refl's I≥2 $\sigma$(I) | 9947 |
| $R_{int}$ | 0.0746 |
| Parameters | 506 |
| Restraints | 0 |
| Largest Peak | 0.8206 |
| Deepest Hole | -0.7201 |
| GooF | 0.9913 |
| $wR_2$ (all data) | 0.0796 |
| $wR_2$ | 0.0725 |
| $R_1$ (all data) | 0.0658 |
| $R_1$ | 0.0400 |

### 9.14.3 Structure Quality Indicators

| | | | | |
|---|---|---|---|---|
| Reflections: | d min (MoKα) 2Θ=64.1° 0.67 | I/σ(I) 16.1 | Rint m=5.20 7.46% | Full 50.5° 100 |
| Refinement: | Shift -0.000 | Max Peak 0.8 | Min Peak -0.7 | GooF 0.991 |

A colourless plate-shaped-shaped crystal with dimensions 0.10 × 0.08 × 0.01 $mm^3$ was mounted on a MITIGEN holder in oil. Data were collected using a Rigaku FRE+ diffractometer with Arc)Sec VHF Varimax confocal mirrors, a UG2 goniometer and HyPix 6000HE detector diffractometer equipped with an Oxford Cryosystems low-temperature device operating at $T$ = 100(2) K.

Data were measured using profile data from $\omega$-scans with Mo $K_\alpha$ radiation. The diffraction pattern was indexed and the total number of runs and images was based on the strategy calculation from the program CrysAlisPro system (CCD 43.143a 64-bit (release 25-10-2024)). The maximum resolution that was achieved was $\Theta = 32.03^{\circ}$ (0.67 Å).

The unit cell was refined using CrysAlisPro 1.171.43.143a (Rigaku OD, 2024) on 21155 reflections, 31% of the observed reflections.

Data reduction, scaling and absorption corrections were performed using CrysAlisPro 1.171.43.143a (Rigaku OD, 2024). The final completeness is 99.97 % out to 32.03° in $\Theta$. A multi-scan absorption correction was performed using CrysAlisPro 1.171.43.143a (Rigaku OD, 2024) Empirical absorption correction using spherical harmonics, implemented in SCALE3 ABSPACK scaling algorithm. The absorption coefficient $\mu$ of this material is 0.960 $mm^{-1}$ at this wavelength ($\lambda$ = 0.71073Å) and the minimum and maximum transmissions are 0.750 and 1.000.

The structure was solved and the space group *P*-1 (# 2) determined by the ShelXT 2018/2 (Sheldrick, 2015) structure solution program using dual methods and refined by full matrix least squares minimisation on $F^2$ using version of olex2.refine 1.5-dev (Bourhis et al., 2015). All non-hydrogen atoms were refined anisotropically. Hydrogen atom positions were calculated geometrically and refined using the riding model.

*_refine_special_details*: OTf- and Br- counterions disordered (ca. 90:10). Solvent masking applied to eliminate the electron contribution equivalent to 0.4 ethyl acetate.

*_olex2_refine_details*: Refinement using NoSpherA2, an implementation of NOn-SPHERical Atom-form-factors in Olex2.Please cite: F. Kleemiss et al. Chem. Sci. DOI 10.1039/D0SC05526C – 2021 NoSpherA2 implementation of HAR makes use of tailor-made aspherical atomic form factors calculated on-the-fly from a Hirshfeld-partitioned electron density (ED) - not from spherical-atom form factors. The ED is calculated from a gaussian basis set single determinant SCF wavefunction - either Hartree-Fock or DFT using selected functionals- for a fragment of the crystal. This fragment can be embedded in an electrostatic crystal field by employing cluster charges or modelled using implicit solvation models, depending on the software used. The following options were used: SOFTWARE: ORCA 6.0 ;;; PARTITIONING: NoSpherA2 ;;; INT ACCURACY: Normal ;;; METHOD:

r2SCAN ;;; BASIS SET: x2c-TZVP ;;; CHARGE: 0 ;;; MULTIPLICITY: 1 ;;; DATE: 2024-11-29_18-01-56

*_exptl_absorpt_process_details*: CrysAlisPro 1.171.43.143a (Rigaku OD, 2024) using spherical harmonics, implemented in SCALE3 ABSPACK scaling algorithm.

*_smtbx_masks_special_details*: A solvent mask was calculated and 36 electrons were found in a volume of 146Å$^3$ in 1 void per unit cell. This is consistent with the presence of 0.4[C4H8O2] per Asymmetric Unit which account for 38 electrons per unit cell.

There is a single formula unit in the asymmetric unit, which is represented by the reported sum formula. In other words: Z is 2 and Z' is 1. The moiety formula is C34 H36 B F5 N4 Sb, 0.897(C F3 O3 S), 0.103(Br), 0.4[C4H8O2].

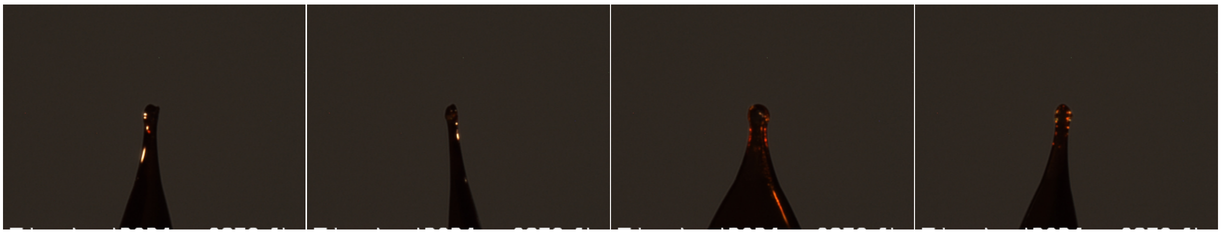

9.14.4 Fractional Atomic Coordinates (×10[4]) and Equivalent Isotropic Displacement Parameters

Table S92. Fractional Atomic Coordinates (×10[4]) and Equivalent Isotropic Displacement Parameters (Å$^2$×10$^3$) for **16e**. $U_{eq}$ is defined as 1/3 of the trace of the orthogonalised $U_{ij}$.

| Atom | x | y | z | $U_{eq}$ |
|---|---|---|---|---|
| Sb1 | 3716.4(3) | 7674.4(2) | 8215.06(17) | 23.21(4) |
| F1 | 6651.2(12) | 6660.4(10) | 7841.6(8) | 36.0(3) |
| F2 | 7788.8(14) | 4791.5(12) | 8246.2(10) | 46.9(3) |
| F3 | 6295.3(15) | 3162.3(11) | 9159.4(10) | 49.9(4) |
| F4 | 3649.3(14) | 3456.1(10) | 9719.2(9) | 44.2(3) |
| F5 | 2497.6(12) | 5323.5(10) | 9344.9(8) | 38.7(3) |
| N1 | 4083.8(14) | 7023.9(12) | 6191.6(9) | 19.8(3) |
| N2 | 5610.7(15) | 8095.5(12) | 6080.2(10) | 21.4(3) |
| N3 | 1836.6(15) | 6617.4(13) | 7415.9(10) | 23.4(3) |
| N4 | 705.0(16) | 7126.1(14) | 8806.0(11) | 29.2(4) |
| C1 | 4576.3(17) | 7601.2(15) | 6690.8(12) | 19.9(4) |
| C2 | 4832.0(18) | 7138.2(15) | 5252.0(12) | 22.9(4) |
| C3 | 5781.3(18) | 7801.9(15) | 5177.5(12) | 23.5(4) |
| C4 | 6493.7(19) | 8873.5(16) | 6212.6(13) | 25.7(4) |
| C5 | 6166(2) | 9125.4(17) | 7242.6(13) | 30.0(4) |
| C6 | 6318(2) | 9885.9(16) | 5583.5(14) | 33.6(5) |
| C7 | 7896(2) | 8401.3(18) | 5895.0(15) | 33.9(5) |
| C8 | 1912.2(19) | 7087.6(16) | 8159.4(12) | 24.6(4) |
| C9 | 562.9(19) | 6347.9(17) | 7590.2(14) | 29.9(4) |
| C10 | -132(2) | 6657.6(18) | 8451.0(14) | 32.7(5) |
| C11 | 222(2) | 7527(2) | 9776.0(14) | 39.5(6) |
| C12 | -973(2) | 8269(2) | 9783.0(17) | 51.3(7) |
| C13 | 1243(3) | 8111(2) | 9994.0(16) | 54.6(8) |
| C14 | -144(3) | 6578(2) | 10530.9(16) | 64.6(9) |
| C15 | 3538.6(18) | 5072.1(15) | 6817.5(11) | 20.4(4) |
| C16 | 4885.6(18) | 4803.8(15) | 6614.9(12) | 22.4(4) |
| C17 | 5368.4(19) | 3791.7(15) | 6850.1(12) | 24.3(4) |
| C18 | 4520(2) | 2999.8(15) | 7285.1(12) | 25.4(4) |
| C19 | 3182(2) | 3244.8(16) | 7482.9(13) | 27.2(4) |
| C20 | 2707.2(19) | 4258.8(16) | 7261.0(12) | 25.3(4) |
| C21 | 5040(3) | 1902.2(18) | 7526.5(16) | 40.9(6) |
| C22 | 2274.5(18) | 6289.9(15) | 5652.6(12) | 21.9(4) |
| C23 | 2103.3(18) | 5428.9(16) | 5226.7(12) | 23.6(4) |
| C24 | 1462.0(19) | 5529.3(17) | 4511.9(13) | 27.9(4) |
| C25 | 973.8(19) | 6507.2(17) | 4187.6(13) | 27.3(4) |
| C26 | 1148.4(19) | 7379.4(18) | 4604.2(13) | 29.5(4) |
| C27 | 1785.2(19) | 7272.5(16) | 5315.8(13) | 27.3(4) |
| C28 | 280(2) | 6624(2) | 3416.7(15) | 40.0(6) |
| C29 | 4528.0(19) | 6071.5(16) | 8549.1(12) | 24.3(4) |
| C30 | 5869(2) | 5887.0(17) | 8304.4(13) | 28.2(4) |
| C31 | 6490(2) | 4923.9(18) | 8498.7(14) | 32.5(5) |
| C32 | 5730(2) | 4094.5(17) | 8967.1(14) | 34.5(5) |
| C33 | 4377(2) | 4245.0(17) | 9242.4(13) | 31.8(5) |
| C34 | 3808(2) | 5225.6(17) | 9035.0(13) | 28.7(4) |
| B1 | 2954(2) | 6225.7(17) | 6515.0(13) | 20.3(4) |
| S51a | 1746.3(6) | 10232.0(5) | 7342.3(4) | 25.16(14) |
| F51a | -635.8(14) | 10559.5(13) | 7165.7(11) | 48.8(4) |
| F52a | -39.1(14) | 8940.5(11) | 7376.2(9) | 39.4(4) |
| F53a | 715.1(15) | 9823.4(13) | 6016.1(9) | 45.9(4) |
| O51a | 2143.2(16) | 11175.6(13) | 6731.8(11) | 35.2(4) |

| | | | | |
|---|---|---|---|---|
| O52a | 2672.3(18) | 9349.1(18) | 7154.3(14) | 36.7(4) |
| O53a | 1167.2(18) | 10345.7(16) | 8339.6(11) | 45.2(5) |
| C51a | 373(2) | 9867.8(19) | 6954.3(15) | 29.5(5) |
| Br51b | 2619(3) | 9871(3) | 7425(3) | 61.8(12) |

9.14.5 Anisotropic Displacement Parameters

Table S93. Anisotropic Displacement Parameters (×$10^4$) for **16e**. The anisotropic displacement factor exponent takes the form: $-2\pi^2[h^2a^{*2} \times U_{11} + ... +2hka^* \times b^* \times U_{12}]$

| Atom | $U_{11}$ | $U_{22}$ | $U_{33}$ | $U_{23}$ | $U_{13}$ | $U_{12}$ |
|---|---|---|---|---|---|---|
| Sb1 | 23.63(7) | 27.24(8) | 18.33(6) | -4.64(5) | -3.24(4) | -4.89(4) |
| F1 | 30.2(7) | 35.5(8) | 42.2(7) | -6.6(6) | -11.0(5) | 1.6(6) |
| F2 | 40.2(8) | 45.9(9) | 57.0(8) | 7.5(7) | -20.0(6) | -6.5(7) |
| F3 | 70.5(11) | 31.9(8) | 53.7(8) | 5.1(7) | -30.6(7) | -3.4(6) |
| F4 | 65.7(10) | 32.0(8) | 36.1(6) | -16.5(7) | -16.1(6) | 5.8(5) |
| F5 | 38.5(7) | 38.7(8) | 33.6(6) | -11.2(6) | -3.5(5) | 6.1(5) |
| N1 | 19.2(8) | 20.6(8) | 18.5(6) | -5.9(6) | -1.9(6) | -2.8(6) |
| N2 | 21.1(8) | 20.2(8) | 22.5(7) | -6.3(6) | -3.6(6) | -3.0(6) |
| N3 | 20.4(8) | 24.4(9) | 22.7(7) | -7.0(6) | -0.4(6) | -2.1(6) |
| N4 | 24.4(9) | 35.7(11) | 22.1(7) | -5.7(7) | 2.8(6) | -2.9(7) |
| C1 | 20.1(9) | 20.3(9) | 19.4(8) | -3.7(7) | -3.7(7) | -4.9(7) |
| C2 | 25.7(10) | 24.5(10) | 17.2(8) | -8.9(8) | -1.2(7) | -4.1(7) |
| C3 | 21.4(9) | 25.3(10) | 21.3(8) | -8.8(8) | 0.9(7) | -3.7(7) |
| C4 | 27.2(10) | 22.8(10) | 27.5(9) | -9.8(8) | -5.6(8) | -3.0(7) |
| C5 | 33.3(11) | 26.9(11) | 31.8(10) | -9.7(9) | -8.1(8) | -6.9(8) |
| C6 | 41.1(13) | 23.8(11) | 36.0(10) | -13.8(9) | -7.8(9) | -1.9(8) |
| C7 | 23.8(10) | 40.5(14) | 38.9(11) | -9.2(9) | -7.5(8) | -7.3(9) |
| C8 | 22.4(10) | 30.3(11) | 18.1(8) | -6.4(8) | 0.0(7) | -1.3(7) |
| C9 | 21.0(10) | 34.8(12) | 31.6(10) | -8.7(9) | -0.7(8) | -5.9(8) |
| C10 | 22.6(10) | 40.7(13) | 29.2(10) | -9.0(9) | 3.6(8) | -3.6(9) |
| C11 | 32.5(12) | 56.0(16) | 24.2(9) | -5.7(11) | 4.1(8) | -9.6(9) |
| C12 | 42.4(15) | 66.1(19) | 41.8(13) | -1.3(13) | -0.2(11) | -21.6(12) |
| C13 | 43.0(15) | 90(2) | 27.7(11) | -8.6(14) | 4.0(10) | -23.4(12) |
| C14 | 65(2) | 82(2) | 28.2(11) | -7.4(17) | 12.3(12) | 6.3(13) |
| C15 | 20.8(9) | 20.3(10) | 19.8(7) | -7.6(7) | -3.4(7) | -1.5(7) |
| C16 | 22.6(9) | 22.3(10) | 21.3(8) | -6.9(8) | -3.1(7) | -1.9(7) |
| C17 | 26.0(10) | 22.1(10) | 23.8(8) | -2.3(8) | -4.4(7) | -4.3(7) |
| C18 | 34.7(11) | 18.8(10) | 22.9(8) | -5.0(8) | -6.6(8) | -3.3(7) |
| C19 | 32.4(11) | 20.2(10) | 27.6(9) | -10.5(8) | -4.9(8) | 1.0(7) |
| C20 | 24.0(10) | 25.3(11) | 26.2(8) | -8.6(8) | -5.2(7) | -0.3(7) |
| C21 | 53.1(15) | 24.8(12) | 41.6(12) | -1.3(10) | -9.2(11) | -2.8(9) |
| C22 | 21.7(9) | 23.5(10) | 21.7(8) | -5.3(7) | -6.2(7) | -3.5(7) |
| C23 | 23.3(9) | 23.9(11) | 23.9(8) | -7.6(8) | -5.6(7) | -1.9(7) |
| C24 | 28.5(10) | 30.8(12) | 26.5(9) | -9.3(9) | -8.0(8) | -4.6(8) |
| C25 | 21.6(9) | 34.9(12) | 27.0(9) | -4.3(8) | -8.7(7) | -2.6(8) |
| C26 | 27.5(10) | 31.9(12) | 31.0(9) | 1.2(9) | -12.7(8) | -1.9(8) |
| C27 | 30.3(11) | 24.4(11) | 29.2(9) | -2.6(8) | -10.9(8) | -3.5(8) |
| C28 | 36.0(13) | 50.6(16) | 39.4(11) | -6.0(11) | -19.3(10) | -4.4(10) |
| C29 | 28.0(10) | 27.5(11) | 18.2(8) | -5.1(8) | -7.0(7) | -1.7(7) |
| C30 | 32.1(11) | 29.6(12) | 25.2(9) | -3.2(9) | -11.2(8) | -3.0(8) |
| C31 | 36.9(12) | 32.5(13) | 31.5(10) | 0.1(10) | -14.6(9) | -5.2(9) |
| C32 | 51.8(15) | 26.2(12) | 30.4(10) | -1.1(10) | -19.1(10) | -4.9(8) |
| C33 | 46.6(14) | 27.9(12) | 24.1(9) | -6.6(10) | -13.6(9) | -2.2(8) |
| C34 | 35.1(11) | 29.3(12) | 21.7(8) | -8.6(9) | -7.2(8) | -0.2(8) |
| B1 | 19.8(10) | 21.2(11) | 19.5(9) | -6.7(8) | -3.5(7) | -1.5(8) |
| S51a | 25.5(3) | 25.3(3) | 24.1(2) | -5.2(2) | -3.3(2) | -6.3(2) |
| F51a | 30.1(8) | 45.4(10) | 69.2(10) | 0.0(7) | -15.9(7) | 2.8(8) |
| F52a | 44.1(9) | 35.0(9) | 41.6(7) | -19.4(7) | -15.9(6) | 7.9(6) |
| F53a | 54.4(10) | 58.3(11) | 29.8(7) | -27.9(8) | -14.7(6) | 2.0(7) |

| | | | | | | |
|---|---|---|---|---|---|---|
| O51a | 37.2(10) | 26.5(9) | 40.1(9) | -11.4(7) | -5.3(7) | -2.6(7) |
| O52a | 30.6(10) | 31.4(12) | 47.7(11) | 2.7(9) | -10.9(8) | -5.5(9) |
| O53a | 48.7(11) | 60.1(13) | 26.8(8) | -13.4(10) | -2.3(7) | -16.8(8) |
| C51a | 27.8(12) | 31.1(13) | 29.4(10) | -9.0(10) | -7.2(9) | 0.7(9) |
| Br51b | 43(2) | 31.7(18) | 110(3) | 2.7(14) | -18.7(17) | -17.4(17) |

9.14.6 Bond lengths

Table S94. Bond Lengths in Å for **16e**.

| Atom | Atom | Length/Å |
|---|---|---|
| Sb1 | C1 | 2.1638(16) |
| Sb1 | C8 | 2.161(2) |
| Sb1 | C29 | 2.215(2) |
| F1 | C30 | 1.341(2) |
| F2 | C31 | 1.330(2) |
| F3 | C32 | 1.328(3) |
| F4 | C33 | 1.330(2) |
| F5 | C34 | 1.340(2) |
| N1 | C1 | 1.341(2) |
| N1 | C2 | 1.373(2) |
| N1 | B1 | 1.583(2) |
| N2 | C1 | 1.355(2) |
| N2 | C3 | 1.378(2) |
| N2 | C4 | 1.504(2) |
| N3 | C8 | 1.337(2) |
| N3 | C9 | 1.379(2) |
| N3 | B1 | 1.602(2) |
| N4 | C8 | 1.361(2) |
| N4 | C10 | 1.373(3) |
| N4 | C11 | 1.505(2) |
| C2 | C3 | 1.357(2) |
| C4 | C5 | 1.517(3) |
| C4 | C6 | 1.526(3) |
| C4 | C7 | 1.527(3) |
| C9 | C10 | 1.353(3) |
| C11 | C12 | 1.522(4) |
| C11 | C13 | 1.508(3) |
| C11 | C14 | 1.535(3) |
| C15 | C16 | 1.402(3) |
| C15 | C20 | 1.403(2) |
| C15 | B1 | 1.616(3) |
| C16 | C17 | 1.395(3) |
| C17 | C18 | 1.391(3) |
| C18 | C19 | 1.389(3) |
| C18 | C21 | 1.507(3) |
| C19 | C20 | 1.390(3) |
| C22 | C23 | 1.390(3) |
| C22 | C27 | 1.409(3) |
| C22 | B1 | 1.621(3) |
| C23 | C24 | 1.396(3) |
| C24 | C25 | 1.397(3) |
| C25 | C26 | 1.396(3) |
| C25 | C28 | 1.507(3) |
| C26 | C27 | 1.388(3) |
| C29 | C30 | 1.378(3) |
| C29 | C34 | 1.386(3) |
| C30 | C31 | 1.388(3) |
| C31 | C32 | 1.384(3) |
| C32 | C33 | 1.386(3) |
| C33 | C34 | 1.386(3) |
| S51a | O51a | 1.4314(16) |
| S51a | O52a | 1.436(2) |
| S51a | O53a | 1.4320(16) |
| S51a | C51a | 1.838(2) |
| F51a | C51a | 1.323(3) |
| F52a | C51a | 1.322(3) |
| F53a | C51a | 1.323(2) |

9.14.7 Bond Angles

Table S95. Bond Angles in ° for **16e**.

| Atom | Atom | Atom | Angle/° |
|---|---|---|---|
| C8 | Sb1 | C1 | 91.45(7) |
| C29 | Sb1 | C1 | 91.53(7) |
| C29 | Sb1 | C8 | 92.04(7) |
| C2 | N1 | C1 | 108.12(14) |
| B1 | N1 | C1 | 131.96(14) |
| B1 | N1 | C2 | 119.67(14) |
| C3 | N2 | C1 | 108.05(15) |
| C4 | N2 | C1 | 132.71(15) |
| C4 | N2 | C3 | 119.20(14) |
| C9 | N3 | C8 | 108.14(15) |
| B1 | N3 | C8 | 131.12(16) |
| B1 | N3 | C9 | 120.17(16) |
| C10 | N4 | C8 | 107.79(16) |
| C11 | N4 | C8 | 131.99(18) |
| C11 | N4 | C10 | 120.17(16) |
| N1 | C1 | Sb1 | 121.79(12) |
| N2 | C1 | Sb1 | 129.59(13) |
| N2 | C1 | N1 | 108.61(14) |
| C3 | C2 | N1 | 108.11(15) |
| C2 | C3 | N2 | 107.11(14) |
| C5 | C4 | N2 | 112.69(14) |
| C6 | C4 | N2 | 107.62(16) |
| C6 | C4 | C5 | 108.21(17) |
| C7 | C4 | N2 | 107.31(16) |
| C7 | C4 | C5 | 109.97(17) |
| C7 | C4 | C6 | 111.05(16) |
| N3 | C8 | Sb1 | 122.18(12) |
| N4 | C8 | Sb1 | 129.23(14) |
| N4 | C8 | N3 | 108.58(17) |
| C10 | C9 | N3 | 107.82(18) |
| C9 | C10 | N4 | 107.67(17) |
| C12 | C11 | N4 | 108.00(18) |
| C13 | C11 | N4 | 112.16(17) |
| C13 | C11 | C12 | 108.8(2) |
| C14 | C11 | N4 | 107.8(2) |
| C14 | C11 | C12 | 110.7(2) |
| C14 | C11 | C13 | 109.5(2) |
| C20 | C15 | C16 | 115.97(18) |
| B1 | C15 | C16 | 122.95(15) |
| B1 | C15 | C20 | 121.01(17) |
| C17 | C16 | C15 | 121.98(17) |
| C18 | C17 | C16 | 120.76(19) |
| C19 | C18 | C17 | 118.26(19) |
| C21 | C18 | C17 | 120.8(2) |
| C21 | C18 | C19 | 120.91(18) |
| C20 | C19 | C18 | 120.67(18) |
| C19 | C20 | C15 | 122.35(19) |
| C27 | C22 | C23 | 116.58(17) |
| B1 | C22 | C23 | 124.45(18) |
| B1 | C22 | C27 | 118.94(17) |
| C24 | C23 | C22 | 121.78(19) |
| C25 | C24 | C23 | 121.13(19) |
| C26 | C25 | C24 | 117.66(18) |
| C28 | C25 | C24 | 121.6(2) |
| C28 | C25 | C26 | 120.8(2) |
| C27 | C26 | C25 | 120.8(2) |
| C26 | C27 | C22 | 122.00(19) |
| C30 | C29 | Sb1 | 118.59(14) |
| C34 | C29 | Sb1 | 125.86(15) |
| C34 | C29 | C30 | 115.47(19) |
| C29 | C30 | F1 | 119.96(19) |
| C31 | C30 | F1 | 116.25(19) |
| C31 | C30 | C29 | 123.78(19) |
| C30 | C31 | F2 | 121.2(2) |
| C32 | C31 | F2 | 120.1(2) |
| C32 | C31 | C30 | 118.7(2) |
| C31 | C32 | F3 | 120.1(2) |
| C33 | C32 | F3 | 120.2(2) |
| C33 | C32 | C31 | 119.7(2) |
| C32 | C33 | F4 | 119.6(2) |
| C34 | C33 | F4 | 121.2(2) |
| C34 | C33 | C32 | 119.2(2) |
| C29 | C34 | F5 | 120.83(19) |
| C33 | C34 | F5 | 116.03(18) |
| C33 | C34 | C29 | 123.1(2) |
| N3 | B1 | N1 | 109.21(14) |
| C15 | B1 | N1 | 109.84(15) |
| C15 | B1 | N3 | 109.30(14) |
| C22 | B1 | N1 | 107.44(14) |
| C22 | B1 | N3 | 105.81(15) |
| C22 | B1 | C15 | 115.05(16) |
| O52a | S51a | O51a | 114.47(11) |
| O53a | S51a | O51a | 116.09(11) |
| O53a | S51a | O52a | 114.47(12) |
| C51a | S51a | O51a | 102.95(10) |
| C51a | S51a | O52a | 103.14(12) |
| C51a | S51a | O53a | 103.25(10) |
| F51a | C51a | S51a | 111.16(17) |
| F52a | C51a | S51a | 111.56(16) |
| F52a | C51a | F51a | 107.45(18) |
| F53a | C51a | S51a | 111.10(15) |
| F53a | C51a | F51a | 107.7(2) |
| F53a | C51a | F52a | 107.70(19) |

9.14.8 Torsion Angles

Table S96. Torsion Angles in ° for **16e**.

| Atom | Atom | Atom | Atom | Angle/° |
|---|---|---|---|---|
| Sb1 | C1 | N1 | C2 | 179.86(15) |
| Sb1 | C1 | N1 | B1 | -6.1(2) |
| Sb1 | C1 | N2 | C3 | -179.74(17) |
| Sb1 | C1 | N2 | C4 | -2.1(2) |
| Sb1 | C8 | N3 | C9 | -179.25(16) |
| Sb1 | C8 | N3 | B1 | 9.6(2) |
| Sb1 | C8 | N4 | C10 | 179.49(19) |
| Sb1 | C8 | N4 | C11 | -3.3(3) |
| Sb1 | C29 | C30 | F1 | -2.47(14) |
| Sb1 | C29 | C30 | C31 | 178.31(13) |
| Sb1 | C29 | C34 | F5 | 0.67(16) |
| Sb1 | C29 | C34 | C33 | -178.43(15) |
| F1 | C30 | C29 | C34 | -179.28(17) |
| F1 | C30 | C31 | F2 | 0.8(2) |
| F1 | C30 | C31 | C32 | -179.40(17) |
| F2 | C31 | C30 | C29 | -179.94(17) |
| F2 | C31 | C32 | F3 | -0.5(2) |
| F2 | C31 | C32 | C33 | 178.87(18) |
| F3 | C32 | C31 | C30 | 179.71(18) |
| F3 | C32 | C33 | F4 | 1.5(2) |
| F3 | C32 | C33 | C34 | 179.92(18) |
| F4 | C33 | C32 | C31 | -177.89(18) |
| F4 | C33 | C34 | F5 | 0.2(2) |
| F4 | C33 | C34 | C29 | 179.33(17) |
| F5 | C34 | C29 | C30 | 177.22(17) |
| F5 | C34 | C33 | C32 | -178.22(16) |
| N1 | C1 | N2 | C3 | -1.11(18) |
| N1 | C1 | N2 | C4 | 176.56(15) |
| N1 | C2 | C3 | N2 | -0.01(17) |
| N1 | B1 | N3 | C8 | -36.3(2) |
| N1 | B1 | N3 | C9 | 153.48(15) |
| N1 | B1 | C15 | C16 | -12.59(17) |
| N1 | B1 | C15 | C20 | 170.75(13) |
| N1 | B1 | C22 | C23 | 126.91(15) |
| N1 | B1 | C22 | C27 | -54.83(17) |
| N3 | C8 | N4 | C10 | 0.50(19) |
| N3 | C8 | N4 | C11 | 177.69(16) |
| N3 | C9 | C10 | N4 | 0.53(19) |
| N3 | B1 | C15 | C16 | -132.39(14) |
| N3 | B1 | C15 | C20 | 50.95(17) |
| N3 | B1 | C22 | C23 | -116.51(15) |
| N3 | B1 | C22 | C27 | 61.75(16) |
| C15 | C16 | C17 | C18 | 1.1(2) |
| C15 | C20 | C19 | C18 | 1.1(2) |
| C15 | B1 | C22 | C23 | 4.25(19) |
| C15 | B1 | C22 | C27 | -177.49(15) |
| C16 | C17 | C18 | C19 | -0.3(2) |
| C16 | C17 | C18 | C21 | 179.09(17) |
| C17 | C18 | C19 | C20 | -0.7(2) |
| C22 | C23 | C24 | C25 | 0.6(2) |
| C22 | C27 | C26 | C25 | -0.4(2) |
| C23 | C24 | C25 | C26 | -0.1(2) |

| | | | | |
|---|---|---|---|---|
| C23 | C24 | C25 | C28 | -179.99(18) |
| C24 | C25 | C26 | C27 | -0.0(2) |
| C29 | C30 | C31 | C32 | -0.2(2) |
| C29 | C34 | C33 | C32 | 0.9(2) |
| C30 | C31 | C32 | C33 | -0.9(2) |
| C31 | C32 | C33 | C34 | 0.5(2) |

### 9.14.9 Hydrogen Fractional Atomic Coordinates and Equivalent Isotropic Displacement Parameters

Table S97. Hydrogen Fractional Atomic Coordinates (×10[4]) and Equivalent Isotropic Displacement Parameters (Å$^2$×10$^3$) for **16e**. $U_{eq}$ is defined as 1/3 of the trace of the orthogonalised $U_{ij}$.

| Atom | x | y | z | $U_{eq}$ |
|---|---|---|---|---|
| H2 | 4689.6(18) | 6763.9(15) | 4671.3(12) | 27.4(5) |
| H3 | 6531.7(18) | 8053.4(15) | 4530.4(12) | 28.2(5) |
| H5a | 5170(5) | 9444(10) | 7465(3) | 45.0(7) |
| H5b | 6277(13) | 8419(3) | 7700.9(19) | 45.0(7) |
| H5c | 6819(9) | 9691(8) | 7293(2) | 45.0(7) |
| H6a | 6576(13) | 9730(3) | 4844.5(18) | 50.5(7) |
| H6b | 5311(4) | 10182(6) | 5801(7) | 50.5(7) |
| H6c | 6941(10) | 10461(4) | 5661(8) | 50.5(7) |
| H7a | 7991(4) | 7688(6) | 6348(7) | 50.9(7) |
| H7b | 8123(5) | 8231(10) | 5162(4) | 50.9(7) |
| H7c | 8563(2) | 8952(5) | 5951(10) | 50.9(7) |
| H9 | 185.6(19) | 5957.8(17) | 7121.5(14) | 35.9(5) |
| H10 | -1162(2) | 6554.5(18) | 8797.6(14) | 39.3(6) |
| H12a | -699(4) | 8923(7) | 9241(9) | 76.9(10) |
| H12b | -1714(7) | 7858(4) | 9635(12) | 76.9(10) |
| H12c | -1353(11) | 8553(11) | 10477(4) | 76.9(10) |
| H13a | 2070(8) | 7581(4) | 10034(13) | 82.0(11) |
| H13b | 1556(13) | 8741(9) | 9434(7) | 82.0(11) |
| H13c | 831(6) | 8430(12) | 10670(7) | 82.0(11) |
| H14a | -922(14) | 6192(10) | 10405(9) | 96.9(13) |
| H14b | 698(6) | 6037(8) | 10481(10) | 96.9(13) |
| H14c | -466(19) | 6839(3) | 11236.5(18) | 96.9(13) |
| H16 | 5571.6(18) | 5400.2(15) | 6265.4(12) | 26.9(5) |
| H17 | 6416.0(19) | 3620.8(15) | 6692.2(12) | 29.2(5) |
| H19 | 2501(2) | 2639.5(16) | 7813.5(13) | 32.7(5) |
| H20 | 1657.8(19) | 4427.2(16) | 7436.7(12) | 30.4(5) |
| H21a | 6046(5) | 1798(4) | 7109(8) | 61.3(8) |
| H21b | 4981(14) | 1780(4) | 8279(3) | 61.3(8) |
| H21c | 4465(9) | 1344.1(18) | 7364(10) | 61.3(8) |
| H23 | 2477.5(18) | 4659.3(16) | 5455.7(12) | 28.3(5) |
| H24 | 1340.9(19) | 4837.2(17) | 4203.5(13) | 33.5(5) |
| H26 | 782.3(19) | 8149.5(18) | 4369.3(13) | 35.4(5) |
| H27 | 1909.5(19) | 7965.7(16) | 5621.3(13) | 32.8(5) |
| H28a | 879(7) | 7033(11) | 2773(4) | 60.0(8) |
| H28b | 106(14) | 5859(2) | 3271(8) | 60.0(8) |
| H28c | -641(7) | 7064(11) | 3655(5) | 60.0(8) |

9.14.10 Atomic Occupancies for all atoms that are not fully occupied

Table S98. Atomic Occupancies for all atoms that are not fully occupied in **16e**.

| Atom | Occupancy |
|---|---|
| S51a | 0.8971(12) |
| F51a | 0.8971(12) |
| F52a | 0.8971(12) |
| F53a | 0.8971(12) |
| O51a | 0.8971(12) |
| O52a | 0.8971(12) |
| O53a | 0.8971(12) |
| C51a | 0.8971(12) |
| Br51b | 0.1029(12) |

### 9.14.11 Solvent masking (PLATON/SQUEEZE) information

Table S99. Solvent masking (PLATON/SQUEEZE) information for **16e**.

| No | x | y | z | V | e | Content |
|---|---|---|---|---|---|---|
| 1 | 0.500 | 0.000 | 0.000 | 146.2 | 35.6 | 0.8C4H8O2 |

## 10 Computational details

Geometry optimizations were performed using the Gaussian 16 software.[16] All geometry optimizations were computed using the functional PBE0[17,18] functional with Grimme dispersion corrections D3[19] and the Becke-Jonson damping function[20] in combination with the def2-SVP basis set.[21] The stationary points were located with the Berny algorithm[22] using redundant internal coordinates. Analytical Hessians were computed to determine the nature of stationary points (one and zero imaginary frequencies for transition states and minima, respectively)[23] and to calculate unscaled zero-point energies (ZPEs) as well as thermal corrections and entropy effects using the standard statistical-mechanics relationships for an ideal gas. The energies for the singlet and triplet gaps were performed at the PBE0-D3(BJ)/def2-TZVPP//PBE0-D3(BJ)/def2-SVP level of theory.

The atomic partial charges were computed with the natural bond orbital (NBO) method.[24,25] Natural Resonance Theory has been employed as implemented in NBO 7.0.[26] The topological quantum theory of atoms in molecules (QTAIM),[27] and Laplacian of the electron density analyses were carried out with AIMAll.[28] All these analysis were performed at the PBE0-D3(BJ)/def2-TZVPP//PBE0-D3(BJ)/def2-SVP level of theory.

Spin-resolved effective fragment orbitals (EFOs)[29] and subsequent (EOS)[30] analysis were performed with the APOST-3D program.[31] EOS analysis calculations were performed using both the Hilbert-space natural population analysis (NPA)[25] and the real space topological fuzzy Voronoi cells (TFVC) atomic definition.[32]

The nature of the chemical bonds were investigated by means of the Energy Decomposition Analysis (EDA) method, which was developed by Morokuma[33] and by Ziegler and Rauk.[34,35] The bonding analysis focuses on the interaction energy $\Delta E_{\text{int}}$ of a bond A–B between two (or more) fragments A and B in the particular electronic reference state and in the frozen geometry AB. This energy is divided into three main components, and one more related to the dispersion according to the level of theory used (Eq. S1).

$$\Delta E_{\text{int}} = \Delta E_{elst} + \Delta E_{Pauli} + \Delta E_{orb} + (\Delta E_{disp}) \quad \text{(S1)}$$

The term $\Delta E_{elst}$ corresponds to the quasiclassical electrostatic interaction between the unperturbed charge distributions of the prepared fragments, and it is usually attractive. The Pauli repulsion $\Delta E_{Pauli}$ is the energy change associated with the transformation from the superposition of the unperturbed wave functions (Slater determinant of the Kohn-Sham orbitals) of the isolated fragments to the wave function $\Psi_0 = N\hat{A}[\Psi^A\Psi^B]$, which properly obeys the Pauli principle through explicit antisymmetrization ($\hat{A}$ operator) and renormalization (N = constant) of the product wave function. It comprises the destabilizing interactions between electrons of the same spin on either fragment. The orbital interaction $\Delta E_{orb}$ accounts for charge

transfer and polarization effects.[36] In the case that the Grimme dispersion corrections[19,20] are computed the term $\Delta E_{disp}$ is added to equation S1. Further details on the EDA method can be found in the literature.[37] The relaxation of the fragments to their equilibrium geometries at the electronic ground state is termed $\Delta E_{prep}$, because it may be considered as preparation energy for chemical bonding. The addition of $\Delta E_{prep}$ to the intrinsic interaction energy $\Delta E_{int}$ gives the total energy $\Delta E$, which is, by definition, the opposite sign of the bond dissociation energy $D_e$:

$$\Delta E(-D_e) = \Delta E_{\text{int}} + \Delta E_{prep} \quad \text{(S2)}$$

The EDA–NOCV method combines the EDA with the natural orbitals for chemical valence (NOCV) to decompose the orbital interaction term $\Delta E_{orb}$ into pairwise contributions.[38–40] The NOCVs $\Psi_i$ are defined as the eigenvector of the valence operator, $\hat{V}$, given by Eq. S3.

$$\hat{V}\Psi_i = v_i \Psi_i \quad \text{(S3)}$$

In the EDA–NOCV scheme the orbital interaction term, $\Delta E_{orb}$, is given by Eq. S4,

$$\Delta \mathrm{E}_{\mathrm{orb}} = \sum_{\mathrm{k}} \Delta \mathrm{E}_{\mathrm{k}} = \sum_{\mathrm{k}=1}^{\mathrm{N}/2} v_k \left[-\mathrm{F}^{\mathrm{TS}}_{\mathrm{-k,k}} + \mathrm{F}^{\mathrm{TS}}_{\mathrm{k,k}}\right] \quad \text{(S4)}$$

in which $\mathrm{F}^{\mathrm{TS}}_{-\mathrm{k},-\mathrm{k}}$ and $\mathrm{F}^{\mathrm{TS}}_{\mathrm{k,k}}$ are diagonal transition state Kohn–Sham matrix elements corresponding to NOCVs with the eigenvalues $-v_k$ and $v_k$, respectively. The $\Delta E_k^{orb}$ term for a particular type of bond is assigned by visual inspection of the shape of the deformation density $\Delta\rho_k$. The latter term is a measure of the size of the charge deformation, and it provides a visual notion of the charge flow that is associated with the pairwise orbital interaction. The EDA–NOCV scheme thus provides both qualitative and quantitative information about the strength of orbital interactions in chemical bonds. The EDA-NOCV calculations were carried out with ADF2019.[41] The basis sets for all elements have triple-ζ quality augmented by two sets of polarizations functions and one set of diffuse functions. Core electrons were treated by the frozen-core approximation. This level of theory is denoted BP86-D3(BJ)/TZ2P.[19,20,42–45] Scalar relativistic effects have been incorporated by applying the zeroth-order regular approximation (ZORA).[45]

## 10.1 Benchmark

Table S100. Benchmarking of compound **4a** with different functionals and their respective computed relevant bond lengths.

| Functional | C1-Sb | C1-N1 | C1-N2 | N2-B | C2-Sb | C2-N3 | C2-N4 | N4-B |
|---|---|---|---|---|---|---|---|---|
| Exp | 2.1521 (8) | 1.3662 (10) | 1.3507 (11) | 1.5771 (11) | 2.1563 (9) | 1.3681 (11) | 1.3516 (10) | 1.5823 (12) |
| BP86 | 2.169 | 1.382 | 1.363 | 1.583 | 2.169 | 1.382 | 1.363 | 1.583 |
| PBE0 | 2.158 | 1.365 | 1.347 | 1.575 | 2.158 | 1.365 | 1.347 | 1.575 |
| wB97xd | 2.172 | 1.365 | 1.346 | 1.582 | 2.172 | 1.365 | 1.346 | 1.582 |
| M062-X | 2.185 | 1.363 | 1.346 | 1.584 | 2.185 | 1.363 | 1.346 | 1.584 |

Table S101. Benchmarking of compound **5a** with different functionals and their respective computed relevant bond lengths.

| Functional | C1-Bi | C1-N1 | C1-N2 | N2-B | C2-Bi | C2-N3 | C2-N4 | N4-B |
|---|---|---|---|---|---|---|---|---|
| Exp | 2.2805 (11) | 1.3633 (14) | 1.3485 (14) | 1.5807 (15) | 2.2878 (11) | 1.3608 (14) | 1.3526 (14) | 1.5856 (16) |
| BP86 | 2.279 | 1.379 | 1.363 | 1.587 | 2.279 | 1.379 | 1.363 | 1.587 |
| PBE0 | 2.269 | 1.363 | 1.347 | 1.579 | 2.269 | 1.363 | 1.346 | 1.579 |
| wB97xd | 2.291 | 1.363 | 1.346 | 1.586 | 2.291 | 1.363 | 1.346 | 1.586 |
| M062-X | 2.300 | 1.361 | 1.346 | 1.589 | 2.300 | 1.361 | 1.346 | 1.589 |

As can be seen on Table S98, the functional PBE0 is the one that represents best the experimental geometry for **4a**. For comparison, we kept the functional for 5a.

## 10.2 Frontier Molecular Orbitals (FMO)

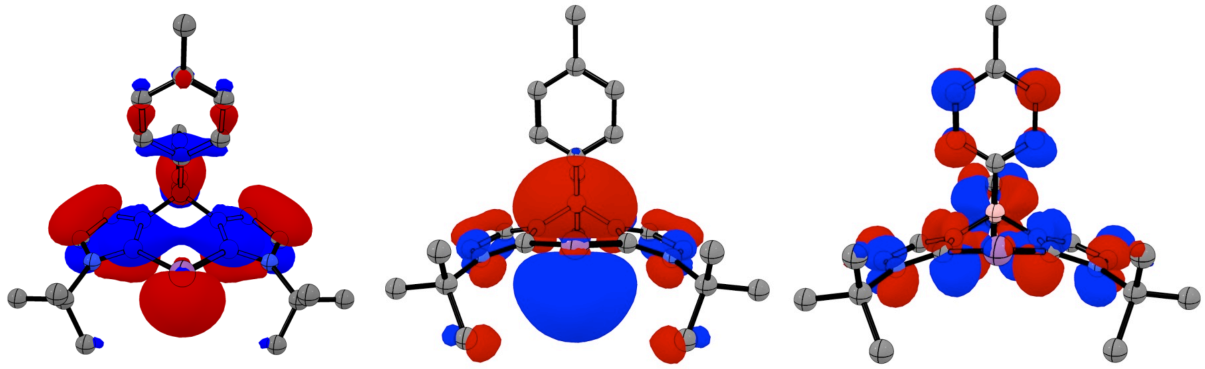

**HOMO-6** ( ε = -7.06 eV) | **HOMO** ( ε = -4.17 eV) | **LUMO** ( ε = -0.11 eV)

**4a**

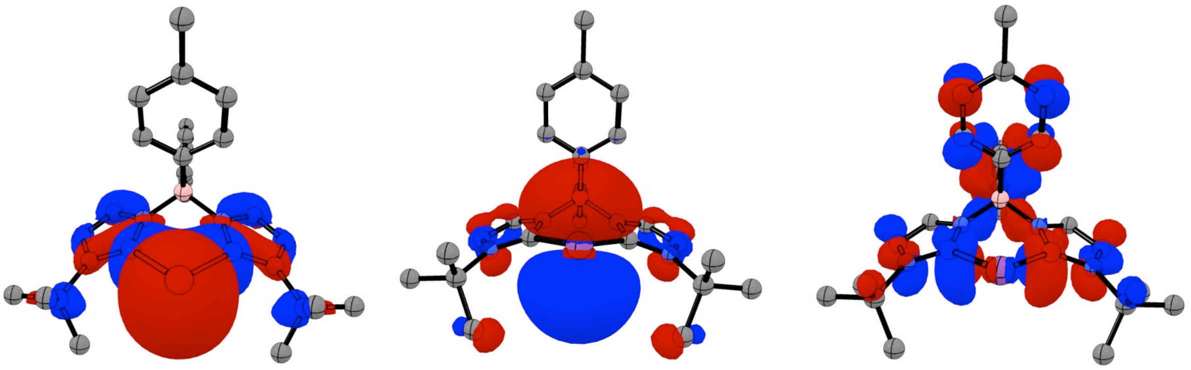

**HOMO-8** ( ε = -7.77 eV) | **HOMO** ( ε = -4.01 eV) | **LUMO** ( ε = -0.08 eV)

**5a**

Figure S40. Shape of the frontier molecular orbitals (isocontour = 0.03 au) for **4a** and **5a** and eigenvalues (in eV) at the PBE0-D3(BJ)/def2-TZVPP//PBE0-D3(BJ)/def2-SVP level of theory. Hydrogen atoms were omitted for clarity.

## 10.3 Natural Bond Orbitals (NBO)

Table S102. NBO results calculated at PBE0-D3(BJ)/def2-TZVPP//PBE0-D3(BJ)/def2-SVP level of theory: partial charges, Q (in e), Wiberg bond order, P (in a.u.).

| | **4a** | **5a** |
|---|---|---|
| **Q(Pn)** | +0.22 | +0.21 |
| **Q($C_{carb}$)** | +0.16 | +0.19 |
| **Q(L)** | -0.22 | -0.21 |
| **P(Pn–C)** | 0.91 | 0.86 |

Table S103. NBO results at the PBE0-D3(BJ)/def2-TZVPP//PBE0-D3(BJ)/def2-SVP of **4a** using the choose resonance structure. Hydrogen atoms were omitted for clarity.

| **Orbital** | **Occ.** | **Contribution from atoms to the orbitals** | **Atomic orbitals** |
|---|---|---|---|
| 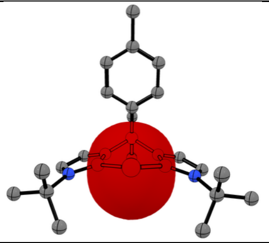 | 1.97 | LP(Sb) | Sb: s(83.11%)p 0.20( 16.87%)<br>d 0.00 (0.01%) f 0.00( 0.00%) |
| 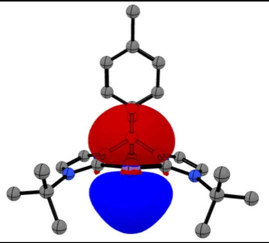 | 1.66 | LP(Sb) | Sb: s( 0.14%)p 99.99<br>( 99.80%)d 0.30(0.04%) f 0.16<br>(0.02%) |
| 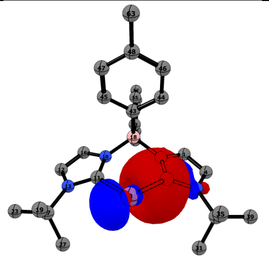 | 1.95 | $C_{carb}$ (71.91%) –<br>Sb (28.09 %) | C: s(37.52%)p 1.66( 62.36%)<br>d 0.00(0.09%) f 0.00(0.03%)<br>Sb: s( 8.62%)p 10.59<br>( 91.24%)d 0.01(0.08%)<br>f 0.01(0.06%) |
| 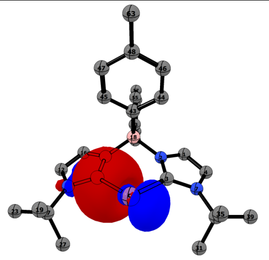 | 1.95 | Sb (28.09 %) –<br>$C_{carb}$ (71.91%) | Sb: s( 8.62%)p10.59( 91.24%)<br>d 0.01( 0.08%) f 0.01( 0.06%)<br>C: s( 37.52%)p 1.66( 62.36%)<br>d 0.00( 0.09%) f 0.00( 0.03%) |

Table S104. NBO results at the PBE0-D3(BJ)/def2-TZVPP//PBE0-D3(BJ)/def2-SVP of **5a** using the default resonance structure. Hydrogen atoms were omitted for clarity.

| Orbital | Occ. | Contribution from atoms to the orbitals | Atomic orbitals |
|---|---|---|---|
| 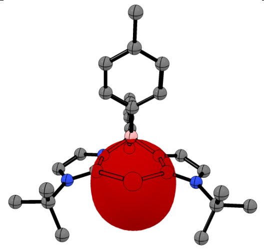 | 1.98 | LP(Bi) | Bi: s(89.69%)p 0.11(10.30%)d 0.00(0.00%) f 0.00(0.01%) |
| 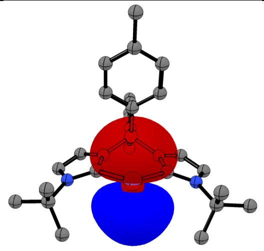 | 1.72 | LP(Bi) | Bi: s(0.07%)p 99.99(99.87%) d 0.33(0.03%) f 0.39(0.03%) |
| 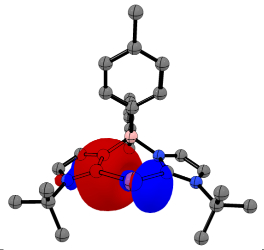 | 1.95 | Bi (26.75%) – $C_{carb}$ (73.25 %) | Bi: s(5.34%) p17.68 (94.49%)d 0.01(0.05%) f 0.02(0.11%) C: s(37.19%)p 1.69(62.73%) d 0.00(0.05%) f 0.00(0.02%) |
| 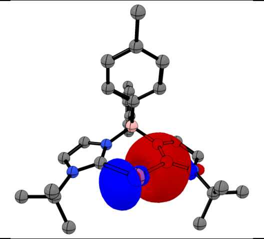 | 1.95 | Bi (26.75%) – $C_{carb}$ (73.25 %) | Bi: s(5.34%)p17.69( 94.49%)d 0.01(0.05%) f 0.02(0.11%) C: s(37.19%)p 1.69(62.73%)d 0.00(0.05%) f 0.00(0.02%) |

## 10.4 Natural Resonance Theory (NRT)

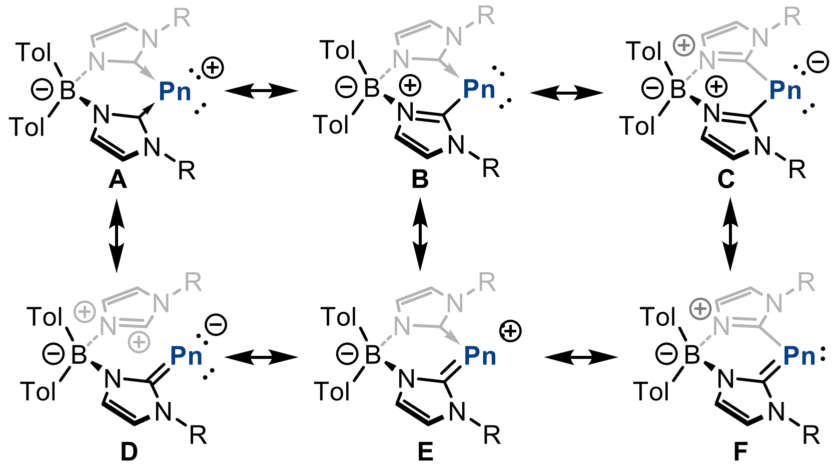


Figure S41. Possible resonance structures for compound **4a** and **5a**.

Table S105. NBO results for **4a** selected Lewis structures at the PBE0-D3(BJ)/def2-TZVPP//PBE0-D3(BJ)/def2-SVP level of theory.

| | 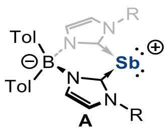 **A** | 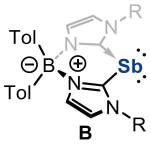 **B** | 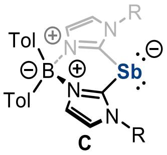 **C** | 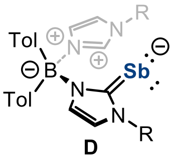 **D** | 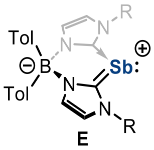 **E** | 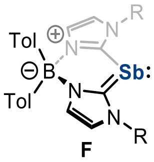 **F** |
|---|---|---|---|---|---|---|
| ***Effective core*** | 28.00 | 28.00 | 28.00 | 28.00 | 28.00 | 28.00 |
| ***Core*** | 84.00 | 84.00 | 84.00 | 84.00 | 84.00 | 84.00 |
| ***Valence Lewis*** | 168.73 | 169.67 | 170.60 | 169.19 | 169.44 | 170.36 |
| ***Total Lewis*** | 280.73 | 281.67 | 282.60 | 281.19 | 281.43 | 282.36 |
| ***Valence non-Lewis*** | 6.88 | 5.95 | 5.02 | 6.39 | 6.17 | 5.24 |
| ***Rydberg non-Lewis*** | 0.39 | 0.39 | 0.39 | 0.41 | 0.39 | 0.39 |
| ***Total non-Lewis*** | 7.27 | 6.33 | 5.40 | 6.81 | 6.57 | 5.64 |

Table S106. NRT results for **4a** selected Lewis structures at the PBE0-D3(BJ)/def2-TZVPP//PBE0-D3(BJ)/def2-SVP level of theory.

| | 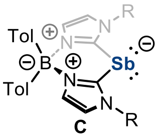 C | 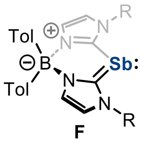 F | 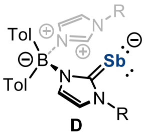 D | 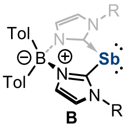 B | 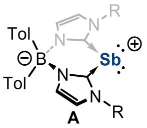 A | 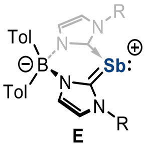 E |
|---|---|---|---|---|---|---|
| ***Weight*** | 73.47 | 17.48 | 8.16 | 0.55 | 0.35 | 0.00 |
| Total ***non-Lewis*** | 5.40 | 5.64 | 6.81 | 6.33 | 7.27 | 6.57 |

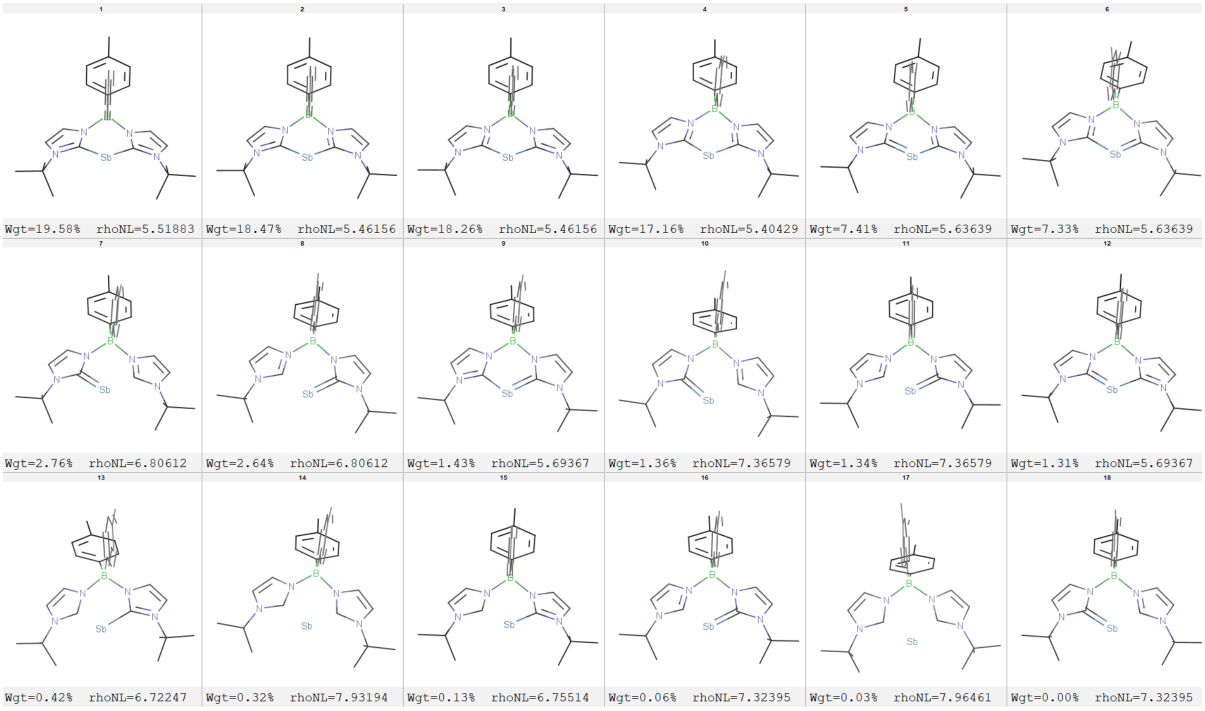


Figure S42. NRT resonance structure weight for 4a at the PBE0-D3(BJ)/def2-TZVPP//PBE0-D3(BJ)/def2-SVP level of theory. Formal charges are not displayed.

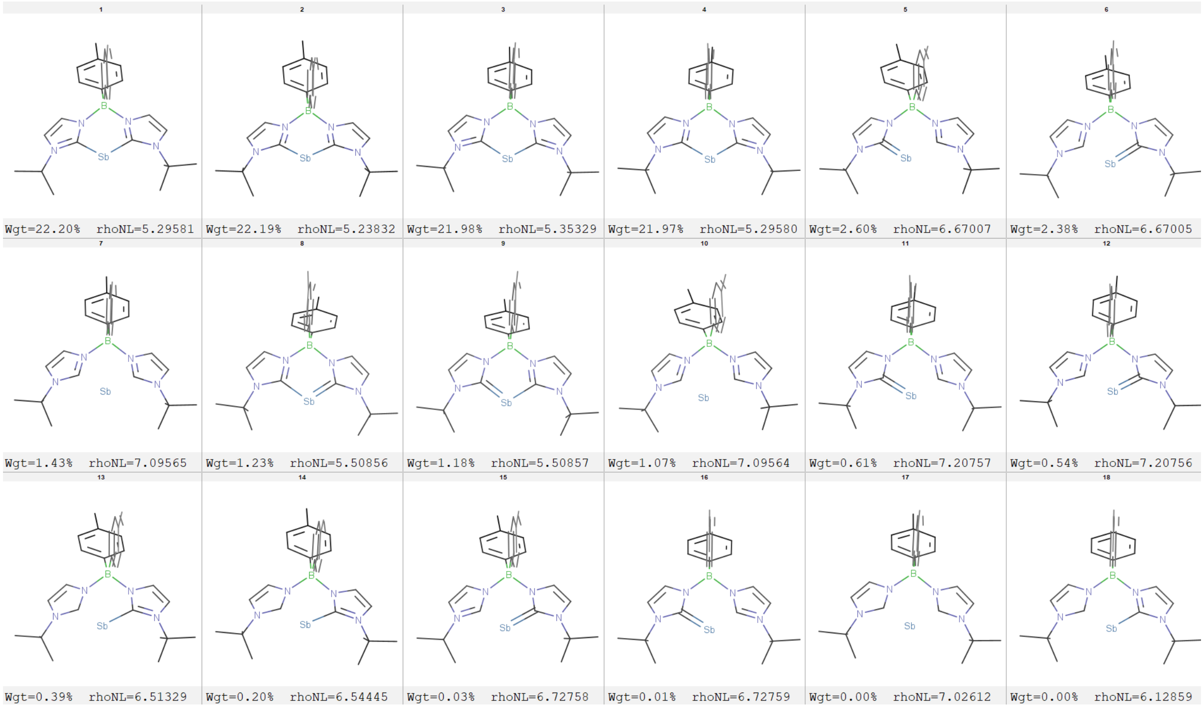


Figure S43. NRT resonance structure weight at the M06-2X/def2-TZVPP//PBE0+D3(BJ)/def2-SVP level of theory. Formal charges are not displayed.

Table S107. NBO results for **5a** selected Lewis structures at the PBE0-D3(BJ)/def2-TZVPP//PBE0-D3(BJ)/def2-SVP level of theory.

| | 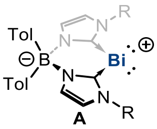 A | 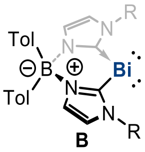 B | 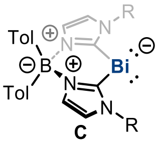 C | 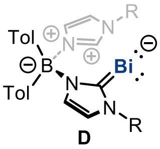 D | 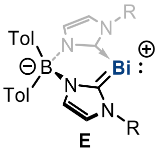 E | 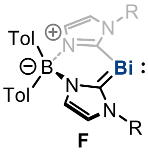 F |
|---|---|---|---|---|---|---|
| ***Effective core*** | 60.00 | 60.00 | 60.00 | 60.00 | 60.00 | 60.00 |
| ***Core*** | 84.00 | 84.00 | 84.00 | 84.00 | 84.00 | 84.00 |
| ***Valence Lewis*** | 168.84 | 169.75 | 170.65 | 169.23 | 169.49 | 170.39 |
| ***Total Lewis*** | 312.84 | 313.75 | 314.65 | 313.23 | 313.49 | 314.39 |
| ***Valence non-Lewis*** | 6.78 | 5.87 | 4.96 | 6.37 | 6.12 | 5.22 |
| ***Rydberg non-Lewis*** | 0.38 | 0.38 | 0.39 | 0.40 | 0.39 | 0.39 |
| ***Total non-Lewis*** | 7.16 | 6.25 | 5.35 | 6.77 | 6.51 | 5.61 |

Table S108. NRT results for **5a** selected Lewis structures at the PBE0-D3(BJ)/def2-TZVPP//PBE0-D3(BJ)/def2-SVP level of theory.

| | 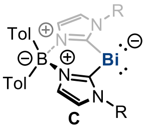 **C** | 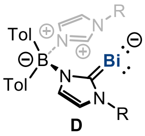 **D** | 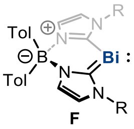 **F** | 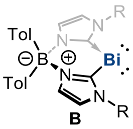 **B** | 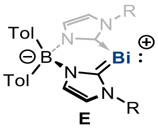 **E** | 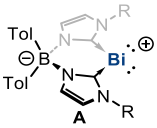 **A** |
|---|---|---|---|---|---|---|
| ***Weight*** | 86.60 | 7.96 | 3.62 | 0.96 | 0.86 | 0.00 |
| ***Total non-Lewis*** | 5.35 | 6.77 | 5.61 | 6.25 | 6.51 | 7.16 |

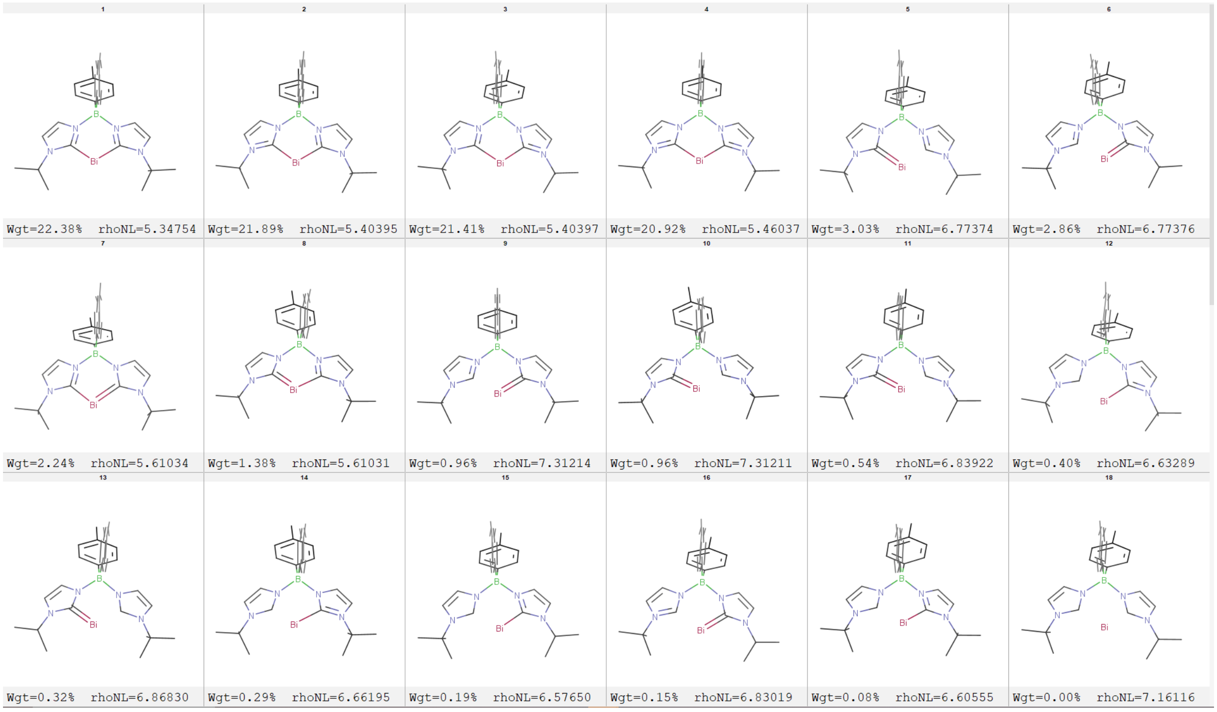


Figure S44. NRT resonance structure weight for **5a** at the PBE0-D3(BJ)/def2-TZVPP//PBE0-D3(BJ)/def2-SVP level of theory. Formal charges are not displayed.

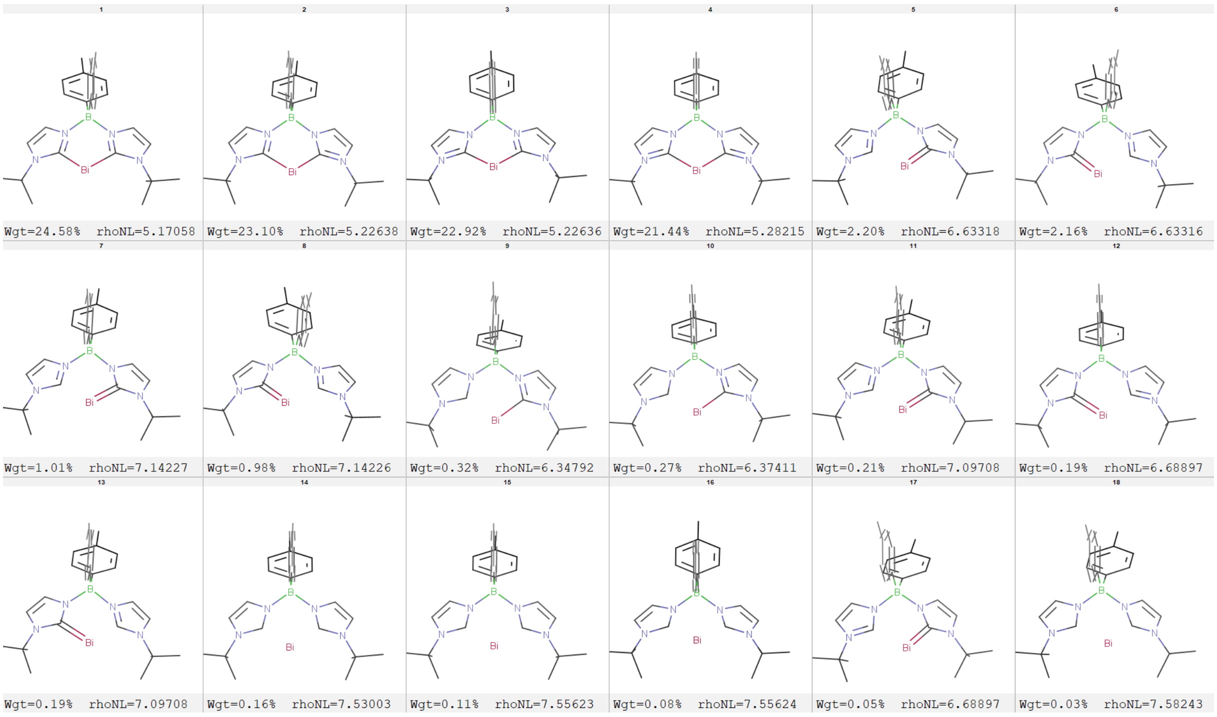


Figure S45. NRT resonance structure weight for **5a** at the M06-2X/def2-TZVPP//PBE0-D3(BJ)/def2-SVP level of theory. Formal charges are not displayed.

**Conclusion from the NRT analysis**

By default, the NBO analysis for **4a** affords **C** as the natural Lewis structure (NLS) with 5.52 non-lewis density at 1.63 occupancy. However, direct natural resonance theory (NRT) calculation does not yield any resonance structure (RS) without specifying them manually. Thus, we repeated the NRT using CHOOSE to specify the previous obtained NLS, at the same 1.63 threshold occupancy, and defining manually the plausible natural resonance structures by NRTSTR (Figure S34). This afforded **C** as the leading resonance Lewis structure (NRLS) for both **4a** and **5a** compounds. Repeating the same procedure with M06-2X/def2-TZVPP//PBE0-D3(BJ)/def2-SVP afforded similar results (Figures S36 and S39). All NRT calculations were done using unlimited cycles (NRTCYC=0), defining the endo-cycle of the compound on the NBO line and considering that all NLRS correspond to the NLS.

## 10.5 Atoms in Molecule (AIM)

Table S109. QTAIM Local topological parameters [in a.u.] of the charge density at the main Bond Critical Points calculated at PBE0-D3(BJ)/def2-TZVPP//PBE0-D3(BJ)/def2-SVP level of theory: Local kinetic energy density (G), Local potential energy density (V), local electronic energy density (H) and ellipticity-

| Compound | BCP | ρ(r) [e/Å$^3$] | $\nabla^2$ρ(r) [e/Å$^5$] | V | G | H | ϵ |
|---|---|---|---|---|---|---|---|
| 4a | Sb-$C_{carb}$ | 0.103 | 0.146 | -0.1186 | 0.0775 | -0.0411 | 0.24 |
| 5a | Bi-$C_{carb}$ | 0.093 | 0.146 | -0.0960 | 0.0660 | -0.0299 | 0.21 |

10.5.1 Laplacian for $PnR_3$ compounds (Pn=N,P,As,Sb and Bi ; R=H and Ph) and PnPh (Pn = Sb and Bi) compounds

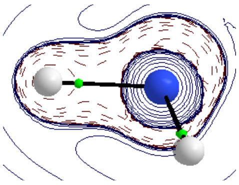

**$NH_3$**

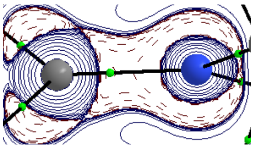

**$NPh_3$**

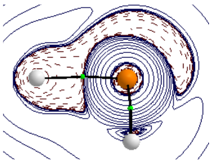

**$PH_3$**

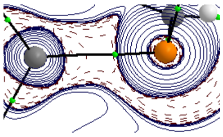

**$PPh_3$**

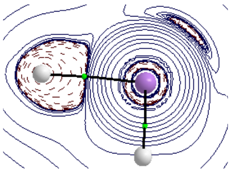

**$AsH_3$**

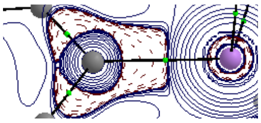

**$AsPh_3$**

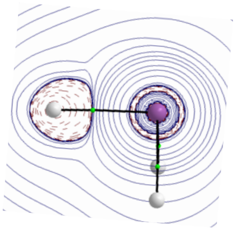

**$SbH_3$**

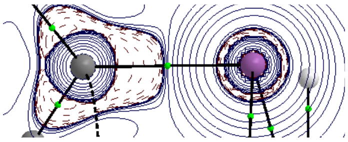

**$SbPh_3$**

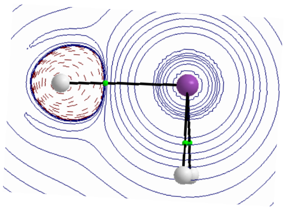

**$BiH_3$**

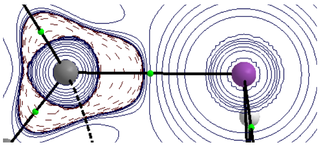

**$BiPh_3$**

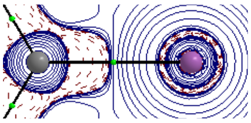

**SbPh**

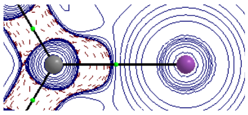

**BiPh**

Figure S46. Laplacian of the electron density ($\nabla^2\rho(r)$) using Quantum Atom in Molecules (QTAIM) at the PBE0-D3(BJ)/def2-TZVPP//PBE0-D3(BJ)/def2-SVP level of theory. For SbPh and BiPh the singlet electronic structure was considered for comparison.

## 10.6 Effective Oxidation States (EOS) and Singlet-triplet Gaps

We fragmented II, VIII, IX, X, **4a** and **5a** in 2 fragments, namely Pn and their own ligand. The rest was defined in 3 fragments, namely Pn and both ligands independently, except 12 which was defined in 4 fragments, namely the two Pn and both ligands.

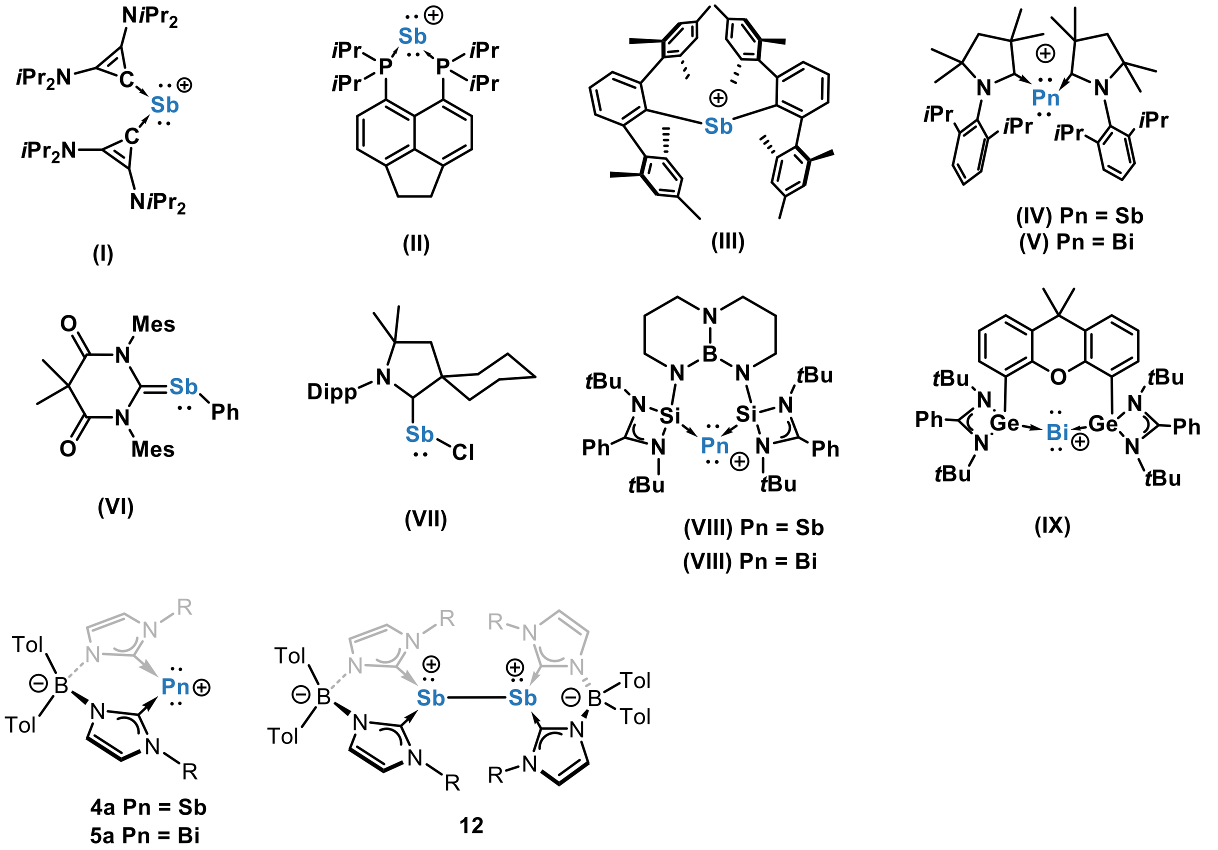


Figure S47. Low-valent pnictogen complexes computed for the effective oxidation state analysis and the singlet-triplet gaps. Pn =Sb, Bi.

Table S110. Oxidation states (OS) and reliability index, R (%) from Hilbert-space natural atomic orbitals (NAO) and real-space topological fuzzy Voronoi cells (TFVC) analyses together with the singlet triplet gap energies ($\Delta E_{S-T}$) in kcal/mol at the PBE0-D3(BJ)/def2-TZVPP//PBE0-D3(BJ)/def2-SVP level of theory.

| **Complex** | **OS (TFVC)** | **R (%)** | **OS (TFVC)** | **R (%)** | **$\Delta E_{S-T}$ Gap** |
|---|---|---|---|---|---|
| **I** | +1 | 93 | 1 | 99 | 37.6 |
| **II** | +1 | 79 | +1 | 91 | 37.1 |
| **III** | +3 | 81 | +3 | 81 | 21.0 |
| **IV** | +1 | 99 | +1 | 77 | 29.6 |
| **V** | +1 | 99 | +1 | 79 | 31.8 |
| **VI** | +1 | 75 | +1 | 55 | 22.1 |
| **VII** | +1 | 87 | +1 | 67 | 22.1 |
| **VIII** | +1 | 72 | +1 | 55 | 48.7 |
| **IX** | +1 | 61 | +1 | 72 | 42.8 |
| **X** | +1 | 61 | +1 | 74 | 37.2 |
| **4a** | +1 | 87 | +1 | 82 | 39.0 |
| **5a** | +1 | 91 | +1 | 87 | 31.8 |
| **12** | +2 | 65 | +2 | 70 | 29.1 |

Table S111. Relevant effective fragment orbitals (EFOs) of $P_n$ for **4a** and **5a** using NAOs fragmentation at the PBE0-D3(BJ)/def2-TZVPP//PBE0-D3(BJ)/def2-SVP level of theory.

| **4a** | **5a** | **12** |
|---|---|---|
| 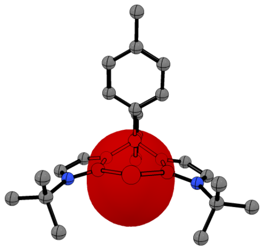 0.98 (occ) | 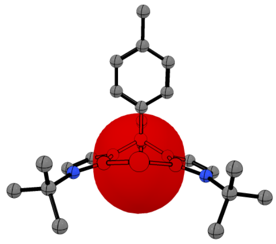 0.99 (occ) | 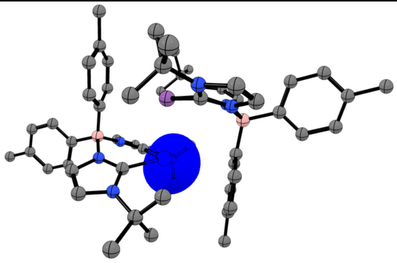 0.99 (occ) |
| 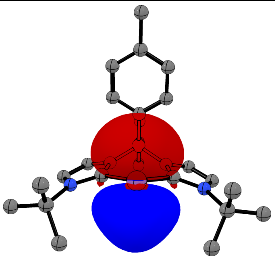 0.83 (occ) | 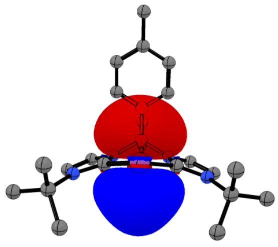 0.86 (occ) | 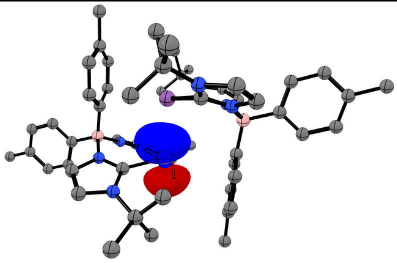 0.50 (occ) |
| 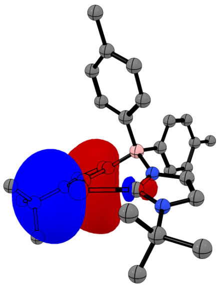 0.31 (unocc) | 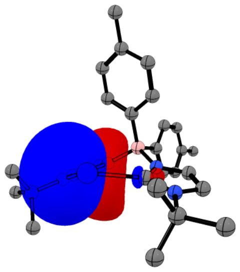 0.30 (unocc) | 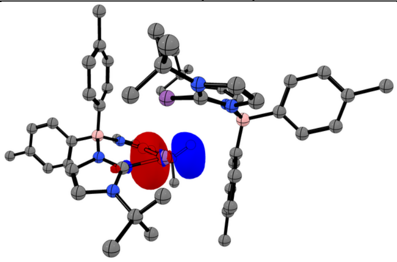 0.30 (unocc) |
| 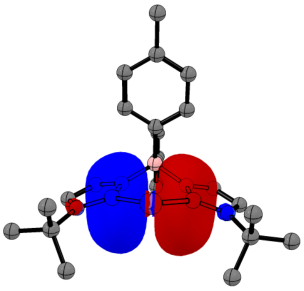 0.26 (unocc) | 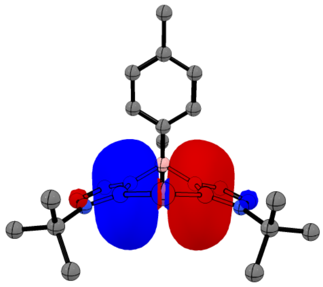 0.25 (unocc) | 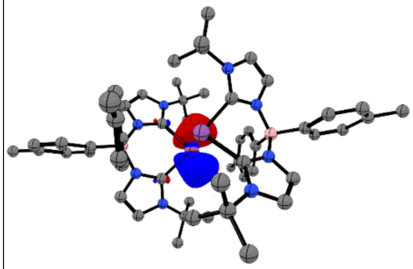 0.27 (unocc) |

## 10.7 Energy Decomposition Analysis (EDA)

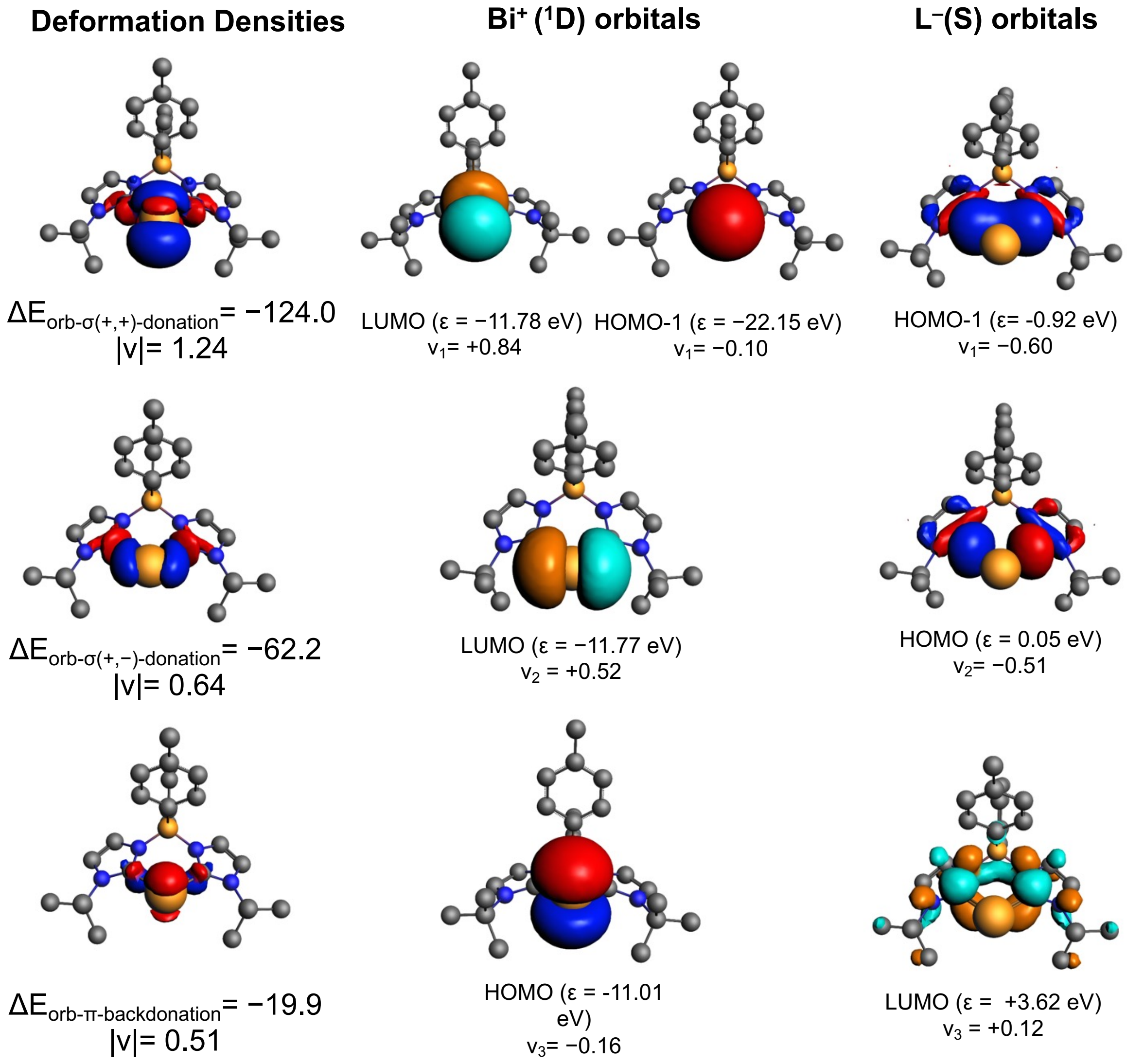


Figure S48. Plot of deformation densities Δρ (isovalue = 0.003) of the pairwise orbital interactions between bis(NHC)borate ligand ($L^{-}$(S)) and $Bi^{+}$($^{1}$D) in **5a**, associated energies ΔE (in kcal/mol) and eigenvalues v (in a.u.). The red colour shows the charge outflow, whereas blue shows charge density accumulation. Shape of the most important interacting occupied and vacant orbitals (isovalue = 0.03) of the fragments. The fragment molecular orbitals the occupied orbitals are shown in blue and yellow, while virtual orbitals are in cyan and pale yellow. Hydrogen atoms are omitted for clarity.

Table S112. EDA-NOCV results at BP86-D3(BJ)/TZ2P. All values are in kcal·mol$^{-1}$.[a]

| | **I-Sb** | **I-Bi** |
|---|---|---|
| fragmentation | $cAAC_2$ (S)[b]; $Sb^+$ ($^1D$, $5s^25p_=^05p_\parallel^05p_\perp^2$) | $cAAC_2$ (S)[b]; $Bi^+$ ($^1D$, $6s^26p_=^06p_\parallel^06p_\perp^2$) |
| $\Delta E_{int}$ | -254.3 | -233.4 |
| $\Delta E_{Pauli}$ | 344.5 | 301.4 |
| $\Delta E_{disp}$[c] | -28.2 (4.7 %) | -30.0 (5.6 %) |
| $\Delta E_{elst}$[c] | -294.5 (49.2 %) | -271.8 (50.8 %) |
| $\Delta E_{orb}$[c] | -276.1 (46.1 %) | -232.9 (43.6 %) |
| $\Delta E_{orb\text{-}\sigma(+,+)\text{-}donation}$[d] | -115.4 (41.8 %) | -91.4 (39.2 %) |
| $\Delta E_{orb\text{-}\sigma(+,-)\text{-}donation}$[d] | -58.5 (21.2 %) | -54.2 (23.3 %) |
| $\Delta E_{orb\text{-}\pi\text{-}backdonation}$[d] | -47.7 (17.3 %) | -41.8 (17.9 %) |
| $\Delta E_{orb\ rest}$[d] | -54.4 (19.7 %) | -45.5 (19.5 %) |
| $\Delta E_{prep}$ | 30.9 | 29.8 |
| $D_e$ | 223.4 | 203.6 |

[a] All calculations were performed on the PBE0-D3(BJ)/def2-SVP optimized structures. [b] S stands for singlet electronic state. [c] The value in parenthesis gives the percentage contribution to the total attractive interactions $\Delta E_{elst}$ + $\Delta E_{orb}$ + $\Delta E_{disp}$. [d] The value in parenthesis gives the percentage contribution to the total orbital interaction term.

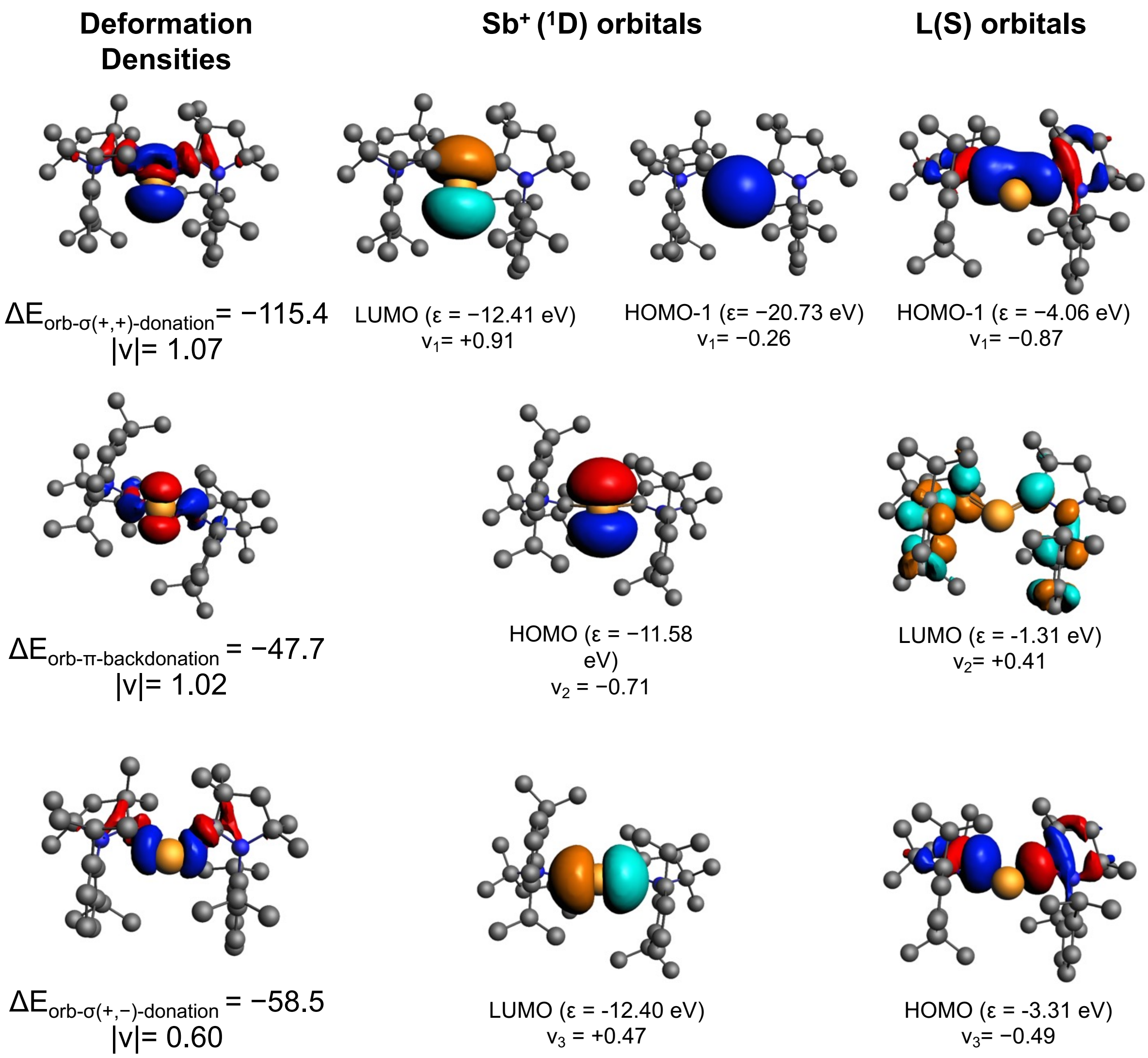


Figure S49. Plot of deformation densities Δρ (isovalue = 0.003) of the pairwise orbital interactions between $cAAC_2$ ($L^-$(S)) and $Sb^+$($^1$D) in **I-Sb**, associated energies ΔE (in kcal/mol) and eigenvalues v (in a.u.). The red colour shows the charge outflow, whereas blue shows charge density accumulation. Shape of the most important interacting occupied and vacant orbitals (isovalue = 0.03) of the fragments. The fragment molecular orbitals the occupied orbitals are shown in blue and yellow, while virtual orbitals are in cyan and pale yellow. Hydrogen atoms are omitted for clarity.

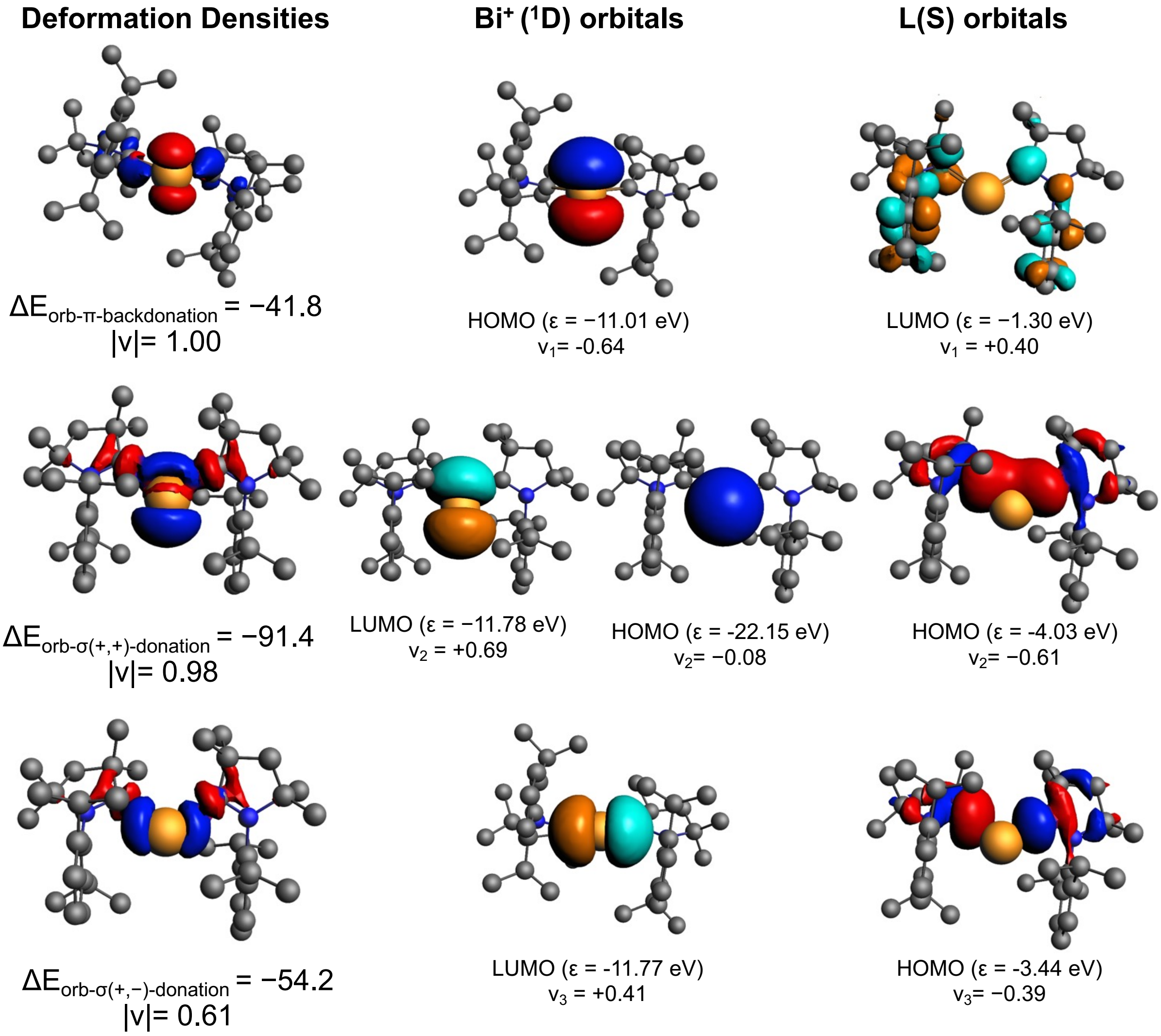


Figure S50. Plot of deformation densities Δρ (isovalue = 0.003) of the pairwise orbital interactions between $cAAC_2$ ($L^-$(S)) and $Bi^+$($^1D$) in **I-Bi**, associated energies ΔE (in kcal/mol) and eigenvalues v (in a.u.). The red colour shows the charge outflow, whereas blue shows charge density accumulation. Shape of the most important interacting occupied and vacant orbitals (isovalue = 0.03) of the fragments. The fragment molecular orbitals the occupied orbitals are shown in blue and yellow, while virtual orbitals are in cyan and pale yellow. Hydrogen atoms are omitted for clarity.

## 10.8 CASSCF calculations

We computed single-point multiconfigurational calculation by the means of Complete Active Space Self Consistent Field (CASSCF) method with the def2-SVP basis set in combination with effective core potentials for Sb and Bi. This was computed to explore the diradical character of 4a and 5a using a simple CASSCF (2,2). The geometries used were computed at the PBE0-D3(BJ)/def2-SVP level of theory. The CASSCF calculations have been performed with the pySCF1.7 package.[47]

Table S113. CI coefficients for **4a** and **5a** obtained at the CASSCF(2,2)/def2-SVP//PBE0 D3(BJ)/def2-SVP level of theory.

| Compound | $C_0$ coefficient | $C_d$ coefficient | Diradical character (%) |
|---|---|---|---|
| 4a | 0.9946 | -0.1037 | 14.7 |
| 5a | -0.9929 | 0.1185 | 16.8 |

## 10.9 TD-DFT results

These calculations have been performed using ORCA 6.0.[48] The level of theory employed is PBE0 as a functional with the ZORA relativistic effects and ZORA-def2-TZVP[49] basis set for all atoms except for Sb and Bi where the SARC-ZORA-TZVP basis sets were used.[50,51] This was combined by the auxiliary basis sets SARC/J and def2/J.[52] All calculations were computed taking into account spin-orbit coupling (SOC).

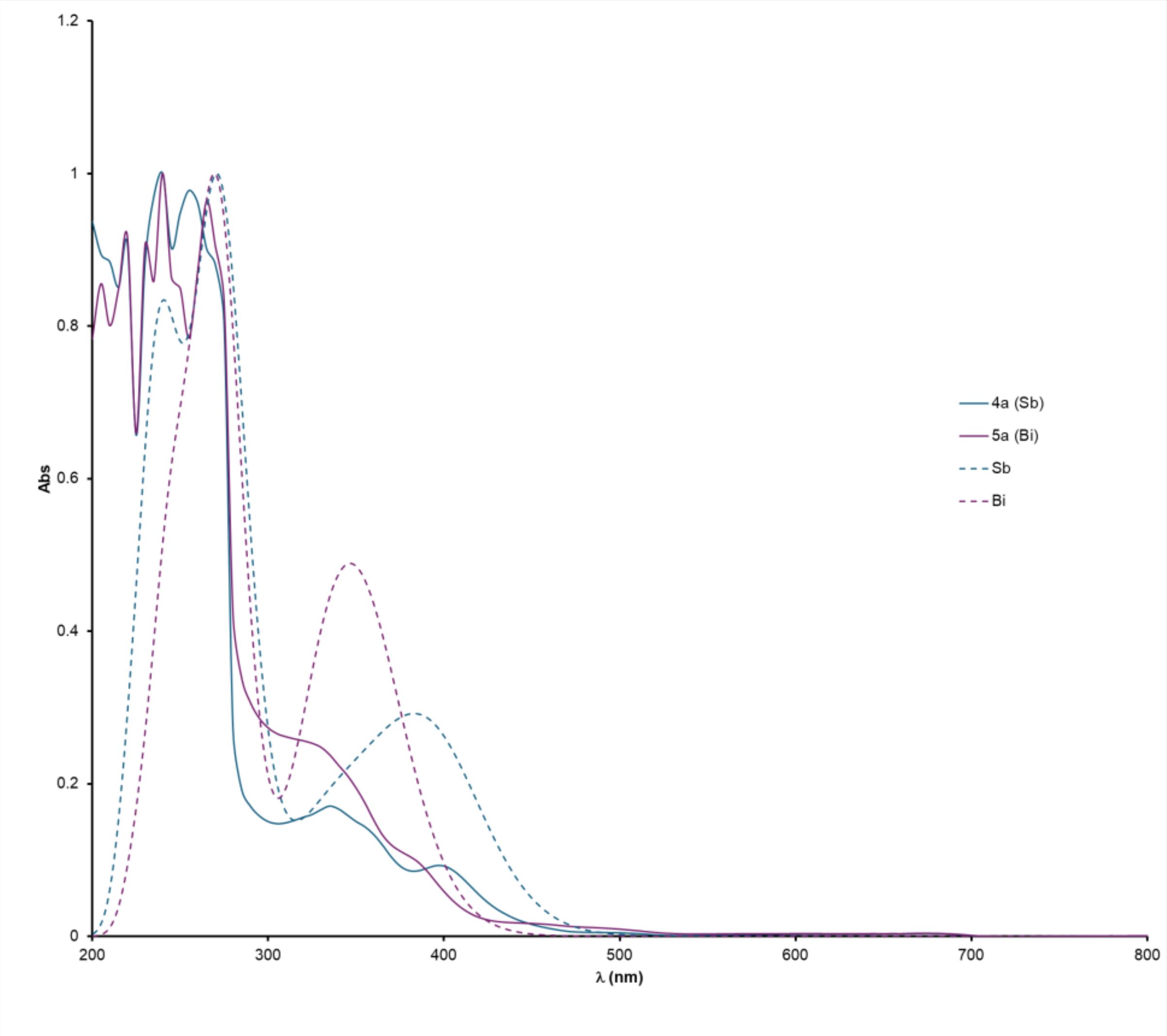


Figure S51. Experimental (full line) and theoretical (PBE0-D3(BJ)/ SARC-ZORA-TZVP dashed line) UV-VIS for **4a** (blue) and **5a** (purple). The standard deviation was arbitrarily set to 0.5 eV.

10.9.1 TD-DFT results for **4a**

Table S114. All selected bands of the SOC-UV-VIS have f(d2) > 0.03. Besides, the selected weighs and f for the SOC, singlet and triplet transitions are bigger than 0.1.

| **UV-vis band** | **Transition (Weight)** | **HOMO/SOMOs** | **LUMOs** |
|---|---|---|---|
| 404nm<br>F(d2)=0.031 | $S_1$ (0.69) | 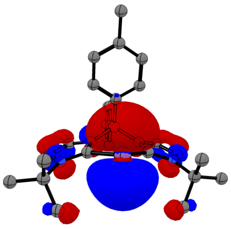<br>HOMO | 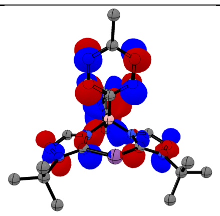<br>LUMO+1<br>f=0.35 |
| | | | 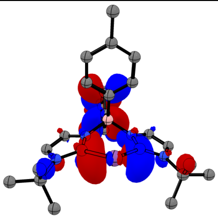<br>LUMO+2<br>f=0.30 |
| | | | 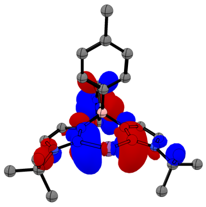<br>LUMO+3<br>F=0.29 |
| | $T_1$ (0.15) | 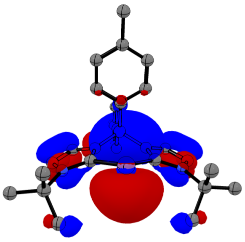<br>SOMO-1 | 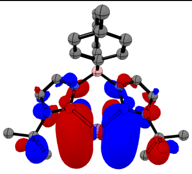<br>SOMO<br>f=0.24 |
| | | | 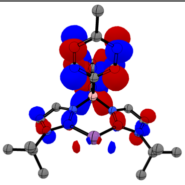<br>LUMO<br>f=0.30 |
| | | | 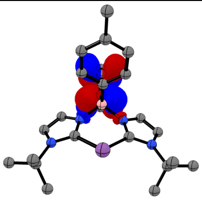<br>LUMO+1<br>f=0.34 |

| | | | |
|---|---|---|---|
| 362 nm<br>F(d2)=0.038 | $S_1$ (0.95) | 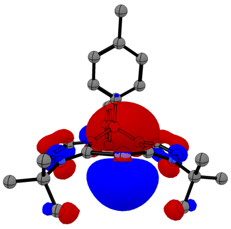<br>HOMO | 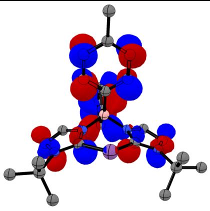<br>LUMO+1<br>f=0.60<br>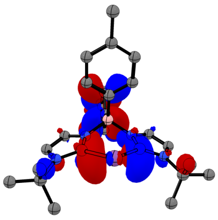<br>LUMO+2<br>f=0.14 |
| 337 nm<br>F(d2)=0.086 | $S_1$ (0.99) | 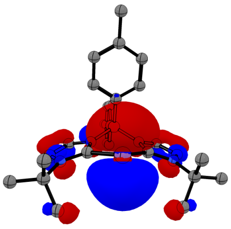<br>HOMO | 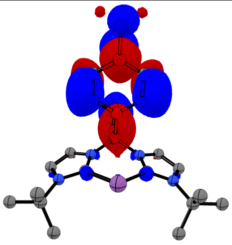<br>LUMO+4<br>f=0.38<br>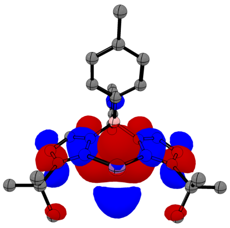<br>LUMO+5<br>f=0.64 |
| 334 nm<br>F(d2)=0.094 | $S_1$ (0.99) | 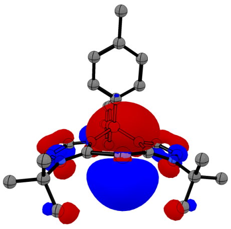<br>HOMO | 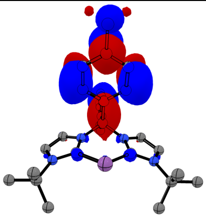<br>LUMO+4<br>f=0.63<br>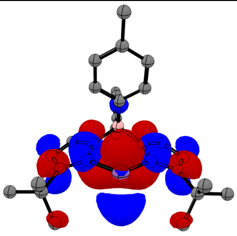<br>LUMO+5<br>f=0.35 |

| | | | |
|---|---|---|---|
| 331 nm<br>F(d2)=0.035 | $S_1$ (0.99) | 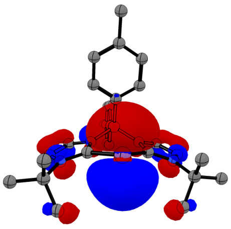<br>HOMO | 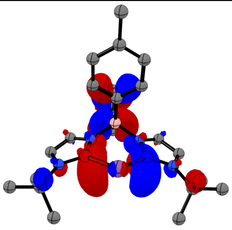<br>LUMO+2<br>f=0.50 |
| | | | 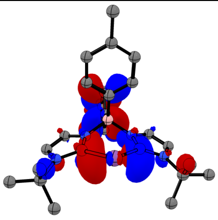<br>LUMO+3<br>f=0.20 |
| | | | 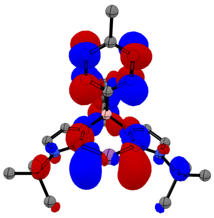<br>LUMO+6<br>f=0.12 |
| | | | 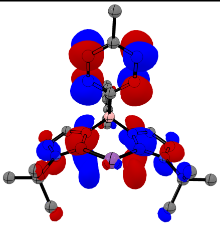<br>LUMO+7<br>f=0.16 |

| | | | |
|---|---|---|---|
| 309 nm<br>F(d2)=0.032 | $S_1$ (0.99) | 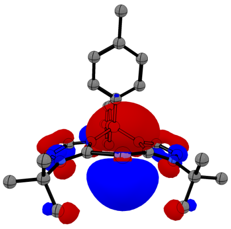<br>HOMO | 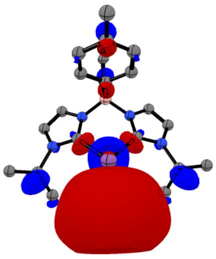<br>LUMO+8<br>f=0.95 |

<table>
<tr><td>279 nm<br>F(d2)=0.043</td><td>$S_1$ (0.99)</td><td>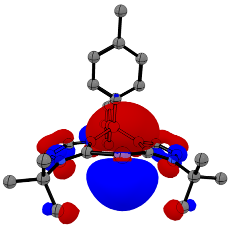<br>HOMO</td><td>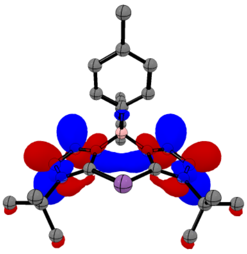<br>LUMO+8<br>f=0.98</td></tr>
<tr><td rowspan="3">271 nm<br>F(d2)=0.063</td><td>$S_1$ (0.74)</td><td>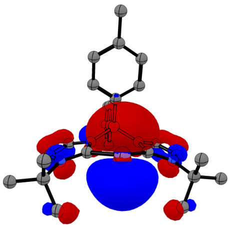<br>HOMO</td><td>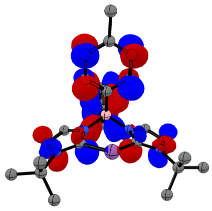<br>LUMO+10<br>f=0.94</td></tr>
<tr><td rowspan="2">$T_1$ (0.20)</td><td rowspan="2">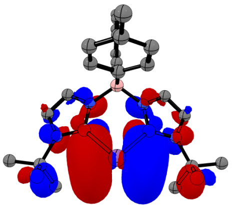<br>SOMO-1</td><td>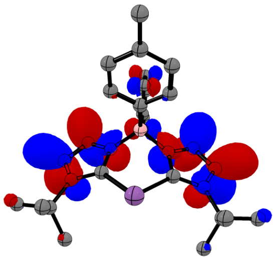<br>LUMO+11<br>f=0.66</td></tr>
<tr><td>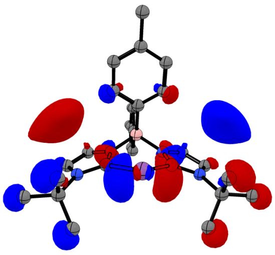<br>LUMO+10<br>f=0.22</td></tr>
<tr><td>244 nm<br>F(d2)=0.087</td><td>$S_1$ (0.99)</td><td>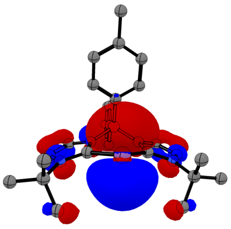<br>HOMO</td><td>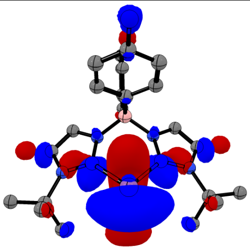<br>LUMO+15<br>f=0.83</td></tr>
</table>

<table>
<tr><td rowspan="3">219 nm<br>F(d2)=0.192</td><td rowspan="3">$S_1$ (0.99)</td><td>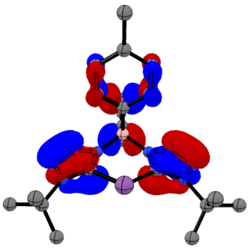<br>HOMO-4</td><td>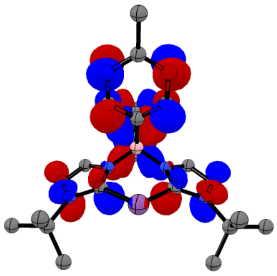<br>LUMO<br>f=0.21</td></tr>
<tr><td rowspan="2">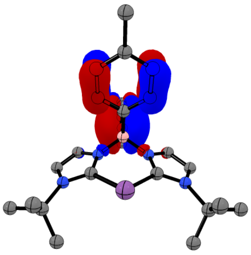<br>HOMO-2</td><td>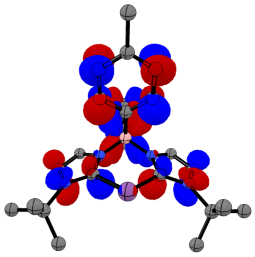<br>LUMO<br>f=0.31</td></tr>
<tr><td>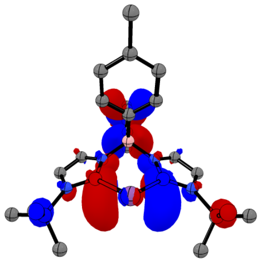<br>LUMO+1<br>f=0.28</td></tr>
</table>

10.9.2 TD-DFT results for **5a**

Table S115. All selected bands of the SOC-UV-VIS have f(d2) > 0.05. Besides, the selected weighs and f for the SOC, singlet and triplet transitions are bigger than 0.1.

<table>
<tr><th>UV-vis band</th><th>Transition (Weight)</th><th>HOMO/SOMOs</th><th>LUMOs</th></tr>
<tr><td rowspan="3">509nm<br>f(d2)=0.36</td><td rowspan="3">$T_1$ (0.89)</td><td rowspan="3">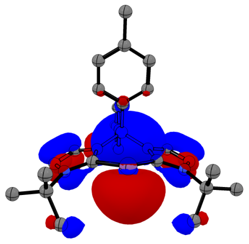<br>SOMO-1</td><td>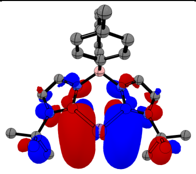<br>SOMO<br>f=0.15</td></tr>
<tr><td>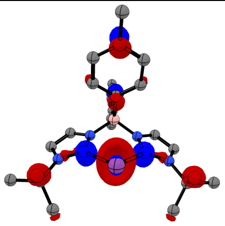<br>LUMO+2<br>f=0.56</td></tr>
<tr><td>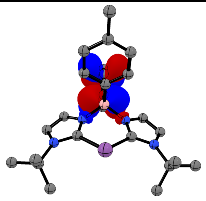<br>LUMO+3<br>f=0.21</td></tr>
<tr><td rowspan="2">353 nm<br>F(d2)=0.15</td><td>$S_1$ (0.49)</td><td>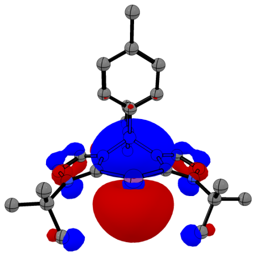<br>HOMO</td><td>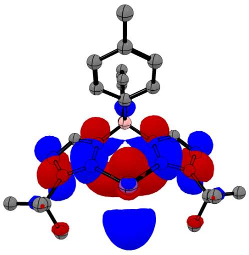<br>LUMO+6<br>f=0.96</td></tr>
<tr><td>$T_1$ (0.22)</td><td>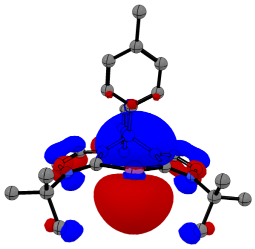<br>SOMO-1</td><td>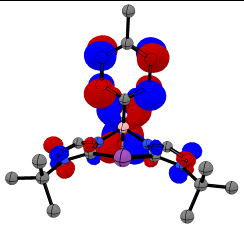<br>LUMO<br>f=0.20</td></tr>
</table>

| | | | |
|---|---|---|---|
| | | | 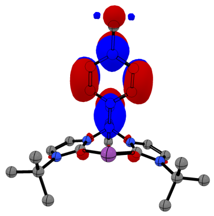<br>LUMO+3<br>f=0.16 |
| | | | 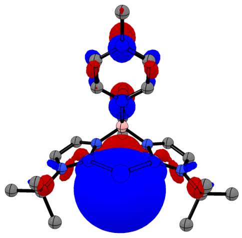<br>LUMO+7<br>f=0.36 |
| 346 nm<br>F(d2)=0.29 | $S_1$ (0.46) | 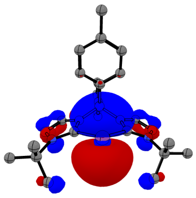<br>HOMO | 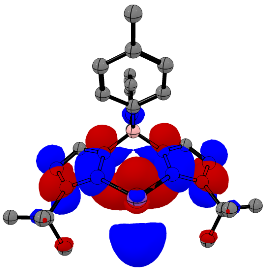<br>LUMO+6<br>f=0.96 |
| | $T_1$ (0.25) | 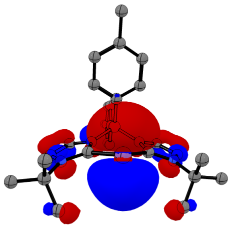<br>SOMO-1 | 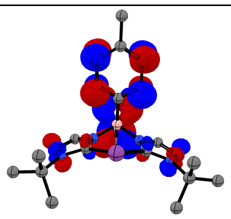<br>LUMO<br>f=0.20 |
| | | | 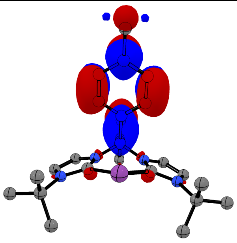<br>LUMO+3<br>f=0.16 |

| | | | |
|---|---|---|---|
| | | | 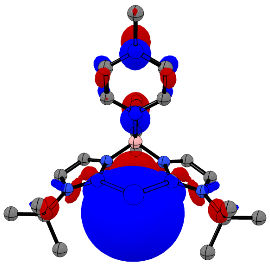<br>LUMO+7<br>f=0.36 |
| 337 nm<br>F(d2)=0.11 | $S_1$ (0.93) | 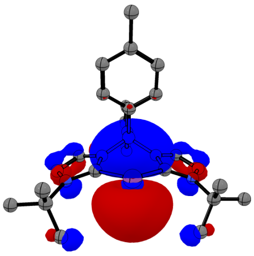<br>HOMO | 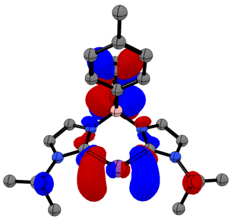<br>LUMO+3<br>f=0.13 |
| | | | 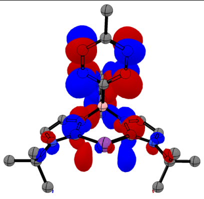<br>LUMO+6<br>f=0.50 |
| | | | 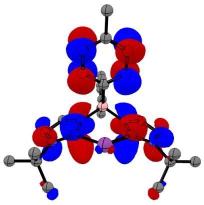<br>LUMO+7<br>f=0.31 |
| 270 nm<br>F(d2)=0.092 | $S_1$ (0.71) | 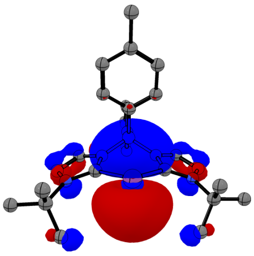<br>HOMO | 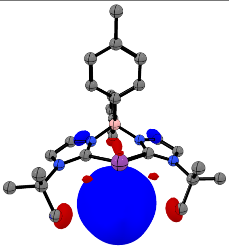<br>LUMO+13<br>f=0.94 |
| 247 nm<br>F(d2)=0.17 | $S_1$ (0.98) | 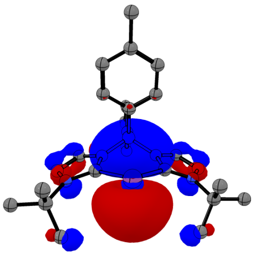<br>HOMO | 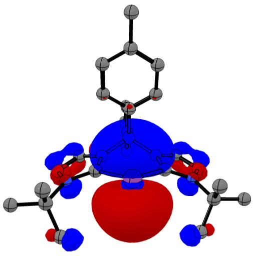<br>LUMO+13 |

| | | | |
|---|---|---|---|
| | | | f=0.25 |
| | | | 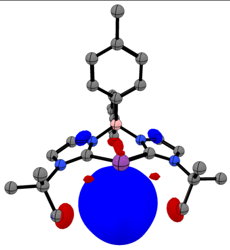 LUMO+14<br>f=0.57 |
| 227 nm<br>F(d2)=0.06 | $S_1$ (0.99) | 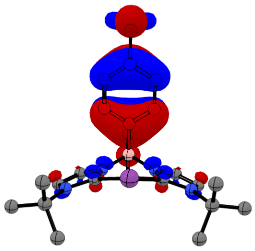 HOMO-1 | 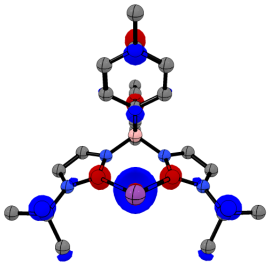 LUMO+1<br>f=0.77 |

### 10.10 Reaction profile of hydrodefluorination of pentafluoropyridine

The exploration of the potential energy surface (PES) was performed at the PBE0-D3(BJ)/def2-SVP level of theory, using CPCM with benzene as implicit solvation. All optimized structure energies were later refined at the PBE0(D3BJ) CPCM($C_6H_6$)/def2-TZVPP level of theory. The nature of the stationary points was characterized with the normal mode analysis (no imaginary frequencies for local minima and one imaginary frequency for transition states). All transition states were confirmed either by IRC or by optimizing the geometries following the negative eigenvalue of the Hessian. The lowest energy pathway for the formation of 13a is presented in Figure S52 and discussed in the main text (Figure 5).

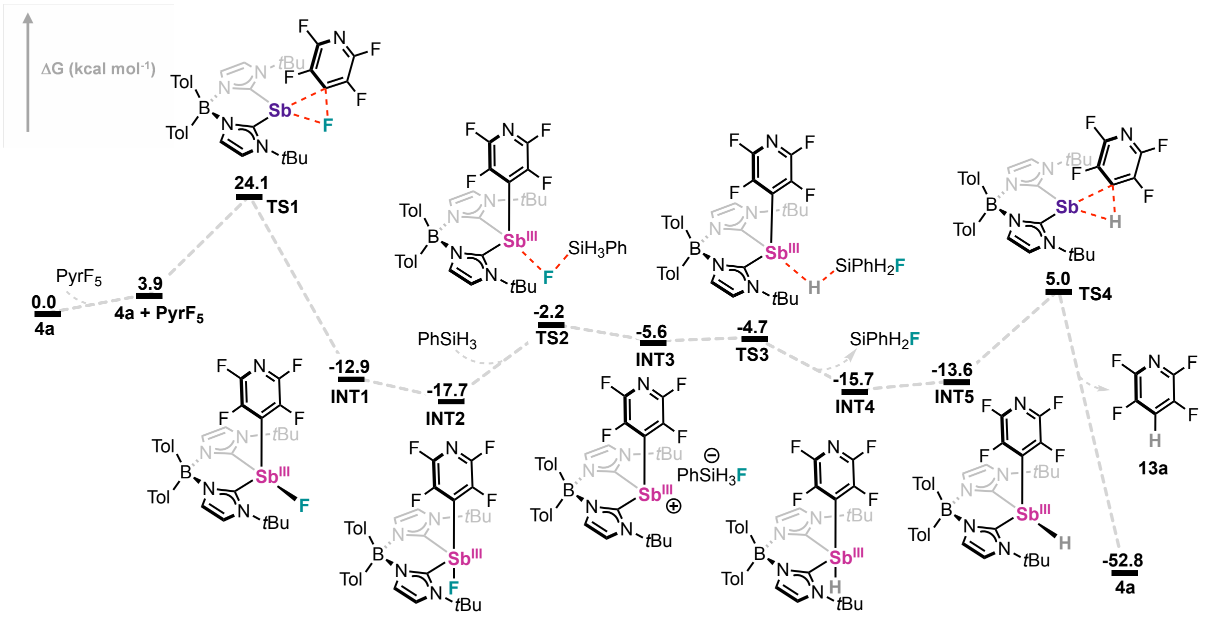


Figure S52. Gibbs energy profile (pathway A) for the stibinidene-catalysed hydrodefluorination of pentafluoropyridine with **4a** with phenylsilane. Thermal free in energy in kcal/mol. Calculated at the PBE0(D3BJ)CPCM($C_6H_6$)/def2-TZVPP//PBE0(D3BJ)/def2-SVP level of theory.

In addition to the stepwise metathesis leading to **INT4** and **INT5** as described in the main text, alternative mechanistic routes to the formation of the Sb(III)–H intermediate and the corresponding product of hydrodefluorination (**13a**) were identified. However, it is important to note that these alternative scenarios outlined involve significantly higher activation barriers compared to the PES in Figure S52. As such, these pathways are considered highly unlikely under the reaction conditions exployed.

In Figure S53, mechanism in which **INT1** undergoes a second oxidative addition is shown. In this case, Si–H bond oxidative addition into Sb(III) species **INT1** overcomes **TS1'** ($\Delta G^{\ddagger}$ = 70 kcal mol$^{-1}$), forming the hexacoordinated Sb(V) species **INT1'**. A subsequent Si–F reductive elimination from Sb(V) through **TS2'** ($\Delta G^{\ddagger}$ = 15.5 kcal mol$^{-1}$) results in **INT5**, which proceeds to the product and **4a** as outlined before.

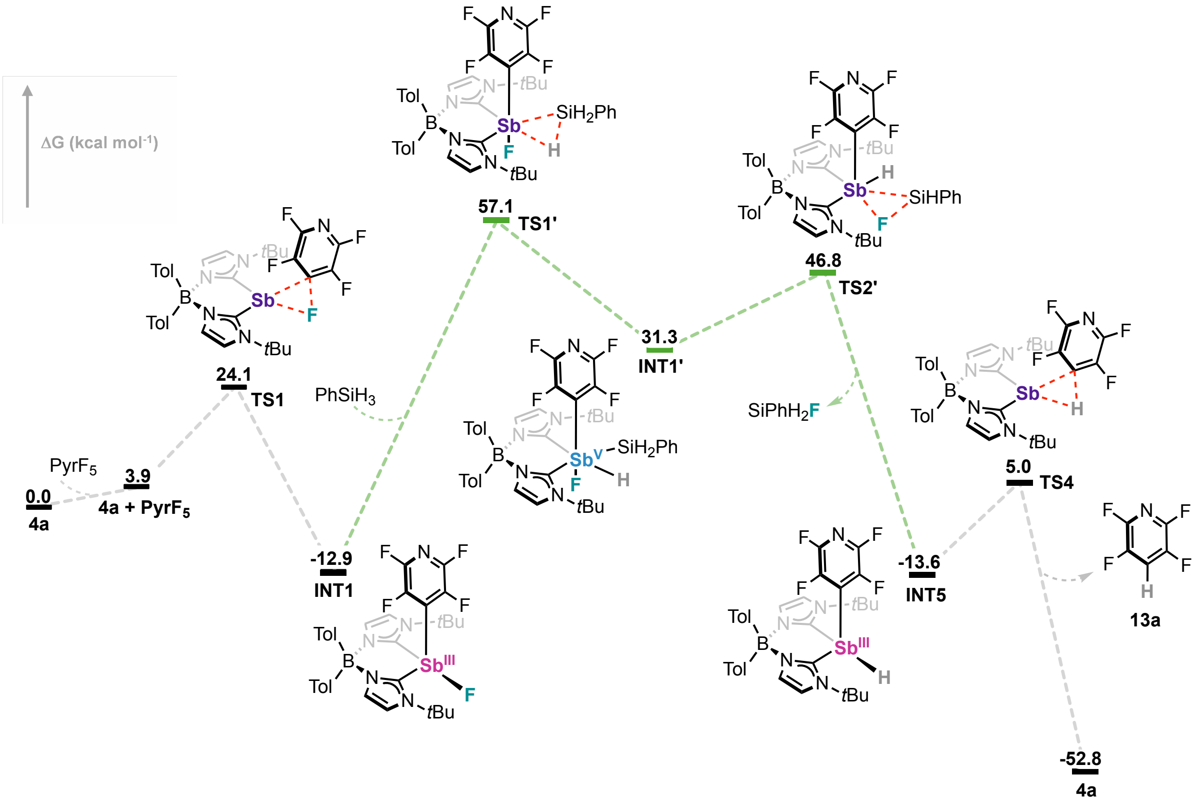


Figure S53. Gibbs energy profile (pathway B) for the stibinidene-catalyzed hydrodefluorination of pentafluoropyridine with **4a** with phenylsilane. Thermal free in energy in kcal/mol. Calculated at the PBE0(D3BJ)CPCM($C_6H_6$)/def2-TZVPP//PBE0(D3BJ)/def2-SVP level of theory.

Another alternative is outlined in Figure S54. In this case, a formal deprotonation of $PhSiH_3$ by the tetrafluoropyridine residue via **TS1”** ($\Delta G^{\ddagger}$ = 44.5 kcal mol$^{-1}$) to form **INT1”** and the hydrodefluorination product **13a**. The corresponding Sb(III) species I**NT1”** undergoes facile Si–F reductive elimination via **TS2”** ($\Delta G^{\ddagger}$ = 4.6 kcal mol$^{-1}$) to recover catalyst **4a** and produce $PhSiH_2F$ as product.

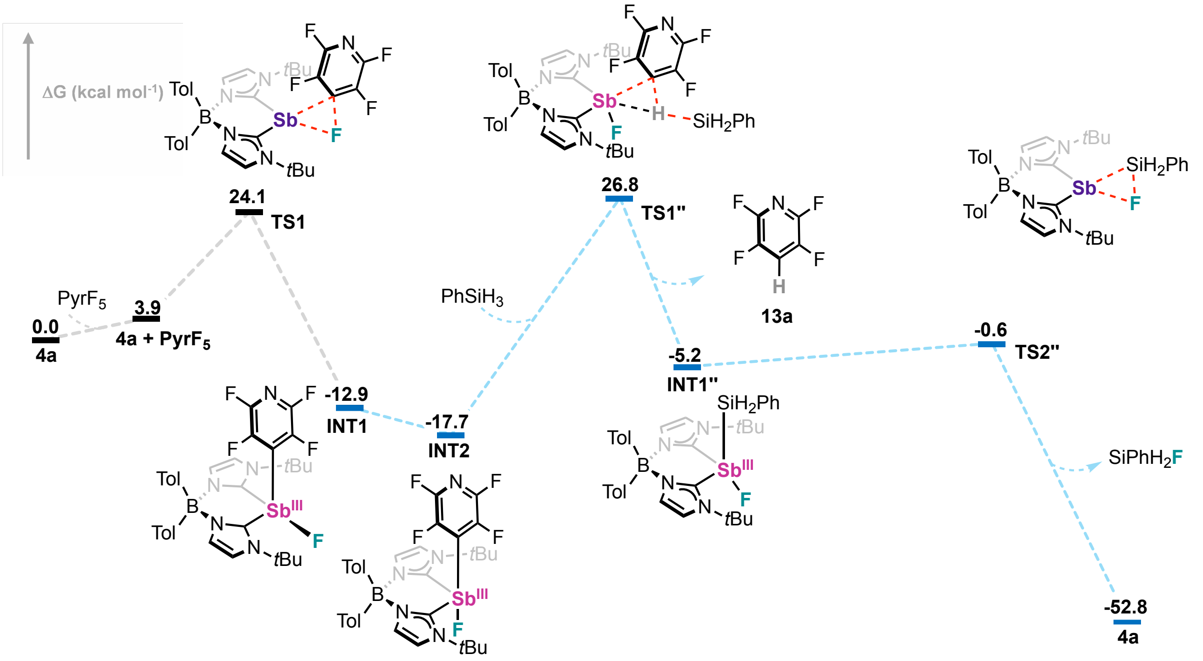


Figure S54. Gibbs energy profile (pathway D) for the stibinidene-catalyzed hydrodefluorination of pentafluoropyridine with **4a** with phenylsilane. Thermal free in energy in kcal/mol. Calculated at the PBE0(D3BJ)CPCM($C_6H_6$)/def2-TZVPP//PBE0(D3BJ)/def2-SVP level of theory.

Finally, after oxidative addition of C–F and formation of **INT1**, addition of phenylsilane can also trigger a concerted ligand metathesis with a five-membered ring transition state **TS1'''** ($\Delta G^{\ddagger}$ = 33.7 kcal mol$^{-1}$). In this concerted mechanism, the fluoride anion is abstracted from the Sb center, which is reduced from Sb(III) to Sb(I), while the H is transferred to the C of the tetrafluoropyridine residue.

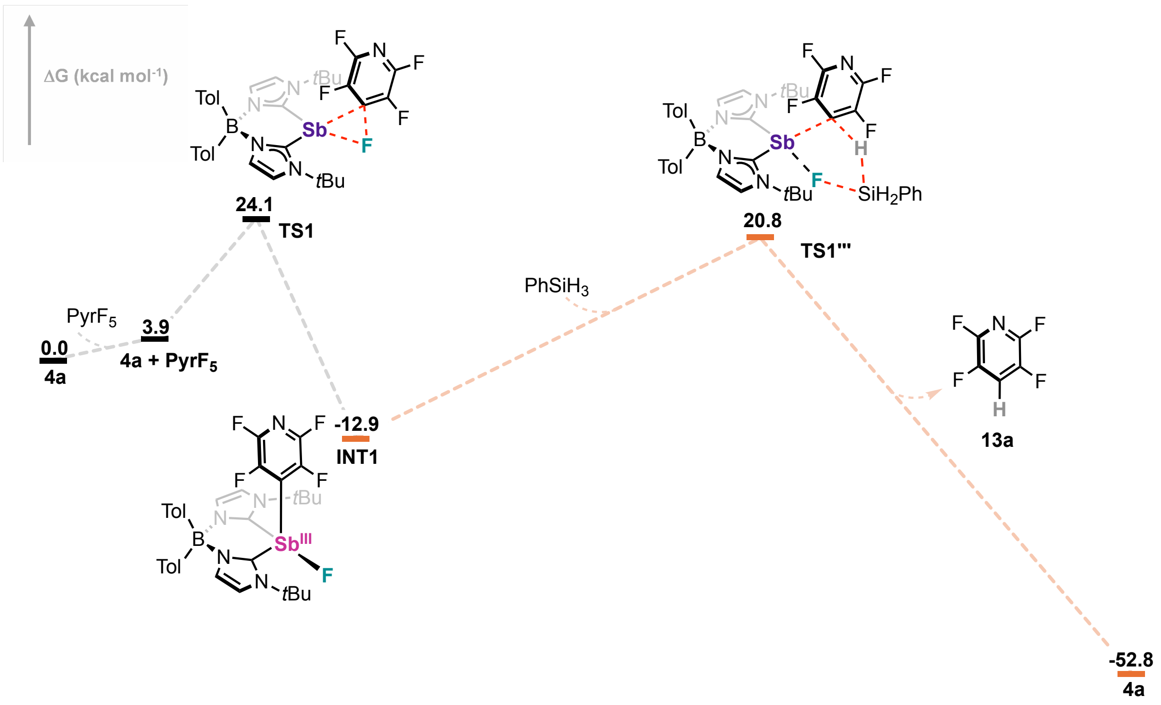


Figure S55. Gibbs energy profile (pathway C) for the stibinidene-catalyzed hydrodefluorination of pentafluoropyridine with **4a** with phenylsilane. Thermal free in energy in kcal/mol. Calculated at the PBE0(D3BJ)CPCM($C_6H_6$)/def2-TZVPP//PBE0(D3BJ)/def2-SVP level of theory.

## 10.11 xyz coordinates (in Å) and Energies (in Hartree)

### 10.11.1 Singlet-triplet gaps

#### *10.11.1.1 **4a** (singlet)*

**Energy(PBE0-D3(BJ)/def2-SVP) =** -1570.44544343

| | | | |
|---|---|---|---|
| 6 | 1.083263 | -2.414389 | -1.021998 |
| 7 | 0.473987 | -1.272687 | -0.561224 |
| 6 | -0.845104 | -1.503130 | -0.415995 |
| 6 | 0.129213 | -3.370360 | -1.158970 |
| 1 | 0.219175 | -4.399766 | -1.482354 |
| 1 | 2.152881 | -2.457197 | -1.201098 |
| 7 | -1.072908 | -2.798826 | -0.780449 |
| 6 | -2.388115 | -3.484137 | -0.840683 |
| 51 | -2.237829 | 0.000002 | 0.261411 |
| 7 | 0.473996 | 1.272709 | -0.561165 |
| 6 | -0.845094 | 1.503154 | -0.415929 |
| 6 | 0.129238 | 3.370412 | -1.158817 |
| 7 | -1.072888 | 2.798868 | -0.780325 |
| 1 | 0.219207 | 4.399831 | -1.482154 |
| 1 | 2.152899 | 2.457233 | -1.200988 |
| 6 | 1.083281 | 2.414427 | -1.021889 |
| 6 | -2.388091 | 3.484190 | -0.840529 |
| 5 | 1.249329 | -0.000003 | -0.050051 |
| 6 | -3.082219 | 3.362170 | 0.515518 |
| 1 | -3.348980 | 2.324224 | 0.760591 |
| 1 | -2.437347 | 3.746965 | 1.318997 |
| 1 | -4.016971 | 3.941179 | 0.505803 |
| 6 | -2.177459 | 4.967884 | -1.135198 |
| 1 | -1.558486 | 5.454602 | -0.366820 |
| 1 | -1.721763 | 5.133297 | -2.122057 |
| 1 | -3.158039 | 5.463448 | -1.140343 |
| 6 | -3.220073 | 2.860368 | -1.961188 |
| 1 | -2.717065 | 2.995928 | -2.930385 |
| 1 | -3.362767 | 1.782047 | -1.791376 |
| 1 | -4.208083 | 3.341494 | -2.009798 |
| 6 | -3.220098 | -2.860249 | -1.961305 |
| 1 | -3.362786 | -1.781937 | -1.791433 |
| 1 | -2.717095 | -2.995758 | -2.930512 |
| 1 | -4.208111 | -3.341367 | -2.009937 |
| 6 | -3.082237 | -3.362188 | 0.515373 |
| 1 | -2.437363 | -3.747028 | 1.318828 |
| 1 | -3.348993 | -2.324254 | 0.760504 |
| 1 | -4.016990 | -3.941193 | 0.505631 |

| | | | |
|---|---|---|---|
| 6 | -2.177493 | -4.967816 | -1.135434 |
| 1 | -1.721804 | -5.133178 | -2.122304 |
| 1 | -1.558516 | -5.454578 | -0.367086 |
| 1 | -3.158075 | -5.463375 | -1.140598 |
| 6 | 1.262108 | -0.000041 | 1.567251 |
| 6 | 1.314980 | -1.191793 | 2.301920 |
| 6 | 1.314949 | 1.191668 | 2.301979 |
| 6 | 1.406851 | -1.196250 | 3.691968 |
| 6 | 1.406821 | 1.196052 | 3.692035 |
| 6 | 1.450351 | -0.000114 | 4.416267 |
| 1 | 1.280189 | -2.150630 | 1.774623 |
| 1 | 1.280134 | 2.150533 | 1.774735 |
| 1 | 1.443624 | -2.149162 | 4.229421 |
| 1 | 1.443568 | 2.148934 | 4.229540 |
| 6 | 2.731808 | 0.000011 | -0.704127 |
| 6 | 2.906745 | 0.000098 | -2.100468 |
| 6 | 3.895297 | -0.000068 | 0.071941 |
| 6 | 4.168505 | 0.000105 | -2.685255 |
| 6 | 5.165841 | -0.000062 | -0.508522 |
| 6 | 5.327658 | 0.000029 | -1.895599 |
| 1 | 2.024530 | 0.000155 | -2.749855 |
| 1 | 3.805539 | -0.000144 | 1.161727 |
| 1 | 4.261741 | 0.000165 | -3.776015 |
| 1 | 6.052382 | -0.000135 | 0.133004 |
| 6 | 1.508366 | -0.000179 | 5.916942 |
| 1 | 2.028604 | -0.890184 | 6.301366 |
| 1 | 2.026909 | 0.890749 | 6.301504 |
| 1 | 0.495241 | -0.001194 | 6.354207 |
| 6 | 6.688831 | 0.000100 | -2.529695 |
| 1 | 6.832538 | -0.884808 | -3.170577 |
| 1 | 6.832983 | 0.885804 | -3.169385 |
| 1 | 7.485667 | -0.000593 | -1.772517 |

*10.11.1.2* ***4a*** *(triplet)*

**Energy(PBE0-D3(BJ)/def2-SVP) =** -1570.38470393

| | | | |
|---|---|---|---|
| 6 | -1.203380 | 2.693831 | -0.209521 |
| 7 | -0.574115 | 1.472704 | -0.300224 |
| 6 | 0.757279 | 1.642504 | -0.440397 |
| 6 | -0.244585 | 3.654489 | -0.304545 |
| 1 | -0.336214 | 4.735412 | -0.299578 |
| 1 | -2.281073 | 2.785521 | -0.104753 |
| 7 | 0.958933 | 2.985962 | -0.457795 |
| 6 | 2.287481 | 3.621460 | -0.532408 |
| 51 | 2.048190 | -0.300009 | 0.807514 |
| 7 | -0.441655 | -0.978371 | -0.919108 |
| 6 | 0.856919 | -1.285866 | -0.746559 |
| 6 | 0.033457 | -2.573238 | -2.367703 |
| 7 | 1.165299 | -2.265818 | -1.639129 |
| 1 | 0.015477 | -3.326094 | -3.145810 |
| 1 | -2.003642 | -1.697064 | -2.209319 |
| 6 | -0.962815 | -1.768203 | -1.908982 |
| 6 | 2.490678 | -2.915491 | -1.818427 |
| 5 | -1.254324 | 0.085008 | -0.061494 |
| 6 | 2.843959 | -3.678227 | -0.542388 |
| 1 | 2.899850 | -2.999928 | 0.323750 |
| 1 | 2.085215 | -4.446483 | -0.330921 |
| 1 | 3.819838 | -4.172161 | -0.659045 |
| 6 | 2.424648 | -3.898776 | -2.984559 |
| 1 | 1.708557 | -4.712623 | -2.799027 |
| 1 | 2.166304 | -3.399842 | -3.930324 |
| 1 | 3.416419 | -4.354962 | -3.108334 |
| 6 | 3.529241 | -1.842438 | -2.142838 |
| 1 | 3.238115 | -1.280761 | -3.042830 |
| 1 | 3.642886 | -1.128473 | -1.312925 |
| 1 | 4.507497 | -2.312277 | -2.320950 |
| 6 | 3.190092 | 2.775812 | -1.427428 |
| 1 | 3.320843 | 1.766227 | -1.014031 |
| 1 | 2.757784 | 2.677979 | -2.434041 |
| 1 | 4.178055 | 3.252788 | -1.510579 |
| 6 | 2.863242 | 3.705842 | 0.882732 |
| 1 | 2.203376 | 4.296886 | 1.535801 |
| 1 | 2.961489 | 2.699023 | 1.315697 |
| 1 | 3.856448 | 4.179525 | 0.871077 |
| 6 | 2.155608 | 5.021138 | -1.130008 |
| 1 | 1.657201 | 4.988842 | -2.110393 |
| 1 | 1.596146 | 5.702941 | -0.472928 |
| 1 | 3.157645 | 5.451807 | -1.267851 |
| 6 | -1.133654 | -0.332927 | 1.499790 |

| | | | |
|---|---|---|---|
| 6 | -1.082967 | 0.625376 | 2.524684 |
| 6 | -1.161086 | -1.677951 | 1.902951 |
| 6 | -1.043657 | 0.263547 | 3.868099 |
| 6 | -1.124645 | -2.046620 | 3.245885 |
| 6 | -1.057689 | -1.081778 | 4.256243 |
| 1 | -1.060679 | 1.687618 | 2.263223 |
| 1 | -1.207619 | -2.464280 | 1.142599 |
| 1 | -0.998061 | 1.041760 | 4.636359 |
| 1 | -1.143985 | -3.106573 | 3.517547 |
| 6 | -2.783685 | 0.132202 | -0.593678 |
| 6 | -3.094189 | 0.689713 | -1.846964 |
| 6 | -3.855275 | -0.392081 | 0.138544 |
| 6 | -4.394841 | 0.715293 | -2.341748 |
| 6 | -5.162205 | -0.369286 | -0.350724 |
| 6 | -5.458273 | 0.185319 | -1.598739 |
| 1 | -2.289824 | 1.116013 | -2.455659 |
| 1 | -3.663520 | -0.830641 | 1.121828 |
| 1 | -4.593361 | 1.155922 | -3.323988 |
| 1 | -5.971854 | -0.791092 | 0.252829 |
| 6 | -0.973857 | -1.472375 | 5.703394 |
| 1 | -1.503849 | -0.754266 | 6.347016 |
| 1 | -1.402946 | -2.469888 | 5.878199 |
| 1 | 0.074565 | -1.502556 | 6.045766 |
| 6 | -6.866408 | 0.241300 | -2.117169 |
| 1 | -7.304135 | 1.243146 | -1.970206 |
| 1 | -6.908790 | 0.026552 | -3.195883 |
| 1 | -7.515836 | -0.479328 | -1.599479 |

*10.11.1.3* ***5a** (singlet)*

**Energy(PBE0-D3(BJ)/def2-SVP) =** -1544.81843379

| | | | |
|---|---|---|---|
| 83 | 2.087667 | -0.000006 | 0.240438 |
| 7 | -0.722927 | -1.283564 | -0.590160 |
| 7 | 0.780328 | -2.841111 | -0.865204 |
| 7 | -0.722946 | 1.283803 | -0.589769 |
| 7 | 0.780342 | 2.841352 | -0.864592 |
| 6 | 0.591892 | -1.549430 | -0.473449 |
| 6 | -1.368830 | -2.404456 | -1.056748 |
| 1 | -2.442504 | -2.422303 | -1.213077 |
| 6 | -0.441020 | -3.379910 | -1.230352 |
| 1 | -0.562202 | -4.401319 | -1.569019 |
| 6 | 2.079684 | -3.548714 | -0.969445 |
| 6 | 2.827188 | -3.432702 | 0.358577 |
| 1 | 3.152953 | -2.404579 | 0.569640 |
| 1 | 2.197191 | -3.772086 | 1.193434 |
| 1 | 3.735026 | -4.052325 | 0.327173 |
| 6 | 2.880588 | -2.941690 | -2.121368 |
| 1 | 2.340868 | -3.071433 | -3.071403 |
| 1 | 3.044465 | -1.864918 | -1.959166 |
| 1 | 3.859185 | -3.437304 | -2.205090 |
| 6 | 1.833016 | -5.030163 | -1.247417 |
| 1 | 1.229672 | -5.499777 | -0.456228 |
| 1 | 1.341645 | -5.192451 | -2.217409 |
| 1 | 2.803010 | -5.545025 | -1.282487 |
| 6 | 0.591897 | 1.549559 | -0.473190 |
| 6 | -1.368876 | 2.404872 | -1.055882 |
| 1 | -2.442571 | 2.422814 | -1.212066 |
| 6 | -0.441043 | 3.380333 | -1.229347 |
| 1 | -0.562226 | 4.401842 | -1.567709 |
| 6 | 2.079725 | 3.548932 | -0.968696 |
| 6 | 2.827026 | 3.432959 | 0.359443 |
| 1 | 2.197012 | 3.772739 | 1.194130 |
| 1 | 3.152358 | 2.404767 | 0.570836 |
| 1 | 3.735079 | 4.052266 | 0.328044 |
| 6 | 2.880767 | 2.941894 | -2.120510 |
| 1 | 3.044676 | 1.865128 | -1.958307 |
| 1 | 2.341144 | 3.071607 | -3.070602 |
| 1 | 3.859354 | 3.437545 | -2.204129 |
| 6 | 1.833137 | 5.030376 | -1.246780 |
| 1 | 1.341794 | 5.192600 | -2.216798 |
| 1 | 1.229808 | 5.500099 | -0.455645 |
| 1 | 2.803162 | 5.545180 | -1.281858 |
| 6 | -1.425573 | -0.000240 | 1.564746 |
| 6 | -1.454734 | -1.191981 | 2.300664 |

| | | | |
|---|---|---|---|
| 1 | -1.438611 | -2.150559 | 1.772074 |
| 6 | -1.498438 | -1.196708 | 3.693008 |
| 1 | -1.517218 | -2.149726 | 4.231254 |
| 6 | -1.516109 | -0.000716 | 4.418659 |
| 6 | -1.498565 | 1.195518 | 3.693405 |
| 1 | -1.517446 | 2.148364 | 4.231954 |
| 6 | -1.454858 | 1.191261 | 2.301058 |
| 1 | -1.438835 | 2.150023 | 1.772805 |
| 6 | -1.521565 | -0.000961 | 5.920459 |
| 1 | -0.493824 | -0.000693 | 6.322221 |
| 1 | -2.027515 | 0.889255 | 6.323092 |
| 1 | -2.026951 | -0.891632 | 6.322796 |
| 6 | -2.978046 | 0.000118 | -0.646992 |
| 6 | -3.210226 | 0.000387 | -2.035249 |
| 1 | -2.355365 | 0.000545 | -2.720246 |
| 6 | -4.494468 | 0.000420 | -2.568700 |
| 1 | -4.631459 | 0.000599 | -3.654844 |
| 6 | -5.620878 | 0.000200 | -1.732934 |
| 6 | -5.402668 | -0.000076 | -0.353731 |
| 1 | -6.262348 | -0.000294 | 0.323388 |
| 6 | -4.109548 | -0.000107 | 0.174887 |
| 1 | -3.976434 | -0.000333 | 1.260187 |
| 6 | -7.006606 | 0.000463 | -2.311382 |
| 1 | -7.177030 | 0.886959 | -2.943501 |
| 1 | -7.175919 | -0.883630 | -2.947122 |
| 1 | -7.772077 | -0.001572 | -1.522490 |
| 5 | -1.469006 | 0.000019 | -0.051702 |

*10.11.1.4* ***5a** (triplet)*

**Energy(PBE0-D3(BJ)/def2-SVP) =** -1544.76976512

| | | | |
|---|---|---|---|
| 83 | 1.952945 | -0.262768 | 0.687144 |
| 7 | -0.680368 | -0.934164 | -1.016724 |
| 7 | 0.897968 | -2.147042 | -1.904143 |
| 7 | -0.858632 | 1.498128 | -0.305335 |
| 7 | 0.581620 | 3.090123 | -0.522136 |
| 6 | 0.628598 | -1.233240 | -0.934097 |
| 6 | -1.245424 | -1.665764 | -2.029461 |
| 1 | -2.301567 | -1.592964 | -2.269163 |
| 6 | -0.267325 | -2.423982 | -2.592557 |
| 1 | -0.319440 | -3.126223 | -3.415243 |
| 6 | 2.219227 | -2.762022 | -2.195334 |
| 6 | 2.660878 | -3.584019 | -0.985454 |
| 1 | 2.763996 | -2.952770 | -0.088891 |
| 1 | 1.925945 | -4.373366 | -0.767754 |
| 1 | 3.633321 | -4.056798 | -1.187553 |
| 6 | 3.220312 | -1.655472 | -2.525034 |
| 1 | 2.866418 | -1.054514 | -3.375783 |
| 1 | 3.373307 | -0.981069 | -1.669068 |
| 1 | 4.193283 | -2.097209 | -2.785465 |
| 6 | 2.098855 | -3.686114 | -3.404956 |
| 1 | 1.404059 | -4.518574 | -3.221029 |
| 1 | 1.780310 | -3.143934 | -4.307503 |
| 1 | 3.087289 | -4.120392 | -3.608671 |
| 6 | 0.453805 | 1.738003 | -0.508948 |
| 6 | -1.545599 | 2.684538 | -0.167605 |
| 1 | -2.619353 | 2.720114 | -0.005508 |
| 6 | -0.644666 | 3.694979 | -0.300545 |
| 1 | -0.792423 | 4.769438 | -0.277558 |
| 6 | 1.872054 | 3.791409 | -0.655182 |
| 6 | 2.532301 | 3.855365 | 0.723496 |
| 1 | 1.887321 | 4.387957 | 1.438759 |
| 1 | 2.705898 | 2.839726 | 1.109776 |
| 1 | 3.498903 | 4.378632 | 0.669190 |
| 6 | 2.753881 | 3.021208 | -1.635180 |
| 1 | 2.943522 | 2.001009 | -1.274811 |
| 1 | 2.267332 | 2.947491 | -2.618908 |
| 1 | 3.716456 | 3.540850 | -1.754004 |
| 6 | 1.639752 | 5.203227 | -1.189870 |
| 1 | 1.082579 | 5.181976 | -2.138394 |
| 1 | 1.092447 | 5.833907 | -0.474068 |
| 1 | 2.610589 | 5.685518 | -1.372833 |
| 6 | -1.233549 | -0.384014 | 1.467884 |
| 6 | -1.187836 | -1.739404 | 1.833006 |

| | | | |
|---|---|---|---|
| 1 | -1.251363 | -2.505853 | 1.053938 |
| 6 | -1.057291 | -2.142403 | 3.160545 |
| 1 | -1.021868 | -3.209244 | 3.401906 |
| 6 | -0.964732 | -1.203293 | 4.192790 |
| 6 | -1.023758 | 0.151726 | 3.843662 |
| 1 | -0.962108 | 0.910239 | 4.630358 |
| 6 | -1.157111 | 0.547657 | 2.515853 |
| 1 | -1.191791 | 1.616469 | 2.284050 |
| 6 | -0.781275 | -1.628666 | 5.620900 |
| 1 | 0.284127 | -1.610237 | 5.907069 |
| 1 | -1.313534 | -0.959150 | 6.313283 |
| 1 | -1.146219 | -2.652728 | 5.787331 |
| 6 | -3.031336 | 0.079238 | -0.492753 |
| 6 | -3.453628 | 0.667491 | -1.698780 |
| 1 | -2.712866 | 1.147429 | -2.346941 |
| 6 | -4.786603 | 0.657863 | -2.098939 |
| 1 | -5.071844 | 1.124737 | -3.047125 |
| 6 | -5.772907 | 0.059152 | -1.303544 |
| 6 | -5.366192 | -0.526750 | -0.102078 |
| 1 | -6.113231 | -1.002333 | 0.540971 |
| 6 | -4.027303 | -0.513490 | 0.292145 |
| 1 | -3.749706 | -0.978356 | 1.242203 |
| 6 | -7.215838 | 0.076069 | -1.718751 |
| 1 | -7.677734 | 1.055911 | -1.510337 |
| 1 | -7.328069 | -0.109643 | -2.797894 |
| 1 | -7.799840 | -0.683267 | -1.178920 |
| 5 | -1.465291 | 0.074381 | -0.069454 |

***10.11.1.5*** ***12$^{2+}$****(singlet)*

**Energy(PBE0-D3(BJ)/def2-SVP) =** -3140.53492024

| | | | |
|---|---|---|---|
| 7 | 3.01057 | 0.57327 | -1.32989 |
| 7 | 3.17221 | 0.71790 | 1.22355 |
| 7 | 1.76798 | 1.63889 | -2.78253 |
| 6 | 1.82700 | 1.18281 | -1.50133 |
| 6 | 5.23926 | -0.01467 | -0.15030 |
| 7 | 2.07143 | 1.84787 | 2.73832 |
| 6 | 1.98958 | 1.28949 | 1.49921 |
| 6 | 3.11306 | -1.63874 | 0.09179 |
| 6 | 5.86479 | 1.23987 | -0.28425 |
| 1 | 5.25485 | 2.14978 | -0.32193 |
| 6 | 3.69838 | 0.61314 | -2.51248 |
| 1 | 4.69615 | 0.19438 | -2.60652 |
| 6 | 2.92821 | 1.27506 | -3.42160 |
| 1 | 3.13864 | 1.52269 | -4.45512 |
| 6 | 6.07519 | -1.13545 | -0.10368 |
| 1 | 5.63850 | -2.13190 | 0.00441 |
| 6 | 2.89248 | -2.24663 | 1.33433 |
| 1 | 3.02413 | -1.66579 | 2.25205 |
| 6 | 2.99074 | -2.46229 | -1.03697 |
| 1 | 3.21092 | -2.05373 | -2.02657 |
| 6 | 8.07641 | 0.23313 | -0.32392 |
| 6 | 4.00329 | 0.88720 | 2.29711 |
| 1 | 5.02096 | 0.50772 | 2.29567 |
| 6 | -0.23448 | 1.89912 | 3.58296 |
| 1 | -0.65539 | 1.56119 | 2.62539 |
| 1 | -0.06008 | 1.02110 | 4.21875 |
| 1 | -1.00346 | 2.52099 | 4.06104 |
| 5 | 3.63649 | -0.12286 | -0.04015 |
| 6 | 7.24541 | 1.36443 | -0.36919 |
| 1 | 7.69593 | 2.35589 | -0.47177 |
| 6 | 0.71200 | 2.50835 | -3.39171 |
| 6 | 2.52950 | -3.58894 | 1.44250 |
| 1 | 2.38190 | -4.03146 | 2.43155 |
| 6 | 7.46402 | -1.01522 | -0.18880 |
| 1 | 8.08575 | -1.91362 | -0.14773 |
| 6 | 1.05116 | 2.70242 | 3.42272 |
| 6 | 0.66273 | 3.82618 | -2.61764 |
| 1 | 0.30441 | 3.69654 | -1.58602 |
| 1 | 1.65610 | 4.29698 | -2.59238 |
| 1 | -0.03165 | 4.51825 | -3.11422 |
| 6 | 2.36586 | -4.39100 | 0.30775 |

| | | | |
|---|---|---|---|
| 6 | 3.32166 | 1.59072 | 3.24464 |
| 1 | 3.64651 | 1.92843 | 4.22146 |
| 6 | -0.62303 | 1.77367 | -3.36229 |
| 1 | -0.59111 | 0.86807 | -3.98223 |
| 1 | -0.93476 | 1.48963 | -2.34747 |
| 1 | -1.40985 | 2.42934 | -3.75887 |
| 6 | 9.56561 | 0.37275 | -0.42259 |
| 1 | 9.85928 | 0.78771 | -1.39989 |
| 1 | 9.95256 | 1.05957 | 0.34596 |
| 1 | 10.07273 | -0.59379 | -0.30143 |
| 6 | 1.07302 | 2.80331 | -4.84467 |
| 1 | 2.00512 | 3.37903 | -4.93388 |
| 1 | 1.15296 | 1.88691 | -5.44741 |
| 1 | 0.27085 | 3.41312 | -5.28143 |
| 6 | 1.55742 | 3.08683 | 4.81025 |
| 1 | 1.75676 | 2.20656 | 5.43840 |
| 1 | 2.45931 | 3.71352 | 4.76551 |
| 1 | 0.77720 | 3.67673 | 5.30966 |
| 6 | 2.61692 | -3.79966 | -0.93787 |
| 1 | 2.53718 | -4.40855 | -1.84325 |
| 6 | 0.85209 | 3.97626 | 2.60113 |
| 1 | 1.81278 | 4.48856 | 2.44709 |
| 1 | 0.39187 | 3.78213 | 1.62167 |
| 1 | 0.18320 | 4.66113 | 3.14072 |
| 6 | 1.99553 | -5.83983 | 0.41658 |
| 1 | 1.53958 | -6.07298 | 1.38895 |
| 1 | 1.29345 | -6.14057 | -0.37536 |
| 1 | 2.88743 | -6.47920 | 0.31367 |
| 51 | 0.33333 | 1.43324 | 0.07719 |
| 7 | -3.17207 | -0.71847 | 1.22312 |
| 7 | -3.01041 | -0.57271 | -1.33023 |
| 7 | -2.07139 | -1.84939 | 2.73727 |
| 6 | -1.98943 | -1.29019 | 1.49854 |
| 6 | -5.23909 | 0.01474 | -0.15038 |
| 7 | -1.76766 | -1.63748 | -2.78336 |
| 6 | -1.82673 | -1.18197 | -1.50197 |
| 6 | -3.11297 | 1.63875 | 0.09231 |
| 6 | -5.86461 | -1.23975 | -0.28487 |
| 1 | -5.25467 | -2.14964 | -0.32295 |
| 6 | -4.00324 | -0.88850 | 2.29650 |
| 1 | -5.02092 | -0.50906 | 2.29520 |
| 6 | -3.32167 | -1.59261 | 3.24364 |
| 1 | -3.64658 | -1.93093 | 4.22022 |
| 6 | -6.07505 | 1.13548 | -0.10323 |

| | | | |
|---|---|---|---|
| 1 | -5.63838 | 2.13190 | 0.00531 |
| 6 | -2.99079 | 2.46262 | -1.03611 |
| 1 | -3.21066 | 2.05432 | -2.02589 |
| 6 | -2.89246 | 2.24631 | 1.33513 |
| 1 | -3.02379 | 1.66502 | 2.25262 |
| 6 | -8.07625 | -0.23303 | -0.32403 |
| 6 | -3.69823 | -0.61219 | -2.51283 |
| 1 | -4.69606 | -0.19351 | -2.60666 |
| 6 | 0.62341 | -1.77165 | -3.36292 |
| 1 | 0.93502 | -1.48822 | -2.34788 |
| 1 | 0.59141 | -0.86564 | -3.98225 |
| 1 | 1.41035 | -2.42695 | -3.75986 |
| 5 | -3.63630 | 0.12289 | -0.04019 |
| 6 | -7.24524 | -1.36428 | -0.36983 |
| 1 | -7.69575 | -2.35571 | -0.47281 |
| 6 | -1.05123 | -2.70449 | 3.42116 |
| 6 | -2.61738 | 3.80016 | -0.93654 |
| 1 | -2.53760 | 4.40935 | -1.84171 |
| 6 | -7.46387 | 1.01527 | -0.18835 |
| 1 | -8.08561 | 1.91364 | -0.14680 |
| 6 | -0.71153 | -2.50646 | -3.39295 |
| 6 | -0.85233 | -3.97781 | 2.59873 |
| 1 | -0.39225 | -3.78308 | 1.61932 |
| 1 | -1.81308 | -4.48995 | 2.44448 |
| 1 | -0.18339 | -4.66311 | 3.13770 |
| 6 | -2.36676 | 4.39120 | 0.30918 |
| 6 | -2.92798 | -1.27358 | -3.42225 |
| 1 | -3.13837 | -1.52075 | -4.45590 |
| 6 | 0.23447 | -1.90141 | 3.58197 |
| 1 | 0.06010 | -1.02375 | 4.21825 |
| 1 | 0.65553 | -1.56296 | 2.62464 |
| 1 | 1.00337 | -2.52363 | 4.05973 |
| 6 | -9.56544 | -0.37258 | -0.42297 |
| 1 | -9.95221 | -1.06219 | 0.34313 |
| 1 | -9.85911 | -0.78410 | -1.40174 |
| 1 | -10.07272 | 0.59345 | -0.29844 |
| 6 | -1.55755 | -3.08971 | 4.80843 |
| 1 | -2.45953 | -3.71626 | 4.76330 |
| 1 | -1.75679 | -2.20982 | 5.43714 |
| 1 | -0.77742 | -3.68005 | 5.30746 |
| 6 | -1.07231 | -2.80058 | -4.84614 |
| 1 | -1.15232 | -1.88382 | -5.44832 |
| 1 | -2.00432 | -3.37639 | -4.93582 |
| 1 | -0.27000 | -3.41002 | -5.28316 |

| | | | |
|---|---|---|---|
| 6 | -2.52991 | 3.58861 | 1.44376 |
| 1 | -2.38229 | 4.03077 | 2.43299 |
| 6 | -0.66222 | -3.82476 | -2.61967 |
| 1 | -1.65552 | -4.29572 | -2.59489 |
| 1 | -0.30414 | -3.69566 | -1.58789 |
| 1 | 0.03237 | -4.51642 | -3.11652 |
| 6 | -1.99865 | 5.84050 | 0.41929 |
| 1 | -2.89569 | 6.47699 | 0.34707 |
| 1 | -1.32037 | 6.15047 | -0.38946 |
| 1 | -1.51674 | 6.06778 | 1.38054 |
| 51 | -0.33314 | -1.43328 | 0.07648 |

*10.11.1.6* ***12***$^{2+}$*(triplet)*

**Energy(PBE0-D3(BJ)/def2-SVP) =** -3140.48677725

| | | | |
|---|---|---|---|
| 7 | 3.74051 | 0.79207 | -1.28859 |
| 7 | 3.77370 | 0.81721 | 1.25343 |
| 7 | 2.67375 | 1.99389 | -2.77018 |
| 6 | 2.62091 | 1.49725 | -1.50738 |
| 6 | 5.57763 | -0.55576 | -0.02738 |
| 7 | 2.73164 | 2.02207 | 2.75013 |
| 6 | 2.65406 | 1.51552 | 1.49282 |
| 6 | 3.00928 | -1.34947 | 0.01678 |
| 6 | 6.60943 | 0.40309 | -0.05262 |
| 1 | 6.36032 | 1.47037 | -0.06327 |
| 6 | 4.51413 | 0.83191 | -2.41455 |
| 1 | 5.47425 | 0.32630 | -2.46716 |
| 6 | 3.85355 | 1.58301 | -3.34420 |
| 1 | 4.14590 | 1.85099 | -4.35278 |
| 6 | 5.96791 | -1.89964 | -0.01384 |
| 1 | 5.20713 | -2.68438 | 0.00676 |
| 6 | 2.65803 | -1.97453 | 1.22386 |
| 1 | 2.92813 | -1.50283 | 2.17420 |
| 6 | 2.64919 | -2.02236 | -1.16320 |
| 1 | 2.91220 | -1.58712 | -2.13280 |
| 6 | 8.33023 | -1.31348 | -0.05100 |
| 6 | 4.57337 | 0.87319 | 2.36035 |
| 1 | 5.53802 | 0.37469 | 2.39497 |
| 6 | 0.44094 | 1.96388 | 3.59918 |
| 1 | -0.00143 | 1.68674 | 2.63026 |
| 1 | 0.67808 | 1.04273 | 4.15109 |
| 1 | -0.33006 | 2.51269 | 4.15829 |
| 5 | 4.03784 | -0.09891 | -0.01233 |
| 6 | 7.95032 | 0.03780 | -0.06424 |
| 1 | 8.72289 | 0.81190 | -0.08380 |
| 6 | 1.63059 | 2.81900 | -3.44868 |
| 6 | 2.03525 | -3.22205 | 1.25371 |
| 1 | 1.82818 | -3.69860 | 2.21581 |
| 6 | 7.31324 | -2.27121 | -0.02545 |
| 1 | 7.57907 | -3.33170 | -0.01417 |
| 6 | 1.69066 | 2.83120 | 3.45083 |
| 6 | 1.40331 | 4.09456 | -2.64094 |
| 1 | 1.00670 | 3.88506 | -1.63643 |
| 1 | 2.33966 | 4.66076 | -2.53343 |
| 1 | 0.67138 | 4.73358 | -3.15453 |
| 6 | 1.72416 | -3.90721 | 0.07208 |

| | | | |
|---|---|---|---|
| 6 | 3.92831 | 1.62630 | 3.29934 |
| 1 | 4.24166 | 1.90452 | 4.29881 |
| 6 | 0.36162 | 1.97574 | -3.56397 |
| 1 | 0.56660 | 1.05204 | -4.12407 |
| 1 | -0.05898 | 1.70438 | -2.58393 |
| 1 | -0.41420 | 2.54005 | -4.10051 |
| 6 | 9.77795 | -1.70229 | -0.06410 |
| 1 | 10.28383 | -1.31619 | -0.96292 |
| 1 | 10.30849 | -1.28470 | 0.80591 |
| 1 | 9.90526 | -2.79298 | -0.04659 |
| 6 | 2.10949 | 3.19590 | -4.84688 |
| 1 | 3.03086 | 3.79513 | -4.81917 |
| 1 | 2.26965 | 2.31373 | -5.48334 |
| 1 | 1.33443 | 3.80896 | -5.32602 |
| 6 | 2.19657 | 3.22090 | 4.83596 |
| 1 | 2.38634 | 2.34388 | 5.47135 |
| 1 | 3.10709 | 3.83494 | 4.78401 |
| 1 | 1.42334 | 3.82308 | 5.33156 |
| 6 | 2.02612 | -3.26854 | -1.13966 |
| 1 | 1.81138 | -3.78198 | -2.08112 |
| 6 | 1.42031 | 4.10008 | 2.64592 |
| 1 | 2.34337 | 4.68276 | 2.51426 |
| 1 | 1.00145 | 3.88362 | 1.65211 |
| 1 | 0.69066 | 4.72709 | 3.17717 |
| 6 | 1.11471 | -5.27754 | 0.09823 |
| 1 | 1.25571 | -5.76234 | 1.07400 |
| 1 | 0.02976 | -5.24868 | -0.09993 |
| 1 | 1.56102 | -5.92696 | -0.66894 |
| 51 | 1.07984 | 1.63449 | 0.00984 |
| 7 | -3.77353 | -0.81746 | 1.25310 |
| 7 | -3.74021 | -0.79100 | -1.28892 |
| 7 | -2.73182 | -2.02330 | 2.74929 |
| 6 | -2.65413 | -1.51629 | 1.49215 |
| 6 | -5.57745 | 0.55602 | -0.02715 |
| 7 | -2.67353 | -1.99185 | -2.77137 |
| 6 | -2.62073 | -1.49621 | -1.50815 |
| 6 | -3.00919 | 1.34993 | 0.01752 |
| 6 | -6.60915 | -0.40293 | -0.05315 |
| 1 | -6.35991 | -1.47016 | -0.06444 |
| 6 | -4.57316 | -0.87359 | 2.36005 |
| 1 | -5.53765 | -0.37481 | 2.39489 |
| 6 | -3.92831 | -1.62727 | 3.29872 |
| 1 | -4.24172 | -1.90574 | 4.29810 |
| 6 | -5.96788 | 1.89986 | -0.01292 |

| | | | |
|---|---|---|---|
| 1 | -5.20718 | 2.68467 | 0.00818 |
| 6 | -2.64920 | 2.02348 | -1.16209 |
| 1 | -2.91236 | 1.58885 | -2.13192 |
| 6 | -2.65780 | 1.97429 | 1.22492 |
| 1 | -2.92784 | 1.50209 | 2.17502 |
| 6 | -8.33013 | 1.31345 | -0.05077 |
| 6 | -4.51367 | -0.82970 | -2.41503 |
| 1 | -5.47366 | -0.32382 | -2.46735 |
| 6 | -0.36114 | -1.97377 | -3.56450 |
| 1 | 0.05931 | -1.70369 | -2.58404 |
| 1 | -0.56558 | -1.04935 | -4.12361 |
| 1 | 0.41458 | -2.53781 | -4.10146 |
| 5 | -4.03762 | 0.09930 | -0.01221 |
| 6 | -7.95008 | -0.03777 | -0.06478 |
| 1 | -8.72256 | -0.81194 | -0.08500 |
| 6 | -1.69109 | -2.83304 | 3.44967 |
| 6 | -2.02610 | 3.26964 | -1.13790 |
| 1 | -1.81149 | 3.78364 | -2.07908 |
| 6 | -7.31324 | 2.27128 | -0.02453 |
| 1 | -7.57920 | 3.33173 | -0.01273 |
| 6 | -1.63049 | -2.81662 | -3.45050 |
| 6 | -1.42127 | -4.10184 | 2.64445 |
| 1 | -1.00231 | -3.88541 | 1.65069 |
| 1 | -2.34455 | -4.68414 | 2.51267 |
| 1 | -0.69186 | -4.72923 | 3.17558 |
| 6 | -1.72400 | 3.90762 | 0.07417 |
| 6 | -3.85309 | -1.58010 | -3.34525 |
| 1 | -4.14534 | -1.84711 | -4.35412 |
| 6 | -0.44102 | -1.96624 | 3.59814 |
| 1 | -0.67771 | -1.04521 | 4.15042 |
| 1 | 0.00129 | -1.68891 | 2.62923 |
| 1 | 0.32986 | -2.51556 | 4.15691 |
| 6 | -9.77789 | 1.70210 | -0.06353 |
| 1 | -10.30736 | 1.28724 | 0.80846 |
| 1 | -10.28481 | 1.31311 | -0.96049 |
| 1 | -9.90527 | 2.79283 | -0.04921 |
| 6 | -2.19702 | -3.22294 | 4.83474 |
| 1 | -3.10781 | -3.83657 | 4.78268 |
| 1 | -2.38635 | -2.34604 | 5.47043 |
| 1 | -1.42401 | -3.82560 | 5.33008 |
| 6 | -2.10914 | -3.19174 | -4.84928 |
| 1 | -2.26880 | -2.30878 | -5.48477 |
| 1 | -3.03071 | -3.79069 | -4.82254 |
| 1 | -1.33415 | -3.80451 | -5.32890 |

| | | | |
|---|---|---|---|
| 6 | -2.03499 | 3.22178 | 1.25542 |
| 1 | -1.82789 | 3.69779 | 2.21778 |
| 6 | -1.40395 | -4.09322 | -2.64420 |
| 1 | -2.34054 | -4.65924 | -2.53775 |
| 1 | -1.00768 | -3.88510 | -1.63927 |
| 1 | -0.67203 | -4.73186 | -3.15827 |
| 6 | -1.11464 | 5.27798 | 0.10104 |
| 1 | -1.56044 | 5.92752 | -0.66631 |
| 1 | -0.02954 | 5.24923 | -0.09631 |
| 1 | -1.25638 | 5.76256 | 1.07681 |
| 51 | -1.08006 | -1.63608 | 0.00916 |

*10.11.1.7 10a1+ (singlet)*

**Energy(PBE0-D3(BJ)/def2-SVP) =** -2028.94628597

| | | | |
|---|---|---|---|
| 51 | -0.16054 | -2.08558 | 0.439573 |
| 6 | 1.436097 | -1.49946 | -0.90238 |
| 6 | 0.434813 | -0.52036 | 1.821281 |
| 6 | -1.63666 | -1.18694 | -1.04619 |
| 7 | 2.020108 | -0.29396 | -0.81288 |
| 7 | 2.037288 | -2.19041 | -1.90207 |
| 7 | 1.153125 | 0.546879 | 1.452884 |
| 7 | 0.255301 | -0.46825 | 3.169338 |
| 8 | -1.44156 | -1.64496 | -2.14212 |
| 6 | -2.76263 | -0.35855 | -0.64561 |
| 6 | 2.998813 | -0.21068 | -1.76745 |
| 5 | 1.528034 | 0.996614 | -0.02356 |
| 6 | 3.012415 | -1.38748 | -2.44932 |
| 6 | 1.733184 | -3.52298 | -2.5102 |
| 6 | 1.420751 | 1.301851 | 2.561015 |
| 6 | 0.862211 | 0.674909 | 3.635355 |
| 6 | -0.37497 | -1.5053 | 4.04471 |
| 6 | -3.75334 | -0.07564 | -1.59995 |
| 6 | -2.90005 | 0.161723 | 0.649169 |
| 1 | 3.602563 | 0.683707 | -1.88675 |
| 6 | 0.210025 | 1.583229 | -0.74089 |
| 6 | 2.753656 | 2.038227 | 0.062153 |
| 1 | 3.633383 | -1.706 | -3.27962 |
| 6 | 0.674986 | -4.27782 | -1.72363 |
| 6 | 3.023927 | -4.34212 | -2.52447 |
| 6 | 1.20936 | -3.26457 | -3.92442 |
| 1 | 1.992956 | 2.222746 | 2.498882 |
| 1 | 0.860466 | 0.961684 | 4.679856 |
| 6 | -0.45394 | -0.97646 | 5.474404 |
| 6 | 0.500518 | -2.7583 | 4.020613 |
| 6 | -1.7938 | -1.79577 | 3.561141 |
| 1 | -3.63561 | -0.47797 | -2.60806 |
| 6 | -4.86018 | 0.688443 | -1.27219 |
| 6 | -3.99622 | 0.926056 | 0.991172 |
| 1 | -2.11816 | -0.01694 | 1.388603 |
| 6 | -0.0716 | 1.372669 | -2.09726 |
| 6 | -0.66046 | 2.433269 | -0.04286 |
| 6 | 3.970992 | 1.674239 | 0.668238 |
| 6 | 2.67258 | 3.332376 | -0.46257 |
| 1 | 0.984197 | -4.47975 | -0.68805 |
| 1 | -0.29304 | -3.75492 | -1.74744 |

| | | | |
|---|---|---|---|
| 1 | 0.517338 | -5.25037 | -2.2101 |
| 1 | 3.809538 | -3.8776 | -3.13602 |
| 1 | 3.413874 | -4.47933 | -1.50505 |
| 1 | 2.820851 | -5.33393 | -2.95176 |
| 1 | 0.301299 | -2.64606 | -3.88292 |
| 1 | 1.95549 | -2.75794 | -4.55257 |
| 1 | 0.960851 | -4.2211 | -4.40581 |
| 1 | 0.540693 | -0.80726 | 5.910552 |
| 1 | -1.04151 | -0.04901 | 5.538829 |
| 1 | -0.95618 | -1.73086 | 6.094601 |
| 1 | 0.547348 | -3.21043 | 3.019469 |
| 1 | 1.524537 | -2.5218 | 4.344331 |
| 1 | 0.087935 | -3.51199 | 4.706134 |
| 1 | -2.41049 | -0.88581 | 3.565933 |
| 1 | -1.82382 | -2.23435 | 2.552522 |
| 1 | -2.26313 | -2.52874 | 4.231945 |
| 1 | -5.6173 | 0.890623 | -2.02936 |
| 6 | -4.98637 | 1.203824 | 0.030559 |
| 1 | -4.11477 | 1.349994 | 1.989377 |
| 1 | 0.573503 | 0.721763 | -2.69481 |
| 6 | -1.16964 | 1.96281 | -2.71926 |
| 6 | -1.75511 | 3.031279 | -0.65878 |
| 1 | -0.48448 | 2.638748 | 1.017945 |
| 1 | 4.08596 | 0.669764 | 1.09121 |
| 6 | 5.043362 | 2.554799 | 0.747235 |
| 6 | 3.746996 | 4.220955 | -0.38596 |
| 1 | 1.746982 | 3.657274 | -0.94515 |
| 8 | -5.99802 | 1.967241 | 0.438594 |
| 1 | -1.36199 | 1.761331 | -3.77692 |
| 6 | -2.03425 | 2.804771 | -2.01164 |
| 1 | -2.41425 | 3.681861 | -0.07693 |
| 1 | 5.973746 | 2.235955 | 1.226601 |
| 6 | 4.950963 | 3.851593 | 0.218616 |
| 1 | 3.646489 | 5.225234 | -0.80706 |
| 6 | -7.03344 | 2.29656 | -0.45854 |
| 6 | -3.20202 | 3.466587 | -2.68052 |
| 6 | 6.113614 | 4.796115 | 0.298352 |
| 1 | -7.73717 | 2.925016 | 0.099317 |
| 1 | -6.65138 | 2.863504 | -1.32374 |
| 1 | -7.55892 | 1.395485 | -0.8152 |
| 1 | -2.91698 | 4.451441 | -3.08649 |
| 1 | -3.5827 | 2.865824 | -3.51911 |
| 1 | -4.02679 | 3.633454 | -1.97198 |
| 1 | 6.460712 | 4.915595 | 1.336505 |

| | | | |
|---|---|---|---|
| 1 | 6.969735 | 4.420625 | -0.28485 |
| 1 | 5.854018 | 5.790629 | -0.08942 |

## 10.11.2 Mechanism

### *10.11.2.1 4a*

**Energy (PBE0-D3(BJ)CPCM($C_6H_6$)/def2-SVP) =** -1571.857347

| | | | |
|---|---|---|---|
| 6 | 1.071162 | -2.41883 | -1.00963 |
| 7 | 0.462354 | -1.27415 | -0.55332 |
| 6 | -0.85632 | -1.50585 | -0.40421 |
| 6 | 0.116787 | -3.37563 | -1.13936 |
| 1 | 0.206049 | -4.40639 | -1.45909 |
| 1 | 2.13997 | -2.46466 | -1.192 |
| 7 | -1.08399 | -2.80275 | -0.76161 |
| 6 | -2.39894 | -3.4933 | -0.81654 |
| 51 | -2.25176 | 0.000068 | 0.268512 |
| 7 | 0.462507 | 1.27419 | -0.5529 |
| 6 | -0.85613 | 1.506028 | -0.40369 |
| 6 | 0.117221 | 3.375863 | -1.13838 |
| 7 | -1.08362 | 2.803068 | -0.76072 |
| 1 | 0.206615 | 4.406693 | -1.45786 |
| 1 | 2.140267 | 2.4646 | -1.19137 |
| 6 | 1.071462 | 2.418897 | -1.00895 |
| 6 | -2.39847 | 3.49383 | -0.81545 |
| 5 | 1.241485 | -0.00011 | -0.05362 |
| 6 | -3.09737 | 3.355807 | 0.536174 |
| 1 | -3.37362 | 2.316481 | 0.764849 |
| 1 | -2.45389 | 3.725957 | 1.347513 |
| 1 | -4.02815 | 3.940889 | 0.530426 |
| 6 | -2.18205 | 4.980397 | -1.08985 |
| 1 | -1.56182 | 5.454122 | -0.31455 |
| 1 | -1.72602 | 5.15819 | -2.07424 |
| 1 | -3.16114 | 5.478615 | -1.08798 |
| 6 | -3.22733 | 2.886699 | -1.94723 |
| 1 | -2.71991 | 3.032261 | -2.9126 |
| 1 | -3.37699 | 1.807557 | -1.79125 |
| 1 | -4.21261 | 3.373179 | -1.99369 |
| 6 | -3.22759 | -2.88595 | -1.94836 |
| 1 | -3.37709 | -1.8068 | -1.79232 |
| 1 | -2.72011 | -3.03154 | -2.91369 |
| 1 | -4.21295 | -3.37227 | -1.99494 |
| 6 | -3.09794 | -3.35525 | 0.535029 |
| 1 | -2.45461 | -3.7256 | 1.346398 |
| 1 | -3.37399 | -2.31588 | 0.76376 |
| 1 | -4.02884 | -3.94014 | 0.529146 |
| 6 | -2.18274 | -4.97988 | -1.09103 |

| | | | |
|---|---|---|---|
| 1 | -1.72662 | -5.15768 | -2.07538 |
| 1 | -1.56267 | -5.45377 | -0.31569 |
| 1 | -3.16192 | -5.47794 | -1.08932 |
| 6 | 1.28403 | -0.00039 | 1.56465 |
| 6 | 1.355208 | -1.1923 | 2.298287 |
| 6 | 1.355097 | 1.191264 | 2.298703 |
| 6 | 1.483732 | -1.19711 | 3.686016 |
| 6 | 1.48362 | 1.195598 | 3.686442 |
| 6 | 1.547679 | -0.00088 | 4.409661 |
| 1 | 1.306643 | -2.15111 | 1.771846 |
| 1 | 1.306441 | 2.150258 | 1.772604 |
| 1 | 1.534403 | -2.15024 | 4.222096 |
| 1 | 1.534202 | 2.148541 | 4.222861 |
| 6 | 2.715612 | -3.1E-05 | -0.72926 |
| 6 | 2.867065 | 0.000713 | -2.1285 |
| 6 | 3.892833 | -0.00062 | 0.025984 |
| 6 | 4.118611 | 0.000824 | -2.73515 |
| 6 | 5.153949 | -0.00051 | -0.57594 |
| 6 | 5.292009 | 0.000212 | -1.96596 |
| 1 | 1.974057 | 0.001215 | -2.76286 |
| 1 | 3.82335 | -0.00119 | 1.117397 |
| 1 | 4.192763 | 0.001398 | -3.82737 |
| 1 | 6.051448 | -0.001 | 0.050278 |
| 6 | 1.648017 | -0.00116 | 5.908213 |
| 1 | 2.178305 | -0.89156 | 6.277523 |
| 1 | 2.176605 | 0.890055 | 6.277972 |
| 1 | 0.64767 | -0.00225 | 6.373777 |
| 6 | 6.641839 | 0.000398 | -2.62387 |
| 1 | 6.77331 | -0.88392 | -3.26807 |
| 1 | 6.774022 | 0.886065 | -3.26608 |
| 1 | 7.451963 | -0.00075 | -1.88101 |

*10.11.2.2 **4a**+$C_5F_5N$*

**Energy (PBE0-D3(BJ)CPCM($C_6H_6$)/def2-SVP) =** -2315.905906

| | | | |
|---|---|---|---|
| 6 | 2.347503 | 0.274744 | 2.53509 |
| 7 | 1.662745 | -0.13719 | 1.416766 |
| 6 | 0.515597 | -0.73056 | 1.797371 |
| 6 | 1.609535 | -0.06179 | 3.623871 |
| 1 | 1.807753 | 0.104539 | 4.675384 |
| 1 | 3.300999 | 0.788217 | 2.464433 |
| 7 | 0.467259 | -0.68828 | 3.159813 |
| 6 | -0.59479 | -1.25651 | 4.031263 |
| 51 | -0.89689 | -1.58874 | 0.409716 |
| 7 | 1.789909 | -0.9319 | -1.00896 |
| 6 | 0.656858 | -1.65538 | -1.08475 |
| 6 | 1.935213 | -2.15146 | -2.84517 |
| 7 | 0.729841 | -2.40989 | -2.21831 |
| 1 | 2.231947 | -2.62264 | -3.77396 |
| 1 | 3.551819 | -0.75776 | -2.23511 |
| 6 | 2.588171 | -1.23426 | -2.08642 |
| 6 | -0.28041 | -3.38593 | -2.70419 |
| 5 | 2.050747 | 0.287201 | -0.04846 |
| 6 | -1.64731 | -2.70658 | -2.76241 |
| 1 | -2.03979 | -2.45641 | -1.76748 |
| 1 | -1.60461 | -1.77872 | -3.35156 |
| 1 | -2.37669 | -3.38415 | -3.22863 |
| 6 | 0.088897 | -3.84086 | -4.11409 |
| 1 | 0.136632 | -2.99562 | -4.81653 |
| 1 | 1.044313 | -4.38422 | -4.13822 |
| 1 | -0.68863 | -4.5306 | -4.46982 |
| 6 | -0.28928 | -4.59358 | -1.76733 |
| 1 | 0.693848 | -5.08747 | -1.76995 |
| 1 | -0.52376 | -4.29174 | -0.73524 |
| 1 | -1.04597 | -5.31996 | -2.09795 |
| 6 | -0.53648 | -2.78158 | 3.949492 |
| 1 | -0.67722 | -3.12878 | 2.914662 |
| 1 | 0.436954 | -3.14701 | 4.309133 |
| 1 | -1.32724 | -3.22101 | 4.574846 |
| 6 | -1.9528 | -0.71711 | 3.58639 |
| 1 | -1.9522 | 0.381877 | 3.558667 |
| 1 | -2.24982 | -1.08177 | 2.593631 |
| 1 | -2.72976 | -1.04853 | 4.289961 |
| 6 | -0.35691 | -0.81904 | 5.474648 |
| 1 | 0.586294 | -1.21443 | 5.877984 |
| 1 | -0.36199 | 0.276306 | 5.575958 |

| | | | |
|---|---|---|---|
| 1 | -1.1709 | -1.21792 | 6.095234 |
| 6 | 1.125131 | 1.533119 | -0.50884 |
| 6 | 0.742105 | 2.534952 | 0.39227 |
| 6 | 0.775139 | 1.734416 | -1.8525 |
| 6 | 0.068037 | 3.683641 | -0.0237 |
| 6 | 0.098421 | 2.875646 | -2.27496 |
| 6 | -0.26051 | 3.88122 | -1.36859 |
| 1 | 0.984671 | 2.424484 | 1.454336 |
| 1 | 1.043546 | 0.97788 | -2.59697 |
| 1 | -0.21089 | 4.444842 | 0.711048 |
| 1 | -0.16048 | 2.992054 | -3.33203 |
| 6 | 3.634792 | 0.632619 | -0.08025 |
| 6 | 4.587635 | -0.33395 | 0.291501 |
| 6 | 4.130409 | 1.879188 | -0.47593 |
| 6 | 5.952961 | -0.0698 | 0.269631 |
| 6 | 5.50064 | 2.152147 | -0.50068 |
| 6 | 6.437619 | 1.185167 | -0.12884 |
| 1 | 4.247393 | -1.32606 | 0.607521 |
| 1 | 3.425757 | 2.660397 | -0.77443 |
| 1 | 6.661946 | -0.84949 | 0.566013 |
| 1 | 5.848977 | 3.140127 | -0.81721 |
| 6 | -0.99698 | 5.104186 | -1.82977 |
| 1 | -1.05889 | 5.861349 | -1.03525 |
| 1 | -0.50674 | 5.562609 | -2.70288 |
| 1 | -2.02535 | 4.843758 | -2.12692 |
| 6 | 7.912093 | 1.468619 | -0.14806 |
| 1 | 8.449578 | 0.752339 | -0.78989 |
| 1 | 8.122718 | 2.48072 | -0.52148 |
| 1 | 8.348654 | 1.384273 | 0.860389 |
| 6 | -3.91848 | 0.473024 | 0.313042 |
| 6 | -3.63667 | 1.733205 | 0.831846 |
| 6 | -3.8426 | 0.265943 | -1.06191 |
| 6 | -3.31509 | 2.739045 | -0.07573 |
| 6 | -3.48072 | 1.350999 | -1.85222 |
| 7 | -3.24978 | 2.542651 | -1.36604 |
| 9 | -3.67534 | 1.949423 | 2.134826 |
| 9 | -4.08393 | -0.92695 | -1.57494 |
| 9 | -3.08396 | 3.953143 | 0.382074 |
| 9 | -3.36712 | 1.178792 | -3.15488 |
| 9 | -4.24647 | -0.5156 | 1.114674 |

*10.11.2.3 TS1*

**Energy (PBE0-D3(BJ)CPCM($C_6H_6$)/def2-SVP) =** -2315.880343

| | | | |
|---|---|---|---|
| 6 | 2.093642 | -0.23398 | 2.404318 |
| 7 | 1.354949 | -0.37727 | 1.258161 |
| 6 | 0.27282 | -1.11894 | 1.536611 |
| 6 | 1.459145 | -0.91239 | 3.39783 |
| 1 | 1.734713 | -1.03488 | 4.437626 |
| 1 | 3.022211 | 0.327282 | 2.421105 |
| 7 | 0.33121 | -1.47577 | 2.846649 |
| 6 | -0.60872 | -2.3709 | 3.586412 |
| 51 | -1.27037 | -1.59651 | 0.082018 |
| 7 | 1.344273 | -0.56065 | -1.30287 |
| 6 | 0.220332 | -1.2706 | -1.477 |
| 6 | 1.415704 | -1.39394 | -3.34381 |
| 7 | 0.255535 | -1.80624 | -2.72858 |
| 1 | 1.680193 | -1.6726 | -4.35614 |
| 1 | 3.036873 | -0.09932 | -2.54528 |
| 6 | 2.088345 | -0.61803 | -2.45153 |
| 6 | -0.69314 | -2.79535 | -3.32303 |
| 5 | 1.758816 | 0.359645 | -0.08774 |
| 6 | -2.12042 | -2.27172 | -3.21887 |
| 1 | -2.48483 | -2.22206 | -2.18422 |
| 1 | -2.21166 | -1.26858 | -3.65575 |
| 1 | -2.7955 | -2.95258 | -3.756 |
| 6 | -0.35881 | -2.98739 | -4.80038 |
| 1 | -0.42062 | -2.04164 | -5.35842 |
| 1 | 0.634577 | -3.4335 | -4.95024 |
| 1 | -1.09505 | -3.67881 | -5.23151 |
| 6 | -0.52271 | -4.12532 | -2.58854 |
| 1 | 0.513398 | -4.48512 | -2.673 |
| 1 | -0.77402 | -4.0322 | -1.52175 |
| 1 | -1.19011 | -4.88015 | -3.02843 |
| 6 | -0.57953 | -3.75555 | 2.939294 |
| 1 | -0.94509 | -3.73481 | 1.902705 |
| 1 | 0.442841 | -4.16182 | 2.940057 |
| 1 | -1.22662 | -4.43913 | 3.507111 |
| 6 | -2.00709 | -1.76179 | 3.580485 |
| 1 | -2.00071 | -0.77214 | 4.05662 |
| 1 | -2.43018 | -1.63045 | 2.576334 |
| 1 | -2.68924 | -2.41949 | 4.13774 |
| 6 | -0.14495 | -2.49994 | 5.035292 |
| 1 | 0.851398 | -2.9586 | 5.114669 |
| 1 | -0.1416 | -1.52999 | 5.553064 |

| | | | |
|---|---|---|---|
| 1 | -0.85291 | -3.15305 | 5.562946 |
| 6 | 1.013304 | 1.779175 | -0.20483 |
| 6 | 1.092462 | 2.706996 | 0.843703 |
| 6 | 0.36809 | 2.203451 | -1.37385 |
| 6 | 0.564834 | 3.989545 | 0.729189 |
| 6 | -0.16928 | 3.482918 | -1.49118 |
| 6 | -0.08378 | 4.40085 | -0.44026 |
| 1 | 1.582362 | 2.426094 | 1.78101 |
| 1 | 0.276266 | 1.521943 | -2.22501 |
| 1 | 0.633782 | 4.681588 | 1.573104 |
| 1 | -0.6927 | 3.768242 | -2.4084 |
| 6 | 3.377711 | 0.474747 | -0.11245 |
| 6 | 4.17809 | -0.67162 | 0.044541 |
| 6 | 4.04869 | 1.681753 | -0.33454 |
| 6 | 5.566251 | -0.61429 | -0.00951 |
| 6 | 5.443323 | 1.74692 | -0.3913 |
| 6 | 6.22867 | 0.603284 | -0.22717 |
| 1 | 3.698347 | -1.64222 | 0.212199 |
| 1 | 3.466297 | 2.597095 | -0.46992 |
| 1 | 6.153382 | -1.52935 | 0.116892 |
| 1 | 5.931214 | 2.71006 | -0.56889 |
| 6 | -0.75238 | 5.737993 | -0.53411 |
| 1 | -0.3681 | 6.440841 | 0.219024 |
| 1 | -0.62555 | 6.189175 | -1.52985 |
| 1 | -1.83451 | 5.610394 | -0.36399 |
| 6 | 7.728028 | 0.667352 | -0.26499 |
| 1 | 8.08062 | 1.637353 | -0.64296 |
| 1 | 8.156436 | 0.5269 | 0.741268 |
| 1 | 8.148524 | -0.12326 | -0.90551 |
| 6 | -2.61111 | 0.3701 | 0.330643 |
| 6 | -2.19455 | 1.41328 | 1.214611 |
| 6 | -3.13133 | 0.903302 | -0.88761 |
| 6 | -2.48191 | 2.731894 | 0.94451 |
| 6 | -3.32156 | 2.254019 | -1.06082 |
| 7 | -3.05074 | 3.172847 | -0.15876 |
| 9 | -1.56925 | 1.082941 | 2.361747 |
| 9 | -3.45123 | 0.039183 | -1.87418 |
| 9 | -2.17849 | 3.645438 | 1.865654 |
| 9 | -3.82034 | 2.680494 | -2.22233 |
| 9 | -3.55681 | -0.56613 | 0.98747 |

*10.11.2.4 INT1*

**Energy (PBE0-D3(BJ)CPCM($C_6H_6$)/def2-SVP) =** -2315.939193

| | | | |
|---|---|---|---|
| 6 | -1.78203 | -0.39943 | -2.48152 |
| 7 | -1.13111 | -0.3941 | -1.27057 |
| 6 | -0.17346 | -1.33349 | -1.29481 |
| 6 | -1.22051 | -1.36727 | -3.24863 |
| 1 | -1.45785 | -1.68585 | -4.2577 |
| 1 | -2.59918 | 0.281759 | -2.69448 |
| 7 | -0.22579 | -1.95481 | -2.49605 |
| 6 | 0.491698 | -3.15655 | -3.01268 |
| 51 | 1.09776 | -1.75857 | 0.427359 |
| 7 | -1.31808 | 0.023941 | 1.276953 |
| 6 | -0.33947 | -0.76883 | 1.729262 |
| 6 | -1.60497 | -0.20163 | 3.455782 |
| 7 | -0.50125 | -0.92588 | 3.064873 |
| 1 | -1.95033 | -0.15542 | 4.480993 |
| 1 | -2.97809 | 1.025927 | 2.213522 |
| 6 | -2.11141 | 0.384948 | 2.338405 |
| 6 | 0.3083 | -1.78743 | 3.977737 |
| 5 | -1.53675 | 0.660433 | -0.15922 |
| 6 | 1.793901 | -1.4834 | 3.784311 |
| 1 | 2.173852 | -1.83468 | 2.813787 |
| 1 | 1.990409 | -0.40606 | 3.865055 |
| 1 | 2.372449 | -2.01105 | 4.555304 |
| 6 | -0.05341 | -1.46077 | 5.424807 |
| 1 | 0.136861 | -0.40456 | 5.666691 |
| 1 | -1.09982 | -1.70338 | 5.657496 |
| 1 | 0.575079 | -2.07619 | 6.082593 |
| 6 | -0.02379 | -3.25032 | 3.681747 |
| 1 | -1.06603 | -3.46456 | 3.96335 |
| 1 | 0.078744 | -3.49435 | 2.613525 |
| 1 | 0.633011 | -3.90563 | 4.272498 |
| 6 | -0.54859 | -4.26989 | -3.15519 |
| 1 | -1.00236 | -4.47477 | -2.17433 |
| 1 | -1.34472 | -4.00332 | -3.86484 |
| 1 | -0.06339 | -5.1874 | -3.51773 |
| 6 | 1.574796 | -3.63151 | -2.05854 |
| 1 | 2.347752 | -2.86547 | -1.90351 |
| 1 | 1.134766 | -3.95259 | -1.10352 |
| 1 | 2.068123 | -4.50379 | -2.50969 |
| 6 | 1.125615 | -2.78605 | -4.35347 |
| 1 | 0.377813 | -2.50881 | -5.10969 |
| 1 | 1.822685 | -1.94526 | -4.22615 |

| | | | |
|---|---|---|---|
| 1 | 1.68514 | -3.6483 | -4.74273 |
| 6 | -0.65122 | 2.001184 | -0.3049 |
| 6 | -0.0081 | 2.348101 | -1.50098 |
| 6 | -0.53237 | 2.910408 | 0.755051 |
| 6 | 0.742072 | 3.514855 | -1.62154 |
| 6 | 0.217357 | 4.079452 | 0.643587 |
| 6 | 0.883865 | 4.397664 | -0.54446 |
| 1 | -0.066 | 1.675794 | -2.36238 |
| 1 | -1.01516 | 2.694274 | 1.712306 |
| 1 | 1.252227 | 3.7349 | -2.56432 |
| 1 | 0.307842 | 4.749193 | 1.503762 |
| 6 | -3.12974 | 0.924946 | -0.31902 |
| 6 | -4.04167 | -0.13925 | -0.19319 |
| 6 | -3.66666 | 2.185706 | -0.59528 |
| 6 | -5.41155 | 0.048418 | -0.33407 |
| 6 | -5.04344 | 2.381062 | -0.73878 |
| 6 | -5.94093 | 1.318684 | -0.61088 |
| 1 | -3.66451 | -1.14528 | 0.020649 |
| 1 | -2.99243 | 3.040099 | -0.70254 |
| 1 | -6.08987 | -0.80422 | -0.2293 |
| 1 | -5.42721 | 3.382547 | -0.95585 |
| 6 | 1.779352 | 5.595897 | -0.64439 |
| 1 | 1.699816 | 6.082558 | -1.62803 |
| 1 | 1.550102 | 6.340554 | 0.131263 |
| 1 | 2.831865 | 5.291563 | -0.51482 |
| 6 | -7.42166 | 1.516491 | -0.76048 |
| 1 | -7.95564 | 1.250402 | 0.16604 |
| 1 | -7.66655 | 2.560321 | -1.00216 |
| 1 | -7.8315 | 0.878528 | -1.55978 |
| 6 | 2.084805 | 0.315298 | 0.016413 |
| 6 | 2.302831 | 1.333613 | 0.929899 |
| 6 | 2.763429 | 0.459471 | -1.18335 |
| 6 | 3.129579 | 2.408703 | 0.606999 |
| 6 | 3.57612 | 1.56206 | -1.42456 |
| 7 | 3.753912 | 2.510264 | -0.53938 |
| 9 | 2.643613 | -0.4717 | -2.14916 |
| 9 | 1.724829 | 1.324711 | 2.137265 |
| 9 | 3.307206 | 3.384499 | 1.487016 |
| 9 | 4.200544 | 1.681968 | -2.58878 |
| 9 | -0.26798 | -3.38008 | 0.544181 |

*10.11.2.5 INT2*

**Energy (PBE0-D3(BJ)CPCM($C_6H_6$)/def2-SVP) =** -2315.946483

| | | | |
|---|---|---|---|
| 6 | 1.698928 | 0.389886 | 2.630343 |
| 7 | 1.098541 | -0.02507 | 1.465755 |
| 6 | 0.043093 | -0.81146 | 1.749192 |
| 6 | 1.011337 | -0.16129 | 3.660255 |
| 1 | 1.173914 | -0.08164 | 4.729817 |
| 1 | 2.571452 | 1.035088 | 2.632841 |
| 7 | -0.00511 | -0.91393 | 3.108371 |
| 6 | -0.91217 | -1.67973 | 4.00532 |
| 51 | -1.3941 | -1.66424 | 0.05874 |
| 7 | 1.387736 | -0.62289 | -1.02464 |
| 6 | 0.419191 | -1.54153 | -1.19281 |
| 6 | 1.882127 | -1.76198 | -2.84702 |
| 7 | 0.734291 | -2.27877 | -2.29375 |
| 1 | 2.337257 | -2.16468 | -3.74323 |
| 1 | 3.16227 | -0.1019 | -2.12579 |
| 6 | 2.286925 | -0.73848 | -2.05339 |
| 6 | 0.045788 | -3.45117 | -2.92424 |
| 5 | 1.609828 | 0.502643 | 0.078217 |
| 6 | -0.87958 | -2.92444 | -4.01991 |
| 1 | -1.6482 | -2.28365 | -3.56716 |
| 1 | -0.30907 | -2.35633 | -4.77031 |
| 1 | -1.36703 | -3.77026 | -4.52745 |
| 6 | 1.115013 | -4.37227 | -3.52038 |
| 1 | 1.631778 | -3.92935 | -4.38273 |
| 1 | 1.862977 | -4.66344 | -2.76755 |
| 1 | 0.620789 | -5.28416 | -3.88203 |
| 6 | -0.73534 | -4.2506 | -1.88689 |
| 1 | -0.11959 | -4.47915 | -1.00337 |
| 1 | -1.65079 | -3.71862 | -1.60376 |
| 1 | -1.03989 | -5.20461 | -2.33928 |
| 6 | -1.94004 | -2.48561 | 3.230731 |
| 1 | -2.61159 | -1.84233 | 2.648299 |
| 1 | -1.46918 | -3.21678 | 2.560086 |
| 1 | -2.55623 | -3.03996 | 3.95261 |
| 6 | -1.63474 | -0.683 | 4.914978 |
| 1 | -0.93386 | -0.12494 | 5.551849 |
| 1 | -2.20791 | 0.036955 | 4.315237 |
| 1 | -2.32966 | -1.22148 | 5.575192 |
| 6 | -0.05488 | -2.64734 | 4.82651 |
| 1 | 0.487517 | -3.33873 | 4.164583 |
| 1 | 0.679566 | -2.12347 | 5.454016 |

| | | | |
|---|---|---|---|
| 1 | -0.7004 | -3.23901 | 5.491228 |
| 6 | 0.85737 | 1.869069 | -0.33252 |
| 6 | 0.217389 | 2.680554 | 0.614329 |
| 6 | 0.897434 | 2.354114 | -1.64706 |
| 6 | -0.37296 | 3.892927 | 0.26572 |
| 6 | 0.30846 | 3.564904 | -2.00451 |
| 6 | -0.35019 | 4.354294 | -1.05545 |
| 1 | 0.159365 | 2.352654 | 1.656651 |
| 1 | 1.385014 | 1.764077 | -2.42834 |
| 1 | -0.88245 | 4.486736 | 1.03078 |
| 1 | 0.341464 | 3.896661 | -3.04633 |
| 6 | 3.219665 | 0.708116 | 0.2177 |
| 6 | 4.045474 | -0.37542 | 0.569909 |
| 6 | 3.851826 | 1.940225 | 0.026526 |
| 6 | 5.420443 | -0.23691 | 0.716462 |
| 6 | 5.234476 | 2.087891 | 0.173132 |
| 6 | 6.044883 | 1.004627 | 0.518669 |
| 1 | 3.594169 | -1.35981 | 0.735508 |
| 1 | 3.249955 | 2.812371 | -0.24336 |
| 1 | 6.028004 | -1.10505 | 0.991176 |
| 1 | 5.691844 | 3.06943 | 0.015696 |
| 6 | -1.07179 | 5.608806 | -1.44703 |
| 1 | -1.01331 | 6.374529 | -0.65931 |
| 1 | -0.66993 | 6.035454 | -2.37734 |
| 1 | -2.14055 | 5.393225 | -1.61521 |
| 6 | 7.531098 | 1.150062 | 0.67376 |
| 1 | 8.071714 | 0.526451 | -0.0566 |
| 1 | 7.851329 | 2.191363 | 0.528446 |
| 1 | 7.862492 | 0.830125 | 1.674547 |
| 6 | -2.0694 | 0.400219 | -0.19721 |
| 6 | -2.77057 | 1.020786 | 0.824893 |
| 6 | -2.05565 | 1.05993 | -1.42105 |
| 6 | -3.40677 | 2.238194 | 0.601663 |
| 6 | -2.73193 | 2.273595 | -1.54403 |
| 7 | -3.38881 | 2.835115 | -0.56047 |
| 9 | -2.85868 | 0.45326 | 2.034431 |
| 9 | -1.40869 | 0.582677 | -2.47115 |
| 9 | -4.06395 | 2.823807 | 1.591524 |
| 9 | -2.72809 | 2.899105 | -2.71019 |
| 9 | -2.46498 | -1.82726 | -1.7316 |

*10.11.2.6 TS2*

**Energy (PBE0-D3(BJ)CPCM($C_6H_6$)/def2-SVP) =** -2838.585612

| | | | |
|---|---|---|---|
| 6 | -1.25004 | 1.791196 | -1.75067 |
| 7 | -0.56766 | 1.043491 | -0.82698 |
| 6 | -0.65333 | -0.23812 | -1.18421 |
| 6 | -1.75144 | 0.940816 | -2.68936 |
| 1 | -2.35362 | 1.152064 | -3.56358 |
| 1 | -1.34071 | 2.868793 | -1.65914 |
| 7 | -1.37718 | -0.33117 | -2.32571 |
| 6 | -1.69943 | -1.56858 | -3.10482 |
| 51 | 0.163428 | -1.83888 | -0.00244 |
| 7 | 0.287037 | 0.783197 | 1.612761 |
| 6 | 0.308553 | -0.54932 | 1.731019 |
| 6 | 0.391735 | 0.310625 | 3.763665 |
| 7 | 0.36474 | -0.86464 | 3.046718 |
| 1 | 0.43829 | 0.333149 | 4.846739 |
| 1 | 0.327344 | 2.403745 | 3.023639 |
| 6 | 0.340149 | 1.330184 | 2.867896 |
| 6 | 0.370004 | -2.17436 | 3.767779 |
| 5 | 0.268747 | 1.723508 | 0.340128 |
| 6 | 0.243246 | -3.35396 | 2.818268 |
| 1 | -0.69977 | -3.32306 | 2.252726 |
| 1 | 1.10391 | -3.43684 | 2.141077 |
| 1 | 0.226762 | -4.27264 | 3.420604 |
| 6 | 1.689545 | -2.27385 | 4.534827 |
| 1 | 2.54342 | -2.22773 | 3.844783 |
| 1 | 1.795198 | -1.46822 | 5.275063 |
| 1 | 1.727428 | -3.23067 | 5.074605 |
| 6 | -0.83082 | -2.18445 | 4.716068 |
| 1 | -0.76038 | -1.40205 | 5.484227 |
| 1 | -1.76614 | -2.03743 | 4.156338 |
| 1 | -0.87761 | -3.1539 | 5.231691 |
| 6 | -2.5936 | -2.48666 | -2.27914 |
| 1 | -2.12771 | -2.80076 | -1.33456 |
| 1 | -3.5416 | -1.99372 | -2.03118 |
| 1 | -2.80919 | -3.39516 | -2.85928 |
| 6 | -0.3986 | -2.26533 | -3.5001 |
| 1 | 0.265753 | -1.59091 | -4.05524 |
| 1 | 0.152747 | -2.65183 | -2.62861 |
| 1 | -0.6337 | -3.13334 | -4.13165 |
| 6 | -2.45012 | -1.17256 | -4.37295 |
| 1 | -3.4096 | -0.68893 | -4.13935 |
| 1 | -1.85245 | -0.51508 | -5.02094 |

| | | | |
|---|---|---|---|
| 1 | -2.6723 | -2.08725 | -4.93863 |
| 6 | 1.786524 | 1.963988 | -0.16157 |
| 6 | 2.057416 | 2.467395 | -1.44182 |
| 6 | 2.896541 | 1.723703 | 0.661332 |
| 6 | 3.358551 | 2.691066 | -1.88726 |
| 6 | 4.200269 | 1.945468 | 0.224439 |
| 6 | 4.458976 | 2.422713 | -1.06583 |
| 1 | 1.231953 | 2.675186 | -2.12863 |
| 1 | 2.748241 | 1.335248 | 1.673383 |
| 1 | 3.526776 | 3.065706 | -2.90105 |
| 1 | 5.039017 | 1.72778 | 0.892768 |
| 6 | -0.52943 | 3.069849 | 0.744535 |
| 6 | -1.84396 | 2.967519 | 1.240635 |
| 6 | 0.004949 | 4.353386 | 0.607286 |
| 6 | -2.58282 | 4.097272 | 1.570806 |
| 6 | -0.73669 | 5.491411 | 0.939538 |
| 6 | -2.04241 | 5.385006 | 1.423869 |
| 1 | -2.28307 | 1.972068 | 1.373152 |
| 1 | 1.024983 | 4.472713 | 0.230344 |
| 1 | -3.6026 | 3.982766 | 1.951512 |
| 1 | -0.2892 | 6.482814 | 0.819747 |
| 6 | 5.864985 | 2.575206 | -1.56363 |
| 1 | 5.915051 | 3.234202 | -2.44203 |
| 1 | 6.52857 | 2.982144 | -0.7861 |
| 1 | 6.273177 | 1.593943 | -1.85935 |
| 6 | -2.84896 | 6.59934 | 1.782989 |
| 1 | -3.13676 | 6.586675 | 2.846578 |
| 1 | -2.28866 | 7.526307 | 1.596103 |
| 1 | -3.78326 | 6.64444 | 1.200752 |
| 6 | 2.250523 | -1.32425 | -0.58924 |
| 6 | 2.661579 | -0.74217 | -1.78203 |
| 6 | 3.26991 | -1.72275 | 0.264879 |
| 6 | 4.024935 | -0.59659 | -2.04537 |
| 6 | 4.603258 | -1.52809 | -0.08182 |
| 7 | 4.958953 | -0.9863 | -1.21755 |
| 9 | 1.79579 | -0.31126 | -2.69254 |
| 9 | 2.981672 | -2.29798 | 1.438392 |
| 9 | 4.403731 | -0.05004 | -3.1861 |
| 9 | 5.555992 | -1.9014 | 0.753789 |
| 1 | -2.20657 | -2.56804 | 0.967143 |
| 14 | -3.42635 | -1.72143 | 1.408428 |
| 1 | -3.56543 | -1.32153 | 2.861066 |
| 9 | -2.31381 | -0.32811 | 1.184371 |
| 6 | -4.61159 | -1.02307 | 0.073589 |

| | | | |
|---|---|---|---|
| 6 | -4.40161 | 0.218124 | -0.55034 |
| 6 | -5.69654 | -1.7923 | -0.37461 |
| 6 | -5.22378 | 0.661963 | -1.58618 |
| 6 | -6.52356 | -1.36001 | -1.41242 |
| 6 | -6.28537 | -0.13006 | -2.02666 |
| 1 | -3.56141 | 0.831663 | -0.22305 |
| 1 | -5.87907 | -2.76294 | 0.096812 |
| 1 | -5.03371 | 1.632579 | -2.05375 |
| 1 | -7.35648 | -1.9855 | -1.74565 |
| 1 | -6.92945 | 0.212856 | -2.84114 |
| 1 | -4.25475 | -3.03526 | 1.581751 |

*10.11.2.7 INT3*

**Energy (PBE0-D3(BJ)CPCM($C_6H_6$)/def2-SVP) =** -2838.589543

| | | | |
|---|---|---|---|
| 6 | -1.43431 | 1.657474 | -1.66667 |
| 7 | -0.67819 | 0.939339 | -0.77852 |
| 6 | -0.64776 | -0.33209 | -1.18689 |
| 6 | -1.86788 | 0.802377 | -2.63428 |
| 1 | -2.50362 | 0.991406 | -3.48963 |
| 1 | -1.62282 | 2.717372 | -1.52903 |
| 7 | -1.37556 | -0.44282 | -2.32552 |
| 6 | -1.58211 | -1.67012 | -3.15723 |
| 51 | 0.334026 | -1.90132 | -0.08231 |
| 7 | 0.20463 | 0.668105 | 1.635487 |
| 6 | 0.309977 | -0.6653 | 1.702051 |
| 6 | 0.309961 | 0.119972 | 3.767671 |
| 7 | 0.368273 | -1.02459 | 3.006461 |
| 1 | 0.336274 | 0.103153 | 4.85152 |
| 1 | 0.122447 | 2.231602 | 3.107182 |
| 6 | 0.207203 | 1.168226 | 2.910617 |
| 6 | 0.423549 | -2.35838 | 3.67636 |
| 5 | 0.121626 | 1.649219 | 0.397906 |
| 6 | 0.411759 | -3.50282 | 2.676675 |
| 1 | -0.50833 | -3.51228 | 2.074564 |
| 1 | 1.301076 | -3.49663 | 2.031217 |
| 1 | 0.436496 | -4.44596 | 3.239506 |
| 6 | 1.714681 | -2.41521 | 4.494179 |
| 1 | 2.590969 | -2.29147 | 3.842671 |
| 1 | 1.744467 | -1.63682 | 5.269447 |
| 1 | 1.786667 | -3.38994 | 4.997147 |
| 6 | -0.81387 | -2.47385 | 4.569386 |
| 1 | -0.81071 | -1.72956 | 5.377708 |
| 1 | -1.73046 | -2.33641 | 3.977422 |
| 1 | -0.83861 | -3.47006 | 5.033144 |
| 6 | -2.30889 | -2.73618 | -2.34478 |
| 1 | -1.74114 | -3.06767 | -1.46503 |
| 1 | -3.28078 | -2.36785 | -1.99331 |
| 1 | -2.4746 | -3.61862 | -2.97879 |
| 6 | -0.22464 | -2.17108 | -3.64702 |
| 1 | 0.316249 | -1.3891 | -4.19578 |
| 1 | 0.416731 | -2.51396 | -2.82076 |
| 1 | -0.37637 | -3.03295 | -4.31182 |
| 6 | -2.44383 | -1.30978 | -4.36394 |
| 1 | -3.43723 | -0.94977 | -4.05904 |
| 1 | -1.96221 | -0.56037 | -5.00862 |

| | | | |
|---|---|---|---|
| 1 | -2.5909 | -2.21824 | -4.96315 |
| 6 | 1.612053 | 2.012878 | -0.10767 |
| 6 | 1.838979 | 2.539926 | -1.38642 |
| 6 | 2.734615 | 1.88378 | 0.72296 |
| 6 | 3.114397 | 2.892358 | -1.82376 |
| 6 | 4.012278 | 2.232935 | 0.293939 |
| 6 | 4.230287 | 2.734911 | -0.99495 |
| 1 | 1.000889 | 2.663927 | -2.0783 |
| 1 | 2.618041 | 1.480941 | 1.73365 |
| 1 | 3.251345 | 3.281241 | -2.83689 |
| 1 | 4.864608 | 2.09831 | 0.966963 |
| 6 | -0.74628 | 2.943733 | 0.837344 |
| 6 | -2.02276 | 2.775683 | 1.409065 |
| 6 | -0.30675 | 4.256168 | 0.638908 |
| 6 | -2.817 | 3.866057 | 1.745549 |
| 6 | -1.10338 | 5.354077 | 0.977259 |
| 6 | -2.37328 | 5.180543 | 1.532265 |
| 1 | -2.3891 | 1.761285 | 1.600396 |
| 1 | 0.682272 | 4.431586 | 0.206234 |
| 1 | -3.80537 | 3.696868 | 2.184505 |
| 1 | -0.72735 | 6.367455 | 0.806417 |
| 6 | 5.617655 | 3.029404 | -1.48128 |
| 1 | 5.606325 | 3.661447 | -2.38051 |
| 1 | 6.218622 | 3.534369 | -0.7101 |
| 1 | 6.139472 | 2.092164 | -1.73845 |
| 6 | -3.23787 | 6.351493 | 1.900049 |
| 1 | -3.44705 | 6.368228 | 2.981906 |
| 1 | -2.76048 | 7.305367 | 1.634698 |
| 1 | -4.21221 | 6.305075 | 1.387939 |
| 6 | 2.388046 | -1.13953 | -0.59495 |
| 6 | 2.779657 | -0.5337 | -1.77994 |
| 6 | 3.414007 | -1.46379 | 0.280397 |
| 6 | 4.132429 | -0.28261 | -2.0138 |
| 6 | 4.736502 | -1.17099 | -0.03685 |
| 7 | 5.075186 | -0.60126 | -1.16475 |
| 9 | 1.897349 | -0.17736 | -2.70979 |
| 9 | 3.140644 | -2.06586 | 1.447006 |
| 9 | 4.495569 | 0.295318 | -3.14553 |
| 9 | 5.698603 | -1.47385 | 0.818164 |
| 1 | -1.7667 | -2.24049 | 0.663693 |
| 14 | -3.23358 | -1.90482 | 1.14975 |
| 1 | -3.63946 | -2.17758 | 2.581101 |
| 9 | -2.54116 | -0.36785 | 1.629656 |
| 6 | -4.46537 | -1.08701 | -0.06213 |

| | | | |
|---|---|---|---|
| 6 | -4.41503 | 0.279782 | -0.37523 |
| 6 | -5.4385 | -1.86301 | -0.71242 |
| 6 | -5.28176 | 0.844804 | -1.31176 |
| 6 | -6.32017 | -1.30605 | -1.63851 |
| 6 | -6.23787 | 0.053033 | -1.94752 |
| 1 | -3.67244 | 0.904236 | 0.121809 |
| 1 | -5.48932 | -2.9342 | -0.49226 |
| 1 | -5.21142 | 1.911646 | -1.54271 |
| 1 | -7.07159 | -1.93355 | -2.12584 |
| 1 | -6.92186 | 0.49347 | -2.6782 |
| 1 | -3.58595 | -3.35251 | 0.671322 |

*10.11.2.8 TS3*

**Energy (PBE0-D3(BJ)CPCM($C_6H_6$)/def2-SVP) =** -2838.58793

| | | | |
|---|---|---|---|
| 6 | -1.19307 | 1.871752 | -1.74217 |
| 7 | -0.57489 | 1.066635 | -0.81888 |
| 6 | -0.64041 | -0.19496 | -1.25362 |
| 6 | -1.63275 | 1.080204 | -2.75901 |
| 1 | -2.17234 | 1.344648 | -3.65937 |
| 1 | -1.28368 | 2.943331 | -1.5952 |
| 7 | -1.28772 | -0.21283 | -2.44379 |
| 6 | -1.57261 | -1.40566 | -3.29954 |
| 51 | 0.100222 | -1.87338 | -0.11607 |
| 7 | 0.211153 | 0.684587 | 1.624767 |
| 6 | 0.220413 | -0.65368 | 1.679154 |
| 6 | 0.291284 | 0.11382 | 3.752362 |
| 7 | 0.26017 | -1.02536 | 2.980689 |
| 1 | 0.324017 | 0.085349 | 4.835759 |
| 1 | 0.255886 | 2.238459 | 3.110556 |
| 6 | 0.258538 | 1.173329 | 2.905265 |
| 6 | 0.188476 | -2.363 | 3.638325 |
| 5 | 0.226221 | 1.690906 | 0.406856 |
| 6 | 0.052332 | -3.49009 | 2.627969 |
| 1 | -0.87159 | -3.4086 | 2.036308 |
| 1 | 0.929478 | -3.56088 | 1.970416 |
| 1 | -0.00687 | -4.43779 | 3.180439 |
| 6 | 1.473433 | -2.55853 | 4.443903 |
| 1 | 2.351413 | -2.52972 | 3.783931 |
| 1 | 1.59283 | -1.78592 | 5.216535 |
| 1 | 1.444918 | -3.53449 | 4.949101 |
| 6 | -1.0452 | -2.35946 | 4.545407 |
| 1 | -0.93486 | -1.66268 | 5.387848 |
| 1 | -1.94062 | -2.06577 | 3.979558 |
| 1 | -1.19968 | -3.36465 | 4.961762 |
| 6 | -2.52547 | -2.3444 | -2.56694 |
| 1 | -2.13793 | -2.67047 | -1.59132 |
| 1 | -3.49222 | -1.85739 | -2.38944 |
| 1 | -2.69066 | -3.24231 | -3.17925 |
| 6 | -0.25677 | -2.09789 | -3.65139 |
| 1 | 0.4429 | -1.40325 | -4.13324 |
| 1 | 0.241319 | -2.52873 | -2.76936 |
| 1 | -0.4617 | -2.93172 | -4.33725 |
| 6 | -2.24275 | -0.94252 | -4.58978 |
| 1 | -3.20818 | -0.45489 | -4.392 |
| 1 | -1.59953 | -0.26534 | -5.1704 |

| | | | |
|---|---|---|---|
| 1 | -2.44171 | -1.82729 | -5.20931 |
| 6 | 1.754932 | 1.972618 | -0.03679 |
| 6 | 2.064209 | 2.534979 | -1.28308 |
| 6 | 2.839198 | 1.709587 | 0.813293 |
| 6 | 3.377192 | 2.792261 | -1.67255 |
| 6 | 4.153958 | 1.964683 | 0.432823 |
| 6 | 4.451556 | 2.500118 | -0.8258 |
| 1 | 1.261837 | 2.760819 | -1.99103 |
| 1 | 2.662073 | 1.271618 | 1.799969 |
| 1 | 3.575586 | 3.21039 | -2.66365 |
| 1 | 4.971543 | 1.724776 | 1.119434 |
| 6 | -0.59299 | 3.015494 | 0.850959 |
| 6 | -1.89212 | 2.89131 | 1.380494 |
| 6 | -0.09299 | 4.311302 | 0.692993 |
| 6 | -2.65079 | 4.00686 | 1.715712 |
| 6 | -0.85324 | 5.435308 | 1.029664 |
| 6 | -2.14595 | 5.305074 | 1.542299 |
| 1 | -2.30869 | 1.892659 | 1.55081 |
| 1 | 0.916083 | 4.451592 | 0.294911 |
| 1 | -3.65744 | 3.871299 | 2.12339 |
| 1 | -0.43009 | 6.435014 | 0.89174 |
| 6 | 5.872265 | 2.685772 | -1.26734 |
| 1 | 5.945333 | 3.375378 | -2.12032 |
| 1 | 6.502962 | 3.072829 | -0.45303 |
| 1 | 6.301898 | 1.719599 | -1.58158 |
| 6 | -2.97185 | 6.504215 | 1.908258 |
| 1 | -3.2155 | 6.507404 | 2.982976 |
| 1 | -2.44593 | 7.441889 | 1.679531 |
| 1 | -3.92987 | 6.510614 | 1.364271 |
| 6 | 2.261052 | -1.25616 | -0.59754 |
| 6 | 2.733606 | -0.63127 | -1.74175 |
| 6 | 3.239463 | -1.69931 | 0.278275 |
| 6 | 4.106727 | -0.48109 | -1.93905 |
| 6 | 4.5891 | -1.50034 | 0.005613 |
| 7 | 5.002159 | -0.91018 | -1.08681 |
| 9 | 1.90889 | -0.15503 | -2.67539 |
| 9 | 2.895016 | -2.33229 | 1.412548 |
| 9 | 4.543131 | 0.114295 | -3.03737 |
| 9 | 5.503547 | -1.91684 | 0.867477 |
| 1 | -1.89912 | -1.88344 | 0.548227 |
| 14 | -3.36104 | -1.75047 | 1.343385 |
| 1 | -4.17858 | -1.86228 | 2.624678 |
| 9 | -2.59095 | -0.35893 | 1.979167 |
| 6 | -4.43822 | -1.05437 | -0.0397 |

| | | | |
|---|---|---|---|
| 6 | -4.19658 | 0.229126 | -0.54814 |
| 6 | -5.46072 | -1.81394 | -0.628 |
| 6 | -4.93269 | 0.730997 | -1.62223 |
| 6 | -6.20609 | -1.31697 | -1.6968 |
| 6 | -5.93734 | -0.04335 | -2.20264 |
| 1 | -3.41007 | 0.841689 | -0.10126 |
| 1 | -5.66761 | -2.82135 | -0.25373 |
| 1 | -4.72076 | 1.732913 | -2.00504 |
| 1 | -6.99686 | -1.92634 | -2.14257 |
| 1 | -6.51699 | 0.346731 | -3.04349 |
| 1 | -3.38458 | -3.27103 | 1.084071 |

*10.11.2.9 INT4*

**Energy (PBE0-D3(BJ)CPCM($C_6H_6$)/def2-SVP) =** -2216.676608

| | | | |
|---|---|---|---|
| 6 | 2.103108 | 0.848647 | 2.194218 |
| 7 | 1.348613 | 0.18349 | 1.257102 |
| 6 | 0.392294 | -0.50128 | 1.893743 |
| 6 | 1.587937 | 0.570723 | 3.420879 |
| 1 | 1.904565 | 0.908699 | 4.399679 |
| 1 | 2.95536 | 1.459361 | 1.914082 |
| 7 | 0.52151 | -0.27923 | 3.223813 |
| 6 | -0.33771 | -0.83527 | 4.308428 |
| 51 | -1.0072 | -1.82472 | 0.883924 |
| 7 | 1.118209 | -0.79938 | -1.14429 |
| 6 | 0.1242 | -1.68706 | -0.98354 |
| 6 | 1.06194 | -2.04847 | -2.96324 |
| 7 | 0.082934 | -2.47142 | -2.08845 |
| 1 | 1.22499 | -2.51313 | -3.92959 |
| 1 | 2.533601 | -0.40514 | -2.71865 |
| 6 | 1.704407 | -1.01203 | -2.36995 |
| 6 | -0.77993 | -3.63493 | -2.43937 |
| 5 | 1.579662 | 0.45444 | -0.28943 |
| 6 | -1.77444 | -3.95364 | -1.33558 |
| 1 | -1.26561 | -4.23553 | -0.40111 |
| 1 | -2.47332 | -3.12404 | -1.16634 |
| 1 | -2.36996 | -4.82215 | -1.64962 |
| 6 | -1.54214 | -3.28386 | -3.71837 |
| 1 | -2.15207 | -2.38354 | -3.56258 |
| 1 | -0.86482 | -3.10709 | -4.56575 |
| 1 | -2.2064 | -4.11657 | -3.9902 |
| 6 | 0.13285 | -4.84517 | -2.6478 |
| 1 | 0.845039 | -4.69086 | -3.4705 |
| 1 | 0.702407 | -5.06045 | -1.73162 |
| 1 | -0.47646 | -5.72644 | -2.89394 |
| 6 | -0.16674 | -2.35261 | 4.346483 |
| 1 | -0.45764 | -2.83498 | 3.402613 |
| 1 | 0.882399 | -2.61782 | 4.543376 |
| 1 | -0.79019 | -2.77424 | 5.147801 |
| 6 | -1.78919 | -0.42721 | 4.059035 |
| 1 | -1.8791 | 0.664162 | 3.984143 |
| 1 | -2.19422 | -0.86194 | 3.133352 |
| 1 | -2.41561 | -0.78504 | 4.888337 |
| 6 | 0.103912 | -0.25504 | 5.649373 |
| 1 | 1.138991 | -0.53001 | 5.898431 |
| 1 | 0.002618 | 0.839556 | 5.675342 |

| | | | |
|---|---|---|---|
| 1 | -0.54487 | -0.66808 | 6.433597 |
| 6 | 0.743908 | 1.759662 | -0.7517 |
| 6 | 0.733723 | 2.927572 | 0.02388 |
| 6 | 0.016047 | 1.804962 | -1.94897 |
| 6 | 0.008489 | 4.057017 | -0.34822 |
| 6 | -0.70867 | 2.930673 | -2.33128 |
| 6 | -0.74237 | 4.075786 | -1.52813 |
| 1 | 1.282182 | 2.95795 | 0.968999 |
| 1 | -0.02032 | 0.924858 | -2.59725 |
| 1 | 0.003723 | 4.935898 | 0.303115 |
| 1 | -1.28686 | 2.910899 | -3.2601 |
| 6 | 3.182843 | 0.603673 | -0.498 |
| 6 | 4.046361 | -0.4363 | -0.10894 |
| 6 | 3.775485 | 1.732548 | -1.07183 |
| 6 | 5.422956 | -0.35111 | -0.28154 |
| 6 | 5.159153 | 1.824936 | -1.24836 |
| 6 | 6.008367 | 0.787616 | -0.85657 |
| 1 | 3.62442 | -1.34096 | 0.342439 |
| 1 | 3.1398 | 2.563329 | -1.39112 |
| 1 | 6.062518 | -1.18173 | 0.033501 |
| 1 | 5.587 | 2.724227 | -1.70185 |
| 6 | -1.61201 | 5.243364 | -1.88592 |
| 1 | -1.29642 | 6.159687 | -1.36658 |
| 1 | -1.60675 | 5.43816 | -2.9689 |
| 1 | -2.65628 | 5.037309 | -1.596 |
| 6 | 7.496438 | 0.878705 | -1.03442 |
| 1 | 8.015663 | 0.869305 | -0.06225 |
| 1 | 7.883981 | 0.024426 | -1.61195 |
| 1 | 7.783943 | 1.800687 | -1.55913 |
| 1 | 0.260499 | -3.12126 | 1.237149 |
| 6 | -2.04838 | 0.325202 | 0.080245 |
| 6 | -2.1512 | 1.541854 | 0.729257 |
| 6 | -2.83751 | 0.228411 | -1.04937 |
| 6 | -2.9702 | 2.556057 | 0.236148 |
| 6 | -3.63654 | 1.280543 | -1.4836 |
| 7 | -3.70081 | 2.422308 | -0.84388 |
| 9 | -2.84438 | -0.91077 | -1.78596 |
| 9 | -1.45096 | 1.803262 | 1.849181 |
| 9 | -3.03116 | 3.724844 | 0.867839 |
| 9 | -4.36743 | 1.155607 | -2.5884 |

*10.11.2.10 INT5*

**Energy (PBE0-D3(BJ)CPCM($C_6H_6$)/def2-SVP) =** -2216.672843

| | | | |
|---|---|---|---|
| 6 | -1.64469 | 0.134267 | -2.61729 |
| 7 | -1.06749 | -0.13757 | -1.39585 |
| 6 | -0.30137 | -1.23717 | -1.48164 |
| 6 | -1.2434 | -0.83634 | -3.47919 |
| 1 | -1.49241 | -0.98068 | -4.52404 |
| 1 | -2.31784 | 0.972375 | -2.77173 |
| 7 | -0.42666 | -1.68766 | -2.76 |
| 6 | 0.111275 | -2.97682 | -3.26151 |
| 51 | 1.400225 | -1.81349 | 0.294029 |
| 7 | -1.24929 | -0.30801 | 1.177551 |
| 6 | -0.3382 | -1.23838 | 1.527974 |
| 6 | -1.78111 | -1.01317 | 3.205358 |
| 7 | -0.67296 | -1.7001 | 2.766621 |
| 1 | -2.24735 | -1.19139 | 4.165942 |
| 1 | -2.96274 | 0.542691 | 2.163184 |
| 6 | -2.13363 | -0.15436 | 2.216536 |
| 6 | 0.004694 | -2.72089 | 3.622203 |
| 5 | -1.41635 | 0.657303 | -0.09916 |
| 6 | 0.547853 | -3.8636 | 2.768026 |
| 1 | -0.22024 | -4.24841 | 2.081063 |
| 1 | 1.440741 | -3.57167 | 2.201617 |
| 1 | 0.855406 | -4.68532 | 3.42958 |
| 6 | 1.111645 | -2.02488 | 4.413057 |
| 1 | 1.842394 | -1.56372 | 3.733692 |
| 1 | 0.686657 | -1.24147 | 5.058085 |
| 1 | 1.631969 | -2.75461 | 5.050683 |
| 6 | -1.02202 | -3.31798 | 4.587985 |
| 1 | -1.37754 | -2.59208 | 5.331828 |
| 1 | -1.88709 | -3.73661 | 4.052412 |
| 1 | -0.53831 | -4.1321 | 5.144428 |
| 6 | -0.30166 | -4.07905 | -2.28535 |
| 1 | 0.14315 | -3.91556 | -1.2936 |
| 1 | -1.39598 | -4.11035 | -2.17657 |
| 1 | 0.041446 | -5.05606 | -2.65675 |
| 6 | 1.63125 | -2.90297 | -3.38198 |
| 1 | 1.931703 | -2.08454 | -4.05053 |
| 1 | 2.101317 | -2.7361 | -2.40396 |
| 1 | 2.016672 | -3.84948 | -3.7889 |
| 6 | -0.48267 | -3.28151 | -4.63462 |
| 1 | -1.58106 | -3.33264 | -4.60342 |
| 1 | -0.18055 | -2.53872 | -5.38745 |

| | | | |
|---|---|---|---|
| 1 | -0.10949 | -4.25928 | -4.96929 |
| 6 | -0.5202 | 1.985395 | 0.084589 |
| 6 | 0.034621 | 2.650366 | -1.0182 |
| 6 | -0.37019 | 2.60903 | 1.330637 |
| 6 | 0.712043 | 3.859468 | -0.88387 |
| 6 | 0.310052 | 3.8172 | 1.474164 |
| 6 | 0.871737 | 4.464109 | 0.368699 |
| 1 | -0.05137 | 2.205544 | -2.01413 |
| 1 | -0.78312 | 2.135877 | 2.226379 |
| 1 | 1.147012 | 4.337132 | -1.7671 |
| 1 | 0.421976 | 4.261124 | 2.467844 |
| 6 | -3.00319 | 1.022769 | -0.20263 |
| 6 | -3.94848 | 0.004831 | -0.426 |
| 6 | -3.50025 | 2.324883 | -0.09422 |
| 6 | -5.3088 | 0.2711 | -0.52529 |
| 6 | -4.86716 | 2.601485 | -0.19634 |
| 6 | -5.79687 | 1.582393 | -0.41241 |
| 1 | -3.6038 | -1.03038 | -0.52381 |
| 1 | -2.80234 | 3.149565 | 0.07523 |
| 1 | -6.01244 | -0.54991 | -0.69652 |
| 1 | -5.21698 | 3.634545 | -0.10656 |
| 6 | 1.678692 | 5.718858 | 0.521613 |
| 1 | 1.538137 | 6.39899 | -0.33166 |
| 1 | 1.417779 | 6.259709 | 1.442831 |
| 1 | 2.753281 | 5.475802 | 0.573463 |
| 6 | -7.26586 | 1.868652 | -0.53349 |
| 1 | -7.6341 | 1.641903 | -1.54747 |
| 1 | -7.85408 | 1.252903 | 0.165279 |
| 1 | -7.48895 | 2.924658 | -0.32547 |
| 1 | 2.360439 | -1.64303 | 1.811756 |
| 6 | 2.209693 | 0.189538 | -0.09 |
| 6 | 2.701384 | 0.548727 | -1.33765 |
| 6 | 2.453269 | 1.090223 | 0.942782 |
| 6 | 3.409864 | 1.738518 | -1.49226 |
| 6 | 3.183331 | 2.250244 | 0.684698 |
| 7 | 3.650427 | 2.552698 | -0.49899 |
| 9 | 2.012903 | 0.88212 | 2.176535 |
| 9 | 2.523054 | -0.23178 | -2.4013 |
| 9 | 3.871992 | 2.068722 | -2.68887 |
| 9 | 3.437578 | 3.088466 | 1.677244 |

*10.11.2.11 TS4*

**Energy (PBE0-D3(BJ)CPCM($C_6H_6$)/def2-SVP) =** -2216.639429

| | | | |
|---|---|---|---|
| 6 | 3.280815 | 0.666205 | -1.64336 |
| 7 | 2.166245 | 0.544116 | -0.84739 |
| 6 | 1.486098 | 1.705813 | -0.82958 |
| 6 | 3.304019 | 1.941956 | -2.11992 |
| 1 | 4.028038 | 2.434145 | -2.7599 |
| 1 | 3.976229 | -0.15287 | -1.80093 |
| 7 | 2.18816 | 2.570692 | -1.60112 |
| 6 | 1.755932 | 3.956483 | -1.89051 |
| 51 | -0.91231 | 1.446385 | -0.23156 |
| 7 | 1.082928 | -0.34438 | 1.255864 |
| 6 | 0.165193 | 0.611709 | 1.491299 |
| 6 | 0.948179 | -0.09568 | 3.442348 |
| 7 | 0.102754 | 0.80808 | 2.834447 |
| 1 | 1.068691 | -0.16407 | 4.515938 |
| 1 | 2.295738 | -1.59612 | 2.516794 |
| 6 | 1.554229 | -0.80661 | 2.45497 |
| 6 | -0.66609 | 1.839953 | 3.595643 |
| 5 | 1.652213 | -0.78432 | -0.17389 |
| 6 | -0.47962 | 3.202038 | 2.9287 |
| 1 | 0.588832 | 3.443372 | 2.826614 |
| 1 | -0.94779 | 3.241239 | 1.937102 |
| 1 | -0.95443 | 3.975578 | 3.548893 |
| 6 | -2.13877 | 1.44345 | 3.677369 |
| 1 | -2.613 | 1.455014 | 2.68683 |
| 1 | -2.24742 | 0.436158 | 4.102438 |
| 1 | -2.67398 | 2.157829 | 4.319726 |
| 6 | -0.1062 | 1.929921 | 5.01604 |
| 1 | -0.28872 | 1.014295 | 5.596353 |
| 1 | 0.970856 | 2.152653 | 5.019988 |
| 1 | -0.62209 | 2.749194 | 5.534629 |
| 6 | 1.294965 | 4.608936 | -0.58859 |
| 1 | 0.430176 | 4.08413 | -0.15969 |
| 1 | 2.102989 | 4.603329 | 0.157737 |
| 1 | 0.999286 | 5.650692 | -0.77943 |
| 6 | 0.614208 | 3.904312 | -2.90653 |
| 1 | 0.940041 | 3.403674 | -3.83045 |
| 1 | -0.24841 | 3.353222 | -2.50403 |
| 1 | 0.28094 | 4.921168 | -3.16102 |
| 6 | 2.924971 | 4.751546 | -2.4646 |
| 1 | 3.787858 | 4.747935 | -1.78214 |
| 1 | 3.244504 | 4.367305 | -3.44409 |

| | | | |
|---|---|---|---|
| 1 | 2.610034 | 5.794287 | -2.60941 |
| 6 | 0.456684 | -1.42857 | -1.05102 |
| 6 | 0.207666 | -1.0673 | -2.38366 |
| 6 | -0.36647 | -2.42851 | -0.51036 |
| 6 | -0.83356 | -1.63241 | -3.11744 |
| 6 | -1.39493 | -3.01414 | -1.24543 |
| 6 | -1.66521 | -2.61 | -2.55757 |
| 1 | 0.831225 | -0.30319 | -2.85879 |
| 1 | -0.21379 | -2.74668 | 0.52542 |
| 1 | -1.0106 | -1.3045 | -4.14632 |
| 1 | -2.01947 | -3.78481 | -0.78342 |
| 6 | 2.912056 | -1.7814 | 0.040814 |
| 6 | 4.091063 | -1.33318 | 0.667002 |
| 6 | 2.908191 | -3.11177 | -0.3922 |
| 6 | 5.189964 | -2.16395 | 0.857979 |
| 6 | 4.007669 | -3.95301 | -0.20444 |
| 6 | 5.168583 | -3.49791 | 0.424595 |
| 1 | 4.151511 | -0.29707 | 1.016726 |
| 1 | 2.021649 | -3.5059 | -0.89595 |
| 1 | 6.085911 | -1.77371 | 1.351149 |
| 1 | 3.961579 | -4.98731 | -0.55866 |
| 6 | -2.83178 | -3.16858 | -3.31912 |
| 1 | -2.62681 | -3.21414 | -4.39892 |
| 1 | -3.09422 | -4.1786 | -2.97256 |
| 1 | -3.72542 | -2.5373 | -3.18239 |
| 6 | 6.357873 | -4.39158 | 0.626908 |
| 1 | 6.635417 | -4.4541 | 1.691246 |
| 1 | 6.160587 | -5.41168 | 0.268535 |
| 1 | 7.240248 | -4.01008 | 0.087987 |
| 1 | -2.45635 | 1.484342 | 0.841104 |
| 6 | -2.64232 | -0.03294 | 0.19964 |
| 6 | -3.62036 | -0.05615 | -0.8018 |
| 6 | -2.73267 | -1.08499 | 1.114716 |
| 6 | -4.56016 | -1.07143 | -0.85375 |
| 6 | -3.72743 | -2.04616 | 0.982931 |
| 7 | -4.61901 | -2.04558 | 0.023748 |
| 9 | -1.84942 | -1.22802 | 2.109259 |
| 9 | -3.61487 | 0.888665 | -1.74842 |
| 9 | -5.43715 | -1.09482 | -1.85211 |
| 9 | -3.76825 | -3.04786 | 1.853136 |

*10.11.2.12 TS1’*

**Energy (PBE0-D3(BJ)CPCM($C_6H_6$)/def2-SVP) =** -2838.48843

| | | | |
|---|---|---|---|
| 6 | -1.41226 | 0.844524 | -2.44464 |
| 7 | -0.85437 | 0.418196 | -1.2659 |
| 6 | -0.32708 | -0.80886 | -1.44343 |
| 6 | -1.21206 | -0.12736 | -3.3676 |
| 1 | -1.50669 | -0.17135 | -4.41023 |
| 1 | -1.90826 | 1.805533 | -2.52706 |
| 7 | -0.52586 | -1.15243 | -2.74826 |
| 6 | -0.17641 | -2.36963 | -3.53816 |
| 51 | 0.882297 | -1.63711 | 0.224211 |
| 7 | -0.92734 | 0.516375 | 1.297647 |
| 6 | -0.3268 | -0.62893 | 1.668497 |
| 6 | -1.50466 | 0.105725 | 3.38251 |
| 7 | -0.66672 | -0.90067 | 2.959117 |
| 1 | -1.92685 | 0.134818 | 4.378091 |
| 1 | -2.24476 | 1.890737 | 2.287077 |
| 6 | -1.66331 | 0.977701 | 2.352767 |
| 6 | -0.19708 | -1.97321 | 3.907569 |
| 5 | -0.72597 | 1.380158 | -0.02237 |
| 6 | 1.187949 | -1.57187 | 4.412434 |
| 1 | 1.91245 | -1.56909 | 3.58997 |
| 1 | 1.154043 | -0.57714 | 4.8821 |
| 1 | 1.520564 | -2.29829 | 5.168515 |
| 6 | -1.17159 | -2.04407 | 5.084956 |
| 1 | -1.14199 | -1.14838 | 5.721229 |
| 1 | -2.20479 | -2.22278 | 4.751622 |
| 1 | -0.87205 | -2.89181 | 5.715159 |
| 6 | -0.18897 | -3.34025 | 3.234978 |
| 1 | -1.17005 | -3.58018 | 2.805827 |
| 1 | 0.590344 | -3.41064 | 2.470489 |
| 1 | 0.049402 | -4.09247 | 4.000284 |
| 6 | 0.589517 | -3.37827 | -2.70178 |
| 1 | 1.543435 | -2.97198 | -2.34124 |
| 1 | -0.02461 | -3.73769 | -1.86553 |
| 1 | 0.825728 | -4.24474 | -3.33428 |
| 6 | 0.685103 | -1.93176 | -4.72553 |
| 1 | 0.141356 | -1.26335 | -5.40763 |
| 1 | 1.592089 | -1.41718 | -4.38258 |
| 1 | 0.983618 | -2.81921 | -5.30142 |
| 6 | -1.47643 | -3.01573 | -4.02274 |
| 1 | -2.09427 | -3.32813 | -3.16913 |
| 1 | -2.06486 | -2.33697 | -4.65609 |

| | | | |
|---|---|---|---|
| 1 | -1.23756 | -3.90686 | -4.6205 |
| 6 | 0.73244 | 2.067603 | 0.001047 |
| 6 | 1.454187 | 2.307392 | -1.17763 |
| 6 | 1.268593 | 2.588748 | 1.185615 |
| 6 | 2.657366 | 3.005622 | -1.17132 |
| 6 | 2.473206 | 3.289371 | 1.200955 |
| 6 | 3.195703 | 3.506105 | 0.022345 |
| 1 | 1.075031 | 1.925615 | -2.13095 |
| 1 | 0.739738 | 2.436954 | 2.131482 |
| 1 | 3.199889 | 3.16008 | -2.10911 |
| 1 | 2.871481 | 3.663767 | 2.148214 |
| 6 | -1.90176 | 2.490127 | -0.10094 |
| 6 | -3.2534 | 2.10605 | -0.16563 |
| 6 | -1.63624 | 3.864092 | -0.11912 |
| 6 | -4.27774 | 3.043007 | -0.23878 |
| 6 | -2.66209 | 4.809472 | -0.19374 |
| 6 | -4.00165 | 4.418208 | -0.25352 |
| 1 | -3.51386 | 1.044715 | -0.15946 |
| 1 | -0.6006 | 4.211096 | -0.07541 |
| 1 | -5.3144 | 2.697369 | -0.28701 |
| 1 | -2.41375 | 5.875048 | -0.20667 |
| 6 | 4.518279 | 4.212368 | 0.028035 |
| 1 | 4.547056 | 5.016231 | -0.72372 |
| 1 | 4.738029 | 4.651661 | 1.010889 |
| 1 | 5.332915 | 3.510878 | -0.21761 |
| 6 | -5.11348 | 5.424057 | -0.32876 |
| 1 | -4.7261 | 6.451471 | -0.37771 |
| 1 | -5.74461 | 5.256512 | -1.21602 |
| 1 | -5.77407 | 5.354198 | 0.550605 |
| 6 | 2.718515 | -0.43295 | -0.13435 |
| 6 | 3.352989 | -0.42394 | -1.36401 |
| 6 | 3.383245 | 0.188314 | 0.912399 |
| 6 | 4.592649 | 0.193715 | -1.50324 |
| 6 | 4.618378 | 0.787826 | 0.668544 |
| 7 | 5.20128 | 0.776946 | -0.50337 |
| 9 | 2.784772 | -0.9977 | -2.43099 |
| 9 | 2.864743 | 0.254469 | 2.129366 |
| 9 | 5.18798 | 0.204197 | -2.68433 |
| 9 | 5.23842 | 1.409781 | 1.656307 |
| 9 | 2.128045 | -2.36347 | 1.66133 |
| 14 | -2.19681 | -2.89661 | 0.065612 |
| 6 | -3.71758 | -1.76264 | -0.11577 |
| 1 | 0.529572 | -3.23637 | -0.07565 |
| 6 | -4.19274 | -1.34848 | -1.3727 |

| | | | |
|---|---|---|---|
| 6 | -4.40758 | -1.29203 | 1.015925 |
| 6 | -5.32139 | -0.53956 | -1.4977 |
| 1 | -3.67788 | -1.67851 | -2.27998 |
| 6 | -5.53705 | -0.4827 | 0.90026 |
| 1 | -4.06271 | -1.58122 | 2.014131 |
| 6 | -6.00644 | -0.11079 | -0.36017 |
| 1 | -5.67192 | -0.24404 | -2.49034 |
| 1 | -6.05831 | -0.14307 | 1.799477 |
| 1 | -6.89825 | 0.513808 | -0.45535 |
| 1 | -2.38306 | -3.88135 | -1.08161 |
| 1 | -2.60125 | -3.75992 | 1.250314 |

*10.11.2.13 INT1’*

**Energy (PBE0-D3(BJ)CPCM($C_6H_6$)/def2-SVP) =** -2838.533301

| | | | |
|---|---|---|---|
| 6 | -1.28817 | 1.102404 | -2.53453 |
| 7 | -0.75123 | 0.575497 | -1.38648 |
| 6 | -0.25051 | -0.64804 | -1.65002 |
| 6 | -1.12856 | 0.188623 | -3.51958 |
| 1 | -1.41524 | 0.238203 | -4.56367 |
| 1 | -1.7396 | 2.087469 | -2.56052 |
| 7 | -0.48661 | -0.90223 | -2.97292 |
| 6 | -0.13695 | -2.02268 | -3.90804 |
| 51 | 0.586995 | -1.87365 | 0.074336 |
| 7 | -0.87421 | 0.683864 | 1.223461 |
| 6 | -0.38979 | -0.50251 | 1.636729 |
| 6 | -1.43133 | 0.478189 | 3.342914 |
| 7 | -0.74404 | -0.65044 | 2.952293 |
| 1 | -1.81592 | 0.621919 | 4.343329 |
| 1 | -1.97982 | 2.27308 | 2.17028 |
| 6 | -1.51244 | 1.300244 | 2.270118 |
| 6 | -0.42259 | -1.72678 | 3.955689 |
| 5 | -0.62795 | 1.508345 | -0.10801 |
| 6 | 1.061217 | -1.6584 | 4.307434 |
| 1 | 1.675627 | -1.925 | 3.44241 |
| 1 | 1.328871 | -0.64938 | 4.652653 |
| 1 | 1.268182 | -2.36983 | 5.120624 |
| 6 | -1.23641 | -1.48727 | 5.230085 |
| 1 | -0.93837 | -0.57154 | 5.760092 |
| 1 | -2.31931 | -1.45958 | 5.036563 |
| 1 | -1.04285 | -2.32897 | 5.908445 |
| 6 | -0.81256 | -3.09631 | 3.418382 |
| 1 | -1.88819 | -3.14721 | 3.19726 |
| 1 | -0.22444 | -3.35934 | 2.535405 |
| 1 | -0.59631 | -3.8516 | 4.187616 |
| 6 | 0.589353 | -3.17646 | -3.24363 |
| 1 | 1.5706 | -2.87669 | -2.86457 |
| 1 | 0.001465 | -3.6233 | -2.43472 |
| 1 | 0.742895 | -3.95469 | -4.00431 |
| 6 | 0.779137 | -1.44158 | -4.99007 |
| 1 | 0.284558 | -0.6655 | -5.59023 |
| 1 | 1.678826 | -1.00907 | -4.53013 |
| 1 | 1.090287 | -2.2445 | -5.67327 |
| 6 | -1.4403 | -2.55411 | -4.5106 |
| 1 | -2.08006 | -2.98665 | -3.7275 |
| 1 | -2.01261 | -1.77919 | -5.03862 |

| | | | |
|---|---|---|---|
| 1 | -1.20742 | -3.3476 | -5.23441 |
| 6 | 0.829124 | 2.198235 | -0.05658 |
| 6 | 1.638575 | 2.340154 | -1.19101 |
| 6 | 1.305778 | 2.773533 | 1.129886 |
| 6 | 2.872461 | 2.984195 | -1.1376 |
| 6 | 2.537471 | 3.420368 | 1.192071 |
| 6 | 3.353953 | 3.525521 | 0.060249 |
| 1 | 1.312827 | 1.915741 | -2.14602 |
| 1 | 0.710722 | 2.698775 | 2.044499 |
| 1 | 3.488492 | 3.049787 | -2.0398 |
| 1 | 2.885508 | 3.834097 | 2.14297 |
| 6 | -1.81294 | 2.615399 | -0.21811 |
| 6 | -3.15874 | 2.208971 | -0.23752 |
| 6 | -1.56956 | 3.988265 | -0.31919 |
| 6 | -4.20118 | 3.120679 | -0.34774 |
| 6 | -2.61362 | 4.911623 | -0.43125 |
| 6 | -3.94757 | 4.497704 | -0.44605 |
| 1 | -3.39574 | 1.144934 | -0.16467 |
| 1 | -0.53793 | 4.350637 | -0.31267 |
| 1 | -5.23485 | 2.760234 | -0.35906 |
| 1 | -2.38391 | 5.978639 | -0.50962 |
| 6 | 4.724469 | 4.127896 | 0.137656 |
| 1 | 4.964611 | 4.709951 | -0.76468 |
| 1 | 4.830989 | 4.784105 | 1.013253 |
| 1 | 5.482521 | 3.33053 | 0.22277 |
| 6 | -5.07623 | 5.481869 | -0.55342 |
| 1 | -5.67707 | 5.50116 | 0.370624 |
| 1 | -4.70675 | 6.50137 | -0.7328 |
| 1 | -5.76145 | 5.21868 | -1.37464 |
| 6 | 2.431908 | -0.58679 | 0.059707 |
| 6 | 3.213774 | -0.57482 | -1.08828 |
| 6 | 2.999323 | 0.042586 | 1.157728 |
| 6 | 4.475808 | 0.010151 | -1.08859 |
| 6 | 4.267842 | 0.614488 | 1.060843 |
| 7 | 4.988484 | 0.582459 | -0.03048 |
| 9 | 2.770634 | -1.10469 | -2.23678 |
| 9 | 2.359657 | 0.14268 | 2.318021 |
| 9 | 5.195996 | 0.008819 | -2.20132 |
| 9 | 4.781704 | 1.227933 | 2.115927 |
| 9 | 1.478766 | -2.84463 | 1.603335 |
| 14 | -1.69419 | -3.20479 | -0.03586 |
| 6 | -3.16957 | -2.03757 | 0.049535 |
| 1 | 1.488759 | -2.95746 | -0.89306 |
| 6 | -3.80685 | -1.64121 | -1.13864 |

| | | | |
|---|---|---|---|
| 6 | -3.7015 | -1.57619 | 1.264706 |
| 6 | -4.95295 | -0.84814 | -1.11309 |
| 1 | -3.41251 | -1.97033 | -2.10361 |
| 6 | -4.84563 | -0.78126 | 1.294987 |
| 1 | -3.22424 | -1.84721 | 2.208804 |
| 6 | -5.48249 | -0.42657 | 0.106004 |
| 1 | -5.4351 | -0.55908 | -2.04997 |
| 1 | -5.2433 | -0.4387 | 2.253204 |
| 1 | -6.38464 | 0.189524 | 0.129401 |
| 1 | -1.75557 | -3.84697 | -1.38711 |
| 1 | -1.8291 | -4.29962 | 0.969511 |

*10.11.2.14 TS2’*

**Energy (PBE0-D3(BJ)CPCM($C_6H_6$)/def2-SVP) =** -2838.507393

| | | | |
|---|---|---|---|
| 6 | -1.5604 | 1.48997 | -2.04791 |
| 7 | -0.85428 | 0.891995 | -1.03264 |
| 6 | -0.93372 | -0.44401 | -1.1766 |
| 6 | -2.09826 | 0.506208 | -2.81076 |
| 1 | -2.71893 | 0.57189 | -3.69674 |
| 1 | -1.62294 | 2.568142 | -2.14621 |
| 7 | -1.724 | -0.69512 | -2.25198 |
| 6 | -2.17253 | -1.97533 | -2.87744 |
| 51 | -0.21988 | -1.69886 | 0.522765 |
| 7 | 0.11776 | 1.205237 | 1.408763 |
| 6 | 0.358468 | -0.03441 | 1.884139 |
| 6 | 0.695484 | 1.402429 | 3.531426 |
| 7 | 0.735722 | 0.06977 | 3.182658 |
| 1 | 0.944265 | 1.757805 | 4.523361 |
| 1 | 0.180576 | 3.168719 | 2.297756 |
| 6 | 0.315134 | 2.101281 | 2.433809 |
| 6 | 1.213428 | -0.96236 | 4.169214 |
| 5 | 0.045614 | 1.788183 | -0.08509 |
| 6 | 1.94118 | -2.10572 | 3.469591 |
| 1 | 1.269131 | -2.67049 | 2.816226 |
| 1 | 2.819349 | -1.74078 | 2.926032 |
| 1 | 2.283533 | -2.80703 | 4.243831 |
| 6 | 2.221226 | -0.29203 | 5.109248 |
| 1 | 3.023663 | 0.208112 | 4.54715 |
| 1 | 1.757407 | 0.428811 | 5.797082 |
| 1 | 2.677721 | -1.07474 | 5.730137 |
| 6 | 0.012169 | -1.46223 | 4.966929 |
| 1 | -0.52424 | -0.62102 | 5.431739 |
| 1 | -0.66465 | -2.02163 | 4.311582 |
| 1 | 0.359299 | -2.13201 | 5.76744 |
| 6 | -1.94144 | -3.15525 | -1.94991 |
| 1 | -0.87639 | -3.34912 | -1.78853 |
| 1 | -2.45578 | -3.01365 | -0.98834 |
| 1 | -2.3673 | -4.05382 | -2.41642 |
| 6 | -1.38998 | -2.1581 | -4.17825 |
| 1 | -1.57616 | -1.32771 | -4.87516 |
| 1 | -0.31177 | -2.21597 | -3.98011 |
| 1 | -1.70581 | -3.08944 | -4.67022 |
| 6 | -3.67327 | -1.86799 | -3.15533 |
| 1 | -4.22449 | -1.62366 | -2.2372 |
| 1 | -3.90984 | -1.11666 | -3.92104 |

| | | | |
|---|---|---|---|
| 1 | -4.03434 | -2.83608 | -3.52945 |
| 6 | 1.535976 | 1.856872 | -0.70915 |
| 6 | 1.826147 | 1.438613 | -2.01568 |
| 6 | 2.601612 | 2.402615 | 0.020515 |
| 6 | 3.109624 | 1.523104 | -2.54995 |
| 6 | 3.888654 | 2.487852 | -0.50415 |
| 6 | 4.172376 | 2.035683 | -1.79788 |
| 1 | 1.034699 | 1.015088 | -2.64092 |
| 1 | 2.433983 | 2.754183 | 1.042443 |
| 1 | 3.296586 | 1.165381 | -3.56699 |
| 1 | 4.697095 | 2.896623 | 0.108818 |
| 6 | -0.66855 | 3.245286 | -0.00212 |
| 6 | -1.9203 | 3.412716 | 0.617854 |
| 6 | -0.10892 | 4.383196 | -0.59328 |
| 6 | -2.57415 | 4.639448 | 0.642727 |
| 6 | -0.76023 | 5.619425 | -0.57376 |
| 6 | -2.00408 | 5.772543 | 0.042949 |
| 1 | -2.40047 | 2.559012 | 1.105278 |
| 1 | 0.862429 | 4.302691 | -1.08861 |
| 1 | -3.5468 | 4.724138 | 1.137234 |
| 1 | -0.28905 | 6.484372 | -1.05026 |
| 6 | 5.570686 | 2.035546 | -2.33861 |
| 1 | 5.591077 | 2.276113 | -3.41197 |
| 1 | 6.211135 | 2.755144 | -1.80883 |
| 1 | 6.023176 | 1.036546 | -2.21942 |
| 6 | -2.70738 | 7.098084 | 0.080223 |
| 1 | -2.76954 | 7.486089 | 1.110056 |
| 1 | -2.18496 | 7.848962 | -0.52897 |
| 1 | -3.7402 | 7.014491 | -0.29283 |
| 6 | 1.951377 | -1.39692 | -0.34171 |
| 6 | 2.17514 | -1.98309 | -1.58035 |
| 6 | 3.08193 | -0.81301 | 0.214813 |
| 6 | 3.428976 | -1.96975 | -2.18235 |
| 6 | 4.309129 | -0.85922 | -0.44805 |
| 7 | 4.47467 | -1.4251 | -1.61551 |
| 9 | 1.171719 | -2.57611 | -2.25 |
| 9 | 3.0599 | -0.1818 | 1.391252 |
| 9 | 3.589867 | -2.52244 | -3.37596 |
| 9 | 5.371696 | -0.30914 | 0.120332 |
| 9 | -0.6118 | -2.94611 | 2.415007 |
| 14 | -2.46359 | -2.13013 | 1.721001 |
| 6 | -3.88968 | -1.31287 | 0.746433 |
| 1 | 0.145348 | -3.29019 | 0.015578 |
| 6 | -5.03293 | -2.03005 | 0.362378 |

| | | | |
|---|---|---|---|
| 6 | -3.8292 | 0.045547 | 0.398686 |
| 6 | -6.07811 | -1.41253 | -0.32816 |
| 1 | -5.11229 | -3.09327 | 0.612167 |
| 6 | -4.859 | 0.669818 | -0.30159 |
| 1 | -2.95086 | 0.631363 | 0.682095 |
| 6 | -5.99179 | -0.06142 | -0.66458 |
| 1 | -6.96287 | -1.98972 | -0.60984 |
| 1 | -4.77704 | 1.728637 | -0.56161 |
| 1 | -6.80605 | 0.421256 | -1.21094 |
| 1 | -2.91072 | -3.55451 | 1.764783 |
| 1 | -2.46141 | -1.38619 | 3.015261 |

*10.11.2.15 TS1”*

**Energy (PBE0-D3(BJ)CPCM($C_6H_6$)/def2-SVP) =** -2838.532563

| | | | |
|---|---|---|---|
| 6 | 2.743258 | -0.09026 | 2.606753 |
| 7 | 2.152172 | -0.32818 | 1.392458 |
| 6 | 1.095563 | -1.13295 | 1.57652 |
| 6 | 2.033133 | -0.76512 | 3.550461 |
| 1 | 2.182948 | -0.83324 | 4.622787 |
| 1 | 3.620655 | 0.541389 | 2.70235 |
| 7 | 1.006929 | -1.41217 | 2.899578 |
| 6 | 0.023388 | -2.24237 | 3.65583 |
| 51 | -0.23332 | -1.75203 | -0.07446 |
| 7 | 2.407437 | -0.45692 | -1.17147 |
| 6 | 1.373686 | -1.24544 | -1.49568 |
| 6 | 2.696903 | -1.09212 | -3.26492 |
| 7 | 1.531441 | -1.64043 | -2.78364 |
| 1 | 3.056151 | -1.25731 | -4.27314 |
| 1 | 4.144316 | 0.242561 | -2.22895 |
| 6 | 3.237456 | -0.35309 | -2.25649 |
| 6 | 0.629053 | -2.53276 | -3.57397 |
| 5 | 2.564011 | 0.478642 | 0.096067 |
| 6 | -0.80391 | -2.01947 | -3.45817 |
| 1 | -1.23704 | -2.16329 | -2.45591 |
| 1 | -0.87015 | -0.95238 | -3.7091 |
| 1 | -1.44994 | -2.5856 | -4.14327 |
| 6 | 1.039916 | -2.48253 | -5.04333 |
| 1 | 0.987189 | -1.4616 | -5.44922 |
| 1 | 2.048013 | -2.88864 | -5.20871 |
| 1 | 0.340861 | -3.10762 | -5.61485 |
| 6 | 0.769182 | -3.96149 | -3.05207 |
| 1 | 1.79741 | -4.3238 | -3.20198 |
| 1 | 0.539233 | -4.03526 | -1.98042 |
| 1 | 0.086801 | -4.62176 | -3.60648 |
| 6 | 0.802659 | -3.33797 | 4.384197 |
| 1 | 1.345657 | -3.96643 | 3.663186 |
| 1 | 1.524622 | -2.92708 | 5.103821 |
| 1 | 0.102242 | -3.97572 | 4.94147 |
| 6 | -0.99119 | -2.892 | 2.728679 |
| 1 | -1.63469 | -2.14701 | 2.237342 |
| 1 | -0.50627 | -3.54428 | 1.987746 |
| 1 | -1.65824 | -3.52204 | 3.332981 |
| 6 | -0.70389 | -1.31903 | 4.634381 |
| 1 | -0.01541 | -0.85477 | 5.354777 |
| 1 | -1.23281 | -0.52468 | 4.088211 |

| | | | |
|---|---|---|---|
| 1 | -1.44409 | -1.89928 | 5.203363 |
| 6 | 1.591555 | 1.75643 | -0.07054 |
| 6 | 0.968206 | 2.364391 | 1.029451 |
| 6 | 1.446657 | 2.401442 | -1.30673 |
| 6 | 0.269475 | 3.56221 | 0.90899 |
| 6 | 0.750545 | 3.602691 | -1.43419 |
| 6 | 0.161671 | 4.217068 | -0.32419 |
| 1 | 1.034872 | 1.899342 | 2.018083 |
| 1 | 1.898698 | 1.964986 | -2.20295 |
| 1 | -0.20648 | 4.001611 | 1.790818 |
| 1 | 0.659271 | 4.074527 | -2.41668 |
| 6 | 4.126231 | 0.875912 | 0.236349 |
| 6 | 5.109343 | -0.1212 | 0.375213 |
| 6 | 4.569761 | 2.202213 | 0.239188 |
| 6 | 6.458 | 0.190559 | 0.505775 |
| 6 | 5.923434 | 2.522181 | 0.371177 |
| 6 | 6.892444 | 1.524967 | 0.505587 |
| 1 | 4.808842 | -1.17467 | 0.381523 |
| 1 | 3.838638 | 3.008768 | 0.135867 |
| 1 | 7.193277 | -0.61328 | 0.611076 |
| 1 | 6.232591 | 3.571706 | 0.369736 |
| 6 | -0.58755 | 5.51086 | -0.43888 |
| 1 | -0.17685 | 6.269512 | 0.246064 |
| 1 | -0.54548 | 5.912853 | -1.46053 |
| 1 | -1.64667 | 5.366626 | -0.17288 |
| 6 | 8.349951 | 1.857298 | 0.642554 |
| 1 | 8.5152 | 2.943539 | 0.665507 |
| 1 | 8.772437 | 1.430131 | 1.565908 |
| 1 | 8.933241 | 1.44505 | -0.19659 |
| 6 | -1.98906 | 0.435341 | -0.39645 |
| 6 | -1.99938 | 1.416666 | -1.38597 |
| 6 | -2.43601 | 0.869582 | 0.852939 |
| 6 | -2.47666 | 2.690264 | -1.10357 |
| 6 | -2.89301 | 2.163129 | 1.041255 |
| 7 | -2.92113 | 3.051268 | 0.075706 |
| 9 | -2.44708 | 0.002241 | 1.880329 |
| 9 | -1.5922 | 1.131197 | -2.62698 |
| 9 | -2.4923 | 3.603309 | -2.06716 |
| 9 | -3.32151 | 2.542267 | 2.237607 |
| 9 | 0.607636 | -3.58047 | 0.091974 |
| 14 | -3.76356 | -2.10539 | -0.78672 |
| 6 | -4.97437 | -0.75779 | -0.25823 |
| 1 | -2.36591 | -0.69162 | -0.6966 |
| 6 | -5.28275 | 0.295416 | -1.14104 |

| | | | |
|---|---|---|---|
| 6 | -5.38481 | -0.62067 | 1.079219 |
| 6 | -5.97181 | 1.426606 | -0.71019 |
| 1 | -4.96472 | 0.234952 | -2.18726 |
| 6 | -6.07374 | 0.510691 | 1.515911 |
| 1 | -5.14901 | -1.41025 | 1.799247 |
| 6 | -6.36761 | 1.540544 | 0.623235 |
| 1 | -6.19044 | 2.232531 | -1.41563 |
| 1 | -6.37543 | 0.593115 | 2.563424 |
| 1 | -6.89546 | 2.433645 | 0.965972 |
| 1 | -4.14916 | -2.57631 | -2.16809 |
| 1 | -3.96324 | -3.27646 | 0.14446 |

*INT1”*

**Energy (PBE0-D3(BJ)CPCM($C_6H_6$)/def2-SVP) =** -2193.709632

| | | | |
|---|---|---|---|
| 6 | 3.413379 | 0.996796 | -1.21582 |
| 7 | 2.235219 | 0.715159 | -0.56559 |
| 6 | 1.415355 | 1.788886 | -0.60639 |
| 6 | 3.333489 | 2.277911 | -1.66327 |
| 1 | 4.061853 | 2.868531 | -2.20633 |
| 1 | 4.218375 | 0.273618 | -1.30317 |
| 7 | 2.095477 | 2.75566 | -1.27787 |
| 6 | 1.60604 | 4.136145 | -1.53053 |
| 51 | -0.91839 | 1.00916 | -0.46823 |
| 7 | 1.055072 | -0.40834 | 1.368366 |
| 6 | -0.10294 | 0.271856 | 1.474186 |
| 6 | 0.517527 | -0.39613 | 3.50223 |
| 7 | -0.43805 | 0.294719 | 2.792938 |
| 1 | 0.474596 | -0.52774 | 4.576056 |
| 1 | 2.348685 | -1.41182 | 2.761102 |
| 6 | 1.444101 | -0.83283 | 2.607888 |
| 6 | -1.65315 | 0.880608 | 3.429607 |
| 5 | 1.853862 | -0.69074 | 0.024118 |
| 6 | -2.83523 | -0.01914 | 3.078732 |
| 1 | -2.93333 | -0.14559 | 1.99353 |
| 1 | -2.69906 | -1.0184 | 3.518762 |
| 1 | -3.77088 | 0.409114 | 3.464758 |
| 6 | -1.48339 | 0.894599 | 4.948314 |
| 1 | -1.43418 | -0.1158 | 5.378892 |
| 1 | -0.59521 | 1.4654 | 5.256778 |
| 1 | -2.36442 | 1.388695 | 5.380237 |
| 6 | -1.82143 | 2.323338 | 2.964941 |
| 1 | -0.96704 | 2.927409 | 3.306269 |
| 1 | -1.88964 | 2.433698 | 1.877771 |
| 1 | -2.73747 | 2.740588 | 3.406528 |
| 6 | 1.253516 | 4.776476 | -0.18901 |
| 1 | 0.443946 | 4.216633 | 0.295017 |
| 1 | 2.135355 | 4.798309 | 0.469323 |
| 1 | 0.914645 | 5.810943 | -0.35007 |
| 6 | 0.386444 | 4.070387 | -2.44939 |
| 1 | 0.620775 | 3.509174 | -3.36651 |
| 1 | -0.46053 | 3.604745 | -1.93158 |
| 1 | 0.081961 | 5.088149 | -2.73535 |
| 6 | 2.702376 | 4.952348 | -2.21007 |
| 1 | 3.609736 | 5.018823 | -1.59148 |
| 1 | 2.97039 | 4.54703 | -3.19724 |

| | | | |
|---|---|---|---|
| 1 | 2.328792 | 5.97418 | -2.36314 |
| 6 | 0.878937 | -1.48865 | -0.99385 |
| 6 | 1.038476 | -1.3832 | -2.38497 |
| 6 | -0.10924 | -2.37569 | -0.53965 |
| 6 | 0.230684 | -2.0892 | -3.27 |
| 6 | -0.9231 | -3.0861 | -1.42196 |
| 6 | -0.7767 | -2.94709 | -2.80555 |
| 1 | 1.802382 | -0.71035 | -2.78678 |
| 1 | -0.26134 | -2.51004 | 0.53608 |
| 1 | 0.3772 | -1.9713 | -4.34817 |
| 1 | -1.69466 | -3.75311 | -1.02749 |
| 6 | 3.200564 | -1.51982 | 0.375392 |
| 6 | 4.253792 | -0.93582 | 1.103528 |
| 6 | 3.37079 | -2.85752 | 0.000153 |
| 6 | 5.403178 | -1.64536 | 1.438049 |
| 6 | 4.521109 | -3.5762 | 0.33175 |
| 6 | 5.560422 | -2.98502 | 1.054977 |
| 1 | 4.171347 | 0.108416 | 1.422921 |
| 1 | 2.58061 | -3.35552 | -0.56855 |
| 1 | 6.19727 | -1.15328 | 2.008407 |
| 1 | 4.612839 | -4.62125 | 0.020371 |
| 6 | -1.67833 | -3.66943 | -3.76397 |
| 1 | -2.15335 | -4.54133 | -3.29183 |
| 1 | -2.48505 | -3.00817 | -4.12401 |
| 1 | -1.12907 | -4.01668 | -4.65193 |
| 6 | 6.812324 | -3.74217 | 1.391659 |
| 1 | 7.140781 | -3.54028 | 2.422589 |
| 1 | 6.669076 | -4.82673 | 1.283419 |
| 1 | 7.642399 | -3.45059 | 0.726568 |
| 9 | -1.25828 | 2.952064 | -0.04389 |
| 14 | -3.60411 | 1.097493 | -0.13345 |
| 6 | -4.41701 | -0.60293 | -0.28145 |
| 6 | -5.30225 | -1.09785 | 0.690739 |
| 6 | -4.0932 | -1.44589 | -1.35927 |
| 6 | -5.84374 | -2.37933 | 0.592808 |
| 1 | -5.58008 | -0.46625 | 1.539852 |
| 6 | -4.63626 | -2.725 | -1.46478 |
| 1 | -3.39979 | -1.10115 | -2.13318 |
| 6 | -5.51247 | -3.19727 | -0.48708 |
| 1 | -6.5332 | -2.7395 | 1.361033 |
| 1 | -4.37199 | -3.35879 | -2.31494 |
| 1 | -5.93809 | -4.2006 | -0.56846 |
| 1 | -4.29518 | 1.844154 | 0.971087 |
| 1 | -3.91733 | 1.838818 | -1.4074 |

*10.11.2.16 TS2”*

**Energy (PBE0-D3(BJ)CPCM($C_6H_6$)/def2-SVP) =** -2193.700931

| | | | |
|---|---|---|---|
| 6 | -2.89448 | 2.186251 | -0.21103 |
| 7 | -1.91751 | 1.220129 | -0.18724 |
| 6 | -0.70954 | 1.807952 | -0.24993 |
| 6 | -2.27381 | 3.392019 | -0.27718 |
| 1 | -2.69376 | 4.390127 | -0.31047 |
| 1 | -3.95035 | 1.941286 | -0.16499 |
| 7 | -0.9149 | 3.147949 | -0.28578 |
| 6 | 0.094928 | 4.238132 | -0.37987 |
| 51 | 1.076254 | 0.439279 | 0.076602 |
| 7 | -1.33395 | -1.16791 | -0.87488 |
| 6 | -0.013 | -1.0641 | -1.11593 |
| 6 | -0.79565 | -2.83 | -2.21831 |
| 7 | 0.328288 | -2.08455 | -1.94667 |
| 1 | -0.78432 | -3.70273 | -2.8592 |
| 1 | -2.874 | -2.53631 | -1.49214 |
| 6 | -1.82804 | -2.25421 | -1.54677 |
| 6 | 1.657317 | -2.3302 | -2.5712 |
| 5 | -2.19376 | -0.29865 | 0.132212 |
| 6 | 2.685158 | -2.52655 | -1.46125 |
| 1 | 2.768805 | -1.65295 | -0.80258 |
| 1 | 2.421464 | -3.39658 | -0.84189 |
| 1 | 3.68044 | -2.69533 | -1.89436 |
| 6 | 1.611776 | -3.61222 | -3.39901 |
| 1 | 1.369686 | -4.49436 | -2.78782 |
| 1 | 0.901624 | -3.54268 | -4.23546 |
| 1 | 2.609933 | -3.77001 | -3.83 |
| 6 | 1.954984 | -1.14997 | -3.4949 |
| 1 | 1.250608 | -1.16114 | -4.34087 |
| 1 | 1.851334 | -0.18405 | -2.98151 |
| 1 | 2.975798 | -1.23192 | -3.89458 |
| 6 | 0.419133 | 4.466909 | -1.85532 |
| 1 | 0.827838 | 3.539095 | -2.28088 |
| 1 | -0.48565 | 4.765134 | -2.40704 |
| 1 | 1.163325 | 5.271503 | -1.95325 |
| 6 | 1.342115 | 3.83091 | 0.391955 |
| 1 | 1.098176 | 3.522073 | 1.419998 |
| 1 | 1.86392 | 3.031329 | -0.14363 |
| 1 | 2.03575 | 4.681707 | 0.444063 |
| 6 | -0.48093 | 5.50667 | 0.249563 |

| | | | |
|---|---|---|---|
| 1 | -1.31446 | 5.927046 | -0.33085 |
| 1 | -0.81763 | 5.330097 | 1.28231 |
| 1 | 0.308707 | 6.270212 | 0.273733 |
| 6 | -1.71375 | -0.62007 | 1.642872 |
| 6 | -1.78738 | 0.345315 | 2.656419 |
| 6 | -1.27585 | -1.89725 | 2.021679 |
| 6 | -1.41967 | 0.060937 | 3.969881 |
| 6 | -0.90682 | -2.18913 | 3.332672 |
| 6 | -0.96292 | -1.21132 | 4.333112 |
| 1 | -2.13301 | 1.3554 | 2.413557 |
| 1 | -1.21 | -2.68951 | 1.268668 |
| 1 | -1.48519 | 0.843651 | 4.73205 |
| 1 | -0.56324 | -3.19661 | 3.587096 |
| 6 | -3.7617 | -0.61273 | -0.12127 |
| 6 | -4.36171 | -0.36848 | -1.37127 |
| 6 | -4.58935 | -1.13781 | 0.876463 |
| 6 | -5.70623 | -0.63349 | -1.60796 |
| 6 | -5.94088 | -1.40676 | 0.64614 |
| 6 | -6.52581 | -1.15973 | -0.59801 |
| 1 | -3.75618 | 0.04223 | -2.18642 |
| 1 | -4.16693 | -1.34376 | 1.863792 |
| 1 | -6.13367 | -0.42968 | -2.59477 |
| 1 | -6.55443 | -1.8182 | 1.453358 |
| 6 | -0.52245 | -1.51022 | 5.737003 |
| 1 | -0.73169 | -2.55455 | 6.012452 |
| 1 | 0.563893 | -1.35595 | 5.852048 |
| 1 | -1.0232 | -0.85592 | 6.465552 |
| 6 | -7.97929 | -1.43516 | -0.85382 |
| 1 | -8.45373 | -1.92742 | 0.006735 |
| 1 | -8.532 | -0.50275 | -1.05388 |
| 1 | -8.11576 | -2.08291 | -1.73416 |
| 9 | 1.485764 | 1.621887 | -1.84504 |
| 14 | 3.513572 | 0.865783 | -0.89636 |
| 6 | 4.733941 | -0.1106 | 0.190444 |
| 6 | 5.715212 | -0.95275 | -0.35709 |
| 6 | 4.630375 | -0.06025 | 1.591527 |
| 6 | 6.562755 | -1.70619 | 0.45512 |
| 1 | 5.822574 | -1.01738 | -1.4449 |
| 6 | 5.473405 | -0.80984 | 2.410836 |
| 1 | 3.873055 | 0.579576 | 2.057901 |
| 6 | 6.443415 | -1.63682 | 1.843309 |
| 1 | 7.32252 | -2.35 | 0.003498 |
| 1 | 5.372996 | -0.75032 | 3.497899 |
| 1 | 7.105191 | -2.22688 | 2.482475 |

| | | | |
|---|---|---|---|
| 1 | 3.948641 | 0.585389 | -2.29614 |
| 1 | 3.776035 | 2.301202 | -0.56009 |

*10.11.2.17 TS1'''*

**Energy (PBE0-D3(BJ)CPCM($C_6H_6$)/def2-SVP) =** -2838.543677

| | | | |
|---|---|---|---|
| 6 | 2.878328 | 0.395557 | 2.315782 |
| 7 | 2.123073 | -0.02898 | 1.252926 |
| 6 | 1.209094 | -0.9095 | 1.691123 |
| 6 | 2.424378 | -0.24267 | 3.425173 |
| 1 | 2.762904 | -0.17958 | 4.453595 |
| 1 | 3.684873 | 1.111613 | 2.196366 |
| 7 | 1.390325 | -1.06095 | 3.03071 |
| 6 | 0.714555 | -1.93143 | 4.040649 |
| 51 | -0.30474 | -1.82669 | 0.359757 |
| 7 | 2.011598 | -0.49221 | -1.28223 |
| 6 | 1.054434 | -1.43318 | -1.32829 |
| 6 | 2.155767 | -1.46901 | -3.25157 |
| 7 | 1.150951 | -2.06792 | -2.52997 |
| 1 | 2.426221 | -1.77801 | -4.2532 |
| 1 | 3.519484 | 0.180444 | -2.65931 |
| 6 | 2.693088 | -0.49656 | -2.46972 |
| 6 | 0.333704 | -3.1819 | -3.11326 |
| 5 | 2.347947 | 0.597219 | -0.18339 |
| 6 | -0.87233 | -2.56659 | -3.81993 |
| 1 | -1.51916 | -2.03838 | -3.11027 |
| 1 | -0.54406 | -1.86637 | -4.60249 |
| 1 | -1.46402 | -3.36312 | -4.29412 |
| 6 | 1.199317 | -3.94325 | -4.12072 |
| 1 | 1.435571 | -3.35063 | -5.01501 |
| 1 | 2.136435 | -4.29784 | -3.66606 |
| 1 | 0.633441 | -4.81975 | -4.4633 |
| 6 | -0.09409 | -4.16533 | -2.02886 |
| 1 | 0.758636 | -4.46957 | -1.40301 |
| 1 | -0.90432 | -3.77321 | -1.40479 |
| 1 | -0.49331 | -5.06561 | -2.51615 |
| 6 | -0.43293 | -2.73123 | 3.446348 |
| 1 | -1.21866 | -2.08343 | 3.033159 |
| 1 | -0.09313 | -3.44753 | 2.686411 |
| 1 | -0.89405 | -3.31427 | 4.255623 |
| 6 | 0.171002 | -1.02848 | 5.15045 |
| 1 | 0.971192 | -0.4898 | 5.67648 |
| 1 | -0.53338 | -0.29433 | 4.737849 |
| 1 | -0.35658 | -1.64386 | 5.89262 |
| 6 | 1.764321 | -2.90383 | 4.58371 |
| 1 | 2.173808 | -3.52492 | 3.773174 |
| 1 | 2.597684 | -2.38086 | 5.073569 |

| | | | |
|---|---|---|---|
| 1 | 1.300974 | -3.5675 | 5.327401 |
| 6 | 1.432584 | 1.902536 | -0.39958 |
| 6 | 1.04847 | 2.724494 | 0.668536 |
| 6 | 1.108248 | 2.352374 | -1.68697 |
| 6 | 0.372924 | 3.924365 | 0.46373 |
| 6 | 0.433543 | 3.552294 | -1.89829 |
| 6 | 0.043116 | 4.357876 | -0.82442 |
| 1 | 1.269362 | 2.421245 | 1.696442 |
| 1 | 1.376642 | 1.749117 | -2.55901 |
| 1 | 0.061168 | 4.522361 | 1.324979 |
| 1 | 0.179107 | 3.857673 | -2.91719 |
| 6 | 3.935342 | 0.922591 | -0.30483 |
| 6 | 4.889318 | -0.10037 | -0.15369 |
| 6 | 4.426263 | 2.208795 | -0.54902 |
| 6 | 6.254007 | 0.147233 | -0.24346 |
| 6 | 5.797314 | 2.465245 | -0.63952 |
| 6 | 6.736226 | 1.442152 | -0.4899 |
| 1 | 4.551942 | -1.12441 | 0.041463 |
| 1 | 3.719463 | 3.034156 | -0.67129 |
| 1 | 6.96524 | -0.67556 | -0.12008 |
| 1 | 6.143661 | 3.485375 | -0.83056 |
| 6 | -0.79017 | 5.585296 | -1.03327 |
| 1 | -0.55148 | 6.371257 | -0.30134 |
| 1 | -0.66274 | 5.998971 | -2.04413 |
| 1 | -1.85532 | 5.328716 | -0.90598 |
| 6 | 8.210641 | 1.705449 | -0.59131 |
| 1 | 8.654783 | 1.163007 | -1.44163 |
| 1 | 8.420265 | 2.77539 | -0.72917 |
| 1 | 8.740441 | 1.369502 | 0.314191 |
| 6 | -1.60403 | -0.04061 | 0.331255 |
| 6 | -1.74769 | 0.757579 | 1.493995 |
| 6 | -1.77553 | 0.715057 | -0.85578 |
| 6 | -2.24102 | 2.041316 | 1.423926 |
| 6 | -2.27642 | 2.001716 | -0.80535 |
| 7 | -2.50947 | 2.67008 | 0.301786 |
| 9 | -1.56037 | 0.172082 | 2.697091 |
| 9 | -1.52512 | 0.147713 | -2.0475 |
| 9 | -2.44201 | 2.712176 | 2.55674 |
| 9 | -2.51638 | 2.63196 | -1.94952 |
| 9 | -2.55668 | -2.42883 | -1.19923 |
| 14 | -3.99407 | -1.97308 | -0.35216 |
| 6 | -4.84076 | -0.28402 | -0.29365 |
| 1 | -2.76212 | -1.08421 | 0.479681 |
| 6 | -5.21531 | 0.392403 | -1.46336 |

| | | | |
|---|---|---|---|
| 6 | -5.02956 | 0.367167 | 0.937454 |
| 6 | -5.75495 | 1.677741 | -1.40886 |
| 1 | -5.07678 | -0.09181 | -2.43557 |
| 6 | -5.60226 | 1.633801 | 1.000337 |
| 1 | -4.71349 | -0.12757 | 1.862099 |
| 6 | -5.95206 | 2.298215 | -0.17629 |
| 1 | -6.01952 | 2.199303 | -2.33235 |
| 1 | -5.74455 | 2.123118 | 1.967299 |
| 1 | -6.37107 | 3.30644 | -0.13104 |
| 1 | -4.82795 | -2.65857 | -1.42556 |
| 1 | -4.26007 | -2.80472 | 0.873547 |

# 11 NMR Characterisation data

## 11.1 NMR characterisation of imidazolium chloride ligands

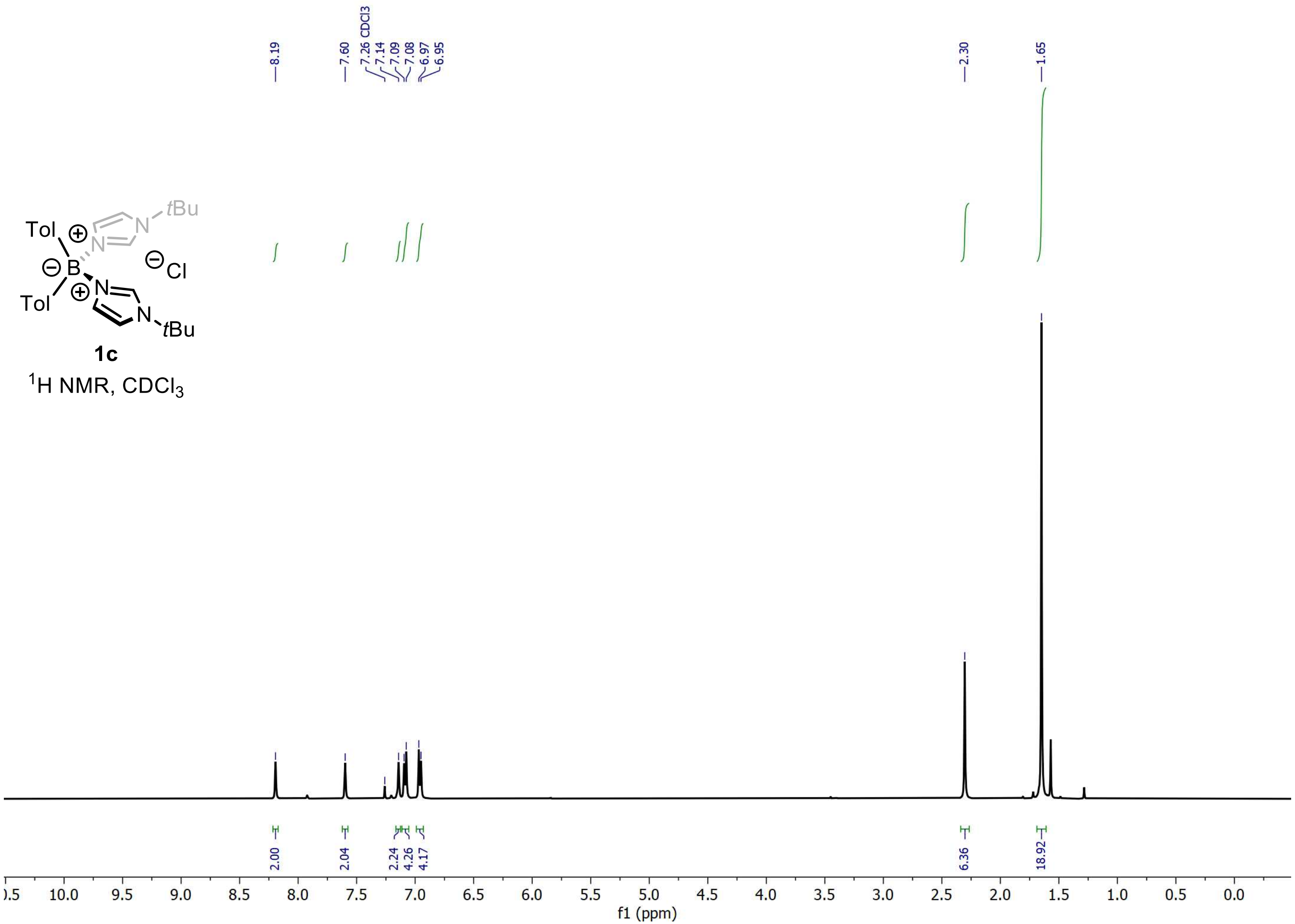

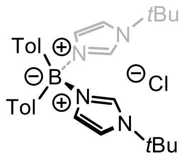

**1c**

$^{11}B$ NMR, $CDCl_3$

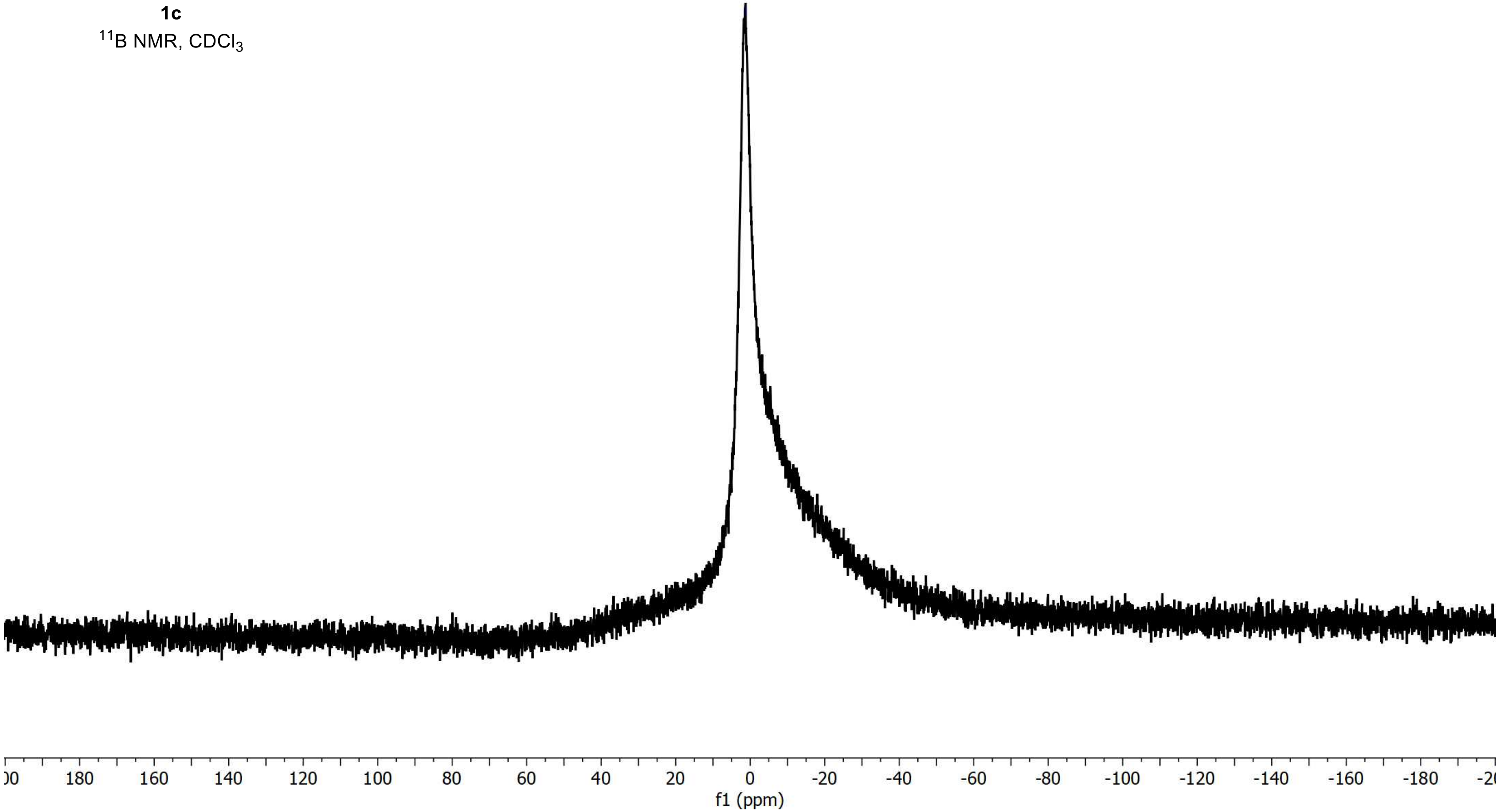

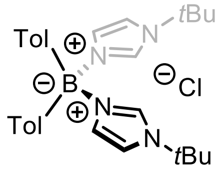

**1c**

$^{13}C$ NMR, $CDCl_3$

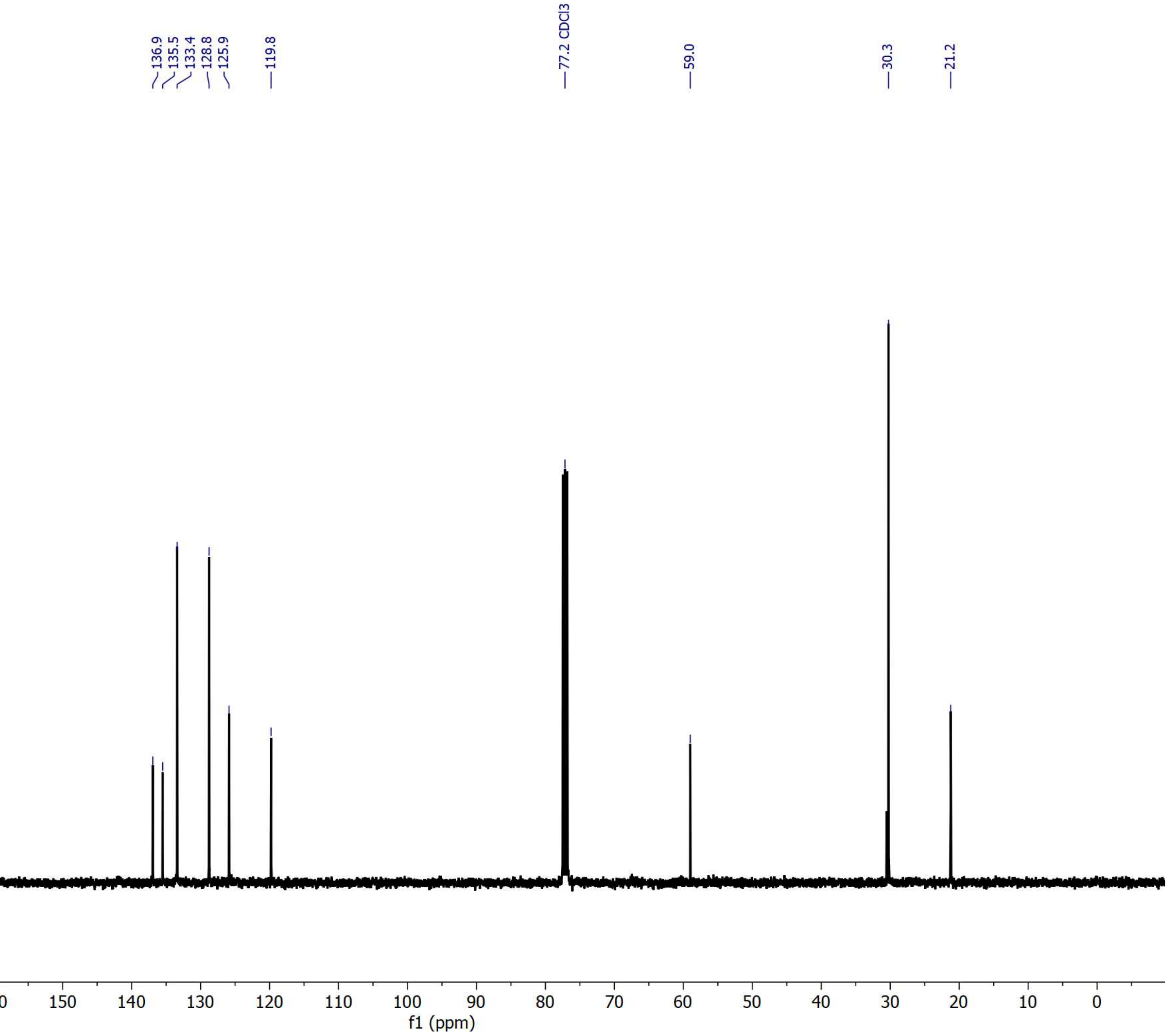

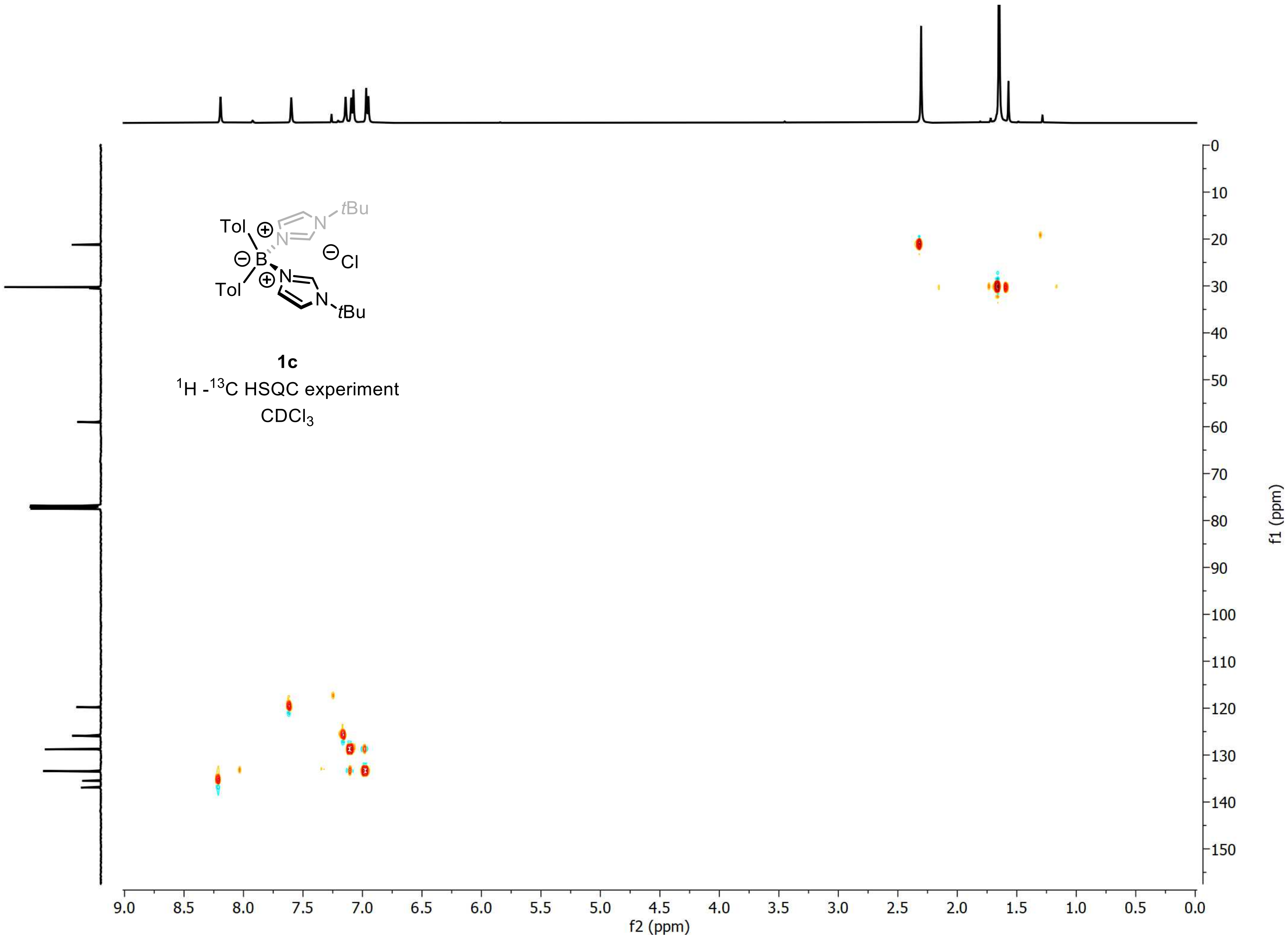
Tol
Tol
B
N
N
N
N
tBu
tBu
Cl
1c
$^1$H -$^{13}$C HSQC experiment
$CDCl_3$
f1 (ppm)
f2 (ppm)
0
10
20
30
40
50
60
70
80
90
100
110
120
130
140
150
9.0
8.5
8.0
7.5
7.0
6.5
6.0
5.5
5.0
4.5
4.0
3.5
3.0
2.5
2.0
1.5
1.0
0.5
0.0

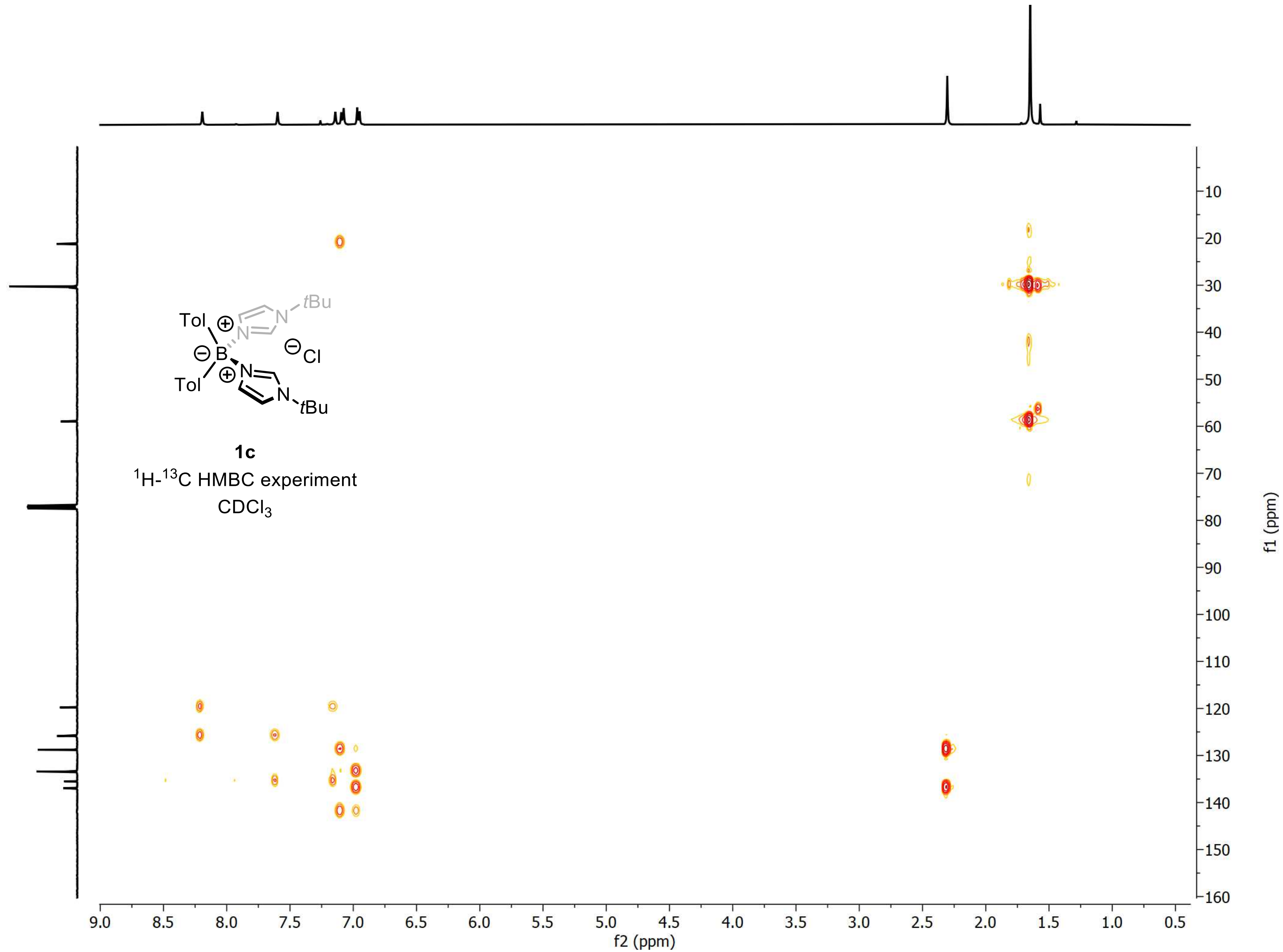


**1c**

$^1$H-$^{13}$C HMBC experiment

$CDCl_3$

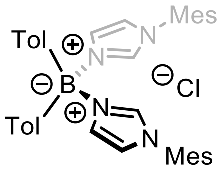


**1b**

$^{1}$H NMR, $CDCl_3$

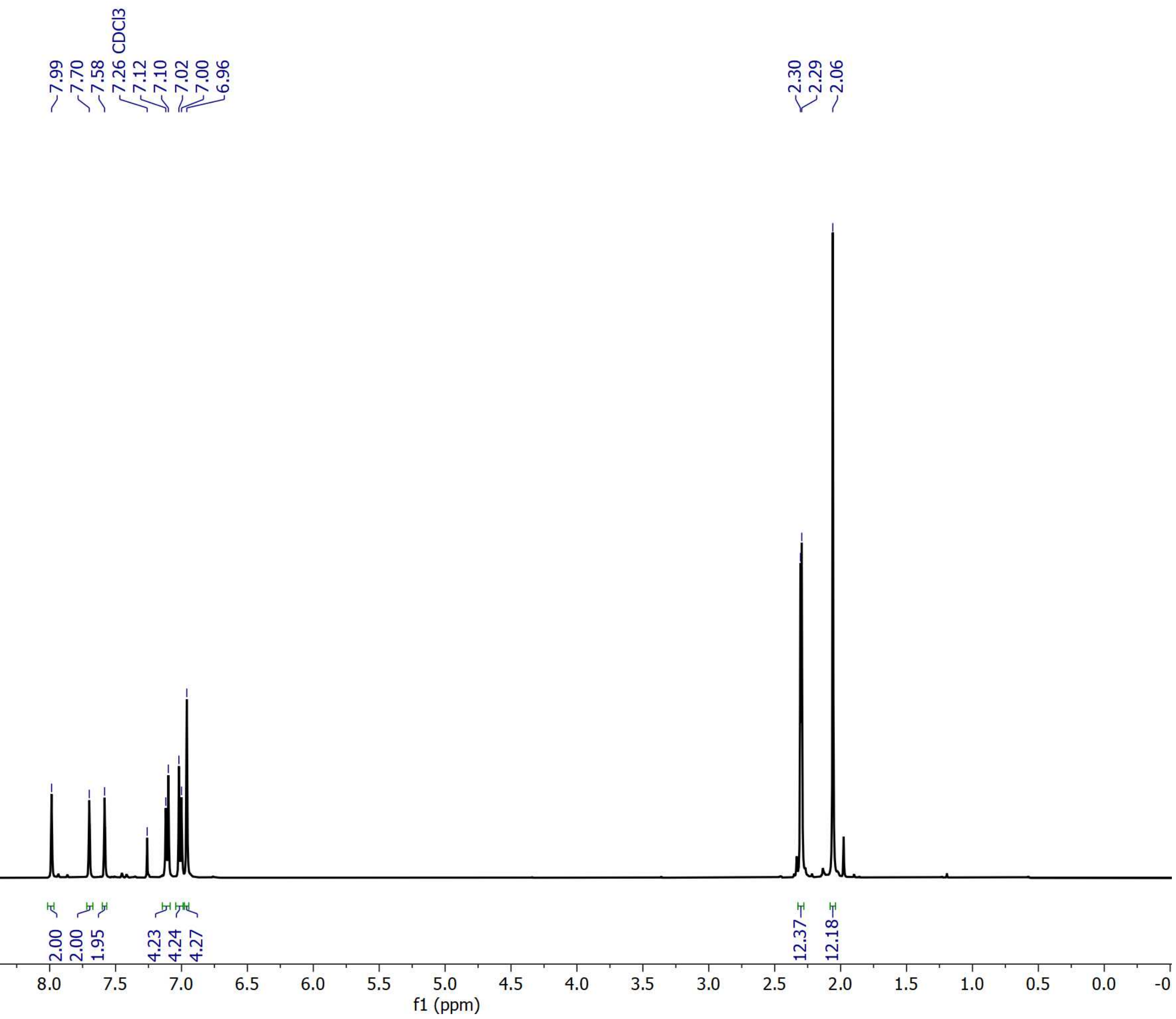

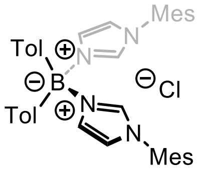


**1b**

$^{11}$B NMR, $CDCl_3$

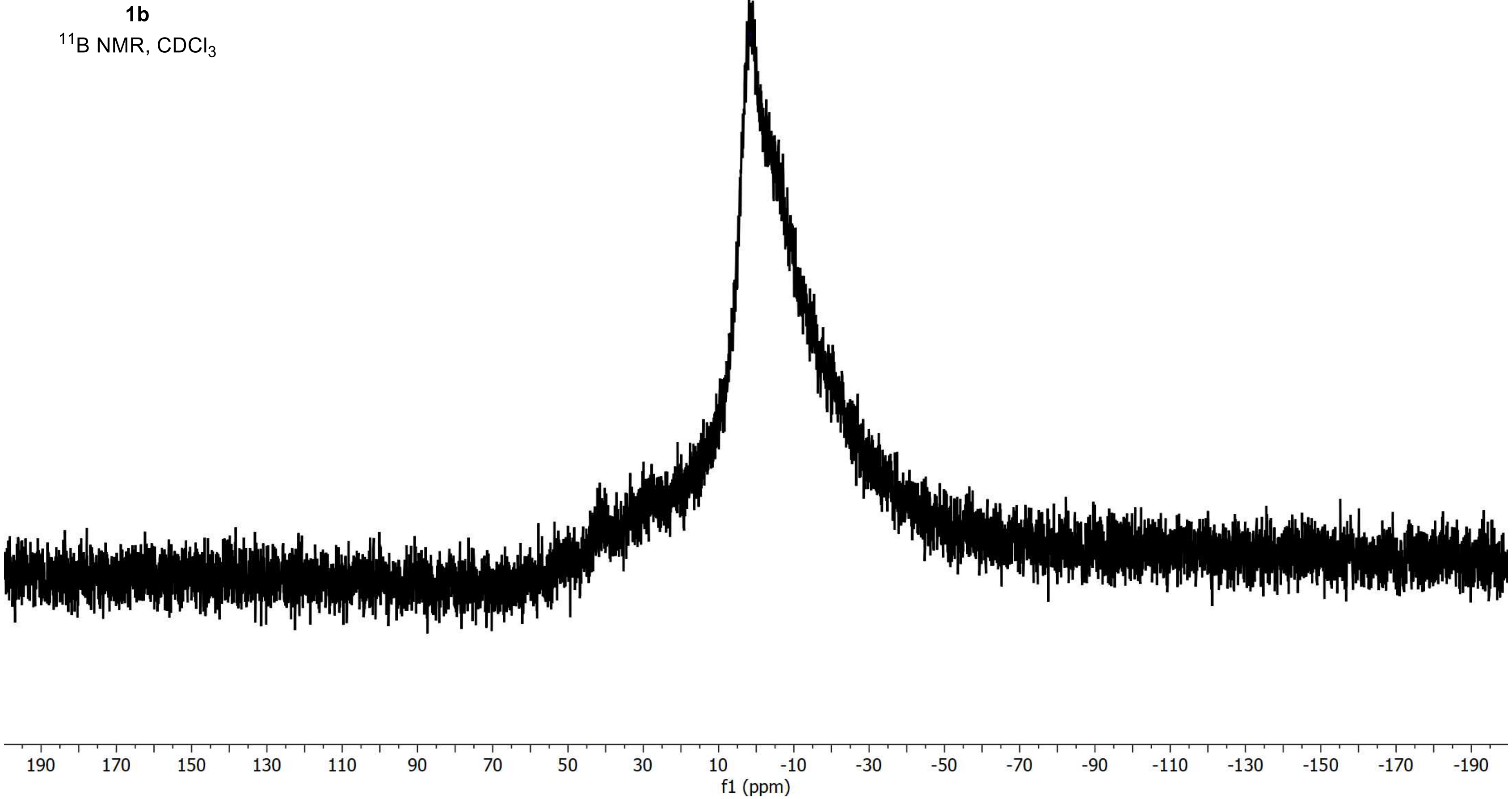

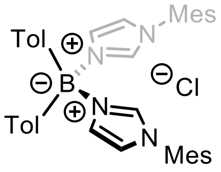

**1b**

$^{13}C$ NMR, $CDCl_3$

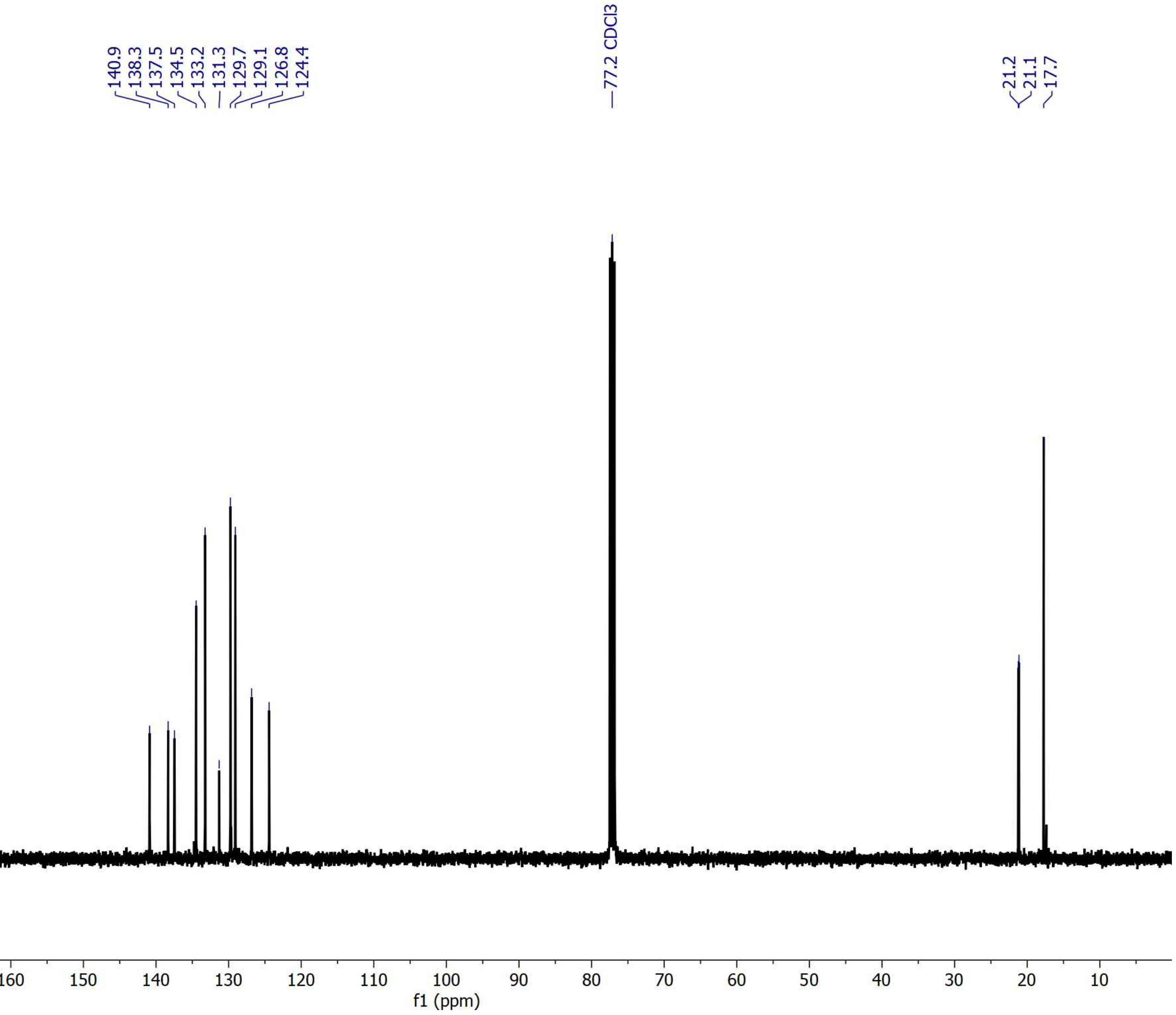

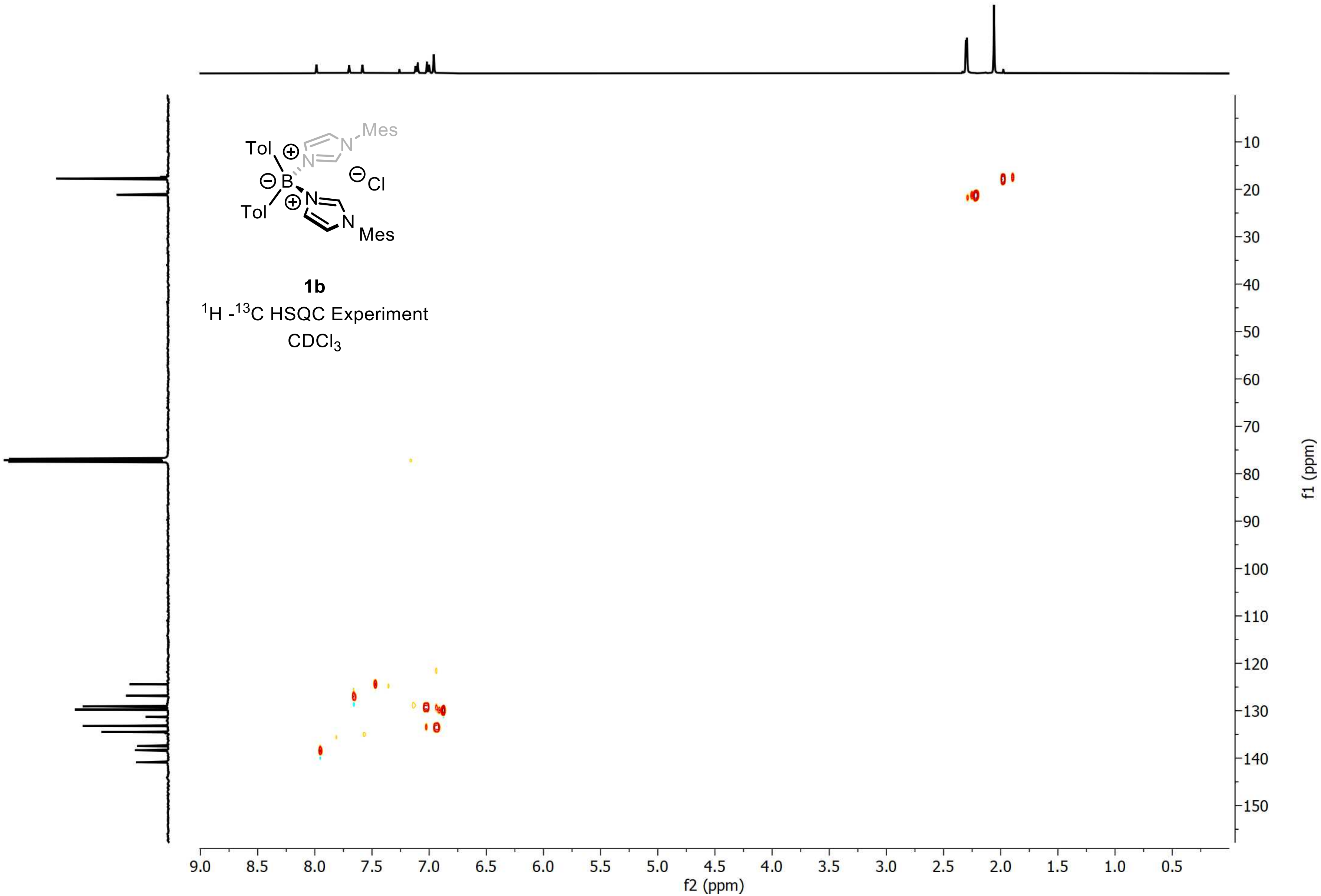
Tol
Tol
B
N
N
Mes
Mes
Cl
1b
$^1$H -$^{13}$C HSQC Experiment
$CDCl_3$
f2 (ppm)
f1 (ppm)
9.0 8.5 8.0 7.5 7.0 6.5 6.0 5.5 5.0 4.5 4.0 3.5 3.0 2.5 2.0 1.5 1.0 0.5
10 20 30 40 50 60 70 80 90 100 110 120 130 140 150

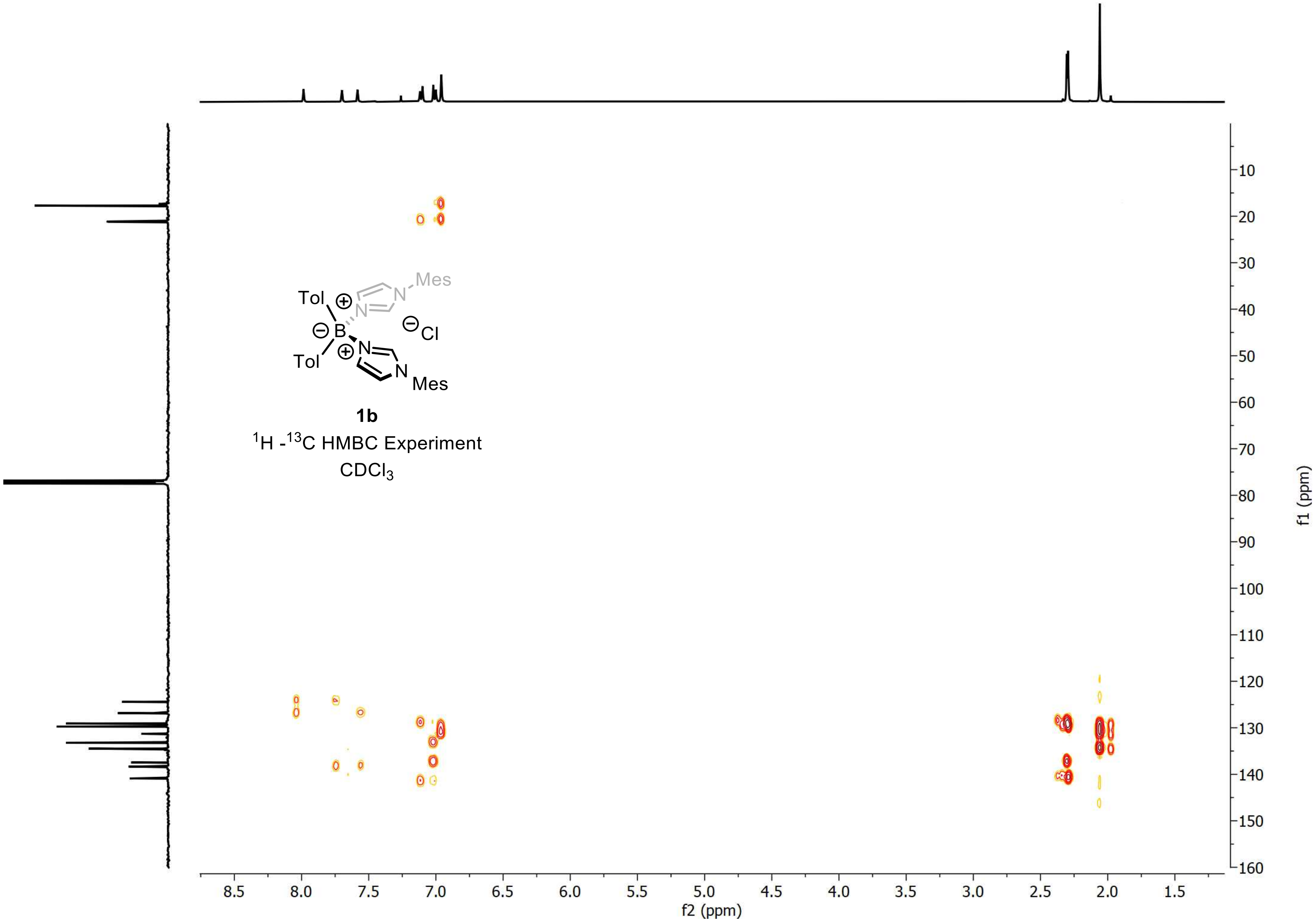

Tol
Tol
B
N
N
Mes
Mes
Cl
1b
$^1$H -$^{13}$C HMBC Experiment
$CDCl_3$
f2 (ppm)
f1 (ppm)

## 11.2 NMR characterisation of imidazolium hexafluorophosphate salt 1a

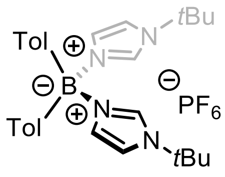


**1a**
$^{1}$H NMR, $CDCl_3$

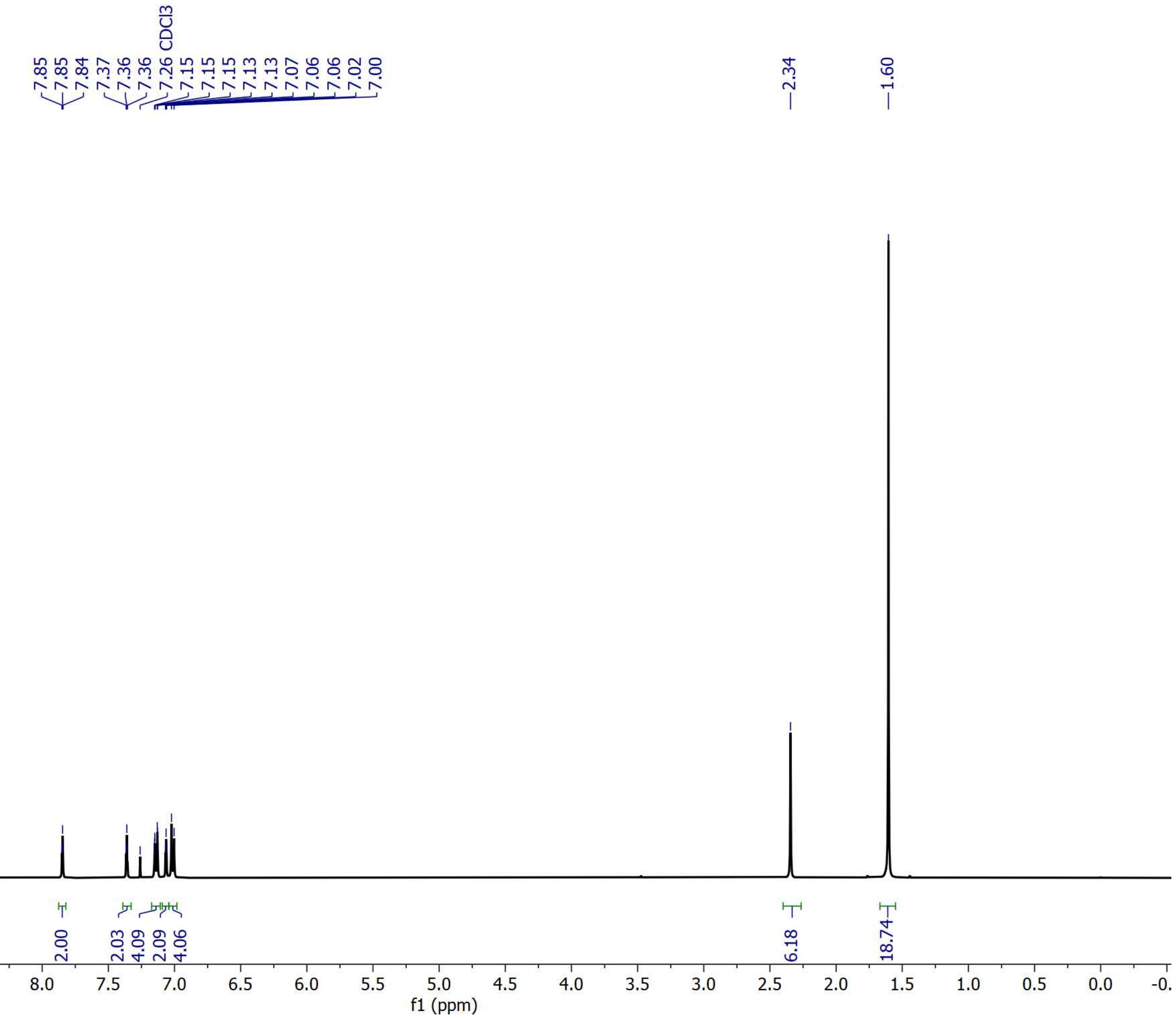

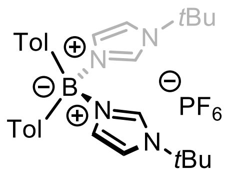


**1a**

$^{19}$F NMR, $CDCl_3$

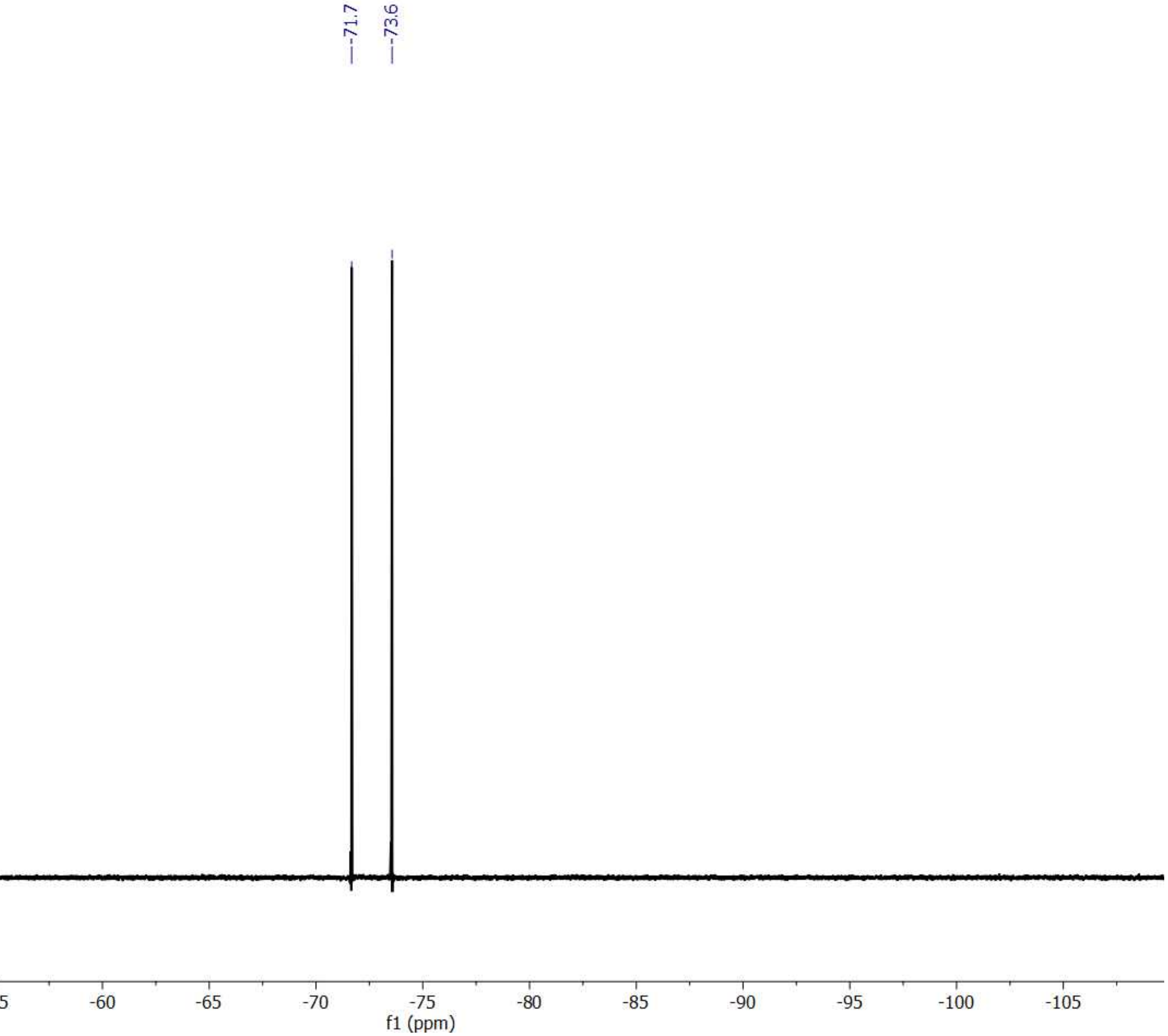

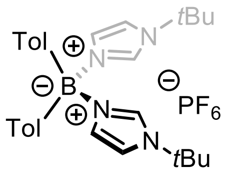


**1a**

$^{31}P$ NMR, $CDCl_3$

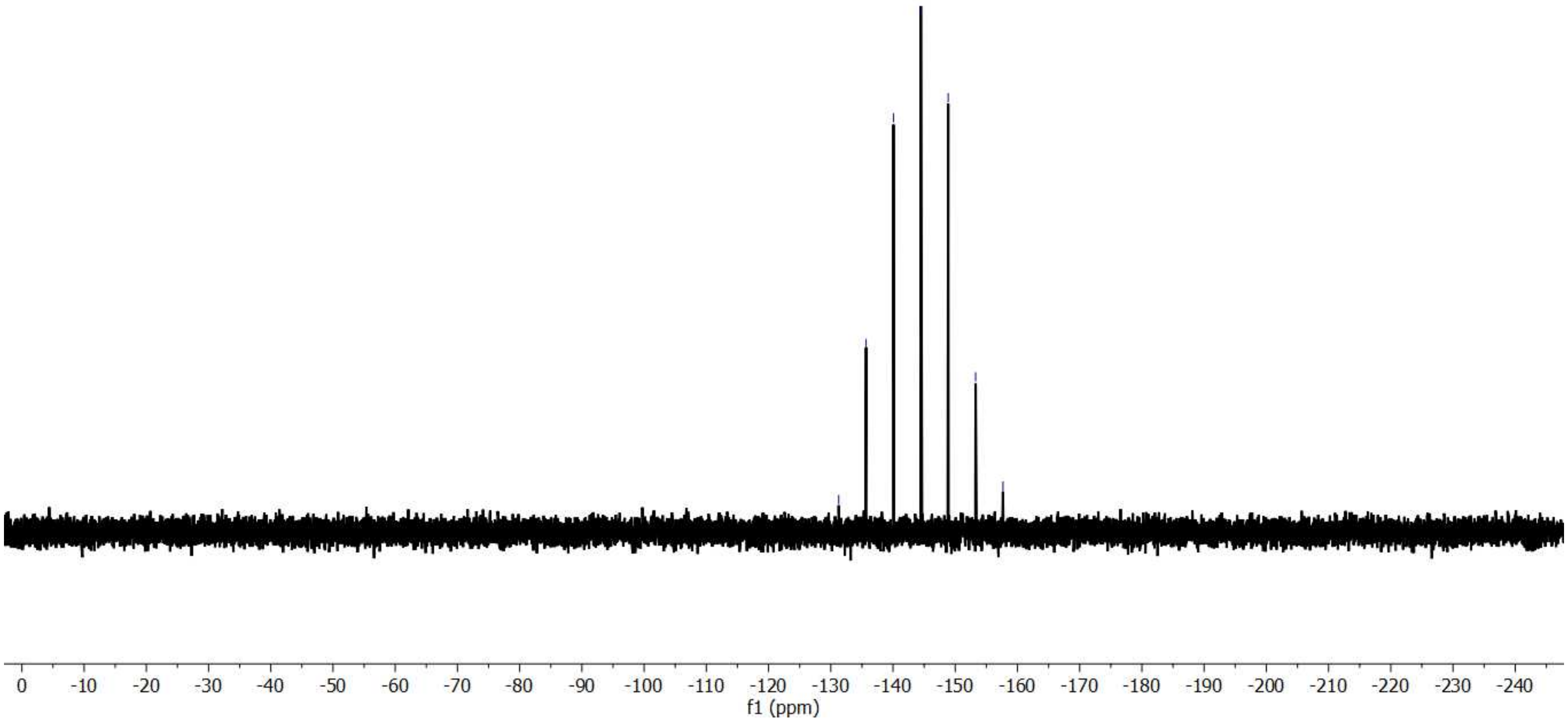

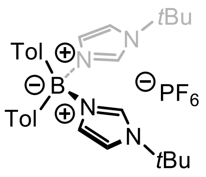


**1a**

$^{11}$B NMR, $CDCl_3$

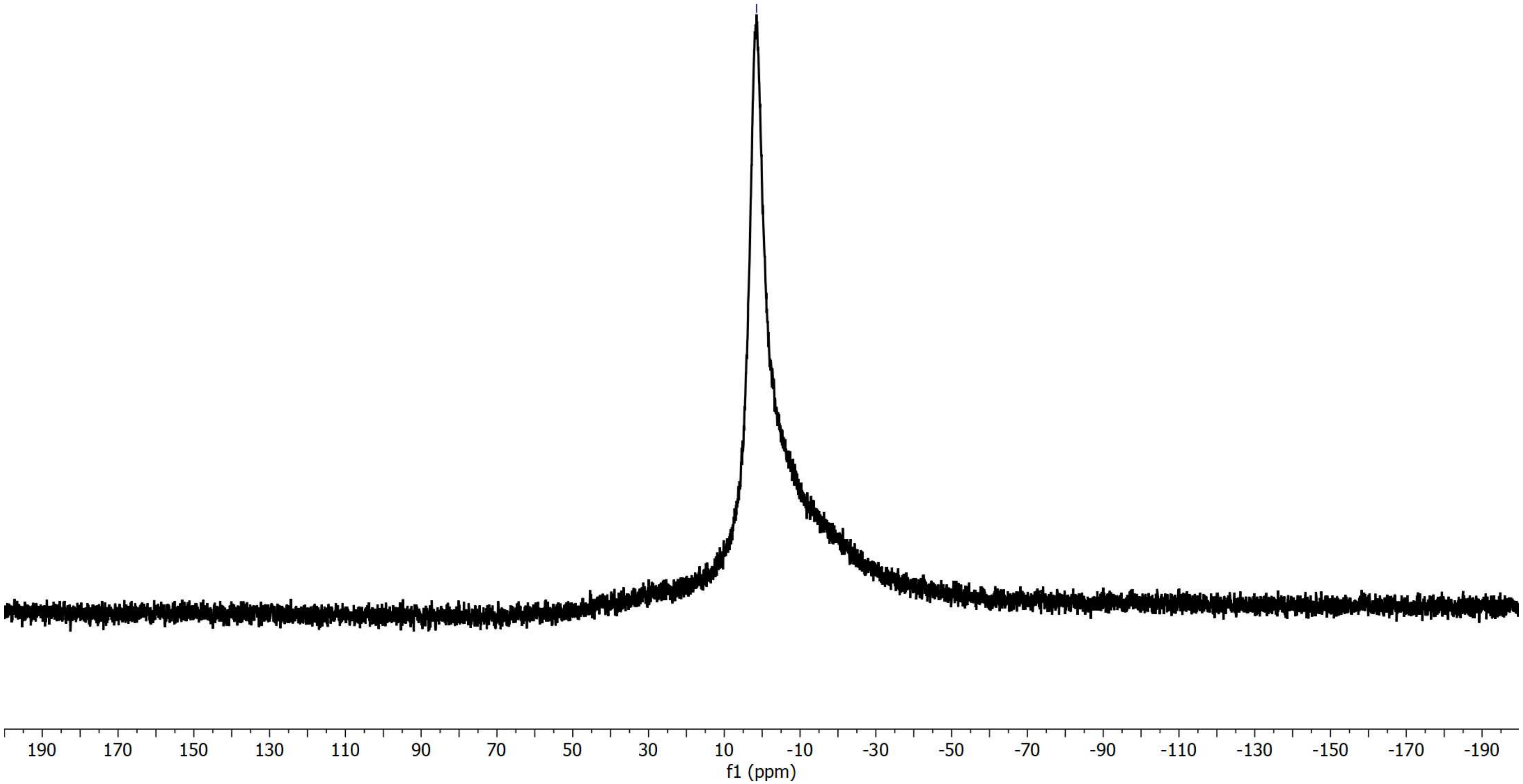

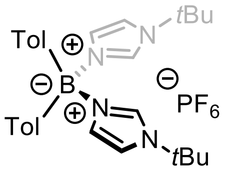


**1a**

$^{13}C$ NMR, $CDCl_3$

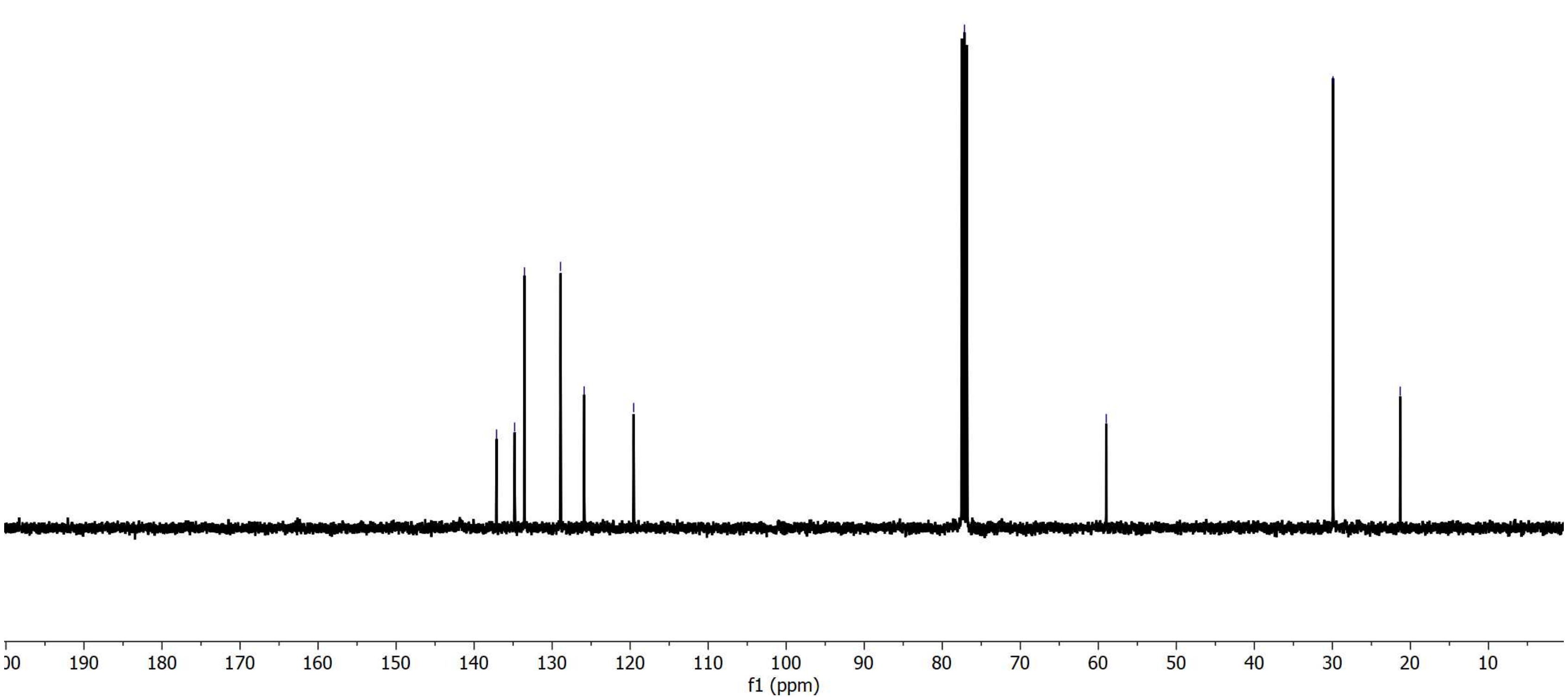

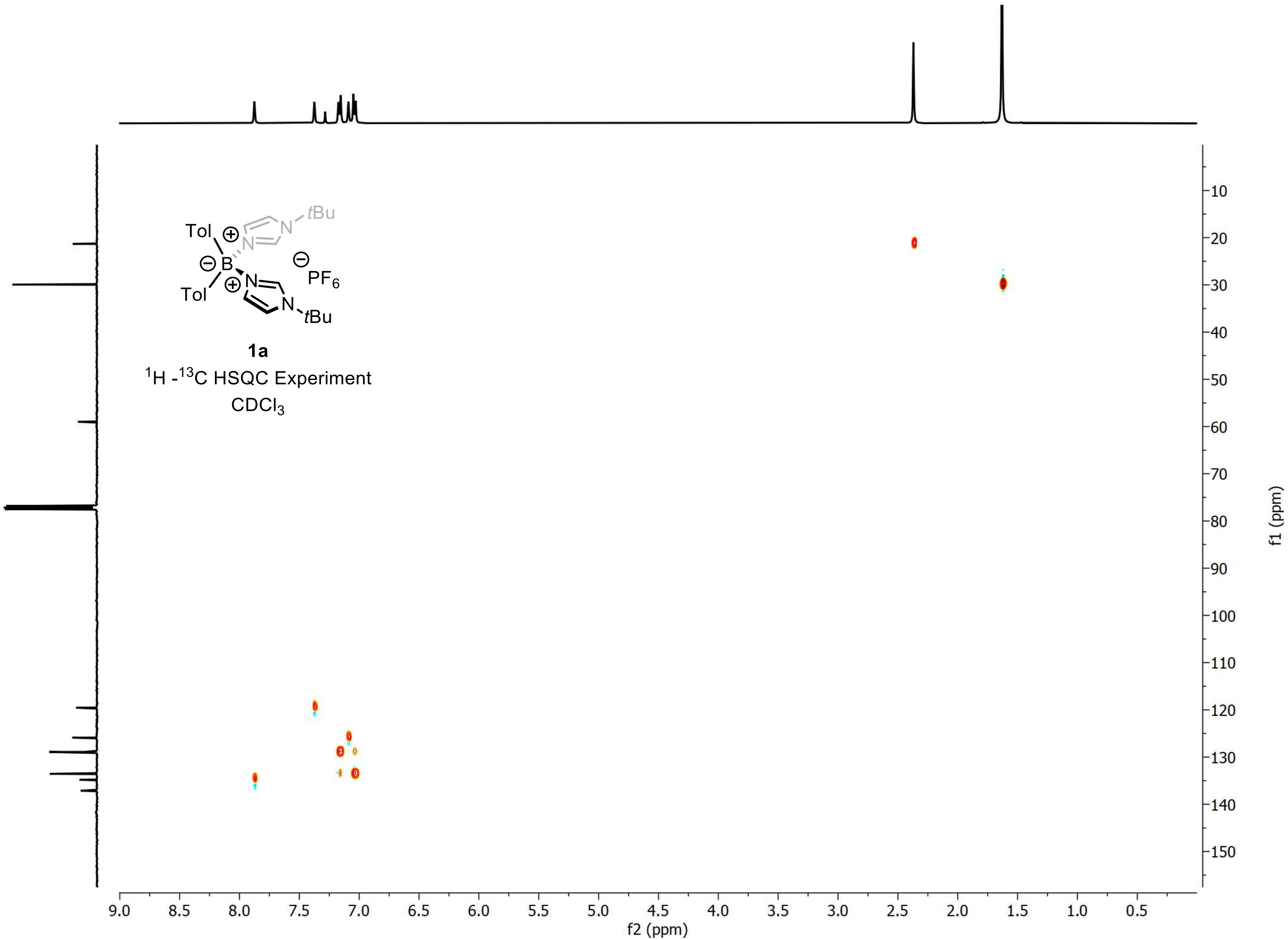

Tol
Tol
tBu
tBu
PF6
1a
1H -13C HSQC Experiment
CDCl3
f1 (ppm)
f2 (ppm)

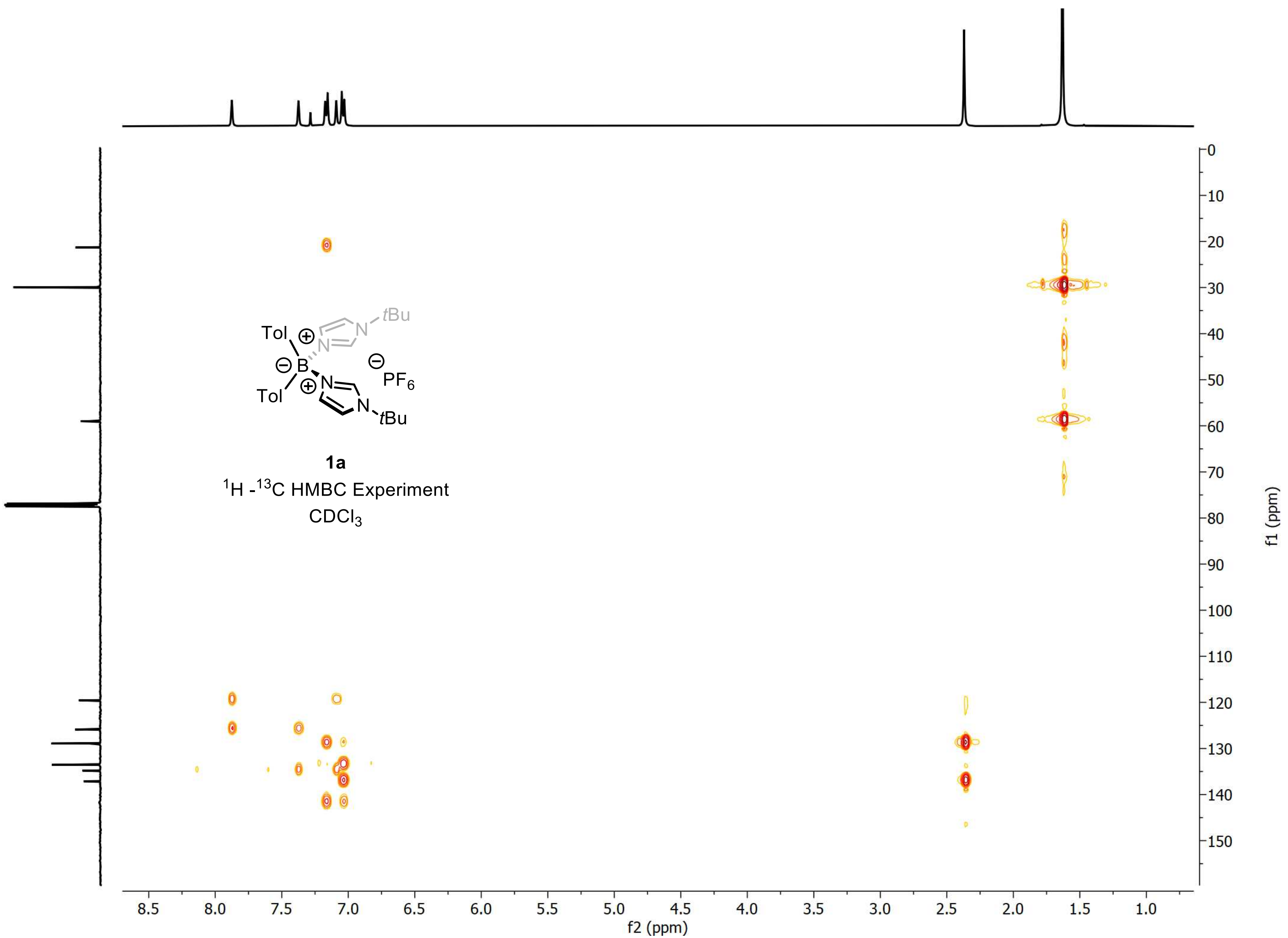
Tol
Tol
B
N
tBu
PF6
1a
1H -13C HMBC Experiment
CDCl3
8.5 8.0 7.5 7.0 6.5 6.0 5.5 5.0 4.5 4.0 3.5 3.0 2.5 2.0 1.5 1.0
f2 (ppm)
0 10 20 30 40 50 60 70 80 90 100 110 120 130 140 150
f1 (ppm)

## 11.3 NMR characterisation of Pn(III) complexes

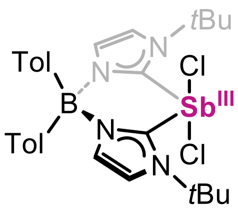


**2a**

$^1$H NMR, $CDCl_3$

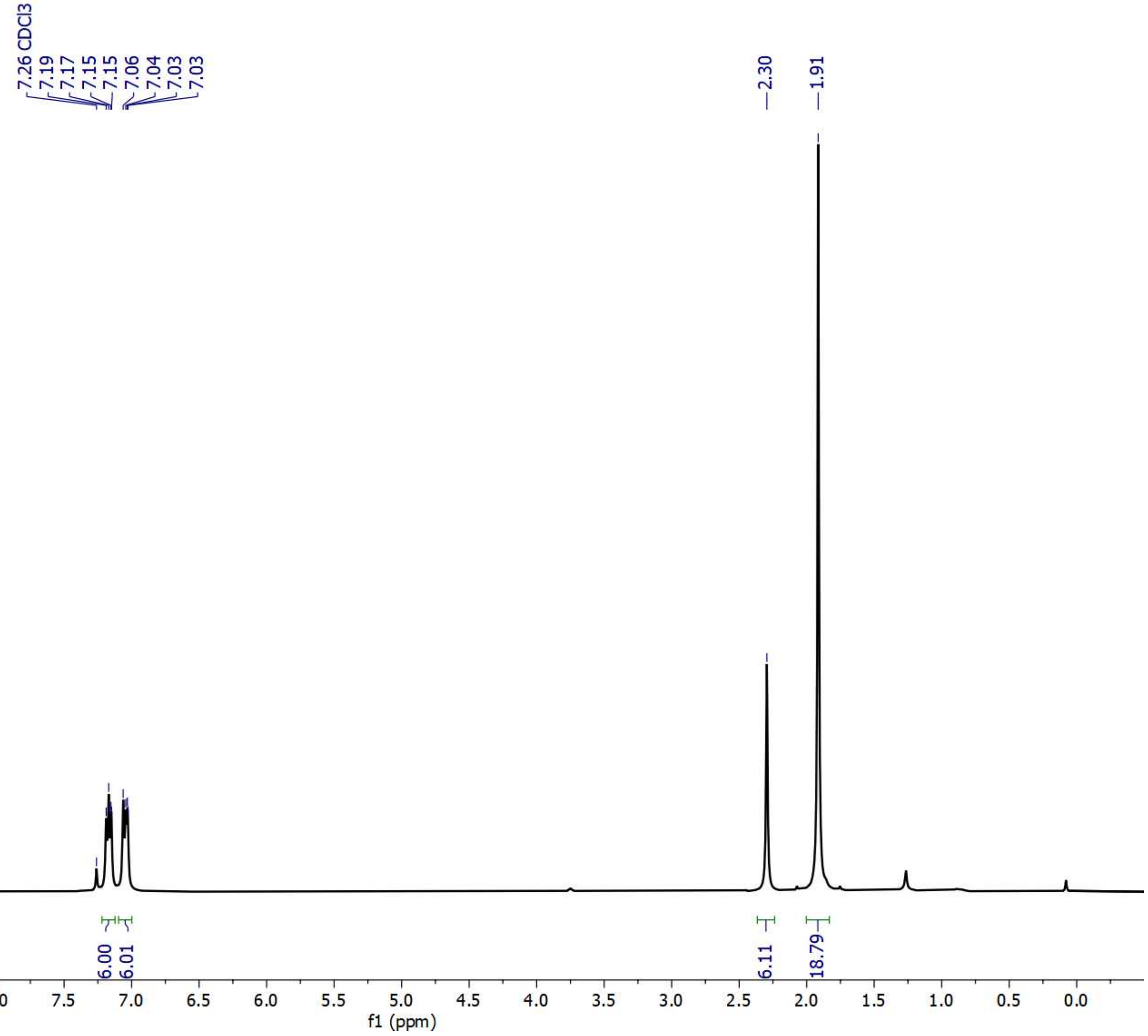

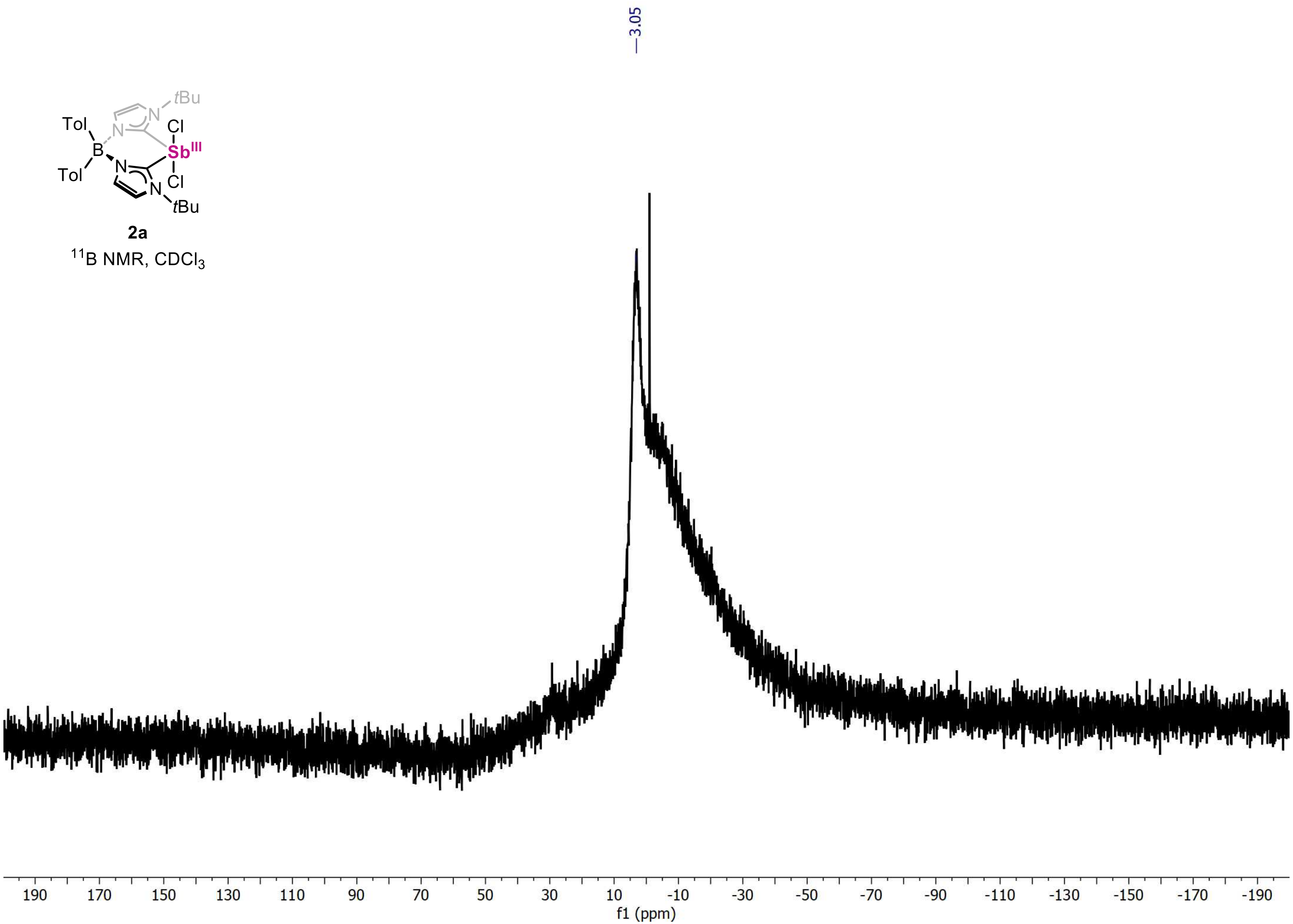

3.05
Tol
Tol
B
N
N
tBu
tBu
Cl
Cl
Sb^III
2a
11B NMR, CDCl3
190 170 150 130 110 90 70 50 30 10 -10 -30 -50 -70 -90 -110 -130 -150 -170 -190
f1 (ppm)

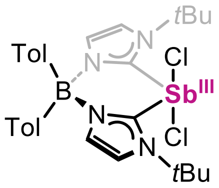


**2a**

$^{13}C$ NMR, $CDCl_3$

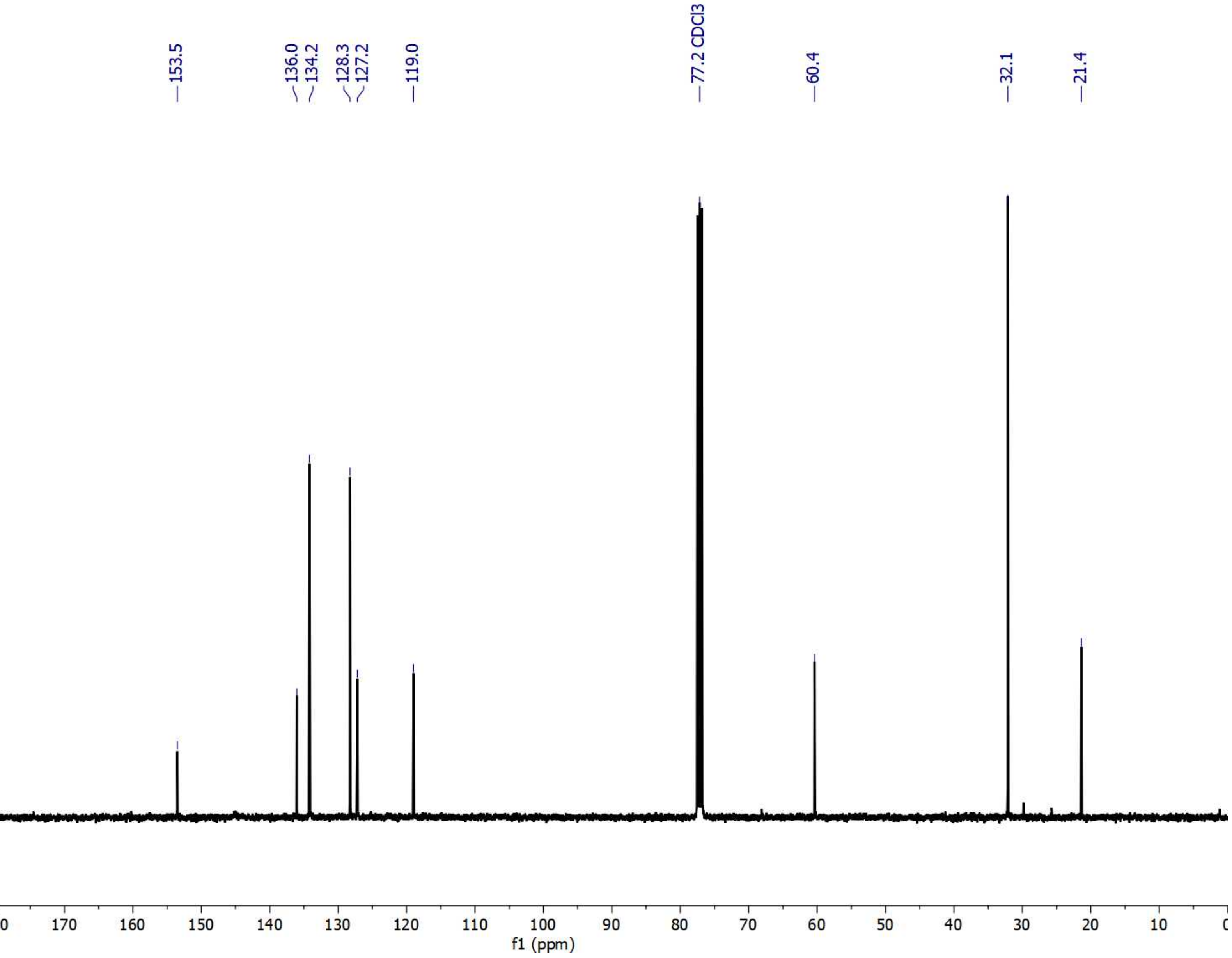

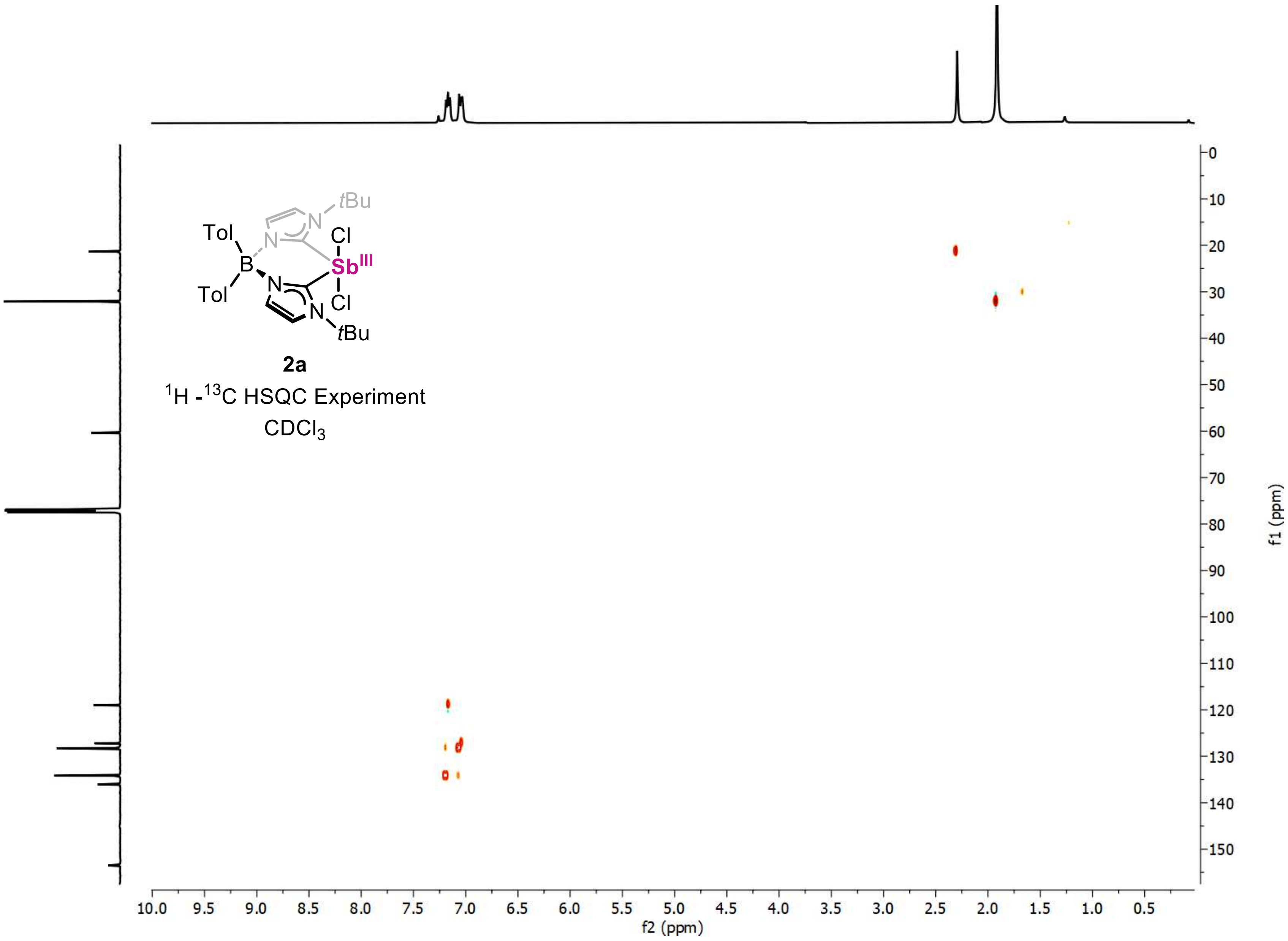
Tol
Tol
B
N
N
tBu
Cl
Sb^III
Cl
N
N
tBu
2a
1H -13C HSQC Experiment
CDCl3
f2 (ppm)
f1 (ppm)

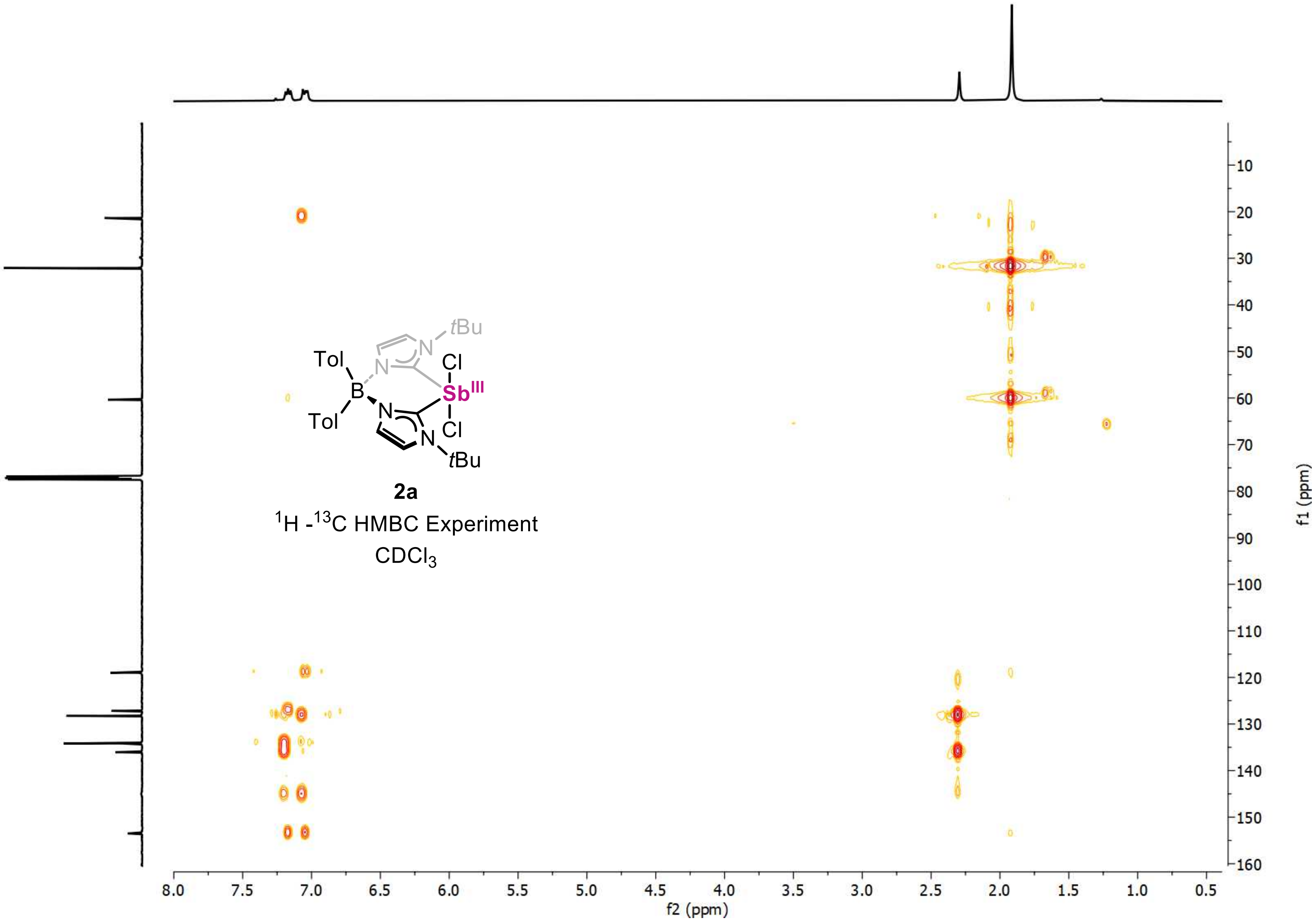


**2a**

$^{1}H$ -$^{13}C$ HMBC Experiment

$CDCl_3$

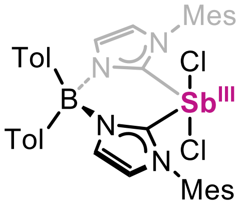


**2b**

$^1$H NMR, $CDCl_3$

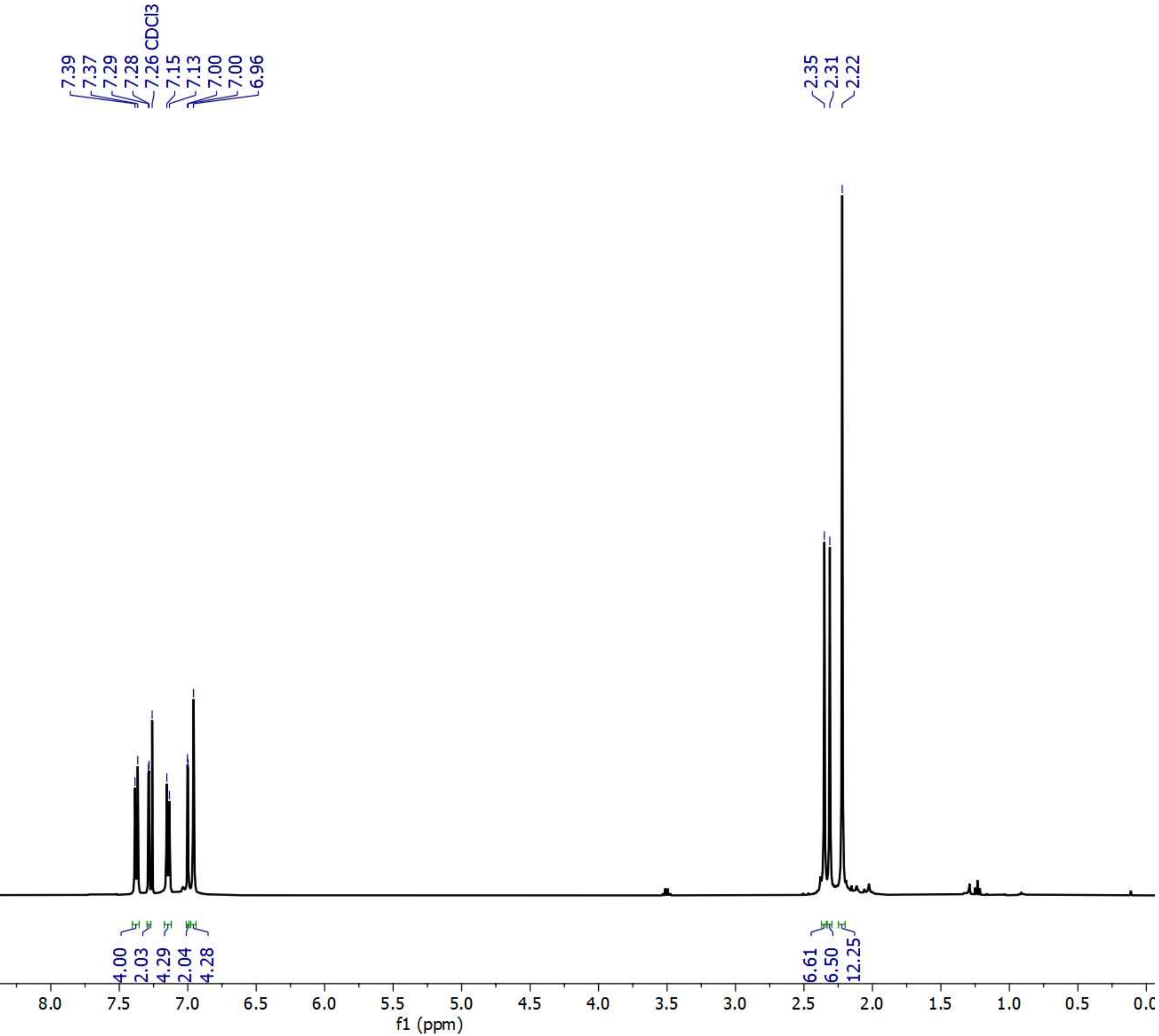

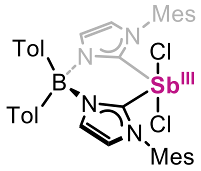


**2b**

$^{11}$B NMR, $CDCl_3$

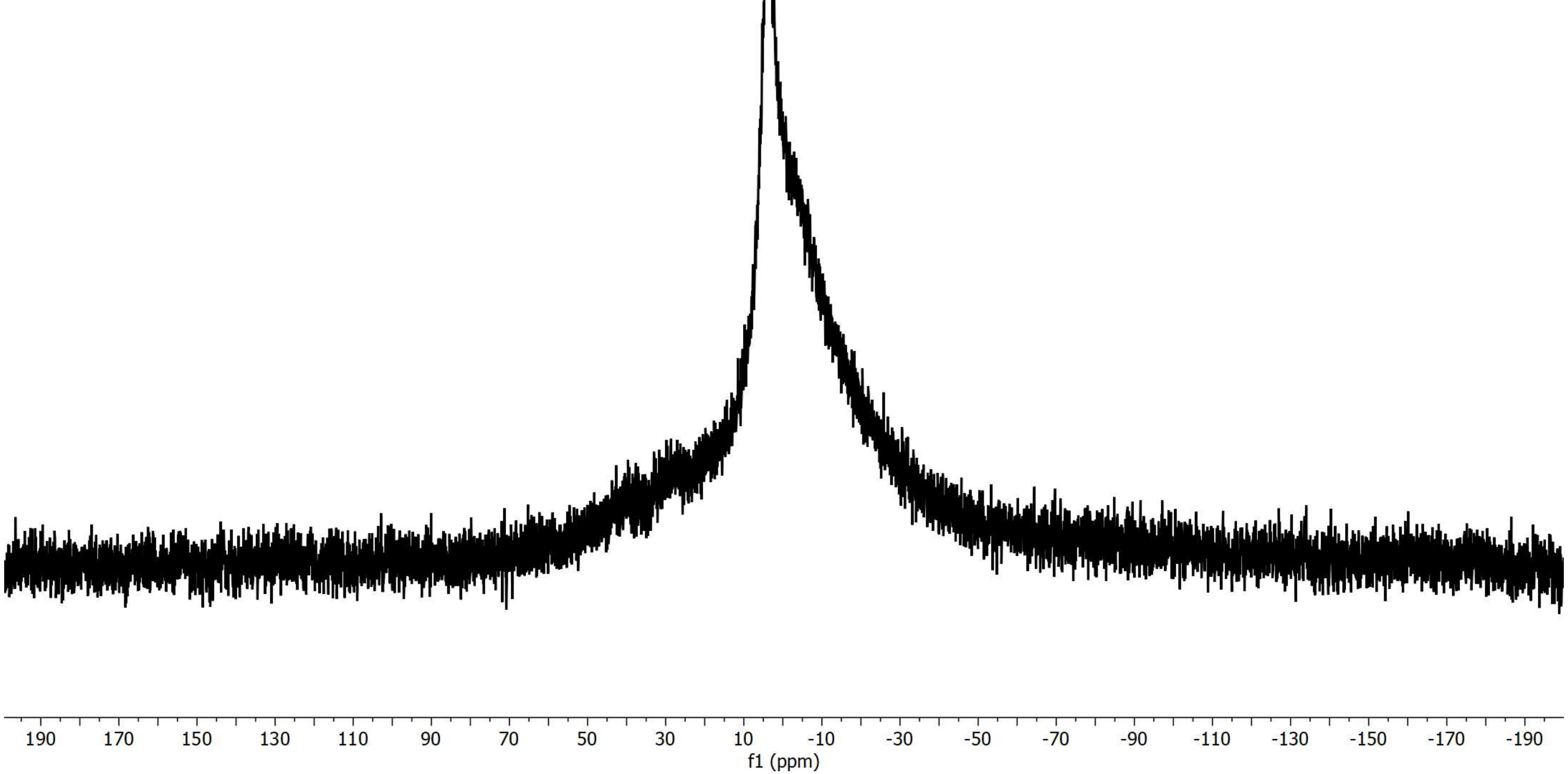

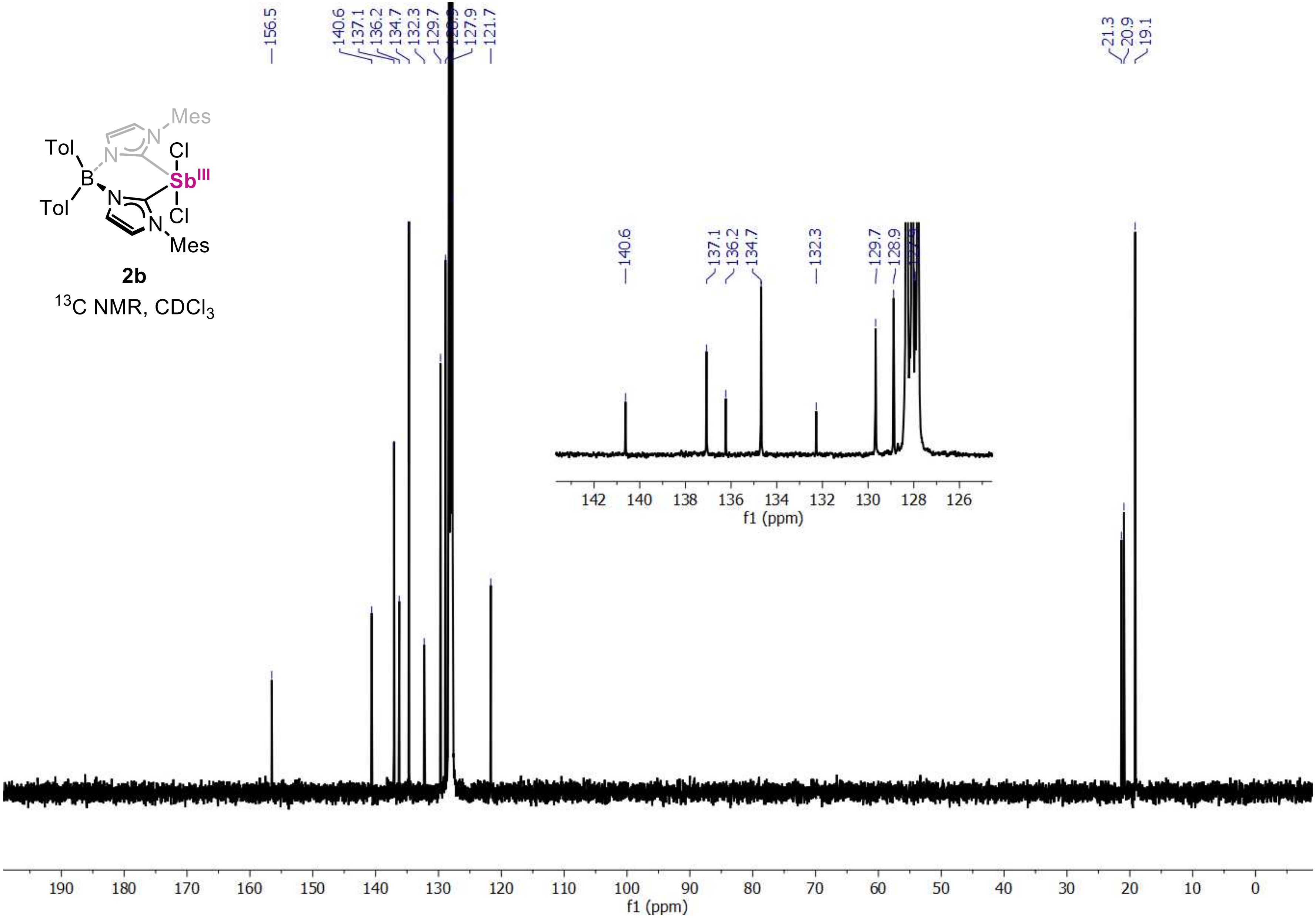


**2b**

$^{13}$C NMR, $CDCl_3$

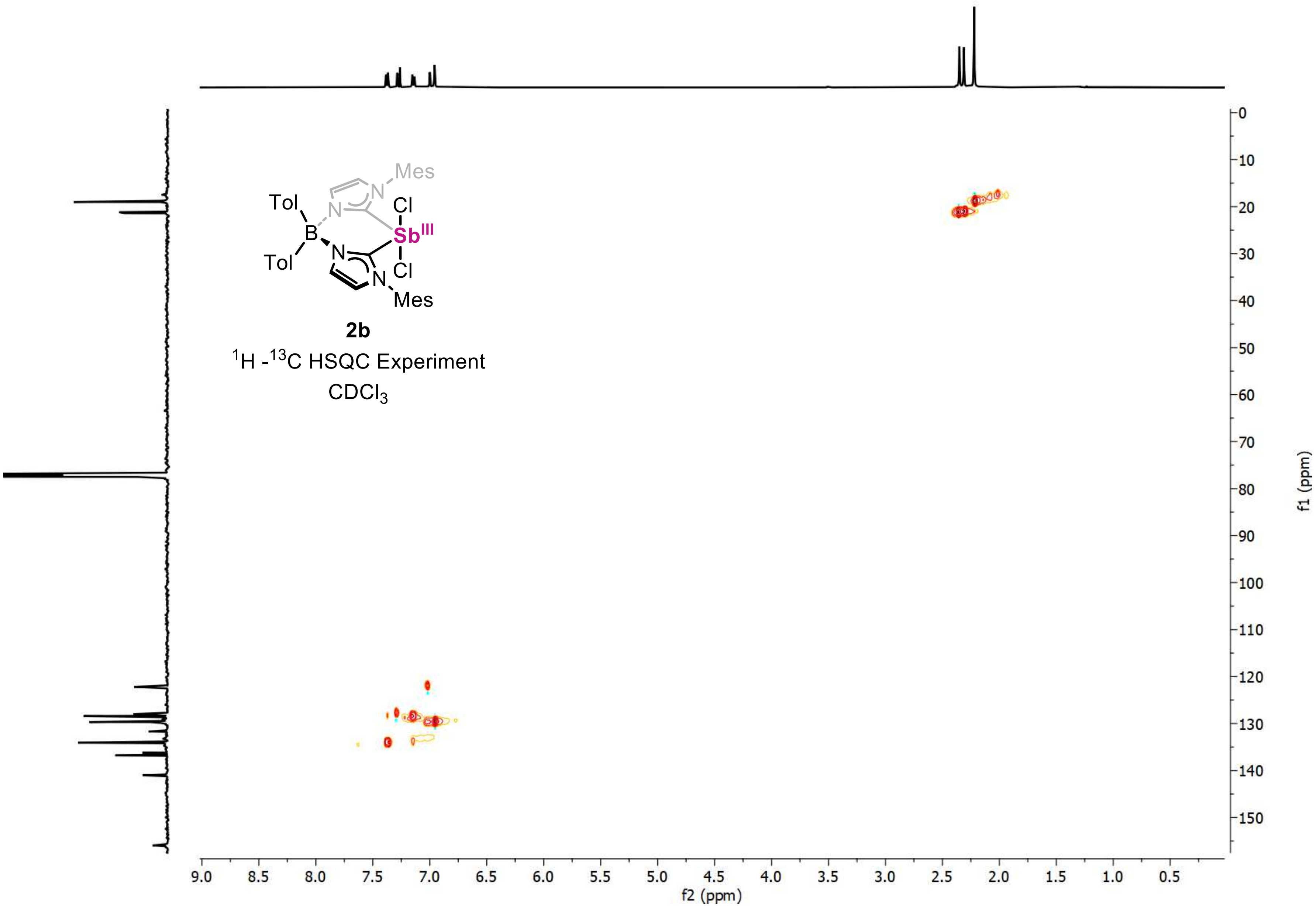
Tol
Tol
B
N
N
Mes
Cl
Sb^III
Cl
N
N
Mes
2b
$^{1}$H -$^{13}$C HSQC Experiment
$CDCl_3$
f1 (ppm)
f2 (ppm)
9.0 8.5 8.0 7.5 7.0 6.5 6.0 5.5 5.0 4.5 4.0 3.5 3.0 2.5 2.0 1.5 1.0 0.5
0 10 20 30 40 50 60 70 80 90 100 110 120 130 140 150

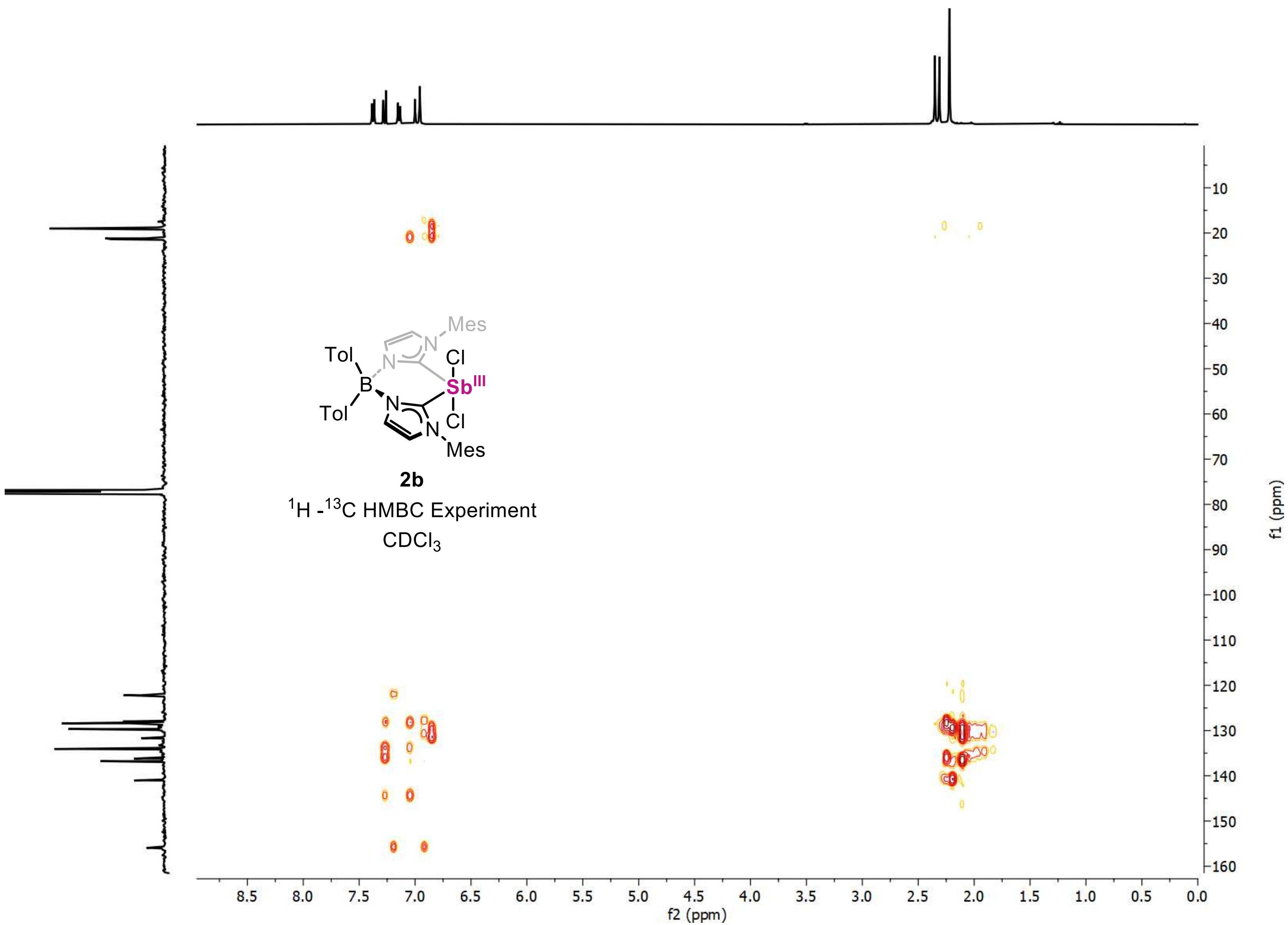


**2b**

$^1$H -$^{13}$C HMBC Experiment

$CDCl_3$

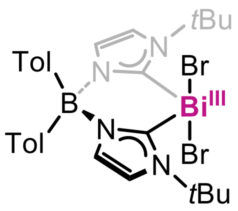


**3a**

$^{1}$H NMR, $CDCl_3$

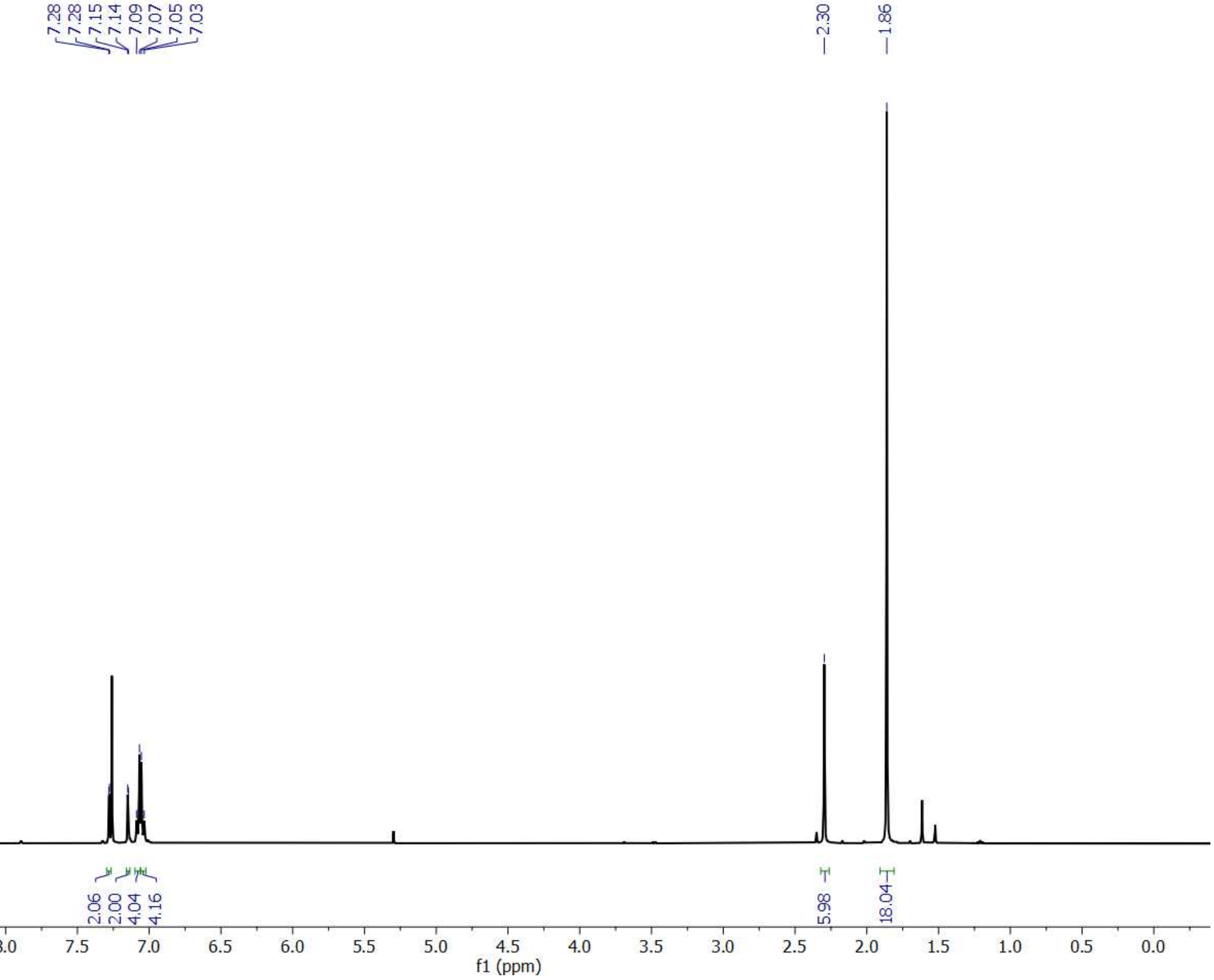

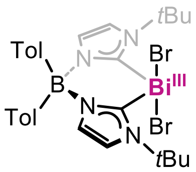


**3a**

$^{11}B$ NMR, $CDCl_3$

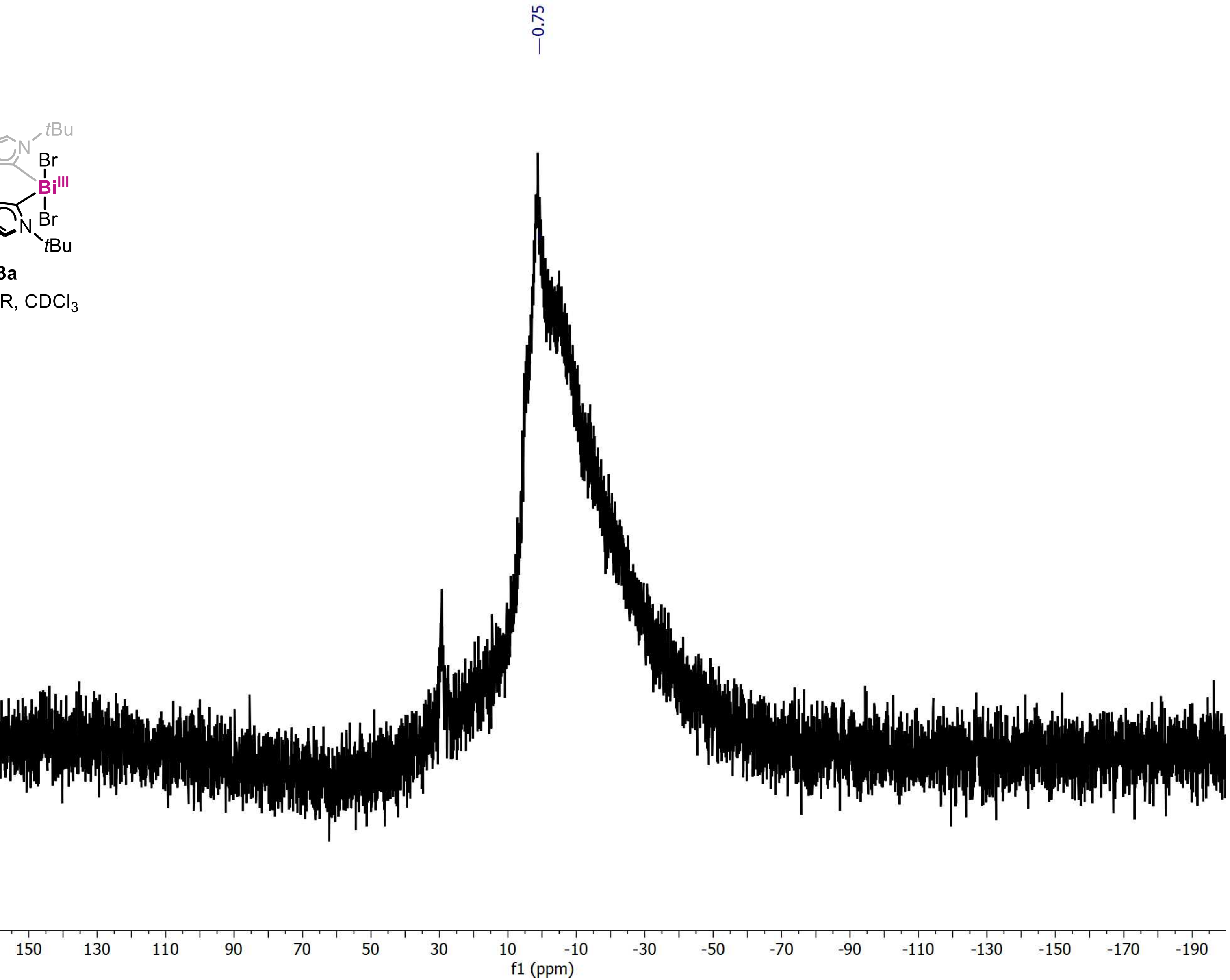

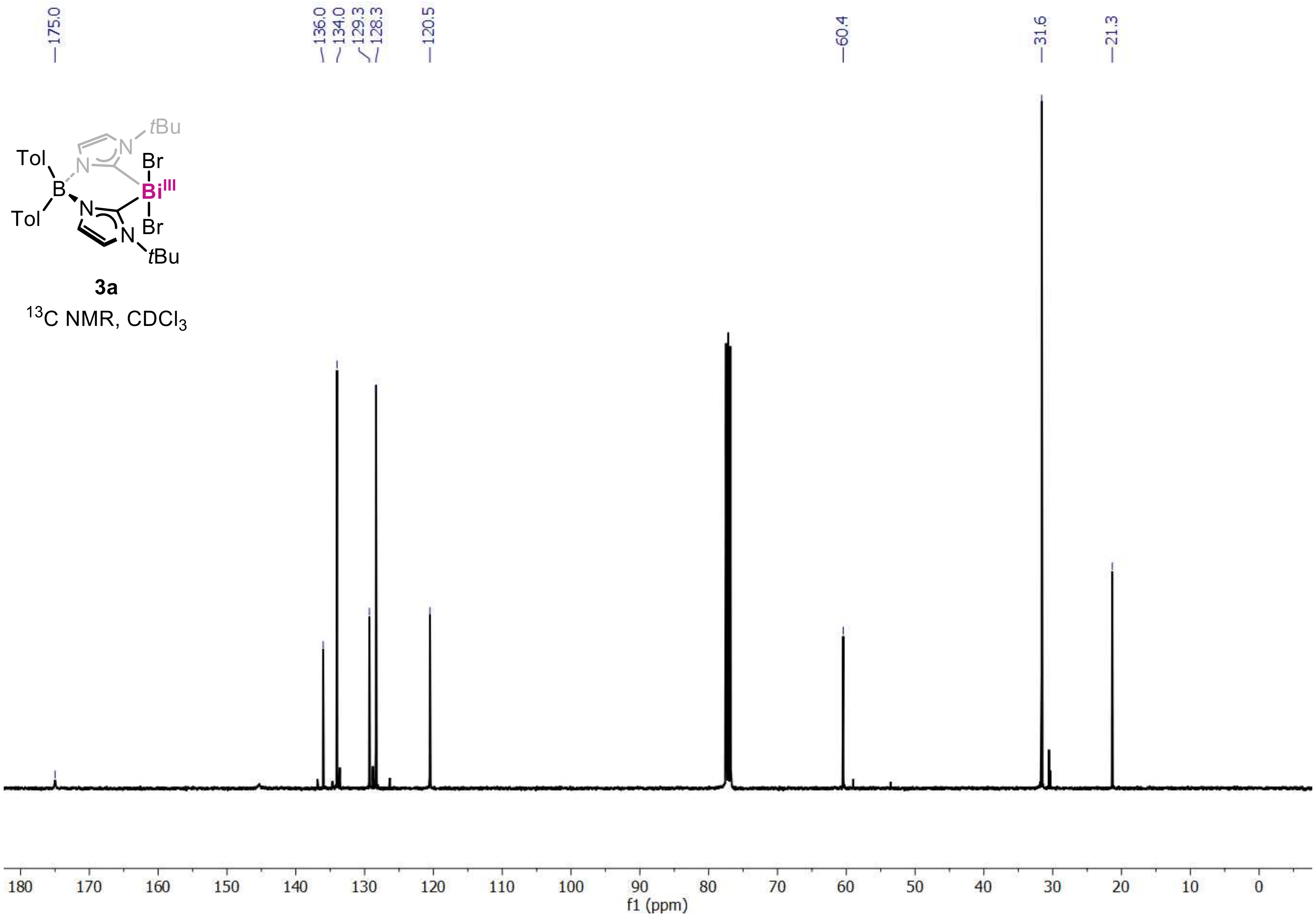

175.0
136.0
134.0
129.3
128.3
120.5
60.4
31.6
21.3
Tol
Tol
B
N
N
tBu
tBu
Br
Br
Bi^III
3a
13C NMR, CDCl3
180 170 160 150 140 130 120 110 100 90 80 70 60 50 40 30 20 10 0
f1 (ppm)

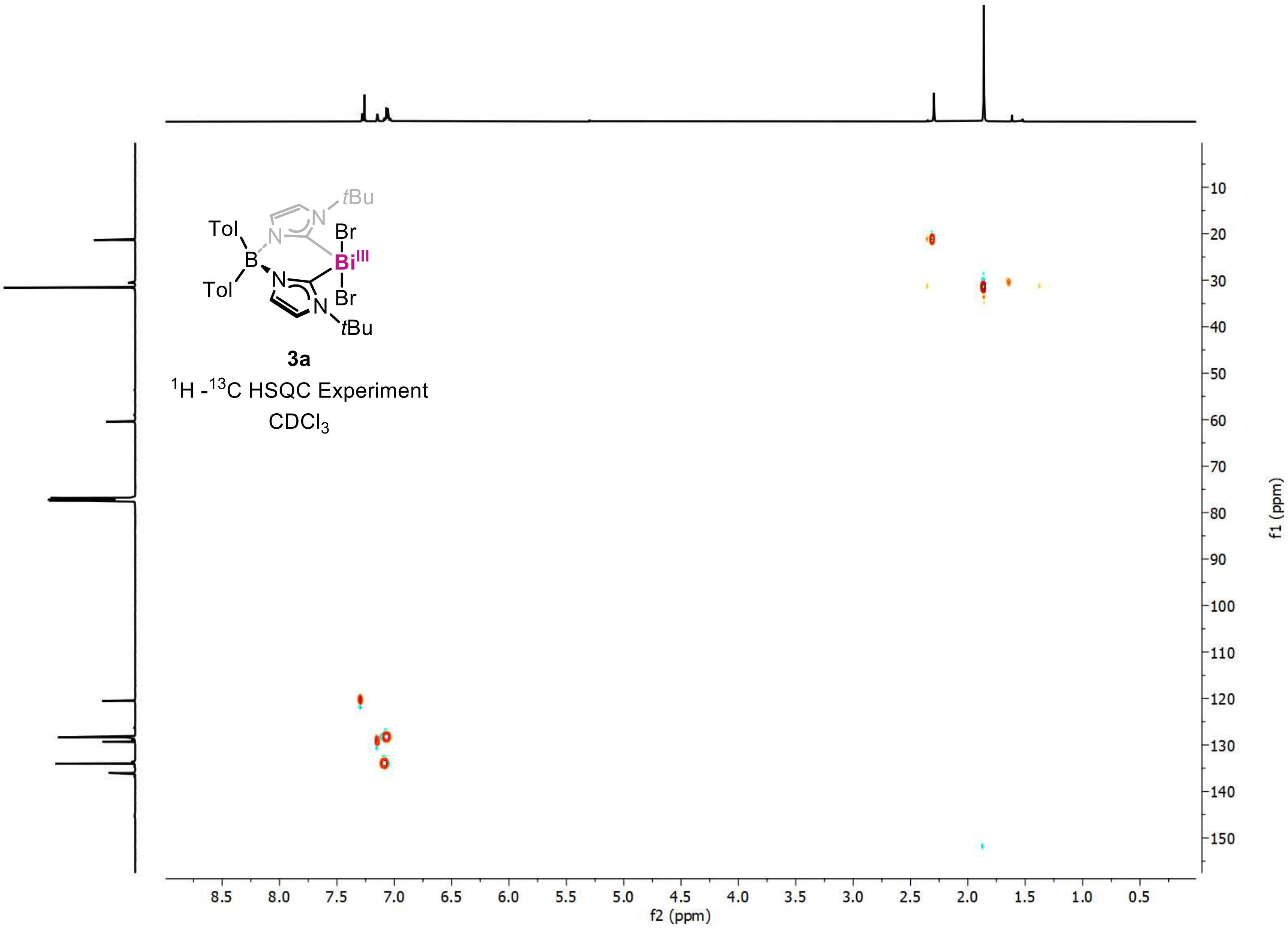

tBu
Tol
N
N
Br
B
Bi^III
Tol
N
N
Br
tBu
3a
¹H -¹³C HSQC Experiment
CDCl₃
f1 (ppm)
f2 (ppm)

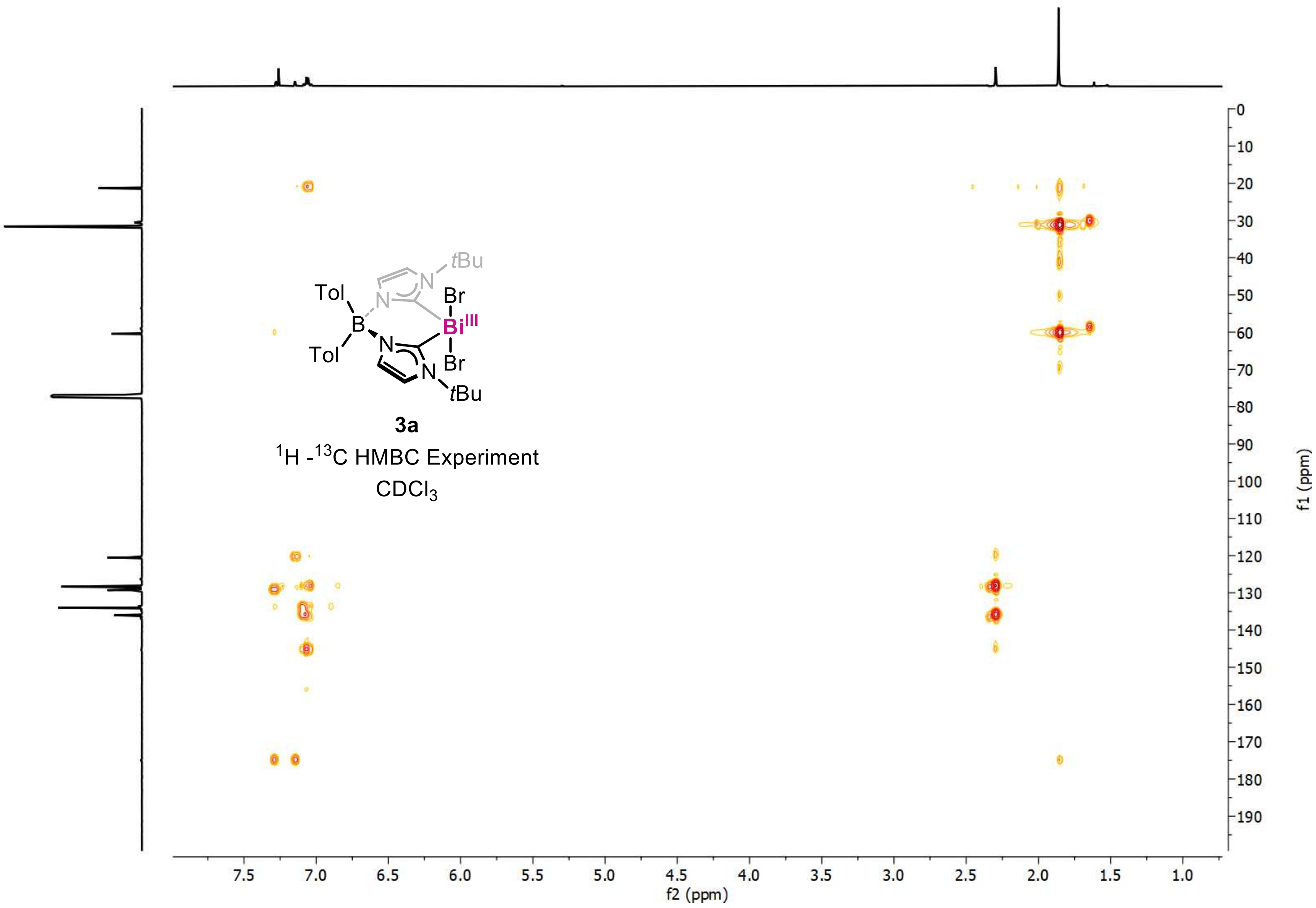
tBu
Tol
N
N
Br
B
Bi
III
Tol
N
Br
N
tBu
3a
$^{1}$H -$^{13}$C HMBC Experiment
CDCl$_3$
f1 (ppm)
f2 (ppm)
0
10
20
30
40
50
60
70
80
90
100
110
120
130
140
150
160
170
180
190
7.5
7.0
6.5
6.0
5.5
5.0
4.5
4.0
3.5
3.0
2.5
2.0
1.5
1.0

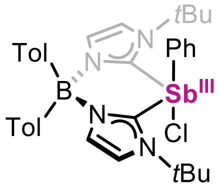


**11b**

$^{1}$H NMR, $CDCl_3$

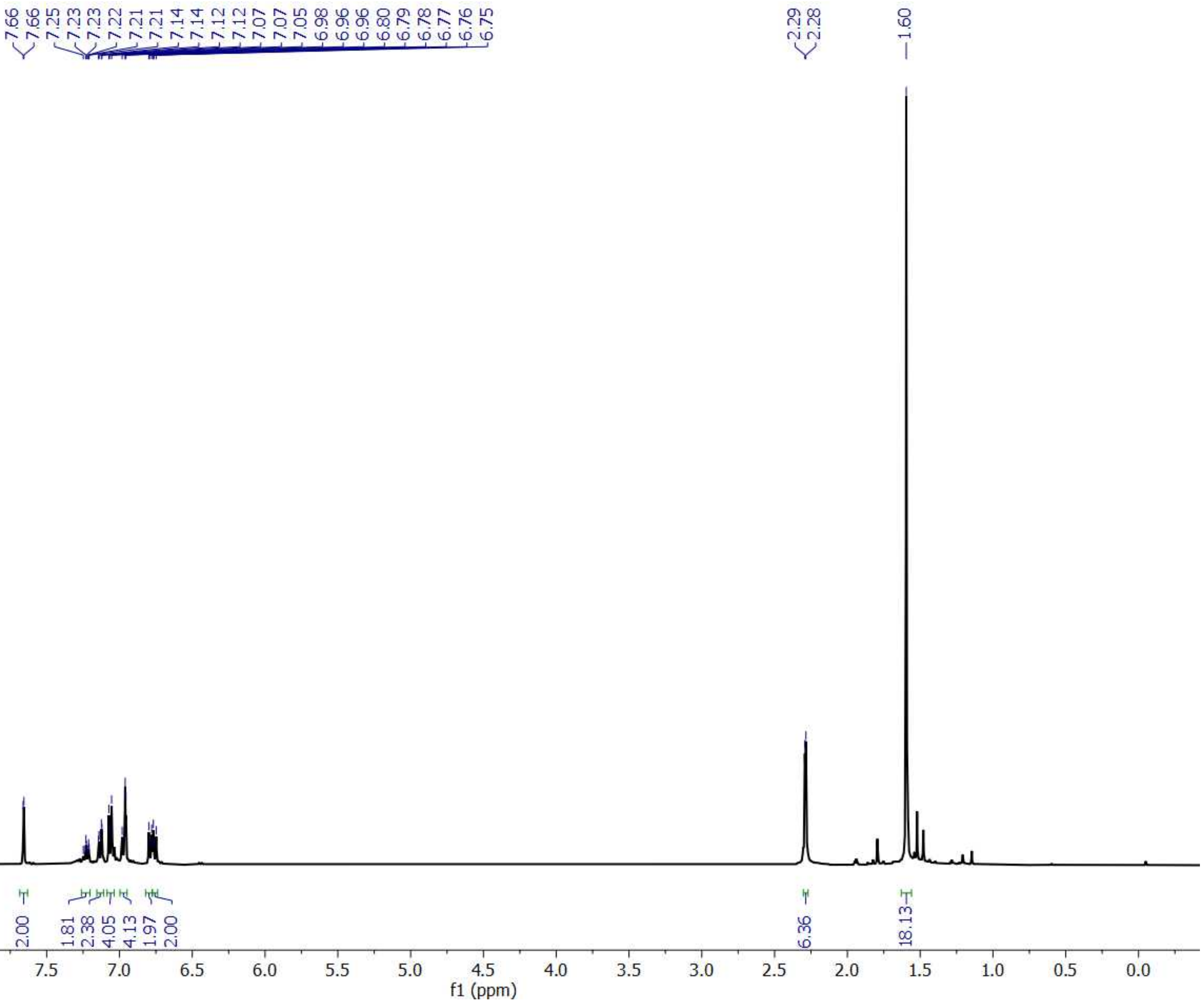

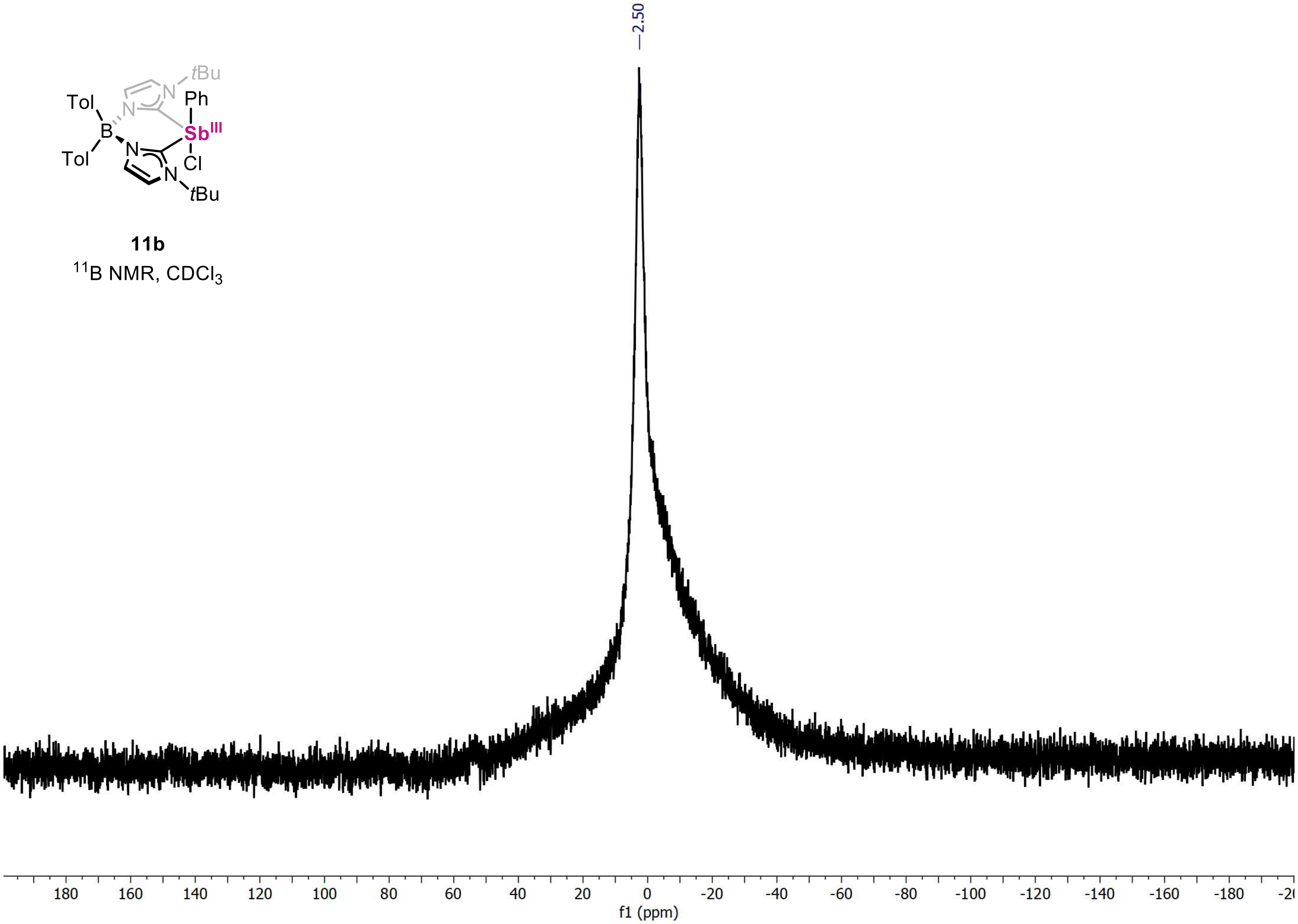


**11b**

$^{11}$B NMR, $CDCl_3$

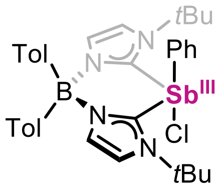


**11b**

$^{13}C$ NMR, $CDCl_3$

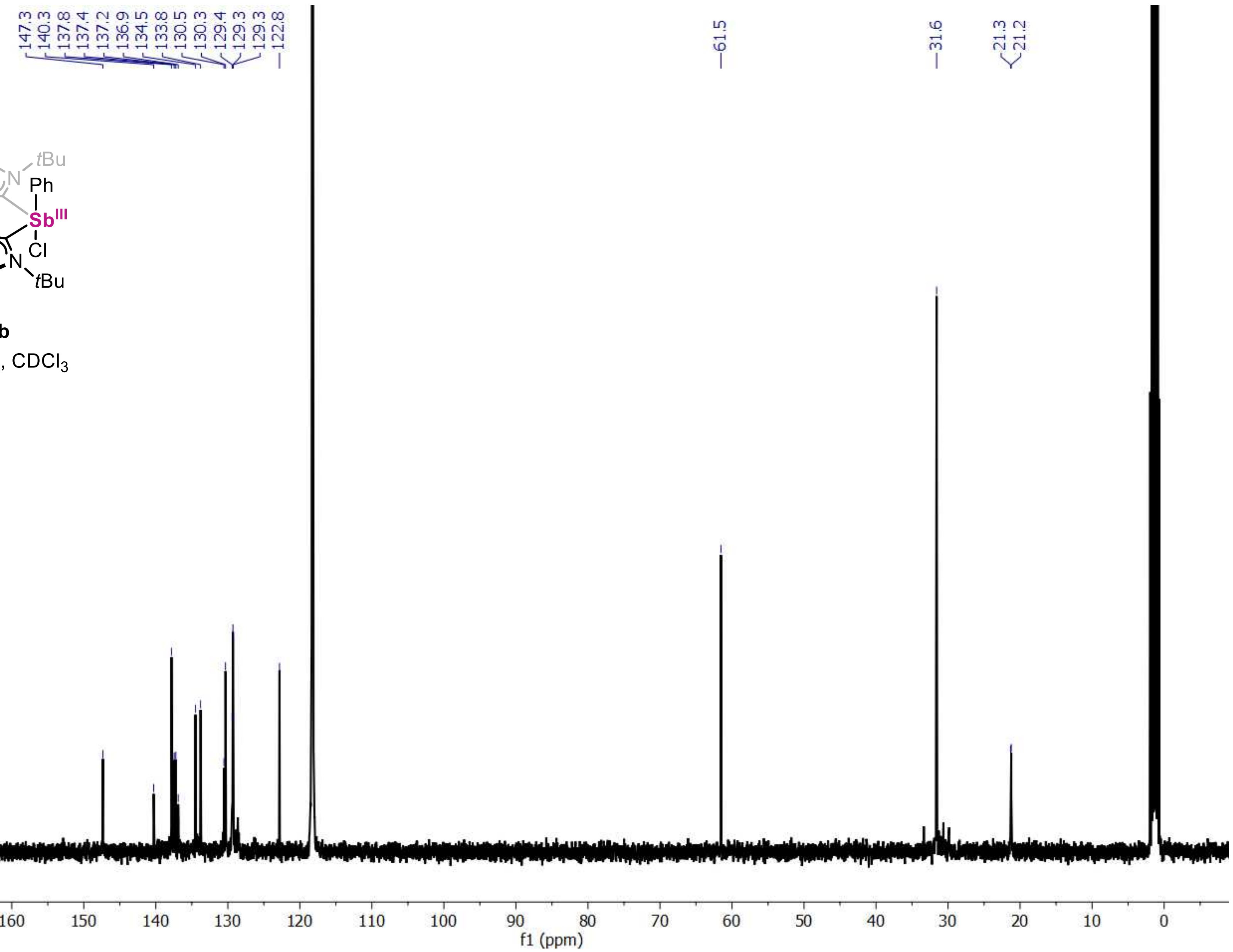

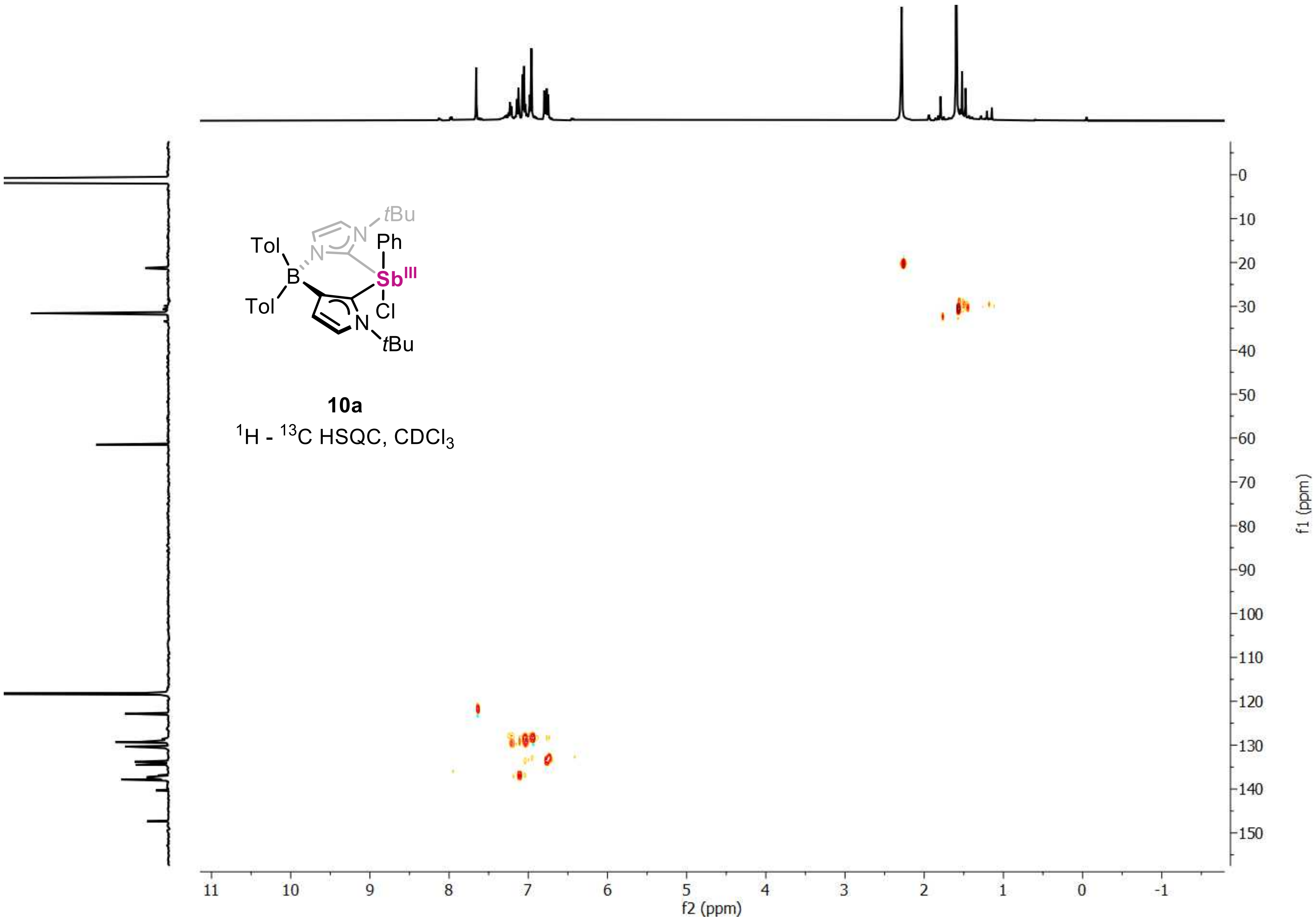
Tol
Tol
B
N
N
tBu
Ph
Sb^III
Cl
N
tBu
10a
1H - 13C HSQC, CDCl3
f2 (ppm)
f1 (ppm)

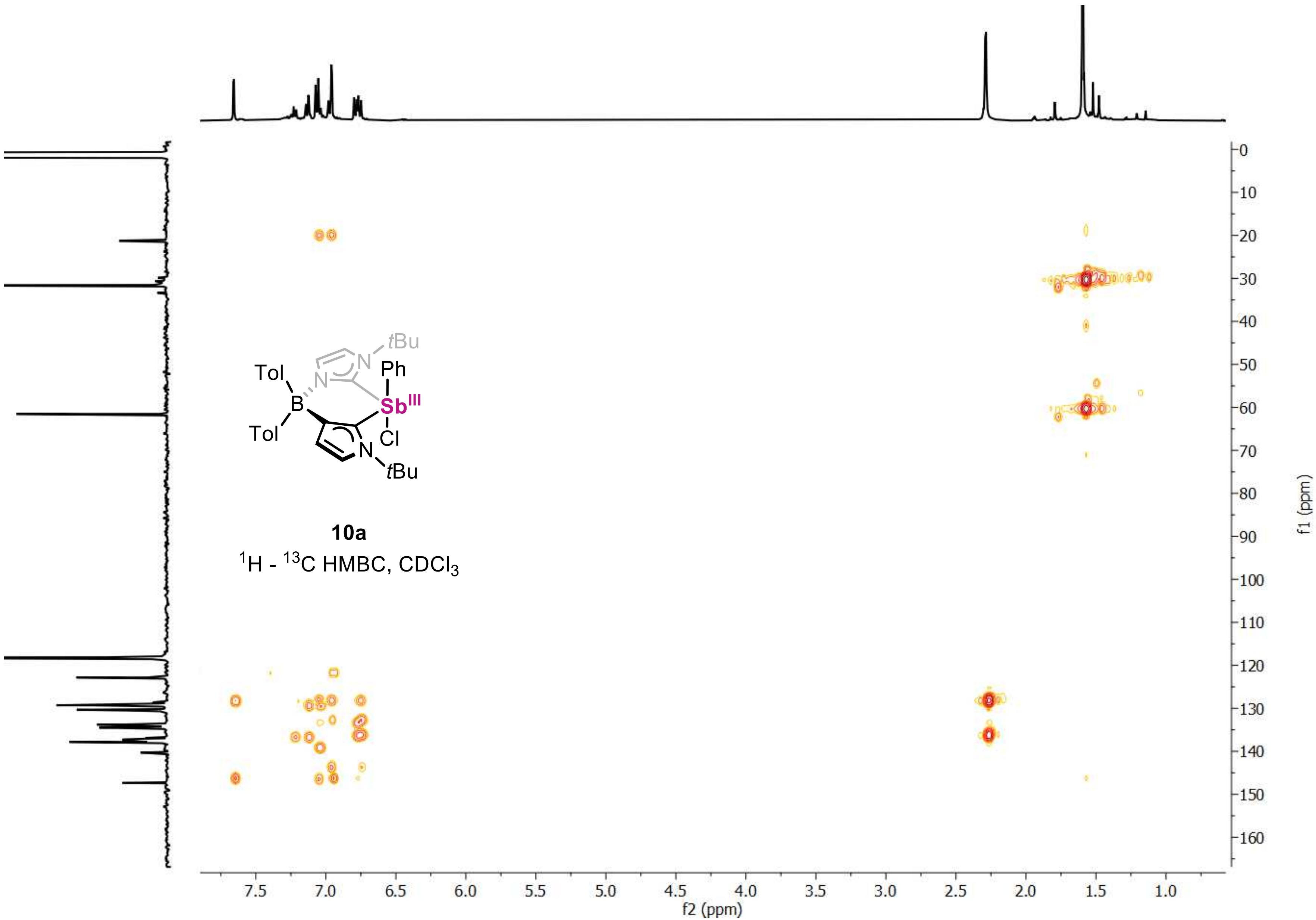

Tol
Tol
B
N
N
tBu
Ph
Sb^III
Cl
N
tBu
10a
1H - 13C HMBC, CDCl3
f1 (ppm)
f2 (ppm)

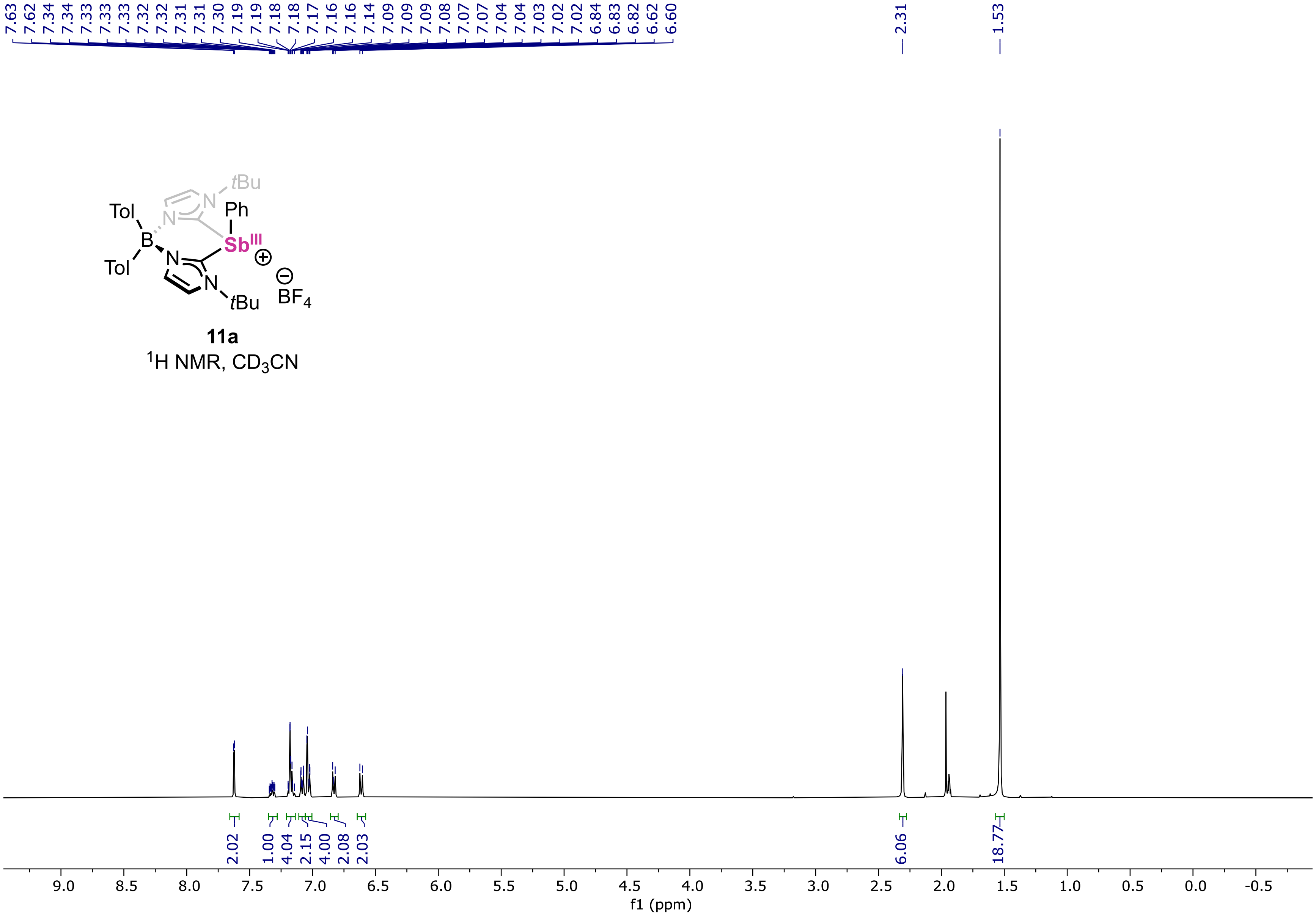
7.63
7.62
7.34
7.34
7.33
7.33
7.33
7.32
7.32
7.31
7.31
7.30
7.19
7.19
7.18
7.18
7.17
7.16
7.16
7.14
7.09
7.09
7.09
7.08
7.07
7.07
7.04
7.04
7.03
7.02
7.02
6.84
6.83
6.82
6.62
6.60
2.31
1.53
tBu
Tol
Ph
B
N
Sb III
Tol
N
N
tBu
BF4
11a
1H NMR, CD3CN
2.02
1.00
4.04
2.15
4.00
2.08
2.03
6.06
18.77
9.0 8.5 8.0 7.5 7.0 6.5 6.0 5.5 5.0 4.5 4.0 3.5 3.0 2.5 2.0 1.5 1.0 0.5 0.0 -0.5
f1 (ppm)

**11a**

$^{19}F$ NMR, $CD_3CN$

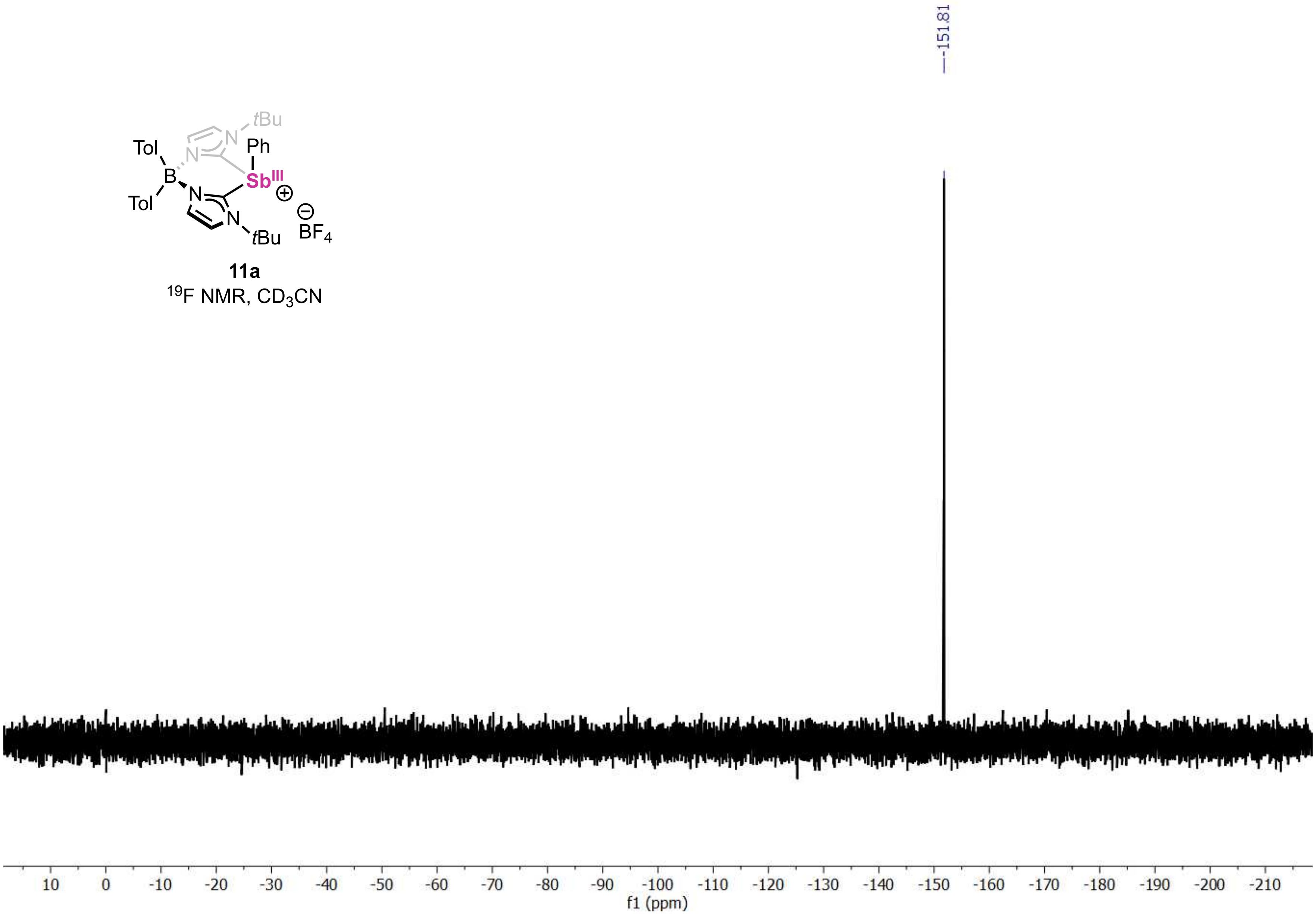

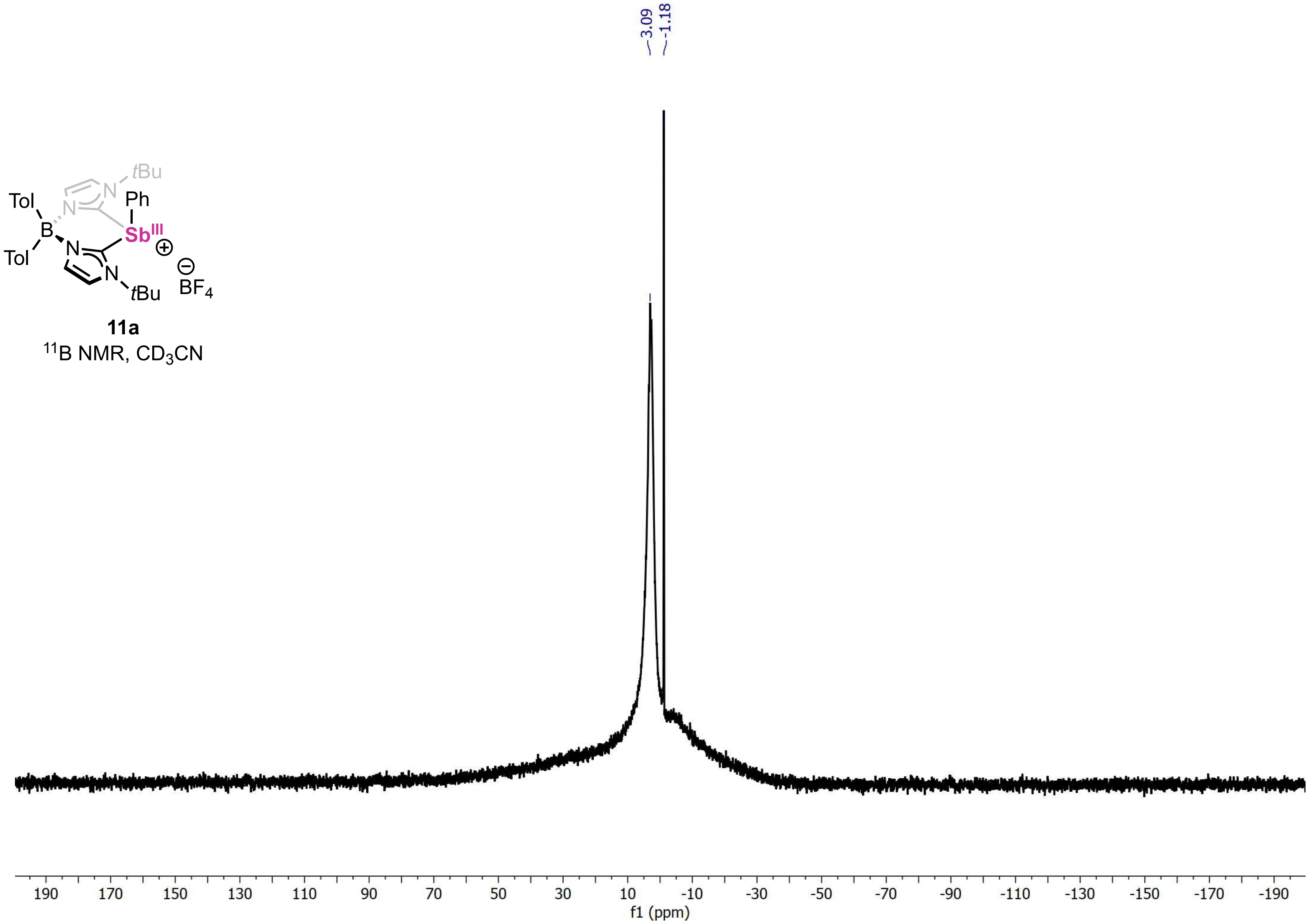


**11a**

$^{11}B$ NMR, $CD_3CN$

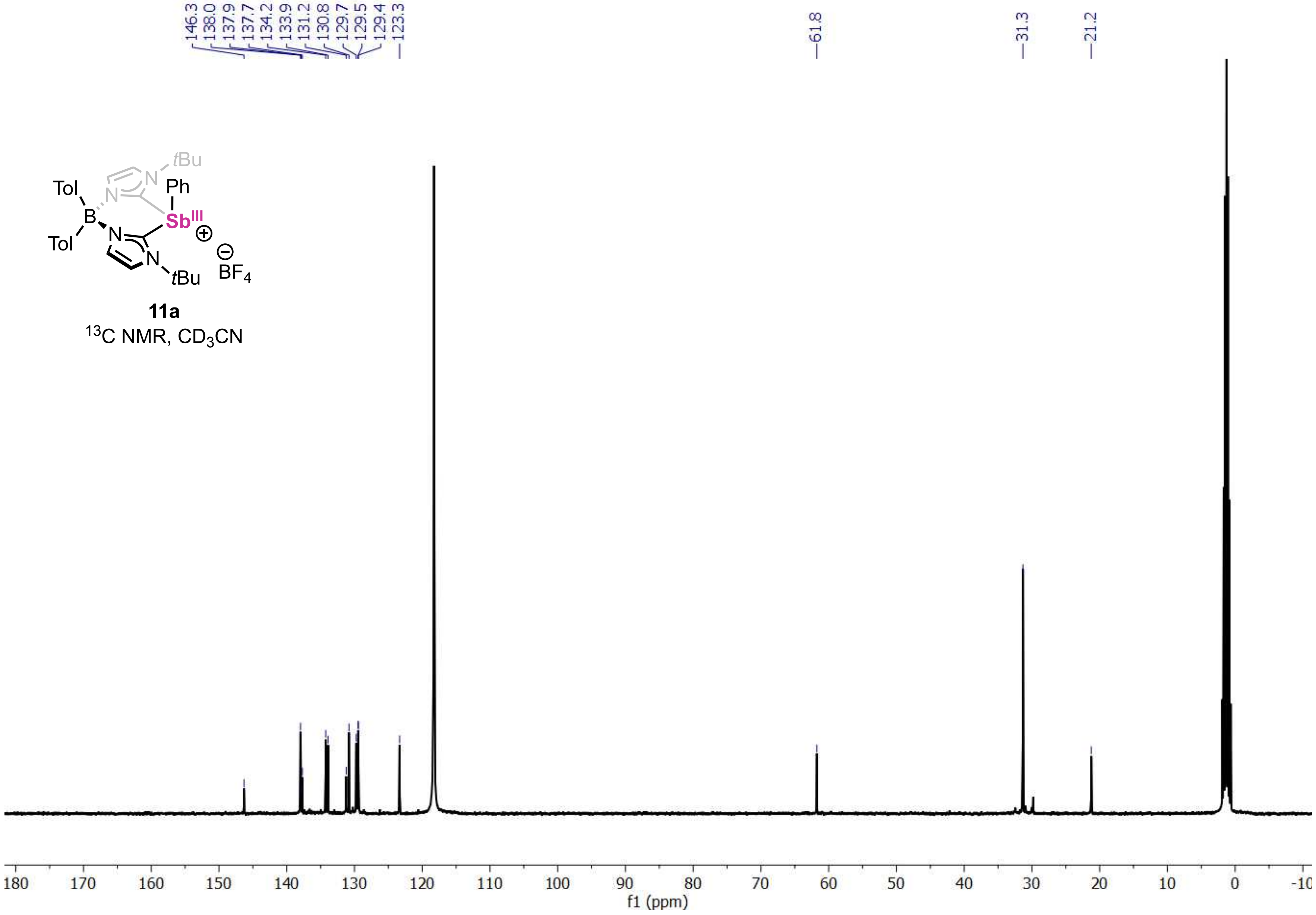

146.3
138.0
137.9
137.7
134.2
133.9
131.2
130.8
129.7
129.5
129.4
123.3
61.8
31.3
21.2
tBu
Tol
Ph
B
Sb
III
Tol
N
tBu
BF4
11a
13C NMR, CD3CN
180
170
160
150
140
130
120
110
100
90
80
70
60
50
40
30
20
10
0
-10
f1 (ppm)

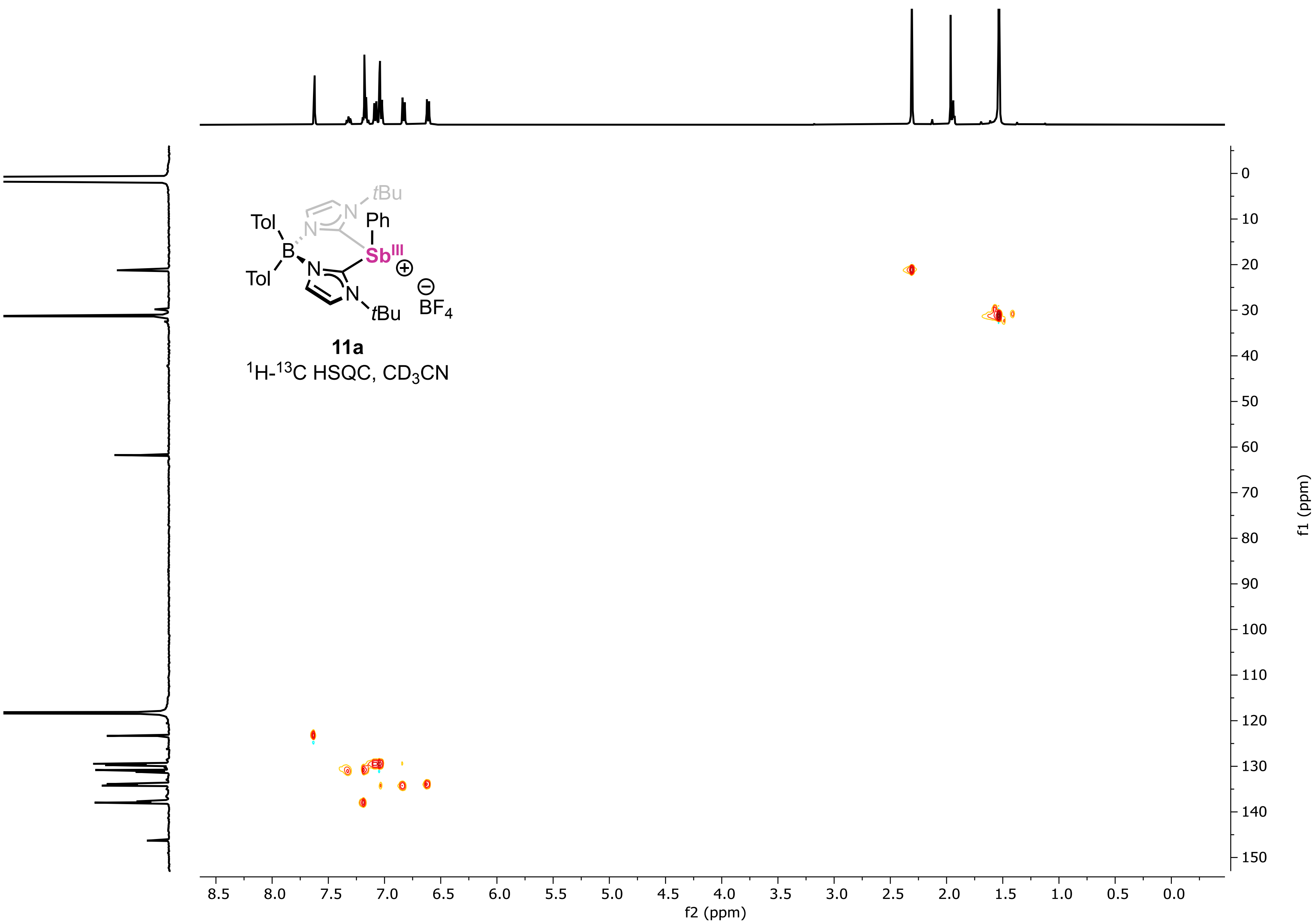

Tol
Tol
B
N
N
N
N
tBu
tBu
Ph
Sb^III
BF4
11a
1H-13C HSQC, CD3CN
f2 (ppm)
f1 (ppm)

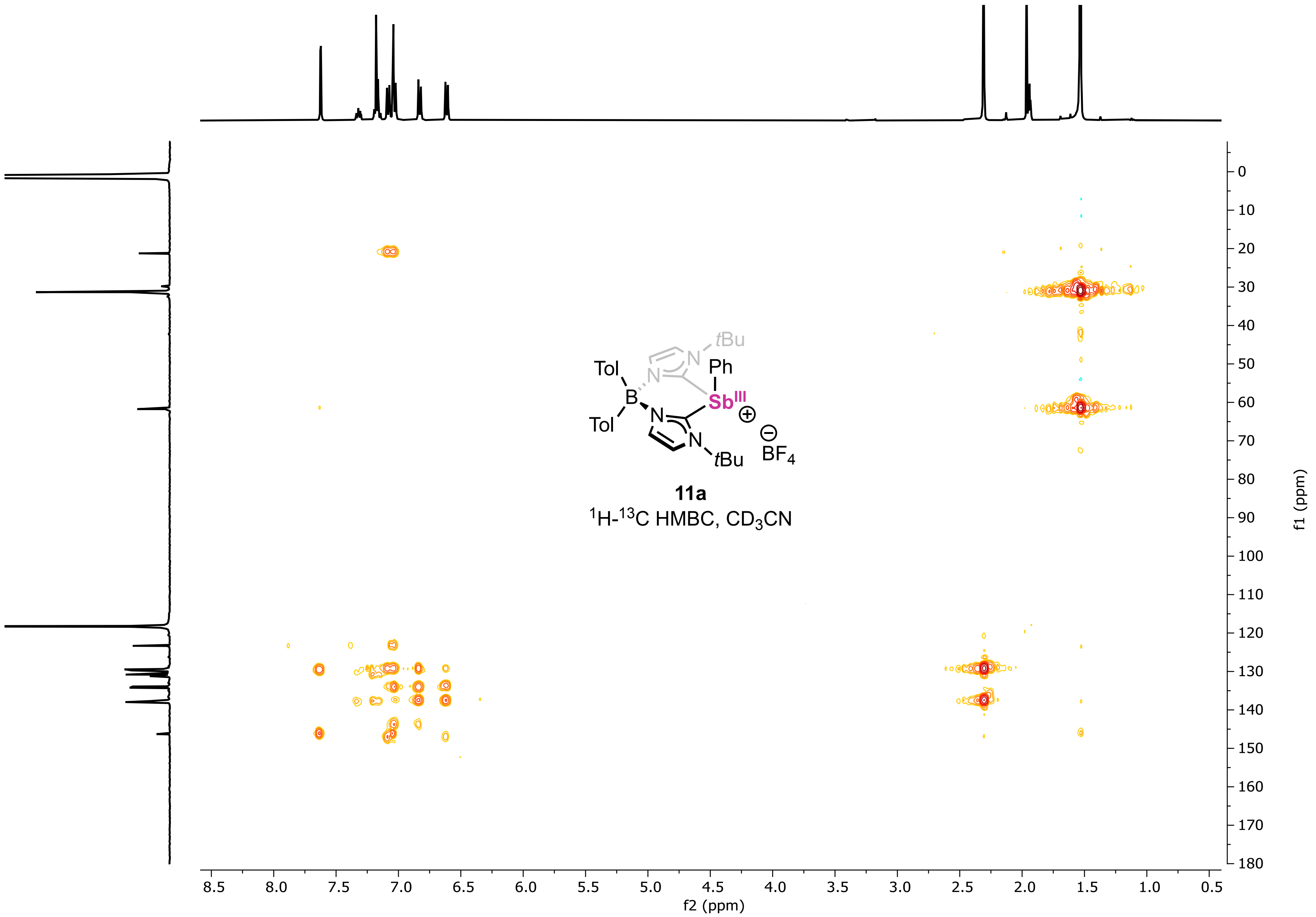

tBu
Tol
Ph
N
B
N
Sb$^{III}$
Tol
N
N
tBu
$BF_4$
**11a**
$^1H$-$^{13}C$ HMBC, $CD_3CN$
f1 (ppm)
f2 (ppm)

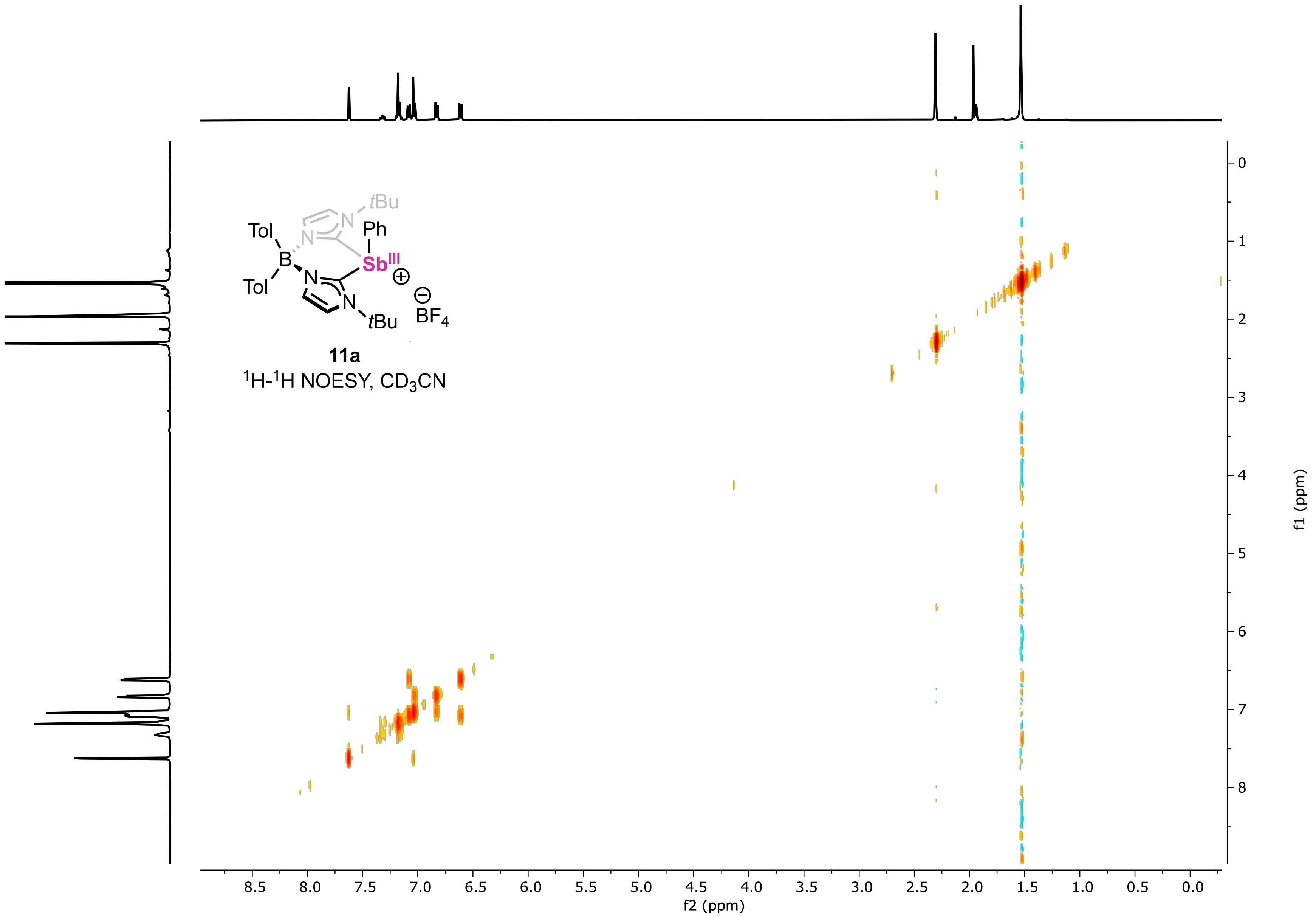
Tol
Tol
B
N
N
tBu
Ph
Sb^III
BF4
11a
1H-1H NOESY, CD3CN
f2 (ppm)
f1 (ppm)

## 11.4 NMR characterisation of Pn(I) complexes

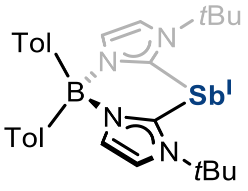


**4a**

$^1$H NMR, benzene-$d_6$

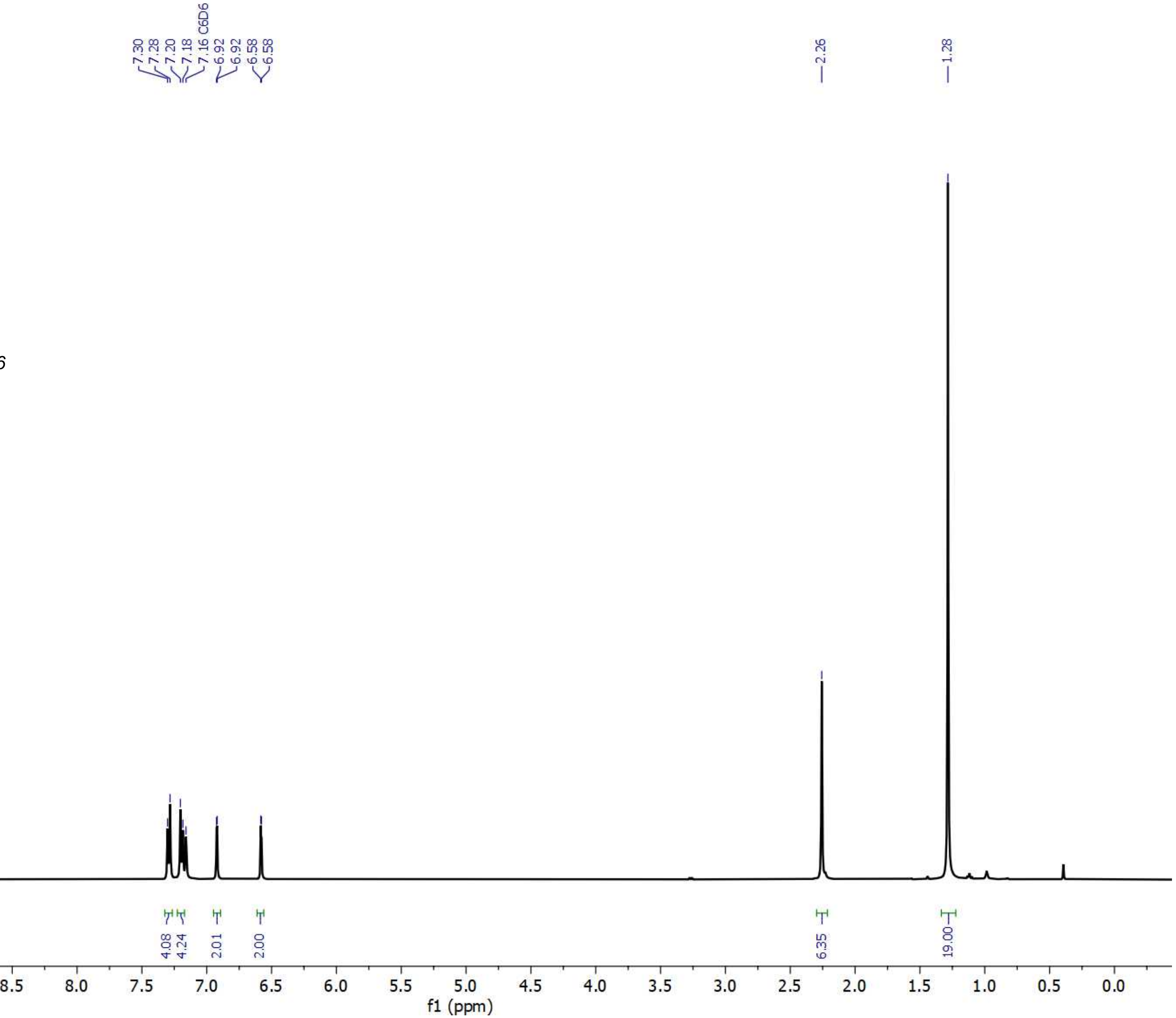

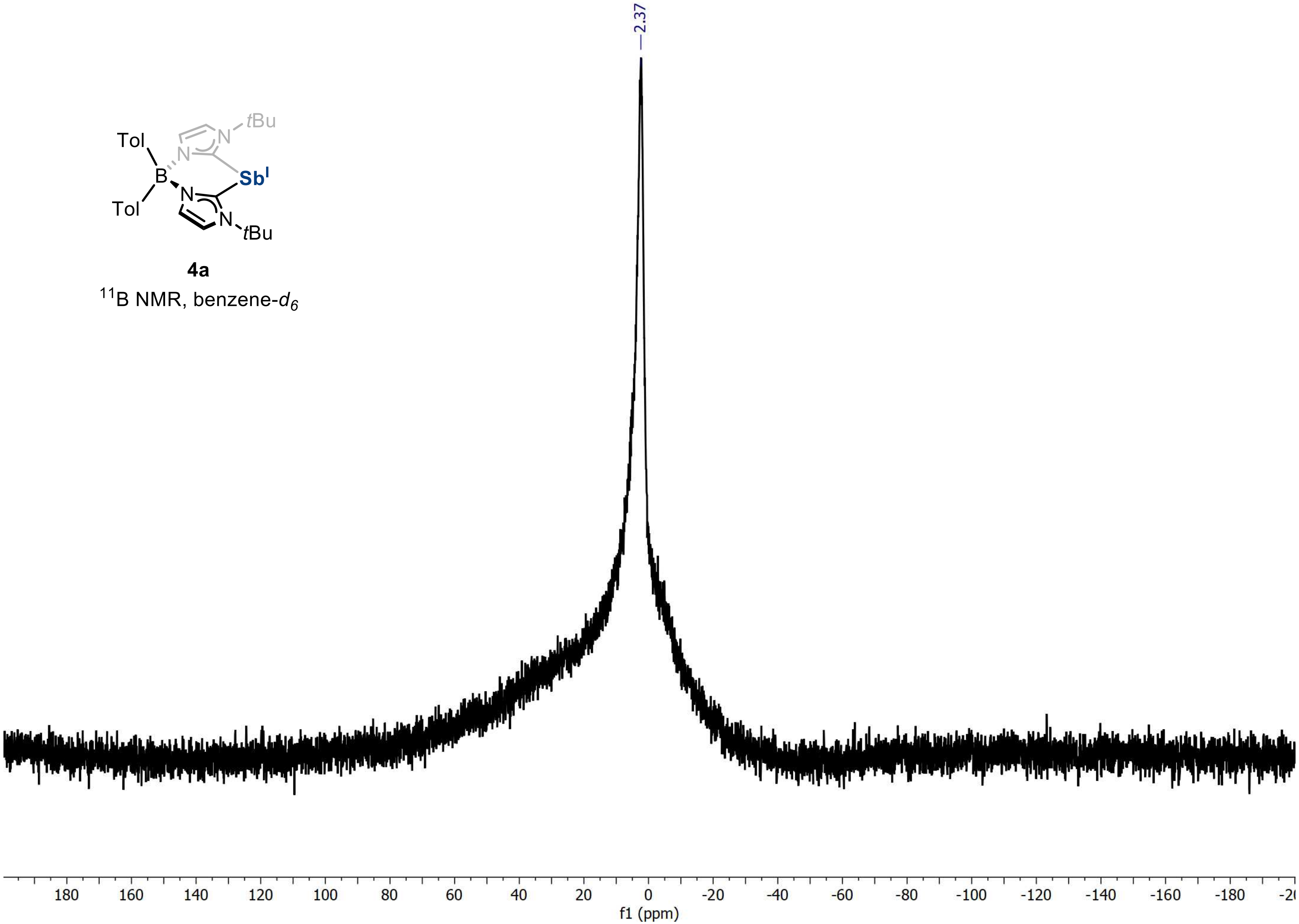


**4a**

$^{11}$B NMR, benzene-$d_6$

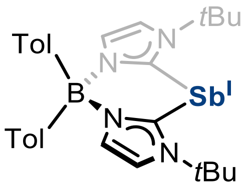

**4a**

$^{13}$C NMR, benzene-$d_6$

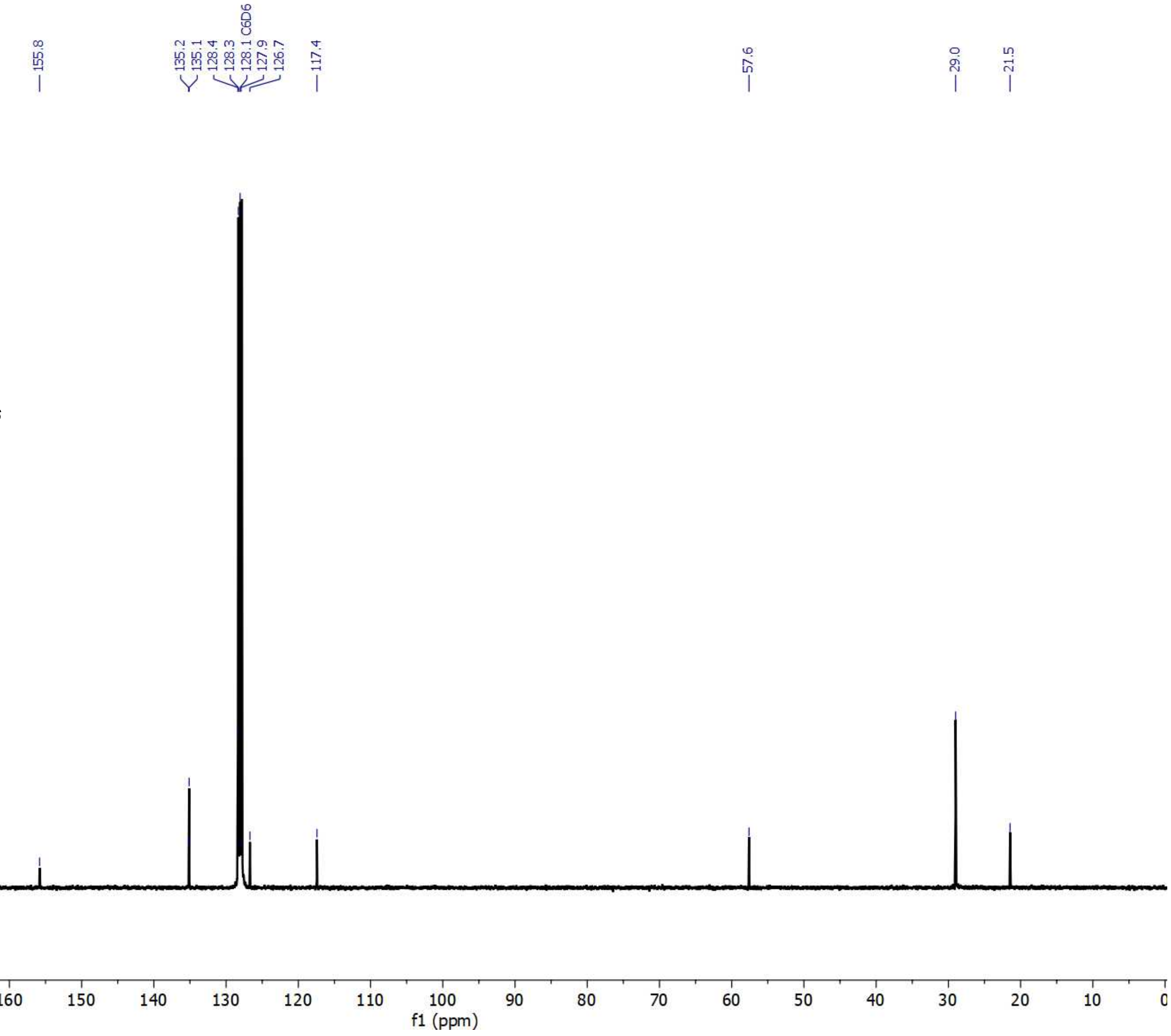

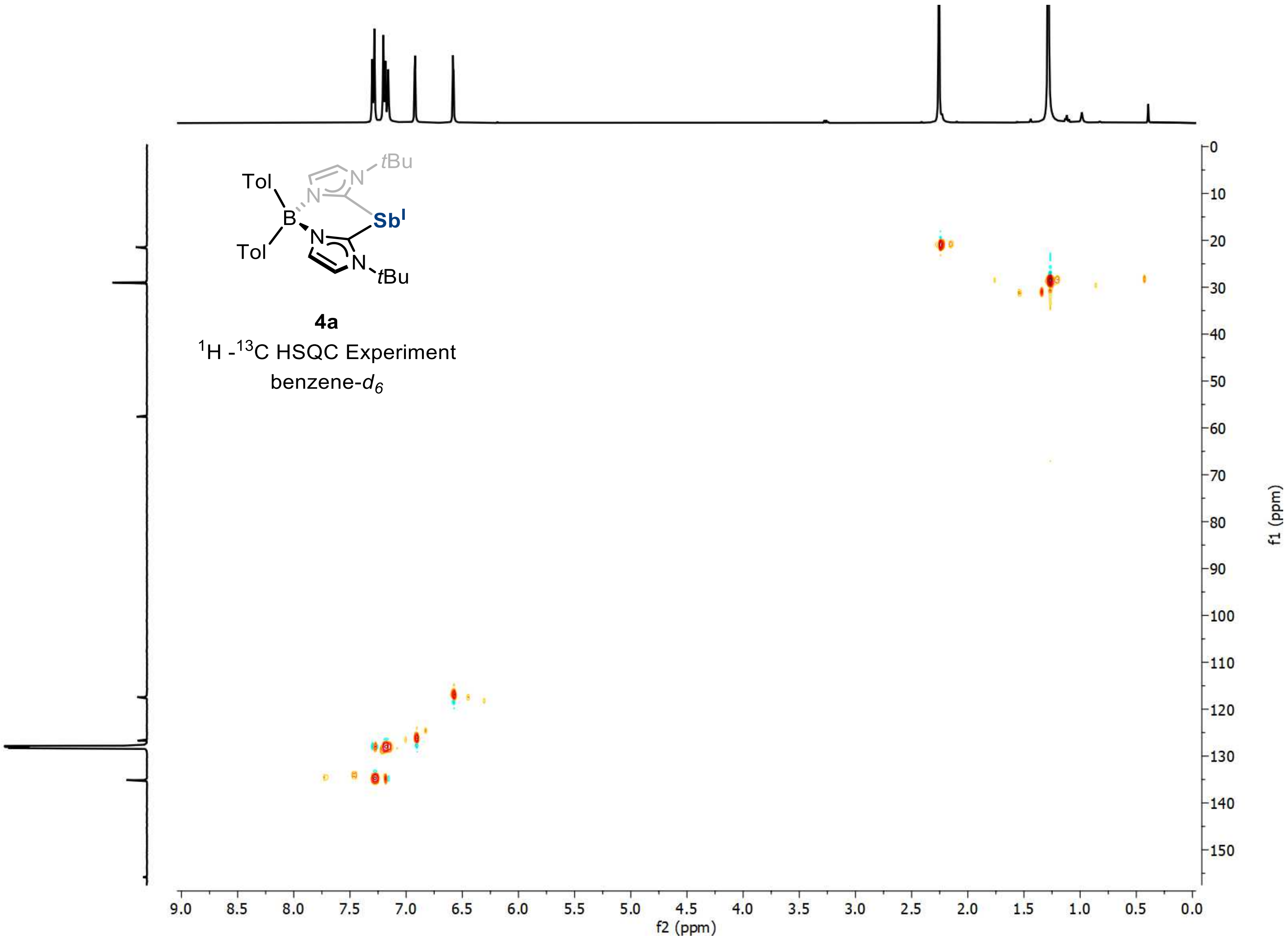


**4a**

$^1$H - $^{13}$C HSQC Experiment

benzene-$d_6$

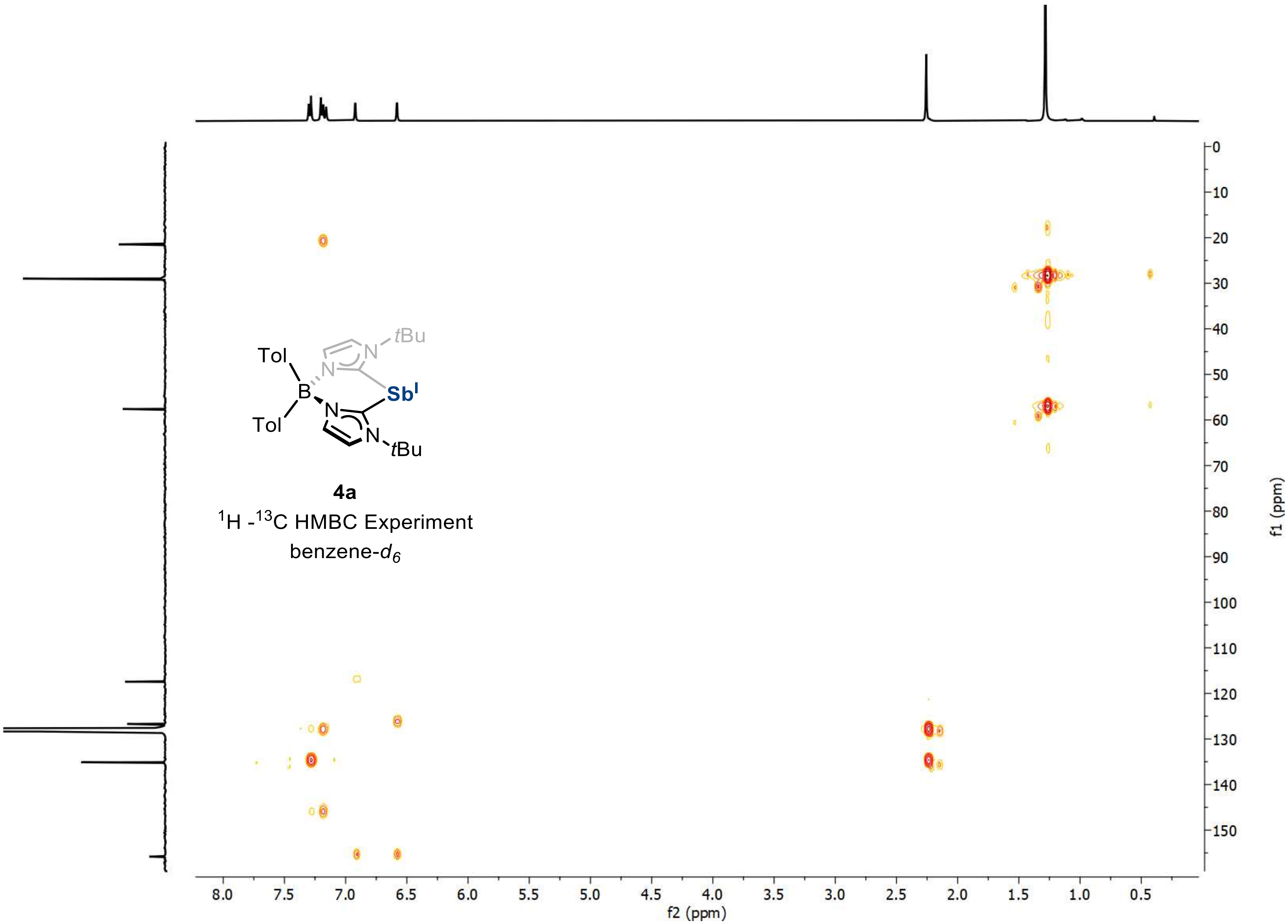

Tol
B
Tol
N
N
tBu
Sb^I
N
N
tBu
4a
1H -13C HMBC Experiment
benzene-d6
f2 (ppm)
f1 (ppm)

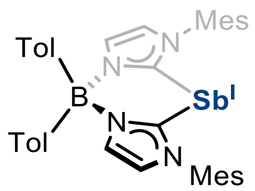


**4b**

$^{1}$H NMR, benzene-$d_6$

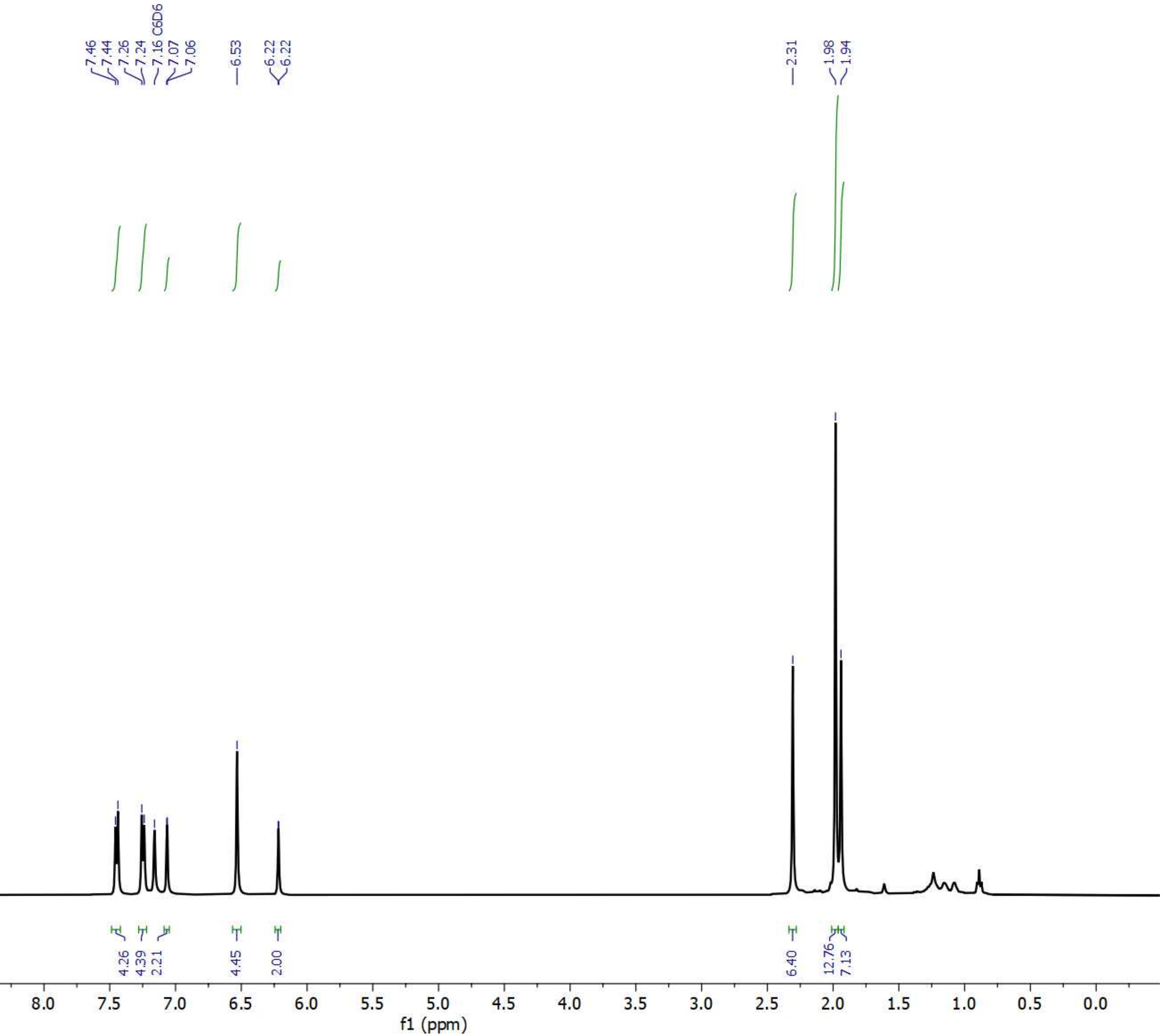

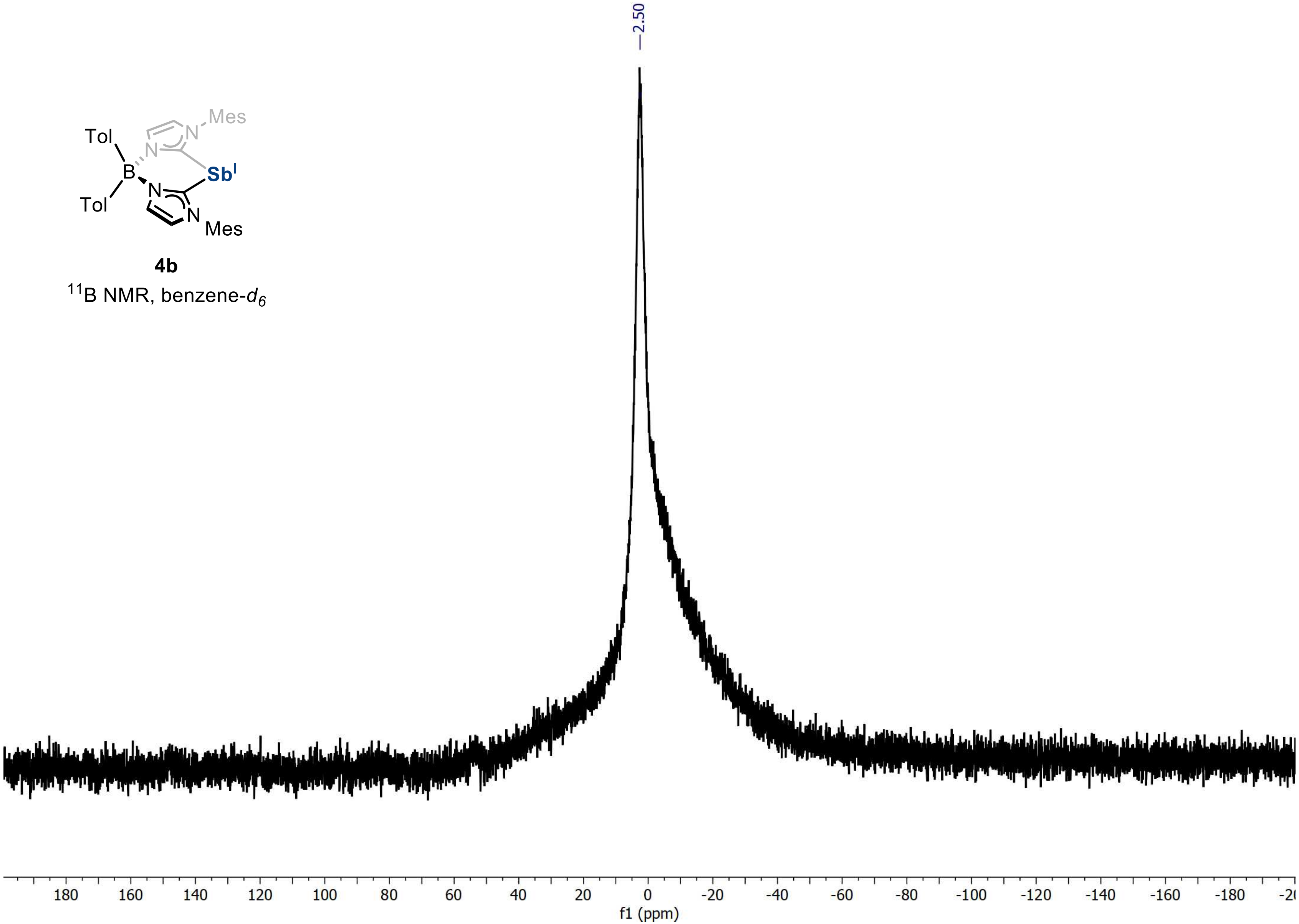


**4b**

$^{11}$B NMR, benzene-$d_6$

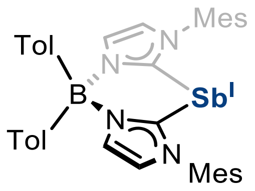


**4b**

$^{13}$C NMR, benzene-$d_6$

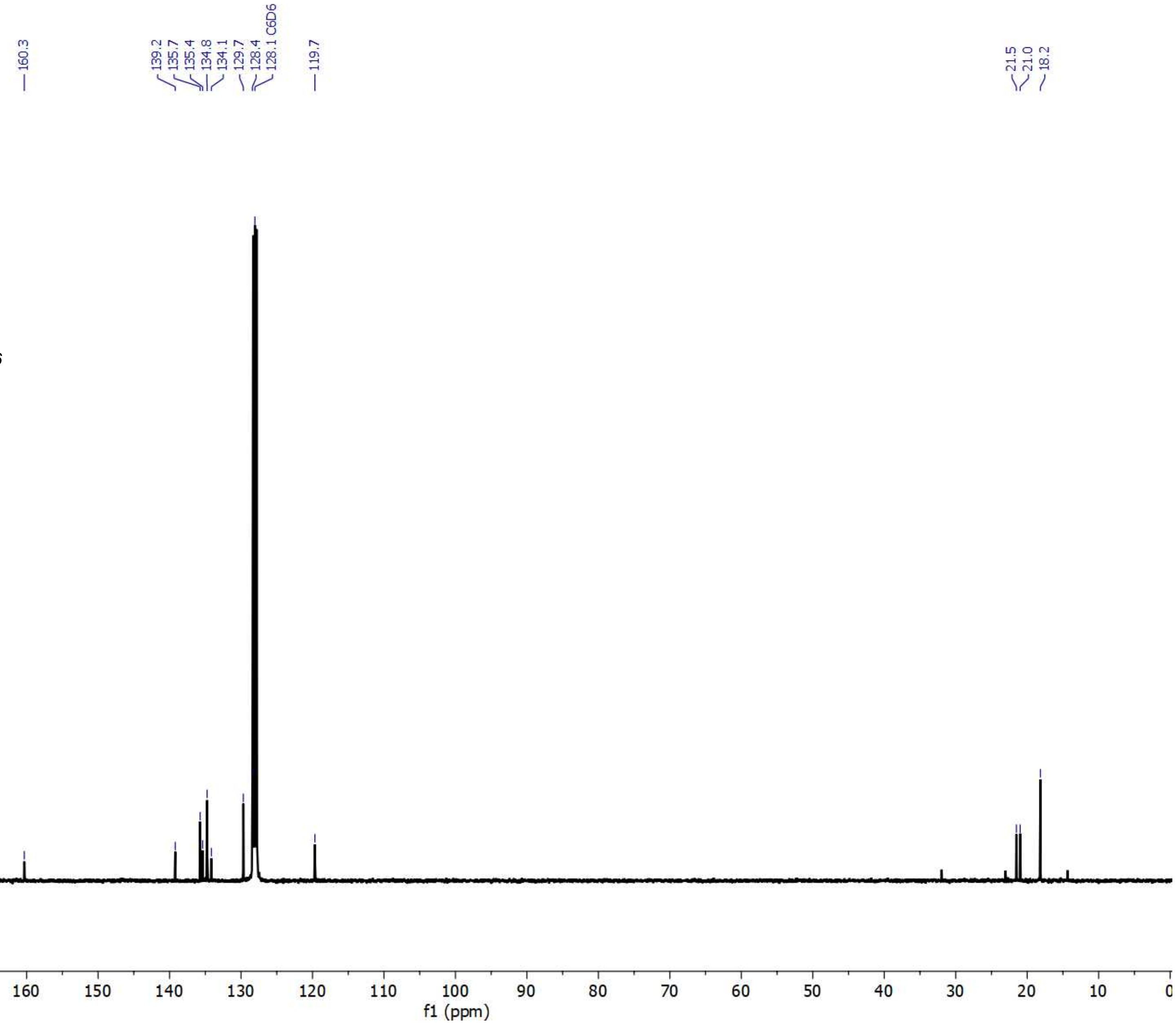

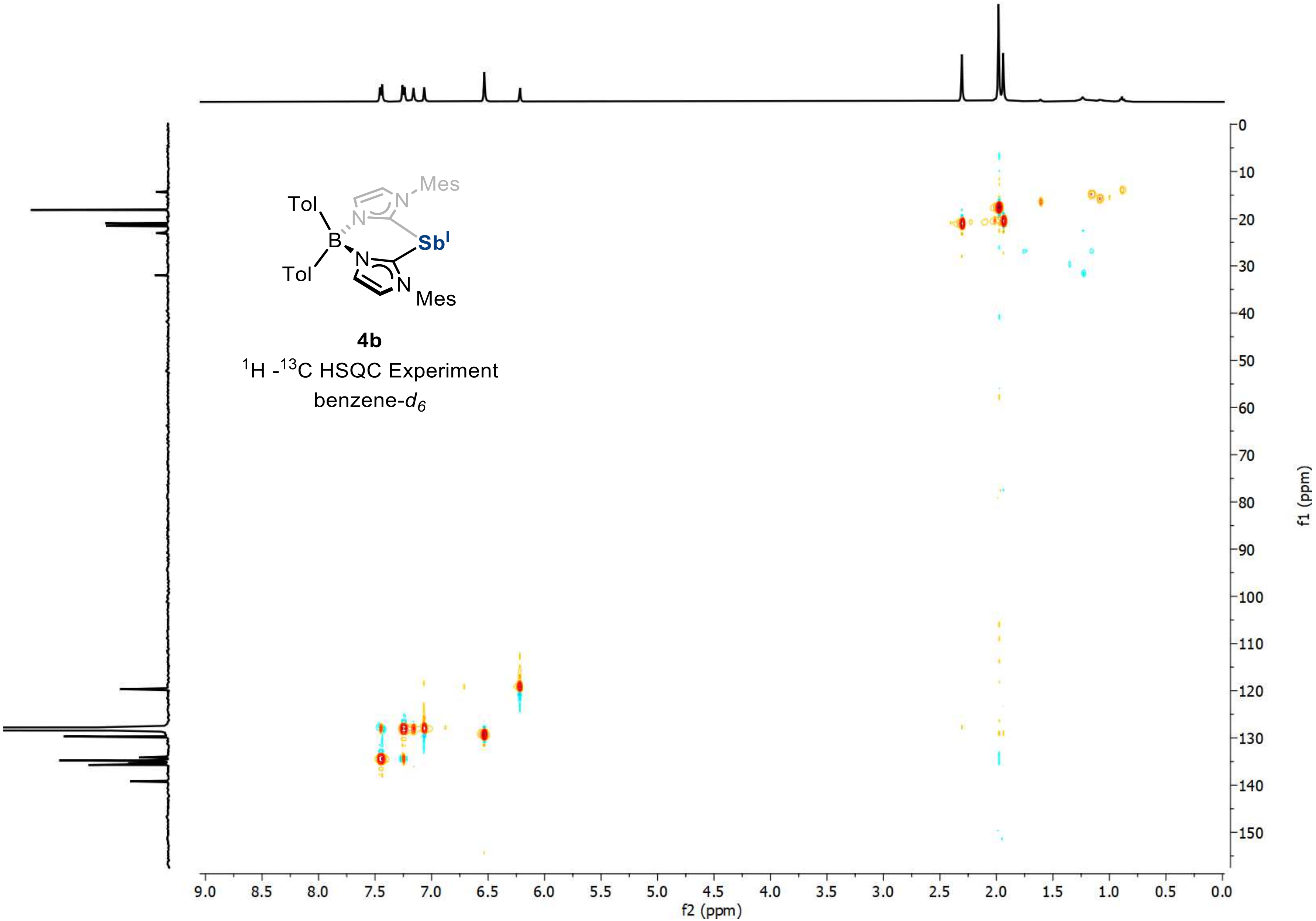


**4b**

$^{1}$H -$^{13}$C HSQC Experiment

benzene-$d_6$

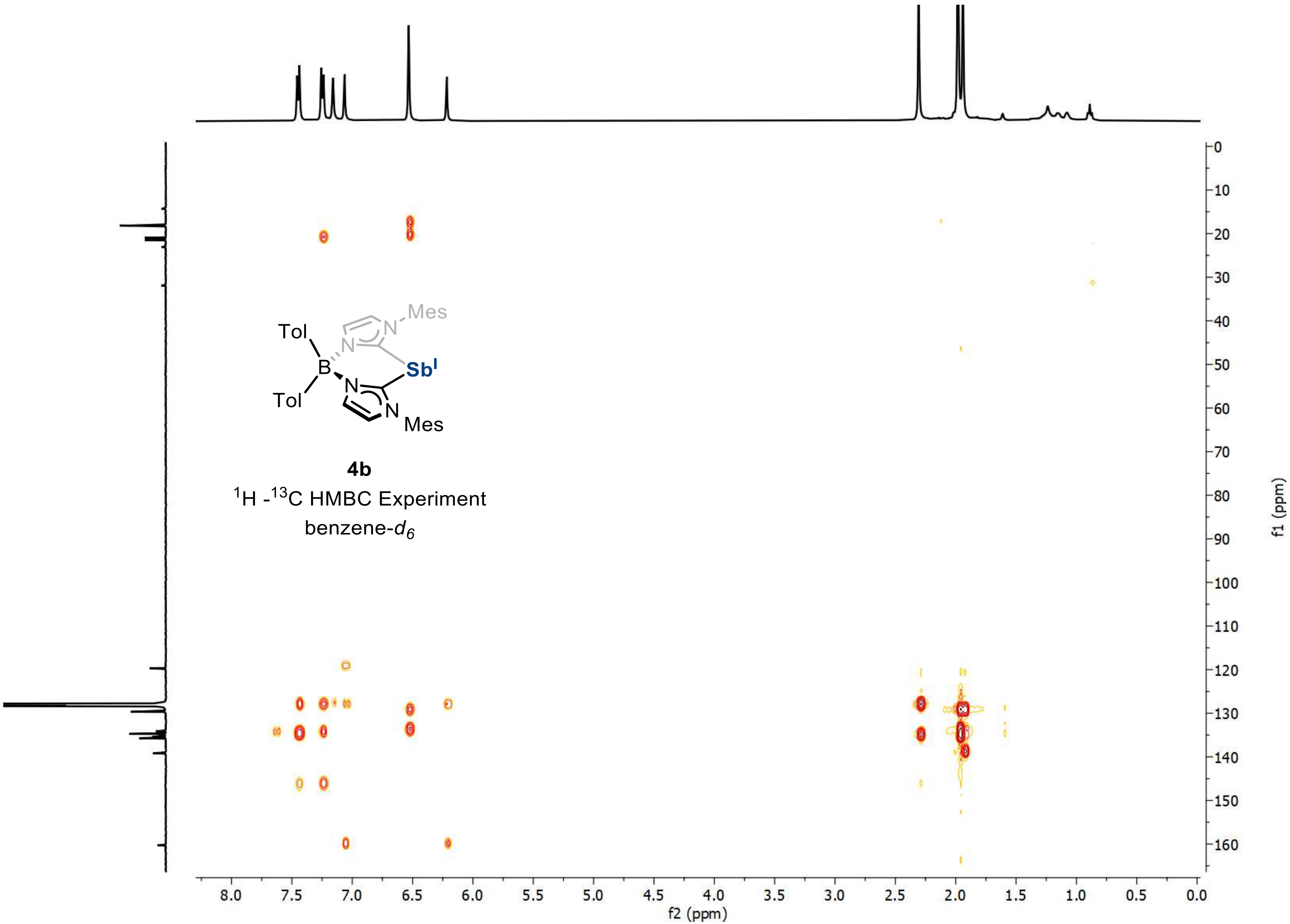


**4b**

$^1$H -$^{13}$C HMBC Experiment

benzene-$d_6$

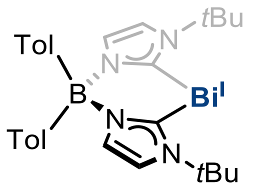


**5a**

$^{1}$H NMR, benzene-$d_6$

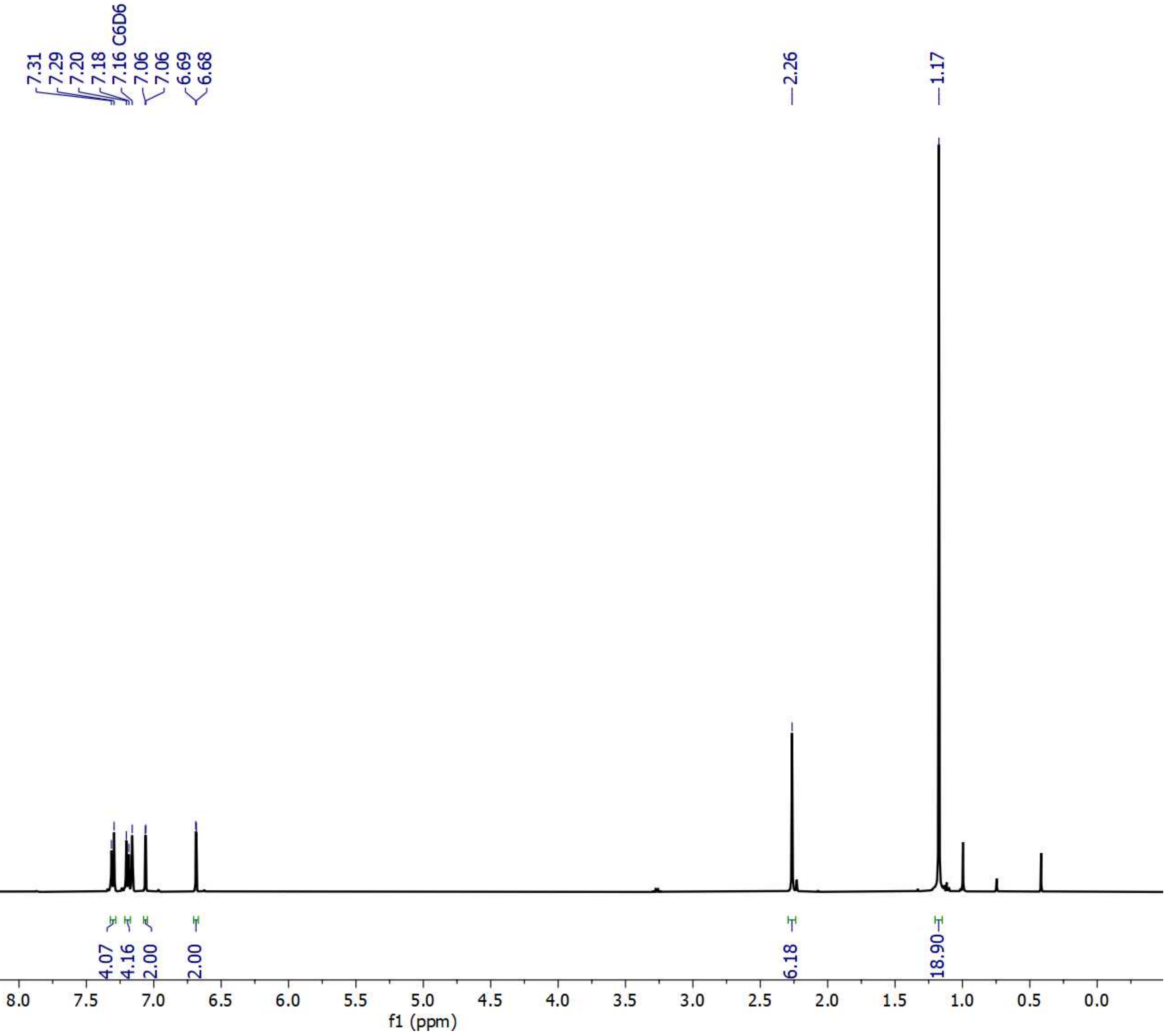

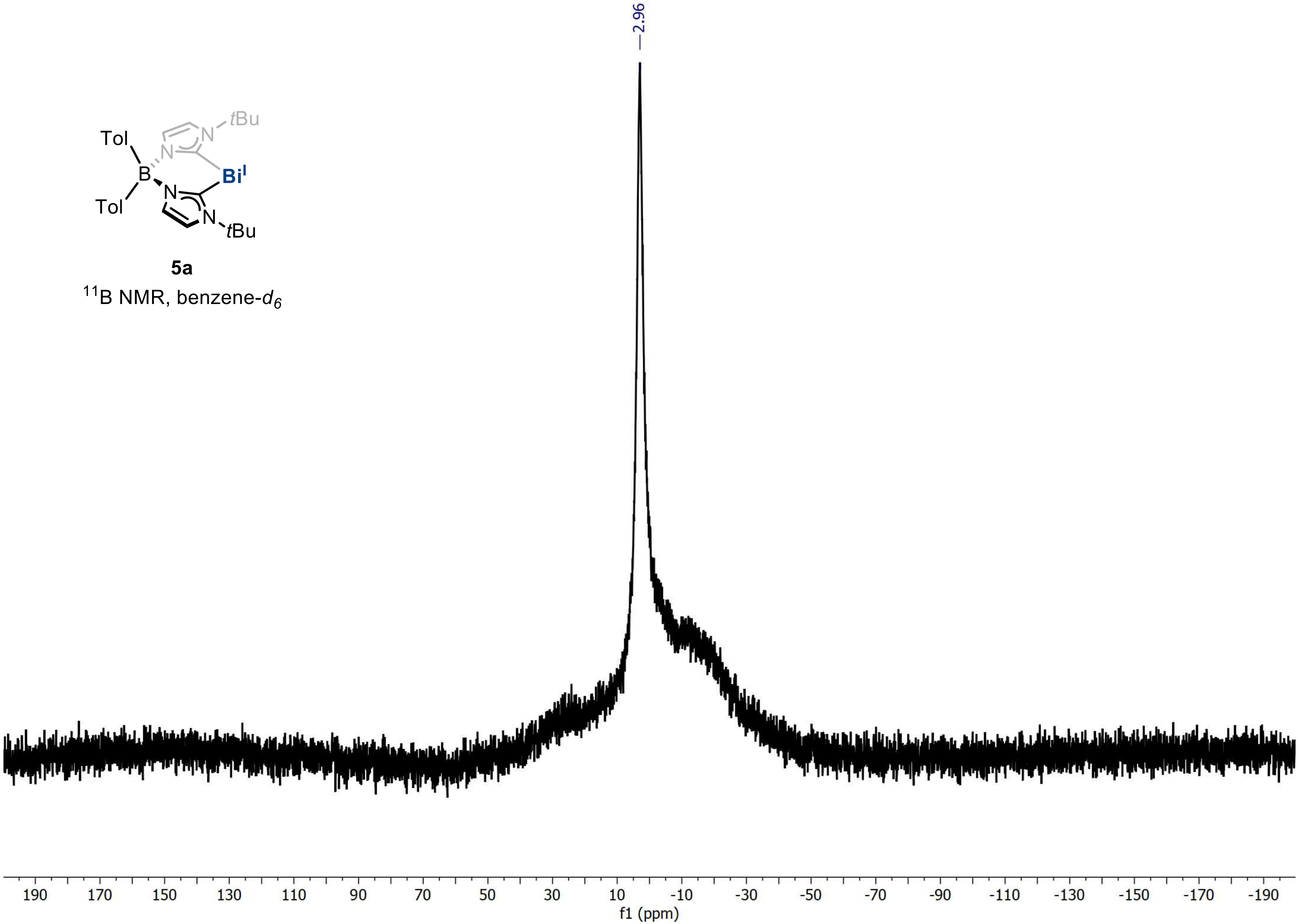


**5a**

$^{11}$B NMR, benzene-$d_6$

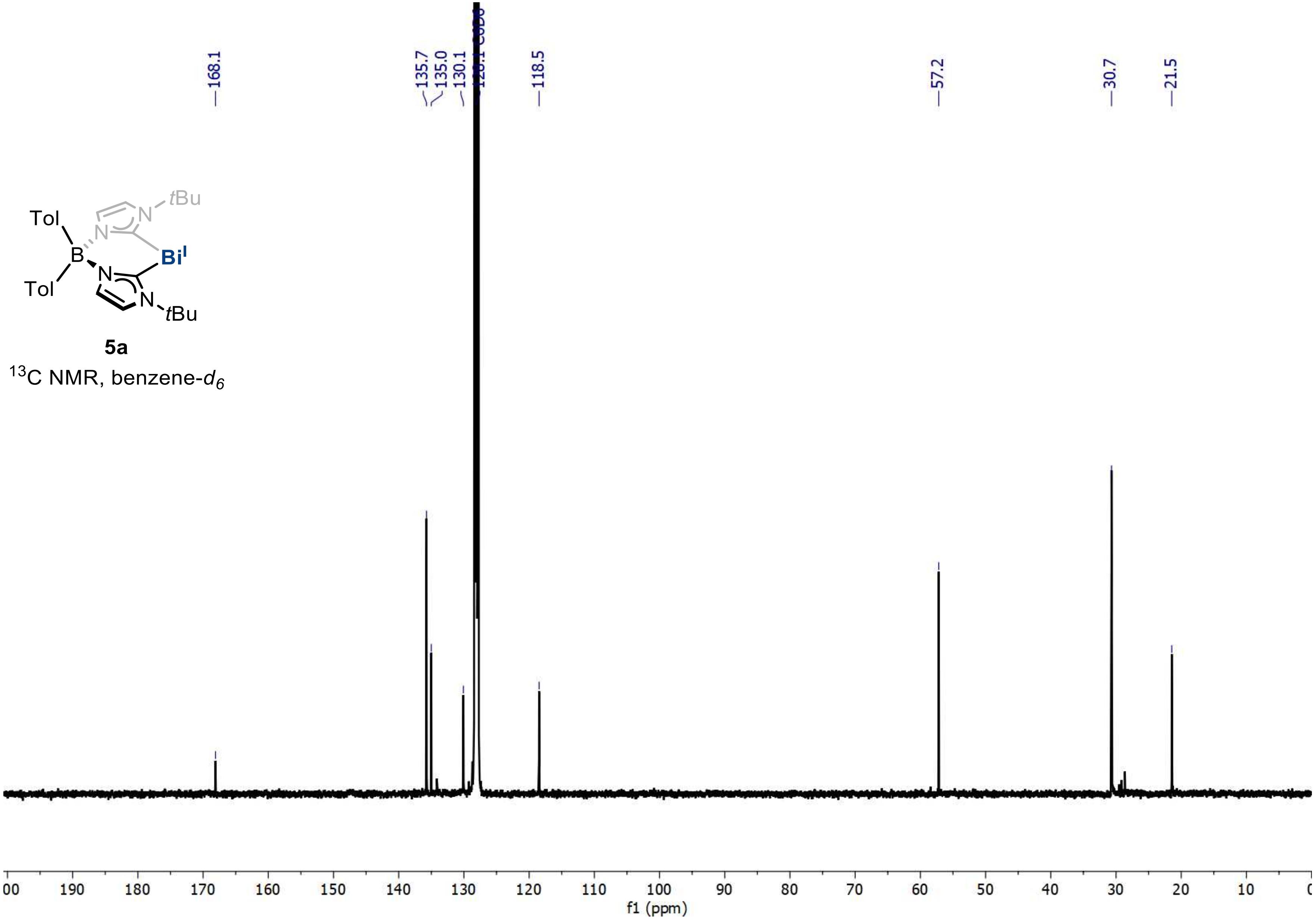


**5a**

$^{13}$C NMR, benzene-$d_6$

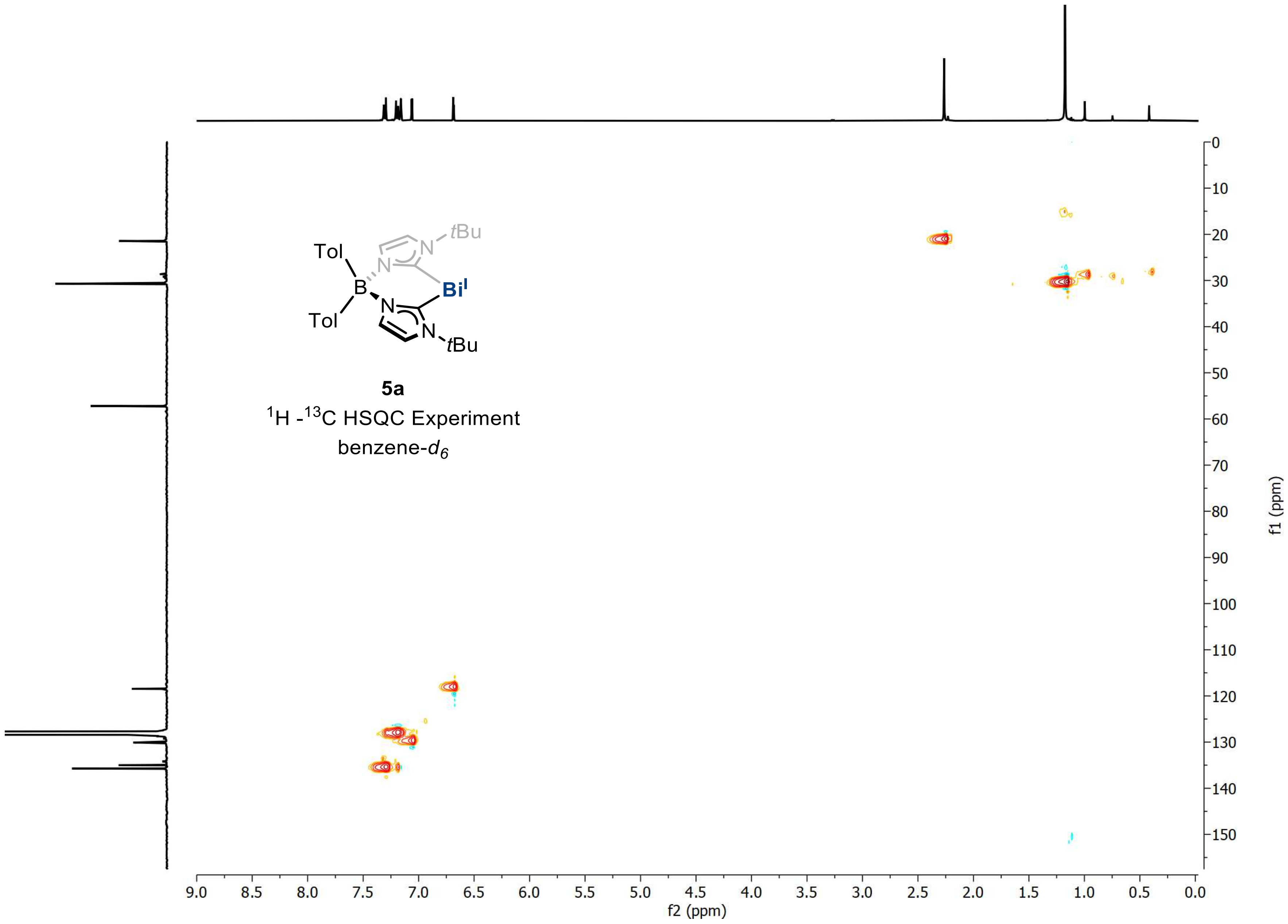
Tol
Tol
B
N
N
N
N
tBu
tBu
Bi^I
5a
1H -13C HSQC Experiment
benzene-d6
f1 (ppm)
f2 (ppm)
0
10
20
30
40
50
60
70
80
90
100
110
120
130
140
150
9.0
8.5
8.0
7.5
7.0
6.5
6.0
5.5
5.0
4.5
4.0
3.5
3.0
2.5
2.0
1.5
1.0
0.5
0.0

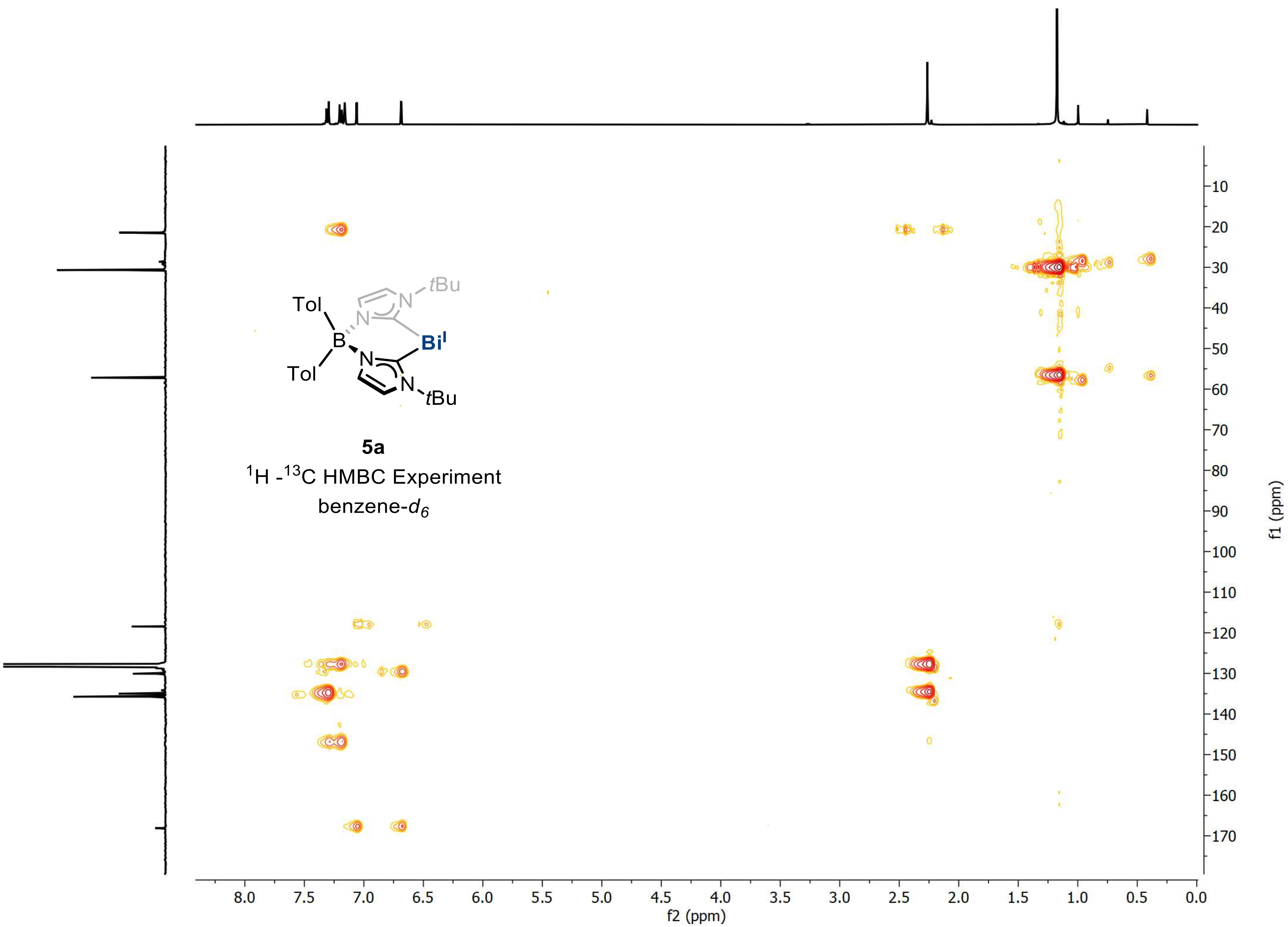

Tol
Tol
B
N
N
N
N
tBu
tBu
Bi$^{I}$
5a
$^{1}$H -$^{13}$C HMBC Experiment
benzene-$d_6$
f1 (ppm)
f2 (ppm)
8.0
7.5
7.0
6.5
6.0
5.5
5.0
4.5
4.0
3.5
3.0
2.5
2.0
1.5
1.0
0.5
0.0
10
20
30
40
50
60
70
80
90
100
110
120
130
140
150
160
170

## 11.5 NMR characterisation of oxidative addition products

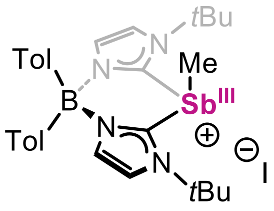


**6**

$^1H$ NMR. $CDCl_3$

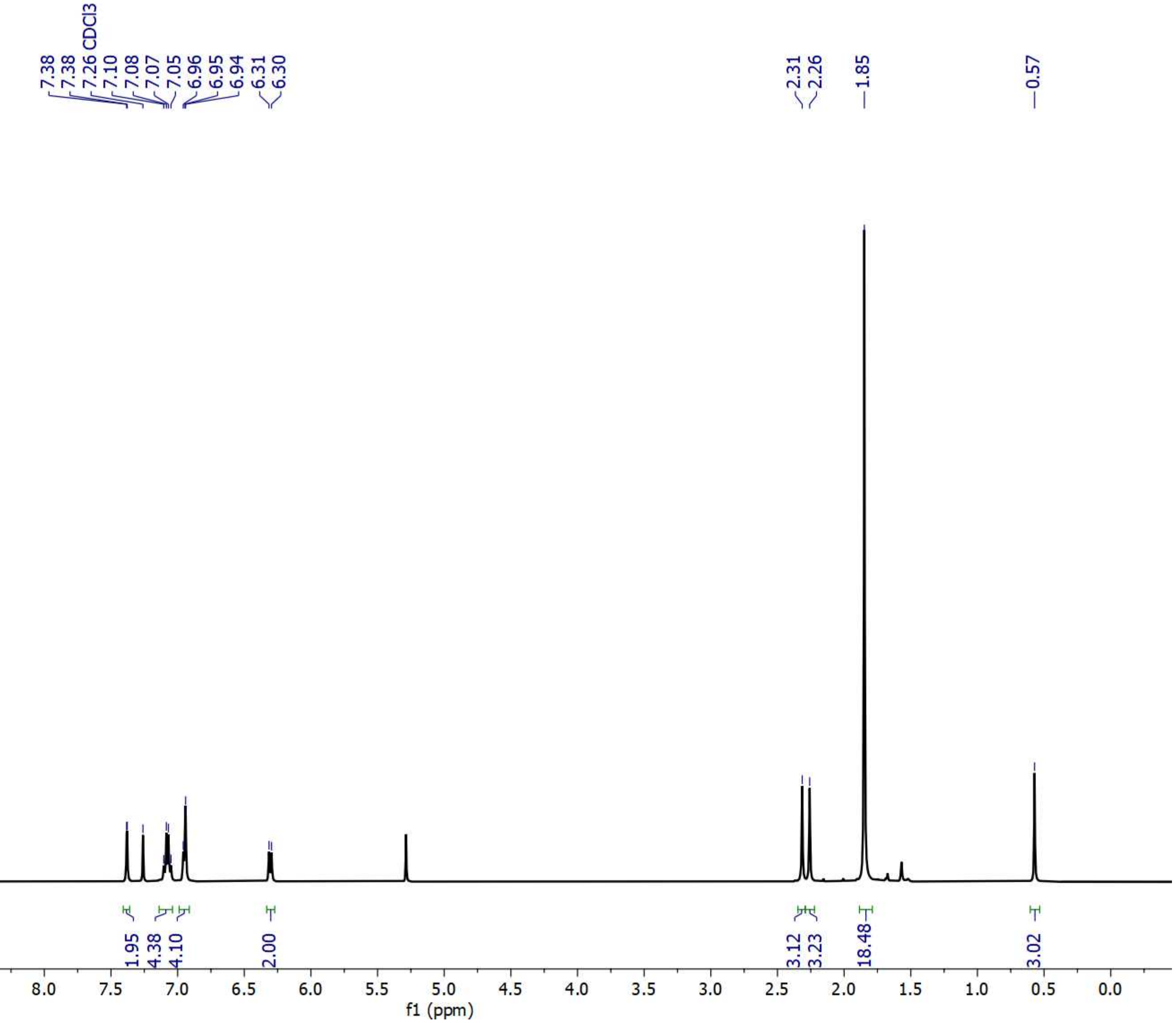

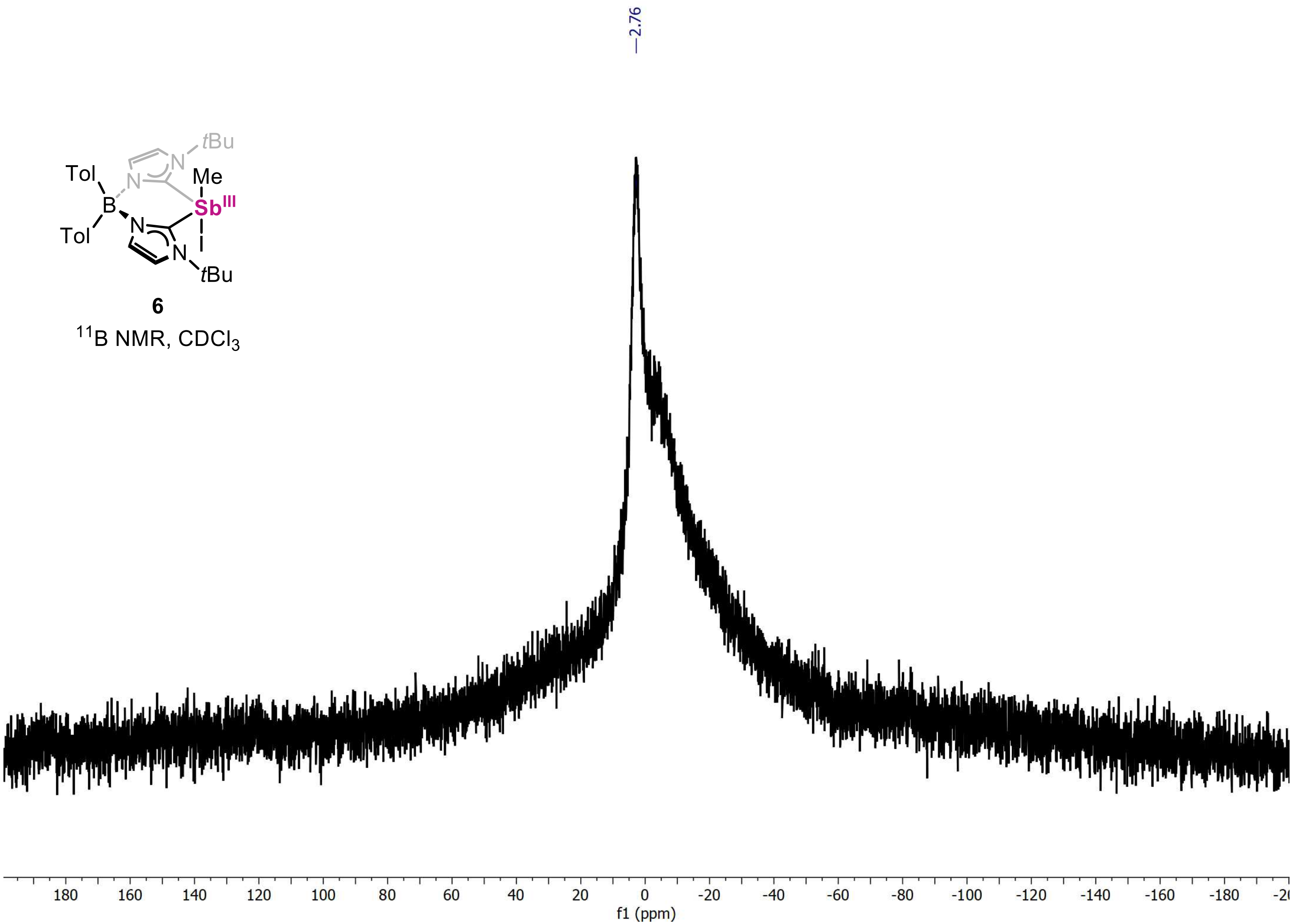

2.76
Tol
Tol
B
N
N
N
N
tBu
tBu
Me
Sb^III
I
6
11B NMR, CDCl3
180 160 140 120 100 80 60 40 20 0 -20 -40 -60 -80 -100 -120 -140 -160 -180
f1 (ppm)

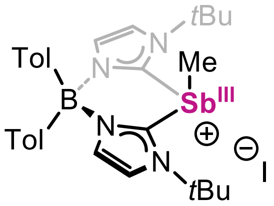


**6**

$^{13}$C NMR, $CDCl_3$

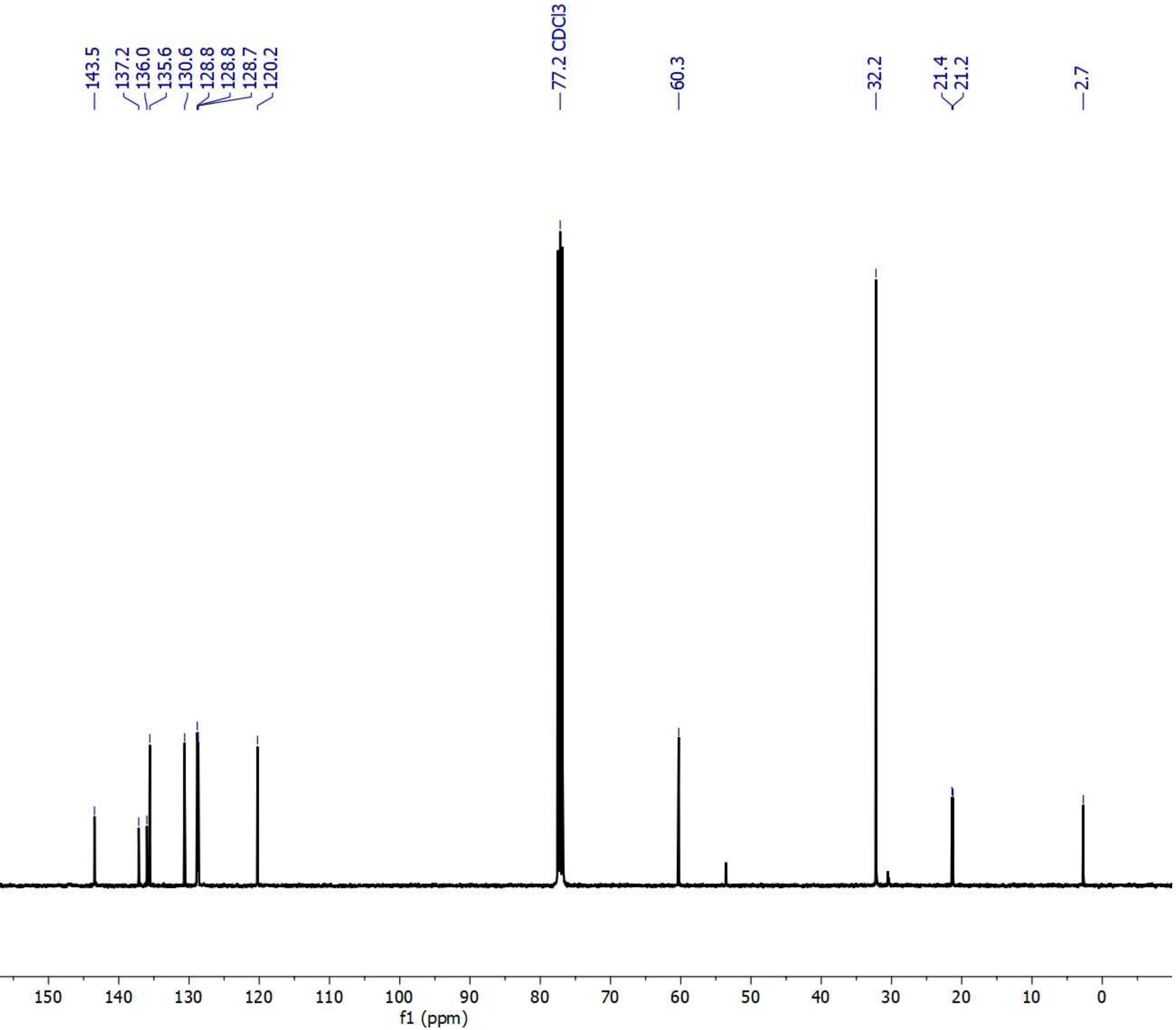

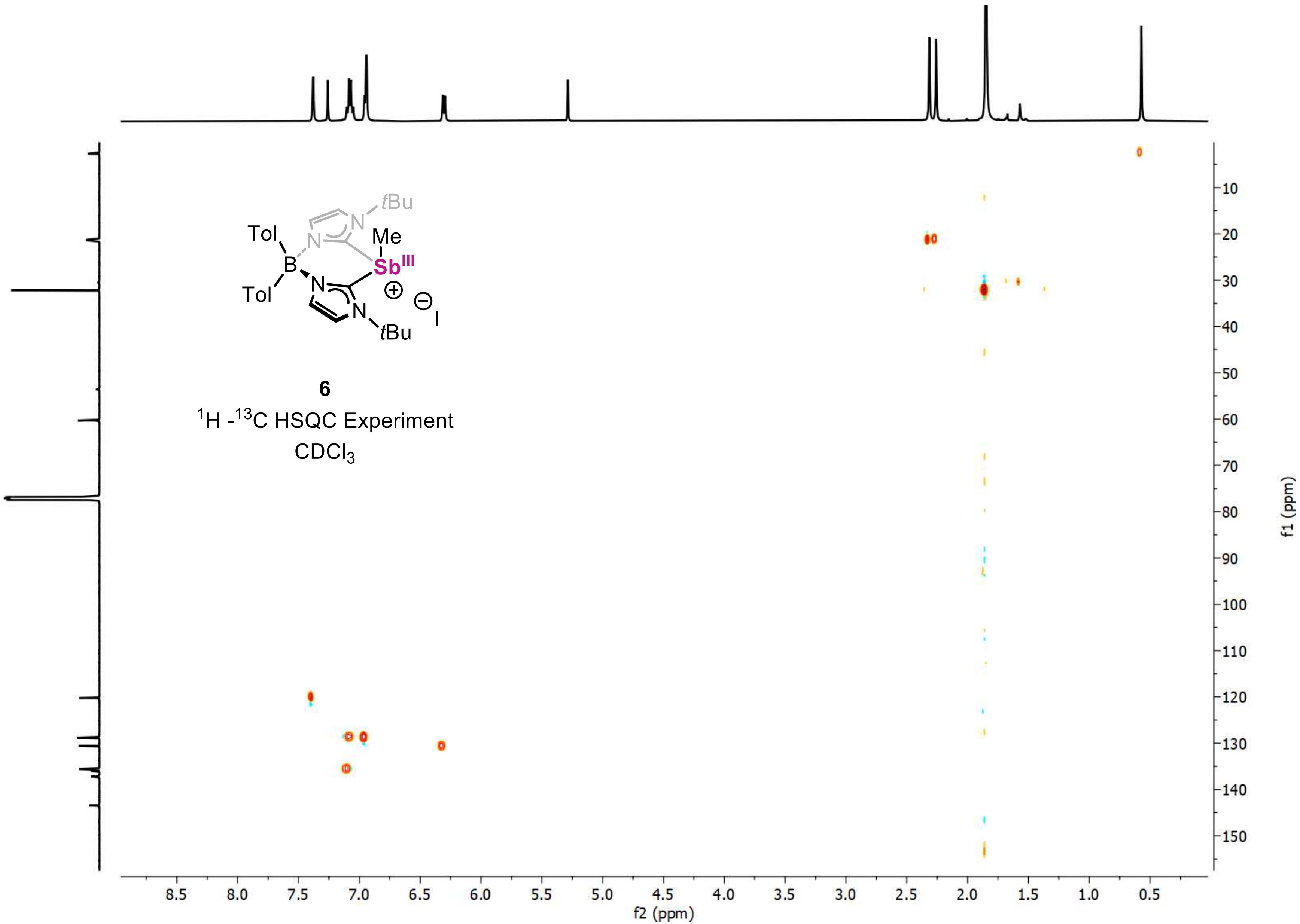

tBu
Tol
N
Me
B
Sb^III
Tol
N
N
tBu
I
6
$^{1}$H -$^{13}$C HSQC Experiment
$CDCl_3$
f2 (ppm)
f1 (ppm)
8.5 8.0 7.5 7.0 6.5 6.0 5.5 5.0 4.5 4.0 3.5 3.0 2.5 2.0 1.5 1.0 0.5
10 20 30 40 50 60 70 80 90 100 110 120 130 140 150

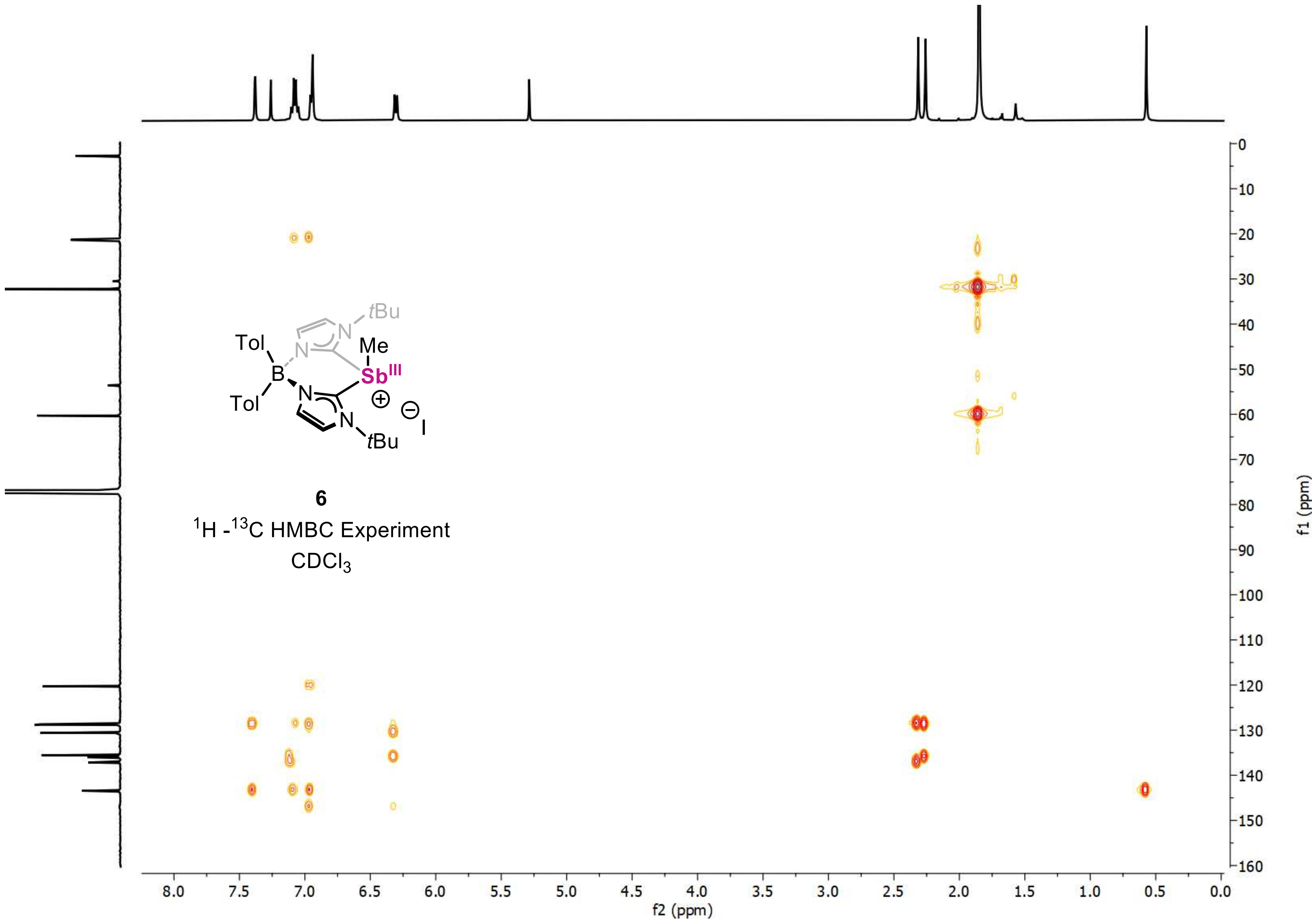

tBu
Tol
Me
N
N
B
Sb$^{III}$
Tol
N
N
tBu
I
6
$^1$H - $^{13}$C HMBC Experiment
$CDCl_3$
f1 (ppm)
f2 (ppm)

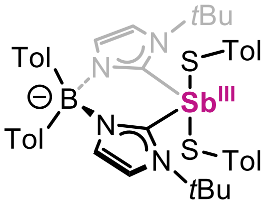

**7**

$^{1}$H NMR, THF-*d*8

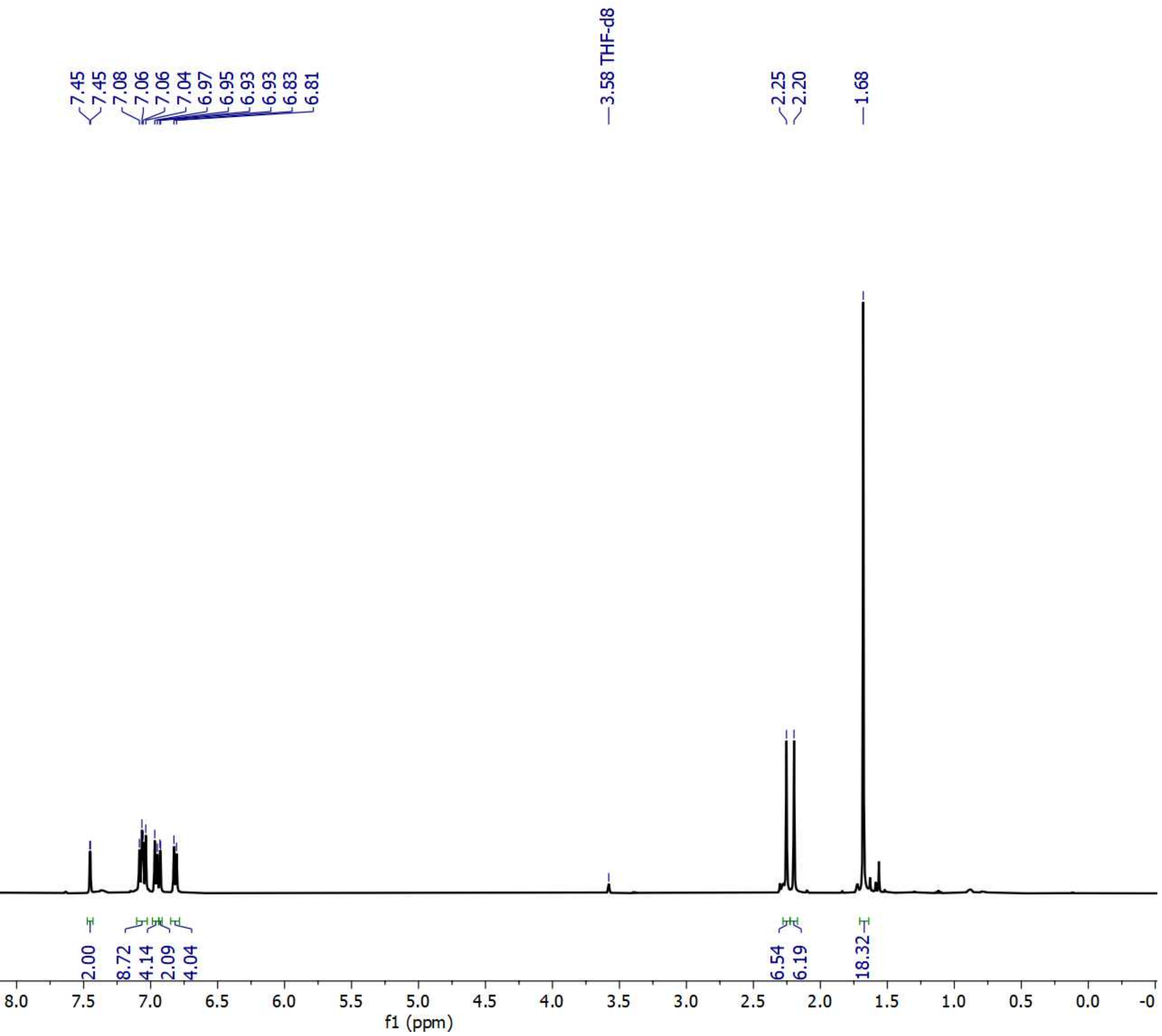

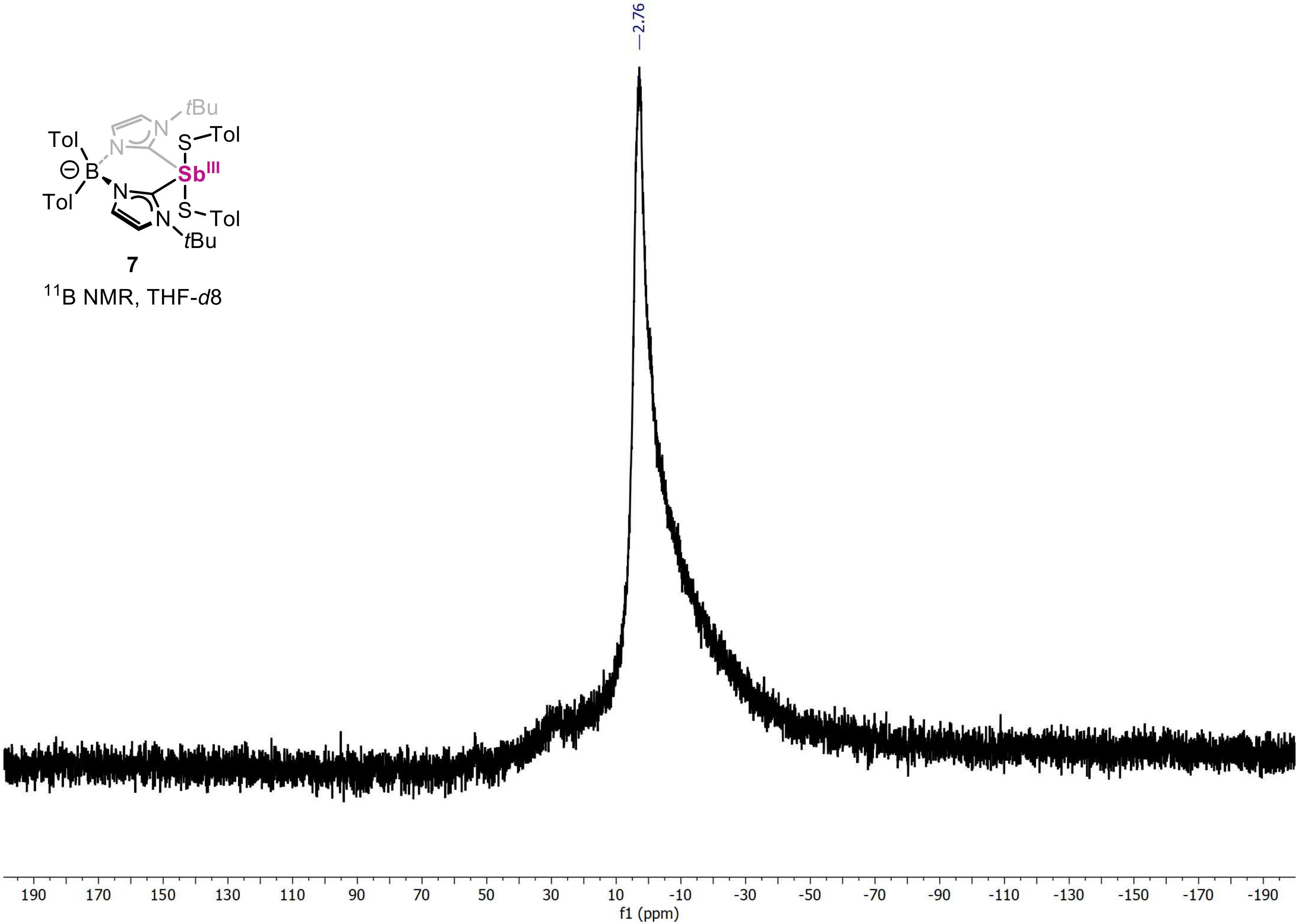


**7**

$^{11}$B NMR, THF-*d*8

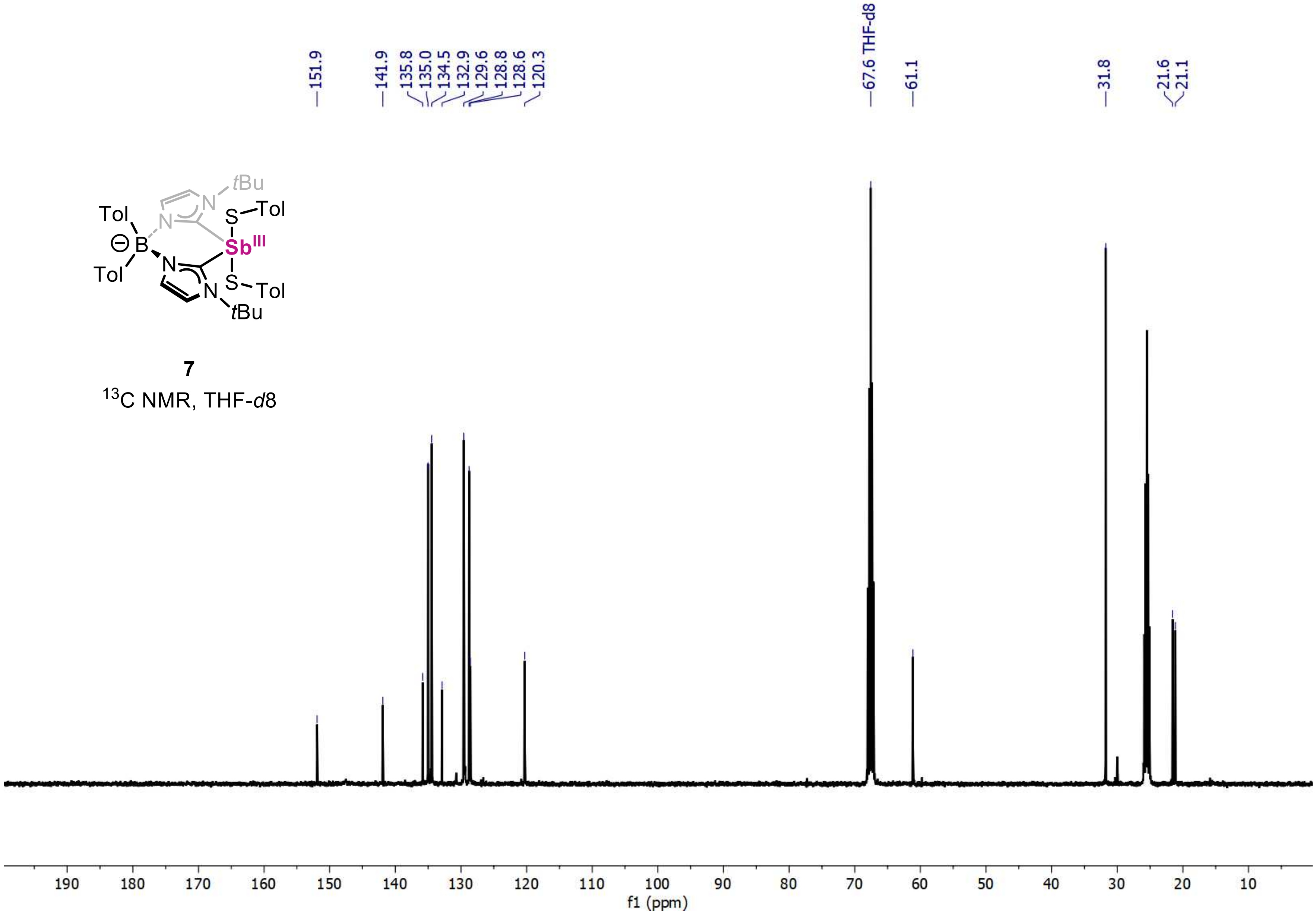
tBu
S
Tol
Tol
B
N
N
Sb III
Tol
N
S
Tol
N
tBu
7
13C NMR, THF-d8
151.9
141.9
135.8
135.0
134.5
132.9
129.6
128.8
128.6
120.3
67.6 THF-d8
61.1
31.8
21.6
21.1
190
180
170
160
150
140
130
120
110
100
90
80
70
60
50
40
30
20
10
f1 (ppm)

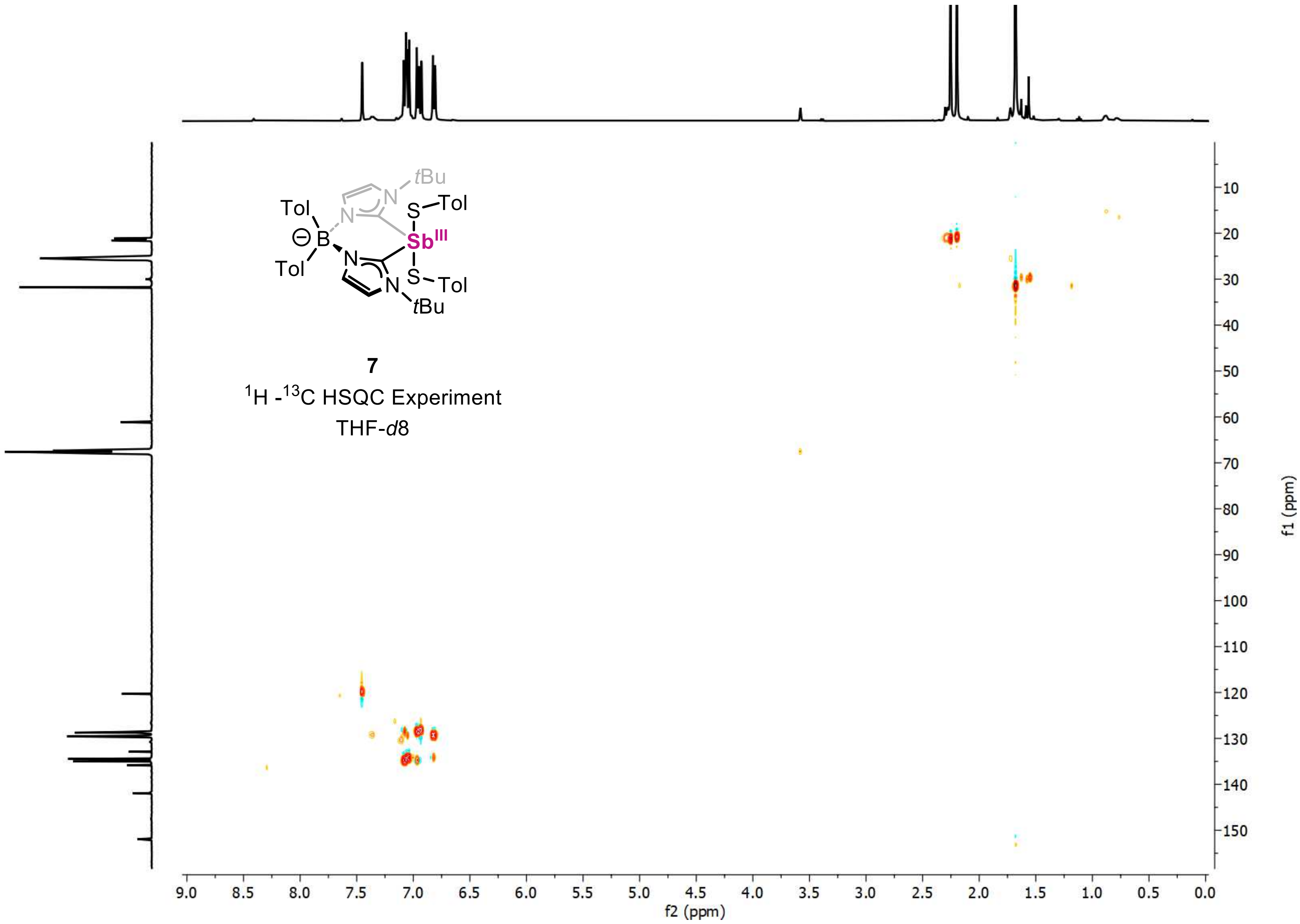

tBu
S
Tol
Tol
N
N
B
Sb^III
Tol
N
S
Tol
N
tBu
7
1H -13C HSQC Experiment
THF-d8
f1 (ppm)
f2 (ppm)

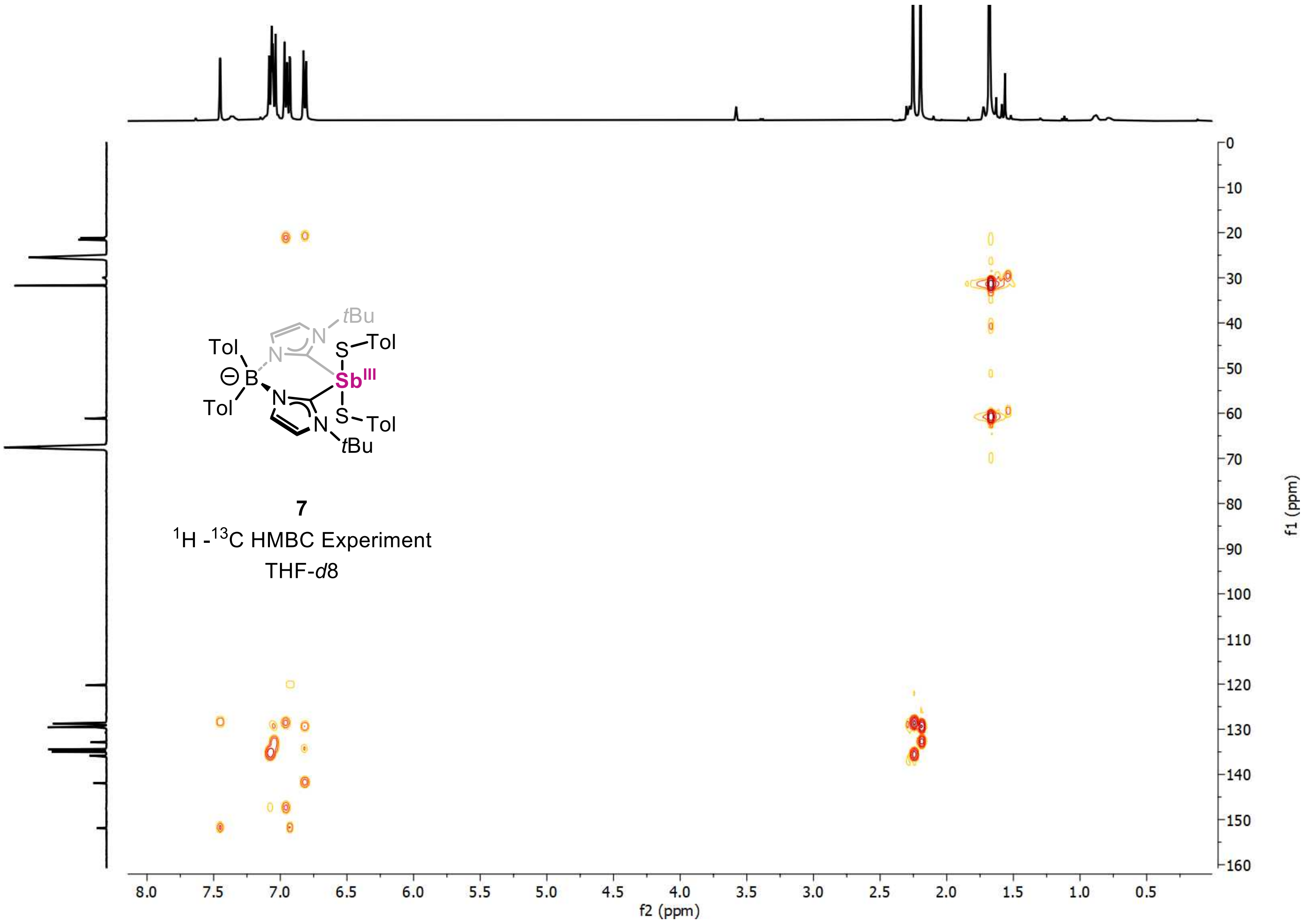
tBu
Tol
N
S
Tol
Sb$^{III}$
B
N
S
Tol
Tol
N
tBu
7
$^{1}$H -$^{13}$C HMBC Experiment
THF-$d$8
f1 (ppm)
f2 (ppm)

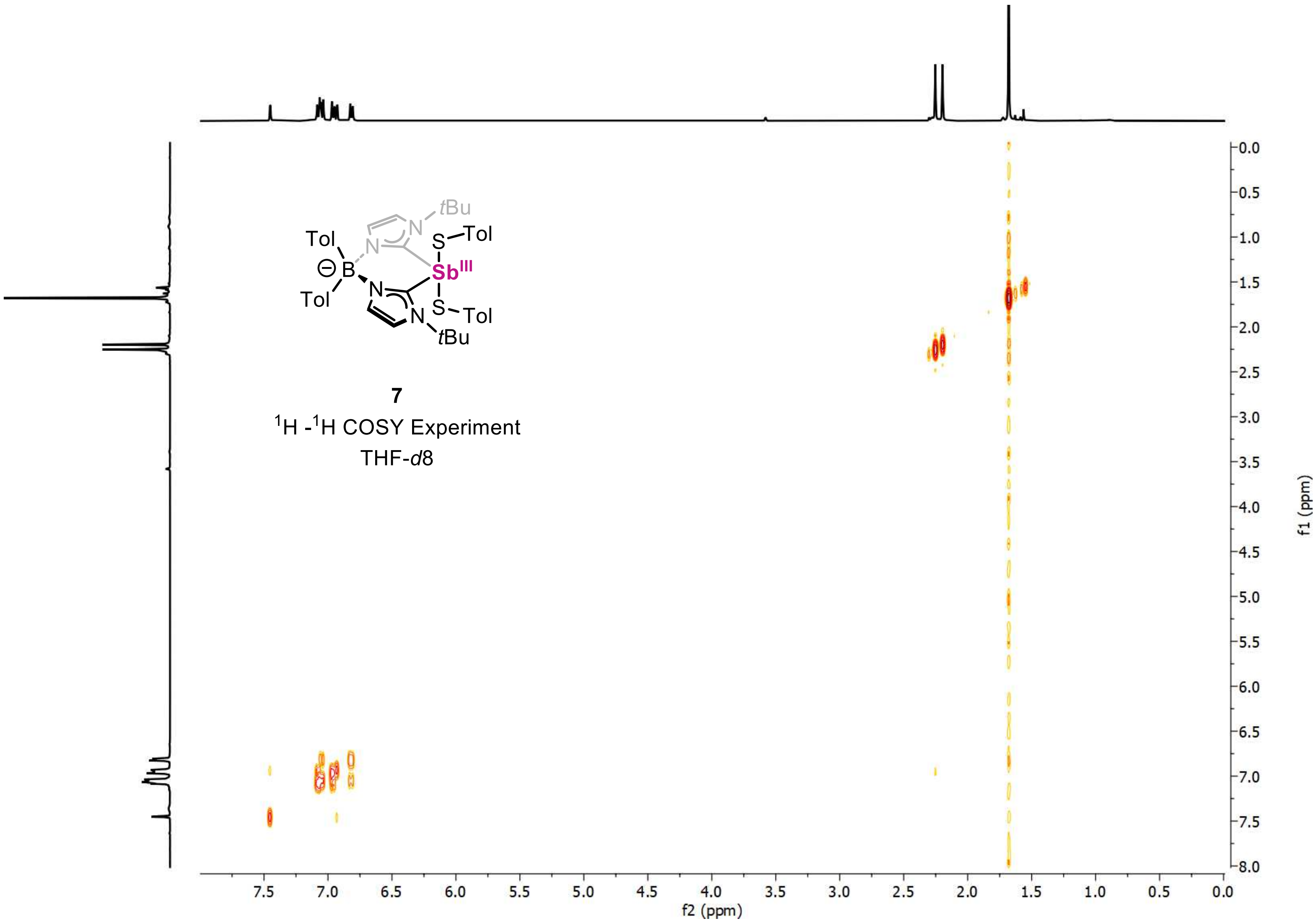

Tol
Tol
B
N
N
tBu
S
Tol
Sb^III
S
Tol
N
N
tBu
7
1H -1H COSY Experiment
THF-d8
7.5
7.0
6.5
6.0
5.5
5.0
4.5
4.0
3.5
3.0
2.5
2.0
1.5
1.0
0.5
0.0
f2 (ppm)
0.0
0.5
1.0
1.5
2.0
2.5
3.0
3.5
4.0
4.5
5.0
5.5
6.0
6.5
7.0
7.5
8.0
f1 (ppm)

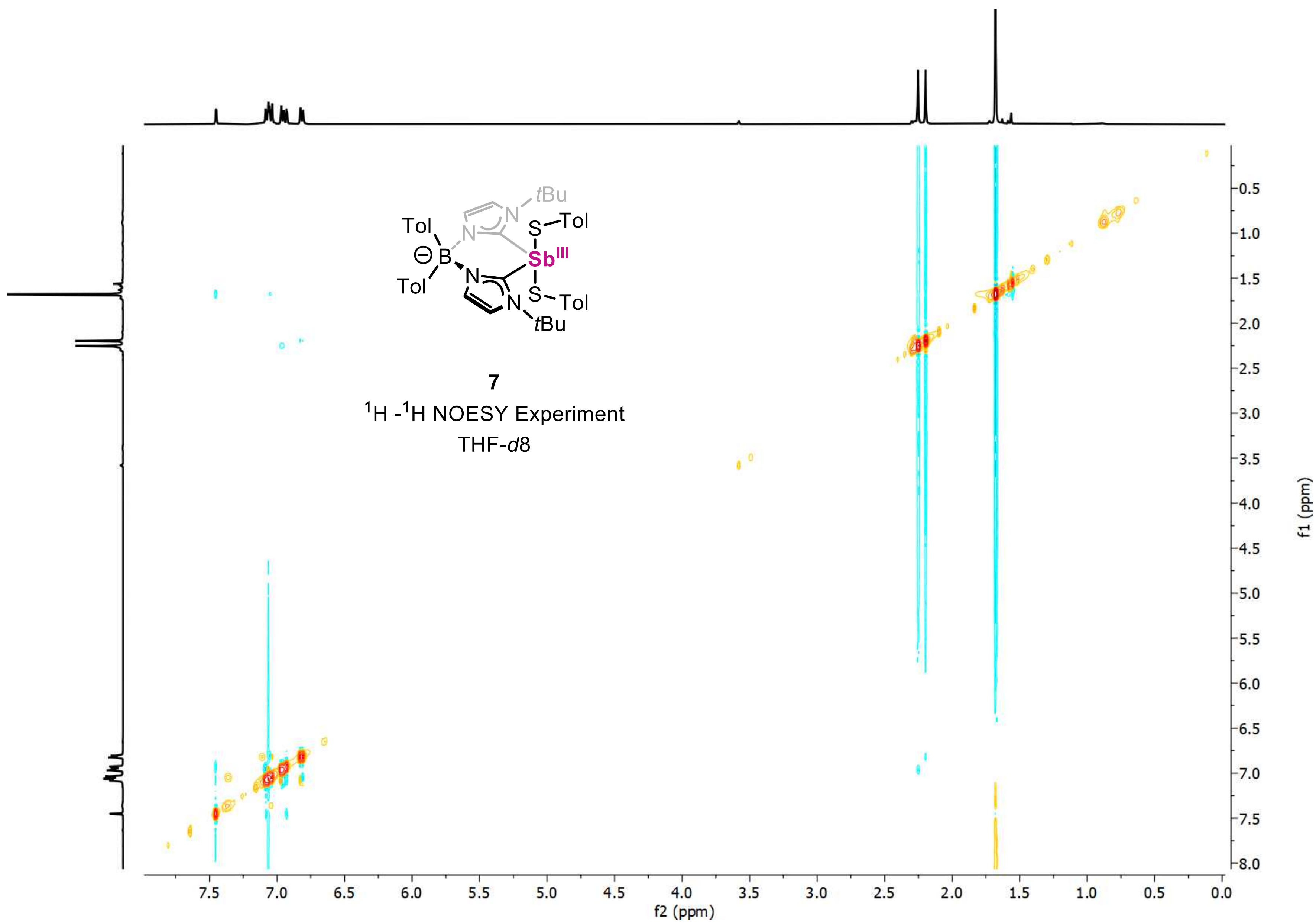
Tol
Tol
B
N
N
N
N
tBu
tBu
S
Tol
S
Tol
Sb^III
7
1H -1H NOESY Experiment
THF-d8
f2 (ppm)
f1 (ppm)

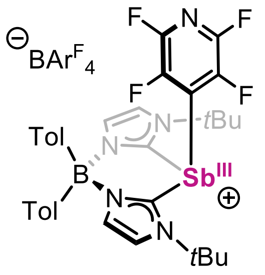

**8a**

$^{1}$H NMR, $CDCl_3$

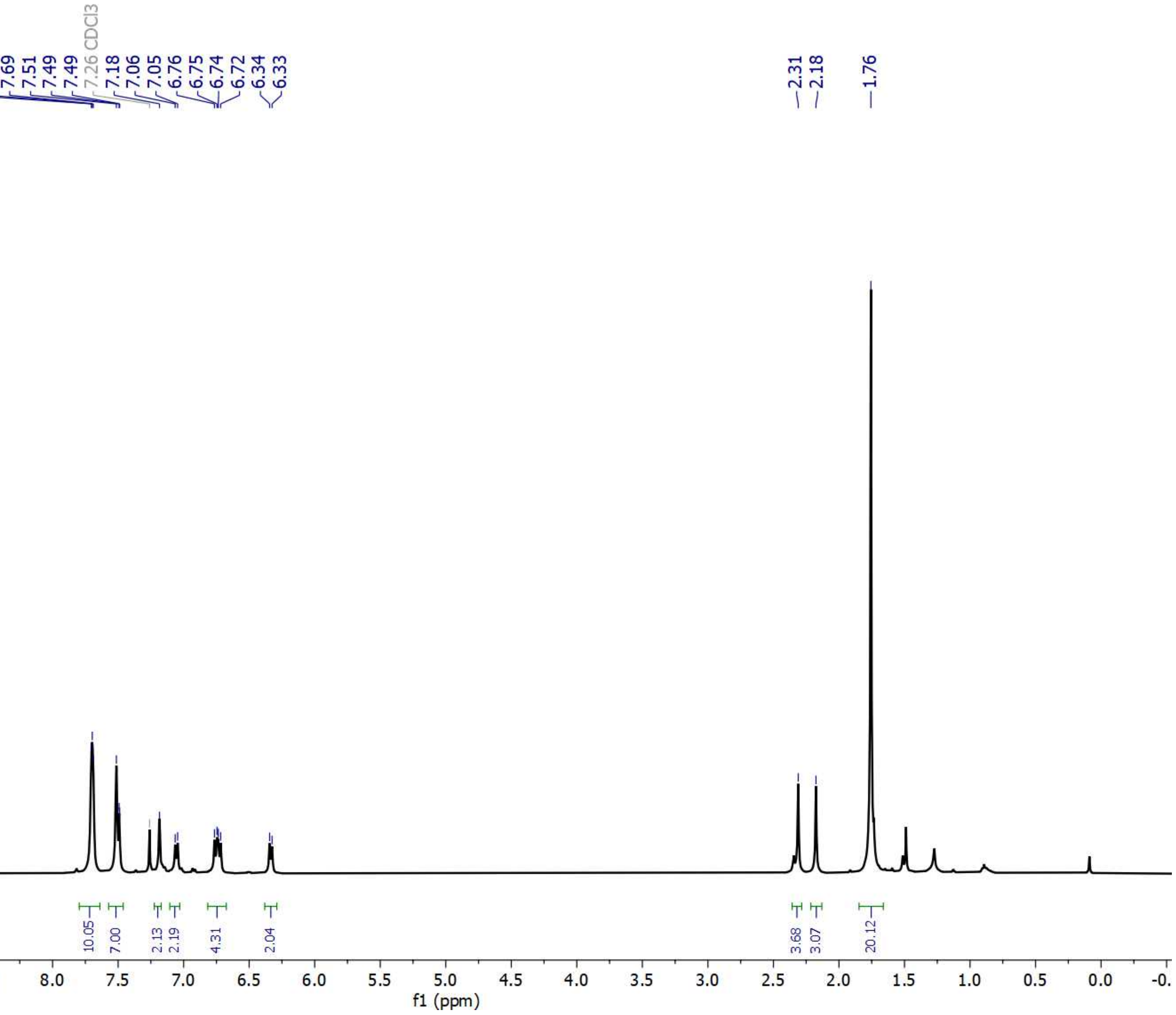

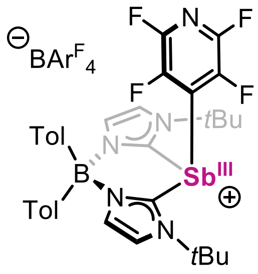


**8a**

$^{19}$F NMR, $CDCl_3$

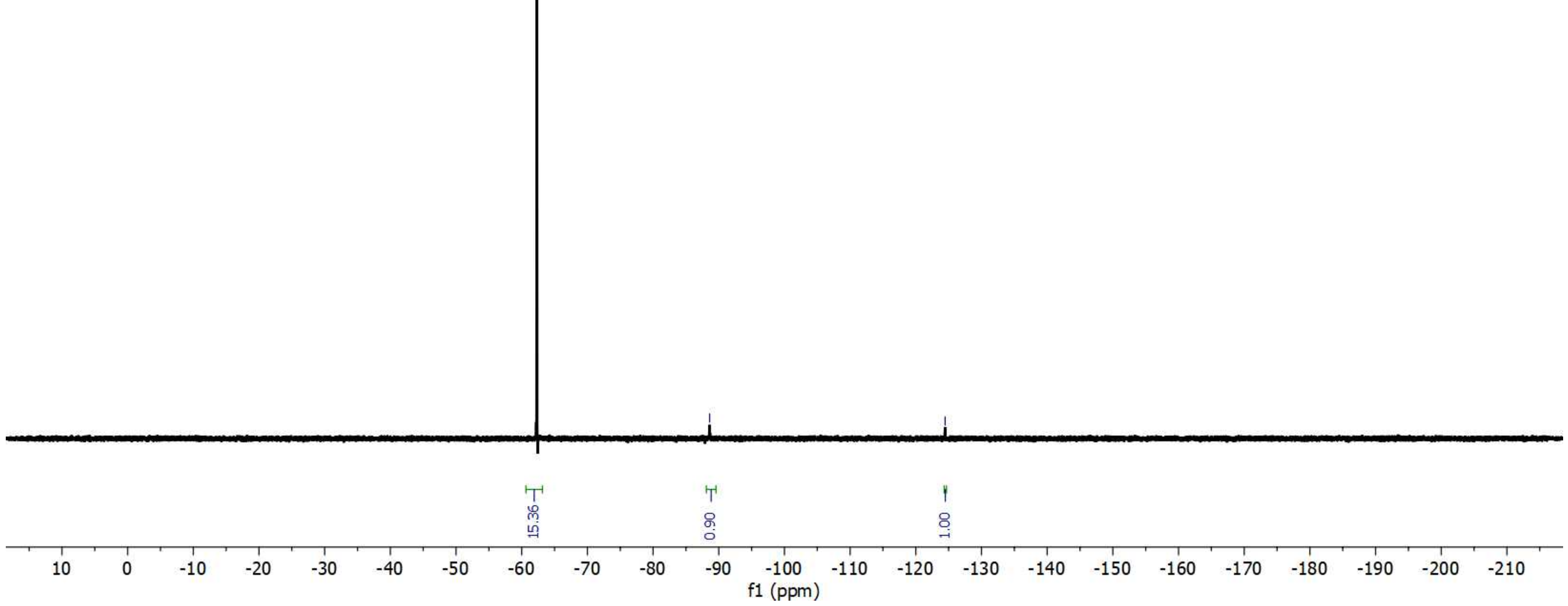

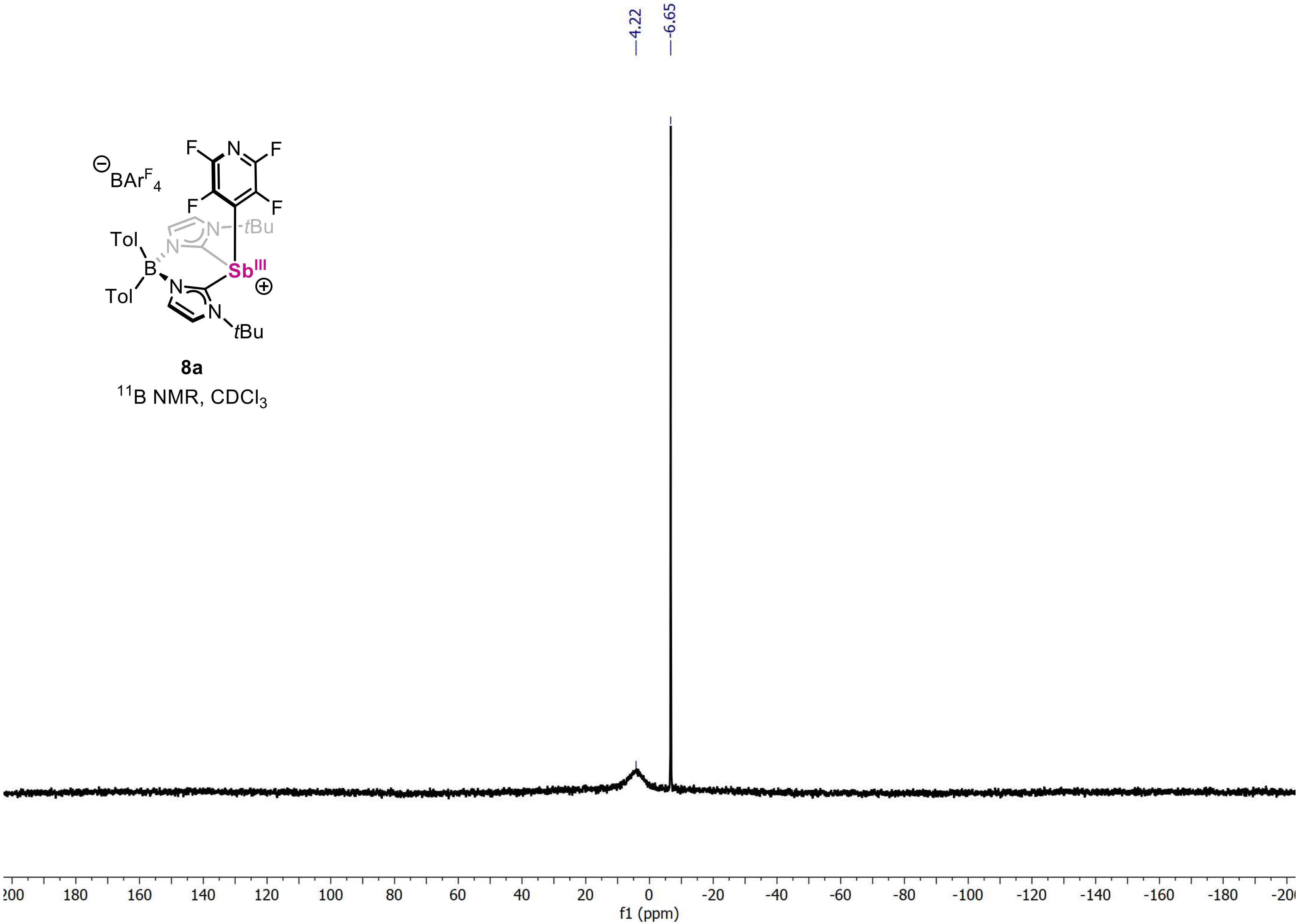


**8a**

$^{11}$B NMR, $CDCl_3$

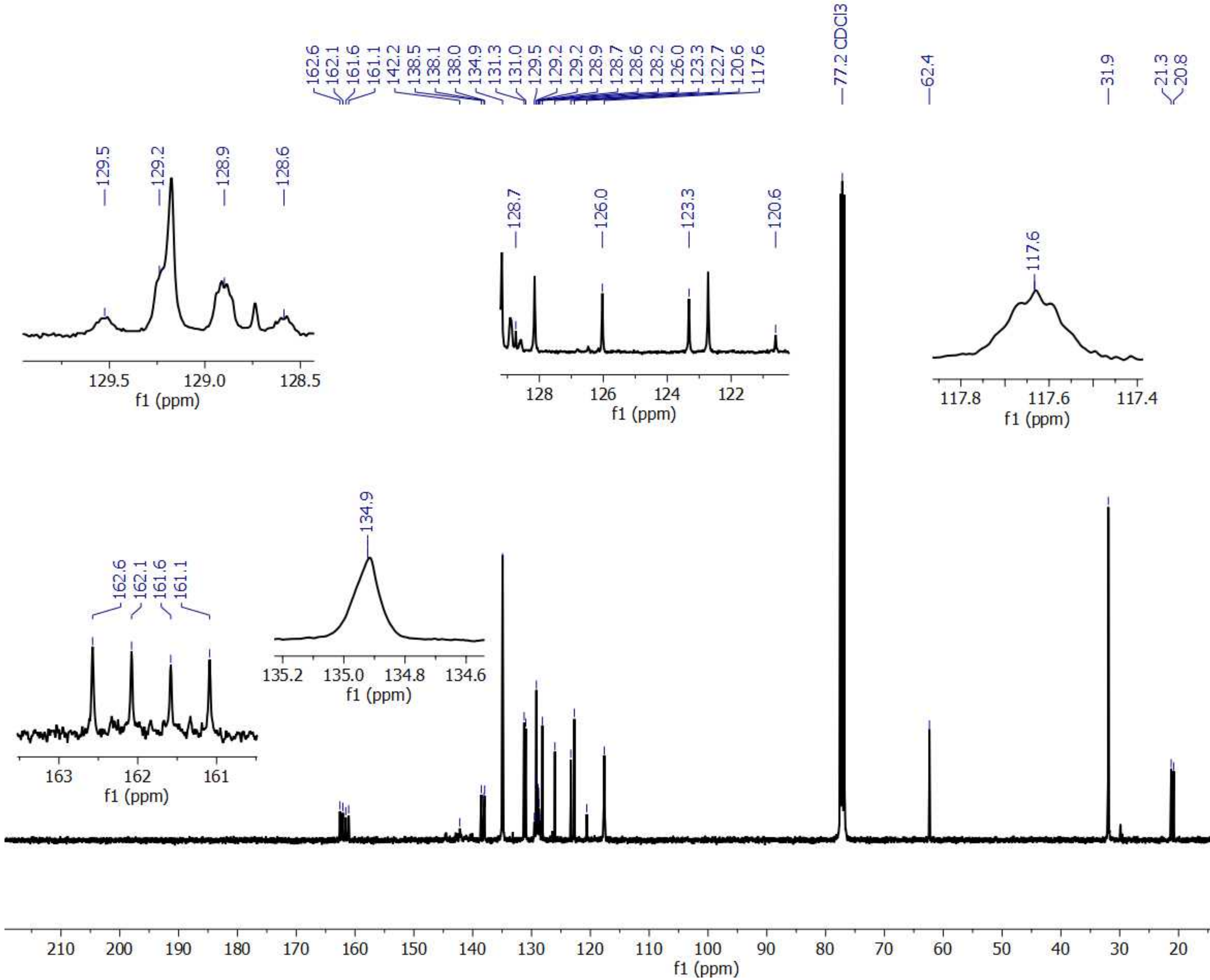


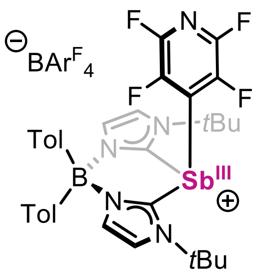


**8a**

$^{13}C$ NMR, $CDCl_3$

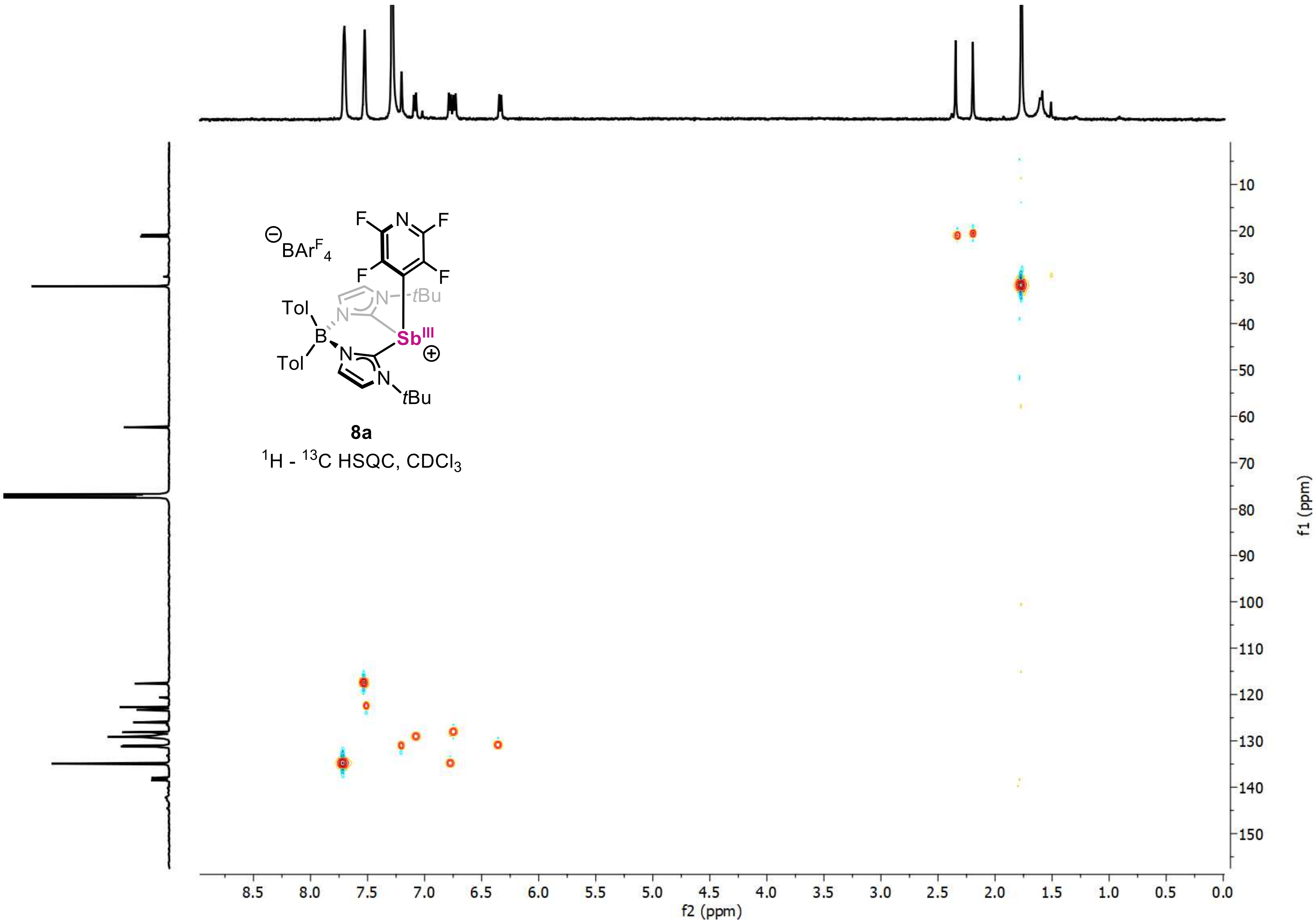

⊖BAr$^F_4$
F
N
F
F
F
N
tBu
Tol
B
Tol
N
Sb$^{III}$
⊕
N
tBu
8a
$^1$H - $^{13}$C HSQC, CDCl$_3$
f1 (ppm)
f2 (ppm)

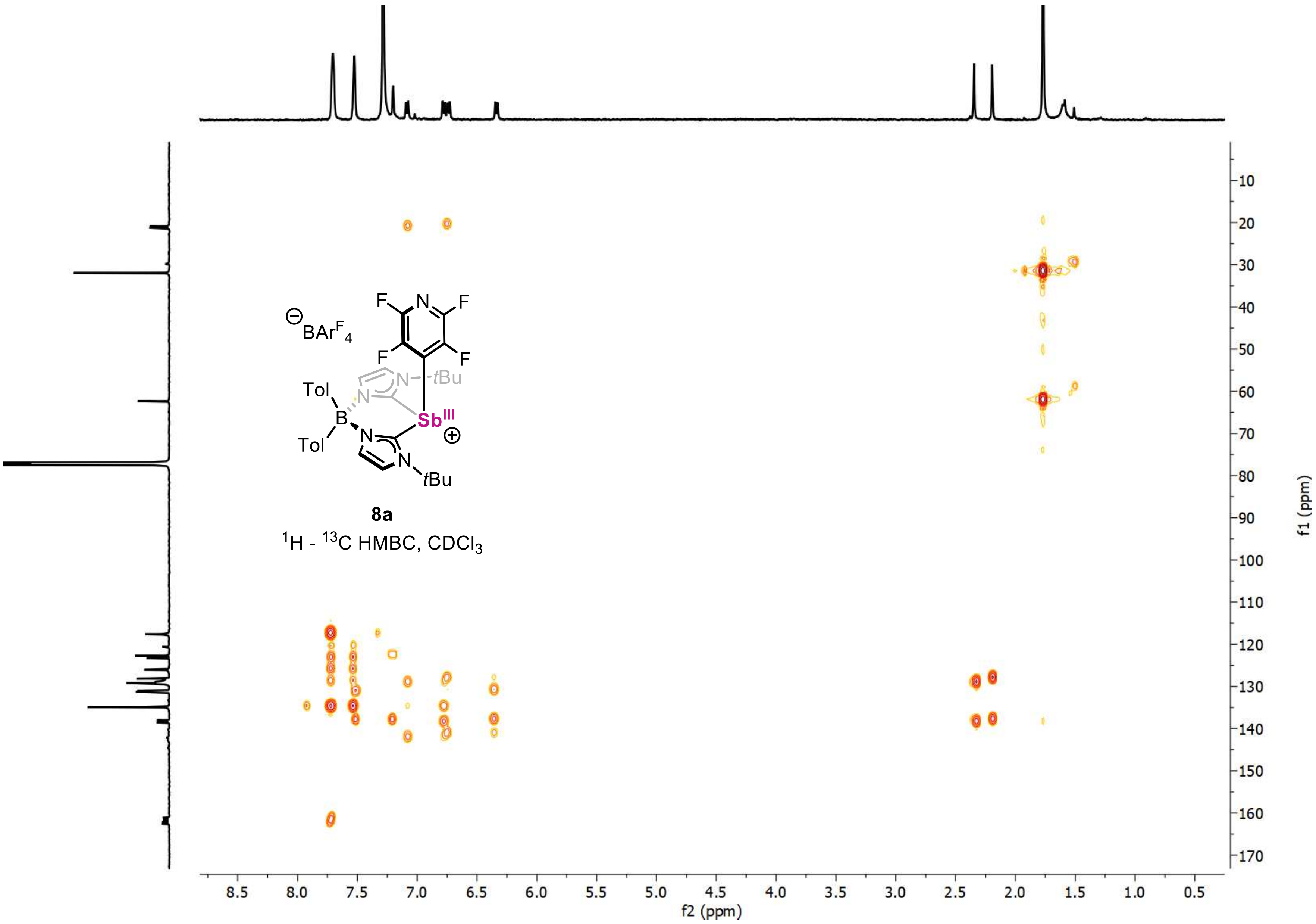

⊖BArF4
F
N
F
F
F
N
tBu
Tol
N
B
Sb III
⊕
Tol
N
N
tBu
8a
1H - 13C HMBC, CDCl3
f1 (ppm)
f2 (ppm)

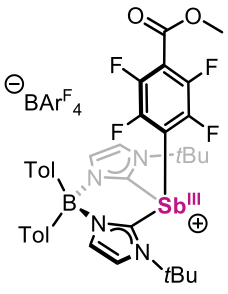


**9a**

$^{1}$H NMR, $CDCl_3$

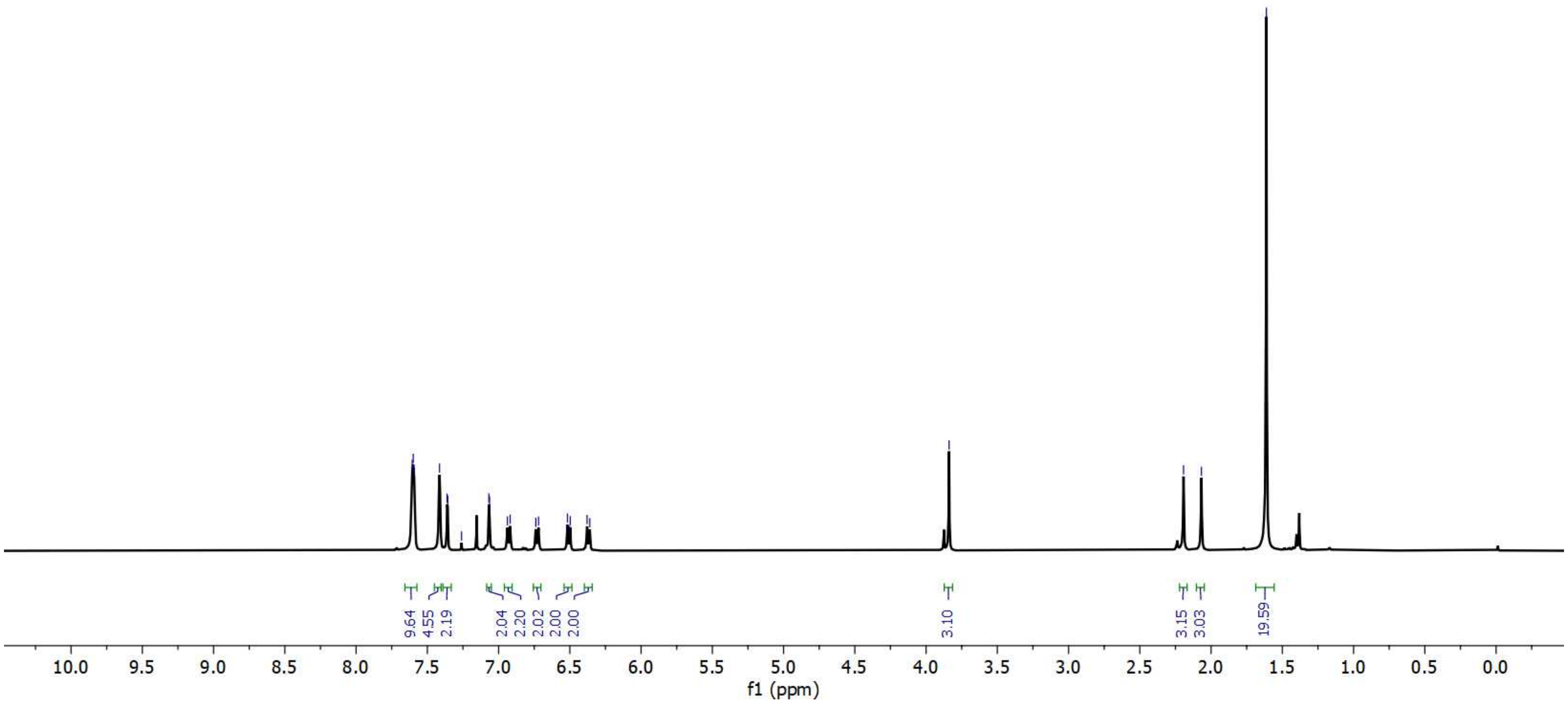

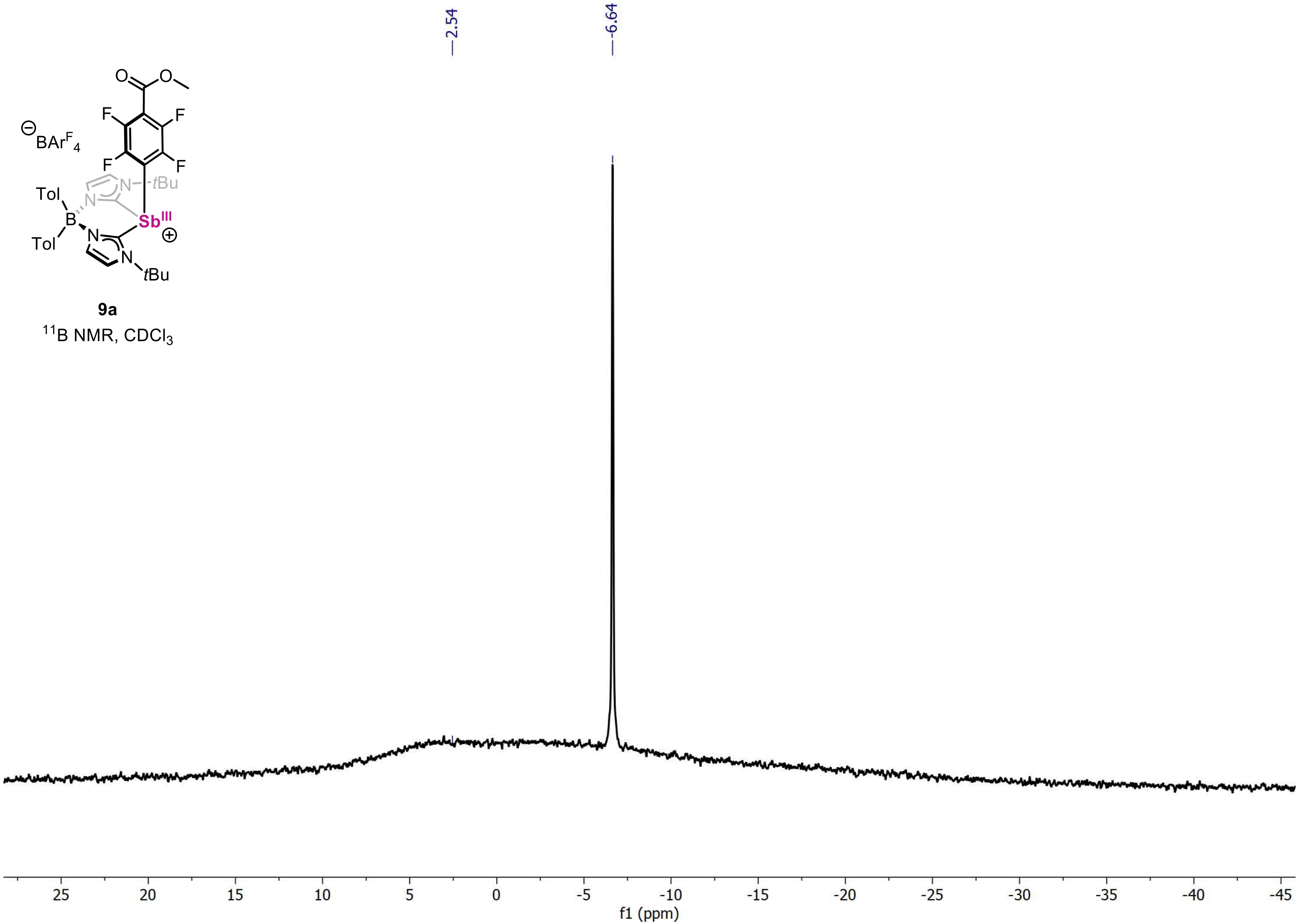


**9a**

$^{11}B$ NMR, $CDCl_3$

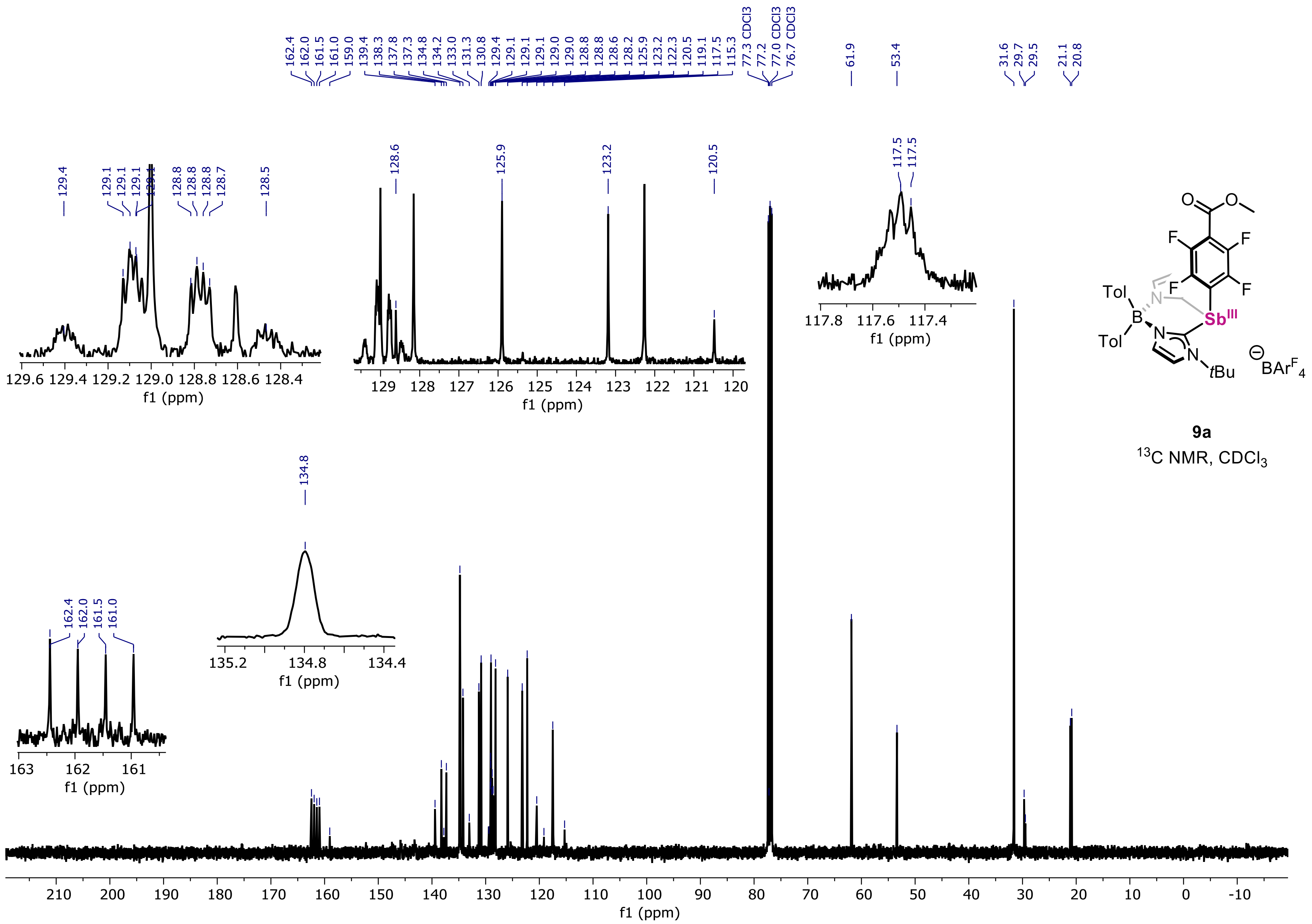


**9a**

$^{13}$C NMR, $CDCl_3$

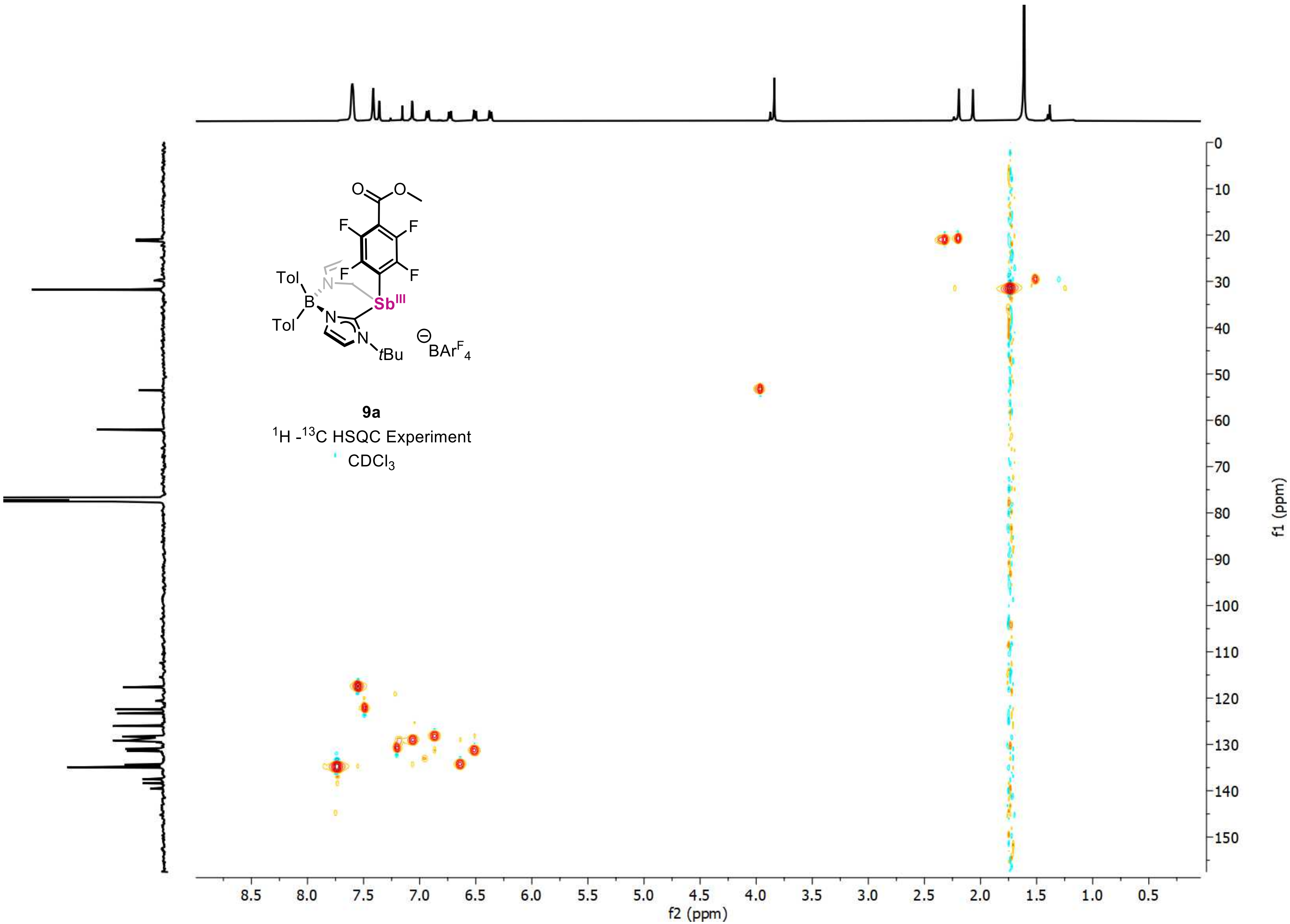

Tol
Tol
B
N
N
N
tBu
Sb^III
F
F
F
F
O
O
⊖BAr^F_4
9a
1H -13C HSQC Experiment
CDCl3
f2 (ppm)
f1 (ppm)

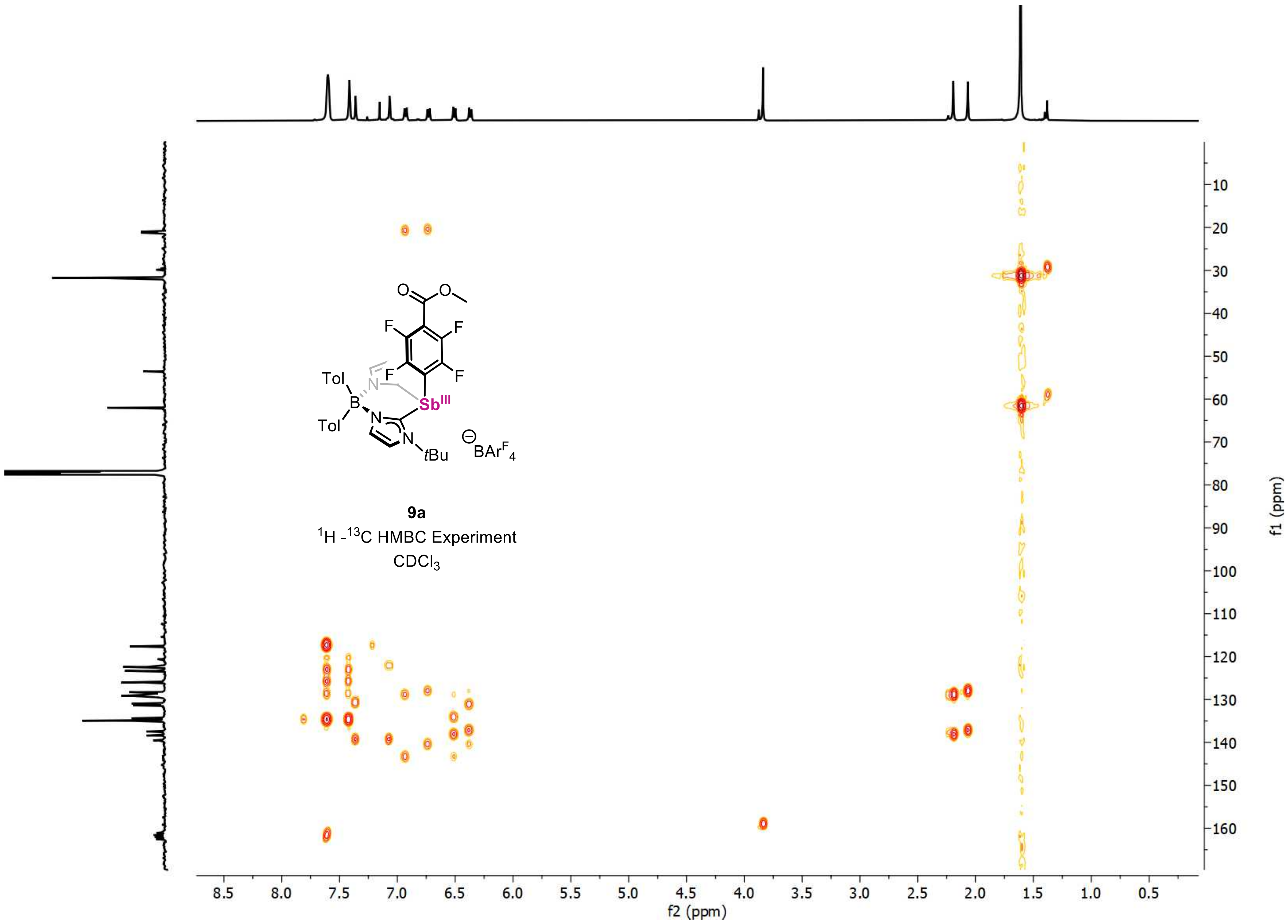
Tol
Tol
B
N
N
N
tBu
Sb^III
F
F
F
F
O
O
BAr^F_4
9a
^1H -^13C HMBC Experiment
CDCl3
f2 (ppm)
f1 (ppm)

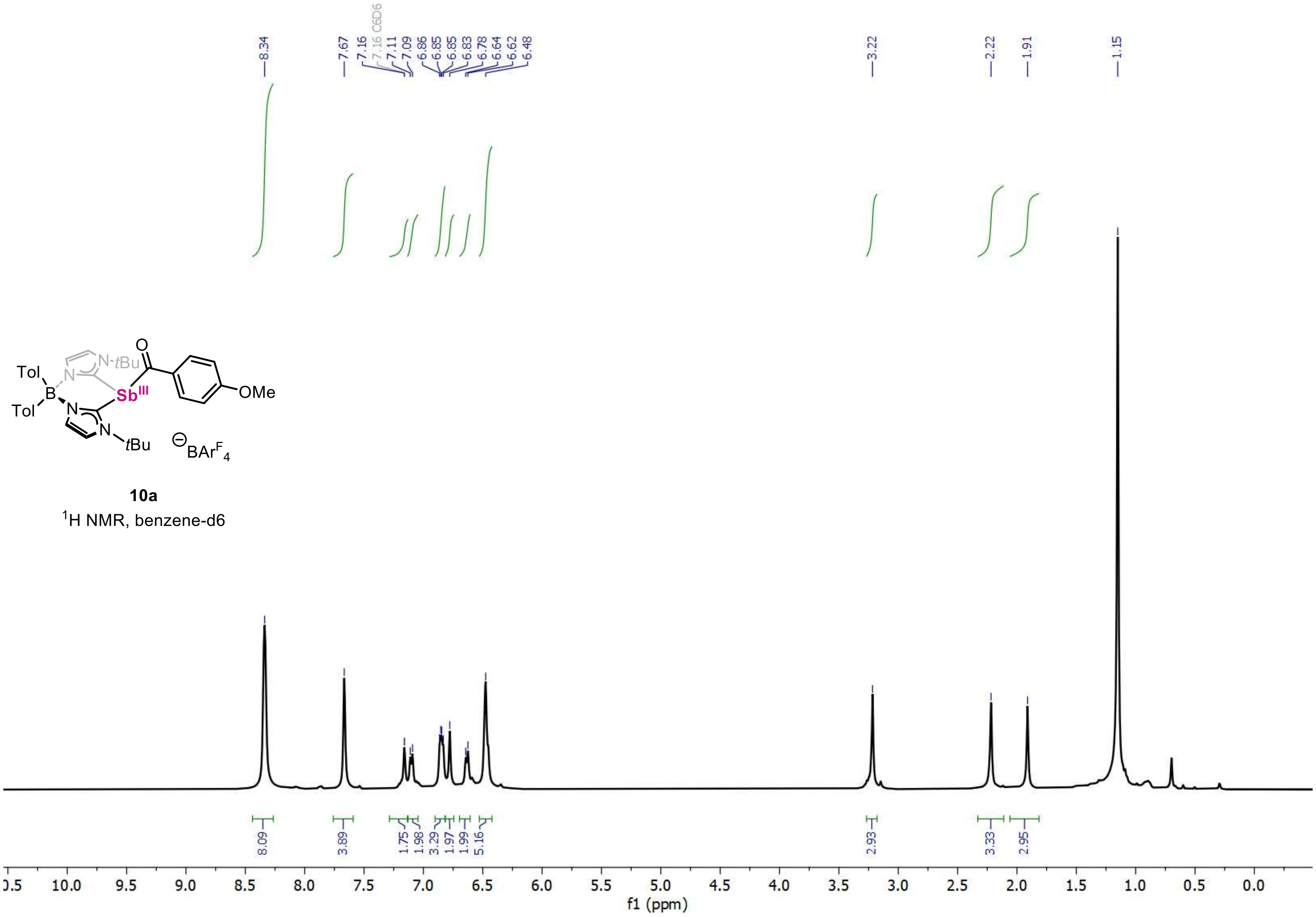


**10a**

$^1$H NMR, benzene-d6

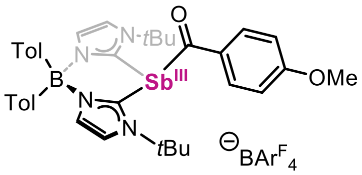


**10a**

$^{19}$F NMR, benzene-d6

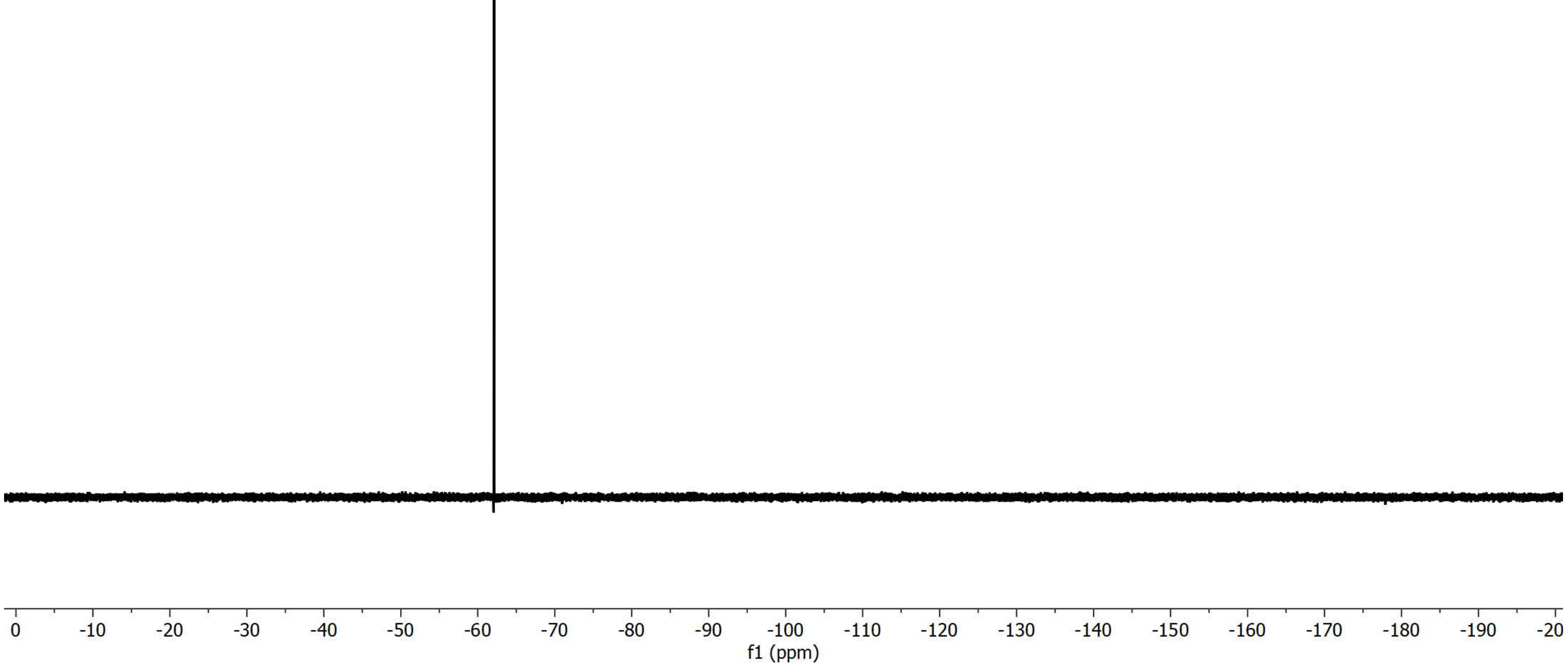

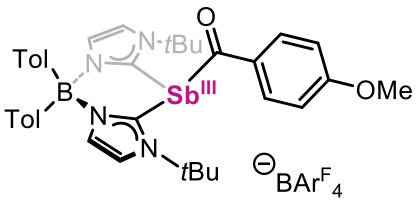


**10a**

$^{11}$B NMR, benzene-d6

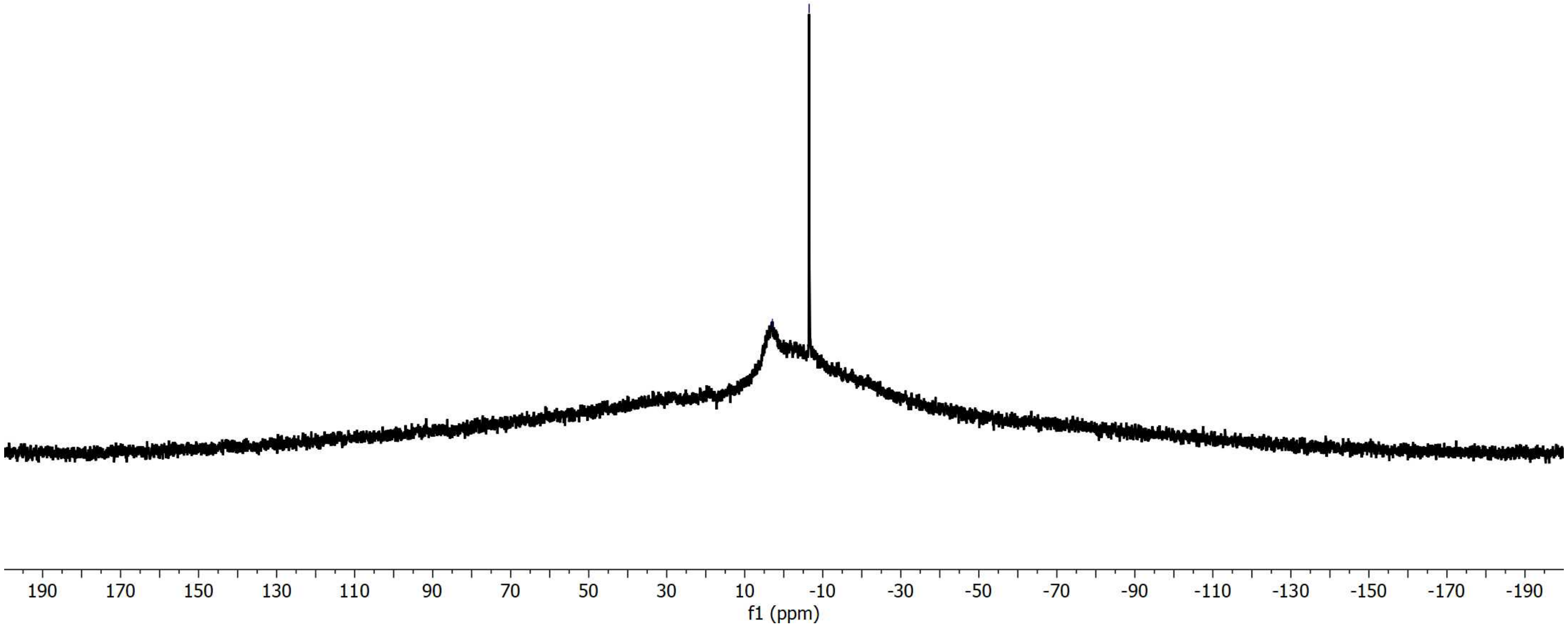

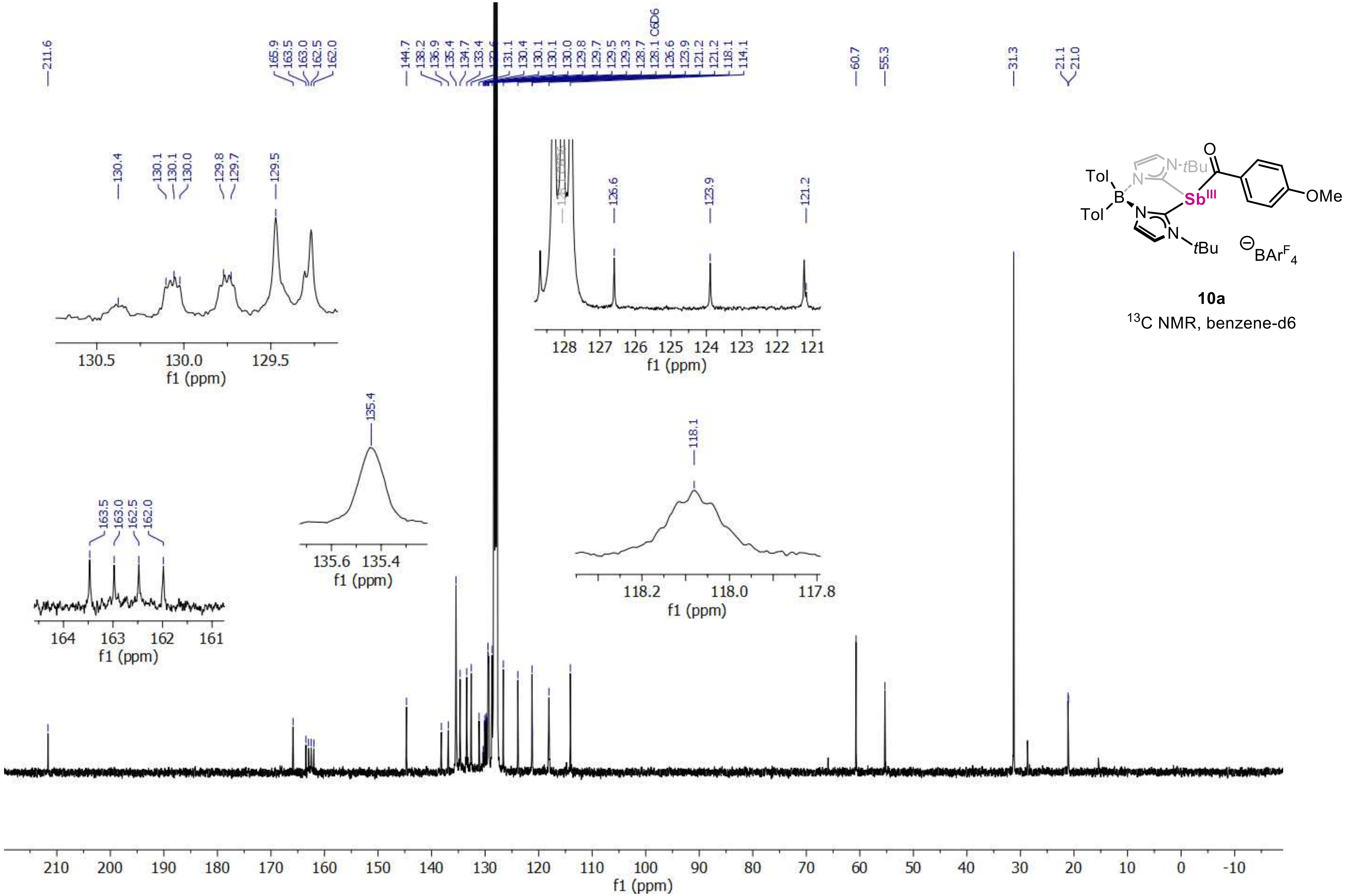


**10a**

$^{13}$C NMR, benzene-d6

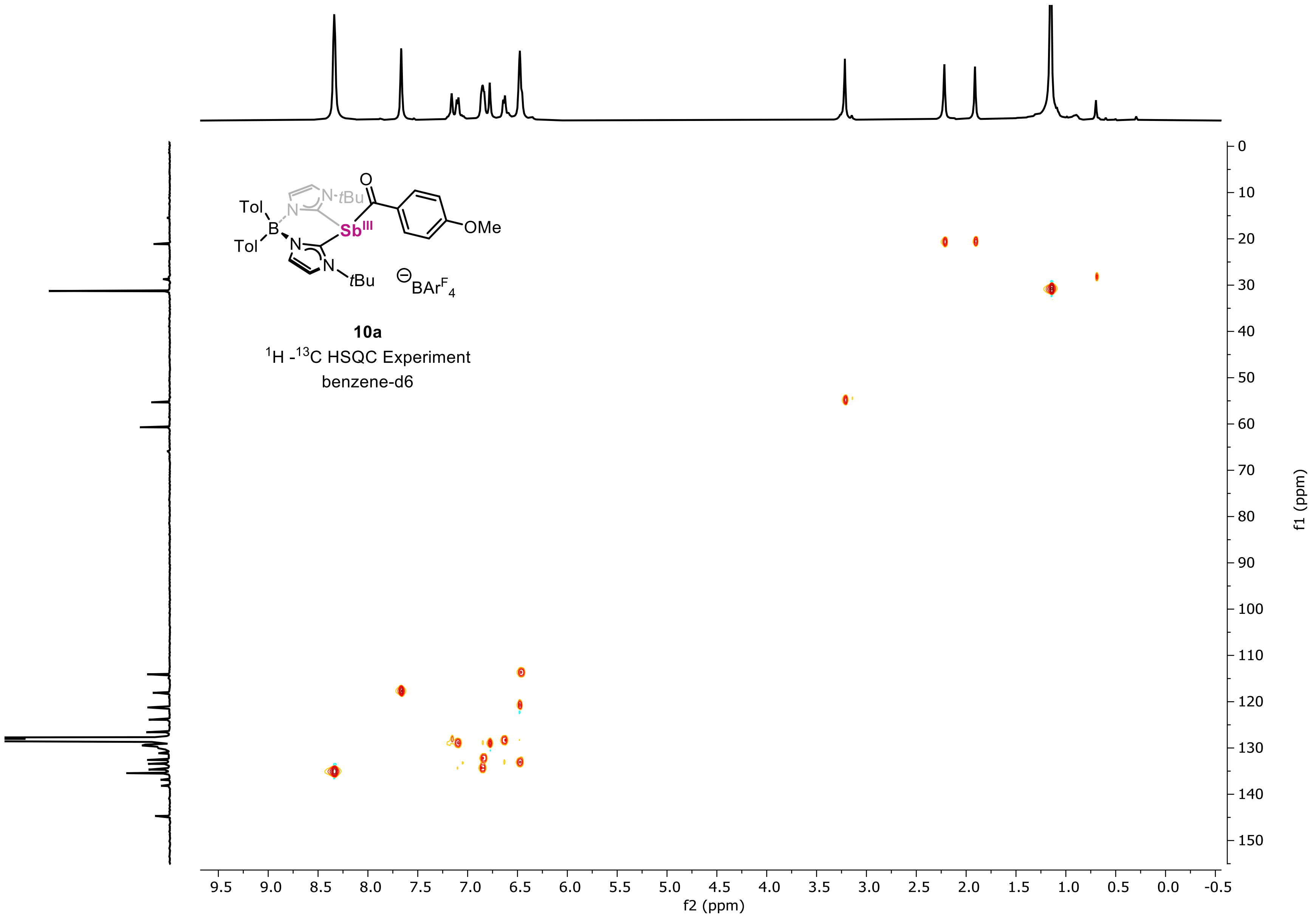

Tol
Tol
B
N
N-tBu
Sb^III
O
OMe
N
tBu
⊖BArF4
10a
1H -13C HSQC Experiment
benzene-d6
f2 (ppm)
f1 (ppm)

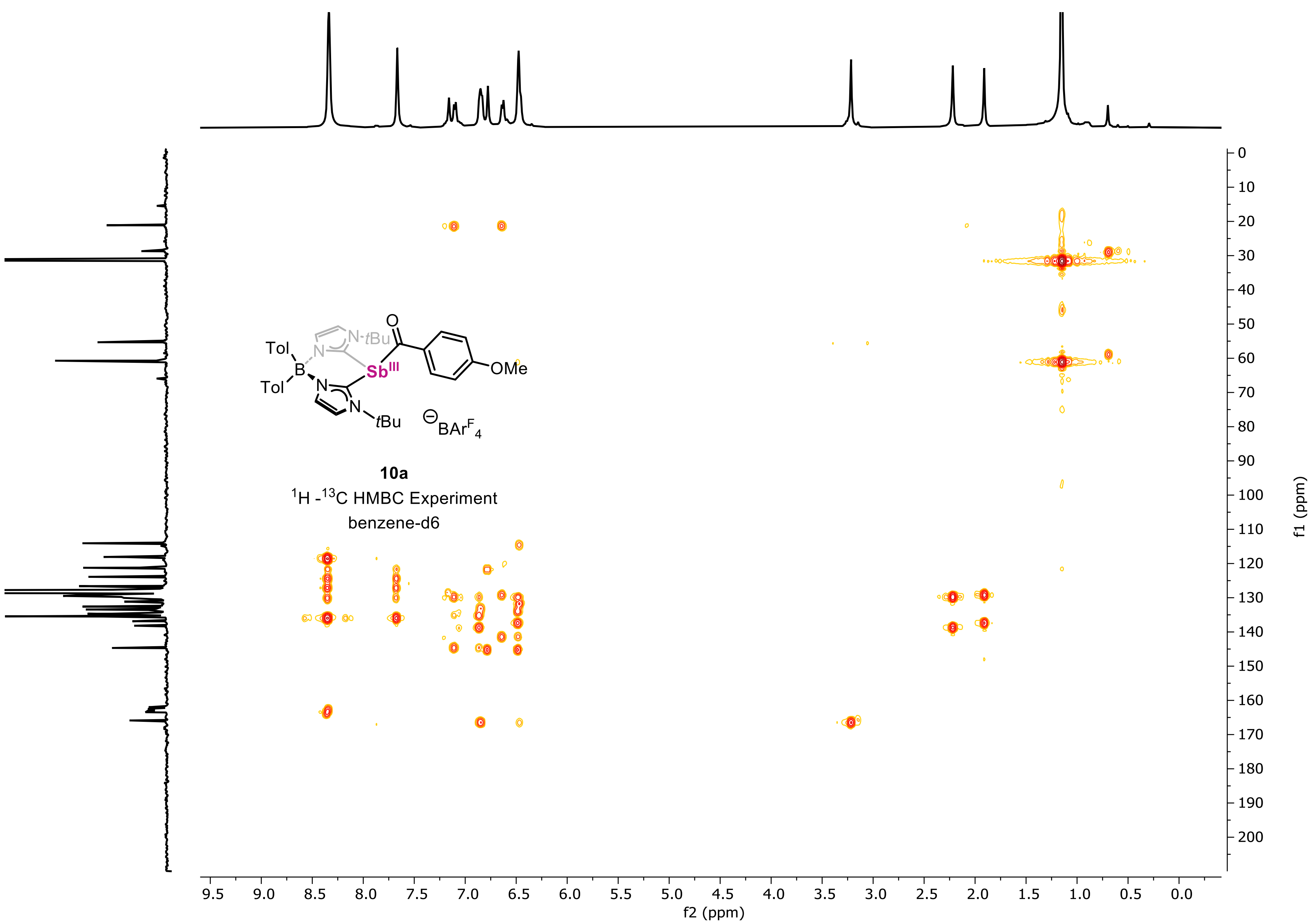
Tol
Tol
B
N
N
N-tBu
N
tBu
Sb^III
O
OMe
⊖BArF4
10a
1H -13C HMBC Experiment
benzene-d6
f1 (ppm)
f2 (ppm)

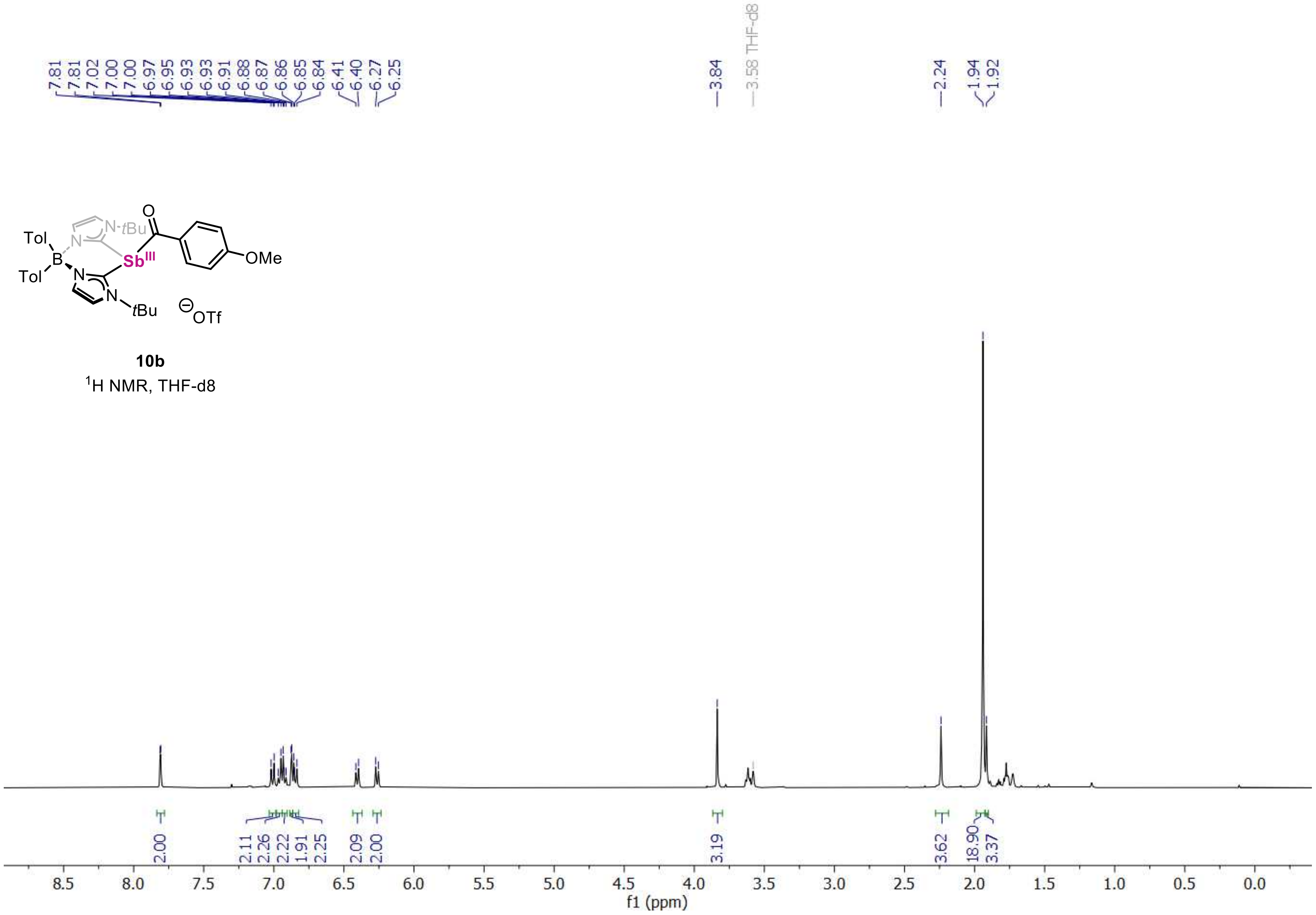


**10b**

$^{1}$H NMR, THF-d8

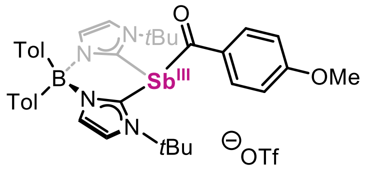


**10b**

$^{19}$F NMR, THF-d8

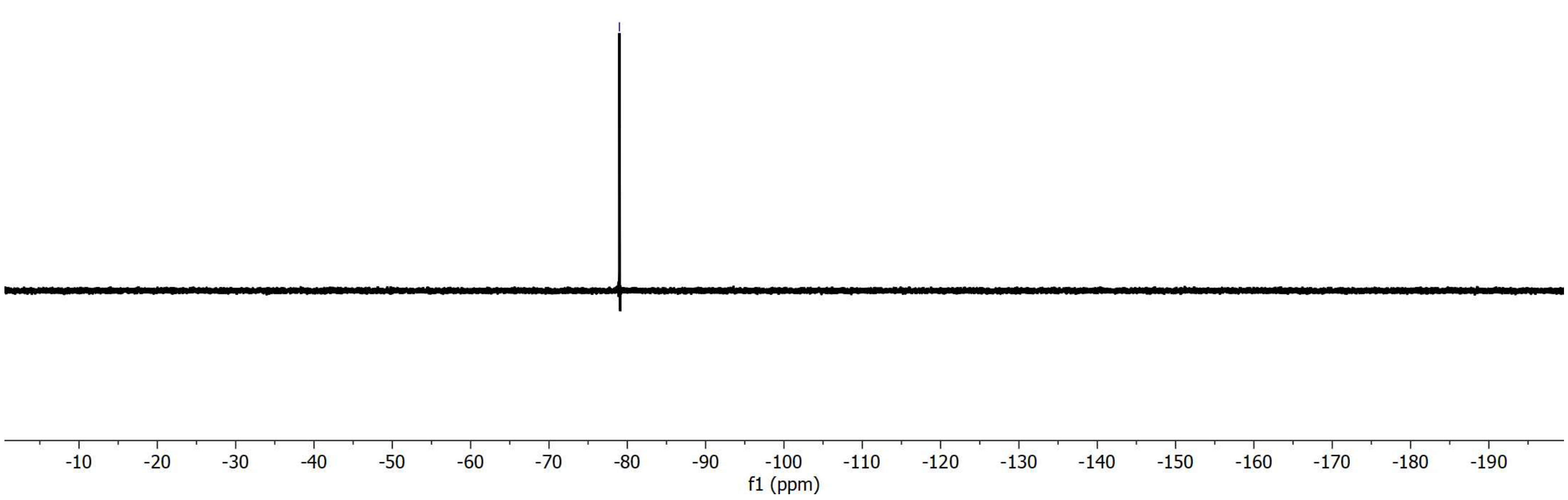

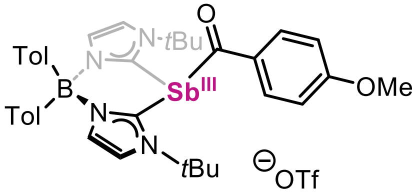


**10b**

$^{11}$B NMR, benzene-d6

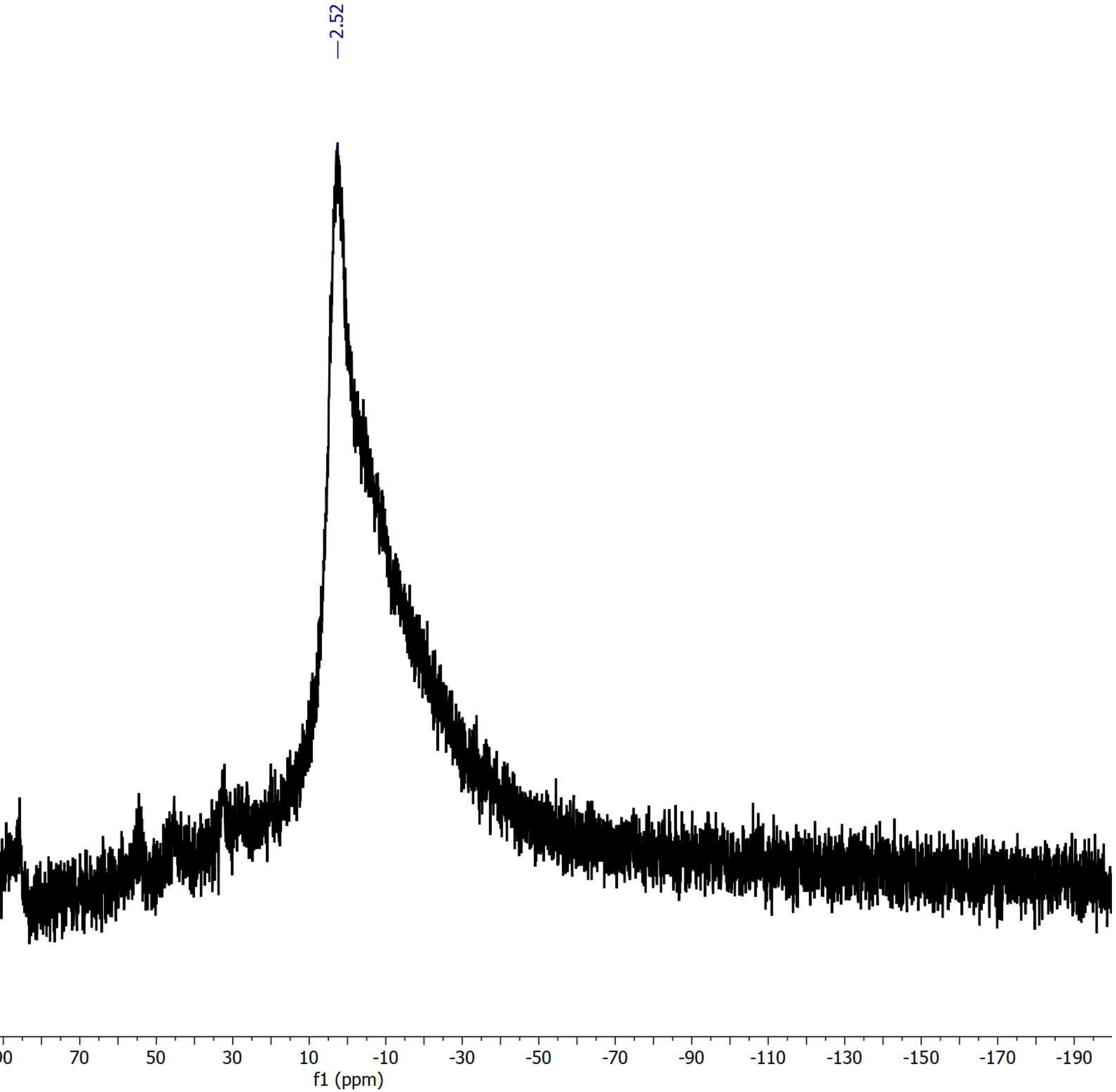

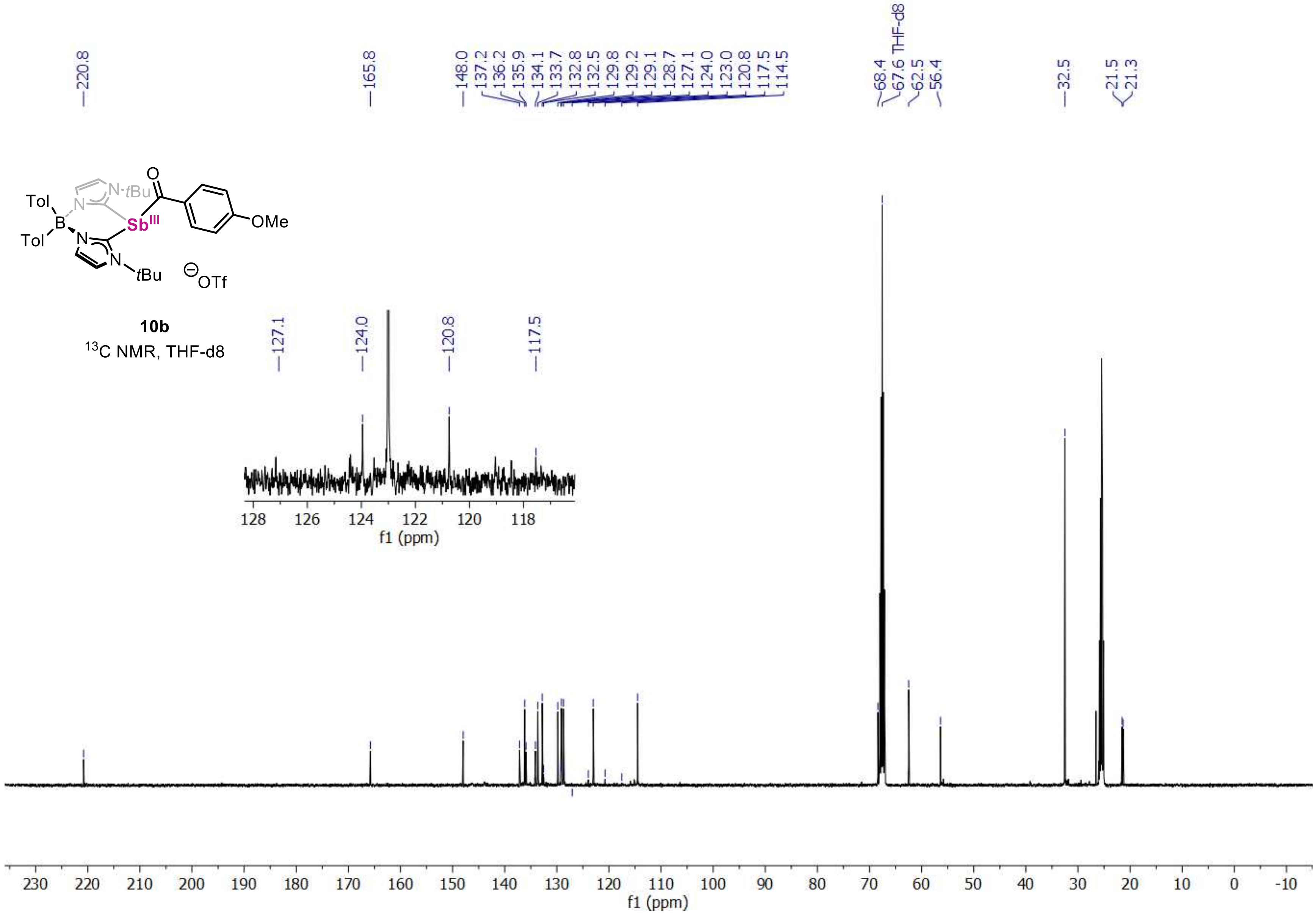

220.8
165.8
148.0
137.2
136.2
135.9
134.1
133.7
132.8
132.5
129.8
129.2
129.1
128.7
127.1
124.0
123.0
120.8
117.5
114.5
68.4
67.6 THF-d8
62.5
56.4
32.5
21.5
21.3
Tol
Tol
B
N
N-tBu
N
N
tBu
O
Sb^III
OMe
⊖OTf
10b
13C NMR, THF-d8
127.1
124.0
120.8
117.5
128
126
124
122
120
118
f1 (ppm)
230
220
210
200
190
180
170
160
150
140
130
120
110
100
90
80
70
60
50
40
30
20
10
0
-10
f1 (ppm)

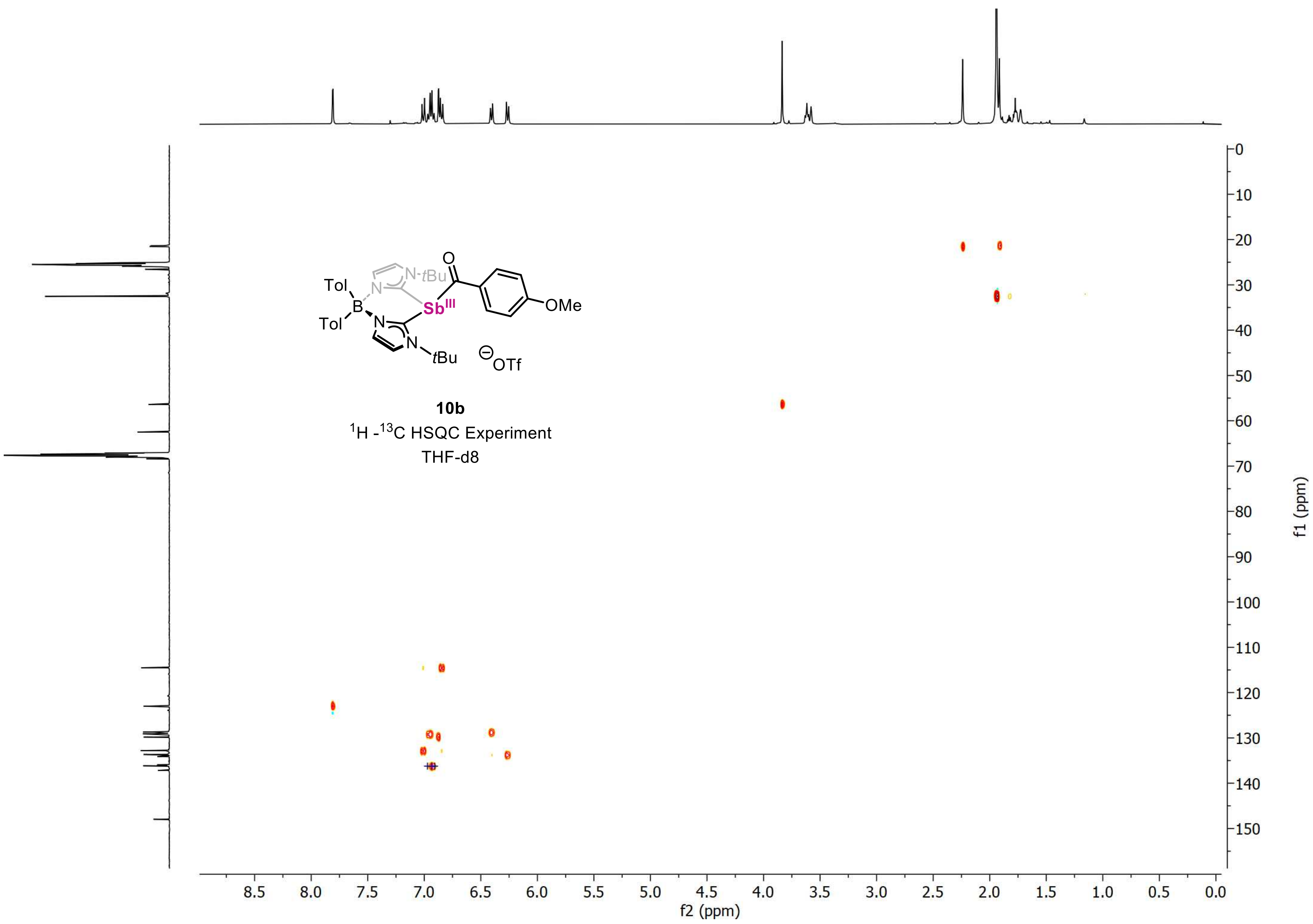
10b
$^{1}H$ -$^{13}C$ HSQC Experiment
THF-d8
Tol
Tol
B
N
N
N-tBu
Sb^III
O
OMe
N
tBu
⊖OTf
f2 (ppm)
f1 (ppm)

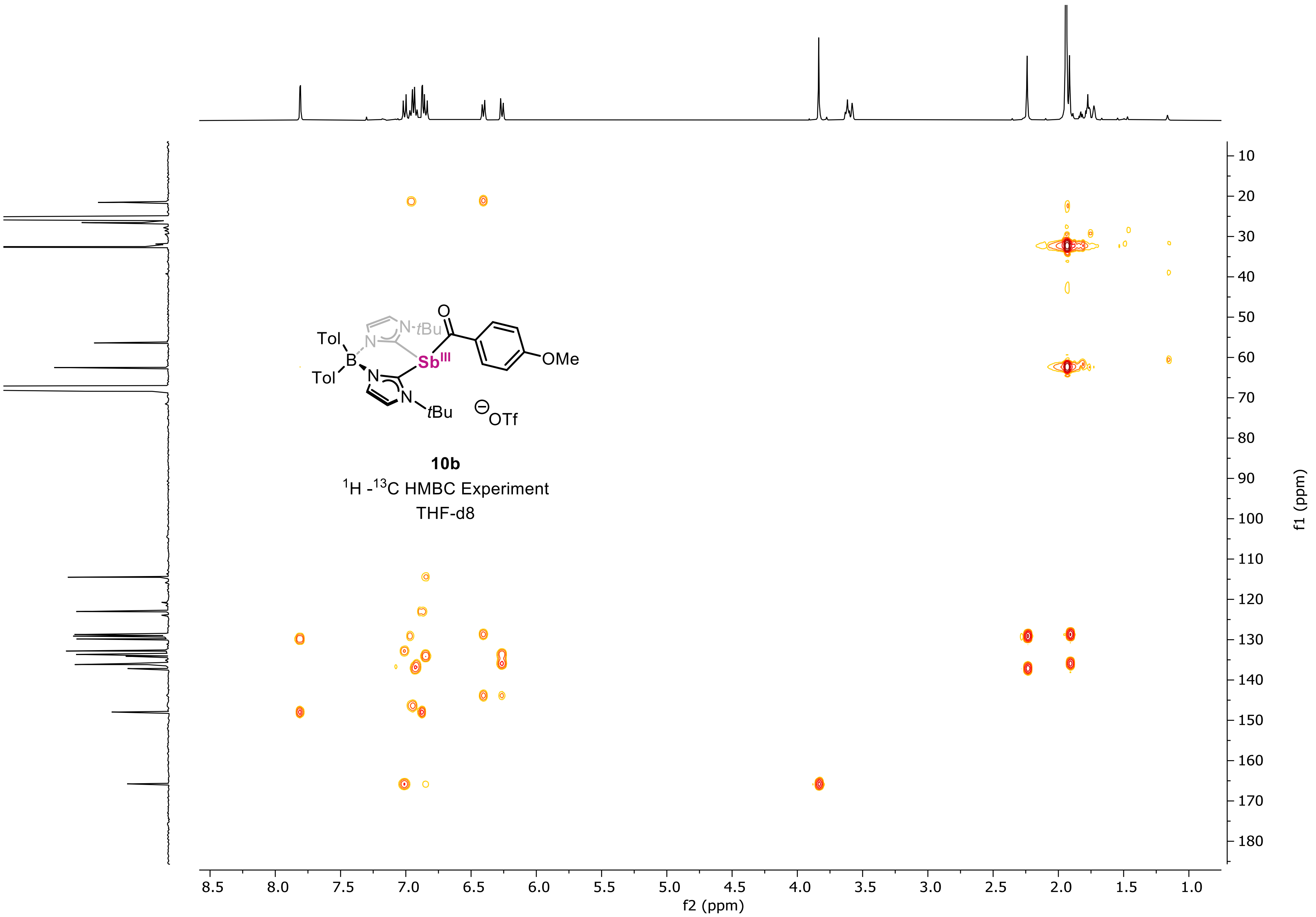


**10b**

$^{1}H$ -$^{13}C$ HMBC Experiment

THF-d8

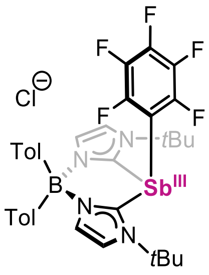


**16a**

$^{1}$H NMR, THF-d8

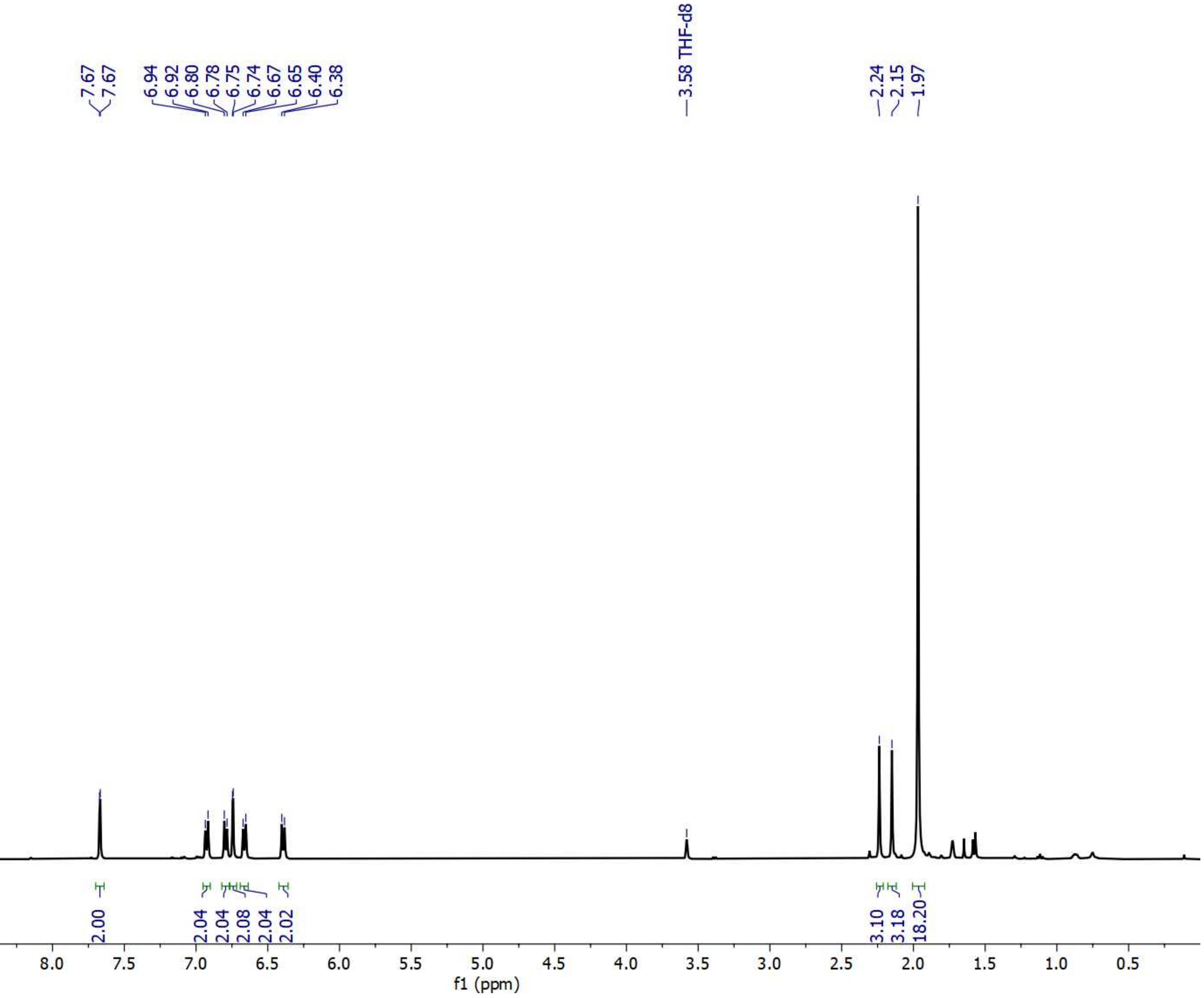

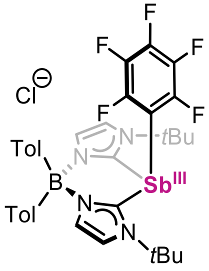


**16a**

$^{19}F$ NMR, THF-d8

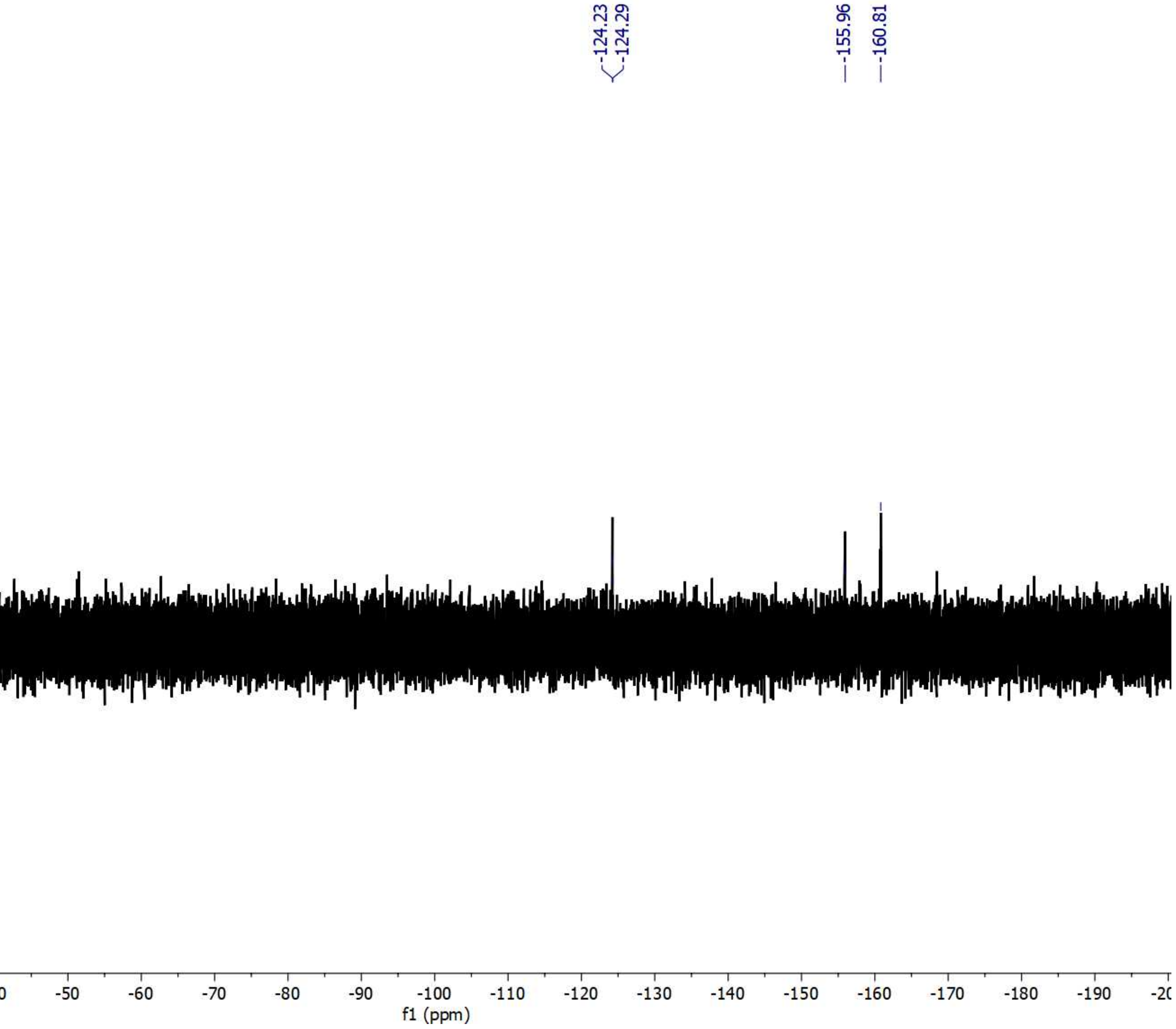

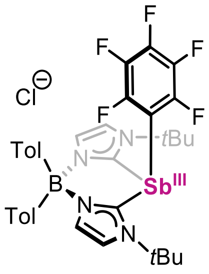


**16a**

$^{11}$B NMR, THF-d8

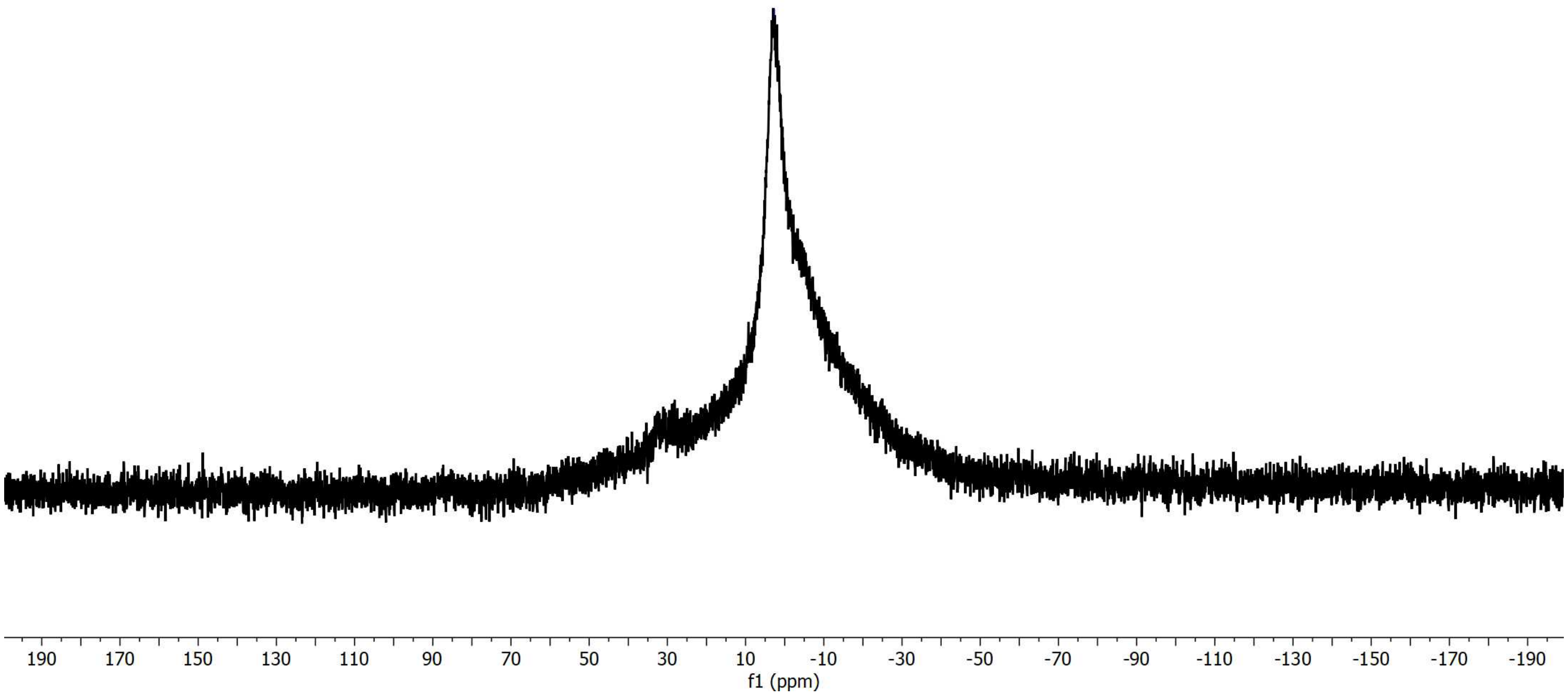

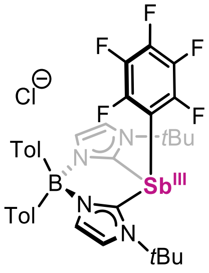


**16a**

$^{13}$C NMR, THF-d8

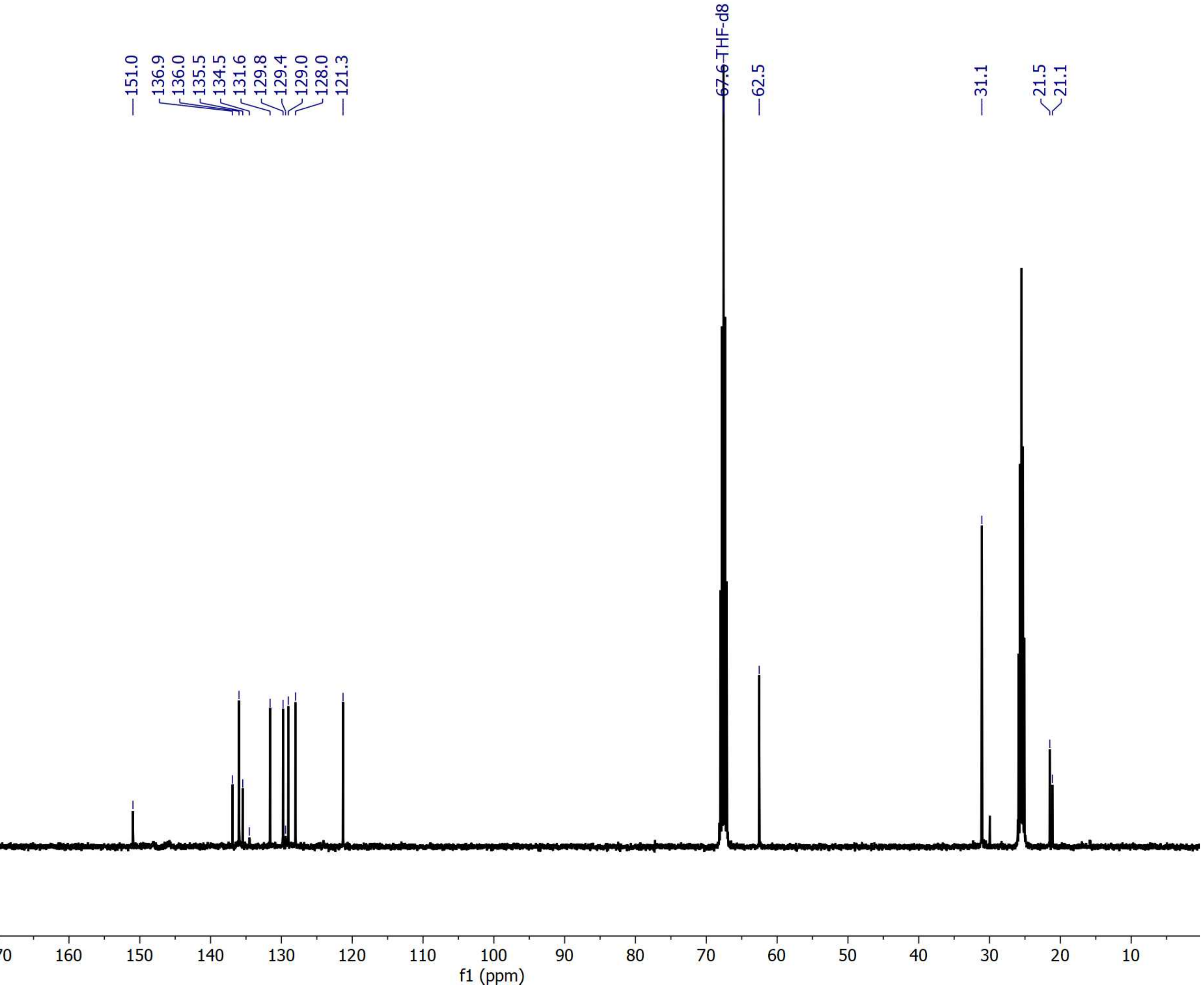

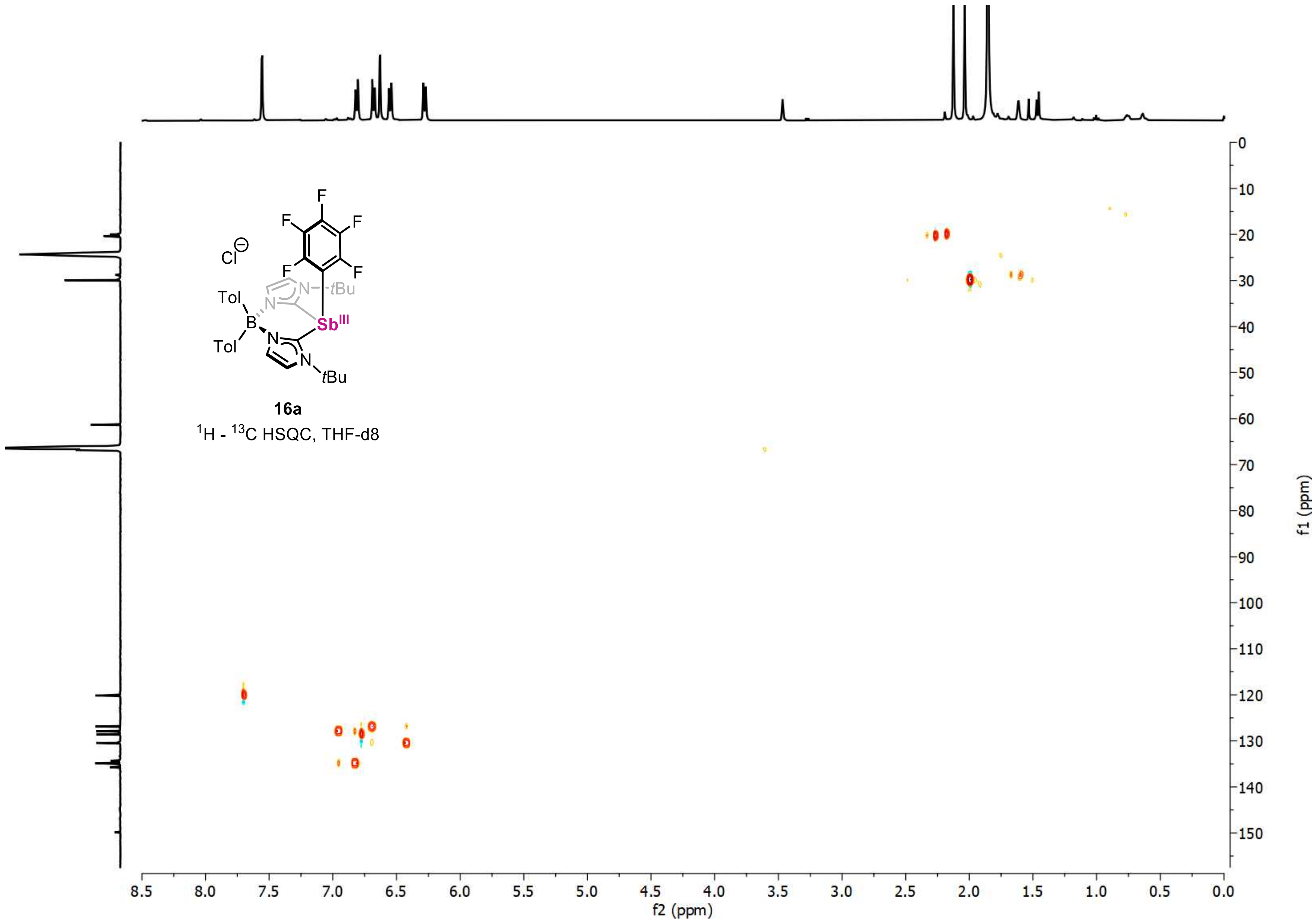

16a
$^{1}$H - $^{13}$C HSQC, THF-d8
f2 (ppm)
f1 (ppm)

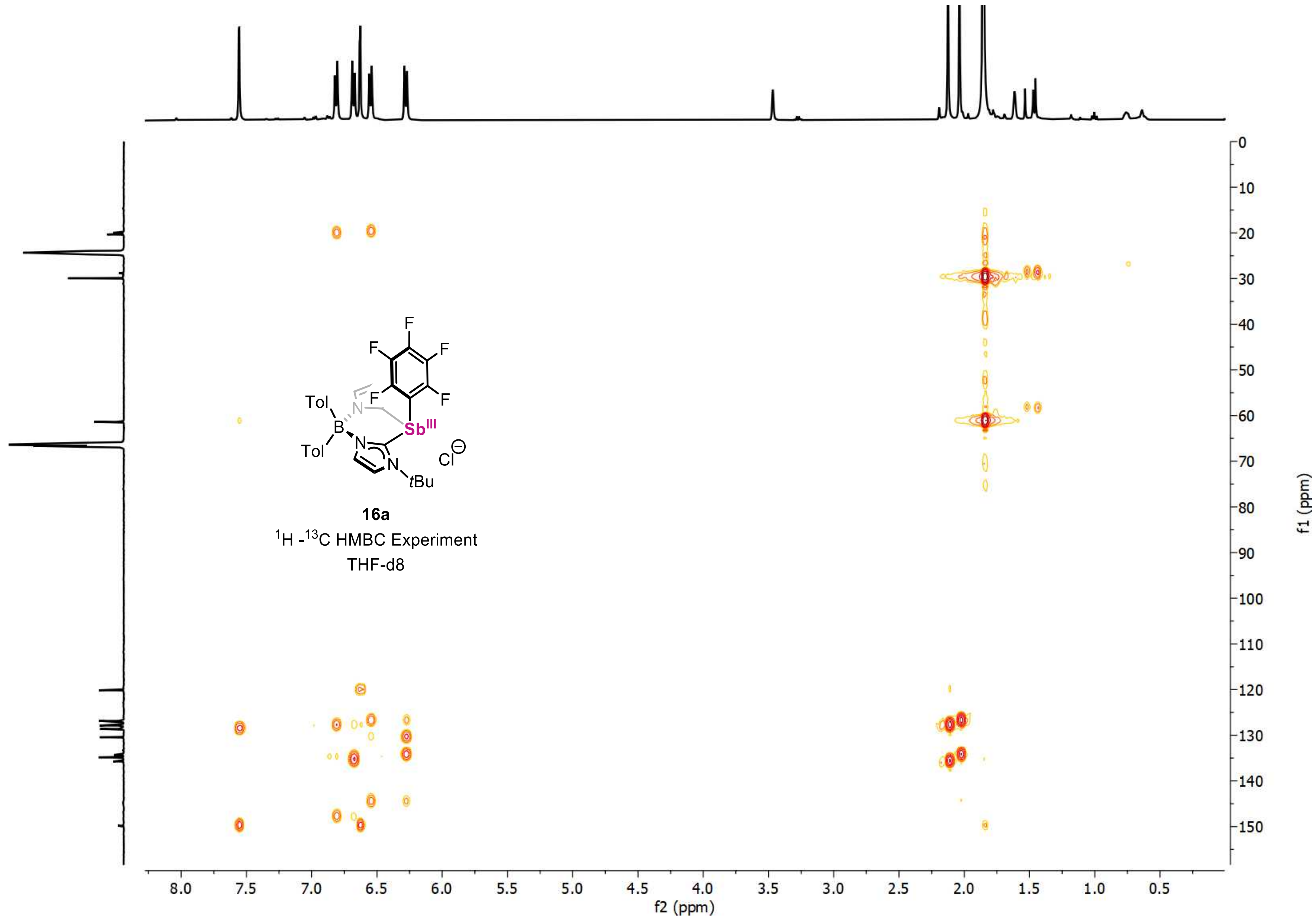
F
F
F
F
F
Tol
B
N
Sb^III
Tol
N
N
tBu
Cl⊖
16a
1H -13C HMBC Experiment
THF-d8
f2 (ppm)
f1 (ppm)

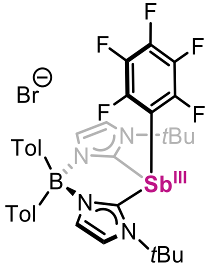


**16b**

$^{1}$H NMR, $CDCl_3$

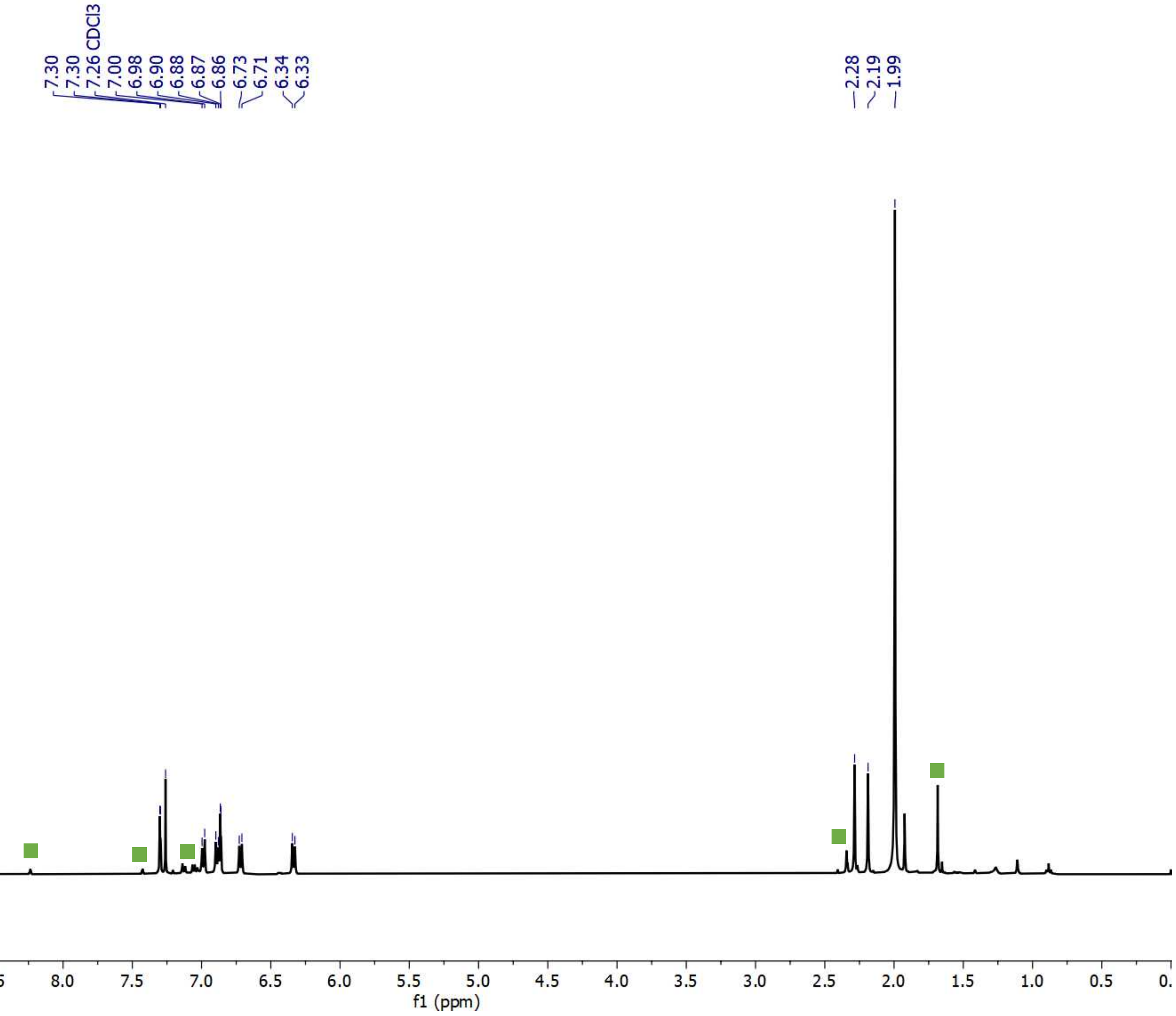

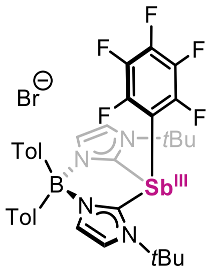

**16b**

$^{19}$F NMR, $CDCl_3$

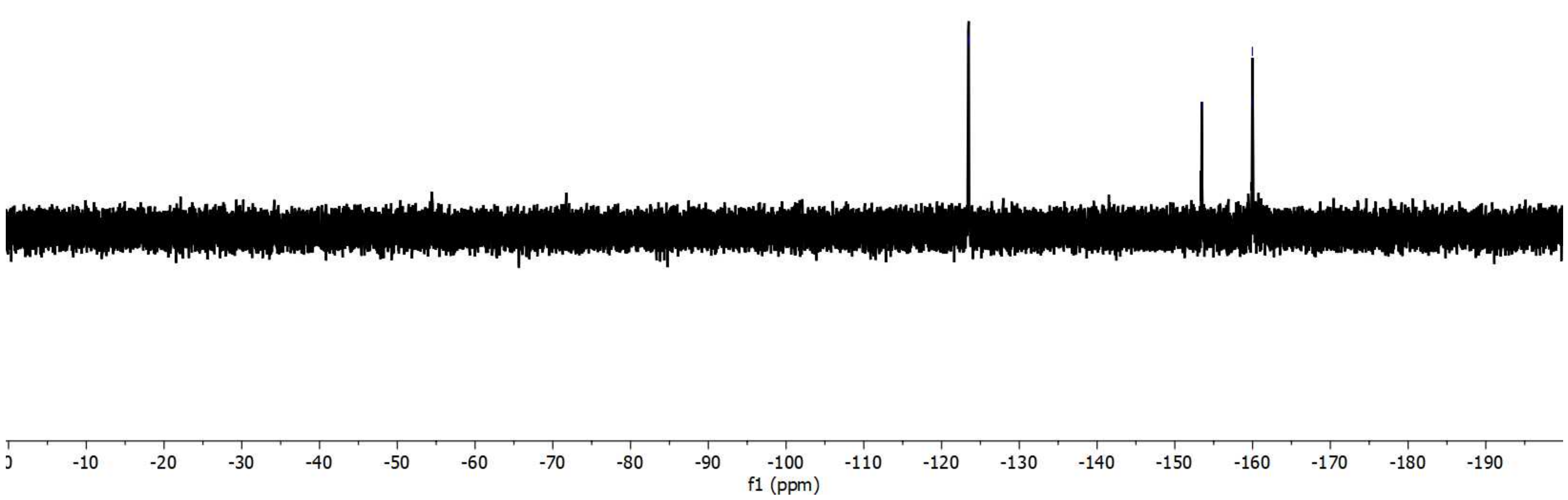

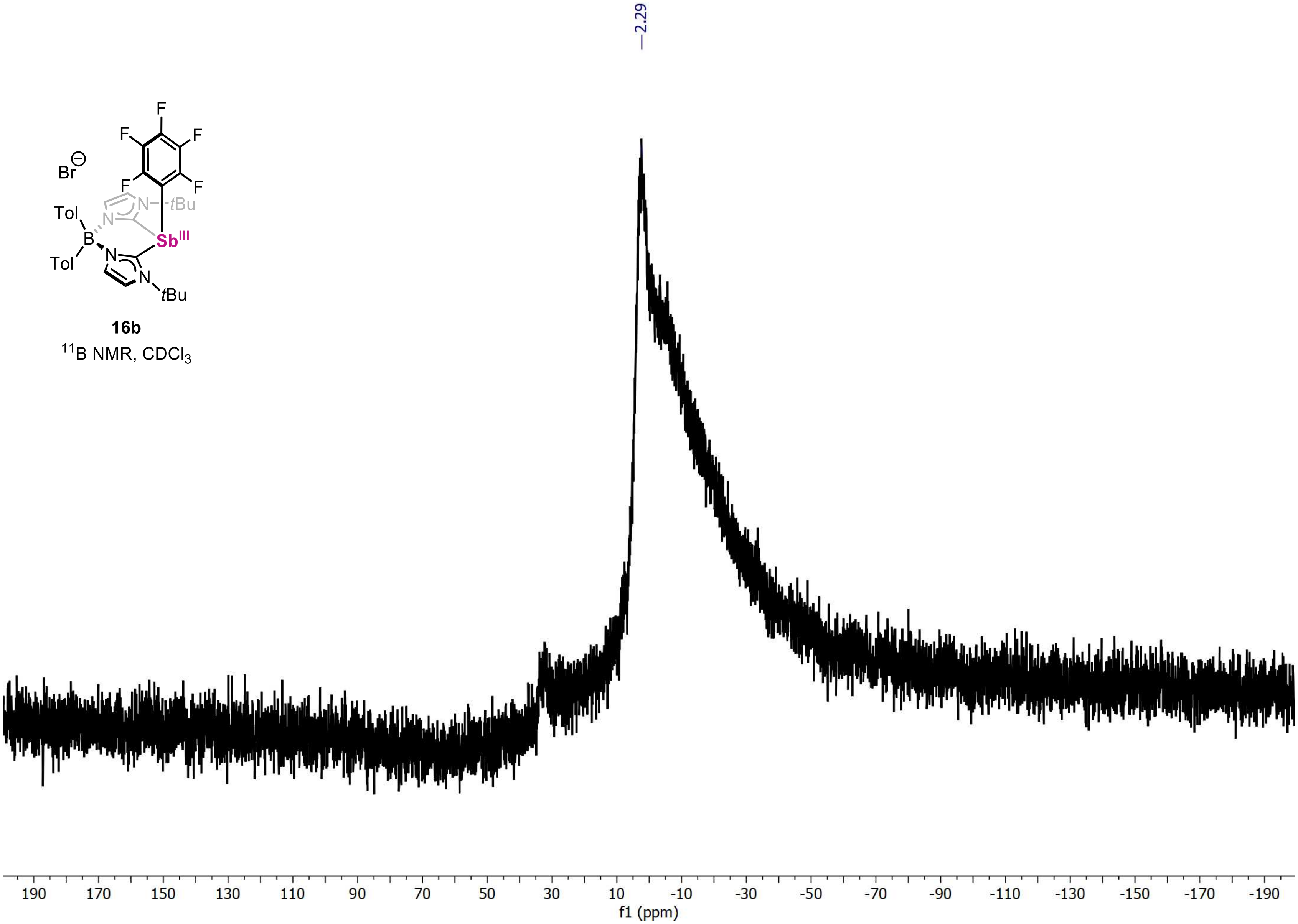


**16b**

$^{11}B$ NMR, $CDCl_3$

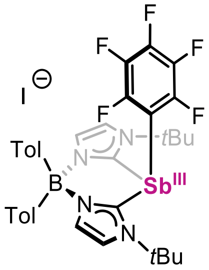


**16c**

$^1$H NMR, $CDCl_3$

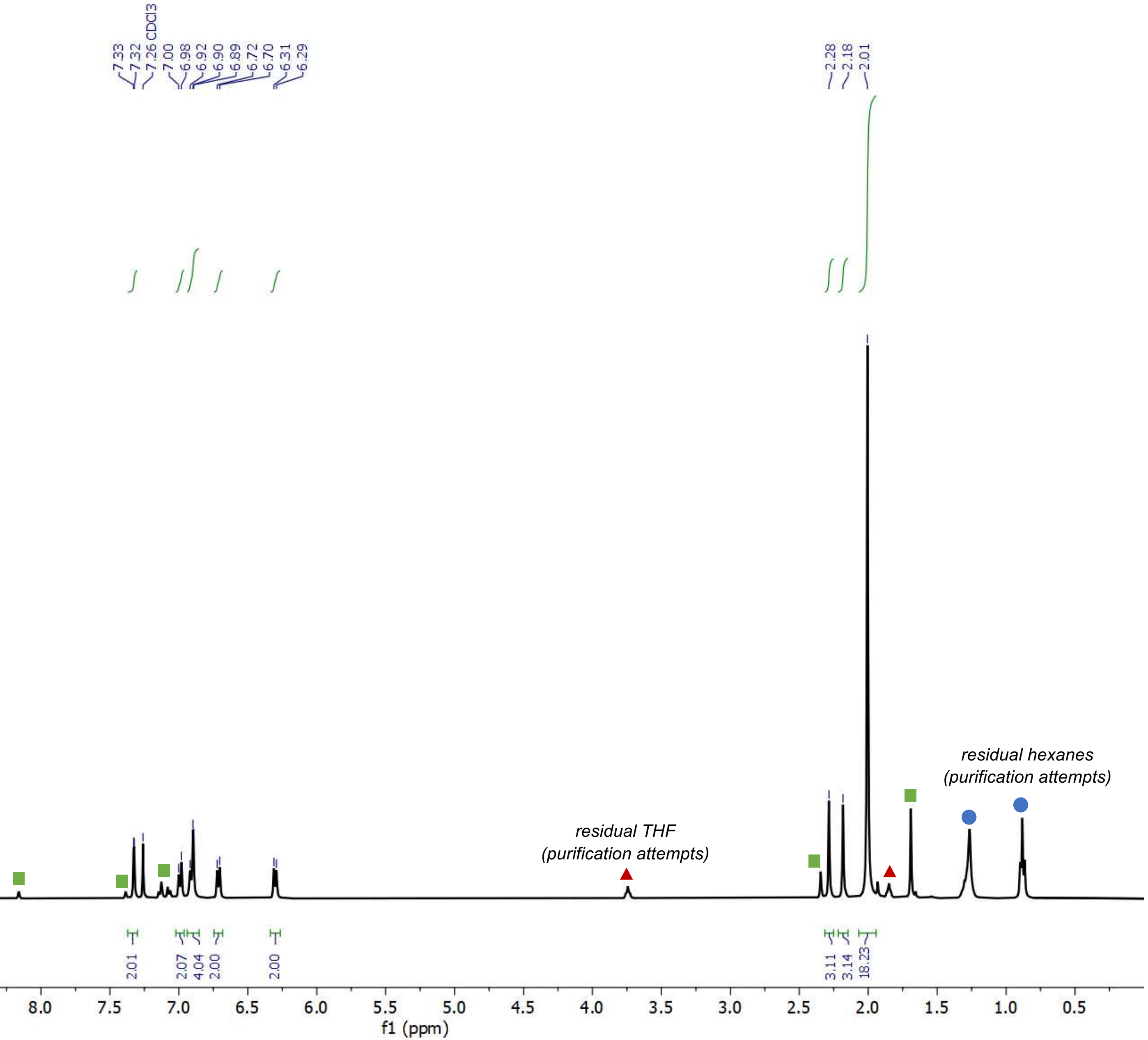

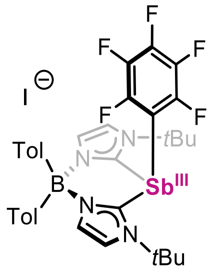


**16c**

$^{19}$F NMR, $CDCl_3$

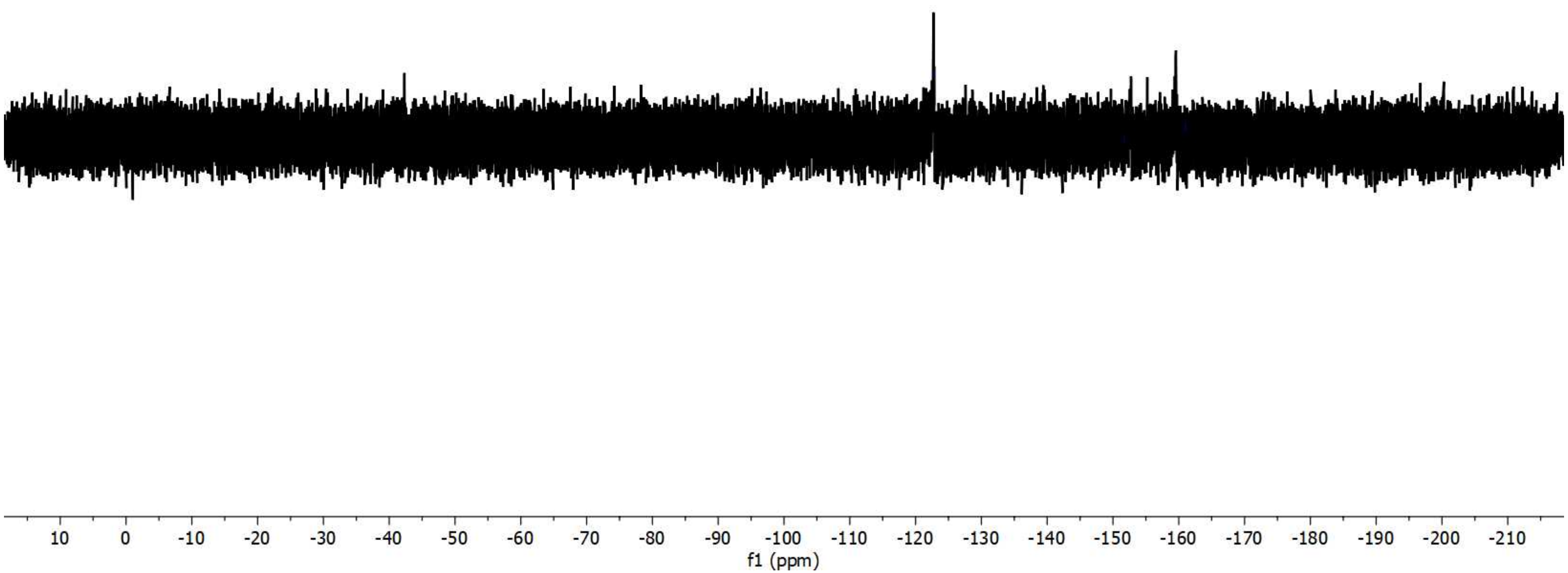

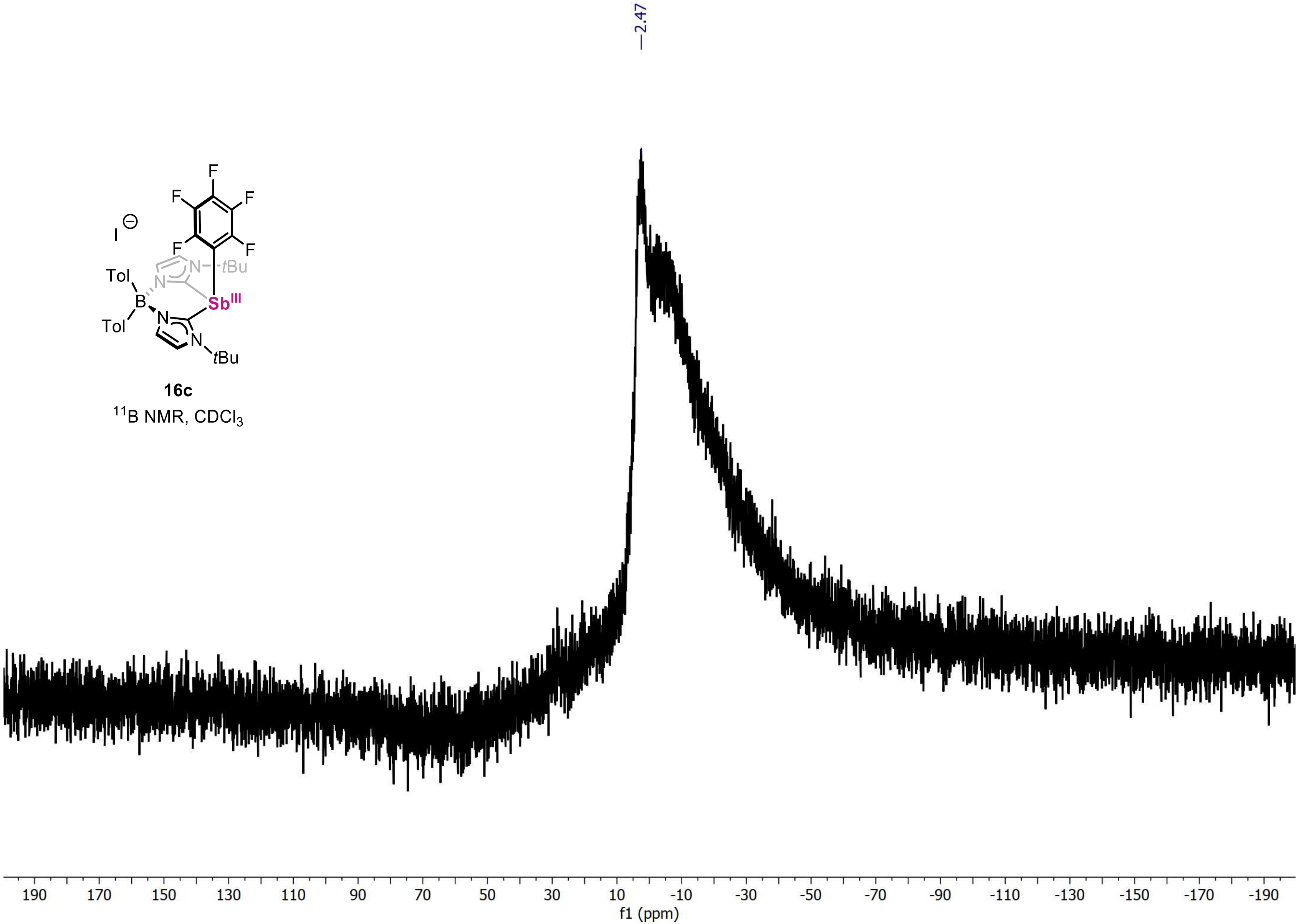


**16c**

$^{11}$B NMR, $CDCl_3$

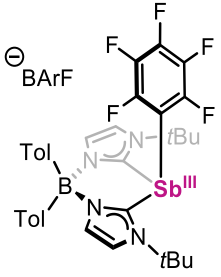


**16d**

$^{1}$H NMR, $C_6D_6$

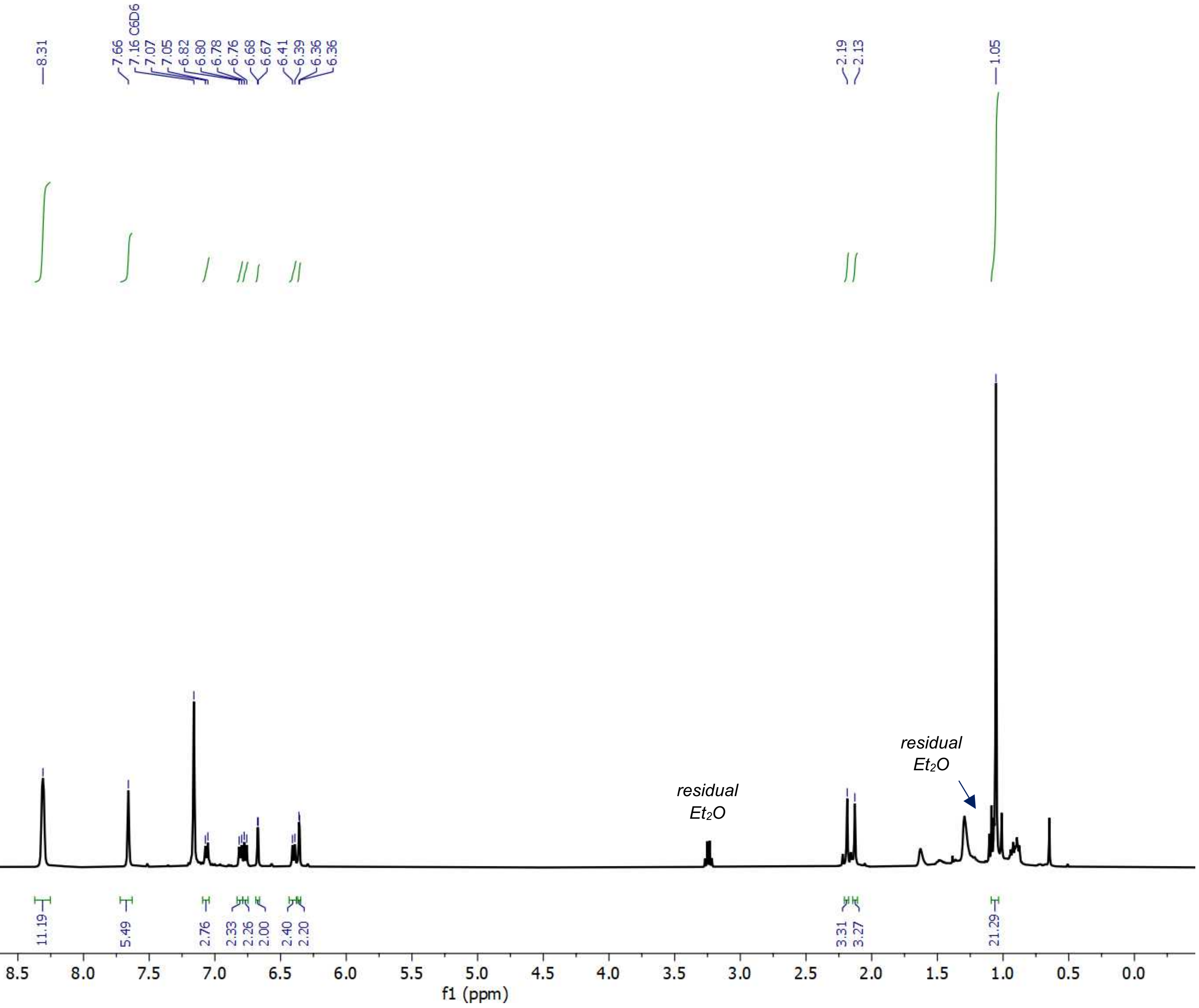

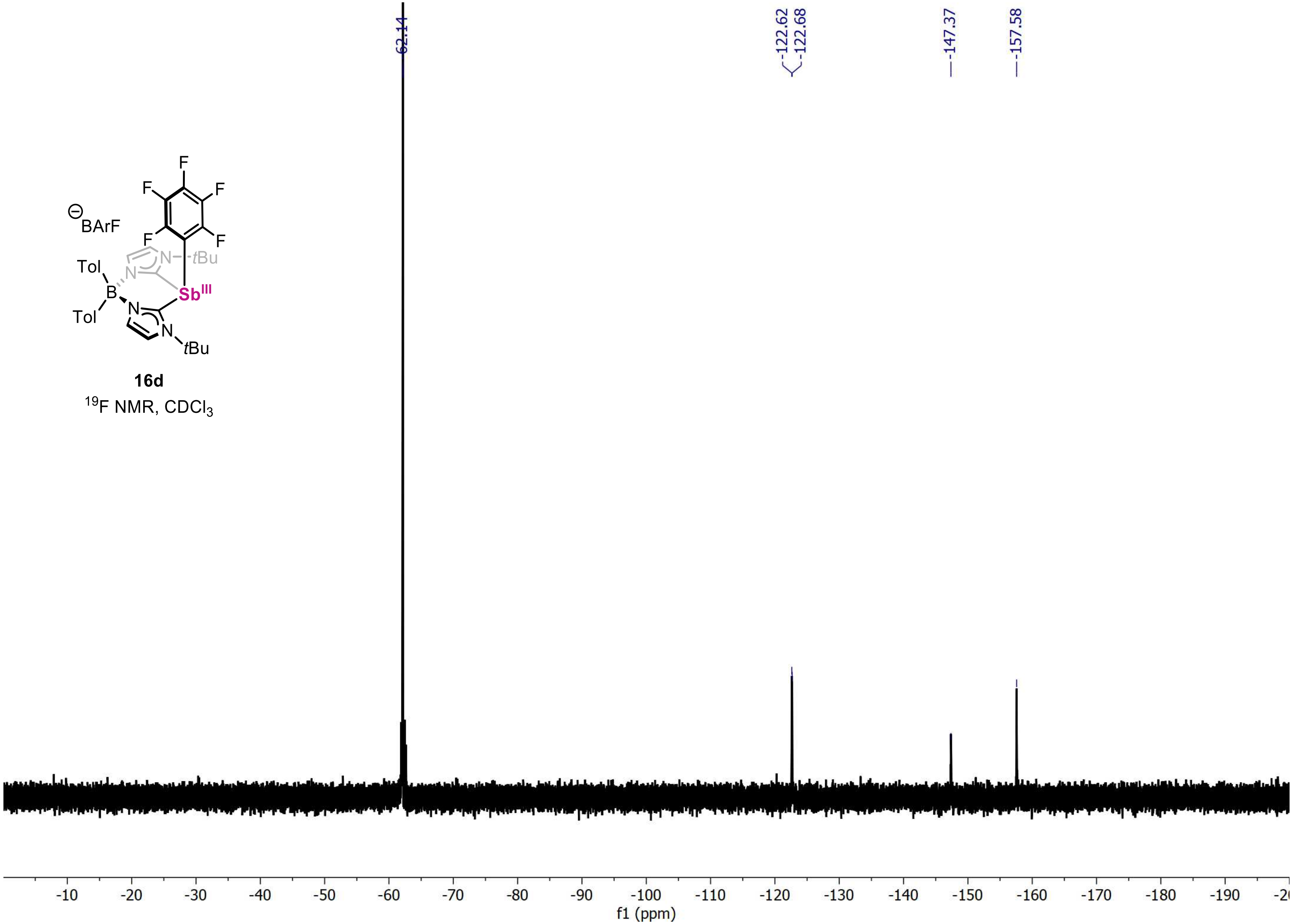


**16d**

$^{19}F$ NMR, $CDCl_3$

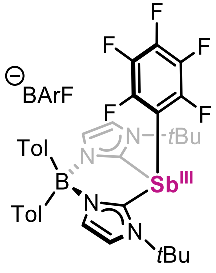


**16d**

$^{11}$B NMR, $CDCl_3$

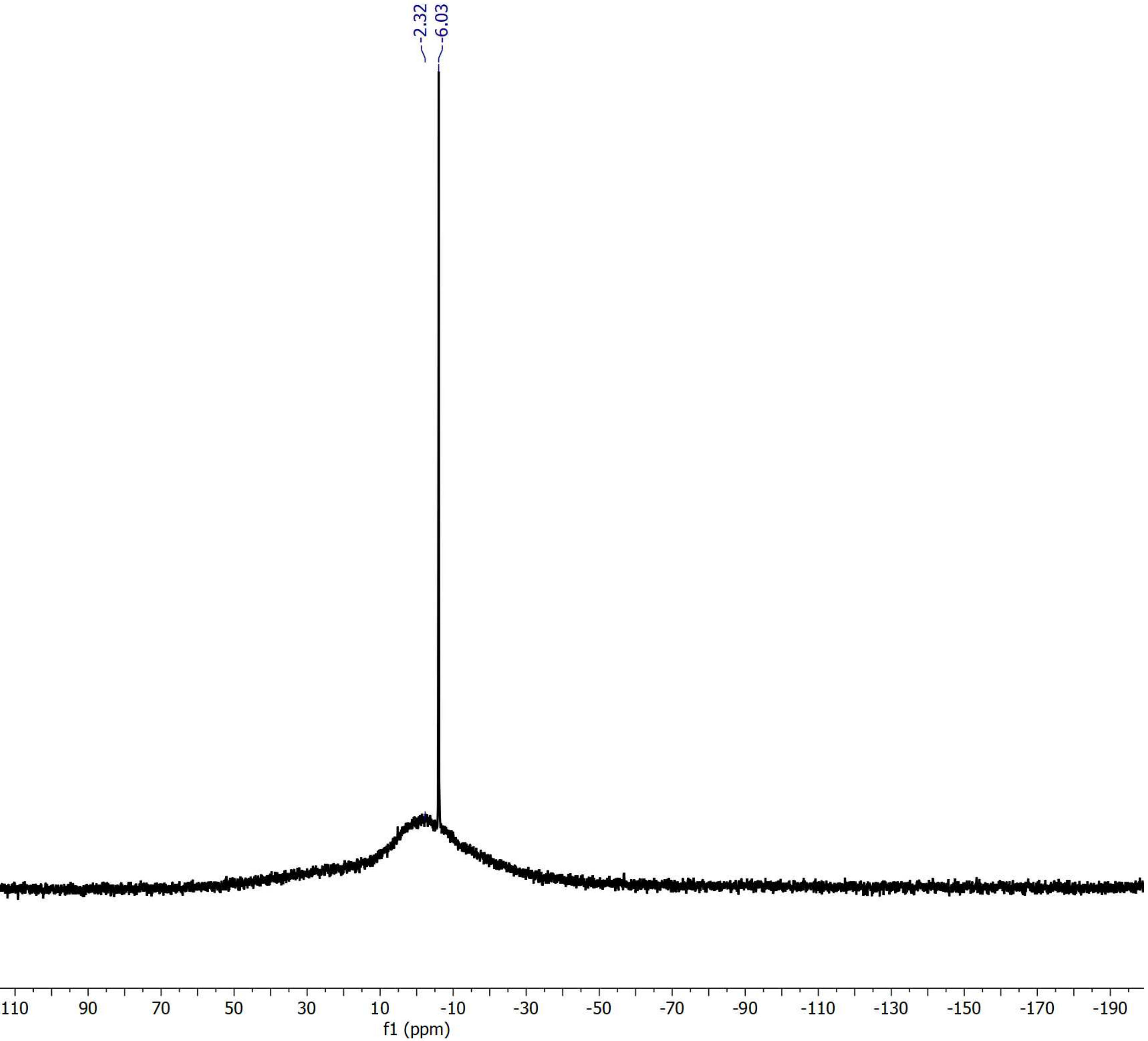

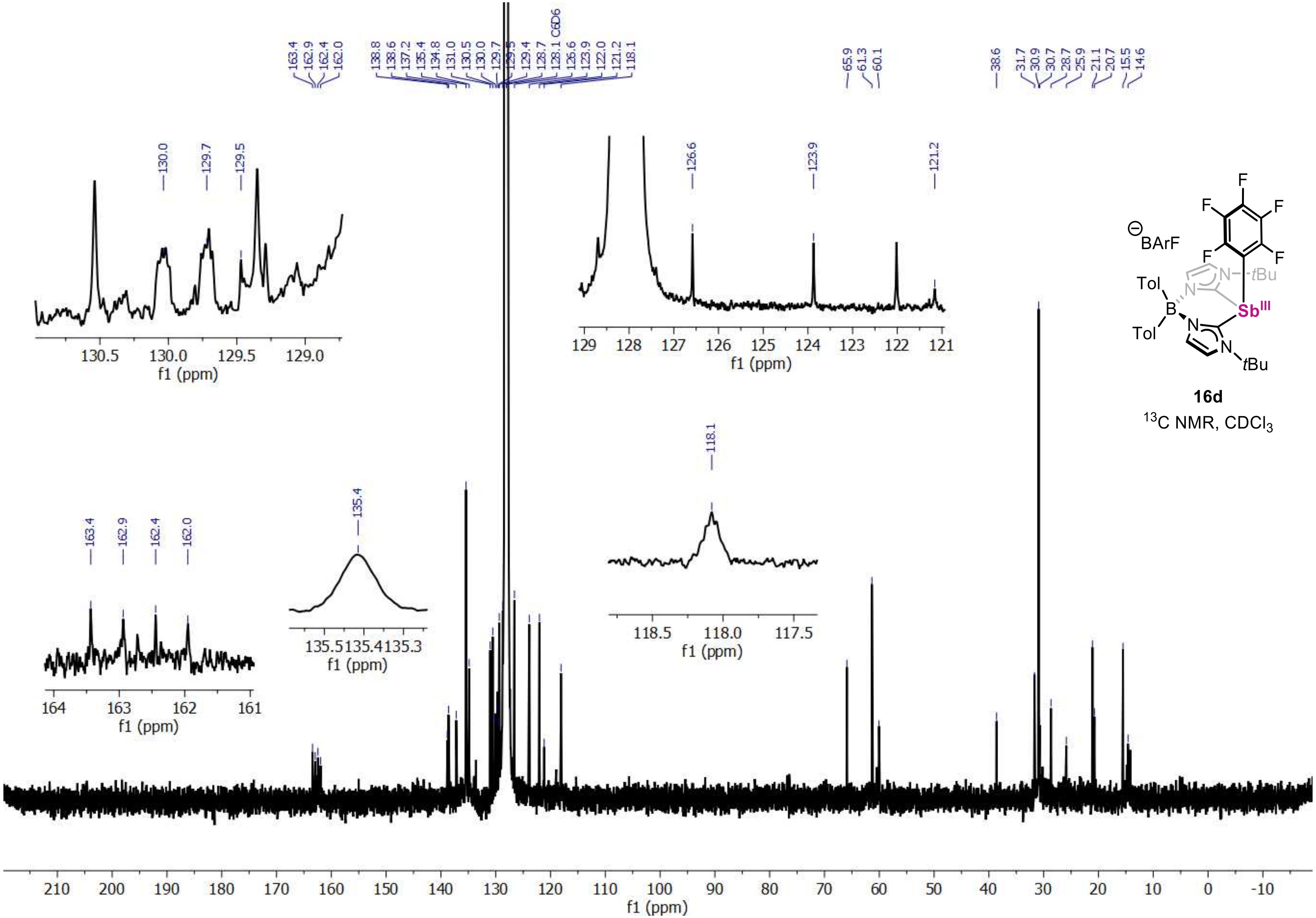


**16d**

$^{13}C$ NMR, $CDCl_3$

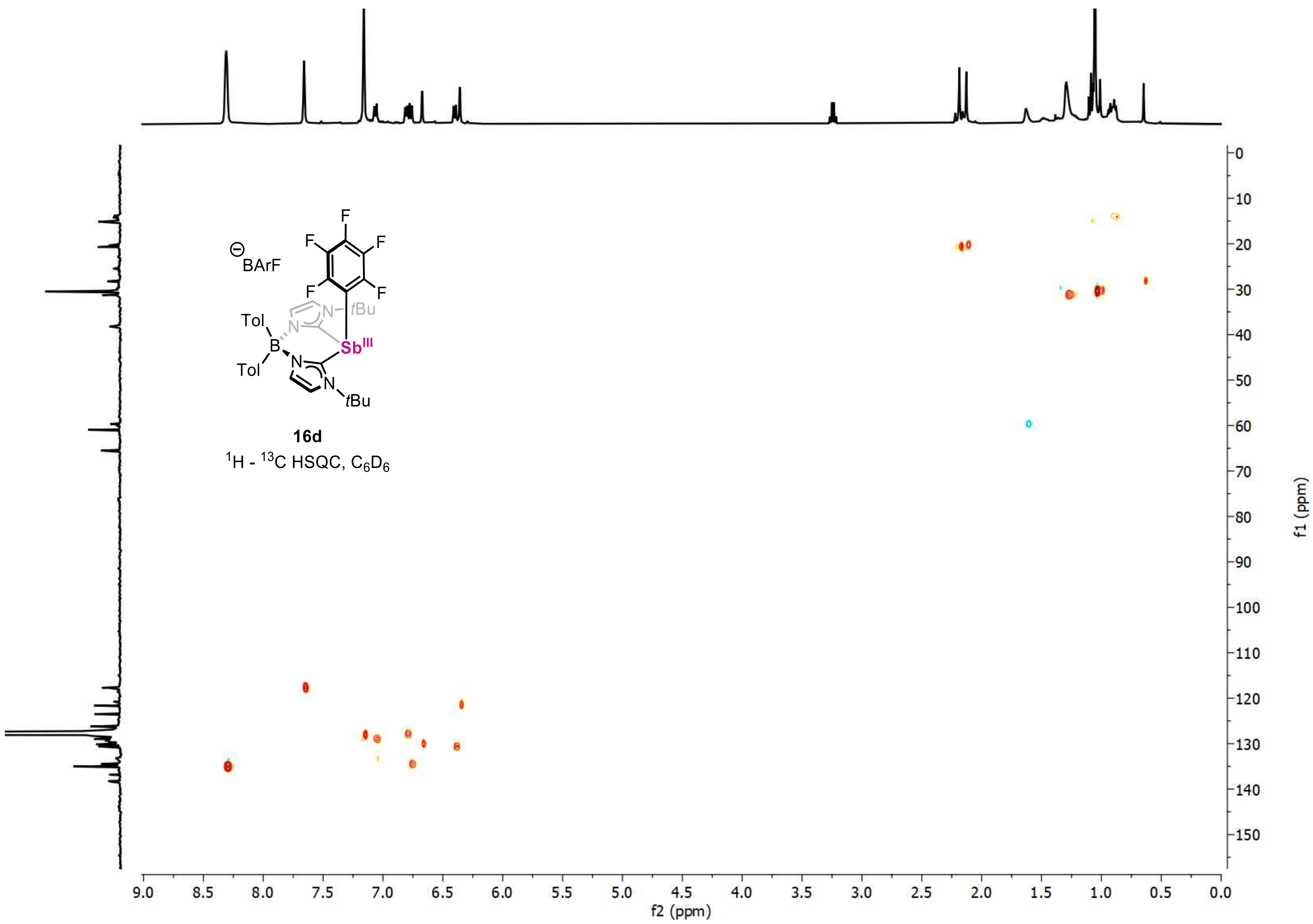
BArF
F
Tol
B
N
tBu
Sb^III
16d
1H - 13C HSQC, C6D6
f2 (ppm)
f1 (ppm)

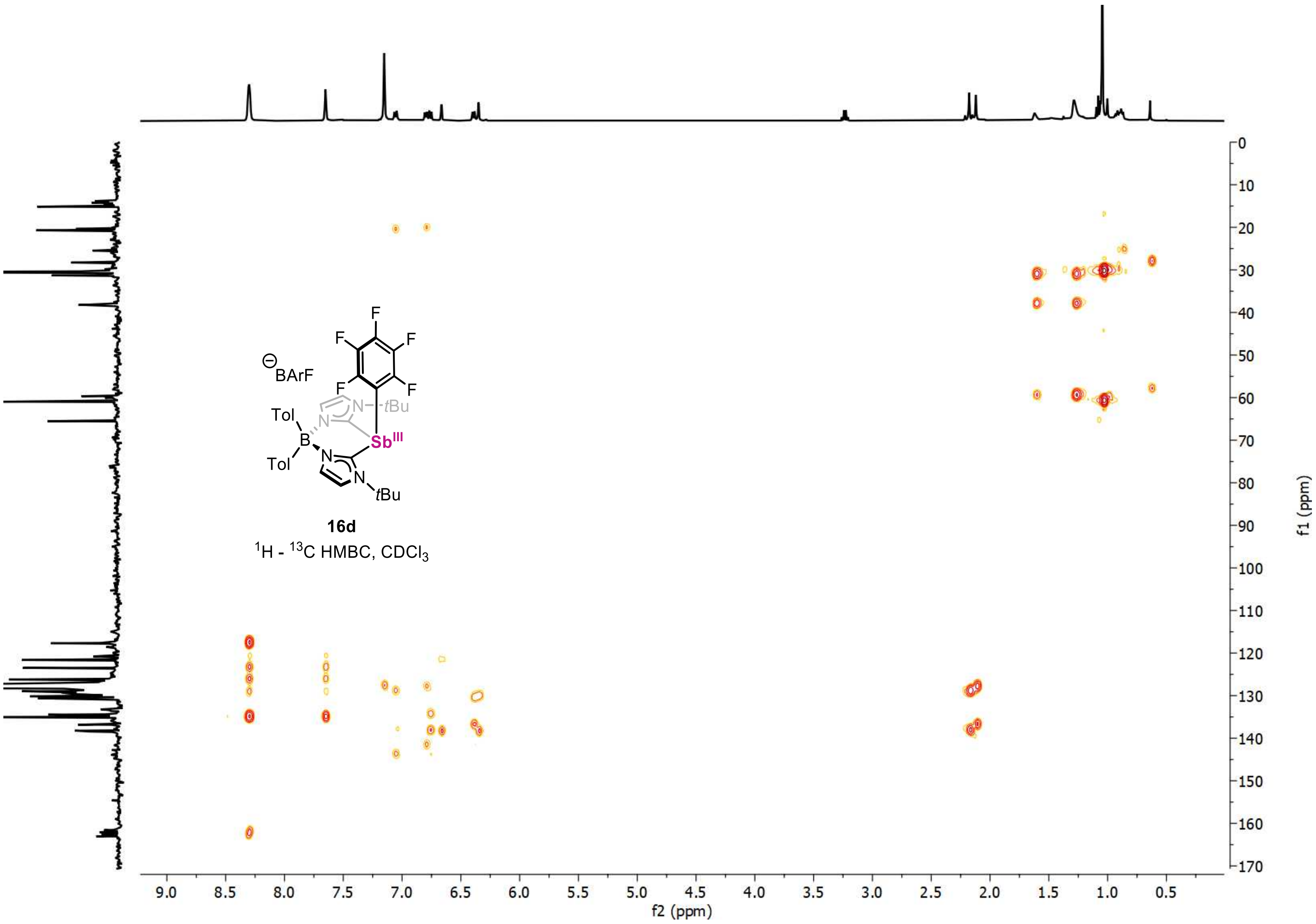
F
F
F
F
F
⊖BArF
N
N
tBu
Tol
B
Tol
N
N
tBu
Sb^III
16d
1H - 13C HMBC, CDCl3
f1 (ppm)
f2 (ppm)
0
10
20
30
40
50
60
70
80
90
100
110
120
130
140
150
160
170
9.0
8.5
8.0
7.5
7.0
6.5
6.0
5.5
5.0
4.5
4.0
3.5
3.0
2.5
2.0
1.5
1.0
0.5

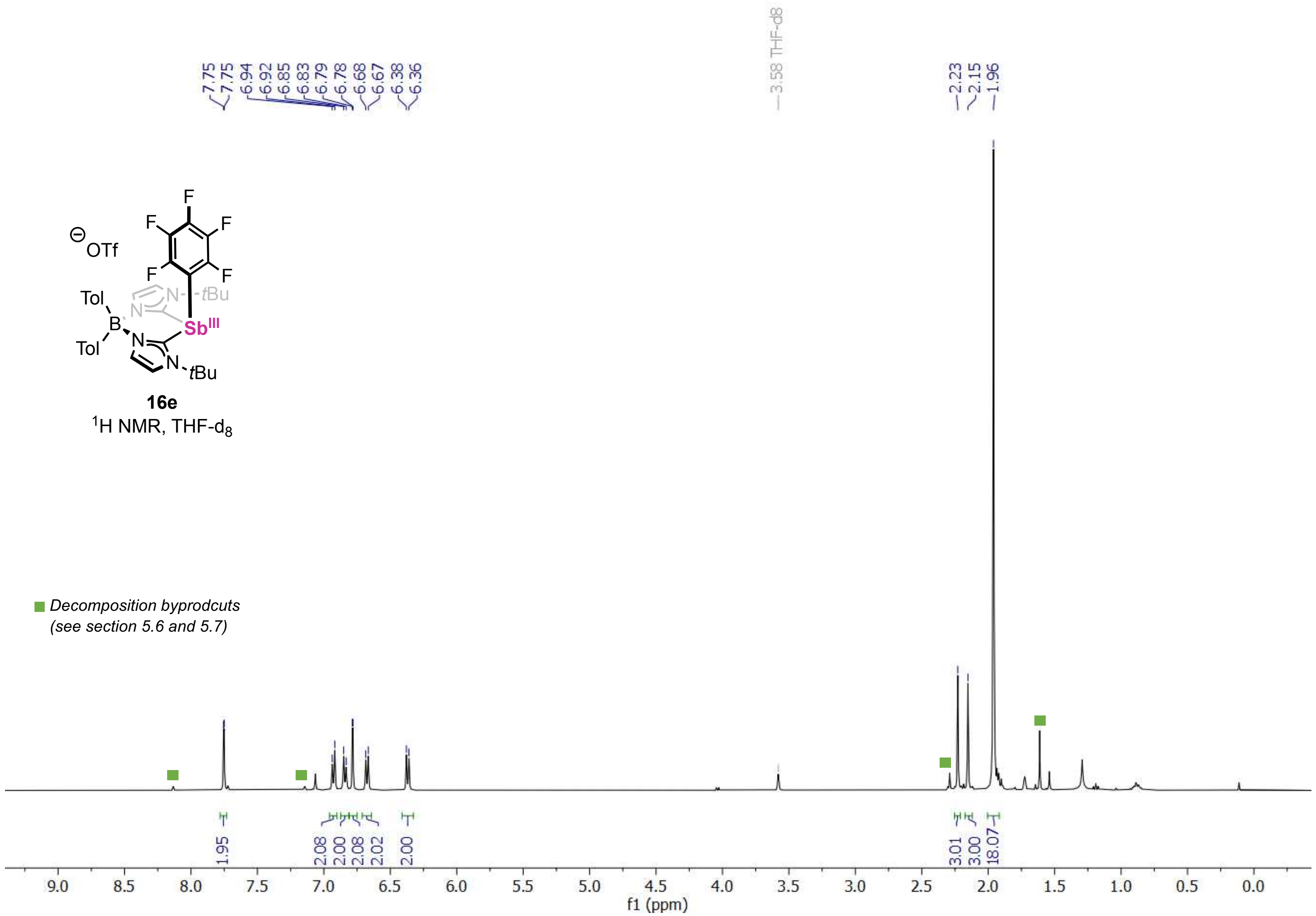

7.75
7.75
6.94
6.92
6.85
6.83
6.79
6.78
6.68
6.67
6.38
6.36
3.58 THF-d8
2.23
2.15
1.96
OTf
F
Tol
B
N
tBu
Sb^III
16e
1H NMR, THF-d8
Decomposition byprodcuts
(see section 5.6 and 5.7)
1.95
2.08
2.00
2.08
2.02
2.00
3.01
3.00
18.07
f1 (ppm)

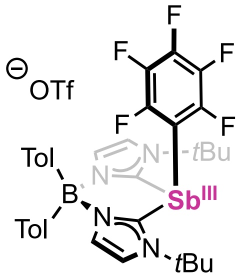

**16e**
$^{19}$F NMR, THF-$d_8$

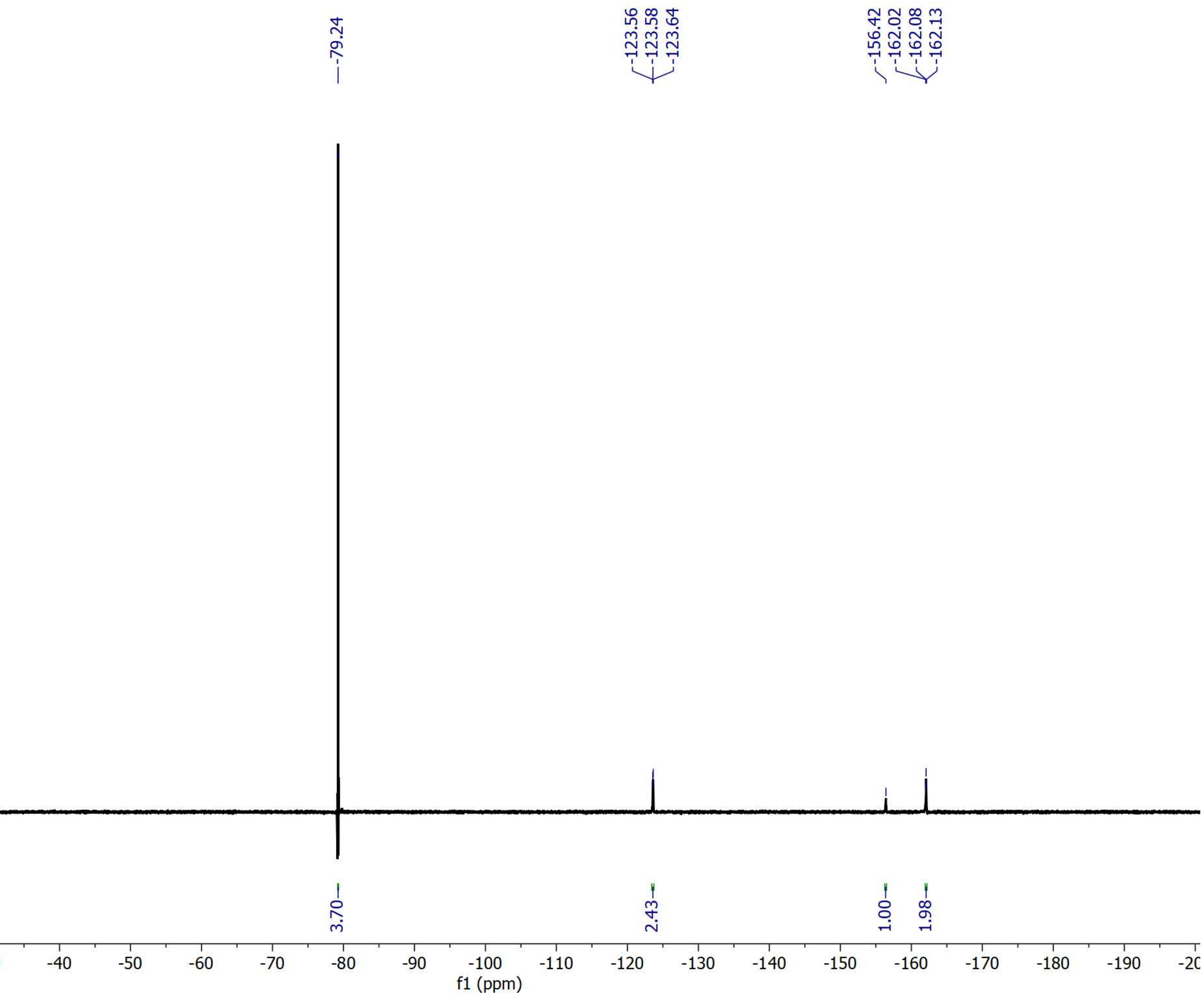

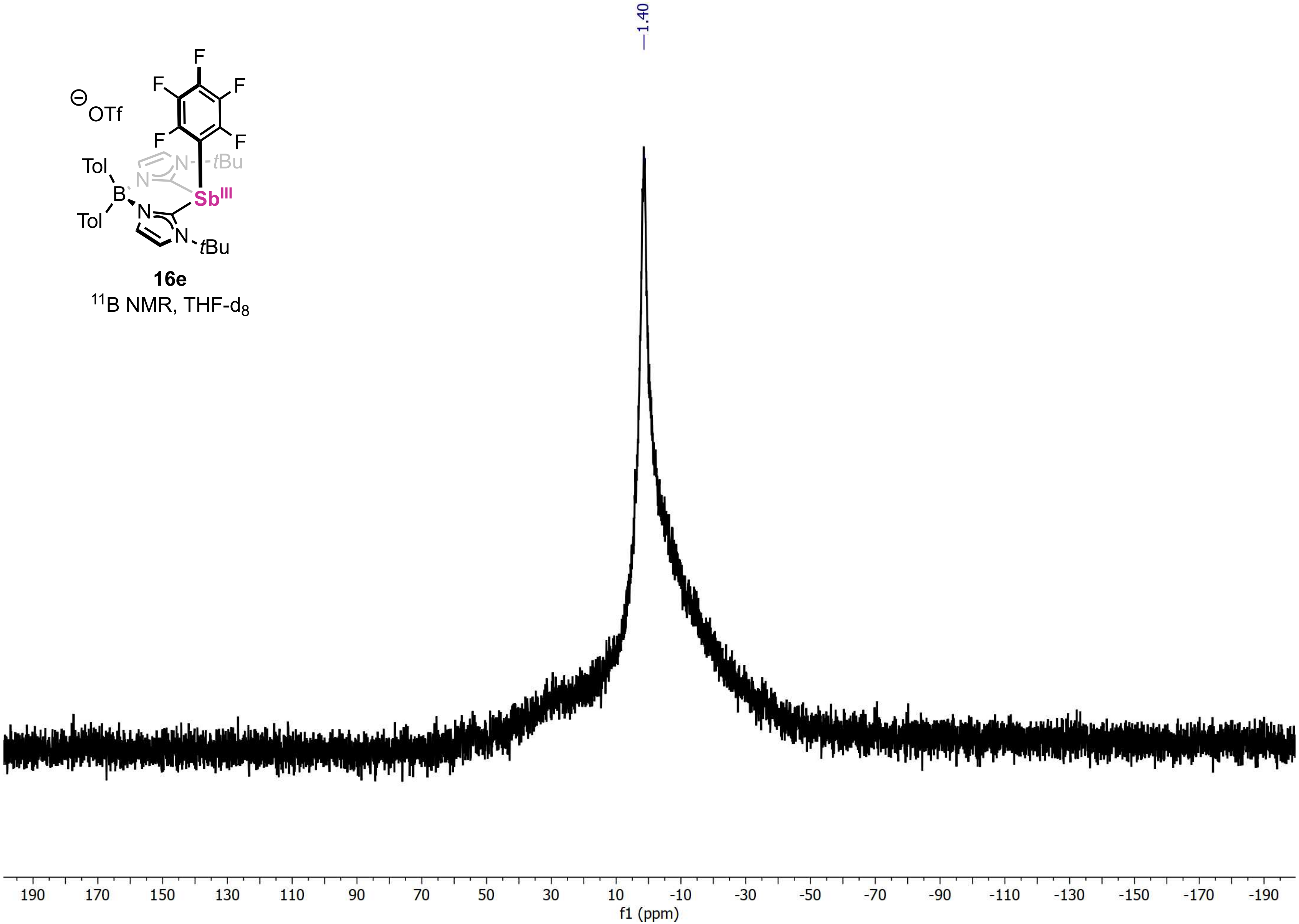


**16e**

$^{11}$B NMR, THF-$d_8$

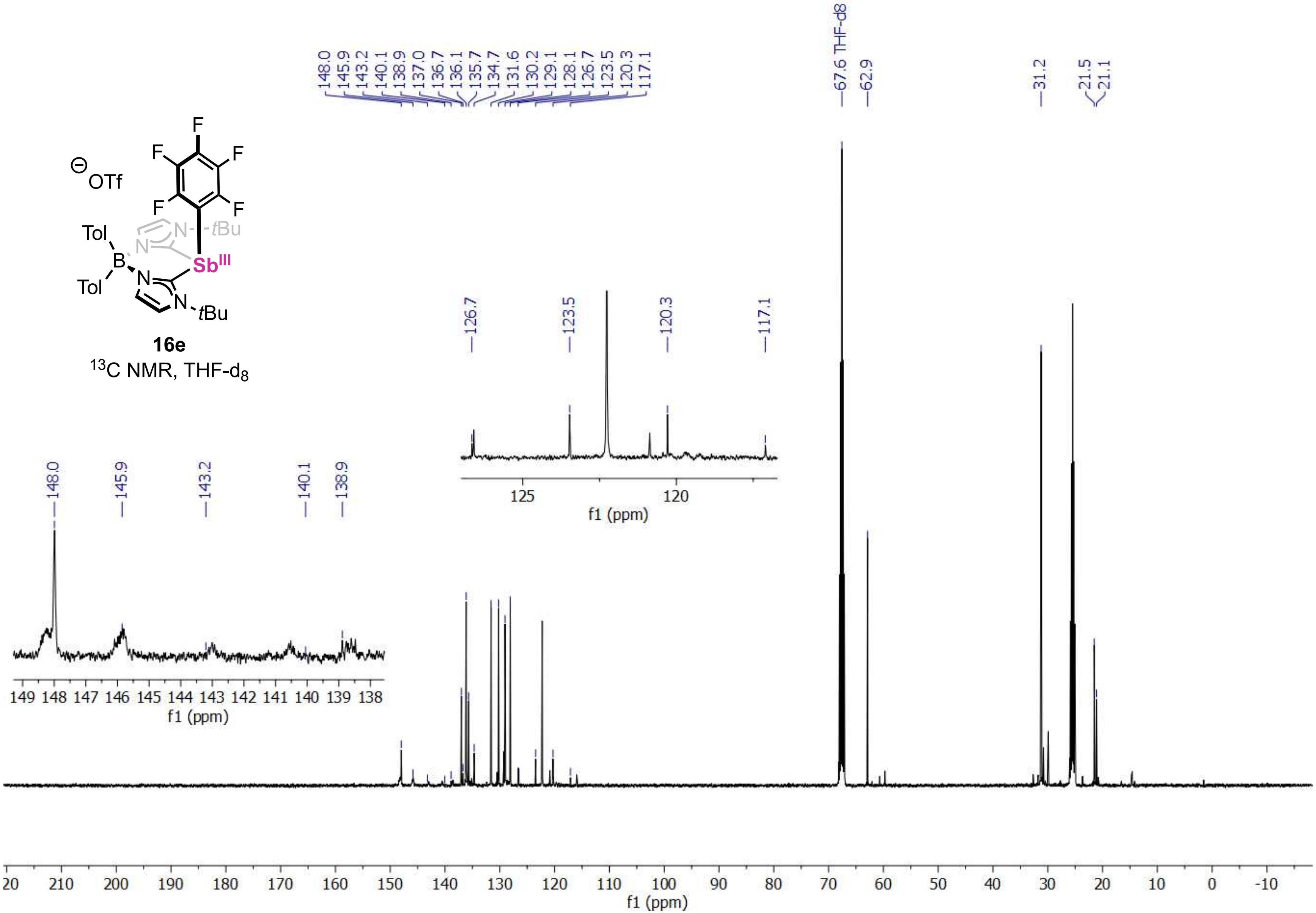
148.0
145.9
143.2
140.1
138.9
137.0
136.7
136.1
135.7
134.7
131.6
130.2
129.1
128.1
126.7
123.5
120.3
117.1
67.6 THF-d8
62.9
31.2
21.5
21.1
OTf
F
Tol
B
N
tBu
Sb^III
16e
$^{13}$C NMR, THF-d$_8$
f1 (ppm)

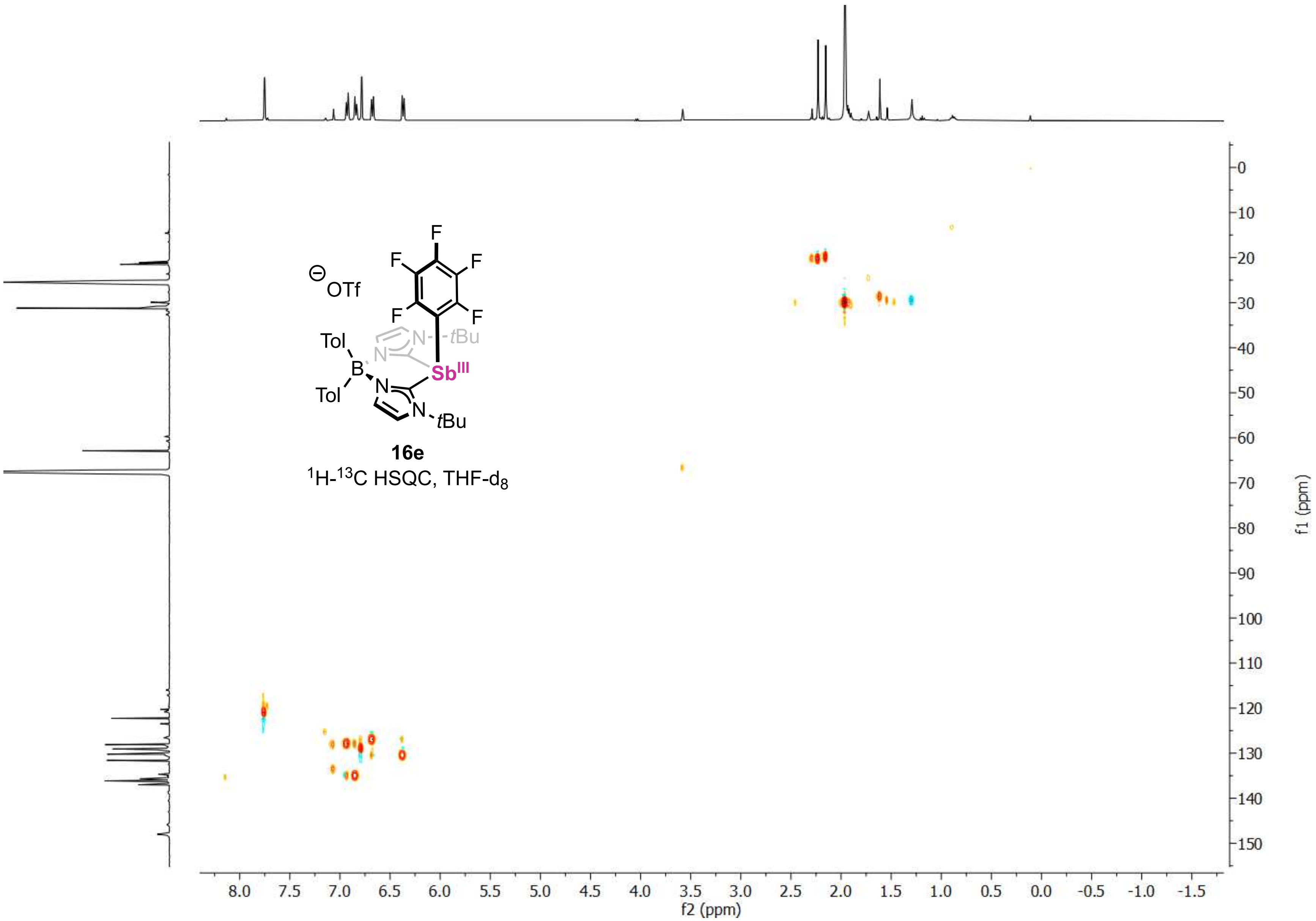


**16e**

$^{1}H$-$^{13}C$ HSQC, THF-$d_8$

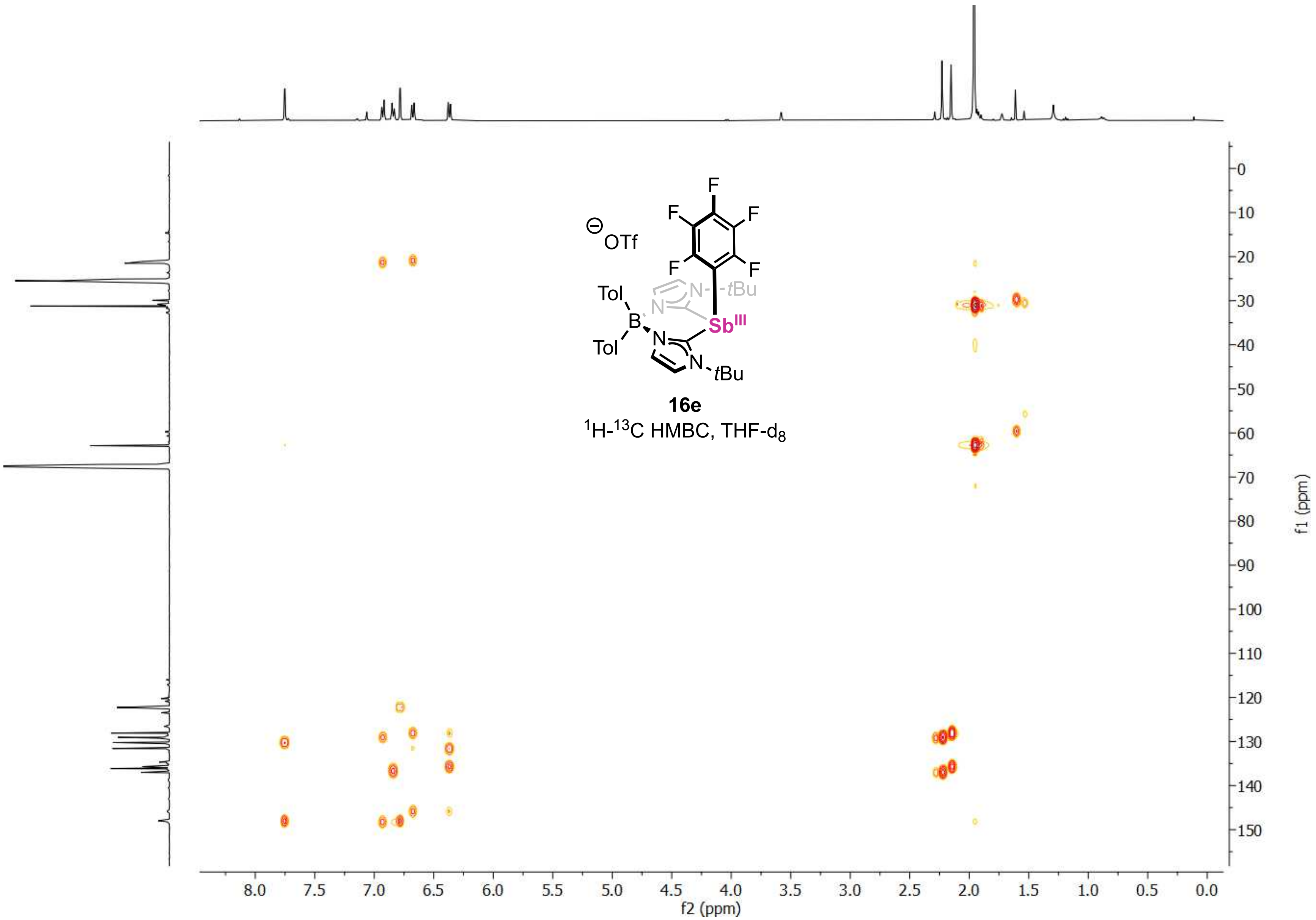

$^{\ominus}$OTf
F
F
F
F
F
N
N
tBu
Tol
B
Tol
N
Sb^III
N
tBu
16e
$^1$H-$^{13}$C HMBC, THF-d$_8$
f1 (ppm)
f2 (ppm)

## 11.6 NMR characterisation of Sb(II) dimer 12

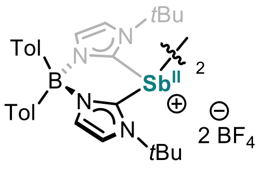


**12**

$^{1}$H NMR, $CD_3CN$

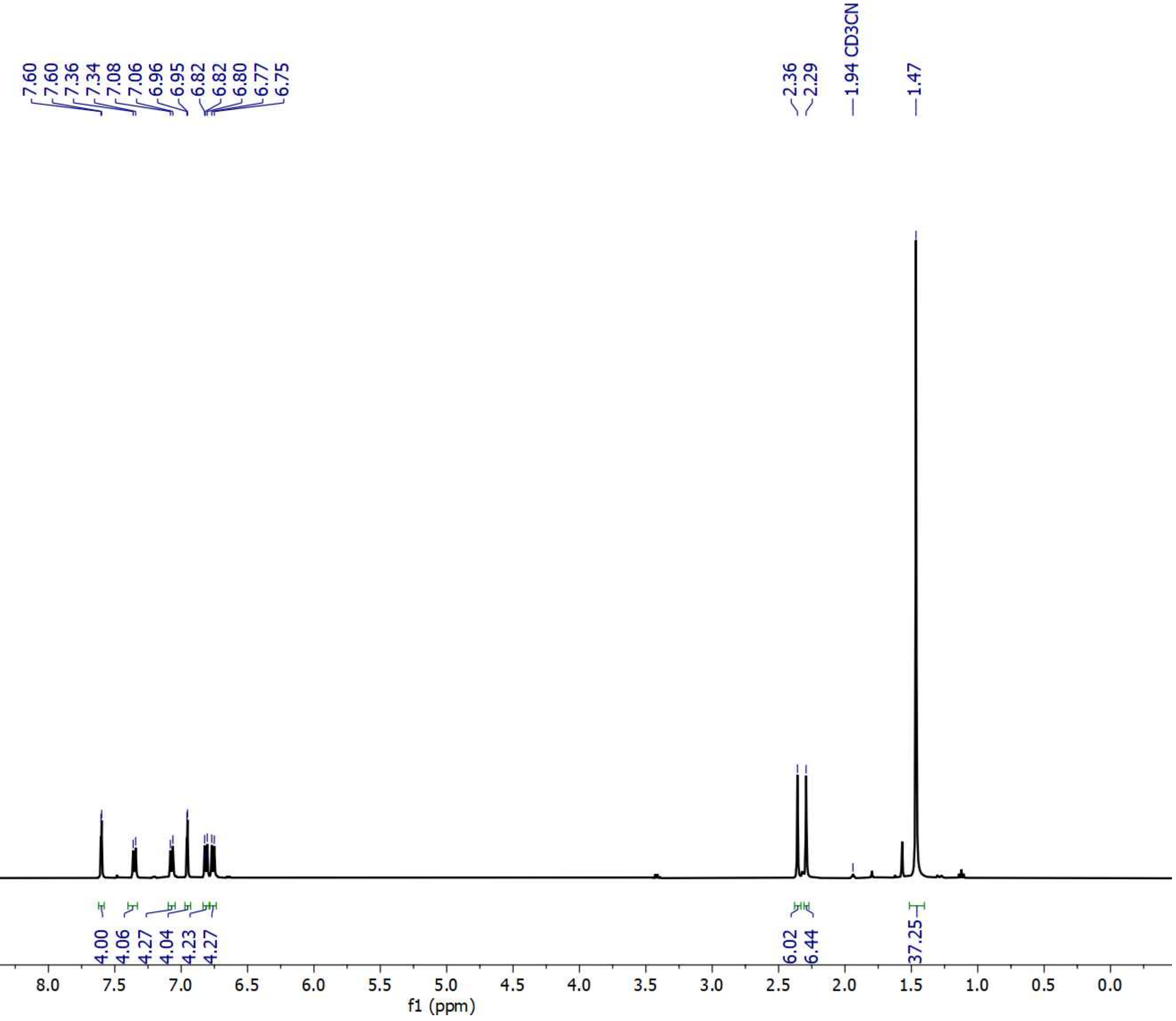

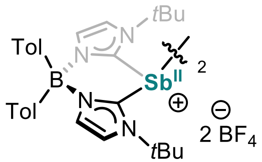


**12**

$^{19}F$ NMR, $CD_3CN$

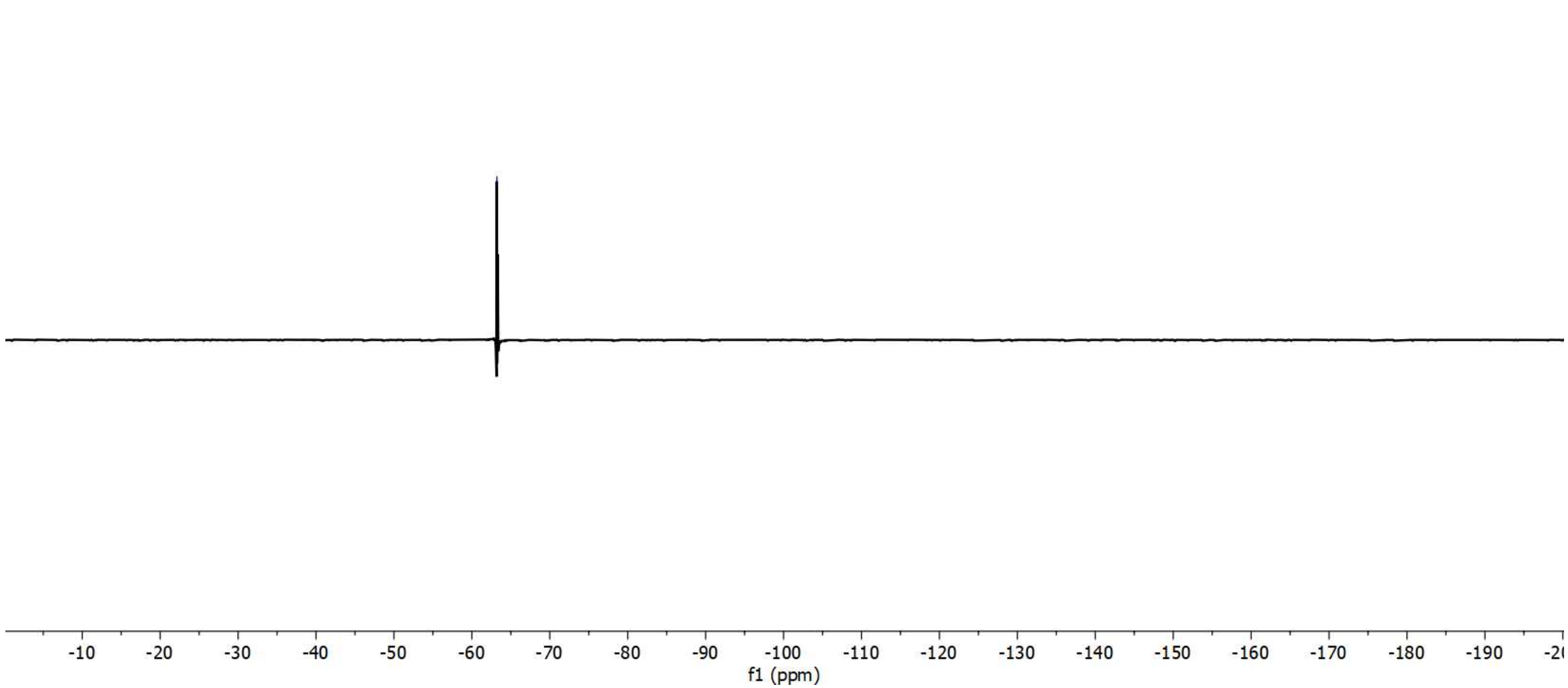

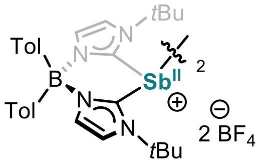

**12**

$^{11}$B NMR, $CD_3CN$

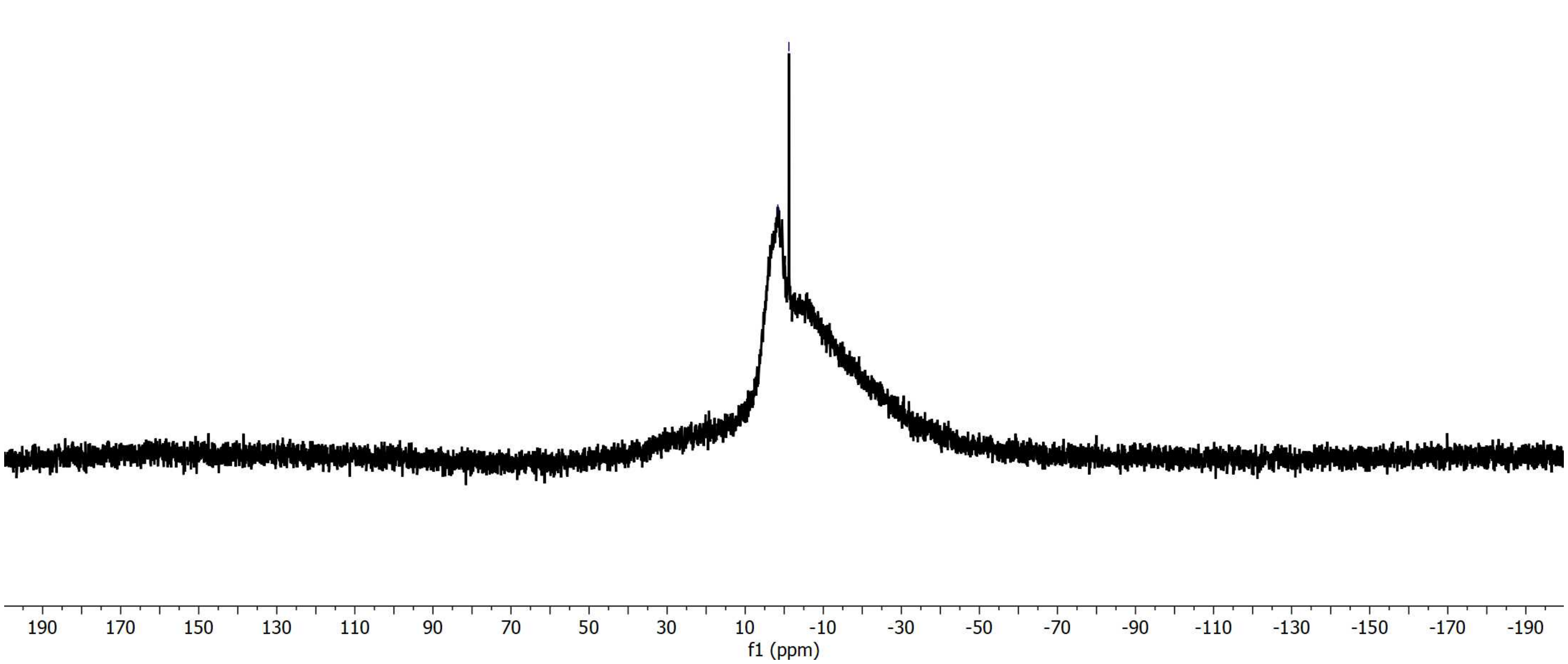

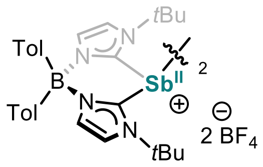


**12**

$^{13}C$ NMR, $CD_3CN$

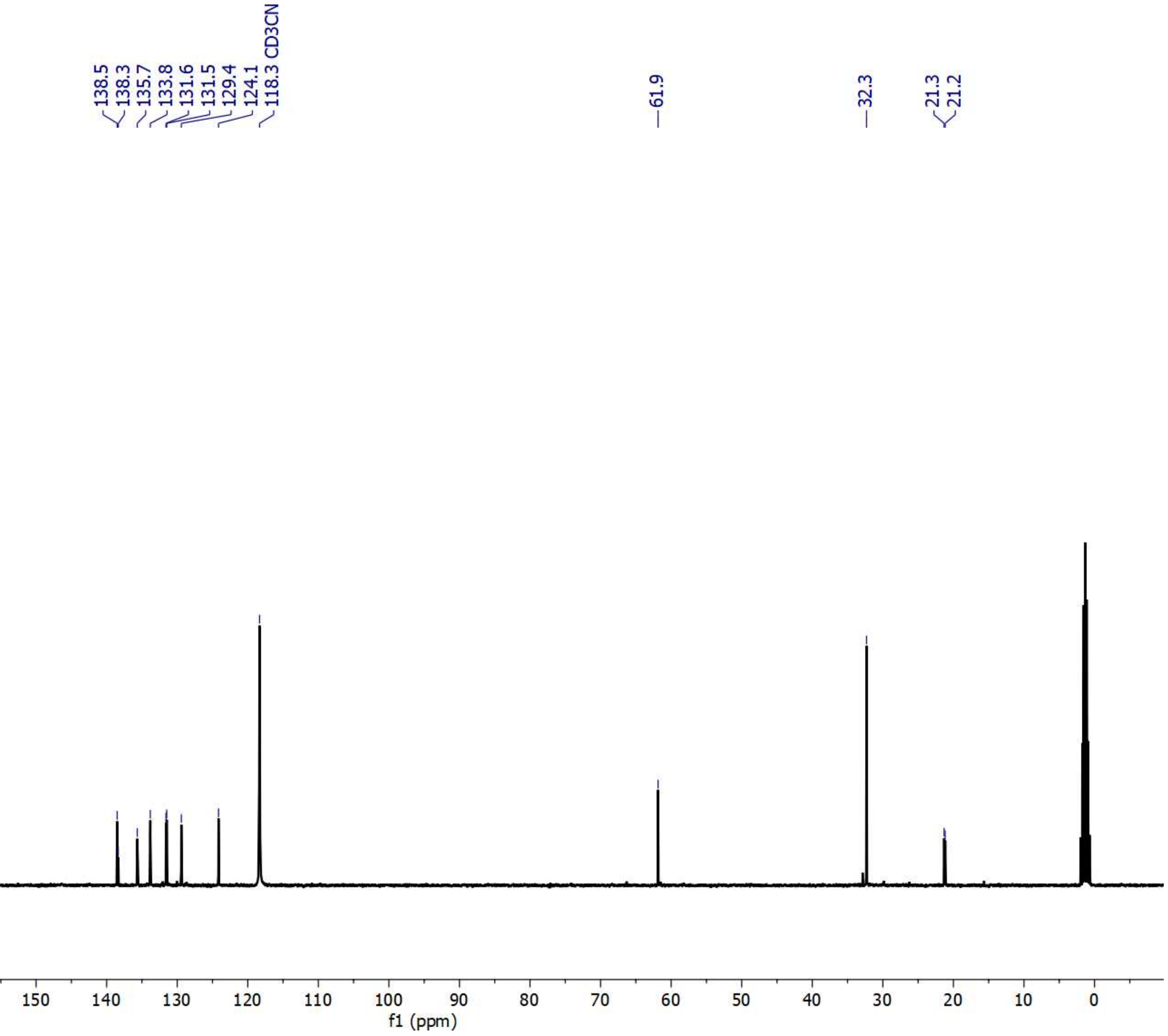

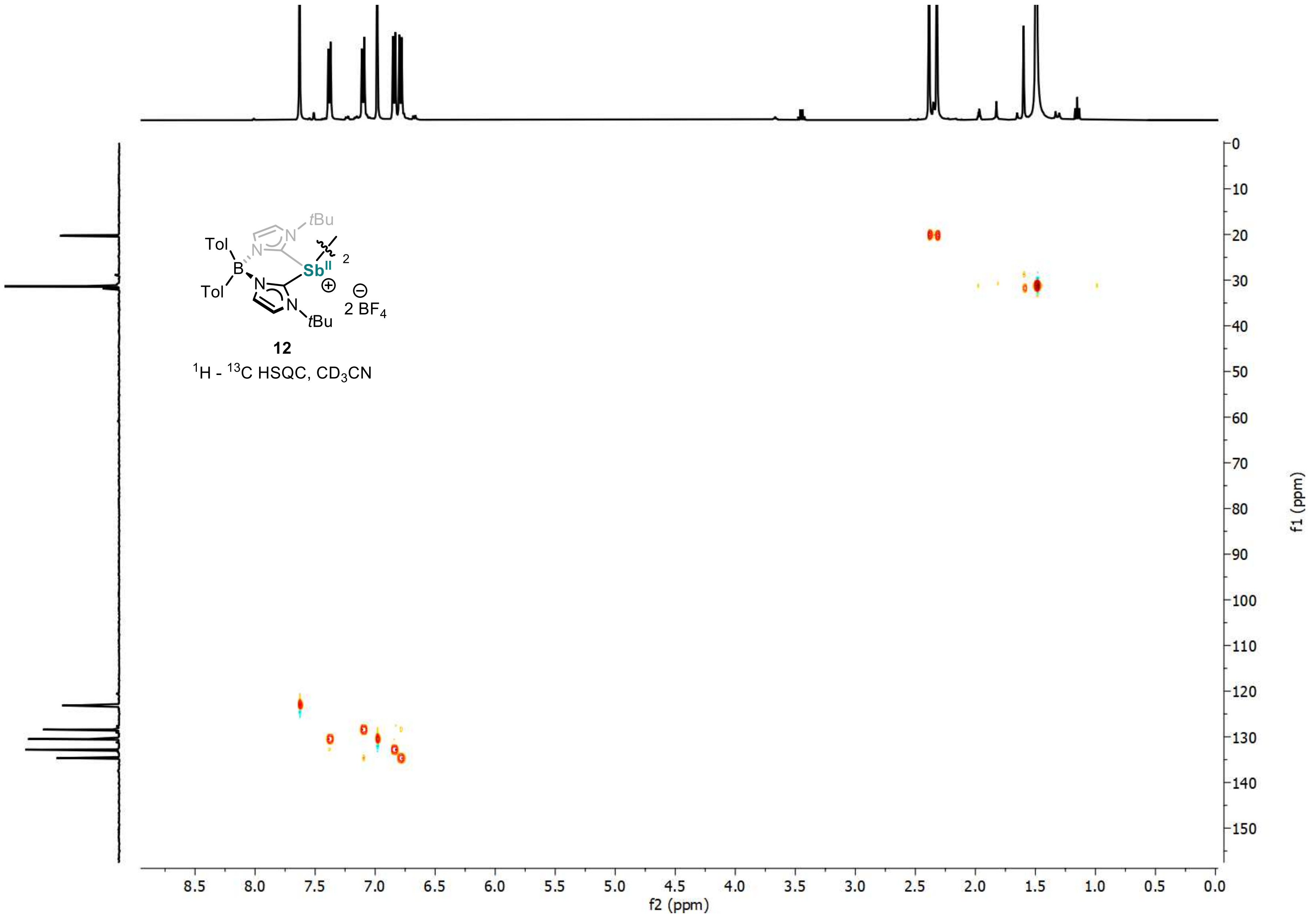
Tol
Tol
B
N
N
N
N
tBu
tBu
Sb^II
2
2 BF4
12
1H - 13C HSQC, CD3CN
f1 (ppm)
f2 (ppm)
0
10
20
30
40
50
60
70
80
90
100
110
120
130
140
150
8.5
8.0
7.5
7.0
6.5
6.0
5.5
5.0
4.5
4.0
3.5
3.0
2.5
2.0
1.5
1.0
0.5
0.0

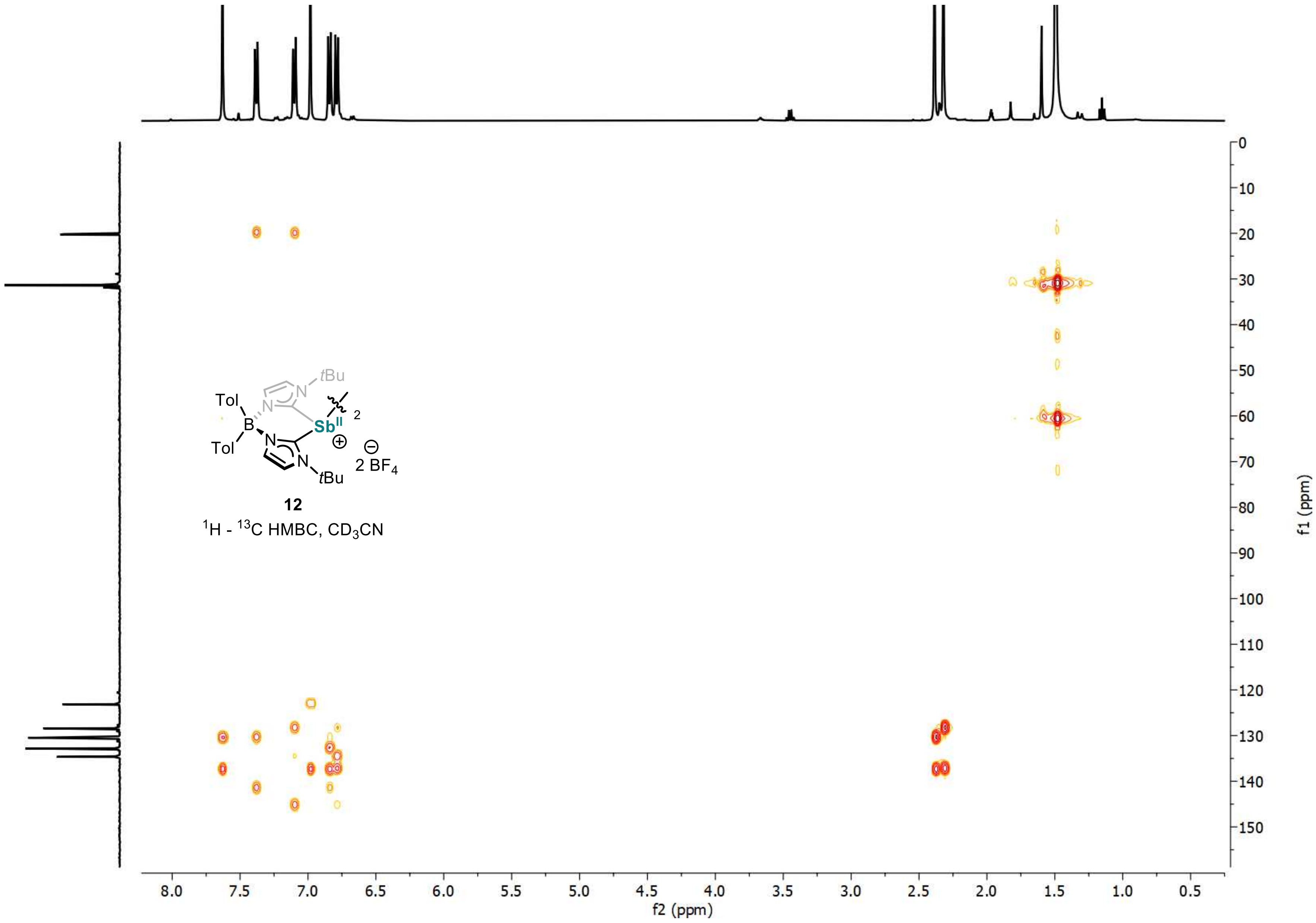
Tol
Tol
B
N
N
N
N
tBu
tBu
Sb^II
2
2 BF4
12
1H - 13C HMBC, CD3CN
f2 (ppm)
f1 (ppm)

## 11.7 $^{19}$F NMR of crude reactions after Sb(I)-catalysed hydrodefluorination with phenylsilane

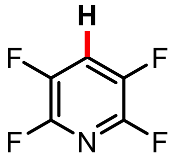

**13a**, <95%
$^{19}$F NMR, benzene-$d_6$

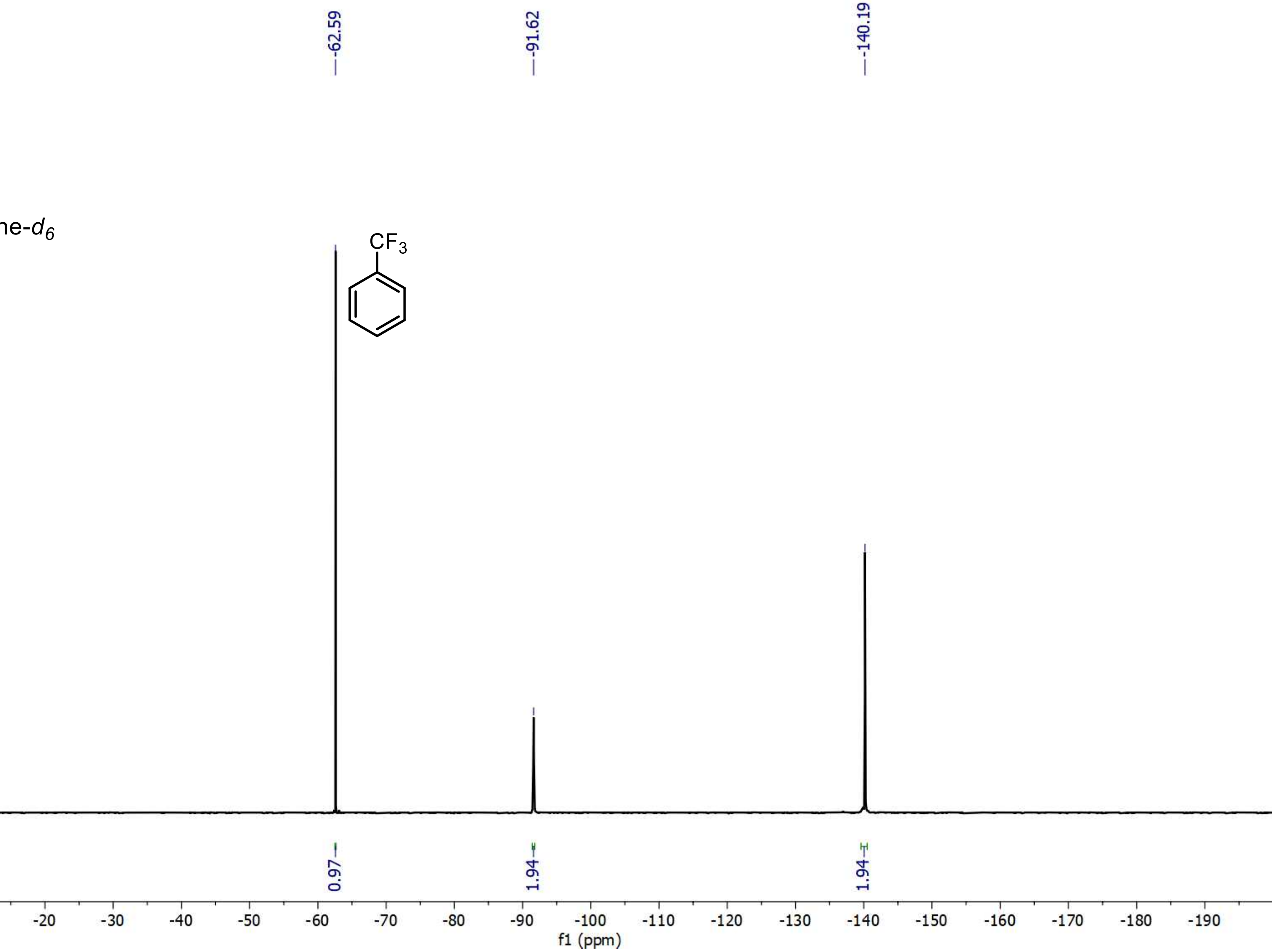

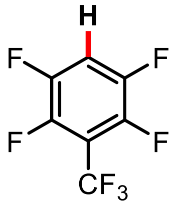


**13b**, >95%
$^{19}F$ NMR, benzene-$d_6$

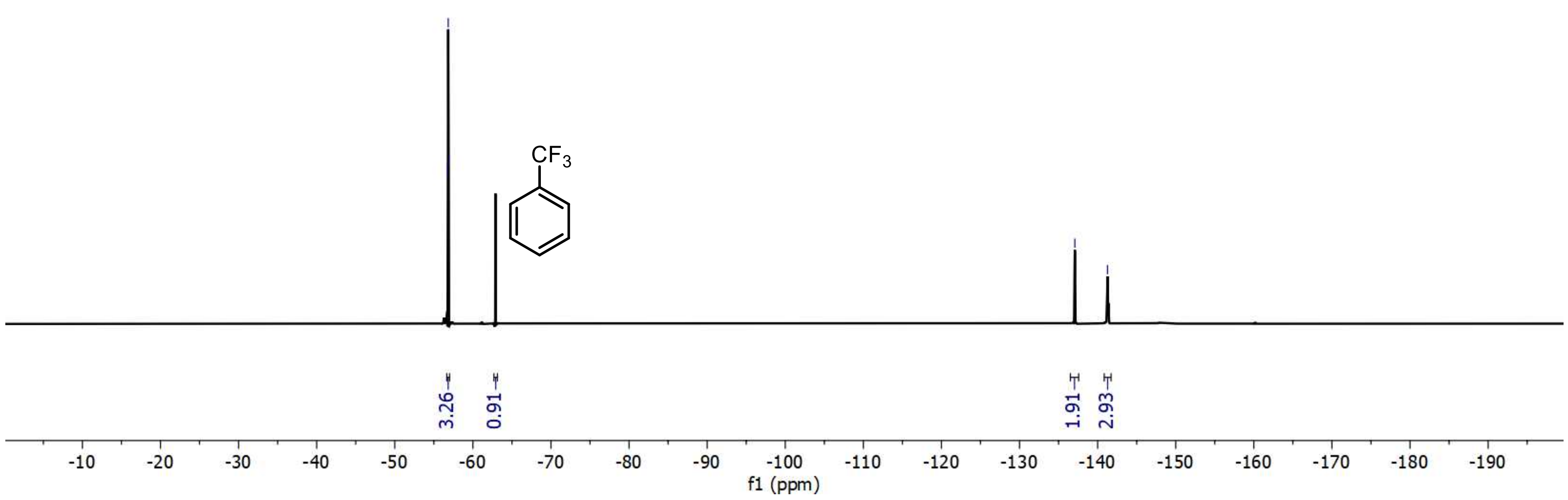

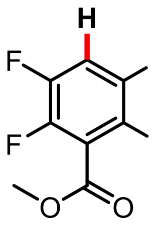


**13c**, 79%

$^{19}$F NMR, benzene-$d_6$

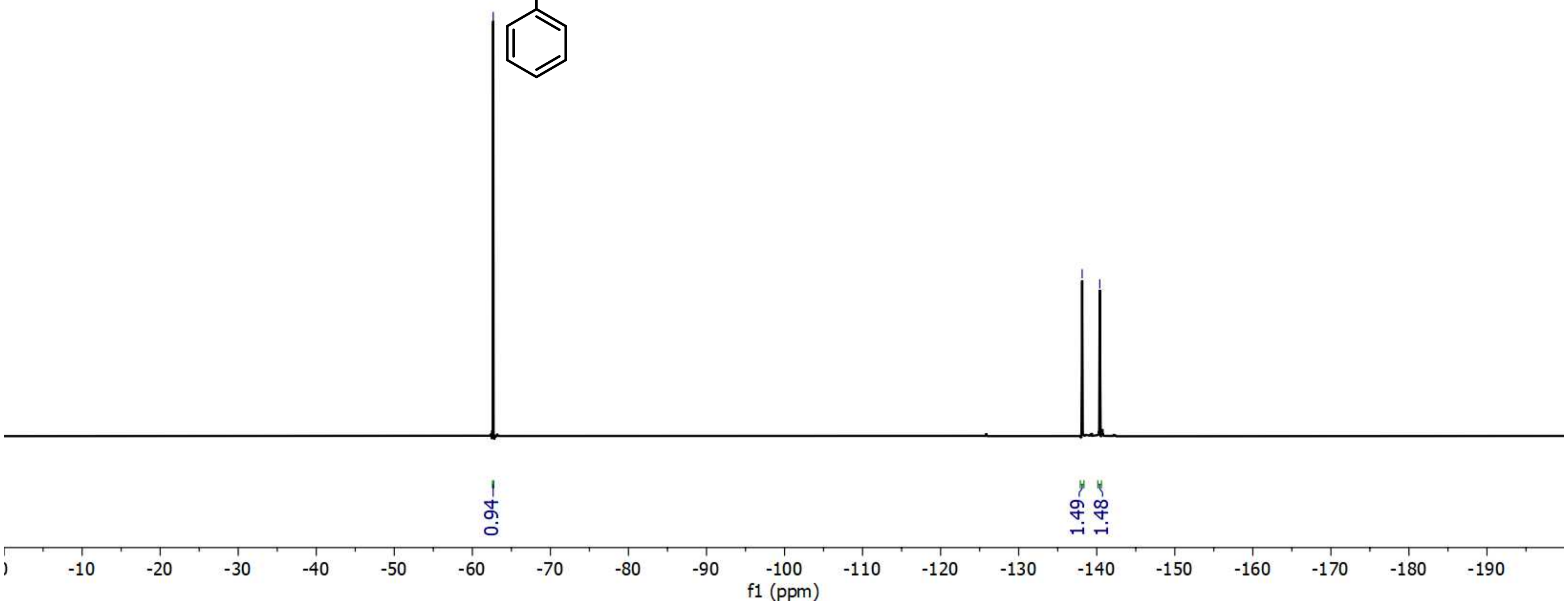

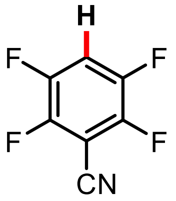


**13d**, >95%

$^{19}$F NMR, benzene-$d_6$

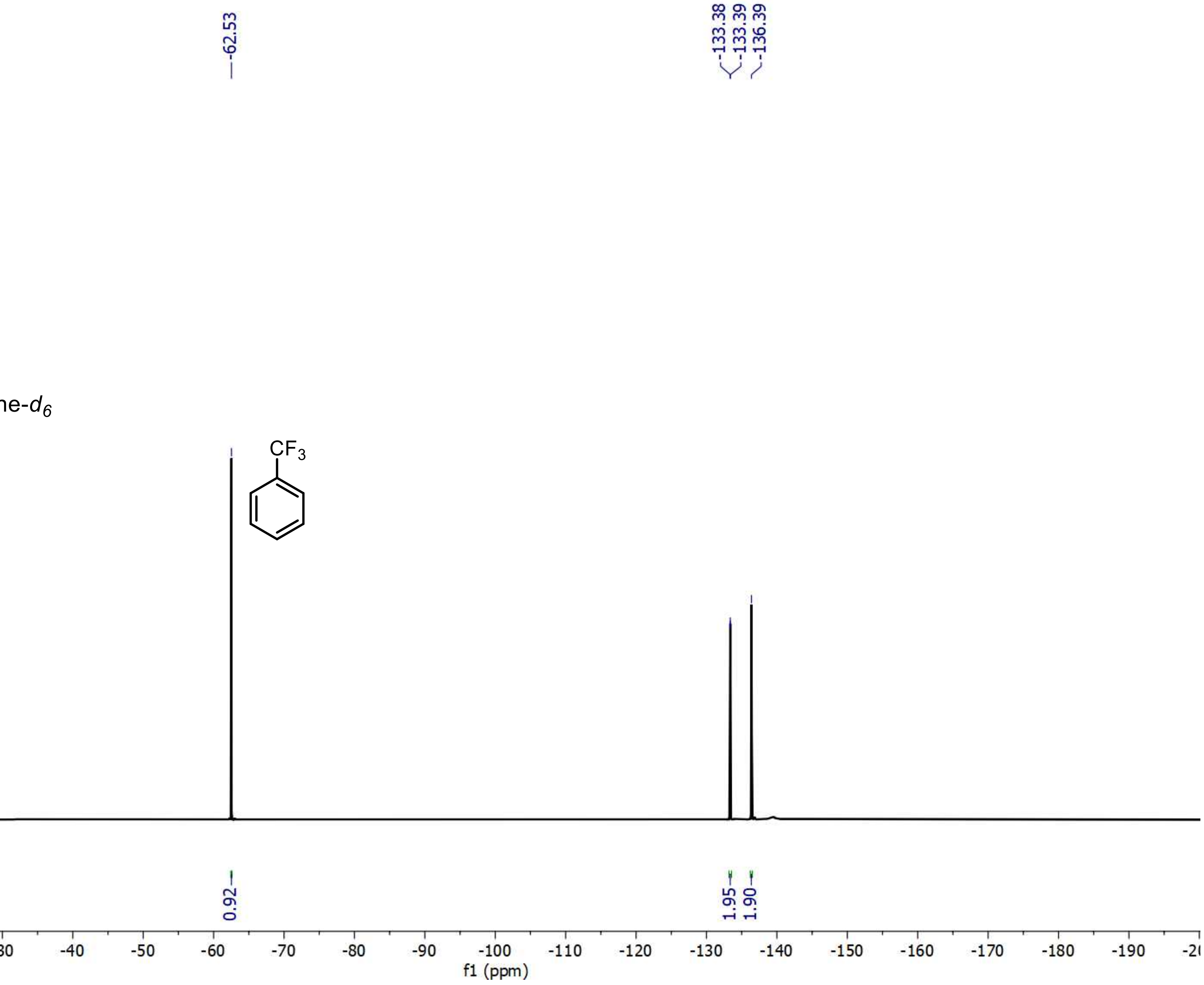

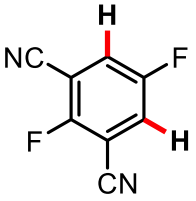


**13e**, >95%

$^{19}$F NMR, benzene-$d_6$

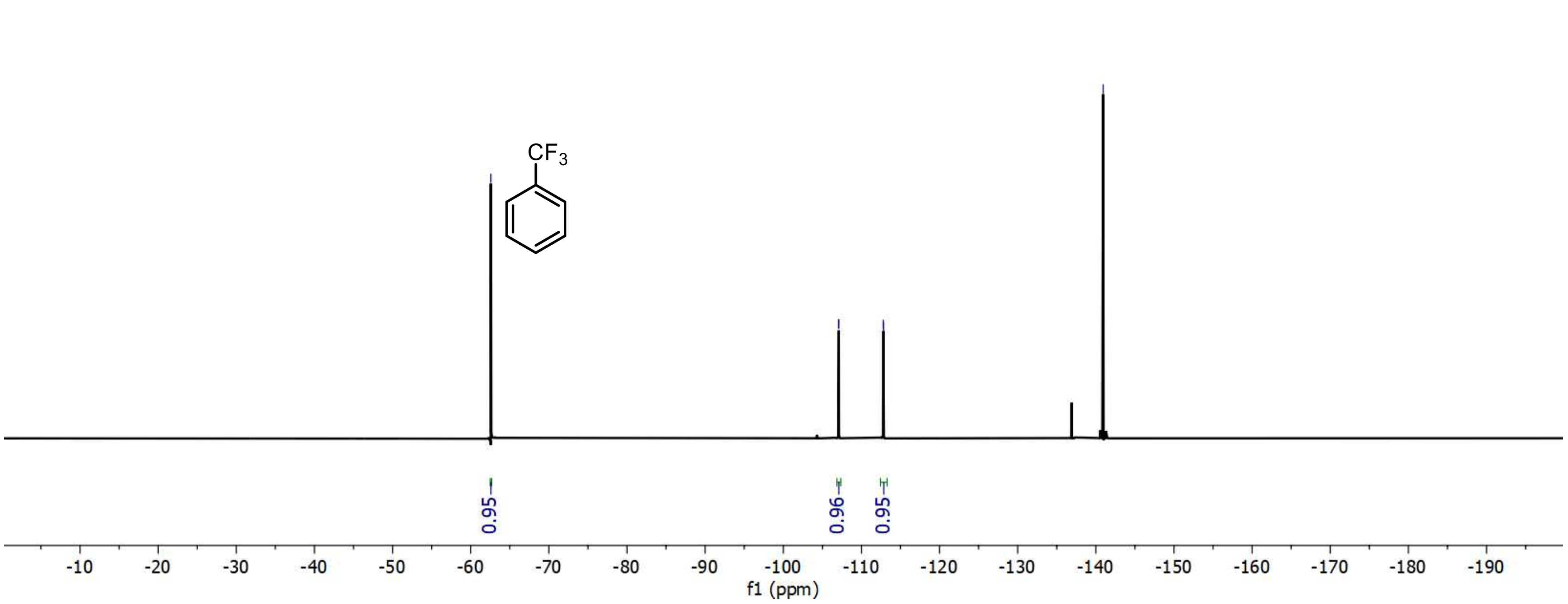

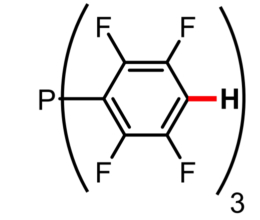


**13f**, 29%

$^{19}$F NMR, benzene-$d_6$

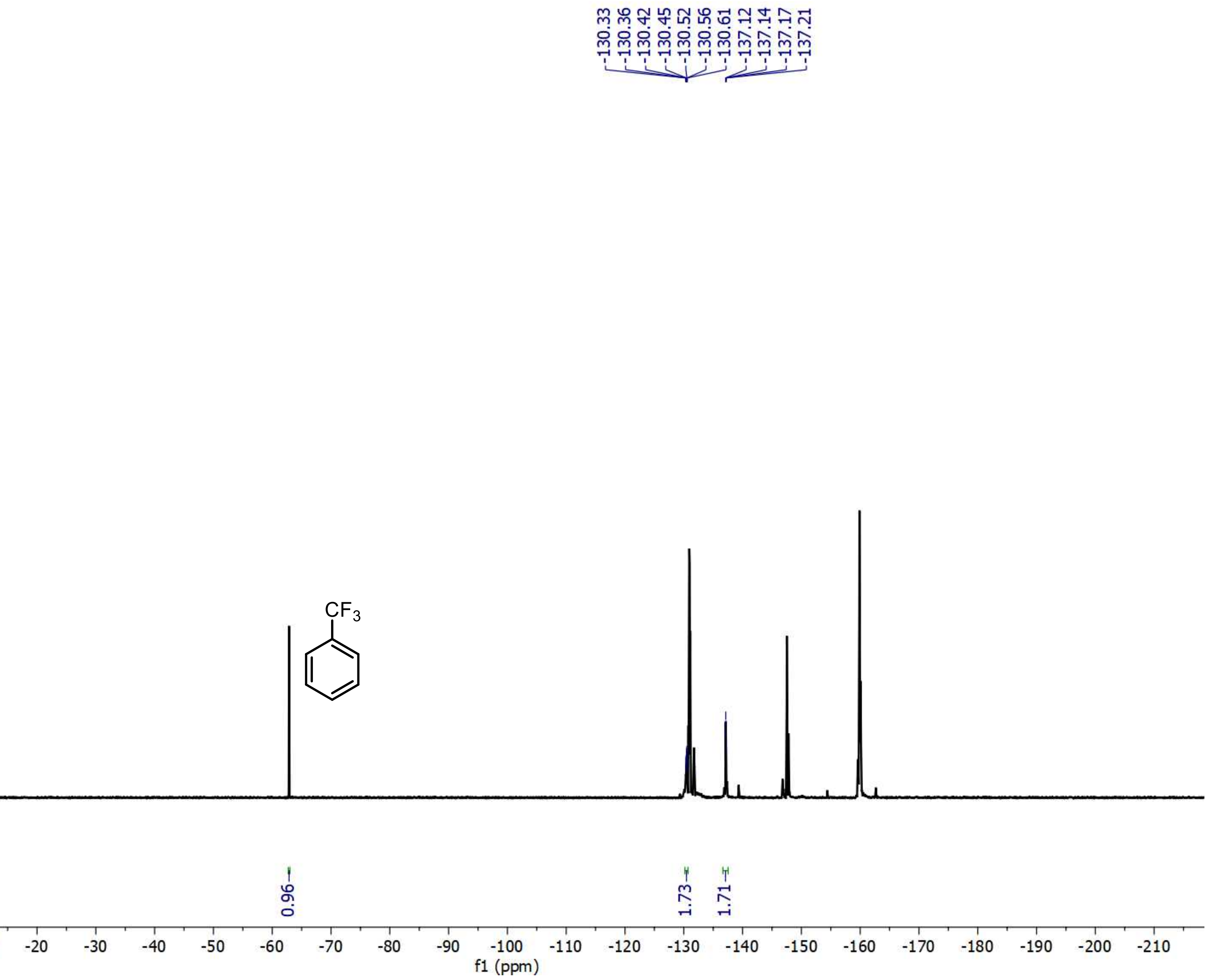

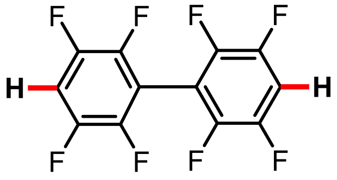

**13g**, 79%

$^{19}$F NMR, benzene-$d_6$

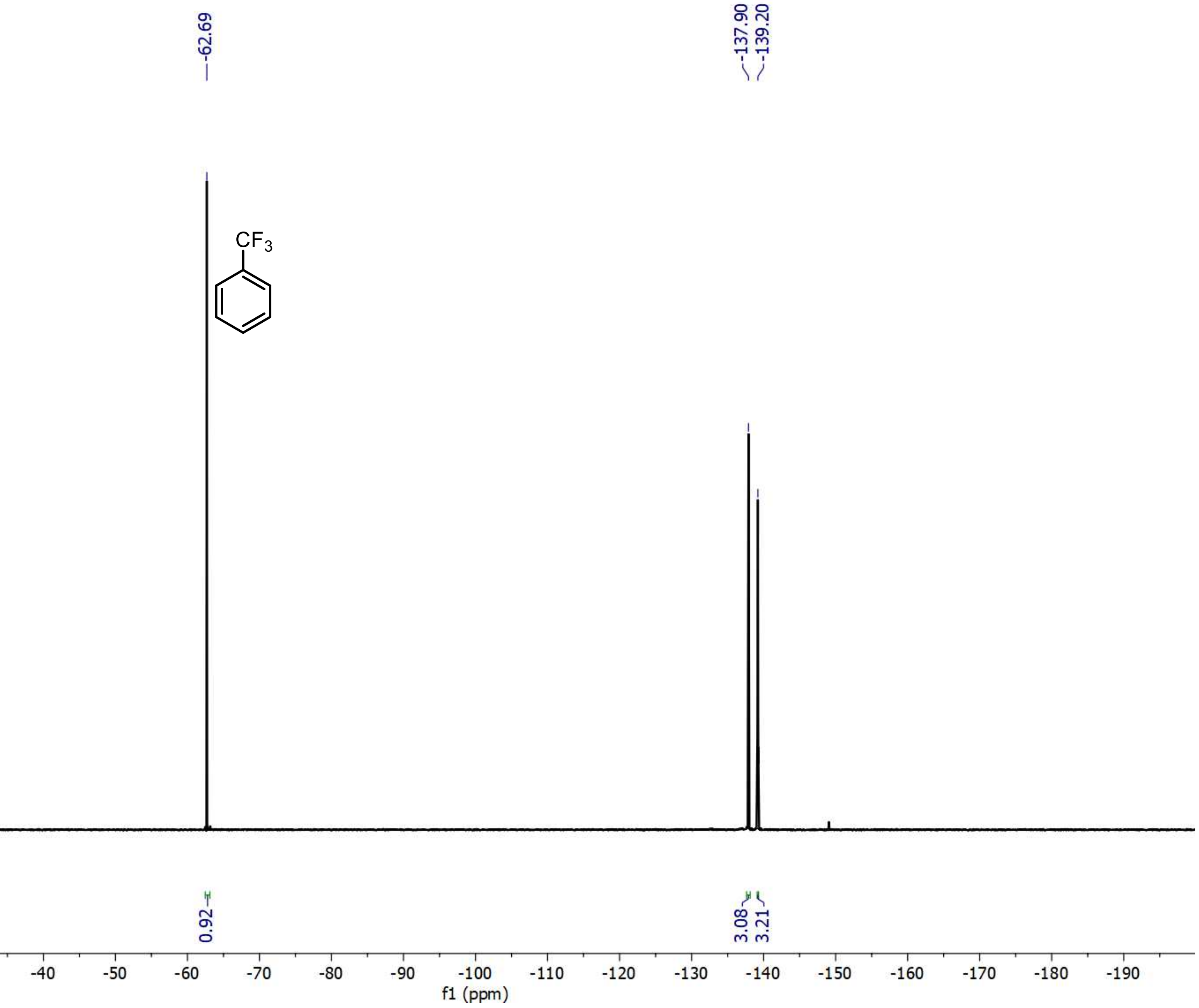

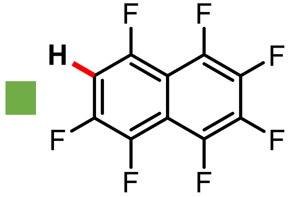


**13h**, 61%
$^{19}$F NMR, benzene-$d_6$

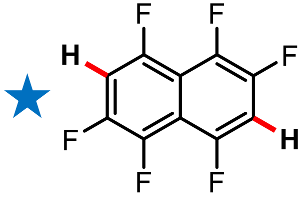


**13i**, 15%
$^{19}$F NMR, benzene-$d_6$

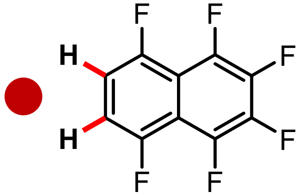


**13j**, >5%
$^{19}$F NMR, benzene-$d_6$

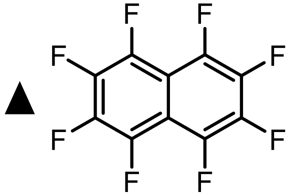


**SM**, 24%
$^{19}$F NMR, benzene-$d_6$

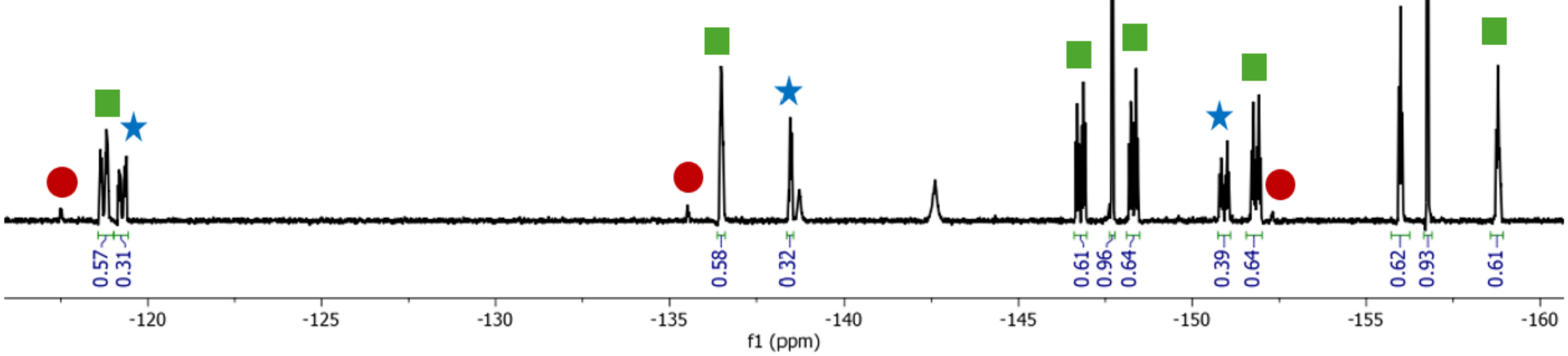

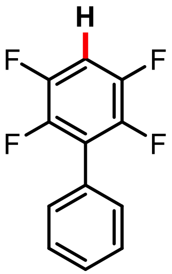


**13m**, 10%
$^{19}F$ NMR, benzene-$d_6$

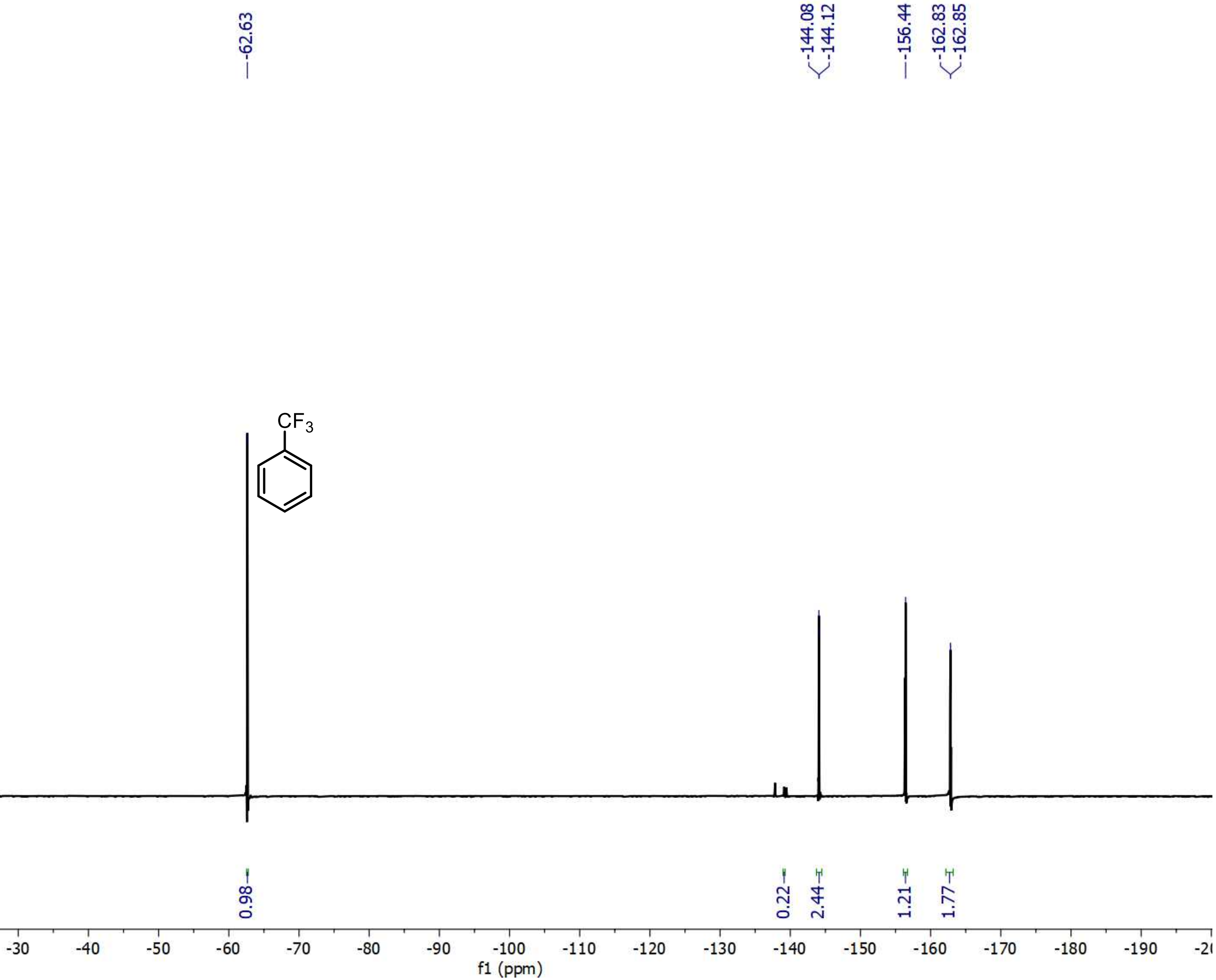

## 11.8 Characterisation of dehydrogenative silylation product

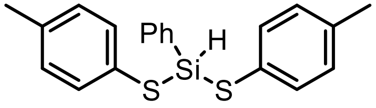


**15**

$^{1}$H NMR, benzene-d6

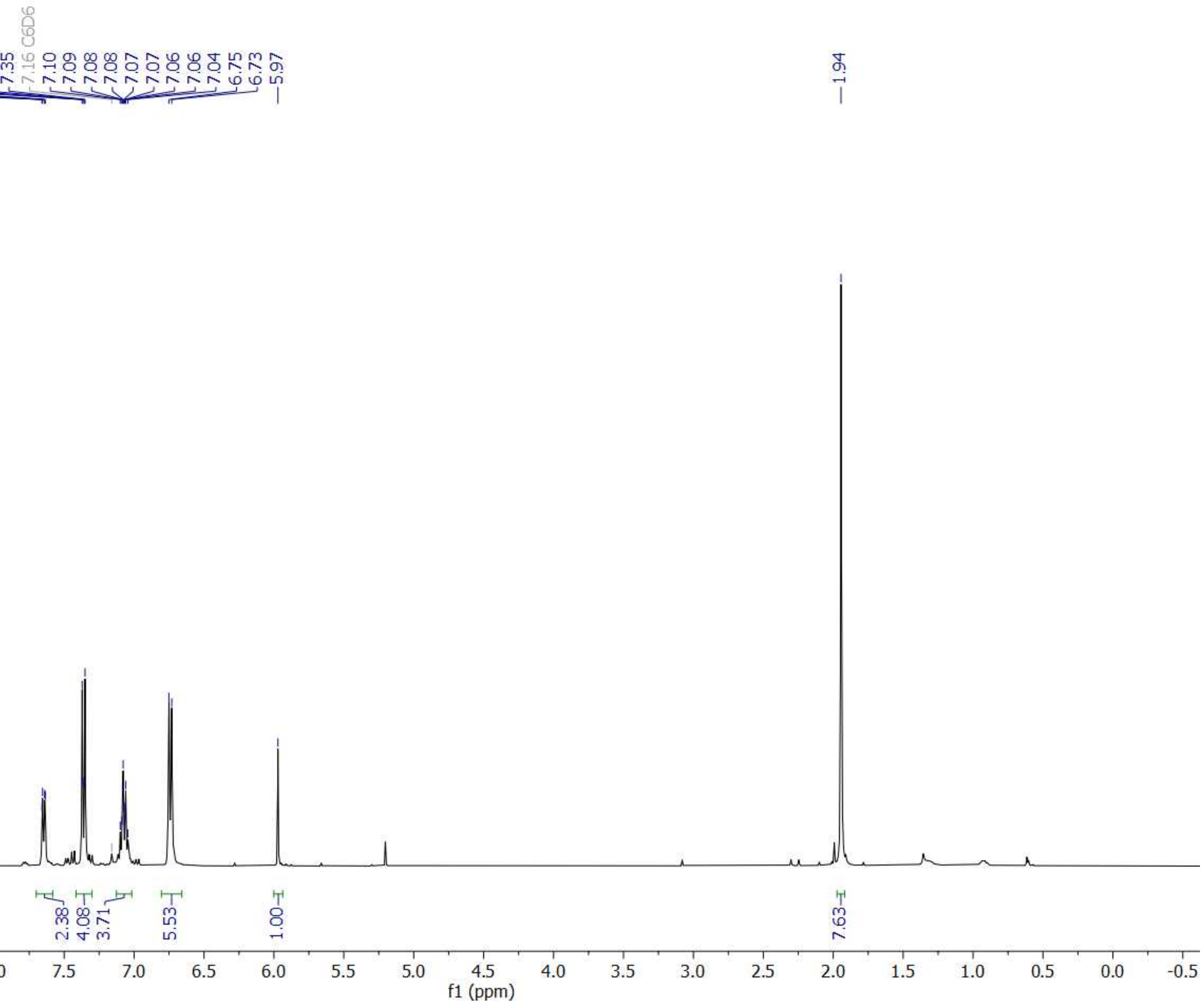

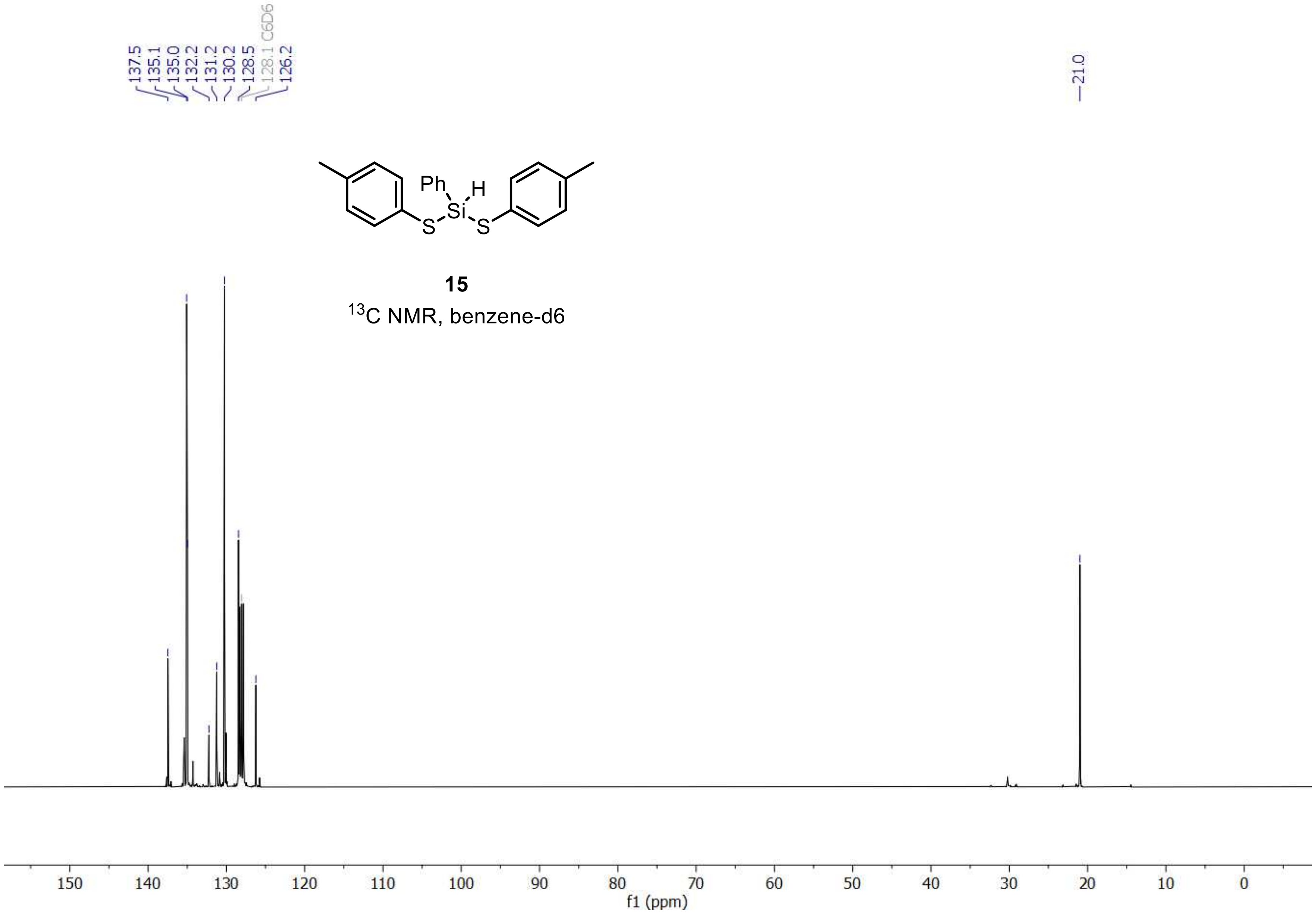


**15**

$^{13}C$ NMR, benzene-d6

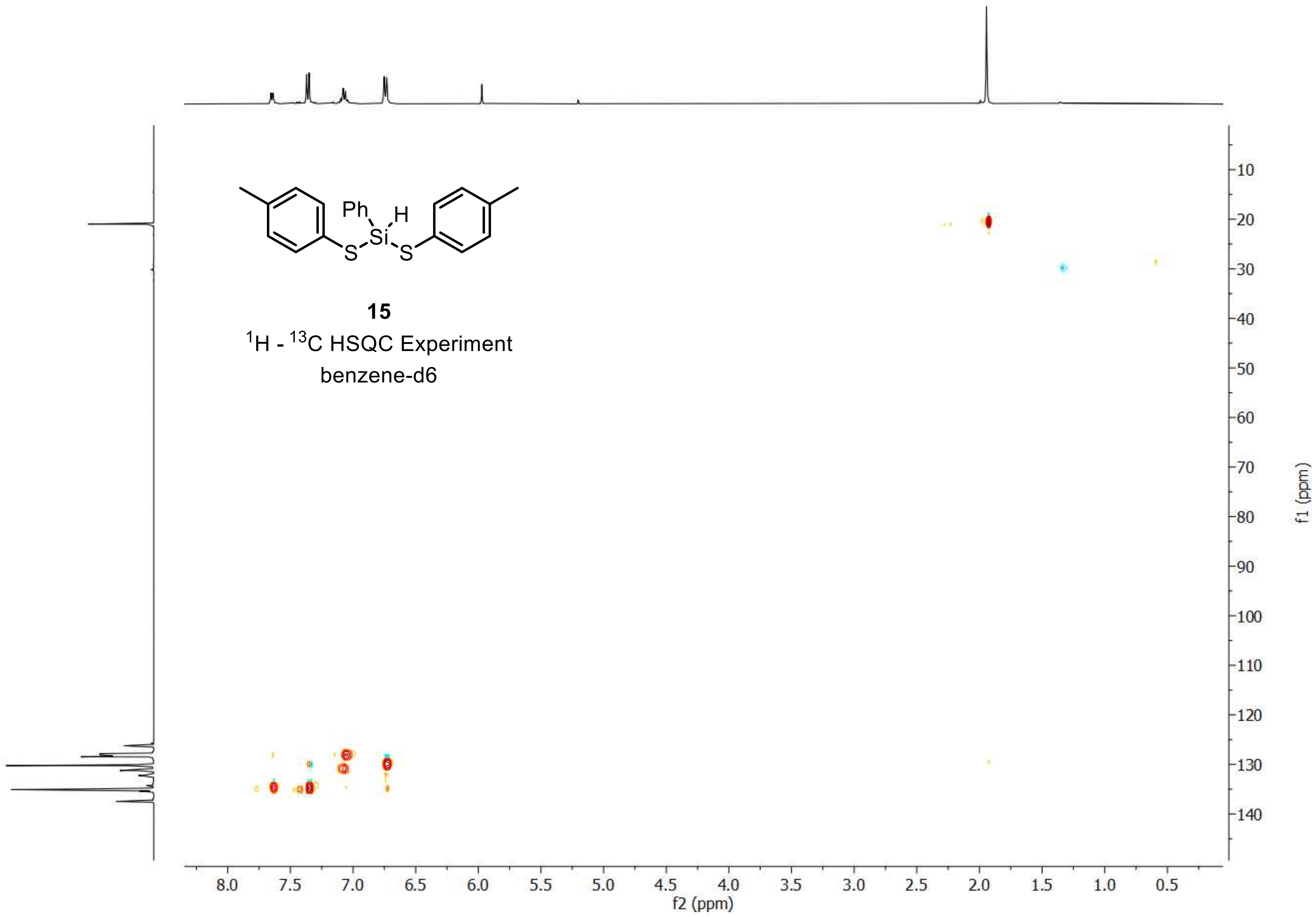

Ph
H
S
Si
S
15
1H - 13C HSQC Experiment
benzene-d6
f1 (ppm)
f2 (ppm)
8.0 7.5 7.0 6.5 6.0 5.5 5.0 4.5 4.0 3.5 3.0 2.5 2.0 1.5 1.0 0.5
10 20 30 40 50 60 70 80 90 100 110 120 130 140

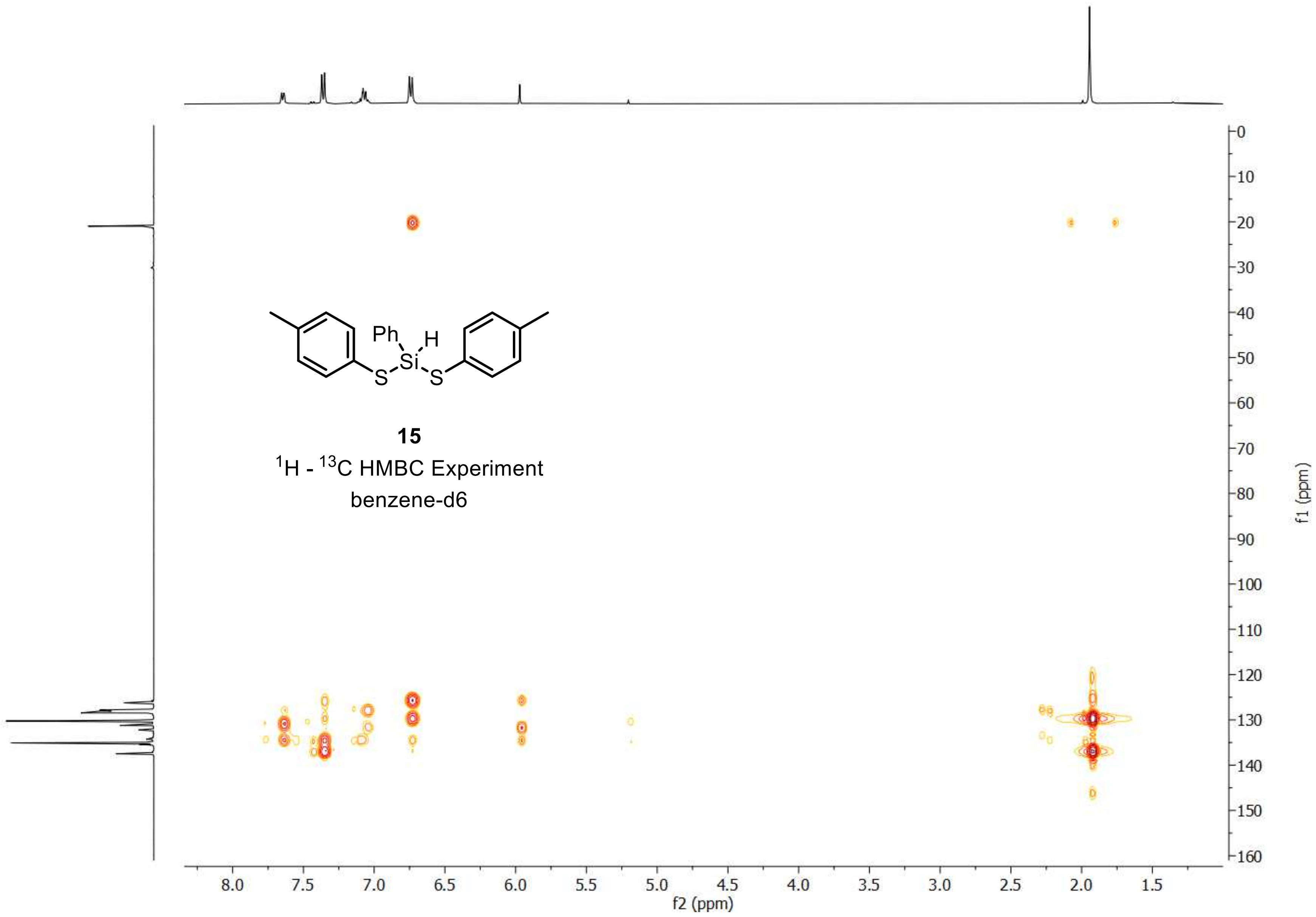
Ph
H
Si
S
S
15
1H - 13C HMBC Experiment
benzene-d6
f1 (ppm)
f2 (ppm)

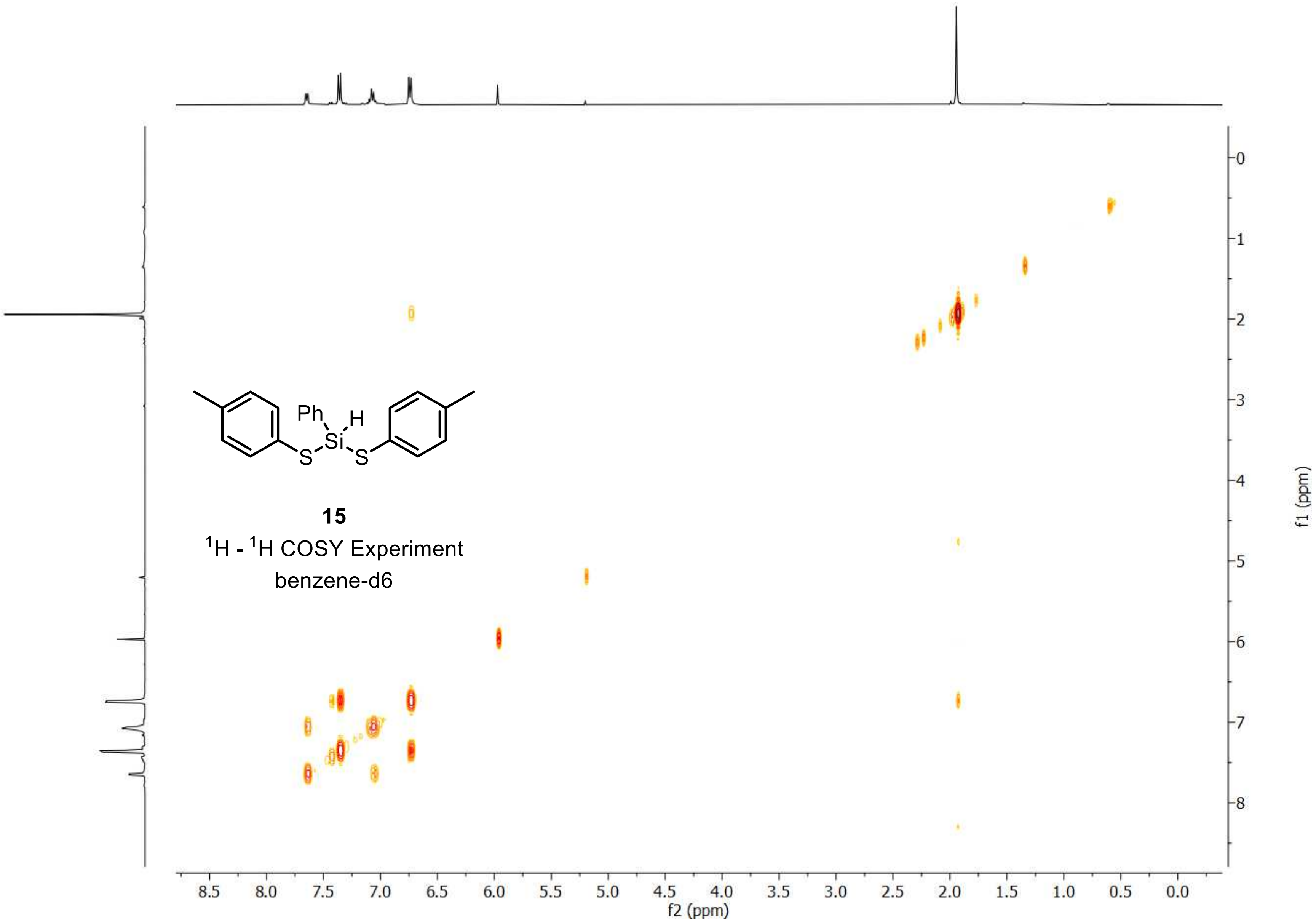
Ph
H
Si
S
S
15
1H - 1H COSY Experiment
benzene-d6
f2 (ppm)
f1 (ppm)

## 12 References


(1) W. V. Taylor, Z.-L. Xie, N. I. Cool, S. A. Shubert, M. J. Rose, Syntheses, Structures, and Characterization of Nickel(II) Stibines: Steric and Electronic Rationale for Metal Deposition. *Inorg. Chem.* **2018**, *57*, 10364.

(2) M. P. Doyle, W. J. Bryker, Alkyl Nitrite-Metal Halide Deamination Reactions. 6. Direct Synthesis of Arenediazonium Tetrafluoroborate Salts from Aromatic Amines, Tert-Butyl Nitrite, and Boron Trifluoride Etherate in Anhydrous Media. *J. Org. Chem.* **1979**, *44*, 1572.

(3) M. Deb, S. Hazra, P. Dolui, A. J. Elias, Ferrocenium Promoted Oxidation of Benzyl Amines to Imines Using Water as the Solvent and Air as the Oxidant. *ACS Sustainable Chem. Eng.* **2019**, *7*, 479.

(4) J. Liu, J. Chen, J. Zhao, Y. Zhao, L. Li, H. Zhang, A Modified Procedure for the Synthesis of 1-Arylimidazoles. *Synthesis 2003*, **2003**, 2661.

(5) J. C. Thomas, J. C. Peters, Bis(Phosphino)Borates: A New Family of Monoanionic Chelating Phosphine Ligands. *Inorg. Chem.* **2003**, *42*, 5055.

(6) G. R. Fulmer, A. J. M. Miller, N. H. Sherden, H. E. Gottlieb, A. Nudelman, B. M. Stoltz, J. E. Bercaw, K. I. Goldberg, NMR Chemical Shifts of Trace Impurities: Common Laboratory Solvents, Organics, and Gases in Deuterated Solvents Relevant to the Organometallic Chemist. *Organometallics* **2010**, *29*, 2176.

(7) I. V. Shishkov, F. Rominger, P. Hofmann, New Structural Motifs of Lithium N-Heterocyclic Carbene Complexes with Bis(3-Tert-Butylimidazol-2-Ylidene)Dialkylborate Ligands. *Organometallics* **2009**, *28*, 3532.

(8) F. W. Friese, A. Studer, New avenues for C–B bond formation via radical intermediates. *Chem. Sci.* **2019**, *10*, 8503

(9) M. Huang, K. Li, Z. Zhang, J. Zhou, Antimony Redox Catalysis: Hydroboration of Disulfides through Unique Sb(I)/Sb(III) Redox Cycling. *J. Am. Chem. Soc.* **2024**, *146*, 20432.

(10) Y. Pang, M. Leutzsch, N. Nöthling, F. Katzenburg, J. Cornella, Catalytic Hydrodefluorination via Oxidative Addition, Ligand Metathesis, and Reductive Elimination at Bi(I)/Bi(III) Centers. *J. Am. Chem. Soc.* **2021**, *143*, 12487.

(11) S. Pietsch, E. C. Neeve, D. C. Apperley, R. Bertermann, F. Mo, D. Qiu, M. S. Cheung, L. Dang, J. Wang, U. Radius, Z. Lin, C. Kleeberg, T. Marder, Synthesis, Structure, and Reactivity of Anionic $sp^2$–$sp^3$ Diboron Compounds: Readily Accessible Boryl Nucleophiles *Chem. Eur. J.* **2015**, *21*, 7082.

(12) P. Erdmann, J. Leitner, J. Schwarz, L. Greb, An Extensive Set of Accurate Fluoride Ion Affinities for p-Block Element Lewis Acids and Basic Design Principles for Strong Fluoride Ion Acceptors. *ChemPhysChem* **2020**, *21*, 987.

(13) L. Kuehn, M. Stang, S. Würtemberger-Pietsch, A. Friedrich, H. Scheneider, U. Radius, T. B. Marder, FBpin and its adducts and their role in catalytic borylations. *Faraday Discuss.* **2019**, *220*, 350.

(14) L. M. Sigmund, S. V. Shree Sowndary, A. Albers, P. Erdmann, R. S. Paton, L. Greb, Predicting Lewis Acidity: Machine Learning the Fluoride Ion Affinity of p-Block-Atom-Based Molecules. *Angew. Chem. Int. Ed*. **2024**, *63*, e202401084.

(15) Z. Liu, Z. Wang, H. Mu, Y. Zhou, J. Zhou, Synthesis and Redox Catalysis of Carbodiphosphorane Ligated Stannylene. *Nat. Commun*. **2024**, *15*, 9849.

(16) Gaussian 09, Revision A.02, M. J. Frisch, G. W. Trucks, H. B. Schlegel, G. E. Scuseria, M. A. Robb, J. R. Cheeseman, G. Scalmani, V. Barone, G. A. Petersson, H. Nakatsuji, X. Li, M. Caricato, A. Marenich, J. Bloino, B. G. Janesko, R. Gomperts, B. Mennucci, H. P. Hratchian, J. V. Ortiz, A. F. Izmaylov, J. L. Sonnenberg, D. Williams-Young, F. Ding, F. Lipparini, F. Egidi, J. Goings, B. Peng, A. Petrone, T. Henderson, D. Ranasinghe, V. G. Zakrzewski, J. Gao, N. Rega, G. Zheng, W. Liang, M. Hada, M. Ehara, K. Toyota, R. Fukuda, J. Hasegawa, M. Ishida, T. Nakajima, Y. Honda, O. Kitao, H. Nakai, T. Vreven, K. Throssell, J. A. Montgomery, Jr., J. E. Peralta, F. Ogliaro, M. Bearpark, J. J. Heyd, E. Brothers, K. N. Kudin, V. N. Staroverov, T. Keith, R. Kobayashi, J. Normand, K. Raghavachari, A. Rendell, J. C. Burant, S. S. Iyengar, J. Tomasi, M. Cossi, J. M. Millam, M. Klene, C. Adamo, R. Cammi, J. W. Ochterski, R. L. Martin, K. Morokuma, O. Farkas, J. B. Foresman, and D. J. Fox, Gaussian, Inc., Wallingford CT, 2016.

(17) C. Adamo, V. Barone, Toward Reliable Density Functional Methods without Adjustable Parameters: The PBE0 Model. *J. Chem. Phys.* **1999**, *110*, 6158.

(18) M. Ernzerhof, G. E. Scuseria, Assessment of the Perdew–Burke–Ernzerhof Exchange-Correlation Functional. *J. Chem. Phys.* **1999**, *110*, 5029.

(19) S. Grimme, J. Antony, S. Ehrlich, H. Krieg, A Consistent and Accurate Ab Initio Parametrization of Density Functional Dispersion Correction (DFT-D) for the 94 Elements H-Pu. *J. Chem. Phys.* **2010**, *132* , 154104.

(20) S. Grimme, S. Ehrlich, L. Goerigk, Effect of the Damping Function in Dispersion Corrected Density Functional Theory. *J. Comput. Chem.* **2011**, *32*, 1456.

(21) F. Weigend, R. Ahlrichs, Balanced Basis Sets of Split Valence, Triple Zeta Valence and Quadruple Zeta Valence Quality for H to Rn: Design and Assessment of Accuracy. *Phys. Chem. Chem. Phys.* **2005**, *7*, 3297.

(22) C. Peng, P. Y. Ayala, H. B. Schlegel, M. J. Frisch, Using Redundant Internal Coordinates to Optimize Equilibrium Geometries and Transition States. *J. Comput. Chem.* **1996**, *17*, 49.

(23) J. W. Jr. McIver, A. Komornicki, Structure of Transition States in Organic Reactions. General Theory and an Application to the Cyclobutene-Butadiene Isomerization Using a Semiempirical Molecular Orbital Method. *J. Am. Chem. Soc.* **1972**, *94*, 2625.

(24) A. E. Reed, L. A. Curtiss, F. Weinhold, Intermolecular Interactions from a Natural Bond Orbital, Donor-Acceptor Viewpoint. *Chem. Rev.* **1988**, *88*, 899.

(25) A. E. Reed, R. B. Weinstock, F. Weinhold, Natural Population Analysis. *J. Chem. Phys.* **1985**, *83*, 735.

(26) E. D. Glendening, J. K. Badenhoop, A. E. Reed, J. E. Carpenter, J. A. Bohmann, C. M. Morales, P. Karafiloglou, C. R. Landis, F. Weinhold, NBO 7.0. Theoretical Chemistry Institute, University of Wisconsin, Madison, **2018**.

(27) R. F. W. Bader, R. F. Bader, Atoms in Molecules: A Quantum Theory; International series of monographs on chemistry; Clarendon Press, **1990**.

(28) AIMAll (Version 19.10.12), Todd A. Keith, TK Gristmill Software, Overland Park KS, USA, **2019** (Aim.Tkgristmill.Com).

(29) E. Ramos-Cordoba, P. Salvador, I. Mayer, The Atomic Orbitals of the Topological Atom. *J. Chem. Phys.* **2013**, 138, 214107.

(30) V. Postils, C. Delgado-Alonso, J. M. Luis, P. Salvador, An Objective Alternative to IUPAC's Approach To Assign Oxidation States. *Angew. Chem. Int. Ed.* **2018**, *57*, 10525.

(31) P. Salvador, E. Ramos-Cordoba, M. Montilla, L. Pujal, M. Gimferrer, APOST-3D: Chemical Concepts from Wavefunction Analysis. *J. Chem. Phys.* **2024**, *160*, 172502.

(32) P. Salvador, E. Ramos-Cordoba, Communication: An Approximation to Bader's Topological Atom. *J. Chem. Phys.* **2013**, *139*, 071103.

(33) K. Morokuma, Molecular Orbital Studies of Hydrogen Bonds. III. C=O···H–O Hydrogen Bond in H2CO···H2O and H2CO···2H2O. *J. Chem. Phys.* **1971***,* *55*, 1236.

(34) T. Ziegler, A. Rauk, Carbon Monoxide, Carbon Monosulfide, Molecular Nitrogen, Phosphorus Trifluoride, and Methyl Isocyanide as Sigma Donors and Pi Acceptors. A Theoretical Study by the Hartree-Fock-Slater Transition-State Method. *Inorg. Chem.* **1979**, *18*, 1755.

(35) T. Ziegler, A. Rauk, A Theoretical Study of the Ethylene-Metal Bond in Complexes between Copper(1+), Silver(1+), Gold(1+), Platinum(0) or Platinum(2+) and Ethylene, Based on the Hartree-Fock-Slater Transition-State Method. *Inorg. Chem.* **1979**, *18*, 1558.

(36) F. M. Bickelhaupt, N. M. M. Nibbering, E. M. Van Wezenbeek, E. J. Baerends, Central Bond in the Three CN.Cntdot.Dimers NC-CN, CN-CN and CN-NC: Electron Pair Bonding and Pauli Repulsion Effects. *J. Chem. Phys.* **1992**, *96*, 4864.

(37) L. Zhao, M. von Hopffgarten, D. M. Andrada, G. Frenking, Energy Decomposition Analysis. G. *WIREs Comput. Mol. Sci.* **2018**, *8*, e1345.

(39) A. Michalak, M. Mitoraj, T. Ziegler, Bond Orbitals from Chemical Valence Theory. *J. Chem. Phys. A.* **2008**, *112*, 1933.

(40) M. Mitoraj, A. Michalak, Applications of Natural Orbitals for Chemical Valence in a Description of Bonding in Conjugated Molecules. *J. Mol. Model.* **2008**, *14*, 681.

(41) M. P. Mitoraj, A. Michalak, T. A. Ziegler, A Combined Charge and Energy Decomposition Scheme for Bond Analysis. *J. Chem. Theory Comput.* **2009**, *5*, 962.

(42) G. te Velde, F. M. Bickelhaupt, E. J. Baerends, C. Fonseca Guerra, S. J. A. van Gisbergen, J. G. Snijders, T. Ziegler, Chemistry with ADF. *J. Comput. Chem.* **2001**, *22*, 931.

(43) J. P. Perdew, Density-Functional Approximation for the Correlation Energy of the Inhomogeneous Electron Gas. *Phys. Rev. B* **1986**, *33*, 8822.

(44) A. D. Becke, Density-Functional Exchange-Energy Approximation with Correct Asymptotic Behavior. *Phys. Rev. A* **1988**, *38*, 3098.

(45) J. Krijn; E. J. Baerends: Fit Functions in the HFS-Method, Internal Report (in Dutch). Amsterdam, the Netherlands: Vrije Universiteit Amsterdam, **1984**.

(46) E. Lenthe, E. J. van Baerends, J. G. Snijders, Relativistic Regular Two-component Hamiltonians. *J. Chem. Phys.* **1993**, *99*, 4597.

(47) Q. Sun, T. C. Berkelbach, N. S. Blunt, G. H. Booth, S. Guo, Z. Li, J. Liu, J. D. McClain, E. R. Sayfutyarova, S. Sharma, S. Wouters, G. K.-L. Chan, PySCF: The Python-Based Simulations of Chemistry Framework. *WIREs Comput. Mol. Sci.* **2018**, *8*, e1340.

(48) F. Neese, Software Update: The ORCA Program System—Version 6.0. *WIREs Comput Mol Sci.* **2025**, *15*, e70019.

(49) F. Weigend, R. Ahlrichs. Balanced basis sets of split valence, triple zeta valence and quadruple zeta valence quality for H to Rn: Design and assessment of accuracy. *Phys. Chem. Chem. Phys.* **2005**, *7*, 3297.

(50) J. D. Rolfes, F. Neese, D. A. Pantazis. All-electron scalar relativistic basis sets for the elements Rb–Xe. *J. Comput. Chem.* **2020**, *41*, 1842.

(51) D. A. Pantazis, F. Neese. All-electron scalar relativistic basis sets for the 6p elements. *Theor. Chem. Acc.* **2012**, *131*, 1292.

(52) D. A. Pantazis, F. Neese. All-electron basis sets for heavy elements. *WIREs Comput Mol Sci.* **2014**, *4*, 363.
